# Quality of Artemisinin-Based Combination Formulations for Malaria Treatment: Prevalence and Risk Factors for Poor Quality Medicines in Public Facilities and Private Sector Drug Outlets in Enugu, Nigeria

**DOI:** 10.1371/journal.pone.0125577

**Published:** 2015-05-27

**Authors:** Harparkash Kaur, Elizabeth Louise Allan, Ibrahim Mamadu, Zoe Hall, Ogochukwu Ibe, Mohamed El Sherbiny, Albert van Wyk, Shunmay Yeung, Isabel Swamidoss, Michael D. Green, Prabha Dwivedi, Maria Julia Culzoni, Siân Clarke, David Schellenberg, Facundo M. Fernández, Obinna Onwujekwe

**Affiliations:** 1 Department of Clinical Research, London School of Hygiene & Tropical Medicine, Keppel Street, London, United Kingdom; 2 Department of Global Health and Development, London School of Hygiene & Tropical Medicine, Tavistock Place, London, United Kingdom; 3 Department of Pharmacology and Therapeutics, University of Nigeria, Enugu, Nigeria; 4 Division of Parasitic Diseases and Malaria, US Centers for Disease Control and Prevention, Atlanta, Georgia, United States of America; 5 Centers for Disease Control and Prevention, Atlanta, Georgia, United States of America; 6 Facultad de Bioquímica y Ciencias Biológicas, Universidad Nacional del Litoral, and Consejo Nacional de Investigaciones Científicas y Técnicas (CONICET), Santa Fe, Argentina; 7 Department of Disease Control, London School of Hygiene & Tropical Medicine, Keppel Street, London, United Kingdom; 8 School of Chemistry and Biochemistry, Georgia Institute of Technology, Atlanta, Georgia, United States of America; Royal Tropical Institute, NETHERLANDS

## Abstract

**Background:**

Artemisinin-based combination therapies are recommended by the World Health Organisation (WHO) as first-line treatment for *Plasmodium falciparum* malaria, yet medication must be of good quality for efficacious treatment. A recent meta-analysis reported 35% (796/2,296) of antimalarial drug samples from 21 Sub-Saharan African countries, purchased from outlets predominantly using convenience sampling, failed chemical content analysis. We used three sampling strategies to purchase artemisinin-containing antimalarials (ACAs) in Enugu metropolis, Nigeria, and compared the resulting quality estimates.

**Methods:**

ACAs were purchased using three sampling approaches - convenience, mystery clients and overt, within a defined area and sampling frame in Enugu metropolis. The active pharmaceutical ingredients were assessed using high-performance liquid chromatography and confirmed by mass spectrometry at three independent laboratories. Results were expressed as percentage of APIs stated on the packaging and used to categorise each sample as acceptable quality, substandard, degraded, or falsified.

**Results:**

Content analysis of 3024 samples purchased from 421 outlets using convenience (n=200), mystery (n=1,919) and overt (n=905) approaches, showed overall 90.8% ACAs to be of acceptable quality, 6.8% substandard, 1.3% degraded and 1.2% falsified. Convenience sampling yielded a significantly higher prevalence of poor quality ACAs, but was not evident by the mystery and overt sampling strategies both of which yielded results that were comparable between each other. Artesunate (n=135; 4 falsified) and dihydroartemisinin (n=14) monotherapy tablets, not recommended by WHO, were also identified.

**Conclusion:**

Randomised sampling identified fewer falsified ACAs than previously reported by convenience approaches. Our findings emphasise the need for specific consideration to be given to sampling frame and sampling approach if representative information on drug quality is to be obtained.

## Introduction

*Plasmodium falciparum* (*Pf*) causes 207 million malaria cases each year, resulting in 562,000 deaths [1]. Artemisinin-based combination therapies (ACTs) are recommended by the World Health Organisation (WHO) as first-line treatment for *Pf* malaria. Efficacious malaria control and treatment requires the use of good quality medication [1] Substandard, degraded and falsified (also referred to as counterfeit or spurious medicines) ACTs pose a threat to malaria patients [2] and may accelerate the spread of drug resistance [3].

*Ad hoc* surveys in South East Asia have found up to half of artesunate monotherapy tablets were falsified [4–9]. Falsified monotherapies and ACTs have also been reported in Africa, where there is a high burden of potentially fatal *Pf* malaria [10]. With 48 million clinical episodes and 180,000 deaths per year, Nigeria is the single most heavily malaria-burdened country in the world. Malaria accounts for 60% of outpatient visits, 30% of hospitalizations under five years of age [11], and 11% of maternal deaths [12]. In 2005 Nigeria adopted the ACT artemether/lumefantrine as the first-line treatment for uncomplicated malaria at public health facilities, and subsequently added artesunate/amodiaquine [13]. In Southeast Nigeria in 2008, 27% of non-ACT antimalarial drugs (60 out of 225) did not meet the United States Pharmacopoeia (USP) quality specifications for dissolution testing [14]; ACT formulations were not obtainable in that region at the time.

The considerable technical, financial and human resources required to inspect, analyse and police the drug supply are lacking in most malaria endemic countries. A systematic review of literature reported that studies that used strong methodology were few and majority did not differentiate between substandard and counterfeit medicines [15]. Sample collection methods require epidemiological knowledge and an adequate sample size to provide a suitable estimate of prevalence which would subsequently justify and promote the political will to put in place mechanisms needed to assure drug quality [16].

This study reports the findings of different sampling approaches (convenience, mystery clients and overt) on the prevalence and, estimates of poor quality artemisinin-containing antimalarials (ACAs) in a single geographical area.

## Methods

### Study area and sample collection

Enugu metropolis (S1 Fig), Southeast Nigeria, with a population of 3.3 million, bears 1.1% of the national malaria burden of 3,300 annual malaria deaths.[13]. *Pf* is the most prevalent parasite and the state is considered holo-endemic for malaria [17].

#### Convenience approach

This frequently-used purposeful sampling involves the surveyors purchasing medicines without specific guidance on which outlets to sample [18]. A geo-referenced list of all government facilities and outlets selling ACAs was obtained from a related project (Research on the Economics of Artemisinin Combination Therapies—REACT) [19]. These 372 outlets were concentrated in three main local government areas: Enugu East, Enugu North and Enugu South. The REACT list was verified in June 2012 to check that they were still operating prior to purchasing the ACAs. A total of 23 outlets (pharmacies and patent medicine vendors) were selected for sampling based on geographical ratio: Enugu East 94 outlets (25%); Enugu North 149 outlets (40%); Enugu South 129 outlets (35%), i.e. 5:8:7. A total of 200 samples were purchased by selecting one sample of every available brand of artemisinin combination therapy (ACT) and artemisinin monotherapy (AMT) from each of the 23 outlets, by four surveyors over seven days.

#### Mystery client approach

Sampling was based on the previous census above and a list obtained from the Ministry of Health (director of pharmacy). Eight teams of two 'mystery client' surveyors updated the two lists in December 2012 to verify which outlets were still trading and to add new outlets. The updated list comprised a total of 489 outlets (138 pharmacies, 307 patent medicine vendors and 44 public health facilities), of which 279 were found to sell ACAs (not all outlets listed sold them). In order to sample for ACTs one surveyor approached the provider masquerading as a patient or their care-giver, or stating that they needed to send the most effective antimalarials to relatives in a village and asked to see all ACAs before purchasing as many as possible without raising suspicion. The other surveyor remained outside the outlet and covertly recorded its Global Positioning System (GPS) co-ordinates.

#### Overt approach

The sampling frame was updated in February 2013, as part of an overt survey undertaken by four teams of two surveyors. A total of 119 outlets were visited using this overt sampling approach, of which 98 had previously been visited during the mystery client survey. The provider was informed of the purpose of the drug purchase and their consent sought to participate in the study. An outlet questionnaire was completed before purchases to document the availability, supply and cost of ACAs, as well as provider's level of training.

### Sample processing and laboratory analyses

For every sample purchased, the outlet type, date of purchase, price paid, brand name, formulation, batch number, manufacture and expiry date were recorded on a standard form. Every sample was placed in an individual zip-lock bag together with data recorded on their respective questionnaires and stored securely in an air conditioned room (≈20°C) pending dispatch using, *DHL* Express, to the London School of Hygiene and Tropical Medicine (LSHTM), London, UK for further processing.

Digital photographs of packaging and contents were taken and sample information (brand name, stated active pharmaceutical ingredients (APIs), dose form, outlet, district, date of purchase, name of stated manufacturer, country of manufacture, National Agency for Food and Drug Administration and Control (NAFDAC) registration number, presence of the Affordable Medicines Facility—malaria (AMFm) logo (green leaf), batch number, date of manufacture, expiry date, number of tablets per packet and price paid) checked with the information collected in the field and logged in a data collection tool (Epi Info v.3.5.) [20]. Tablets were weighed and their dimensions recorded prior to laboratory analysis.

### Chemical content analysis

For quantitative analysis by high performance liquid chromatography (HPLC), tablets were dissolved in a solvent (S1 Table), sonicated and centrifuged. The supernatant was then injected into the HPLC column and the amount of APIs present in the tablet was determined. Injectable forms of antimalarials and suppositories were dissolved in methanol prior to HPLC analysis. HPLC analysis was conducted using a Dionex Ultimate 3000 system (Thermofisher, Hemel Hempstead, UK) and separation achieved using a GENESIS AQ 4 μm column (150 x 4.6 mm, Grace Materials Technologies, Cranforth, UK) or Acclaim 120, C18, 5 μm Analytical (4.6 x 150 mm) from Fisher Scientific, Leicestershire, UK. The mobile phase was a gradient of ammonium formate (10 mM, pH 2.7) and acetonitrile (v/v; 15:85 to 85:15 over 7.0 min). A photo-diode array detector (UV-PDA; DAD 3000) set at 204 nm for the artemisinin and the derivatives artesunate, artemether and dihydroartemisinin, 360 nm for piperaquine, amodiaquine and lumafantrine; 275 nm for sulfadoxine, sulfamethoxypyrazine and pyramethamine; 259 nm for mefloquine. In all cases, the flow rate used was 1.0 mL/min. A duplicate sample of the ones analysed at LSHTM from each packet were sent to the US Centres for Disease Control and Prevention (CDC) Laboratories Atlanta, USA for confirmatory HPLC analyses, and to the Georgia Institute of Technology (GT), Atlanta, USA for ambient mass spectrometry (MS) analyses to verify the ingredients present and identify any unstated compounds [21, 22]. In general there was good agreement between the results from the two laboratories where quantitation work was carried out (CDC and LSHTM) as shown by the Bland Altman plots (S2 Fig).

### Classification of samples

Laboratory results were expressed as a percentage of the API (% API) stated on the packaging for both APIs, the artemisinin derivative (AD) and the partner drug (PD) which is a non-artemisinin antimalarial. To accommodate the variation in HPLC laboratory testing of less than 10 samples of each ACA formulation, we adopted the tolerance band of 85% to 115% for the purposes of this study. If the % API of a drug was found to be outside of this tolerance band then a second sample from the same packet was reanalysed (with the exception of suspensions as the entire bottle was used in the first analysis) and the majority outcome reported. The % API used to classify each sample as one of the following: being of acceptable quality (85–115% for both APIs); substandard (>0% but <85%, or >115% of either API); degraded (products of degradation of either or both APIs present); or falsified (0% APIs). Classification of analysed drugs as “degraded” was achieved through comparison of HPLC retention times and MS patterns of good quality drugs with that obtained from artificially degraded samples. Artificial degradation was achieved by placing good quality artemether/lumefantrine (Coartem, Novartis, China) and artesunate/amodiaquine tablets (Winthrop, Maphar Laboratories, Morocco) in an oven at 60°C for 21 days [23].

### Data analysis

Laboratory data were combined with survey and sample data (S1 Database) and analysed using STATA v12 (Statacorp, College station, Texas). Proportions were evaluated using Chi-square tests and crude associations between poor quality ACAs and variables collected from the questionnaire were examined. A univariate logistic model assessed associations with poor quality products and factors associated with a *p*-value <0.1 on crude analysis were included in a multivariate model. The svy command was used to account for clustering at the outlet level.

### Ethics approvals

The study was approved by the London School Hygiene & Tropical Medicine, Ethics Committee (Ref: 5804) and the Enugu State Ethics Committee (Ref: UNTH/CSA.329/VOL.5). This study did not involve patients and hence consent is not applicable. Verbal consent was given by the person in charge of the outlet during the overt sampling approach which is recorded on the database. Findings were reported to the Ministry of Health, Enugu state; manufacturers whose products were found to have been falsified; Global Fund drug quality section and the World Health Organisation (rapidalert@who.int).

## Results

A total of 3,024 ACA samples were analysed of the 3065 purchased using the three sampling approaches from 421 outlets (35.6% pharmacies, 60.6% patent medicine vendors, 3.6% public health facilities, and 0.2% market stalls). Overall, approximately half of the pharmacies and patent medicine vendors had an ACA available at the time of the overt survey, and typically had 2–3 staff of which at least one had a local secondary school qualification (Table 1). Pharmacies were more likely than patent medicine vendors to have staff with a health-related qualification and recent malaria training (past 12 months), and on average stocked almost twice as many ACA samples of varying brands than patent medicine vendors.

**Table 1:** Description of outlets sampled using the overt sampling approach.

					Outlets with ≥1 staff member with:			
Outlet types	Outlets enumerated	Outlets with ≥1 ACA in stock	Number of Staff surveyed	Median number of staff per outlet (IQR)	secondary school qualification	health related qualification	malaria training (past 12 months)	Mean number of ACA samples per outlet
Pharmacies	83	54 (65.1%)	53 (98.1%)	3 (2,6)	51 (94.4%)	31 (57.4%)	33 (61.1%)	10.2
PMVs	163	65 (39.9%)	63 (96.9%)	2 (1,2)	60 (92.3%)	25 (38.5%)	33 (50.8%)	5.7
χ^2^ *p*-value	-	0.28	0.73	-	0.54	0.14	0.40	-
**Total**	246	119 (48.4%)	116 (97.5%)	2 (1,3)	111 (93.3%)	56 (47.1%)	66 (55.5%)	-

ACA = artemisinin-containing antimalarial; IQR = interquartile range; PMVs = patent medicine vendors.

Table 2 shows the quality of ACAs found using the alternative sampling strategies. Convenience sampling approach yielded 190 ACTs and 10 AMTs, representing 49 different brands, purchased from 23 outlets. In the convenience sample 3.0% of the ACAs purchased were found to be falsified falsified, 2.0% degraded, and 10.5% substandard (4.0% with respect to API 1; 6.0% for API 2; 0.5% for both APIs), while 84.5% were of acceptable quality. The mystery clients approach yielded 102 brands purchased from 277 outlets (1,794 ACT and 125 AMT samples); of which 1.2% were falsified, 1.3% degraded, 6.4% substandard (2.9% API 1; 1.7% API 2; 1.8% both APIs), and 91.1% were of acceptable quality drugs. The overt sampling approach resulted in 846 ACTs and 59 AMTs, including 79 brands from 119 outlets. From these samples, 0.6% were falsified, 1.0% degraded, 7.0% substandard (3.8% API 1; 2.3% API 2; 0.9% both APIs), and 91.5% were of acceptable quality. Mass Spectrometry analyses of falsified samples confirmed the absence of the stated APIs and tentatively identified the presence of other potentially harmful compounds; including *bis* (2-ethylhexyl) adipate, dioctyl adipate, chlorzoxazone, ciprofloxacin and acetaminophen (S2 Table).

**Table 2:** Quality of Artemisinin-Containing Antimalarials (ACAs) purchased per outlet using convenience, mystery clients and overt sampling approaches in Enugu, Nigeria; n = 3024.

Outlets (n)	Acceptable quality for both AD & PD	Substandard AD only	Substandard PD only	Substandard AD & PD	Degraded	Falsified	Total
**Convenience (n = 200; total brands = 49; brands per outlet = 2.1)**							
Pharmacies (4)	62 (88.6%)	1 (1.4%)	3 (4.3%)	0	2 (2.9%)	2 (2.9%)	70
PMVs (16)	97 (81.5%)	6 (5.0%)	9 (7.6%)	1 (0.8%)	2 (1.7%)	4 (3.4%)	119
Public health facilities (2)	4 (80.0%)	1 (20.0%)	0	0	0	0	5
Market stalls (1)	6 (100.0%)	0	0	0	0	0	6
All outlets (23)	169 (84.5%)^a^ ^,^ ^b^	8 (4.0%)	12 (6.0%)^c^ ^,^ ^d^	1 (0.5%)	4 (2.0%)	6 (3.0%)^e^ ^,^ ^f^	200
**Mystery clients (n = 1919; total brands = 102; brands per outlet = 0.4)**							
Pharmacies (92)	803 (90.0%)	23 (2.6%)	25 (2.8%)	20 (2.2%)	16 (1.8%)	5 (0.6%)	892
PMVs (174)	94 (91.9%)	29 (3.0%)	7 (0.7%)	15 (1.5%)	9 (0.9%)	19 (2.0%)	973
Public health facilities (13)	51 (94.4%)	3 (5.6%)	Acceptable 0	0	0	0	54
All outlets (279)	1748 (91.1%)^a^	55 (2.9%)	32 (1.7%)^c^	35 (1.8%)	25 (1.3%)	24 (1.2%)^e^	1919
**Overt (n = 905; total brands = 79; brands per outlet = 0.7)**							
Pharmacies (54)	488 (89.4%)	27 (4.9%)	16 (2.9%)	7 (1.3%)	8 (1.5%)	0	546
PMVs (65)	340 (94.7%)	7 (1.9%)	5 (1.4%)	1 (0.3%)	1 (0.3%)	5 (1.4%)	359
Public health facilities (0)	-	-	-	-	-	-	-
All outlets (119)	828 (91.5%)^b^	34 (3.8%)	21 (2.3%)^d^	8 (0.9%)	9 (1.0%)	5 (0.6%)	905

ACAs = artemisinin containing antimalarial; AD = artemisinin derivative (always the ACAs); PD = partner drug (always the non-ACAs); PMVs = patent medicine vendors. Substandard = genuine medicines produced by legitimate manufacturers that do not satisfy the pharma set quality specifications. Degraded = result from exposure of good quality medicines to excessive heat and humidity. Falsified = deliberately and fraudulently mislabelled with respect to identity and/or source. Include both branded and generic products and may include wrong ingredients or no active ingredients or counterfeited packaging. Significant difference between sampling approaches for each quality category are indicated as follows: between convenience and mystery clients =

^a^ (*p* = 0.002),

^c^ (*p*<0.001),

^e^ (*p* = 0.046); between convenience and overt =

^b, f^ (*p* = 0.002),

^d^ (*p* = 0.006). Other comparisons were not significantly different (*p*>0.05).

All the sampling approaches used detected falsified drugs; one of the falsified products was detected amongst samples collected by at least two of the different sampling approaches. A significantly higher proportion of falsified drugs were found in samples purchased using the convenience approach compared to mystery and overt approaches (Table 2, *p* = 0.002 and 0.046, respectively). The proportion of substandard and degraded drugs, however, did not vary significantly between the three sampling approaches (*p*>0.05 in all cases).A subsample of 98 outlets were visited during both mystery and overt sampling (Table 3). From these outlets a similar number of samples (≈720), brands (≈75; *p* = 0.35), and poor quality drugs (*p*>0.05 in all cases) were purchased using each approach. As mystery and overt sampling approaches showed no difference in the proportion of poor quality drugs purchased, unlike the convenience approach, all subsequent STATA analyses were restricted to the overt and mystery clients data sets.

**Table 3:** Quality of Artemisinin-Containing Antimalarials (ACAs) at 98 outlets visited during both mystery clients and overt sampling in Enugu, Nigeria.

	Sampling method		
Variable	Mystery clients	Overt	*p*-value
Outlets	98 of 277 (35.4%)	98 of 119 (82.4%)	
Samples	720	721	
Brands	78 (72.9%)	72 (67.3%)	0.37
Acceptable quality	669 (92.9%)	665 (92.2%)	0.62
Substandard	35 (4.9%)	46 (6.4%)	0.21
Degraded	7 (1.0%)	5 (0.7%)	0.56
Falsified	9 (1.3%)	5 (0.7%)	0.28

Total number of brands purchased were 107

During the mystery and overt sampling over 80% of the ACAs collected were sold as tablets and approximately a quarter of medicines were manufactured by WHO prequalified companies (Table 4). Both ACTs (2,679) and AMTs (186), including monotherapy suspensions (36), injections (1) and tablets (149), were purchased for testing. The availability of monotherapy tablets (artesunate and dihydroartemisinin) did not differ according to sampling approach [107/1819 (5.9%) by mystery and 42/860 (4.9% by overt; *p* = 0.29). The proportion of medications not produced by WHO prequalified manufacturers was significantly higher in pharmacies than patent medicine vendors in both sampling approaches (*p*<0.001 for both).

**Table 4:** Description of all samples purchased using mystery clients and overt sampling approaches in Enugu, Nigeria; N = 2865.

	Mystery clients					Overt			
Variable	Total samples	Pharmacies	PMVs	*p*-value^a^	PHFs	Total samples	Pharmacies	PMVs	*p*-value^a^
**Combination therapies**									
AM-LUM	1145 (62.9%)	509 (59.0%)	597 (66.0%)	0.04	39 (73.6%)	581 (67.6%)	344 (66.4%)	237 (69.3%)	0.44
AS-ADQ	239 (13.1%)	117 (13.6%)	108 (11.9%)	0.72	14 (26.4%)	78 (9.1%)	48 (9.3%)	30 (8.8%)	0.97
AS-MEF	47 (2.6%)	32 (3.7%)	15 (1.7%)	0.71	0	25 (2.9%)	19 (3.7%)	6 (1.8%)	0.81
AS-SULDOX-PYR	12 (0.7%)	8 (0.9%)	4 (0.4%)	0.97	0	4 (0.5%)	2 (0.4%)	2 (0.6%)	0.97
AS-SULFMEX-PYR	28 (1.5%)	24 (2.8%)	4 (0.4%)	0.93	0	21 (2.4%)	18 (3.5%)	3 (0.9%)	0.83
DHA-PIP	344 (18.9%)	171 (19.8%)	173 (19.1%)	0.85	0	151 (17.6%)	87 (16.8%)	64 (18.7%)	0.73
ART-PIP	1 (0.1%)	0	1 (0.1%)	-	0	0	0	0	
DHA-ADQ	3 (0.2%)	1 (0.1%)	2 (0.2%)	-	0	0	0	0	
**Total**	1819^b^	862	904		53	860^b^	518	342	
**All monotherapies (suspensions n = 36, injectables n = 1, and tablets n = 149)**									
AM	1 (0.8%)	0	0	0	1 (50.0%)	0	0	0	
DHA	14 (11.0%)	10 (21.3%)	4 (5.1%)	0.1	0	1 (1.7%)	0	1 (4.3%)	1.00
AS	112 (88.2%)	37 (78.7%)	74 (94.9%)	<0.001	1 (50.0%)	58 (98.3%)	36 (100.0%)	22 (95.7%)	0.07
**Total**	127^b^	47	78		2	59^b^	36	23	
**Monotherapy tablets (n = 149)**									
AM	0	0	0		0	0	0	0	
DHA	13 (12.1%)	10 (23.3%)	3 (4.8%)	<0.01	0	1 (2.4%)	0	1 (5.0%)	0.48
AS	94 (87.9%)	33 (76.7%)	60 (95.2%)	<0.01	1 (100.0%)	41 (97.6%)	22 (100.0%)	19 (95.0%)	0.48
**Total**	107^b^	43	63		1	42^b^	22	20	
**Dose form**									
Tablets	1710 (87.9%)	792 (87.2%)	866 (88.1%)	0.57	52 (94.5%)	774 (84.2%)	446 (80.5%)	328 (89.9%)	<0.001
Capsules	5 (0.3%)	4 (4.4%)	0	-	1 (1.8%)	3 (0.3%)	2 (0.4%)	1 (0.3%)	
Suspensions	187 (9.6%)	87 (9.6%)	99 (10.1%)	0.92	1 (1.8%)	131 (14.3%)	97 (17.5%)	34 (9.3%)	0.24
Injectables	1 (0.1%)	0	0	-	1 (1.8%)	0	0	0	
Granules/powders	40 (2.1%)	24 (2.6%)	16 (1.6%)	0.83	0	11 (1.2%)	9 (1.6%)	2 (0.5%)	0.91
Soft gels	3 (0.2%)	1 (0.1%)	2 (0.2%)	-	0	0	0	0	
**Total**	1946	908	983		55	919	554	365	
**WHO prequalification**									
Not WHO prequalified	1386 (71.2%)	727 (80.0%)	638 (64.9%)	<0.001	21 (38.2%)	702 (76.4%)	450 (81.2%)	252 (69.0%)	<0.001
**Stated region of manufacture**									
Africa	381 (19.6%)	182 (20.0%)	182 (18.5%)	0.66	17 (30.9%)	168 (18.3%)	96 (17.3%)	72 (19.7%)	0.69
Asia	1324 (68.0%)	619 (68.2%)	671 (68.3%)	1	34 (61.8%)	644 (70.1%)	387 (69.9%)	257 (70.4%)	0.89
Europe	90 (4.6%)	65 (7.2%)	25 (2.5%)	0.39	0	60 (6.5%)	47 (8.5%)	13 (3.6%)	0.30
North America	149 (7.7%)	42 (4.6%)	104 (10.6%)	0.25	3 (5.5%)	44 (4.8%)	21 (3.8%)	23 (6.3%)	0.70
Unknown	2 (0.1%)	0	1 (0.1%)	-	1 (1.8%)	3 (0.3%)	3 (0.5%)	0	
**Total**	1946	908	983		55	919	554	365	

AM = artemether; ADQ = -amodiaquine; ART = artemisinin; AS = artesunate; DHA = dihydroartemisinin; LUM = lumefantrine; MEF = mefloquine; PIP = piperaquine; PYR = pyrimethamine; SULDOX = sulfadoxine; SULFMEX = sulfamethoxypyridazine; PHCs = public health facilities; PMVs = patent medicine vendors; WHO = World Health Organisation.

^a^only pharmacies and patent medicine vendors were compared as public health facilities were not visited during the overt sampling approach.

^b^no significant difference (*p* = 0.29) between the proportion of monotherapy tablets purchased using the mystery clients (5.5%) and overt (4.6%) sampling approaches.

### Risk factors for substandard, degraded and falsified drugs

Generic dihydroartemisinin formulations were more likely to be substandard, degraded or falsified than artemether or artesunate formulations (Table 5). Samples labelled with the AMFm logo or from WHO prequalified manufacturers were more likely to be of good quality than those that were not. However, four Coartem samples, batch number F2261, allegedly manufactured by Novartis and with the AMFm logo, were found to be falsified, containing chloroxazone instead of the stated APIs. The risk of a drug being of poor quality—predominantly substandard and degraded—also differed according to stated country of manufacture while the risk of buying a falsified drug was five times greater if it had been manufactured locally (stated on the packaging as Nigeria) after controlling for other factors; adjusted odds ratio 5.0 (95% CI 1.9–13.2). In addition, the risk of buying a falsified drug amongst drugs purchased from a patent medicine vendor than amongst drugs purchased from a pharmacy; adjusted odds ratio 3.9 (95% CI 1.5–10.1).

**Table 5:** Univariate and multivariate analyses of associations of substandard, degraded and falsified samples with key risk factors (significant associations presented); N = 2824.

Variable		Total samples	Poor quality samples	Crude odds ratios (95% CI)	Adjusted odds ratios (95% CI)
**The following are significant risk factors for poor quality (substandard, degraded and falsified) ACAs** ^**b**^					
Generic type	AM	1701	80 (4.7%)	1	1
	DHA	501	72 (14.4%)	3.4 (2.4,4.9)	2.4 (1.6,3.4)
	AS	622	43 (6.9%)	1.5 (1.0,2.2)	1.4 (0.9,2.2)
WHO prequalified/ QAACT	not prequalified	2047	190 (9.3%)	1	1
	Prequalified	777	5 (0.6%)	0.06 (0.02,0.15)	0.08 (0.02,0.3)
AMFm	non AMFm drugs	2072	191 (9.3%)	1	1
	AMFm drugs	752	4 (0.5%)	0.05 (0.02,0.13)	0.24 (0.1,0.8)
Region of stated country of manufacture	Asia	1940	159 (8.2%)	1	1
	Africa	546	30 (5.5%)	0.7 (0.4,1.0)	2.1 (1.3,3.2)
	Europe	141	1 (0.7%)	0.08 (0.01,0.6)	0.04 (0.06,0.4)
	North America/unknown	197	5 (2.6%)	0.3 (0.1,0.7)	12.5 (2.7,56.9)
Expired at time of analysis	not expired	2537	132 (5.2%)	1	1
	expired^a^	275	59 (21.5%)	5.0 (3.6,6.9)	6.4 (4.4,9.3)
**The following are significant risk factors specifically for falsified ACAs** ^**c**^					
Outlet type	Pharmacies	1438	5 (0.4%)	1	1
	PMVs	1332	24 (1.8%)	4.2 (2.0,12.0)	3.9 (1.5,10.1)
	public health facilities	54	0	1	1
Generic type	AM	1701	8 (0.5%)	1	1
	DHA	501	18 (3.6%)	7.8 (3.4,18.3)	5.9 (1.9,18.1)
	AS	622	3 (0.5%)	1.0 (0.3,4.1)	0.9 (0.2,3.5)
Region of stated country of manufacture	Asia	1940	7 (0.4%)	1	1
	Africa	546	17 (3.1%)	8.9 (3.5,22.4)	5.0 (1.9,13.2)
	Europe	141	0	1	1
	North America/unknown	197	5 (2.5%)	7.2 (2.4,21.1)	27.9 (5.2,149.4)

ACAs = artemisinin-containing antimalarials; AM = artemether; AMFm = Affordable Medicines Facility—malaria; AS = artesunate;

CI = confidence interval; DHA = dihydroartemisinin; LR = likelihood-ratio; PMVs = patent medicine vendors; QAACT = quality-assured

artemisinin combination therapy; WHO = World Health Organisation.

^a^of these 59 expired samples 58% were suspensions that were substandard, while only 8% were tablets that were degraded.

^b^adjusted for generic type, WHO prequalification, AMFm status, manufacture region, expired at time of analysis.

^c^adjusted for outlet type, provider health related qualification, generic type, AMFm status, manufacture region.

At the time of analysis approximately 10% of the samples had exceeded their expiry date, a factor also associated with poor quality. Of these 275 out of date samples, 59 (21.5%) were determined to be of poor quality (substandard, degraded and falsified). Almost a third of these out of date samples were suspensions as they were the last dose form type to be analysed due to their complexity. As a quarter of these expired suspensions contained >115% of AD and/or PD they therefore do not provide a reliable idea with regards to the relationship between drug quality and expiration unlike degraded samples. Hence, it is unlikely that the inclusion of expired samples would have substantially affected the estimate of the overall proportion of poor quality drugs found in this study.

## Discussion

Reports of the quality of antimalarial drugs are not available for the majority of malaria endemic countries [24]. This assessment of the quality of ACTs and AMTs purchased from pharmacies, patent medicine vendors and public health facilities in Enugu metropolis, Nigeria, found that 9.2% of the drugs purchased were of poor quality, with 1.2% falsified, 1.3% degraded, and 6.8% substandard in one or more of the APIs. The reliability of these findings are supported by the use of standardised sampling procedures, based on accurate contemporaneous sampling frames. The chemical analyses were corroborated in three independent laboratories. Although these results raise concerns, they are reassuring in comparison with a previous meta-analysis which concluded that 35% of 2296 antimalarial drugs, purchased mainly as a small sample size in 21 countries in Sub-Saharan Africa using the convenience method, failed chemical content analysis [24, 25].

When comparing the estimates obtained in our study using different sampling approaches the prevalence of poor quality drugs was highest for ACAs purchased using the convenience sampling approach. This yielded a significantly higher proportion of poor quality (substandard and falsified) drugs than the mystery clients and overt sampling approaches (*p*<0.05 in both cases). Though the latter mystery clients and overt approaches captured a larger number of brands, which may have been the consequence of the greater number of outlets surveyed and numbers of samples purchased Rather than the sampling methodology *per se*. Our findings draw attention to the importance of representative sampling approaches to monitor the quality of drugs accurately in a given geographic region and suggest that the prevalence of poor quality drugs in sub-Saharan Africa could be overestimated from previous reports, which were largely based on convenience sampling [24, 25].

Although direct comparison between the mystery and overt approaches is hampered by the time between the surveys and rapid pace of change in the drug markets during this two-month interval, resulting in variation both in number of outlets surveyed and variability in brands and number of samples purchased, the quality of the drugs tested did not vary from the 98 outlets visited by both approaches. As the quality of drugs purchased did not vary significantly between the mystery clients and overt approach our findings indicate that either strategy could be effectively used to determine drug quality in a geographical region. However, as each approach has its advantages and disadvantages the survey objectives must be considered to ensure that the most appropriate sampling strategy is chosen (Table 6). For example, the overt approach has the advantage of collecting additional information through the use of outlet questionnaires, enabling detailed data to collect on sources as well as risk factors for poor quality drugs, at minimal added cost. However, there is greater potential risk of sampling bias with the overt approach, if for example outlet staff are aware of which drugs may be of poor quality, and therefore either refuse to be sampled or differentially withhold some samples. The mystery clients approach has a lower inherent risk of sampling bias as outlets are unaware of the survey, and could thus be a more effective way to identify falsified products. In our study both the mystery and overt approaches yielded similar results for each classification of drug quality (acceptable; substandard; degraded and falsified) indicating that the selection bias often assumed to be the limitation of overt sampling was not a major concern in this setting. This would further suggest that vendors are not necessarily aware of which drugs may be of poor quality.

**Table 6:** Comparative strengths and weaknesses of the three sampling approaches used.

Sampling approach	Advantages	Disadvantages
***Convenience***	•Rapid •Low cost	**•Lack of defined sampling frame of standardised approach** •Uncertainty in whether sampling is representative and therefore reliability of the estimates of drug quality obtained •Generalisability of findings may be weak •Results may be difficult to replicate
***Mystery clients***	•Use of defined sampling frame •Can yield representative sample from all types of outlets and/or brands •**Low risk of sampling bias in samples collected, as outlets are unaware of survey** •Reliability and generalisability of results should be strong •Results can be replicated	•Sample will only be as comprehensive and/or representative as the sampling frame that was used •Need to authenticate and update sampling frame increases time and cost of survey •**Information on sources of poor quality drugs is limited to brand, batch and country of manufacture as stated on packaging**
***Overt***	•Use of defined sampling frame •Can yield representative sample from all types of outlets and/or brands •Results can be replicated •**Can collect additional information at minimal additional cost to mystery approach**	•Sample will only be as comprehensive and/or representative as the sampling frame that was used •Need to authenticate and update sampling frame increases time and cost of survey •Possible risk of sampling bias in samples collected, if some outlets refuse to be sampled or are aware of which samples might be poor quality and differentially withhold these •**Reliability and generalisability of results may be compromised if sampling bias occurs**

Analyses of falsified samples using both HPLC and MS techniques confirmed the absence of the stated APIs and examination of the packaging also showed them to be counterfeited e.g. most notably the packets on AMFm logoed Coartem had a longer expiry date than used by Novartis; packets of dihydroartemisinin/piperaquine manufactured in Nigeria and branded “Waipa ACT Tablets” had a missing hologram. This existence of counterfeit products is a major concern, since sick patients would be left untreated, thus representing a serious public health risk. Moreover, a number of compounds other than the stated APIs on the packaging were tentatively identified in these samples that have the potential to produce adverse effects and could be toxic. A further concern is the availability of monotherapy tablets on two counts, firstly these are not recommended by WHO for the treatment of uncomplicated *Pf* malaria; secondly, four of the 135 monotherapy samples were found to be falsified. The absence of one of the stated API in an ACT and the availability of monotherapies increases the risk of drug resistance and adverse effects [26].

Our results show that falsified drugs in this setting were most likely to be dihydroartemisinin/piperaquine tablets, and that the risk of purchasing a falsified drug was greater from a patent medicine vendor than other sources. The PMV category included drug purchased from small shops and grocery shops as well as specialised drug outlets. Our study did not however sample drugs sold by market stalls or other unlicensed traders, and given the findings above there is a potential that the risk of poor quality drugs may be greatest in this highly unregulated sector. Obtaining reliable estimates of drug quality amongst unlicensed providers is particularly challenging, hampered by factors such as lack of sampling frame, rapid turnover of outlets, providers fearing regulation and/or reprisal, staff safety and anonymity; but nevertheless should be a priority for research in countries where a major share of antimalarial treatments are sought from unregulated providers. It is reassuring that samples labelled with the AMFm logo or from WHO pre-qualified manufacturers were less likely to have quality concerns. However, recent findings in Angola where packets of Coartem bearing the AMFm logo, allegedly manufactured by Novartis, were found to be falsified together with the findings in this study reinforce the need to remain vigilant [27, 28, 29].

In conclusion, although the situation in Enugu metropolis, Nigeria was not as alarming has been reported elsewhere in Sub-Saharan Africa [24, 25, 26, 27, 28, 30]—falsified, degraded and substandard drugs were circulating in the market and highlights the need for routine monitoring of drug quality. Furthermore, whilst convenience sampling can be useful to help identify the presence of poor quality drugs and “hot spots” where these are sold, this unstandardised method may overestimate their prevalence. Our findings demonstrate that a representative sampling approach, is essential, and that either a mystery client or overt sampling approach, can be used to accurately quantify and track the scale of ineffective drugs which jeopardise treatment of a life threatening disease.

## Supporting Information

S1 Fig Map of Enugu Metropolis—Nigeria. (DOCX)

S2 Fig Bland Altman plot of the inter-laboratory comparison of all drugs analysed at CDC and LSTMH (n = 497). (DOCX)

S1 Table Solvents used to extract the active pharmaceutical ingredients (APIs) from the formulation and high-performance liquid chromatography (HPLC) conditions used for the determination. (DOCX)

S2 Table Details of falsified samples collected using convenience (CoS), mystery clients (MyS) and overt (OvS) sampling approaches in Enugu, Nigeria. (DOCX)

S1 Database Enugu Nigeria Data base. (PDF)
