# Supplementary figures and images for: Quality of Artemisinin-Based Combination Formulations for Malaria Treatment: Prevalence and Risk Factors for Poor Quality Medicines in Public Facilities and Private Sector Drug Outlets in Enugu, Nigeria
Source: PLoS One. 2015 May 27;10(5):e0125577. doi: 10.1371/journal.pone.0125577 (PMC4446036; doi:10.1371/journal.pone.0125577)

**S1 Fig. Enugu Metropolis - Nigeria.**


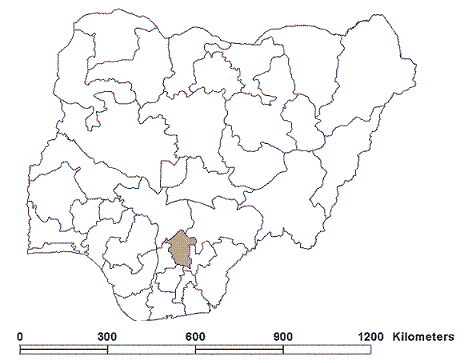

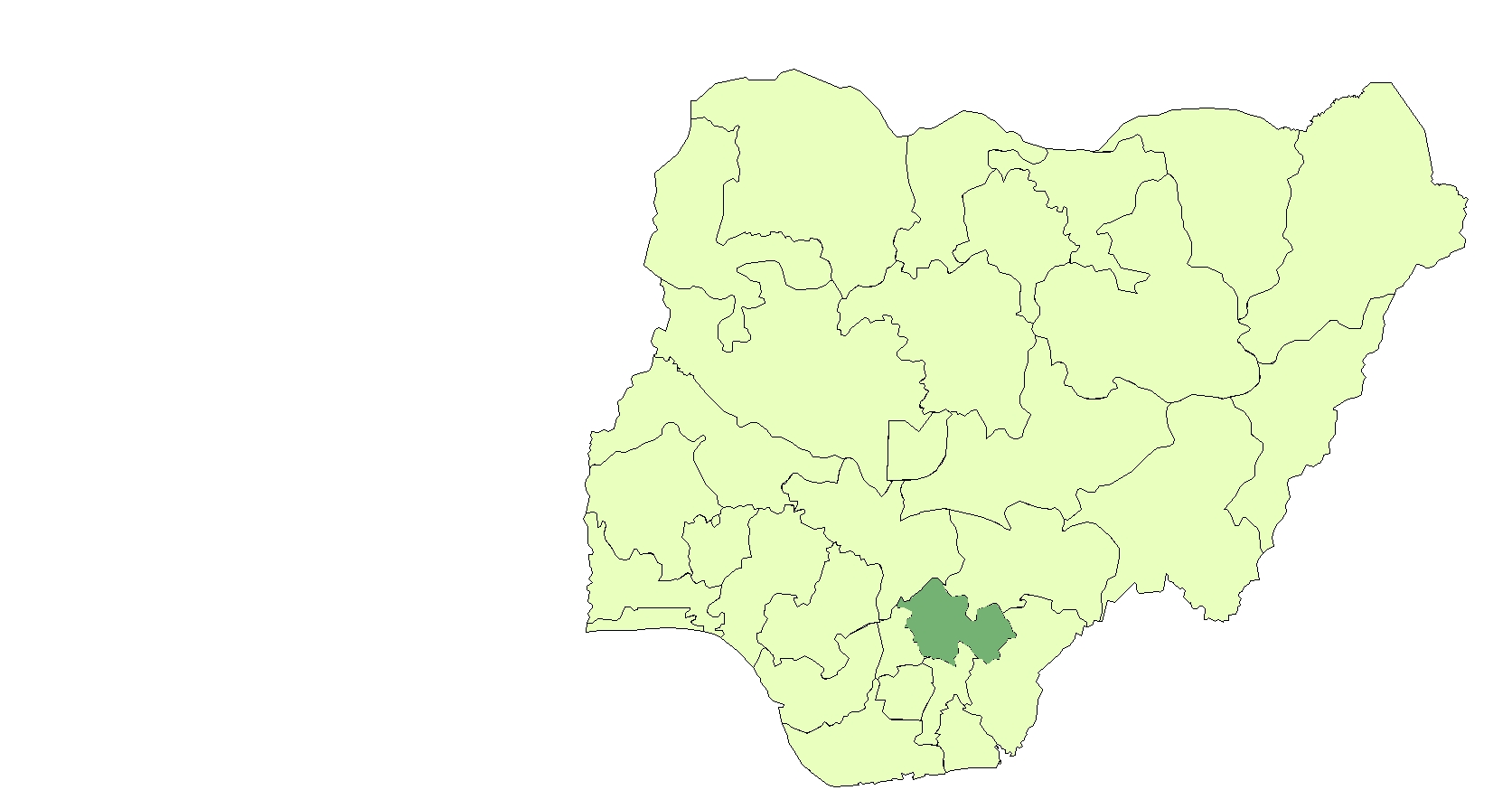


**Enugu**


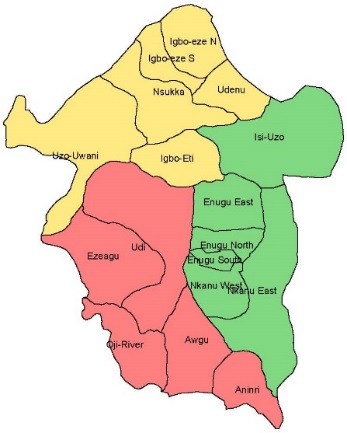

Supplement: S1 Fig — (DOCX) [file pone.0125577.s001.docx]
