# Supplementary material for: Quality of Artemisinin-Based Combination Formulations for Malaria Treatment: Prevalence and Risk Factors for Poor Quality Medicines in Public Facilities and Private Sector Drug Outlets in Enugu, Nigeria
Source: PLoS One. 2015 May 27;10(5):e0125577. doi: 10.1371/journal.pone.0125577 (PMC4446036; doi:10.1371/journal.pone.0125577)
Supplement: S2 Fig — (DOCX) [file pone.0125577.s002.docx]

**S2 Fig.** Bland Altman plot of the inter-laboratory comparison of all drugs analysed at CDC and LSTMH (n=497).

The inter-laboratory bias is 2.06% (95% CI = 1.09 to 3.03) that is the average difference of the API values between laboratories.

The upper and lower 95% limits of agreement are -21 to 25 %, an excellent agreement for independent laboratories with

distinct analytical methodologies (HPLC columns, eluents).
