# Supplementary material for: Quality of Artemisinin-Based Combination Formulations for Malaria Treatment: Prevalence and Risk Factors for Poor Quality Medicines in Public Facilities and Private Sector Drug Outlets in Enugu, Nigeria
Source: PLoS One. 2015 May 27;10(5):e0125577. doi: 10.1371/journal.pone.0125577 (PMC4446036; doi:10.1371/journal.pone.0125577)
Supplement: S1 Table — (DOCX) [file pone.0125577.s003.docx]

**S1 Table. Solvents used to extract the active pharmaceutical ingredients (APIs) from the formulation and high-performance liquid chromatography (HPLC) conditions used for the determination.**

| **Formulation** | **Solvent for API extraction** | **Injected sample concentration (mg/ml)** | **Column** | **Wavelength (nm)** |
| --- | --- | --- | --- | --- |
| ART-PIP | ART = MeOH  PIP = MeOH/0.1 M HCl (1:1; v/v) | ART = 20  PIP = 0.4 | Genesis AQ 4 µm  Genesis AQ 4 µm | ART = 275  PIP = 350 |
| AS | AS = MeOH | AS= 10 | Genesis AQ 4 µm | AS = 204 |
| AM-LUM | AM = MeOH  LUM = 10% acetic acid in MeOH | AM = 10  LUM = 0.6 | Dionex Acclaim  Dionex Acclaim | AM = 204  LUM = 275 |
| AS-ADQ | AS = MeOH | AS = 10  ADQ = 0.6 | Genesis AQ 4 µm  Genesis AQ 4 µm | AS = 204 |
|  | ADQ = MeOH |  |  | ADQ = 360 |
| AS-MEF | AS = MeOH  MEF = MeOH/2.0 N HCl (80:20; v/v) | AS = 10  MEF = 0.5 | Genesis AQ 4 µm  Genesis AQ 4 µm | AS = 204  MEF = 275 |
| AS-SULMEX-PYR | AS = MeOH  SULMEX-PYR = MeOH | AS = 10  SULMEX-PYR = 0.5 & 0.25 | Genesis AQ 4 µm  Genesis AQ 4 µm | AS = 204  SULMEX-PYR = 275 |
| AS-SULDOX-PYR | AS = MeOH  SULDOX-PYR = MeOH | AS = 10  SULDOX-PYR = 0.5 & 0.25 | Genesis AQ 4 µm  Genesis AQ 4 µm | AS = 204  SULDOX-PYR = 275 |
| DHA-PIP | DHA = MeOH  PIP = MeOH/0.1 M HCl (1:1; v/v) | DHA = 5  PIP = 0.4 | Genesis AQ 4 µm  Genesis AQ 4 µm | DHA = 193  PIP = 350 |

ADQ = amodiaquine dichlorodihydrate; AM = artemether; ART = artemisinin; AS = artesunate; DHA = dihydroartemisinin;

HCl = hydrochloric acid; LUM = lumefantrine; MeOH = methanol; MEF = mefloquine; PIP = piperaquine; PYR = pyrimethamine;

SULDOX = sulfadoxine; SULMEX = sulfamethoxypyridazine.

Reference standards of ART, AS, AM, DHA, ADQ and PYR were purchased from Sigma Aldich, UK. LUM was purchased from WHO, Switzerland.

MEF and SULDOX were a gift from Roche, Basle, Switzerland, and SULMEX was provided by Dafra PHARMA, Belgium.
