# Supplementary material for: Quality of Artemisinin-Based Combination Formulations for Malaria Treatment: Prevalence and Risk Factors for Poor Quality Medicines in Public Facilities and Private Sector Drug Outlets in Enugu, Nigeria
Source: PLoS One. 2015 May 27;10(5):e0125577. doi: 10.1371/journal.pone.0125577 (PMC4446036; doi:10.1371/journal.pone.0125577)
Supplement: S2 Table — (DOCX) [file pone.0125577.s004.docx]

**S2 Table.** Details of falsified samples collected using convenience (CoS), mystery clients’ (MyS) and overt (OvS) sampling approaches in

Enugu, Nigeria.

| **Stated brand** | **Stated country of manufacture** | **Stated**  **manufacturer** | **Manufacture**  **and expiry date** | | | **Batch**  **number** | | **Number of samples and sampling approach** | **Stated**  **API** | **Compound found** |
| --- | --- | --- | --- | --- | --- | --- | --- | --- | --- | --- |
| Artesunat® | Vietnam | Mekophar | Sep 10 - Aug 13 | | | 07015FX | | 1 CoS; 1 MyS | AS | DEHA or DOA |
| Artesunat® | Vietnam | Mekophar | Jul 11 - Jul 14 | | | 07015FX | | 1 MyS | AS | DEHA or DOA |
| Artesunat® | Vietnam | Mekophar | Jul 09 - Jul 12 | | | 09015FX | | 1 MyS | AS | acetaminophen |
| Artesmequine® | China | Greenfield | Jul 09 - Jul 12 | | | 090701 | | 1 CoS | AS-MEF | Unidentified |
| Coartem**®** (USA) | USA | Novartis | Mar 11 - Feb 13 | | | F1901 | | 1 CoS | AM-LUM | chlorzoxazone |
| Coartem**®** (USA) | USA | Novartis | Jan 12 - Jan 14 | | | F1901 | | 1 MyS | AM-LUM | chlorzoxazone |
| Coartem**®** (USA) | USA | Novartis | Jan 12 - Jan 14 | | | F2261* | | 2 MyS; 2 OvS | AM-LUM | chlorzoxazone |
| Lonart®-DS | India | Bliss GVS | Mar 11 - Feb 13 | | | LD-189 | | 1 CoS; 2 MyS | AM-LUM | ciprofloxacin |
| Lonart®-DS | India | Bliss GVS | Nov 11 - Dec 13 | | | LD-189 | | 1 MyS | AM-LUM | ciprofloxacin |
| Duo-Cotecxin ® | China | Zheijang Holley | Feb 11 - Jan 14 | | | 151109 | | 1 MyS | DHA-PIP | DEHA or DOA |
| Waipa Act | Nigeria | Kunimed | Nov 10 - Oct 14 | | | XMW46 | | 2 CoS; 14 MyS; 3OvS | DHA-PIP | acetaminophen |
| **Total number of falsified samples: 35 (6 CoS; 24 MyS; 5 OvS)** | | | |  |  | |  |  | | |

*Note:* AM = artemether; AS = artesunate; DEHA = *bis* (2-ethylhexyl) adipate; DHA = dihydroartemisinin; DOA = dioctyl adipate; LUM = lumefantrine; MEF = mefloquine; PIP = piperaquine; USA = United States of America.

*samples with the Affordable Medicines Facility - malaria (AMFm) logo
