# Supplementary material for: Quality of Artemisinin-Based Combination Formulations for Malaria Treatment: Prevalence and Risk Factors for Poor Quality Medicines in Public Facilities and Private Sector Drug Outlets in Enugu, Nigeria
Source: PLoS One. 2015 May 27;10(5):e0125577. doi: 10.1371/journal.pone.0125577 (PMC4446036; doi:10.1371/journal.pone.0125577)
Supplement: S1 Database — (PDF) [file pone.0125577.s005.pdf]

| Sampling | country | dateofpurchase | interviewcode | districtcode | wardcode |
|---|---|---|---|---|---|
| Convenience | Nigeria (Enugu) | 01/06/2012 | 1 | 2 | 8 |
| Convenience | Nigeria (Enugu) | 06/06/2012 | 1 | 2 | 6 |
| Convenience | Nigeria (Enugu) | 12/06/2012 | 1 | 3 | 11 |
| Convenience | Nigeria (Enugu) | 11/06/2012 | 1 | 1 | 1 |
| Convenience | Nigeria (Enugu) | 05/06/2012 | 1 | 1 | 2 |
| Convenience | Nigeria (Enugu) | 05/06/2012 | 1 | 1 | 1 |
| Convenience | Nigeria (Enugu) | 05/06/2012 | 1 | 1 | 2 |
| Convenience | Nigeria (Enugu) | 06/06/2012 | 1 | 2 | 6 |
| Convenience | Nigeria (Enugu) | 05/06/2012 | 1 | 1 | 2 |
| Convenience | Nigeria (Enugu) | 11/06/2012 | 1 | 3 | 10 |
| Convenience | Nigeria (Enugu) | 01/06/2012 | 1 | 2 | 8 |
| Convenience | Nigeria (Enugu) | 06/06/2012 | 1 | 2 | 6 |
| Convenience | Nigeria (Enugu) | 11/06/2012 | 1 | 2 | 9 |
| Convenience | Nigeria (Enugu) | 11/06/2012 | 1 | 2 | 9 |
| Convenience | Nigeria (Enugu) | 11/06/2012 | 1 | 1 | 1 |
| Convenience | Nigeria (Enugu) | 07/06/2012 | 1 | 1 | 3 |
| Convenience | Nigeria (Enugu) | 05/06/2012 | 1 | 1 | 2 |
| Convenience | Nigeria (Enugu) | 05/06/2012 | 1 | 1 | 1 |
| Convenience | Nigeria (Enugu) | 06/06/2012 | 1 | 2 | 5 |
| Convenience | Nigeria (Enugu) | 06/06/2012 | 1 | 2 | 6 |
| Convenience | Nigeria (Enugu) | 11/06/2012 | 1 | 2 | 9 |
| Convenience | Nigeria (Enugu) | 07/06/2012 | 1 | 1 | 3 |
| Convenience | Nigeria (Enugu) | 05/06/2012 | 1 | 1 | 2 |
| Convenience | Nigeria (Enugu) | 05/06/2012 | 1 | 1 | 2 |
| Convenience | Nigeria (Enugu) | 05/06/2012 | 1 | 1 | 1 |
| Convenience | Nigeria (Enugu) | 07/06/2012 | 1 | 2 | 7 |
| Convenience | Nigeria (Enugu) | 12/06/2012 | 1 | 3 | 13 |
| Convenience | Nigeria (Enugu) | 13/06/2012 | 1 | 3 | 12 |
| Convenience | Nigeria (Enugu) | 07/06/2012 | 1 | 2 | 8 |
| Convenience | Nigeria (Enugu) | 14/06/2012 | 1 | 1 | 0 |
| Convenience | Nigeria (Enugu) | 06/06/2012 | 1 | 2 | 6 |
| Convenience | Nigeria (Enugu) | 07/06/2012 | 1 | 2 | 7 |
| Convenience | Nigeria (Enugu) | 11/06/2012 | 1 | 3 | 16 |
| Convenience | Nigeria (Enugu) | 13/06/2012 | 1 | 3 | 15 |
| Convenience | Nigeria (Enugu) | 11/06/2012 | 1 | 3 | 10 |
| Convenience | Nigeria (Enugu) | 11/06/2012 | 1 | 3 | 10 |
| Convenience | Nigeria (Enugu) | 12/06/2012 | 1 | 3 | 11 |
| Convenience | Nigeria (Enugu) | 11/06/2012 | 1 | 1 | 1 |
| Convenience | Nigeria (Enugu) | 12/06/2012 | 1 | 3 | 13 |
| Convenience | Nigeria (Enugu) | 11/06/2012 | 1 | 3 | 16 |
| Convenience | Nigeria (Enugu) | 13/06/2012 | 1 | 3 | 15 |
| Convenience | Nigeria (Enugu) | 06/06/2012 | 1 | 2 | 5 |
| Convenience | Nigeria (Enugu) | 13/06/2012 | 1 | 1 | 0 |
| Convenience | Nigeria (Enugu) | 14/06/2012 | 1 | 1 | 0 |
| Convenience | Nigeria (Enugu) | 07/06/2012 | 1 | 2 | 7 |
| Convenience | Nigeria (Enugu) | 07/06/2012 | 1 | 2 | 4 |

| | | | | | |
|---|---|---|---|---|---|
| Convenience | Nigeria (Enugu) | 05/06/2012 | 1 | 1 | 1 |
| Convenience | Nigeria (Enugu) | 06/06/2012 | 1 | 2 | 6 |
| Convenience | Nigeria (Enugu) | 11/06/2012 | 1 | 2 | 9 |
| Convenience | Nigeria (Enugu) | 07/06/2012 | 1 | 1 | 3 |
| Convenience | Nigeria (Enugu) | 05/06/2012 | 1 | 1 | 1 |
| Convenience | Nigeria (Enugu) | 07/06/2012 | 1 | 2 | 4 |
| Convenience | Nigeria (Enugu) | 12/06/2012 | 1 | 3 | 14 |
| Convenience | Nigeria (Enugu) | 11/06/2012 | 1 | 3 | 16 |
| Convenience | Nigeria (Enugu) | 11/06/2012 | 1 | 3 | 10 |
| Convenience | Nigeria (Enugu) | 01/06/2012 | 1 | 2 | 8 |
| Convenience | Nigeria (Enugu) | 05/06/2012 | 1 | 1 | 2 |
| Convenience | Nigeria (Enugu) | 06/06/2012 | 1 | 2 | 6 |
| Convenience | Nigeria (Enugu) | 01/06/2012 | 1 | 2 | 8 |
| Convenience | Nigeria (Enugu) | 06/06/2012 | 1 | 2 | 6 |
| Convenience | Nigeria (Enugu) | 07/06/2012 | 1 | 2 | 8 |
| Convenience | Nigeria (Enugu) | 06/06/2012 | 1 | 2 | 5 |
| Convenience | Nigeria (Enugu) | 01/06/2012 | 1 | 2 | 8 |
| Convenience | Nigeria (Enugu) | 11/06/2012 | 1 | 1 | 1 |
| Convenience | Nigeria (Enugu) | 07/06/2012 | 1 | 2 | 4 |
| Convenience | Nigeria (Enugu) | 06/06/2012 | 1 | 2 | 5 |
| Convenience | Nigeria (Enugu) | 01/06/2012 | 1 | 2 | 8 |
| Convenience | Nigeria (Enugu) | 11/06/2012 | 1 | 1 | 1 |
| Convenience | Nigeria (Enugu) | 07/06/2012 | 1 | 1 | 3 |
| Convenience | Nigeria (Enugu) | 07/06/2012 | 1 | 2 | 8 |
| Convenience | Nigeria (Enugu) | 01/06/2012 | 1 | 2 | 8 |
| Convenience | Nigeria (Enugu) | 07/06/2012 | 1 | 1 | 3 |
| Convenience | Nigeria (Enugu) | 01/06/2012 | 1 | 2 | 8 |
| Convenience | Nigeria (Enugu) | 06/06/2012 | 1 | 2 | 6 |
| Convenience | Nigeria (Enugu) | 11/06/2012 | 1 | 2 | 9 |
| Convenience | Nigeria (Enugu) | 11/06/2012 | 1 | 3 | 10 |
| Convenience | Nigeria (Enugu) | 06/06/2012 | 1 | 2 | 5 |
| Convenience | Nigeria (Enugu) | 01/06/2012 | 1 | 2 | 8 |
| Convenience | Nigeria (Enugu) | 06/06/2012 | 1 | 2 | 6 |
| Convenience | Nigeria (Enugu) | 13/06/2012 | 1 | 3 | 15 |
| Convenience | Nigeria (Enugu) | 01/06/2012 | 1 | 2 | 8 |
| Convenience | Nigeria (Enugu) | 06/06/2012 | 1 | 2 | 6 |
| Convenience | Nigeria (Enugu) | 01/06/2012 | 1 | 2 | 8 |
| Convenience | Nigeria (Enugu) | 06/06/2012 | 1 | 2 | 6 |
| Convenience | Nigeria (Enugu) | 11/06/2012 | 1 | 2 | 9 |
| Convenience | Nigeria (Enugu) | 11/06/2012 | 1 | 2 | 9 |
| Convenience | Nigeria (Enugu) | 12/06/2012 | 1 | 3 | 14 |
| Convenience | Nigeria (Enugu) | 01/06/2012 | 1 | 2 | 8 |
| Convenience | Nigeria (Enugu) | 06/06/2012 | 1 | 2 | 6 |
| Convenience | Nigeria (Enugu) | 11/06/2012 | 1 | 2 | 9 |
| Convenience | Nigeria (Enugu) | 12/06/2012 | 1 | 3 | 11 |
| Convenience | Nigeria (Enugu) | 11/06/2012 | 1 | 1 | 1 |
| Convenience | Nigeria (Enugu) | 07/06/2012 | 1 | 1 | 3 |
| Convenience | Nigeria (Enugu) | 05/06/2012 | 1 | 1 | 2 |
| Convenience | Nigeria (Enugu) | 05/06/2012 | 1 | 1 | 2 |
| Convenience | Nigeria (Enugu) | 05/06/2012 | 1 | 1 | 1 |

| | | | | | |
|---|---|---|---|---|---|
| Convenience | Nigeria (Enugu) | 07/06/2012 | 1 | 2 | 7 |
| Convenience | Nigeria (Enugu) | 12/06/2012 | 1 | 3 | 14 |
| Convenience | Nigeria (Enugu) | 07/06/2012 | 1 | 2 | 8 |
| Convenience | Nigeria (Enugu) | 11/06/2012 | 1 | 3 | 10 |
| Convenience | Nigeria (Enugu) | 06/06/2012 | 1 | 2 | 5 |
| Convenience | Nigeria (Enugu) | 14/06/2012 | 1 | 1 | 0 |
| Convenience | Nigeria (Enugu) | 01/06/2012 | 1 | 2 | 8 |
| Convenience | Nigeria (Enugu) | 06/06/2012 | 1 | 2 | 6 |
| Convenience | Nigeria (Enugu) | 11/06/2012 | 1 | 1 | 1 |
| Convenience | Nigeria (Enugu) | 11/06/2012 | 1 | 3 | 10 |
| Convenience | Nigeria (Enugu) | 06/06/2012 | 1 | 2 | 6 |
| Convenience | Nigeria (Enugu) | 01/06/2012 | 1 | 2 | 8 |
| Convenience | Nigeria (Enugu) | 06/06/2012 | 1 | 2 | 6 |
| Convenience | Nigeria (Enugu) | 05/06/2012 | 1 | 1 | 2 |
| Convenience | Nigeria (Enugu) | 07/06/2012 | 1 | 2 | 8 |
| Convenience | Nigeria (Enugu) | 11/06/2012 | 1 | 3 | 10 |
| Convenience | Nigeria (Enugu) | 06/06/2012 | 1 | 2 | 5 |
| Convenience | Nigeria (Enugu) | 14/06/2012 | 1 | 1 | 0 |
| Convenience | Nigeria (Enugu) | 01/06/2012 | 1 | 2 | 8 |
| Convenience | Nigeria (Enugu) | 06/06/2012 | 1 | 2 | 6 |
| Convenience | Nigeria (Enugu) | 11/06/2012 | 1 | 2 | 9 |
| Convenience | Nigeria (Enugu) | 05/06/2012 | 1 | 1 | 2 |
| Convenience | Nigeria (Enugu) | 01/06/2012 | 1 | 2 | 8 |
| Convenience | Nigeria (Enugu) | 06/06/2012 | 1 | 2 | 6 |
| Convenience | Nigeria (Enugu) | 11/06/2012 | 1 | 2 | 9 |
| Convenience | Nigeria (Enugu) | 07/06/2012 | 1 | 1 | 3 |
| Convenience | Nigeria (Enugu) | 05/06/2012 | 1 | 1 | 2 |
| Convenience | Nigeria (Enugu) | 07/06/2012 | 1 | 2 | 8 |
| Convenience | Nigeria (Enugu) | 11/06/2012 | 1 | 3 | 10 |
| Convenience | Nigeria (Enugu) | 11/06/2012 | 1 | 1 | 1 |
| Convenience | Nigeria (Enugu) | 13/06/2012 | 1 | 3 | 12 |
| Convenience | Nigeria (Enugu) | 11/06/2012 | 1 | 3 | 16 |
| Convenience | Nigeria (Enugu) | 06/06/2012 | 1 | 2 | 5 |
| Convenience | Nigeria (Enugu) | 06/06/2012 | 1 | 2 | 5 |
| Convenience | Nigeria (Enugu) | 12/06/2012 | 1 | 3 | 11 |
| Convenience | Nigeria (Enugu) | 12/06/2012 | 1 | 3 | 13 |
| Convenience | Nigeria (Enugu) | 11/06/2012 | 1 | 3 | 10 |
| Convenience | Nigeria (Enugu) | 11/06/2012 | 1 | 3 | 16 |
| Convenience | Nigeria (Enugu) | 12/06/2012 | 1 | 3 | 13 |
| Convenience | Nigeria (Enugu) | 07/06/2012 | 1 | 1 | 3 |
| Convenience | Nigeria (Enugu) | 05/06/2012 | 1 | 1 | 2 |
| Convenience | Nigeria (Enugu) | 07/06/2012 | 1 | 2 | 7 |
| Convenience | Nigeria (Enugu) | 12/06/2012 | 1 | 3 | 14 |
| Convenience | Nigeria (Enugu) | 06/06/2012 | 1 | 2 | 6 |
| Convenience | Nigeria (Enugu) | 11/06/2012 | 1 | 3 | 10 |
| Convenience | Nigeria (Enugu) | 13/06/2012 | 1 | 1 | 0 |
| Convenience | Nigeria (Enugu) | 14/06/2012 | 1 | 1 | 0 |
| Convenience | Nigeria (Enugu) | 01/06/2012 | 1 | 2 | 8 |
| Convenience | Nigeria (Enugu) | 06/06/2012 | 1 | 2 | 6 |

| | | | | | |
|---|---|---|---|---|---|
| Convenience | Nigeria (Enugu) | 11/06/2012 | 1 | 2 | 9 |
| Convenience | Nigeria (Enugu) | 05/06/2012 | 1 | 1 | 2 |
| Convenience | Nigeria (Enugu) | 05/06/2012 | 1 | 1 | 1 |
| Convenience | Nigeria (Enugu) | 07/06/2012 | 1 | 2 | 4 |
| Convenience | Nigeria (Enugu) | 01/06/2012 | 1 | 2 | 8 |
| Convenience | Nigeria (Enugu) | 13/06/2012 | 1 | 1 | 0 |
| Convenience | Nigeria (Enugu) | 07/06/2012 | 1 | 2 | 4 |
| Convenience | Nigeria (Enugu) | 06/06/2012 | 1 | 2 | 6 |
| Convenience | Nigeria (Enugu) | 07/06/2012 | 1 | 2 | 8 |
| Convenience | Nigeria (Enugu) | 07/06/2012 | 1 | 1 | 3 |
| Convenience | Nigeria (Enugu) | 05/06/2012 | 1 | 1 | 2 |
| Convenience | Nigeria (Enugu) | 01/06/2012 | 1 | 2 | 8 |
| Convenience | Nigeria (Enugu) | 07/06/2012 | 1 | 2 | 4 |
| Convenience | Nigeria (Enugu) | 11/06/2012 | 1 | 3 | 10 |
| Convenience | Nigeria (Enugu) | 01/06/2012 | 1 | 2 | 8 |
| Convenience | Nigeria (Enugu) | 11/06/2012 | 1 | 2 | 9 |
| Convenience | Nigeria (Enugu) | 11/06/2012 | 1 | 2 | 9 |
| Convenience | Nigeria (Enugu) | 07/06/2012 | 1 | 1 | 3 |
| Convenience | Nigeria (Enugu) | 11/06/2012 | 1 | 3 | 10 |
| Convenience | Nigeria (Enugu) | 06/06/2012 | 1 | 2 | 5 |
| Convenience | Nigeria (Enugu) | 13/06/2012 | 1 | 3 | 16 |
| Convenience | Nigeria (Enugu) | 01/06/2012 | 1 | 2 | 8 |
| Convenience | Nigeria (Enugu) | 01/06/2012 | 1 | 2 | 8 |
| Convenience | Nigeria (Enugu) | 01/06/2012 | 1 | 2 | 8 |
| Convenience | Nigeria (Enugu) | 07/06/2012 | 1 | 1 | 3 |
| Convenience | Nigeria (Enugu) | 01/06/2012 | 1 | 2 | 8 |
| Convenience | Nigeria (Enugu) | 06/06/2012 | 1 | 2 | 6 |
| Convenience | Nigeria (Enugu) | 07/06/2012 | 1 | 1 | 3 |
| Convenience | Nigeria (Enugu) | 11/06/2012 | 1 | 3 | 10 |
| Convenience | Nigeria (Enugu) | 13/06/2012 | 1 | 3 | 16 |
| Convenience | Nigeria (Enugu) | 01/06/2012 | 1 | 2 | 8 |
| Convenience | Nigeria (Enugu) | 06/06/2012 | 1 | 2 | 6 |
| Convenience | Nigeria (Enugu) | 12/06/2012 | 1 | 3 | 11 |
| Convenience | Nigeria (Enugu) | 11/06/2012 | 1 | 2 | 9 |
| Convenience | Nigeria (Enugu) | 11/06/2012 | 1 | 1 | 1 |
| Convenience | Nigeria (Enugu) | 07/06/2012 | 1 | 1 | 3 |
| Convenience | Nigeria (Enugu) | 05/06/2012 | 1 | 1 | 2 |
| Convenience | Nigeria (Enugu) | 05/06/2012 | 1 | 1 | 2 |
| Convenience | Nigeria (Enugu) | 07/06/2012 | 1 | 2 | 7 |
| Convenience | Nigeria (Enugu) | 12/06/2012 | 1 | 3 | 14 |
| Convenience | Nigeria (Enugu) | 12/06/2012 | 1 | 3 | 13 |
| Convenience | Nigeria (Enugu) | 13/06/2012 | 1 | 3 | 12 |
| Convenience | Nigeria (Enugu) | 11/06/2012 | 1 | 3 | 16 |
| Convenience | Nigeria (Enugu) | 06/06/2012 | 1 | 2 | 5 |
| Convenience | Nigeria (Enugu) | 14/06/2012 | 1 | 1 | 0 |
| Convenience | Nigeria (Enugu) | 01/06/2012 | 1 | 2 | 8 |
| Convenience | Nigeria (Enugu) | 06/06/2012 | 1 | 2 | 6 |
| Convenience | Nigeria (Enugu) | 11/06/2012 | 1 | 2 | 9 |

| | | | | | |
|---|---|---|---|---|---|
| Convenience | Nigeria (Enugu) | 11/06/2012 | 1 | 1 | 1 |
| Convenience | Nigeria (Enugu) | 07/06/2012 | 1 | 1 | 3 |
| Convenience | Nigeria (Enugu) | 05/06/2012 | 1 | 1 | 1 |
| Convenience | Nigeria (Enugu) | 11/06/2012 | 1 | 3 | 16 |
| Convenience | Nigeria (Enugu) | 07/06/2012 | 1 | 2 | 8 |
| Convenience | Nigeria (Enugu) | 01/06/2012 | 1 | 2 | 8 |
| Convenience | Nigeria (Enugu) | 05/06/2012 | 1 | 1 | 2 |

| Sampling meth | country | interviewcode | districtcode | wardcode | outletcode |
|---|---|---|---|---|---|
| mystery | Nigeria (Enugu) | 1 | 3 | 1 | 57 |
| mystery | Nigeria (Enugu) | 5 | 1 | 2 | 17 |
| mystery | Nigeria (Enugu) | 15 | 2 | 3 | 34 |
| mystery | Nigeria (Enugu) | 1 | 3 | 1 | 4 |
| mystery | Nigeria (Enugu) | 1 | 3 | 1 | 21 |
| mystery | Nigeria (Enugu) | 1 | 3 | 1 | 80 |
| mystery | Nigeria (Enugu) | 2 | 3 | 5 | 7 |
| mystery | Nigeria (Enugu) | 2 | 3 | 5 | 29 |
| mystery | Nigeria (Enugu) | 3 | 2 | 1 | 2 |
| mystery | Nigeria (Enugu) | 3 | 2 | 4 | 42 |
| mystery | Nigeria (Enugu) | 4 | 1 | 2 | 12 |
| mystery | Nigeria (Enugu) | 5 | 1 | 2 | 0 |
| mystery | Nigeria (Enugu) | 5 | 1 | 2 | 21 |
| mystery | Nigeria (Enugu) | 5 | 1 | 2 | 30 |
| mystery | Nigeria (Enugu) | 5 | 1 | 2 | 31 |
| mystery | Nigeria (Enugu) | 5 | 1 | 2 | 46 |
| mystery | Nigeria (Enugu) | 5 | 1 | 2 | 49 |
| mystery | Nigeria (Enugu) | 5 | 2 | 1 | 0 |
| mystery | Nigeria (Enugu) | 6 | 2 | 1 | 26 |
| mystery | Nigeria (Enugu) | 6 | 2 | 1 | 28 |
| mystery | Nigeria (Enugu) | 6 | 2 | 1 | 28 |
| mystery | Nigeria (Enugu) | 6 | 2 | 3 | 6 |
| mystery | Nigeria (Enugu) | 6 | 2 | 3 | 39 |
| mystery | Nigeria (Enugu) | 7 | 2 | 4 | 20 |
| mystery | Nigeria (Enugu) | 7 | 3 | 5 | 0 |
| mystery | Nigeria (Enugu) | 8 | 2 | 1 | 0 |
| mystery | Nigeria (Enugu) | 8 | 2 | 4 | 44 |
| mystery | Nigeria (Enugu) | 9 | 2 | 1 | 0 |
| mystery | Nigeria (Enugu) | 9 | 2 | 2 | 10 |
| mystery | Nigeria (Enugu) | 10 | 3 | 1 | 18 |
| mystery | Nigeria (Enugu) | 10 | 3 | 1 | 31 |
| mystery | Nigeria (Enugu) | 10 | 3 | 1 | 53 |
| mystery | Nigeria (Enugu) | 10 | 3 | 1 | 65 |
| mystery | Nigeria (Enugu) | 11 | 2 | 5 | 2 |
| mystery | Nigeria (Enugu) | 12 | 2 | 5 | 24 |
| mystery | Nigeria (Enugu) | 12 | 3 | 7 | 18 |
| mystery | Nigeria (Enugu) | 13 | 2 | 1 | 0 |
| mystery | Nigeria (Enugu) | 14 | 1 | 1 | 7 |
| mystery | Nigeria (Enugu) | 14 | 1 | 1 | 7 |

| | | | | | |
|---|---|---|---|---|---|
| mystery | Nigeria (Enugu) | 14 | 1 | 1 | 18 |
| mystery | Nigeria (Enugu) | 14 | 1 | 3 | 5 |
| mystery | Nigeria (Enugu) | 14 | 1 | 3 | 10 |
| mystery | Nigeria (Enugu) | 14 | 1 | 3 | 23 |
| mystery | Nigeria (Enugu) | 15 | 2 | 1 | 0 |
| mystery | Nigeria (Enugu) | 16 | 1 | 1 | 7 |
| mystery | Nigeria (Enugu) | 16 | 2 | 3 | 0 |
| mystery | Nigeria (Enugu) | 1 | 3 | 1 | 21 |
| mystery | Nigeria (Enugu) | 2 | 3 | 5 | 28 |
| mystery | Nigeria (Enugu) | 2 | 3 | 5 | 29 |
| mystery | Nigeria (Enugu) | 3 | 3 | 71 | 0 |
| mystery | Nigeria (Enugu) | 4 | 1 | 2 | 5 |
| mystery | Nigeria (Enugu) | 4 | 1 | 2 | 9 |
| mystery | Nigeria (Enugu) | 8 | 2 | 1 | 0 |
| mystery | Nigeria (Enugu) | 9 | 2 | 1 | 0 |
| mystery | Nigeria (Enugu) | 10 | 3 | 1 | 25 |
| mystery | Nigeria (Enugu) | 10 | 3 | 1 | 31 |
| mystery | Nigeria (Enugu) | 10 | 3 | 1 | 65 |
| mystery | Nigeria (Enugu) | 11 | 2 | 5 | 26 |
| mystery | Nigeria (Enugu) | 12 | 2 | 5 | 24 |
| mystery | Nigeria (Enugu) | 13 | 2 | 1 | 0 |
| mystery | Nigeria (Enugu) | 15 | 2 | 1 | 0 |
| mystery | Nigeria (Enugu) | 16 | 2 | 3 | 0 |
| mystery | Nigeria (Enugu) | 0 | 0 | 0 | 0 |
| mystery | Nigeria (Enugu) | 1 | 3 | 1 | 4 |
| mystery | Nigeria (Enugu) | 1 | 3 | 1 | 57 |
| mystery | Nigeria (Enugu) | 2 | 3 | 2 | 5 |
| mystery | Nigeria (Enugu) | 2 | 3 | 2 | 7 |
| mystery | Nigeria (Enugu) | 2 | 3 | 2 | 7 |
| mystery | Nigeria (Enugu) | 2 | 3 | 2 | 8 |
| mystery | Nigeria (Enugu) | 2 | 3 | 5 | 7 |
| mystery | Nigeria (Enugu) | 2 | 3 | 5 | 34 |
| mystery | Nigeria (Enugu) | 3 | 2 | 4 | 42 |
| mystery | Nigeria (Enugu) | 4 | 1 | 2 | 38 |
| mystery | Nigeria (Enugu) | 5 | 1 | 2 | 0 |
| mystery | Nigeria (Enugu) | 5 | 1 | 2 | 2 |
| mystery | Nigeria (Enugu) | 5 | 1 | 2 | 21 |
| mystery | Nigeria (Enugu) | 5 | 1 | 2 | 22 |
| mystery | Nigeria (Enugu) | 5 | 2 | 1 | 0 |
| mystery | Nigeria (Enugu) | 6 | 2 | 1 | 28 |
| mystery | Nigeria (Enugu) | 6 | 2 | 3 | 1 |
| mystery | Nigeria (Enugu) | 6 | 2 | 3 | 14 |
| mystery | Nigeria (Enugu) | 6 | 2 | 3 | 39 |
| mystery | Nigeria (Enugu) | 7 | 2 | 4 | 20 |
| mystery | Nigeria (Enugu) | 7 | 2 | 4 | 22 |
| mystery | Nigeria (Enugu) | 7 | 2 | 4 | 36 |
| mystery | Nigeria (Enugu) | 7 | 3 | 5 | 0 |
| mystery | Nigeria (Enugu) | 8 | 2 | 1 | 0 |
| mystery | Nigeria (Enugu) | 9 | 2 | 1 | 0 |
| mystery | Nigeria (Enugu) | 9 | 2 | 2 | 10 |

| | | | | | |
|---|---|---|---|---|---|
| mystery | Nigeria (Enugu) | 9 | 2 | 5 | 11 |
| mystery | Nigeria (Enugu) | 9 | 3 | 3 | 3 |
| mystery | Nigeria (Enugu) | 10 | 3 | 1 | 18 |
| mystery | Nigeria (Enugu) | 10 | 3 | 1 | 21 |
| mystery | Nigeria (Enugu) | 10 | 3 | 1 | 31 |
| mystery | Nigeria (Enugu) | 10 | 3 | 1 | 33 |
| mystery | Nigeria (Enugu) | 10 | 3 | 1 | 65 |
| mystery | Nigeria (Enugu) | 13 | 2 | 1 | 0 |
| mystery | Nigeria (Enugu) | 14 | 1 | 1 | 5 |
| mystery | Nigeria (Enugu) | 14 | 1 | 1 | 6 |
| mystery | Nigeria (Enugu) | 14 | 1 | 1 | 16 |
| mystery | Nigeria (Enugu) | 15 | 2 | 1 | 0 |
| mystery | Nigeria (Enugu) | 16 | 1 | 1 | 7 |
| mystery | Nigeria (Enugu) | 16 | 1 | 3 | 30 |
| mystery | Nigeria (Enugu) | 16 | 2 | 3 | 0 |
| mystery | Nigeria (Enugu) | 1 | 3 | 1 | 10 |
| mystery | Nigeria (Enugu) | 1 | 3 | 1 | 33 |
| mystery | Nigeria (Enugu) | 1 | 3 | 1 | 42 |
| mystery | Nigeria (Enugu) | 1 | 3 | 1 | 57 |
| mystery | Nigeria (Enugu) | 1 | 3 | 1 | 60 |
| mystery | Nigeria (Enugu) | 1 | 3 | 1 | 63 |
| mystery | Nigeria (Enugu) | 1 | 3 | 1 | 69 |
| mystery | Nigeria (Enugu) | 1 | 3 | 1 | 76 |
| mystery | Nigeria (Enugu) | 2 | 3 | 2 | 5 |
| mystery | Nigeria (Enugu) | 2 | 3 | 2 | 6 |
| mystery | Nigeria (Enugu) | 2 | 3 | 5 | 33 |
| mystery | Nigeria (Enugu) | 3 | 2 | 4 | 7 |
| mystery | Nigeria (Enugu) | 3 | 2 | 4 | 13 |
| mystery | Nigeria (Enugu) | 3 | 2 | 4 | 14 |
| mystery | Nigeria (Enugu) | 3 | 2 | 4 | 15 |
| mystery | Nigeria (Enugu) | 3 | 2 | 4 | 39 |
| mystery | Nigeria (Enugu) | 3 | 2 | 4 | 42 |
| mystery | Nigeria (Enugu) | 3 | 2 | 4 | 43 |
| mystery | Nigeria (Enugu) | 4 | 1 | 2 | 23 |
| mystery | Nigeria (Enugu) | 5 | 1 | 2 | 22 |
| mystery | Nigeria (Enugu) | 5 | 1 | 2 | 55 |
| mystery | Nigeria (Enugu) | 5 | 1 | 2 | 56 |
| mystery | Nigeria (Enugu) | 5 | 2 | 1 | 0 |
| mystery | Nigeria (Enugu) | 6 | 2 | 1 | 26 |
| mystery | Nigeria (Enugu) | 6 | 2 | 1 | 28 |
| mystery | Nigeria (Enugu) | 6 | 2 | 3 | 37 |
| mystery | Nigeria (Enugu) | 7 | 2 | 4 | 6 |
| mystery | Nigeria (Enugu) | 7 | 2 | 4 | 27 |
| mystery | Nigeria (Enugu) | 7 | 2 | 4 | 36 |
| mystery | Nigeria (Enugu) | 7 | 3 | 5 | 0 |
| mystery | Nigeria (Enugu) | 8 | 2 | 1 | 0 |
| mystery | Nigeria (Enugu) | 8 | 2 | 4 | 16 |
| mystery | Nigeria (Enugu) | 8 | 2 | 4 | 34 |
| mystery | Nigeria (Enugu) | 9 | 2 | 1 | 0 |
| mystery | Nigeria (Enugu) | 9 | 2 | 2 | 2 |

| | | | | | |
|---|---|---|---|---|---|
| mystery | Nigeria (Enugu) | 9 | 3 | 5 | 8 |
| mystery | Nigeria (Enugu) | 10 | 3 | 1 | 14 |
| mystery | Nigeria (Enugu) | 10 | 3 | 1 | 18 |
| mystery | Nigeria (Enugu) | 10 | 3 | 1 | 65 |
| mystery | Nigeria (Enugu) | 10 | 3 | 1 | 67 |
| mystery | Nigeria (Enugu) | 11 | 2 | 5 | 22 |
| mystery | Nigeria (Enugu) | 11 | 3 | 7 | 15 |
| mystery | Nigeria (Enugu) | 12 | 2 | 5 | 24 |
| mystery | Nigeria (Enugu) | 12 | 3 | 7 | 18 |
| mystery | Nigeria (Enugu) | 13 | 2 | 1 | 0 |
| mystery | Nigeria (Enugu) | 13 | 2 | 3 | 16 |
| mystery | Nigeria (Enugu) | 13 | 3 | 7 | 1 |
| mystery | Nigeria (Enugu) | 14 | 1 | 3 | 25 |
| mystery | Nigeria (Enugu) | 15 | 2 | 1 | 0 |
| mystery | Nigeria (Enugu) | 15 | 2 | 3 | 20 |
| mystery | Nigeria (Enugu) | 15 | 2 | 3 | 26 |
| mystery | Nigeria (Enugu) | 16 | 1 | 3 | 11 |
| mystery | Nigeria (Enugu) | 16 | 2 | 3 | 0 |
| mystery | Nigeria (Enugu) | 1 | 3 | 1 | 12 |
| mystery | Nigeria (Enugu) | 3 | 2 | 4 | 2 |
| mystery | Nigeria (Enugu) | 3 | 2 | 4 | 8 |
| mystery | Nigeria (Enugu) | 3 | 2 | 4 | 15 |
| mystery | Nigeria (Enugu) | 4 | 1 | 2 | 9 |
| mystery | Nigeria (Enugu) | 4 | 1 | 2 | 23 |
| mystery | Nigeria (Enugu) | 4 | 1 | 2 | 36 |
| mystery | Nigeria (Enugu) | 5 | 2 | 3 | 12 |
| mystery | Nigeria (Enugu) | 7 | 2 | 4 | 6 |
| mystery | Nigeria (Enugu) | 7 | 2 | 4 | 36 |
| mystery | Nigeria (Enugu) | 9 | 2 | 2 | 2 |
| mystery | Nigeria (Enugu) | 14 | 1 | 3 | 32 |
| mystery | Nigeria (Enugu) | 15 | 2 | 1 | 4 |
| mystery | Nigeria (Enugu) | 15 | 2 | 1 | 14 |
| mystery | Nigeria (Enugu) | 15 | 2 | 3 | 10 |
| mystery | Nigeria (Enugu) | 1 | 3 | 1 | 4 |
| mystery | Nigeria (Enugu) | 2 | 3 | 5 | 33 |
| mystery | Nigeria (Enugu) | 4 | 1 | 2 | 1 |
| mystery | Nigeria (Enugu) | 5 | 1 | 2 | 8 |
| mystery | Nigeria (Enugu) | 6 | 2 | 1 | 11 |
| mystery | Nigeria (Enugu) | 6 | 2 | 3 | 36 |
| mystery | Nigeria (Enugu) | 7 | 2 | 4 | 6 |
| mystery | Nigeria (Enugu) | 7 | 2 | 4 | 27 |
| mystery | Nigeria (Enugu) | 11 | 2 | 5 | 22 |
| mystery | Nigeria (Enugu) | 13 | 2 | 5 | 9 |
| mystery | Nigeria (Enugu) | 15 | 2 | 3 | 38 |
| mystery | Nigeria (Enugu) | 5 | 1 | 2 | 2 |
| mystery | Nigeria (Enugu) | 1 | 3 | 1 | 75 |
| mystery | Nigeria (Enugu) | 2 | 3 | 5 | 33 |
| mystery | Nigeria (Enugu) | 4 | 2 | 3 | 3 |
| mystery | Nigeria (Enugu) | 5 | 1 | 2 | 8 |
| mystery | Nigeria (Enugu) | 5 | 1 | 2 | 10 |

| | | | | | |
|---|---|---|---|---|---|
| mystery | Nigeria (Enugu) | 5 | 1 | 2 | 59 |
| mystery | Nigeria (Enugu) | 9 | 2 | 2 | 11 |
| mystery | Nigeria (Enugu) | 10 | 3 | 1 | 8 |
| mystery | Nigeria (Enugu) | 11 | 2 | 5 | 1 |
| mystery | Nigeria (Enugu) | 11 | 2 | 5 | 2 |
| mystery | Nigeria (Enugu) | 16 | 1 | 3 | 2 |
| mystery | Nigeria (Enugu) | 16 | 1 | 3 | 26 |
| mystery | Nigeria (Enugu) | 1 | 3 | 1 | 74 |
| mystery | Nigeria (Enugu) | 3 | 2 | 1 | 2 |
| mystery | Nigeria (Enugu) | 4 | 2 | 3 | 3 |
| mystery | Nigeria (Enugu) | 6 | 2 | 1 | 1 |
| mystery | Nigeria (Enugu) | 6 | 2 | 1 | 11 |
| mystery | Nigeria (Enugu) | 6 | 2 | 3 | 6 |
| mystery | Nigeria (Enugu) | 7 | 2 | 4 | 6 |
| mystery | Nigeria (Enugu) | 10 | 3 | 1 | 37 |
| mystery | Nigeria (Enugu) | 12 | 2 | 5 | 10 |
| mystery | Nigeria (Enugu) | 14 | 1 | 1 | 16 |
| mystery | Nigeria (Enugu) | 14 | 1 | 3 | 4 |
| mystery | Nigeria (Enugu) | 15 | 2 | 3 | 38 |
| mystery | Nigeria (Enugu) | 4 | 1 | 2 | 4 |
| mystery | Nigeria (Enugu) | 4 | 2 | 3 | 3 |
| mystery | Nigeria (Enugu) | 5 | 1 | 2 | 31 |
| mystery | Nigeria (Enugu) | 11 | 2 | 5 | 6 |
| mystery | Nigeria (Enugu) | 14 | 1 | 3 | 4 |
| mystery | Nigeria (Enugu) | 1 | 3 | 1 | 12 |
| mystery | Nigeria (Enugu) | 1 | 3 | 1 | 42 |
| mystery | Nigeria (Enugu) | 1 | 3 | 1 | 77 |
| mystery | Nigeria (Enugu) | 1 | 3 | 1 | 83 |
| mystery | Nigeria (Enugu) | 2 | 3 | 6 | 1 |
| mystery | Nigeria (Enugu) | 3 | 2 | 1 | 2 |
| mystery | Nigeria (Enugu) | 3 | 2 | 4 | 2 |
| mystery | Nigeria (Enugu) | 3 | 2 | 6 | 6 |
| mystery | Nigeria (Enugu) | 4 | 1 | 2 | 9 |
| mystery | Nigeria (Enugu) | 5 | 1 | 2 | 57 |
| mystery | Nigeria (Enugu) | 5 | 2 | 3 | 7 |
| mystery | Nigeria (Enugu) | 6 | 2 | 1 | 1 |
| mystery | Nigeria (Enugu) | 6 | 2 | 1 | 11 |
| mystery | Nigeria (Enugu) | 6 | 2 | 1 | 28 |
| mystery | Nigeria (Enugu) | 6 | 2 | 3 | 9 |
| mystery | Nigeria (Enugu) | 6 | 2 | 3 | 25 |
| mystery | Nigeria (Enugu) | 6 | 2 | 3 | 31 |
| mystery | Nigeria (Enugu) | 6 | 2 | 3 | 37 |
| mystery | Nigeria (Enugu) | 7 | 2 | 4 | 6 |
| mystery | Nigeria (Enugu) | 8 | 2 | 1 | 8 |
| mystery | Nigeria (Enugu) | 8 | 2 | 4 | 10 |
| mystery | Nigeria (Enugu) | 9 | 2 | 2 | 1 |
| mystery | Nigeria (Enugu) | 10 | 3 | 1 | 8 |
| mystery | Nigeria (Enugu) | 10 | 3 | 1 | 33 |
| mystery | Nigeria (Enugu) | 10 | 3 | 1 | 71 |
| mystery | Nigeria (Enugu) | 11 | 2 | 5 | 1 |

| | | | | | |
|---|---|---|---|---|---|
| mystery | Nigeria (Enugu) | 11 | 2 | 5 | 11 |
| mystery | Nigeria (Enugu) | 11 | 2 | 5 | 22 |
| mystery | Nigeria (Enugu) | 11 | 2 | 5 | 26 |
| mystery | Nigeria (Enugu) | 13 | 2 | 3 | 16 |
| mystery | Nigeria (Enugu) | 13 | 3 | 7 | 33 |
| mystery | Nigeria (Enugu) | 14 | 1 | 3 | 1 |
| mystery | Nigeria (Enugu) | 14 | 1 | 3 | 5 |
| mystery | Nigeria (Enugu) | 14 | 1 | 3 | 35 |
| mystery | Nigeria (Enugu) | 15 | 2 | 1 | 7 |
| mystery | Nigeria (Enugu) | 15 | 2 | 3 | 10 |
| mystery | Nigeria (Enugu) | 15 | 2 | 3 | 11 |
| mystery | Nigeria (Enugu) | 15 | 2 | 3 | 24 |
| mystery | Nigeria (Enugu) | 15 | 2 | 3 | 38 |
| mystery | Nigeria (Enugu) | 16 | 1 | 3 | 38 |
| mystery | Nigeria (Enugu) | 3 | 2 | 4 | 21 |
| mystery | Nigeria (Enugu) | 4 | 1 | 2 | 4 |
| mystery | Nigeria (Enugu) | 4 | 1 | 2 | 12 |
| mystery | Nigeria (Enugu) | 6 | 2 | 1 | 13 |
| mystery | Nigeria (Enugu) | 8 | 2 | 4 | 5 |
| mystery | Nigeria (Enugu) | 9 | 3 | 5 | 31 |
| mystery | Nigeria (Enugu) | 10 | 3 | 1 | 67 |
| mystery | Nigeria (Enugu) | 10 | 3 | 1 | 1 |
| mystery | Nigeria (Enugu) | 11 | 2 | 5 | 1 |
| mystery | Nigeria (Enugu) | 14 | 1 | 1 | 15 |
| mystery | Nigeria (Enugu) | 15 | 2 | 3 | 24 |
| mystery | Nigeria (Enugu) | 15 | 2 | 3 | 38 |
| mystery | Nigeria (Enugu) | 1 | 3 | 1 | 36 |
| mystery | Nigeria (Enugu) | 1 | 3 | 1 | 4 |
| mystery | Nigeria (Enugu) | 1 | 3 | 1 | 57 |
| mystery | Nigeria (Enugu) | 1 | 3 | 1 | 59 |
| mystery | Nigeria (Enugu) | 1 | 3 | 1 | 60 |
| mystery | Nigeria (Enugu) | 1 | 3 | 1 | 69 |
| mystery | Nigeria (Enugu) | 1 | 3 | 1 | 81 |
| mystery | Nigeria (Enugu) | 2 | 3 | 4 | 3 |
| mystery | Nigeria (Enugu) | 2 | 3 | 5 | 25 |
| mystery | Nigeria (Enugu) | 3 | 2 | 4 | 15 |
| mystery | Nigeria (Enugu) | 3 | 2 | 4 | 18 |
| mystery | Nigeria (Enugu) | 3 | 2 | 4 | 2 |
| mystery | Nigeria (Enugu) | 3 | 2 | 4 | 7 |
| mystery | Nigeria (Enugu) | 3 | 2 | 6 | 6 |
| mystery | Nigeria (Enugu) | 3 | 3 | 7 | 0 |
| mystery | Nigeria (Enugu) | 4 | 1 | 2 | 23 |
| mystery | Nigeria (Enugu) | 4 | 1 | 2 | 35 |
| mystery | Nigeria (Enugu) | 4 | 1 | 2 | 36 |
| mystery | Nigeria (Enugu) | 4 | 1 | 2 | 53 |
| mystery | Nigeria (Enugu) | 4 | 1 | 2 | 5 |
| mystery | Nigeria (Enugu) | 5 | 1 | 2 | 33 |
| mystery | Nigeria (Enugu) | 5 | 1 | 2 | 37 |
| mystery | Nigeria (Enugu) | 5 | 1 | 2 | 55 |
| mystery | Nigeria (Enugu) | 5 | 1 | 2 | 57 |

| | | | | | |
|---|---|---|---|---|---|
| mystery | Nigeria (Enugu) | 5 | 1 | 2 | 59 |
| mystery | Nigeria (Enugu) | 6 | 2 | 1 | 13 |
| mystery | Nigeria (Enugu) | 6 | 2 | 1 | 15 |
| mystery | Nigeria (Enugu) | 6 | 2 | 1 | 26 |
| mystery | Nigeria (Enugu) | 6 | 2 | 3 | 1 |
| mystery | Nigeria (Enugu) | 6 | 2 | 3 | 39 |
| mystery | Nigeria (Enugu) | 7 | 2 | 4 | 1 |
| mystery | Nigeria (Enugu) | 7 | 2 | 4 | 1 |
| mystery | Nigeria (Enugu) | 7 | 2 | 4 | 22 |
| mystery | Nigeria (Enugu) | 8 | 2 | 4 | 16 |
| mystery | Nigeria (Enugu) | 8 | 2 | 4 | 23 |
| mystery | Nigeria (Enugu) | 8 | 2 | 4 | 34 |
| mystery | Nigeria (Enugu) | 8 | 2 | 4 | 5 |
| mystery | Nigeria (Enugu) | 9 | 2 | 2 | 11 |
| mystery | Nigeria (Enugu) | 2 | 3 | 2 | 5 |
| mystery | Nigeria (Enugu) | 9 | 3 | 5 | 20 |
| mystery | Nigeria (Enugu) | 9 | 3 | 5 | 31 |
| mystery | Nigeria (Enugu) | 9 | 3 | 6 | 8 |
| mystery | Nigeria (Enugu) | 1 | 3 | 1 | 21 |
| mystery | Nigeria (Enugu) | 10 | 3 | 1 | 1 |
| mystery | Nigeria (Enugu) | 10 | 3 | 1 | 8 |
| mystery | Nigeria (Enugu) | 11 | 2 | 5 | 21 |
| mystery | Nigeria (Enugu) | 12 | 2 | 5 | 10 |
| mystery | Nigeria (Enugu) | 13 | 2 | 3 | 21 |
| mystery | Nigeria (Enugu) | 13 | 2 | 5 | 25 |
| mystery | Nigeria (Enugu) | 13 | 2 | 5 | 9 |
| mystery | Nigeria (Enugu) | 13 | 3 | 7 | 28 |
| mystery | Nigeria (Enugu) | 13 | 3 | 7 | 32 |
| mystery | Nigeria (Enugu) | 14 | 1 | 1 | 20 |
| mystery | Nigeria (Enugu) | 14 | 1 | 3 | 23 |
| mystery | Nigeria (Enugu) | 14 | 1 | 3 | 4 |
| mystery | Nigeria (Enugu) | 14 | 1 | 3 | 5 |
| mystery | Nigeria (Enugu) | 15 | 2 | 1 | 4 |
| mystery | Nigeria (Enugu) | 15 | 2 | 1 | 7 |
| mystery | Nigeria (Enugu) | 15 | 2 | 3 | 10 |
| mystery | Nigeria (Enugu) | 15 | 2 | 3 | 26 |
| mystery | Nigeria (Enugu) | 16 | 1 | 1 | 28 |
| mystery | Nigeria (Enugu) | 16 | 1 | 3 | 11 |
| mystery | Nigeria (Enugu) | 16 | 1 | 3 | 6 |
| mystery | Nigeria (Enugu) | 16 | 1 | 3 | 7 |
| mystery | Nigeria (Enugu) | 5 | 1 | 2 | 27 |
| mystery | Nigeria (Enugu) | 1 | 3 | 1 | 21 |
| mystery | Nigeria (Enugu) | 1 | 3 | 1 | 60 |
| mystery | Nigeria (Enugu) | 1 | 3 | 1 | 75 |
| mystery | Nigeria (Enugu) | 2 | 3 | 2 | 5 |
| mystery | Nigeria (Enugu) | 2 | 3 | 2 | 6 |
| mystery | Nigeria (Enugu) | 2 | 3 | 5 | 34 |
| mystery | Nigeria (Enugu) | 3 | 2 | 4 | 2 |
| mystery | Nigeria (Enugu) | 3 | 3 | 7 | 0 |
| mystery | Nigeria (Enugu) | 5 | 1 | 2 | 21 |

| | | | | | |
|---|---|---|---|---|---|
| mystery | Nigeria (Enugu) | 5 | 1 | 2 | 22 |
| mystery | Nigeria (Enugu) | 5 | 1 | 2 | 33 |
| mystery | Nigeria (Enugu) | 5 | 1 | 2 | 49 |
| mystery | Nigeria (Enugu) | 5 | 2 | 3 | 12 |
| mystery | Nigeria (Enugu) | 6 | 2 | 1 | 13 |
| mystery | Nigeria (Enugu) | 6 | 2 | 3 | 37 |
| mystery | Nigeria (Enugu) | 8 | 2 | 4 | 16 |
| mystery | Nigeria (Enugu) | 8 | 2 | 4 | 23 |
| mystery | Nigeria (Enugu) | 9 | 3 | 5 | 20 |
| mystery | Nigeria (Enugu) | 9 | 3 | 5 | 31 |
| mystery | Nigeria (Enugu) | 9 | 3 | 6 | 8 |
| mystery | Nigeria (Enugu) | 10 | 3 | 1 | 1 |
| mystery | Nigeria (Enugu) | 10 | 3 | 4 | 20 |
| mystery | Nigeria (Enugu) | 12 | 3 | 7 | 6 |
| mystery | Nigeria (Enugu) | 13 | 2 | 3 | 21 |
| mystery | Nigeria (Enugu) | 13 | 3 | 7 | 28 |
| mystery | Nigeria (Enugu) | 13 | 3 | 7 | 8 |
| mystery | Nigeria (Enugu) | 13 | 3 | 7 | 9 |
| mystery | Nigeria (Enugu) | 14 | 1 | 1 | 1 |
| mystery | Nigeria (Enugu) | 14 | 1 | 1 | 7 |
| mystery | Nigeria (Enugu) | 15 | 2 | 1 | 17 |
| mystery | Nigeria (Enugu) | 15 | 2 | 1 | 5 |
| mystery | Nigeria (Enugu) | 15 | 2 | 3 | 26 |
| mystery | Nigeria (Enugu) | 15 | 2 | 3 | 38 |
| mystery | Nigeria (Enugu) | 16 | 1 | 1 | 17 |
| mystery | Nigeria (Enugu) | 16 | 1 | 3 | 2 |
| mystery | Nigeria (Enugu) | 16 | 1 | 3 | 31 |
| mystery | Nigeria (Enugu) | 1 | 3 | 1 | 12 |
| mystery | Nigeria (Enugu) | 1 | 3 | 1 | 21 |
| mystery | Nigeria (Enugu) | 1 | 3 | 1 | 42 |
| mystery | Nigeria (Enugu) | 1 | 3 | 1 | 59 |
| mystery | Nigeria (Enugu) | 1 | 3 | 1 | 60 |
| mystery | Nigeria (Enugu) | 2 | 3 | 2 | 5 |
| mystery | Nigeria (Enugu) | 2 | 3 | 2 | 6 |
| mystery | Nigeria (Enugu) | 2 | 3 | 5 | 34 |
| mystery | Nigeria (Enugu) | 3 | 2 | 1 | 2 |
| mystery | Nigeria (Enugu) | 3 | 2 | 6 | 6 |
| mystery | Nigeria (Enugu) | 3 | 3 | 7 | 0 |
| mystery | Nigeria (Enugu) | 4 | 1 | 2 | 23 |
| mystery | Nigeria (Enugu) | 5 | 1 | 2 | 2 |
| mystery | Nigeria (Enugu) | 5 | 1 | 2 | 8 |
| mystery | Nigeria (Enugu) | 5 | 1 | 2 | 10 |
| mystery | Nigeria (Enugu) | 7 | 2 | 4 | 22 |
| mystery | Nigeria (Enugu) | 8 | 2 | 4 | 34 |
| mystery | Nigeria (Enugu) | 9 | 2 | 2 | 6 |
| mystery | Nigeria (Enugu) | 9 | 3 | 5 | 31 |
| mystery | Nigeria (Enugu) | 9 | 3 | 6 | 8 |
| mystery | Nigeria (Enugu) | 10 | 3 | 1 | 14 |
| mystery | Nigeria (Enugu) | 10 | 3 | 1 | 15 |
| mystery | Nigeria (Enugu) | 10 | 3 | 1 | 33 |

| | | | | | |
|---|---|---|---|---|---|
| mystery | Nigeria (Enugu) | 10 | 3 | 1 | 53 |
| mystery | Nigeria (Enugu) | 10 | 3 | 1 | 67 |
| mystery | Nigeria (Enugu) | 11 | 2 | 5 | 2 |
| mystery | Nigeria (Enugu) | 12 | 2 | 5 | 24 |
| mystery | Nigeria (Enugu) | 13 | 2 | 3 | 21 |
| mystery | Nigeria (Enugu) | 13 | 2 | 5 | 18 |
| mystery | Nigeria (Enugu) | 14 | 1 | 3 | 21 |
| mystery | Nigeria (Enugu) | 16 | 1 | 3 | 27 |
| mystery | Nigeria (Enugu) | 16 | 1 | 3 | 30 |
| mystery | Nigeria (Enugu) | 5 | 1 | 2 | 8 |
| mystery | Nigeria (Enugu) | 14 | 1 | 3 | 5 |
| mystery | Nigeria (Enugu) | 15 | 2 | 1 | 7 |
| mystery | Nigeria (Enugu) | 5 | 1 | 2 | 8 |
| mystery | Nigeria (Enugu) | 5 | 2 | 3 | 7 |
| mystery | Nigeria (Enugu) | 6 | 2 | 1 | 1 |
| mystery | Nigeria (Enugu) | 7 | 2 | 4 | 1 |
| mystery | Nigeria (Enugu) | 8 | 2 | 4 | 10 |
| mystery | Nigeria (Enugu) | 11 | 2 | 5 | 1 |
| mystery | Nigeria (Enugu) | 11 | 2 | 5 | 6 |
| mystery | Nigeria (Enugu) | 12 | 2 | 5 | 10 |
| mystery | Nigeria (Enugu) | 15 | 2 | 1 | 29 |
| mystery | Nigeria (Enugu) | 15 | 2 | 3 | 38 |
| mystery | Nigeria (Enugu) | 5 | 1 | 2 | 33 |
| mystery | Nigeria (Enugu) | 3 | 1 | 2 | 0 |
| mystery | Nigeria (Enugu) | 5 | 1 | 2 | 30 |
| mystery | Nigeria (Enugu) | 6 | 2 | 3 | 14 |
| mystery | Nigeria (Enugu) | 3 | 2 | 4 | 7 |
| mystery | Nigeria (Enugu) | 10 | 3 | 1 | 18 |
| mystery | Nigeria (Enugu) | 10 | 3 | 1 | 21 |
| mystery | Nigeria (Enugu) | 16 | 1 | 1 | 7 |
| mystery | Nigeria (Enugu) | 1 | 3 | 1 | 57 |
| mystery | Nigeria (Enugu) | 3 | 2 | 1 | 2 |
| mystery | Nigeria (Enugu) | 4 | 1 | 2 | 12 |
| mystery | Nigeria (Enugu) | 5 | 1 | 2 | 8 |
| mystery | Nigeria (Enugu) | 12 | 3 | 7 | 6 |
| mystery | Nigeria (Enugu) | 13 | 2 | 5 | 25 |
| mystery | Nigeria (Enugu) | 13 | 3 | 7 | 28 |
| mystery | Nigeria (Enugu) | 14 | 1 | 1 | 16 |
| mystery | Nigeria (Enugu) | 16 | 1 | 3 | 27 |
| mystery | Nigeria (Enugu) | 11 | 3 | 7 | 24 |
| mystery | Nigeria (Enugu) | 12 | 2 | 5 | 28 |
| mystery | Nigeria (Enugu) | 5 | 1 | 2 | 22 |
| mystery | Nigeria (Enugu) | 7 | 2 | 4 | 22 |
| mystery | Nigeria (Enugu) | 9 | 2 | 1 | 0 |
| mystery | Nigeria (Enugu) | 6 | 2 | 3 | 6 |
| mystery | Nigeria (Enugu) | 2 | 3 | 2 | 5 |
| mystery | Nigeria (Enugu) | 3 | 2 | 4 | 7 |
| mystery | Nigeria (Enugu) | 3 | 2 | 4 | 21 |
| mystery | Nigeria (Enugu) | 4 | 1 | 2 | 19 |
| mystery | Nigeria (Enugu) | 4 | 1 | 2 | 38 |

| | | | | | |
|---|---|---|---|---|---|
| mystery | Nigeria (Enugu) | 5 | 1 | 2 | 8 |
| mystery | Nigeria (Enugu) | 5 | 1 | 2 | 10 |
| mystery | Nigeria (Enugu) | 5 | 1 | 2 | 21 |
| mystery | Nigeria (Enugu) | 5 | 1 | 2 | 22 |
| mystery | Nigeria (Enugu) | 5 | 2 | 3 | 12 |
| mystery | Nigeria (Enugu) | 7 | 2 | 4 | 20 |
| mystery | Nigeria (Enugu) | 7 | 2 | 4 | 36 |
| mystery | Nigeria (Enugu) | 8 | 2 | 1 | 8 |
| mystery | Nigeria (Enugu) | 9 | 2 | 2 | 6 |
| mystery | Nigeria (Enugu) | 11 | 2 | 5 | 21 |
| mystery | Nigeria (Enugu) | 11 | 3 | 7 | 24 |
| mystery | Nigeria (Enugu) | 14 | 1 | 3 | 25 |
| mystery | Nigeria (Enugu) | 15 | 2 | 3 | 11 |
| mystery | Nigeria (Enugu) | 15 | 2 | 3 | 26 |
| mystery | Nigeria (Enugu) | 1 | 3 | 1 | 4 |
| mystery | Nigeria (Enugu) | 1 | 3 | 1 | 39 |
| mystery | Nigeria (Enugu) | 2 | 3 | 2 | 5 |
| mystery | Nigeria (Enugu) | 4 | 1 | 2 | 8 |
| mystery | Nigeria (Enugu) | 4 | 1 | 2 | 38 |
| mystery | Nigeria (Enugu) | 4 | 2 | 3 | 3 |
| mystery | Nigeria (Enugu) | 5 | 1 | 2 | 49 |
| mystery | Nigeria (Enugu) | 6 | 2 | 3 | 14 |
| mystery | Nigeria (Enugu) | 7 | 2 | 4 | 1 |
| mystery | Nigeria (Enugu) | 7 | 2 | 4 | 22 |
| mystery | Nigeria (Enugu) | 8 | 2 | 4 | 10 |
| mystery | Nigeria (Enugu) | 8 | 2 | 4 | 17 |
| mystery | Nigeria (Enugu) | 9 | 2 | 2 | 10 |
| mystery | Nigeria (Enugu) | 2 | 3 | 2 | 5 |
| mystery | Nigeria (Enugu) | 9 | 3 | 5 | 19 |
| mystery | Nigeria (Enugu) | 10 | 3 | 1 | 14 |
| mystery | Nigeria (Enugu) | 13 | 2 | 5 | 25 |
| mystery | Nigeria (Enugu) | 13 | 2 | 5 | 25 |
| mystery | Nigeria (Enugu) | 13 | 3 | 7 | 4 |
| mystery | Nigeria (Enugu) | 13 | 3 | 7 | 32 |
| mystery | Nigeria (Enugu) | 14 | 1 | 1 | 7 |
| mystery | Nigeria (Enugu) | 14 | 1 | 3 | 1 |
| mystery | Nigeria (Enugu) | 15 | 2 | 1 | 14 |
| mystery | Nigeria (Enugu) | 6 | 2 | 3 | 36 |
| mystery | Nigeria (Enugu) | 10 | 3 | 1 | 66 |
| mystery | Nigeria (Enugu) | 15 | 2 | 1 | 7 |
| mystery | Nigeria (Enugu) | 1 | 3 | 1 | 4 |
| mystery | Nigeria (Enugu) | 1 | 3 | 1 | 12 |
| mystery | Nigeria (Enugu) | 1 | 3 | 1 | 13 |
| mystery | Nigeria (Enugu) | 1 | 3 | 1 | 21 |
| mystery | Nigeria (Enugu) | 1 | 3 | 1 | 42 |
| mystery | Nigeria (Enugu) | 1 | 3 | 1 | 57 |
| mystery | Nigeria (Enugu) | 1 | 3 | 1 | 60 |
| mystery | Nigeria (Enugu) | 1 | 3 | 1 | 74 |
| mystery | Nigeria (Enugu) | 1 | 3 | 1 | 77 |
| mystery | Nigeria (Enugu) | 1 | 3 | 1 | 80 |

| | | | | | |
|---|---|---|---|---|---|
| mystery | Nigeria (Enugu) | 1 | 3 | 1 | 81 |
| mystery | Nigeria (Enugu) | 2 | 3 | 5 | 28 |
| mystery | Nigeria (Enugu) | 2 | 3 | 5 | 29 |
| mystery | Nigeria (Enugu) | 2 | 3 | 5 | 33 |
| mystery | Nigeria (Enugu) | 2 | 3 | 5 | 34 |
| mystery | Nigeria (Enugu) | 2 | 3 | 6 | 2 |
| mystery | Nigeria (Enugu) | 3 | 2 | 1 | 2 |
| mystery | Nigeria (Enugu) | 3 | 2 | 4 | 14 |
| mystery | Nigeria (Enugu) | 3 | 2 | 4 | 43 |
| mystery | Nigeria (Enugu) | 3 | 2 | 6 | 6 |
| mystery | Nigeria (Enugu) | 4 | 1 | 2 | 9 |
| mystery | Nigeria (Enugu) | 4 | 1 | 2 | 15 |
| mystery | Nigeria (Enugu) | 4 | 1 | 2 | 18 |
| mystery | Nigeria (Enugu) | 4 | 1 | 2 | 23 |
| mystery | Nigeria (Enugu) | 4 | 1 | 2 | 35 |
| mystery | Nigeria (Enugu) | 4 | 1 | 2 | 38 |
| mystery | Nigeria (Enugu) | 5 | 1 | 2 | 21 |
| mystery | Nigeria (Enugu) | 5 | 1 | 2 | 30 |
| mystery | Nigeria (Enugu) | 5 | 1 | 2 | 55 |
| mystery | Nigeria (Enugu) | 5 | 1 | 2 | 57 |
| mystery | Nigeria (Enugu) | 6 | 2 | 1 | 15 |
| mystery | Nigeria (Enugu) | 6 | 2 | 3 | 14 |
| mystery | Nigeria (Enugu) | 6 | 2 | 3 | 36 |
| mystery | Nigeria (Enugu) | 6 | 2 | 3 | 39 |
| mystery | Nigeria (Enugu) | 7 | 2 | 4 | 25 |
| mystery | Nigeria (Enugu) | 8 | 2 | 1 | 8 |
| mystery | Nigeria (Enugu) | 8 | 2 | 4 | 5 |
| mystery | Nigeria (Enugu) | 8 | 2 | 4 | 16 |
| mystery | Nigeria (Enugu) | 8 | 2 | 4 | 44 |
| mystery | Nigeria (Enugu) | 9 | 2 | 2 | 10 |
| mystery | Nigeria (Enugu) | 9 | 2 | 2 | 11 |
| mystery | Nigeria (Enugu) | 9 | 3 | 5 | 8 |
| mystery | Nigeria (Enugu) | 9 | 3 | 5 | 16 |
| mystery | Nigeria (Enugu) | 9 | 3 | 5 | 19 |
| mystery | Nigeria (Enugu) | 10 | 3 | 1 | 5 |
| mystery | Nigeria (Enugu) | 10 | 3 | 1 | 14 |
| mystery | Nigeria (Enugu) | 10 | 3 | 1 | 24 |
| mystery | Nigeria (Enugu) | 10 | 3 | 1 | 33 |
| mystery | Nigeria (Enugu) | 10 | 3 | 1 | 37 |
| mystery | Nigeria (Enugu) | 10 | 3 | 1 | 66 |
| mystery | Nigeria (Enugu) | 10 | 3 | 1 | 67 |
| mystery | Nigeria (Enugu) | 10 | 3 | 1 | 78 |
| mystery | Nigeria (Enugu) | 11 | 2 | 5 | 5 |
| mystery | Nigeria (Enugu) | 11 | 2 | 5 | 11 |
| mystery | Nigeria (Enugu) | 11 | 2 | 5 | 21 |
| mystery | Nigeria (Enugu) | 11 | 2 | 5 | 26 |
| mystery | Nigeria (Enugu) | 11 | 3 | 7 | 14 |
| mystery | Nigeria (Enugu) | 11 | 3 | 7 | 15 |
| mystery | Nigeria (Enugu) | 12 | 2 | 5 | 3 |
| mystery | Nigeria (Enugu) | 12 | 3 | 7 | 12 |

| | | | | | |
|---|---|---|---|---|---|
| mystery | Nigeria (Enugu) | 13 | 2 | 3 | 21 |
| mystery | Nigeria (Enugu) | 13 | 2 | 5 | 25 |
| mystery | Nigeria (Enugu) | 13 | 3 | 7 | 28 |
| mystery | Nigeria (Enugu) | 14 | 1 | 1 | 18 |
| mystery | Nigeria (Enugu) | 14 | 1 | 3 | 4 |
| mystery | Nigeria (Enugu) | 14 | 1 | 3 | 5 |
| mystery | Nigeria (Enugu) | 15 | 2 | 1 | 5 |
| mystery | Nigeria (Enugu) | 15 | 2 | 1 | 7 |
| mystery | Nigeria (Enugu) | 15 | 2 | 1 | 14 |
| mystery | Nigeria (Enugu) | 15 | 2 | 1 | 19 |
| mystery | Nigeria (Enugu) | 15 | 2 | 3 | 10 |
| mystery | Nigeria (Enugu) | 15 | 2 | 3 | 20 |
| mystery | Nigeria (Enugu) | 15 | 2 | 3 | 26 |
| mystery | Nigeria (Enugu) | 15 | 2 | 3 | 30 |
| mystery | Nigeria (Enugu) | 16 | 1 | 1 | 7 |
| mystery | Nigeria (Enugu) | 16 | 1 | 1 | 28 |
| mystery | Nigeria (Enugu) | 16 | 1 | 3 | 4 |
| mystery | Nigeria (Enugu) | 16 | 1 | 3 | 7 |
| mystery | Nigeria (Enugu) | 16 | 1 | 3 | 27 |
| mystery | Nigeria (Enugu) | 2 | 3 | 6 | 1 |
| mystery | Nigeria (Enugu) | 2 | 3 | 6 | 1 |
| mystery | Nigeria (Enugu) | 4 | 1 | 2 | 9 |
| mystery | Nigeria (Enugu) | 11 | 2 | 5 | 1 |
| mystery | Nigeria (Enugu) | 14 | 1 | 3 | 5 |
| mystery | Nigeria (Enugu) | 11 | 2 | 5 | 1 |
| mystery | Nigeria (Enugu) | 11 | 2 | 5 | 1 |
| mystery | Nigeria (Enugu) | 1 | 3 | 1 | 21 |
| mystery | Nigeria (Enugu) | 1 | 3 | 1 | 59 |
| mystery | Nigeria (Enugu) | 4 | 1 | 2 | 1 |
| mystery | Nigeria (Enugu) | 4 | 1 | 2 | 4 |
| mystery | Nigeria (Enugu) | 4 | 1 | 2 | 9 |
| mystery | Nigeria (Enugu) | 4 | 1 | 2 | 36 |
| mystery | Nigeria (Enugu) | 5 | 1 | 2 | 7 |
| mystery | Nigeria (Enugu) | 5 | 1 | 2 | 8 |
| mystery | Nigeria (Enugu) | 5 | 1 | 2 | 17 |
| mystery | Nigeria (Enugu) | 5 | 2 | 3 | 12 |
| mystery | Nigeria (Enugu) | 7 | 2 | 4 | 6 |
| mystery | Nigeria (Enugu) | 14 | 1 | 3 | 4 |
| mystery | Nigeria (Enugu) | 15 | 2 | 1 | 5 |
| mystery | Nigeria (Enugu) | 15 | 2 | 3 | 10 |
| mystery | Nigeria (Enugu) | 16 | 1 | 3 | 2 |
| mystery | Nigeria (Enugu) | 4 | 1 | 2 | 23 |
| mystery | Nigeria (Enugu) | 5 | 1 | 2 | 37 |
| mystery | Nigeria (Enugu) | 12 | 2 | 5 | 10 |
| mystery | Nigeria (Enugu) | 4 | 1 | 2 | 8 |
| mystery | Nigeria (Enugu) | 5 | 1 | 2 | 8 |
| mystery | Nigeria (Enugu) | 6 | 2 | 1 | 11 |
| mystery | Nigeria (Enugu) | 8 | 2 | 4 | 5 |
| mystery | Nigeria (Enugu) | 12 | 3 | 7 | 6 |
| mystery | Nigeria (Enugu) | 13 | 3 | 7 | 3 |

| | | | | | |
|---|---|---|---|---|---|
| mystery | Nigeria (Enugu) | 1 | 3 | 1 | 21 |
| mystery | Nigeria (Enugu) | 1 | 3 | 1 | 59 |
| mystery | Nigeria (Enugu) | 2 | 3 | 5 | 34 |
| mystery | Nigeria (Enugu) | 4 | 1 | 2 | 36 |
| mystery | Nigeria (Enugu) | 5 | 1 | 2 | 8 |
| mystery | Nigeria (Enugu) | 6 | 2 | 1 | 11 |
| mystery | Nigeria (Enugu) | 6 | 2 | 3 | 6 |
| mystery | Nigeria (Enugu) | 9 | 2 | 2 | 11 |
| mystery | Nigeria (Enugu) | 9 | 3 | 5 | 8 |
| mystery | Nigeria (Enugu) | 10 | 3 | 1 | 2 |
| mystery | Nigeria (Enugu) | 11 | 2 | 5 | 6 |
| mystery | Nigeria (Enugu) | 13 | 2 | 5 | 9 |
| mystery | Nigeria (Enugu) | 13 | 3 | 7 | 7 |
| mystery | Nigeria (Enugu) | 14 | 1 | 3 | 1 |
| mystery | Nigeria (Enugu) | 15 | 2 | 1 | 29 |
| mystery | Nigeria (Enugu) | 15 | 2 | 3 | 38 |
| mystery | Nigeria (Enugu) | 15 | 2 | 1 | 19 |
| mystery | Nigeria (Enugu) | 15 | 2 | 3 | 28 |
| mystery | Nigeria (Enugu) | 3 | 2 | 4 | 13 |
| mystery | Nigeria (Enugu) | 4 | 1 | 2 | 36 |
| mystery | Nigeria (Enugu) | 5 | 2 | 3 | 7 |
| mystery | Nigeria (Enugu) | 11 | 2 | 5 | 1 |
| mystery | Nigeria (Enugu) | 12 | 2 | 5 | 16 |
| mystery | Nigeria (Enugu) | 14 | 1 | 3 | 4 |
| mystery | Nigeria (Enugu) | 4 | 1 | 2 | 1 |
| mystery | Nigeria (Enugu) | 4 | 1 | 2 | 1 |
| mystery | Nigeria (Enugu) | 5 | 2 | 3 | 7 |
| mystery | Nigeria (Enugu) | 1 | 3 | 1 | 10 |
| mystery | Nigeria (Enugu) | 1 | 3 | 1 | 81 |
| mystery | Nigeria (Enugu) | 2 | 3 | 6 | 2 |
| mystery | Nigeria (Enugu) | 3 | 2 | 1 | 2 |
| mystery | Nigeria (Enugu) | 5 | 2 | 3 | 7 |
| mystery | Nigeria (Enugu) | 6 | 2 | 3 | 6 |
| mystery | Nigeria (Enugu) | 7 | 2 | 4 | 1 |
| mystery | Nigeria (Enugu) | 8 | 2 | 4 | 5 |
| mystery | Nigeria (Enugu) | 9 | 2 | 2 | 1 |
| mystery | Nigeria (Enugu) | 9 | 3 | 6 | 8 |
| mystery | Nigeria (Enugu) | 10 | 3 | 1 | 2 |
| mystery | Nigeria (Enugu) | 10 | 3 | 1 | 15 |
| mystery | Nigeria (Enugu) | 11 | 2 | 5 | 5 |
| mystery | Nigeria (Enugu) | 11 | 2 | 5 | 12 |
| mystery | Nigeria (Enugu) | 12 | 2 | 5 | 16 |
| mystery | Nigeria (Enugu) | 14 | 1 | 1 | 1 |
| mystery | Nigeria (Enugu) | 14 | 1 | 3 | 29 |
| mystery | Nigeria (Enugu) | 15 | 2 | 3 | 20 |
| mystery | Nigeria (Enugu) | 15 | 2 | 3 | 38 |
| mystery | Nigeria (Enugu) | 16 | 1 | 3 | 7 |
| mystery | Nigeria (Enugu) | 16 | 1 | 3 | 11 |
| mystery | Nigeria (Enugu) | 16 | 1 | 3 | 14 |
| mystery | Nigeria (Enugu) | 16 | 1 | 3 | 30 |

| | | | | | |
|---|---|---|---|---|---|
| mystery | Nigeria (Enugu) | 4 | 1 | 2 | 9 |
| mystery | Nigeria (Enugu) | 4 | 1 | 2 | 53 |
| mystery | Nigeria (Enugu) | 5 | 1 | 2 | 31 |
| mystery | Nigeria (Enugu) | 5 | 1 | 2 | 59 |
| mystery | Nigeria (Enugu) | 6 | 2 | 3 | 36 |
| mystery | Nigeria (Enugu) | 7 | 2 | 4 | 1 |
| mystery | Nigeria (Enugu) | 7 | 2 | 4 | 25 |
| mystery | Nigeria (Enugu) | 9 | 3 | 5 | 8 |
| mystery | Nigeria (Enugu) | 10 | 3 | 1 | 33 |
| mystery | Nigeria (Enugu) | 10 | 3 | 1 | 71 |
| mystery | Nigeria (Enugu) | 11 | 2 | 5 | 1 |
| mystery | Nigeria (Enugu) | 11 | 2 | 5 | 5 |
| mystery | Nigeria (Enugu) | 11 | 2 | 5 | 6 |
| mystery | Nigeria (Enugu) | 12 | 2 | 5 | 3 |
| mystery | Nigeria (Enugu) | 12 | 2 | 5 | 16 |
| mystery | Nigeria (Enugu) | 12 | 2 | 5 | 28 |
| mystery | Nigeria (Enugu) | 14 | 1 | 1 | 1 |
| mystery | Nigeria (Enugu) | 14 | 1 | 3 | 4 |
| mystery | Nigeria (Enugu) | 16 | 1 | 3 | 38 |
| mystery | Nigeria (Enugu) | 3 | 2 | 1 | 2 |
| mystery | Nigeria (Enugu) | 6 | 2 | 1 | 11 |
| mystery | Nigeria (Enugu) | 6 | 2 | 3 | 6 |
| mystery | Nigeria (Enugu) | 9 | 2 | 2 | 1 |
| mystery | Nigeria (Enugu) | 9 | 2 | 2 | 11 |
| mystery | Nigeria (Enugu) | 11 | 2 | 5 | 1 |
| mystery | Nigeria (Enugu) | 16 | 1 | 3 | 2 |
| mystery | Nigeria (Enugu) | 7 | 2 | 4 | 1 |
| mystery | Nigeria (Enugu) | 11 | 2 | 5 | 1 |
| mystery | Nigeria (Enugu) | 11 | 2 | 5 | 6 |
| mystery | Nigeria (Enugu) | 4 | 1 | 2 | 19 |
| mystery | Nigeria (Enugu) | 6 | 2 | 3 | 9 |
| mystery | Nigeria (Enugu) | 15 | 2 | 3 | 38 |
| mystery | Nigeria (Enugu) | 5 | 2 | 3 | 12 |
| mystery | Nigeria (Enugu) | 9 | 2 | 2 | 10 |
| mystery | Nigeria (Enugu) | 15 | 2 | 1 | 19 |
| mystery | Nigeria (Enugu) | 5 | 1 | 2 | 57 |
| mystery | Nigeria (Enugu) | 1 | 3 | 1 | 60 |
| mystery | Nigeria (Enugu) | 2 | 3 | 5 | 30 |
| mystery | Nigeria (Enugu) | 4 | 1 | 2 | 1 |
| mystery | Nigeria (Enugu) | 9 | 3 | 5 | 11 |
| mystery | Nigeria (Enugu) | 4 | 1 | 2 | 1 |
| mystery | Nigeria (Enugu) | 4 | 1 | 2 | 36 |
| mystery | Nigeria (Enugu) | 5 | 1 | 2 | 31 |
| mystery | Nigeria (Enugu) | 9 | 3 | 6 | 8 |
| mystery | Nigeria (Enugu) | 12 | 2 | 5 | 16 |
| mystery | Nigeria (Enugu) | 14 | 1 | 3 | 9 |
| mystery | Nigeria (Enugu) | 15 | 2 | 1 | 5 |
| mystery | Nigeria (Enugu) | 5 | 1 | 2 | 57 |
| mystery | Nigeria (Enugu) | 1 | 3 | 1 | 81 |
| mystery | Nigeria (Enugu) | 3 | 2 | 4 | 42 |

| | | | | | |
|---|---|---|---|---|---|
| mystery | Nigeria (Enugu) | 7 | 3 | 7 | 0 |
| mystery | Nigeria (Enugu) | 8 | 2 | 1 | 8 |
| mystery | Nigeria (Enugu) | 8 | 2 | 4 | 10 |
| mystery | Nigeria (Enugu) | 10 | 3 | 1 | 2 |
| mystery | Nigeria (Enugu) | 14 | 1 | 1 | 1 |
| mystery | Nigeria (Enugu) | 14 | 1 | 1 | 16 |
| mystery | Nigeria (Enugu) | 14 | 1 | 3 | 4 |
| mystery | Nigeria (Enugu) | 15 | 2 | 1 | 5 |
| mystery | Nigeria (Enugu) | 15 | 2 | 1 | 14 |
| mystery | Nigeria (Enugu) | 15 | 2 | 3 | 10 |
| mystery | Nigeria (Enugu) | 1 | 3 | 1 | 77 |
| mystery | Nigeria (Enugu) | 3 | 2 | 4 | 21 |
| mystery | Nigeria (Enugu) | 4 | 1 | 2 | 1 |
| mystery | Nigeria (Enugu) | 5 | 1 | 2 | 7 |
| mystery | Nigeria (Enugu) | 5 | 2 | 3 | 7 |
| mystery | Nigeria (Enugu) | 6 | 2 | 3 | 31 |
| mystery | Nigeria (Enugu) | 7 | 2 | 4 | 1 |
| mystery | Nigeria (Enugu) | 7 | 2 | 4 | 25 |
| mystery | Nigeria (Enugu) | 8 | 2 | 4 | 44 |
| mystery | Nigeria (Enugu) | 9 | 2 | 2 | 1 |
| mystery | Nigeria (Enugu) | 9 | 3 | 6 | 8 |
| mystery | Nigeria (Enugu) | 10 | 3 | 1 | 15 |
| mystery | Nigeria (Enugu) | 11 | 2 | 5 | 1 |
| mystery | Nigeria (Enugu) | 11 | 2 | 5 | 5 |
| mystery | Nigeria (Enugu) | 12 | 2 | 5 | 16 |
| mystery | Nigeria (Enugu) | 14 | 1 | 1 | 18 |
| mystery | Nigeria (Enugu) | 16 | 1 | 3 | 11 |
| mystery | Nigeria (Enugu) | 1 | 3 | 1 | 4 |
| mystery | Nigeria (Enugu) | 1 | 3 | 1 | 57 |
| mystery | Nigeria (Enugu) | 1 | 3 | 1 | 82 |
| mystery | Nigeria (Enugu) | 5 | 1 | 2 | 56 |
| mystery | Nigeria (Enugu) | 9 | 3 | 6 | 8 |
| mystery | Nigeria (Enugu) | 10 | 3 | 1 | 2 |
| mystery | Nigeria (Enugu) | 10 | 3 | 1 | 21 |
| mystery | Nigeria (Enugu) | 10 | 3 | 1 | 3 |
| mystery | Nigeria (Enugu) | 11 | 3 | 7 | 24 |
| mystery | Nigeria (Enugu) | 12 | 2 | 5 | 16 |
| mystery | Nigeria (Enugu) | 6 | 2 | 3 | 14 |
| mystery | Nigeria (Enugu) | 4 | 1 | 2 | 8 |
| mystery | Nigeria (Enugu) | 4 | 1 | 2 | 53 |
| mystery | Nigeria (Enugu) | 4 | 1 | 2 | 19 |
| mystery | Nigeria (Enugu) | 8 | 2 | 4 | 5 |
| mystery | Nigeria (Enugu) | 9 | 2 | 2 | 6 |
| mystery | Nigeria (Enugu) | 15 | 2 | 1 | 29 |
| mystery | Nigeria (Enugu) | 1 | 3 | 1 | 57 |
| mystery | Nigeria (Enugu) | 3 | 2 | 4 | 14 |
| mystery | Nigeria (Enugu) | 5 | 1 | 2 | 31 |
| mystery | Nigeria (Enugu) | 6 | 2 | 1 | 11 |
| mystery | Nigeria (Enugu) | 6 | 2 | 3 | 6 |
| mystery | Nigeria (Enugu) | 8 | 2 | 4 | 10 |

| | | | | | |
|---|---|---|---|---|---|
| mystery | Nigeria (Enugu) | 9 | 2 | 2 | 1 |
| mystery | Nigeria (Enugu) | 11 | 2 | 5 | 1 |
| mystery | Nigeria (Enugu) | 11 | 2 | 5 | 5 |
| mystery | Nigeria (Enugu) | 13 | 3 | 7 | 9 |
| mystery | Nigeria (Enugu) | 15 | 2 | 1 | 7 |
| mystery | Nigeria (Enugu) | 4 | 1 | 2 | 4 |
| mystery | Nigeria (Enugu) | 4 | 2 | 3 | 3 |
| mystery | Nigeria (Enugu) | 3 | 2 | 4 | 7 |
| mystery | Nigeria (Enugu) | 4 | 1 | 2 | 23 |
| mystery | Nigeria (Enugu) | 5 | 1 | 2 | 7 |
| mystery | Nigeria (Enugu) | 5 | 1 | 2 | 17 |
| mystery | Nigeria (Enugu) | 5 | 1 | 2 | 22 |
| mystery | Nigeria (Enugu) | 6 | 2 | 1 | 28 |
| mystery | Nigeria (Enugu) | 6 | 2 | 3 | 1 |
| mystery | Nigeria (Enugu) | 7 | 2 | 4 | 20 |
| mystery | Nigeria (Enugu) | 7 | 2 | 4 | 25 |
| mystery | Nigeria (Enugu) | 10 | 3 | 1 | 33 |
| mystery | Nigeria (Enugu) | 11 | 2 | 5 | 1 |
| mystery | Nigeria (Enugu) | 11 | 2 | 5 | 5 |
| mystery | Nigeria (Enugu) | 11 | 2 | 5 | 6 |
| mystery | Nigeria (Enugu) | 11 | 2 | 5 | 11 |
| mystery | Nigeria (Enugu) | 13 | 2 | 3 | 16 |
| mystery | Nigeria (Enugu) | 13 | 2 | 5 | 9 |
| mystery | Nigeria (Enugu) | 13 | 2 | 5 | 25 |
| mystery | Nigeria (Enugu) | 15 | 2 | 1 | 5 |
| mystery | Nigeria (Enugu) | 15 | 2 | 1 | 19 |
| mystery | Nigeria (Enugu) | 15 | 2 | 3 | 20 |
| mystery | Nigeria (Enugu) | 15 | 2 | 3 | 34 |
| mystery | Nigeria (Enugu) | 15 | 2 | 1 | 19 |
| mystery | Nigeria (Enugu) | 3 | 2 | 4 | 7 |
| mystery | Nigeria (Enugu) | 6 | 2 | 3 | 37 |
| mystery | Nigeria (Enugu) | 7 | 2 | 4 | 1 |
| mystery | Nigeria (Enugu) | 8 | 2 | 4 | 5 |
| mystery | Nigeria (Enugu) | 15 | 2 | 1 | 5 |
| mystery | Nigeria (Enugu) | 15 | 2 | 3 | 28 |
| mystery | Nigeria (Enugu) | 1 | 3 | 1 | 77 |
| mystery | Nigeria (Enugu) | 2 | 3 | 2 | 5 |
| mystery | Nigeria (Enugu) | 4 | 1 | 2 | 53 |
| mystery | Nigeria (Enugu) | 5 | 1 | 2 | 57 |
| mystery | Nigeria (Enugu) | 5 | 1 | 2 | 59 |
| mystery | Nigeria (Enugu) | 5 | 2 | 3 | 12 |
| mystery | Nigeria (Enugu) | 6 | 2 | 1 | 11 |
| mystery | Nigeria (Enugu) | 6 | 2 | 3 | 37 |
| mystery | Nigeria (Enugu) | 7 | 2 | 4 | 1 |
| mystery | Nigeria (Enugu) | 7 | 2 | 4 | 25 |
| mystery | Nigeria (Enugu) | 9 | 2 | 2 | 1 |
| mystery | Nigeria (Enugu) | 11 | 2 | 5 | 1 |
| mystery | Nigeria (Enugu) | 11 | 2 | 5 | 5 |
| mystery | Nigeria (Enugu) | 12 | 2 | 5 | 10 |
| mystery | Nigeria (Enugu) | 13 | 2 | 5 | 9 |

| | | | | | |
|---|---|---|---|---|---|
| mystery | Nigeria (Enugu) | 15 | 2 | 1 | 14 |
| mystery | Nigeria (Enugu) | 15 | 2 | 3 | 38 |
| mystery | Nigeria (Enugu) | 1 | 3 | 1 | 4 |
| mystery | Nigeria (Enugu) | 1 | 3 | 1 | 12 |
| mystery | Nigeria (Enugu) | 1 | 3 | 1 | 13 |
| mystery | Nigeria (Enugu) | 1 | 3 | 1 | 33 |
| mystery | Nigeria (Enugu) | 1 | 3 | 1 | 36 |
| mystery | Nigeria (Enugu) | 1 | 3 | 1 | 38 |
| mystery | Nigeria (Enugu) | 1 | 3 | 1 | 59 |
| mystery | Nigeria (Enugu) | 1 | 3 | 1 | 60 |
| mystery | Nigeria (Enugu) | 1 | 3 | 1 | 69 |
| mystery | Nigeria (Enugu) | 1 | 3 | 1 | 77 |
| mystery | Nigeria (Enugu) | 1 | 3 | 1 | 80 |
| mystery | Nigeria (Enugu) | 1 | 3 | 1 | 82 |
| mystery | Nigeria (Enugu) | 1 | 3 | 1 | 83 |
| mystery | Nigeria (Enugu) | 1 | 3 | 1 | 84 |
| mystery | Nigeria (Enugu) | 2 | 3 | 2 | 5 |
| mystery | Nigeria (Enugu) | 2 | 3 | 6 | 2 |
| mystery | Nigeria (Enugu) | 3 | 2 | 1 | 2 |
| mystery | Nigeria (Enugu) | 3 | 2 | 4 | 2 |
| mystery | Nigeria (Enugu) | 3 | 2 | 4 | 8 |
| mystery | Nigeria (Enugu) | 3 | 2 | 4 | 14 |
| mystery | Nigeria (Enugu) | 3 | 2 | 4 | 15 |
| mystery | Nigeria (Enugu) | 3 | 2 | 4 | 42 |
| mystery | Nigeria (Enugu) | 3 | 2 | 6 | 6 |
| mystery | Nigeria (Enugu) | 4 | 1 | 2 | 15 |
| mystery | Nigeria (Enugu) | 4 | 1 | 2 | 23 |
| mystery | Nigeria (Enugu) | 4 | 1 | 2 | 53 |
| mystery | Nigeria (Enugu) | 4 | 2 | 3 | 3 |
| mystery | Nigeria (Enugu) | 5 | 1 | 2 | 8 |
| mystery | Nigeria (Enugu) | 5 | 1 | 2 | 46 |
| mystery | Nigeria (Enugu) | 5 | 1 | 2 | 56 |
| mystery | Nigeria (Enugu) | 5 | 1 | 2 | 57 |
| mystery | Nigeria (Enugu) | 5 | 1 | 2 | 59 |
| mystery | Nigeria (Enugu) | 5 | 2 | 1 | 0 |
| mystery | Nigeria (Enugu) | 5 | 2 | 3 | 12 |
| mystery | Nigeria (Enugu) | 6 | 2 | 1 | 1 |
| mystery | Nigeria (Enugu) | 6 | 2 | 1 | 11 |
| mystery | Nigeria (Enugu) | 6 | 2 | 3 | 1 |
| mystery | Nigeria (Enugu) | 6 | 2 | 3 | 9 |
| mystery | Nigeria (Enugu) | 6 | 2 | 3 | 25 |
| mystery | Nigeria (Enugu) | 6 | 2 | 3 | 27 |
| mystery | Nigeria (Enugu) | 6 | 2 | 3 | 31 |
| mystery | Nigeria (Enugu) | 6 | 2 | 3 | 37 |
| mystery | Nigeria (Enugu) | 7 | 2 | 4 | 1 |
| mystery | Nigeria (Enugu) | 7 | 2 | 4 | 22 |
| mystery | Nigeria (Enugu) | 8 | 2 | 1 | 8 |
| mystery | Nigeria (Enugu) | 8 | 2 | 4 | 5 |
| mystery | Nigeria (Enugu) | 8 | 2 | 4 | 10 |
| mystery | Nigeria (Enugu) | 8 | 2 | 4 | 16 |

| | | | | | |
|---|---|---|---|---|---|
| mystery | Nigeria (Enugu) | 8 | 2 | 4 | 23 |
| mystery | Nigeria (Enugu) | 9 | 2 | 2 | 2 |
| mystery | Nigeria (Enugu) | 9 | 2 | 2 | 10 |
| mystery | Nigeria (Enugu) | 9 | 2 | 2 | 11 |
| mystery | Nigeria (Enugu) | 9 | 3 | 5 | 19 |
| mystery | Nigeria (Enugu) | 9 | 3 | 6 | 8 |
| mystery | Nigeria (Enugu) | 10 | 3 | 1 | 1 |
| mystery | Nigeria (Enugu) | 10 | 3 | 1 | 2 |
| mystery | Nigeria (Enugu) | 10 | 3 | 1 | 5 |
| mystery | Nigeria (Enugu) | 10 | 3 | 1 | 8 |
| mystery | Nigeria (Enugu) | 10 | 3 | 1 | 14 |
| mystery | Nigeria (Enugu) | 10 | 3 | 1 | 21 |
| mystery | Nigeria (Enugu) | 10 | 3 | 1 | 31 |
| mystery | Nigeria (Enugu) | 10 | 3 | 1 | 33 |
| mystery | Nigeria (Enugu) | 10 | 3 | 1 | 37 |
| mystery | Nigeria (Enugu) | 10 | 3 | 1 | 53 |
| mystery | Nigeria (Enugu) | 10 | 3 | 1 | 66 |
| mystery | Nigeria (Enugu) | 10 | 3 | 1 | 71 |
| mystery | Nigeria (Enugu) | 10 | 3 | 1 | 78 |
| mystery | Nigeria (Enugu) | 10 | 3 | 1 | 79 |
| mystery | Nigeria (Enugu) | 11 | 2 | 5 | 1 |
| mystery | Nigeria (Enugu) | 11 | 2 | 5 | 3 |
| mystery | Nigeria (Enugu) | 11 | 2 | 5 | 5 |
| mystery | Nigeria (Enugu) | 11 | 2 | 5 | 6 |
| mystery | Nigeria (Enugu) | 11 | 2 | 5 | 11 |
| mystery | Nigeria (Enugu) | 11 | 2 | 5 | 12 |
| mystery | Nigeria (Enugu) | 11 | 2 | 5 | 22 |
| mystery | Nigeria (Enugu) | 11 | 2 | 5 | 26 |
| mystery | Nigeria (Enugu) | 11 | 3 | 7 | 15 |
| mystery | Nigeria (Enugu) | 12 | 2 | 5 | 10 |
| mystery | Nigeria (Enugu) | 12 | 2 | 5 | 16 |
| mystery | Nigeria (Enugu) | 12 | 3 | 7 | 18 |
| mystery | Nigeria (Enugu) | 12 | 3 | 7 | 23 |
| mystery | Nigeria (Enugu) | 13 | 2 | 3 | 16 |
| mystery | Nigeria (Enugu) | 13 | 3 | 7 | 3 |
| mystery | Nigeria (Enugu) | 13 | 2 | 5 | 9 |
| mystery | Nigeria (Enugu) | 13 | 3 | 7 | 4 |
| mystery | Nigeria (Enugu) | 13 | 3 | 7 | 8 |
| mystery | Nigeria (Enugu) | 13 | 3 | 7 | 28 |
| mystery | Nigeria (Enugu) | 13 | 3 | 7 | 31 |
| mystery | Nigeria (Enugu) | 13 | 3 | 7 | 33 |
| mystery | Nigeria (Enugu) | 14 | 1 | 1 | 1 |
| mystery | Nigeria (Enugu) | 14 | 1 | 1 | 6 |
| mystery | Nigeria (Enugu) | 14 | 1 | 1 | 7 |
| mystery | Nigeria (Enugu) | 14 | 1 | 3 | 4 |
| mystery | Nigeria (Enugu) | 14 | 1 | 3 | 5 |
| mystery | Nigeria (Enugu) | 14 | 1 | 3 | 9 |
| mystery | Nigeria (Enugu) | 14 | 1 | 3 | 10 |
| mystery | Nigeria (Enugu) | 14 | 1 | 3 | 23 |
| mystery | Nigeria (Enugu) | 14 | 1 | 3 | 35 |

| | | | | | |
|---|---|---|---|---|---|
| mystery | Nigeria (Enugu) | 15 | 2 | 1 | 4 |
| mystery | Nigeria (Enugu) | 15 | 2 | 1 | 5 |
| mystery | Nigeria (Enugu) | 15 | 2 | 1 | 7 |
| mystery | Nigeria (Enugu) | 15 | 2 | 1 | 14 |
| mystery | Nigeria (Enugu) | 15 | 2 | 1 | 17 |
| mystery | Nigeria (Enugu) | 15 | 2 | 1 | 29 |
| mystery | Nigeria (Enugu) | 15 | 2 | 3 | 10 |
| mystery | Nigeria (Enugu) | 15 | 2 | 3 | 15 |
| mystery | Nigeria (Enugu) | 15 | 2 | 3 | 20 |
| mystery | Nigeria (Enugu) | 15 | 2 | 3 | 30 |
| mystery | Nigeria (Enugu) | 15 | 2 | 3 | 38 |
| mystery | Nigeria (Enugu) | 16 | 1 | 1 | 7 |
| mystery | Nigeria (Enugu) | 16 | 1 | 3 | 6 |
| mystery | Nigeria (Enugu) | 16 | 1 | 3 | 7 |
| mystery | Nigeria (Enugu) | 16 | 1 | 3 | 11 |
| mystery | Nigeria (Enugu) | 16 | 1 | 3 | 30 |
| mystery | Nigeria (Enugu) | 16 | 1 | 3 | 38 |
| mystery | Nigeria (Enugu) | 5 | 1 | 2 | 2 |
| mystery | Nigeria (Enugu) | 4 | 1 | 2 | 53 |
| mystery | Nigeria (Enugu) | 5 | 1 | 2 | 7 |
| mystery | Nigeria (Enugu) | 13 | 2 | 5 | 9 |
| mystery | Nigeria (Enugu) | 14 | 1 | 3 | 4 |
| mystery | Nigeria (Enugu) | 14 | 1 | 3 | 29 |
| mystery | Nigeria (Enugu) | 15 | 2 | 3 | 38 |
| mystery | Nigeria (Enugu) | 1 | 3 | 1 | 33 |
| mystery | Nigeria (Enugu) | 2 | 3 | 6 | 1 |
| mystery | Nigeria (Enugu) | 4 | 1 | 2 | 36 |
| mystery | Nigeria (Enugu) | 5 | 1 | 2 | 8 |
| mystery | Nigeria (Enugu) | 5 | 1 | 2 | 57 |
| mystery | Nigeria (Enugu) | 6 | 2 | 1 | 11 |
| mystery | Nigeria (Enugu) | 6 | 2 | 3 | 37 |
| mystery | Nigeria (Enugu) | 7 | 2 | 4 | 6 |
| mystery | Nigeria (Enugu) | 8 | 2 | 4 | 5 |
| mystery | Nigeria (Enugu) | 10 | 3 | 1 | 21 |
| mystery | Nigeria (Enugu) | 10 | 3 | 1 | 33 |
| mystery | Nigeria (Enugu) | 11 | 2 | 5 | 6 |
| mystery | Nigeria (Enugu) | 11 | 2 | 5 | 11 |
| mystery | Nigeria (Enugu) | 12 | 3 | 7 | 23 |
| mystery | Nigeria (Enugu) | 13 | 3 | 7 | 33 |
| mystery | Nigeria (Enugu) | 14 | 1 | 3 | 9 |
| mystery | Nigeria (Enugu) | 15 | 2 | 3 | 38 |
| mystery | Nigeria (Enugu) | 16 | 1 | 3 | 4 |
| mystery | Nigeria (Enugu) | 16 | 1 | 3 | 11 |
| mystery | Nigeria (Enugu) | 2 | 3 | 5 | 33 |
| mystery | Nigeria (Enugu) | 5 | 1 | 2 | 8 |
| mystery | Nigeria (Enugu) | 5 | 1 | 2 | 31 |
| mystery | Nigeria (Enugu) | 11 | 2 | 5 | 1 |
| mystery | Nigeria (Enugu) | 14 | 1 | 3 | 4 |
| mystery | Nigeria (Enugu) | 2 | 3 | 5 | 33 |
| mystery | Nigeria (Enugu) | 6 | 2 | 3 | 6 |

| | | | | | |
|---|---|---|---|---|---|
| mystery | Nigeria (Enugu) | 10 | 3 | 1 | 15 |
| mystery | Nigeria (Enugu) | 16 | 1 | 3 | 2 |
| mystery | Nigeria (Enugu) | 1 | 3 | 1 | 10 |
| mystery | Nigeria (Enugu) | 3 | 2 | 4 | 7 |
| mystery | Nigeria (Enugu) | 3 | 2 | 4 | 42 |
| mystery | Nigeria (Enugu) | 4 | 1 | 2 | 1 |
| mystery | Nigeria (Enugu) | 4 | 1 | 2 | 9 |
| mystery | Nigeria (Enugu) | 5 | 1 | 2 | 8 |
| mystery | Nigeria (Enugu) | 6 | 2 | 1 | 11 |
| mystery | Nigeria (Enugu) | 7 | 2 | 4 | 6 |
| mystery | Nigeria (Enugu) | 10 | 3 | 1 | 2 |
| mystery | Nigeria (Enugu) | 10 | 3 | 1 | 8 |
| mystery | Nigeria (Enugu) | 10 | 3 | 1 | 15 |
| mystery | Nigeria (Enugu) | 10 | 3 | 1 | 33 |
| mystery | Nigeria (Enugu) | 10 | 3 | 1 | 37 |
| mystery | Nigeria (Enugu) | 10 | 3 | 1 | 71 |
| mystery | Nigeria (Enugu) | 11 | 2 | 5 | 6 |
| mystery | Nigeria (Enugu) | 11 | 2 | 5 | 12 |
| mystery | Nigeria (Enugu) | 11 | 2 | 5 | 26 |
| mystery | Nigeria (Enugu) | 12 | 2 | 5 | 16 |
| mystery | Nigeria (Enugu) | 13 | 3 | 7 | 33 |
| mystery | Nigeria (Enugu) | 15 | 2 | 3 | 38 |
| mystery | Nigeria (Enugu) | 16 | 1 | 3 | 2 |
| mystery | Nigeria (Enugu) | 3 | 2 | 4 | 8 |
| mystery | Nigeria (Enugu) | 4 | 1 | 2 | 9 |
| mystery | Nigeria (Enugu) | 5 | 1 | 2 | 8 |
| mystery | Nigeria (Enugu) | 5 | 1 | 2 | 11 |
| mystery | Nigeria (Enugu) | 6 | 2 | 3 | 25 |
| mystery | Nigeria (Enugu) | 6 | 2 | 3 | 31 |
| mystery | Nigeria (Enugu) | 7 | 2 | 4 | 6 |
| mystery | Nigeria (Enugu) | 8 | 2 | 4 | 5 |
| mystery | Nigeria (Enugu) | 10 | 3 | 1 | 21 |
| mystery | Nigeria (Enugu) | 11 | 2 | 5 | 5 |
| mystery | Nigeria (Enugu) | 15 | 2 | 3 | 30 |
| mystery | Nigeria (Enugu) | 16 | 1 | 3 | 26 |
| mystery | Nigeria (Enugu) | 3 | 2 | 1 | 2 |
| mystery | Nigeria (Enugu) | 3 | 2 | 4 | 14 |
| mystery | Nigeria (Enugu) | 3 | 2 | 6 | 6 |
| mystery | Nigeria (Enugu) | 4 | 1 | 2 | 8 |
| mystery | Nigeria (Enugu) | 5 | 1 | 2 | 8 |
| mystery | Nigeria (Enugu) | 6 | 2 | 1 | 28 |
| mystery | Nigeria (Enugu) | 6 | 2 | 3 | 6 |
| mystery | Nigeria (Enugu) | 8 | 2 | 4 | 23 |
| mystery | Nigeria (Enugu) | 9 | 3 | 5 | 20 |
| mystery | Nigeria (Enugu) | 11 | 2 | 5 | 1 |
| mystery | Nigeria (Enugu) | 11 | 2 | 5 | 6 |
| mystery | Nigeria (Enugu) | 14 | 1 | 3 | 32 |
| mystery | Nigeria (Enugu) | 15 | 2 | 1 | 17 |
| mystery | Nigeria (Enugu) | 15 | 2 | 3 | 34 |
| mystery | Nigeria (Enugu) | 1 | 3 | 1 | 82 |

| | | | | | |
|---|---|---|---|---|---|
| mystery | Nigeria (Enugu) | 3 | 2 | 4 | 2 |
| mystery | Nigeria (Enugu) | 3 | 2 | 6 | 6 |
| mystery | Nigeria (Enugu) | 4 | 1 | 2 | 1 |
| mystery | Nigeria (Enugu) | 5 | 1 | 2 | 31 |
| mystery | Nigeria (Enugu) | 5 | 2 | 3 | 12 |
| mystery | Nigeria (Enugu) | 6 | 2 | 3 | 14 |
| mystery | Nigeria (Enugu) | 8 | 2 | 4 | 5 |
| mystery | Nigeria (Enugu) | 10 | 3 | 1 | 2 |
| mystery | Nigeria (Enugu) | 10 | 3 | 1 | 31 |
| mystery | Nigeria (Enugu) | 10 | 3 | 1 | 71 |
| mystery | Nigeria (Enugu) | 12 | 2 | 5 | 16 |
| mystery | Nigeria (Enugu) | 14 | 1 | 3 | 32 |
| mystery | Nigeria (Enugu) | 15 | 2 | 1 | 7 |
| mystery | Nigeria (Enugu) | 10 | 3 | 1 | 79 |
| mystery | Nigeria (Enugu) | 5 | 1 | 2 | 22 |
| mystery | Nigeria (Enugu) | 1 | 3 | 1 | 4 |
| mystery | Nigeria (Enugu) | 1 | 3 | 1 | 10 |
| mystery | Nigeria (Enugu) | 1 | 3 | 1 | 12 |
| mystery | Nigeria (Enugu) | 1 | 3 | 1 | 13 |
| mystery | Nigeria (Enugu) | 1 | 3 | 1 | 38 |
| mystery | Nigeria (Enugu) | 1 | 3 | 1 | 39 |
| mystery | Nigeria (Enugu) | 1 | 3 | 1 | 57 |
| mystery | Nigeria (Enugu) | 1 | 3 | 1 | 68 |
| mystery | Nigeria (Enugu) | 1 | 3 | 1 | 69 |
| mystery | Nigeria (Enugu) | 1 | 3 | 1 | 70 |
| mystery | Nigeria (Enugu) | 1 | 3 | 1 | 74 |
| mystery | Nigeria (Enugu) | 1 | 3 | 1 | 81 |
| mystery | Nigeria (Enugu) | 1 | 3 | 1 | 82 |
| mystery | Nigeria (Enugu) | 2 | 3 | 2 | 8 |
| mystery | Nigeria (Enugu) | 2 | 3 | 4 | 3 |
| mystery | Nigeria (Enugu) | 2 | 3 | 5 | 4 |
| mystery | Nigeria (Enugu) | 2 | 3 | 5 | 25 |
| mystery | Nigeria (Enugu) | 2 | 3 | 6 | 1 |
| mystery | Nigeria (Enugu) | 2 | 3 | 6 | 2 |
| mystery | Nigeria (Enugu) | 3 | 2 | 4 | 26 |
| mystery | Nigeria (Enugu) | 4 | 1 | 2 | 9 |
| mystery | Nigeria (Enugu) | 4 | 1 | 2 | 15 |
| mystery | Nigeria (Enugu) | 4 | 1 | 2 | 19 |
| mystery | Nigeria (Enugu) | 4 | 1 | 2 | 53 |
| mystery | Nigeria (Enugu) | 5 | 1 | 2 | 11 |
| mystery | Nigeria (Enugu) | 5 | 1 | 2 | 27 |
| mystery | Nigeria (Enugu) | 6 | 2 | 1 | 11 |
| mystery | Nigeria (Enugu) | 6 | 2 | 1 | 13 |
| mystery | Nigeria (Enugu) | 6 | 2 | 1 | 15 |
| mystery | Nigeria (Enugu) | 6 | 2 | 3 | 14 |
| mystery | Nigeria (Enugu) | 6 | 2 | 3 | 39 |
| mystery | Nigeria (Enugu) | 16 | 1 | 3 | 38 |
| mystery | Nigeria (Enugu) | 9 | 2 | 2 | 2 |
| mystery | Nigeria (Enugu) | 9 | 2 | 2 | 7 |
| mystery | Nigeria (Enugu) | 9 | 2 | 2 | 10 |

| | | | | | |
|---|---|---|---|---|---|
| mystery | Nigeria (Enugu) | 11 | 2 | 5 | 5 |
| mystery | Nigeria (Enugu) | 9 | 3 | 3 | 3 |
| mystery | Nigeria (Enugu) | 9 | 3 | 5 | 8 |
| mystery | Nigeria (Enugu) | 9 | 3 | 5 | 11 |
| mystery | Nigeria (Enugu) | 9 | 3 | 5 | 31 |
| mystery | Nigeria (Enugu) | 9 | 3 | 6 | 8 |
| mystery | Nigeria (Enugu) | 10 | 3 | 1 | 20 |
| mystery | Nigeria (Enugu) | 10 | 3 | 1 | 25 |
| mystery | Nigeria (Enugu) | 10 | 3 | 1 | 33 |
| mystery | Nigeria (Enugu) | 10 | 3 | 1 | 66 |
| mystery | Nigeria (Enugu) | 10 | 3 | 1 | 72 |
| mystery | Nigeria (Enugu) | 11 | 2 | 5 | 11 |
| mystery | Nigeria (Enugu) | 11 | 3 | 7 | 27 |
| mystery | Nigeria (Enugu) | 12 | 2 | 5 | 4 |
| mystery | Nigeria (Enugu) | 12 | 2 | 5 | 24 |
| mystery | Nigeria (Enugu) | 12 | 3 | 7 | 12 |
| mystery | Nigeria (Enugu) | 13 | 2 | 3 | 16 |
| mystery | Nigeria (Enugu) | 13 | 3 | 7 | 3 |
| mystery | Nigeria (Enugu) | 13 | 3 | 7 | 4 |
| mystery | Nigeria (Enugu) | 13 | 3 | 7 | 9 |
| mystery | Nigeria (Enugu) | 13 | 3 | 7 | 32 |
| mystery | Nigeria (Enugu) | 14 | 1 | 1 | 5 |
| mystery | Nigeria (Enugu) | 14 | 1 | 1 | 6 |
| mystery | Nigeria (Enugu) | 14 | 1 | 1 | 6 |
| mystery | Nigeria (Enugu) | 14 | 1 | 1 | 7 |
| mystery | Nigeria (Enugu) | 14 | 1 | 1 | 15 |
| mystery | Nigeria (Enugu) | 14 | 1 | 3 | 1 |
| mystery | Nigeria (Enugu) | 14 | 1 | 3 | 5 |
| mystery | Nigeria (Enugu) | 14 | 1 | 3 | 9 |
| mystery | Nigeria (Enugu) | 14 | 1 | 3 | 25 |
| mystery | Nigeria (Enugu) | 14 | 1 | 3 | 35 |
| mystery | Nigeria (Enugu) | 15 | 2 | 1 | 4 |
| mystery | Nigeria (Enugu) | 15 | 2 | 1 | 7 |
| mystery | Nigeria (Enugu) | 15 | 2 | 1 | 14 |
| mystery | Nigeria (Enugu) | 15 | 2 | 1 | 29 |
| mystery | Nigeria (Enugu) | 15 | 2 | 3 | 15 |
| mystery | Nigeria (Enugu) | 15 | 2 | 3 | 38 |
| mystery | Nigeria (Enugu) | 16 | 1 | 1 | 17 |
| mystery | Nigeria (Enugu) | 16 | 1 | 1 | 28 |
| mystery | Nigeria (Enugu) | 16 | 1 | 3 | 7 |
| mystery | Nigeria (Enugu) | 16 | 1 | 3 | 26 |
| mystery | Nigeria (Enugu) | 16 | 1 | 3 | 31 |
| mystery | Nigeria (Enugu) | 13 | 3 | 7 | 8 |
| mystery | Nigeria (Enugu) | 3 | 2 | 4 | 13 |
| mystery | Nigeria (Enugu) | 4 | 1 | 2 | 4 |
| mystery | Nigeria (Enugu) | 5 | 1 | 2 | 33 |
| mystery | Nigeria (Enugu) | 6 | 2 | 1 | 28 |
| mystery | Nigeria (Enugu) | 7 | 2 | 4 | 27 |
| mystery | Nigeria (Enugu) | 13 | 2 | 5 | 25 |
| mystery | Nigeria (Enugu) | 3 | 1 | 2 | 0 |

| | | | | | |
|---|---|---|---|---|---|
| mystery | Nigeria (Enugu) | 4 | 1 | 2 | 9 |
| mystery | Nigeria (Enugu) | 5 | 1 | 2 | 8 |
| mystery | Nigeria (Enugu) | 5 | 1 | 2 | 59 |
| mystery | Nigeria (Enugu) | 15 | 2 | 3 | 38 |
| mystery | Nigeria (Enugu) | 6 | 2 | 1 | 28 |
| mystery | Nigeria (Enugu) | 9 | 3 | 5 | 31 |
| mystery | Nigeria (Enugu) | 15 | 2 | 1 | 5 |
| mystery | Nigeria (Enugu) | 1 | 3 | 1 | 38 |
| mystery | Nigeria (Enugu) | 2 | 3 | 2 | 7 |
| mystery | Nigeria (Enugu) | 4 | 2 | 3 | 2 |
| mystery | Nigeria (Enugu) | 5 | 1 | 2 | 2 |
| mystery | Nigeria (Enugu) | 5 | 1 | 2 | 31 |
| mystery | Nigeria (Enugu) | 6 | 2 | 1 | 1 |
| mystery | Nigeria (Enugu) | 6 | 2 | 1 | 11 |
| mystery | Nigeria (Enugu) | 6 | 2 | 3 | 14 |
| mystery | Nigeria (Enugu) | 7 | 2 | 4 | 4 |
| mystery | Nigeria (Enugu) | 7 | 3 | 5 | 0 |
| mystery | Nigeria (Enugu) | 8 | 2 | 1 | 0 |
| mystery | Nigeria (Enugu) | 9 | 2 | 1 | 0 |
| mystery | Nigeria (Enugu) | 9 | 2 | 5 | 0 |
| mystery | Nigeria (Enugu) | 9 | 3 | 5 | 31 |
| mystery | Nigeria (Enugu) | 10 | 3 | 1 | 21 |
| mystery | Nigeria (Enugu) | 10 | 3 | 1 | 53 |
| mystery | Nigeria (Enugu) | 10 | 3 | 1 | 72 |
| mystery | Nigeria (Enugu) | 11 | 2 | 5 | 12 |
| mystery | Nigeria (Enugu) | 12 | 2 | 5 | 3 |
| mystery | Nigeria (Enugu) | 13 | 2 | 3 | 16 |
| mystery | Nigeria (Enugu) | 13 | 2 | 5 | 25 |
| mystery | Nigeria (Enugu) | 16 | 1 | 3 | 2 |
| mystery | Nigeria (Enugu) | 16 | 1 | 3 | 11 |
| mystery | Nigeria (Enugu) | 16 | 1 | 3 | 26 |
| mystery | Nigeria (Enugu) | 4 | 1 | 2 | 1 |
| mystery | Nigeria (Enugu) | 7 | 2 | 4 | 1 |
| mystery | Nigeria (Enugu) | 2 | 3 | 5 | 27 |
| mystery | Nigeria (Enugu) | 4 | 1 | 2 | 53 |
| mystery | Nigeria (Enugu) | 5 | 1 | 2 | 21 |
| mystery | Nigeria (Enugu) | 5 | 1 | 2 | 49 |
| mystery | Nigeria (Enugu) | 6 | 2 | 1 | 15 |
| mystery | Nigeria (Enugu) | 6 | 2 | 1 | 26 |
| mystery | Nigeria (Enugu) | 10 | 3 | 1 | 21 |
| mystery | Nigeria (Enugu) | 12 | 2 | 5 | 3 |
| mystery | Nigeria (Enugu) | 12 | 2 | 5 | 4 |
| mystery | Nigeria (Enugu) | 12 | 2 | 5 | 24 |
| mystery | Nigeria (Enugu) | 13 | 3 | 7 | 1 |
| mystery | Nigeria (Enugu) | 14 | 1 | 3 | 4 |
| mystery | Nigeria (Enugu) | 14 | 1 | 3 | 25 |
| mystery | Nigeria (Enugu) | 16 | 1 | 1 | 7 |
| mystery | Nigeria (Enugu) | 1 | 3 | 1 | 75 |
| mystery | Nigeria (Enugu) | 6 | 2 | 3 | 14 |
| mystery | Nigeria (Enugu) | 1 | 3 | 1 | 21 |

| | | | | | |
|---|---|---|---|---|---|
| mystery | Nigeria (Enugu) | 1 | 3 | 1 | 63 |
| mystery | Nigeria (Enugu) | 2 | 3 | 2 | 5 |
| mystery | Nigeria (Enugu) | 2 | 3 | 2 | 8 |
| mystery | Nigeria (Enugu) | 4 | 1 | 2 | 9 |
| mystery | Nigeria (Enugu) | 5 | 1 | 2 | 2 |
| mystery | Nigeria (Enugu) | 5 | 1 | 2 | 21 |
| mystery | Nigeria (Enugu) | 5 | 1 | 2 | 33 |
| mystery | Nigeria (Enugu) | 5 | 1 | 2 | 59 |
| mystery | Nigeria (Enugu) | 7 | 2 | 4 | 22 |
| mystery | Nigeria (Enugu) | 9 | 3 | 3 | 3 |
| mystery | Nigeria (Enugu) | 14 | 1 | 3 | 21 |
| mystery | Nigeria (Enugu) | 5 | 2 | 3 | 12 |
| mystery | Nigeria (Enugu) | 5 | 2 | 1 | 0 |
| mystery | Nigeria (Enugu) | 9 | 2 | 1 | 0 |
| mystery | Nigeria (Enugu) | 3 | 2 | 4 | 39 |
| mystery | Nigeria (Enugu) | 1 | 3 | 1 | 77 |
| mystery | Nigeria (Enugu) | 7 | 2 | 4 | 1 |
| mystery | Nigeria (Enugu) | 16 | 1 | 3 | 7 |
| mystery | Nigeria (Enugu) | 5 | 1 | 2 | 7 |
| mystery | Nigeria (Enugu) | 5 | 1 | 2 | 22 |
| mystery | Nigeria (Enugu) | 2 | 3 | 5 | 28 |
| mystery | Nigeria (Enugu) | 6 | 2 | 3 | 37 |
| mystery | Nigeria (Enugu) | 13 | 3 | 7 | 28 |
| mystery | Nigeria (Enugu) | 1 | 3 | 1 | 81 |
| mystery | Nigeria (Enugu) | 2 | 3 | 2 | 7 |
| mystery | Nigeria (Enugu) | 4 | 1 | 2 | 19 |
| mystery | Nigeria (Enugu) | 5 | 1 | 2 | 11 |
| mystery | Nigeria (Enugu) | 5 | 2 | 1 | 0 |
| mystery | Nigeria (Enugu) | 6 | 2 | 1 | 1 |
| mystery | Nigeria (Enugu) | 6 | 2 | 1 | 11 |
| mystery | Nigeria (Enugu) | 6 | 2 | 3 | 14 |
| mystery | Nigeria (Enugu) | 7 | 2 | 4 | 4 |
| mystery | Nigeria (Enugu) | 9 | 2 | 1 | 0 |
| mystery | Nigeria (Enugu) | 10 | 3 | 1 | 21 |
| mystery | Nigeria (Enugu) | 10 | 3 | 1 | 72 |
| mystery | Nigeria (Enugu) | 13 | 2 | 3 | 16 |
| mystery | Nigeria (Enugu) | 13 | 2 | 5 | 25 |
| mystery | Nigeria (Enugu) | 14 | 1 | 1 | 1 |
| mystery | Nigeria (Enugu) | 14 | 1 | 3 | 4 |
| mystery | Nigeria (Enugu) | 14 | 1 | 3 | 10 |
| mystery | Nigeria (Enugu) | 16 | 1 | 3 | 11 |
| mystery | Nigeria (Enugu) | 1 | 3 | 1 | 38 |
| mystery | Nigeria (Enugu) | 1 | 3 | 1 | 60 |
| mystery | Nigeria (Enugu) | 1 | 3 | 1 | 63 |
| mystery | Nigeria (Enugu) | 2 | 3 | 2 | 7 |
| mystery | Nigeria (Enugu) | 2 | 3 | 5 | 33 |
| mystery | Nigeria (Enugu) | 3 | 2 | 4 | 21 |
| mystery | Nigeria (Enugu) | 3 | 2 | 4 | 39 |
| mystery | Nigeria (Enugu) | 3 | 2 | 4 | 42 |
| mystery | Nigeria (Enugu) | 3 | 3 | 71 | 0 |

| | | | | | |
|---|---|---|---|---|---|
| mystery | Nigeria (Enugu) | 4 | 1 | 2 | 4 |
| mystery | Nigeria (Enugu) | 4 | 1 | 2 | 18 |
| mystery | Nigeria (Enugu) | 4 | 1 | 2 | 35 |
| mystery | Nigeria (Enugu) | 4 | 1 | 2 | 53 |
| mystery | Nigeria (Enugu) | 5 | 1 | 2 | 0 |
| mystery | Nigeria (Enugu) | 5 | 1 | 2 | 7 |
| mystery | Nigeria (Enugu) | 6 | 2 | 1 | 1 |
| mystery | Nigeria (Enugu) | 6 | 2 | 3 | 14 |
| mystery | Nigeria (Enugu) | 6 | 2 | 3 | 25 |
| mystery | Nigeria (Enugu) | 7 | 2 | 4 | 1 |
| mystery | Nigeria (Enugu) | 7 | 2 | 4 | 27 |
| mystery | Nigeria (Enugu) | 7 | 3 | 5 | 0 |
| mystery | Nigeria (Enugu) | 8 | 2 | 1 | 0 |
| mystery | Nigeria (Enugu) | 8 | 2 | 4 | 17 |
| mystery | Nigeria (Enugu) | 8 | 2 | 4 | 23 |
| mystery | Nigeria (Enugu) | 8 | 2 | 4 | 44 |
| mystery | Nigeria (Enugu) | 9 | 2 | 1 | 0 |
| mystery | Nigeria (Enugu) | 9 | 3 | 5 | 8 |
| mystery | Nigeria (Enugu) | 9 | 3 | 5 | 20 |
| mystery | Nigeria (Enugu) | 9 | 3 | 5 | 31 |
| mystery | Nigeria (Enugu) | 10 | 3 | 1 | 1 |
| mystery | Nigeria (Enugu) | 10 | 3 | 1 | 14 |
| mystery | Nigeria (Enugu) | 10 | 3 | 1 | 21 |
| mystery | Nigeria (Enugu) | 10 | 3 | 1 | 43 |
| mystery | Nigeria (Enugu) | 10 | 3 | 1 | 53 |
| mystery | Nigeria (Enugu) | 10 | 3 | 1 | 78 |
| mystery | Nigeria (Enugu) | 11 | 3 | 7 | 14 |
| mystery | Nigeria (Enugu) | 12 | 2 | 5 | 4 |
| mystery | Nigeria (Enugu) | 12 | 3 | 7 | 6 |
| mystery | Nigeria (Enugu) | 13 | 2 | 3 | 16 |
| mystery | Nigeria (Enugu) | 13 | 2 | 5 | 25 |
| mystery | Nigeria (Enugu) | 13 | 3 | 7 | 8 |
| mystery | Nigeria (Enugu) | 14 | 1 | 1 | 1 |
| mystery | Nigeria (Enugu) | 14 | 1 | 1 | 15 |
| mystery | Nigeria (Enugu) | 14 | 1 | 3 | 4 |
| mystery | Nigeria (Enugu) | 15 | 2 | 1 | 4 |
| mystery | Nigeria (Enugu) | 15 | 2 | 1 | 14 |
| mystery | Nigeria (Enugu) | 15 | 2 | 3 | 30 |
| mystery | Nigeria (Enugu) | 16 | 1 | 3 | 2 |
| mystery | Nigeria (Enugu) | 1 | 3 | 1 | 38 |
| mystery | Nigeria (Enugu) | 3 | 2 | 6 | 6 |
| mystery | Nigeria (Enugu) | 4 | 1 | 2 | 19 |
| mystery | Nigeria (Enugu) | 7 | 2 | 4 | 20 |
| mystery | Nigeria (Enugu) | 16 | 1 | 3 | 26 |
| mystery | Nigeria (Enugu) | 4 | 1 | 2 | 9 |
| mystery | Nigeria (Enugu) | 7 | 2 | 4 | 20 |
| mystery | Nigeria (Enugu) | 1 | 3 | 1 | 42 |
| mystery | Nigeria (Enugu) | 2 | 3 | 5 | 28 |
| mystery | Nigeria (Enugu) | 2 | 3 | 6 | 1 |
| mystery | Nigeria (Enugu) | 3 | 2 | 4 | 11 |

| | | | | | |
|---|---|---|---|---|---|
| mystery | Nigeria (Enugu) | 3 | 22 | 6 | 6 |
| mystery | Nigeria (Enugu) | 4 | 1 | 2 | 9 |
| mystery | Nigeria (Enugu) | 4 | 1 | 2 | 12 |
| mystery | Nigeria (Enugu) | 4 | 1 | 2 | 53 |
| mystery | Nigeria (Enugu) | 5 | 1 | 2 | 8 |
| mystery | Nigeria (Enugu) | 6 | 2 | 3 | 14 |
| mystery | Nigeria (Enugu) | 6 | 2 | 3 | 37 |
| mystery | Nigeria (Enugu) | 10 | 3 | 1 | 5 |
| mystery | Nigeria (Enugu) | 11 | 2 | 5 | 12 |
| mystery | Nigeria (Enugu) | 12 | 3 | 7 | 6 |
| mystery | Nigeria (Enugu) | 13 | 2 | 3 | 16 |
| mystery | Nigeria (Enugu) | 14 | 1 | 1 | 1 |
| mystery | Nigeria (Enugu) | 14 | 1 | 1 | 15 |
| mystery | Nigeria (Enugu) | 14 | 1 | 3 | 1 |
| mystery | Nigeria (Enugu) | 15 | 2 | 3 | 10 |
| mystery | Nigeria (Enugu) | 15 | 2 | 3 | 11 |
| mystery | Nigeria (Enugu) | 15 | 2 | 3 | 30 |
| mystery | Nigeria (Enugu) | 15 | 2 | 3 | 38 |
| mystery | Nigeria (Enugu) | 16 | 1 | 3 | 2 |
| mystery | Nigeria (Enugu) | 16 | 1 | 3 | 14 |
| mystery | Nigeria (Enugu) | 16 | 1 | 3 | 30 |
| mystery | Nigeria (Enugu) | 1 | 3 | 1 | 12 |
| mystery | Nigeria (Enugu) | 1 | 3 | 1 | 13 |
| mystery | Nigeria (Enugu) | 1 | 3 | 1 | 42 |
| mystery | Nigeria (Enugu) | 3 | 2 | 1 | 2 |
| mystery | Nigeria (Enugu) | 3 | 2 | 4 | 11 |
| mystery | Nigeria (Enugu) | 3 | 2 | 4 | 21 |
| mystery | Nigeria (Enugu) | 3 | 2 | 4 | 39 |
| mystery | Nigeria (Enugu) | 4 | 1 | 2 | 6 |
| mystery | Nigeria (Enugu) | 6 | 2 | 1 | 13 |
| mystery | Nigeria (Enugu) | 6 | 2 | 3 | 6 |
| mystery | Nigeria (Enugu) | 7 | 2 | 4 | 25 |
| mystery | Nigeria (Enugu) | 9 | 2 | 2 | 1 |
| mystery | Nigeria (Enugu) | 10 | 3 | 1 | 5 |
| mystery | Nigeria (Enugu) | 10 | 3 | 1 | 71 |
| mystery | Nigeria (Enugu) | 11 | 2 | 5 | 12 |
| mystery | Nigeria (Enugu) | 13 | 2 | 3 | 21 |
| mystery | Nigeria (Enugu) | 13 | 3 | 7 | 8 |
| mystery | Nigeria (Enugu) | 13 | 3 | 7 | 28 |
| mystery | Nigeria (Enugu) | 15 | 2 | 3 | 10 |
| mystery | Nigeria (Enugu) | 15 | 2 | 3 | 38 |
| mystery | Nigeria (Enugu) | 16 | 1 | 3 | 7 |
| mystery | Nigeria (Enugu) | 16 | 1 | 3 | 38 |
| mystery | Nigeria (Enugu) | 10 | 3 | 1 | 21 |
| mystery | Nigeria (Enugu) | 4 | 1 | 2 | 8 |
| mystery | Nigeria (Enugu) | 11 | 2 | 5 | 5 |
| mystery | Nigeria (Enugu) | 14 | 1 | 3 | 21 |
| mystery | Nigeria (Enugu) | 9 | 2 | 1 | 0 |
| mystery | Nigeria (Enugu) | 4 | 2 | 3 | 3 |
| mystery | Nigeria (Enugu) | 5 | 1 | 2 | 2 |

| | | | | | |
|---|---|---|---|---|---|
| mystery | Nigeria (Enugu) | 10 | 3 | 1 | 21 |
| mystery | Nigeria (Enugu) | 3 | 2 | 6 | 6 |
| mystery | Nigeria (Enugu) | 9 | 3 | 5 | 31 |
| mystery | Nigeria (Enugu) | 2 | 3 | 5 | 28 |
| mystery | Nigeria (Enugu) | 2 | 3 | 6 | 1 |
| mystery | Nigeria (Enugu) | 6 | 2 | 1 | 13 |
| mystery | Nigeria (Enugu) | 6 | 2 | 3 | 37 |
| mystery | Nigeria (Enugu) | 13 | 2 | 3 | 16 |
| mystery | Nigeria (Enugu) | 14 | 1 | 1 | 7 |
| mystery | Nigeria (Enugu) | 15 | 2 | 3 | 10 |
| mystery | Nigeria (Enugu) | 15 | 2 | 3 | 30 |
| mystery | Nigeria (Enugu) | 1 | 3 | 1 | 80 |
| mystery | Nigeria (Enugu) | 2 | 3 | 6 | 1 |
| mystery | Nigeria (Enugu) | 4 | 1 | 2 | 23 |
| mystery | Nigeria (Enugu) | 4 | 2 | 3 | 3 |
| mystery | Nigeria (Enugu) | 5 | 1 | 2 | 2 |
| mystery | Nigeria (Enugu) | 5 | 1 | 2 | 7 |
| mystery | Nigeria (Enugu) | 5 | 1 | 2 | 17 |
| mystery | Nigeria (Enugu) | 5 | 2 | 3 | 7 |
| mystery | Nigeria (Enugu) | 6 | 2 | 1 | 13 |
| mystery | Nigeria (Enugu) | 6 | 2 | 3 | 9 |
| mystery | Nigeria (Enugu) | 6 | 2 | 3 | 14 |
| mystery | Nigeria (Enugu) | 6 | 2 | 3 | 37 |
| mystery | Nigeria (Enugu) | 9 | 3 | 5 | 31 |
| mystery | Nigeria (Enugu) | 10 | 3 | 1 | 14 |
| mystery | Nigeria (Enugu) | 13 | 2 | 5 | 9 |
| mystery | Nigeria (Enugu) | 13 | 3 | 7 | 3 |
| mystery | Nigeria (Enugu) | 14 | 1 | 1 | 15 |
| mystery | Nigeria (Enugu) | 14 | 1 | 3 | 1 |
| mystery | Nigeria (Enugu) | 14 | 1 | 3 | 9 |
| mystery | Nigeria (Enugu) | 14 | 1 | 3 | 23 |
| mystery | Nigeria (Enugu) | 14 | 1 | 3 | 35 |
| mystery | Nigeria (Enugu) | 16 | 1 | 1 | 28 |
| mystery | Nigeria (Enugu) | 16 | 1 | 3 | 27 |
| mystery | Nigeria (Enugu) | 16 | 1 | 3 | 30 |
| mystery | Nigeria (Enugu) | 16 | 1 | 3 | 38 |
| mystery | Nigeria (Enugu) | 1 | 3 | 1 | 13 |
| mystery | Nigeria (Enugu) | 1 | 3 | 1 | 21 |
| mystery | Nigeria (Enugu) | 4 | 1 | 2 | 9 |
| mystery | Nigeria (Enugu) | 5 | 1 | 2 | 8 |
| mystery | Nigeria (Enugu) | 5 | 1 | 2 | 17 |
| mystery | Nigeria (Enugu) | 6 | 2 | 3 | 9 |
| mystery | Nigeria (Enugu) | 11 | 2 | 5 | 12 |
| mystery | Nigeria (Enugu) | 11 | 2 | 5 | 26 |
| mystery | Nigeria (Enugu) | 15 | 2 | 3 | 38 |
| mystery | Nigeria (Enugu) | 1 | 3 | 1 | 74 |
| mystery | Nigeria (Enugu) | 5 | 1 | 2 | 8 |
| mystery | Nigeria (Enugu) | 14 | 1 | 3 | 4 |
| mystery | Nigeria (Enugu) | 1 | 3 | 1 | 13 |
| mystery | Nigeria (Enugu) | 1 | 3 | 1 | 21 |

| | | | | | |
|---|---|---|---|---|---|
| mystery | Nigeria (Enugu) | 1 | 3 | 1 | 42 |
| mystery | Nigeria (Enugu) | 2 | 3 | 6 | 1 |
| mystery | Nigeria (Enugu) | 3 | 2 | 4 | 14 |
| mystery | Nigeria (Enugu) | 5 | 1 | 2 | 2 |
| mystery | Nigeria (Enugu) | 5 | 1 | 2 | 57 |
| mystery | Nigeria (Enugu) | 6 | 2 | 1 | 11 |
| mystery | Nigeria (Enugu) | 7 | 2 | 4 | 1 |
| mystery | Nigeria (Enugu) | 7 | 2 | 4 | 4 |
| mystery | Nigeria (Enugu) | 8 | 2 | 4 | 5 |
| mystery | Nigeria (Enugu) | 10 | 3 | 1 | 2 |
| mystery | Nigeria (Enugu) | 10 | 3 | 1 | 33 |
| mystery | Nigeria (Enugu) | 11 | 2 | 5 | 6 |
| mystery | Nigeria (Enugu) | 11 | 2 | 5 | 12 |
| mystery | Nigeria (Enugu) | 11 | 2 | 5 | 22 |
| mystery | Nigeria (Enugu) | 11 | 2 | 5 | 26 |
| mystery | Nigeria (Enugu) | 12 | 3 | 7 | 6 |
| mystery | Nigeria (Enugu) | 13 | 3 | 7 | 28 |
| mystery | Nigeria (Enugu) | 13 | 3 | 7 | 33 |
| mystery | Nigeria (Enugu) | 14 | 1 | 1 | 1 |
| mystery | Nigeria (Enugu) | 14 | 1 | 1 | 20 |
| mystery | Nigeria (Enugu) | 14 | 1 | 3 | 1 |
| mystery | Nigeria (Enugu) | 14 | 1 | 3 | 29 |
| mystery | Nigeria (Enugu) | 14 | 1 | 3 | 35 |
| mystery | Nigeria (Enugu) | 15 | 2 | 1 | 7 |
| mystery | Nigeria (Enugu) | 15 | 2 | 1 | 14 |
| mystery | Nigeria (Enugu) | 15 | 2 | 3 | 10 |
| mystery | Nigeria (Enugu) | 15 | 2 | 3 | 11 |
| mystery | Nigeria (Enugu) | 15 | 2 | 3 | 15 |
| mystery | Nigeria (Enugu) | 15 | 2 | 3 | 28 |
| mystery | Nigeria (Enugu) | 15 | 2 | 3 | 38 |
| mystery | Nigeria (Enugu) | 16 | 1 | 3 | 7 |
| mystery | Nigeria (Enugu) | 16 | 1 | 3 | 38 |
| mystery | Nigeria (Enugu) | 5 | 1 | 2 | 7 |
| mystery | Nigeria (Enugu) | 11 | 2 | 5 | 12 |
| mystery | Nigeria (Enugu) | 1 | 3 | 1 | 68 |
| mystery | Nigeria (Enugu) | 3 | 2 | 6 | 6 |
| mystery | Nigeria (Enugu) | 4 | 1 | 2 | 9 |
| mystery | Nigeria (Enugu) | 4 | 1 | 2 | 19 |
| mystery | Nigeria (Enugu) | 5 | 1 | 2 | 57 |
| mystery | Nigeria (Enugu) | 9 | 3 | 5 | 31 |
| mystery | Nigeria (Enugu) | 7 | 2 | 4 | 1 |
| mystery | Nigeria (Enugu) | 14 | 1 | 3 | 29 |
| mystery | Nigeria (Enugu) | 4 | 1 | 2 | 1 |
| mystery | Nigeria (Enugu) | 7 | 2 | 4 | 1 |
| mystery | Nigeria (Enugu) | 5 | 1 | 2 | 2 |
| mystery | Nigeria (Enugu) | 5 | 1 | 2 | 8 |
| mystery | Nigeria (Enugu) | 5 | 1 | 2 | 17 |
| mystery | Nigeria (Enugu) | 6 | 2 | 1 | 11 |
| mystery | Nigeria (Enugu) | 6 | 2 | 3 | 6 |
| mystery | Nigeria (Enugu) | 7 | 2 | 4 | 20 |

| | | | | | |
|---|---|---|---|---|---|
| mystery | Nigeria (Enugu) | 11 | 2 | 5 | 12 |
| mystery | Nigeria (Enugu) | 14 | 1 | 3 | 35 |
| mystery | Nigeria (Enugu) | 15 | 2 | 1 | 29 |
| mystery | Nigeria (Enugu) | 10 | 3 | 1 | 21 |
| mystery | Nigeria (Enugu) | 1 | 2 | 4 | 1 |
| mystery | Nigeria (Enugu) | 1 | 3 | 1 | 59 |
| mystery | Nigeria (Enugu) | 4 | 1 | 2 | 4 |
| mystery | Nigeria (Enugu) | 4 | 1 | 2 | 9 |
| mystery | Nigeria (Enugu) | 5 | 2 | 3 | 7 |
| mystery | Nigeria (Enugu) | 6 | 2 | 1 | 11 |
| mystery | Nigeria (Enugu) | 6 | 2 | 3 | 6 |
| mystery | Nigeria (Enugu) | 8 | 2 | 4 | 5 |
| mystery | Nigeria (Enugu) | 11 | 2 | 5 | 11 |
| mystery | Nigeria (Enugu) | 11 | 2 | 5 | 12 |
| mystery | Nigeria (Enugu) | 12 | 3 | 7 | 6 |
| mystery | Nigeria (Enugu) | 13 | 3 | 7 | 7 |
| mystery | Nigeria (Enugu) | 14 | 1 | 3 | 1 |
| mystery | Nigeria (Enugu) | 15 | 2 | 1 | 29 |
| mystery | Nigeria (Enugu) | 15 | 2 | 3 | 10 |
| mystery | Nigeria (Enugu) | 15 | 2 | 3 | 11 |
| mystery | Nigeria (Enugu) | 15 | 2 | 3 | 38 |
| mystery | Nigeria (Enugu) | 16 | 1 | 1 | 28 |
| mystery | Nigeria (Enugu) | 16 | 1 | 3 | 38 |
| mystery | Nigeria (Enugu) | 2 | 3 | 5 | 33 |
| mystery | Nigeria (Enugu) | 4 | 1 | 2 | 9 |
| mystery | Nigeria (Enugu) | 4 | 1 | 2 | 23 |
| mystery | Nigeria (Enugu) | 7 | 2 | 4 | 22 |
| mystery | Nigeria (Enugu) | 9 | 2 | 2 | 1 |
| mystery | Nigeria (Enugu) | 13 | 2 | 3 | 16 |
| mystery | Nigeria (Enugu) | 14 | 1 | 1 | 15 |
| mystery | Nigeria (Enugu) | 14 | 1 | 3 | 4 |
| mystery | Nigeria (Enugu) | 14 | 1 | 3 | 5 |
| mystery | Nigeria (Enugu) | 15 | 2 | 3 | 38 |
| mystery | Nigeria (Enugu) | 4 | 1 | 2 | 1 |
| mystery | Nigeria (Enugu) | 5 | 2 | 3 | 7 |
| mystery | Nigeria (Enugu) | 13 | 3 | 7 | 1 |
| mystery | Nigeria (Enugu) | 2 | 3 | 5 | 27 |
| mystery | Nigeria (Enugu) | 4 | 1 | 2 | 9 |
| mystery | Nigeria (Enugu) | 5 | 1 | 2 | 2 |
| mystery | Nigeria (Enugu) | 1 | 3 | 1 | 10 |
| mystery | Nigeria (Enugu) | 1 | 3 | 1 | 12 |
| mystery | Nigeria (Enugu) | 1 | 3 | 1 | 13 |
| mystery | Nigeria (Enugu) | 1 | 3 | 1 | 36 |
| mystery | Nigeria (Enugu) | 1 | 3 | 1 | 59 |
| mystery | Nigeria (Enugu) | 1 | 3 | 1 | 68 |
| mystery | Nigeria (Enugu) | 1 | 3 | 1 | 69 |
| mystery | Nigeria (Enugu) | 1 | 3 | 1 | 70 |
| mystery | Nigeria (Enugu) | 1 | 3 | 1 | 76 |
| mystery | Nigeria (Enugu) | 1 | 3 | 1 | 80 |
| mystery | Nigeria (Enugu) | 1 | 3 | 1 | 81 |

| | | | | | |
|---|---|---|---|---|---|
| mystery | Nigeria (Enugu) | 1 | 3 | 1 | 82 |
| mystery | Nigeria (Enugu) | 1 | 3 | 1 | 84 |
| mystery | Nigeria (Enugu) | 2 | 3 | 2 | 5 |
| mystery | Nigeria (Enugu) | 2 | 3 | 2 | 7 |
| mystery | Nigeria (Enugu) | 2 | 3 | 2 | 8 |
| mystery | Nigeria (Enugu) | 2 | 3 | 4 | 3 |
| mystery | Nigeria (Enugu) | 2 | 3 | 5 | 4 |
| mystery | Nigeria (Enugu) | 2 | 3 | 5 | 25 |
| mystery | Nigeria (Enugu) | 2 | 3 | 6 | 1 |
| mystery | Nigeria (Enugu) | 3 | 2 | 4 | 2 |
| mystery | Nigeria (Enugu) | 3 | 2 | 4 | 7 |
| mystery | Nigeria (Enugu) | 3 | 2 | 4 | 11 |
| mystery | Nigeria (Enugu) | 3 | 2 | 4 | 15 |
| mystery | Nigeria (Enugu) | 3 | 2 | 4 | 21 |
| mystery | Nigeria (Enugu) | 3 | 2 | 4 | 26 |
| mystery | Nigeria (Enugu) | 3 | 2 | 4 | 43 |
| mystery | Nigeria (Enugu) | 3 | 2 | 6 | 6 |
| mystery | Nigeria (Enugu) | 4 | 1 | 2 | 4 |
| mystery | Nigeria (Enugu) | 4 | 1 | 2 | 5 |
| mystery | Nigeria (Enugu) | 4 | 1 | 2 | 8 |
| mystery | Nigeria (Enugu) | 4 | 1 | 2 | 9 |
| mystery | Nigeria (Enugu) | 4 | 1 | 2 | 15 |
| mystery | Nigeria (Enugu) | 4 | 1 | 2 | 19 |
| mystery | Nigeria (Enugu) | 4 | 1 | 2 | 23 |
| mystery | Nigeria (Enugu) | 4 | 1 | 2 | 35 |
| mystery | Nigeria (Enugu) | 4 | 1 | 2 | 36 |
| mystery | Nigeria (Enugu) | 4 | 1 | 2 | 53 |
| mystery | Nigeria (Enugu) | 5 | 1 | 2 | 2 |
| mystery | Nigeria (Enugu) | 5 | 1 | 2 | 7 |
| mystery | Nigeria (Enugu) | 5 | 1 | 2 | 8 |
| mystery | Nigeria (Enugu) | 5 | 1 | 2 | 10 |
| mystery | Nigeria (Enugu) | 5 | 1 | 2 | 21 |
| mystery | Nigeria (Enugu) | 5 | 1 | 2 | 22 |
| mystery | Nigeria (Enugu) | 5 | 1 | 2 | 30 |
| mystery | Nigeria (Enugu) | 5 | 1 | 2 | 31 |
| mystery | Nigeria (Enugu) | 5 | 1 | 2 | 33 |
| mystery | Nigeria (Enugu) | 5 | 1 | 2 | 46 |
| mystery | Nigeria (Enugu) | 5 | 1 | 2 | 49 |
| mystery | Nigeria (Enugu) | 5 | 1 | 2 | 56 |
| mystery | Nigeria (Enugu) | 5 | 1 | 2 | 59 |
| mystery | Nigeria (Enugu) | 5 | 1 | 2 | 59 |
| mystery | Nigeria (Enugu) | 5 | 2 | 3 | 7 |
| mystery | Nigeria (Enugu) | 5 | 2 | 3 | 12 |
| mystery | Nigeria (Enugu) | 6 | 2 | 1 | 1 |
| mystery | Nigeria (Enugu) | 6 | 2 | 1 | 13 |
| mystery | Nigeria (Enugu) | 6 | 2 | 1 | 15 |
| mystery | Nigeria (Enugu) | 6 | 2 | 1 | 28 |
| mystery | Nigeria (Enugu) | 6 | 2 | 3 | 1 |
| mystery | Nigeria (Enugu) | 6 | 2 | 3 | 14 |
| mystery | Nigeria (Enugu) | 6 | 2 | 3 | 25 |

| | | | | | |
|---|---|---|---|---|---|
| mystery | Nigeria (Enugu) | 6 | 2 | 3 | 37 |
| mystery | Nigeria (Enugu) | 7 | 2 | 4 | 1 |
| mystery | Nigeria (Enugu) | 7 | 2 | 4 | 22 |
| mystery | Nigeria (Enugu) | 8 | 2 | 1 | 8 |
| mystery | Nigeria (Enugu) | 8 | 2 | 4 | 5 |
| mystery | Nigeria (Enugu) | 8 | 2 | 4 | 5 |
| mystery | Nigeria (Enugu) | 8 | 2 | 4 | 10 |
| mystery | Nigeria (Enugu) | 8 | 2 | 4 | 16 |
| mystery | Nigeria (Enugu) | 8 | 2 | 4 | 17 |
| mystery | Nigeria (Enugu) | 8 | 2 | 4 | 23 |
| mystery | Nigeria (Enugu) | 8 | 2 | 4 | 33 |
| mystery | Nigeria (Enugu) | 8 | 2 | 4 | 44 |
| mystery | Nigeria (Enugu) | 9 | 2 | 2 | 1 |
| mystery | Nigeria (Enugu) | 9 | 2 | 2 | 7 |
| mystery | Nigeria (Enugu) | 9 | 3 | 5 | 11 |
| mystery | Nigeria (Enugu) | 9 | 3 | 5 | 16 |
| mystery | Nigeria (Enugu) | 9 | 3 | 5 | 19 |
| mystery | Nigeria (Enugu) | 10 | 3 | 1 | 1 |
| mystery | Nigeria (Enugu) | 10 | 3 | 1 | 5 |
| mystery | Nigeria (Enugu) | 10 | 3 | 1 | 14 |
| mystery | Nigeria (Enugu) | 10 | 3 | 1 | 15 |
| mystery | Nigeria (Enugu) | 10 | 3 | 1 | 20 |
| mystery | Nigeria (Enugu) | 10 | 3 | 1 | 25 |
| mystery | Nigeria (Enugu) | 10 | 3 | 1 | 33 |
| mystery | Nigeria (Enugu) | 10 | 3 | 1 | 37 |
| mystery | Nigeria (Enugu) | 10 | 3 | 1 | 53 |
| mystery | Nigeria (Enugu) | 10 | 3 | 1 | 65 |
| mystery | Nigeria (Enugu) | 10 | 3 | 1 | 79 |
| mystery | Nigeria (Enugu) | 11 | 2 | 5 | 1 |
| mystery | Nigeria (Enugu) | 11 | 2 | 5 | 5 |
| mystery | Nigeria (Enugu) | 11 | 2 | 5 | 22 |
| mystery | Nigeria (Enugu) | 11 | 2 | 5 | 26 |
| mystery | Nigeria (Enugu) | 11 | 3 | 7 | 14 |
| mystery | Nigeria (Enugu) | 11 | 3 | 7 | 15 |
| mystery | Nigeria (Enugu) | 11 | 3 | 7 | 24 |
| mystery | Nigeria (Enugu) | 11 | 3 | 7 | 27 |
| mystery | Nigeria (Enugu) | 12 | 2 | 5 | 2 |
| mystery | Nigeria (Enugu) | 12 | 2 | 5 | 10 |
| mystery | Nigeria (Enugu) | 12 | 2 | 5 | 16 |
| mystery | Nigeria (Enugu) | 12 | 2 | 5 | 24 |
| mystery | Nigeria (Enugu) | 12 | 3 | 7 | 6 |
| mystery | Nigeria (Enugu) | 12 | 3 | 7 | 23 |
| mystery | Nigeria (Enugu) | 13 | 2 | 3 | 16 |
| mystery | Nigeria (Enugu) | 13 | 2 | 3 | 21 |
| mystery | Nigeria (Enugu) | 13 | 2 | 5 | 25 |
| mystery | Nigeria (Enugu) | 13 | 3 | 7 | 1 |
| mystery | Nigeria (Enugu) | 13 | 3 | 7 | 4 |
| mystery | Nigeria (Enugu) | 13 | 3 | 7 | 7 |
| mystery | Nigeria (Enugu) | 13 | 3 | 7 | 8 |
| mystery | Nigeria (Enugu) | 13 | 3 | 7 | 28 |

| | | | | | |
|---|---|---|---|---|---|
| mystery | Nigeria (Enugu) | 13 | 3 | 7 | 31 |
| mystery | Nigeria (Enugu) | 13 | 3 | 7 | 32 |
| mystery | Nigeria (Enugu) | 13 | 3 | 7 | 33 |
| mystery | Nigeria (Enugu) | 14 | 1 | 1 | 1 |
| mystery | Nigeria (Enugu) | 14 | 1 | 1 | 5 |
| mystery | Nigeria (Enugu) | 14 | 1 | 1 | 6 |
| mystery | Nigeria (Enugu) | 14 | 1 | 1 | 7 |
| mystery | Nigeria (Enugu) | 14 | 1 | 1 | 15 |
| mystery | Nigeria (Enugu) | 14 | 1 | 1 | 16 |
| mystery | Nigeria (Enugu) | 14 | 1 | 1 | 18 |
| mystery | Nigeria (Enugu) | 14 | 1 | 1 | 20 |
| mystery | Nigeria (Enugu) | 14 | 1 | 3 | 5 |
| mystery | Nigeria (Enugu) | 14 | 1 | 3 | 9 |
| mystery | Nigeria (Enugu) | 14 | 1 | 3 | 21 |
| mystery | Nigeria (Enugu) | 14 | 1 | 3 | 23 |
| mystery | Nigeria (Enugu) | 14 | 1 | 3 | 25 |
| mystery | Nigeria (Enugu) | 14 | 1 | 3 | 32 |
| mystery | Nigeria (Enugu) | 14 | 1 | 3 | 35 |
| mystery | Nigeria (Enugu) | 15 | 2 | 1 | 4 |
| mystery | Nigeria (Enugu) | 15 | 2 | 1 | 5 |
| mystery | Nigeria (Enugu) | 15 | 2 | 1 | 7 |
| mystery | Nigeria (Enugu) | 15 | 2 | 1 | 14 |
| mystery | Nigeria (Enugu) | 15 | 2 | 3 | 10 |
| mystery | Nigeria (Enugu) | 15 | 2 | 3 | 15 |
| mystery | Nigeria (Enugu) | 15 | 2 | 3 | 20 |
| mystery | Nigeria (Enugu) | 15 | 2 | 3 | 24 |
| mystery | Nigeria (Enugu) | 15 | 2 | 3 | 26 |
| mystery | Nigeria (Enugu) | 15 | 2 | 3 | 30 |
| mystery | Nigeria (Enugu) | 15 | 2 | 3 | 34 |
| mystery | Nigeria (Enugu) | 15 | 2 | 3 | 38 |
| mystery | Nigeria (Enugu) | 16 | 1 | 1 | 17 |
| mystery | Nigeria (Enugu) | 16 | 1 | 3 | 7 |
| mystery | Nigeria (Enugu) | 16 | 1 | 3 | 11 |
| mystery | Nigeria (Enugu) | 16 | 1 | 3 | 14 |
| mystery | Nigeria (Enugu) | 16 | 1 | 3 | 26 |
| mystery | Nigeria (Enugu) | 16 | 1 | 3 | 30 |
| mystery | Nigeria (Enugu) | 16 | 1 | 3 | 31 |
| mystery | Nigeria (Enugu) | 16 | 1 | 3 | 38 |
| mystery | Nigeria (Enugu) | 4 | 1 | 2 | 53 |
| mystery | Nigeria (Enugu) | 5 | 1 | 2 | 2 |
| mystery | Nigeria (Enugu) | 5 | 1 | 2 | 8 |
| mystery | Nigeria (Enugu) | 10 | 3 | 1 | 14 |
| mystery | Nigeria (Enugu) | 11 | 2 | 5 | 22 |
| mystery | Nigeria (Enugu) | 15 | 2 | 1 | 4 |
| mystery | Nigeria (Enugu) | 4 | 1 | 2 | 4 |
| mystery | Nigeria (Enugu) | 4 | 1 | 2 | 9 |
| mystery | Nigeria (Enugu) | 4 | 1 | 2 | 53 |
| mystery | Nigeria (Enugu) | 5 | 1 | 2 | 8 |
| mystery | Nigeria (Enugu) | 5 | 2 | 3 | 12 |
| mystery | Nigeria (Enugu) | 15 | 2 | 1 | 17 |

| | | | | | |
|---|---|---|---|---|---|
| mystery | Nigeria (Enugu) | 1 | 3 | 1 | 59 |
| mystery | Nigeria (Enugu) | 13 | 3 | 7 | 7 |
| mystery | Nigeria (Enugu) | 3 | 2 | 4 | 21 |
| mystery | Nigeria (Enugu) | 6 | 2 | 1 | 11 |
| mystery | Nigeria (Enugu) | 6 | 2 | 1 | 28 |
| mystery | Nigeria (Enugu) | 4 | 1 | 2 | 53 |
| mystery | Nigeria (Enugu) | 5 | 1 | 2 | 8 |
| mystery | Nigeria (Enugu) | 13 | 3 | 7 | 3 |
| mystery | Nigeria (Enugu) | 14 | 1 | 1 | 18 |
| mystery | Nigeria (Enugu) | 15 | 2 | 3 | 38 |
| mystery | Nigeria (Enugu) | 8 | 2 | 1 | 8 |
| mystery | Nigeria (Enugu) | 5 | 1 | 2 | 2 |
| mystery | Nigeria (Enugu) | 14 | 1 | 3 | 1 |
| mystery | Nigeria (Enugu) | 11 | 2 | 5 | 5 |
| mystery | Nigeria (Enugu) | 15 | 2 | 1 | 29 |
| mystery | Nigeria (Enugu) | 15 | 2 | 3 | 26 |
| mystery | Nigeria (Enugu) | 4 | 1 | 2 | 9 |
| mystery | Nigeria (Enugu) | 5 | 2 | 3 | 12 |
| mystery | Nigeria (Enugu) | 6 | 2 | 3 | 1 |
| mystery | Nigeria (Enugu) | 6 | 2 | 3 | 6 |
| mystery | Nigeria (Enugu) | 1 | 3 | 1 | 10 |
| mystery | Nigeria (Enugu) | 1 | 3 | 1 | 13 |
| mystery | Nigeria (Enugu) | 1 | 3 | 1 | 38 |
| mystery | Nigeria (Enugu) | 1 | 3 | 1 | 42 |
| mystery | Nigeria (Enugu) | 1 | 3 | 1 | 59 |
| mystery | Nigeria (Enugu) | 1 | 3 | 1 | 75 |
| mystery | Nigeria (Enugu) | 1 | 3 | 1 | 83 |
| mystery | Nigeria (Enugu) | 1 | 3 | 1 | 84 |
| mystery | Nigeria (Enugu) | 2 | 3 | 2 | 5 |
| mystery | Nigeria (Enugu) | 2 | 3 | 5 | 30 |
| mystery | Nigeria (Enugu) | 3 | 2 | 4 | 2 |
| mystery | Nigeria (Enugu) | 3 | 2 | 4 | 7 |
| mystery | Nigeria (Enugu) | 3 | 2 | 4 | 8 |
| mystery | Nigeria (Enugu) | 3 | 2 | 4 | 13 |
| mystery | Nigeria (Enugu) | 3 | 2 | 4 | 15 |
| mystery | Nigeria (Enugu) | 3 | 2 | 4 | 21 |
| mystery | Nigeria (Enugu) | 3 | 2 | 6 | 6 |
| mystery | Nigeria (Enugu) | 4 | 1 | 2 | 15 |
| mystery | Nigeria (Enugu) | 4 | 1 | 2 | 19 |
| mystery | Nigeria (Enugu) | 4 | 1 | 2 | 36 |
| mystery | Nigeria (Enugu) | 5 | 1 | 2 | 17 |
| mystery | Nigeria (Enugu) | 5 | 1 | 2 | 57 |
| mystery | Nigeria (Enugu) | 5 | 1 | 2 | 59 |
| mystery | Nigeria (Enugu) | 5 | 2 | 3 | 7 |
| mystery | Nigeria (Enugu) | 5 | 2 | 3 | 12 |
| mystery | Nigeria (Enugu) | 6 | 2 | 1 | 15 |
| mystery | Nigeria (Enugu) | 6 | 2 | 1 | 28 |
| mystery | Nigeria (Enugu) | 6 | 2 | 3 | 1 |
| mystery | Nigeria (Enugu) | 6 | 2 | 3 | 25 |
| mystery | Nigeria (Enugu) | 6 | 2 | 3 | 31 |

| | | | | | |
|---|---|---|---|---|---|
| mystery | Nigeria (Enugu) | 6 | 2 | 3 | 37 |
| mystery | Nigeria (Enugu) | 7 | 2 | 4 | 4 |
| mystery | Nigeria (Enugu) | 7 | 2 | 4 | 22 |
| mystery | Nigeria (Enugu) | 7 | 2 | 4 | 25 |
| mystery | Nigeria (Enugu) | 8 | 2 | 4 | 16 |
| mystery | Nigeria (Enugu) | 9 | 2 | 2 | 1 |
| mystery | Nigeria (Enugu) | 9 | 3 | 5 | 19 |
| mystery | Nigeria (Enugu) | 9 | 3 | 5 | 20 |
| mystery | Nigeria (Enugu) | 10 | 3 | 1 | 5 |
| mystery | Nigeria (Enugu) | 10 | 3 | 1 | 8 |
| mystery | Nigeria (Enugu) | 10 | 3 | 1 | 14 |
| mystery | Nigeria (Enugu) | 10 | 3 | 1 | 20 |
| mystery | Nigeria (Enugu) | 10 | 3 | 1 | 33 |
| mystery | Nigeria (Enugu) | 10 | 3 | 1 | 66 |
| mystery | Nigeria (Enugu) | 10 | 3 | 1 | 71 |
| mystery | Nigeria (Enugu) | 10 | 3 | 1 | 78 |
| mystery | Nigeria (Enugu) | 10 | 3 | 1 | 79 |
| mystery | Nigeria (Enugu) | 11 | 2 | 5 | 1 |
| mystery | Nigeria (Enugu) | 11 | 2 | 5 | 5 |
| mystery | Nigeria (Enugu) | 11 | 2 | 5 | 11 |
| mystery | Nigeria (Enugu) | 11 | 2 | 5 | 22 |
| mystery | Nigeria (Enugu) | 11 | 2 | 5 | 26 |
| mystery | Nigeria (Enugu) | 12 | 3 | 7 | 18 |
| mystery | Nigeria (Enugu) | 13 | 2 | 3 | 16 |
| mystery | Nigeria (Enugu) | 13 | 2 | 5 | 9 |
| mystery | Nigeria (Enugu) | 13 | 3 | 7 | 1 |
| mystery | Nigeria (Enugu) | 13 | 3 | 7 | 33 |
| mystery | Nigeria (Enugu) | 14 | 1 | 1 | 6 |
| mystery | Nigeria (Enugu) | 14 | 1 | 1 | 20 |
| mystery | Nigeria (Enugu) | 14 | 1 | 3 | 1 |
| mystery | Nigeria (Enugu) | 14 | 1 | 3 | 4 |
| mystery | Nigeria (Enugu) | 14 | 1 | 3 | 5 |
| mystery | Nigeria (Enugu) | 14 | 1 | 3 | 29 |
| mystery | Nigeria (Enugu) | 14 | 1 | 3 | 35 |
| mystery | Nigeria (Enugu) | 15 | 2 | 1 | 5 |
| mystery | Nigeria (Enugu) | 15 | 2 | 1 | 7 |
| mystery | Nigeria (Enugu) | 15 | 2 | 1 | 19 |
| mystery | Nigeria (Enugu) | 15 | 2 | 1 | 29 |
| mystery | Nigeria (Enugu) | 15 | 2 | 3 | 10 |
| mystery | Nigeria (Enugu) | 15 | 2 | 3 | 11 |
| mystery | Nigeria (Enugu) | 15 | 2 | 3 | 20 |
| mystery | Nigeria (Enugu) | 15 | 2 | 3 | 30 |
| mystery | Nigeria (Enugu) | 15 | 2 | 3 | 38 |
| mystery | Nigeria (Enugu) | 16 | 1 | 1 | 7 |
| mystery | Nigeria (Enugu) | 16 | 1 | 3 | 2 |
| mystery | Nigeria (Enugu) | 16 | 1 | 3 | 6 |
| mystery | Nigeria (Enugu) | 16 | 1 | 3 | 27 |
| mystery | Nigeria (Enugu) | 16 | 1 | 3 | 30 |
| mystery | Nigeria (Enugu) | 16 | 1 | 3 | 31 |
| mystery | Nigeria (Enugu) | 16 | 1 | 3 | 38 |

| | | | | | |
|---|---|---|---|---|---|
| mystery | Nigeria (Enugu) | 1 | 3 | 1 | 10 |
| mystery | Nigeria (Enugu) | 5 | 1 | 2 | 7 |
| mystery | Nigeria (Enugu) | 6 | 2 | 1 | 11 |
| mystery | Nigeria (Enugu) | 11 | 2 | 5 | 1 |
| mystery | Nigeria (Enugu) | 13 | 3 | 7 | 1 |
| mystery | Nigeria (Enugu) | 13 | 3 | 7 | 33 |
| mystery | Nigeria (Enugu) | 1 | 3 | 1 | 4 |
| mystery | Nigeria (Enugu) | 9 | 2 | 2 | 6 |
| mystery | Nigeria (Enugu) | 10 | 3 | 1 | 21 |
| mystery | Nigeria (Enugu) | 11 | 3 | 7 | 24 |
| mystery | Nigeria (Enugu) | 12 | 2 | 5 | 16 |
| mystery | Nigeria (Enugu) | 1 | 3 | 1 | 39 |
| mystery | Nigeria (Enugu) | 1 | 3 | 1 | 74 |
| mystery | Nigeria (Enugu) | 3 | 2 | 6 | 6 |
| mystery | Nigeria (Enugu) | 6 | 2 | 1 | 28 |
| mystery | Nigeria (Enugu) | 11 | 2 | 5 | 22 |
| mystery | Nigeria (Enugu) | 12 | 2 | 5 | 3 |
| mystery | Nigeria (Enugu) | 13 | 3 | 7 | 9 |
| mystery | Nigeria (Enugu) | 15 | 2 | 1 | 4 |
| mystery | Nigeria (Enugu) | 15 | 2 | 1 | 5 |
| mystery | Nigeria (Enugu) | 3 | 2 | 4 | 21 |
| mystery | Nigeria (Enugu) | 4 | 1 | 2 | 15 |
| mystery | Nigeria (Enugu) | 4 | 1 | 2 | 53 |
| mystery | Nigeria (Enugu) | 5 | 1 | 2 | 22 |
| mystery | Nigeria (Enugu) | 6 | 2 | 1 | 11 |
| mystery | Nigeria (Enugu) | 14 | 1 | 3 | 9 |
| mystery | Nigeria (Enugu) | 15 | 2 | 1 | 29 |
| mystery | Nigeria (Enugu) | 15 | 2 | 3 | 28 |
| mystery | Nigeria (Enugu) | 16 | 1 | 3 | 7 |
| mystery | Nigeria (Enugu) | 16 | 1 | 3 | 11 |
| mystery | Nigeria (Enugu) | 16 | 1 | 3 | 38 |
| mystery | Nigeria (Enugu) | 5 | 1 | 2 | 59 |
| mystery | Nigeria (Enugu) | 6 | 2 | 1 | 11 |
| mystery | Nigeria (Enugu) | 6 | 2 | 3 | 37 |
| mystery | Nigeria (Enugu) | 6 | 2 | 1 | 11 |
| mystery | Nigeria (Enugu) | 16 | 1 | 3 | 4 |
| mystery | Nigeria (Enugu) | 4 | 1 | 2 | 8 |
| mystery | Nigeria (Enugu) | 5 | 1 | 2 | 17 |
| mystery | Nigeria (Enugu) | 9 | 2 | 2 | 2 |
| mystery | Nigeria (Enugu) | 9 | 3 | 5 | 16 |
| mystery | Nigeria (Enugu) | 16 | 1 | 1 | 28 |
| mystery | Nigeria (Enugu) | 1 | 3 | 1 | 60 |
| mystery | Nigeria (Enugu) | 1 | 3 | 1 | 60 |
| mystery | Nigeria (Enugu) | 5 | 1 | 2 | 7 |
| mystery | Nigeria (Enugu) | 5 | 1 | 2 | 55 |
| mystery | Nigeria (Enugu) | 5 | 1 | 2 | 59 |
| mystery | Nigeria (Enugu) | 14 | 1 | 1 | 15 |
| mystery | Nigeria (Enugu) | 14 | 1 | 3 | 9 |
| mystery | Nigeria (Enugu) | 15 | 2 | 3 | 38 |
| mystery | Nigeria (Enugu) | 16 | 1 | 3 | 7 |

| | | | | | |
|---|---|---|---|---|---|
| mystery | Nigeria (Enugu) | 13 | 2 | 5 | 25 |
| mystery | Nigeria (Enugu) | 3 | 2 | 4 | 21 |
| mystery | Nigeria (Enugu) | 4 | 1 | 2 | 9 |
| mystery | Nigeria (Enugu) | 4 | 1 | 2 | 19 |
| mystery | Nigeria (Enugu) | 5 | 1 | 2 | 55 |
| mystery | Nigeria (Enugu) | 5 | 2 | 1 | 0 |
| mystery | Nigeria (Enugu) | 6 | 2 | 3 | 37 |
| mystery | Nigeria (Enugu) | 9 | 2 | 2 | 1 |
| mystery | Nigeria (Enugu) | 9 | 3 | 6 | 8 |
| mystery | Nigeria (Enugu) | 15 | 2 | 1 | 14 |
| mystery | Nigeria (Enugu) | 16 | 1 | 3 | 11 |
| mystery | Nigeria (Enugu) | 5 | 1 | 2 | 59 |
| mystery | Nigeria (Enugu) | 4 | 1 | 2 | 53 |
| mystery | Nigeria (Enugu) | 5 | 1 | 2 | 7 |
| mystery | Nigeria (Enugu) | 6 | 2 | 3 | 14 |
| mystery | Nigeria (Enugu) | 15 | 2 | 1 | 14 |
| mystery | Nigeria (Enugu) | 2 | 3 | 5 | 4 |
| mystery | Nigeria (Enugu) | 6 | 2 | 1 | 11 |
| mystery | Nigeria (Enugu) | 9 | 3 | 5 | 8 |
| mystery | Nigeria (Enugu) | 13 | 3 | 7 | 8 |
| mystery | Nigeria (Enugu) | 16 | 1 | 3 | 11 |
| mystery | Nigeria (Enugu) | 5 | 1 | 2 | 8 |
| mystery | Nigeria (Enugu) | 15 | 2 | 3 | 38 |
| mystery | Nigeria (Enugu) | 6 | 2 | 3 | 14 |
| mystery | Nigeria (Enugu) | 1 | 3 | 1 | 60 |
| mystery | Nigeria (Enugu) | 2 | 3 | 2 | 6 |
| mystery | Nigeria (Enugu) | 2 | 3 | 2 | 7 |
| mystery | Nigeria (Enugu) | 2 | 3 | 6 | 1 |
| mystery | Nigeria (Enugu) | 3 | 2 | 4 | 13 |
| mystery | Nigeria (Enugu) | 3 | 2 | 4 | 21 |
| mystery | Nigeria (Enugu) | 4 | 1 | 2 | 9 |
| mystery | Nigeria (Enugu) | 4 | 1 | 2 | 19 |
| mystery | Nigeria (Enugu) | 6 | 2 | 3 | 1 |
| mystery | Nigeria (Enugu) | 6 | 2 | 1 | 11 |
| mystery | Nigeria (Enugu) | 6 | 2 | 1 | 15 |
| mystery | Nigeria (Enugu) | 6 | 2 | 1 | 28 |
| mystery | Nigeria (Enugu) | 6 | 2 | 3 | 31 |
| mystery | Nigeria (Enugu) | 6 | 2 | 3 | 36 |
| mystery | Nigeria (Enugu) | 8 | 2 | 4 | 16 |
| mystery | Nigeria (Enugu) | 8 | 2 | 4 | 44 |
| mystery | Nigeria (Enugu) | 9 | 2 | 2 | 1 |
| mystery | Nigeria (Enugu) | 9 | 2 | 2 | 10 |
| mystery | Nigeria (Enugu) | 9 | 2 | 2 | 11 |
| mystery | Nigeria (Enugu) | 9 | 3 | 5 | 16 |
| mystery | Nigeria (Enugu) | 9 | 3 | 5 | 19 |
| mystery | Nigeria (Enugu) | 9 | 3 | 6 | 8 |
| mystery | Nigeria (Enugu) | 14 | 1 | 1 | 6 |
| mystery | Nigeria (Enugu) | 14 | 1 | 1 | 15 |
| mystery | Nigeria (Enugu) | 14 | 1 | 3 | 10 |
| mystery | Nigeria (Enugu) | 14 | 1 | 3 | 5 |

| | | | | | |
|---|---|---|---|---|---|
| mystery | Nigeria (Enugu) | 14 | 1 | 3 | 9 |
| mystery | Nigeria (Enugu) | 15 | 2 | 1 | 29 |
| mystery | Nigeria (Enugu) | 15 | 2 | 3 | 10 |
| mystery | Nigeria (Enugu) | 15 | 2 | 3 | 20 |
| mystery | Nigeria (Enugu) | 16 | 1 | 1 | 28 |
| mystery | Nigeria (Enugu) | 16 | 1 | 3 | 11 |
| mystery | Nigeria (Enugu) | 16 | 1 | 3 | 2 |
| mystery | Nigeria (Enugu) | 16 | 1 | 3 | 31 |
| mystery | Nigeria (Enugu) | 16 | 1 | 3 | 38 |
| mystery | Nigeria (Enugu) | 4 | 1 | 2 | 4 |
| mystery | Nigeria (Enugu) | 4 | 1 | 2 | 19 |
| mystery | Nigeria (Enugu) | 5 | 1 | 2 | 22 |
| mystery | Nigeria (Enugu) | 15 | 2 | 3 | 30 |
| mystery | Nigeria (Enugu) | 4 | 1 | 2 | 9 |
| mystery | Nigeria (Enugu) | 7 | 2 | 4 | 4 |
| mystery | Nigeria (Enugu) | 9 | 2 | 1 | 0 |
| mystery | Nigeria (Enugu) | 11 | 2 | 5 | 1 |
| mystery | Nigeria (Enugu) | 12 | 2 | 5 | 4 |
| mystery | Nigeria (Enugu) | 6 | 2 | 3 | 6 |
| mystery | Nigeria (Enugu) | 4 | 1 | 2 | 53 |
| mystery | Nigeria (Enugu) | 3 | 2 | 4 | 21 |
| mystery | Nigeria (Enugu) | 5 | 1 | 2 | 11 |
| mystery | Nigeria (Enugu) | 13 | 3 | 7 | 32 |
| mystery | Nigeria (Enugu) | 15 | 2 | 1 | 5 |
| mystery | Nigeria (Enugu) | 1 | 3 | 1 | 60 |
| mystery | Nigeria (Enugu) | 2 | 3 | 5 | 27 |
| mystery | Nigeria (Enugu) | 6 | 2 | 1 | 15 |
| mystery | Nigeria (Enugu) | 9 | 2 | 2 | 1 |
| mystery | Nigeria (Enugu) | 9 | 2 | 2 | 7 |
| mystery | Nigeria (Enugu) | 9 | 2 | 2 | 10 |
| mystery | Nigeria (Enugu) | 9 | 3 | 5 | 20 |
| mystery | Nigeria (Enugu) | 9 | 3 | 6 | 8 |
| mystery | Nigeria (Enugu) | 14 | 1 | 1 | 15 |
| mystery | Nigeria (Enugu) | 14 | 1 | 3 | 1 |
| mystery | Nigeria (Enugu) | 14 | 1 | 3 | 23 |
| mystery | Nigeria (Enugu) | 14 | 1 | 3 | 35 |
| mystery | Nigeria (Enugu) | 15 | 2 | 3 | 38 |
| mystery | Nigeria (Enugu) | 16 | 1 | 1 | 28 |
| mystery | Nigeria (Enugu) | 16 | 1 | 3 | 11 |
| mystery | Nigeria (Enugu) | 16 | 1 | 3 | 31 |
| mystery | Nigeria (Enugu) | 9 | 2 | 2 | 10 |
| mystery | Nigeria (Enugu) | 15 | 2 | 3 | 28 |
| mystery | Nigeria (Enugu) | 15 | 2 | 1 | 29 |
| mystery | Nigeria (Enugu) | 1 | 3 | 1 | 4 |
| mystery | Nigeria (Enugu) | 1 | 3 | 1 | 13 |
| mystery | Nigeria (Enugu) | 1 | 3 | 1 | 60 |
| mystery | Nigeria (Enugu) | 4 | 1 | 2 | 12 |
| mystery | Nigeria (Enugu) | 4 | 1 | 2 | 23 |
| mystery | Nigeria (Enugu) | 4 | 1 | 2 | 38 |
| mystery | Nigeria (Enugu) | 6 | 2 | 1 | 11 |

| | | | | | |
|---|---|---|---|---|---|
| mystery | Nigeria (Enugu) | 6 | 2 | 1 | 15 |
| mystery | Nigeria (Enugu) | 6 | 2 | 1 | 28 |
| mystery | Nigeria (Enugu) | 6 | 2 | 3 | 9 |
| mystery | Nigeria (Enugu) | 6 | 2 | 3 | 25 |
| mystery | Nigeria (Enugu) | 6 | 2 | 3 | 37 |
| mystery | Nigeria (Enugu) | 8 | 2 | 4 | 16 |
| mystery | Nigeria (Enugu) | 8 | 2 | 4 | 23 |
| mystery | Nigeria (Enugu) | 8 | 2 | 4 | 33 |
| mystery | Nigeria (Enugu) | 9 | 2 | 2 | 1 |
| mystery | Nigeria (Enugu) | 9 | 2 | 2 | 7 |
| mystery | Nigeria (Enugu) | 9 | 3 | 5 | 19 |
| mystery | Nigeria (Enugu) | 9 | 3 | 5 | 20 |
| mystery | Nigeria (Enugu) | 9 | 3 | 6 | 8 |
| mystery | Nigeria (Enugu) | 10 | 3 | 1 | 1 |
| mystery | Nigeria (Enugu) | 13 | 3 | 7 | 32 |
| mystery | Nigeria (Enugu) | 14 | 1 | 1 | 15 |
| mystery | Nigeria (Enugu) | 14 | 1 | 3 | 1 |
| mystery | Nigeria (Enugu) | 14 | 1 | 3 | 5 |
| mystery | Nigeria (Enugu) | 14 | 1 | 3 | 9 |
| mystery | Nigeria (Enugu) | 14 | 1 | 3 | 23 |
| mystery | Nigeria (Enugu) | 15 | 2 | 1 | 19 |
| mystery | Nigeria (Enugu) | 15 | 2 | 1 | 29 |
| mystery | Nigeria (Enugu) | 15 | 2 | 3 | 10 |
| mystery | Nigeria (Enugu) | 15 | 2 | 3 | 38 |
| mystery | Nigeria (Enugu) | 16 | 1 | 1 | 28 |
| mystery | Nigeria (Enugu) | 16 | 1 | 3 | 7 |
| mystery | Nigeria (Enugu) | 16 | 1 | 3 | 11 |
| mystery | Nigeria (Enugu) | 16 | 1 | 3 | 4 |
| mystery | Nigeria (Enugu) | 16 | 1 | 3 | 31 |
| mystery | Nigeria (Enugu) | 16 | 1 | 3 | 38 |
| mystery | Nigeria (Enugu) | 1 | 3 | 1 | 4 |
| mystery | Nigeria (Enugu) | 5 | 1 | 2 | 8 |
| mystery | Nigeria (Enugu) | 6 | 2 | 1 | 11 |
| mystery | Nigeria (Enugu) | 6 | 2 | 3 | 14 |
| mystery | Nigeria (Enugu) | 16 | 1 | 3 | 38 |
| mystery | Nigeria (Enugu) | 6 | 2 | 3 | 9 |
| overt | Nigeria (Enugu) | 4 | 2 | 3 | 34 |
| overt | Nigeria (Enugu) | 3 | 1 | 3 | 10 |
| overt | Nigeria (Enugu) | 3 | 1 | 3 | 21 |
| mystery | Nigeria (Enugu) | 4 | 1 | 2 | 43 |
| overt | Nigeria (Enugu) | 4 | 2 | 3 | 1 |
| overt | Nigeria (Enugu) | 11 | 3 | 7 | 4 |
| overt | Nigeria (Enugu) | 11 | 3 | 7 | 29 |
| overt | Nigeria (Enugu) | 12 | 2 | 1 | 2 |
| overt | Nigeria (Enugu) | 12 | 2 | 1 | 27 |
| overt | Nigeria (Enugu) | 12 | 2 | 1 | 28 |
| overt | Nigeria (Enugu) | 14 | 2 | 5 | 22 |
| overt | Nigeria (Enugu) | 14 | 3 | 1 | 53 |
| overt | Nigeria (Enugu) | 15 | 2 | 4 | 17 |
| overt | Nigeria (Enugu) | 15 | 2 | 4 | 23 |

| | | | | | |
|---|---|---|---|---|---|
| overt | Nigeria (Enugu) | 16 | 3 | 1 | 72 |
| overt | Nigeria (Enugu) | 16 | 3 | 1 | 83 |
| overt | Nigeria (Enugu) | 3 | 1 | 3 | 34 |
| overt | Nigeria (Enugu) | 11 | 3 | 7 | 9 |
| overt | Nigeria (Enugu) | 11 | 3 | 7 | 33 |
| overt | Nigeria (Enugu) | 15 | 2 | 4 | 17 |
| overt | Nigeria (Enugu) | 3 | 1 | 3 | 30 |
| overt | Nigeria (Enugu) | 4 | 2 | 3 | 27 |
| overt | Nigeria (Enugu) | 9 | 1 | 1 | 5 |
| overt | Nigeria (Enugu) | 11 | 3 | 5 | 20 |
| overt | Nigeria (Enugu) | 11 | 3 | 7 | 33 |
| overt | Nigeria (Enugu) | 12 | 2 | 6 | 6 |
| overt | Nigeria (Enugu) | 15 | 2 | 4 | 17 |
| overt | Nigeria (Enugu) | 15 | 2 | 4 | 42 |
| overt | Nigeria (Enugu) | 16 | 3 | 2 | 5 |
| overt | Nigeria (Enugu) | 17 | 1 | 2 | 51 |
| overt | Nigeria (Enugu) | 3 | 1 | 3 | 27 |
| overt | Nigeria (Enugu) | 4 | 2 | 3 | 20 |
| overt | Nigeria (Enugu) | 4 | 2 | 3 | 26 |
| overt | Nigeria (Enugu) | 4 | 2 | 3 | 37 |
| overt | Nigeria (Enugu) | 11 | 3 | 7 | 1 |
| overt | Nigeria (Enugu) | 11 | 3 | 7 | 28 |
| overt | Nigeria (Enugu) | 11 | 3 | 7 | 31 |
| overt | Nigeria (Enugu) | 12 | 2 | 1 | 28 |
| overt | Nigeria (Enugu) | 14 | 2 | 5 | 22 |
| overt | Nigeria (Enugu) | 14 | 3 | 1 | 14 |
| overt | Nigeria (Enugu) | 15 | 2 | 4 | 11 |
| overt | Nigeria (Enugu) | 15 | 2 | 4 | 14 |
| overt | Nigeria (Enugu) | 15 | 2 | 4 | 17 |
| overt | Nigeria (Enugu) | 15 | 2 | 4 | 34 |
| overt | Nigeria (Enugu) | 15 | 2 | 4 | 36 |
| overt | Nigeria (Enugu) | 15 | 2 | 4 | 39 |
| overt | Nigeria (Enugu) | 15 | 2 | 4 | 42 |
| overt | Nigeria (Enugu) | 16 | 3 | 1 | 69 |
| overt | Nigeria (Enugu) | 16 | 3 | 1 | 72 |
| overt | Nigeria (Enugu) | 16 | 3 | 2 | 5 |
| overt | Nigeria (Enugu) | 17 | 1 | 3 | 2 |
| overt | Nigeria (Enugu) | 3 | 1 | 3 | 10 |
| overt | Nigeria (Enugu) | 3 | 1 | 3 | 32 |
| overt | Nigeria (Enugu) | 4 | 2 | 1 | 10 |
| overt | Nigeria (Enugu) | 9 | 1 | 1 | 6 |
| overt | Nigeria (Enugu) | 9 | 1 | 2 | 29 |
| overt | Nigeria (Enugu) | 12 | 2 | 6 | 5 |
| overt | Nigeria (Enugu) | 14 | 2 | 5 | 2 |
| overt | Nigeria (Enugu) | 15 | 2 | 4 | 8 |
| overt | Nigeria (Enugu) | 16 | 3 | 1 | 74 |
| overt | Nigeria (Enugu) | 17 | 1 | 2 | 36 |
| overt | Nigeria (Enugu) | 3 | 1 | 3 | 34 |
| overt | Nigeria (Enugu) | 9 | 1 | 2 | 18 |
| overt | Nigeria (Enugu) | 15 | 2 | 4 | 11 |

| | | | | | |
|---|---|---|---|---|---|
| overt | Nigeria (Enugu) | 17 | 1 | 2 | 19 |
| overt | Nigeria (Enugu) | 11 | 3 | 7 | 1 |
| overt | Nigeria (Enugu) | 14 | 2 | 5 | 2 |
| overt | Nigeria (Enugu) | 3 | 1 | 3 | 21 |
| overt | Nigeria (Enugu) | 9 | 1 | 1 | 6 |
| overt | Nigeria (Enugu) | 12 | 2 | 1 | 10 |
| overt | Nigeria (Enugu) | 17 | 1 | 2 | 59 |
| overt | Nigeria (Enugu) | 16 | 3 | 1 | 10 |
| overt | Nigeria (Enugu) | 4 | 2 | 3 | 28 |
| overt | Nigeria (Enugu) | 4 | 2 | 3 | 38 |
| overt | Nigeria (Enugu) | 3 | 1 | 3 | 9 |
| overt | Nigeria (Enugu) | 3 | 1 | 3 | 10 |
| overt | Nigeria (Enugu) | 3 | 1 | 3 | 11 |
| overt | Nigeria (Enugu) | 3 | 1 | 3 | 21 |
| overt | Nigeria (Enugu) | 3 | 1 | 3 | 33 |
| overt | Nigeria (Enugu) | 3 | 1 | 3 | 34 |
| overt | Nigeria (Enugu) | 3 | 2 | 3 | 3 |
| overt | Nigeria (Enugu) | 4 | 2 | 1 | 10 |
| overt | Nigeria (Enugu) | 4 | 2 | 3 | 8 |
| overt | Nigeria (Enugu) | 4 | 2 | 3 | 16 |
| overt | Nigeria (Enugu) | 4 | 2 | 3 | 20 |
| overt | Nigeria (Enugu) | 4 | 2 | 3 | 31 |
| overt | Nigeria (Enugu) | 4 | 2 | 3 | 38 |
| overt | Nigeria (Enugu) | 9 | 1 | 1 | 16 |
| overt | Nigeria (Enugu) | 11 | 3 | 7 | 28 |
| overt | Nigeria (Enugu) | 12 | 2 | 1 | 1 |
| overt | Nigeria (Enugu) | 12 | 2 | 1 | 10 |
| overt | Nigeria (Enugu) | 12 | 2 | 1 | 14 |
| overt | Nigeria (Enugu) | 12 | 2 | 6 | 1 |
| overt | Nigeria (Enugu) | 12 | 2 | 6 | 5 |
| overt | Nigeria (Enugu) | 14 | 3 | 1 | 8 |
| overt | Nigeria (Enugu) | 14 | 3 | 1 | 31 |
| overt | Nigeria (Enugu) | 14 | 3 | 1 | 62 |
| overt | Nigeria (Enugu) | 14 | 3 | 2 | 1 |
| overt | Nigeria (Enugu) | 15 | 2 | 4 | 8 |
| overt | Nigeria (Enugu) | 15 | 2 | 4 | 14 |
| overt | Nigeria (Enugu) | 15 | 2 | 4 | 25 |
| overt | Nigeria (Enugu) | 16 | 3 | 1 | 74 |
| overt | Nigeria (Enugu) | 16 | 3 | 1 | 76 |
| overt | Nigeria (Enugu) | 17 | 1 | 2 | 36 |
| overt | Nigeria (Enugu) | 17 | 1 | 2 | 57 |
| overt | Nigeria (Enugu) | 17 | 1 | 3 | 2 |
| overt | Nigeria (Enugu) | 17 | 1 | 3 | 3 |
| overt | Nigeria (Enugu) | 3 | 1 | 3 | 14 |
| overt | Nigeria (Enugu) | 9 | 1 | 2 | 20 |
| overt | Nigeria (Enugu) | 11 | 3 | 7 | 4 |
| overt | Nigeria (Enugu) | 16 | 3 | 1 | 72 |
| overt | Nigeria (Enugu) | 16 | 3 | 3 | 5 |
| overt | Nigeria (Enugu) | 3 | 1 | 3 | 7 |
| overt | Nigeria (Enugu) | 3 | 1 | 3 | 10 |

| | | | | | |
|---|---|---|---|---|---|
| overt | Nigeria (Enugu) | 3 | 1 | 3 | 27 |
| overt | Nigeria (Enugu) | 3 | 1 | 3 | 31 |
| overt | Nigeria (Enugu) | 4 | 2 | 1 | 10 |
| overt | Nigeria (Enugu) | 4 | 2 | 3 | 1 |
| overt | Nigeria (Enugu) | 4 | 2 | 3 | 26 |
| overt | Nigeria (Enugu) | 4 | 2 | 3 | 27 |
| overt | Nigeria (Enugu) | 4 | 2 | 3 | 28 |
| overt | Nigeria (Enugu) | 4 | 2 | 3 | 37 |
| mystery | Nigeria (Enugu) | 5 | 1 | 2 | 44 |
| mystery | Nigeria (Enugu) | 5 | 1 | 2 | 51 |
| overt | Nigeria (Enugu) | 9 | 1 | 2 | 28 |
| overt | Nigeria (Enugu) | 11 | 3 | 5 | 20 |
| overt | Nigeria (Enugu) | 11 | 3 | 7 | 25 |
| overt | Nigeria (Enugu) | 12 | 2 | 1 | 10 |
| overt | Nigeria (Enugu) | 12 | 2 | 6 | 6 |
| overt | Nigeria (Enugu) | 14 | 2 | 5 | 2 |
| overt | Nigeria (Enugu) | 14 | 2 | 5 | 6 |
| overt | Nigeria (Enugu) | 14 | 2 | 5 | 25 |
| overt | Nigeria (Enugu) | 14 | 3 | 1 | 10 |
| overt | Nigeria (Enugu) | 14 | 3 | 1 | 21 |
| overt | Nigeria (Enugu) | 14 | 3 | 1 | 59 |
| overt | Nigeria (Enugu) | 15 | 2 | 4 | 34 |
| overt | Nigeria (Enugu) | 16 | 3 | 2 | 5 |
| overt | Nigeria (Enugu) | 17 | 1 | 2 | 33 |
| overt | Nigeria (Enugu) | 17 | 1 | 2 | 60 |
| overt | Nigeria (Enugu) | 3 | 1 | 3 | 10 |
| overt | Nigeria (Enugu) | 3 | 1 | 3 | 34 |
| overt | Nigeria (Enugu) | 4 | 2 | 1 | 10 |
| overt | Nigeria (Enugu) | 4 | 2 | 3 | 27 |
| overt | Nigeria (Enugu) | 4 | 2 | 3 | 28 |
| mystery | Nigeria (Enugu) | 5 | 1 | 2 | 42 |
| overt | Nigeria (Enugu) | 9 | 1 | 1 | 7 |
| overt | Nigeria (Enugu) | 11 | 3 | 5 | 20 |
| overt | Nigeria (Enugu) | 11 | 3 | 7 | 9 |
| overt | Nigeria (Enugu) | 12 | 2 | 1 | 17 |
| mystery | Nigeria (Enugu) | 4 | 1 | 2 | 43 |
| overt | Nigeria (Enugu) | 9 | 1 | 1 | 6 |
| overt | Nigeria (Enugu) | 11 | 3 | 7 | 25 |
| overt | Nigeria (Enugu) | 12 | 2 | 1 | 2 |
| overt | Nigeria (Enugu) | 14 | 3 | 1 | 14 |
| overt | Nigeria (Enugu) | 16 | 3 | 2 | 5 |
| overt | Nigeria (Enugu) | 4 | 2 | 1 | 10 |
| overt | Nigeria (Enugu) | 4 | 2 | 3 | 8 |
| overt | Nigeria (Enugu) | 12 | 2 | 1 | 1 |
| overt | Nigeria (Enugu) | 12 | 2 | 1 | 10 |
| overt | Nigeria (Enugu) | 14 | 2 | 5 | 6 |
| overt | Nigeria (Enugu) | 14 | 3 | 2 | 1 |
| overt | Nigeria (Enugu) | 15 | 2 | 4 | 7 |
| mystery | Nigeria (Enugu) | 5 | 1 | 2 | 42 |
| overt | Nigeria (Enugu) | 14 | 3 | 1 | 21 |

| | | | | | |
|---|---|---|---|---|---|
| overt | Nigeria (Enugu) | 9 | 1 | 1 | 6 |
| overt | Nigeria (Enugu) | 11 | 3 | 7 | 28 |
| overt | Nigeria (Enugu) | 12 | 2 | 1 | 2 |
| overt | Nigeria (Enugu) | 14 | 3 | 1 | 14 |
| overt | Nigeria (Enugu) | 15 | 2 | 4 | 36 |
| overt | Nigeria (Enugu) | 16 | 3 | 1 | 75 |
| overt | Nigeria (Enugu) | 17 | 1 | 2 | 19 |
| mystery | Nigeria (Enugu) | 4 | 1 | 2 | 45 |
| mystery | Nigeria (Enugu) | 5 | 1 | 2 | 42 |
| overt | Nigeria (Enugu) | 9 | 1 | 1 | 6 |
| overt | Nigeria (Enugu) | 14 | 3 | 1 | 14 |
| overt | Nigeria (Enugu) | 15 | 2 | 4 | 7 |
| overt | Nigeria (Enugu) | 15 | 2 | 4 | 36 |
| overt | Nigeria (Enugu) | 16 | 3 | 2 | 5 |
| overt | Nigeria (Enugu) | 3 | 1 | 3 | 10 |
| overt | Nigeria (Enugu) | 3 | 1 | 3 | 30 |
| overt | Nigeria (Enugu) | 4 | 2 | 3 | 27 |
| overt | Nigeria (Enugu) | 4 | 2 | 3 | 28 |
| overt | Nigeria (Enugu) | 4 | 2 | 3 | 34 |
| mystery | Nigeria (Enugu) | 5 | 1 | 2 | 44 |
| overt | Nigeria (Enugu) | 9 | 1 | 1 | 7 |
| overt | Nigeria (Enugu) | 9 | 1 | 2 | 29 |
| overt | Nigeria (Enugu) | 11 | 3 | 7 | 4 |
| overt | Nigeria (Enugu) | 12 | 2 | 1 | 27 |
| overt | Nigeria (Enugu) | 16 | 3 | 2 | 5 |
| overt | Nigeria (Enugu) | 16 | 3 | 1 | 83 |
| overt | Nigeria (Enugu) | 3 | 1 | 3 | 7 |
| overt | Nigeria (Enugu) | 3 | 1 | 3 | 21 |
| overt | Nigeria (Enugu) | 3 | 1 | 3 | 34 |
| mystery | Nigeria (Enugu) | 4 | 1 | 2 | 13 |
| overt | Nigeria (Enugu) | 4 | 2 | 3 | 26 |
| overt | Nigeria (Enugu) | 4 | 2 | 3 | 34 |
| overt | Nigeria (Enugu) | 9 | 1 | 1 | 16 |
| overt | Nigeria (Enugu) | 9 | 1 | 1 | 11 |
| overt | Nigeria (Enugu) | 11 | 3 | 7 | 29 |
| overt | Nigeria (Enugu) | 11 | 3 | 7 | 9 |
| overt | Nigeria (Enugu) | 11 | 3 | 7 | 12 |
| overt | Nigeria (Enugu) | 14 | 3 | 1 | 10 |
| overt | Nigeria (Enugu) | 14 | 3 | 1 | 21 |
| overt | Nigeria (Enugu) | 15 | 2 | 4 | 27 |
| overt | Nigeria (Enugu) | 15 | 2 | 4 | 44 |
| overt | Nigeria (Enugu) | 16 | 3 | 1 | 69 |
| overt | Nigeria (Enugu) | 16 | 3 | 2 | 5 |
| overt | Nigeria (Enugu) | 17 | 1 | 2 | 15 |
| overt | Nigeria (Enugu) | 3 | 1 | 3 | 10 |
| overt | Nigeria (Enugu) | 3 | 1 | 3 | 14 |
| overt | Nigeria (Enugu) | 3 | 1 | 3 | 27 |
| mystery | Nigeria (Enugu) | 4 | 1 | 2 | 39 |
| mystery | Nigeria (Enugu) | 4 | 1 | 2 | 45 |
| overt | Nigeria (Enugu) | 4 | 2 | 1 | 10 |

| | | | | | |
|---|---|---|---|---|---|
| overt | Nigeria (Enugu) | 4 | 2 | 3 | 1 |
| overt | Nigeria (Enugu) | 4 | 2 | 3 | 24 |
| overt | Nigeria (Enugu) | 4 | 2 | 3 | 27 |
| overt | Nigeria (Enugu) | 4 | 2 | 3 | 28 |
| overt | Nigeria (Enugu) | 4 | 2 | 3 | 31 |
| overt | Nigeria (Enugu) | 9 | 1 | 1 | 10 |
| overt | Nigeria (Enugu) | 9 | 1 | 2 | 19 |
| overt | Nigeria (Enugu) | 9 | 1 | 2 | 25 |
| overt | Nigeria (Enugu) | 9 | 1 | 2 | 28 |
| overt | Nigeria (Enugu) | 11 | 3 | 6 | 1 |
| overt | Nigeria (Enugu) | 11 | 3 | 7 | 4 |
| overt | Nigeria (Enugu) | 11 | 3 | 7 | 28 |
| overt | Nigeria (Enugu) | 11 | 3 | 7 | 31 |
| overt | Nigeria (Enugu) | 12 | 2 | 1 | 10 |
| overt | Nigeria (Enugu) | 12 | 2 | 6 | 6 |
| overt | Nigeria (Enugu) | 14 | 2 | 5 | 2 |
| overt | Nigeria (Enugu) | 14 | 2 | 5 | 25 |
| overt | Nigeria (Enugu) | 15 | 2 | 4 | 22 |
| overt | Nigeria (Enugu) | 15 | 2 | 4 | 34 |
| overt | Nigeria (Enugu) | 16 | 3 | 1 | 74 |
| overt | Nigeria (Enugu) | 16 | 3 | 3 | 5 |
| overt | Nigeria (Enugu) | 17 | 1 | 2 | 18 |
| overt | Nigeria (Enugu) | 17 | 1 | 2 | 39 |
| overt | Nigeria (Enugu) | 17 | 1 | 2 | 57 |
| overt | Nigeria (Enugu) | 14 | 2 | 5 | 2 |
| overt | Nigeria (Enugu) | 17 | 1 | 2 | 33 |
| overt | Nigeria (Enugu) | 3 | 1 | 3 | 33 |
| overt | Nigeria (Enugu) | 3 | 1 | 3 | 11 |
| mystery | Nigeria (Enugu) | 4 | 1 | 2 | 43 |
| overt | Nigeria (Enugu) | 9 | 1 | 1 | 16 |
| overt | Nigeria (Enugu) | 9 | 1 | 2 | 18 |
| overt | Nigeria (Enugu) | 9 | 1 | 2 | 20 |
| overt | Nigeria (Enugu) | 15 | 2 | 4 | 4 |
| overt | Nigeria (Enugu) | 14 | 2 | 5 | 6 |
| overt | Nigeria (Enugu) | 3 | 1 | 3 | 33 |
| overt | Nigeria (Enugu) | 14 | 2 | 5 | 2 |
| overt | Nigeria (Enugu) | 15 | 2 | 4 | 4 |
| overt | Nigeria (Enugu) | 3 | 1 | 3 | 10 |
| overt | Nigeria (Enugu) | 3 | 1 | 3 | 31 |
| overt | Nigeria (Enugu) | 3 | 1 | 3 | 32 |
| overt | Nigeria (Enugu) | 3 | 1 | 3 | 33 |
| overt | Nigeria (Enugu) | 3 | 2 | 3 | 3 |
| overt | Nigeria (Enugu) | 3 | 2 | 5 | 12 |
| overt | Nigeria (Enugu) | 11 | 3 | 7 | 9 |
| overt | Nigeria (Enugu) | 11 | 3 | 7 | 28 |
| overt | Nigeria (Enugu) | 12 | 2 | 1 | 14 |
| overt | Nigeria (Enugu) | 14 | 3 | 1 | 42 |
| overt | Nigeria (Enugu) | 15 | 2 | 4 | 7 |
| overt | Nigeria (Enugu) | 16 | 3 | 1 | 77 |
| overt | Nigeria (Enugu) | 16 | 3 | 1 | 83 |

| | | | | | |
|---|---|---|---|---|---|
| overt | Nigeria (Enugu) | 17 | 1 | 2 | 36 |
| overt | Nigeria (Enugu) | 17 | 1 | 2 | 59 |
| overt | Nigeria (Enugu) | 17 | 1 | 3 | 2 |
| overt | Nigeria (Enugu) | 17 | 1 | 2 | 57 |
| overt | Nigeria (Enugu) | 15 | 2 | 4 | 4 |
| overt | Nigeria (Enugu) | 3 | 2 | 3 | 3 |
| overt | Nigeria (Enugu) | 14 | 3 | 1 | 42 |
| overt | Nigeria (Enugu) | 9 | 1 | 2 | 18 |
| overt | Nigeria (Enugu) | 9 | 1 | 2 | 18 |
| overt | Nigeria (Enugu) | 9 | 1 | 2 | 20 |
| overt | Nigeria (Enugu) | 3 | 1 | 3 | 14 |
| overt | Nigeria (Enugu) | 3 | 1 | 3 | 31 |
| overt | Nigeria (Enugu) | 3 | 1 | 3 | 33 |
| overt | Nigeria (Enugu) | 3 | 2 | 3 | 3 |
| overt | Nigeria (Enugu) | 3 | 2 | 5 | 12 |
| overt | Nigeria (Enugu) | 4 | 2 | 1 | 10 |
| overt | Nigeria (Enugu) | 4 | 2 | 3 | 37 |
| overt | Nigeria (Enugu) | 9 | 1 | 2 | 19 |
| overt | Nigeria (Enugu) | 11 | 3 | 7 | 28 |
| overt | Nigeria (Enugu) | 12 | 2 | 1 | 10 |
| overt | Nigeria (Enugu) | 12 | 2 | 1 | 14 |
| overt | Nigeria (Enugu) | 14 | 3 | 1 | 8 |
| overt | Nigeria (Enugu) | 14 | 3 | 1 | 62 |
| overt | Nigeria (Enugu) | 15 | 2 | 4 | 25 |
| overt | Nigeria (Enugu) | 15 | 2 | 4 | 34 |
| overt | Nigeria (Enugu) | 15 | 2 | 4 | 36 |
| overt | Nigeria (Enugu) | 16 | 3 | 1 | 76 |
| overt | Nigeria (Enugu) | 16 | 3 | 1 | 83 |
| overt | Nigeria (Enugu) | 17 | 1 | 3 | 2 |
| overt | Nigeria (Enugu) | 3 | 1 | 3 | 30 |
| overt | Nigeria (Enugu) | 3 | 1 | 3 | 34 |
| overt | Nigeria (Enugu) | 3 | 2 | 5 | 12 |
| mystery | Nigeria (Enugu) | 4 | 1 | 2 | 43 |
| overt | Nigeria (Enugu) | 9 | 1 | 1 | 16 |
| overt | Nigeria (Enugu) | 9 | 1 | 2 | 28 |
| overt | Nigeria (Enugu) | 14 | 3 | 1 | 31 |
| overt | Nigeria (Enugu) | 14 | 3 | 1 | 37 |
| overt | Nigeria (Enugu) | 3 | 1 | 3 | 11 |
| overt | Nigeria (Enugu) | 3 | 1 | 3 | 21 |
| overt | Nigeria (Enugu) | 3 | 1 | 3 | 32 |
| overt | Nigeria (Enugu) | 14 | 3 | 1 | 62 |
| overt | Nigeria (Enugu) | 14 | 3 | 2 | 1 |
| overt | Nigeria (Enugu) | 17 | 1 | 3 | 2 |
| overt | Nigeria (Enugu) | 3 | 2 | 3 | 3 |
| overt | Nigeria (Enugu) | 3 | 2 | 3 | 6 |
| overt | Nigeria (Enugu) | 4 | 2 | 3 | 38 |
| overt | Nigeria (Enugu) | 15 | 2 | 4 | 4 |
| mystery | Nigeria (Enugu) | 4 | 1 | 2 | 13 |
| overt | Nigeria (Enugu) | 16 | 3 | 5 | 11 |
| overt | Nigeria (Enugu) | 9 | 1 | 1 | 7 |

| | | | | | |
|---|---|---|---|---|---|
| overt | Nigeria (Enugu) | 9 | 1 | 2 | 18 |
| overt | Nigeria (Enugu) | 17 | 1 | 2 | 36 |
| overt | Nigeria (Enugu) | 14 | 3 | 1 | 42 |
| overt | Nigeria (Enugu) | 16 | 3 | 1 | 77 |
| overt | Nigeria (Enugu) | 17 | 1 | 2 | 57 |
| overt | Nigeria (Enugu) | 4 | 2 | 3 | 28 |
| overt | Nigeria (Enugu) | 11 | 3 | 7 | 9 |
| overt | Nigeria (Enugu) | 12 | 2 | 1 | 27 |
| overt | Nigeria (Enugu) | 14 | 2 | 5 | 2 |
| overt | Nigeria (Enugu) | 15 | 2 | 4 | 4 |
| overt | Nigeria (Enugu) | 15 | 2 | 4 | 11 |
| overt | Nigeria (Enugu) | 15 | 2 | 4 | 42 |
| overt | Nigeria (Enugu) | 3 | 1 | 3 | 11 |
| overt | Nigeria (Enugu) | 3 | 2 | 5 | 12 |
| overt | Nigeria (Enugu) | 4 | 2 | 3 | 16 |
| overt | Nigeria (Enugu) | 4 | 2 | 3 | 21 |
| overt | Nigeria (Enugu) | 4 | 2 | 3 | 31 |
| overt | Nigeria (Enugu) | 9 | 1 | 1 | 7 |
| overt | Nigeria (Enugu) | 11 | 3 | 7 | 28 |
| overt | Nigeria (Enugu) | 11 | 3 | 6 | 1 |
| overt | Nigeria (Enugu) | 11 | 3 | 7 | 28 |
| overt | Nigeria (Enugu) | 12 | 2 | 6 | 5 |
| overt | Nigeria (Enugu) | 17 | 1 | 2 | 19 |
| overt | Nigeria (Enugu) | 3 | 1 | 3 | 31 |
| overt | Nigeria (Enugu) | 4 | 2 | 3 | 38 |
| overt | Nigeria (Enugu) | 9 | 1 | 1 | 16 |
| overt | Nigeria (Enugu) | 11 | 3 | 7 | 28 |
| overt | Nigeria (Enugu) | 12 | 2 | 1 | 27 |
| overt | Nigeria (Enugu) | 3 | 2 | 5 | 12 |
| overt | Nigeria (Enugu) | 4 | 2 | 3 | 1 |
| overt | Nigeria (Enugu) | 4 | 2 | 3 | 20 |
| overt | Nigeria (Enugu) | 4 | 2 | 3 | 21 |
| overt | Nigeria (Enugu) | 9 | 1 | 2 | 25 |
| overt | Nigeria (Enugu) | 12 | 2 | 1 | 28 |
| overt | Nigeria (Enugu) | 14 | 3 | 2 | 1 |
| overt | Nigeria (Enugu) | 15 | 2 | 4 | 4 |
| overt | Nigeria (Enugu) | 15 | 2 | 4 | 7 |
| overt | Nigeria (Enugu) | 15 | 2 | 4 | 25 |
| overt | Nigeria (Enugu) | 15 | 2 | 4 | 42 |
| overt | Nigeria (Enugu) | 15 | 2 | 4 | 11 |
| overt | Nigeria (Enugu) | 9 | 1 | 1 | 6 |
| overt | Nigeria (Enugu) | 15 | 2 | 4 | 4 |
| overt | Nigeria (Enugu) | 4 | 2 | 3 | 21 |
| overt | Nigeria (Enugu) | 4 | 2 | 3 | 28 |
| overt | Nigeria (Enugu) | 4 | 2 | 3 | 34 |
| overt | Nigeria (Enugu) | 4 | 2 | 3 | 37 |
| overt | Nigeria (Enugu) | 12 | 2 | 1 | 28 |
| overt | Nigeria (Enugu) | 12 | 2 | 6 | 5 |
| overt | Nigeria (Enugu) | 15 | 2 | 4 | 7 |
| overt | Nigeria (Enugu) | 15 | 2 | 4 | 11 |

| | | | | | |
|---|---|---|---|---|---|
| overt | Nigeria (Enugu) | 16 | 3 | 2 | 5 |
| overt | Nigeria (Enugu) | 3 | 1 | 3 | 7 |
| overt | Nigeria (Enugu) | 3 | 1 | 3 | 9 |
| overt | Nigeria (Enugu) | 3 | 1 | 3 | 11 |
| overt | Nigeria (Enugu) | 3 | 2 | 5 | 12 |
| overt | Nigeria (Enugu) | 4 | 2 | 3 | 30 |
| overt | Nigeria (Enugu) | 4 | 2 | 3 | 37 |
| mystery | Nigeria (Enugu) | 5 | 1 | 2 | 44 |
| overt | Nigeria (Enugu) | 9 | 1 | 2 | 29 |
| overt | Nigeria (Enugu) | 11 | 3 | 7 | 28 |
| overt | Nigeria (Enugu) | 12 | 2 | 1 | 27 |
| overt | Nigeria (Enugu) | 12 | 2 | 6 | 5 |
| overt | Nigeria (Enugu) | 14 | 3 | 2 | 1 |
| overt | Nigeria (Enugu) | 16 | 3 | 2 | 5 |
| overt | Nigeria (Enugu) | 17 | 1 | 2 | 36 |
| overt | Nigeria (Enugu) | 17 | 1 | 2 | 59 |
| overt | Nigeria (Enugu) | 3 | 1 | 3 | 7 |
| overt | Nigeria (Enugu) | 3 | 1 | 3 | 10 |
| overt | Nigeria (Enugu) | 3 | 1 | 3 | 14 |
| overt | Nigeria (Enugu) | 3 | 1 | 3 | 27 |
| overt | Nigeria (Enugu) | 3 | 1 | 3 | 30 |
| overt | Nigeria (Enugu) | 3 | 1 | 3 | 31 |
| overt | Nigeria (Enugu) | 3 | 1 | 3 | 32 |
| overt | Nigeria (Enugu) | 3 | 1 | 3 | 33 |
| overt | Nigeria (Enugu) | 3 | 1 | 3 | 34 |
| overt | Nigeria (Enugu) | 3 | 2 | 3 | 3 |
| overt | Nigeria (Enugu) | 3 | 2 | 3 | 6 |
| overt | Nigeria (Enugu) | 3 | 2 | 5 | 12 |
| mystery | Nigeria (Enugu) | 4 | 1 | 2 | 13 |
| mystery | Nigeria (Enugu) | 4 | 1 | 2 | 39 |
| overt | Nigeria (Enugu) | 4 | 2 | 1 | 10 |
| overt | Nigeria (Enugu) | 4 | 2 | 3 | 8 |
| overt | Nigeria (Enugu) | 4 | 2 | 3 | 16 |
| overt | Nigeria (Enugu) | 4 | 2 | 3 | 24 |
| overt | Nigeria (Enugu) | 4 | 2 | 3 | 30 |
| overt | Nigeria (Enugu) | 4 | 2 | 3 | 31 |
| overt | Nigeria (Enugu) | 4 | 2 | 3 | 37 |
| overt | Nigeria (Enugu) | 4 | 2 | 3 | 38 |
| mystery | Nigeria (Enugu) | 5 | 1 | 2 | 42 |
| overt | Nigeria (Enugu) | 9 | 1 | 1 | 10 |
| overt | Nigeria (Enugu) | 9 | 1 | 1 | 16 |
| overt | Nigeria (Enugu) | 9 | 1 | 2 | 19 |
| overt | Nigeria (Enugu) | 9 | 1 | 2 | 20 |
| overt | Nigeria (Enugu) | 9 | 1 | 2 | 25 |
| overt | Nigeria (Enugu) | 9 | 1 | 2 | 28 |
| overt | Nigeria (Enugu) | 9 | 1 | 2 | 29 |
| overt | Nigeria (Enugu) | 11 | 3 | 5 | 20 |
| overt | Nigeria (Enugu) | 11 | 3 | 6 | 1 |
| overt | Nigeria (Enugu) | 11 | 3 | 7 | 4 |
| overt | Nigeria (Enugu) | 11 | 3 | 7 | 28 |

| | | | | | |
|---|---|---|---|---|---|
| overt | Nigeria (Enugu) | 11 | 3 | 7 | 33 |
| overt | Nigeria (Enugu) | 12 | 2 | 1 | 2 |
| overt | Nigeria (Enugu) | 12 | 2 | 1 | 10 |
| overt | Nigeria (Enugu) | 12 | 2 | 1 | 14 |
| overt | Nigeria (Enugu) | 12 | 2 | 1 | 15 |
| overt | Nigeria (Enugu) | 12 | 2 | 6 | 5 |
| overt | Nigeria (Enugu) | 14 | 2 | 5 | 6 |
| overt | Nigeria (Enugu) | 14 | 2 | 5 | 22 |
| overt | Nigeria (Enugu) | 14 | 2 | 5 | 25 |
| overt | Nigeria (Enugu) | 14 | 3 | 1 | 8 |
| overt | Nigeria (Enugu) | 14 | 3 | 1 | 10 |
| overt | Nigeria (Enugu) | 14 | 3 | 1 | 14 |
| overt | Nigeria (Enugu) | 14 | 3 | 1 | 21 |
| overt | Nigeria (Enugu) | 14 | 3 | 1 | 31 |
| overt | Nigeria (Enugu) | 14 | 3 | 1 | 37 |
| overt | Nigeria (Enugu) | 14 | 3 | 1 | 53 |
| overt | Nigeria (Enugu) | 14 | 3 | 1 | 59 |
| overt | Nigeria (Enugu) | 14 | 3 | 1 | 62 |
| overt | Nigeria (Enugu) | 14 | 3 | 2 | 1 |
| overt | Nigeria (Enugu) | 15 | 2 | 4 | 8 |
| overt | Nigeria (Enugu) | 15 | 2 | 4 | 11 |
| overt | Nigeria (Enugu) | 15 | 2 | 4 | 25 |
| overt | Nigeria (Enugu) | 15 | 2 | 4 | 34 |
| overt | Nigeria (Enugu) | 15 | 2 | 4 | 36 |
| overt | Nigeria (Enugu) | 15 | 2 | 4 | 42 |
| overt | Nigeria (Enugu) | 15 | 2 | 4 | 44 |
| overt | Nigeria (Enugu) | 16 | 3 | 1 | 10 |
| overt | Nigeria (Enugu) | 16 | 3 | 1 | 74 |
| overt | Nigeria (Enugu) | 16 | 3 | 1 | 75 |
| overt | Nigeria (Enugu) | 16 | 3 | 1 | 76 |
| overt | Nigeria (Enugu) | 16 | 3 | 2 | 5 |
| overt | Nigeria (Enugu) | 16 | 3 | 3 | 5 |
| overt | Nigeria (Enugu) | 17 | 1 | 2 | 15 |
| overt | Nigeria (Enugu) | 17 | 1 | 2 | 19 |
| overt | Nigeria (Enugu) | 17 | 1 | 2 | 36 |
| overt | Nigeria (Enugu) | 17 | 1 | 2 | 39 |
| overt | Nigeria (Enugu) | 17 | 1 | 2 | 46 |
| overt | Nigeria (Enugu) | 17 | 1 | 2 | 51 |
| overt | Nigeria (Enugu) | 17 | 1 | 2 | 57 |
| overt | Nigeria (Enugu) | 17 | 1 | 3 | 2 |
| overt | Nigeria (Enugu) | 17 | 1 | 3 | 3 |
| overt | Nigeria (Enugu) | 3 | 1 | 3 | 11 |
| overt | Nigeria (Enugu) | 3 | 1 | 3 | 30 |
| overt | Nigeria (Enugu) | 3 | 2 | 5 | 12 |
| overt | Nigeria (Enugu) | 14 | 3 | 1 | 42 |
| overt | Nigeria (Enugu) | 3 | 1 | 3 | 9 |
| overt | Nigeria (Enugu) | 3 | 1 | 3 | 14 |
| overt | Nigeria (Enugu) | 3 | 1 | 3 | 31 |
| overt | Nigeria (Enugu) | 3 | 1 | 3 | 34 |
| overt | Nigeria (Enugu) | 3 | 2 | 3 | 3 |

| | | | | | |
|---|---|---|---|---|---|
| mystery | Nigeria (Enugu) | 4 | 1 | 2 | 39 |
| overt | Nigeria (Enugu) | 4 | 2 | 1 | 10 |
| overt | Nigeria (Enugu) | 9 | 1 | 1 | 16 |
| overt | Nigeria (Enugu) | 9 | 1 | 2 | 20 |
| overt | Nigeria (Enugu) | 12 | 2 | 1 | 10 |
| overt | Nigeria (Enugu) | 14 | 3 | 1 | 42 |
| overt | Nigeria (Enugu) | 16 | 3 | 1 | 83 |
| overt | Nigeria (Enugu) | 17 | 1 | 2 | 36 |
| overt | Nigeria (Enugu) | 17 | 1 | 2 | 39 |
| overt | Nigeria (Enugu) | 17 | 1 | 2 | 57 |
| overt | Nigeria (Enugu) | 3 | 1 | 3 | 31 |
| overt | Nigeria (Enugu) | 9 | 1 | 2 | 25 |
| overt | Nigeria (Enugu) | 14 | 2 | 5 | 6 |
| overt | Nigeria (Enugu) | 15 | 2 | 4 | 4 |
| overt | Nigeria (Enugu) | 17 | 1 | 2 | 15 |
| overt | Nigeria (Enugu) | 17 | 1 | 3 | 2 |
| overt | Nigeria (Enugu) | 3 | 1 | 3 | 11 |
| overt | Nigeria (Enugu) | 4 | 2 | 3 | 30 |
| overt | Nigeria (Enugu) | 11 | 3 | 7 | 31 |
| overt | Nigeria (Enugu) | 14 | 2 | 5 | 6 |
| overt | Nigeria (Enugu) | 14 | 3 | 1 | 8 |
| overt | Nigeria (Enugu) | 15 | 2 | 4 | 4 |
| overt | Nigeria (Enugu) | 15 | 2 | 4 | 34 |
| overt | Nigeria (Enugu) | 15 | 2 | 4 | 42 |
| overt | Nigeria (Enugu) | 16 | 3 | 1 | 76 |
| overt | Nigeria (Enugu) | 17 | 1 | 3 | 2 |
| overt | Nigeria (Enugu) | 3 | 1 | 3 | 21 |
| overt | Nigeria (Enugu) | 4 | 2 | 3 | 31 |
| overt | Nigeria (Enugu) | 4 | 2 | 3 | 38 |
| mystery | Nigeria (Enugu) | 5 | 1 | 2 | 44 |
| overt | Nigeria (Enugu) | 11 | 3 | 7 | 9 |
| overt | Nigeria (Enugu) | 14 | 3 | 1 | 42 |
| overt | Nigeria (Enugu) | 16 | 3 | 1 | 10 |
| overt | Nigeria (Enugu) | 3 | 1 | 3 | 32 |
| overt | Nigeria (Enugu) | 4 | 2 | 3 | 16 |
| overt | Nigeria (Enugu) | 11 | 3 | 7 | 9 |
| overt | Nigeria (Enugu) | 11 | 3 | 7 | 12 |
| overt | Nigeria (Enugu) | 12 | 2 | 1 | 15 |
| overt | Nigeria (Enugu) | 12 | 2 | 1 | 17 |
| overt | Nigeria (Enugu) | 12 | 2 | 1 | 28 |
| overt | Nigeria (Enugu) | 15 | 2 | 4 | 14 |
| overt | Nigeria (Enugu) | 14 | 2 | 5 | 6 |
| overt | Nigeria (Enugu) | 3 | 1 | 3 | 33 |
| overt | Nigeria (Enugu) | 4 | 2 | 1 | 10 |
| overt | Nigeria (Enugu) | 9 | 1 | 2 | 18 |
| overt | Nigeria (Enugu) | 12 | 2 | 1 | 10 |
| overt | Nigeria (Enugu) | 12 | 2 | 6 | 6 |
| overt | Nigeria (Enugu) | 14 | 3 | 2 | 1 |
| overt | Nigeria (Enugu) | 15 | 2 | 4 | 39 |
| overt | Nigeria (Enugu) | 17 | 1 | 2 | 22 |

| | | | | | |
|---|---|---|---|---|---|
| overt | Nigeria (Enugu) | 3 | 1 | 3 | 10 |
| overt | Nigeria (Enugu) | 3 | 1 | 3 | 26 |
| overt | Nigeria (Enugu) | 3 | 1 | 3 | 27 |
| overt | Nigeria (Enugu) | 3 | 1 | 3 | 33 |
| overt | Nigeria (Enugu) | 3 | 1 | 3 | 34 |
| overt | Nigeria (Enugu) | 4 | 2 | 3 | 16 |
| overt | Nigeria (Enugu) | 4 | 2 | 3 | 20 |
| overt | Nigeria (Enugu) | 9 | 1 | 1 | 5 |
| overt | Nigeria (Enugu) | 9 | 1 | 2 | 19 |
| overt | Nigeria (Enugu) | 9 | 1 | 2 | 22 |
| overt | Nigeria (Enugu) | 9 | 1 | 2 | 28 |
| overt | Nigeria (Enugu) | 12 | 2 | 1 | 2 |
| overt | Nigeria (Enugu) | 12 | 2 | 1 | 14 |
| overt | Nigeria (Enugu) | 12 | 2 | 6 | 1 |
| overt | Nigeria (Enugu) | 12 | 2 | 6 | 5 |
| overt | Nigeria (Enugu) | 14 | 2 | 5 | 2 |
| overt | Nigeria (Enugu) | 14 | 3 | 1 | 10 |
| overt | Nigeria (Enugu) | 14 | 3 | 1 | 31 |
| overt | Nigeria (Enugu) | 14 | 3 | 1 | 37 |
| overt | Nigeria (Enugu) | 14 | 3 | 2 | 1 |
| overt | Nigeria (Enugu) | 15 | 2 | 4 | 7 |
| overt | Nigeria (Enugu) | 15 | 2 | 4 | 17 |
| overt | Nigeria (Enugu) | 15 | 2 | 4 | 22 |
| overt | Nigeria (Enugu) | 15 | 2 | 4 | 39 |
| overt | Nigeria (Enugu) | 16 | 3 | 1 | 10 |
| overt | Nigeria (Enugu) | 16 | 3 | 1 | 69 |
| overt | Nigeria (Enugu) | 16 | 3 | 1 | 72 |
| overt | Nigeria (Enugu) | 17 | 1 | 2 | 18 |
| overt | Nigeria (Enugu) | 17 | 1 | 2 | 19 |
| overt | Nigeria (Enugu) | 17 | 1 | 2 | 36 |
| overt | Nigeria (Enugu) | 17 | 1 | 2 | 39 |
| overt | Nigeria (Enugu) | 17 | 1 | 2 | 46 |
| overt | Nigeria (Enugu) | 17 | 1 | 2 | 51 |
| overt | Nigeria (Enugu) | 17 | 1 | 2 | 57 |
| overt | Nigeria (Enugu) | 17 | 1 | 2 | 59 |
| overt | Nigeria (Enugu) | 3 | 1 | 3 | 30 |
| overt | Nigeria (Enugu) | 17 | 1 | 2 | 33 |
| overt | Nigeria (Enugu) | 9 | 1 | 1 | 16 |
| overt | Nigeria (Enugu) | 17 | 1 | 2 | 59 |
| overt | Nigeria (Enugu) | 3 | 1 | 3 | 10 |
| overt | Nigeria (Enugu) | 9 | 1 | 2 | 19 |
| overt | Nigeria (Enugu) | 12 | 2 | 1 | 1 |
| overt | Nigeria (Enugu) | 9 | 1 | 2 | 18 |
| overt | Nigeria (Enugu) | 3 | 1 | 3 | 9 |
| overt | Nigeria (Enugu) | 3 | 1 | 3 | 10 |
| mystery | Nigeria (Enugu) | 4 | 1 | 2 | 45 |
| overt | Nigeria (Enugu) | 9 | 1 | 1 | 10 |
| overt | Nigeria (Enugu) | 15 | 2 | 4 | 7 |
| overt | Nigeria (Enugu) | 3 | 1 | 3 | 30 |
| overt | Nigeria (Enugu) | 17 | 1 | 2 | 33 |

| | | | | | |
|---|---|---|---|---|---|
| mystery | Nigeria (Enugu) | 5 | 1 | 2 | 42 |
| mystery | Nigeria (Enugu) | 4 | 1 | 2 | 45 |
| overt | Nigeria (Enugu) | 15 | 2 | 4 | 4 |
| overt | Nigeria (Enugu) | 3 | 1 | 3 | 7 |
| overt | Nigeria (Enugu) | 3 | 1 | 3 | 26 |
| overt | Nigeria (Enugu) | 3 | 1 | 3 | 31 |
| overt | Nigeria (Enugu) | 3 | 2 | 3 | 6 |
| overt | Nigeria (Enugu) | 4 | 2 | 3 | 21 |
| overt | Nigeria (Enugu) | 4 | 2 | 3 | 26 |
| overt | Nigeria (Enugu) | 4 | 2 | 3 | 30 |
| overt | Nigeria (Enugu) | 11 | 3 | 5 | 20 |
| overt | Nigeria (Enugu) | 11 | 3 | 7 | 1 |
| overt | Nigeria (Enugu) | 12 | 2 | 1 | 15 |
| overt | Nigeria (Enugu) | 12 | 2 | 1 | 28 |
| overt | Nigeria (Enugu) | 14 | 3 | 1 | 14 |
| overt | Nigeria (Enugu) | 14 | 3 | 1 | 53 |
| overt | Nigeria (Enugu) | 15 | 2 | 4 | 4 |
| overt | Nigeria (Enugu) | 15 | 2 | 4 | 7 |
| overt | Nigeria (Enugu) | 15 | 2 | 4 | 17 |
| overt | Nigeria (Enugu) | 15 | 2 | 4 | 22 |
| overt | Nigeria (Enugu) | 15 | 2 | 4 | 27 |
| overt | Nigeria (Enugu) | 15 | 2 | 4 | 39 |
| overt | Nigeria (Enugu) | 15 | 2 | 4 | 42 |
| overt | Nigeria (Enugu) | 16 | 3 | 1 | 10 |
| overt | Nigeria (Enugu) | 16 | 3 | 2 | 5 |
| overt | Nigeria (Enugu) | 16 | 3 | 3 | 63 |
| overt | Nigeria (Enugu) | 17 | 1 | 2 | 18 |
| overt | Nigeria (Enugu) | 17 | 1 | 3 | 2 |
| overt | Nigeria (Enugu) | 3 | 1 | 3 | 26 |
| mystery | Nigeria (Enugu) | 4 | 1 | 2 | 45 |
| overt | Nigeria (Enugu) | 12 | 2 | 1 | 1 |
| overt | Nigeria (Enugu) | 17 | 1 | 2 | 51 |
| overt | Nigeria (Enugu) | 11 | 3 | 7 | 28 |
| overt | Nigeria (Enugu) | 17 | 1 | 2 | 19 |
| overt | Nigeria (Enugu) | 12 | 2 | 6 | 6 |
| overt | Nigeria (Enugu) | 3 | 1 | 3 | 7 |
| overt | Nigeria (Enugu) | 3 | 1 | 3 | 10 |
| overt | Nigeria (Enugu) | 3 | 1 | 3 | 14 |
| overt | Nigeria (Enugu) | 3 | 1 | 3 | 30 |
| overt | Nigeria (Enugu) | 3 | 1 | 3 | 31 |
| overt | Nigeria (Enugu) | 4 | 2 | 3 | 1 |
| overt | Nigeria (Enugu) | 4 | 2 | 3 | 37 |
| overt | Nigeria (Enugu) | 11 | 3 | 7 | 28 |
| overt | Nigeria (Enugu) | 12 | 2 | 6 | 1 |
| overt | Nigeria (Enugu) | 12 | 2 | 6 | 6 |
| overt | Nigeria (Enugu) | 14 | 2 | 5 | 2 |
| overt | Nigeria (Enugu) | 14 | 2 | 5 | 6 |
| overt | Nigeria (Enugu) | 14 | 3 | 2 | 1 |
| overt | Nigeria (Enugu) | 15 | 2 | 4 | 39 |
| overt | Nigeria (Enugu) | 17 | 1 | 2 | 59 |

| | | | | | |
|---|---|---|---|---|---|
| overt | Nigeria (Enugu) | 17 | 1 | 3 | 3 |
| overt | Nigeria (Enugu) | 3 | 1 | 3 | 7 |
| overt | Nigeria (Enugu) | 3 | 1 | 3 | 10 |
| overt | Nigeria (Enugu) | 3 | 1 | 3 | 14 |
| overt | Nigeria (Enugu) | 12 | 2 | 6 | 1 |
| overt | Nigeria (Enugu) | 17 | 1 | 2 | 59 |
| overt | Nigeria (Enugu) | 3 | 1 | 3 | 21 |
| overt | Nigeria (Enugu) | 3 | 2 | 5 | 12 |
| overt | Nigeria (Enugu) | 3 | 1 | 3 | 11 |
| overt | Nigeria (Enugu) | 11 | 3 | 7 | 28 |
| overt | Nigeria (Enugu) | 16 | 3 | 1 | 77 |
| overt | Nigeria (Enugu) | 3 | 1 | 3 | 11 |
| overt | Nigeria (Enugu) | 3 | 2 | 3 | 3 |
| overt | Nigeria (Enugu) | 12 | 2 | 1 | 27 |
| overt | Nigeria (Enugu) | 12 | 2 | 6 | 1 |
| overt | Nigeria (Enugu) | 17 | 1 | 2 | 59 |
| overt | Nigeria (Enugu) | 3 | 1 | 3 | 9 |
| overt | Nigeria (Enugu) | 3 | 1 | 3 | 21 |
| overt | Nigeria (Enugu) | 3 | 1 | 3 | 33 |
| overt | Nigeria (Enugu) | 3 | 1 | 3 | 34 |
| overt | Nigeria (Enugu) | 3 | 2 | 3 | 6 |
| overt | Nigeria (Enugu) | 9 | 1 | 2 | 28 |
| overt | Nigeria (Enugu) | 11 | 3 | 7 | 25 |
| overt | Nigeria (Enugu) | 11 | 3 | 7 | 28 |
| overt | Nigeria (Enugu) | 12 | 2 | 1 | 14 |
| overt | Nigeria (Enugu) | 12 | 2 | 1 | 27 |
| overt | Nigeria (Enugu) | 12 | 2 | 6 | 1 |
| overt | Nigeria (Enugu) | 12 | 2 | 6 | 5 |
| overt | Nigeria (Enugu) | 14 | 2 | 5 | 2 |
| overt | Nigeria (Enugu) | 14 | 3 | 1 | 14 |
| overt | Nigeria (Enugu) | 16 | 3 | 1 | 77 |
| overt | Nigeria (Enugu) | 17 | 1 | 2 | 18 |
| overt | Nigeria (Enugu) | 17 | 1 | 2 | 36 |
| overt | Nigeria (Enugu) | 17 | 1 | 2 | 57 |
| overt | Nigeria (Enugu) | 17 | 1 | 3 | 3 |
| overt | Nigeria (Enugu) | 3 | 1 | 3 | 33 |
| overt | Nigeria (Enugu) | 12 | 2 | 6 | 1 |
| overt | Nigeria (Enugu) | 3 | 1 | 3 | 33 |
| overt | Nigeria (Enugu) | 12 | 2 | 6 | 6 |
| overt | Nigeria (Enugu) | 17 | 1 | 2 | 57 |
| overt | Nigeria (Enugu) | 3 | 1 | 3 | 7 |
| overt | Nigeria (Enugu) | 3 | 1 | 3 | 31 |
| overt | Nigeria (Enugu) | 3 | 1 | 3 | 34 |
| overt | Nigeria (Enugu) | 12 | 2 | 6 | 1 |
| overt | Nigeria (Enugu) | 14 | 2 | 5 | 2 |
| overt | Nigeria (Enugu) | 14 | 3 | 1 | 59 |
| overt | Nigeria (Enugu) | 1 | 1 | 3 | 9 |
| overt | Nigeria (Enugu) | 3 | 1 | 3 | 11 |
| overt | Nigeria (Enugu) | 3 | 1 | 3 | 30 |
| overt | Nigeria (Enugu) | 3 | 1 | 3 | 33 |

| | | | | | |
|---|---|---|---|---|---|
| overt | Nigeria (Enugu) | 3 | 2 | 3 | 6 |
| overt | Nigeria (Enugu) | 3 | 2 | 5 | 12 |
| overt | Nigeria (Enugu) | 4 | 2 | 1 | 10 |
| overt | Nigeria (Enugu) | 4 | 2 | 3 | 16 |
| overt | Nigeria (Enugu) | 4 | 2 | 3 | 30 |
| overt | Nigeria (Enugu) | 12 | 2 | 1 | 27 |
| overt | Nigeria (Enugu) | 11 | 3 | 7 | 28 |
| overt | Nigeria (Enugu) | 12 | 2 | 6 | 1 |
| overt | Nigeria (Enugu) | 16 | 3 | 1 | 77 |
| overt | Nigeria (Enugu) | 17 | 1 | 2 | 59 |
| overt | Nigeria (Enugu) | 17 | 1 | 3 | 3 |
| overt | Nigeria (Enugu) | 17 | 1 | 2 | 51 |
| overt | Nigeria (Enugu) | 3 | 1 | 3 | 7 |
| overt | Nigeria (Enugu) | 3 | 1 | 3 | 9 |
| overt | Nigeria (Enugu) | 3 | 1 | 3 | 10 |
| overt | Nigeria (Enugu) | 3 | 1 | 3 | 11 |
| overt | Nigeria (Enugu) | 3 | 1 | 3 | 21 |
| overt | Nigeria (Enugu) | 3 | 1 | 3 | 26 |
| overt | Nigeria (Enugu) | 3 | 1 | 3 | 27 |
| overt | Nigeria (Enugu) | 3 | 1 | 3 | 32 |
| overt | Nigeria (Enugu) | 3 | 1 | 3 | 34 |
| mystery | Nigeria (Enugu) | 4 | 1 | 2 | 13 |
| mystery | Nigeria (Enugu) | 4 | 1 | 2 | 39 |
| mystery | Nigeria (Enugu) | 4 | 1 | 2 | 40 |
| mystery | Nigeria (Enugu) | 4 | 1 | 2 | 43 |
| mystery | Nigeria (Enugu) | 4 | 1 | 2 | 45 |
| overt | Nigeria (Enugu) | 4 | 2 | 1 | 10 |
| overt | Nigeria (Enugu) | 4 | 2 | 3 | 1 |
| overt | Nigeria (Enugu) | 4 | 2 | 3 | 16 |
| overt | Nigeria (Enugu) | 4 | 2 | 3 | 20 |
| overt | Nigeria (Enugu) | 4 | 2 | 3 | 24 |
| overt | Nigeria (Enugu) | 4 | 2 | 3 | 26 |
| overt | Nigeria (Enugu) | 4 | 2 | 3 | 37 |
| mystery | Nigeria (Enugu) | 5 | 1 | 2 | 44 |
| overt | Nigeria (Enugu) | 9 | 1 | 1 | 5 |
| overt | Nigeria (Enugu) | 9 | 1 | 1 | 7 |
| overt | Nigeria (Enugu) | 9 | 1 | 1 | 10 |
| overt | Nigeria (Enugu) | 9 | 1 | 2 | 25 |
| overt | Nigeria (Enugu) | 9 | 1 | 2 | 28 |
| overt | Nigeria (Enugu) | 11 | 3 | 5 | 20 |
| overt | Nigeria (Enugu) | 11 | 3 | 7 | 1 |
| overt | Nigeria (Enugu) | 11 | 3 | 7 | 4 |
| overt | Nigeria (Enugu) | 11 | 3 | 7 | 25 |
| overt | Nigeria (Enugu) | 11 | 3 | 7 | 28 |
| overt | Nigeria (Enugu) | 11 | 3 | 7 | 33 |
| overt | Nigeria (Enugu) | 12 | 2 | 1 | 2 |
| overt | Nigeria (Enugu) | 12 | 2 | 1 | 10 |
| overt | Nigeria (Enugu) | 12 | 2 | 1 | 14 |
| overt | Nigeria (Enugu) | 12 | 2 | 1 | 15 |
| overt | Nigeria (Enugu) | 12 | 2 | 6 | 1 |

| | | | | | |
|---|---|---|---|---|---|
| overt | Nigeria (Enugu) | 12 | 2 | 6 | 5 |
| overt | Nigeria (Enugu) | 14 | 2 | 5 | 2 |
| overt | Nigeria (Enugu) | 14 | 2 | 5 | 6 |
| overt | Nigeria (Enugu) | 14 | 2 | 5 | 22 |
| overt | Nigeria (Enugu) | 14 | 2 | 5 | 25 |
| overt | Nigeria (Enugu) | 14 | 3 | 1 | 8 |
| overt | Nigeria (Enugu) | 14 | 3 | 1 | 10 |
| overt | Nigeria (Enugu) | 14 | 3 | 1 | 21 |
| overt | Nigeria (Enugu) | 14 | 3 | 1 | 37 |
| overt | Nigeria (Enugu) | 14 | 3 | 1 | 53 |
| overt | Nigeria (Enugu) | 14 | 3 | 1 | 59 |
| overt | Nigeria (Enugu) | 14 | 3 | 2 | 1 |
| overt | Nigeria (Enugu) | 15 | 2 | 4 | 7 |
| overt | Nigeria (Enugu) | 15 | 2 | 4 | 8 |
| overt | Nigeria (Enugu) | 15 | 2 | 4 | 11 |
| overt | Nigeria (Enugu) | 15 | 2 | 4 | 14 |
| overt | Nigeria (Enugu) | 15 | 2 | 4 | 22 |
| overt | Nigeria (Enugu) | 15 | 2 | 4 | 23 |
| overt | Nigeria (Enugu) | 15 | 2 | 4 | 25 |
| overt | Nigeria (Enugu) | 15 | 2 | 4 | 39 |
| overt | Nigeria (Enugu) | 15 | 2 | 4 | 44 |
| overt | Nigeria (Enugu) | 16 | 3 | 1 | 74 |
| overt | Nigeria (Enugu) | 16 | 3 | 1 | 80 |
| overt | Nigeria (Enugu) | 16 | 3 | 1 | 83 |
| overt | Nigeria (Enugu) | 16 | 3 | 3 | 5 |
| overt | Nigeria (Enugu) | 17 | 1 | 2 | 15 |
| overt | Nigeria (Enugu) | 17 | 1 | 2 | 33 |
| overt | Nigeria (Enugu) | 17 | 1 | 2 | 36 |
| overt | Nigeria (Enugu) | 17 | 1 | 2 | 39 |
| overt | Nigeria (Enugu) | 17 | 1 | 2 | 57 |
| overt | Nigeria (Enugu) | 17 | 1 | 3 | 3 |
| mystery | Nigeria (Enugu) | 4 | 1 | 2 | 43 |
| overt | Nigeria (Enugu) | 17 | 1 | 2 | 22 |
| overt | Nigeria (Enugu) | 9 | 1 | 2 | 20 |
| overt | Nigeria (Enugu) | 12 | 2 | 1 | 17 |
| overt | Nigeria (Enugu) | 14 | 3 | 1 | 59 |
| mystery | Nigeria (Enugu) | 5 | 1 | 2 | 42 |
| overt | Nigeria (Enugu) | 3 | 1 | 3 | 7 |
| overt | Nigeria (Enugu) | 12 | 2 | 6 | 1 |
| overt | Nigeria (Enugu) | 14 | 3 | 7 | 62 |
| overt | Nigeria (Enugu) | 15 | 2 | 4 | 25 |
| overt | Nigeria (Enugu) | 17 | 1 | 2 | 22 |
| overt | Nigeria (Enugu) | 3 | 1 | 3 | 10 |
| overt | Nigeria (Enugu) | 3 | 1 | 3 | 14 |
| overt | Nigeria (Enugu) | 3 | 1 | 3 | 21 |
| overt | Nigeria (Enugu) | 3 | 1 | 3 | 27 |
| overt | Nigeria (Enugu) | 3 | 1 | 3 | 30 |
| overt | Nigeria (Enugu) | 3 | 1 | 3 | 34 |
| overt | Nigeria (Enugu) | 3 | 2 | 5 | 12 |
| mystery | Nigeria (Enugu) | 4 | 1 | 2 | 43 |

| | | | | | |
|---|---|---|---|---|---|
| overt | Nigeria (Enugu) | 4 | 2 | 1 | 10 |
| overt | Nigeria (Enugu) | 4 | 2 | 3 | 8 |
| overt | Nigeria (Enugu) | 4 | 2 | 3 | 20 |
| overt | Nigeria (Enugu) | 4 | 2 | 3 | 21 |
| overt | Nigeria (Enugu) | 9 | 1 | 1 | 6 |
| overt | Nigeria (Enugu) | 9 | 1 | 1 | 16 |
| overt | Nigeria (Enugu) | 9 | 1 | 2 | 19 |
| overt | Nigeria (Enugu) | 9 | 1 | 2 | 29 |
| overt | Nigeria (Enugu) | 11 | 3 | 5 | 20 |
| overt | Nigeria (Enugu) | 11 | 3 | 7 | 1 |
| overt | Nigeria (Enugu) | 11 | 3 | 7 | 28 |
| overt | Nigeria (Enugu) | 11 | 3 | 7 | 33 |
| overt | Nigeria (Enugu) | 12 | 2 | 1 | 2 |
| overt | Nigeria (Enugu) | 12 | 2 | 1 | 14 |
| overt | Nigeria (Enugu) | 12 | 2 | 1 | 28 |
| overt | Nigeria (Enugu) | 12 | 2 | 6 | 1 |
| overt | Nigeria (Enugu) | 14 | 2 | 5 | 2 |
| overt | Nigeria (Enugu) | 14 | 2 | 5 | 6 |
| overt | Nigeria (Enugu) | 14 | 3 | 1 | 37 |
| overt | Nigeria (Enugu) | 14 | 3 | 1 | 59 |
| overt | Nigeria (Enugu) | 14 | 3 | 2 | 1 |
| overt | Nigeria (Enugu) | 15 | 2 | 4 | 8 |
| overt | Nigeria (Enugu) | 15 | 2 | 4 | 14 |
| overt | Nigeria (Enugu) | 15 | 2 | 4 | 22 |
| overt | Nigeria (Enugu) | 15 | 2 | 4 | 25 |
| overt | Nigeria (Enugu) | 15 | 2 | 4 | 36 |
| overt | Nigeria (Enugu) | 15 | 2 | 4 | 42 |
| overt | Nigeria (Enugu) | 16 | 3 | 1 | 77 |
| overt | Nigeria (Enugu) | 16 | 3 | 1 | 83 |
| overt | Nigeria (Enugu) | 16 | 3 | 2 | 5 |
| overt | Nigeria (Enugu) | 17 | 1 | 2 | 15 |
| overt | Nigeria (Enugu) | 17 | 1 | 2 | 19 |
| overt | Nigeria (Enugu) | 17 | 1 | 2 | 22 |
| overt | Nigeria (Enugu) | 17 | 1 | 2 | 36 |
| overt | Nigeria (Enugu) | 17 | 1 | 2 | 46 |
| overt | Nigeria (Enugu) | 17 | 1 | 2 | 57 |
| overt | Nigeria (Enugu) | 17 | 1 | 2 | 59 |
| overt | Nigeria (Enugu) | 17 | 1 | 3 | 2 |
| overt | Nigeria (Enugu) | 17 | 1 | 3 | 3 |
| overt | Nigeria (Enugu) | 11 | 3 | 6 | 1 |
| overt | Nigeria (Enugu) | 3 | 1 | 3 | 10 |
| overt | Nigeria (Enugu) | 4 | 2 | 3 | 16 |
| overt | Nigeria (Enugu) | 12 | 2 | 6 | 6 |
| overt | Nigeria (Enugu) | 15 | 2 | 4 | 4 |
| overt | Nigeria (Enugu) | 15 | 2 | 4 | 4 |
| overt | Nigeria (Enugu) | 15 | 2 | 4 | 4 |
| overt | Nigeria (Enugu) | 11 | 3 | 7 | 31 |
| overt | Nigeria (Enugu) | 11 | 3 | 7 | 33 |
| overt | Nigeria (Enugu) | 3 | 1 | 3 | 9 |
| overt | Nigeria (Enugu) | 3 | 1 | 3 | 14 |

| | | | | | |
|---|---|---|---|---|---|
| overt | Nigeria (Enugu) | 4 | 2 | 3 | 37 |
| overt | Nigeria (Enugu) | 12 | 2 | 6 | 5 |
| overt | Nigeria (Enugu) | 14 | 3 | 1 | 8 |
| overt | Nigeria (Enugu) | 16 | 3 | 1 | 83 |
| overt | Nigeria (Enugu) | 12 | 2 | 6 | 5 |
| overt | Nigeria (Enugu) | 3 | 1 | 3 | 10 |
| overt | Nigeria (Enugu) | 4 | 2 | 1 | 10 |
| overt | Nigeria (Enugu) | 12 | 2 | 1 | 10 |
| overt | Nigeria (Enugu) | 3 | 2 | 3 | 3 |
| overt | Nigeria (Enugu) | 12 | 2 | 1 | 27 |
| overt | Nigeria (Enugu) | 3 | 1 | 3 | 30 |
| overt | Nigeria (Enugu) | 16 | 3 | 1 | 10 |
| overt | Nigeria (Enugu) | 9 | 1 | 1 | 6 |
| overt | Nigeria (Enugu) | 9 | 1 | 2 | 29 |
| overt | Nigeria (Enugu) | 12 | 2 | 1 | 27 |
| overt | Nigeria (Enugu) | 12 | 2 | 6 | 5 |
| overt | Nigeria (Enugu) | 3 | 1 | 3 | 10 |
| overt | Nigeria (Enugu) | 3 | 1 | 3 | 11 |
| overt | Nigeria (Enugu) | 3 | 1 | 3 | 33 |
| overt | Nigeria (Enugu) | 4 | 2 | 1 | 10 |
| overt | Nigeria (Enugu) | 4 | 2 | 3 | 16 |
| overt | Nigeria (Enugu) | 4 | 2 | 3 | 28 |
| overt | Nigeria (Enugu) | 9 | 1 | 2 | 29 |
| overt | Nigeria (Enugu) | 11 | 3 | 7 | 33 |
| overt | Nigeria (Enugu) | 12 | 2 | 1 | 1 |
| overt | Nigeria (Enugu) | 12 | 2 | 1 | 27 |
| overt | Nigeria (Enugu) | 12 | 2 | 6 | 1 |
| overt | Nigeria (Enugu) | 14 | 2 | 5 | 2 |
| overt | Nigeria (Enugu) | 16 | 3 | 5 | 11 |
| overt | Nigeria (Enugu) | 15 | 2 | 4 | 4 |
| overt | Nigeria (Enugu) | 3 | 1 | 3 | 11 |
| mystery | Nigeria (Enugu) | 4 | 1 | 2 | 13 |
| mystery | Nigeria (Enugu) | 4 | 1 | 2 | 43 |
| overt | Nigeria (Enugu) | 9 | 1 | 2 | 25 |
| overt | Nigeria (Enugu) | 12 | 2 | 6 | 5 |
| mystery | Nigeria (Enugu) | 4 | 1 | 2 | 43 |
| overt | Nigeria (Enugu) | 12 | 2 | 1 | 14 |
| overt | Nigeria (Enugu) | 17 | 1 | 2 | 22 |
| overt | Nigeria (Enugu) | 14 | 3 | 1 | 8 |
| overt | Nigeria (Enugu) | 14 | 3 | 1 | 14 |
| overt | Nigeria (Enugu) | 14 | 3 | 2 | 1 |
| overt | Nigeria (Enugu) | 4 | 2 | 3 | 38 |
| overt | Nigeria (Enugu) | 16 | 3 | 1 | 83 |
| overt | Nigeria (Enugu) | 3 | 1 | 3 | 30 |
| overt | Nigeria (Enugu) | 3 | 1 | 3 | 9 |
| overt | Nigeria (Enugu) | 3 | 1 | 3 | 10 |
| overt | Nigeria (Enugu) | 3 | 1 | 3 | 11 |
| overt | Nigeria (Enugu) | 3 | 1 | 3 | 14 |
| overt | Nigeria (Enugu) | 3 | 1 | 3 | 21 |
| overt | Nigeria (Enugu) | 3 | 1 | 3 | 27 |

| | | | | | |
|---|---|---|---|---|---|
| overt | Nigeria (Enugu) | 3 | 1 | 3 | 31 |
| overt | Nigeria (Enugu) | 3 | 1 | 3 | 33 |
| overt | Nigeria (Enugu) | 3 | 1 | 3 | 34 |
| overt | Nigeria (Enugu) | 4 | 2 | 1 | 10 |
| overt | Nigeria (Enugu) | 9 | 1 | 1 | 7 |
| overt | Nigeria (Enugu) | 9 | 1 | 1 | 10 |
| overt | Nigeria (Enugu) | 9 | 1 | 2 | 20 |
| overt | Nigeria (Enugu) | 9 | 1 | 2 | 29 |
| overt | Nigeria (Enugu) | 11 | 3 | 7 | 33 |
| overt | Nigeria (Enugu) | 12 | 2 | 1 | 10 |
| overt | Nigeria (Enugu) | 12 | 2 | 1 | 14 |
| overt | Nigeria (Enugu) | 12 | 2 | 1 | 15 |
| overt | Nigeria (Enugu) | 12 | 2 | 1 | 27 |
| overt | Nigeria (Enugu) | 12 | 2 | 1 | 28 |
| overt | Nigeria (Enugu) | 14 | 2 | 5 | 2 |
| overt | Nigeria (Enugu) | 14 | 3 | 1 | 8 |
| overt | Nigeria (Enugu) | 14 | 3 | 1 | 10 |
| overt | Nigeria (Enugu) | 14 | 3 | 1 | 14 |
| overt | Nigeria (Enugu) | 14 | 3 | 1 | 42 |
| overt | Nigeria (Enugu) | 15 | 2 | 4 | 7 |
| overt | Nigeria (Enugu) | 15 | 2 | 4 | 8 |
| overt | Nigeria (Enugu) | 15 | 2 | 4 | 11 |
| overt | Nigeria (Enugu) | 15 | 2 | 4 | 14 |
| overt | Nigeria (Enugu) | 15 | 2 | 4 | 22 |
| overt | Nigeria (Enugu) | 15 | 2 | 4 | 42 |
| overt | Nigeria (Enugu) | 15 | 2 | 4 | 44 |
| overt | Nigeria (Enugu) | 16 | 3 | 1 | 76 |
| overt | Nigeria (Enugu) | 16 | 3 | 1 | 83 |
| mystery | Nigeria (Enugu) | 5 | 1 | 2 | 42 |
| overt | Nigeria (Enugu) | 12 | 2 | 6 | 6 |
| overt | Nigeria (Enugu) | 3 | 1 | 3 | 14 |
| overt | Nigeria (Enugu) | 3 | 1 | 3 | 30 |
| overt | Nigeria (Enugu) | 12 | 2 | 6 | 1 |
| overt | Nigeria (Enugu) | 3 | 1 | 3 | 10 |
| overt | Nigeria (Enugu) | 3 | 1 | 3 | 11 |
| overt | Nigeria (Enugu) | 3 | 1 | 3 | 14 |
| overt | Nigeria (Enugu) | 3 | 1 | 3 | 34 |
| mystery | Nigeria (Enugu) | 4 | 1 | 2 | 43 |
| mystery | Nigeria (Enugu) | 5 | 1 | 2 | 44 |
| overt | Nigeria (Enugu) | 9 | 1 | 2 | 28 |
| overt | Nigeria (Enugu) | 12 | 2 | 6 | 1 |
| overt | Nigeria (Enugu) | 3 | 1 | 3 | 9 |
| overt | Nigeria (Enugu) | 3 | 1 | 3 | 10 |
| overt | Nigeria (Enugu) | 3 | 1 | 3 | 21 |
| overt | Nigeria (Enugu) | 3 | 1 | 3 | 33 |
| overt | Nigeria (Enugu) | 3 | 1 | 3 | 34 |
| overt | Nigeria (Enugu) | 3 | 2 | 3 | 3 |
| mystery | Nigeria (Enugu) | 4 | 1 | 2 | 43 |
| mystery | Nigeria (Enugu) | 5 | 1 | 2 | 51 |
| overt | Nigeria (Enugu) | 9 | 1 | 1 | 10 |

| | | | | | |
|---|---|---|---|---|---|
| overt | Nigeria (Enugu) | 9 | 1 | 2 | 25 |
| overt | Nigeria (Enugu) | 12 | 2 | 6 | 1 |
| overt | Nigeria (Enugu) | 12 | 2 | 6 | 5 |
| overt | Nigeria (Enugu) | 14 | 3 | 1 | 10 |
| overt | Nigeria (Enugu) | 14 | 3 | 1 | 14 |
| overt | Nigeria (Enugu) | 14 | 3 | 1 | 21 |
| overt | Nigeria (Enugu) | 15 | 2 | 4 | 8 |
| overt | Nigeria (Enugu) | 15 | 2 | 4 | 14 |
| overt | Nigeria (Enugu) | 15 | 2 | 4 | 27 |
| overt | Nigeria (Enugu) | 17 | 1 | 3 | 3 |
| overt | Nigeria (Enugu) | 3 | 1 | 3 | 10 |
| overt | Nigeria (Enugu) | 3 | 1 | 3 | 26 |
| overt | Nigeria (Enugu) | 3 | 1 | 3 | 34 |
| overt | Nigeria (Enugu) | 3 | 2 | 5 | 12 |
| mystery | Nigeria (Enugu) | 4 | 1 | 2 | 13 |
| overt | Nigeria (Enugu) | 4 | 2 | 1 | 10 |
| overt | Nigeria (Enugu) | 4 | 2 | 3 | 8 |
| overt | Nigeria (Enugu) | 4 | 2 | 3 | 16 |
| overt | Nigeria (Enugu) | 4 | 2 | 3 | 38 |
| overt | Nigeria (Enugu) | 9 | 1 | 1 | 6 |
| overt | Nigeria (Enugu) | 9 | 1 | 1 | 16 |
| overt | Nigeria (Enugu) | 12 | 2 | 1 | 15 |
| overt | Nigeria (Enugu) | 12 | 2 | 6 | 1 |
| overt | Nigeria (Enugu) | 15 | 2 | 4 | 14 |
| overt | Nigeria (Enugu) | 15 | 2 | 4 | 7 |
| overt | Nigeria (Enugu) | 15 | 2 | 4 | 8 |
| overt | Nigeria (Enugu) | 15 | 2 | 4 | 11 |
| overt | Nigeria (Enugu) | 15 | 2 | 4 | 27 |
| overt | Nigeria (Enugu) | 15 | 2 | 4 | 44 |
| overt | Nigeria (Enugu) | 16 | 3 | 1 | 83 |
| overt | Nigeria (Enugu) | 3 | 1 | 3 | 10 |
| overt | Nigeria (Enugu) | 3 | 1 | 3 | 11 |
| overt | Nigeria (Enugu) | 3 | 2 | 5 | 12 |
| overt | Nigeria (Enugu) | 12 | 2 | 6 | 1 |

| outletcode | productid | brandname | genname1 | strengthgen1 | permlgen1 |
|---|---|---|---|---|---|
| | 1 | X | Artemether | 20 | |
| | 1 | X | Artemether | 20 | |
| | 2 | X | Artemether | 20 | |
| | 2 | X | Artemether | 20 | |
| | 2 | X | Artemether | 20 | |
| | 1 | X | Artemether | 20 | |
| | 2 | X | Artemether | 20 | |
| | 1 | X | Artemether | 20 | |
| | 2 | X | Artemether | 20 | |
| | 2 | X | Artemether | 20 | |
| | 1 | X | Artemether | 20 | |
| | 1 | X | Artemether | 20 | |
| | 2 | X | Artemether | 20 | |
| | 2 | X | Artemether | 20 | |
| | 2 | X | Artemether | 20 | |
| | 2 | X | Artemether | 20 | |
| | 2 | X | Artemether | 20 | |
| | 1 | X | Artemether | 20 | |
| | 1 | X | Artemether | 20 | |
| | 1 | X | Artemether | 20 | |
| | 2 | X | Artemether | 20 | |
| | 2 | X | Artemether | 20 | |
| | 2 | X | Artemether | 20 | |
| | 2 | X | Artemether | 20 | |
| | 1 | X | Artemether | 20 | |
| | 2 | X | Artemether | 20 | |
| | 2 | X | Artemether | 20 | |
| | 2 | X | Artemether | 20 | |
| | 2 | X | Artemether | 20 | |
| | 4 | X | Artemether | 20 | |
| | 1 | X | Artemether | 20 | |
| | 2 | X | Artemether | 20 | |
| | 2 | X | Artemether | 20 | |
| | 2 | X | Artemether | 20 | |
| | 2 | X | Artemether | 20 | |
| | 2 | X | Artemether | 20 | |
| | 2 | X | Artemether | 20 | |
| | 2 | X | Artemether | 20 | |
| | 2 | X | Artemether | 20 | |
| | 2 | X | Artemether | 20 | |
| | 2 | X | Artemether | 20 | |
| | 1 | X | Artemether | 20 | |
| | 3 | X | Artemether | 20 | |
| | 4 | X | Artemether | 20 | |
| | 2 | X | Artemether | 20 | |
| | 2 | X | Artemether | 20 | |

| | | | |
|---|---|---|---|
| 1 | X | Artemether | 20 |
| 1 | X | Artemether | 20 |
| 2 | X | Artemether | 20 |
| 2 | X | Artemether | 20 |
| 1 | X | Artemether | 20 |
| 2 | X | Artemether | 20 |
| 2 | X | Artemether | 20 |
| 2 | X | Artemether | 20 |
| 2 | X | Artemether | 20 |
| 1 | X | Artemether | 20 |
| 2 | X | Artemether | 20 |
| 1 | X | Artemether | 20 |
| 1 | X | Artemether | 40 |
| 1 | X | Artemether | 40 |
| 2 | X | Artemether | 40 |
| 1 | X | Artemether | 40 |
| 1 | X | Artemether | 40 |
| 2 | X | Artemether | 40 |
| 2 | X | Artemether | 40 |
| 1 | X | Artemether | 80 |
| 1 | X | Artemether | 80 |
| 2 | X | Artemether | 80 |
| 2 | X | Artemether | 80 |
| 2 | X | Artemether | 80 |
| 1 | X | Artemether | 80 |
| 2 | X | Artemether | 80 |
| 1 | X | Artemether | 80 |
| 1 | X | Artemether | 80 |
| 2 | X | Artemether | 80 |
| 2 | X | Artemether | 80 |
| 1 | X | Artemether | 80 |
| 1 | X | Artemether | 80 |
| 1 | X | Artemether | 80 |
| 2 | X | Artemether | 80 |
| 1 | X | Artemether | 80 |
| 1 | X | Artemether | 80 |
| 1 | X | Artemether | 80 |
| 1 | X | Artemether | 80 |
| 2 | X | Artemether | 80 |
| 2 | X | Artemether | 80 |
| 2 | X | Artemether | 80 |
| 1 | X | Artemether | 80 |
| 1 | X | Artemether | 80 |
| 2 | X | Artemether | 80 |
| 2 | X | Artemether | 80 |
| 2 | X | Artemether | 80 |
| 2 | X | Artemether | 80 |
| 2 | X | Artemether | 80 |
| 2 | X | Artemether | 80 |
| 1 | X | Artemether | 80 |

| | | | | |
|---|---|---|---|---|
| 2 | X | Artemether | 80 | |
| 2 | X | Artemether | 80 | |
| 2 | X | Artemether | 80 | |
| 2 | X | Artemether | 80 | |
| 1 | X | Artemether | 80 | |
| 4 | X | Artemether | 80 | |
| 1 | X | Artemether | 80 | |
| 1 | X | Artemether | 80 | |
| 2 | X | Artemether | 80 | |
| 2 | X | Artemether | 80 | |
| 1 | X | Artemether | 80 | |
| 1 | X | Artemether | 80 | |
| 1 | X | Artemether | 80 | |
| 2 | X | Artemether | 80 | |
| 2 | X | Artemether | 80 | |
| 2 | X | Artemether | 80 | |
| 1 | X | Artemether | 80 | |
| 4 | X | Artemether | 80 | |
| 1 | X | Artemether | 80 | |
| 1 | X | Artemether | 80 | |
| 2 | X | Artemether | 80 | |
| 2 | X | Artemether | 80 | |
| 1 | X | Artemether | 80 | |
| 1 | X | Artemether | 80 | |
| 2 | X | Artemether | 80 | |
| 2 | X | Artemether | 80 | |
| 2 | X | Artemether | 80 | |
| 2 | X | Artemether | 80 | |
| 2 | X | Artemether | 80 | |
| 2 | X | Artemether | | 20/5 |
| 2 | X | Artemether | | 20/5 |
| 2 | X | Artemether | | 20/5 |
| 1 | X | Artemether | | 20/5 |
| 1 | X | Artesunate | 50 | |
| 2 | X | Artesunate | 50 | |
| 2 | X | Artesunate | 50 | |
| 2 | X | Artesunate | 50 | |
| 2 | X | Artesunate | 50 | |
| 2 | X | Artesunate | 50 | |
| 2 | X | Artesunate | 50 | |
| 2 | X | Artesunate | 50 | |
| 2 | X | Artesunate | 50 | |
| 2 | X | Artesunate | 50 | |
| 1 | X | Artesunate | 100 | |
| 2 | X | Artesunate | 100 | |
| 3 | X | Artesunate | 100 | |
| 4 | X | Artesunate | 100 | |
| 1 | X | Artesunate | 100 | |
| 1 | X | Artesunate | 100 | |

| | | | |
|---|---|---|---|
| 2 | X | Artesunate | 100 |
| 2 | X | Artesunate | 100 |
| 1 | X | Artesunate | 100 |
| 2 | X | Artesunate | 100 |
| 1 | X | Artesunate | 100 |
| 3 | X | Artesunate | 100 |
| 2 | X | Artesunate | 100 |
| 1 | X | Artesunate | 100 |
| 2 | X | Artesunate | 100 |
| 2 | X | Artesunate | 100 |
| 2 | X | Artesunate | 100 |
| 1 | X | Artesunate | 200 |
| 2 | X | Artesunate | 200 |
| 2 | X | Artesunate | 200 |
| 1 | X | Artesunate | 200 |
| 2 | X | Artesunate | 200 |
| 2 | X | Artesunate | 200 |
| 2 | X | Artesunate | 200 |
| 2 | X | Artesunate | 200 |
| 1 | X | Artesunate | 200 |
| 3 | X | Artesunate | 200 |
| 1 | X | Artesunate | 200 |
| 1 | X | Artesunate | 200 |
| 1 | X | Artesunate | 200 |
| 2 | X | Artesunate | 200 |
| 1 | X | Artesunate | 200 |
| 1 | X | Artesunate | 200 |
| 2 | X | Artesunate | 200 |
| 2 | X | Artesunate | 200 |
| 3 | X | Artesunate | 200 |
| 1 | X | Dihydroartemisinin | 30 |
| 1 | X | Dihydroartemisinin | 30 |
| 2 | X | Dihydroartemisinin | 30 |
| 2 | X | Dihydroartemisinin | 30 |
| 2 | X | Dihydroartemisinin | 30 |
| 2 | X | Dihydroartemisinin | 30 |
| 2 | X | Dihydroartemisinin | 30 |
| 2 | X | Dihydroartemisinin | 30 |
| 2 | X | Dihydroartemisinin | 30 |
| 2 | X | Dihydroartemisinin | 30 |
| 2 | X | Dihydroartemisinin | 30 |
| 2 | X | Dihydroartemisinin | 30 |
| 2 | X | Dihydroartemisinin | 30 |
| 1 | X | Dihydroartemisinin | 30 |
| 4 | X | Dihydroartemisinin | 30 |
| 1 | X | Dihydroartemisinin | 40 |
| 1 | X | Dihydroartemisinin | 40 |
| 2 | X | Dihydroartemisinin | 40 |

| | | | |
|---|---|---|---|
| 2 | X | Dihydroartemisinin | 40 |
| 2 | X | Dihydroartemisinin | 40 |
| 1 | X | Dihydroartemisinin | 40 |
| 2 | X | Dihydroartemisinin | 40 |
| 2 | X | Dihydroartemisinin | 40 |
| 1 | X | Dihydroartemisinin | 60 |
| 2 | X | Dihydroartemisinin | 60 |

| productid | outlet_type | samplecode | brandname | otherbname | dateofpurchase |
|---|---|---|---|---|---|
| 57-67 | PMV | 1-3-1-57-57-67 | x | | 15-Dec-12 |
| 17-67b | Pharmacy | 5-1-2-17-17-67b | x | | 18-Dec-12 |
| 34-67a | PMV | 15-2-3-34-34-67a | x | | 08-Dec-12 |
| 4-46 | Pharmacy | 1-3-1-4-04-46 | x | | 10-Dec-12 |
| 21-46A | PMV | 1-3-1-21-21-46A | x | | 13-Dec-12 |
| 80-46 | PMV | 1-3-1-80-80-46 | x | | 18-Dec-12 |
| 7-46 | PMV | 2-3-5-7-7-46 | x | | 12-Dec-12 |
| 29-46 | PMV | 2-3-5-29-29-46 | x | | 11-Dec-12 |
| 2-26 | Pharmacy | 3-2-1-2-02-26 | x | | 10-Dec-12 |
| 42-46 | PMV | 3-2-4-42-42-46 | x | | 13-Dec-12 |
| 12-46 | Pharmacy | 4-1-2-12-12-46 | x | | 14-Dec-12 |
| 0-46 | Publichealth fa | 5-1-2-0-0-46 | x | | 21-Dec-12 |
| 21-46 | Pharmacy | 5-1-2-21-21-46 | x | | 14-Dec-12 |
| 30-46 | PMV | 5-1-2-30-30-46 | x | | 10-Dec-12 |
| 31-46 | PMV | 5-1-2-31-31-46 | x | | 12-Dec-12 |
| 46-46 | PMV | 5-1-2-46-46-46 | x | | 14-Dec-12 |
| 49-496 | PMV | 5-1-2-49-49-496 | x | | 14-Dec-12 |
| 0-40 | Publichealth fa | 5-2-1-0-0-40 | x | | 20-Dec-12 |
| 26-46 | PMV | 6-2-1-26-26-46 | x | | 14-Dec-12 |
| 28-46 | Pharmacy | 6-2-1-28-28-46 | x | | 14-Dec-12 |
| 28-67A | Pharmacy | 6-2-1-28-28-67A | x | | 14-Dec-12 |
| 6-46 | Pharmacy | 6-2-3-6-06-46 | x | | 11-Dec-12 |
| 39-67 | PMV | 6-2-3-39-39-67 | x | | 13-Dec-12 |
| 20-44 | PMV | 7-2-4-20-20-44 | x | | 10-Dec-12 |
| 0-46 | Publichealth fa | 7-3-5-0-0-46 | x | | 20-Dec-12 |
| 0-46b | Publichealth fa | 8-2-1-0-00-46b | x | | 20-Dec-12 |
| 44-46 | PMV | 8-2-4-44-44-46 | x | | 12-Dec-12 |
| 0-46A | Publichealth fa | 9-2-1-0-0-46A | x | | 21-Dec-12 |
| 10-46 | PMV | 9-2-2-10-10-46 | x | | 18-Dec-12 |
| 18-44 | PMV | 10-3-1-18-18-44 | x | | 14-Dec-12 |
| 31-46 | PMV | 10-3-1-31-31-46 | x | | 14-Dec-12 |
| 53-46 | PMV | 10-3-1-53-53-46 | x | | 18-Dec-12 |
| 65-46a | PMV | 10-3-1-65-65-46a | x | | 14-Dec-12 |
| 2-46 | Pharmacy | 11-2-5-2-02-46 | x | | 12-Dec-12 |
| 24-44 | PMV | 12-2-5-24-24-44 | x | | 10-Dec-12 |
| 18-46 | PMV | 12-3-7-18-18-46 | x | | 11-Dec-12 |
| 0-46 | Publichealth fa | 13-2-1-0-0-46 | x | | 21-Dec-12 |
| 7-44 | Pharmacy | 14-1-1-7-07-44 | x | | 14-Dec-12 |
| 7-46 | Pharmacy | 14-1-1-7-07-46 | x | | 14-Dec-12 |

| | | | | |
|---|---|---|---|---|
| 18-44 | PMV | 14-1-1-18-18-44 | x | 14-Dec-12 |
| 5-45 | Pharmacy | 14-1-3-5-05-45 | x | 11-Dec-12 |
| 10-46 | Pharmacy | 14-1-3-10-10-46 | x | 13-Dec-12 |
| 23-44 | Pharmacy | 14-1-3-23-23-44 | x | 12-Dec-12 |
| 0-46 | Publichealth fa | 15-2-1-0-0-46 | x | 20-Dec-12 |
| 7-45 | Pharmacy | 16-1-1-7-07-45 | x | 14-Dec-12 |
| 0-46a | Publichealth fa | 16-2-3-0-0-46a | x | 10-Dec-12 |
| 21-46B | PMV | 1-3-1-21-21-46B | x | 13-Dec-12 |
| 28-44 | PMV | 2-3-5-28-28-44 | x | 11-Dec-12 |
| 29-44 | PMV | 2-3-5-29-29-44 | x | 11-Dec-12 |
| 0-46 | Publichealth fa | 3-3-71-0-0-46 | x | 20-Dec-12 |
| 5-44 | Pharmacy | 4-1-2-5-5-44 | x | 13-Dec-12 |
| 9-46 | Pharmacy | 4-1-2-9-09-46 | x | 11-Dec-12 |
| 0-46a | Publichealth fa | 8-2-1-0-00-46a | x | 20-Dec-12 |
| 0-46B | Publichealth fa | 9-2-1-0-0-46B | x | 21-Dec-12 |
| 25-44 | PMV | 10-3-1-25-25-44 | x | 15-Dec-12 |
| 31-45 | PMV | 10-3-1-31-31-45 | x | 14-Dec-12 |
| 65-44 | PMV | 10-3-1-65-65-44 | x | 14-Dec-12 |
| 26-46 | PMV | 11-2-5-26-26-46 | x | 10-Dec-12 |
| 24-46 | PMV | 12-2-5-24-24-46 | x | 10-Dec-12 |
| 0-46 | Publichealth fa | 13-2-1-0-0-46 | x | 21-Dec-12 |
| 0-67 | Publichealth fa | 15-2-1-0-0-67 | x | 20-Dec-12 |
| 0-46B | Publichealth fa | 16-2-3-0-0-46B | x | 10-Dec-12 |
| 0-00A | Publichealth fa | 00-0-0-0-0-00A | x | no date |
| 4-45 | Pharmacy | 1-3-1-4-04-45 | x | 10-Dec-12 |
| 57-44a | PMV | 1-3-1-57-57-44a | x | 15-Dec-12 |
| 5-44 | PMV | 2-3-2-5-05-44 | x | 10-Dec-12 |
| 7-44 | PMV | 2-3-2-7-07-44 | x | 10-Dec-12 |
| 7-45 | PMV | 2-3-2-7-07-45 | x | 10-Dec-12 |
| 8-45 | PMV | 2-3-2-8-08-45 | x | 10-Dec-12 |
| 7-45 | PMV | 2-3-5-7-7-45 | x | 12-Dec-12 |
| 34-44 | PMV | 2-3-5-34-34-44 | x | 12-Dec-12 |
| 42-45 | PMV | 3-2-4-42-42-45 | x | 13-Dec-12 |
| 38-46 | PMV | 4-1-2-38-38-46 | x | 14-Dec-12 |
| 0-45 | Publichealth fa | 5-1-2-0-0-45 | x | 21-Dec-12 |
| 2-45 | Pharmacy | 5-1-2-2-02-45 | x | 10-Dec-12 |
| 21-45 | PMV | 5-1-2-21-21-45 | x | 14-Dec-12 |
| 22-45 | PMV | 5-1-2-22-22-45 | x | 04-Dec-12 |
| 0-45 | Publichealth fa | 5-2-1-0-0-45 | x | 20-Dec-12 |
| 28-45 | Pharmacy | 6-2-1-28-28-45 | x | 14-Dec-12 |
| 1-46 | Pharmacy | 6-2-3-1-01-46 | x | 12-Dec-12 |
| 14-45 | Pharmacy | 6-2-3-14-14-45 | x | 10-Dec-12 |
| 39-46 | PMV | 6-2-3-39-39-46 | x | 13-Dec-12 |
| 20-45 | Pharmacy | 7-2-4-20-20-45 | x | 10-Dec-12 |
| 22-45 | Pharmacy | 7-2-4-22-22-45 | x | 13-Dec-12 |
| 36-46 | PMV | 7-2-4-36-36-46 | x | 13-Dec-12 |
| 0-46 | Publichealth fa | 7-3-5-0-0-46 | x | 20-Dec-12 |
| 0-45 | Publichealth fa | 8-2-1-0-00-45 | x | 20-Dec-12 |
| 0-45 | Publichealth fa | 9-2-1-0-0-45 | x | 21-Dec-12 |
| 10-45 | PMV | 9-2-2-10-10-45 | x | 18-Dec-12 |

| | | | | |
|---|---|---|---|---|
| 11-45 | Pharmacy | 9-2-5-11-11-45 | x | 20-Dec-12 |
| 3-46 | PMV | 9-3-3-3-03-46 | x | 13-Dec-12 |
| 18-45 | PMV | 10-3-1-18-18-45 | x | 14-Dec-12 |
| 21-45 | PMV | 10-3-1-21-21-45 | x | 13-Dec-12 |
| 31-44 | PMV | 10-3-1-31-31-44 | x | 14-Dec-12 |
| 33-45 | PMV | 10-3-1-33-33-45 | x | 15-Dec-12 |
| 65-45 | PMV | 10-3-1-65-65-45 | x | 14-Dec-12 |
| 0-45 | Publichealth fa | 13-2-1-0-0-45 | x | 21-Dec-12 |
| 5-45 | PMV | 14-1-1-5-05-45 | x | 14-Dec-12 |
| 6-14 | PMV | 14-1-1-6-6-14 | x | 14-Dec-12 |
| 16-45 | PMV | 14-1-1-16-16-45 | x | 13-Dec-12 |
| 0-45 | Publichealth fa | 15-2-1-0-0-45 | x | 20-Dec-12 |
| 7-44 | PMV | 16-1-1-7-07-44 | x | 14-Dec-12 |
| 30-44 | PMV | 16-1-3-30-30-44 | x | 18-Dec-12 |
| 0-45A | Publichealth fa | 16-2-3-0-0-45A | x | 10-Dec-12 |
| 10-44 | Pharmacy | 1-3-1-10-10-044 | x | 14-Dec-12 |
| 33-44 | PMV | 1-3-1-33-33-44 | x | 15-Dec-12 |
| 42-44 | PMV | 1-3-1-42-42-44 | x | 13-Dec-12 |
| 57-44b | PMV | 1-3-1-57-57-44b | x | 15-Dec-12 |
| 60-44 | PMV | 1-3-1-60-60-44 | x | 13-Dec-12 |
| 63-44 | PMV | 1-3-1-63-63-44 | x | 13-Dec-12 |
| 69-44 | PMV | 1-3-1-69-69-44 | x | 14-Dec-12 |
| 76-44 | PMV | 1-3-1-76-76-44 | x | 18-Dec-12 |
| 5-67A | PMV | 2-3-2-5-05-67A | x | 10-Dec-12 |
| 6-44 | PMV | 2-3-2-6-06-44 | x | 10-Dec-12 |
| 33-46 | Pharmacy | 2-3-5-33-33-46 | x | 12-Dec-12 |
| 7-44 | Pharmacy | 3-2-4-7-07-44 | x | 11-Dec-12 |
| 13 | PMV | 3-2-4-13-13 | x | 13-Dec-12 |
| 14-44 | PMV | 3-2-4-14-14-44 | x | 12-Dec-12 |
| 15-44 | PMV | 3-2-4-15-15-44 | x | 11-Dec-12 |
| 39-44 | PMV | 3-2-4-39-39-44 | x | 12-Dec-12 |
| 42-44 | PMV | 3-2-4-42-42-44 | x | 13-Dec-12 |
| 43-44 | PMV | 3-2-4-43-43-44 | x | 13-Dec-12 |
| 23-44 | Pharmacy | 4-1-2-23-23-44 | x | 10-Dec-12 |
| 22-44 | Pharmacy | 5-1-2-22-22-44 | x | 04-Dec-12 |
| 55-44 | PMV | 5-1-2-55-55-44 | x | 13-Dec-12 |
| 56-44 | PMV | 5-1-2-56-56-44 | x | 13-Dec-12 |
| 0-44 | Publichealth fa | 5-2-1-0-0-44 | x | 20-Dec-12 |
| 26-44 | PMV | 6-2-1-26-26-44 | x | 14-Dec-12 |
| 28-44 | PMV | 6-2-1-28-28-44 | x | 14-Dec-12 |
| 37-44 | Pharmacy | 6-2-3-37-37-44 | x | 12-Dec-12 |
| 6-45 | Pharmacy | 7-2-4-6-06-45 | x | 11-Dec-12 |
| 27-44 | PMV | 7-2-4-27-27-44 | x | 12-Dec-12 |
| 36-44 | PMV | 7-2-4-36-36-44 | x | 13-Dec-12 |
| 0-44 | Publichealth fa | 7-3-5-0-0-44 | x | 20-Dec-12 |
| 0-44 | Publichealth fa | 8-2-1-0-0-44 | x | 20-Dec-12 |
| 16-44 | PMV | 8-2-4-16-16-44 | x | 13-Dec-12 |
| 34-45 | PMV | 8-2-4-34-34-45 | x | 11-Dec-12 |
| 0-44 | Publichealth fa | 9-2-1-0-0-44 | x | 21-Dec-12 |
| 76-44 | PMV | 9-2-2-2-76-44 | x | 18-Dec-12 |

| | | | | |
|---|---|---|---|---|
| 8-67 | PMV | 9-3-5-8-8-67 | x | 11-Dec-12 |
| 14-45 | Pharmacy | 10-3-1-14-14-45 | x | 18-Dec-12 |
| 18-46 | PMV | 10-3-1-18-18-46 | x | 14-Dec-12 |
| 65-46b | PMV | 10-3-1-65-65-46b | x | 14-Dec-12 |
| 67-46 | PMV | 10-3-1-67-67-46 | x | 14-Dec-12 |
| 22-45 | PMV | 11-2-5-22-22-45 | x | 10-Dec-12 |
| 15-45 | PMV | 11-3-7-15-15-45 | x | 11-Dec-12 |
| 24-45 | PMV | 12-2-5-24-24-45 | x | 10-Dec-12 |
| 18-45 | PMV | 12-3-7-18-18-45 | x | 11-Dec-12 |
| 0-44 | Publichealth fa | 13-2-1-0-0-44 | x | 21-Dec-12 |
| 16-46 | Pharmacy | 13-2-3-16-16-46 | x | 08-Dec-12 |
| 1-45 | Pharmacy | 13-3-7-1-01-45 | x | 11-Dec-12 |
| 25-44 | PMV | 14-1-3-25-25-44 | x | 14-Dec-12 |
| 0-44 | Publichealth fa | 15-2-1-0-0-44 | x | 20-Dec-12 |
| 20-44 | PMV | 15-2-3-20-20-44 | x | 12-Dec-12 |
| 26-44 | PMV | 15-2-3-26-26-44 | x | 08-Dec-12 |
| 11-44 | Pharmacy | 16-1-3-11-11-44 | x | 10-Dec-12 |
| 0-44 | Publichealth fa | 16-2-3-0-0-44 | x | 10-Dec-12 |
| 12-3 | Pharmacy | 1-3-1-12-12-3 | x | 13-Dec-12 |
| 2-3 | Pharmacy | 3-2-4-2-02-03 | x | 10-Dec-12 |
| 8-3 | Pharmacy | 3-2-4-8-08-03 | x | 11-Dec-12 |
| 15-3 | PMV | 3-2-4-15-15-03 | x | 11-Dec-12 |
| 9-3 | Pharmacy | 4-1-2-9-9-3 | x | 11-Dec-12 |
| 23-3 | Pharmacy | 4-1-2-23-23-03 | x | 10-Dec-12 |
| 36-3 | PMV | 4-1-2-36-36-03 | x | 12-Dec-12 |
| 12-3 | Pharmacy | 5-2-3-12-12-03 | x | 08-Dec-12 |
| 6-3 | Pharmacy | 7-2-4-6-06-03 | x | 11-Dec-12 |
| 36-3 | PMV | 7-2-4-36-36-3 | x | 13-Dec-12 |
| 2-3 | PMV | 9-2-2-2-02-03 | x | 18-Dec-12 |
| 32-4 | PMV | 14-1-3-32-32-04 | x | 14-Dec-12 |
| 4-3 | Pharmacy | 15-2-1-4-04-03 | x | 12-Dec-12 |
| 14-3 | PMV | 15-2-1-14-14-03 | x | 17-Dec-12 |
| 10-3 | Pharmacy | 15-2-3-10-10-3 | x | no date |
| 4-5 | Pharmacy | 1-3-1-4-04-05 | x | 10-Dec-12 |
| 33-5 | PMV | 2-3-5-33-33-05 | x | 12-Dec-12 |
| 1-5 | Pharmacy | 4-1-2-1-01-05 | x | 10-Dec-12 |
| 8-5 | Pharmacy | 5-1-2-8-08-05 | x | 11-Dec-12 |
| 11-5 | Pharmacy | 6-2-1-11-11-05 | x | 18-Dec-12 |
| 36-05C | PMV | 6-2-3-36-36-05C | x | 08-Dec-12 |
| 6-5 | Pharmacy | 7-2-4-6-06-05 | x | 11-Dec-12 |
| 27-5 | PMV | 7-2-4-27-27-05 | x | 12-Dec-12 |
| 22-5 | PMV | 11-2-5-22-22-5 | x | 10-Dec-12 |
| 9-5 | Pharmacy | 13-2-5-9-09-05 | x | 10-Dec-12 |
| 38-5 | Pharmacy | 15-2-3-38-38-05 | x | 13-Dec-12 |
| 2-67 | Pharmacy | 5-1-2-2-02-67 | x | 10-Dec-12 |
| 75-11 | PMV | 1-3-1-75-75-11 | x | 15-Dec-12 |
| 33-67A | PMV | 2-3-5-33-33-67A | x | 12-Dec-12 |
| 3-11 | Pharmacy | 4-2-3-3-03-11 | x | 08-Dec-12 |
| 8-11 | Pharmacy | 5-1-2-8-08-11 | x | 11-Dec-12 |
| 10-11 | Pharmacy | 5-1-2-10-10-11 | x | 14-Dec-12 |

| | | | | |
|---|---|---|---|---|
| 59-11 | Pharmacy | 5-1-2-59-59-11 | x | 13-Dec-12 |
| 11-11 | PMV | 9-2-2-11-11-11 | x | 18-Dec-12 |
| 8-11 | Pharmacy | 10-3-1-8-08-11 | x | 18-Dec-12 |
| 1-67 | Pharmacy | 11-2-5-1-01-67 | x | 13-Dec-12 |
| 2-11 | Pharmacy | 11-2-5-2-02-11 | x | 12-Dec-12 |
| 2-11 | Pharmacy | 16-1-3-2-02-11 | x | 11-Dec-12 |
| 26-11 | Pharmacy | 16-1-3-26-26-11 | x | 12-Dec-12 |
| 74-67b | PMV | 1-3-1-74-74-67b | x | 14-Dec-12 |
| 2-18 | Pharmacy | 3-2-1-2-02-18 | x | 10-Dec-12 |
| 3-18 | Pharmacy | 4-2-3-3-03-18 | x | 08-Dec-12 |
| 1-18 | Pharmacy | 6-2-1-1-01-18 | x | 14-Dec-12 |
| 11-18 | Pharmacy | 6-2-1-11-11-18 | x | 18-Dec-12 |
| 6-18 | Pharmacy | 6-2-3-6-06-18 | x | 11-Dec-12 |
| 6-18 | Pharmacy | 7-2-4-6-06-18 | x | 11-Dec-12 |
| 37-18 | PMV | 10-3-1-37-37-18 | x | 11-Dec-12 |
| 10-18 | Pharmacy | 12-2-5-10-10-18 | x | 12-Dec-12 |
| 16-18 | PMV | 14-1-1-16-16-18 | x | 13-Dec-12 |
| 4-18 | Pharmacy | 14-1-3-4-4-18 | x | 10-Dec-12 |
| 38-18 | Pharmacy | 15-2-3-38-38-18 | x | 13-Dec-12 |
| 4-23 | Pharmacy | 4-1-2-4-04-23 | x | 12-Dec-12 |
| 3-23 | Pharmacy | 4-2-3-3-03-23 | x | 08-Dec-12 |
| 31-23 | PMV | 5-1-2-31-31-23 | x | 12-Dec-12 |
| 6-23 | Pharmacy | 11-2-5-6-06-23 | x | 21-Dec-12 |
| 4-23 | Pharmacy | 14-1-3-4-4-23 | x | 10-Dec-12 |
| 12-25 | Pharmacy | 1-3-1-12-12-25 | x | 13-Dec-12 |
| 42-25 | PMV | 1-3-1-42-42-25 | x | 13-Dec-12 |
| 77-25 | PMV | 1-3-1-77-77-25 | x | 18-Dec-12 |
| 83-25 | PMV | 1-3-1-83-83-25 | x | 18-Dec-12 |
| 1-25 | Pharmacy | 2-3-6-1-01-25 | x | 10-Dec-12 |
| 2-25 | PMV | 3-2-1-2-02-25 | x | 10-Dec-12 |
| 2-25 | Pharmacy | 3-2-4-2-02-25 | x | 10-Dec-12 |
| 6-25A | PMV | 3-2-6-6-06-25A | x | 19-Dec-12 |
| 9-25 | Pharmacy | 4-1-2-9-09-25 | x | 11-Dec-12 |
| 57-25 | PMV | 5-1-2-57-57-25 | x | 14-Dec-12 |
| 7-25 | Pharmacy | 5-2-3-7-07-25 | x | 08-Dec-12 |
| 1-25 | Pharmacy | 6-2-1-1-01-25 | x | 14-Dec-12 |
| 11-25 | Pharmacy | 6-2-1-11-11-25 | x | 18-Dec-12 |
| 28-25 | Pharmacy | 6-2-1-28-28-25 | x | 14-Dec-12 |
| 9-25 | Pharmacy | 6-2-3-9-09-25 | x | 11-Dec-12 |
| 25-25 | PMV | 6-2-3-25-25-25 | x | 08-Dec-12 |
| 31-25 | PMV | 6-2-3-31-31-25 | x | 10-Dec-12 |
| 37-25 | Pharmacy | 6-2-3-37-37-25 | x | 12-Dec-12 |
| 6-25 | Pharmacy | 7-2-4-6-06-25 | x | 11-Dec-12 |
| 8-25 | Pharmacy | 8-2-1-8-08-25 | x | 10-Dec-12 |
| 10-25 | Pharmacy | 8-2-4-10-10-25 | x | 12-Dec-12 |
| 1-25 | Pharmacy | 9-2-2-1-01-25 | x | 18-Dec-12 |
| 8-25 | Pharmacy | 10-3-1-8-08-25 | x | 18-Dec-12 |
| 33-25B | PMV | 10-3-1-33-33-25B | x | 15-Dec-12 |
| 71-25 | PMV | 10-3-1-71-71-25 | x | 15-Dec-12 |
| 1-25 | Pharmacy | 11-2-5-1-01-25 | x | 13-Dec-12 |

| | | | | |
|---|---|---|---|---|
| 11-25 | Pharmacy | 11-2-5-11-11-25 | x | 12-Dec-12 |
| 22-25 | PMV | 11-2-5-22-22-25 | x | 10-Dec-12 |
| 26-26 | PMV | 11-2-5-26-26-26 | x | 10-Dec-12 |
| 16-25 | Pharmacy | 13-2-3-16-16-25 | x | 08-Dec-12 |
| 33-25 | PMV | 13-3-7-33-33-25 | x | 14-Dec-12 |
| 1-25 | Pharmacy | 14-1-3-1-01-25 | x | 11-Dec-12 |
| 5-25 | Pharmacy | 14-1-3-5-05-25 | x | 11-Dec-12 |
| 35-23 | PMV | 14-1-3-35-35-23 | x | 14-Dec-12 |
| 7-25 | Pharmacy | 15-2-1-7-07-25 | x | 14-Dec-12 |
| 10-25 | Pharmacy | 15-2-3-10-10-25 | x | 11-Dec-12 |
| 11-25 | Pharmacy | 15-2-3-11-11-25 | x | 12-Dec-12 |
| 24-25 | PMV | 15-2-3-24-24-25 | x | 11-Dec-12 |
| 38-25 | Pharmacy | 15-2-3-38-38-25 | x | 13-Dec-12 |
| 38-25 | PMV | 16-1-3-38-38-25 | x | 14-Dec-12 |
| 21-27 | PMV | 3-2-4-21-21-27 | x | 10-Dec-12 |
| 4-27 | Pharmacy | 4-1-2-4-04-27 | x | 12-Dec-12 |
| 12-27 | Pharmacy | 4-1-2-12-12-27 | x | 14-Dec-12 |
| 13-26A | PMV | 6-2-1-13-13-26A | x | 13-Dec-12 |
| 5-27 | Pharmacy | 8-2-4-5-05-27 | x | 11-Dec-12 |
| 31-26A | PMV | 9-3-5-31-31-26A | x | 12-Dec-12 |
| 67-26 | PMV | 10-3-1-67-67-26 | x | 14-Dec-12 |
| 1-27B | Pharmacy | 10-3-1-1-01-27B | x | 14-Dec-12 |
| 1-26 | Pharmacy | 11-2-5-1-01-26 | x | 13-Dec-12 |
| 15-26 | PMV | 14-1-1-15-15-26 | x | 13-Dec-12 |
| 24-27 | PMV | 15-2-3-24-24-27 | x | 11-Dec-12 |
| 38-67B | PMV | 15-2-3-38-38-67B | x | 13-Dec-12 |
| 36-25 | PMV | 1-3-1-36-36-25 | x | 15-Dec-12 |
| 4-26 | Pharmacy | 1-3-1-4-04-26 | x | 10-Dec-12 |
| 57-26 | PMV | 1-3-1-57-57-26 | x | 15-Dec-12 |
| 59-26 | PMV | 1-3-1-59-59-26 | x | 11-Dec-12 |
| 60-26 | PMV | 1-3-1-60-60-26 | x | 13-Dec-12 |
| 69-26 | PMV | 1-3-1-69-69-26 | x | 14-Dec-12 |
| 81-26 | PMV | 1-3-1-81-81-26 | x | 18-Dec-12 |
| 3-26 | PMV | 2-3-4-3-3-26 | x | 10-Dec-12 |
| 25-26 | PMV | 2-3-5-25-25-26 | x | 11-Dec-12 |
| 15-26 | PMV | 3-2-4-15-15-26 | x | 11-Dec-12 |
| 18-26 | PMV | 3-2-4-18-18-26 | x | 13-Dec-12 |
| 2-26 | PMV | 3-2-4-2-02-26 | x | 10-Dec-12 |
| 7-26 | Pharmacy | 3-2-4-7-07-26 | x | 11-Dec-12 |
| 6-25B | PMV | 3-2-6-6-06-25B | x | 19-Dec-12 |
| 0-26 | Publichealth fa | 3-3-7-0-0-26 | x | 20-Dec-12 |
| 23-26 | Pharmacy | 4-1-2-23-23-26 | x | 10-Dec-12 |
| 35-26 | PMV | 4-1-2-35-35-26 | x | 13-Dec-12 |
| 36-26 | PMV | 4-1-2-36-36-26 | x | 12-Dec-12 |
| 53-26 | PMV | 4-1-2-53-53-26 | x | 18-Dec-12 |
| 5-26 | Pharmacy | 4-1-2-5-5-26 | x | 13-Dec-12 |
| 33-26 | PMV | 5-1-2-33-33-26 | x | 12-Dec-12 |
| 37-25 | PMV | 5-1-2-37-37-25 | x | 13-Dec-12 |
| 55-26 | PMV | 5-1-2-55-55-26 | x | 13-Dec-12 |
| 57-26 | PMV | 5-1-2-57-57-26 | x | 13-Dec-12 |

| | | | | | |
|---|---|---|---|---|---|
| 59-7 | PMV | 5-1-2-59-59-07 | x | | 20-Dec-12 |
| 13-26B | PMV | 6-2-1-13-13-26B | x | | 13-Dec-12 |
| 15-26 | PMV | 6-2-1-15-15-26 | x | | 17-Dec-12 |
| 26-26 | PMV | 6-2-1-26-26-26 | x | | 14-Dec-12 |
| 1-26 | Pharmacy | 6-2-3-1-01-26 | x | | 12-Dec-12 |
| 39-26 | PMV | 6-2-3-39-39-26 | x | | 13-Dec-12 |
| 1-26A | Pharmacy | 7-2-4-1-01-26A | x | | 13-Dec-12 |
| 1-26B | Pharmacy | 7-2-4-1-01-26B | x | | 13-Dec-12 |
| 22-26 | PMV | 7-2-4-22-22-26 | x | | 10-Dec-12 |
| 16-26 | PMV | 8-2-4-16-16-26 | x | | 13-Dec-12 |
| 23-26 | PMV | 8-2-4-23-23-26 | x | | 10-Dec-12 |
| 34-26 | PMV | 8-2-4-34-34-26 | x | | 11-Dec-12 |
| 5-26 | Pharmacy | 8-2-4-5-05-26 | x | | 11-Dec-12 |
| 11-25 | PMV | 9-2-2-11-11-25 | x | | 18-Dec-12 |
| 5-26 | PMV | 2-3-2-5-05-26 | x | | 10-Dec-12 |
| 20-25 | PMV | 9-3-5-20-20-25 | x | | 11-Dec-12 |
| 31-25 | PMV | 9-3-5-31-31-25 | x | | 12-Dec-12 |
| 8-25 | PMV | 9-3-6-8-08-25 | x | | 13-Dec-12 |
| 21-26 | PMV | 1-3-1-21-21-26 | x | | 13-Dec-12 |
| 1-26 | Pharmacy | 10-3-1-1-01-26 | x | | 14-Dec-12 |
| 8-26 | Pharmacy | 10-3-1-8-8-26 | x | | 18-Dec-12 |
| 21-62 | PMV | 11-2-5-21-21-62 | x | | 10-Dec-12 |
| 10-26 | Pharmacy | 12-2-5-10-10-26 | x | | 12-Dec-12 |
| 21-26 | PMV | 13-2-3-21-21-26 | x | | 08-Dec-12 |
| 25-26 | PMV | 13-2-5-25-25-26 | x | | 10-Dec-12 |
| 9-26 | Pharmacy | 13-2-5-9-09-26 | x | | 10-Dec-12 |
| 28-26 | PMV | 13-3-7-28-28-26 | x | | 13-Dec-12 |
| 32-26 | PMV | 13-3-7-32-32-26 | x | | 14-Dec-12 |
| 20-26 | PMV | 14-1-1-20-20-26 | x | | 20-Dec-12 |
| 23-26 | PMV | 14-1-3-23-23-26 | x | | 10-Dec-12 |
| 4-25 | Pharmacy | 14-1-3-4-4-25 | x | | 12-Dec-12 |
| 5-26 | Pharmacy | 14-1-3-5-05-26 | x | | 11-Dec-12 |
| 4-26 | Pharmacy | 15-2-1-4-04-26 | x | | 17-Dec-12 |
| 7-26 | Pharmacy | 15-2-1-7-07-26 | x | | 14-Dec-12 |
| 10-26 | PMV | 15-2-3-10-10-26 | x | | 11-Dec-12 |
| 26-26 | PMV | 15-2-3-26-26-26 | x | | 08-Dec-12 |
| 28-26 | PMV | 16-1-1-28-28-26 | x | | 14-Dec-12 |
| 11-26 | Pharmacy | 16-1-3-11-11-26 | x | | 10-Dec-12 |
| 6-26 | Pharmacy | 16-1-3-6-06-026 | x | | 12-Dec-12 |
| 7-26 | Pharmacy | 16-1-3-7-07-26 | x | | 11-Dec-12 |
| 27-26 | PMV | 5-1-2-27-27-26 | x | old packaging | 14-Dec-12 |
| 21-27A | PMV | 1-3-1-21-21-27A | x | | 13-Dec-12 |
| 60-27B | PMV | 1-3-1-60-60-27B | x | | 11-Dec-12 |
| 75-26 | PMV | 1-3-1-75-75-26 | x | | 15-Dec-12 |
| 5-67 | PMV | 2-3-2-5-5-67 | x | | 10-Dec-12 |
| 6-27A | PMV | 2-3-2-6-06-27A | x | | 10-Dec-12 |
| 34-27a | PMV | 2-3-5-34-34-27a | x | | 12-Dec-12 |
| 2-27 | Pharmacy | 3-2-4-2-02-27 | x | | 10-Dec-12 |
| 0-27a | Publichealth fa | 3-3-7-0-0-27a | x | | 20-Dec-12 |
| 21-27 | PMV | 5-1-2-21-21-27 | x | | 14-Dec-12 |

| | | | | |
|---|---|---|---|---|
| 22-27 | PMV | 5-1-2-22-22-27 | x | 04-Dec-12 |
| 33-27 | PMV | 5-1-2-33-33-27 | x | 12-Dec-12 |
| 49-27 | PMV | 5-1-2-49-49-27 | x | 14-Dec-12 |
| 12-27 | Pharmacy | 5-2-3-12-12-27 | x | 08-Dec-12 |
| 13-27 | PMV | 6-2-1-13-13-27 | x | 13-Dec-12 |
| 37-67C | Pharmacy | 6-2-3-37-37-67C | x | 12-Dec-12 |
| 16-27 | PMV | 8-2-4-16-16-27 | x | 13-Dec-12 |
| 23-27 | PMV | 8-2-4-23-23-27 | x | 13-Dec-12 |
| 20-27 | PMV | 9-3-5-20-20-27 | x | 11-Dec-12 |
| 31-27 | PMV | 9-3-5-31-31-27 | x | 12-Dec-12 |
| 8-27 | PMV | 9-3-6-8-08-27 | x | 13-Dec-12 |
| 1-27A | Pharmacy | 10-3-1-1-01-27A | x | 14-Dec-12 |
| 20-17A | PMV | 10-3-4-20-20-17A | x | 13-Dec-12 |
| 6-77 | Pharmacy | 12-3-7-6-06-77 | x | 11-Dec-12 |
| 21-27 | PMV | 13-2-3-21-21-27 | x | 08-Dec-12 |
| 28-25 | PMV | 13-3-7-28-28-25 | x | 13-Dec-12 |
| 8-27 | Pharmacy | 13-3-7-8-08-27 | x | 11-Dec-12 |
| 9-27 | Pharmacy | 13-3-7-9-09-27 | x | 11-Dec-12 |
| 1-25 | Pharmacy | 14-1-1-1-01-25 | x | 13-Dec-12 |
| 7-25 | Pharmacy | 14-1-1-7-07-25 | x | 14-Dec-12 |
| 17-67A | PMV | 15-2-1-17-17-67A | x | 14-Dec-12 |
| 5-67b | Pharmacy | 15-2-1-5-05-67b | x | 14-Dec-12 |
| 26-27 | PMV | 15-2-3-26-26-27 | x | 08-Dec-12 |
| 38-67a | PMV | 15-2-3-38-38-67a | x | 13-Dec-12 |
| 17-27 | PMV | 16-1-1-17-17-27 | x | 14-Dec-12 |
| 2-26 | Pharmacy | 16-1-3-2-02-26 | x | 11-Dec-12 |
| 31-27 | PMV | 16-1-3-31-31-27 | x | 18-Dec-12 |
| 12-27 | Pharmacy | 1-3-1-12-12-27 | x | 13-Dec-12 |
| 21-27B | PMV | 1-3-1-21-21-27B | x | 13-Dec-12 |
| 42-27 | PMV | 1-3-1-42-42-27 | x | 13-Dec-12 |
| 59-67b | PMV | 1-3-1-59-59-67b | x | 11-Dec-12 |
| 60-27A | PMV | 1-3-1-60-60-27A | x | 11-Dec-12 |
| 5-27 | PMV | 2-3-2-5-05-27 | x | 10-Dec-12 |
| 6-27B | PMV | 2-3-2-6-06-27B | x | 10-Dec-12 |
| 34-27b | PMV | 2-3-5-34-34-27b | x | 12-Dec-12 |
| 2-27 | Pharmacy | 3-2-1-2-02-27 | x | 10-Dec-12 |
| 6-27 | PMV | 3-2-6-6-06-27 | x | 19-Dec-12 |
| 0-27b | Publichealth fa | 3-3-7-0-00-27b | x | 20-Dec-12 |
| 23-27 | PMV | 4-1-2-23-23-27 | x | 10-Dec-12 |
| 2-25 | Pharmacy | 5-1-2-2-02-25 | x | 10-Dec-12 |
| 8-27 | Pharmacy | 5-1-2-8-08-27 | x | 11-Dec-12 |
| 10-27 | Pharmacy | 5-1-2-10-10-27 | x | 14-Dec-12 |
| 22-27 | PMV | 7-2-4-22-22-27 | x | 13-Dec-12 |
| 34-27 | PMV | 8-2-4-34-34-27 | x | 11-Dec-12 |
| 6-27 | PMV | 9-2-2-6-06-27 | x | 18-Dec-12 |
| 31-26B | PMV | 9-3-5-31-31-26B | x | 12-Dec-12 |
| 8-26 | PMV | 9-3-6-8-08-26 | x | 13-Dec-12 |
| 14-26 | Pharmacy | 10-3-1-14-14-26 | x | 18-Dec-12 |
| 15-27 | Pharmacy | 10-3-1-15-15-27 | x | 10-Dec-12 |
| 33-27 | PMV | 10-3-1-33-33-27 | x | 15-Dec-12 |

| | | | | |
|---|---|---|---|---|
| 53-26 | PMV | 10-3-1-53-53-26 | x | 18-Dec-12 |
| 67-27 | PMV | 10-3-1-67-67-27 | x | 14-Dec-12 |
| 2-27 | PMV | 11-2-5-2-02-27 | x | 12-Dec-12 |
| 24-27 | PMV | 12-2-5-24-24-27 | x | 10-Dec-12 |
| 21-25 | PMV | 13-2-3-21-21-25 | x | 08-Dec-12 |
| 18-25 | PMV | 13-2-5-18-18-25 | x | 10-Dec-12 |
| 21-26 | PMV | 14-1-3-21-21-26 | x | 12-Dec-12 |
| 27-27 | PMV | 16-1-3-27-27-27 | x | 12-Dec-12 |
| 30-27 | PMV | 16-1-3-30-30-27 | x | 18-Dec-12 |
| 8-67C | Pharmacy | 5-1-2-8-08-67C | x | 11-Dec-12 |
| 5-67A | Pharmacy | 14-1-3-5-05-67A | x | 11-Dec-12 |
| 7-67a | Pharmacy | 15-2-1-7-07-67a | x | 14-Dec-12 |
| 8-67D | Pharmacy | 5-1-2-8-08-67D | x | 11-Dec-12 |
| 7-67 | Pharmacy | 5-2-3-7-07-67 | x | 08-Dec-12 |
| 1-67a | Pharmacy | 6-2-1-1-01-67a | x | 14-Dec-12 |
| 1-7 | Pharmacy | 7-2-4-1-01-07 | x | 14-Dec-12 |
| 10-67A | Pharmacy | 8-2-4-10-10-67A | x | 12-Dec-12 |
| 1-67A | Pharmacy | 11-2-5-1-01-67A | x | 13-Dec-12 |
| 6-67B | Pharmacy | 11-2-5-6-06-67B | x | 21-Dec-12 |
| 10-67A | Pharmacy | 12-2-5-10-10-67A | x | 12-Dec-12 |
| 29-67b | PMV | 15-2-1-29-29-67b | x | 14-Dec-12 |
| 38-67A | PMV | 15-2-3-38-38-67A | x | 13-Dec-12 |
| 33-31 | PMV | 5-1-2-33-33-31 | x | 12-Dec-12 |
| 0-31 | Publichealth fa | 3-1-2-0-00-31 | x | 21-Dec-12 |
| 30-31 | PMV | 5-1-2-30-30-31 | x | 10-Dec-12 |
| 14-31 | PMV | 6-2-3-14-14-31 | x | 10-Dec-12 |
| 7-31 | Pharmacy | 3-2-4-7-07-31 | x | 11-Dec-12 |
| 18-31 | PMV | 10-3-1-18-18-31 | x | 14-Dec-12 |
| 21-31 | PMV | 10-3-1-21-21-31 | x | 13-Dec-12 |
| 7-20 | Pharmacy | 16-1-1-7-07-20 | x | 14-Dec-12 |
| 57-31 | PMV | 1-3-1-57-57-31 | x | 15-Dec-12 |
| 2-31 | Pharmacy | 3-2-1-2-02-31 | x | 10-Dec-12 |
| 12-31 | Pharmacy | 4-1-2-12-12-31 | x | 14-Dec-12 |
| 8-31 | Pharmacy | 5-1-2-8-08-31 | x | 11-Dec-12 |
| 6-31 | Pharmacy | 12-3-7-6-06-31 | x | 11-Dec-12 |
| 25-31 | PMV | 13-2-5-25-25-31 | x | 10-Dec-12 |
| 28-31 | PMV | 13-3-7-28-28-31 | x | 13-Dec-12 |
| 16-31 | PMV | 14-1-1-16-16-31 | x | 13-Dec-12 |
| 27-31A | PMV | 16-1-3-27-27-31A | x | 12-Dec-12 |
| 24-67a | PMV | 11-3-7-24-24-67a | x | 11-Dec-12 |
| 28-67 | PMV | 12-2-5-28-28-67 | x | 10-Dec-12 |
| 22-67b | PMV | 5-1-2-22-22-67b | x | 04-Dec-12 |
| 22-67 | PMV | 7-2-4-22-22-67 | x | 13-Dec-12 |
| 0-48 | Publichealth fa | 9-2-1-0-0-48 | x | 21-Dec-12 |
| 6-67A | PMV | 6-2-3-6-06-67A | x | 11-Dec-12 |
| 5-52a | Pharmacy | 2-3-2-5-5-52a | x | 10-Dec-12 |
| 7-52 | Pharmacy | 3-2-4-7-07-52 | x | 11-Dec-12 |
| 21-52 | PMV | 3-2-4-21-21-52 | x | 10-Dec-12 |
| 19-52 | Pharmacy | 4-1-2-19-19-52 | x | 14-Dec-12 |
| 38-52a | PMV | 4-1-2-38-38-52a | x | 14-Dec-12 |

| | | | | |
|---|---|---|---|---|
| 8-52 | Pharmacy | 5-1-2-8-08-52 | x | 11-Dec-12 |
| 10-52 | Pharmacy | 5-1-2-10-10-52 | x | 14-Dec-12 |
| 21-67 | PMV | 5-1-2-21-21-67 | x | 14-Dec-12 |
| 22-52 | PMV | 5-1-2-22-22-52 | x | 04-Dec-12 |
| 12-67e | PMV | 5-2-3-12-12-67e | x | 08-Dec-12 |
| 20-52 | PMV | 7-2-4-20-20-52 | x | 10-Dec-12 |
| 36-52 | PMV | 7-2-4-36-36-52 | x | 13-Dec-12 |
| 8-67B | Pharmacy | 8-2-1-8-08-67B | x | 10-Dec-12 |
| 6-52 | Pharmacy | 9-2-2-6-06-52 | x | 18-Dec-12 |
| 21-52a | PMV | 11-2-5-21-21-52a | x | 10-Dec-12 |
| 24-52 | PMV | 11-3-7-24-24-52 | x | 11-Dec-12 |
| 25-52 | PMV | 14-1-3-25-25-52 | x | 14-Dec-12 |
| 11-46 | Pharmacy | 15-2-3-11-11-46 | x | 12-Dec-12 |
| 26-67A | PMV | 15-2-3-26-26-67A | x | 08-Dec-12 |
| 4-52A | Pharmacy | 1-3-1-4-04-52A | x | 10-Dec-12 |
| 39-52 | PMV | 1-3-1-39-39-52 | x | 14-Dec-12 |
| 5-52b | PMV | 2-3-2-5-5-52b | x | 10-Dec-12 |
| 8-52 | Pharmacy | 4-1-2-8-08-52 | x | 13-Dec-12 |
| 38-52b | PMV | 4-1-2-38-38-52b | x | 14-Dec-12 |
| 3-52 | Pharmacy | 4-2-3-3-03-52 | x | 08-Dec-12 |
| 49-52 | PMV | 5-1-2-49-49-52 | x | 14-Dec-12 |
| 14-67A | Pharmacy | 6-2-3-14-14-67A | x | 10-Dec-12 |
| 1-52 | Pharmacy | 7-2-4-1-01-52 | x | 13-Dec-12 |
| 22-52 | PMV | 7-2-4-22-22-52 | x | 13-Dec-12 |
| 10-52 | Pharmacy | 8-2-4-10-10-52 | x | 12-Dec-12 |
| 17-52 | PMV | 8-2-4-17-17-52 | x | 13-Dec-12 |
| 10-52a | PMV | 9-2-2-10-10-52a | x | 18-Dec-12 |
| 5-517 | Pharmacy | 2-3-2-5-5-517 | x | 10-Dec-12 |
| 19-50A | PMV | 9-3-5-19-19-50A | x | 11-Dec-12 |
| 14-52a | Pharmacy | 10-3-1-14-14-52a | x | 18-Dec-12 |
| 25-52A | PMV | 13-2-5-25-25-52A | x | 10-Dec-12 |
| 25-52B | PMV | 13-2-5-25-25-52B | x | 10-Dec-12 |
| 4-52 | Pharmacy | 13-3-7-4-04-52 | x | 11-Dec-12 |
| 32-52 | PMV | 13-3-7-32-32-52 | x | 14-Dec-12 |
| 7-52 | PMV | 14-1-1-7-07-52 | x | 14-Dec-12 |
| 1-52 | Pharmacy | 14-1-3-1-01-52 | x | 11-Dec-12 |
| 14-67B | PMV | 15-2-1-14-14-67B | x | 17-Dec-12 |
| 36-52A | PMV | 6-2-3-36-36-52A | x | 08-Dec-12 |
| 66-52A | PMV | 10-3-1-66-66-52A | x | 14-Dec-12 |
| 7-67b | Pharmacy | 15-2-1-7-07-67b | x | 14-Dec-12 |
| 4-52 | Pharmacy | 1-3-1-4-04-52 | x | 10-Dec-12 |
| 15-52 | Pharmacy | 1-3-1-12-15-52 | x | 13-Dec-12 |
| 13-52 | Pharmacy | 1-3-1-13-13-52 | x | 14-Dec-12 |
| 21-52 | PMV | 1-3-1-21-21-52 | x | 13-Dec-12 |
| 42-52 | PMV | 1-3-1-42-42-52 | x | 13-Dec-12 |
| 57-52 | PMV | 1-3-1-57-57-52 | x | 15-Dec-12 |
| 60-52 | PMV | 1-3-1-60-60-52 | x | 11-Dec-12 |
| 74-52 | PMV | 1-3-1-74-74-52 | x | 14-Dec-12 |
| 77-52 | PMV | 1-3-1-77-77-52 | x | 18-Dec-12 |
| 80-52 | PMV | 1-3-1-80-80-52 | x | 18-Dec-12 |

| | | | | |
|---|---|---|---|---|
| 1-52 | PMV | 1-3-1-81-01-52 | x | 18-Dec-12 |
| 28-52 | PMV | 2-3-5-28-28-52 | x | 11-Dec-12 |
| 29-52 | PMV | 2-3-5-29-29-52 | x | 11-Dec-12 |
| 33-52 | PMV | 2-3-5-33-33-52 | x | 12-Dec-12 |
| 34-52 | PMV | 2-3-5-34-34-52 | x | 12-Dec-12 |
| 2-52 | PMV | 2-3-6-2-02-52 | x | 10-Dec-12 |
| 2-52 | Pharmacy | 3-2-1-2-02-52 | x | 10-Dec-12 |
| 14-52 | PMV | 3-2-4-14-14-52 | x | 12-Dec-12 |
| 43-52 | PMV | 3-2-4-43-43-52 | x | 13-Dec-12 |
| 6-52 | Pharmacy | 3-2-6-6-06-52 | x | 19-Dec-12 |
| 9-52 | Pharmacy | 4-1-2-9-09-52 | x | 11-Dec-12 |
| 15-52 | Pharmacy | 4-1-2-15-15-52 | x | 12-Dec-12 |
| 18-52 | PMV | 4-1-2-18-18-52 | x | 14-Dec-12 |
| 23-52 | Pharmacy | 4-1-2-23-23-52 | x | 10-Dec-12 |
| 35-52 | PMV | 4-1-2-35-35-52 | x | 13-Dec-12 |
| 38-52c | PMV | 4-1-2-38-38-52c | x | 14-Dec-12 |
| 21-0 | Pharmacy | 5-1-2-21-21-00 | x | 14-Dec-12 |
| 30-52 | PMV | 5-1-2-30-30-52 | x | 10-Dec-12 |
| 55-52 | PMV | 5-1-2-55-55-52 | x | 13-Dec-12 |
| 57-51b | Pharmacy | 5-1-2-57-57-51b | x | 14-Dec-12 |
| 15-52 | PMV | 6-2-1-15-15-52 | x | 17-Dec-12 |
| 14-52 | PMV | 6-2-3-14-14-52 | x | 10-Dec-12 |
| 36-52B | PMV | 6-2-3-36-36-52B | x | 08-Dec-12 |
| 39-52 | PMV | 6-2-3-39-39-52 | x | 13-Dec-12 |
| 25-52 | PMV | 7-2-4-25-25-52 | x | 13-Dec-12 |
| 8-52 | Pharmacy | 8-2-1-8-08-52 | x | 10-Dec-12 |
| 5-52 | Pharmacy | 8-2-4-5-05-52 | x | 11-Dec-12 |
| 16-52 | PMV | 8-2-4-16-16-52 | x | 13-Dec-12 |
| 44-52 | PMV | 8-2-4-44-44-52 | x | 12-Dec-12 |
| 10-52b | PMV | 9-2-2-10-10-52b | x | 18-Dec-12 |
| 11-52 | PMV | 9-2-2-11-11-52 | x | 18-Dec-12 |
| 8-52 | PMV | 9-3-5-8-8-52 | x | 11-Dec-12 |
| 16-52 | PMV | 9-3-5-16-16-52 | x | 12-Dec-12 |
| 19-52 | PMV | 9-3-5-19-19-52 | x | 11-Dec-12 |
| 5-52 | Pharmacy | 10-3-1-5-05-52 | x | 13-Dec-12 |
| 14-52b | Pharmacy | 10-3-1-14-14-52b | x | 18-Dec-12 |
| 24-52 | PMV | 10-3-1-24-24-52 | x | 15-Dec-12 |
| 33-52 | PMV | 10-3-1-33-33-52 | x | 15-Dec-12 |
| 37-52 | PMV | 10-3-1-37-37-52 | x | 11-Dec-12 |
| 66-52B | PMV | 10-3-1-66-66-52B | x | 14-Dec-12 |
| 67-52 | PMV | 10-3-1-67-67-52 | x | 14-Dec-12 |
| 78-52 | PMV | 10-3-1-78-78-52 | x | 13-Dec-12 |
| 5-52 | Pharmacy | 11-2-5-5-05-52 | x | 12-Dec-12 |
| 11-52 | Pharmacy | 11-2-5-11-11-52 | x | 12-Dec-12 |
| 21-52b | PMV | 11-2-5-21-21-52b | x | 10-Dec-12 |
| 26-52 | PMV | 11-2-5-26-26-52 | x | 10-Dec-12 |
| 14-52 | PMV | 11-3-7-14-14-52 | x | 11-Dec-12 |
| 15-52 | PMV | 11-3-7-15-15-52 | x | 11-Dec-12 |
| 3-52 | Pharmacy | 12-2-5-3-03-52 | x | 12-Dec-12 |
| 12-52 | PMV | 12-3-7-12-12-52 | x | 11-Dec-12 |

| | | | | |
|---|---|---|---|---|
| 21-52 | PMV | 13-2-3-21-21-52 | x | 08-Dec-12 |
| 25-52 | PMV | 13-2-5-25-25-52 | x | 10-Dec-12 |
| 28-52 | PMV | 13-3-7-28-28-52 | x | 13-Dec-12 |
| 18-52 | PMV | 14-1-1-18-18-52 | x | 14-Dec-12 |
| 4-52 | Pharmacy | 14-1-3-4-4-52 | x | 10-Dec-12 |
| 5-52 | Pharmacy | 14-1-3-5-05-52 | x | 11-Dec-12 |
| 5-52 | Pharmacy | 15-2-1-5-05-52 | x | 14-Dec-12 |
| 7-52 | Pharmacy | 15-2-1-7-07-52 | x | 14-Dec-12 |
| 14-52 | PMV | 15-2-1-14-14-52 | x | 17-Dec-12 |
| 19-52 | PMV | 15-2-1-19-19-52 | x | 18-Dec-12 |
| 10-52 | Pharmacy | 15-2-3-10-10-52 | x | 11-Dec-12 |
| 20-52 | PMV | 15-2-3-20-20-52 | x | 12-Dec-12 |
| 26-52 | PMV | 15-2-3-26-26-52 | x | 08-Dec-12 |
| 30-52 | PMV | 15-2-3-30-30-52 | x | 10-Dec-12 |
| 7-52 | Pharmacy | 16-1-1-7-07-52 | x | 14-Dec-12 |
| 28-52 | PMV | 16-1-1-28-28-52 | x | 14-Dec-12 |
| 14-52 | Pharmacy | 16-1-3-4-014-052 | x | 12-Dec-12 |
| 7-52 | Pharmacy | 16-1-3-7-07-52 | x | 11-Dec-12 |
| 27-52 | PMV | 16-1-3-27-27-52 | x | 12-Dec-12 |
| 1-57 | Pharmacy | 2-3-6-1-01-57 | x | 10-Dec-12 |
| 1-67 | Pharmacy | 2-3-6-1-01-67 | x | 10-Dec-12 |
| 9-67B | Pharmacy | 4-1-2-9-09-67B | x | 11-Dec-12 |
| 1-67E | Pharmacy | 11-2-5-1-01-67E | x | 13-Dec-12 |
| 5-67B | Pharmacy | 14-1-3-5-05-67B | x | 11-Dec-12 |
| 1-67F | Pharmacy | 11-2-5-1-01-67F | x | 13-Dec-12 |
| 1-67G | Pharmacy | 11-2-5-1-01-67G | x | 13-Dec-12 |
| 21-59 | PMV | 1-3-1-21-21-59 | x | 13-Dec-12 |
| 59-59 | PMV | 1-3-1-59-59-59 | x | 11-Dec-12 |
| 1-59 | Pharmacy | 4-1-2-1-01-59 | x | 10-Dec-12 |
| 4-59 | Pharmacy | 4-1-2-4-04-59 | x | 12-Dec-12 |
| 9-59 | Pharmacy | 4-1-2-9-9-59 | x | 11-Dec-12 |
| 36-59 | PMV | 4-1-2-36-36-59 | x | 12-Dec-12 |
| 7-59 | Pharmacy | 5-1-2-7-07-59 | x | 12-Dec-12 |
| 8-59 | Pharmacy | 5-1-2-8-08-59 | x | 11-Dec-12 |
| 17-59 | Pharmacy | 5-1-2-17-17-59 | x | 18-Dec-12 |
| 12-59 | Pharmacy | 5-2-3-12-12-59 | x | 08-Dec-12 |
| 6-59 | Pharmacy | 7-2-4-6-06-59 | x | 11-Dec-12 |
| 4-59 | Pharmacy | 14-1-3-4-04-59 | x | 10-Dec-12 |
| 5-59 | Pharmacy | 15-2-1-5-05-59 | x | 14-Dec-12 |
| 10-59 | Pharmacy | 15-2-3-10-10-59 | x | 11-Dec-12 |
| 2-59 | Pharmacy | 16-1-3-2-02-59 | x | 11-Dec-12 |
| 23-61 | PMV | 4-1-2-23-23-61 | x | 10-Dec-12 |
| 37-67 | PMV | 5-1-2-37-37-67 | x | 13-Dec-12 |
| 10-67B | Pharmacy | 12-2-5-10-10-67B | x | 12-Dec-12 |
| 8-67A | Pharmacy | 4-1-2-8-08-67A | x | 13-Dec-12 |
| 8-67G | Pharmacy | 5-1-2-8-08-67G | x | 11-Dec-12 |
| 11-67F | Pharmacy | 6-2-1-11-11-67F | x | 18-Dec-12 |
| 5-067a | Pharmacy | 8-2-4-5-05-067a | x | 13-Dec-12 |
| 6-67 | Pharmacy | 12-3-7-6-06-67 | x | 11-Dec-12 |
| 3-67A | Pharmacy | 13-3-7-3-03-67A | x | 11-Dec-12 |

| | | | | |
|---|---|---|---|---|
| 21-10 | Pharmacy | 1-3-1-21-21-10 | x | 13-Dec-12 |
| 59-10 | PMV | 1-3-1-59-59-10 | x | 11-Dec-12 |
| 34-10 | PMV | 2-3-5-34-34-10 | x | 12-Dec-12 |
| 36-10 | PMV | 4-1-2-36-36-10 | x | 12-Dec-12 |
| 8-10 | Pharmacy | 5-1-2-8-08-10 | x | 11-Dec-12 |
| 11-10 | Pharmacy | 6-2-1-11-11-10 | x | 18-Dec-12 |
| 6-10 | Pharmacy | 6-2-3-6-06-10 | x | 11-Dec-12 |
| 11-10 | PMV | 9-2-2-11-11-10 | x | 18-Dec-12 |
| 8-10 | PMV | 9-3-5-8-8-10 | x | 11-Dec-12 |
| 2-10 | PMV | 10-3-1-2-02-10 | x | 15-Dec-12 |
| 6-10 | Pharmacy | 11-2-5-6-06-10 | x | 21-Dec-12 |
| 9-10 | Pharmacy | 13-2-5-9-09-10 | x | 10-Dec-12 |
| 7-10 | Pharmacy | 13-3-7-7-07-10 | x | 11-Dec-12 |
| 1-10 | Pharmacy | 14-1-3-1-01-10 | x | 11-Dec-12 |
| 29-10 | Pharmacy | 15-2-1-29-29-10 | x | 14-Dec-12 |
| 38-10 | Pharmacy | 15-2-3-38-38-10 | x | 13-Dec-12 |
| 19-67B | PMV | 15-2-1-19-19-67B | x | 18-Dec-12 |
| 28-67b | PMV | 15-2-3-28-28-67b | x | 08-Dec-12 |
| 13-47 | PMV | 3-2-4-13-13-47 | x | 13-Dec-12 |
| 36-47 | PMV | 4-1-2-36-36-47 | x | 12-Dec-12 |
| 7-47 | Pharmacy | 5-2-3-7-07-47 | x | 08-Dec-12 |
| 1-47 | Pharmacy | 11-2-5-1-1-47 | x | 13-Dec-12 |
| 16-47 | Pharmacy | 12-2-5-16-16-47 | x | 10-Dec-12 |
| 4-47 | Pharmacy | 14-1-3-4-4-47 | x | 10-Dec-12 |
| 1-67C | Pharmacy | 4-1-2-1-01-67C | x | 10-Dec-12 |
| 1-61 | Pharmacy | 4-1-2-1-01-61 | x | 10-Dec-12 |
| 7-61 | Pharmacy | 5-2-3-7-07-61 | x | 08-Dec-12 |
| 10-2 | Pharmacy | 1-3-1-10-10-02 | x | 14-Dec-12 |
| 81-2 | Pharmacy | 1-3-1-81-81-02 | x | 18-Dec-12 |
| 2-2 | PMV | 2-3-6-2-02-02 | x | 10-Dec-12 |
| 2-2 | Pharmacy | 3-2-1-2-02-02 | x | 10-Dec-12 |
| 7-2 | Pharmacy | 5-2-3-7-07-02 | x | 08-Dec-12 |
| 6-2 | Pharmacy | 6-2-3-6-06-02 | x | 11-Dec-12 |
| 1-2 | Pharmacy | 7-2-4-1-01-02 | x | 14-Dec-12 |
| 5-2 | Pharmacy | 8-2-4-5-05-02 | x | 11-Dec-12 |
| 1-2 | Pharmacy | 9-2-2-1-01-02 | x | 18-Dec-12 |
| 8-2 | PMV | 9-3-6-8-08-02 | x | 13-Dec-12 |
| 2-2 | Pharmacy | 10-3-1-2-02-02 | x | 15-Dec-12 |
| 15-2 | Pharmacy | 10-3-1-15-15-02 | x | 10-Dec-12 |
| 5-2 | Pharmacy | 11-2-5-5-05-02 | x | 12-Dec-12 |
| 12-2 | Pharmacy | 11-2-5-12-12-2 | x | 12-Dec-12 |
| 16-2 | PMV | 12-2-5-16-16-02 | x | 10-Dec-12 |
| 1-2 | Pharmacy | 14-1-1-1-01-02 | x | 13-Dec-12 |
| 29-2 | PMV | 14-1-3-29-29-02 | x | 13-Dec-12 |
| 20-2 | PMV | 15-2-3-20-20-02 | x | 12-Dec-12 |
| 38-2 | Pharmacy | 15-2-3-38-38-02 | x | 13-Dec-12 |
| 7-2 | Pharmacy | 16-1-3-7-07-02 | x | 11-Dec-12 |
| 11-2 | Pharmacy | 16-1-3-11-11-2 | x | 10-Dec-12 |
| 14-2 | PMV | 16-1-3-14-14-02 | x | 12-Dec-12 |
| 30-2 | PMV | 16-1-3-30-30-2 | x | 18-Dec-12 |

| | | | | |
|---|---|---|---|---|
| 9-5 | Pharmacy | 4-1-2-9-9-5 | x | 11-Dec-12 |
| 53-5 | PMV | 4-1-2-53-53-05 | x | 18-Dec-12 |
| 31-5 | PMV | 5-1-2-31-31-05 | x | 12-Dec-12 |
| 59-5 | Pharmacy | 5-1-2-59-59-05 | x | 13-Dec-12 |
| 36-05b | PMV | 6-2-3-36-36-05b | x | 08-Dec-12 |
| 1-5 | Pharmacy | 7-2-4-1-01-05 | x | 14-Dec-12 |
| 25-5 | PMV | 7-2-4-25-25-05 | x | 13-Dec-12 |
| 8-5 | PMV | 9-3-5-8-08-05 | x | 11-Dec-12 |
| 33-25A | PMV | 10-3-1-33-33-25A | x | 15-Dec-12 |
| 71-5 | Pharmacy | 10-3-1-71-71-05 | x | 15-Dec-12 |
| 1-5 | Pharmacy | 11-2-5-1-01-05 | x | 13-Dec-12 |
| 5-5 | Pharmacy | 11-2-5-5-05-5 | x | 12-Dec-12 |
| 6-5 | Pharmacy | 11-2-5-6-06-05 | x | 21-Dec-12 |
| 3-5 | Pharmacy | 12-2-5-3-03-05 | x | 12-Dec-12 |
| 16-5 | PMV | 12-2-5-16-16-05 | x | 10-Dec-12 |
| 28-5 | PMV | 12-2-5-28-28-05 | x | 10-Dec-12 |
| 1-5 | Pharmacy | 14-1-1-1-01-05 | x | 13-Dec-12 |
| 4-5 | Pharmacy | 14-1-3-4-04-05 | x | 10-Dec-12 |
| 38-5 | Pharmacy | 16-1-3-38-38-05 | x | 14-Dec-12 |
| 2-6 | Pharmacy | 3-2-1-2-02-06 | x | 10-Dec-12 |
| 11-6 | Pharmacy | 6-2-1-11-11-06 | x | 18-Dec-12 |
| 6-6 | Pharmacy | 6-2-3-6-06-06 | x | 11-Dec-12 |
| 1-6 | Pharmacy | 9-2-2-1-01-06 | x | 18-Dec-12 |
| 11-6 | PMV | 9-2-2-11-11-06 | x | 18-Dec-12 |
| 1-6 | Pharmacy | 11-2-5-1-01-06 | x | 13-Dec-12 |
| 2-6 | Pharmacy | 16-1-3-2-02-06 | x | 11-Dec-12 |
| 1-9 | Pharmacy | 7-2-4-1-01-09 | x | 14-Dec-12 |
| 1-9 | Pharmacy | 11-2-5-1-01-09 | x | 13-Dec-12 |
| 6-9 | Pharmacy | 11-2-5-6-06-09 | x | 21-Dec-12 |
| 19-67E | Pharmacy | 4-1-2-19-19-67E | x | 14-Dec-12 |
| 9-67A | Pharmacy | 6-2-3-9-09-67A | x | 11-Dec-12 |
| 38-67B | Pharmacy | 15-2-3-38-38-67B | x | 13-Dec-12 |
| 12-67d | Pharmacy | 5-2-3-12-12-67d | x | 08-Dec-12 |
| 10-67a | Pharmacy | 9-2-2-10-10-67a | x | 18-Dec-12 |
| 19-67A | Pharmacy | 15-2-1-19-19-67A | x | 18-Dec-12 |
| 57-67a | PMV | 5-1-2-57-57-67a | x | 14-Dec-12 |
| 60-67B | PMV | 1-3-1-60-60-67B | x | 11-Dec-12 |
| 30-67 | PMV | 2-3-5-30-30-67 | x | 11-Dec-12 |
| 1-67B | Pharmacy | 4-1-2-1-01-67B | x | 10-Dec-12 |
| 11-67 | PMV | 9-3-5-11-11-67 | x | 11-Dec-12 |
| 1-19 | Pharmacy | 4-1-2-1-01-19 | x | 10-Dec-12 |
| 36-19 | PMV | 4-1-2-36-36-19 | x | 12-Dec-12 |
| 31-19 | PMV | 5-1-2-31-31-19 | x | 12-Dec-12 |
| 8-19 | PMV | 9-3-6-8-08-19 | x | 13-Dec-12 |
| 16-19 | PMV | 12-2-5-16-16-19 | x | 10-Dec-12 |
| 9-19 | Pharmacy | 14-1-3-9-9-19 | x | 12-Dec-12 |
| 5-19 | Pharmacy | 15-2-1-5-05-19 | x | 14-Dec-12 |
| 57-67b | PMV | 5-1-2-57-57-67b | x | 14-Dec-12 |
| 81-67 | Pharmacy | 1-3-1-81-81-67 | x | 18-Dec-12 |
| 42-67 | PMV | 3-2-4-42-42-67 | x | 13-Dec-12 |

| | | | | |
|---|---|---|---|---|
| 0-67 | Publichealth fa | 7-3-7-0-0-67 | x | 21-Dec-12 |
| 8-67C | Pharmacy | 8-2-1-8-08-67C | x | 10-Dec-12 |
| 10-67B | Pharmacy | 8-2-4-10-10-67B | x | 12-Dec-12 |
| 2-67 | PMV | 10-3-1-2-02-67 | x | 15-Dec-12 |
| 1-67 | Pharmacy | 14-1-1-1-01-67 | x | 13-Dec-12 |
| 16-67 | PMV | 14-1-1-16-16-67 | x | 13-Dec-12 |
| 4-67D | Pharmacy | 14-1-3-4-04-67D | x | 10-Dec-12 |
| 5-67a | Pharmacy | 15-2-1-5-05-67a | x | 14-Dec-12 |
| 14-67A | PMV | 15-2-1-14-14-67A | x | 17-Dec-12 |
| 10-67A | Pharmacy | 15-2-3-10-10-67A | x | 11-Dec-12 |
| 77-29 | PMV | 1-3-1-77-77-29 | x | 18-Dec-12 |
| 21-29 | PMV | 3-2-4-21-21-29 | x | 10-Dec-12 |
| 1-29 | Pharmacy | 4-1-2-1-01-29 | x | 10-Dec-12 |
| 7-29 | Pharmacy | 5-1-2-7-07-29 | x | 12-Dec-12 |
| 7-29 | Pharmacy | 5-2-3-7-07-29 | x | 08-Dec-12 |
| 31-29 | PMV | 6-2-3-31-31-29 | x | 10-Dec-12 |
| 1-29 | Pharmacy | 7-2-4-1-01-29 | x | 14-Dec-12 |
| 25-29 | PMV | 7-2-4-25-25-29 | x | 13-Dec-12 |
| 44-29 | PMV | 8-2-4-44-44-29 | x | 12-Dec-12 |
| 1-29 | Pharmacy | 9-2-2-1-01-29 | x | 18-Dec-12 |
| 8-29 | PMV | 9-3-6-8-08-29 | x | 13-Dec-12 |
| 15-29 | PMV | 10-3-1-15-15-29 | x | 10-Dec-12 |
| 1-32 | Pharmacy | 11-2-5-1-01-32 | x | 13-Dec-12 |
| 5-29 | Pharmacy | 11-2-5-5-05-29 | x | 12-Dec-12 |
| 16-29 | PMV | 12-2-5-16-16-29 | x | 10-Dec-12 |
| 18-28 | Pharmacy | 14-1-1-18-18-28 | x | 14-Dec-12 |
| 11-29 | Pharmacy | 16-1-3-11-11-29 | x | 10-Dec-12 |
| 4-32 | Pharmacy | 1-3-1-4-04-32 | x | 10-Dec-12 |
| 57-32 | PMV | 1-3-1-57-57-32 | x | 15-Dec-12 |
| 82-32 | PMV | 1-3-1-82-82-32 | x | 18-Dec-12 |
| 56-32 | PMV | 5-1-2-56-56-32 | x | 13-Dec-12 |
| 8-32 | PMV | 9-3-6-8-08-32 | x | 13-Dec-12 |
| 2-32 | Pharmacy | 10-3-1-2-02-32 | x | 15-Dec-12 |
| 21-67A | PMV | 10-3-1-21-21-67A | x | 13-Dec-12 |
| 31-32 | Pharmacy | 10-3-1-3-31-32 | x | 14-Dec-12 |
| 24-32 | PMV | 11-3-7-24-24-32 | x | 11-Dec-12 |
| 16-37 | PMV | 12-2-5-16-16-37 | x | 10-Dec-12 |
| 14-67B | PMV | 6-2-3-14-14-67B | x | 10-Dec-12 |
| 8-67C | Pharmacy | 4-1-2-8-08-67C | x | 13-Dec-12 |
| 53-67B | PMV | 4-1-2-53-53-67B | x | 18-Dec-12 |
| 19-67C | PMV | 4-1-2-19-19-67C | x | 14-Dec-12 |
| 5-067b | Pharmacy | 8-2-4-5-05-067b | x | 13-Dec-12 |
| 6-67A | Pharmacy | 9-2-2-6-06-67A | x | 18-Dec-12 |
| 29-67C | PMV | 15-2-1-29-29-67C | x | 14-Dec-12 |
| 57-40 | PMV | 1-3-1-57-57-40 | x | 15-Dec-12 |
| 14-40 | PMV | 3-2-4-14-14-40 | x | 12-Dec-12 |
| 31-40 | PMV | 5-1-2-31-31-40 | x | 12-Dec-12 |
| 11-40 | Pharmacy | 6-2-1-11-11-40 | x | 18-Dec-12 |
| 6-40 | Pharmacy | 6-2-3-6-06-40 | x | 11-Dec-12 |
| 10-40 | Pharmacy | 8-2-4-10-10-40 | x | 12-Dec-12 |

| | | | | |
|---|---|---|---|---|
| 1-40 | Pharmacy | 9-2-2-1-01-40 | x | 18-Dec-12 |
| 1-40 | Pharmacy | 11-2-5-1-01-40 | x | 13-Dec-12 |
| 5-40 | Pharmacy | 11-2-5-5-05-40 | x | 12-Dec-12 |
| 9-40 | Pharmacy | 13-3-7-9-09-40 | x | 11-Dec-12 |
| 7-40 | Pharmacy | 15-2-1-7-07-40 | x | 14-Dec-12 |
| 4-41 | Pharmacy | 4-1-2-4-04-41 | x | 12-Dec-12 |
| 3-33 | Pharmacy | 4-2-3-3-03-33 | x | 08-Dec-12 |
| 7-67a | Pharmacy | 3-2-4-7-07-67a | x | 12-Dec-12 |
| 23-67 | PMV | 4-1-2-23-23-67 | x | 18-Dec-12 |
| 7-67C | Pharmacy | 5-1-2-7-07-67C | x | 12-Dec-12 |
| 17-67a | PMV | 5-1-2-17-17-67a | x | 13-Dec-12 |
| 22-67c | PMV | 5-1-2-22-22-67c | x | 13-Dec-12 |
| 28-67D | PMV | 6-2-1-28-28-67D | x | 12-Dec-12 |
| 1-67A | Pharmacy | 6-2-3-1-01-67A | x | 21-Dec-12 |
| 20-67B | PMV | 7-2-4-20-20-67B | x | 08-Dec-12 |
| 25-67 | PMV | 7-2-4-25-25-67 | x | 14-Dec-12 |
| 33-67 | PMV | 10-3-1-33-33-67 | x | 08-Dec-12 |
| 1-67B | Pharmacy | 11-2-5-1-01-67B | x | 11-Dec-12 |
| 5-67A | Pharmacy | 11-2-5-5-05-67A | x | 10-Dec-12 |
| 6-67A | Pharmacy | 11-2-5-6-06-67A | x | 04-Dec-12 |
| 11-67 | Pharmacy | 11-2-5-11-11-67 | x | 14-Dec-12 |
| 16-67 | Pharmacy | 13-2-3-16-16-67 | x | 10-Dec-12 |
| 9-67 | Pharmacy | 13-2-5-9-09-67 | x | 15-Dec-12 |
| 25-67 | PMV | 13-2-5-25-25-67 | x | 12-Dec-12 |
| 5-67c | Pharmacy | 15-2-1-5-05-67c | x | 10-Dec-12 |
| 19-67C | PMV | 15-2-1-19-19-67C | x | 10-Dec-12 |
| 20-67 | PMV | 15-2-3-20-20-67 | x | 18-Dec-12 |
| 34-67b | PMV | 15-2-3-34-34-67b | x | 12-Dec-12 |
| 19-67D | PMV | 15-2-1-19-19-67D | x | 18-Dec-12 |
| 7-67b | Pharmacy | 3-2-4-7-07-67b | x | 11-Dec-12 |
| 37-67F | PMV | 6-2-3-37-37-67F | x | 12-Dec-12 |
| 1-67C | Pharmacy | 7-2-4-1-01-67C | x | 13-Dec-12 |
| 5-067c | Pharmacy | 8-2-4-5-05-067c | x | 13-Dec-12 |
| 5-67d | Pharmacy | 15-2-1-5-05-67d | x | 14-Dec-12 |
| 28-67a | PMV | 15-2-3-28-28-67a | x | 08-Dec-12 |
| 77-48 | PMV | 1-3-1-77-77-48 | x | 18-Dec-12 |
| 5-48 | PMV | 2-3-2-5-05-48 | x | 10-Dec-12 |
| 53-48 | PMV | 4-1-2-53-53-48 | x | 18-Dec-12 |
| 57-48 | PMV | 5-1-2-57-57-48 | x | 14-Dec-12 |
| 59-48 | PMV | 5-1-2-59-59-48 | x | 13-Dec-12 |
| 12-48 | Pharmacy | 5-2-3-12-12-48 | x | 08-Dec-12 |
| 11-48 | Pharmacy | 6-2-1-11-11-48 | x | 18-Dec-12 |
| 37-48 | PMV | 6-2-3-37-37-48 | x | 12-Dec-12 |
| 1-48 | Pharmacy | 7-2-4-1-01-48 | x | 13-Dec-12 |
| 25-48 | PMV | 7-2-4-25-25-48 | x | 13-Dec-12 |
| 1-45 | Pharmacy | 9-2-2-1-01-45 | x | 18-Dec-12 |
| 1-48 | Pharmacy | 11-2-5-1-01-48 | x | 13-Dec-12 |
| 5-48 | Pharmacy | 11-2-5-5-05-48 | x | 12-Dec-12 |
| 10-47 | Pharmacy | 12-2-5-10-10-47 | x | 12-Dec-12 |
| 9-48 | Pharmacy | 13-2-5-9-09-48 | x | 10-Dec-12 |

| | | | | |
|---|---|---|---|---|
| 14-48 | PMV | 15-2-1-14-14-48 | x | 17-Dec-12 |
| 38-48 | PMV | 15-2-3-38-38-48 | x | 13-Dec-12 |
| 4-51 | Pharmacy | 1-3-1-4-04-51 | x | 10-Dec-12 |
| 12-51 | Pharmacy | 1-3-1-12-12-51 | x | 13-Dec-12 |
| 13-51 | Pharmacy | 1-3-1-13-13-51 | x | 14-Dec-12 |
| 33-51 | PMV | 1-3-1-33-33-51 | x | 15-Dec-12 |
| 36-51 | PMV | 1-3-1-36-36-51 | x | 18-Dec-12 |
| 38-51 | PMV | 1-3-1-38-38-51 | x | 14-Dec-12 |
| 59-51 | PMV | 1-3-1-59-59-51 | x | 11-Dec-12 |
| 60-51A | PMV | 1-3-1-60-60-51A | x | 13-Dec-12 |
| 69-31 | PMV | 1-3-1-69-69-31 | x | 14-Dec-12 |
| 77-51 | PMV | 1-3-1-77-77-51 | x | 18-Dec-12 |
| 80-51 | PMV | 1-3-1-80-80-51 | x | 18-Dec-12 |
| 82-51 | PMV | 1-3-1-82-82-51 | x | 18-Dec-12 |
| 83-51 | PMV | 1-3-1-83-83-51 | x | 18-Dec-12 |
| 84-51 | PMV | 1-3-1-84-84-51 | x | 18-Dec-12 |
| 5-49 | PMV | 2-3-2-5-05-49 | x | 10-Dec-12 |
| 2-49 | PMV | 2-3-6-2-02-49 | x | 10-Dec-12 |
| 2-51 | Pharmacy | 3-2-1-2-02-51 | x | 10-Dec-12 |
| 2-51 | Pharmacy | 3-2-4-2-02-51 | x | 10-Dec-12 |
| 8-51 | Pharmacy | 3-2-4-8-08-51 | x | 11-Dec-12 |
| 14-51 | Pharmacy | 3-2-4-14-14-51 | x | 12-Dec-12 |
| 15-51 | PMV | 3-2-4-15-15-51 | x | 11-Dec-12 |
| 42-51 | PMV | 3-2-4-42-42-51 | x | 13-Dec-12 |
| 6-51 | PMV | 3-2-6-6-06-51 | x | 19-Dec-12 |
| 15-51 | Pharmacy | 4-1-2-15-15-51 | x | 12-Dec-12 |
| 23-51 | Pharmacy | 4-1-2-23-23-51 | x | 10-Dec-12 |
| 53-51 | PMV | 4-1-2-53-53-51 | x | 18-Dec-12 |
| 3-49 | Pharmacy | 4-2-3-3-03-49 | x | 08-Dec-12 |
| 8-51 | Pharmacy | 5-1-2-8-08-51 | x | 11-Dec-12 |
| 46-51 | PMV | 5-1-2-46-46-51 | x | 14-Dec-12 |
| 56-51 | PMV | 5-1-2-56-56-51 | x | 13-Dec-12 |
| 57-51a | PMV | 5-1-2-57-57-51a | x | 14-Dec-12 |
| 59-51 | PMV | 5-1-2-59-59-51 | x | 13-Dec-12 |
| 0-51 | Publichealth fa | 5-2-1-0-0-51 | x | 20-Dec-12 |
| 12-51 | Pharmacy | 5-2-3-12-12-51 | x | 08-Dec-12 |
| 1-51 | Pharmacy | 6-2-1-1-01-51 | x | 14-Dec-12 |
| 11-51 | Pharmacy | 6-2-1-11-11-51 | x | 18-Dec-12 |
| 1-51 | Pharmacy | 6-2-3-1-01-51 | x | 12-Dec-12 |
| 9-51 | Pharmacy | 6-2-3-9-09-51 | x | 11-Dec-12 |
| 25-51 | PMV | 6-2-3-25-25-51 | x | 08-Dec-12 |
| 27-51 | PMV | 6-2-3-27-27-51 | x | 08-Dec-12 |
| 31-51 | PMV | 6-2-3-31-31-51 | x | 10-Dec-12 |
| 37-51 | PMV | 6-2-3-37-37-51 | x | 12-Dec-12 |
| 1-51 | Pharmacy | 7-2-4-1-01-51 | x | 14-Dec-12 |
| 22-51A | PMV | 7-2-4-22-22-51A | x | 13-Dec-12 |
| 8-51 | Pharmacy | 8-2-1-8-08-51 | x | 10-Dec-12 |
| 5-51 | Pharmacy | 8-2-4-5-05-51 | x | 11-Dec-12 |
| 10-51 | Pharmacy | 8-2-4-10-10-51 | x | 12-Dec-12 |
| 16-49 | PMV | 8-2-4-16-16-49 | x | 13-Dec-12 |

| | | | | |
|---|---|---|---|---|
| 23-51 | PMV | 8-2-4-23-23-51 | x | 13-Dec-12 |
| 2-51 | Pharmacy | 9-2-2-2-02-51 | x | 18-Dec-12 |
| 10-51 | Pharmacy | 9-2-2-10-10-51 | x | 18-Dec-12 |
| 11-51 | Pharmacy | 9-2-2-11-11-51 | x | 18-Dec-12 |
| 19-51 | PMV | 9-3-5-19-19-51 | x | 11-Dec-12 |
| 8-51 | PMV | 9-3-6-8-08-51 | x | 13-Dec-12 |
| 1-51 | PMV | 10-3-1-1-01-51 | x | 14-Dec-12 |
| 2-51 | Pharmacy | 10-3-1-2-02-51 | x | 15-Dec-12 |
| 5-51 | Pharmacy | 10-3-1-5-05-51 | x | 13-Dec-12 |
| 8-51 | Pharmacy | 10-3-1-8-08-51 | x | 18-Dec-12 |
| 14-51 | Pharmacy | 10-3-1-14-14-51 | x | 18-Dec-12 |
| 21-51 | PMV | 10-3-1-21-21-51 | x | 13-Dec-12 |
| 31-51 | PMV | 10-3-1-31-31-51 | x | 14-Dec-12 |
| 33-51 | PMV | 10-3-1-33-33-51 | x | 15-Dec-12 |
| 37-51 | PMV | 10-3-1-37-37-51 | x | 11-Dec-12 |
| 53-51 | PMV | 10-3-1-53-53-51 | x | 18-Dec-12 |
| 66-51 | PMV | 10-3-1-66-66-51 | x | 14-Dec-12 |
| 71-51 | PMV | 10-3-1-71-71-51 | x | 15-Dec-12 |
| 78-51 | PMV | 10-3-1-78-78-51 | x | 13-Dec-12 |
| 79-51 | PMV | 10-3-1-79-79-51 | x | 11-Dec-12 |
| 1-51 | Pharmacy | 11-2-5-1-01-51 | x | 13-Dec-12 |
| 6-51 | Pharmacy | 11-2-5-3-06-51 | x | 12-Dec-12 |
| 5-51 | Pharmacy | 11-2-5-5-05-51 | x | 12-Dec-12 |
| 6-51 | Pharmacy | 11-2-5-6-06-51 | x | 21-Dec-12 |
| 11-51 | Pharmacy | 11-2-5-11-11-51 | x | 12-Dec-12 |
| 12-51 | PMV | 11-2-5-12-12-51 | x | 12-Dec-12 |
| 22-51 | PMV | 11-2-5-22-22-51 | x | 10-Dec-12 |
| 26-51 | PMV | 11-2-5-26-26-51 | x | 10-Dec-12 |
| 15-51 | PMV | 11-3-7-15-15-51 | x | 11-Dec-12 |
| 10-51 | Pharmacy | 12-2-5-10-10-51 | x | 12-Dec-12 |
| 16-51 | PMV | 12-2-5-16-16-51 | x | 10-Dec-12 |
| 18-51 | PMV | 12-3-7-18-18-51 | x | 11-Dec-12 |
| 23-51 | PMV | 12-3-7-23-23-51 | x | 11-Dec-12 |
| 16-51 | PMV | 13-2-3-16-16-51 | x | 08-Dec-12 |
| 3-49 | Pharmacy | 13-3-7-3-03-49 | x | 11-Dec-12 |
| 9-49 | Pharmacy | 13-2-5-9-09-49 | x | 10-Dec-12 |
| 4-51 | Pharmacy | 13-3-7-4-04-51 | x | 11-Dec-12 |
| 8-51 | Pharmacy | 13-3-7-8-08-51 | x | 11-Dec-12 |
| 28-57 | PMV | 13-3-7-28-28-57 | x | 13-Dec-12 |
| 31-51 | PMV | 13-3-7-31-31-51 | x | 14-Dec-12 |
| 33-51 | PMV | 13-3-7-33-33-51 | x | 14-Dec-12 |
| 1-51 | PMV | 14-1-1-1-01-51 | x | 13-Dec-12 |
| 6-51 | PMV | 14-1-1-6-6-51 | x | 14-Dec-12 |
| 7-51 | PMV | 14-1-1-7-07-51 | x | 14-Dec-12 |
| 4-51 | Pharmacy | 14-1-3-4-04-51 | x | 10-Dec-12 |
| 5-51 | Pharmacy | 14-1-3-5-05-51 | x | 11-Dec-12 |
| 9-51 | Pharmacy | 14-1-3-9-9-51 | x | 12-Dec-12 |
| 10-51 | Pharmacy | 14-1-3-10-10-51 | x | 13-Dec-12 |
| 23-51 | PMV | 14-1-3-23-23-51 | x | 12-Dec-12 |
| 35-51 | PMV | 14-1-3-35-35-51 | x | 14-Dec-12 |

| | | | | |
|---|---|---|---|---|
| 4-51 | Pharmacy | 15-2-1-4-4-51 | x | 12-Dec-12 |
| 5-51 | Pharmacy | 15-2-1-5-05-51 | x | 14-Dec-12 |
| 7-51 | Pharmacy | 15-2-1-7-07-51 | x | 14-Dec-12 |
| 14-51 | Pharmacy | 15-2-1-14-14-51 | x | 17-Dec-12 |
| 17-51 | PMV | 15-2-1-17-17-51 | x | 14-Dec-12 |
| 29-51 | PMV | 15-2-1-29-29-51 | x | 14-Dec-12 |
| 10-51 | Pharmacy | 15-2-3-10-10-51 | x | 11-Dec-12 |
| 15-51 | Pharmacy | 15-2-3-15-15-51 | x | 10-Dec-12 |
| 20-51 | PMV | 15-2-3-20-20-51 | x | 12-Dec-12 |
| 30-51 | PMV | 15-2-3-30-30-51 | x | 10-Dec-12 |
| 38-51 | PMV | 15-2-3-38-38-51 | x | 13-Dec-12 |
| 7-51 | Pharmacy | 16-1-1-7-07-51 | x | 14-Dec-12 |
| 6-51 | Pharmacy | 16-1-3-6-06-051 | x | 12-Dec-12 |
| 7-51 | Pharmacy | 16-1-3-7-07-51 | x | 11-Dec-12 |
| 11-51 | Pharmacy | 16-1-3-11-11-51 | x | 10-Dec-12 |
| 30-51 | PMV | 16-1-3-30-30-51 | x | 18-Dec-12 |
| 38-51 | PMV | 16-1-3-38-38-51 | x | 14-Dec-12 |
| 2-19 | Pharmacy | 5-1-2-2-2-19 | x | 10-Dec-12 |
| 53-67C | PMV | 4-1-2-53-53-67C | x | 18-Dec-12 |
| 7-67E | Pharmacy | 5-1-2-7-7-67E | x | 12-Dec-12 |
| 9-67 | Pharmacy | 13-2-5-9-09-67 | x | 10-Dec-12 |
| 4-67A | Pharmacy | 14-1-3-4-4-67A | x | 10-Dec-12 |
| 29-67 | PMV | 14-1-3-29-29-67 | x | 13-Dec-12 |
| 38-67J | PMV | 15-2-3-38-38-67J | x | 13-Dec-12 |
| 33-53 | PMV | 1-3-1-33-33-53 | x | 15-Dec-12 |
| 1-53 | Pharmacy | 2-3-6-1-01-53 | x | 10-Dec-12 |
| 36-53 | PMV | 4-1-2-36-36-53 | x | 12-Dec-12 |
| 8-53 | Pharmacy | 5-1-2-8-08-53 | x | 11-Dec-12 |
| 57-53 | PMV | 5-1-2-57-57-53 | x | 14-Dec-12 |
| 11-53 | Pharmacy | 6-2-1-11-11-53 | x | 18-Dec-12 |
| 37-53 | PMV | 6-2-3-37-37-53 | x | 12-Dec-12 |
| 6-53 | Pharmacy | 7-2-4-6-06-53 | x | 11-Dec-12 |
| 5-53 | Pharmacy | 8-2-4-5-05-053 | x | 13-Dec-12 |
| 21-53 | PMV | 10-3-1-21-21-53 | x | 13-Dec-12 |
| 33-53 | PMV | 10-3-1-33-33-53 | x | 15-Dec-12 |
| 6-53 | Pharmacy | 11-2-5-6-06-53 | x | 21-Dec-12 |
| 11-53 | Pharmacy | 11-2-5-11-11-53 | x | 12-Dec-12 |
| 23-53 | PMV | 12-3-7-23-23-53 | x | 11-Dec-12 |
| 33-53 | PMV | 13-3-7-33-33-53 | x | 14-Dec-12 |
| 9-55 | Pharmacy | 14-1-3-9-9-55 | x | 12-Dec-12 |
| 38-53 | PMV | 15-2-3-38-38-53 | x | 13-Dec-12 |
| 14-53 | Pharmacy | 16-1-3-4-014-053 | x | 12-Dec-12 |
| 11-53 | Pharmacy | 16-1-3-11-11-53 | x | 10-Dec-12 |
| 33-67B | PMV | 2-3-5-33-33-67B | x | 12-Dec-12 |
| 8-67H | Pharmacy | 5-1-2-8-8-67H | x | 11-Dec-12 |
| 31-67 | PMV | 5-1-2-31-31-67 | x | 12-Dec-12 |
| 1-67C | Pharmacy | 11-2-5-1-01-67C | x | 13-Dec-12 |
| 4-067B | Pharmacy | 14-1-3-4-04-067B | x | 10-Dec-12 |
| 33-55 | PMV | 2-3-5-33-33-55 | x | 12-Dec-12 |
| 6-55 | PMV | 6-2-3-6-06-55 | x | 11-Dec-12 |

| | | | | |
|---|---|---|---|---|
| 15-55 | Pharmacy | 10-3-1-15-15-55 | x | 10-Dec-12 |
| 2-55 | Pharmacy | 16-1-3-2-02-55 | x | 11-Dec-12 |
| 10-56 | Pharmacy | 1-3-1-10-10-056 | x | 14-Dec-12 |
| 7-56 | Pharmacy | 3-2-4-7-07-56 | x | 11-Dec-12 |
| 42-56 | PMV | 3-2-4-42-42-56 | x | 13-Dec-12 |
| 1-56 | Pharmacy | 4-1-2-1-01-56 | x | 10-Dec-12 |
| 9-56 | Pharmacy | 4-1-2-9-09-56 | x | 11-Dec-12 |
| 8-56 | Pharmacy | 5-1-2-8-08-56 | x | 11-Dec-12 |
| 11-56 | Pharmacy | 6-2-1-11-11-56 | x | 18-Dec-12 |
| 6-56 | Pharmacy | 7-2-4-6-06-56 | x | 11-Dec-12 |
| 2-56 | Pharmacy | 10-3-1-2-02-56 | x | 15-Dec-12 |
| 8-56 | Pharmacy | 10-3-1-8-08-56 | x | 18-Dec-12 |
| 15-56 | Pharmacy | 10-3-1-15-15-56 | x | 10-Dec-12 |
| 33-56 | PMV | 10-3-1-33-33-56 | x | 15-Dec-12 |
| 37-56 | PMV | 10-3-1-37-37-56 | x | 11-Dec-12 |
| 71-56 | PMV | 10-3-1-71-71-56 | x | 15-Dec-12 |
| 6-56 | Pharmacy | 11-2-5-6-06-56 | x | 21-Dec-12 |
| 12-26 | Pharmacy | 11-2-5-12-12-26 | x | 12-Dec-12 |
| 26-67b | PMV | 11-2-5-26-26-67b | x | 10-Dec-12 |
| 16-56 | PMV | 12-2-5-16-16-56 | x | 10-Dec-12 |
| 33-56 | PMV | 13-3-7-33-33-56 | x | 14-Dec-12 |
| 38-56 | PMV | 15-2-3-38-38-56 | x | 13-Dec-12 |
| 2-56 | Pharmacy | 16-1-3-2-02-56 | x | 11-Dec-12 |
| 8-61 | Pharmacy | 3-2-4-8-08-61 | x | 11-Dec-12 |
| 9-61 | Pharmacy | 4-1-2-9-09-61 | x | 11-Dec-12 |
| 8-61 | Pharmacy | 5-1-2-8-08-61 | x | 11-Dec-12 |
| 11-61 | Pharmacy | 5-1-2-11-11-61 | x | 12-Dec-12 |
| 25-61 | PMV | 6-2-3-25-25-61 | x | 08-Dec-12 |
| 31-61 | PMV | 6-2-3-31-31-61 | x | 10-Dec-12 |
| 6-61 | Pharmacy | 7-2-4-6-06-61 | x | 11-Dec-12 |
| 5-61 | Pharmacy | 8-2-4-5-05-61 | x | 11-Dec-12 |
| 21-61 | PMV | 10-3-1-21-21-61 | x | 13-Dec-12 |
| 5-61 | Pharmacy | 11-2-5-5-05-61 | x | 12-Dec-12 |
| 30-61 | PMV | 15-2-3-30-30-61 | x | 10-Dec-12 |
| 26-61 | PMV | 16-1-3-26-26-61 | x | 12-Dec-12 |
| 2-62 | Pharmacy | 3-2-1-2-02-62 | x | 10-Dec-12 |
| 14-62 | PMV | 3-2-4-14-14-62 | x | 12-Dec-12 |
| 6-62 | PMV | 3-2-6-6-06-62 | x | 19-Dec-12 |
| 8-62 | Pharmacy | 4-1-2-8-08-62 | x | 13-Dec-12 |
| 8-62 | Pharmacy | 5-1-2-8-08-62 | x | 11-Dec-12 |
| 28-62 | PMV | 6-2-1-28-28-62 | x | 14-Dec-12 |
| 6-62 | Pharmacy | 6-2-3-6-06-62 | x | 11-Dec-12 |
| 23-62 | PMV | 8-2-4-23-23-62 | x | 13-Dec-12 |
| 20-62 | PMV | 9-3-5-20-20-62 | x | 11-Dec-12 |
| 1-62 | Pharmacy | 11-2-5-1-01-62 | x | 13-Dec-12 |
| 6-62 | Pharmacy | 11-2-5-6-06-62 | x | 21-Dec-12 |
| 32-62 | PMV | 14-1-3-32-32-62 | x | 14-Dec-12 |
| 17-62 | PMV | 15-2-1-17-17-62 | x | 14-Dec-12 |
| 34-62 | PMV | 15-2-3-34-34-62 | x | 08-Dec-12 |
| 82-66 | PMV | 1-3-1-82-82-66 | x | 18-Dec-12 |

| | | | | |
|---|---|---|---|---|
| 2-66 | Pharmacy | 3-2-4-2-02-66 | x | 10-Dec-12 |
| 6-66 | PMV | 3-2-6-6-06-66 | x | 19-Dec-12 |
| 1-66 | Pharmacy | 4-1-2-1-01-66 | x | 10-Dec-12 |
| 31-66 | PMV | 5-1-2-31-31-66 | x | 12-Dec-12 |
| 12-66 | Pharmacy | 5-2-3-12-12-66 | x | 08-Dec-12 |
| 14-66 | Pharmacy | 6-2-3-14-14-66 | x | 10-Dec-12 |
| 5-66 | Pharmacy | 8-2-4-5-05-66 | x | 11-Dec-12 |
| 2-66 | Pharmacy | 10-3-1-2-02-66 | x | 15-Dec-12 |
| 31-12 | PMV | 10-3-1-31-31-12 | x | 14-Dec-12 |
| 71-66 | PMV | 10-3-1-71-71-66 | x | 15-Dec-12 |
| 16-66 | PMV | 12-2-5-16-16-66 | x | 10-Dec-12 |
| 32-66 | PMV | 14-1-3-32-32-66 | x | 14-Dec-12 |
| 7-66 | Pharmacy | 15-2-1-7-07-66 | x | 14-Dec-12 |
| 79-17 | PMV | 10-3-1-79-79-17 | x | 11-Dec-12 |
| 22-67a | Pharmacy | 5-1-2-22-22-67a | x | 04-Dec-12 |
| 4-17 | Pharmacy | 1-3-1-4-04-17 | x | 10-Dec-12 |
| 10-17 | Pharmacy | 1-3-1-10-10-017 | x | 14-Dec-12 |
| 12-17 | Pharmacy | 1-3-1-12-12-17 | x | 13-Dec-12 |
| 13-17 | Pharmacy | 1-3-1-13-13-17 | x | 14-Dec-12 |
| 38-17 | PMV | 1-3-1-38-38-17 | x | 14-Dec-12 |
| 39-17 | PMV | 1-3-1-39-39-17 | x | 14-Dec-12 |
| 57-17 | PMV | 1-3-1-57-57-17 | x | 15-Dec-12 |
| 68-17 | PMV | 1-3-1-68-68-17 | x | 14-Dec-12 |
| 69-17 | PMV | 1-3-1-69-69-17 | x | 14-Dec-12 |
| 70-17 | PMV | 1-3-1-70-70-17 | x | 15-Dec-12 |
| 74-17 | PMV | 1-3-1-74-74-17 | x | 14-Dec-12 |
| 81-17 | PMV | 1-3-1-81-81-17 | x | 18-Dec-12 |
| 82-17 | PMV | 1-3-1-82-82-17 | x | 18-Dec-12 |
| 8-17 | PMV | 2-3-2-8-08-17 | x | 10-Dec-12 |
| 3-17 | PMV | 2-3-4-3-3-17 | x | 10-Dec-12 |
| 4-17 | PMV | 2-3-5-4-04-17 | x | 11-Dec-12 |
| 25-27 | PMV | 2-3-5-25-25-27 | x | 11-Dec-12 |
| 1-17 | Pharmacy | 2-3-6-1-01-17 | x | 10-Dec-12 |
| 2-17 | PMV | 2-3-6-2-02-17 | x | 10-Dec-12 |
| 26-17 | PMV | 3-2-4-26-26-17 | x | 14-Dec-12 |
| 9-17 | Pharmacy | 4-1-2-9-9-17 | x | 11-Dec-12 |
| 15-17 | PMV | 4-1-2-15-15-17 | x | 12-Dec-12 |
| 19-17 | PMV | 4-1-2-19-19-17 | x | 14-Dec-12 |
| 53-12 | PMV | 4-1-2-53-53-12 | x | 18-Dec-12 |
| 11-17 | Pharmacy | 5-1-2-11-11-17 | x | 12-Dec-12 |
| 27-17 | PMV | 5-1-2-27-27-17 | x | 14-Dec-12 |
| 11-17 | Pharmacy | 6-2-1-11-11-17 | x | 18-Dec-12 |
| 13-17 | PMV | 6-2-1-13-13-17 | x | 13-Dec-12 |
| 15-17 | PMV | 6-2-1-15-15-17 | x | 17-Dec-12 |
| 14-17 | PMV | 6-2-3-14-14-17 | x | 10-Dec-12 |
| 39-17 | PMV | 6-2-3-39-39-17 | x | 13-Dec-12 |
| 38-17 | PMV | 16-1-3-38-38-17 | x | 14-Dec-12 |
| 2-17 | PMV | 9-2-2-2-02-17 | x | 18-Dec-12 |
| 7-17b | PMV | 9-2-2-7-07-17b | x | 18-Dec-12 |
| 10-17a | PMV | 9-2-2-10-10-17a | x | 18-Dec-12 |

| | | | | |
|---|---|---|---|---|
| 5-17 | Pharmacy | 11-2-5-5-05-17 | x | 12-Dec-12 |
| 3-17 | PMV | 9-3-3-3-03-17 | x | 13-Dec-12 |
| 8-17 | PMV | 9-3-5-8-08-17 | x | 11-Dec-12 |
| 11-17 | PMV | 9-3-5-11-11-17 | x | 11-Dec-12 |
| 31-17 | PMV | 9-3-5-31-31-17 | x | 12-Dec-12 |
| 8-17A | PMV | 9-3-6-8-08-17A | x | 13-Dec-12 |
| 20-17B | PMV | 10-3-1-20-20-17B | x | 13-Dec-12 |
| 25-17 | PMV | 10-3-1-25-25-17 | x | 15-Dec-12 |
| 33-17 | PMV | 10-3-1-33-33-17 | x | 15-Dec-12 |
| 66-17 | PMV | 10-3-1-66-66-17 | x | 14-Dec-12 |
| 72-17 | PMV | 10-3-1-72-72-17 | x | 15-Dec-12 |
| 11-17 | Pharmacy | 11-2-5-11-11-17 | x | 12-Dec-12 |
| 27-17 | PMV | 11-3-7-27-27-17 | x | 11-Dec-12 |
| 4-17 | Pharmacy | 12-2-5-4-04-17 | x | 12-Dec-12 |
| 24-17 | PMV | 12-2-5-24-24-17 | x | 10-Dec-12 |
| 12-17 | PMV | 12-3-7-12-12-17 | x | 11-Dec-12 |
| 16-17 | Pharmacy | 13-2-3-16-16-17 | x | 08-Dec-12 |
| 3-17 | Pharmacy | 13-3-7-3-03-17 | x | 11-Dec-12 |
| 4-17 | Pharmacy | 13-3-7-4-04-17 | x | 11-Dec-12 |
| 9-17 | Pharmacy | 13-3-7-9-09-17 | x | 11-Dec-12 |
| 32-17 | PMV | 13-3-7-32-32-17 | x | 14-Dec-12 |
| 5-17 | Pharmacy | 14-1-1-5-05-17 | x | 14-Dec-12 |
| 6-17A | Pharmacy | 14-1-1-6-06-17A | x | 14-Dec-12 |
| 6-17B | Pharmacy | 14-1-1-6-06-17B | x | 14-Dec-12 |
| 7-17 | Pharmacy | 14-1-1-7-07-17 | x | 14-Dec-12 |
| 15-17a | PMV | 14-1-1-15-15-17a | x | 13-Dec-12 |
| 1-17 | Pharmacy | 14-1-3-1-01-17 | x | 11-Dec-12 |
| 5-17 | Pharmacy | 14-1-3-5-05-17 | x | 11-Dec-12 |
| 9-17 | Pharmacy | 14-1-3-9-9-17 | x | 12-Dec-12 |
| 25-14 | PMV | 14-1-3-25-25-14 | x | 14-Dec-12 |
| 35-17a | PMV | 14-1-3-35-35-17a | x | 14-Dec-12 |
| 4-17 | Pharmacy | 15-2-1-4-04-17 | x | 12-Dec-12 |
| 7-17 | Pharmacy | 15-2-1-7-07-17 | x | 14-Dec-12 |
| 14-17 | PMV | 15-2-1-14-14-17 | x | 17-Dec-12 |
| 29-17 | PMV | 15-2-1-29-29-17 | x | 14-Dec-12 |
| 15-17 | Pharmacy | 15-2-3-15-15-17 | x | 10-Dec-12 |
| 38-17 | PMV | 15-2-3-38-38-17 | x | 13-Dec-12 |
| 17-17 | PMV | 16-1-1-17-17-17 | x | 14-Dec-12 |
| 28-17A | PMV | 16-1-1-28-28-17A | x | 14-Dec-12 |
| 7-17 | Pharmacy | 16-1-3-7-07-17 | x | 11-Dec-12 |
| 26-17 | PMV | 16-1-3-26-26-17 | x | 12-Dec-12 |
| 31-17A | PMV | 16-1-3-31-31-17A | x | 18-Dec-12 |
| 8-17 | Pharmacy | 13-3-7-8-08-17 | x | 11-Dec-12 |
| 13-42 | PMV | 3-2-4-13-13-42 | x | 13-Dec-12 |
| 4-42 | Pharmacy | 4-1-2-4-04-42 | x | 12-Dec-12 |
| 33-42 | PMV | 5-1-2-33-33-42 | x | 12-Dec-12 |
| 28-42 | PMV | 6-2-1-28-28-42 | x | 14-Dec-12 |
| 27-42 | PMV | 7-2-4-27-27-42 | x | 12-Dec-12 |
| 25-42 | PMV | 13-2-5-25-25-42 | x | 10-Dec-12 |
| 0-17 | Publichealth fa | 3-1-2-0-00-17 | x | 21-Dec-12 |

| | | | | |
|---|---|---|---|---|
| 9-67C | Pharmacy | 4-1-2-9-09-67C | x | 11-Dec-12 |
| 8-67K | Pharmacy | 5-1-2-8-8-67K | x | 11-Dec-12 |
| 59-43 | PMV | 5-1-2-59-59-43 | x | 13-Dec-12 |
| 38-43 | PMV | 15-2-3-38-38-43 | x | 13-Dec-12 |
| 28-67B | PMV | 6-2-1-28-28-67B | x | 14-Dec-12 |
| 31-65A | PMV | 9-3-5-31-31-65A | x | 12-Dec-12 |
| 5-67f | Pharmacy | 15-2-1-5-05-67f | x | 14-Dec-12 |
| 38-65 | Pharmacy | 1-3-1-38-38-65 | x | 14-Dec-12 |
| 7-64 | Pharmacy | 2-3-2-7-07-64 | x | 10-Dec-12 |
| 2-65 | Pharmacy | 4-2-3-2-02-65 | x | 08-Dec-12 |
| 2-65 | Pharmacy | 5-1-2-2-02-65 | x | 10-Dec-12 |
| 31-65 | PMV | 5-1-2-31-31-65 | x | 12-Dec-12 |
| 1-65 | Pharmacy | 6-2-1-1-01-65 | x | 14-Dec-12 |
| 11-65 | Pharmacy | 6-2-1-11-11-65 | x | 18-Dec-12 |
| 14-65 | Pharmacy | 6-2-3-14-14-65 | x | 10-Dec-12 |
| 4-65A | Pharmacy | 7-2-4-4-04-65A | x | 12-Dec-12 |
| 0-65 | Publichealth fa | 7-3-5-0-0-65 | x | 20-Dec-12 |
| 0-65 | Publichealth fa | 8-2-1-0-00-65 | x | 20-Dec-12 |
| 0-65B | Publichealth fa | 9-2-1-0-0-65B | x | 21-Dec-12 |
| 0-65 | Publichealth fa | 9-2-5-0-0-65 | x | 20-Dec-12 |
| 31-65B | PMV | 9-3-5-31-31-65B | x | 12-Dec-12 |
| 21-65A | PMV | 10-3-1-21-21-65A | x | 13-Dec-12 |
| 53-65 | PMV | 10-3-1-53-53-65 | x | 18-Dec-12 |
| 72-65 | PMV | 10-3-1-72-72-65 | x | 15-Dec-12 |
| 12-65 | Pharmacy | 11-2-5-12-12-65 | x | 12-Dec-12 |
| 3-65 | Pharmacy | 12-2-5-3-03-65 | x | 12-Dec-12 |
| 16-65 | Pharmacy | 13-2-3-16-16-65 | x | 08-Dec-12 |
| 25-64 | PMV | 13-2-5-25-25-64 | x | 10-Dec-12 |
| 2-65 | Pharmacy | 16-1-3-2-02-65 | x | 11-Dec-12 |
| 11-65 | Pharmacy | 16-1-3-11-11-65 | x | 10-Dec-12 |
| 26-64 | PMV | 16-1-3-26-26-64 | x | 12-Dec-12 |
| 1-67A | Pharmacy | 4-1-2-1-01-67A | x | 10-Dec-12 |
| 1-67A | Pharmacy | 7-2-4-1-01-67A | x | 13-Dec-12 |
| 27-7 | PMV | 2-3-5-27-27-07 | x | 11-Dec-12 |
| 53-8 | PMV | 4-1-2-53-53-08 | x | 18-Dec-12 |
| 21-08A | PMV | 5-1-2-21-21-08A | x | 14-Dec-12 |
| 49-8 | PMV | 5-1-2-49-49-08 | x | 14-Dec-12 |
| 15-8 | PMV | 6-2-1-15-15-8 | x | 17-Dec-12 |
| 26-67 | PMV | 6-2-1-26-26-67 | x | 14-Dec-12 |
| 21-08B | PMV | 10-3-1-21-21-08B | x | 13-Dec-12 |
| 3-8 | Pharmacy | 12-2-5-3-03-08 | x | 12-Dec-12 |
| 4-8 | Pharmacy | 12-2-5-4-04-08 | x | 12-Dec-12 |
| 24-8 | PMV | 12-2-5-24-24-8 | x | 10-Dec-12 |
| 1-8 | Pharmacy | 13-3-7-1-01-08 | x | 11-Dec-12 |
| 4-7 | Pharmacy | 14-1-3-4-04-07 | x | 10-Dec-12 |
| 25-7 | PMV | 14-1-3-25-25-07 | x | 14-Dec-12 |
| 7-7 | Pharmacy | 16-1-1-7-07-07 | x | 14-Dec-12 |
| 75-7 | PMV | 1-3-1-75-75-07 | x | 15-Dec-12 |
| 14-7 | Pharmacy | 6-2-3-14-14-7 | x | 10-Dec-12 |
| 21-07A | PMV | 1-3-1-21-21-07A | x | 13-Dec-12 |

| | | | | |
|---|---|---|---|---|
| 63-7 | PMV | 1-3-1-63-63-07 | x | 13-Dec-12 |
| 5-7 | PMV | 2-3-2-5-05-07 | x | 10-Dec-12 |
| 8-7 | PMV | 2-3-2-8-08-07 | x | 10-Dec-12 |
| 9-7 | Pharmacy | 4-1-2-9-09-07 | x | 11-Dec-12 |
| 2-7 | Pharmacy | 5-1-2-2-02-07 | x | 10-Dec-12 |
| 21-07B | PMV | 5-1-2-21-21-07B | x | 14-Dec-12 |
| 33-7 | PMV | 5-1-2-33-33-07 | x | 12-Dec-12 |
| 59-26 | PMV | 5-1-2-59-59-26 | x | 20-Dec-12 |
| 22-7 | PMV | 7-2-4-22-22-7 | x | 13-Dec-12 |
| 3-7 | PMV | 9-3-3-3-03-07 | x | 13-Dec-12 |
| 21-07C | PMV | 14-1-3-21-21-07C | x | 12-Dec-12 |
| 12-67c | Pharmacy | 5-2-3-12-12-67c | x | 08-Dec-12 |
| 0-35 | Publichealth fa | 5-2-1-0-0-35 | x | 20-Dec-12 |
| 0-35A | Publichealth fa | 9-2-1-0-0-35A | x | 21-Dec-12 |
| 39-67 | PMV | 3-2-4-39-39-67 | x | 12-Dec-12 |
| 77-34 | PMV | 1-3-1-77-77-34 | x | 18-Dec-12 |
| 1-34 | Pharmacy | 7-2-4-1-01-34 | x | 13-Dec-12 |
| 7-33 | Pharmacy | 16-1-3-7-07-33 | x | 11-Dec-12 |
| 7-67D | Pharmacy | 5-1-2-7-07-67D | x | 12-Dec-12 |
| 22-67d | Pharmacy | 5-1-2-22-22-67d | x | 04-Dec-12 |
| 28-67 | PMV | 2-3-5-28-28-67 | x | 11-Dec-12 |
| 37-67E | Pharmacy | 6-2-3-37-37-67E | x | 12-Dec-12 |
| 28-67 | PMV | 13-3-7-28-28-67 | x | 13-Dec-12 |
| 81-65 | Pharmacy | 1-3-1-81-81-65 | x | 18-Dec-12 |
| 7-67b | PMV | 2-3-2-7-07-67b | x | 10-Dec-12 |
| 19-64 | Pharmacy | 4-1-2-19-19-64 | x | 14-Dec-12 |
| 11-65 | Pharmacy | 5-1-2-11-11-65 | x | 12-Dec-12 |
| 0-65 | Publichealth fa | 5-2-1-0-0-65 | x | 20-Dec-12 |
| 1-67b | Pharmacy | 6-2-1-1-01-67b | x | 14-Dec-12 |
| 11-67H | Pharmacy | 6-2-1-11-11-67H | x | 18-Dec-12 |
| 14-67C | Pharmacy | 6-2-3-14-14-67C | x | 10-Dec-12 |
| 4-65B | Pharmacy | 7-2-4-4-04-65B | x | 12-Dec-12 |
| 0-65T | Publichealth fa | 9-2-1-0-0-65T | x | 21-Dec-12 |
| 21-65B | PMV | 10-3-1-21-21-65B | x | 13-Dec-12 |
| 72-64 | PMV | 10-3-1-72-72-64 | x | 15-Dec-12 |
| 16-64A | Pharmacy | 13-2-3-16-16-64A | x | 08-Dec-12 |
| 25-64B | PMV | 13-2-5-25-25-64B | x | 10-Dec-12 |
| 1-64A | Pharmacy | 14-1-1-1-01-64A | x | 13-Dec-12 |
| 4-64A | Pharmacy | 14-1-3-4-04-64A | x | 10-Dec-12 |
| 10-64 | Pharmacy | 14-1-3-10-10-64 | x | 13-Dec-12 |
| 11-64 | Pharmacy | 16-1-3-11-11-64 | x | 10-Dec-12 |
| 38-64 | PMV | 1-3-1-38-38-64 | x | 14-Dec-12 |
| 60-64 | PMV | 1-3-1-60-60-64 | x | 13-Dec-12 |
| 63-64 | PMV | 1-3-1-63-63-64 | x | 13-Dec-12 |
| 7-67a | PMV | 2-3-2-7-07-67a | x | 10-Dec-12 |
| 33-67a | PMV | 2-3-5-33-33-67a | x | 12-Dec-12 |
| 21-64 | PMV | 3-2-4-21-21-64 | x | 10-Dec-12 |
| 39-64 | PMV | 3-2-4-39-39-64 | x | 12-Dec-12 |
| 42-64 | PMV | 3-2-4-42-42-64 | x | 13-Dec-12 |
| 0-64 | Publichealth fa | 3-3-71-0-0-64 | x | 20-Dec-12 |

| | | | | |
|---|---|---|---|---|
| 4-64 | Pharmacy | 4-1-2-4-04-64 | x | 12-Dec-12 |
| 18-64 | Pharmacy | 4-1-2-18-18-64 | x | 14-Dec-12 |
| 35-64 | PMV | 4-1-2-35-35-64 | x | 13-Dec-12 |
| 53-65 | PMV | 4-1-2-53-53-65 | x | 18-Dec-12 |
| 0-65 | Publichealth fa | 5-1-2-0-0-65 | x | 21-Dec-12 |
| 7-64 | Pharmacy | 5-1-2-7-07-64 | x | 12-Dec-12 |
| 1-64 | Pharmacy | 6-2-1-1-01-64 | x | 14-Dec-12 |
| 14-67E | Pharmacy | 6-2-3-14-14-67E | x | 10-Dec-12 |
| 25-64 | PMV | 6-2-3-25-25-64 | x | 08-Dec-12 |
| 1-64 | Pharmacy | 7-2-4-1-01-64 | x | 13-Dec-12 |
| 27-64 | PMV | 7-2-4-27-27-64 | x | 12-Dec-12 |
| 0-64 | Publichealth fa | 7-3-5-0-0-64 | x | 20-Dec-12 |
| 0-64 | Publichealth fa | 8-2-1-0-00-64 | x | 20-Dec-12 |
| 17-64 | PMV | 8-2-4-17-17-64 | x | 13-Dec-12 |
| 23-64 | PMV | 8-2-4-23-23-64 | x | 13-Dec-12 |
| 44-64 | PMV | 8-2-4-44-44-64 | x | 12-Dec-12 |
| 0-64 | Publichealth fa | 9-2-1-0-0-64 | x | 21-Dec-12 |
| 8-64 | PMV | 9-3-5-8-08-64 | x | 11-Dec-12 |
| 20-64 | PMV | 9-3-5-20-20-64 | x | 11-Dec-12 |
| 31-64 | PMV | 9-3-5-31-31-64 | x | 12-Dec-12 |
| 1-64 | Pharmacy | 10-3-1-1-01-64 | x | 14-Dec-12 |
| 14-64 | Pharmacy | 10-3-1-14-14-64 | x | 18-Dec-12 |
| 21-64 | PMV | 10-3-1-21-21-64 | x | 13-Dec-12 |
| 43-64 | PMV | 10-3-1-43-43-64 | x | 18-Dec-12 |
| 53-64 | PMV | 10-3-1-53-53-64 | x | 18-Dec-12 |
| 78-64 | PMV | 10-3-1-78-78-64 | x | 13-Dec-12 |
| 14-64 | PMV | 11-3-7-14-14-64 | x | 11-Dec-12 |
| 4-64 | Pharmacy | 12-2-5-4-04-64 | x | 12-Dec-12 |
| 6-64 | Pharmacy | 12-3-7-6-06-64 | x | 11-Dec-12 |
| 16-64B | Pharmacy | 13-2-3-16-16-64B | x | 08-Dec-12 |
| 25-64 | PMV | 13-2-5-25-25-64 | x | 10-Dec-12 |
| 8-65 | Pharmacy | 13-3-7-8-08-65 | x | 11-Dec-12 |
| 1-64R | Pharmacy | 14-1-1-1-01-64R | x | 13-Dec-12 |
| 15-26 | PMV | 14-1-1-15-15-26 | x | 13-Dec-12 |
| 4-64 | Pharmacy | 14-1-3-4-4-64 | x | 10-Dec-12 |
| 4-64 | Pharmacy | 15-2-1-4-04-64 | x | 12-Dec-12 |
| 14-64 | Pharmacy | 15-2-1-14-14-64 | x | 17-Dec-12 |
| 30-64 | PMV | 15-2-3-30-30-64 | x | 10-Dec-12 |
| 2-64 | Pharmacy | 16-1-3-2-02-64 | x | 11-Dec-12 |
| 38-67 | PMV | 1-3-1-38-38-67 | x | 14-Dec-12 |
| 6-07B | Pharmacy | 3-2-6-6-06-07B | x | 19-Dec-12 |
| 19-67D | PMV | 4-1-2-19-19-67D | x | 14-Dec-12 |
| 20-67A | PMV | 7-2-4-20-20-67A | x | 10-Dec-12 |
| 26-67 | PMV | 16-1-3-26-26-67 | x | 12-Dec-12 |
| 9-67E | Pharmacy | 4-1-2-9-9-67E | x | 11-Dec-12 |
| 20-67C | PMV | 7-2-4-20-20-67C | x | 10-Dec-12 |
| 42-22 | PMV | 1-3-1-42-42-22 | x | 13-Dec-12 |
| 28-22 | PMV | 2-3-5-28-28-22 | x | 11-Dec-12 |
| 1-21 | Pharmacy | 2-3-6-1-01-21 | x | 10-Dec-12 |
| 11-22 | Pharmacy | 3-2-4-11-11-22 | x | 12-Dec-12 |

| | | | | |
|---|---|---|---|---|
| 6-22 | PMV | 3-22-6-6-06-22 | x | 19-Dec-12 |
| 9-22 | Pharmacy | 4-1-2-9-9-22 | x | 11-Dec-12 |
| 12-22 | Pharmacy | 4-1-2-12-12-22 | x | 14-Dec-12 |
| 53-22 | PMV | 4-1-2-53-53-22 | x | 18-Dec-12 |
| 8-22 | Pharmacy | 5-1-2-8-08-22 | x | 11-Dec-12 |
| 14-22 | Pharmacy | 6-2-3-14-14-22 | x | 10-Dec-12 |
| 37-22 | Pharmacy | 6-2-3-37-37-22 | x | 12-Dec-12 |
| 5-22 | Pharmacy | 10-3-1-5-05-22 | x | 13-Dec-12 |
| 12-22 | Pharmacy | 11-2-5-12-12-22 | x | 12-Dec-12 |
| 6-22 | Pharmacy | 12-3-7-6-06-22 | x | 11-Dec-12 |
| 16-22 | Pharmacy | 13-2-3-16-16-22 | x | 08-Dec-12 |
| 1-22 | Pharmacy | 14-1-1-1-01-22 | x | 13-Dec-12 |
| 15-22 | PMV | 14-1-1-15-15-22 | x | 13-Dec-12 |
| 1-22 | Pharmacy | 14-1-3-1-01-22 | x | 11-Dec-12 |
| 10-22 | Pharmacy | 15-2-3-10-10-22 | x | 11-Dec-12 |
| 11-22 | Pharmacy | 15-2-3-11-11-22 | x | 12-Dec-12 |
| 30-22 | PMV | 15-2-3-30-30-22 | x | 10-Dec-12 |
| 38-22 | Pharmacy | 15-2-3-38-38-22 | x | 13-Dec-12 |
| 2-22 | Pharmacy | 16-1-3-2-02-22 | x | 11-Dec-12 |
| 14-22 | PMV | 16-1-3-14-014-022 | x | 12-Dec-12 |
| 30-22 | PMV | 16-1-3-30-30-22 | x | 18-Dec-12 |
| 12-20 | Pharmacy | 1-3-1-12-12-20 | x | 13-Dec-12 |
| 13-20 | Pharmacy | 1-3-1-13-13-20 | x | 14-Dec-12 |
| 42-20 | PMV | 1-3-1-42-42-20 | x | 13-Dec-12 |
| 2-20 | Pharmacy | 3-2-1-2-02-20 | x | 10-Dec-12 |
| 11-20 | PMV | 3-2-4-11-11-20 | x | 12-Dec-12 |
| 21-20 | PMV | 3-2-4-21-21-20 | x | 10-Dec-12 |
| 39-20 | PMV | 3-2-4-39-39-20 | x | 12-Dec-12 |
| 6-20 | Pharmacy | 4-1-2-6-06-20 | x | 13-Dec-12 |
| 13-20 | PMV | 6-2-1-13-13-20 | x | 13-Dec-12 |
| 6-20 | Pharmacy | 6-2-3-6-06-20 | x | 11-Dec-12 |
| 25-20 | PMV | 7-2-4-25-25-20 | x | 13-Dec-12 |
| 1-21 | Pharmacy | 9-2-2-1-01-21 | x | 18-Dec-12 |
| 5-20 | Pharmacy | 10-3-1-5-05-20 | x | 13-Dec-12 |
| 71-21 | PMV | 10-3-1-71-71-21 | x | 15-Dec-12 |
| 12-20 | Pharmacy | 11-2-5-12-12-20 | x | 12-Dec-12 |
| 21-20 | PMV | 13-2-3-21-21-20 | x | 08-Dec-12 |
| 8-20 | Pharmacy | 13-3-7-8-08-20 | x | 11-Dec-12 |
| 28-20 | PMV | 13-3-7-28-28-20 | x | 13-Dec-12 |
| 10-20 | Pharmacy | 15-2-3-10-10-20 | x | 11-Dec-12 |
| 38-20 | Pharmacy | 15-2-3-38-38-20 | x | 13-Dec-12 |
| 7-20 | Pharmacy | 16-1-3-7-07-20 | x | 11-Dec-12 |
| 38-20 | Pharmacy | 16-1-3-38-38-20 | x | 14-Dec-12 |
| 21-67B | PMV | 10-3-1-21-21-67B | x | 13-Dec-12 |
| 8-67B | Pharmacy | 4-1-2-8-08-67B | x | 13-Dec-12 |
| 5-67B | Pharmacy | 11-2-5-5-05-67B | x | 12-Dec-12 |
| 21-67 | PMV | 14-1-3-21-21-67 | x | 12-Dec-12 |
| 0-35B | Publichealth fa | 9-2-1-0-0-35B | x | 21-Dec-12 |
| 3-33 | Pharmacy | 4-2-3-3-03-33 | x | 08-Dec-12 |
| 2-33 | Pharmacy | 5-1-2-2-02-33 | x | 10-Dec-12 |

| | | | | |
|---|---|---|---|---|
| 21-33 | PMV | 10-3-1-21-21-33 | x | 13-Dec-12 |
| 6-67A | Pharmacy | 3-2-6-6-06-67A | x | 19-Dec-12 |
| 31-67A | PMV | 9-3-5-31-31-67A | x | 12-Dec-12 |
| 28-21 | PMV | 2-3-5-28-28-21 | x | 11-Dec-12 |
| 1-22b | Pharmacy | 2-3-6-1-01-22b | x | 10-Dec-12 |
| 13-67 | PMV | 6-2-1-13-13-67 | x | 13-Dec-12 |
| 37-67B | Pharmacy | 6-2-3-37-37-67B | x | 12-Dec-12 |
| 16-20 | Pharmacy | 13-2-3-16-16-20 | x | 08-Dec-12 |
| 7-22 | PMV | 14-1-1-7-07-22 | x | 14-Dec-12 |
| 10-67B | Pharmacy | 15-2-3-10-10-67B | x | 11-Dec-12 |
| 30-67 | PMV | 15-2-3-30-30-67 | x | 10-Dec-12 |
| 80-21 | PMV | 1-3-1-80-80-21 | x | 18-Dec-12 |
| 1-22a | Pharmacy | 2-3-6-1-01-22a | x | 10-Dec-12 |
| 23-20 | Pharmacy | 4-1-2-23-23-20 | x | 10-Dec-12 |
| 3-21 | Pharmacy | 4-2-3-3-03-21 | x | 08-Dec-12 |
| 2-21 | Pharmacy | 5-1-2-2-02-21 | x | 10-Dec-12 |
| 7-21 | Pharmacy | 5-1-2-7-07-21 | x | 12-Dec-12 |
| 17-21 | Pharmacy | 5-1-2-17-17-21 | x | 18-Dec-12 |
| 7-21 | Pharmacy | 5-2-3-7-07-21 | x | 08-Dec-12 |
| 13-21 | PMV | 6-2-1-13-13-21 | x | 13-Dec-12 |
| 9-21 | Pharmacy | 6-2-3-9-09-21 | x | 11-Dec-12 |
| 14-21 | Pharmacy | 6-2-3-14-14-21 | x | 10-Dec-12 |
| 37-21 | Pharmacy | 6-2-3-37-37-21 | x | 12-Dec-12 |
| 31-21 | PMV | 9-3-5-31-31-21 | x | 12-Dec-12 |
| 14-21 | Pharmacy | 10-3-1-14-14-21 | x | 18-Dec-12 |
| 9-21 | Pharmacy | 13-2-5-9-09-21 | x | 10-Dec-12 |
| 3-21 | Pharmacy | 13-3-7-3-03-21 | x | 11-Dec-12 |
| 15-21 | PMV | 14-1-1-15-15-21 | x | 13-Dec-12 |
| 1-21 | Pharmacy | 14-1-3-1-01-21 | x | 11-Dec-12 |
| 9-21 | Pharmacy | 14-1-3-9-9-21 | x | 12-Dec-12 |
| 23-21 | Pharmacy | 14-1-3-23-23-21 | x | 12-Dec-12 |
| 35-21 | PMV | 14-1-3-35-35-21 | x | 14-Dec-12 |
| 28-22 | PMV | 16-1-1-28-28-22 | x | 14-Dec-12 |
| 27-31T | PMV | 16-1-3-27-27-31T | x | 12-Dec-12 |
| 30-21 | PMV | 16-1-3-30-30-21 | x | 18-Dec-12 |
| 38-24 | Pharmacy | 16-1-3-38-38-24 | x | 14-Dec-12 |
| 13-15 | Pharmacy | 1-3-1-13-13-15 | x | 14-Dec-12 |
| 21-15 | PMV | 1-3-1-21-21-15 | x | 13-Dec-12 |
| 9-14 | Pharmacy | 4-1-2-9-09-14 | x | 11-Dec-12 |
| 8-15 | Pharmacy | 5-1-2-8-08-15 | x | 11-Dec-12 |
| 17-15 | Pharmacy | 5-1-2-17-17-15 | x | 18-Dec-12 |
| 9-15 | Pharmacy | 6-2-3-9-09-15 | x | 11-Dec-12 |
| 12-15 | Pharmacy | 11-2-5-12-12-15 | x | 12-Dec-12 |
| 26-15 | PMV | 11-2-5-26-26-15 | x | 10-Dec-12 |
| 38-67C | Pharmacy | 15-2-3-38-38-67C | x | 13-Dec-12 |
| 74-67a | PMV | 1-3-1-74-74-67a | x | 14-Dec-12 |
| 8-67A | Pharmacy | 5-1-2-8-08-67A | x | 11-Dec-12 |
| 4-067C | Pharmacy | 14-1-3-4-04-067C | x | 10-Dec-12 |
| 13-14 | Pharmacy | 1-3-1-13-13-14 | x | 14-Dec-12 |
| 21-14 | PMV | 1-3-1-21-21-14 | x | 13-Dec-12 |

| | | | | |
|---|---|---|---|---|
| 42-14 | PMV | 1-3-1-42-42-14 | x | 13-Dec-12 |
| 1-14 | Pharmacy | 2-3-6-1-01-14 | x | 10-Dec-12 |
| 14-14 | PMV | 3-2-4-14-14-14 | x | 12-Dec-12 |
| 2-14 | Pharmacy | 5-1-2-2-02-14 | x | 10-Dec-12 |
| 57-14 | PMV | 5-1-2-57-57-14 | x | 14-Dec-12 |
| 11-14 | Pharmacy | 6-2-1-11-11-14 | x | 18-Dec-12 |
| 1-14 | Pharmacy | 7-2-4-1-01-14 | x | 14-Dec-12 |
| 4-14 | Pharmacy | 7-2-4-4-04-14 | x | 12-Dec-12 |
| 5-14 | Pharmacy | 8-2-4-5-05-14 | x | 11-Dec-12 |
| 2-14 | Pharmacy | 10-3-1-2-02-14 | x | 15-Dec-12 |
| 33-14 | PMV | 10-3-1-33-33-14 | x | 15-Dec-12 |
| 6-14 | Pharmacy | 11-2-5-6-06-14 | x | 21-Dec-12 |
| 12-14 | Pharmacy | 11-2-5-12-12-14 | x | 12-Dec-12 |
| 22-14 | PMV | 11-2-5-22-22-14 | x | 10-Dec-12 |
| 26-14 | PMV | 11-2-5-26-26-14 | x | 10-Dec-12 |
| 6-14 | Pharmacy | 12-3-7-6-06-14 | x | 11-Dec-12 |
| 28-14 | PMV | 13-3-7-28-28-14 | x | 13-Dec-12 |
| 33-14 | PMV | 13-3-7-33-33-14 | x | 14-Dec-12 |
| 1-14 | Pharmacy | 14-1-1-1-01-14 | x | 13-Dec-12 |
| 20-14 | Pharmacy | 14-1-1-20-20-14 | x | 20-Dec-12 |
| 1-14 | Pharmacy | 14-1-3-1-01-14 | x | 11-Dec-12 |
| 29-14 | Pharmacy | 14-1-3-29-29-14 | x | 13-Dec-12 |
| 35-14 | PMV | 14-1-3-35-35-14 | x | 14-Dec-12 |
| 7-14 | Pharmacy | 15-2-1-7-07-14 | x | 14-Dec-12 |
| 14-14 | PMV | 15-2-1-14-14-14 | x | 17-Dec-12 |
| 10-14 | Pharmacy | 15-2-3-10-10-14 | x | 11-Dec-12 |
| 11-14 | Pharmacy | 15-2-3-11-11-14 | x | 12-Dec-12 |
| 15-14 | Pharmacy | 15-2-3-15-15-14 | x | 10-Dec-12 |
| 28-14 | PMV | 15-2-3-28-28-14 | x | 08-Dec-12 |
| 38-14 | Pharmacy | 15-2-3-38-38-14 | x | 13-Dec-12 |
| 7-14 | Pharmacy | 16-1-3-7-07-14 | x | 11-Dec-12 |
| 38-14 | Pharmacy | 16-1-3-38-38-14 | x | 14-Dec-12 |
| 7-67B | Pharmacy | 5-1-2-7-07-67B | x | 12-Dec-12 |
| 12-39 | Pharmacy | 11-2-5-12-12-39 | x | 12-Dec-12 |
| 68-67 | PMV | 1-3-1-68-68-67 | x | 14-Dec-12 |
| 6-67C | Pharmacy | 3-2-6-6-06-67C | x | 19-Dec-12 |
| 9-67F | Pharmacy | 4-1-2-9-9-67F | x | 11-Dec-12 |
| 19-67B | Pharmacy | 4-1-2-19-19-67B | x | 14-Dec-12 |
| 57-67c | PMV | 5-1-2-57-57-67c | x | 14-Dec-12 |
| 31-67B | PMV | 9-3-5-31-31-67B | x | 12-Dec-12 |
| 1-38 | Pharmacy | 7-2-4-1-01-38 | x | 13-Dec-12 |
| 29-32 | Pharmacy | 14-1-3-29-29-32 | x | 13-Dec-12 |
| 1-54 | Pharmacy | 4-1-2-1-01-54 | x | 10-Dec-12 |
| 1-54 | Pharmacy | 7-2-4-1-01-54 | x | 13-Dec-12 |
| 2-24 | Pharmacy | 5-1-2-2-02-24 | x | 10-Dec-12 |
| 8-24 | Pharmacy | 5-1-2-8-08-24 | x | 11-Dec-12 |
| 17-24 | Pharmacy | 5-1-2-17-17-24 | x | 18-Dec-12 |
| 11-67I | Pharmacy | 6-2-1-11-11-67I | x | 18-Dec-12 |
| 6-67L | Pharmacy | 6-2-3-6-06-67L | x | 11-Dec-12 |
| 20-24 | PMV | 7-2-4-20-20-24 | x | 10-Dec-12 |

| | | | | |
|---|---|---|---|---|
| 12-67 | Pharmacy | 11-2-5-12-12-67 | x | 12-Dec-12 |
| 35-67 | PMV | 14-1-3-35-35-67 | x | 14-Dec-12 |
| 29-67E | Pharmacy | 15-2-1-29-29-67E | x | 14-Dec-12 |
| 21-63 | PMV | 10-3-1-21-21-63 | x | 13-Dec-12 |
| 1-24 | Pharmacy | 1-2-4-1-01-24 | x | 13-Dec-12 |
| 59-24 | PMV | 1-3-1-59-59-24 | x | 11-Dec-12 |
| 4-24 | Pharmacy | 4-1-2-4-04-24 | x | 12-Dec-12 |
| 9-24 | Pharmacy | 4-1-2-9-09-24 | x | 11-Dec-12 |
| 7-24 | Pharmacy | 5-2-3-7-07-24 | x | 08-Dec-12 |
| 11-24 | Pharmacy | 6-2-1-11-11-24 | x | 18-Dec-12 |
| 6-24 | Pharmacy | 6-2-3-6-06-24 | x | 11-Dec-12 |
| 5-24 | Pharmacy | 8-2-4-5-05-24 | x | 11-Dec-12 |
| 11-24 | Pharmacy | 11-2-5-11-11-24 | x | 12-Dec-12 |
| 12-24 | Pharmacy | 11-2-5-12-12-24 | x | 12-Dec-12 |
| 6-24 | Pharmacy | 12-3-7-6-06-24 | x | 11-Dec-12 |
| 7-24 | Pharmacy | 13-3-7-7-07-24 | x | 11-Dec-12 |
| 1-24 | Pharmacy | 14-1-3-1-01-24 | x | 11-Dec-12 |
| 29-24 | Pharmacy | 15-2-1-29-29-24 | x | 14-Dec-12 |
| 10-24 | Pharmacy | 15-2-3-10-10-24 | x | 11-Dec-12 |
| 11-24 | Pharmacy | 15-2-3-11-11-24 | x | 12-Dec-12 |
| 38-24 | Pharmacy | 15-2-3-38-38-24 | x | 13-Dec-12 |
| 28-24 | Pharmacy | 16-1-1-28-28-24 | x | 14-Dec-12 |
| 38-24 | Pharmacy | 16-1-3-38-38-24 | x | 14-Dec-12 |
| 33-1 | PMV | 2-3-5-33-33-01 | x | 12-Dec-12 |
| 9-1 | Pharmacy | 4-1-2-9-09-01 | x | 11-Dec-12 |
| 23-1 | Pharmacy | 4-1-2-23-23-01 | x | 10-Dec-12 |
| 22-1 | PMV | 7-2-4-22-22-1 | x | 13-Dec-12 |
| 1-1 | Pharmacy | 9-2-2-1-01-01 | x | 18-Dec-12 |
| 16-1 | Pharmacy | 13-2-3-16-16-01 | x | 08-Dec-12 |
| 15-1 | PMV | 14-1-1-15-15-01 | x | 13-Dec-12 |
| 4-1 | Pharmacy | 14-1-3-4-4-1 | x | 10-Dec-12 |
| 5-1 | Pharmacy | 14-1-3-5-05-01 | x | 11-Dec-12 |
| 38-1 | Pharmacy | 15-2-3-38-38-01 | x | 13-Dec-12 |
| 1-30 | Pharmacy | 4-1-2-1-01-30 | x | 10-Dec-12 |
| 7-30 | Pharmacy | 5-2-3-7-07-30 | x | 08-Dec-12 |
| 1-30 | Pharmacy | 13-3-7-1-01-30 | x | 11-Dec-12 |
| 27-67b | PMV | 2-3-5-27-27-67b | x | 11-Dec-12 |
| 9-67D | Pharmacy | 4-1-2-9-9-67D | x | 11-Dec-12 |
| 2-67C | PMV | 5-1-2-2-02-67C | x | 10-Dec-12 |
| 10-67 | Pharmacy | 1-3-1-10-10-067 | x | 14-Dec-12 |
| 12-63 | Pharmacy | 1-3-1-12-12-63 | x | 13-Dec-12 |
| 13-63 | Pharmacy | 1-3-1-13-13-63 | x | 14-Dec-12 |
| 36-63 | PMV | 1-3-1-36-36-63 | x | 18-Dec-12 |
| 59-63 | PMV | 1-3-1-59-59-63 | x | 11-Dec-12 |
| 68-63 | Pharmacy | 1-3-1-68-68-63 | x | 14-Dec-12 |
| 69-63 | PMV | 1-3-1-69-69-63 | x | 14-Dec-12 |
| 70-63 | PMV | 1-3-1-70-70-63 | x | 15-Dec-12 |
| 76-63 | PMV | 1-3-1-76-76-63 | x | 18-Dec-12 |
| 80-63 | PMV | 1-3-1-80-80-63 | x | 18-Dec-12 |
| 81-63 | Pharmacy | 1-3-1-81-81-63 | x | 18-Dec-12 |

| | | | | |
|---|---|---|---|---|
| 82-63 | PMV | 1-3-1-82-82-63 | x | 18-Dec-12 |
| 84-63 | PMV | 1-3-1-84-84-63 | x | 18-Dec-12 |
| 5-63 | PMV | 2-3-2-5-05-63 | x | 10-Dec-12 |
| 7-63 | PMV | 2-3-2-7-07-63 | x | 10-Dec-12 |
| 8-63 | PMV | 2-3-2-8-08-63 | x | 10-Dec-12 |
| 3-63 | Pharmacy | 2-3-4-3-3-63 | x | 10-Dec-12 |
| 4-63 | Pharmacy | 2-3-5-4-04-63 | x | 11-Dec-12 |
| 25-63 | PMV | 2-3-5-25-25-63 | x | 11-Dec-12 |
| 1-63 | Pharmacy | 2-3-6-1-01-63 | x | 10-Dec-12 |
| 2-63 | Pharmacy | 3-2-4-2-02-63 | x | 10-Dec-12 |
| 7-63 | Pharmacy | 3-2-4-7-07-63 | x | 11-Dec-12 |
| 11-63 | Pharmacy | 3-2-4-11-11-63 | x | 12-Dec-12 |
| 15-63 | PMV | 3-2-4-15-15-63 | x | 11-Dec-12 |
| 21-63 | PMV | 3-2-4-21-21-63 | x | 10-Dec-12 |
| 26-63 | PMV | 3-2-4-26-26-63 | x | 14-Dec-12 |
| 43-63 | PMV | 3-2-4-43-43-63 | x | 13-Dec-12 |
| 6-63 | PMV | 3-2-6-6-06-63 | x | 19-Dec-12 |
| 4-63 | Pharmacy | 4-1-2-4-04-63 | x | 12-Dec-12 |
| 5-63 | Pharmacy | 4-1-2-5-5-63 | x | 13-Dec-12 |
| 8-63 | Pharmacy | 4-1-2-8-08-63 | x | 13-Dec-12 |
| 9-63 | Pharmacy | 4-1-2-9-9-63 | x | 11-Dec-12 |
| 15-63 | Pharmacy | 4-1-2-15-15-63 | x | 12-Dec-12 |
| 19-63 | Pharmacy | 4-1-2-19-19-63 | x | 14-Dec-12 |
| 23-63 | Pharmacy | 4-1-2-23-23-63 | x | 10-Dec-12 |
| 35-63 | PMV | 4-1-2-35-35-63 | x | 13-Dec-12 |
| 36-63 | PMV | 4-1-2-36-36-63 | x | 12-Dec-12 |
| 53-63 | PMV | 4-1-2-53-53-63 | x | 18-Dec-12 |
| 2-63 | Pharmacy | 5-1-2-2-02-63 | x | 10-Dec-12 |
| 7-63 | Pharmacy | 5-1-2-7-07-63 | x | 12-Dec-12 |
| 8-63 | Pharmacy | 5-1-2-8-08-63 | x | 11-Dec-12 |
| 10-63 | Pharmacy | 5-1-2-10-10-63 | x | 14-Dec-12 |
| 21-63 | Pharmacy | 5-1-2-21-21-63 | x | 14-Dec-12 |
| 22-63 | Pharmacy | 5-1-2-22-22-63 | x | 04-Dec-12 |
| 30-63 | PMV | 5-1-2-30-30-63 | x | 10-Dec-12 |
| 31-63 | PMV | 5-1-2-31-31-63 | x | 12-Dec-12 |
| 33-63 | PMV | 5-1-2-33-33-63 | x | 12-Dec-12 |
| 46-63 | PMV | 5-1-2-46-46-63 | x | 14-Dec-12 |
| 49-63 | PMV | 5-1-2-49-49-63 | x | 14-Dec-12 |
| 56-63 | PMV | 5-1-2-56-56-63 | x | 13-Dec-12 |
| 59-63A | Pharmacy | 5-1-2-59-59-63A | x | 13-Dec-12 |
| 59-63B | Pharmacy | 5-1-2-59-59-63B | x | 20-Dec-12 |
| 7-63 | Pharmacy | 5-2-3-7-07-63 | x | 08-Dec-12 |
| 12-63 | Pharmacy | 5-2-3-12-12-63 | x | 08-Dec-12 |
| 1-63 | Pharmacy | 6-2-1-1-01-63 | x | 14-Dec-12 |
| 13-63 | PMV | 6-2-1-13-13-63 | x | 13-Dec-12 |
| 15-63 | PMV | 6-2-1-15-15-63 | x | 17-Dec-12 |
| 28-63 | Pharmacy | 6-2-1-28-28-63 | x | 14-Dec-12 |
| 1-63 | Pharmacy | 6-2-3-1-01-63 | x | 12-Dec-12 |
| 14-63 | Pharmacy | 6-2-3-14-14-63 | x | 10-Dec-12 |
| 25-63 | PMV | 6-2-3-25-25-63 | x | 08-Dec-12 |

| | | | | |
|---|---|---|---|---|
| 37-63 | PMV | 6-2-3-37-37-63 | x | 12-Dec-12 |
| 1-63 | Pharmacy | 7-2-4-1-01-63 | x | 13-Dec-12 |
| 22-63 | PMV | 7-2-4-22-22-63 | x | 13-Dec-12 |
| 8-63 | Pharmacy | 8-2-1-8-08-63 | x | 10-Dec-12 |
| 5-63A | Pharmacy | 8-2-4-5-05-63A | x | 11-Dec-12 |
| 5-063B | Pharmacy | 8-2-4-5-05-063B | x | 13-Dec-12 |
| 10-63 | Pharmacy | 8-2-4-10-10-63 | x | 12-Dec-12 |
| 16-63 | PMV | 8-2-4-16-16-63 | x | 13-Dec-12 |
| 17-63 | PMV | 8-2-4-17-17-63 | x | 13-Dec-12 |
| 23-63 | PMV | 8-2-4-23-23-63 | x | 13-Dec-12 |
| 33-63 | PMV | 8-2-4-33-33-63 | x | 13-Dec-12 |
| 44-63 | PMV | 8-2-4-44-44-63 | x | 12-Dec-12 |
| 1-63 | Pharmacy | 9-2-2-1-01-63 | x | 18-Dec-12 |
| 7-63 | PMV | 9-2-2-7-07-63 | x | 18-Dec-12 |
| 11-63 | PMV | 9-3-5-11-11-63 | x | 11-Dec-12 |
| 16-63 | PMV | 9-3-5-16-16-63 | x | 12-Dec-12 |
| 19-63 | PMV | 9-3-5-19-19-63 | x | 11-Dec-12 |
| 1-63 | Pharmacy | 10-3-1-1-01-63 | x | 14-Dec-12 |
| 5-63 | Pharmacy | 10-3-1-5-05-63 | x | 13-Dec-12 |
| 14-63 | Pharmacy | 10-3-1-14-14-63 | x | 18-Dec-12 |
| 15-63 | Pharmacy | 10-3-1-15-15-63 | x | 10-Dec-12 |
| 20-63 | PMV | 10-3-1-20-20-63 | x | 13-Dec-12 |
| 25-63 | PMV | 10-3-1-25-25-63 | x | 15-Dec-12 |
| 33-63 | Pharmacy | 10-3-1-33-33-63 | x | 15-Dec-12 |
| 37-63 | PMV | 10-3-1-37-37-63 | x | 11-Dec-12 |
| 53-63 | PMV | 10-3-1-53-53-63 | x | 18-Dec-12 |
| 65-63 | PMV | 10-3-1-65-65-63 | x | 14-Dec-12 |
| 79-63 | PMV | 10-3-1-79-79-63 | x | 11-Dec-12 |
| 1-63 | Pharmacy | 11-2-5-1-01-63 | x | 13-Dec-12 |
| 5-63 | Pharmacy | 11-2-5-5-05-63 | x | 12-Dec-12 |
| 22-63 | PMV | 11-2-5-22-22-63 | x | 10-Dec-12 |
| 26-63 | PMV | 11-2-5-26-26-63 | x | 10-Dec-12 |
| 14-63 | PMV | 11-3-7-14-14-63 | x | 11-Dec-12 |
| 15-63 | PMV | 11-3-7-15-15-63 | x | 11-Dec-12 |
| 24-63 | PMV | 11-3-7-24-24-63 | x | 11-Dec-12 |
| 27-63 | PMV | 11-3-7-27-27-63 | x | 11-Dec-12 |
| 2-63 | Pharmacy | 12-2-5-02-02-63 | x | 12-Dec-12 |
| 10-63 | Pharmacy | 12-2-5-10-10-63 | x | 12-Dec-12 |
| 16-63 | PMV | 12-2-5-16-16-63 | x | 10-Dec-12 |
| 24-63 | PMV | 12-2-5-24-24-63 | x | 10-Dec-12 |
| 6-63 | Pharmacy | 12-3-7-6-06-63 | x | 11-Dec-12 |
| 23-63 | PMV | 12-3-7-23-23-63 | x | 11-Dec-12 |
| 16-63 | Pharmacy | 13-2-3-16-16-63 | x | 08-Dec-12 |
| 21-63 | PMV | 13-2-3-21-21-63 | x | 08-Dec-12 |
| 25-63 | PMV | 13-2-5-25-25-63 | x | 10-Dec-12 |
| 1-63 | Pharmacy | 13-3-7-1-01-63 | x | 11-Dec-12 |
| 4-63 | Pharmacy | 13-3-7-4-04-63 | x | 11-Dec-12 |
| 7-63 | Pharmacy | 13-3-7-7-07-63 | x | 11-Dec-12 |
| 8-63 | Pharmacy | 13-3-7-8-08-63 | x | 11-Dec-12 |
| 28-63 | PMV | 13-3-7-28-28-63 | x | 13-Dec-12 |

| | | | | |
|---|---|---|---|---|
| 31-63 | PMV | 13-3-7-31-31-63 | x | 14-Dec-12 |
| 32-63 | PMV | 13-3-7-32-32-63 | x | 14-Dec-12 |
| 33-63 | PMV | 13-3-7-33-33-63 | x | 14-Dec-12 |
| 1-63 | Pharmacy | 14-1-1-1-01-63 | x | 13-Dec-12 |
| 5-63 | PMV | 14-1-1-5-05-63 | x | 14-Dec-12 |
| 6-63 | PMV | 14-1-1-6-06-63 | x | 14-Dec-12 |
| 7-63 | PMV | 14-1-1-7-07-63 | x | 14-Dec-12 |
| 15-63 | PMV | 14-1-1-15-15-63 | x | 13-Dec-12 |
| 16-63 | PMV | 14-1-1-16-16-63 | x | 13-Dec-12 |
| 18-63 | PMV | 14-1-1-18-18-63 | x | 14-Dec-12 |
| 20-63 | Pharmacy | 14-1-1-20-20-63 | x | 20-Dec-12 |
| 5-63 | Pharmacy | 14-1-3-5-05-63 | x | 11-Dec-12 |
| 9-63 | Pharmacy | 14-1-3-9-9-63 | x | 12-Dec-12 |
| 21-63 | PMV | 14-1-3-21-21-63 | x | 12-Dec-12 |
| 23-63 | PMV | 14-1-3-23-23-63 | x | 12-Dec-12 |
| 25-63 | Pharmacy | 14-1-3-25-25-63 | x | 14-Dec-12 |
| 32-63 | Pharmacy | 14-1-3-32-32-63 | x | 14-Dec-12 |
| 35-63 | Pharmacy | 14-1-3-35-35-63 | x | 14-Dec-12 |
| 4-63 | Pharmacy | 15-2-1-4-04-63 | x | 12-Dec-12 |
| 5-63 | Pharmacy | 15-2-1-5-05-63 | x | 14-Dec-12 |
| 7-63 | Pharmacy | 15-2-1-7-07-63 | x | 14-Dec-12 |
| 14-63 | PMV | 15-2-1-14-14-63 | x | 17-Dec-12 |
| 10-63 | Pharmacy | 15-2-3-10-10-63 | x | 11-Dec-12 |
| 15-63 | Pharmacy | 15-2-3-15-15-63 | x | 10-Dec-12 |
| 20-63 | PMV | 15-2-3-20-20-63 | x | 12-Dec-12 |
| 24-63 | PMV | 15-2-3-24-24-63 | x | 11-Dec-12 |
| 26-63 | PMV | 15-2-3-26-26-63 | x | 08-Dec-12 |
| 30-63 | PMV | 15-2-3-30-30-63 | x | 10-Dec-12 |
| 34-63 | PMV | 15-2-3-34-34-63 | x | 08-Dec-12 |
| 38-63 | Pharmacy | 15-2-3-38-38-63 | x | 13-Dec-12 |
| 17-63 | PMV | 16-1-1-17-17-63 | x | 14-Dec-12 |
| 7-63 | Pharmacy | 16-1-3-7-07-63 | x | 11-Dec-12 |
| 11-63 | Pharmacy | 16-1-3-11-11-63 | x | 10-Dec-12 |
| 14-63 | PMV | 16-1-3-14-014-063 | x | 12-Dec-12 |
| 26-63 | PMV | 16-1-3-26-26-63 | x | 12-Dec-12 |
| 30-63 | PMV | 16-1-3-30-30-63 | x | 18-Dec-12 |
| 31-63 | Pharmacy | 16-1-3-31-31-63 | x | 18-Dec-12 |
| 38-63 | Pharmacy | 16-1-3-38-38-63 | x | 14-Dec-12 |
| 53-67E | PMV | 4-1-2-53-53-67E | x | 18-Dec-12 |
| 21-67b | Pharmacy | 5-1-2-2-21-67b | x | 10-Dec-12 |
| 8-67B | Pharmacy | 5-1-2-8-08-67B | x | 11-Dec-12 |
| 14-67 | Pharmacy | 10-3-1-14-14-67 | x | 18-Dec-12 |
| 22-67 | PMV | 11-2-5-22-22-67 | x | 10-Dec-12 |
| 4-67A | Pharmacy | 15-2-1-4-04-67A | x | 12-Dec-12 |
| 4-67 | Pharmacy | 4-1-2-4-04-67 | x | 12-Dec-12 |
| 9-67G | Pharmacy | 4-1-2-9-9-67G | x | 11-Dec-12 |
| 53-67F | PMV | 4-1-2-53-53-67F | x | 18-Dec-12 |
| 8-67E | Pharmacy | 5-1-2-8-08-67E | x | 11-Dec-12 |
| 12-67b | Pharmacy | 5-2-3-12-12-67b | x | 08-Dec-12 |
| 17-67B | PMV | 15-2-1-17-17-67B | x | 14-Dec-12 |

| | | | | |
|---|---|---|---|---|
| 59-67a | PMV | 1-3-1-59-59-67a | x | 11-Dec-12 |
| 7-67 | Pharmacy | 13-3-7-7-07-67 | x | 11-Dec-12 |
| 21-67C | Pharmacy | 3-2-4-21-21-67C | x | 10-Dec-12 |
| 11-67E | Pharmacy | 6-2-1-11-11-67E | x | 18-Dec-12 |
| 28-67C | Pharmacy | 6-2-1-28-28-67C | x | 14-Dec-12 |
| 53-67G | PMV | 4-1-2-53-53-67G | x | 18-Dec-12 |
| 8-67F | Pharmacy | 5-1-2-8-08-67F | x | 11-Dec-12 |
| 3-67B | Pharmacy | 13-3-7-3-03-67B | x | 11-Dec-12 |
| 18-67 | PMV | 14-1-1-18-18-67 | x | 14-Dec-12 |
| 38-67F | Pharmacy | 15-2-3-38-38-67F | x | 13-Dec-12 |
| 8-67A | Pharmacy | 8-2-1-8-08-67A | x | 10-Dec-12 |
| 2-67A | PMV | 5-1-2-2-02-67A | x | 10-Dec-12 |
| 1-67 | Pharmacy | 14-1-3-1-01-067 | x | 11-Dec-12 |
| 5-67C | Pharmacy | 11-2-5-5-05-67C | x | 12-Dec-12 |
| 29-67D | Pharmacy | 15-2-1-29-29-67D | x | 14-Dec-12 |
| 26-67B | PMV | 15-2-3-26-26-67B | x | 08-Dec-12 |
| 9-67H | Pharmacy | 4-1-2-9-09-67H | x | 11-Dec-12 |
| 12-67a | Pharmacy | 5-2-3-12-12-67a | x | 08-Dec-12 |
| 1-67B | Pharmacy | 6-2-3-1-01-67B | x | 12-Dec-12 |
| 6-67C | Pharmacy | 6-2-3-6-06-67C | x | 11-Dec-12 |
| 10-57 | Pharmacy | 1-3-1-10-10-57 | x | 14-Dec-12 |
| 13-57 | Pharmacy | 1-3-1-13-13-57 | x | 14-Dec-12 |
| 38-57 | PMV | 1-3-1-38-38-57 | x | 14-Dec-12 |
| 42-57 | PMV | 1-3-1-42-42-57 | x | 13-Dec-12 |
| 59-57 | PMV | 1-3-1-59-59-57 | x | 11-Dec-12 |
| 75-57 | PMV | 1-3-1-75-75-57 | x | 15-Dec-12 |
| 83-57 | PMV | 1-3-1-83-83-57 | x | 18-Dec-12 |
| 84-57 | PMV | 1-3-1-84-84-57 | x | 18-Dec-12 |
| 5-57 | PMV | 2-3-2-5-5-57 | x | 10-Dec-12 |
| 30-57 | PMV | 2-3-5-30-30-57 | x | 11-Dec-12 |
| 2-54 | Pharmacy | 3-2-4-2-02-54 | x | 10-Dec-12 |
| 7-57 | Pharmacy | 3-2-4-7-07-57 | x | 11-Dec-12 |
| 8-57 | Pharmacy | 3-2-4-8-08-57 | x | 11-Dec-12 |
| 13-57 | PMV | 3-2-4-13-13-57 | x | 13-Dec-12 |
| 15-57 | PMV | 3-2-4-15-15-57 | x | 11-Dec-12 |
| 21-57 | PMV | 3-2-4-21-21-57 | x | 10-Dec-12 |
| 6-57 | Pharmacy | 3-2-6-6-06-57 | x | 19-Dec-12 |
| 15-57 | Pharmacy | 4-1-2-15-15-57 | x | 12-Dec-12 |
| 19-57 | Pharmacy | 4-1-2-19-19-57 | x | 14-Dec-12 |
| 36-57 | PMV | 4-1-2-36-36-57 | x | 12-Dec-12 |
| 17-57 | Pharmacy | 5-1-2-17-17-57 | x | 18-Dec-12 |
| 57-57 | PMV | 5-1-2-57-57-57 | x | 14-Dec-12 |
| 59-57 | Pharmacy | 5-1-2-59-59-57 | x | 13-Dec-12 |
| 7-57 | Pharmacy | 5-2-3-7-07-57 | x | 08-Dec-12 |
| 12-57 | Pharmacy | 5-2-3-12-12-57 | x | 08-Dec-12 |
| 15-57 | PMV | 6-2-1-15-15-57 | x | 17-Dec-12 |
| 28-57 | Pharmacy | 6-2-1-28-28-57 | x | 14-Dec-12 |
| 1-57 | Pharmacy | 6-2-3-1-01-57 | x | 12-Dec-12 |
| 25-57 | PMV | 6-2-3-25-25-57 | x | 08-Dec-12 |
| 31-57 | PMV | 6-2-3-31-31-57 | x | 10-Dec-12 |

| | | | | |
|---|---|---|---|---|
| 37-57 | Pharmacy | 6-2-3-37-37-57 | x | 12-Dec-12 |
| 4-57 | Pharmacy | 7-2-4-4-04-57 | x | 12-Dec-12 |
| 22-57B | PMV | 7-2-4-22-22-57B | x | 13-Dec-12 |
| 25-27 | PMV | 7-2-4-25-25-27 | x | 13-Dec-12 |
| 16-57 | PMV | 8-2-4-16-16-57 | x | 13-Dec-12 |
| 1-57 | Pharmacy | 9-2-2-1-01-57 | x | 18-Dec-12 |
| 11-57 | PMV | 9-3-5-19-11-57 | x | 11-Dec-12 |
| 20-57 | PMV | 9-3-5-20-20-57 | x | 11-Dec-12 |
| 5-1 | Pharmacy | 10-3-1-5-05-01 | x | 13-Dec-12 |
| 8-57 | Pharmacy | 10-3-1-8-08-57 | x | 18-Dec-12 |
| 14-57 | Pharmacy | 10-3-1-14-14-57 | x | 18-Dec-12 |
| 20-57 | PMV | 10-3-1-20-20-57 | x | 13-Dec-12 |
| 33-57 | Pharmacy | 10-3-1-33-33-57 | x | 15-Dec-12 |
| 66-1 | PMV | 10-3-1-66-66-01 | x | 14-Dec-12 |
| 71-57 | Pharmacy | 10-3-1-71-71-57 | x | 15-Dec-12 |
| 78-1 | PMV | 10-3-1-78-78-01 | x | 13-Dec-12 |
| 79-57 | PMV | 10-3-1-79-79-57 | x | 11-Dec-12 |
| 1-57 | Pharmacy | 11-2-5-1-01-57 | x | 13-Dec-12 |
| 5-57 | Pharmacy | 11-2-5-5-05-57 | x | 12-Dec-12 |
| 11-57 | Pharmacy | 11-2-5-11-11-57 | x | 12-Dec-12 |
| 22-57 | PMV | 11-2-5-22-22-57 | x | 10-Dec-12 |
| 26-57 | PMV | 11-2-5-26-26-57 | x | 10-Dec-12 |
| 18-57 | PMV | 12-3-7-18-18-57 | x | 11-Dec-12 |
| 16-57 | Pharmacy | 13-2-3-16-16-57 | x | 08-Dec-12 |
| 9-57 | Pharmacy | 13-2-5-9-09-57 | x | 10-Dec-12 |
| 1-57 | Pharmacy | 13-3-7-1-01-57 | x | 11-Dec-12 |
| 33-57 | PMV | 13-3-7-33-33-57 | x | 14-Dec-12 |
| 6-57 | PMV | 14-1-1-6-06-57 | x | 14-Dec-12 |
| 20-57 | Pharmacy | 14-1-1-20-20-57 | x | 20-Dec-12 |
| 1-57 | Pharmacy | 14-1-3-1-01-57 | x | 11-Dec-12 |
| 4-27 | Pharmacy | 14-1-3-4-4-27 | x | 10-Dec-12 |
| 5-37 | Pharmacy | 14-1-3-5-05-37 | x | 11-Dec-12 |
| 29-57 | Pharmacy | 14-1-3-29-29-57 | x | 13-Dec-12 |
| 35-57 | Pharmacy | 14-1-3-35-35-57 | x | 14-Dec-12 |
| 5-57 | Pharmacy | 15-2-1-5-05-57 | x | 14-Dec-12 |
| 7-57 | Pharmacy | 15-2-1-7-07-57 | x | 14-Dec-12 |
| 19-57 | PMV | 15-2-1-19-19-57 | x | 18-Dec-12 |
| 29-57 | PMV | 15-2-1-29-29-57 | x | 14-Dec-12 |
| 10-57 | Pharmacy | 15-2-3-10-10-57 | x | 11-Dec-12 |
| 11-57 | Pharmacy | 15-2-3-11-11-57 | x | 12-Dec-12 |
| 20-57 | PMV | 15-2-3-20-20-57 | x | 12-Dec-12 |
| 30-57 | PMV | 15-2-3-30-30-57 | x | 10-Dec-12 |
| 38-57 | PMV | 15-2-3-38-38-57 | x | 13-Dec-12 |
| 7-57 | PMV | 16-1-1-7-07-57 | x | 14-Dec-12 |
| 2-57 | Pharmacy | 16-1-3-2-02-57 | x | 11-Dec-12 |
| 6-57 | Pharmacy | 16-1-3-6-06-057 | x | 12-Dec-12 |
| 27-57 | PMV | 16-1-3-27-27-57 | x | 12-Dec-12 |
| 30-57 | PMV | 16-1-3-30-30-57 | x | 18-Dec-12 |
| 31-57 | Pharmacy | 16-1-3-31-31-57 | x | 18-Dec-12 |
| 38-57 | Pharmacy | 16-1-3-38-38-57 | x | 14-Dec-12 |

| | | | | |
|---|---|---|---|---|
| 10-67 | Pharmacy | 1-3-1-10-10-067 | x | 14-Dec-12 |
| 7-67F | Pharmacy | 5-1-2-7-7-67F | x | 12-Dec-12 |
| 11-67B | Pharmacy | 6-2-1-11-11-67B | x | 18-Dec-12 |
| 1-67D | Pharmacy | 11-2-5-1-01-67D | x | 13-Dec-12 |
| 1-67 | Pharmacy | 13-3-7-1-01-67 | x | 11-Dec-12 |
| 33-67 | PMV | 13-3-7-33-33-67 | x | 14-Dec-12 |
| 4-67 | Pharmacy | 1-3-1-4-04-67 | x | 10-Dec-12 |
| 6-67B | PMV | 9-2-2-6-06-67B | x | 18-Dec-12 |
| 21-67A | PMV | 10-3-1-21-21-67A | x | 13-Dec-12 |
| 24-67b | PMV | 11-3-7-24-24-67b | x | 11-Dec-12 |
| 16-67 | PMV | 12-2-5-16-16-67 | x | 10-Dec-12 |
| 39-67 | PMV | 1-3-1-39-39-67 | x | 14-Dec-12 |
| 74-67c | PMV | 1-3-1-74-74-67c | x | 14-Dec-12 |
| 6-67D | PMV | 3-2-6-6-06-67D | x | 19-Dec-12 |
| 28-67E | Pharmacy | 6-2-1-28-28-67E | x | 14-Dec-12 |
| 22-67 | PMV | 11-2-5-22-22-67 | x | 10-Dec-12 |
| 3-67 | Pharmacy | 12-2-5-3-3-67 | x | 12-Dec-12 |
| 9-67 | Pharmacy | 13-3-7-9-09-67 | x | 11-Dec-12 |
| 4-67B | Pharmacy | 15-2-1-4-04-67B | x | 12-Dec-12 |
| 5-67e | Pharmacy | 15-2-1-5-05-67e | x | 14-Dec-12 |
| 21-30 | PMV | 3-2-4-21-21-30 | x | 10-Dec-12 |
| 15-28 | Pharmacy | 4-1-2-15-15-28 | x | 12-Dec-12 |
| 53-28 | PMV | 4-1-2-53-53-28 | x | 18-Dec-12 |
| 22-28 | Pharmacy | 5-1-2-22-22-28 | x | 04-Dec-12 |
| 11-28 | Pharmacy | 6-2-1-11-11-28 | x | 18-Dec-12 |
| 9-28 | Pharmacy | 14-1-3-9-9-28 | x | 12-Dec-12 |
| 29-28 | PMV | 15-2-1-29-29-28 | x | 14-Dec-12 |
| 28-28 | PMV | 15-2-3-28-28-28 | x | 08-Dec-12 |
| 7-28 | Pharmacy | 16-1-3-7-07-28 | x | 11-Dec-12 |
| 11-28 | Pharmacy | 16-1-3-11-11-28 | x | 10-Dec-12 |
| 38-28 | PMV | 16-1-3-38-38-28 | x | 14-Dec-12 |
| 59-67A | Pharmacy | 5-1-2-59-59-67A | x | 13-Dec-12 |
| 11-67A | Pharmacy | 6-2-1-11-11-67A | x | 18-Dec-12 |
| 37-67A | Pharmacy | 6-2-3-37-37-67A | x | 12-Dec-12 |
| 11-67C | Pharmacy | 6-2-1-11-11-67C | x | 18-Dec-12 |
| 14-067A | Pharmacy | 16-1-3-4-014-067A | x | 12-Dec-12 |
| 8-12 | Pharmacy | 4-1-2-8-08-12 | x | 13-Dec-12 |
| 17-12 | Pharmacy | 5-1-2-17-17-12 | x | 18-Dec-12 |
| 2-12 | Pharmacy | 9-2-2-2-02-12 | x | 18-Dec-12 |
| 16-12 | PMV | 9-3-5-16-16-12 | x | 12-Dec-12 |
| 28-12 | PMV | 16-1-1-28-28-12 | x | 14-Dec-12 |
| 60-67A | PMV | 1-3-1-60-60-67A | x | 11-Dec-12 |
| 60-67D | PMV | 1-3-1-60-60-67D | x | 13-Dec-12 |
| 7-67A | Pharmacy | 5-1-2-7-07-67A | x | 12-Dec-12 |
| 55-67A | PMV | 5-1-2-55-55-67A | x | 13-Dec-12 |
| 59-67B | Pharmacy | 5-1-2-59-59-67B | x | 13-Dec-12 |
| 15-67 | PMV | 14-1-1-15-15-67 | x | 13-Dec-12 |
| 9-67 | Pharmacy | 14-1-3-9-9-67 | x | 12-Dec-12 |
| 38-67G | Pharmacy | 15-2-3-38-38-67G | x | 13-Dec-12 |
| 7-67 | Pharmacy | 16-1-3-7-07-67 | x | 11-Dec-12 |

| | | | | |
|---|---|---|---|---|
| 25-67 | PMV | 13-2-5-25-25-67 | x | 10-Dec-12 |
| 21-67W | PMV | 3-2-4-21-21-67W | x | 10-Dec-12 |
| 9-48 | Pharmacy | 4-1-2-9-09-48 | x | 11-Dec-12 |
| 19-67A | Pharmacy | 4-1-2-19-19-67A | x | 14-Dec-12 |
| 55-67B | PMV | 5-1-2-55-55-67B | x | 13-Dec-12 |
| 0-67 | Publichealth fa | 5-2-1-0-0-67 | x | 20-Dec-12 |
| 37-67D | PMV | 6-2-3-37-37-67D | x | 12-Dec-12 |
| 1-48 | Pharmacy | 9-2-2-1-01-48 | x | 18-Dec-12 |
| 8-48 | PMV | 9-3-6-8-08-48 | x | 13-Dec-12 |
| 14-67C | PMV | 15-2-1-14-14-67C | x | 17-Dec-12 |
| 11-48 | Pharmacy | 16-1-3-11-11-48 | x | 10-Dec-12 |
| 59-67C | PMV | 5-1-2-59-59-67C | x | 13-Dec-12 |
| 53-67D | PMV | 4-1-2-53-53-67D | x | 18-Dec-12 |
| 7-67 | Pharmacy | 5-1-2-7-7-67 | x | 12-Dec-12 |
| 14-67A | PMV | 6-2-3-14-14-67A | x | 10-Dec-12 |
| 14-67D | PMV | 15-2-1-14-14-67D | x | 17-Dec-12 |
| 4-56 | Pharmacy | 2-3-5-4-04-56 | x | 11-Dec-12 |
| 11-67G | Pharmacy | 6-2-1-11-11-67G | x | 18-Dec-12 |
| 8-56 | PMV | 9-3-5-8-8-56 | x | 11-Dec-12 |
| 8-56 | Pharmacy | 13-3-7-8-08-56 | x | 11-Dec-12 |
| 11-67 | Pharmacy | 16-1-3-11-11-67 | x | 10-Dec-12 |
| 8-67J | Pharmacy | 5-1-2-8-08-67J | x | 11-Dec-12 |
| 38-67D | PMV | 15-2-3-38-38-67D | x | 13-Dec-12 |
| 14-67D | Pharmacy | 6-2-3-14-14-67D | x | 10-Dec-12 |
| 60-51B | PMV | 1-3-1-60-60-51B | x | 13-Dec-12 |
| 6-50 | PMV | 2-3-2-6-06-50 | x | 10-Dec-12 |
| 7-50 | PMV | 2-3-2-7-07-50 | x | 10-Dec-12 |
| 1-50 | Pharmacy | 2-3-6-1-01-50 | x | 10-Dec-12 |
| 13-50 | PMV | 3-2-4-13-13-50 | x | 13-Dec-12 |
| 21-50 | PMV | 3-2-4-21-21-50 | x | 10-Dec-12 |
| 9-50 | Pharmacy | 4-1-2-9-09-50 | x | 11-Dec-12 |
| 19-50 | Pharmacy | 4-1-2-19-19-50 | x | 14-Dec-12 |
| 1-50 | Pharmacy | 6-2-3-1-01-50 | x | 12-Dec-12 |
| 11-50 | Pharmacy | 6-2-1-11-11-50 | x | 18-Dec-12 |
| 15-50 | PMV | 6-2-1-15-15-50 | x | 17-Dec-12 |
| 28-50 | PMV | 6-2-1-28-28-50 | x | 14-Dec-12 |
| 31-50 | PMV | 6-2-3-31-31-50 | x | 10-Dec-12 |
| 36-49 | PMV | 6-2-3-36-36-49 | x | 08-Dec-12 |
| 16-50 | PMV | 8-2-4-16-16-50 | x | 13-Dec-12 |
| 44-50 | PMV | 8-2-4-44-44-50 | x | 12-Dec-12 |
| 1-50 | Pharmacy | 9-2-2-1-01-50 | x | 18-Dec-12 |
| 10-50 | PMV | 9-2-2-10-10-50 | x | 18-Dec-12 |
| 11-50 | PMV | 9-2-2-11-11-50 | x | 18-Dec-12 |
| 16-50 | PMV | 9-3-5-16-16-50 | x | 12-Dec-12 |
| 19-50B | PMV | 9-3-5-19-19-50B | x | 11-Dec-12 |
| 8-50 | PMV | 9-3-6-8-08-50 | x | 13-Dec-12 |
| 6-49 | PMV | 14-1-1-6-06-49 | x | 14-Dec-12 |
| 15-63 | PMV | 14-1-1-15-15-63 | x | 13-Dec-12 |
| 13-49 | PMV | 14-1-3-10-13-49 | x | 13-Dec-12 |
| 5-50 | Pharmacy | 14-1-3-5-05-50 | x | 11-Dec-12 |

| | | | | |
|---|---|---|---|---|
| 9-50 | Pharmacy | 14-1-3-9-9-50 | x | 12-Dec-12 |
| 29-50 | PMV | 15-2-1-29-29-50 | x | 14-Dec-12 |
| 10-50 | Pharmacy | 15-2-3-10-10-50 | x | 11-Dec-12 |
| 20-50 | PMV | 15-2-3-20-20-50 | x | 12-Dec-12 |
| 28-50 | PMV | 16-1-1-28-28-50 | x | 14-Dec-12 |
| 11-50 | PMV | 16-1-3-11-11-50 | x | 10-Dec-12 |
| 2-50 | Pharmacy | 16-1-3-2-02-50 | x | 11-Dec-12 |
| 31-50 | PMV | 16-1-3-31-31-50 | x | 18-Dec-12 |
| 38-50 | PMV | 16-1-3-38-38-50 | x | 14-Dec-12 |
| 4-60 | Pharmacy | 4-1-2-4-04-60 | x | 12-Dec-12 |
| 19-60 | PMV | 4-1-2-19-19-60 | x | 14-Dec-12 |
| 22-67e | PMV | 5-1-2-22-22-67e | x | 04-Dec-12 |
| 30-60 | PMV | 15-2-3-30-30-60 | x | 10-Dec-12 |
| 9-37 | Pharmacy | 4-1-2-9-9-37 | x | 11-Dec-12 |
| 4-37 | Pharmacy | 7-2-4-4-04-37 | x | 12-Dec-12 |
| 0-37 | Publichealth fa | 9-2-1-0-0-37 | x | 21-Dec-12 |
| 1-37 | Pharmacy | 11-2-5-1-01-37 | x | 13-Dec-12 |
| 4-37 | Pharmacy | 12-2-5-4-04-37 | x | 12-Dec-12 |
| 6-67B | Pharmacy | 6-2-3-6-06-67B | x | 11-Dec-12 |
| 53-67A | PMV | 4-1-2-53-53-67A | x | 18-Dec-12 |
| 21-67B | PMV | 3-2-4-21-21-67B | x | 10-Dec-12 |
| 11-67 | Pharmacy | 5-1-2-11-11-67 | x | 12-Dec-12 |
| 32-67d | PMV | 13-3-7-32-32-67d | x | 14-Dec-12 |
| 5-60 | Pharmacy | 15-2-1-5-05-60 | x | 14-Dec-12 |
| 60-67C | PMV | 1-3-1-60-60-67C | x | 11-Dec-12 |
| 27-67a | PMV | 2-3-5-27-27-67a | x | 11-Dec-12 |
| 15-67 | PMV | 6-2-1-15-15-67 | x | 17-Dec-12 |
| 1-17 | Pharmacy | 9-2-2-1-01-17 | x | 18-Dec-12 |
| 7-17a | PMV | 9-2-2-7-07-17a | x | 18-Dec-12 |
| 10-17b | PMV | 9-2-2-10-10-17b | x | 18-Dec-12 |
| 20-17 | PMV | 9-3-5-20-20-17 | x | 11-Dec-12 |
| 8-17B | Pharmacy | 9-3-6-8-08-17B | x | 13-Dec-12 |
| 15-17b | PMV | 14-1-1-15-15-17b | x | 13-Dec-12 |
| 1-17B | Pharmacy | 14-1-3-1-01-17B | x | 11-Dec-12 |
| 23-17 | PMV | 14-1-3-23-23-17 | x | 12-Dec-12 |
| 35-17b | PMV | 14-1-3-35-35-17b | x | 14-Dec-12 |
| 38-67E | PMV | 15-2-3-38-38-67E | x | 13-Dec-12 |
| 28-17B | PMV | 16-1-1-28-28-17B | x | 14-Dec-12 |
| 11-17 | Pharmacy | 16-1-3-11-11-17 | x | 10-Dec-12 |
| 31-17B | PMV | 16-1-3-31-31-17B | x | 18-Dec-12 |
| 10-67b | PMV | 9-2-2-10-10-67b | x | 18-Dec-12 |
| 28-67c | PMV | 15-2-3-28-28-67c | x | 08-Dec-12 |
| 29-67a | PMV | 15-2-1-29-29-67a | x | 14-Dec-12 |
| 4-58 | Pharmacy | 1-3-1-4-04-58 | x | 10-Dec-12 |
| 13-58 | Pharmacy | 1-3-1-13-13-58 | x | 14-Dec-12 |
| 60-58 | PMV | 1-3-1-60-60-58 | x | 13-Dec-12 |
| 12-57 | Pharmacy | 4-1-2-12-12-57 | x | 14-Dec-12 |
| 23-57 | Pharmacy | 4-1-2-23-23-57 | x | 10-Dec-12 |
| 38-58 | PMV | 4-1-2-38-38-58 | x | 14-Dec-12 |
| 11-58 | Pharmacy | 6-2-1-11-11-58 | x | 18-Dec-12 |

| | | | | |
|---|---|---|---|---|
| 15-58 | PMV | 6-2-1-15-15-58 | x | 17-Dec-12 |
| 28-58 | PMV | 6-2-1-28-28-58 | x | 14-Dec-12 |
| 9-58 | Pharmacy | 6-2-3-9-09-58 | x | 11-Dec-12 |
| 25-58 | PMV | 6-2-3-25-25-58 | x | 08-Dec-12 |
| 37-58 | PMV | 6-2-3-37-37-58 | x | 12-Dec-12 |
| 16-58 | PMV | 8-2-4-16-16-58 | x | 13-Dec-12 |
| 23-58 | PMV | 8-2-4-23-23-58 | x | 13-Dec-12 |
| 33-58 | PMV | 8-2-4-33-33-58 | x | 13-Dec-12 |
| 1-58 | Pharmacy | 9-2-2-1-01-58 | x | 18-Dec-12 |
| 7-58 | PMV | 9-2-2-7-07-58 | x | 18-Dec-12 |
| 19-58 | PMV | 9-3-5-19-19-58 | x | 11-Dec-12 |
| 20-58 | PMV | 9-3-5-20-20-58 | x | 11-Dec-12 |
| 8-58 | PMV | 9-3-6-8-08-58 | x | 13-Dec-12 |
| 1-1 | Pharmacy | 10-3-1-1-01-01 | x | 14-Dec-12 |
| 32-58 | PMV | 13-3-7-32-32-58 | x | 14-Dec-12 |
| 15-58 | PMV | 14-1-1-15-15-58 | x | 13-Dec-12 |
| 1-57 | Pharmacy | 14-1-3-1-01-57 | x | 11-Dec-12 |
| 5-58 | Pharmacy | 14-1-3-5-05-58 | x | 11-Dec-12 |
| 9-58 | Pharmacy | 14-1-3-9-9-58 | x | 12-Dec-12 |
| 23-53 | PMV | 14-1-3-23-23-53 | x | 12-Dec-12 |
| 19-58 | PMV | 15-2-1-19-19-58 | x | 18-Dec-12 |
| 29-58 | Pharmacy | 15-2-1-29-29-58 | x | 14-Dec-12 |
| 10-58 | Pharmacy | 15-2-3-10-10-58 | x | 11-Dec-12 |
| 38-58 | Pharmacy | 15-2-3-38-38-58 | x | 13-Dec-12 |
| 28-58 | Pharmacy | 16-1-1-28-28-58 | x | 14-Dec-12 |
| 7-57 | Pharmacy | 16-1-3-7-07-57 | x | 11-Dec-12 |
| 11-57 | Pharmacy | 16-1-3-11-11-57 | x | 10-Dec-12 |
| 14-58 | Pharmacy | 16-1-3-4-014-058 | x | 12-Dec-12 |
| 31-58 | Pharmacy | 16-1-3-31-31-58 | x | 18-Dec-12 |
| 38-58 | Pharmacy | 16-1-3-38-38-58 | x | 14-Dec-12 |
| 4-67 | PMV | 1-3-1-4-04-67 | x | 10-Dec-12 |
| 8-67L | Pharmacy | 5-1-2-8-8-67L | x | 11-Dec-12 |
| 11-67D | Pharmacy | 6-2-1-11-11-67D | x | 18-Dec-12 |
| 14-67D | Pharmacy | 6-2-3-14-14-67D | x | 10-Dec-12 |
| 38-67 | PMV | 16-1-3-38-38-67 | x | 14-Dec-12 |
| 9-67B | PMV | 6-2-3-9-09-67B | x | 11-Dec-12 |
| 34-67A | PMV | 4-2-3-34-34-67A | x | 06-Feb-13 |
| 10-46 | Pharmacy | 3-1-3-10-10-46 | x | 06-Feb-13 |
| 21-46 | Pharmacy | 3-1-3-21-21-46 | x | 07-Feb-13 |
| 43-11 | PMV | 4-1-2-43-43-11 | x | 15-Dec-12 |
| 1-46 | Pharmacy | 4-2-3-1-1-46 | x | 05-Feb-13 |
| 4-46 | PMV | 11-3-7-4-4-46 | x | 06-Feb-13 |
| 29-46 | PMV | 11-3-7-29-29-46 | x | 06-Feb-13 |
| 2-46 | Pharmacy | 12-2-1-2-2-46 | x | 06-Feb-13 |
| 27-46 | Pharmacy | 12-2-1-27-27-46 | x | 06-Feb-13 |
| 28-44 | PMV | 12-2-1-28-28-44 | x | 05-Feb-13 |
| 22-46 | PMV | 14-2-5-22-22-46 | x | 07-Feb-13 |
| 53-46 | PMV | 14-3-1-53-53-46 | x | 07-Feb-13 |
| 17-46 | PMV | 15-2-4-17-17-46 | x | 07-Feb-13 |
| 23-67 | PMV | 15-2-4-23-23-67 | x | 07-Feb-13 |

| | | | | |
|---|---|---|---|---|
| 72-46 | PMV | 16-3-1-72-72-46 | x | 09-Feb-13 |
| 83-46 | Pharmacy | 16-3-1-83-83-46 | x | 05-Feb-13 |
| 34-44 | Pharmacy | 3-1-3-34-34-44 | x | 05-Feb-13 |
| 9-44 | Pharmacy | 11-3-7-9-9-44 | x | 05-Feb-13 |
| 33-46 | PMV | 11-3-7-33-33-46 | x | 06-Feb-13 |
| 17-67 | PMV | 15-2-4-17-17-67 | x | 07-Feb-13 |
| 30-52 | Pharmacy | 3-1-3-30-30-52 | x | 09-Feb-13 |
| 27-44 | PMV | 4-2-3-27-27-44 | x | 08-Feb-13 |
| 5-45 | PMV | 9-1-1-5-5-45 | x | 07-Feb-13 |
| 20-45 | PMV | 11-3-5-20-20-45 | x | 06-Feb-13 |
| 33-45 | PMV | 11-3-7-33-33-45 | x | 06-Feb-13 |
| 6-45 | PMV | 12-2-6-6-6-45 | x | 06-Feb-13 |
| 17-45 | PMV | 15-2-4-17-17-45 | x | 07-Feb-13 |
| 42-45 | PMV | 15-2-4-42-42-45 | x | 05-Feb-13 |
| 5-45 | PMV | 16-3-2-5-5-45 | x | 08-Feb-13 |
| 51-45 | PMV | 17-1-2-51-51-45 | x | 05-Feb-13 |
| 27-44 | PMV | 3-1-3-27-27-44 | x | 08-Feb-13 |
| 20-44 | PMV | 4-2-3-20-20-44 | x | 06-Feb-13 |
| 26-44 | PMV | 4-2-3-26-26-44 | x | 06-Feb-13 |
| 37-45 | PMV | 4-2-3-37-37-45 | x | 06-Feb-13 |
| 1-45 | Pharmacy | 11-3-7-1-1-45 | x | 05-Feb-13 |
| 28-44 | PMV | 11-3-7-28-28-44 | x | 05-Feb-13 |
| 31-45 | PMV | 11-3-7-31-31-45 | x | 07-Feb-13 |
| 28-45 | PMV | 12-2-1-28-28-45 | x | 05-Feb-13 |
| 22-45 | PMV | 14-2-5-22-22-45 | x | 07-Feb-13 |
| 14-46 | Pharmacy | 14-3-1-14-14-46 | x | 05-Jan-13 |
| 11-44 | Pharmacy | 15-2-4-11-11-44 | x | 05-Feb-13 |
| 14-44 | PMV | 15-2-4-14-14-44 | x | 06-Feb-13 |
| 17-44 | PMV | 15-2-4-17-17-44 | x | 07-Feb-13 |
| 34-44 | PMV | 15-2-4-34-34-44 | x | 08-Feb-13 |
| 36-44 | PMV | 15-2-4-36-36-44 | x | 06-Feb-13 |
| 39-44 | PMV | 15-2-4-39-39-44 | x | 05-Feb-13 |
| 42-44 | PMV | 15-2-4-42-42-44 | x | 05-Feb-13 |
| 69-44 | PMV | 16-3-1-69-69-44 | x | 09-Feb-13 |
| 72-44 | PMV | 16-3-1-72-72-44 | x | 09-Feb-13 |
| 5-44 | PMV | 16-3-2-5-5-44 | x | 08-Feb-13 |
| 2-67 | PMV | 17-1-3-2-2-67 | x | 06-Feb-13 |
| 10-3 | Pharmacy | 3-1-3-10-10-3 | x | 06-Feb-13 |
| 32-3 | Pharmacy | 3-1-3-32-32-3 | x | 08-Feb-13 |
| 10-3 | Pharmacy | 4-2-1-10-10-3 | x | 07-Feb-13 |
| 6-3 | PMV | 9-1-1-6-6-3 | x | 07-Feb-13 |
| 29-3 | Pharmacy | 9-1-2-29-29-3 | x | 05-Feb-13 |
| 5-3 | Pharmacy | 12-2-6-5-5-3 | x | 09-Feb-13 |
| 2-3 | Pharmacy | 14-2-5-2-2-3 | x | 06-Feb-13 |
| 8-3 | Pharmacy | 15-2-4-8-8-3 | x | 06-Feb-13 |
| 74-67 | PMV | 16-3-1-74-74-67 | x | 09-Feb-13 |
| 36-3 | PMV | 17-1-2-36-36-3 | x | 08-Feb-13 |
| 34-05B | Pharmacy | 3-1-3-34-34-05B | x | 05-Feb-13 |
| 18-5 | Pharmacy | 9-1-2-18-18-5 | x | 05-Feb-13 |
| 11-5 | Pharmacy | 15-2-4-11-11-5 | x | 05-Feb-13 |

| | | | | |
|---|---|---|---|---|
| 19-5 | Pharmacy | 17-1-2-19-19-5 | x | 08-Jan-13 |
| 1-11 | Pharmacy | 11-3-7-1-1-11 | x | 05-Feb-13 |
| 2-11 | Pharmacy | 14-2-5-2-2-11 | x | 06-Feb-13 |
| 21-11 | Pharmacy | 3-1-3-21-21-11 | x | 07-Feb-13 |
| 6-11 | PMV | 9-1-1-6-6-11 | x | 07-Feb-13 |
| 10-11 | Pharmacy | 12-2-1-10-10-11 | x | 04-Feb-13 |
| 59-11 | Pharmacy | 17-1-2-59-59-11 | x | 07-Feb-13 |
| 10-67A | PMV | 16-3-1-10-10-67A | x | 06-Feb-13 |
| 28-18 | PMV | 4-2-3-28-28-18 | x | 06-Feb-13 |
| 38-18 | Pharmacy | 4-2-3-38-38-18 | x | 05-Feb-13 |
| 9-25 | Pharmacy | 3-1-3-9-9-25 | x | 09-Feb-13 |
| 10-25 | Pharmacy | 3-1-3-10-10-25 | x | 06-Feb-13 |
| 11-25 | Pharmacy | 3-1-3-11-11-25 | x | 07-Feb-13 |
| 21-25 | Pharmacy | 3-1-3-21-21-25 | x | 07-Feb-13 |
| 33-25 | Pharmacy | 3-1-3-33-33-25 | x | 07-Feb-13 |
| 34-25 | Pharmacy | 3-1-3-34-34-25 | x | 05-Feb-13 |
| 3-25 | Pharmacy | 3-2-3-3-3-25 | x | 08-Feb-13 |
| 10-25 | Pharmacy | 4-2-1-10-10-25 | x | 07-Feb-13 |
| 8-25 | Pharmacy | 4-2-3-8-8-25 | x | 05-Feb-13 |
| 16-25 | Pharmacy | 4-2-3-16-16-25 | x | 06-Feb-13 |
| 20-25 | PMV | 4-2-3-20-20-25 | x | 06-Feb-13 |
| 31-25 | PMV | 4-2-3-31-31-25 | x | 05-Feb-13 |
| 38-25 | Pharmacy | 4-2-3-38-38-25 | x | 05-Feb-13 |
| 16-25 | Pharmacy | 9-1-1-16-16-25 | x | 06-Feb-13 |
| 28-25 | PMV | 11-3-7-28-28-25 | x | 05-Feb-13 |
| 1-28B | Pharmacy | 12-2-1-1-1-28B | x | 05-Feb-13 |
| 10-25 | Pharmacy | 12-2-1-10-10-25 | x | 05-Feb-13 |
| 14-25 | PMV | 12-2-1-14-14-25 | x | 06-Feb-13 |
| 1-25 | Pharmacy | 12-2-6-1-1-25 | x | 08-Feb-13 |
| 5-25 | Pharmacy | 12-2-6-5-5-25 | x | 09-Feb-13 |
| 8-25 | Pharmacy | 14-3-1-8-8-25 | x | 05-Jan-13 |
| 31-25 | PMV | 14-3-1-31-31-25 | x | 07-Feb-13 |
| 62-25 | Pharmacy | 14-3-1-62-62-25 | x | 08-Feb-13 |
| 1-25 | Pharmacy | 14-3-2-1-1-25 | x | 06-Jan-13 |
| 8-25 | Pharmacy | 15-2-4-8-8-25 | x | 06-Feb-13 |
| 14-25 | PMV | 15-2-4-14-14-25 | x | 06-Feb-13 |
| 25-25 | PMV | 15-2-4-25-25-25 | x | 07-Feb-13 |
| 74-25 | PMV | 16-3-1-74-74-25 | x | 09-Feb-13 |
| 76-25 | PMV | 16-3-1-76-76-25 | x | 09-Feb-13 |
| 36-25 | PMV | 17-1-2-36-36-25 | x | 08-Feb-13 |
| 57-25 | PMV | 17-1-2-57-57-25 | x | 06-Feb-13 |
| 2-25 | PMV | 17-1-3-2-2-25 | x | 06-Feb-13 |
| 3-25 | Pharmacy | 17-1-3-3-3-25 | x | 06-Feb-13 |
| 14-26 | Pharmacy | 3-1-3-14-14-26 | x | 05-Feb-13 |
| 20-26 | Pharmacy | 9-1-2-20-20-26 | x | 05-Jan-13 |
| 4-26 | PMV | 11-3-7-4-4-26 | x | 06-Feb-13 |
| 72-27 | PMV | 16-3-1-72-72-27 | x | 09-Feb-13 |
| 5-26 | PMV | 16-3-3-5-5-26 | x | 09-Feb-13 |
| 7-26 | Pharmacy | 3-1-3-7-7-26 | x | 05-Feb-13 |
| 10-26 | Pharmacy | 3-1-3-10-10-26 | x | 06-Feb-13 |

| | | | | |
|---|---|---|---|---|
| 27-26 | PMV | 3-1-3-27-27-26 | x | 08-Feb-13 |
| 31-26 | Pharmacy | 3-1-3-31-31-26 | x | 08-Feb-13 |
| 10-0 | Pharmacy | 4-2-1-10-10-0 | x | 07-Feb-13 |
| 1-26 | Pharmacy | 4-2-3-1-1-26 | x | 05-Feb-13 |
| 26-26 | PMV | 4-2-3-26-26-26 | x | 06-Feb-13 |
| 27-26 | PMV | 4-2-3-27-27-26 | x | 08-Feb-13 |
| 28-26 | PMV | 4-2-3-28-28-26 | x | 06-Feb-13 |
| 37-26 | PMV | 4-2-3-37-37-26 | x | 06-Feb-13 |
| 44-26 | PMV | 5-1-2-44-44-26 | x | 15-Dec-12 |
| 51-26 | PMV | 5-1-2-51-51-26 | x | 15-Dec-12 |
| 28-26 | Pharmacy | 9-1-2-28-28-26 | x | 05-Feb-13 |
| 20-26 | PMV | 11-3-5-20-20-26 | x | 06-Feb-13 |
| 25-26 | PMV | 11-3-7-25-25-26 | x | 08-Feb-13 |
| 10-26 | Pharmacy | 12-2-1-10-10-26 | x | 04-Feb-13 |
| 6-26 | PMV | 12-2-6-6-6-26 | x | 06-Feb-13 |
| 2-27 | Pharmacy | 14-2-5-2-2-27 | x | 06-Feb-13 |
| 6-27 | Pharmacy | 14-2-5-6-6-27 | x | 06-Feb-13 |
| 25-27 | Pharmacy | 14-2-5-25-25-27 | x | 06-Feb-13 |
| 10-25 | Pharmacy | 14-3-1-10-10-25 | x | 05-Jan-13 |
| 21-26 | Pharmacy | 14-3-1-21-21-26 | x | 09-Feb-13 |
| 59-27 | Pharmacy | 14-3-1-59-59-27 | x | 08-Feb-13 |
| 34-26 | PMV | 15-2-4-34-34-26 | x | 08-Feb-13 |
| 5-25 | PMV | 16-3-2-5-5-25 | x | 08-Feb-13 |
| 33-25 | PMV | 17-1-2-33-33-25 | x | 05-Feb-13 |
| 60-25 | PMV | 17-1-2-60-60-25 | x | 06-Feb-13 |
| 10-27 | Pharmacy | 3-1-3-10-10-27 | x | 06-Feb-13 |
| 34-27 | Pharmacy | 3-1-3-34-34-27 | x | 05-Feb-13 |
| 10-27 | Pharmacy | 4-2-1-10-10-27 | x | 07-Feb-13 |
| 27-27 | PMV | 4-2-3-27-27-27 | x | 08-Feb-13 |
| 28-25 | PMV | 4-2-3-28-28-25 | x | 06-Feb-13 |
| 42-27 | PMV | 5-1-2-42-42-27 | x | 15-Dec-12 |
| 7-27 | PMV | 9-1-1-7-7-27 | x | 06-Feb-13 |
| 20-27 | PMV | 11-3-5-20-20-27 | x | 06-Feb-13 |
| 9-27 | Pharmacy | 11-3-7-9-9-27 | x | 05-Feb-13 |
| 17-27 | PMV | 12-2-1-17-17-27 | x | 06-Feb-13 |
| 43-25 | PMV | 4-1-2-43-43-25 | x | 15-Dec-12 |
| 6-27 | PMV | 9-1-1-6-6-27 | x | 07-Feb-13 |
| 25-27 | PMV | 11-3-7-25-25-27 | x | 09-Feb-13 |
| 2-25 | Pharmacy | 12-2-1-2-2-25 | x | 06-Feb-13 |
| 14-27 | Pharmacy | 14-3-1-14-14-27 | x | 05-Jan-13 |
| 5-27 | PMV | 16-3-2-5-5-27 | x | 08-Feb-13 |
| 10-67 | Pharmacy | 4-2-1-10-10-67 | x | 07-Feb-13 |
| 8-67 | Pharmacy | 4-2-3-8-8-67 | x | 05-Feb-13 |
| 1-67 | Pharmacy | 12-2-1-1-1-67 | x | 05-Feb-13 |
| 10-68 | Pharmacy | 12-2-1-10-10-68 | x | 04-Feb-13 |
| 6-67B | Pharmacy | 14-2-5-6-6-67B | x | 06-Feb-13 |
| 1-67B | Pharmacy | 14-3-2-1-1-67B | x | 06-Jan-13 |
| 7-31 | Pharmacy | 15-2-4-7-7-31 | x | 06-Feb-13 |
| 42-31 | PMV | 5-1-2-42-42-31 | x | 15-Dec-12 |
| 21-31 | Pharmacy | 14-3-1-21-21-31 | x | 09-Feb-13 |

| | | | | | |
|---|---|---|---|---|---|
| 6-31 | PMV | 9-1-1-6-6-31 | x | | 07-Feb-13 |
| 28-31 | PMV | 11-3-7-28-28-31 | x | | 05-Feb-13 |
| 2-31 | Pharmacy | 12-2-1-2-2-31 | x | | 06-Feb-13 |
| 14-57 | Pharmacy | 14-3-1-14-14-57 | x | | 05-Jan-13 |
| 36-67B | PMV | 15-2-4-36-36-67B | x | | 06-Feb-13 |
| 75-52 | PMV | 16-3-1-75-75-52 | x | | 06-Feb-13 |
| 19-52 | Pharmacy | 17-1-2-19-19-52 | x | | 08-Jan-13 |
| 45-67B | PMV | 4-1-2-45-45-67B | x | OP | 15-Dec-12 |
| 42-67B | PMV | 5-1-2-42-42-67B | x | OP | 15-Dec-12 |
| 6-52 | PMV | 9-1-1-6-6-52 | x | OP | 07-Feb-13 |
| 14-52 | Pharmacy | 14-3-1-14-14-52 | x | OP | 05-Jan-13 |
| 7-67C | Pharmacy | 15-2-4-7-7-67C | x | OP | 06-Feb-13 |
| 36-67A | PMV | 15-2-4-36-36-67A | x | OP | 06-Feb-13 |
| 5-52C | PMV | 16-3-2-5-5-52C | x | OP | 08-Feb-13 |
| 10-52B | Pharmacy | 3-1-3-10-10-52B | x | NP | 06-Feb-13 |
| 30-52 | Pharmacy | 3-1-3-30-30-52 | x | NP | 09-Feb-13 |
| 27-52A | PMV | 4-2-3-27-27-52A | x | NP | 08-Feb-13 |
| 28-52 | PMV | 4-2-3-28-28-52 | x | NP | 06-Feb-13 |
| 34-46 | PMV | 4-2-3-34-34-46 | x | NP | 06-Feb-13 |
| 44-67 | PMV | 5-1-2-44-44-67 | x | NP | 15-Dec-12 |
| 7-52 | PMV | 9-1-1-7-7-52 | x | NP | 06-Feb-13 |
| 29-52 | Pharmacy | 9-1-2-29-29-52 | x | NP | 05-Feb-13 |
| 4-52 | PMV | 11-3-7-4-4-52 | x | NP | 06-Feb-13 |
| 27-52 | Pharmacy | 12-2-1-27-27-52 | x | NP | 06-Feb-13 |
| 5-52A | PMV | 16-3-2-5-5-52A | x | NP | 08-Feb-13 |
| 83-52 | Pharmacy | 16-3-1-83-83-52 | x | | 05-Feb-13 |
| 7-52 | Pharmacy | 3-1-3-7-7-52 | x | OP | 05-Feb-13 |
| 21-52 | Pharmacy | 3-1-3-21-21-52 | x | OP | 07-Feb-13 |
| 34-52 | Pharmacy | 3-1-3-34-34-52 | x | OP | 05-Feb-13 |
| 13-52 | Pharmacy | 4-1-2-13-13-52 | x | OP | 15-Dec-12 |
| 26-52 | PMV | 4-2-3-26-26-52 | x | OP | 06-Feb-13 |
| 34-52 | PMV | 4-2-3-34-34-52 | x | OP | 06-Feb-13 |
| 16-52 | Pharmacy | 9-1-1-16-16-52 | x | OP | 06-Feb-13 |
| 11-52 | PMV | 9-1-1-11-11-52 | x | OP | 06-Feb-13 |
| 29-52 | PMV | 11-3-7-29-29-52 | x | OP | 06-Feb-13 |
| 9-52 | Pharmacy | 11-3-7-9-9-52 | x | OP | 05-Feb-13 |
| 12-52 | PMV | 11-3-7-12-12-52 | x | OP | 06-Feb-13 |
| 10-52 | Pharmacy | 14-3-1-10-10-52 | x | OP | 05-Jan-13 |
| 21-52 | Pharmacy | 14-3-1-21-21-52 | x | OP | 09-Feb-13 |
| 27-52 | PMV | 15-2-4-27-27-52 | x | OP | 07-Feb-13 |
| 44-52 | PMV | 15-2-4-44-44-52 | x | OP | 05-Feb-13 |
| 69-52 | PMV | 16-3-1-69-69-52 | x | OP | 09-Feb-13 |
| 5-52B | PMV | 16-3-2-5-5-52B | x | OP | 08-Feb-13 |
| 15-52 | Pharmacy | 17-1-2-15-15-52 | x | OP | 07-Feb-13 |
| 10-52A | Pharmacy | 3-1-3-10-10-52A | x | NP | 06-Feb-13 |
| 14-52 | Pharmacy | 3-1-3-14-14-52 | x | NP | 05-Feb-13 |
| 27-52 | PMV | 3-1-3-27-27-52 | x | NP | 08-Feb-13 |
| 39-59 | PMV | 4-1-2-39-39-59 | x | NP | 15-Dec-12 |
| 45-52 | PMV | 4-1-2-45-45-52 | x | NP | 15-Dec-12 |
| 10-52 | Pharmacy | 4-2-1-10-10-52 | x | NP | 07-Feb-13 |

| | | | | | |
|---|---|---|---|---|---|
| 1-52 | Pharmacy | 4-2-3-1-1-52 | x | NP | 05-Feb-13 |
| 24-52 | PMV | 4-2-3-24-24-52 | x | NP | 06-Feb-13 |
| 27-52B | PMV | 4-2-3-27-27-52B | x | NP | 08-Feb-13 |
| 28-52 | PMV | 4-2-3-28-28-52 | x | NP | 06-Feb-13 |
| 31-52 | PMV | 4-2-3-31-31-52 | x | NP | 05-Feb-13 |
| 10-52 | PMV | 9-1-1-10-10-52 | x | NP | 06-Feb-13 |
| 19-52 | Pharmacy | 9-1-2-19-19-52 | x | NP | 05-Feb-13 |
| 25-52 | Pharmacy | 9-1-2-25-25-52 | x | NP | 06-Feb-13 |
| 28-52 | Pharmacy | 9-1-2-28-28-52 | x | NP | 05-Feb-13 |
| 1-44 | Pharmacy | 11-3-6-1-1-44 | x | NP | 07-Feb-13 |
| 4-67 | PMV | 11-3-7-4-4-67 | x | NP | 06-Feb-13 |
| 28-52 | PMV | 11-3-7-28-28-52 | x | NP | 05-Feb-13 |
| 31-51 | PMV | 11-3-7-31-31-51 | x | NP | 07-Feb-13 |
| 10-52 | Pharmacy | 12-2-1-10-10-52 | x | NP | 05-Feb-13 |
| 6-52 | PMV | 12-2-6-6-6-52 | x | NP | 06-Feb-13 |
| 2-52 | Pharmacy | 14-2-5-2-2-52 | x | NP | 06-Feb-13 |
| 25-52 | Pharmacy | 14-2-5-25-25-52 | x | NP | 06-Feb-13 |
| 22-52 | PMV | 15-2-4-22-22-52 | x | NP | 08-Feb-13 |
| 34-52 | PMV | 15-2-4-34-34-52 | x | NP | 08-Feb-13 |
| 74-52 | PMV | 16-3-1-74-74-52 | x | NP | 09-Feb-13 |
| 5-52 | PMV | 16-3-3-5-5-52 | x | NP | 08-Feb-13 |
| 18-52 | Pharmacy | 17-1-2-18-18-52 | x | NP | 05-Feb-13 |
| 39-52 | PMV | 17-1-2-39-39-52 | x | NP | 05-Feb-13 |
| 57-52 | PMV | 17-1-2-57-57-52 | x | NP | 06-Feb-13 |
| 2-67A | Pharmacy | 14-2-5-2-2-67A | x | | 06-Feb-13 |
| 33-67B | PMV | 17-1-2-33-33-67B | x | | 05-Feb-13 |
| 33-56 | Pharmacy | 3-1-3-33-33-56 | x | | 07-Feb-13 |
| 11-59 | Pharmacy | 3-1-3-11-11-59 | x | | 07-Feb-13 |
| 43-59 | PMV | 4-1-2-43-43-59 | x | | 15-Dec-12 |
| 16-59 | Pharmacy | 9-1-1-16-16-59 | x | | 06-Feb-13 |
| 18-59 | Pharmacy | 9-1-2-18-18-59 | x | | 05-Feb-13 |
| 20-59 | Pharmacy | 9-1-2-20-20-59 | x | | 05-Feb-13 |
| 4-59 | Pharmacy | 15-2-4-4-4-59 | x | | 07-Feb-13 |
| 6-67C | Pharmacy | 14-2-5-6-6-67C | x | | 06-Feb-13 |
| 33-67 | Pharmacy | 3-1-3-33-33-67 | x | | 07-Feb-13 |
| 2-5 | Pharmacy | 14-2-5-2-2-5 | x | | 06-Feb-13 |
| 4-67F | Pharmacy | 15-2-4-4-4-67F | x | | 07-Feb-13 |
| 10-10 | Pharmacy | 3-1-3-10-10-10 | x | | 06-Feb-13 |
| 31-10 | Pharmacy | 3-1-3-31-31-10 | x | | 08-Feb-13 |
| 32-10 | Pharmacy | 3-1-3-32-32-10 | x | | 08-Feb-13 |
| 33-10 | Pharmacy | 3-1-3-33-33-10 | x | | 07-Feb-13 |
| 3-10 | Pharmacy | 3-2-3-3-03-10 | x | | 08-Feb-13 |
| 12-10 | Pharmacy | 3-2-5-12-12-10 | x | | 11-Feb-13 |
| 9-10 | Pharmacy | 11-3-7-9-9-10 | x | | 05-Feb-13 |
| 28-10 | PMV | 11-3-7-28-28-10 | x | | 05-Feb-13 |
| 14-10 | PMV | 12-2-1-14-14-10 | x | | 06-Feb-13 |
| 42-10 | Pharmacy | 14-3-1-42-42-10 | x | | 06-Feb-13 |
| 7-10 | Pharmacy | 15-2-4-7-7-10 | x | | 06-Feb-13 |
| 77-10 | PMV | 16-3-1-77-77-10 | x | | 06-Feb-13 |
| 83-10 | Pharmacy | 16-3-1-83-83-10 | x | | 05-Feb-13 |

| | | | | |
|---|---|---|---|---|
| 36-10 | PMV | 17-1-2-36-36-10 | x | 08-Feb-13 |
| 59-10 | Pharmacy | 17-1-2-59-59-10 | x | 07-Feb-13 |
| 2-10 | PMV | 17-1-3-2-2-10 | x | 06-Feb-13 |
| 57-67a | PMV | 17-1-2-57-57-67a | x | 06-Feb-13 |
| 4-67H | Pharmacy | 15-2-4-4-4-67H | x | 07-Feb-13 |
| 3-47 | Pharmacy | 3-2-3-3-3-47 | x | 08-Feb-13 |
| 42-47 | Pharmacy | 14-3-1-42-42-47 | x | 06-Feb-13 |
| 18-67A | Pharmacy | 9-1-2-18-18-67A | x | 05-Feb-13 |
| 18-60 | Pharmacy | 9-1-2-18-18-60 | x | 05-Feb-13 |
| 20-61 | Pharmacy | 9-1-2-20-20-61 | x | 05-Jan-13 |
| 14-2 | Pharmacy | 3-1-3-14-14-2 | x | 05-Feb-13 |
| 31-2 | Pharmacy | 3-1-3-31-31-2 | x | 08-Feb-13 |
| 33-2 | Pharmacy | 3-1-3-33-33-2 | x | 07-Feb-13 |
| 3-67B | Pharmacy | 3-2-3-3-3-67B | x | 08-Feb-13 |
| 12-2 | Pharmacy | 3-2-5-12-12-2 | x | 11-Feb-13 |
| 10-62 | Pharmacy | 4-2-1-10-10-62 | x | 07-Feb-13 |
| 37-2 | PMV | 4-2-3-37-37-2 | x | 06-Feb-13 |
| 19-2 | Pharmacy | 9-1-2-19-19-2 | x | 05-Feb-13 |
| 28-2 | PMV | 11-3-7-28-28-2 | x | 05-Feb-13 |
| 10-2 | Pharmacy | 12-2-1-10-10-2 | x | 05-Feb-13 |
| 14-2 | PMV | 12-2-1-14-14-2 | x | 06-Feb-13 |
| 8-2 | Pharmacy | 14-3-1-8-8-2 | x | 05-Jan-13 |
| 62-2 | Pharmacy | 14-3-1-62-62-2 | x | 08-Feb-13 |
| 25-2 | PMV | 15-2-4-25-25-2 | x | 07-Feb-13 |
| 34-2 | PMV | 15-2-4-34-34-2 | x | 08-Feb-13 |
| 36-2 | PMV | 15-2-4-36-36-2 | x | 06-Feb-13 |
| 76-2 | PMV | 16-3-1-76-76-2 | x | 09-Feb-13 |
| 83-2 | Pharmacy | 16-3-1-83-83-2 | x | 05-Feb-13 |
| 2-2 | PMV | 17-1-3-2-2-2 | x | 06-Feb-13 |
| 30-5 | Pharmacy | 3-1-3-30-30-5 | x | 09-Feb-13 |
| 34-5A | Pharmacy | 3-1-3-34-34-5A | x | 05-Feb-13 |
| 12-5 | Pharmacy | 3-2-5-12-12-5 | x | 11-Feb-13 |
| 43-5 | PMV | 4-1-2-43-43-5 | x | 15-Dec-12 |
| 16-5 | Pharmacy | 9-1-1-16-16-5 | x | 06-Feb-13 |
| 28-5 | Pharmacy | 9-1-2-28-28-5 | x | 05-Feb-13 |
| 31-5 | PMV | 14-3-1-31-31-5 | x | 07-Feb-13 |
| 37-5 | Pharmacy | 14-3-1-37-37-5 | x | 09-Feb-13 |
| 11-6 | Pharmacy | 3-1-3-11-11-6 | x | 07-Feb-13 |
| 21-6 | Pharmacy | 3-1-3-21-21-6 | x | 07-Feb-13 |
| 32-6 | Pharmacy | 3-1-3-32-32-6 | x | 08-Feb-13 |
| 62-6 | Pharmacy | 14-3-1-62-62-6 | x | 08-Feb-13 |
| 1-6 | Pharmacy | 14-3-2-1-1-6 | x | 06-Jan-13 |
| 2-6 | PMV | 17-1-3-2-2-6 | x | 06-Feb-13 |
| 3-67A | Pharmacy | 3-2-3-3-3-67A | x | 08-Feb-13 |
| 6-67 | Pharmacy | 3-2-3-6-6-67 | x | 11-Feb-13 |
| 38-67A | Pharmacy | 4-2-3-38-38-67A | x | 05-Feb-13 |
| 4-67K | Pharmacy | 15-2-4-4-4-67K | x | 07-Feb-13 |
| 13-67 | PMV | 4-1-2-13-13-67 | x | 15-Dec-12 |
| 11-67A | PMV | 16-3-5-11-11-67A | x | 05-Feb-13 |
| 7-19 | PMV | 9-1-1-7-7-19 | x | 06-Feb-13 |

| | | | | |
|---|---|---|---|---|
| 18-19 | Pharmacy | 9-1-2-18-18-19 | x | 05-Feb-13 |
| 36-57 | PMV | 17-1-2-36-36-57 | x | 08-Feb-13 |
| 42-67A | Pharmacy | 14-3-1-42-42-67A | x | 06-Feb-13 |
| 77-67 | PMV | 16-3-1-77-77-67 | x | 06-Feb-13 |
| 57-67b | PMV | 17-1-2-57-57-67b | x | 06-Feb-13 |
| 28-67A | PMV | 4-2-3-28-28-67A | x | 06-Feb-13 |
| 9-67 | Pharmacy | 11-3-7-9-9-67 | x | 05-Feb-13 |
| 27-67A | Pharmacy | 12-2-1-27-27-67A | x | 06-Feb-13 |
| 2-67 | Pharmacy | 14-2-5-2-2-67 | x | 06-Feb-13 |
| 4-67L | Pharmacy | 15-2-4-4-4-67L | x | 07-Feb-13 |
| 11-67A | Pharmacy | 15-2-4-11-11-67A | x | 05-Feb-13 |
| 42-67 | PMV | 15-2-4-42-42-67 | x | 05-Feb-13 |
| 11-29 | Pharmacy | 3-1-3-11-11-29 | x | 07-Feb-13 |
| 12-29 | Pharmacy | 3-2-5-12-12-29 | x | 11-Feb-13 |
| 16-29 | Pharmacy | 4-2-3-16-16-29 | x | 06-Feb-13 |
| 21-29 | PMV | 4-2-3-21-21-29 | x | 05-Feb-13 |
| 31-29 | PMV | 4-2-3-31-31-29 | x | 05-Feb-13 |
| 7-29 | PMV | 9-1-1-7-7-29 | x | 06-Feb-13 |
| 28-29 | PMV | 11-3-7-28-28-29 | x | 05-Feb-13 |
| 1-32 | Pharmacy | 11-3-6-1-1-32 | x | 07-Feb-13 |
| 28-32 | PMV | 11-3-7-28-28-32 | x | 05-Feb-13 |
| 5-67A | Pharmacy | 12-2-6-5-5-67A | x | 09-Feb-13 |
| 19-67 | Pharmacy | 17-1-2-19-19-67 | x | 08-Jan-13 |
| 31-40 | Pharmacy | 3-1-3-31-31-40 | x | 08-Feb-13 |
| 38-40 | Pharmacy | 4-2-3-38-38-40 | x | 05-Feb-13 |
| 16-40 | Pharmacy | 9-1-1-16-16-40 | x | 06-Feb-13 |
| 28-40 | PMV | 11-3-7-28-28-40 | x | 05-Feb-13 |
| 27-40 | Pharmacy | 12-2-1-27-27-40 | x | 06-Feb-13 |
| 12-67C | Pharmacy | 3-2-5-12-12-67C | x | 11-Feb-13 |
| 1-67 | Pharmacy | 4-2-3-1-1-67 | x | 05-Feb-13 |
| 20-67A | PMV | 4-2-3-20-20-67A | x | 06-Feb-13 |
| 21-67B | PMV | 4-2-3-21-21-67B | x | 05-Feb-13 |
| 25-67B | Pharmacy | 9-1-2-25-25-67B | x | 06-Feb-13 |
| 28-67B | PMV | 12-2-1-28-28-67B | x | 05-Feb-13 |
| 1-67A | Pharmacy | 14-3-2-1-1-67A | x | 06-Jan-13 |
| 4-67D | Pharmacy | 15-2-4-4-4-67D | x | 07-Feb-13 |
| 7-67B | Pharmacy | 15-2-4-7-7-67B | x | 06-Feb-13 |
| 25-67A | PMV | 15-2-4-25-25-67A | x | 07-Feb-13 |
| 42-67 | PMV | 15-2-4-42-42-67 | x | 05-Feb-13 |
| 11-67B | Pharmacy | 15-2-4-11-11-67B | x | 05-Feb-13 |
| 6-67A | PMV | 9-1-1-6-6-67A | x | 07-Feb-13 |
| 4-67B | Pharmacy | 15-2-4-4-4-67B | x | 07-Feb-13 |
| 21-67A | PMV | 4-2-3-21-21-67A | x | 05-Feb-13 |
| 28-67 | PMV | 4-2-3-28-28-67 | x | 06-Feb-13 |
| 34-67 | PMV | 4-2-3-34-34-67 | x | 06-Feb-13 |
| 37-67 | PMV | 4-2-3-37-37-67 | x | 06-Feb-13 |
| 28-67A | PMV | 12-2-1-28-28-67A | x | 05-Feb-13 |
| 5-67C | Pharmacy | 12-2-6-5-5-67C | x | 09-Feb-13 |
| 7-67A | Pharmacy | 15-2-4-7-7-67A | x | 06-Feb-13 |
| 11-67C | Pharmacy | 15-2-4-11-11-67C | x | 05-Feb-13 |

| | | | | |
|---|---|---|---|---|
| 5-67 | PMV | 16-3-2-5-5-67 | x | 08-Feb-13 |
| 7-51 | Pharmacy | 3-1-3-7-7-51 | x | 05-Feb-13 |
| 9-48 | Pharmacy | 3-1-3-9-9-48 | x | 09-Feb-13 |
| 11-48B | Pharmacy | 3-1-3-11-11-48B | x | 07-Feb-13 |
| 12-48 | Pharmacy | 3-2-5-12-12-48 | x | 11-Feb-13 |
| 30-48 | PMV | 4-2-3-30-30-48 | x | 05-Feb-13 |
| 37-48 | PMV | 4-2-3-37-37-48 | x | 06-Feb-13 |
| 44-48 | PMV | 5-1-2-44-44-48 | x | 15-Dec-12 |
| 29-48 | Pharmacy | 9-1-2-29-29-48 | x | 05-Feb-13 |
| 28-48 | PMV | 11-3-7-28-28-48 | x | 05-Feb-13 |
| 27-48 | Pharmacy | 12-2-1-27-27-48 | x | 06-Feb-13 |
| 5-48 | Pharmacy | 12-2-6-5-5-48 | x | 09-Feb-13 |
| 1-48 | Pharmacy | 14-3-2-1-1-48 | x | 06-Jan-13 |
| 5-48 | PMV | 16-3-2-5-5-48 | x | 08-Feb-13 |
| 36-48 | PMV | 17-1-2-36-36-48 | x | 08-Feb-13 |
| 59-48 | Pharmacy | 17-1-2-59-59-48 | x | 07-Feb-13 |
| 7-51 | Pharmacy | 3-1-3-7-7-51 | x | 05-Feb-13 |
| 10-51 | Pharmacy | 3-1-3-10-10-51 | x | 06-Feb-13 |
| 14-51 | Pharmacy | 3-1-3-14-14-51 | x | 05-Feb-13 |
| 27-51 | PMV | 3-1-3-27-27-51 | x | 08-Feb-13 |
| 30-51 | Pharmacy | 3-1-3-30-30-51 | x | 09-Feb-13 |
| 31-51 | Pharmacy | 3-1-3-31-31-51 | x | 08-Feb-13 |
| 32-51 | Pharmacy | 3-1-3-32-32-51 | x | 08-Feb-13 |
| 33-51 | Pharmacy | 3-1-3-33-33-51 | x | 07-Feb-13 |
| 34-51 | Pharmacy | 3-1-3-34-34-51 | x | 05-Feb-13 |
| 3-57 | Pharmacy | 3-2-3-3-3-57 | x | 08-Feb-13 |
| 6-51 | Pharmacy | 3-2-3-6-6-51 | x | 11-Feb-13 |
| 12-51 | Pharmacy | 3-2-5-12-12-51 | x | 11-Feb-13 |
| 13-51 | Pharmacy | 4-1-2-13-13-51 | x | 15-Dec-12 |
| 39-51 | PMV | 4-1-2-39-39-51 | x | 15-Dec-12 |
| 10-51 | Pharmacy | 4-2-1-10-10-51 | x | 07-Feb-13 |
| 8-51 | Pharmacy | 4-2-3-8-8-51 | x | 05-Feb-13 |
| 16-51 | Pharmacy | 4-2-3-16-16-51 | x | 06-Feb-13 |
| 24-51 | PMV | 4-2-3-24-24-51 | x | 06-Feb-13 |
| 30-51 | PMV | 4-2-3-30-30-51 | x | 05-Feb-13 |
| 31-51 | PMV | 4-2-3-31-31-51 | x | 05-Feb-13 |
| 37-51 | PMV | 4-2-3-37-37-51 | x | 06-Feb-13 |
| 38-51 | Pharmacy | 4-2-3-38-38-51 | x | 05-Feb-13 |
| 42-51 | PMV | 5-1-2-42-42-51 | x | 15-Dec-12 |
| 10-51 | PMV | 9-1-1-10-10-51 | x | 06-Feb-13 |
| 16-51 | Pharmacy | 9-1-1-16-16-51 | x | 06-Feb-13 |
| 19-51 | Pharmacy | 9-1-2-19-19-51 | x | 05-Feb-13 |
| 20-51 | Pharmacy | 9-1-2-20-20-51 | x | 05-Feb-13 |
| 25-51 | Pharmacy | 9-1-2-25-25-51 | x | 06-Feb-13 |
| 28-51 | Pharmacy | 9-1-2-28-28-51 | x | 05-Feb-13 |
| 29-51 | Pharmacy | 9-1-2-29-29-51 | x | 05-Feb-13 |
| 20-51 | PMV | 11-3-5-20-20-51 | x | 06-Feb-13 |
| 1-51 | Pharmacy | 11-3-6-1-1-51 | x | 07-Feb-13 |
| 4-51 | PMV | 11-3-7-4-4-51 | x | 06-Feb-13 |
| 28-52 | PMV | 11-3-7-28-28-52 | x | 05-Feb-13 |

| | | | | |
|---|---|---|---|---|
| 33-51 | PMV | 11-3-7-33-33-51 | x | 06-Feb-13 |
| 2-51 | Pharmacy | 12-2-1-2-2-51 | x | 06-Feb-13 |
| 10-51 | Pharmacy | 12-2-1-10-10-51 | x | 04-Feb-13 |
| 14-51 | PMV | 12-2-1-14-14-51 | x | 06-Feb-13 |
| 15-51 | PMV | 12-2-1-15-15-51 | x | 06-Feb-13 |
| 5-51 | Pharmacy | 12-2-6-5-5-51 | x | 09-Feb-13 |
| 6-51 | Pharmacy | 14-2-5-6-6-51 | x | 06-Feb-13 |
| 22-51 | PMV | 14-2-5-22-22-51 | x | 07-Feb-13 |
| 25-51 | Pharmacy | 14-2-5-25-25-51 | x | 06-Feb-13 |
| 8-51 | Pharmacy | 14-3-1-8-8-51 | x | 05-Jan-13 |
| 10-49 | Pharmacy | 14-3-1-10-10-49 | x | 05-Jan-13 |
| 14-51 | Pharmacy | 14-3-1-14-14-51 | x | 05-Jan-13 |
| 21-51 | Pharmacy | 14-3-1-21-21-51 | x | 09-Feb-13 |
| 31-51 | PMV | 14-3-1-31-31-51 | x | 07-Feb-13 |
| 37-51 | Pharmacy | 14-3-1-37-37-51 | x | 09-Feb-13 |
| 53-51 | PMV | 14-3-1-53-53-51 | x | 07-Feb-13 |
| 59-51 | Pharmacy | 14-3-1-59-59-51 | x | 08-Feb-13 |
| 62-51 | Pharmacy | 14-3-1-62-62-51 | x | 08-Feb-13 |
| 1-51 | Pharmacy | 14-3-2-1-1-51 | x | 06-Jan-13 |
| 8-51 | Pharmacy | 15-2-4-8-8-51 | x | 06-Feb-13 |
| 11-51 | Pharmacy | 15-2-4-11-11-51 | x | 05-Feb-13 |
| 25-51 | PMV | 15-2-4-25-25-51 | x | 07-Feb-13 |
| 34-51 | PMV | 15-2-4-34-34-51 | x | 08-Feb-13 |
| 36-51 | PMV | 15-2-4-36-36-51 | x | 06-Feb-13 |
| 42-51 | PMV | 15-2-4-42-42-51 | x | 05-Feb-13 |
| 44-51 | PMV | 15-2-4-44-44-51 | x | 05-Feb-13 |
| 10-51 | PMV | 16-3-1-10-10-51 | x | 06-Feb-13 |
| 74-49 | PMV | 16-3-1-74-74-49 | x | 09-Feb-13 |
| 75-51 | PMV | 16-3-1-75-75-51 | x | 06-Feb-13 |
| 76-50 | PMV | 16-3-1-76-76-50 | x | 09-Feb-13 |
| 5-51 | PMV | 16-3-2-5-5-51 | x | 08-Feb-13 |
| 5-49 | PMV | 16-3-3-5-5-49 | x | 08-Feb-13 |
| 15-51 | Pharmacy | 17-1-2-15-15-51 | x | 07-Feb-13 |
| 19-51 | Pharmacy | 17-1-2-19-19-51 | x | 08-Feb-13 |
| 36-51 | PMV | 17-1-2-36-36-51 | x | 08-Feb-13 |
| 39-51 | PMV | 17-1-2-39-39-51 | x | 05-Feb-13 |
| 46-51 | PMV | 17-1-2-46-46-51 | x | 05-Feb-13 |
| 51-51 | PMV | 17-1-2-51-51-51 | x | 05-Feb-13 |
| 57-51 | PMV | 17-1-2-57-57-51 | x | 06-Feb-13 |
| 2-51 | PMV | 17-1-3-2-2-51 | x | 06-Feb-13 |
| 3-51 | Pharmacy | 17-1-3-3-3-51 | x | 06-Feb-13 |
| 11-67B | Pharmacy | 3-1-3-11-11-67B | x | 07-Feb-13 |
| 30-67A | Pharmacy | 3-1-3-30-30-67A | x | 09-Feb-13 |
| 12-67D | Pharmacy | 3-2-5-12-12-67D | x | 11-Feb-13 |
| 42-67B | Pharmacy | 14-3-1-42-42-67B | x | 06-Feb-13 |
| 9-53 | Pharmacy | 3-1-3-9-9-53 | x | 09-Feb-13 |
| 14-53 | Pharmacy | 3-1-3-14-14-53 | x | 05-Feb-13 |
| 31-53 | Pharmacy | 3-1-3-31-31-53 | x | 08-Feb-13 |
| 34-53 | Pharmacy | 3-1-3-34-34-53 | x | 05-Feb-13 |
| 3-53 | Pharmacy | 3-2-3-3-3-53 | x | 08-Feb-13 |

| | | | | |
|---|---|---|---|---|
| 39-53 | PMV | 4-1-2-39-39-53 | x | 15-Dec-12 |
| 10-53 | Pharmacy | 4-2-1-10-10-53 | x | 07-Feb-13 |
| 16-53 | Pharmacy | 9-1-1-16-16-53 | x | 06-Feb-13 |
| 20-53 | Pharmacy | 9-1-2-20-20-53 | x | 05-Jan-13 |
| 10-53 | Pharmacy | 12-2-1-10-10-53 | x | 05-Feb-13 |
| 42-53 | Pharmacy | 14-3-1-42-42-53 | x | 06-Feb-13 |
| 83-53 | Pharmacy | 16-3-1-83-83-53 | x | 05-Feb-13 |
| 36-53 | PMV | 17-1-2-36-36-53 | x | 08-Feb-13 |
| 39-53 | PMV | 17-1-2-39-39-53 | x | 05-Feb-13 |
| 57-53 | PMV | 17-1-2-57-57-53 | x | 06-Feb-13 |
| 31-67 | Pharmacy | 3-1-3-31-31-67 | x | 08-Feb-13 |
| 25-67A | Pharmacy | 9-1-2-25-25-67A | x | 06-Feb-13 |
| 6-67A | Pharmacy | 14-2-5-6-6-67A | x | 06-Feb-13 |
| 4-67E | Pharmacy | 15-2-4-4-4-67E | x | 07-Feb-13 |
| 15-67 | Pharmacy | 17-1-2-15-15-67 | x | 07-Feb-13 |
| 2-55 | PMV | 17-1-3-2-2-55 | x | 06-Feb-13 |
| 11-56 | Pharmacy | 3-1-3-11-11-56 | x | 07-Feb-13 |
| 30-56 | PMV | 4-2-3-30-30-56 | x | 05-Feb-13 |
| 31-56 | PMV | 11-3-7-31-31-56 | x | 07-Feb-13 |
| 6-56 | Pharmacy | 14-2-5-6-6-56 | x | 06-Feb-13 |
| 8-56 | Pharmacy | 14-3-1-8-8-56 | x | 05-Jan-13 |
| 4-56 | Pharmacy | 15-2-4-4-4-56 | x | 07-Feb-13 |
| 34-56 | PMV | 15-2-4-34-34-56 | x | 08-Feb-13 |
| 42-56 | PMV | 15-2-4-42-42-56 | x | 05-Feb-13 |
| 76-56 | PMV | 16-3-1-76-76-56 | x | 09-Feb-13 |
| 2-56 | PMV | 17-1-3-2-2-56 | x | 06-Feb-13 |
| 21-61 | Pharmacy | 3-1-3-21-21-61 | x | 07-Feb-13 |
| 31-61 | PMV | 4-2-3-31-31-61 | x | 05-Feb-13 |
| 38-61 | Pharmacy | 4-2-3-38-38-61 | x | 05-Feb-13 |
| 44-61 | PMV | 5-1-2-44-44-61 | x | 15-Dec-12 |
| 9-61 | Pharmacy | 11-3-7-9-9-61 | x | 05-Feb-13 |
| 42-61 | Pharmacy | 14-3-1-42-42-61 | x | 06-Feb-13 |
| 10-61 | PMV | 16-3-1-10-10-61 | x | 06-Feb-13 |
| 32-62 | Pharmacy | 3-1-3-32-32-62 | x | 08-Feb-13 |
| 16-62 | Pharmacy | 4-2-3-16-16-62 | x | 06-Feb-13 |
| 9-62 | Pharmacy | 11-3-7-9-9-62 | x | 05-Feb-13 |
| 12-62 | PMV | 11-3-7-12-12-62 | x | 06-Feb-13 |
| 15-62 | PMV | 12-2-1-15-15-62 | x | 06-Feb-13 |
| 17-62 | PMV | 12-2-1-17-17-62 | x | 06-Feb-13 |
| 28-62 | PMV | 12-2-1-28-28-62 | x | 05-Feb-13 |
| 14-62 | PMV | 15-2-4-14-14-62 | x | 06-Feb-13 |
| 6-62 | Pharmacy | 14-2-5-6-6-62 | x | 06-Feb-13 |
| 33-66 | Pharmacy | 3-1-3-33-33-66 | x | 07-Feb-13 |
| 10-66 | Pharmacy | 4-2-1-10-10-66 | x | 07-Feb-13 |
| 18-66 | Pharmacy | 9-1-2-18-18-66 | x | 05-Feb-13 |
| 10-66 | Pharmacy | 12-2-1-10-10-66 | x | 04-Feb-13 |
| 6-66 | PMV | 12-2-6-6-6-66 | x | 06-Feb-13 |
| 1-66 | Pharmacy | 14-3-2-1-1-66 | x | 06-Jan-13 |
| 39-67 | PMV | 15-2-4-39-39-67 | x | 05-Feb-13 |
| 22-67B | Pharmacy | 17-1-2-22-22-67B | x | 05-Feb-13 |

| | | | | |
|---|---|---|---|---|
| 10-17 | Pharmacy | 3-1-3-10-10-17 | x | 06-Feb-13 |
| 26-17 | PMV | 3-1-3-26-26-17 | x | 08-Feb-13 |
| 27-17 | PMV | 3-1-3-27-27-17 | x | 08-Feb-13 |
| 33-17 | Pharmacy | 3-1-3-33-33-17 | x | 07-Feb-13 |
| 34-17 | Pharmacy | 3-1-3-34-34-17 | x | 05-Feb-13 |
| 16-17 | Pharmacy | 4-2-3-16-16-17 | x | 06-Feb-13 |
| 20-16 | PMV | 4-2-3-20-20-16 | x | 06-Feb-13 |
| 5-17 | PMV | 9-1-1-5-05-17 | x | 07-Feb-13 |
| 19-17 | Pharmacy | 9-1-2-19-19-17 | x | 05-Feb-13 |
| 22-17 | Pharmacy | 9-1-2-22-22-17 | x | 05-Feb-13 |
| 28-17 | Pharmacy | 9-1-2-28-28-17 | x | 05-Feb-13 |
| 2-17 | Pharmacy | 12-2-1-2-02-17 | x | 06-Feb-13 |
| 14-17 | PMV | 12-2-1-14-14-17 | x | 06-Feb-13 |
| 1-17A | Pharmacy | 12-2-6-1-01-17A | x | 08-Feb-13 |
| 5-17 | Pharmacy | 12-2-6-5-05-17 | x | 09-Feb-13 |
| 2-17 | Pharmacy | 14-2-5-2-02-17 | x | 06-Feb-13 |
| 10-17A | Pharmacy | 14-3-1-10-10-17A | x | 05-Jan-13 |
| 31-17 | PMV | 14-3-1-31-31-17 | x | 07-Feb-13 |
| 37-17 | Pharmacy | 14-3-1-37-37-17 | x | 09-Feb-13 |
| 1-17 | Pharmacy | 14-3-2-1-01-17 | x | 06-Jan-13 |
| 7-17 | Pharmacy | 15-2-4-7-07-17 | x | 06-Feb-13 |
| 17-17 | PMV | 15-2-4-17-17-17 | x | 07-Feb-13 |
| 22-17 | PMV | 15-2-4-22-22-17 | x | 08-Feb-13 |
| 39-17 | PMV | 15-2-4-39-39-17 | x | 05-Feb-13 |
| 10-17 | PMV | 16-3-1-10-10-17 | x | 06-Feb-13 |
| 69-17 | PMV | 16-3-1-69-69-17 | x | 09-Feb-13 |
| 72-17 | PMV | 16-3-1-72-72-17 | x | 09-Feb-13 |
| 18-17 | Pharmacy | 17-1-2-18-18-17 | x | 05-Feb-13 |
| 19-17 | Pharmacy | 17-1-2-19-19-17 | x | 08-Jan-13 |
| 36-17 | PMV | 17-1-2-36-36-17 | x | 08-Feb-13 |
| 39-17 | PMV | 17-1-2-39-39-17 | x | 05-Feb-13 |
| 46-17 | PMV | 17-1-2-46-46-17 | x | 05-Feb-13 |
| 51-17B | PMV | 17-1-2-51-51-17B | x | 05-Feb-13 |
| 57-17 | PMV | 17-1-2-57-57-17 | x | 06-Feb-13 |
| 59-17 | Pharmacy | 17-1-2-59-59-17 | x | 07-Feb-13 |
| 30-42 | Pharmacy | 3-1-3-30-30-42 | x | 09-Feb-13 |
| 33-42 | PMV | 17-1-2-33-33-42 | x | 05-Feb-13 |
| 16-43 | Pharmacy | 9-1-1-16-16-43 | x | 06-Feb-13 |
| 59-43 | Pharmacy | 17-1-2-59-59-43 | x | 07-Feb-13 |
| 10-65 | Pharmacy | 3-1-3-10-10-65 | x | 06-Feb-13 |
| 19-65 | Pharmacy | 9-1-2-19-19-65 | x | 05-Feb-13 |
| 1-65 | Pharmacy | 12-2-1-1-01-65 | x | 05-Feb-13 |
| 18-67B | Pharmacy | 9-1-2-18-18-67B | x | 05-Feb-13 |
| 9-7 | Pharmacy | 3-1-3-9-09-07 | x | 09-Feb-13 |
| 10-8 | Pharmacy | 3-1-3-10-10-08 | x | 06-Feb-13 |
| 45-7 | PMV | 4-1-2-45-45-07 | x | 15-Dec-12 |
| 10-8 | PMV | 9-1-1-10-10-08 | x | 06-Feb-13 |
| 7-8 | Pharmacy | 15-2-4-7-07-08 | x | 06-Feb-13 |
| 30-7 | Pharmacy | 3-1-3-30-30-07 | x | 09-Feb-13 |
| 33-67A | PMV | 17-1-2-33-33-67A | x | 05-Feb-13 |

| | | | | |
|---|---|---|---|---|
| 42-67C | PMV | 5-1-2-42-42-67C | x | 15-Dec-12 |
| 45-67B | PMV | 4-1-2-45-45-67B | x | 15-Dec-12 |
| 4-67I | Pharmacy | 15-2-4-4-04-67I | x | 07-Feb-13 |
| 7-33 | Pharmacy | 3-1-3-7-07-33 | x | 05-Feb-13 |
| 26-64 | PMV | 3-1-3-26-26-64 | x | 08-Feb-13 |
| 31-64 | Pharmacy | 3-1-3-31-31-64 | x | 08-Feb-13 |
| 6-64 | Pharmacy | 3-2-3-6-06-64 | x | 11-Feb-13 |
| 21-64 | PMV | 4-2-3-21-21-64 | x | 05-Feb-13 |
| 26-64 | PMV | 4-2-3-26-26-64 | x | 06-Feb-13 |
| 30-64 | PMV | 4-2-3-30-30-64 | x | 05-Feb-13 |
| 20-64 | PMV | 11-3-5-20-20-64 | x | 06-Feb-13 |
| 1-64 | Pharmacy | 11-3-7-1-01-64 | x | 05-Feb-13 |
| 15-64 | PMV | 12-2-1-15-15-64 | x | 06-Feb-13 |
| 28-64 | PMV | 12-2-1-28-28-64 | x | 05-Feb-13 |
| 14-64 | Pharmacy | 14-3-1-14-14-64 | x | 05-Jan-13 |
| 53-64 | PMV | 14-3-1-53-53-64 | x | 07-Feb-13 |
| 4-64 | Pharmacy | 15-2-4-4-04-64 | x | 07-Feb-13 |
| 7-64 | Pharmacy | 15-2-4-7-07-64 | x | 06-Feb-13 |
| 17-64 | PMV | 15-2-4-17-17-64 | x | 07-Feb-13 |
| 22-64 | PMV | 15-2-4-22-22-64 | x | 08-Feb-13 |
| 27-64 | PMV | 15-2-4-27-27-64 | x | 07-Feb-13 |
| 39-64 | PMV | 15-2-4-39-39-64 | x | 05-Feb-13 |
| 42-64 | PMV | 15-2-4-42-42-64 | x | 05-Feb-13 |
| 10-64 | PMV | 16-3-1-10-10-64 | x | 06-Feb-13 |
| 5-64 | PMV | 16-3-2-5-05-64 | x | 08-Feb-13 |
| 63-64 | PMV | 16-3-3-63-63-64 | x | 08-Feb-13 |
| 18-64 | Pharmacy | 17-1-2-18-18-64 | x | 05-Feb-13 |
| 2-64 | PMV | 17-1-3-2-02-64 | x | 06-Feb-13 |
| 26-65 | PMV | 3-1-3-26-26-65 | x | 08-Feb-13 |
| 45-64 | PMV | 4-1-2-45-45-64 | x | 15-Dec-12 |
| 1-64 | Pharmacy | 12-2-1-1-01-64 | x | 05-Feb-13 |
| 51-17A | PMV | 17-1-2-51-51-17A | x | 05-Feb-13 |
| 28-67 | PMV | 11-3-7-28-28-67 | x | 05-Feb-13 |
| 19-67 | Pharmacy | 17-1-2-19-19-67 | x | 08-Jan-13 |
| 6-67C | PMV | 12-2-6-6-06-67C | x | 06-Feb-13 |
| 7-20 | Pharmacy | 3-1-3-7-07-20 | x | 05-Feb-13 |
| 10-20 | Pharmacy | 3-1-3-10-10-20 | x | 06-Feb-13 |
| 14-20 | Pharmacy | 3-1-3-14-14-20 | x | 05-Feb-13 |
| 30-20 | Pharmacy | 3-1-3-30-30-20 | x | 09-Feb-13 |
| 31-20 | Pharmacy | 3-1-3-31-31-20 | x | 08-Feb-13 |
| 1-20 | Pharmacy | 4-2-3-1-01-20 | x | 05-Feb-13 |
| 37-20 | PMV | 4-2-3-37-37-20 | x | 06-Feb-13 |
| 28-20 | PMV | 11-3-7-28-28-20 | x | 05-Feb-13 |
| 1-20 | Pharmacy | 12-2-6-1-01-20 | x | 08-Feb-13 |
| 6-20 | PMV | 12-2-6-6-06-20 | x | 06-Feb-13 |
| 2-20 | Pharmacy | 14-2-5-2-02-20 | x | 06-Feb-13 |
| 6-19 | Pharmacy | 14-2-5-6-06-19 | x | 06-Feb-13 |
| 1-20 | Pharmacy | 14-3-2-1-01-20 | x | 06-Jan-13 |
| 39-20 | PMV | 15-2-4-39-39-20 | x | 05-Feb-13 |
| 59-20 | Pharmacy | 17-1-2-59-59-20 | x | 07-Feb-13 |

| | | | | |
|---|---|---|---|---|
| 3-20 | Pharmacy | 17-1-3-3-03-20 | x | 06-Feb-13 |
| 7-22 | Pharmacy | 3-1-3-7-07-22 | x | 05-Feb-13 |
| 10-22 | Pharmacy | 3-1-3-10-10-22 | x | 06-Feb-13 |
| 14-22 | Pharmacy | 3-1-3-14-14-22 | x | 05-Feb-13 |
| 1-22 | Pharmacy | 12-2-6-1-01-22 | x | 08-Feb-13 |
| 59-22 | Pharmacy | 17-1-2-59-59-22 | x | 07-Feb-13 |
| 21-67B | Pharmacy | 3-1-3-21-21-67B | x | 07-Feb-13 |
| 12-67A | Pharmacy | 3-2-5-12-12-67A | x | 11-Feb-13 |
| 11-33 | Pharmacy | 3-1-3-11-11-33 | x | 07-Feb-13 |
| 28-33 | PMV | 11-3-7-28-28-33 | x | 05-Feb-13 |
| 77-33 | PMV | 16-3-1-77-77-33 | x | 06-Feb-13 |
| 11-15 | Pharmacy | 3-1-3-11-11-15 | x | 07-Feb-13 |
| 3-14 | Pharmacy | 3-2-3-3-03-14 | x | 08-Feb-13 |
| 27-15 | Pharmacy | 12-2-1-27-27-15 | x | 06-Feb-13 |
| 1-15 | Pharmacy | 12-2-6-1-01-15 | x | 08-Feb-13 |
| 59-14 | Pharmacy | 17-1-2-59-59-14 | x | 07-Feb-13 |
| 9-14 | Pharmacy | 3-1-3-9-09-14 | x | 09-Feb-13 |
| 21-14 | Pharmacy | 3-1-3-21-21-14 | x | 07-Feb-13 |
| 33-14 | Pharmacy | 3-1-3-33-33-14 | x | 07-Feb-13 |
| 34-14 | Pharmacy | 3-1-3-34-34-14 | x | 05-Feb-13 |
| 6-14 | Pharmacy | 3-2-3-6-06-14 | x | 11-Feb-13 |
| 28-14 | Pharmacy | 9-1-2-28-28-14 | x | 05-Feb-13 |
| 25-14 | PMV | 11-3-7-25-25-14 | x | 08-Feb-13 |
| 28-14 | PMV | 11-3-7-28-28-14 | x | 05-Feb-13 |
| 14-14 | PMV | 12-2-1-14-14-14 | x | 06-Feb-13 |
| 27-14 | Pharmacy | 12-2-1-27-27-14 | x | 06-Feb-13 |
| 1-14 | Pharmacy | 12-2-6-1-01-14 | x | 08-Feb-13 |
| 5-14 | Pharmacy | 12-2-6-5-05-14 | x | 09-Feb-13 |
| 2-14 | Pharmacy | 14-2-5-2-02-14 | x | 06-Feb-13 |
| 14-14 | Pharmacy | 14-3-1-14-14-14 | x | 05-Jan-13 |
| 77-14 | PMV | 16-3-1-77-77-14 | x | 06-Feb-13 |
| 18-14 | Pharmacy | 17-1-2-18-18-14 | x | 05-Feb-13 |
| 36-14 | PMV | 17-1-2-36-36-14 | x | 08-Feb-13 |
| 57-14 | PMV | 17-1-2-57-57-14 | x | 06-Feb-13 |
| 3-14 | Pharmacy | 17-1-3-3-03-14 | x | 06-Feb-13 |
| 33-61 | Pharmacy | 3-1-3-33-33-61 | x | 07-Feb-13 |
| 1-38 | Pharmacy | 12-2-6-1-01-38 | x | 08-Feb-13 |
| 33-54 | Pharmacy | 3-1-3-33-33-54 | x | 07-Feb-13 |
| 6-67B | PMV | 12-2-6-6-06-67B | x | 06-Feb-13 |
| 57-67 | PMV | 17-1-2-57-57-67 | x | 06-Feb-13 |
| 7-24 | Pharmacy | 3-1-3-7-07-24 | x | 05-Feb-13 |
| 31-24 | Pharmacy | 3-1-3-31-31-24 | x | 08-Feb-13 |
| 34-24 | Pharmacy | 3-1-3-34-34-24 | x | 05-Feb-13 |
| 1-24 | Pharmacy | 12-2-6-1-01-24 | x | 08-Feb-13 |
| 2-24 | Pharmacy | 14-2-5-2-02-24 | x | 06-Feb-13 |
| 59-24 | Pharmacy | 14-3-1-59-59-24 | x | 08-Feb-13 |
| 9-24 | Pharmacy | 1-1-3-9-9-24 | x | 09-Feb-13 |
| 11-24 | Pharmacy | 3-1-3-11-11-24 | x | 07-Feb-13 |
| 30-24 | Pharmacy | 3-1-3-30-30-24 | x | 09-Feb-13 |
| 33-24 | Pharmacy | 3-1-3-33-33-24 | x | 07-Feb-13 |

| | | | | |
|---|---|---|---|---|
| 6-24 | Pharmacy | 3-2-3-6-06-24 | x | 11-Feb-13 |
| 12-24 | Pharmacy | 3-2-5-12-12-24 | x | 11-Feb-13 |
| 10-50 | Pharmacy | 4-2-1-10-10-50 | x | 07-Feb-13 |
| 16-24 | Pharmacy | 4-2-3-16-16-24 | x | 06-Feb-13 |
| 30-24 | PMV | 4-2-3-30-30-24 | x | 05-Feb-13 |
| 27-24 | Pharmacy | 12-2-1-27-27-24 | x | 06-Feb-13 |
| 28-24 | PMV | 11-3-7-28-28-24 | x | 05-Feb-13 |
| 1-24 | Pharmacy | 12-2-6-1-01-24 | x | 08-Feb-13 |
| 77-24 | PMV | 16-3-1-77-77-24 | x | 06-Feb-13 |
| 59-24 | Pharmacy | 17-1-2-59-59-24 | x | 07-Feb-13 |
| 3-24 | Pharmacy | 17-1-3-3-03-24 | x | 06-Feb-13 |
| 51-1 | PMV | 17-1-2-51-51-1 | x | 05-Feb-13 |
| 7-63 | Pharmacy | 3-1-3-7-7-63 | x | 05-Feb-13 |
| 9-63 | Pharmacy | 3-1-3-9-9-63 | x | 09-Feb-13 |
| 10-63 | Pharmacy | 3-1-3-10-10-63 | x | 06-Feb-13 |
| 11-63 | Pharmacy | 3-1-3-11-11-63 | x | 07-Feb-13 |
| 21-63 | Pharmacy | 3-1-3-21-21-63 | x | 07-Feb-13 |
| 26-63 | PMV | 3-1-3-26-26-63 | x | 08-Feb-13 |
| 27-63 | PMV | 3-1-3-27-27-63 | x | 08-Feb-13 |
| 32-63 | Pharmacy | 3-1-3-32-32-63 | x | 08-Feb-13 |
| 34-63 | Pharmacy | 3-1-3-34-34-63 | x | 05-Feb-13 |
| 13-63 | Pharmacy | 4-1-2-13-13-63 | x | 15-Dec-12 |
| 39-63 | PMV | 4-1-2-39-39-63 | x | 15-Dec-12 |
| 40-63 | PMV | 4-1-2-40-40-63 | x | 15-Dec-12 |
| 43-63 | PMV | 4-1-2-43-43-63 | x | 15-Dec-12 |
| 45-63 | PMV | 4-1-2-45-45-63 | x | 15-Dec-12 |
| 10-63 | Pharmacy | 4-2-1-10-10-63 | x | 07-Feb-13 |
| 1-63 | Pharmacy | 4-2-3-1-1-63 | x | 05-Feb-13 |
| 16-63 | Pharmacy | 4-2-3-16-16-63 | x | 06-Feb-13 |
| 20-63 | PMV | 4-2-3-20-20-63 | x | 06-Feb-13 |
| 24-63 | PMV | 4-2-3-24-24-63 | x | 06-Feb-13 |
| 26-63 | PMV | 4-2-3-26-26-63 | x | 06-Feb-13 |
| 37-63 | PMV | 4-2-3-37-37-63 | x | 06-Feb-13 |
| 44-63 | PMV | 5-1-2-44-44-63 | x | 15-Dec-12 |
| 5-63 | PMV | 9-1-1-5-5-63 | x | 07-Feb-13 |
| 7-63 | PMV | 9-1-1-7-7-63 | x | 06-Feb-13 |
| 10-63 | PMV | 9-1-1-10-10-63 | x | 06-Feb-13 |
| 25-63 | Pharmacy | 9-1-2-25-25-63 | x | 06-Feb-13 |
| 28-63 | Pharmacy | 9-1-2-28-28-63 | x | 05-Feb-13 |
| 20-63 | PMV | 11-3-5-20-20-63 | x | 06-Feb-13 |
| 1-63 | Pharmacy | 11-3-7-1-1-63 | x | 05-Feb-13 |
| 4-63 | PMV | 11-3-7-4-4-63 | x | 06-Feb-13 |
| 25-63 | PMV | 11-3-7-25-25-63 | x | 08-Feb-13 |
| 28-63 | PMV | 11-3-7-28-28-63 | x | 05-Feb-13 |
| 33-63 | PMV | 11-3-7-33-33-63 | x | 06-Feb-13 |
| 2-63 | Pharmacy | 12-2-1-2-2-63 | x | 06-Feb-13 |
| 10-63 | Pharmacy | 12-2-1-10-10-63 | x | 05-Feb-13 |
| 14-63 | PMV | 12-2-1-14-14-63 | x | 06-Feb-13 |
| 15-63 | PMV | 12-2-1-15-15-63 | x | 06-Feb-13 |
| 1-63 | Pharmacy | 12-2-6-1-1-63 | x | 08-Feb-13 |

| | | | | |
|---|---|---|---|---|
| 5-63 | Pharmacy | 12-2-6-5-5-63 | x | 09-Feb-13 |
| 2-63 | Pharmacy | 14-2-5-2-2-63 | x | 06-Feb-13 |
| 6-63 | Pharmacy | 14-2-5-6-6-63 | x | 06-Feb-13 |
| 22-63 | PMV | 14-2-5-22-22-63 | x | 07-Feb-13 |
| 25-63 | Pharmacy | 14-2-5-25-25-63 | x | 06-Feb-13 |
| 8-63 | Pharmacy | 14-3-1-8-8-63 | x | 05-Jan-13 |
| 10-63 | Pharmacy | 14-3-1-10-10-63 | x | 05-Jan-13 |
| 21-63 | Pharmacy | 14-3-1-21-21-63 | x | 09-Feb-13 |
| 37-63 | Pharmacy | 14-3-1-37-37-63 | x | 09-Feb-13 |
| 53-63 | PMV | 14-3-1-53-53-63 | x | 07-Feb-13 |
| 59-63 | Pharmacy | 14-3-1-59-59-63 | x | 08-Feb-13 |
| 1-63 | Pharmacy | 14-3-2-1-1-63 | x | 06-Jan-13 |
| 7-63 | Pharmacy | 15-2-4-7-7-63 | x | 06-Feb-13 |
| 8-63 | Pharmacy | 15-2-4-8-8-63 | x | 06-Feb-13 |
| 11-63 | Pharmacy | 15-2-4-11-11-63 | x | 05-Feb-13 |
| 14-63 | PMV | 15-2-4-14-14-63 | x | 06-Feb-13 |
| 22-63 | PMV | 15-2-4-22-22-63 | x | 08-Feb-13 |
| 23-63 | PMV | 15-2-4-23-23-63 | x | 07-Feb-13 |
| 25-63 | PMV | 15-2-4-25-25-63 | x | 07-Feb-13 |
| 39-63 | PMV | 15-2-4-39-39-63 | x | 05-Feb-13 |
| 164-63 | PMV | 15-2-4-44-164-63 | x | 05-Feb-13 |
| 74-63 | PMV | 16-3-1-74-74-63 | x | 09-Feb-13 |
| 80-63 | PMV | 16-3-1-80-80-63 | x | 06-Feb-13 |
| 83-63 | Pharmacy | 16-3-1-83-83-63 | x | 05-Feb-13 |
| 5-63 | PMV | 16-3-3-5-5-63 | x | 08-Feb-13 |
| 15-63 | Pharmacy | 17-1-2-15-15-63 | x | 07-Feb-13 |
| 33-63 | PMV | 17-1-2-33-33-63 | x | 05-Feb-13 |
| 36-63 | PMV | 17-1-2-36-36-63 | x | 08-Feb-13 |
| 39-63 | PMV | 17-1-2-39-39-63 | x | 05-Feb-13 |
| 57-63 | PMV | 17-1-2-57-57-63 | x | 06-Feb-13 |
| 3-63 | Pharmacy | 17-1-3-3-3-63 | x | 06-Feb-13 |
| 43-67B | PMV | 4-1-2-43-43-67B | x | 15-Dec-12 |
| 22-67A | Pharmacy | 17-1-2-22-22-67A | x | 05-Feb-13 |
| 20-67 | Pharmacy | 9-1-2-20-20-67 | x | 05-Jan-13 |
| 17-67 | PMV | 12-2-1-17-17-67 | x | 06-Feb-13 |
| 59-67 | Pharmacy | 14-3-1-59-59-67 | x | 08-Feb-13 |
| 42-67D | PMV | 5-1-2-42-42-67D | x | 15-Dec-12 |
| 7-67 | Pharmacy | 3-1-3-7-7-67 | x | 05-Feb-13 |
| 1-67A | Pharmacy | 12-2-6-1-1-67A | x | 08-Feb-13 |
| 62-67 | Pharmacy | 14-3-7-62-62-67 | x | 08-Feb-13 |
| 25-67B | PMV | 15-2-4-25-25-67B | x | 07-Feb-13 |
| 22-67C | Pharmacy | 17-1-2-22-22-67C | x | 05-Feb-13 |
| 10-57 | Pharmacy | 3-1-3-10-10-57 | x | 06-Feb-13 |
| 14-57 | Pharmacy | 3-1-3-14-14-57 | x | 05-Feb-13 |
| 21-57 | Pharmacy | 3-1-3-21-21-57 | x | 07-Feb-13 |
| 27-57 | PMV | 3-1-3-27-27-57 | x | 08-Feb-13 |
| 30-57 | Pharmacy | 3-1-3-30-30-57 | x | 09-Feb-13 |
| 34-57 | Pharmacy | 3-1-3-34-34-57 | x | 05-Feb-13 |
| 12-57 | Pharmacy | 3-2-5-12-12-57 | x | 11-Feb-13 |
| 43-57 | PMV | 4-1-2-43-43-57 | x | 15-Dec-12 |

| | | | | |
|---|---|---|---|---|
| 10-57 | Pharmacy | 4-2-1-10-10-57 | x | 07-Feb-13 |
| 8-57 | Pharmacy | 4-2-3-8-8-57 | x | 05-Feb-13 |
| 20-67B | PMV | 4-2-3-20-20-67B | x | 06-Feb-13 |
| 21-57 | PMV | 4-2-3-21-21-57 | x | 05-Feb-13 |
| 6-57 | PMV | 9-1-1-6-6-57 | x | 07-Feb-13 |
| 16-57 | Pharmacy | 9-1-1-16-16-57 | x | 06-Feb-13 |
| 19-57 | Pharmacy | 9-1-2-19-19-57 | x | 05-Feb-13 |
| 29-57 | Pharmacy | 9-1-2-29-29-57 | x | 05-Feb-13 |
| 20-57 | PMV | 11-3-5-20-20-57 | x | 06-Feb-13 |
| 1-57 | Pharmacy | 11-3-7-1-1-57 | x | 05-Feb-13 |
| 28-57 | PMV | 11-3-7-28-28-57 | x | 05-Feb-13 |
| 33-57 | PMV | 11-3-7-33-33-57 | x | 06-Feb-13 |
| 2-56 | Pharmacy | 12-2-1-2-2-56 | x | 06-Feb-13 |
| 14-57 | PMV | 12-2-1-14-14-57 | x | 06-Feb-13 |
| 28-57 | PMV | 12-2-1-28-28-57 | x | 05-Feb-13 |
| 1-57 | Pharmacy | 12-2-6-1-1-57 | x | 08-Feb-13 |
| 2-57 | Pharmacy | 14-2-5-2-2-57 | x | 06-Feb-13 |
| 6-57 | Pharmacy | 14-2-5-6-6-57 | x | 06-Feb-13 |
| 37-57 | Pharmacy | 14-3-1-37-37-57 | x | 09-Feb-13 |
| 59-56 | Pharmacy | 14-3-1-59-59-56 | x | 08-Feb-13 |
| 1-57 | Pharmacy | 14-3-2-1-1-57 | x | 06-Jan-13 |
| 8-57 | Pharmacy | 15-2-4-8-8-57 | x | 06-Feb-13 |
| 14-57 | PMV | 15-2-4-14-14-57 | x | 06-Feb-13 |
| 22-57 | PMV | 15-2-4-22-22-57 | x | 08-Feb-13 |
| 25-57 | PMV | 15-2-4-25-25-57 | x | 07-Feb-13 |
| 36-57 | PMV | 15-2-4-36-36-57 | x | 06-Feb-13 |
| 42-57 | PMV | 15-2-4-42-42-57 | x | 05-Feb-13 |
| 77-57 | PMV | 16-3-1-77-77-57 | x | 06-Feb-13 |
| 83-57 | Pharmacy | 16-3-1-83-83-57 | x | 05-Feb-13 |
| 5-57 | PMV | 16-3-2-5-5-57 | x | 08-Feb-13 |
| 15-57 | Pharmacy | 17-1-2-15-15-57 | x | 07-Feb-13 |
| 19-57 | Pharmacy | 17-1-2-19-19-57 | x | 08-Jan-13 |
| 22-57 | Pharmacy | 17-1-2-22-22-57 | x | 05-Feb-13 |
| 36-19 | PMV | 17-1-2-36-36-19 | x | 08-Feb-13 |
| 46-57 | PMV | 17-1-2-46-46-57 | x | 05-Feb-13 |
| 57-57 | PMV | 17-1-2-57-57-57 | x | 06-Feb-13 |
| 59-57 | Pharmacy | 17-1-2-59-59-57 | x | 07-Feb-13 |
| 2-57 | PMV | 17-1-3-2-2-57 | x | 06-Feb-13 |
| 3-57 | Pharmacy | 17-1-3-3-3-57 | x | 06-Feb-13 |
| 1-69 | Pharmacy | 11-3-6-1-1-69 | x | 07-Feb-13 |
| 10-67D | Pharmacy | 3-1-3-10-10-67D | x | 06-Feb-13 |
| 16-67 | Pharmacy | 4-2-3-16-16-67 | x | 06-Feb-13 |
| 6-67D | PMV | 12-2-6-6-6-67D | x | 06-Feb-13 |
| 4-67A | Pharmacy | 15-2-4-4-4-67A | x | 07-Feb-13 |
| 4-67J | Pharmacy | 15-2-4-4-4-67J | x | 07-Feb-13 |
| 4-67G | Pharmacy | 15-2-4-4-4-67G | x | 07-Feb-13 |
| 31-67 | PMV | 11-3-7-31-31-67 | x | 07-Feb-13 |
| 33-67 | PMV | 11-3-7-33-33-67 | x | 06-Feb-13 |
| 9-67 | Pharmacy | 3-1-3-9-9-67 | x | 09-Feb-13 |
| 14-3 | Pharmacy | 3-1-3-14-14-3 | x | 05-Feb-13 |

| | | | | |
|---|---|---|---|---|
| 37-67 | PMV | 4-2-3-37-37-67 | x | 06-Feb-13 |
| 5-2 | Pharmacy | 12-2-6-5-5-2 | x | 09-Feb-13 |
| 8-3 | Pharmacy | 14-3-1-8-8-3 | x | 05-Jan-13 |
| 83-67 | Pharmacy | 16-3-1-83-83-67 | x | 05-Feb-13 |
| 5-67B | Pharmacy | 12-2-6-5-5-67B | x | 09-Feb-13 |
| 10-67B | Pharmacy | 3-1-3-10-10-67B | x | 06-Feb-13 |
| 10-67 | Pharmacy | 4-2-1-10-10-67 | x | 07-Feb-13 |
| 10-67 | Pharmacy | 12-2-1-10-10-67 | x | 05-Feb-13 |
| 3-0 | Pharmacy | 3-2-3-3-3-0 | x | 08-Feb-13 |
| 27-12 | Pharmacy | 12-2-1-27-27-12 | x | 06-Feb-13 |
| 30-67B | Pharmacy | 3-1-3-30-30-67B | x | 09-Feb-13 |
| 10-67B | PMV | 16-3-1-10-10-67B | x | 06-Feb-13 |
| 6-7 | PMV | 9-1-1-6-6-7 | x | 07-Feb-13 |
| 29-19 | Pharmacy | 9-1-2-29-29-19 | x | 05-Feb-13 |
| 27-67B | Pharmacy | 12-2-1-27-27-67B | x | 06-Feb-13 |
| 5-67 | Pharmacy | 12-2-6-5-5-67 | x | 09-Feb-13 |
| 10-67C | Pharmacy | 3-1-3-10-10-67C | x | 06-Feb-13 |
| 11-28 | Pharmacy | 3-1-3-11-11-28 | x | 07-Feb-13 |
| 33-28 | Pharmacy | 3-1-3-33-33-28 | x | 07-Feb-13 |
| 10-28 | Pharmacy | 4-2-1-10-10-28 | x | 07-Feb-13 |
| 16-28 | Pharmacy | 4-2-3-16-16-28 | x | 06-Feb-13 |
| 28-28 | PMV | 4-2-3-28-28-28 | x | 06-Feb-13 |
| 29-28 | Pharmacy | 9-1-2-29-29-28 | x | 05-Feb-13 |
| 33-28 | PMV | 11-3-7-33-33-28 | x | 06-Feb-13 |
| 1-28A | Pharmacy | 12-2-1-1-1-28A | x | 05-Feb-13 |
| 27-28 | Pharmacy | 12-2-1-27-27-28 | x | 06-Feb-13 |
| 1-28 | Pharmacy | 12-2-6-1-1-28 | x | 06-Feb-13 |
| 2-28 | Pharmacy | 14-2-5-2-2-28 | x | 06-Feb-13 |
| 11-67B | PMV | 16-3-5-11-11-67B | x | 05-Feb-13 |
| 4-67C | Pharmacy | 15-2-4-4-4-67C | x | 07-Feb-13 |
| 11-48A | Pharmacy | 3-1-3-11-11-48A | x | 07-Feb-13 |
| 13-48 | Pharmacy | 4-1-2-13-13-48 | x | 15-Dec-12 |
| 43-48 | PMV | 4-1-2-43-43-48 | x | 15-Dec-12 |
| 25-48 | Pharmacy | 9-1-2-25-25-48 | x | 06-Feb-13 |
| 5-56 | Pharmacy | 12-2-6-5-5-56 | x | 09-Feb-13 |
| 43-67A | PMV | 4-1-2-43-43-67A | x | 15-Dec-12 |
| 14-67 | PMV | 12-2-1-14-14-67 | x | 06-Feb-13 |
| 22-67D | Pharmacy | 17-1-2-22-22-67D | x | 05-Feb-13 |
| 8-56 | Pharmacy | 14-3-1-8-8-56 | x | 05-Jan-13 |
| 14-56 | Pharmacy | 14-3-1-14-14-56 | x | 05-Jan-13 |
| 1-56 | Pharmacy | 14-3-2-1-1-56 | x | 06-Jan-13 |
| 38-67B | Pharmacy | 4-2-3-38-38-67B | x | 05-Feb-13 |
| 83-28 | Pharmacy | 16-3-1-83-83-28 | x | 05-Feb-13 |
| 30-67C | Pharmacy | 3-1-3-30-30-67C | x | 09-Feb-13 |
| 9-50 | Pharmacy | 3-1-3-9-9-50 | x | 09-Feb-13 |
| 10-50 | Pharmacy | 3-1-3-10-10-50 | x | 06-Feb-13 |
| 11-50 | Pharmacy | 3-1-3-11-11-50 | x | 07-Feb-13 |
| 14-50 | Pharmacy | 3-1-3-14-14-50 | x | 05-Feb-13 |
| 21-50 | Pharmacy | 3-1-3-21-21-50 | x | 07-Feb-13 |
| 27-50 | PMV | 3-1-3-27-27-50 | x | 08-Feb-13 |

| | | | | |
|---|---|---|---|---|
| 31-50 | Pharmacy | 3-1-3-31-31-50 | x | 08-Feb-13 |
| 33-50 | Pharmacy | 3-1-3-33-33-50 | x | 07-Feb-13 |
| 34-50 | Pharmacy | 3-1-3-34-34-50 | x | 05-Feb-13 |
| 10-50 | Pharmacy | 4-2-1-10-10-50 | x | 07-Feb-13 |
| 7-50 | PMV | 9-1-1-7-7-50 | x | 06-Feb-13 |
| 10-50 | PMV | 9-1-1-10-10-50 | x | 06-Feb-13 |
| 20-50 | Pharmacy | 9-1-2-20-20-50 | x | 05-Feb-13 |
| 29-50 | Pharmacy | 9-1-2-29-29-50 | x | 05-Feb-13 |
| 33-50 | PMV | 11-3-7-33-33-50 | x | 06-Feb-13 |
| 10-50 | Pharmacy | 12-2-1-10-10-50 | x | 05-Feb-13 |
| 14-50 | PMV | 12-2-1-14-14-50 | x | 06-Feb-13 |
| 15-50 | PMV | 12-2-1-15-15-50 | x | 06-Feb-13 |
| 27-50 | Pharmacy | 12-2-1-27-27-50 | x | 06-Feb-13 |
| 28-50 | PMV | 12-2-1-28-28-50 | x | 05-Feb-13 |
| 2-50 | Pharmacy | 14-2-5-2-2-50 | x | 06-Feb-13 |
| 8-50 | Pharmacy | 14-3-1-8-8-50 | x | 05-Jan-13 |
| 10-50 | Pharmacy | 14-3-1-10-10-50 | x | 05-Jan-13 |
| 14-50 | Pharmacy | 14-3-1-14-14-50 | x | 05-Jan-13 |
| 42-50 | Pharmacy | 14-3-1-42-42-50 | x | 06-Feb-13 |
| 7-50 | Pharmacy | 15-2-4-7-7-50 | x | 06-Feb-13 |
| 8-50 | Pharmacy | 15-2-4-8-8-50 | x | 06-Feb-13 |
| 11-50 | Pharmacy | 15-2-4-11-11-50 | x | 05-Feb-13 |
| 14-50 | PMV | 15-2-4-14-14-50 | x | 06-Feb-13 |
| 22-50 | PMV | 15-2-4-22-22-50 | x | 08-Feb-13 |
| 42-50 | PMV | 15-2-4-42-42-50 | x | 05-Feb-13 |
| 44-50 | PMV | 15-2-4-44-44-50 | x | 05-Feb-13 |
| 76-50 | PMV | 16-3-1-76-76-50 | x | 09-Feb-13 |
| 83-49 | Pharmacy | 16-3-1-83-83-49 | x | 05-Feb-13 |
| 42-67A | PMV | 5-1-2-42-42-67A | x | 15-Dec-12 |
| 6-67A | PMV | 12-2-6-6-06-67A | x | 06-Feb-13 |
| 14-21B | Pharmacy | 3-1-3-14-14-21B | x | 05-Feb-13 |
| 30-21 | Pharmacy | 3-1-3-30-30-21 | x | 09-Feb-13 |
| 1-21A | Pharmacy | 12-2-6-1-01-21A | x | 08-Feb-13 |
| 10-21 | Pharmacy | 3-1-3-10-10-21 | x | 06-Feb-13 |
| 11-21 | Pharmacy | 3-1-3-11-11-21 | x | 07-Feb-13 |
| 14-21A | Pharmacy | 3-1-3-14-14-21A | x | 05-Feb-13 |
| 34-21 | Pharmacy | 3-1-3-34-34-21 | x | 05-Feb-13 |
| 43-21 | PMV | 4-1-2-43-43-21 | x | 15-Dec-12 |
| 44-21 | PMV | 5-1-2-44-44-21 | x | 15-Dec-12 |
| 28-21 | Pharmacy | 9-1-2-28-28-21 | x | 05-Feb-13 |
| 1-21B | Pharmacy | 12-2-6-1-01-21B | x | 08-Feb-13 |
| 9-17 | Pharmacy | 3-1-3-9-09-17 | x | 09-Feb-13 |
| 10-17B | Pharmacy | 3-1-3-10-10-17B | x | 06-Feb-13 |
| 21-17 | Pharmacy | 3-1-3-21-21-17 | x | 07-Feb-13 |
| 33-17B | Pharmacy | 3-1-3-33-33-17B | x | 07-Feb-13 |
| 34-67 | Pharmacy | 3-1-3-34-34-67 | x | 05-Feb-13 |
| 3-11 | Pharmacy | 3-2-3-3-03-11 | x | 08-Feb-13 |
| 43-67 | PMV | 4-1-2-43-43-67 | x | 15-Dec-12 |
| 51-67 | PMV | 5-1-2-51-51-67 | x | 15-Dec-12 |
| 10-17 | PMV | 9-1-1-10-10-17 | x | 06-Feb-13 |

| | | | | |
|---|---|---|---|---|
| 25-17 | Pharmacy | 9-1-2-25-25-17 | x | 06-Feb-13 |
| 1-17 | Pharmacy | 12-2-6-1-01-17 | x | 08-Feb-13 |
| 5-17 | Pharmacy | 12-2-6-5-05-17 | x | 09-Feb-13 |
| 10-17B | Pharmacy | 14-3-1-10-10-17B | x | 05-Jan-13 |
| 14-17 | Pharmacy | 14-3-1-14-14-17 | x | 05-Jan-13 |
| 21-17 | Pharmacy | 14-3-1-21-21-17 | x | 09-Feb-13 |
| 8-67 | Pharmacy | 15-2-4-8-08-67 | x | 06-Feb-13 |
| 14-58 | PMV | 15-2-4-14-14-58 | x | 05-Feb-13 |
| 27-67 | PMV | 15-2-4-27-27-67 | x | 07-Feb-13 |
| 3-17 | Pharmacy | 17-1-3-3-03-17 | x | 06-Feb-13 |
| 10-58 | Pharmacy | 3-1-3-10-10-58 | x | 06-Feb-13 |
| 26-58 | PMV | 3-1-3-26-26-58 | x | 08-Feb-13 |
| 34-58 | Pharmacy | 3-1-3-34-34-58 | x | 05-Feb-13 |
| 12-58 | Pharmacy | 3-2-5-12-12-58 | x | 11-Feb-13 |
| 13-58 | Pharmacy | 4-1-2-13-13-58 | x | 15-Dec-12 |
| 10-58 | Pharmacy | 4-2-1-10-10-58 | x | 07-Feb-13 |
| 8-58 | Pharmacy | 4-2-3-8-08-58 | x | 05-Feb-13 |
| 16-58 | Pharmacy | 4-2-3-16-16-58 | x | 06-Feb-13 |
| 38-27 | Pharmacy | 4-2-3-38-38-27 | x | 05-Feb-13 |
| 6-58 | PMV | 9-1-1-6-06-58 | x | 07-Feb-13 |
| 16-58 | Pharmacy | 9-1-1-16-16-58 | x | 06-Feb-13 |
| 15-58 | PMV | 12-2-1-15-15-58 | x | 06-Feb-13 |
| 1-58 | Pharmacy | 12-2-6-1-01-58 | x | 08-Feb-13 |
| 14-58 | PMV | 15-2-4-14-14-58 | x | 06-Feb-13 |
| 7-58 | Pharmacy | 15-2-4-7-07-58 | x | 06-Feb-13 |
| 8-58 | Pharmacy | 15-2-4-8-08-58 | x | 06-Feb-13 |
| 11-58 | Pharmacy | 15-2-4-11-11-58 | x | 05-Feb-13 |
| 27-58 | PMV | 15-2-4-27-27-58 | x | 07-Feb-13 |
| 44-58 | PMV | 15-2-4-44-44-58 | x | 05-Feb-13 |
| 83-58 | Pharmacy | 16-3-1-83-83-58 | x | 05-Feb-13 |
| 10-67A | Pharmacy | 3-1-3-10-10-67A | x | 06-Feb-13 |
| 11-67A | Pharmacy | 3-1-3-11-11-67A | x | 07-Feb-13 |
| 12-67B | Pharmacy | 3-2-5-12-12-67B | x | 11-Feb-13 |
| 1-67 | Pharmacy | 12-2-6-1-01-67 | x | 08-Feb-13 |

| HPLC - LSHTM | CDC results | GT MS | genname2 |
|---|---|---|---|
| **107.7** | | Yes | Lumefantrine |
| **105.1** | | Yes | Lumefantrine |
| **110.5** | | Yes | Lumefantrine |
| **91.6** | | Yes | Lumefantrine |
| **107.8** | | Yes | Lumefantrine |
| **100.6** | | Yes | Lumefantrine |
| **97.2** | | | Lumefantrine |
| **97.7** | | Yes | Lumefantrine |
| **108.6** | | Yes | Lumefantrine |
| **94.6** | | | Lumefantrine |
| **94.6** | | Yes | Lumefantrine |
| **86.1** | | Yes | Lumefantrine |
| **89.5** | | | Lumefantrine |
| **91.7** | | | Lumefantrine |
| **84.5** | | | Lumefantrine |
| **94.6** | | Yes | Lumefantrine |
| **98.8** | | Yes | Lumefantrine |
| **102.0** | | Yes | Lumefantrine |
| **98.2** | | Yes | Lumefantrine |
| **107.4** | | Yes | Lumefantrine |
| **92.3** | | Yes | Lumefantrine |
| **97.2** | | Yes | Lumefantrine |
| **93.7** | | Yes | Lumefantrine |
| **99.3** | | Yes | Lumefantrine |
| **111.9** | | Yes | Lumefantrine |
| **Not detectable** | **Not detectable** | | Lumefantrine |
| **105.5** | | Yes | Lumefantrine |
| **99.0** | | Yes | Lumefantrine |
| **91.8** | | Yes | Lumefantrine |
| **97.2** | | Yes | Lumefantrine |
| **90.0** | | Yes | Lumefantrine |
| **99.0** | | Yes | Lumefantrine |
| **97.4** | | Yes | Lumefantrine |
| **108.9** | | Yes | Lumefantrine |
| **94.7** | | Yes | Lumefantrine |
| 90.9 | | Yes | Lumefantrine |
| **96.6** | | | Lumefantrine |
| **108.2** | | | Lumefantrine |
| **102.2** | | | Lumefantrine |
| **92.0** | | | Lumefantrine |
| **100.1** | | | Lumefantrine |
| **92.9** | | | Lumefantrine |
| **101.7** | | | Lumefantrine |
| **91.1** | | | Lumefantrine |
| **89.8** | | Yes | Lumefantrine |
| **102.2** | | Yes | Lumefantrine |

| | | |
|---|---|---|
| **99.0** | | Lumefantrine |
| **91.6** | | Lumefantrine |
| **91.0** | | Lumefantrine |
| **95.6** | Yes | Lumefantrine |
| **91.1** | | Lumefantrine |
| **97.2** | Yes | Lumefantrine |
| **91.3** | | Lumefantrine |
| **98.7** | | Lumefantrine |
| **91.1** | | Lumefantrine |
| 95 | | Lumefantrine |
| **90.0** | | Lumefantrine |
| **93.0** | Yes | Lumefantrine |
| **93.3** | | Lumefantrine |
| **91.6** | | Lumefantrine |
| **94.9** | Yes | Lumefantrine |
| **93.0** | | Lumefantrine |
| **94.2** | Yes | Lumefantrine |
| **96.3** | | Lumefantrine |
| **97.5** | | Lumefantrine |
| **89.1** | | Lumefantrine |
| **97.6** | Yes | Lumefantrine |
| **93.1** | Yes | Lumefantrine |
| **85.6** | Yes | Lumefantrine |
| **93.6** | Yes | Lumefantrine |
| **Not detectable** | | Lumefantrine |
| **92.3** | Yes | Lumefantrine |
| **90.9** | | Lumefantrine |
| **109.7** | Yes | Lumefantrine |
| **98.9** | Yes | Lumefantrine |
| **87.2** | Yes | Lumefantrine |
| **92.5** | Yes | Lumefantrine |
| 94.9 | Yes | Lumefantrine |
| 101.2 | Yes | Lumefantrine |
| 92.2 | | Lumefantrine |
| 88 | Yes | Lumefantrine |
| 91.6 | Yes | Lumefantrine |
| **96.3** | **Yes** | Lumefantrine |
| **97.2** | **Yes** | Lumefantrine |
| **93.8** | **Yes** | Lumefantrine |
| **85.3** | **Yes** | Lumefantrine |
| **89.9** | **Yes** | Lumefantrine |
| **91.6** | | Lumefantrine |
| **91.0** | | Lumefantrine |
| **95.6** | | Lumefantrine |
| **91.1** | | Lumefantrine |
| **97.2** | | Lumefantrine |
| **91.3** | yes | Lumefantrine |
| **98.7** | | Lumefantrine |
| **91.1** | | Lumefantrine |
| **Degraded** | | Lumefantrine |

| | | | | |
|---|---|---|---|---|
| **88.6** | | | yes | Lumefantrine |
| **90.4** | | | | Lumefantrine |
| **93.3** | | | yes | Lumefantrine |
| **91.6** | | | | Lumefantrine |
| **94.9** | | | | Lumefantrine |
| **93.0** | | | | Lumefantrine |
| **94.2** | | | | Lumefantrine |
| **96.3** | | | | Lumefantrine |
| **97.5** | | | | Lumefantrine |
| **89.1** | | | | Lumefantrine |
| 91.8 | | | Yes | Lumefantrine |
| 87.4 | | | | Lumefantrine |
| 33.7 | | 15 | | Lumefantrine |
| 89.9 | | | | Lumefantrine |
| **Degraded** | | | | Lumefantrine |
| 91.8 | | | Yes | Lumefantrine |
| 95.9 | | | Yes | Lumefantrine |
| 91.2 | | | Yes | Lumefantrine |
| **96.1** | | | | Lumefantrine |
| **98.1** | | | | Lumefantrine |
| **102.9** | | | | Lumefantrine |
| **90.8** | | | | Lumefantrine |
| **95.6** | | | Yes | Lumefantrine |
| **95.1** | | | | Lumefantrine |
| **91.6** | | | Yes | Lumefantrine |
| **90.1** | | | | Lumefantrine |
| **98.4** | | | | Lumefantrine |
| **96.8** | | | Yes | Lumefantrine |
| **94.7** | | | Yes | Lumefantrine |
| **94.2** | | | | Lumefantrine |
| **96.3** | | | | Lumefantrine |
| **97.5** | | | | Lumefantrine |
| **89.1** | | | | Lumefantrine |
| **101.1** | | | | Sulfadoxine |
| **Not detectable** | **Not detectable** | | | Amodiaquine |
| 99 | 99.7 | | | Amodiaquine |
| 92 | 88 | | | Amodiaquine |
| 102 | 102.8 | | | Amodiaquine |
| **96.7** | 98.4 | | | Amodiaquine |
| **93.0** | no sample | | | Amodiaquine |
| **95.2** | 96.6 | | | Amodiaquine |
| **90.7** | 91 | | | Amodiaquine |
| **95.1** | 92.3 | | Yes | Amodiaquine |
| **98.6** | 57.3 | | yes | Amodiaquine |
| **91.6** | 88 | | yes | Amodiaquine |
| 99.8 | 99.7 | | yes | Amodiaquine |
| 99.5 | 99 | | yes | Amodiaquine |
| **96.4** | 94.9 | | Yes | Amodiaquine |
| **102.5** | | | | Amodiaquine |

| | | | |
|---|---|---|---|
| **101.1** | | | Amodiaquine |
| 97.8 | | Yes | Amodiaquine |
| **98.2** | 94.7 | | Amodiaquine |
| **89.6** | not detected | | Amodiaquine |
| 102.5 | 87.3 | Yes | Amodiaquine |
| 89.5 | | | Amodiaquine |
| 99.6 | | Yes | Amodiquine |
| 107.9 | 99 | Yes | Mefloquine |
| 101.4 | 97.7 | Yes | Mefloquine |
| 90.5 mono | 94.7/- | | |
| **89.6** | 89.2 | | |
| 101.1 | 101.6 | Yes | Amodiaquine |
| 96.7 | 22.3 | | Amodiaquine |
| 93.0 | 100.4 | | Amodiaquine |
| 93 | 96.7 | Yes | Mefloquine |
| 83.9 | 96.5 | Yes | Mefloquine |
| 91.6 | 96.2 | Yes | Mefloquine |
| 86.4 | 97.1 | Yes | Mefloquine |
| 98.1 | 98 | Yes | Mefloquine |
| 87.5 | 97.1 | Yes | Mefloquine |
| 86.4 | 96.4 | Yes | Mefloquine |
| **Not detectable** | | | Mefloquine |
| 96.1 | 69.7 | | Sulfadoxine |
| **Degraded** | 53.9 | | Sulfadoxine |
| **Degraded** | 67.1 | | Sulfadoxine |
| **96.5** | 93.6 | | Sulfamethoxypyrazine |
| **99.3** | | | Sulfamethoxypyrazine |
| **98.6** | 92.5 | yes | Sulfamethoxypyrazine |
| **96.3** | 93.1 | | Sulfamethoxypyrazine |
| **99.5** | 90.4 | | Sulfamethoxypyrazine |
| **89.6** | 87.2 | Yes | Piperaquine phosphate |
| **100.4** | | Yes | Piperaquine phosphate |
| **Not detectable** | **Not detectable** | | Piperaquine phosphate |
| **99.6** | 71.8 | Yes | Piperaquine phosphate |
| **97.6** | 74.2 | Yes | Piperaquine phosphate |
| **100.5** | 62.8 | Yes | Piperaquine phosphate |
| **106.5** | 67.1 | Yes | Piperaquine phosphate |
| **Not detectable** | **Not detectable** | | Piperaquine phosphate |
| **91.2** | 78.6 | yes | Piperaquine phosphate |
| **97.6** | 71.3 | yes | Piperaquine phosphate |
| **96.8** | | yes | Piperaquine phosphate |
| **91.1** | 74.8 | yes | Piperaquine phosphate |
| **94.8** | 90.1 | yes | Piperaquine phosphate |
| **93.4** | 72.4 | yes | Piperaquine phosphate |
| **96.5** | 73.2 | yes | Piperaquine phosphate |
| **89.3** | | yes | Piperaquine phosphate |
| **99.1** | 96.4 | yes | Piperaquine phosphate |
| **94.6** | 97.6 | yes | Piperaquine phosphate |

| | | | |
|---|---|---|---|
| **104.8** | 98 | yes | Piperaquine phosphate |
| **95.8** | 94.2 | yes | Piperaquine phosphate |
| **80.1** | 100.1 | yes | Piperaquine phosphate |
| **95.6** | 95.3 | yes | Piperaquine phosphate |
| **96.8** | 97.4 | yes | Piperaquine phosphate |
| **89.3** | 96.8 | yes | |
| **79.3** | 72.5 | yes | |

| **genname1** | **strengthgen1** | **Weight (g)** | **Width (mm)** |
|---|---|---|---|
| artemether | 20 | 0.2929 | 9.28 |
| artemether | 20 | 0.2953 | 9.22 |
| artemether | 20 | 0.2929 | 9.24 |
| artemether | 20 | 0.2446 | 9.15 |
| artemether | 20 | 0.2468 | 9.10 |
| artemether | 20 | 0.2474 | 9.10 |
| artemether | 20 | 0.2473 | 9.18 |
| artemether | 20 | 0.2484 | 9.17 |
| artemether | 20 | 0.2445 | 9.18 |
| artemether | 20 | 0.2517 | 9.17 |
| artemether | 20 | 0.2488 | 9.15 |
| artemether | 20 | 0.2489 | 9.15 |
| artemether | 20 | 0.2443 | 9.08 |
| artemether | 20 | 0.2475 | 9.17 |
| artemether | 20 | 0.2513 | 9.18 |
| artemether | 20 | 0.2487 | 9.16 |
| artemether | 20 | 0.2466 | 9.16 |
| artemether | 20 | 0.2458 | 9.11 |
| artemether | 20 | 0.2469 | 9.13 |
| artemether | 20 | 0.2492 | 9.20 |
| artemether | 20 | 0.2510 | 9.15 |
| artemether | 20 | 0.2490 | 9.10 |
| artemether | 20 | 0.2517 | 9.14 |
| artemether | 20 | 0.2493 | 9.10 |
| artemether | 20 | 0.2485 | 9.16 |
| artemether | 20 | 0.2506 | 9.19 |
| artemether | 20 | 0.2501 | 9.12 |
| artemether | 20 | 0.2485 | 9.18 |
| artemether | 20 | 0.2496 | 9.08 |
| artemether | 20 | 0.2482 | 9.13 |
| artemether | 20 | 0.2532 | 9.17 |
| artemether | 20 | 0.2457 | 9.09 |
| artemether | 20 | 0.2472 | 9.09 |
| artemether | 20 | 0.2478 | 9.06 |
| artemether | 20 | 0.2492 | 9.09 |
| artemether | 20 | 0.2499 | 9.06 |
| artemether | 20 | 0.2521 | 9.08 |
| artemether | 20 | 0.2464 | 9.09 |
| artemether | 20 | 0.2463 | 9.15 |

| | | | | |
|---|---|---|---|---|
| artemether | 20 | | 0.2473 | 9.04 |
| artemether | 20 | | 0.2474 | 9.04 |
| artemether | 20 | | 0.2485 | 9.06 |
| artemether | 20 | | 0.2498 | 9.17 |
| artemether | 20 | | 0.2513 | 9.18 |
| artemether | 20 | | 0.2466 | 9.13 |
| artemether | 20 | | 0.2478 | 9.17 |
| artemether | 20 | | 0.2461 | 9.15 |
| artemether | 20 | | 0.2522 | 9.16 |
| artemether | 20 | | 0.2468 | 9.15 |
| artemether | 20 | | 0.2498 | 9.14 |
| artemether | 20 | | 0.2531 | 9.12 |
| artemether | 20 | | 0.2506 | 9.08 |
| artemether | 20 | | 0.2472 | 9.05 |
| artemether | 20 | | 0.2501 | 9.21 |
| artemether | 20 | | 0.2473 | 9.04 |
| artemether | 20 | | 0.2459 | 9.04 |
| artemether | 20 | | 0.2531 | 9.06 |
| artemether | 20 | | 0.2493 | 9.17 |
| artemether | 20 | | 0.2520 | 9.13 |
| artemether | 20 | | 0.2519 | 9.05 |
| artemether | 20 | | 0.2499 | 9.14 |
| artemether | 20 | | 0.2493 | 9.04 |
| artemether | | 20 | 0.2534 | 9.10 |
| artemether | 20 | | 0.2472 | 9.10 |
| artemether | 20 | | 0.2468 | 9.10 |
| artemether | 20 | | 0.2504 | 9.14 |
| artemether | 20 | | 0.2452 | 9.09 |
| artemether | 20 | | 0.2518 | 9.09 |
| artemether | 20 | | 0.2503 | 9.13 |
| artemether | 20 | | 0.2444 | 9.13 |
| artemether | 20 | | 0.2500 | 9.09 |
| artemether | 20 | | 0.2467 | 9.15 |
| artemether | 20 | | 0.2510 | 9.11 |
| artemether | 20 | | 0.2490 | 9.14 |
| artemether | 20 | | 0.2533 | 9.16 |
| artemether | 20 | | 0.2479 | 9.13 |
| artemether | 20 | | 0.2484 | 9.15 |
| artemether | 20 | | 0.2510 | 9.20 |
| artemether | 20 | | 0.2498 | 9.14 |
| artemether | 20 | | 0.2457 | 9.11 |
| artemether | 20 | | 0.2465 | 9.17 |
| artemether | 20 | | 0.2486 | 9.12 |
| artemether | 20 | | 0.2460 | 9.13 |
| artemether | 20 | | 0.2488 | 9.13 |
| artemether | 20 | | 0.2483 | 9.12 |
| artemether | 20 | | 0.2486 | 9.15 |
| artemether | 20 | | 0.2510 | 9.10 |
| artemether | 20 | | 0.2474 | 9.18 |
| artemether | 20 | | 0.2499 | 9.16 |

| | | | |
|---|---|---|---|
| artemether | 20 | 0.2473 | 9.10 |
| artemether | 20 | 0.2486 | 9.10 |
| artemether | 20 | 0.2449 | 9.11 |
| artemether | 20 | 0.2474 | 9.15 |
| artemether | 20 | 0.2497 | 9.15 |
| artemether | 20 | 0.2505 | 9.15 |
| artemether | 20 | 0.2483 | 9.12 |
| artemether | 20 | 0.2494 | 9.13 |
| artemether | 20 | 0.2461 | 9.12 |
| artemether | 20 | 0.2474 | 9.16 |
| artemether | 20 | 0.2471 | 9.14 |
| artemether | 20 | 0.2475 | 9.13 |
| artemether | 20 | 0.2455 | 9.11 |
| artemether | 20 | 0.2483 | 9.15 |
| artemether | 20 | 0.2528 | 9.19 |
| artemether | 20 | 0.2542 | 9.11 |
| artemether | 20 | 0.2509 | 9.18 |
| artemether | 20 | 0.2438 | 9.08 |
| artemether | 20 | 0.2464 | 9.09 |
| artemether | 20 | 0.2516 | 9.08 |
| artemether | 20 | 0.2447 | 9.12 |
| artemether | 20 | 0.2475 | 9.15 |
| artemether | 20 | 0.2466 | 9.16 |
| artemether | 20 | 0.2447 | 9.06 |
| artemether | 20 | 0.2451 | 9.13 |
| artemether | 20 | 0.2432 | 9.06 |
| artemether | 20 | 0.2489 | 9.18 |
| artemether | 20 | 0.2496 | 9.17 |
| artemether | 20 | 0.2442 | 9.10 |
| artemether | 20 | 0.2481 | 9.06 |
| artemether | 20 | 0.2511 | 9.13 |
| artemether | 20 | 0.2492 | 9.10 |
| artemether | 20 | 0.2533 | 9.17 |
| artemether | 20 | 0.2528 | 9.16 |
| artemether | 20 | 0.2457 | 9.09 |
| artemether | 20 | 0.2485 | 9.10 |
| artemether | 20 | 0.2440 | 9.12 |
| artemether | 20 | 0.2476 | 9.15 |
| artemether | 20 | 0.2453 | 9.14 |
| artemether | 20 | 0.2500 | 9.12 |
| artemether | 20 | 0.2507 | 9.11 |
| artemether | 20 | 0.2470 | 9.13 |
| artemether | 20 | 0.2469 | 9.16 |
| artemether | 20 | 0.2451 | 9.08 |
| artemether | 20 | 0.2484 | 9.15 |
| artemether | 20 | 0.2448 | 9.10 |
| artemether | 20 | 0.2448 | 9.07 |
| artemether | 20 | 0.2491 | 9.08 |
| artemether | 20 | 0.2483 | 9.12 |
| artemether | 20 | 0.2450 | 9.12 |

| | | | | |
|---|---|---|---|---|
| artemether | 20 | | 0.2470 | 9.14 |
| artemether | 20 | | 0.2485 | 9.18 |
| artemether | 20 | | 0.2492 | 9.12 |
| artemether | 20 | | 0.2486 | 9.12 |
| artemether | 20 | | 0.2469 | 9.05 |
| artemether | 20 | | 0.2496 | 9.10 |
| artemether | 20 | | 0.2482 | 9.07 |
| artemether | 20 | | 0.2462 | 9.15 |
| artemether | 20 | | 0.2531 | 9.10 |
| artemether | 20 | | 0.2489 | 9.15 |
| artemether | 20 | | 0.2476 | 9.06 |
| artemether | 20 | | 0.2461 | 9.04 |
| artemether | 20 | | 0.2444 | 9.06 |
| artemether | 20 | | 0.2485 | 9.15 |
| artemether | 20 | | 0.2477 | 9.16 |
| artemether | 20 | | 0.2460 | 9.05 |
| artemether | 20 | | 0.2468 | 9.07 |
| artemether | 20 | | 0.2467 | 9.11 |
| artemether | 20 | | 0.3303 | 9.65 |
| artemether | 20 | | 0.3272 | 9.65 |
| artemether | 20 | | 0.3326 | 9.63 |
| artemether | 20 | | 0.3201 | 9.58 |
| artemether | 20 | | 0.3343 | 9.64 |
| artemether | 20 | | 0.3226 | 9.67 |
| artemether | 20 | | 0.3341 | 9.61 |
| artemether | 20 | | 0.3313 | 9.65 |
| artemether | 20 | | 0.3282 | 9.64 |
| artemether | 20 | | 0.3308 | 9.66 |
| artemether | 20 | | 0.3336 | 9.71 |
| artemether | 20 | | 0.3292 | 9.62 |
| artemether | 20 | | 0.3333 | 9.64 |
| artemether | 20 | | 0.3263 | 9.68 |
| artemether | | 20 | 0.3337 | 9.65 |
| artemether | 20 | | 0.3000 | 9.25 |
| artemether | 20 | | 0.2802 | 9.22 |
| artemether | 20 | | 0.2816 | 9.29 |
| artemether | 80 | | 0.2913 | 9.25 |
| artemether | 20 | | 0.3056 | 9.25 |
| artemether | 20 | | 0.2957 | 9.35 |
| artemether | 20 | | 0.2966 | 9.27 |
| artemether | 20 | | 0.2968 | 9.23 |
| artemether | 20 | | 0.2997 | 9.28 |
| artemether | 20 | | 0.2877 | 9.28 |
| artemether | 20 | | 0.2753 | 9.27 |
| artemether | 20 | | 0.2382 | 9.21 |
| artemether | 20 | | 0.3495 | 9.99 |
| artemether | 20 | | 0.3527 | 9.93 |
| artemether | 20 | | 0.3571 | 9.86 |
| artemether | 20 | | 0.3483 | 9.88 |
| artemether | 20 | | 0.3564 | 9.89 |

| | | | |
|---|---|---|---|
| artemether | 20 | 0.3564 | 9.88 |
| artemether | 20 | 0.3509 | 9.87 |
| artemether | 20 | 0.3543 | 9.93 |
| artemether | 20 | 0.3532 | 9.88 |
| artemether | 20 | 0.3588 | 9.96 |
| artemether | 20 | 0.3571 | 9.99 |
| artemether | 20 | 0.3626 | 9.98 |
| artemether | 20 | 0.3492 | 10.81 |
| artemether | 20 | 0.2426 | 9.08 |
| artemether | 20 | 0.2404 | 9.06 |
| artemether | 20 | 0.2418 | 9.08 |
| artemether | 20 | 0.2374 | 9.07 |
| artemether | 20 | 0.2333 | 9.15 |
| artemether | 20 | 0.2435 | 9.07 |
| artemether | 20 | 0.2394 | 9.11 |
| artemether | 20 | 0.3401 | 9.12 |
| artemether | 20 | 0.2348 | 9.10 |
| artemether | 20 | 0.2390 | 9.04 |
| artemether | 20 | 0.2528 | 9.08 |
| artemether | 20 | 0.2598 | 9.37 |
| artemether | 20 | 0.2616 | 9.23 |
| artemether | 20 | 0.2639 | 9.23 |
| artemether | 20 | 0.2631 | 9.25 |
| artemether | 20 | 0.2645 | 9.25 |
| artemether | 20 | 0.2421 | 9.05 |
| artemether | 20 | 0.2424 | 9.04 |
| artemether | 20 | 0.2376 | 9.05 |
| artemether | 20 | 0.2436 | 9.03 |
| artemether | 20 | 0.2413 | 9.05 |
| artemether | 20 | 0.2423 | 9.11 |
| artemether | 20 | 0.2437 | 9.05 |
| artemether | 20 | 0.2436 | 9.05 |
| artemether | 20 | 0.2407 | 9.04 |
| artemether | 20 | 0.2412 | 9.05 |
| artemether | 20 | 0.2404 | 9.03 |
| artemether | 20 | 0.2411 | 9.03 |
| artemether | 20 | 0.2402 | 9.04 |
| artemether | 20 | 0.2453 | 9.08 |
| artemether | 20 | 0.2433 | 9.03 |
| artemether | 20 | 0.2412 | 9.05 |
| artemether | 20 | 0.2439 | 9.05 |
| artemether | 20 | 0.2401 | 9.04 |
| artemether | 20 | 0.2414 | 9.04 |
| artemether | 20 | 0.2425 | 9.05 |
| artemether | 20 | 0.2418 | 9.04 |
| artemether | 20 | 0.2423 | 9.09 |
| artemether | 20 | 0.2409 | 9.06 |
| artemether | 20 | 0.2421 | 9.05 |
| artemether | 20 | 0.2412 | 9.05 |
| artemether | 20 | 0.2396 | 9.05 |

| | | | |
|---|---|---|---|
| artemether | 20 | 0.2406 | 9.06 |
| artemether | 20 | 0.2429 | 9.05 |
| artemether | 20 | 0.2432 | 9.06 |
| artemether | 20 | 0.2429 | 9.06 |
| artemether | 20 | 0.2424 | 9.05 |
| artemether | 20 | 0.2405 | 9.04 |
| artemether | 20 | 0.2433 | 9.05 |
| artemether | 20 | 0.2393 | 9.06 |
| artemether | 20 | 0.2405 | 9.08 |
| artemether | 20 | 0.2435 | 9.07 |
| artemether | 20 | 0.2428 | 9.06 |
| artemether | 20 | 0.2410 | 9.03 |
| artemether | 20 | 0.2410 | 9.06 |
| artemether | 20 | 0.2401 | 9.05 |
| artemether | 20 | 0.3466 | 9.04 |
| artemether | 20 | 0.2435 | 9.02 |
| artemether | 20 | 0.2401 | 9.10 |
| artemether | 20 | 0.2427 | 9.02 |
| artemether | 20 | 0.2369 | 8.99 |
| artemether | 20 | 0.2432 | 8.99 |
| artemether | 20 | 0.2402 | 9.01 |
| artemether | 20 | 0.2383 | 8.98 |
| artemether | 20 | 0.2418 | 9.01 |
| artemether | 20 | 0.2447 | 9.01 |
| artemether | 20 | 0.2399 | 9.01 |
| artemether | 20 | 0.2389 | 8.99 |
| artemether | 20 | 0.2401 | 9.06 |
| artemether | 20 | 0.2432 | 9.08 |
| artemether | 20 | 0.2434 | 9.08 |
| artemether | 20 | 0.2398 | 9.11 |
| artemether | 20 | 0.2381 | 9.12 |
| artemether | 20 | 0.2396 | 9.10 |
| artemether | 20 | 0.2397 | 9.08 |
| artemether | 20 | 0.2397 | 9.10 |
| artemether | 20 | 0.2399 | 9.11 |
| artemether | 20 | 0.2394 | 9.11 |
| artemether | 20 | 0.2436 | 9.08 |
| artemether | 20 | 0.2381 | 9.06 |
| artemether | 20 | 0.2403 | 9.07 |
| artemether | 20 | 0.2438 | 9.04 |
| artemether | 20 | 0.2400 | 9.08 |
| artemether | 20 | 0.2406 | 9.12 |
| artemether | 20 | 0.2385 | 9.06 |
| artemether | 20 | 0.2391 | 9.11 |
| artemether | 20 | 0.2417 | 9.08 |
| artemether | 20 | 0.2400 | 9.06 |
| artemether | 20 | 0.2469 | 9.07 |
| artemether | 20 | 0.3240 | 9.47 |
| artemether | 20 | 0.2446 | 9.10 |
| artemether | 20 | 0.2395 | 9.07 |

| | | | |
|---|---|---|---|
| artemether | 20 | 0.2457 | 9.13 |
| artemether | 20 | 0.2420 | 9.09 |
| artemether | 20 | 0.2442 | 9.06 |
| artemether | 20 | 0.2438 | 9.06 |
| artemether | 20 | 0.2368 | 9.06 |
| artemether | 20 | 0.2412 | 9.12 |
| artemether | 20 | 0.2450 | 9.10 |
| artemether | 20 | 0.2480 | 9.08 |
| artemether | 20 | 0.2420 | 9.08 |
| artemether | 20 | 0.2394 | 9.09 |
| artemether | 20 | 0.2381 | 9.10 |
| artemether | 20 | 0.2406 | 9.04 |
| artemether | 20 | 0.2438 | 9.05 |
| artemether | 20 | 0.2410 | 9.04 |
| artemether | 20 | 0.2421 | 9.08 |
| artemether | 20 | 0.2435 | 9.07 |
| artemether | 20 | 0.2410 | 9.05 |
| artemether | 20 | 0.2449 | 9.06 |
| artemether | 20 | 0.2440 | 9.10 |
| artemether | 20 | 0.2387 | 9.06 |
| artemether | 20 | 0.2410 | 9.03 |
| artemether | 20 | 0.2425 | 9.04 |
| artemether | 20 | 0.2435 | 9.08 |
| artemether | 20 | 0.2428 | 9.03 |
| artemether | 20 | 0.2430 | 9.03 |
| artemether | 20 | 0.2419 | 9.09 |
| artemether | 20 | 0.2444 | 9.07 |
| artemether | 20 | 0.2417 | 9.08 |
| artemether | 20 | 0.2402 | 9.10 |
| artemether | 20 | 0.2454 | 9.04 |
| artemether | 20 | 0.2419 | 9.06 |
| artemether | 20 | 0.3099 | 9.50 |
| artemether | 20 | 0.2465 | 9.05 |
| artemether | 20 | 0.2333 | 9.07 |
| artemether | 20 | 0.2465 | 9.05 |
| artemether | 20 | 0.2433 | 9.07 |
| artemether | 20 | 0.2448 | 9.05 |
| artemether | 20 | 0.2410 | 9.08 |
| artemether | 20 | 0.2453 | 9.05 |
| artemether | 20 | 0.2447 | 9.11 |
| artemether | 20 | 0.2399 | 9.04 |
| artemether | 20 | 0.2863 | 9.15 |
| artemether | 20 | 0.2884 | 9.14 |
| artemether | 20 | 0.2923 | 9.13 |
| artemether | 20 | 0.2871 | 9.16 |
| artemether | 20 | 0.2864 | 9.18 |
| artemether | 20 | 0.2861 | 9.16 |
| artemether | 20 | 0.2885 | 9.14 |
| artemether | 20 | 0.2873 | 9.10 |
| artemether | 20 | 0.2887 | 9.18 |

| | | | |
|---|---|---|---|
| artemether | 20 | 0.2860 | 9.16 |
| artemether | 20 | 0.2850 | 9.11 |
| artemether | 20 | 0.2870 | 9.16 |
| artemether | 20 | 0.2856 | 9.14 |
| artemether | 20 | 0.2891 | 9.15 |
| artemether | 20 | 0.2855 | 9.14 |
| artemether | 20 | 0.2572 | 9.16 |
| artemether | 20 | 0.2856 | 9.13 |
| artemether | 20 | 0.2862 | 9.13 |
| artemether | 20 | 0.2862 | 9.15 |
| artemether | 20 | 0.2865 | 9.16 |
| artemether | 20 | 0.2846 | 9.12 |
| artemether | 20 | 0.2884 | 9.19 |
| artemether | 20 | 0.2880 | 9.13 |
| artemether | 20 | 0.2897 | 9.17 |
| artemether | 20 | 0.2854 | 9.15 |
| artemether | 20 | 0.2855 | 9.19 |
| artemether | 20 | 0.2882 | 9.14 |
| artemether | 20 | 0.2857 | 9.14 |
| artemether | 20 | 0.2863 | 9.16 |
| artemether | 20 | 0.2874 | 9.16 |
| artemether | 20 | 0.2896 | 9.15 |
| artemether | 20 | 0.2859 | 9.11 |
| artemether | 20 | 0.2939 | 9.17 |
| artemether | 20 | 0.2855 | 9.14 |
| artemether | 20 | 0.2893 | 9.13 |
| artemether | 20 | 0.2890 | 9.13 |
| artemether | 20 | 0.2880 | 9.12 |
| artemether | 20 | 0.2860 | 9.13 |
| artemether | 20 | 0.2902 | 9.14 |
| artemether | 20 | 0.2856 | 9.13 |
| artemether | 20 | 0.2882 | 9.11 |
| artemether | 20 | 0.2894 | 9.15 |
| artemether | 20 | 0.2880 | 9.13 |
| artemether | 20 | 0.2886 | 9.18 |
| artemether | 20 | 0.2824 | 9.19 |
| artemether | 20 | 0.2832 | 9.10 |
| artemether | 20 | 0.2860 | 9.09 |
| artemether | 20 | 0.2865 | 9.07 |
| artemether | 20 | 0.2831 | 9.10 |
| artemether | 20 | 0.2892 | 9.11 |
| artemether | 20 | 0.2875 | 9.07 |
| artemether | 20 | 0.2881 | 9.16 |
| artemether | 20 | 0.2894 | 9.14 |
| artemether | 20 | 0.2859 | 9.11 |
| artemether | 20 | 0.2845 | 9.10 |
| artemether | 20 | 0.2880 | 9.12 |
| artemether | 20 | 0.2886 | 9.12 |
| artemether | 20 | 0.2886 | 9.12 |
| artemether | 20 | 0.2881 | 9.13 |

| | | | | |
|---|---|---|---|---|
| artemether | 20 | | 0.2899 | 9.13 |
| artemether | 20 | | 0.2888 | 9.15 |
| artemether | 20 | | 0.2864 | 9.07 |
| artemether | 20 | | 0.2864 | 9.09 |
| artemether | 20 | | 0.2854 | 9.00 |
| artemether | 20 | | 0.2865 | 9.10 |
| artemether | 20 | | 0.2918 | 9.05 |
| artemether | 20 | | 0.2887 | 9.11 |
| artemether | 20 | | 0.2875 | 9.09 |
| artemether | 20 | | 0.2956 | 9.31 |
| artemether | 20 | | 0.3066 | 9.26 |
| artemether | 20 | | 0.2942 | 9.28 |
| artemether | 20 | | 0.2488 | 9.27 |
| artemether | 20 | | 0.2447 | 9.16 |
| artemether | 20 | | 0.2471 | 9.15 |
| artemether | 20 | | 0.2466 | 9.16 |
| artemether | 20 | | 0.2453 | 9.18 |
| artemether | 20 | | 0.2501 | 9.12 |
| artemether | 20 | | 0.2477 | 9.16 |
| artemether | 20 | | 0.2517 | 9.17 |
| artemether | 20 | | 0.2448 | 9.20 |
| artemether | 20 | | 0.2472 | 9.24 |
| artemether | 20 | | 0.2452 | 9.22 |
| artemether | 20 | | 0.2451 | 9.14 |
| artemether | 20 | | 0.2450 | 9.14 |
| artemether | 20 | | 0.2413 | 9.09 |
| artemether | 20 | | 0.2508 | 9.13 |
| artemether | 20 | | 0.2406 | 9.11 |
| artemether | 20 | | 0.2466 | 9.09 |
| artemether | 20 | | 0.2402 | 9.11 |
| artemether | 20 | | 0.2408 | 9.08 |
| artemether | 20 | | 0.2441 | 9.16 |
| artemether | 20 | | 0.2397 | 9.11 |
| artemether | 20 | | 0.2348 | 9.12 |
| artemether | 20 | | 0.2461 | 9.14 |
| artemether | 20 | | 0.2358 | 9.12 |
| artemether | 20 | | 0.2413 | 9.10 |
| artemether | 20 | | 0.2477 | 9.08 |
| artemether | 20 | | 0.2436 | 9.10 |
| artemether | 20 | | 0.2973 | 9.21 |
| artemether | 20 | | 0.3057 | 9.23 |
| artemether | 20 | | 0.3443 | 10.62 |
| artemether | 20 | | 0.3189 | 9.33 |
| artemether | 20 | | 0.3069 | 9.64 |
| artemether | | 20 | 0.2965 | 10.37 |
| artemether | 20 | | 0.3490 | 10.07 |
| artemether | 20 | | 0.3517 | 10.03 |
| artemether | 20 | | 0.3514 | 10.04 |
| artemether | 20 | | 0.3541 | 10.10 |
| artemether | 20 | | 0.3547 | 10.11 |

| | | | | |
|---|---|---|---|---|
| artemether | 20 | | 0.3452 | 10.05 |
| artemether | 20 | | 0.3567 | 10.15 |
| artemether | 20 | | 0.3540 | 10.04 |
| artemether | 20 | | 0.3540 | 10.18 |
| artemether | 20 | | 0.3578 | 10.10 |
| artemether | 20 | | 0.3513 | 10.09 |
| artemether | 20 | | 0.3536 | 10.11 |
| artemether | 20 | | 0.3545 | 10.15 |
| artemether | 20 | | 0.3524 | 10.05 |
| artemether | 20 | | 0.3567 | 10.06 |
| artemether | 20 | | 0.3584 | 10.11 |
| artemether | 20 | | 0.3652 | 10.05 |
| artemether | 20 | | 0.3514 | 10.10 |
| artemether | 20 | | 0.3518 | 10.02 |
| artemether | 20 | | 0.3574 | 10.06 |
| artemether | 20 | | 0.3497 | 10.06 |
| artemether | 20 | | 0.3550 | 10.06 |
| artemether | 20 | | 0.3509 | 10.05 |
| artemether | 20 | | 0.3511 | 10.11 |
| artemether | 20 | | 0.3544 | 10.18 |
| artemether | 20 | | 0.3548 | 10.07 |
| artemether | 20 | | 0.3546 | 10.05 |
| artemether | 20 | | 0.3541 | 10.13 |
| artemether | 20 | | 0.3524 | 10.11 |
| artemether | 20 | | 0.3595 | 10.08 |
| artemether | 20 | | 0.3482 | 10.10 |
| artemether | 20 | | 0.3571 | 10.11 |
| artesunate | 50 | | | |
| artemether | 20 | | 0.3544 | 10.06 |
| artemether | 20 | | 0.3518 | 10.08 |
| artemether | 20 | | 0.3514 | 10.05 |
| artemether | 20 | | 0.3472 | 10.07 |
| artemether | | 20 | 0.3559 | 10.16 |
| artemether | 20 | | 0.3570 | 10.10 |
| artemether | 20 | | 0.3548 | 10.06 |
| artemether | 20 | | 0.3511 | 10.03 |
| artemether | 20 | | 0.3515 | 10.04 |
| artemether | 20 | | 0.3557 | 10.18 |
| artemether | 20 | | 0.3532 | 10.13 |
| artemether | 20 | | 0.3524 | 10.16 |
| artemether | 20 | | 0.3514 | 10.04 |
| artemether | 20 | | 0.3497 | 10.10 |
| artemether | 20 | | 0.3577 | 10.15 |
| artemether | 20 | | 0.3400 | 10.11 |
| artemether | 20 | | 0.3547 | 10.11 |
| artemether | 20 | | 0.3552 | 10.08 |
| artemether | 20 | | 0.3450 | 10.06 |
| artemether | 20 | | 0.3393 | 10.03 |
| artemether | 20 | | 0.3524 | 10.10 |
| artemether | 20 | | 0.3529 | 10.10 |

| | | | |
|---|---|---|---|
| artemether | 20 | 0.3525 | 10.06 |
| artemether | 20 | 0.3540 | 10.03 |
| artemether | 20 | 0.3427 | 10.07 |
| artemether | 20 | 0.3555 | 10.12 |
| artemether | 20 | 0.3527 | 10.03 |
| artemether | 20 | 0.3499 | 10.03 |
| artemether | 20 | 0.3570 | 10.04 |
| artemether | 20 | 0.3466 | 10.04 |
| artemether | 20 | 0.3563 | 10.10 |
| artemether | 20 | 0.3530 | 10.08 |
| artemether | 20 | 0.3490 | 10.13 |
| artemether | 20 | 0.3541 | 10.05 |
| artemether | 20 | 0.3528 | 10.03 |
| artemether | 20 | 0.3462 | 10.09 |
| artemether | 20 | 0.3483 | 10.07 |
| artemether | 20 | 0.3524 | 10.10 |
| artemether | 20 | 0.3482 | 10.07 |
| artemether | 20 | 0.3497 | 10.07 |
| artemether | 20 | 0.3509 | 10.01 |
| artemether | 20 | 0.3479 | 10.05 |
| artemether | 20 | 0.3495 | 10.06 |
| artemether | 20 | 0.3554 | 10.02 |
| artemether | 20 | 0.3541 | 10.05 |
| artemether | 20 | 0.3500 | 10.06 |
| artemether | 20 | 0.3554 | 10.12 |
| artemether | 20 | 0.3499 | 10.06 |
| artemether | 20 | 0.3530 | 10.09 |
| artemether | 20 | 0.3498 | 10.04 |
| artemether | 20 | 0.3478 | 10.05 |
| artemether | 20 | 0.3522 | 10.07 |
| artemether | 20 | 0.3522 | 10.05 |
| artemether | 20 | 0.3585 | 10.01 |
| artemether | 20 | 0.3418 | 10.11 |
| artemether | 20 | 0.3539 | 10.04 |
| artemether | 20 | 0.3552 | 10.08 |
| artemether | 20 | 0.3567 | 10.07 |
| artemether | 20 | 0.3517 | 10.07 |
| artemether | 20 | 0.3502 | 10.07 |
| artemether | 20 | 0.3494 | 10.04 |
| artemether | 20 | 0.3515 | 10.02 |
| artemether | 20 | 0.3530 | 10.10 |
| artemether | 20 | 0.3502 | 10.16 |
| artemether | 20 | 0.3577 | 10.07 |
| artemether | 20 | 0.3551 | 10.04 |
| artemether | 20 | 0.3519 | 10.08 |
| artemether | 20 | 0.3457 | 10.08 |
| artemether | 20 | 0.3464 | 10.08 |
| artemether | 20 | 0.3534 | 10.09 |
| artemether | 20 | 0.3539 | 10.09 |
| artemether | 20 | 0.3537 | 10.07 |

| | | | |
|---|---|---|---|
| artemether | 20 | 0.3485 | 10.07 |
| artemether | 20 | 0.3486 | 10.07 |
| artemether | 20 | 0.3501 | 10.07 |
| artemether | 20 | 0.3529 | 10.07 |
| artemether | 20 | 0.3501 | 10.08 |
| artemether | 20 | 0.3461 | 10.09 |
| artemether | 20 | 0.3546 | 10.09 |
| artemether | 20 | 0.3446 | 10.05 |
| artemether | 20 | 0.3406 | 10.12 |
| artemether | 20 | 0.3510 | 10.02 |
| artemether | 20 | 0.3463 | 10.02 |
| artemether | 20 | 0.3547 | 10.06 |
| artemether | 20 | 0.3582 | 10.00 |
| artemether | 20 | 0.3528 | 10.05 |
| artemether | 20 | 0.3488 | 10.09 |
| artemether | 20 | 0.3503 | 10.06 |
| artemether | 20 | 0.3550 | 10.06 |
| artemether | 20 | 0.3536 | 10.05 |
| artemether | 20 | 0.3407 | 10.07 |
| artemether | 20 | 0.2738 | 9.26 |
| artemether | 20 | 0.2668 | 9.23 |
| artemether | 20 | 0.2717 | 9.20 |
| artemether | 20 | 0.2111 | 8.07 |
| artemether | 20 | 0.2038 | 8.08 |
| artemether | 20 | 0.2109 | 8.06 |
| artemether | 20 | 0.2099 | 8.06 |
| artemether | 20 | 0.2652 | 9.13 |
| artemether | 20 | 0.2616 | 9.12 |
| artemether | 20 | 0.2694 | 9.11 |
| artemether | 20 | 0.2546 | 9.10 |
| artemether | 20 | 0.2607 | 9.14 |
| artemether | 20 | 0.2744 | 9.14 |
| artemether | 20 | 0.2716 | 9.13 |
| artemether | 20 | 0.2636 | 9.10 |
| artemether | 20 | 0.2763 | 9.10 |
| artemether | 20 | 0.2581 | 9.08 |
| artemether | 20 | 0.2677 | 9.13 |
| artemether | 20 | 0.2716 | 9.10 |
| artemether | 20 | 0.2790 | 9.13 |
| artemether | 20 | 0.2638 | 9.15 |
| artemether | 20 | 0.2701 | 9.20 |
| artemether | 20 | 0.2664 | 9.13 |
| artemether | 20 | 0.2663 | 9.08 |
| artemether | 20 | 0.2872 | 9.15 |
| artemether | 40 | 0.3686 | 10.23 |
| artemether | 40 | 0.4030 | 10.16 |
| artemether | 40 | 0.3891 | 10.08 |
| artemether | 40 | 0.3785 | 10.12 |
| artemether | 40 | 0.3883 | 10.11 |
| artemether | 40 | 0.3572 | 10.15 |

| | | | | |
|---|---|---|---|---|
| artemether | | 40 | 0.4611 | 11.02 |
| artemether | 40 | | 0.4496 | 11.03 |
| artemether | | 40 | 0.4549 | 11.01 |
| artemether | 40 | | 0.4452 | 11.03 |
| artemether | | 40 | 0.4587 | 10.98 |
| artemether | | 40 | 0.4601 | 11.03 |
| artemether | 40 | | 0.4560 | 11.02 |
| artemether | 40 | | 0.4639 | 11.02 |
| artemether | 40 | | 0.4193 | 11.03 |
| artemether | 40 | | 0.4670 | 11.04 |
| artemether | 40 | | 0.4460 | 11.01 |
| artemether | | 40 | 0.4516 | 11.02 |
| artemether | | 40 | 0.4674 | 11.03 |
| artemether | | 40 | 0.4577 | 11.01 |
| artemether | 40 | | 0.4535 | 11.05 |
| artemether | | 40 | 0.3782 | 11.02 |
| artemether | 40 | | 0.5583 | 12.62 |
| artemether | 40 | | 0.5625 | 12.66 |
| artemether | 40 | | 0.5800 | 13.13 |
| artemether | 40 | | 0.6076 | 13.09 |
| artemether | 40 | | 0.6358 | 13.07 |
| artemether | 40 | | 0.6167 | 13.12 |
| artemether | 40 | | 0.5407 | 13.09 |
| artemether | 40 | | 0.4911 | 13.16 |
| artemether | 40 | | 0.3985 | 11.08 |
| artemether | 40 | | 0.4502 | 10.13 |
| artemether | 40 | | 0.3694 | 10.05 |
| artemether | 80 | | 0.7310 | 12.54 |
| artemether | 80 | | 0.7441 | 12.51 |
| artemether | 80 | | 0.7338 | 12.50 |
| artemether | 80 | | 0.7151 | 12.48 |
| artemether | 80 | | 0.7467 | 12.50 |
| artemether | 80 | | 0.7282 | 12.49 |
| artemether | 80 | | 0.7353 | 12.51 |
| artemether | 80 | | 0.7292 | 12.47 |
| artemether | 80 | | 0.7275 | 12.49 |
| artemether | 80 | | 0.7352 | 12.52 |
| artemether | 80 | | 0.7228 | 12.44 |
| artemether | 80 | | 0.7369 | 12.55 |
| artemether | 80 | | 0.7382 | 12.53 |
| artemether | 80 | | 0.7384 | 12.51 |
| artemether | 80 | | 0.7417 | 12.49 |
| artemether | 80 | | 0.7271 | 12.52 |
| artemether | 80 | | 0.7521 | 12.48 |
| artemether | 80 | | 0.7295 | 12.50 |
| artemether | 80 | | 0.7411 | 12.46 |
| artemether | 80 | | 0.7277 | 12.51 |
| artemether | 80 | | 0.7190 | 12.53 |
| artemether | 80 | | 0.7311 | 12.51 |
| artemether | 80 | | 0.7369 | 12.50 |

| | | | | |
|---|---|---|---|---|
| artemether | 80 | | 0.7643 | 12.97 |
| artemether | 80 | | 0.7632 | 12.80 |
| artemether | 80 | | 0.7869 | 12.89 |
| artemether | 80 | | 0.7754 | 12.90 |
| artemether | 80 | | 0.7356 | 12.87 |
| artemether | 80 | | 0.7718 | 12.89 |
| artemether | 80 | | 0.7764 | 12.92 |
| artemether | 80 | | 0.7493 | 12.86 |
| artemether | 80 | | 0.7989 | 12.97 |
| artemether | 80 | | 0.7633 | 12.89 |
| artemether | 80 | | 0.7774 | 12.91 |
| artemether | 80 | | 0.7737 | 12.89 |
| artemether | 80 | | 0.7694 | 12.92 |
| artemether | 80 | | 0.7633 | 12.95 |
| artemether | 80 | | 0.7764 | 12.90 |
| artemether | 80 | | 0.7582 | 12.82 |
| artemether | 80 | | 0.7724 | 12.89 |
| artemether | 80 | | 0.7638 | 12.90 |
| artemether | 80 | | 0.7671 | 12.89 |
| artemether | 80 | | 0.6694 | 12.03 |
| artemether | 80 | | 0.6966 | 12.07 |
| artemether | 80 | | 0.6951 | 12.10 |
| artemether | 80 | | 0.6887 | 12.02 |
| artemether | 80 | | 0.6740 | 12.13 |
| artemether | 80 | | 0.7036 | 12.01 |
| artemether | 80 | | 0.7028 | 12.09 |
| artemether | 80 | | 0.6824 | 13.03 |
| artemether | 80 | | 0.7224 | 13.06 |
| artemether | 80 | | 0.6723 | 13.03 |
| artemether | | 80 | 0.6866 | 7.75 |
| artemether | 80 | | 0.6604 | 7.70 |
| artemether | 80 | | 0.6778 | 7.73 |
| artemether | 80 | | 0.8063 | 13.19 |
| artemether | 80 | | 0.8223 | 13.22 |
| artemether | 80 | | 0.6371 | 12.29 |
| artemether | | 40 | | |
| artemether | 80 | | 0.6181 | 12.64 |
| artemether | 80 | | 0.5936 | 12.61 |
| artemether | 80 | | | |
| artemether | 80 | | | |
| artemether | 80 | | 0.7274 | 12.58 |
| artemether | 80 | | 0.7625 | 12.66 |
| artemether | 80 | | | |
| artemether | 80 | | | |
| artemether | 80 | | | |
| artemether | 80 | | | |
| artemether | 80 | | | |
| artemether | 80 | | 0.6636 | 12.24 |
| artemether | 80 | | 0.6318 | 12.68 |
| artemether | 80 | | | |

| | | | |
|---|---|---|---|
| artemether | 80 | 0.6120 | 12.70 |
| artemether | 80 | | |
| artemether | 80 | | |
| artemether | 80 | | |
| artemether | 80 | | |
| artemether | 80 | | |
| artemether | 80 | | |
| artemether | 80 | | |
| artemether | 80 | | |
| artemether | 80 | | |
| artemether | 80 | 0.8441 | 14.23 |
| artemether | 80 | 0.8415 | 14.33 |
| artemether | 80 | | |
| artemether | 80 | | |
| artemether | 80 | | |
| artemether | 80 | | |
| artemether | 80 | | |
| artemether | 80 | | |
| artemether | 80 | | |
| artemether | 80 | | |
| artemether | 80 | | |
| artemether | 80 | | |
| artemether | 80 | | |
| artemether | 80 | | |
| artemether | 80 | | |
| artemether | 80 | | |
| artemether | 80 | | |
| artemether | 80 | 0.7708 | 12.20 |
| artemether | 80 | 0.7816 | 12.23 |
| artemether | 80 | | |
| artemether | 80 | | |
| artemether | 80 | | |
| artemether | 80 | | |
| artemether | 80 | | |
| artemether | 80 | | |
| artemether | 80 | | |
| artemether | 80 | | |
| artemether | 80 | 0.9760 | 9.20 |
| artemether | 80 | 0.6582 | 12.80 |
| artemether | 80 | 0.6609 | 12.79 |
| artemether | 80 | 0.7022 | 12.03 |
| artemether | 80 | 0.7133 | 12.04 |
| artemether | 80 | | |
| artemether | 80 | | |
| artemether | 80 | 0.7663 | 13.00 |
| artemether | 80 | 0.7571 | 12.95 |
| artemether | 80 | | |
| artemether | 80 | | |
| artemether | 80 | | |
| artemether | 80 | | |

| | | | |
|---|---|---|---|
| artemether | 80 | | |
| artemether | 80 | | |
| artemether | 80 | | |
| artemether | 80 | | |
| artemether | 80 | | |
| artemether | 80 | 0.6416 | 12.07 |
| artemether | 80 | 0.6525 | 12.06 |
| artemether | 80 | 0.6816 | 12.13 |
| artemether | 80 | 0.6785 | 12.24 |
| artemether | 80 | | |
| artemether | 80 | | |
| artemether | 80 | | |
| artemether | 80 | | |
| artemether | 80 | | |
| artemether | 80 | | |
| artemether | 80 | | |
| artemether | 80 | | |
| artemether | 80 | | |
| artemether | 80 | | |
| artemether | 80 | | |
| artemether | 80 | | |
| artemether | 80 | | |
| artemether | 80 | | |
| artemether | 80 | | |
| artemether | 80 | | |
| artemether | 80 | | |
| artemether | 80 | | |
| artemether | 80 | | |
| artemether | 80 | 0.6698 | 9.15 |
| artemether | 80 | 0.6960 | 12.07 |
| artemether | 80 | 0.6861 | 11.96 |
| artemether | 80 | | |
| artemether | 80 | | |
| artemether | 80 | | |
| artemether | 80 | | |
| artemether | 80 | 0.7420 | 6.04 |
| artemether | 80 | | |
| artemether | 80 | 0.7634 | 6.25 |
| artemether | 80 | | |
| artemether | 80 | | |
| artemether | 80 | | |
| artemether | 80 | | |
| artemether | 80 | | |
| artemether | 80 | | |
| artemether | 80 | | |
| artemether | 80 | | |
| artemether | 80 | | |
| artemether | 80 | | |
| artemether | 80 | | |
| artemether | 80 | | |

| | | | |
|---|---|---|---|
| artemether | 80 | | |
| artemether | 80 | | |
| artemether | 80 | | |
| artemether | 80 | | |
| artemether | 80 | | |
| artemether | 80 | | |
| artemether | 80 | | |
| artemether | 80 | | |
| artemether | 80 | | |
| artemether | 80 | | |
| artemether | 80 | | |
| artemether | 80 | | |
| artemether | 80 | | |
| artemether | 80 | | |
| artemether | 80 | | |
| artemether | 80 | | |
| artemether | 80 | | |
| artemether | 80 | | |
| artemether | 80 | | |
| artemether | 80 | | |
| artemether | 80 | | |
| artemether | 80 | | |
| artemether | 80 | | |
| artemether | 80 | | |
| artemether | 80 | | |
| artemether | 80 | | |
| artemether | 80 | | |
| artemether | 80 | | |
| artemether | 80 | | |
| artemether | 80 | | |
| artemether | 80 | | |
| artemether | 80 | | |
| artemether | 80 | | |
| artemether | 80 | | |
| artemether | 80 | | |
| artemether | 80 | | |
| artemether | 80 | | |
| artemether | 80 | | |
| artemether | 80 | | |
| artemether | 80 | | |
| artemether | 80 | | |
| artemether | 80 | | |
| artemether | 80 | | |
| artemether | 80 | | |
| artemether | 80 | | |
| artemether | 80 | | |
| artemether | 80 | | |
| artemether | 80 | | |
| artemether | 80 | 0.6828 | 12.21 |
| artemether | 80 | 0.6816 | 12.17 |

| | |
|---|---|
| artemether | 80 |
| artemether | 80 |
| artemether | 80 |
| artemether | 80 |
| artemether | 80 |
| artemether | 80 |
| artemether | 80 |
| artemether | 80 |
| artemether | 80 |
| artemether | 80 |
| artemether | 80 |
| artemether | 80 |
| artemether | 80 |
| artemether | 80 |
| artemether | 80 |
| artemether | 80 |
| artemether | 80 |
| artemether | 80 |
| artemether | 80 |
| artemether | 80 |
| artemether | 80 |
| artemether | 80 |
| artemether | 80 |
| artemether | 80 |
| artemether | 80 |
| artemether | 80 |
| artemether | 80 |
| artemether | 80 |
| artemether | 80 |
| artemether | 80 |
| artemether | 80 |
| artemether | 80 |
| artemether | 80 |
| artemether | 80 |
| artemether | 80 |
| artemether | 80 |
| artemether | 80 |
| artemether | 80 |
| artemether | 80 |
| artemether | 80 |
| artemether | 80 |
| artemether | 80 |
| artemether | 80 |
| artemether | 80 |
| artemether | 80 |
| artemether | 80 |
| artemether | 80 |
| artemether | 80 |
| artemether | 80 |
| artemether | 80 |

| | | | | |
|---|---|---|---|---|
| artemether | 80 | | | |
| artemether | 80 | | | |
| artemether | 80 | | | |
| artemether | 80 | | | |
| artemether | 80 | | | |
| artemether | 80 | | | |
| artemether | 80 | | | |
| artemether | 80 | | | |
| artemether | 80 | | | |
| artemether | 80 | | | |
| artemether | 80 | | | |
| artemether | 80 | | | |
| artemether | 80 | | | |
| artemether | 80 | | | |
| artemether | 80 | | | |
| artemether | 80 | | | |
| artemether | 80 | | | |
| artemether | | 20 | | |
| artemether | 80 | | 0.7278 | 12.16 |
| artemether | 80 | | 0.6834 | 12.23 |
| artemether | 80 | | | |
| artemether | 80 | | | |
| artemether | 80 | | | |
| artemether | 80 | | | |
| artemether | 80 | | 0.7141 | 7.60 |
| artemether | 80 | | 0.7800 | 7.59 |
| artemether | 80 | | | |
| artemether | 80 | | | |
| artemether | 80 | | | |
| artemether | 80 | | | |
| artemether | 80 | | | |
| artemether | 80 | | | |
| artemether | 80 | | | |
| artemether | 80 | | | |
| artemether | 80 | | | |
| artemether | 80 | | | |
| artemether | 80 | | | |
| artemether | 80 | | | |
| artemether | 80 | | | |
| artemether | 80 | | | |
| artemether | 80 | | | |
| artemether | 80 | | | |
| artemether | 80 | | | |
| artemether | 80 | | 0.6525 | 9.51 |
| artemether | 180 | | 0.7232 | 13.62 |
| artemether | 80 | | 0.7438 | 13.64 |
| artemether | 180 | | | |
| artemether | 80 | | | |
| artemether | 80 | | 0.7890 | 12.89 |
| artemether | 80 | | 0.7823 | 12.84 |

| | | | |
|---|---|---|---|
| artemether | 80 | | |
| artemether | 80 | | |
| artemether | 80 | 0.6666 | 12.01 |
| artemether | 80 | 0.6678 | 12.04 |
| artemether | 80 | | |
| artemether | 80 | | |
| artemether | 80 | | |
| artemether | 80 | | |
| artemether | 80 | | |
| artemether | 80 | | |
| artemether | 80 | | |
| artemether | 80 | | |
| artemether | 80 | | |
| artemether | 80 | | |
| artemether | 80 | | |
| artemether | 80 | | |
| artemether | 80 | | |
| artemether | 80 | | |
| artemether | 80 | | |
| artemether | 80 | | |
| artemether | 80 | | |
| artemether | 80 | | |
| artemether | 80 | | |
| artemether | 80 | 0.8263 | 14.27 |
| artemether | 80 | 0.8396 | 14.17 |
| artemether | 80 | | |
| artemether | 80 | | |
| artemether | 80 | | |
| artemether | 80 | | |
| artemether | 80 | | |
| artemether | 80 | | |
| artemether | 80 | | |
| artemether | 80 | | |
| artemether | 80 | | |
| artemether | 80 | | |
| artemether | 80 | 0.6725 | 12.03 |
| artemether | 80 | 0.6583 | 12.01 |
| artemether | 80 | | |
| artemether | 80 | | |
| artemether | 80 | | |
| artemether | 80 | | |
| artemether | 80 | | |
| artemether | 80 | | |
| artemether | 80 | | |
| artemether | 80 | | |
| artemether | 80 | | |
| artemether | 80 | | |
| artemether | 80 | | |
| artemether | 80 | | |
| artemether | 80 | 0.8235 | 8.10 |

| | | | |
|---|---|---|---|
| artemether | 80 | 0.8082 | 8.12 |
| artemether | 80 | | |
| artemether | 80 | | |
| artemether | 80 | | |
| artemether | 80 | | |
| artemether | 80 | | |
| artemether | 80 | | |
| artemether | 80 | | |
| artemether | 80 | | |
| artemether | 80 | | |
| artemether | 80 | | |
| artemether | 80 | | |
| artemether | 80 | | |
| artesunate | 50 | 0.2830 | 9.54 |
| artesunate | 50 | 0.2623 | 9.72 |
| artesunate | 50 | 0.2950 | 10.06 |
| artesunate | 50 | 0.2993 | 10.09 |
| artesunate | 50 | 0.2975 | 10.11 |
| artesunate | 50 | 0.2921 | 10.07 |
| artesunate | 50 | 0.3111 | 10.04 |
| artesunate | 50 | | |
| artesunate | 50 | | |
| artesunate | 50 | | |
| artesunate | 50 | | |
| artesunate | 50 | | |
| artesunate | 50 | | |
| artesunate | 50 | | |
| artesunate | 50 | | |
| artesunate | 50 | | |
| artesunate | 50 | | |
| artesunate | 50 | | |
| artesunate | 50 | | |
| artesunate | 50 | | |
| artesunate | 50 | | |
| artesunate | 50 | | |
| artesunate | 50 | | |
| artesunate | 50 | | |
| artesunate | 50 | | |
| artesunate | 50 | | |
| artesunate | 50 | | |
| artesunate | 50 | | |
| artesunate | 50 | | |
| artesunate | 50 | | |
| artesunate | 50 | | |
| artesunate | 50 | | |
| artesunate | 50 | | |
| artesunate | 50 | | |
| artesunate | 50 | | |
| artesunate | 50 | | |
| artesunate | 50 | | |

| | | | |
|---|---|---|---|
| artesunate | 50 | | |
| artesunate | 50 | | |
| artesunate | 50 | | |
| artesunate | 50 | | |
| artesunate | 50 | | |
| artesunate | 50 | | |
| artesunate | 50 | | |
| artesunate | 50 | | |
| artesunate | 50 | | |
| artesunate | 50 | | |
| artesunate | 50 | | |
| artesunate | 50 | | |
| artesunate | 50 | | |
| artesunate | 50 | | |
| artesunate | 50 | | |
| artesunate | 50 | | |
| artesunate | 50 | | |
| artesunate | 50 | | |
| artesunate | 50 | | |
| artesunate | 50 | | |
| artesunate | 50 | | |
| artesunate | 50 | | |
| artesunate | 50 | | |
| artesunate | 50 | | |
| artesunate | 50 | | |
| artesunate | 50 | | |
| artesunate | 50 | | |
| artesunate | 50 | | |
| artesunate | 50 | | |
| artesunate | 50 | | |
| artesunate | 50 | | |
| artesunate | 50 | | |
| artesunate | 50 | | |
| artesunate | 50 | | |
| artesunate | 50 | | |
| artesunate | 50 | | |
| artesunate | 50 | | |
| artesunate | 50 | | |
| artesunate | 50 | | |
| artesunate | 50 | | |
| artesunate | 50 | | |
| artesunate | 50 | | |
| artesunate | 50 | 0.2970 | 9.53 |
| artesunate | 50 | 0.2635 | 9.29 |
| artesunate | 50 | 0.2924 | 9.39 |
| artesunate | 50 | 0.2748 | 9.30 |
| artesunate | 50 | 0.2876 | 9.28 |
| artesunate | 50 | 0.2795 | 9.28 |
| artesunate | 50 | 0.2775 | 9.28 |
| artesunate | 50 | 0.2949 | 9.08 |

| | | | |
|---|---|---|---|
| artesunate | 50 | 0.2964 | 9.09 |
| artesunate | 50 | 0.2998 | 9.13 |
| artesunate | 100 | 0.2045 | 8.24 |
| artesunate | 100 | 0.2062 | 8.31 |
| artesunate | 100 | 0.2560 | 9.12 |
| artesunate | 25 | 0.1744 | 8.08 |
| artesunate | 25 | 0.1778 | 8.06 |
| artesunate | 50 | 0.3450 | 10.08 |
| artesunate | 50 | 0.3572 | 10.09 |
| artesunate | 50 | 0.3550 | 10.08 |
| artesunate | 50 | 0.3533 | 10.09 |
| artesunate | 50 | 0.3574 | 10.09 |
| artesunate | 50 | | |
| artesunate | 50 | | |
| artesunate | 50 | | |
| artesunate | 50 | | |
| artesunate | 50 | | |
| artesunate | 50 | | |
| artesunate | 50 | | |
| artesunate | 50 | | |
| artesunate | 50 | | |
| artesunate | 50 | | |
| artesunate | 50 | | |
| artesunate | 50 | | |
| artesunate | 50 | | |
| artesunate | 50 | | |
| artesunate | 50 | | |
| artesunate | 50 | | |
| artesunate | 50 | | |
| artesunate | 50 | | |
| artesunate | 50 | | |
| artesunate | 50 | 0.2956 | 9.33 |
| artesunate | 50 | 0.3115 | 9.19 |
| artesunate | 50 | 0.2670 | 9.26 |
| artesunate | 50 | 0.2646 | 9.21 |
| artesunate | 50 | 0.2728 | 9.22 |
| artesunate | 50 | 0.2692 | 9.18 |
| artesunate | 50 | 0.2728 | 9.22 |
| artesunate | 50 | | |
| artesunate | 50 | | |
| artesunate | 50 | | |
| artesunate | 50 | | |
| artesunate | 50 | | |
| artesunate | 50 | | |
| artesunate | 50 | | |
| artesunate | 50 | | |
| artesunate | 50 | | |
| artesunate | 50 | 0.2747 | 9.23 |
| artesunate | 50 | 0.2699 | 9.26 |
| artesunate | 50 | 0.2567 | 9.19 |

| | | | |
|---|---|---|---|
| artesunate | 50 | 0.2681 | 9.31 |
| artesunate | 50 | 0.2680 | 9.20 |
| artesunate | 50 | 0.2713 | 9.21 |
| artesunate | 50 | 0.2676 | 9.28 |
| artesunate | 50 | | |
| artesunate | 50 | | |
| artesunate | 50 | | |
| artesunate | 50 | | |
| artesunate | 50 | | |
| artesunate | 50 | | |
| artesunate | 50 | | |
| artesunate | 50 | 0.3888 | 11.27 |
| artesunate | 50 | 0.3268 | 9.63 |
| artesunate | 50 | 0.3310 | 9.71 |
| artesunate | 50 | 0.2711 | 9.14 |
| artesunate | 50 | 0.1815 | 8.15 |
| artesunate | 50 | 0.1900 | 8.10 |
| artesunate | 50 | 0.1909 | 8.10 |
| artesunate | 50 | 0.2860 | 9.51 |
| artesunate | 50 | 0.2691 | 9.48 |
| artesunate | 50 | 0.2811 | 9.44 |
| artesunate | 50 | 0.2946 | 9.54 |
| artesunate | 50 | 0.2829 | 9.47 |
| artesunate | 100 | 0.7002 | 13.11 |
| artesunate | 100 | 0.7061 | 13.13 |
| artesunate | 100 | 0.7148 | 13.11 |
| artesunate | 100 | 0.7076 | 13.11 |
| artesunate | 100 | 0.7074 | 13.15 |
| artesunate | 100 | | |
| artesunate | 100 | | |
| artesunate | 100 | | |
| artesunate | 100 | | |
| artesunate | 100 | | |
| artesunate | 100 | | |
| artesunate | 100 | | |
| artesunate | 100 | | |
| artesunate | 100 | | |
| artesunate | 100 | | |
| artesunate | 100 | | |
| artesunate | 100 | | |
| artesunate | 100 | | |
| artesunate | 100 | 0.7085 | 13.13 |
| artesunate | 100 | 0.7094 | 13.14 |
| artesunate | 100 | 0.7000 | 13.10 |
| artesunate | 100 | 0.7041 | 13.11 |
| artesunate | 100 | 0.6968 | 13.12 |
| artesunate | 100 | | |
| artesunate | 100 | | |
| artesunate | 100 | | |
| artesunate | 100 | | |

| | | | | |
|---|---|---|---|---|
| artesunate | 100 | | | |
| artesunate | 100 | | | |
| artesunate | 100 | | | |
| artesunate | 100 | | | |
| artesunate | 100 | | | |
| artesunate | 100 | | | |
| artesunate | 100 | | | |
| artesunate | 100 | | | |
| artesunate | 100 | | | |
| artesunate | 100 | | | |
| artesunate | 100 | | | |
| artesunate | 100 | | | |
| artesunate | 100 | | | |
| artesunate | 100 | | | |
| artesunate | 100 | | | |
| artesunate | 100 | | | |
| artesunate | 100 | | | |
| artesunate | 100 | | | |
| artesunate | 100 | | | |
| artesunate | 100 | | | |
| artesunate | 100 | | | |
| artesunate | 100 | | | |
| artesunate | 100 | | | |
| artesunate | 100 | | | |
| artesunate | 100 | | | |
| artesunate | 100 | | | |
| artesunate | 100 | | | |
| artesunate | 100 | | | |
| artesunate | 100 | | | |
| artesunate | 100 | | | |
| artesunate | 100 | | | |
| artesunate | 100 | | | |
| artesunate | 100 | | | |
| artesunate | 100 | | | |
| artesunate | 100 | | | |
| artesunate | 100 | | | |
| artesunate | 100 | | | |
| artesunate | 100 | | | |
| artesunate | 100 | | | |
| artesunate | | 100 | 0.2766 | 9.15 |
| artesunate | 100 | | 0.2757 | 9.14 |
| artesunate | 100 | | 0.2884 | 9.14 |
| artesunate | 100 | | 0.2824 | 9.13 |
| artesunate | 100 | | 0.2929 | 9.18 |
| artesunate | | 100 | 0.2795 | 9.26 |
| artesunate | 100 | | 0.2774 | 9.12 |
| artesunate | 100 | | 0.3026 | 9.11 |
| artesunate | | 100 | 0.3040 | 9.15 |
| artesunate | 100 | | 0.2849 | 9.20 |
| artesunate | 100 | | 0.3079 | 9.13 |

| | | | | |
|---|---|---|---|---|
| artesunate | | 100 | 0.2988 | 9.18 |
| artesunate | | 100 | | |
| artesunate | | 100 | | |
| artesunate | | 100 | | |
| artesunate | | 100 | | |
| artesunate | | 100 | | |
| artesunate | | 100 | | |
| artesunate | 100 | | | |
| artesunate | | 100 | | |
| artesunate | | 100 | | |
| artesunate | 100 | | | |
| artesunate | | 100 | | |
| artesunate | 100 | | | |
| artesunate | | 100 | | |
| artesunate | | 100 | | |
| artesunate | | 100 | | |
| artesunate | | 100 | | |
| artesunate | | 100 | | |
| artesunate | 100 | | | |
| artesunate | | 100 | | |
| artesunate | | 100 | | |
| artesunate | | 100 | 0.3003 | 9.19 |
| artesunate | 100 | | 0.2935 | 9.18 |
| artesunate | 100 | | 0.3014 | 9.16 |
| artesunate | | 100 | 0.3006 | 9.16 |
| artesunate | 100 | | 0.3041 | 9.18 |
| artesunate | | 100 | | |
| artesunate | | 100 | | |
| artesunate | | 100 | | |
| artesunate | | 100 | | |
| artesunate | 100 | | | |
| artesunate | | 100 | | |
| artesunate | | 100 | | |
| artesunate | 100 | | | |
| artesunate | | 100 | | |
| artesunate | | 100 | | |
| artesunate | 100 | | | |
| artesunate | | 100 | | |
| artesunate | | 100 | | |
| artesunate | | 100 | | |
| artesunate | | 100 | | |
| artesunate | 100 | | | |
| artesunate | | 100 | | |
| artesunate | 100 | | 0.2390 | 9.30 |
| artesunate | 100 | | 0.2728 | 9.19 |
| artesunate | 100 | | 0.2434 | 9.30 |
| artesunate | 100 | | 0.2606 | 9.27 |
| artesunate | 100 | | | |
| artesunate | 200 | | 0.4983 | 16.24 |
| artesunate | 200 | | 0.4830 | 18.67 |

| | | | | |
|---|---|---|---|---|
| artesunate | | 200 | 0.5094 | 16.29 |
| artesunate | | 25 | | |
| artesunate | | 25 | 0.5073 | |
| artesunate | | 25 | 0.4779 | |
| artesunate | | 25 | 0.4885 | |
| artesunate | | 25 | 0.4849 | |
| artesunate | | 25 | 0.5118 | |
| artesunate | | 25 | 0.4873 | |
| artesunate | | 25 | | |
| artesunate | | 25 | | |
| artesunate | | 25 | | |
| artesunate | 50 | | 1.0272 | |
| artesunate | 50 | | 1.0439 | |
| artesunate | | 50 | 1.0049 | |
| artesunate | 50 | | 1.0612 | |
| artesunate | | 50 | 0.9848 | |
| artesunate | 50 | | | |
| artesunate | 50 | | | |
| artesunate | 50 | | | |
| artesunate | 50 | | | |
| artesunate | | 50 | | |
| artesunate | | 50 | | |
| artesunate | | 50 | | |
| artesunate | 50 | | | |
| artesunate | | 50 | | |
| artesunate | | 50 | | |
| artesunate | 50 | | | |
| artesunate | 50 | | | |
| artesunate | | 50 | | |
| artesunate | 50 | | | |
| artesunate | 50 | | | |
| artesunate | 50 | | | |
| artesunate | | 50 | | |
| artesunate | 50 | | | |
| artesunate | | 50 | | |
| artesunate | | 50 | | |
| artesunate | 100 | | 0.2824 | 13.10 |
| artesunate | 100 | | 0.2815 | 13.09 |
| artesunate | 100 | | 0.2839 | 13.08 |
| artesunate | 100 | | 0.2804 | 13.11 |
| artesunate | 100 | | 0.2800 | 13.09 |
| artesunate | 100 | | | |
| artesunate | 100 | | | |
| artesunate | 100 | | | |
| artesunate | 100 | | | |
| artesunate | 200 | | 0.5720 | 18.37 |
| artesunate | 200 | | 0.5597 | 18.40 |
| artesunate | 200 | | 0.5464 | 18.48 |
| artesunate | 200 | | 0.5545 | 19.16 |
| artesunate | 200 | | 0.5629 | 19.13 |

| | | | | |
|---|---|---|---|---|
| artesunate | 200 | | 0.5586 | 19.17 |
| artesunate | 200 | | 0.5536 | 19.15 |
| artesunate | 200 | | 0.5642 | 19.16 |
| artesunate | 200 | | | |
| artesunate | 200 | | | |
| artesunate | 200 | | | |
| artesunate | 200 | | | |
| artesunate | 200 | | | |
| artesunate | 200 | | | |
| artesunate | 200 | | | |
| artesunate | 200 | | | |
| artesunate | 200 | | | |
| artesunate | 200 | | | |
| artesunate | 200 | | | |
| artesunate | 200 | | | |
| artesunate | 200 | | | |
| artesunate | 200 | | | |
| artesunate | 200 | | | |
| artesunate | 200 | | | |
| artesunate | 200 | | | |
| artesunate | 200 | | | |
| artesunate | 200 | | | |
| artesunate | 200 | | | |
| artesunate | 200 | | | |
| artesunate | 200 | | | |
| artesunate | 200 | | | |
| artesunate | 200 | | | |
| artesunate | 200 | | | |
| artesunate | 200 | | | |
| artesunate | 200 | | | |
| artesunate | 200 | | | |
| artesunate | 200 | | | |
| artesunate | 200 | | 0.5428 | 12.93 |
| artesunate | 50 | | 0.1922 | 8.31 |
| artesunate | 100 | | 0.3894 | 11.43 |
| artesunate | 100 | | 0.2831 | 10.22 |
| artesunate | 100 | | 0.2810 | 10.18 |
| artesunate | 100 | | 0.2879 | 10.26 |
| artesunate | 100 | | 0.2797 | 10.27 |
| artesunate | 100 | | 0.2863 | 10.22 |
| artesunate | 200 | | 0.4881 | 16.17 |
| artesunate | 200 | | 0.5188 | 16.25 |
| artesunate | 200 | | 0.5300 | 12.22 |
| artesunate | 200 | | 0.4716 | |
| artesunate | 100 | | 0.6055 | 12.62 |
| artesunate | | 100 | 0.6062 | 12.58 |
| artesunate | 100 | | 0.6006 | 12.60 |
| artesunate | 100 | | 0.6004 | 12.60 |
| artesunate | 100 | | 0.5901 | 12.57 |
| artesunate | | 100 | | |

| | | | | |
|---|---|---|---|---|
| artesunate | 100 | | | |
| artesunate | 100 | | | |
| artesunate | 100 | | | |
| dihydroartemisinin | 30 | | | |
| artesunate | 200 | | 1.2034 | 21.86 |
| artesunate | 200 | | 1.1977 | 21.82 |
| artesunate | 200 | | 1.2224 | 21.81 |
| artesunate | 200 | | 1.2178 | 21.79 |
| artesunate | 200 | | 1.2069 | 21.79 |
| artesunate | 200 | | | |
| artesunate | | 200 | | |
| artesunate | 200 | | | |
| artesunate | 200 | | | |
| artesunate | 200 | | | |
| artesunate | 200 | | | |
| artesunate | 200 | | | |
| artesunate | 200 | | | |
| artesunate | 200 | | | |
| artesunate | 200 | | | |
| artesunate | 200 | | | |
| artesunate | 200 | | | |
| artesunate | 100 | | | |
| artesunate | 200 | | | |
| dihydroartemisinin | 60 | | 0.1969 | 8.21 |
| dihydroartemisinin | 60 | | 0.2022 | 8.25 |
| dihydroartemisinin | 60 | | 0.2032 | 8.17 |
| dihydroartemisinin | 60 | | | |
| dihydroartemisinin | 60 | | | |
| dihydroartemisinin | 60 | | | |
| dihydroartemisinin | 60 | | | |
| dihydroartemisinin | 60 | | | |
| dihydroartemisinin | 60 | | | |
| dihydroartemisinin | 60 | | | |
| dihydroartemisinin | 60 | | 0.2374 | 9.03 |
| dihydroartemisinin | 60 | | 0.2376 | 9.07 |
| dihydroartemisinin | 60 | | 0.2486 | 9.08 |
| dihydroartemisinin | 60 | | 0.2498 | 9.28 |
| dihydroartemisinin | 60 | | 0.2551 | 9.26 |
| dihydroartemisinin | 60 | | 0.2514 | 9.28 |
| dihydroartemisinin | 30 | | 0.5437 | 12.65 |
| dihydroartemisinin | 30 | | 0.5283 | 12.69 |
| dihydroartemisinin | 30 | | 0.5333 | 12.63 |
| dihydroartemisinin | 30 | | 0.5476 | 12.68 |
| dihydroartemisinin | 30 | | 0.5250 | 12.65 |
| dihydroartemisinin | 30 | | 0.5271 | 12.68 |
| dihydroartemisinin | 30 | | 0.6118 | 12.22 |
| dihydroartemisinin | 30 | | 0.5730 | 12.65 |
| dihydroartemisinin | 30 | | 0.5466 | 12.70 |
| dihydroartemisinin | 30 | | 0.5334 | 12.68 |
| dihydroartemisinin | 30 | | | |

| | |
|---|---|
| dihydroartemisinin | 30 |
| dihydroartemisinin | 30 |
| dihydroartemisinin | 30 |
| dihydroartemisinin | 30 |
| dihydroartemisinin | 30 |
| dihydroartemisinin | 30 |
| dihydroartemisinin | 30 |
| dihydroartemisinin | 30 |
| dihydroartemisinin | 30 |
| dihydroartemisinin | 30 |
| dihydroartemisinin | 30 |
| dihydroartemisinin | 30 |
| dihydroartemisinin | 30 |
| dihydroartemisinin | 30 |
| dihydroartemisinin | 30 |
| dihydroartemisinin | 30 |
| dihydroartemisinin | 30 |
| dihydroartemisinin | 30 |
| dihydroartemisinin | 30 |
| dihydroartemisinin | 30 |
| dihydroartemisinin | 30 |
| dihydroartemisinin | 30 |
| dihydroartemisinin | 30 |
| dihydroartemisinin | 30 |
| dihydroartemisinin | 30 |
| dihydroartemisinin | 30 |
| dihydroartemisinin | 30 |
| dihydroartemisinin | 30 |
| dihydroartemisinin | 30 |
| dihydroartemisinin | 30 |
| dihydroartemisinin | 30 |
| dihydroartemisinin | 30 |
| dihydroartemisinin | 30 |
| dihydroartemisinin | 30 |
| dihydroartemisinin | 30 |
| dihydroartemisinin | 30 |
| dihydroartemisinin | 30 |
| dihydroartemisinin | 30 |
| dihydroartemisinin | 30 |
| dihydroartemisinin | 30 |
| dihydroartemisinin | 30 |
| dihydroartemisinin | 30 |
| dihydroartemisinin | 30 |
| dihydroartemisinin | 30 |
| dihydroartemisinin | 30 |
| dihydroartemisinin | 30 |
| dihydroartemisinin | 30 |
| dihydroartemisinin | 30 |
| dihydroartemisinin | 30 |
| dihydroartemisinin | 30 |

| | |
|---|---|
| dihydroartemisinin | 30 |
| dihydroartemisinin | 30 |
| dihydroartemisinin | 30 |
| dihydroartemisinin | 30 |
| dihydroartemisinin | 30 |
| dihydroartemisinin | 30 |
| dihydroartemisinin | 30 |
| dihydroartemisinin | 30 |
| dihydroartemisinin | 30 |
| dihydroartemisinin | 30 |
| dihydroartemisinin | 30 |
| dihydroartemisinin | 30 |
| dihydroartemisinin | 30 |
| dihydroartemisinin | 30 |
| dihydroartemisinin | 30 |
| dihydroartemisinin | 30 |
| dihydroartemisinin | 30 |
| dihydroartemisinin | 30 |
| dihydroartemisinin | 30 |
| dihydroartemisinin | 30 |
| dihydroartemisinin | 30 |
| dihydroartemisinin | 30 |
| dihydroartemisinin | 30 |
| dihydroartemisinin | 30 |
| dihydroartemisinin | 30 |
| dihydroartemisinin | 30 |
| dihydroartemisinin | 30 |
| dihydroartemisinin | 30 |
| dihydroartemisinin | 30 |
| dihydroartemisinin | 30 |
| dihydroartemisinin | 30 |
| dihydroartemisinin | 30 |
| dihydroartemisinin | 30 |
| dihydroartemisinin | 30 |
| dihydroartemisinin | 30 |
| dihydroartemisinin | 30 |
| dihydroartemisinin | 30 |
| dihydroartemisinin | 30 |
| dihydroartemisinin | 30 |
| dihydroartemisinin | 30 |
| dihydroartemisinin | 30 |
| dihydroartemisinin | 30 |
| dihydroartemisinin | 30 |
| dihydroartemisinin | 30 |
| dihydroartemisinin | 30 |
| dihydroartemisinin | 30 |
| dihydroartemisinin | 30 |
| dihydroartemisinin | 30 |
| dihydroartemisinin | 30 |
| dihydroartemisinin | 30 |

| | | | |
|---|---|---|---|
| dihydroartemisinin | 30 | | |
| dihydroartemisinin | 30 | | |
| dihydroartemisinin | 30 | | |
| dihydroartemisinin | 30 | | |
| dihydroartemisinin | 30 | | |
| dihydroartemisinin | 30 | | |
| dihydroartemisinin | 30 | | |
| dihydroartemisinin | 30 | | |
| dihydroartemisinin | 30 | | |
| dihydroartemisinin | 30 | | |
| dihydroartemisinin | 30 | | |
| dihydroartemisinin | 30 | | |
| dihydroartemisinin | 30 | | |
| dihydroartemisinin | 30 | | |
| dihydroartemisinin | 30 | | |
| dihydroartemisinin | 30 | | |
| dihydroartemisinin | 30 | | |
| dihydroartemisinin | 30 | | |
| dihydroartemisinin | 30 | | |
| dihydroartemisinin | 30 | | |
| dihydroartemisinin | 30 | | |
| dihydroartemisinin | 30 | | |
| dihydroartemisinin | 30 | | |
| dihydroartemisinin | 30 | | |
| dihydroartemisinin | 30 | | |
| dihydroartemisinin | 30 | | |
| dihydroartemisinin | 30 | | |
| dihydroartemisinin | 30 | | |
| dihydroartemisinin | 30 | | |
| dihydroartemisinin | 30 | | |
| dihydroartemisinin | 30 | | |
| dihydroartemisinin | 30 | | |
| dihydroartemisinin | 30 | | |
| dihydroartemisinin | 30 | | |
| dihydroartemisinin | 30 | | |
| dihydroartemisinin | 30 | | |
| dihydroartemisinin | 30 | | |
| dihydroartemisinin | 30 | | |
| dihydroartemisinin | 32 | 0.5126 | 16.82 |
| dihydroartemisinin | 32 | 0.5444 | 16.82 |
| dihydroartemisinin | 32 | 0.5390 | 16.88 |
| dihydroartemisinin | 32 | | |
| dihydroartemisinin | 32 | | |
| dihydroartemisinin | 32 | | |
| dihydroartemisinin | 32 | 0.6060 | 16.82 |
| dihydroartemisinin | 32 | 0.5970 | 16.84 |
| dihydroartemisinin | 32 | 0.5970 | 16.87 |
| dihydroartemisinin | 32 | | |
| dihydroartemisinin | 32 | | |
| dihydroartemisinin | 32 | | |

| | | | | |
|---|---|---|---|---|
| dihydroartemisinin | 40 | | 0.5981 | 16.34 |
| dihydroartemisinin | | 40 | 0.6064 | 16.36 |
| dihydroartemisinin | 40 | | 0.6872 | 18.59 |
| dihydroartemisinin | 40 | | 0.6746 | 18.25 |
| dihydroartemisinin | 40 | | 0.6632 | 18.31 |
| dihydroartemisinin | 40 | | 0.4677 | 10.59 |
| dihydroartemisinin | 40 | | 0.4644 | 10.60 |
| dihydroartemisinin | 40 | | 0.4710 | 10.60 |
| dihydroartemisinin | 40 | | | |
| dihydroartemisinin | 40 | | | |
| dihydroartemisinin | 40 | | 0.4214 | 11.07 |
| dihydroartemisinin | 40 | | 0.6942 | 12.10 |
| dihydroartemisinin | 40 | | 0.5200 | 12.16 |
| dihydroartemisinin | 40 | | 0.5251 | 12.23 |
| dihydroartemisinin | 40 | | 0.6876 | 16.61 |
| dihydroartemisinin | 40 | | 0.6881 | 16.68 |
| dihydroartemisinin | 40 | | 0.5055 | 20.54 |
| dihydroartemisinin | 40 | | 0.5096 | 20.34 |
| dihydroartemisinin | 40 | | 0.5130 | 20.47 |
| dihydroartemisinin | 40 | | | |
| dihydroartemisinin | 40 | | | |
| dihydroartemisinin | 40 | | | |
| dihydroartemisinin | 40 | | | |
| dihydroartemisinin | 40 | | | |
| dihydroartemisinin | 40 | | | |
| dihydroartemisinin | 40 | | | |
| dihydroartemisinin | 40 | | | |
| dihydroartemisinin | 40 | | | |
| dihydroartemisinin | 40 | | | |
| dihydroartemisinin | 40 | | | |
| dihydroartemisinin | 40 | | | |
| dihydroartemisinin | 40 | | | |
| dihydroartemisinin | 40 | | | |
| dihydroartemisinin | 40 | | | |
| dihydroartemisinin | 40 | | | |
| dihydroartemisinin | 40 | | | |
| dihydroartemisinin | 40 | | | |
| dihydroartemisinin | 40 | | | |
| dihydroartemisinin | 40 | | | |
| dihydroartemisinin | 40 | | | |
| dihydroartemisinin | 40 | | | |
| dihydroartemisinin | 40 | | | |
| dihydroartemisinin | 40 | | | |
| dihydroartemisinin | 40 | | | |
| dihydroartemisinin | 40 | | | |
| dihydroartemisinin | 40 | | | |
| dihydroartemisinin | 40 | | | |
| dihydroartemisinin | 40 | | | |
| dihydroartemisinin | 40 | | | |
| dihydroartemisinin | 40 | | | |

| | | | |
|---|---|---|---|
| dihydroartemisinin | 40 | | |
| dihydroartemisinin | 40 | | |
| dihydroartemisinin | 40 | | |
| dihydroartemisinin | 40 | | |
| dihydroartemisinin | 40 | | |
| dihydroartemisinin | 40 | | |
| dihydroartemisinin | 40 | | |
| dihydroartemisinin | 40 | | |
| dihydroartemisinin | 40 | | |
| dihydroartemisinin | 40 | | |
| dihydroartemisinin | 40 | | |
| dihydroartemisinin | 40 | | |
| dihydroartemisinin | 40 | | |
| dihydroartemisinin | 40 | | |
| dihydroartemisinin | 40 | | |
| dihydroartemisinin | 40 | | |
| dihydroartemisinin | 40 | | |
| dihydroartemisinin | 40 | | |
| dihydroartemisinin | 40 | | |
| dihydroartemisinin | 40 | | |
| dihydroartemisinin | 40 | | |
| dihydroartemisinin | 40 | | |
| dihydroartemisinin | 40 | | |
| dihydroartemisinin | 40 | | |
| dihydroartemisinin | 40 | | |
| dihydroartemisinin | 40 | | |
| dihydroartemisinin | 40 | | |
| dihydroartemisinin | 40 | | |
| dihydroartemisinin | 40 | | |
| dihydroartemisinin | 40 | | |
| dihydroartemisinin | 40 | | |
| dihydroartemisinin | 40 | | |
| dihydroartemisinin | 40 | | |
| dihydroartemisinin | 40 | | |
| dihydroartemisinin | 40 | | |
| dihydroartemisinin | 40 | | |
| dihydroartemisinin | 40 | | |
| dihydroartemisinin | 40 | | |
| dihydroartemisinin | 40 | | |
| dihydroartemisinin | 40 | | |
| dihydroartemisinin | 40 | | |
| dihydroartemisinin | 40 | | |
| dihydroartemisinin | 40 | | |
| dihydroartemisinin | 40 | | |
| dihydroartemisinin | 40 | | |
| dihydroartemisinin | 40 | 0.5521 | 12.18 |
| dihydroartemisinin | 40 | 0.5559 | 12.19 |
| dihydroartemisinin | 40 | 0.5574 | 12.16 |
| dihydroartemisinin | 40 | 0.5421 | 12.18 |
| dihydroartemisinin | 40 | 0.5575 | 12.13 |

| | | | | |
|---|---|---|---|---|
| dihydroartemisinin | 40 | | 0.5226 | 16.56 |
| dihydroartemisinin | | 40 | 0.5175 | 16.67 |
| dihydroartemisinin | 40 | | 0.5476 | 16.66 |
| dihydroartemisinin | 40 | | | |
| dihydroartemisinin | 40 | | | |
| dihydroartemisinin | 40 | | | |
| dihydroartemisinin | 40 | | 0.5615 | 12.21 |
| dihydroartemisinin | 40 | | 0.5625 | 12.22 |
| dihydroartemisinin | 40 | | 0.5436 | 12.22 |
| dihydroartemisinin | 40 | | | |
| dihydroartemisinin | 40 | | | |
| dihydroartemisinin | 40 | | 0.5241 | 16.16 |
| dihydroartemisinin | 40 | | 0.5306 | 16.24 |
| dihydroartemisinin | 40 | | 0.5307 | 16.27 |
| dihydroartemisinin | 40 | | | |
| dihydroartemisinin | 40 | | | |
| dihydroartemisinin | 40 | | | |
| dihydroartemisinin | 40 | | | |
| dihydroartemisinin | 40 | | | |
| dihydroartemisinin | 40 | | | |
| dihydroartemisinin | 40 | | 0.6710 | 18.45 |
| artemether | | 180 | | |
| artemether | | 180 | | |
| artemether | | 180 | | |
| artemether | | 180 | | |
| artemether | | 180 | | |
| artemether | | 180 | | |
| artemether | | 180 | | |
| artemether | | 180 | | |
| artemether | | 180 | | |
| artemether | | 180 | | |
| artemether | 180 | | | |
| artemether | 180 | | | |
| artemether | 180 | | | |
| artemether | 180 | | | |
| artemether | 180 | | | |
| artemether | 180 | | | |
| artemether | 180 | | | |
| artemether | 180 | | | |
| artemether | 180 | | | |
| artemether | 180 | | | |
| artemether | 180 | | | |
| artemether | 180 | | | |
| artemether | 180 | | | |
| artemether | 180 | | | |
| artemether | 180 | | | |
| artemether | 180 | | | |
| artemether | 180 | | | |
| artemether | 180 | | | |
| artemether | 180 | | | |

| | | |
|---|---|---|
| artemether | 180 | |
| artemether | 80 | |
| artemether | 180 | |
| artemether | 180 | |
| artemether | 180 | |
| artemether | 180 | |
| artemether | 180 | |
| artemether | 180 | |
| artemether | | 180 |
| artemether | 180 | |
| artemether | 180 | |
| artemether | 180 | |
| artemether | 180 | |
| artemether | 180 | |
| artemether | 180 | |
| artemether | 180 | |
| artemether | 180 | |
| artemether | 180 | |
| artemether | 180 | |
| artemether | 180 | |
| artemether | 180 | |
| artemether | 180 | |
| artemether | 15 | |
| artemether | 180 | |
| artemether | 240 | |
| artemether | 240 | |
| artemether | 240 | |
| artemether | 240 | |
| artemether | 240 | |
| artemether | 240 | |
| artemether | 240 | |
| artemether | 240 | |
| artemether | 240 | |
| artemether | 240 | |
| artemether | 240 | |
| artemether | 240 | |
| artemether | 240 | |
| artemether | 240 | |
| artemether | 240 | |
| artemether | 240 | |
| artemether | 240 | |
| artemether | 240 | |
| artemether | 240 | |
| artemether | 240 | |
| artemether | 240 | |
| artemether | 240 | |
| artemether | 240 | |
| artemether | 240 | |
| artemether | 240 | |
| artemether | 240 | |

| | | | | |
|---|---|---|---|---|
| artemether | 240 | | | |
| artemether | 240 | | | |
| artemether | 240 | | | |
| artemether | 240 | | | |
| artemether | 240 | | | |
| artemether | 240 | | | |
| artemether | 240 | | | |
| artemether | 240 | | | |
| artemether | 240 | | | |
| artemether | 240 | | | |
| artemether | 240 | | | |
| artemether | 240 | | | |
| artemether | | 240 | | |
| artemether | 40 | | 0.2453 | 18.13 |
| artemether | 40 | | 0.2221 | 18.33 |
| artemether | 40 | | | |
| artemether | 40 | | | |
| artemether | 40 | | | |
| artemether | 80 | | 1.3184 | 21.55 |
| artemether | 40 | | 0.6777 | 13.54 |
| artemether | 40 | | 0.6809 | 14.15 |
| artemether | 20 | | 0.9841 | |
| artemether | 20 | | 0.9531 | |
| artemether | 20 | | | |
| artesunate | 160 | | | |
| artesunate | 160 | | | |
| artesunate | 160 | | | |
| artesunate | | 160 | | |
| artesunate | 160 | | | |
| artesunate | 160 | | | |
| artesunate | 160 | | | |
| artesunate | 160 | | | |
| artesunate | 160 | | | |
| artesunate | 160 | | | |
| artesunate | 160 | | | |
| artesunate | 160 | | | |
| artesunate | 160 | | | |
| artesunate | 160 | | | |
| artesunate | 160 | | | |
| artesunate | 160 | | | |
| artesunate | 200 | | | |
| artesunate | 200 | | | |
| dihydroartemisinin | 80 | | | |
| dihydroartemisinin | 80 | | | |
| dihydroartemisinin | 80 | | | |
| dihydroartemisinin | 80 | | | |
| dihydroartemisinin | 80 | | | |
| dihydroartemisinin | 80 | | | |
| dihydroartemisinin | 80 | | | |
| dihydroartemisinin | 80 | | | |

| | | | | |
|---|---|---|---|---|
| dihydroartemisinin | 80 | | | |
| dihydroartemisinin | 80 | | | |
| dihydroartemisinin | 80 | | | |
| dihydroartemisinin | 80 | | | |
| dihydroartemisinin | 80 | | | |
| dihydroartemisinin | 80 | | | |
| dihydroartemisinin | 80 | | | |
| dihydroartemisinin | 80 | | | |
| dihydroartemisinin | 80 | | | |
| dihydroartemisinin | 80 | | | |
| dihydroartemisinin | 80 | | | |
| dihydroartemisinin | 80 | | | |
| dihydroartemisinin | 80 | | | |
| dihydroartemisinin | 80 | | | |
| dihydroartemisinin | 80 | | | |
| dihydroartemisinin | 80 | | | |
| dihydroartemisinin | 80 | | | |
| dihydroartemisinin | 80 | | | |
| dihydroartemisinin | | 80 | | |
| dihydroartemisinin | 80 | | | |
| dihydroartemisinin | 80 | | | |
| dihydroartemisinin | 80 | | | |
| dihydroartemisinin | 80 | | | |
| dihydroartemisinin | 80 | | | |
| dihydroartemisinin | 80 | | | |
| dihydroartemisinin | 80 | | | |
| dihydroartemisinin | 80 | | | |
| dihydroartemisinin | 80 | | | |
| dihydroartemisinin | 80 | | | |
| dihydroartemisinin | 80 | | | |
| dihydroartemisinin | | 80 | | |
| dihydroartemisinin | 90 | | | |
| dihydroartemisinin | 90 | | | |
| dihydroartemisinin | 90 | | | |
| dihydroartemisinin | 90 | | | |
| dihydroartemisinin | 240 | | | |
| artemether | 20 | | 0.2941 | 9.25 |
| artemether | 20 | | 0.2480 | 9.06 |
| artemether | 20 | | 0.2515 | 9.15 |
| artemether | 20 | | 0.262 | 9.16 |
| artemether | 20 | | 0.2430 | 9.1 |
| artemether | 20 | | 0.2506 | 9.09 |
| artemether | 20 | | | |
| artemether | 20 | | | |
| artemether | 20 | | | |
| artemether | 20 | | | |
| artemether | 20 | | | |
| artemether | 20 | | | |
| artemether | 20 | | | |
| artemether | 20 | | | |

| | | | |
|---|---|---|---|
| artemether | 20 | | |
| artemether | 20 | | |
| artemether | 20 | | |
| artemether | 20 | | |
| artemether | 20 | | |
| artemether | 20 | | |
| artemether | 20 | | |
| artemether | 20 | | |
| artemether | 20 | | |
| artemether | 20 | | |
| artemether | 20 | | |
| artemether | 20 | | |
| artemether | 20 | | |
| artemether | 20 | | |
| artemether | 20 | | |
| artemether | 20 | | |
| artemether | 20 | | |
| artemether | 20 | | |
| artemether | 20 | | |
| artemether | 20 | | |
| artemether | 20 | | |
| artemether | 20 | | |
| artemether | 20 | | |
| artemether | 20 | | |
| artemether | 20 | | |
| artemether | 20 | | |
| artemether | 20 | | |
| artemether | 20 | | |
| artemether | 20 | | |
| artemether | 20 | | |
| artemether | 20 | | |
| artemether | 20 | | |
| artemether | 20 | | |
| artemether | 20 | | |
| artemether | 20 | | |
| artemether | 20 | | |
| artemether | 20 | | |
| artemether | 20 | 0.3272 | 9.60 |
| artemether | 20 | 0.3297 | 9.65 |
| artemether | 20 | 0.3280 | 9.64 |
| artemether | 20 | 0.3316 | 9.61 |
| artemether | 20 | 0.3294 | 9.62 |
| artemether | 20 | | |
| artemether | 20 | | |
| artemether | 20 | | |
| artemether | 20 | | |
| artemether | 20 | | |
| artemether | 20 | 0.2966 | 9.20 |
| artemether | 20 | 0.2840 | 9.14 |
| artemether | 20 | 0.2878 | 9.21 |

| | | | |
|---|---|---|---|
| artemether | 20 | 0.3001 | 9.24 |
| artemether | 20 | 0.3456 | 10.15 |
| artemether | 20 | 0.3563 | 10.14 |
| artemether | 20 | 0.3569 | 10.08 |
| artemether | 20 | 0.3520 | 10.06 |
| artemether | 20 | 0.3539 | 10.10 |
| artemether | 20 | 0.3545 | 10.08 |
| artemether | 20 | 0.2963 | 10.95 |
| artemether | 20 | 0.2437 | 9.02 |
| artemether | 20 | 0.2406 | 9.05 |
| artemether | 20 | 0.2382 | 9.15 |
| artemether | 20 | 0.2447 | 9.06 |
| artemether | 20 | 0.2426 | 9.05 |
| artemether | 20 | 0.2412 | 9.07 |
| artemether | 20 | 0.2444 | 9.07 |
| artemether | 20 | | |
| artemether | 20 | | |
| artemether | 20 | | |
| artemether | 20 | | |
| artemether | 20 | | |
| artemether | 20 | | |
| artemether | 20 | | |
| artemether | 20 | | |
| artemether | 20 | | |
| artemether | 20 | | |
| artemether | 20 | | |
| artemether | 20 | | |
| artemether | 20 | | |
| artemether | 20 | | |
| artemether | 20 | | |
| artemether | 20 | | |
| artemether | 20 | | |
| artemether | 20 | | |
| artemether | 20 | | |
| artemether | 20 | | |
| artemether | 20 | | |
| artemether | 20 | | |
| artemether | 20 | | |
| artemether | 20 | | |
| artemether | 20 | | |
| artemether | 20 | | |
| artemether | 20 | | |
| artemether | 20 | | |
| artemether | 20 | | |
| artemether | 20 | | |
| artemether | 20 | | |
| artemether | 20 | | |
| artemether | 20 | | |
| artemether | 20 | | |
| artemether | 20 | | |

| | | | |
|---|---|---|---|
| artemether | 20 | | |
| artemether | 20 | | |
| artemether | 20 | | |
| artemether | 20 | | |
| artemether | 20 | | |
| artemether | 20 | | |
| artemether | 20 | | |
| artemether | 20 | 0.3360 | 9.44 |
| artemether | 20 | 0.3356 | 9.43 |
| artemether | 20 | | |
| artemether | 20 | | |
| artemether | 20 | 0.2427 | 9.08 |
| artemether | 20 | | |
| artemether | 20 | 0.2401 | 9.06 |
| artemether | 20 | | |
| artemether | 20 | | |
| artemether | 20 | | |
| artemether | 20 | | |
| artemether | 20 | | |
| artemether | 20 | | |
| artemether | 20 | | |
| artemether | 20 | | |
| artemether | 20 | | |
| artemether | 20 | | |
| artemether | 20 | | |
| artemether | 20 | 0.2863 | 9.2 |
| artemether | 20 | 0.2845 | 9.18 |
| artemether | 20 | 0.2879 | 9.24 |
| artemether | 20 | 0.2861 | 9.15 |
| artemether | 20 | 0.2863 | 9.17 |
| artemether | 20 | | |
| artemether | 20 | | |
| artemether | 20 | | |
| artemether | 20 | | |
| artemether | 20 | | |
| artemether | 20 | | |
| artemether | 20 | | |
| artemether | 20 | | |
| artemether | 20 | | |
| artemether | 20 | | |
| artemether | 20 | | |
| artemether | 20 | 0.2461 | 9.25 |
| artemether | 20 | 0.2502 | 9.17 |
| artemether | 20 | 0.2485 | 9.17 |
| artemether | 20 | 0.2494 | 9.17 |
| artemether | 20 | 0.2483 | 9.16 |
| artemether | 20 | 0.2450 | 9.16 |
| artemether | 20 | 0.2458 | 9.17 |
| artemether | 20 | 0.2437 | 9.22 |
| artemether | 20 | 0.2446 | 9.10 |

| | | | |
|---|---|---|---|
| artemether | 20 | 0.2410 | 9.15 |
| artemether | 20 | 0.2470 | 9.08 |
| artemether | 20 | 0.2460 | 9.11 |
| artemether | 20 | 0.3571 | 10.18 |
| artemether | 20 | 0.3512 | 10.21 |
| artemether | 20 | 0.3539 | 10.09 |
| artemether | 20 | 0.3532 | 10.08 |
| artemether | 20 | 0.3455 | 10.11 |
| artemether | 20 | 0.3504 | 10.06 |
| artemether | 20 | | |
| artemether | 20 | | |
| artemether | 20 | | |
| artemether | 20 | | |
| artemether | 20 | | |
| artemether | 20 | | |
| artemether | 20 | | |
| artemether | 20 | | |
| artemether | 20 | | |
| artemether | 20 | | |
| artemether | 20 | | |
| artemether | 20 | | |
| artemether | 20 | | |
| artemether | 20 | | |
| artemether | 20 | | |
| artemether | 20 | | |
| artemether | 20 | | |
| artemether | 20 | | |
| artemether | 20 | | |
| artemether | 20 | | |
| artemether | 20 | | |
| artemether | 20 | | |
| artemether | 20 | | |
| artemether | 20 | | |
| artemether | 20 | | |
| artemether | 20 | | |
| artemether | 20 | | |
| artemether | 20 | | |
| artemether | 20 | | |
| artemether | 20 | | |
| artemether | 20 | | |
| artemether | 20 | | |
| artemether | 20 | | |
| artemether | 20 | | |
| artemether | 20 | | |
| artemether | 20 | | |
| artemether | 20 | | |
| artemether | 20 | | |
| artemether | 20 | | |
| artemether | 20 | | |
| artemether | 20 | | |

| | | | |
|---|---|---|---|
| artemether | 20 | | |
| artemether | 20 | | |
| artemether | 20 | | |
| artemether | 20 | | |
| artemether | 20 | | |
| artemether | 20 | | |
| artemether | 20 | | |
| artemether | 20 | | |
| artemether | 20 | | |
| artemether | 20 | | |
| artemether | 20 | | |
| artemether | 20 | | |
| artemether | 20 | | |
| artemether | 20 | | |
| artemether | 20 | | |
| artemether | 20 | | |
| artemether | 20 | | |
| artemether | 20 | | |
| artemether | 20 | | |
| artemether | 20 | | |
| artemether | 20 | | |
| artemether | 20 | | |
| artemether | 20 | | |
| artemether | 20 | | |
| artemether | 20 | 0.2267 | 8.87 |
| artemether | 20 | 0.1989 | 8.81 |
| artemether | 20 | 0.3067 | 10.09 |
| artemether | 20 | 0.2655 | 9.09 |
| artemether | 20 | 0.2660 | 9.27 |
| artemether | 20 | 0.2780 | 9.11 |
| artemether | 20 | 0.2603 | 9.12 |
| artemether | 20 | 0.2702 | 9.12 |
| artemether | 20 | 0.2781 | 9.05 |
| artemether | 20 | 0.2691 | 9.17 |
| artemether | 40 | 0.6062 | 12.12 |
| artemether | 40 | 0.3717 | 10.14 |
| artemether | 40 | 0.3821 | 10.06 |
| artemether | 40 | 0.4561 | 11.05 |
| artemether | 40 | 0.4451 | 11.01 |
| artemether | 40 | 0.4517 | 11.02 |
| artemether | 40 | 0.4485 | 11.03 |
| artemether | 40 | 0.4493 | 11.02 |
| artemether | 40 | | |
| artemether | 40 | | |
| artemether | 40 | | |
| artemether | 40 | | |
| artemether | 40 | | |
| artemether | 40 | | |
| artemether | 40 | | |
| artemether | 40 | | |

| | | | | |
|---|---|---|---|---|
| artemether | 40 | | | |
| artemether | 40 | | | |
| artemether | 40 | | | |
| artemether | | 40 | 0.6414 | 12.29 |
| artemether | 40 | | 0.5534 | 12.65 |
| artemether | 40 | | 0.5832 | 13.08 |
| artemether | 40 | | 0.5926 | 13.2 |
| artemether | 40 | | 0.3793 | 11.11 |
| artemether | 40 | | 0.4367 | 10.17 |
| artemether | 40 | | 0.3516 | 10.1 |
| artemether | 80 | | 0.7373 | 12.50 |
| artemether | 80 | | 0.7294 | 12.52 |
| artemether | 80 | | 0.7456 | 12.52 |
| artemether | 80 | | 0.7491 | 12.62 |
| artemether | 80 | | 0.7161 | 12.52 |
| artemether | 80 | | | |
| artemether | 80 | | | |
| artemether | 80 | | | |
| artemether | 80 | | | |
| artemether | 80 | | | |
| artemether | 80 | | | |
| artemether | 80 | | | |
| artemether | 80 | | | |
| artemether | 80 | | | |
| artemether | 80 | | | |
| artemether | 80 | | | |
| artemether | 80 | | | |
| artemether | 80 | | | |
| artemether | 80 | | | |
| artemether | 80 | | 0.7708 | 12.90 |
| artemether | 80 | | 0.7481 | 12.91 |
| artemether | 80 | | 0.7790 | 12.93 |
| artemether | 80 | | 0.7549 | 13.00 |
| artemether | 80 | | 0.7730 | 12.91 |
| artemether | 80 | | | |
| artemether | 80 | | | |
| artemether | 80 | | | |
| artemether | 80 | | 0.6810 | 12.02 |
| artemether | 80 | | 0.6782 | 12.10 |
| artemether | 80 | | 0.6972 | 12.23 |
| artemether | 80 | | 0.7137 | 12.12 |
| artemether | 80 | | 0.6982 | 12.00 |
| artemether | 80 | | | |
| artemether | 80 | | 0.6677 | 16.30 |
| artemether | 80 | | 0.6782 | 16.46 |
| artemether | 80 | | 0.6775 | 16.44 |
| artemether | 80 | | 0.6751 | 16.38 |
| artemether | 80 | | 0.7904 | 13.18 |
| artemether | 80 | | 0.6280 | 12.64 |
| artemether | 80 | | 0.7702 | 12.72 |

| | | | |
|---|---|---|---|
| artemether | 80 | 0.7296 | 12.78 |
| artemether | 80 | 0.7527 | 12.75 |
| artemether | 80 | 0.6919 | 12.24 |
| artemether | 80 | 0.6982 | 12.29 |
| artemether | 80 | 0.7117 | 12.28 |
| artemether | 80 | 0.6156 | 12.72 |
| artemether | 80 | 0.6405 | 12.86 |
| artemether | 80 | 0.6254 | 12.72 |
| artemether | 80 | 0.6384 | 12.74 |
| artemether | 80 | 0.6405 | 12.71 |
| artemether | 80 | | |
| artemether | 80 | | |
| artemether | 80 | 0.8415 | 14.24 |
| artemether | 80 | 0.8309 | 14.27 |
| artemether | 80 | 0.8456 | 14.22 |
| artemether | 80 | 0.8552 | 14.14 |
| artemether | 80 | 0.8480 | 14.19 |
| artemether | 80 | | |
| artemether | 80 | | |
| artemether | 80 | 0.7738 | 12.25 |
| artemether | 80 | 0.7618 | 12.23 |
| artemether | 80 | 0.7470 | 12.00 |
| artemether | 80 | 0.6640 | 12.01 |
| artemether | 80 | 0.7738 | 12.89 |
| artemether | 80 | 0.7619 | 12.99 |
| artemether | 80 | 0.7807 | 12.88 |
| artemether | 80 | 0.7586 | 12.96 |
| artemether | 80 | 0.7688 | 12.87 |
| artemether | 80 | 0.6909 | 12.29 |
| artemether | 80 | 0.6929 | 12.13 |
| artemether | 80 | 0.6828 | 12.16 |
| artemether | 80 | 0.6990 | 12.15 |
| artemether | 80 | 0.6769 | 12.13 |
| artemether | 80 | 0.6840 | 12.16 |
| artemether | 80 | | |
| artemether | 80 | | |
| artemether | 80 | | |
| artemether | 80 | | |
| artemether | 80 | | |
| artemether | 80 | 0.6700 | 16.10 |
| artemether | 80 | 0.7018 | 12.49 |
| artemether | 80 | 0.6957 | 12.48 |
| artemether | 80 | 0.7015 | 11.92 |
| artemether | 80 | 0.6820 | 11.91 |
| artemether | 80 | 0.7750 | 11.92 |
| artemether | 80 | 0.6704 | 11.95 |
| artemether | 80 | 0.7126 | 11.92 |
| artemether | 80 | | |
| artemether | 80 | | |
| artemether | 80 | | |

| | | | |
|---|---|---|---|
| artemether | 80 | | |
| artemether | 80 | 0.7700 | 17.47 |
| artemether | 80 | 0.7594 | 17.54 |
| artemether | 80 | 0.7755 | 17.60 |
| artemether | 80 | 0.7487 | 17.60 |
| artemether | 80 | 0.7666 | 17.46 |
| artemether | 80 | | |
| artemether | 80 | | |
| artemether | 80 | | |
| artemether | 80 | | |
| artemether | 80 | | |
| artemether | 80 | | |
| artemether | 80 | | |
| artemether | 80 | | |
| artemether | 80 | | |
| artemether | 80 | | |
| artemether | 80 | 0.6799 | 12.19 |
| artemether | 80 | 0.6834 | 12.21 |
| artemether | 80 | 0.6733 | 12.19 |
| artemether | 80 | 0.6963 | 12.13 |
| artemether | 80 | 0.6781 | 12.15 |
| artemether | 80 | | |
| artemether | 80 | | |
| artemether | 80 | | |
| artemether | 80 | | |
| artemether | 80 | | |
| artemether | 80 | | |
| artemether | 80 | | |
| artemether | 80 | | |
| artemether | 80 | | |
| artemether | 80 | | |
| artemether | 80 | | |
| artemether | 80 | | |
| artemether | 80 | | |
| artemether | 80 | | |
| artemether | 80 | | |
| artemether | 80 | | |
| artemether | 80 | | |
| artemether | 80 | | |
| artemether | 80 | | |
| artemether | 80 | | |
| artemether | 80 | | |
| artemether | 80 | | |
| artemether | 80 | | |
| artemether | 80 | | |
| artemether | 80 | | |
| artemether | 80 | | |
| artemether | 80 | | |
| artemether | 80 | | |
| artemether | 80 | | |

| | | |
|---|---|---|
| artemether | 80 | |
| artemether | 80 | |
| artemether | 80 | |
| artemether | 80 | |
| artemether | 80 | |
| artemether | 80 | |
| artemether | 80 | |
| artemether | 80 | |
| artemether | 80 | |
| artemether | 80 | |
| artemether | 80 | |
| artemether | 80 | |
| artemether | 80 | |
| artemether | 80 | |
| artemether | 80 | |
| artemether | 80 | |
| artemether | 80 | |
| artemether | 80 | |
| artemether | 80 | |
| artemether | 80 | |
| artemether | 80 | |
| artemether | 80 | |
| artemether | 80 | |
| artemether | 80 | |
| artemether | 80 | |
| artemether | 80 | |
| artemether | 80 | |
| artemether | 80 | |
| artemether | 80 | |
| artemether | 80 | |
| artemether | 80 | |
| artemether | 80 | |
| artemether | 80 | |
| artemether | 80 | |
| artemether | 80 | |
| artemether | 80 | |
| artemether | 80 | |
| artemether | 80 | |
| artemether | 80 | |
| artemether | 80 | |
| artemether | 80 | |
| artemether | 80 | 12.32 |
| artemether | 80 | 12.21 |
| artemether | 80 | 12.18 |
| artemether | 80 | 12.17 |
| artemether | 80 | 18.09 |
| artemether | 80 | 18.08 |
| artemether | 80 | 18.06 |
| artemether | 80 | 18.07 |
| artemether | 80 | 18.06 |

| | | | |
|---|---|---|---|
| artemether | 80 | | |
| artemether | 80 | | |
| artemether | 80 | | |
| artemether | 80 | | |
| artemether | 80 | | |
| artemether | 80 | | |
| artemether | 80 | | |
| artemether | 80 | | |
| artemether | 80 | | |
| artemether | 80 | | |
| artemether | 80 | | 13.64 |
| artemether | 80 | | 13.63 |
| artemether | 80 | | 13.61 |
| artemether | 80 | | 13.59 |
| artemether | 80 | | 13.63 |
| artemether | 80 | | 12.92 |
| artemether | 80 | | 12.13 |
| artemether | 80 | | 12.04 |
| artemether | 80 | | 12.04 |
| artemether | 80 | | 12.06 |
| artemether | 80 | | 12.11 |
| artemether | 80 | | |
| artemether | 80 | | |
| artemether | 80 | | |
| artemether | 80 | | |
| artemether | 80 | | |
| artemether | 80 | | 14.17 |
| artemether | 80 | | 14.24 |
| artemether | 80 | | 14.19 |
| artemether | 80 | | 14.2 |
| artemether | 80 | | 14.17 |
| artemether | 80 | | |
| artemether | 80 | | |
| artemether | 80 | | 12.02 |
| artemether | 80 | | 11.95 |
| artemether | 80 | | 11.91 |
| artemether | 80 | | 12.04 |
| artemether | 80 | | 12.04 |
| artemether | 80 | | |
| artemether | 80 | | |
| artemether | 80 | | |
| artemether | 80 | | |
| artemether | 80 | | 19.1 |
| artemether | 80 | | 19.22 |
| artemether | 80 | | 19.2 |
| artemether | 80 | | 19.23 |
| artemether | 80 | | 19.02 |
| artemether | 80 | | |
| artesunate | 50 | 0.2927 | 9.08 |
| artesunate | 50 | 0.2776 | 9.73 |

| | | | | |
|---|---|---|---|---|
| artesunate | | 50 | 0.2948 | 10.04 |
| artesunate | 50 | | 0.3019 | 10.09 |
| artesunate | 50 | | 0.2937 | 10.12 |
| artesunate | 50 | | 0.2959 | 10.03 |
| artesunate | 50 | | 0.3012 | 10.07 |
| artesunate | 50 | | | |
| artesunate | 50 | | | |
| artesunate | 50 | | | |
| artesunate | 50 | | | |
| artesunate | 50 | | | |
| artesunate | 50 | | | |
| artesunate | 50 | | | |
| artesunate | 50 | | | |
| artesunate | 50 | | | |
| artesunate | 50 | | | |
| artesunate | 50 | | | |
| artesunate | 50 | | | |
| artesunate | 50 | | | |
| artesunate | 50 | | | |
| artesunate | 50 | | | |
| artesunate | 50 | | | |
| artesunate | 50 | | | |
| artesunate | 50 | | | |
| artesunate | 50 | | | |
| artesunate | 50 | | | |
| artesunate | 50 | | | |
| artesunate | 50 | | | |
| artesunate | 50 | | | |
| artesunate | 50 | | | |
| artesunate | 50 | | | |
| artesunate | 50 | | | |
| artesunate | 50 | | | |
| artesunate | 50 | | | |
| artesunate | 50 | | | |
| artesunate | 50 | | | |
| artesunate | 50 | | 0.2714 | 9.26 |
| artesunate | 50 | | 0.2678 | 9.28 |
| artesunate | 100 | | 0.1974 | 8.28 |
| artesunate | 100 | | 0.2119 | 8.24 |
| artesunate | 50 | | 0.3545 | 10.17 |
| artesunate | 50 | | 0.3601 | 10.11 |
| artesunate | 50 | | 0.3572 | 10.10 |
| artesunate | 50 | | | |
| artesunate | 50 | | | |
| artesunate | 50 | | | |
| artesunate | 50 | | | |
| artesunate | 50 | | | |
| artesunate | 50 | | | |
| artesunate | 50 | | | |
| artesunate | 50 | | | |

| | | | | |
|---|---|---|---|---|
| artesunate | 50 | | 0.3952 | 11.22 |
| artesunate | 50 | | | |
| artesunate | 50 | | | |
| artesunate | 50 | | | |
| artesunate | 100 | | 0.7086 | 13.16 |
| artesunate | 100 | | 0.7092 | 13.11 |
| artesunate | 100 | | 0.7126 | 13.22 |
| artesunate | 100 | | 0.7078 | 13.11 |
| artesunate | 100 | | 0.7176 | 13.11 |
| artesunate | 100 | | | |
| artesunate | 100 | | | |
| artesunate | 100 | | | |
| artesunate | 100 | | | |
| artesunate | 100 | | | |
| artesunate | 100 | | | |
| artesunate | 100 | | | |
| artesunate | 100 | | | |
| artesunate | 100 | | | |
| artesunate | 100 | | | |
| artesunate | 100 | | | |
| artesunate | 100 | | | |
| artesunate | 100 | | | |
| artesunate | 100 | | | |
| artesunate | 100 | | | |
| artesunate | 100 | | | |
| artesunate | 100 | | | |
| artesunate | 100 | | | |
| artesunate | 100 | | 0.7091 | 13.08 |
| artesunate | 100 | | 0.7062 | 13.16 |
| artesunate | 100 | | 0.6993 | 13.26 |
| artesunate | 100 | | 0.7070 | 13.13 |
| artesunate | | 100 | 0.2823 | 9.10 |
| artesunate | | 100 | 0.2901 | 9.14 |
| artesunate | | 100 | 0.2930 | 9.14 |
| artesunate | 100 | | 0.3040 | 9.12 |
| artesunate | 100 | | 0.2769 | 9.13 |
| artesunate | 100 | | 0.2992 | 9.17 |
| artesunate | 100 | | 0.2891 | 9.10 |
| artesunate | 100 | | 0.2924 | 9.17 |
| artesunate | 100 | | | |
| artesunate | 100 | | | |
| artesunate | 100 | | | |
| artesunate | 100 | | | |
| artesunate | 100 | | | |
| artesunate | 100 | | | |
| artesunate | 100 | | | |
| artesunate | 100 | | | |
| artesunate | 100 | | | |
| artesunate | 100 | | | |

| | | | |
|---|---|---|---|
| artesunate | 100 | | |
| artesunate | 100 | 0.2916 | 9.01 |
| artesunate | 100 | 0.2964 | 9.07 |
| artesunate | 100 | 0.2878 | 9.05 |
| artesunate | 100 | 0.2921 | 9.16 |
| artesunate | 100 | 0.2947 | 9.06 |
| artesunate | 100 | 0.2920 | 9.32 |
| artesunate | 100 | 0.2452 | 9.29 |
| artesunate | 200 | 0.5054 | 16.31 |
| artesunate | 200 | 0.5046 | 16.31 |
| artesunate | 200 | 0.4803 | 16.30 |
| artesunate | 100 | 0.2827 | 13.08 |
| artesunate | 100 | 0.2818 | 13.06 |
| artesunate | 100 | 0.2800 | 13.06 |
| artesunate | 100 | 0.2779 | 13.07 |
| artesunate | 100 | 0.2831 | 13.06 |
| artesunate | 200 | 0.5592 | 19.12 |
| artesunate | 200 | 0.5640 | 19.03 |
| artesunate | 200 | 0.5536 | 19.12 |
| artesunate | 200 | 0.5561 | 19.08 |
| artesunate | 200 | 0.5540 | 19.12 |
| artesunate | 200 | | |
| artesunate | 200 | | |
| artesunate | 200 | | |
| artesunate | 200 | | |
| artesunate | 200 | | |
| artesunate | 200 | | |
| artesunate | 200 | | |
| artesunate | 200 | | |
| artesunate | 200 | | |
| artesunate | 200 | | |
| artesunate | 200 | | |
| artesunate | 200 | | |
| artesunate | 200 | | |
| artesunate | 200 | | |
| artesunate | 200 | 0.5292 | |
| artesunate | 200 | 0.5103 | 16.17 |
| artesunate | 200 | 0.4365 | |
| artesunate | 100 | 0.2867 | 10.23 |
| artesunate | 100 | | |
| artesunate | 100 | 0.5942 | 12.56 |
| artesunate | 100 | 0.6033 | 12.56 |
| artesunate | 100 | 0.6009 | 12.60 |
| artesunate | 100 | 0.6014 | 12.58 |
| artesunate | 100 | 0.5951 | 12.62 |
| artesunate | 100 | | |
| artesunate | 200 | 1.1915 | 21.82 |
| artesunate | 200 | 1.2038 | 21.88 |
| artesunate | 200 | 1.1989 | 21.81 |
| artesunate | 200 | 1.2081 | 21.81 |

| | | | |
|---|---|---|---|
| artesunate | 200 | 1.2167 | 21.86 |
| artesunate | 200 | | |
| artesunate | 200 | | |
| artesunate | 200 | | |
| artesunate | 200 | | |
| artesunate | 200 | | |
| artesunate | 200 | | |
| artesunate | 200 | | |
| artesunate | 200 | | |
| artesunate | 200 | | |
| artesunate | 200 | | |
| dihydroartemisinin | 60 | 0.2130 | 8.19 |
| dihydroartemisinin | 30 | 0.5317 | 12.69 |
| dihydroartemisinin | 30 | 0.5381 | 12.66 |
| dihydroartemisinin | 30 | 0.5538 | 12.66 |
| dihydroartemisinin | 30 | 0.5245 | 12.71 |
| dihydroartemisinin | 30 | 0.5463 | 12.75 |
| dihydroartemisinin | 30 | | |
| dihydroartemisinin | 30 | | |
| dihydroartemisinin | 30 | | |
| dihydroartemisinin | 30 | | |
| dihydroartemisinin | 30 | | |
| dihydroartemisinin | 30 | | |
| dihydroartemisinin | 30 | | |
| dihydroartemisinin | 30 | | |
| dihydroartemisinin | 30 | | |
| dihydroartemisinin | 30 | | |
| dihydroartemisinin | 30 | | |
| dihydroartemisinin | 30 | | |
| dihydroartemisinin | 30 | | |
| dihydroartemisinin | 30 | | |
| dihydroartemisinin | 30 | | |
| dihydroartemisinin | 30 | | |
| dihydroartemisinin | 30 | | |
| dihydroartemisinin | 30 | | |
| dihydroartemisinin | 30 | | |
| dihydroartemisinin | 30 | | |
| dihydroartemisinin | 30 | | |
| dihydroartemisinin | 30 | | |
| dihydroartemisinin | 30 | | |
| dihydroartemisinin | 30 | | |
| dihydroartemisinin | 30 | | |
| dihydroartemisinin | 30 | | |
| dihydroartemisinin | 30 | | |
| dihydroartemisinin | 30 | | |
| dihydroartemisinin | 30 | | |
| dihydroartemisinin | 30 | | |
| dihydroartemisinin | 30 | | |
| dihydroartemisinin | 30 | | |

| | | | |
|---|---|---|---|
| dihydroartemisinin | 30 | | |
| dihydroartemisinin | 30 | | |
| dihydroartemisinin | 30 | | |
| dihydroartemisinin | 30 | | |
| dihydroartemisinin | 30 | | |
| dihydroartemisinin | 30 | | |
| dihydroartemisinin | 30 | | |
| dihydroartemisinin | 30 | | |
| dihydroartemisinin | 30 | | |
| dihydroartemisinin | 30 | | |
| dihydroartemisinin | 30 | | |
| dihydroartemisinin | 30 | | |
| dihydroartemisinin | 30 | | |
| dihydroartemisinin | 30 | | |
| dihydroartemisinin | 30 | | |
| dihydroartemisinin | 30 | | |
| dihydroartemisinin | 30 | | |
| dihydroartemisinin | 30 | | |
| dihydroartemisinin | 30 | | |
| dihydroartemisinin | 30 | | |
| dihydroartemisinin | 30 | | |
| dihydroartemisinin | 30 | | |
| dihydroartemisinin | 30 | | |
| dihydroartemisinin | 30 | | |
| dihydroartemisinin | 30 | | |
| dihydroartemisinin | 30 | | |
| dihydroartemisinin | 30 | | |
| dihydroartemisinin | 30 | | |
| dihydroartemisinin | 30 | | |
| dihydroartemisinin | 30 | | |
| dihydroartemisinin | 30 | | |
| dihydroartemisinin | 32 | 0.5712 | 16.83 |
| dihydroartemisinin | 32 | 0.5460 | 17.00 |
| dihydroartemisinin | 32 | 0.5734 | 16.89 |
| dihydroartemisinin | 32 | 0.6005 | 16.94 |
| dihydroartemisinin | 40 | 0.6003 | 16.38 |
| dihydroartemisinin | 40 | 0.4971 | 20.52 |
| dihydroartemisinin | 40 | 0.5177 | 16.56 |
| dihydroartemisinin | 40 | 0.5290 | 16.59 |
| dihydroartemisinin | 40 | 0.5132 | 16.60 |
| dihydroartemisinin | 40 | 0.5255 | 16.61 |
| dihydroartemisinin | 40 | 0.5293 | 16.59 |
| dihydroartemisinin | 40 | 0.5582 | 12.17 |
| dihydroartemisinin | 40 | 0.5656 | 12.15 |
| dihydroartemisinin | 40 | 0.5489 | 12.22 |
| dihydroartemisinin | 40 | 0.5570 | 12.15 |
| dihydroartemisinin | 40 | 0.5790 | 12.15 |
| dihydroartemisinin | 40 | | |
| dihydroartemisinin | 40 | | |
| dihydroartemisinin | 40 | | |

| | | | |
|---|---|---|---|
| dihydroartemisinin | 40 | | |
| dihydroartemisinin | 40 | | |
| dihydroartemisinin | 40 | | |
| dihydroartemisinin | 40 | | |
| dihydroartemisinin | 40 | | |
| dihydroartemisinin | 40 | | |
| dihydroartemisinin | 40 | | |
| dihydroartemisinin | 40 | | |
| dihydroartemisinin | 40 | | |
| dihydroartemisinin | 40 | | |
| dihydroartemisinin | 40 | | |
| dihydroartemisinin | 40 | | |
| dihydroartemisinin | 40 | | |
| dihydroartemisinin | 40 | | |
| dihydroartemisinin | 40 | | |
| dihydroartemisinin | 40 | | |
| dihydroartemisinin | 40 | | |
| dihydroartemisinin | 40 | | |
| dihydroartemisinin | 40 | | |
| dihydroartemisinin | 40 | | |
| dihydroartemisinin | 40 | | |
| dihydroartemisinin | 40 | | |
| dihydroartemisinin | 40 | | |
| dihydroartemisinin | 40 | | |
| dihydroartemisinin | 40 | | |
| dihydroartemisinin | 40 | | |
| dihydroartemisinin | 40 | | |
| dihydroartemisinin | 40 | | |
| dihydroartemisinin | 40 | | |
| dihydroartemisinin | 40 | | |
| dihydroartemisinin | 40 | | |
| dihydroartemisinin | 40 | | |
| dihydroartemisinin | 40 | | |
| dihydroartemisinin | 40 | | |
| dihydroartemisinin | 40 | | |
| dihydroartemisinin | 40 | | |
| dihydroartemisinin | 40 | | |
| dihydroartemisinin | 40 | | |
| dihydroartemisinin | 40 | 0.5731 | 12.25 |
| dihydroartemisinin | 40 | 0.5296 | 16.31 |
| dihydroartemisinin | 40 | 0.5342 | 16.24 |
| dihydroartemisinin | 40 | 0.5350 | 16.26 |
| dihydroartemisinin | 40 | 0.5404 | 16.28 |
| artemether | 40 | 0.3413 | 18.5 |
| artemether | 40 | 0.6574 | 13.96 |
| artemether | 40 | 0.6757 | 13.5 |
| artemether | 20 | 1.0068 | |
| artemether | 180 | | |
| artemether | 180 | | |

| | | |
|---|---|---|
| artemether | 180 | |
| artemether | 180 | |
| artemether | 180 | |
| artemether | 180 | |
| artemether | 180 | |
| artemether | 180 | |
| artemether | 180 | |
| artemether | 180 | |
| artemether | | 180 |
| artemether | | 180 |
| artemether | 180 | |
| artemether | 180 | |
| artemether | 180 | |
| artemether | 180 | |
| artemether | 180 | |
| artemether | 180 | |
| artemether | | 180 |
| artemether | | 180 |
| artemether | | 180 |
| artemether | | 180 |
| artemether | | 180 |
| artemether | | 180 |
| artemether | | 180 |
| artemether | | 180 |
| artemether | | 180 |
| artemether | | 180 |
| artemether | | 180 |
| artemether | | 180 |
| artemether | | 180 |
| artemether | | 180 |
| artemether | 180 | |
| artemether | 180 | |
| artemether | 180 | |
| artemether | 180 | |
| artemether | 180 | |
| artemether | 180 | |
| artemether | 180 | |
| artemether | 180 | |
| artemether | 180 | |
| artemether | 180 | |
| artemether | 180 | |
| artemether | | 180 |
| artemether | 360 | |
| artemether | 240 | |
| artemether | 240 | |
| artemether | 240 | |
| artemether | 240 | |
| artemether | 240 | |
| artemether | 240 | |
| artemether | 240 | |

| | | | |
|---|---|---|---|
| artemether | 240 | | |
| artemether | 240 | | |
| artemether | 240 | | |
| artemether | 240 | | |
| artemether | 240 | | |
| artemether | 240 | | |
| artemether | 240 | | |
| artemether | 240 | | |
| artemether | 240 | | |
| artemether | 240 | | |
| artemether | 240 | | |
| artemether | 240 | | |
| artemether | 240 | | |
| artemether | 240 | | |
| artemether | 240 | | |
| artemether | 240 | | |
| artemether | 240 | | |
| artemether | 240 | | |
| artemether | 240 | | |
| artemether | 240 | | |
| artemether | 240 | | |
| artemether | 240 | | |
| artemether | 240 | | |
| artemether | 240 | | |
| artemether | 240 | | |
| artemether | 240 | | |
| artemether | 240 | | |
| artemether | 240 | | |
| artemether | 240 | | |
| artesunate | | 25 | 0.5075 |
| artesunate | 25 | | 0.4848 |
| artesunate | 25 | | 0.5046 |
| artesunate | 25 | | 0.5128 |
| artesunate | 50 | | 0.9120 |
| artesunate | 50 | | 1.0412 |
| artesunate | 50 | | 1.0130 |
| artesunate | 50 | | 0.9891 |
| artesunate | 50 | | 0.9918 |
| artesunate | 50 | | |
| artesunate | 50 | | |
| artesunate | 50 | | |
| artesunate | 160 | | |
| artesunate | | 160 | |
| artesunate | 160 | | |
| artesunate | 160 | | |
| artesunate | 160 | | |
| artesunate | 160 | | |
| artesunate | 160 | | |
| artesunate | 160 | | |
| artesunate | 160 | | |

| | |
|---|---|
| artesunate | 160 |
| artesunate | 160 |
| artesunate | 160 |
| artesunate | 160 |
| artesunate | 160 |
| artesunate | 160 |
| artesunate | 160 |
| artesunate | 160 |
| artesunate | 160 |
| artesunate | 160 |
| dihydroartemisinin | 80 |
| dihydroartemisinin | 80 |
| dihydroartemisinin | 80 |
| dihydroartemisinin | 80 |
| dihydroartemisinin | 80 |
| dihydroartemisinin | 80 |
| dihydroartemisinin | 80 |
| dihydroartemisinin | 80 |
| dihydroartemisinin | 80 |
| dihydroartemisinin | 80 |
| dihydroartemisinin | 80 |
| dihydroartemisinin | 80 |
| dihydroartemisinin | 80 |
| dihydroartemisinin | 80 |
| dihydroartemisinin | 80 |
| dihydroartemisinin | 80 |
| dihydroartemisinin | 80 |
| dihydroartemisinin | 80 |
| dihydroartemisinin | 80 |
| dihydroartemisinin | 80 |
| dihydroartemisinin | 90 |
| dihydroartemisinin | 90 |
| dihydroartemisinin | 90 |
| dihydroartemisinin | 90 |

## Enug[illegible]

### Data of Con[illegible]

| LSHTM | CDC | GT MS2 | strengthegen2 |
|---|---|---|---|
| **103.1** | | | 120 |
| **90.3** | | | 120 |
| **94.7** | | | 120 |
| **89.7** | | | 120 |
| **92.5** | | | 120 |
| **97.0** | | | 120 |
| **97.5** | | | 120 |
| 96.6 | | | 120 |
| 97.3 | | | 120 |
| 97.9 | | | 120 |
| **92.3** | | | 120 |
| **100.8** | | | 120 |
| **90.7** | | | 120 |
| **93.5** | | | 120 |
| **95.6** | | | 120 |
| **98.6** | | | 120 |
| **93.7** | | | 120 |
| **91.4** | | | 120 |
| **109.7** | | | 120 |
| **99.2** | | | 120 |
| **89.4** | | | 120 |
| **100.1** | | | 120 |
| **94.6** | | | 120 |
| **98.7** | | | 120 |
| **99.5** | | | 120 |
| **Not detectable** | | | 120 |
| **100.2** | | | 120 |
| **94.2** | | | 120 |
| **97.4** | | | 120 |
| **100.5** | | | 120 |
| **92.6** | | | 120 |
| **98.1** | | | 120 |
| **99.4** | | | 120 |
| **99.6** | | | 120 |
| **102.7** | | | 120 |
| 100.3 | | | 120 |
| **97.9** | | | 120 |
| **96.5** | | | 120 |
| **99.8** | | | 120 |
| **98.4** | | | 120 |
| **104.6** | | | 120 |
| **106.2** | | | 120 |
| **103.0** | | | 120 |
| **107.7** | | | 120 |
| **104.8** | | | 120 |
| **112.4** | | | 120 |

| | | |
|---|---|---|
| **123.1** | | 120 |
| **95.8** | | 120 |
| **99.8** | | 120 |
| **88.3** | | 120 |
| **92.4** | | 120 |
| **95.3** | | 120 |
| **90.6** | | 120 |
| **92.7** | | 120 |
| **98.8** | | 120 |
| **98.4** | | 120 |
| **104.1** | | 120 |
| 124.6 | | 120 |
| 99.7 | | 240 |
| 97.7 | | 240 |
| 96 | | 240 |
| 93.8 | | 240 |
| 106.3 | | 240 |
| | | 240 |
| | | 240 |
| **114.5** | | 480 |
| **105.6** | | 480 |
| **99.4** | | 480 |
| **95.0** | | 480 |
| **108.0** | | 480 |
| **Not detectable** | | 480 |
| **101.6** | | 480 |
| | 101.6 | 480 |
| **112.6** | | 480 |
| **102.0** | | 480 |
| **108.1** | | 480 |
| **106.7** | | 480 |
| 103.7 | | 480 |
| 95.1 | | 480 |
| 109.9 | | 480 |
| 110 | | 480 |
| 105.3 | | 480 |
| **103.2** | | 480 |
| **100.7** | | 480 |
| **86.7** | | 480 |
| **94.4** | | 480 |
| **92.9** | | 480 |
| **95.8** | | 480 |
| **99.8** | | 480 |
| **88.3** | | 480 |
| **92.4** | | 480 |
| **95.3** | | 480 |
| **90.6** | | 480 |
| **92.7** | | 480 |
| **98.8** | | 480 |
| **Degraded** | | 480 |

| | | | |
|---|---|---|---|
| **94.8** | | | 480 |
| **96.5** | | | 480 |
| **86.8** | | | 480 |
| **98.3** | | | 480 |
| **91.7** | | | 480 |
| **95.3** | | | 480 |
| **90.6** | | | 480 |
| **92.9** | | | 480 |
| **90.1** | | | 480 |
| **91.9** | | | 480 |
| 92.7 | | | 480 |
| 101.1 | | | 480 |
| 98.8 | 10.2 | | 480 |
| 100.4 | | | 480 |
| **Degraded** | | | 480 |
| 96.5 | | | 480 |
| 86.8 | | | 480 |
| 98.3 | | | 480 |
| **91.7** | | | 480 |
| **95.3** | | | 480 |
| **90.6** | | | 480 |
| **92.9** | | | 480 |
| **90.1** | | | 480 |
| **91.9** | | | 480 |
| **92.7** | | | 480 |
| **98.8** | | | 480 |
| **100.4** | | | 480 |
| **92.7** | | | 480 |
| **90.3** | | | 480 |
| **90.6** | | | |
| **92.9** | | | |
| **90.1** | | | |
| **91.9** | | | |
| 102.8 | 102.9 | | 500 |
| **Not detectable** | | | |
| 99.4 | 90.5 | Yes | |
| 102.5 | 101.3 | Yes | |
| 99.9 | 99 | Yes | |
| | | | |
| 95.6 | 93.1 | | 270 |
| 99.6 | 102.5 | | 270 |
| 98.3 | 95 | | 270 |
| 96.5 | 96.5 | | 270 |
| 94.8 | 98.9 | | 300 |
| | | | 300 |

| | | | |
|---|---|---|---|
| | | | 300 |
| 98.9 | 101.2 | | 300 |
| 97.5 | 103.4 | | 300 |
| | | | 300 |
| 99.6 | 99.5 | | 300 |
| 87.9 | | | 306.3 |
| 89.6 | 100.3 | | 300 |
| 96.3 | 91.2 | | 125 |
| 99.5 | 102.1 | | 125 |
| | | | |
| 98.6 | 94.2 | | |
| 102.8 | 102.9 | | 600 |
| 96.3 | 63.4 | | 600 |
| | 68/99 | | 780 |
| | | | 250 |
| 99.6 | 100.2 | | 250 |
| 96.3 | 108.7 | | 250 |
| 99.3 | 104.3 | | 250 |
| 98.5 | 104.4 | | 250 |
| 100.2 | 102.8 | | 250 |
| 103.2 | 104.7 | | 250 |
| **Not detectable** | | | 250 |
| 91.7 | | | 500 |
| **Degraded** | 73.3 / 47.9 | | 500 |
| **Degraded** | 16.8 / 91.5 | | 500 |
| **99.6/98.6** | 93.1/102.8 | | 500 |
| **98.6/99.6** | | | 500 |
| **98.8/100.2** | 85.8/102.2 | | 500 |
| **98.6/99.5** | 96.3/96 | | 500 |
| **89.3/96.3** | 99.4/98.1 | | 500 |
| 102.3 | 101 | Yes | 225 |
| 99.6 | 7 | Yes | 225 |
| **Not detectable** | **Not detectable** | **Not detectable** | 225 |
| **105.3** | 104.8 | Yes | 225 |
| **103.5** | 106.8 | Yes | 225 |
| **98.6** | 101 | Yes | 225 |
| **110.3** | 127.6 | Yes | 225 |
| **Not detectable** | **Not detectable** | **Not detectable** | 225 |
| **78.3** | 110.9 | Yes | 225 |
| **98.5** | 99.6 | Yes | 225 |
| **100.3** | 109.6 | Yes | 225 |
| **95.8** | 106.6 | Yes | 225 |
| **96.8** | 109.9 | Yes | 225 |
| **87.3** | 107 | Yes | 225 |
| **89.6** | 104.9 | Yes | 225 |
| **87.9** | 78.1 | Yes | 320 |
| **88.9** | 81.7 | Yes | 320 |
| **89.2** | 88 | Yes | 320 |

| | | | |
|---|---|---|---|
| **98.6** | 63.7 | Yes | 320 |
| **90.3** | 78.7 | Yes | 320 |
| **89.6** | 73.9 | Yes | 320 |
| **92.6** | 77.6 | Yes | 320 |
| **85.6** | 81.4 | Yes | 320 |

| Height (mm) | Length (mm) | analysis date | HPLC (%) mean of t |
|---|---|---|---|
| 3.58 | | 06/02/2013 | **88.8** |
| 3.51 | | 06/02/2013 | **112.3** |
| 3.61 | | 06/02/2013 | **104.2** |
| 3.03 | | 01/02/2013 | **97.0** |
| 3.03 | | 01/02/2013 | **110.2** |
| 3.03 | | 01/02/2013 | **101.2** |
| 3.01 | | 01/02/2013 | **97.2** |
| 3.06 | | 01/02/2013 | **93.4** |
| 3.00 | | 01/02/2013 | **102.2** |
| 3.09 | | 01/02/2013 | **97.8** |
| 9.04 | | 01/02/2013 | **97.3** |
| 3.06 | | 01/02/2013 | **101.8** |
| 3.02 | | 01/02/2013 | **99.2** |
| 3.06 | | 01/02/2013 | **103.1** |
| 3.16 | | 01/02/2013 | **97.7** |
| 3.09 | | 01/02/2013 | **100.6** |
| 3.10 | | 01/02/2013 | **96.2** |
| 3.07 | | 01/02/2013 | **105.5** |
| 3.08 | | 01/02/2013 | **91.7** |
| 3.05 | | 01/02/2013 | **103.0** |
| 3.07 | | 01/02/2013 | **93.4** |
| 3.08 | | 01/02/2013 | **100.0** |
| 3.10 | | 01/02/2013 | **97.4** |
| 3.05 | | 01/02/2013 | **103.0** |
| 3.07 | | 01/02/2013 | **101.2** |
| 3.06 | | 01/02/2013 | **98.5** |
| 3.06 | | 01/02/2013 | **112.5** |
| 3.08 | | 01/02/2013 | **110.3** |
| 3.04 | | 01/02/2013 | **104.1** |
| 3.04 | | 01/02/2013 | **101.4** |
| 3.10 | | 01/02/2013 | **102.5** |
| 3.05 | | 01/02/2013 | **98.2** |
| 3.05 | | 01/02/2013 | **92.7** |
| 3.09 | | 01/02/2013 | **94.9** |
| 3.07 | | 01/02/2013 | **97.0** |
| 3.08 | | 01/02/2013 | **104.3** |
| 3.09 | | 01/02/2013 | **88.8** |
| 3.02 | | 01/02/2013 | **87.0** |
| 3.10 | | 01/02/2013 | **90.1** |

| | | |
|---|---|---|
| 3.02 | 01/02/2013 | **109.3** |
| 3.04 | 01/02/2013 | **112.0** |
| 3.13 | 01/02/2013 | **101.4** |
| 3.08 | 01/02/2013 | **107.3** |
| 3.07 | 01/02/2013 | **93.5** |
| 3.04 | 01/02/2013 | **98.4** |
| 3.03 | 01/02/2013 | **96.9** |
| 3.08 | 04/02/2013 | **90.9** |
| 3.10 | 04/02/2013 | **97.3** |
| 3.04 | 04/02/2013 | **96.3** |
| 3.17 | 04/02/2013 | **95.9** |
| 3.07 | 04/02/2013 | **93.8** |
| 3.09 | 04/02/2013 | **96.1** |
| 2.98 | 04/02/2013 | **92.7** |
| 3.04 | 04/02/2013 | **90.6** |
| 3.03 | 04/02/2013 | **92.4** |
| 2.95 | 04/02/2013 | **96.2** |
| 3.06 | 04/02/2013 | **97.9** |
| 3.06 | 04/02/2013 | **89.0** |
| 3.05 | 04/02/2013 | **95.2** |
| 3.10 | 04/02/2013 | **94.1** |
| 3.02 | 04/02/2013 | **97.7** |
| 3.06 | 04/02/2013 | **95.2** |
| 3.08 | 04/02/2013 | **92.9** |
| 3.02 | 04/02/2013 | **96.1** |
| 3.03 | 04/02/2013 | **97.8** |
| 3.10 | 04/02/2013 | **95.4** |
| 3.04 | 04/02/2013 | **102.4** |
| 3.08 | 04/02/2013 | **95.7** |
| 3.02 | 04/02/2013 | **98.3** |
| 3.04 | 04/02/2013 | **91.5** |
| 3.09 | 04/02/2013 | **94.6** |
| 3.05 | 04/02/2013 | **93.9** |
| 3.08 | 04/02/2013 | **93.8** |
| 3.10 | 04/02/2013 | **95.1** |
| 3.10 | 04/02/2013 | **100.7** |
| 3.09 | 04/02/2013 | **96.8** |
| 3.13 | 04/02/2013 | **106.1** |
| 3.10 | 04/02/2013 | **100.5** |
| 3.04 | 04/02/2013 | **85.4** |
| 3.05 | 04/02/2013 | **109.7** |
| 3.10 | 04/02/2013 | **100.2** |
| 3.70 | 04/02/2013 | **105.4** |
| 3.04 | 04/02/2013 | **108.5** |
| 3.18 | 04/02/2013 | **113.2** |
| 3.13 | 04/02/2013 | **102.6** |
| 3.00 | 04/02/2013 | **106.3** |
| 3.09 | 04/02/2013 | **102.1** |
| 3.07 | 04/02/2013 | **100.4** |
| 3.10 | 04/02/2013 | **102.7** |

| | | |
|---|---|---|
| 3.08 | 04/02/2013 | **93.5** |
| 3.12 | 04/02/2013 | **114.1** |
| 3.14 | 04/02/2013 | **98.1** |
| 3.05 | 04/02/2013 | **103.7** |
| 3.03 | 04/02/2013 | **103.0** |
| 3.14 | 04/02/2013 | **103.7** |
| 3.09 | 04/02/2013 | **96.8** |
| 3.11 | 04/02/2013 | **100.5** |
| 3.03 | 04/02/2013 | **95.7** |
| 3.09 | 04/02/2013 | **102.4** |
| 3.05 | 04/02/2013 | **98.9** |
| 3.09 | 04/02/2013 | **110.6** |
| 3.06 | 04/02/2013 | **97.4** |
| 3.07 | 04/02/2013 | **96.5** |
| 3.12 | 04/02/2013 | **95.1** |
| 3.11 | 05/02/2013 | **92.1** |
| 3.06 | 05/02/2013 | **94.5** |
| 3.00 | 05/02/2013 | **98.6** |
| 3.04 | 05/02/2013 | **86.8** |
| 3.10 | 05/02/2013 | **95.5** |
| 3.02 | 05/02/2013 | **92.8** |
| 3.04 | 05/02/2013 | **90.1** |
| 3.06 | 05/02/2013 | **90.5** |
| 3.00 | 05/02/2013 | **96.1** |
| 3.02 | 05/02/2013 | **93.6** |
| 2.99 | 05/02/2013 | **96.4** |
| 3.10 | 05/02/2013 | **103.8** |
| 3.07 | 05/02/2013 | **98.1** |
| 3.01 | 05/02/2013 | **99.0** |
| 3.04 | 05/02/2013 | **104.2** |
| 3.07 | 05/02/2013 | **92.0** |
| 3.10 | 05/02/2013 | **102.7** |
| 3.12 | 05/02/2013 | **99.8** |
| 3.11 | 05/02/2013 | **101.6** |
| 3.02 | 05/02/2013 | **107.5** |
| 3.13 | 05/02/2013 | **103.3** |
| 3.01 | 05/02/2013 | **96.1** |
| 3.05 | 05/02/2013 | **110.5** |
| 3.00 | 05/02/2013 | **99.2** |
| 3.02 | 05/02/2013 | **106.9** |
| 3.13 | 05/02/2013 | **98.2** |
| 3.07 | 05/02/2013 | **98.5** |
| 3.00 | 05/02/2013 | **104.6** |
| 3.01 | 05/02/2013 | **108.4** |
| 3.04 | 05/02/2013 | **105.9** |
| 3.08 | 05/02/2013 | **97.9** |
| 3.02 | 05/02/2013 | **101.2** |
| 3.13 | 05/02/2013 | **109.0** |
| 3.14 | 05/02/2013 | **98.0** |
| 3.05 | 05/02/2013 | **100.1** |

| | | |
|---|---|---|
| 3.06 | 05/02/2013 | **100.5** |
| 3.06 | 05/02/2013 | **99.9** |
| 3.07 | 05/02/2013 | **97.5** |
| 3.06 | 05/02/2013 | **103.1** |
| 3.01 | 05/02/2013 | **100.0** |
| 3.14 | 05/02/2013 | **100.3** |
| 3.03 | 05/02/2013 | **106.6** |
| 3.00 | 05/02/2013 | **104.1** |
| 3.17 | 05/02/2013 | **107.2** |
| 3.07 | 05/02/2013 | **108.7** |
| 3.02 | 05/02/2013 | **111.5** |
| 3.03 | 05/02/2013 | **104.2** |
| 3.03 | 05/02/2013 | **114.2** |
| 3.09 | 05/02/2013 | **112.3** |
| 3.08 | 05/02/2013 | **114.7** |
| 3.03 | 05/02/2013 | **114.5** |
| 3.02 | 05/02/2013 | **100.8** |
| 3.12 | 05/02/2013 | **100.5** |
| 3.67 | 06/02/2013 | **84.3** |
| 3.62 | 06/02/2013 | **87.1** |
| 3.67 | 06/02/2013 | **94.6** |
| 3.63 | 06/02/2013 | **64.6** |
| 3.71 | 06/02/2013 | **93.1** |
| 3.64 | 06/02/2013 | **97.1** |
| 3.73 | 06/02/2013 | **98.9** |
| 3.66 | 06/02/2013 | **94.9** |
| 3.65 | 06/02/2013 | **105.2** |
| 3.68 | 06/02/2013 | **95.2** |
| 3.72 | 06/02/2013 | **109.8** |
| 3.69 | 06/02/2013 | **101.5** |
| 3.75 | 06/02/2013 | **43.0** |
| 3.65 | 06/02/2013 | **90.7** |
| 3.66 | 06/02/2013 | **111.6** |
| 4.68 | 06/02/2013 | **0.3** |
| 4.56 | 06/02/2013 | **0.8** |
| 4.65 | 06/02/2013 | **1.1** |
| 4.68 | 06/02/2013 | **0.5** |
| 4.80 | 06/02/2013 | **0.3** |
| 4.65 | 06/02/2013 | **1.2** |
| 4.70 | 06/02/2013 | **0.3** |
| 4.76 | 06/02/2013 | **0.9** |
| 4.67 | 06/02/2013 | **0.1** |
| 4.66 | 06/02/2013 | **0.5** |
| 4.57 | 06/02/2013 | **0.4** |
| 3.15 | 06/02/2013 | **105.1** |
| 3.42 | 06/02/2013 | **103.9** |
| 3.34 | 06/02/2013 | **106.7** |
| 3.30 | 06/02/2013 | **109.5** |
| 3.24 | 06/02/2013 | **111.3** |
| 3.28 | 06/02/2013 | **109.3** |

| | | |
|---|---|---|
| 3.28 | 06/02/2013 | **107.9** |
| 3.25 | 06/02/2013 | **108.4** |
| 3.26 | 06/02/2013 | **106.3** |
| 3.24 | 06/02/2013 | **111.8** |
| 3.45 | 06/02/2013 | **106.8** |
| 3.47 | 06/02/2013 | **106.8** |
| 3.46 | 06/02/2013 | **94.2** |
| 3.18 | 06/02/2013 | **99.0** |
| 3.25 | 07/02/2013 | **101.8** |
| 3.25 | 07/02/2013 | **100.5** |
| 3.08 | 07/02/2013 | **103.4** |
| 3.09 | 07/02/2013 | **103.5** |
| 2.99 | 07/02/2013 | **100.3** |
| 3.12 | 07/02/2013 | **104.9** |
| 3.04 | 07/02/2013 | **103.9** |
| 3.07 | 07/02/2013 | **84.6** |
| 3.04 | 07/02/2013 | **68.4** |
| 3.11 | 07/02/2013 | **62.9** |
| 3.23 | 07/02/2013 | **42.5** |
| 3.23 | 07/02/2013 | **102.9** |
| 3.16 | 07/02/2013 | **107.2** |
| 3.19 | 07/02/2013 | **107.1** |
| 3.18 | 07/02/2013 | **107.3** |
| 3.17 | 07/02/2013 | **88.5** |
| 3.16 | 08/02/2013 | **107.6** |
| 3.15 | 08/02/2013 | **107.5** |
| 3.10 | 08/02/2013 | **104.7** |
| 3.18 | 08/02/2013 | **108.4** |
| 3.16 | 08/02/2013 | **110.4** |
| 3.21 | 08/02/2013 | **103.8** |
| 3.18 | 08/02/2013 | **107.7** |
| 3.19 | 08/02/2013 | **112.7** |
| 3.14 | 08/02/2013 | **107.1** |
| 3.16 | 08/02/2013 | **108.8** |
| 3.14 | 08/02/2013 | **111.3** |
| 3.18 | 08/02/2013 | **109.4** |
| 3.18 | 08/02/2013 | **114.3** |
| 3.20 | 08/02/2013 | **100.0** |
| 3.17 | 08/02/2013 | **106.9** |
| 3.16 | 08/02/2013 | **109.7** |
| 3.16 | 08/02/2013 | **105.8** |
| 3.13 | 08/02/2013 | **109.0** |
| 3.15 | 08/02/2013 | **104.3** |
| 3.16 | 08/02/2013 | **104.3** |
| 3.14 | 08/02/2013 | **103.7** |
| 3.15 | 08/02/2013 | **92.1** |
| 3.16 | 08/02/2013 | **96.3** |
| 3.17 | 08/02/2013 | **94.1** |
| 3.16 | 08/02/2013 | **91.6** |
| 3.13 | 08/02/2013 | **91.5** |

| | | |
|---|---|---|
| 3.17 | 08/02/2013 | **87.4** |
| 3.16 | 08/02/2013 | **101.8** |
| 3.16 | 08/02/2013 | **96.4** |
| 3.16 | 08/02/2013 | **93.0** |
| 3.16 | 08/02/2013 | **96.3** |
| 3.15 | 08/02/2013 | **98.3** |
| 3.17 | 08/02/2013 | **94.2** |
| 3.14 | 08/02/2013 | **89.2** |
| 3.15 | 08/02/2013 | **99.4** |
| 3.17 | 08/02/2013 | **91.4** |
| 3.18 | 08/02/2013 | **92.5** |
| 3.16 | 08/02/2013 | **111.1** |
| 3.17 | 08/02/2013 | **95.0** |
| 3.18 | 08/02/2013 | **112.1** |
| 3.08 | 11/02/2013 | **106.8** |
| 3.09 | 11/02/2013 | **110.3** |
| 3.06 | 11/02/2013 | **107.3** |
| 3.09 | 11/02/2013 | **107.8** |
| 2.99 | 11/02/2013 | **106.0** |
| 3.11 | 11/02/2013 | **99.7** |
| 3.07 | 11/02/2013 | **99.9** |
| 3.09 | 11/02/2013 | **99.9** |
| 3.05 | 11/02/2013 | **110.3** |
| 3.15 | 11/02/2013 | **112.7** |
| 3.07 | 11/02/2013 | **106.2** |
| 3.04 | 11/02/2013 | **112.0** |
| 3.15 | 11/02/2013 | **86.4** |
| 3.22 | 11/02/2013 | **92.7** |
| 3.10 | 11/02/2013 | **94.8** |
| 3.18 | 11/02/2013 | **87.0** |
| 3.07 | 11/02/2013 | **94.4** |
| 3.16 | 11/02/2013 | **93.2** |
| 3.17 | 11/02/2013 | **87.1** |
| 3.13 | 11/02/2013 | **85.6** |
| 3.13 | 11/02/2013 | **90.6** |
| 3.19 | 11/02/2013 | **96.1** |
| 3.15 | 11/02/2013 | **95.6** |
| 3.14 | 11/02/2013 | **102.2** |
| 3.19 | 11/02/2013 | **105.6** |
| 3.22 | 11/02/2013 | **93.5** |
| 3.14 | 11/02/2013 | **90.9** |
| 3.11 | 11/02/2013 | **93.3** |
| 3.16 | 11/02/2013 | **103.5** |
| 3.16 | 11/02/2013 | **101.0** |
| 3.19 | 11/02/2013 | **108.6** |
| 3.17 | 11/02/2013 | **93.8** |
| 3.17 | 11/02/2013 | **90.1** |
| 3.40 | 11/02/2013 | **Not detectable** |
| 3.17 | 11/02/2013 | **106.1** |
| 3.10 | 11/02/2013 | **104.1** |

| | | |
|---|---|---|
| 3.18 | 11/02/2013 | **91.3** |
| 3.13 | 11/02/2013 | **97.2** |
| 3.18 | 11/02/2013 | **100.7** |
| 3.20 | 11/02/2013 | **92.5** |
| 3.13 | 11/02/2013 | **96.7** |
| 3.15 | 11/02/2013 | **98.2** |
| 3.19 | 11/02/2013 | **97.2** |
| 3.22 | 11/02/2013 | **92.7** |
| 3.15 | 11/02/2013 | **105.3** |
| 3.09 | 11/02/2013 | **100.0** |
| 3.06 | 11/02/2013 | **87.5** |
| 3.11 | 11/02/2013 | **109.3** |
| 3.14 | 11/02/2013 | **113.3** |
| 3.16 | 11/02/2013 | **107.6** |
| 3.11 | 11/02/2013 | **109.1** |
| 3.13 | 11/02/2013 | **110.6** |
| 3.13 | 11/02/2013 | **108.1** |
| 3.21 | 11/02/2013 | **100.2** |
| 3.10 | 11/02/2013 | **94.1** |
| 3.13 | 11/02/2013 | **96.1** |
| 3.15 | 11/02/2013 | **109.3** |
| 3.19 | 11/02/2013 | **98.2** |
| 3.12 | 11/02/2013 | **109.5** |
| 3.16 | 11/02/2013 | **92.6** |
| 3.18 | 11/02/2013 | **110.7** |
| 3.10 | 11/02/2013 | **91.8** |
| 3.12 | 11/02/2013 | **97.6** |
| 3.11 | 11/02/2013 | **96.3** |
| 3.12 | 11/02/2013 | **112.4** |
| 3.15 | 11/02/2013 | **114.8** |
| 3.21 | 11/02/2013 | **109.0** |
| 3.31 | 11/02/2013 | **Not detectable** |
| 3.18 | 11/02/2013 | **108.1** |
| 3.01 | 11/02/2013 | **100.3** |
| 3.18 | 11/02/2013 | **94.8** |
| 3.01 | 11/02/2013 | **101.6** |
| 3.16 | 11/02/2013 | **99.2** |
| 3.11 | 11/02/2013 | **92.4** |
| 3.21 | 11/02/2013 | **99.9** |
| 3.23 | 11/02/2013 | **91.9** |
| 3.10 | 11/02/2013 | **99.3** |
| 4.17 | 12/02/2013 | **106.5** |
| 4.28 | 12/02/2013 | **104.2** |
| 4.24 | 12/02/2013 | **102.2** |
| 4.25 | 12/02/2013 | **102.7** |
| 4.25 | 12/02/2013 | **104.5** |
| 4.14 | 12/02/2013 | **102.9** |
| 4.22 | 12/02/2013 | **105.0** |
| 4.21 | 12/02/2013 | **106.2** |
| 4.24 | 12/02/2013 | **102.0** |

| | | |
|---|---|---|
| 4.20 | 12/02/2013 | **105.4** |
| 4.26 | 12/02/2013 | **97.4** |
| 4.27 | 12/02/2013 | **104.2** |
| 4.22 | 12/02/2013 | **100.7** |
| 4.23 | 12/02/2013 | **109.1** |
| 4.33 | 12/02/2013 | **106.1** |
| 4.18 | 12/02/2013 | **106.2** |
| 4.19 | 12/02/2013 | **107.9** |
| 4.23 | 12/02/2013 | **106.8** |
| 4.19 | 12/02/2013 | **101.1** |
| 4.20 | 12/02/2013 | **103.7** |
| 4.19 | 12/02/2013 | **98.1** |
| 4.27 | 12/02/2013 | **104.7** |
| 4.09 | 12/02/2013 | **114.7** |
| 4.17 | 12/02/2013 | **104.3** |
| 4.13 | 12/02/2013 | **106.9** |
| 4.22 | 12/02/2013 | **105.2** |
| 4.27 | 12/02/2013 | **104.1** |
| 4.20 | 12/02/2013 | **102.8** |
| 4.20 | 12/02/2013 | **107.3** |
| 4.19 | 12/02/2013 | **109.3** |
| 4.15 | 12/02/2013 | **106.8** |
| 4.15 | 12/02/2013 | **106.8** |
| 4.23 | 12/02/2013 | **102.3** |
| 4.16 | 12/02/2013 | **102.8** |
| 4.19 | 12/02/2013 | **103.6** |
| 4.24 | 12/02/2013 | **104.7** |
| 4.32 | 12/02/2013 | **103.1** |
| 4.21 | 12/02/2013 | **109.0** |
| 4.34 | 12/02/2013 | **108.9** |
| 4.22 | 12/02/2013 | **88.3** |
| 4.35 | 12/02/2013 | **100.1** |
| 4.20 | 12/02/2013 | **86.9** |
| 4.18 | 12/02/2013 | **102.8** |
| 4.29 | 12/02/2013 | **106.4** |
| 4.20 | 12/02/2013 | **108.8** |
| 4.24 | 12/02/2013 | **99.6** |
| 4.28 | 12/02/2013 | **113.0** |
| 4.23 | 12/02/2013 | **105.5** |
| 4.13 | 12/02/2013 | **105.3** |
| 4.20 | 12/02/2013 | **100.1** |
| 4.25 | 12/02/2013 | **109.2** |
| 4.21 | 12/02/2013 | **102.2** |
| 4.18 | 12/02/2013 | **104.1** |
| 4.33 | 12/02/2013 | **96.2** |
| 4.15 | 12/02/2013 | **107.3** |
| 4.15 | 12/02/2013 | **106.1** |
| 4.22 | 12/02/2013 | **101.8** |
| 4.22 | 12/02/2013 | **107.7** |
| 4.23 | 12/02/2013 | **104.2** |

| | | |
|---|---|---|
| 4.21 | 12/02/2013 | **107.8** |
| 4.21 | 12/02/2013 | **104.0** |
| 4.28 | 12/02/2013 | **102.0** |
| 4.25 | 12/02/2013 | **104.8** |
| 4.18 | 12/02/2013 | **114.6** |
| 4.18 | 12/02/2013 | **104.3** |
| 4.19 | 12/02/2013 | **104.2** |
| 4.19 | 12/02/2013 | **109.9** |
| 4.29 | 12/02/2013 | **106.8** |
| 3.78 | 07/02/2013 | **113.7** |
| 3.85 | 07/02/2013 | **111.4** |
| 3.48 | 07/02/2013 | **101.7** |
| 3.13 | 07/02/2013 | **113.0** |
| 3.09 | 07/02/2013 | **106.3** |
| 3.11 | 07/02/2013 | **106.4** |
| 3.11 | 07/02/2013 | **95.8** |
| 3.09 | 07/02/2013 | **111.9** |
| 3.16 | 07/02/2013 | **97.8** |
| 3.11 | 07/02/2013 | **99.3** |
| 3.18 | 07/02/2013 | **111.8** |
| 3.09 | 07/02/2013 | **113.7** |
| 3.12 | 07/02/2013 | **92.7** |
| 3.23 | 07/02/2013 | **110.3** |
| 3.17 | 07/02/2013 | **106.5** |
| 3.20 | 07/02/2013 | **86.2** |
| 3.06 | 07/02/2013 | **108.3** |
| 3.23 | 07/02/2013 | **111.2** |
| 3.13 | 07/02/2013 | **100.4** |
| 3.17 | 07/02/2013 | **88.6** |
| 3.14 | 07/02/2013 | **110.5** |
| 3.11 | 07/02/2013 | **112.8** |
| 3.15 | 07/02/2013 | **105.1** |
| 3.11 | 07/02/2013 | **98.4** |
| 3.12 | 07/02/2013 | **99.5** |
| 3.22 | 07/02/2013 | **91.4** |
| 3.07 | 07/02/2013 | **113.6** |
| 3.13 | 07/02/2013 | **109.7** |
| 3.24 | 07/02/2013 | **92.1** |
| 3.16 | 07/02/2013 | **96.8** |
| 3.85 | 07/02/2013 | **95.7** |
| 3.93 | 07/02/2013 | **100.0** |
| 3.80 | 07/02/2013 | **100.6** |
| 4.95 | 07/02/2013 | **0.1** |
| 4.54 | 07/02/2013 | **94.0** |
| 4.43 | 19/02/2013 | **113.7** |
| 3.47 | 13/02/2013 | **92.2** |
| 3.46 | 13/02/2013 | **97.9** |
| 3.44 | 13/02/2013 | **100.4** |
| 3.61 | 13/02/2013 | **98.7** |
| 3.47 | 13/02/2013 | **96.8** |

| | | |
|---|---|---|
| 3.41 | 13/02/2013 | **101.7** |
| 3.56 | 13/02/2013 | **111.8** |
| 3.56 | 13/02/2013 | **100.0** |
| 3.58 | 13/02/2013 | **94.6** |
| 3.63 | 13/02/2013 | **102.8** |
| 3.53 | 13/02/2013 | **92.1** |
| 3.57 | 13/02/2013 | **96.8** |
| 3.54 | 13/02/2013 | **105.6** |
| 3.54 | 13/02/2013 | **110.0** |
| 3.50 | 13/02/2013 | **106.6** |
| 3.62 | 13/02/2013 | **97.1** |
| 3.58 | 13/02/2013 | **100.8** |
| 3.52 | 13/02/2013 | **98.6** |
| 3.49 | 13/02/2013 | **97.8** |
| 3.68 | 13/02/2013 | **111.2** |
| 3.42 | 13/02/2013 | **99.6** |
| 3.66 | 13/02/2013 | **96.4** |
| 3.57 | 13/02/2013 | **108.1** |
| 3.54 | 13/02/2013 | **112.8** |
| 3.55 | 13/02/2013 | **107.2** |
| 3.64 | 13/02/2013 | **107.3** |
| 3.50 | 13/02/2013 | **100.5** |
| 3.56 | 13/02/2013 | **105.5** |
| 3.61 | 13/02/2013 | **106.9** |
| 3.70 | 13/02/2013 | **103.3** |
| 3.41 | 13/02/2013 | **102.7** |
| 3.66 | 13/02/2013 | **108.7** |
| | 23/05/2013 | **105.5** |
| 3.66 | 13/02/2013 | **99.1** |
| 3.63 | 13/02/2013 | **100.9** |
| 3.47 | 13/02/2013 | **107.1** |
| 3.55 | 13/02/2013 | **111.3** |
| 3.67 | 13/02/2013 | **98.3** |
| 3.63 | 13/02/2013 | **107.1** |
| 3.63 | 13/02/2013 | **98.8** |
| 3.43 | 13/02/2013 | **105.9** |
| 3.45 | 13/02/2013 | **97.8** |
| 3.60 | 13/02/2013 | **104.2** |
| 3.48 | 13/02/2013 | **108.4** |
| 3.50 | 13/02/2013 | **108.8** |
| 3.53 | 14/02/2013 | **96.4** |
| 3.58 | 14/02/2013 | **98.4** |
| 3.62 | 14/02/2013 | **100.0** |
| 3.33 | 14/02/2013 | **98.0** |
| 3.51 | 14/02/2013 | **94.5** |
| 3.58 | 14/02/2013 | **101.5** |
| 3.48 | 14/02/2013 | **99.5** |
| 3.45 | 14/02/2013 | **96.8** |
| 3.56 | 14/02/2013 | **96.7** |
| 3.43 | 14/02/2013 | **97.3** |

| | | |
|---|---|---|
| 3.48 | 14/02/2013 | **97.2** |
| 3.56 | 14/02/2013 | **105.5** |
| 3.45 | 14/02/2013 | **96.0** |
| 3.61 | 14/02/2013 | **100.1** |
| 3.57 | 14/02/2013 | **104.6** |
| 3.54 | 14/02/2013 | **100.0** |
| 3.53 | 14/02/2013 | **93.8** |
| 3.51 | 14/02/2013 | **94.6** |
| 3.62 | 14/02/2013 | **97.6** |
| 3.59 | 14/02/2013 | **96.0** |
| 3.54 | 14/02/2013 | **92.8** |
| 3.60 | 14/02/2013 | **87.0** |
| 3.59 | 14/02/2013 | **102.6** |
| 3.35 | 14/02/2013 | **100.5** |
| 3.45 | 14/02/2013 | **92.0** |
| 3.53 | 14/02/2013 | **90.7** |
| 3.43 | 14/02/2013 | **93.0** |
| 3.55 | 14/02/2013 | **93.0** |
| 3.55 | 14/02/2013 | **103.9** |
| 3.48 | 14/02/2013 | **100.0** |
| 3.47 | 14/02/2013 | **94.1** |
| 3.47 | 14/02/2013 | **92.4** |
| 3.58 | 14/02/2013 | **93.6** |
| 3.56 | 14/02/2013 | **96.1** |
| 3.60 | 14/02/2013 | **95.2** |
| 3.55 | 14/02/2013 | **87.1** |
| 3.51 | 14/02/2013 | **99.5** |
| 3.58 | 14/02/2013 | **101.5** |
| 3.46 | 14/02/2013 | **98.3** |
| 3.59 | 14/02/2013 | **100.1** |
| 3.57 | 14/02/2013 | **102.1** |
| 3.62 | 14/02/2013 | **103.6** |
| 3.50 | 14/02/2013 | **85.5** |
| 3.60 | 14/02/2013 | **101.0** |
| 3.63 | 14/02/2013 | **89.1** |
| 3.59 | 14/02/2013 | **91.7** |
| 3.59 | 14/02/2013 | **99.9** |
| 3.56 | 14/02/2013 | **88.9** |
| 3.54 | 14/02/2013 | **90.2** |
| 3.49 | 14/02/2013 | **89.6** |
| 3.53 | 14/02/2013 | **85.2** |
| 3.55 | 14/02/2013 | **102.4** |
| 3.63 | 14/02/2013 | **95.2** |
| 3.60 | 14/02/2013 | **98.3** |
| 3.59 | 14/02/2013 | **103.5** |
| 3.51 | 14/02/2013 | **95.5** |
| 3.41 | 14/02/2013 | **109.9** |
| 3.55 | 14/02/2013 | **110.0** |
| 3.59 | 14/02/2013 | **89.3** |
| 3.62 | 14/02/2013 | **103.9** |

| | | |
|---|---|---|
| 3.48 | 14/02/2013 | **98.5** |
| 3.52 | 14/02/2013 | **96.3** |
| 3.53 | 14/02/2013 | **91.4** |
| 3.58 | 14/02/2013 | **103.2** |
| 3.49 | 14/02/2013 | **114.1** |
| 3.49 | 14/02/2013 | **94.5** |
| 3.55 | 14/02/2013 | **92.9** |
| 3.42 | 14/02/2013 | **93.8** |
| 3.47 | 14/02/2013 | **109.7** |
| 3.58 | 14/02/2013 | **109.7** |
| 3.43 | 14/02/2013 | **103.5** |
| 3.59 | 14/02/2013 | **100.1** |
| 3.59 | 14/02/2013 | **93.8** |
| 3.57 | 14/02/2013 | **93.8** |
| 3.57 | 14/02/2013 | **106.0** |
| 3.56 | 14/02/2013 | **99.8** |
| 3.56 | 14/02/2013 | **92.0** |
| 3.62 | 14/02/2013 | **109.6** |
| 3.46 | 14/02/2013 | **103.1** |
| 3.23 | 18/02/2013 | **100.4** |
| 3.18 | 18/02/2013 | **90.0** |
| 3.26 | 18/02/2013 | **95.1** |
| 3.36 | 18/02/2013 | **87.9** |
| 3.20 | 18/02/2013 | **93.2** |
| 3.33 | 18/02/2013 | **93.8** |
| 3.31 | 18/02/2013 | **90.5** |
| 3.44 | 18/02/2013 | **86.3** |
| 3.44 | 18/02/2013 | **90.7** |
| 3.46 | 18/02/2013 | **103.7** |
| 3.37 | 18/02/2013 | **87.5** |
| 3.39 | 18/02/2013 | **89.1** |
| 3.50 | 18/02/2013 | **91.2** |
| 3.55 | 18/02/2013 | **86.6** |
| 3.35 | 18/02/2013 | **101.1** |
| 3.61 | 18/02/2013 | **85.4** |
| 3.52 | 18/02/2013 | **91.1** |
| 3.51 | 18/02/2013 | **87.7** |
| 3.59 | 18/02/2013 | **102.9** |
| 3.55 | 18/02/2013 | **88.0** |
| 3.39 | 18/02/2013 | **89.8** |
| 3.46 | 18/02/2013 | **94.9** |
| 3.43 | 18/02/2013 | **92.5** |
| 3.43 | 18/02/2013 | **90.7** |
| 3.66 | 18/02/2013 | **103.2** |
| 4.60 | 19/02/2013 | **89.7** |
| 4.99 | 19/02/2013 | **105.0** |
| 4.89 | 19/02/2013 | **101.2** |
| 4.83 | 19/02/2013 | **100.4** |
| 4.94 | 19/02/2013 | **98.2** |
| 4.61 | 19/02/2013 | **94.0** |

| | | |
|---|---|---|
| 5.07 | 19/02/2013 | **104.4** |
| 4.97 | 19/02/2013 | **99.5** |
| 5.00 | 19/02/2013 | **100.4** |
| 4.98 | 19/02/2013 | **106.3** |
| 5.00 | 19/02/2013 | **98.6** |
| 5.03 | 19/02/2013 | **100.8** |
| 5.04 | 19/02/2013 | **102.2** |
| 5.12 | 19/02/2013 | **96.9** |
| 4.87 | 19/02/2013 | **104.6** |
| 5.07 | 19/02/2013 | **101.0** |
| 4.95 | 19/02/2013 | **103.8** |
| 4.99 | 19/02/2013 | **100.5** |
| 5.11 | 19/02/2013 | **103.3** |
| 5.05 | 19/02/2013 | **99.2** |
| 5.05 | 19/02/2013 | **107.9** |
| 4.69 | 19/02/2013 | **110.1** |
| 4.07 | 19/02/2013 | **100.6** |
| 4.07 | 19/02/2013 | **101.3** |
| 3.61 | 19/02/2013 | **109.6** |
| 3.80 | 19/02/2013 | **100.5** |
| 3.85 | 19/02/2013 | **106.2** |
| 3.84 | 19/02/2013 | **105.6** |
| 3.38 | 19/02/2013 | **102.4** |
| 3.21 | 19/02/2013 | **87.3** |
| 4.56 | 19/02/2013 | **103.8** |
| 5.52 | 19/02/2013 | **98.5** |
| 4.75 | 19/02/2013 | **96.7** |
| 4.94 | 20/02/2013 | **91.7** |
| 4.96 | 20/02/2013 | **99.7** |
| 4.94 | 20/02/2013 | **99.7** |
| 4.98 | 20/02/2013 | **106.9** |
| 4.96 | 20/02/2013 | **94.4** |
| 4.85 | 20/02/2013 | **89.6** |
| 4.99 | 20/02/2013 | **94.0** |
| 4.93 | 20/02/2013 | **96.9** |
| 4.90 | 20/02/2013 | **98.1** |
| 4.98 | 20/02/2013 | **94.7** |
| 4.93 | 20/02/2013 | **0.1** |
| 4.89 | 20/02/2013 | **102.5** |
| 4.90 | 20/02/2013 | **97.0** |
| 4.85 | 20/02/2013 | **95.1** |
| 4.97 | 20/02/2013 | **107.5** |
| 4.94 | 20/02/2013 | **99.3** |
| 4.99 | 20/02/2013 | **105.4** |
| 4.91 | 20/02/2013 | **98.5** |
| 4.99 | 20/02/2013 | **97.1** |
| 4.92 | 20/02/2013 | **98.2** |
| 4.94 | 20/02/2013 | **94.8** |
| 4.95 | 20/02/2013 | **92.5** |
| 4.93 | 20/02/2013 | **102.4** |

| | | | |
|---|---|---|---|
| 6.22 | | 20/02/2013 | **109.3** |
| 6.26 | | 20/02/2013 | **100.2** |
| 6.21 | | 20/02/2013 | **107.5** |
| 6.21 | | 20/02/2013 | **111.8** |
| 6.13 | | 20/02/2013 | **98.7** |
| 6.24 | | 20/02/2013 | **107.2** |
| 6.11 | | 20/02/2013 | **102.2** |
| 6.19 | | 20/02/2013 | **100.6** |
| 6.22 | | 20/02/2013 | **106.4** |
| 6.26 | | 20/02/2013 | **103.6** |
| 6.13 | | 20/02/2013 | **101.6** |
| 6.25 | | 20/02/2013 | **105.4** |
| 6.19 | | 20/02/2013 | **102.3** |
| 6.08 | | 20/02/2013 | **99.7** |
| 6.27 | | 20/02/2013 | **103.9** |
| 6.17 | | 20/02/2013 | **106.7** |
| 6.24 | | 20/02/2013 | **109.5** |
| 6.06 | | 20/02/2013 | **96.9** |
| 6.24 | | 20/02/2013 | **108.9** |
| 6.15 | | 20/02/2013 | **111.0** |
| 6.04 | | 20/02/2013 | **100.4** |
| 6.19 | | 20/02/2013 | **100.4** |
| 6.29 | | 20/02/2013 | **105.4** |
| 5.90 | | 20/02/2013 | **86.0** |
| 6.39 | | 20/02/2013 | **91.4** |
| 6.34 | | 20/02/2013 | **112.3** |
| 5.46 | | 20/02/2013 | **104.3** |
| 5.69 | | 20/02/2013 | **85.0** |
| 5.53 | | 20/02/2013 | **98.1** |
| 5.59 | 16.29 | 20/02/2013 | **103.6** |
| 6.19 | 16.33 | 20/02/2013 | **110.4** |
| 5.78 | 16.31 | 20/02/2013 | **107.7** |
| 5.75 | | 22/02/2013 | **95.8** |
| 6.80 | | 22/02/2013 | **108.5** |
| 6.45 | | 22/02/2013 | **111.1** |
| | | 22/02/2013 | **112.4** |
| 4.67 | | 22/02/2013 | **46.3** |
| 4.46 | | 22/02/2013 | **53.8** |
| | | 22/02/2013 | **40.0** |
| | | 22/02/2013 | **49.4** |
| 6.39 | | 22/02/2013 | **110.0** |
| 6.49 | | 22/02/2013 | **111.0** |
| | | 22/02/2013 | **100.0** |
| | | 22/02/2013 | **92.5** |
| | | 22/02/2013 | **90.5** |
| | | 22/02/2013 | **92.5** |
| | | 22/02/2013 | **95.0** |
| 6.21 | | 22/02/2013 | **91.5** |
| 4.29 | | 22/02/2013 | **96.2** |
| | | 22/02/2013 | **95.8** |

| | | | |
|---|---|---|---|
| 4.22 | | 22/02/2013 | **92.3** |
| | | 22/02/2013 | **93.0** |
| | | 22/02/2013 | **93.1** |
| | | 22/02/2013 | **92.6** |
| | | 22/02/2013 | **93.6** |
| | | 22/02/2013 | **90.1** |
| | | 22/02/2013 | **90.1** |
| | | 22/02/2013 | **88.5** |
| | | 22/02/2013 | **89.3** |
| | | 22/02/2013 | **92.5** |
| 6.09 | | 22/02/2013 | **89.5** |
| 6.01 | | 22/02/2013 | **88.9** |
| | | 22/02/2013 | **95.5** |
| | | 22/02/2013 | **103.1** |
| | | 22/02/2013 | **109.6** |
| | | 22/02/2013 | **99.3** |
| | | 22/02/2013 | **87.7** |
| | | 22/02/2013 | **86.6** |
| | | 22/02/2013 | **89.7** |
| | | 22/02/2013 | **102.8** |
| | | 22/02/2013 | **90.9** |
| | | 22/02/2013 | **89.5** |
| | | 22/02/2013 | **96.7** |
| | | 22/02/2013 | **89.5** |
| | | 22/02/2013 | **85.8** |
| | | 22/02/2013 | **89.8** |
| | | 22/02/2013 | **98.0** |
| 5.89 | | 22/02/2013 | **104.0** |
| 5.98 | | 22/02/2013 | **89.6** |
| | | 22/02/2013 | **87.6** |
| | | 22/02/2013 | **95.6** |
| | | 22/02/2013 | **102.1** |
| | | 22/02/2013 | **92.7** |
| | | 22/02/2013 | **89.8** |
| | | 22/02/2013 | **87.1** |
| | | 22/02/2013 | **103.8** |
| | | 22/02/2013 | **93.7** |
| 6.89 | 19.76 | 22/02/2013 | **88.3** |
| 5.25 | | 25/02/2013 | **92.8** |
| 5.22 | | 25/02/2013 | **91.8** |
| 6.08 | | 25/02/2013 | **107.7** |
| 6.32 | | 25/02/2013 | **101.2** |
| | | 25/02/2013 | **94.4** |
| | | 25/02/2013 | **91.6** |
| 6.09 | | 25/02/2013 | **99.4** |
| 5.97 | | 25/02/2013 | **102.7** |
| | | 25/02/2013 | **101.1** |
| | | 25/02/2013 | **103.7** |
| | | 25/02/2013 | **95.2** |
| | | 25/02/2013 | **108.5** |

| | | | |
|---|---|---|---|
| | | 25/02/2013 | **99.4** |
| | | 25/02/2013 | **85.1** |
| | | 25/02/2013 | **99.0** |
| | | 25/02/2013 | **97.3** |
| | | 25/02/2013 | **104.3** |
| 5.96 | | 25/02/2013 | **96.1** |
| 5.93 | | 25/02/2013 | **108.0** |
| 6.04 | | 25/02/2013 | **100.9** |
| 6.10 | | 25/02/2013 | **102.5** |
| | | 25/02/2013 | **101.6** |
| | | 25/02/2013 | **102.2** |
| | | 25/02/2013 | **89.5** |
| | | 25/02/2013 | **90.0** |
| | | 25/02/2013 | **103.9** |
| | | 25/02/2013 | **103.3** |
| | | 25/02/2013 | **106.6** |
| | | 25/02/2013 | **96.0** |
| | | 25/02/2013 | **106.8** |
| | | 25/02/2013 | **103.8** |
| | | 25/02/2013 | **103.8** |
| | | 25/02/2013 | **102.3** |
| | | 25/02/2013 | **106.0** |
| | | 25/02/2013 | **106.9** |
| | | 25/02/2013 | **93.9** |
| | | 25/02/2013 | **107.8** |
| | | 25/02/2013 | **105.1** |
| | | 25/02/2013 | **106.0** |
| | | 25/02/2013 | **106.3** |
| 5.73 | 16.15 | 25/02/2013 | **89.2** |
| 6.16 | | 25/02/2013 | **99.2** |
| 6.35 | | 25/02/2013 | **110.0** |
| | | 25/02/2013 | **101.0** |
| | | 25/02/2013 | **100.9** |
| | | 25/02/2013 | **104.8** |
| | | 25/02/2013 | **104.3** |
| 7.23 | 17.40 | 25/02/2013 | **105.7** |
| | | 25/02/2013 | **95.8** |
| 7.10 | 17.50 | 25/02/2013 | **104.4** |
| | | 25/02/2013 | **100.0** |
| | | 25/02/2013 | **106.2** |
| | | 25/02/2013 | **103.8** |
| | | 25/02/2013 | **104.1** |
| | | 25/02/2013 | **106.8** |
| | | 25/02/2013 | **99.5** |
| | | 25/02/2013 | **102.7** |
| | | 25/02/2013 | **113.5** |
| | | 25/02/2013 | **105.9** |
| | | 25/02/2013 | **99.0** |
| | | 25/02/2013 | **106.4** |
| | | 25/02/2013 | **102.8** |

| | | | |
|---|---|---|---|
| | | 25/02/2013 | **104.0** |
| | | 25/02/2013 | **101.7** |
| | | 27/02/2013 | **110.0** |
| | | 27/02/2013 | **108.7** |
| | | 27/02/2013 | **107.1** |
| | | 27/02/2013 | **107.1** |
| | | 27/02/2013 | **105.3** |
| | | 27/02/2013 | **104.5** |
| | | 27/02/2013 | **100.4** |
| | | 27/02/2013 | **106.9** |
| | | 27/02/2013 | **107.1** |
| | | 27/02/2013 | **106.1** |
| | | 27/02/2013 | Not detectable |
| | | 27/02/2013 | **106.9** |
| | | 27/02/2013 | **108.9** |
| | | 27/02/2013 | **107.9** |
| | | 27/02/2013 | **112.6** |
| | | 27/02/2013 | **108.9** |
| | | 27/02/2013 | **104.9** |
| | | 27/02/2013 | **95.4** |
| | | 27/02/2013 | **109.8** |
| | | 27/02/2013 | **107.5** |
| | | 27/02/2013 | **108.7** |
| | | 27/02/2013 | **110.0** |
| | | 27/02/2013 | **110.0** |
| | | 27/02/2013 | **111.1** |
| | | 27/02/2013 | **100.7** |
| | | 27/02/2013 | **111.1** |
| | | 27/02/2013 | **109.6** |
| | | 27/02/2013 | **106.9** |
| | | 27/02/2013 | **109.9** |
| | | 27/02/2013 | **111.4** |
| | | 27/02/2013 | **113.4** |
| | | 27/02/2013 | **102.3** |
| | | 27/02/2013 | **113.6** |
| | | 27/02/2013 | **110.9** |
| | | 27/02/2013 | **110.7** |
| | | 27/02/2013 | **108.0** |
| | | 27/02/2013 | **111.0** |
| | | 27/02/2013 | **112.3** |
| | | 27/02/2013 | **110.8** |
| | | 27/02/2013 | **113.8** |
| | | 27/02/2013 | **109.7** |
| | | 27/02/2013 | Not detectable |
| | | 27/02/2013 | **103.8** |
| | | 27/02/2013 | **110.2** |
| | | 27/02/2013 | **105.0** |
| | | 27/02/2013 | **111.6** |
| 6.28 | | 27/02/2013 | **105.9** |
| 6.47 | | 28/02/2013 | **101.3** |

| | |
|---|---|
| 28/02/2013 | **102.2** |
| 28/02/2013 | **96.7** |
| 28/02/2013 | **104.4** |
| 28/02/2013 | **98.5** |
| 28/02/2013 | **106.5** |
| 28/02/2013 | **93.5** |
| 28/02/2013 | **106.3** |
| 28/02/2013 | **107.6** |
| 28/02/2013 | **107.7** |
| 28/02/2013 | **97.8** |
| 28/02/2013 | **107.8** |
| 28/02/2013 | **108.4** |
| 28/02/2013 | **107.2** |
| 28/02/2013 | **102.6** |
| 28/02/2013 | **104.2** |
| 28/02/2013 | **112.4** |
| 28/02/2013 | **110.3** |
| 28/02/2013 | **86.3** |
| 28/02/2013 | **107.9** |
| 28/02/2013 | **108.9** |
| 28/02/2013 | **109.5** |
| 28/02/2013 | **110.0** |
| 28/02/2013 | **114.9** |
| 28/02/2013 | **111.2** |
| 28/02/2013 | **108.6** |
| 28/02/2013 | **111.9** |
| 28/02/2013 | **112.6** |
| 28/02/2013 | **110.4** |
| 28/02/2013 | **102.5** |
| 28/02/2013 | **109.9** |
| 28/02/2013 | **96.1** |
| 28/02/2013 | **110.7** |
| 28/02/2013 | **109.5** |
| 28/02/2013 | **108.3** |
| 28/02/2013 | **109.3** |
| 28/02/2013 | **109.8** |
| 28/02/2013 | **110.0** |
| 28/02/2013 | **107.8** |
| 28/02/2013 | **112.1** |
| 28/02/2013 | **112.5** |
| 28/02/2013 | **110.0** |
| 28/02/2013 | **104.6** |
| 28/02/2013 | **109.6** |
| 28/02/2013 | **111.4** |
| 28/02/2013 | **90.6** |
| 28/02/2013 | **113.6** |
| 28/02/2013 | **114.6** |
| 28/02/2013 | **110.8** |
| 28/02/2013 | **112.1** |
| 28/02/2013 | **111.2** |

| | | | |
|---|---|---|---|
| | | 28/02/2013 | **111.7** |
| | | 28/02/2013 | **112.0** |
| | | 28/02/2013 | **113.0** |
| | | 28/02/2013 | **111.6** |
| | | 28/02/2013 | **Not detectable** |
| | | 28/02/2013 | **97.0** |
| | | 28/02/2013 | **105.6** |
| | | 28/02/2013 | **110.1** |
| | | 28/02/2013 | **109.8** |
| | | 28/02/2013 | **95.6** |
| | | 28/02/2013 | **105.2** |
| | | 28/02/2013 | **110.0** |
| | | 28/02/2013 | **113.8** |
| | | 28/02/2013 | **106.0** |
| | | 28/02/2013 | **95.9** |
| | | 28/02/2013 | **106.3** |
| | | 28/02/2013 | **114.9** |
| | | 28/02/2013 | **90.3** |
| 6.29 | | 25/02/2013 | **96.5** |
| 5.97 | | 25/02/2013 | **108.4** |
| | | 25/02/2013 | **100.3** |
| | | 25/02/2013 | **98.0** |
| | | 25/02/2013 | **106.0** |
| | | 25/02/2013 | **107.4** |
| 5.86 | 18.07 | 26/02/2013 | **107.5** |
| 6.06 | 18.04 | 26/02/2013 | **108.8** |
| | | 26/02/2013 | **104.4** |
| | | 26/02/2013 | **103.1** |
| | | 26/02/2013 | **106.6** |
| | | 26/02/2013 | **103.0** |
| | | 26/02/2013 | **103.0** |
| | | 26/02/2013 | **109.7** |
| | | 26/02/2013 | **102.8** |
| | | 26/02/2013 | **112.4** |
| | | 26/02/2013 | **106.2** |
| | | 26/02/2013 | **104.7** |
| | | 26/02/2013 | **110.7** |
| | | 26/02/2013 | **92.2** |
| | | 26/02/2013 | **106.2** |
| | | 26/02/2013 | **112.2** |
| | | 26/02/2013 | **92.9** |
| | | 26/02/2013 | **93.6** |
| | | 26/02/2013 | **97.0** |
| 5.64 | 18.99 | 26/02/2013 | **93.1** |
| 5.64 | | 26/02/2013 | **94.9** |
| 5.67 | | 26/02/2013 | **98.2** |
| | | 26/02/2013 | **86.6** |
| | | 26/02/2013 | **92.3** |
| 6.08 | | 26/02/2013 | **94.6** |
| 6.18 | | 26/02/2013 | **85.5** |

| | | | |
|---|---|---|---|
| | | 26/02/2013 | **95.3** |
| | | 26/02/2013 | **92.5** |
| 6.14 | | 26/02/2013 | **96.2** |
| 5.95 | | 26/02/2013 | **107.6** |
| | | 26/02/2013 | **96.0** |
| | | 26/02/2013 | **93.2** |
| | | 26/02/2013 | **98.8** |
| | | 26/02/2013 | **108.8** |
| | | 26/02/2013 | **89.9** |
| | | 26/02/2013 | **113.9** |
| | | 26/02/2013 | **0.1** |
| | | 26/02/2013 | **105.2** |
| | | 26/02/2013 | **110.4** |
| | | 26/02/2013 | **107.2** |
| | | 26/02/2013 | **94.7** |
| | | 26/02/2013 | **114.9** |
| | | 26/02/2013 | **94.5** |
| | | 26/02/2013 | **97.7** |
| | | 26/02/2013 | **91.2** |
| | | 26/02/2013 | **96.0** |
| | | 26/02/2013 | **99.4** |
| | | 26/02/2013 | **93.7** |
| | | 26/02/2013 | **95.5** |
| 5.94 | | 26/02/2013 | **102.1** |
| 6.04 | | 26/02/2013 | **101.2** |
| | | 26/02/2013 | **104.8** |
| | | 26/02/2013 | **102.9** |
| | | 26/02/2013 | **109.1** |
| | | 26/02/2013 | **99.7** |
| | | 26/02/2013 | **105.1** |
| | | 26/02/2013 | **105.7** |
| | | 26/02/2013 | **107.6** |
| | | 26/02/2013 | **98.4** |
| | | 26/02/2013 | **113.2** |
| | | 26/02/2013 | **105.8** |
| 6.2300 | | 26/02/2013 | **100.0** |
| 6.21 | | 26/02/2013 | **108.6** |
| | | 26/02/2013 | **101.4** |
| | | 26/02/2013 | **95.8** |
| | | 26/02/2013 | **107.1** |
| | | 26/02/2013 | **104.2** |
| | | 26/02/2013 | **109.5** |
| | | 26/02/2013 | **106.3** |
| | | 26/02/2013 | **110.1** |
| | | 26/02/2013 | **107.7** |
| | | 26/02/2013 | **102.4** |
| | | 26/02/2013 | **97.4** |
| | | 26/02/2013 | **108.9** |
| | | 26/02/2013 | **107.0** |
| 5.93 | 19.08 | 26/02/2013 | **104.8** |

| | | | |
|---|---|---|---|
| 5.85 | 19.17 | 26/02/2013 | **104.6** |
| | | 26/02/2013 | **108.4** |
| | | 26/02/2013 | **101.1** |
| | | 26/02/2013 | **111.6** |
| | | 26/02/2013 | **108.0** |
| | | 26/02/2013 | **101.9** |
| | | 26/02/2013 | **105.0** |
| | | 26/02/2013 | **102.4** |
| | | 26/02/2013 | **111.8** |
| | | 26/02/2013 | **102.4** |
| | | 26/02/2013 | **105.3** |
| | | 26/02/2013 | **107.2** |
| | | 26/02/2013 | **103.3** |
| 4.38 | | 23/05/2013 | **96.4** |
| 4.29 | | 23/05/2013 | **90.2** |
| 2.91 | | 23/05/2013 | **97.0** |
| 2.97 | | 23/05/2013 | **96.4** |
| 3.11 | | 23/05/2013 | **97.0** |
| 2.91 | | 23/05/2013 | **97.5** |
| 3.12 | | 23/05/2013 | **103.2** |
| | | 23/05/2013 | **99.4** |
| | | 23/05/2013 | **98.9** |
| | | 23/05/2013 | **Not detectable** |
| | | 23/05/2013 | **99.0** |
| | | 23/05/2013 | **101.4** |
| | | 23/05/2013 | **97.3** |
| | | 23/05/2013 | **100.9** |
| | | 23/05/2013 | **104.1** |
| | | 23/05/2013 | **99.5** |
| | | 23/05/2013 | **100.1** |
| | | 23/05/2013 | **100.1** |
| | | 23/05/2013 | **Not detectable** |
| | | 23/05/2013 | **102.0** |
| | | 23/05/2013 | **102.3** |
| | | 23/05/2013 | **106.5** |
| | | 23/05/2013 | **101.2** |
| | | 23/05/2013 | **99.4** |
| | | 23/05/2013 | **101.4** |
| | | 23/05/2013 | **97.6** |
| | | 23/05/2013 | **104.2** |
| | | 24/05/2013 | **108.1** |
| | | 24/05/2013 | **107.8** |
| | | 24/05/2013 | **107.9** |
| | | 24/05/2013 | **89.7** |
| | | 24/05/2013 | **90.3** |
| | | 24/05/2013 | **94.0** |
| | | 24/05/2013 | **101.1** |
| | | 24/05/2013 | **96.3** |
| | | 24/05/2013 | **97.1** |
| | | 24/05/2013 | **93.0** |

| | | |
|---|---|---|
| | 24/05/2013 | **100.0** |
| | 24/05/2013 | **Not detectable** |
| | 24/05/2013 | **100.9** |
| | 24/05/2013 | **99.6** |
| | 24/05/2013 | **99.1** |
| | 24/05/2013 | **101.6** |
| | 24/05/2013 | **98.6** |
| | 24/05/2013 | **98.6** |
| | 24/05/2013 | **99.3** |
| | 24/05/2013 | **99.0** |
| | 24/05/2013 | **99.5** |
| | 24/05/2013 | **103.5** |
| | 24/05/2013 | **98.0** |
| | 24/05/2013 | **99.3** |
| | 24/05/2013 | **103.6** |
| | 24/05/2013 | **104.1** |
| | 24/05/2013 | **96.3** |
| | 24/05/2013 | **99.9** |
| | 24/05/2013 | **100.4** |
| | 24/05/2013 | **102.8** |
| | 24/05/2013 | **101.7** |
| | 24/05/2013 | **104.3** |
| | 24/05/2013 | **103.2** |
| | 24/05/2013 | **101.6** |
| | 24/05/2013 | **100.7** |
| | 24/05/2013 | **101.5** |
| | 24/05/2013 | **101.8** |
| | 24/05/2013 | **100.3** |
| | 24/05/2013 | **96.5** |
| | 24/05/2013 | **100.7** |
| | 24/05/2013 | **102.9** |
| | 24/05/2013 | **99.3** |
| | 24/05/2013 | **84.6** |
| | 24/05/2013 | **103.8** |
| | 24/05/2013 | **101.8** |
| | 24/05/2013 | **102.8** |
| | 24/05/2013 | **85.7** |
| | 24/05/2013 | **101.0** |
| | 24/05/2013 | **101.1** |
| | 24/05/2013 | **98.8** |
| | 24/05/2013 | **99.6** |
| | 24/05/2013 | **102.0** |
| 4.76 | 24/05/2013 | **80.0** |
| 4.45 | 24/05/2013 | **95.2** |
| 4.87 | 24/05/2013 | **98.0** |
| 4.51 | 24/05/2013 | **97.1** |
| 4.68 | 24/05/2013 | **94.5** |
| 4.42 | 24/05/2013 | **100.1** |
| 4.34 | 24/05/2013 | **98.8** |
| 4.33 | 24/05/2013 | **98.2** |

| | | |
|---|---|---|
| 4.24 | 24/05/2013 | **98.5** |
| 4.38 | 24/05/2013 | **100.2** |
| 3.92 | 24/05/2013 | **102.2** |
| 4.03 | 24/05/2013 | **101.2** |
| 4.18 | 24/05/2013 | **90.5** |
| 2.77 | 03/06/2013 | **99.3** |
| 2.79 | 03/06/2013 | **100.6** |
| 3.42 | 03/06/2013 | **100.1** |
| 3.53 | 03/06/2013 | **100.1** |
| 3.51 | 03/06/2013 | **103.6** |
| 3.47 | 03/06/2013 | **101.1** |
| 3.55 | 03/06/2013 | **105.7** |
| | 03/06/2013 | **99.3** |
| | 03/06/2013 | **102.0** |
| | 03/06/2013 | **107.5** |
| | 03/06/2013 | **100.3** |
| | 03/06/2013 | **101.1** |
| | 03/06/2013 | **103.0** |
| | 03/06/2013 | **101.6** |
| | 03/06/2013 | **103.7** |
| | 03/06/2013 | **104.8** |
| | 03/06/2013 | **105.3** |
| | 03/06/2013 | **103.5** |
| | 03/06/2013 | **105.4** |
| | 03/06/2013 | **104.6** |
| | 03/06/2013 | **102.0** |
| | 03/06/2013 | **105.8** |
| | 03/06/2013 | **106.6** |
| | 03/06/2013 | **106.2** |
| | 03/06/2013 | **106.4** |
| | 03/06/2013 | **106.6** |
| 4.41 | 29/05/2013 | **94.8** |
| 4.48 | 29/05/2013 | **92.3** |
| 3.38 | 29/05/2013 | **101.0** |
| 3.35 | 29/05/2013 | **97.7** |
| 3.42 | 29/05/2013 | **97.9** |
| 3.37 | 29/05/2013 | **104.0** |
| 3.38 | 29/05/2013 | **99.1** |
| | 29/05/2013 | **109.1** |
| | 29/05/2013 | **101.5** |
| | 29/05/2013 | **103.8** |
| | 29/05/2013 | **102.0** |
| | 29/05/2013 | **107.4** |
| | 29/05/2013 | **108.0** |
| | 29/05/2013 | **108.0** |
| | 29/05/2013 | **98.0** |
| | 29/05/2013 | **104.7** |
| 3.47 | 29/05/2013 | **105.3** |
| 3.36 | 29/05/2013 | **106.0** |
| 3.26 | 29/05/2013 | **105.9** |

| | | |
|---|---|---|
| 3.42 | 29/05/2013 | **107.3** |
| 3.38 | 29/05/2013 | **107.7** |
| 3.40 | 29/05/2013 | **107.8** |
| 3.36 | 29/05/2013 | **105.1** |
| | 29/05/2013 | **107.1** |
| | 29/05/2013 | **105.5** |
| | 29/05/2013 | **106.4** |
| | 29/05/2013 | **111.4** |
| | 29/05/2013 | **111.3** |
| | 29/05/2013 | **109.6** |
| | 29/05/2013 | **109.0** |
| 3.66 | 04/06/2013 | **70.4** |
| 4.80 | 04/06/2013 | **76.8** |
| 4.88 | 04/06/2013 | **72.0** |
| 3.38 | 29/05/2013 | **105.7** |
| 2.77 | 29/05/2013 | **105.0** |
| 2.87 | 29/05/2013 | **108.4** |
| 2.91 | 29/05/2013 | **107.6** |
| 4.33 | 29/05/2013 | **108.1** |
| 4.20 | 29/05/2013 | **103.3** |
| 4.32 | 29/05/2013 | **106.4** |
| 4.40 | 29/05/2013 | **111.4** |
| 4.36 | 29/05/2013 | **107.4** |
| 4.19 | 03/06/2013 | **103.7** |
| 4.24 | 03/06/2013 | **103.1** |
| 4.28 | 03/06/2013 | **103.4** |
| 4.28 | 03/06/2013 | **105.3** |
| 4.26 | 03/06/2013 | **105.0** |
| | 03/06/2013 | **106.1** |
| | 03/06/2013 | **100.5** |
| | 03/06/2013 | **104.8** |
| | 03/06/2013 | **105.7** |
| | 03/06/2013 | **107.4** |
| | 03/06/2013 | **102.0** |
| | 03/06/2013 | **104.6** |
| | 03/06/2013 | **104.9** |
| | 03/06/2013 | **104.1** |
| | 03/06/2013 | **100.9** |
| | 03/06/2013 | **104.1** |
| | 03/06/2013 | **103.0** |
| | 03/06/2013 | **105.2** |
| 4.25 | 04/06/2013 | **105.0** |
| 4.26 | 04/06/2013 | **104.3** |
| 4.23 | 04/06/2013 | **101.5** |
| 4.22 | 04/06/2013 | **102.9** |
| 4.28 | 04/06/2013 | **101.4** |
| | 04/06/2013 | **103.1** |
| | 04/06/2013 | **100.8** |
| | 04/06/2013 | **104.3** |
| | 04/06/2013 | **104.1** |

| | | |
|---|---|---|
| | 04/06/2013 | **102.8** |
| | 04/06/2013 | **104.2** |
| | 04/06/2013 | **99.5** |
| | 04/06/2013 | **105.9** |
| | 04/06/2013 | **103.2** |
| | 04/06/2013 | **104.4** |
| | 04/06/2013 | **105.5** |
| | 04/06/2013 | **106.1** |
| | 04/06/2013 | **108.3** |
| | 04/06/2013 | **103.3** |
| | 04/06/2013 | **105.8** |
| | 04/06/2013 | **106.2** |
| | 04/06/2013 | **104.6** |
| | 04/06/2013 | **105.7** |
| | 04/06/2013 | **103.3** |
| | 04/06/2013 | **103.9** |
| | 04/06/2013 | **104.6** |
| | 04/06/2013 | **107.0** |
| | 04/06/2013 | **104.7** |
| | 04/06/2013 | **107.0** |
| | 04/06/2013 | **105.7** |
| | 04/06/2013 | **107.1** |
| | 04/06/2013 | **107.9** |
| | 04/06/2013 | **105.5** |
| | 04/06/2013 | **103.3** |
| | 04/06/2013 | **105.5** |
| | 04/06/2013 | **104.4** |
| | 04/06/2013 | **102.0** |
| | 04/06/2013 | **102.9** |
| | 04/06/2013 | **105.6** |
| | 04/06/2013 | **106.5** |
| | 04/06/2013 | **99.4** |
| | 04/06/2013 | **106.2** |
| | 04/06/2013 | **107.1** |
| | 04/06/2013 | **103.4** |
| | 04/06/2013 | **105.8** |
| | 04/06/2013 | **103.8** |
| | 04/06/2013 | **100.7** |
| | 04/06/2013 | **100.7** |
| 4.17 | 29/05/2013 | **102.9** |
| 4.23 | 29/05/2013 | **103.3** |
| 4.20 | 29/05/2013 | **107.0** |
| 4.10 | 29/05/2013 | **105.5** |
| 4.29 | 29/05/2013 | **108.1** |
| 4.44 | 29/05/2013 | **92.2** |
| 4.07 | 29/05/2013 | **101.3** |
| 4.34 | 30/05/2013 | **102.2** |
| 4.55 | 30/05/2013 | **102.2** |
| 4.41 | 30/05/2013 | **92.5** |
| 4.52 | 30/05/2013 | **102.3** |

| | | |
|---|---|---|
| 4.61 | 30/05/2013 | **100.3** |
| | 30/05/2013 | **102.1** |
| | 30/05/2013 | **93.8** |
| | 30/05/2013 | **102.2** |
| | 30/05/2013 | **100.5** |
| | 30/05/2013 | **97.2** |
| | 30/05/2013 | **98.9** |
| | 30/05/2013 | **96.0** |
| | 30/05/2013 | **100.6** |
| | 30/05/2013 | **101.1** |
| | 30/05/2013 | **100.7** |
| | 30/05/2013 | **101.8** |
| | 30/05/2013 | **104.0** |
| | 30/05/2013 | **104.1** |
| | 30/05/2013 | **102.1** |
| | 30/05/2013 | **100.0** |
| | 30/05/2013 | **102.6** |
| | 30/05/2013 | **100.9** |
| | 30/05/2013 | **103.2** |
| | 30/05/2013 | **93.9** |
| | 30/05/2013 | **98.6** |
| 4.52 | 30/05/2013 | **100.1** |
| 4.38 | 30/05/2013 | **96.7** |
| 4.45 | 30/05/2013 | **101.7** |
| 4.47 | 30/05/2013 | **101.9** |
| 4.51 | 30/05/2013 | **102.2** |
| | 30/05/2013 | **104.8** |
| | 30/05/2013 | **103.7** |
| | 30/05/2013 | **102.9** |
| | 30/05/2013 | **102.6** |
| | 30/05/2013 | **101.4** |
| | 30/05/2013 | **102.0** |
| | 30/05/2013 | **101.7** |
| | 30/05/2013 | **102.9** |
| | 30/05/2013 | **101.1** |
| | 30/05/2013 | **102.9** |
| | 30/05/2013 | **96.5** |
| | 30/05/2013 | **100.6** |
| | 30/05/2013 | **104.2** |
| | 30/05/2013 | **101.9** |
| | 30/05/2013 | **106.1** |
| | 30/05/2013 | **104.6** |
| | 30/05/2013 | **103.9** |
| 3.95 | 05/06/2013 | **100.0** |
| 4.34 | 05/06/2013 | **86.7** |
| 3.93 | 05/06/2013 | **102.0** |
| 4.07 | 05/06/2013 | **107.4** |
| | 05/06/2013 | **81.5** |
| 7.15 | 05/06/2013 | **105.3** |
| 7.17 | 05/06/2013 | **104.5** |

| | | |
|---|---|---|
| 7.15 | 05/06/2013 | **108.7** |
| | 05/06/2013 | **67.9** |
| | 05/06/2013 | **85.6** |
| | 05/06/2013 | **75.8** |
| | 05/06/2013 | **77.2** |
| | 05/06/2013 | **77.1** |
| | 05/06/2013 | **82.3** |
| | 05/06/2013 | **87.6** |
| | 05/06/2013 | **81.3** |
| | 05/06/2013 | **77.1** |
| | 05/06/2013 | **84.2** |
| | 05/06/2013 | **83.1** |
| | 05/06/2013 | **82.3** |
| | 05/06/2013 | **79.2** |
| | 05/06/2013 | **113.1** |
| | 05/06/2013 | **84.4** |
| | 05/06/2013 | **92.6** |
| | 05/06/2013 | **113.9** |
| | 05/06/2013 | **111.9** |
| | 05/06/2013 | **66.6** |
| | 05/06/2013 | **82.8** |
| | 05/06/2013 | **71.6** |
| | 05/06/2013 | **112.5** |
| | 05/06/2013 | **77.4** |
| | 05/06/2013 | **89.0** |
| | 05/06/2013 | **113.2** |
| | 05/06/2013 | **86.4** |
| | 05/06/2013 | **106.0** |
| | 05/06/2013 | **82.1** |
| | 05/06/2013 | **107.5** |
| | 05/06/2013 | **74.3** |
| | 05/06/2013 | **87.0** |
| | 05/06/2013 | **108.5** |
| | 05/06/2013 | **81.6** |
| | 05/06/2013 | **84.8** |
| | 05/06/2013 | **112.8** |
| 5.56 | 18/06/2013 | **95.4** |
| 5.59 | 18/06/2013 | **104.3** |
| 5.57 | 18/06/2013 | **105.5** |
| 5.58 | 18/06/2013 | **102.0** |
| 5.58 | 18/06/2013 | **102.9** |
| | 18/06/2013 | **104.0** |
| | 18/06/2013 | **106.5** |
| | 18/06/2013 | **109.1** |
| | 18/06/2013 | **106.8** |
| 8.12 | 18/06/2013 | **102.3** |
| 8.13 | 18/06/2013 | **98.2** |
| 8.13 | 18/06/2013 | **95.1** |
| 7.17 | 18/06/2013 | **102.1** |
| 7.16 | 18/06/2013 | **110.2** |

| | | |
|---|---|---|
| 7.17 | 18/06/2013 | **104.5** |
| 7.18 | 18/06/2013 | **104.8** |
| 7.19 | 18/06/2013 | **106.0** |
| | 18/06/2013 | **106.9** |
| | 18/06/2013 | **107.2** |
| | 18/06/2013 | **110.5** |
| | 18/06/2013 | **109.9** |
| | 18/06/2013 | **106.8** |
| | 18/06/2013 | **109.7** |
| | 18/06/2013 | **108.8** |
| | 18/06/2013 | **106.8** |
| | 18/06/2013 | **107.5** |
| | 18/06/2013 | **109.3** |
| | 18/06/2013 | **111.1** |
| | 18/06/2013 | **108.3** |
| | 18/06/2013 | **110.5** |
| | 18/06/2013 | **109.5** |
| | 18/06/2013 | **109.5** |
| | 18/06/2013 | **108.6** |
| | 18/06/2013 | **109.8** |
| | 18/06/2013 | **109.1** |
| | 18/06/2013 | **109.3** |
| | 18/06/2013 | **109.8** |
| | 18/06/2013 | **106.8** |
| | 18/06/2013 | **109.1** |
| | 18/06/2013 | **110.5** |
| | 18/06/2013 | **109.4** |
| | 18/06/2013 | **111.4** |
| | 18/06/2013 | **111.5** |
| | 18/06/2013 | **111.2** |
| | 18/06/2013 | **105.9** |
| | 18/06/2013 | **109.3** |
| 4.98 | 18/06/2013 | **102.8** |
| 2.93 | 18/06/2013 | **112.2** |
| 3.43 | 18/06/2013 | **99.6** |
| 3.79 | 18/06/2013 | **103.5** |
| 3.69 | 18/06/2013 | **102.8** |
| 3.77 | 18/06/2013 | **105.1** |
| 3.86 | 18/06/2013 | **100.5** |
| 3.88 | 18/06/2013 | **102.6** |
| 7.11 | 18/06/2013 | **111.2** |
| 7.14 | 18/06/2013 | **113.0** |
| 4.44 | 18/06/2013 | **98.8** |
| | 18/06/2013 | **54.1** |
| 4.29 | 25/06/2013 | **93.5** |
| 4.18 | 25/06/2013 | **99.2** |
| 4.25 | 25/06/2013 | **96.4** |
| 4.15 | 25/06/2013 | **92.0** |
| 4.17 | 25/06/2013 | **102.2** |
| | 25/06/2013 | **91.7** |

| | | |
|---|---|---|
| | 25/06/2013 | **88.9** |
| | 25/06/2013 | **90.8** |
| | 25/06/2013 | **91.9** |
| | 27/03/2013 | **106.2** |
| 10.46 | 25/06/2013 | **97.1** |
| 10.46 | 25/06/2013 | **86.5** |
| 10.42 | 25/06/2013 | **85.7** |
| 10.42 | 25/06/2013 | **99.0** |
| 10.44 | 25/06/2013 | **93.6** |
| | 25/06/2013 | **95.0** |
| | 25/06/2013 | **96.3** |
| | 25/06/2013 | **100.2** |
| | 25/06/2013 | **92.6** |
| | 25/06/2013 | **92.6** |
| | 25/06/2013 | **89.1** |
| | 25/06/2013 | **97.5** |
| | 25/06/2013 | **98.7** |
| | 25/06/2013 | **99.8** |
| | 25/06/2013 | **95.7** |
| | 25/06/2013 | **92.1** |
| | 25/06/2013 | **90.5** |
| | 25/06/2013 | **93.0** |
| | 25/06/2013 | **88.8** |
| 3.76 | 09/04/2013 | **77.8** |
| 4.01 | 09/04/2013 | **80.8** |
| 3.88 | 09/04/2013 | **76.2** |
| | 09/04/2013 | **76.7** |
| | 09/04/2013 | **96.6** |
| | 09/04/2013 | **85.2** |
| | 09/04/2013 | **88.9** |
| | 09/04/2013 | **75.7** |
| | 09/04/2013 | **86.2** |
| | 09/04/2013 | **80.7** |
| 3.80 | 09/04/2013 | **110.6** |
| 3.77 | 09/04/2013 | **78.0** |
| 3.83 | 09/04/2013 | **105.6** |
| 4.03 | 09/04/2013 | **88.0** |
| 4.08 | 09/04/2013 | **102.7** |
| 4.02 | 09/04/2013 | **102.0** |
| 3.55 | 26/03/2013 | **90.8** |
| 3.56 | 26/03/2013 | **92.0** |
| 3.45 | 26/03/2013 | **91.5** |
| 3.68 | 26/03/2013 | **92.3** |
| 3.51 | 26/03/2013 | **99.2** |
| 3.47 | 26/03/2013 | **98.8** |
| 4.00 | 26/03/2013 | **Not detectable** |
| 3.56 | 26/03/2013 | **88.7** |
| 3.58 | 26/03/2013 | **91.2** |
| 3.51 | 26/03/2013 | **97.6** |
| | 26/03/2013 | **96.8** |

| | |
|---|---|
| 26/03/2013 | **91.1** |
| 26/03/2013 | **94.8** |
| 26/03/2013 | **93.4** |
| 26/03/2013 | **96.5** |
| 26/03/2013 | **99.1** |
| 26/03/2013 | **94.6** |
| 26/03/2013 | **104.8** |
| 26/03/2013 | **105.5** |
| 26/03/2013 | **94.7** |
| 26/03/2013 | **88.6** |
| 26/03/2013 | **98.9** |
| 26/03/2013 | **99.1** |
| 26/03/2013 | **99.0** |
| 26/03/2013 | **99.9** |
| 26/03/2013 | **101.1** |
| 26/03/2013 | **99.2** |
| 26/03/2013 | **98.7** |
| 26/03/2013 | **97.6** |
| 26/03/2013 | **95.7** |
| 26/03/2013 | **98.8** |
| 26/03/2013 | **97.6** |
| 26/03/2013 | **105.8** |
| 26/03/2013 | **96.1** |
| 26/03/2013 | **103.3** |
| 26/03/2013 | **96.5** |
| 26/03/2013 | **93.5** |
| 26/03/2013 | **93.8** |
| 26/03/2013 | **92.4** |
| 26/03/2013 | **98.2** |
| 26/03/2013 | **101.4** |
| 26/03/2013 | **90.1** |
| 26/03/2013 | **Not detectable** |
| 26/03/2013 | **94.4** |
| 26/03/2013 | **98.7** |
| 26/03/2013 | **96.4** |
| 26/03/2013 | **97.6** |
| 26/03/2013 | **101.5** |
| 26/03/2013 | **Not detectable** |
| 26/03/2013 | **94.0** |
| 26/03/2013 | **97.2** |
| 26/03/2013 | **92.4** |
| 26/03/2013 | **99.9** |
| 26/03/2013 | **100.9** |
| 26/03/2013 | **108.2** |
| 26/03/2013 | **95.8** |
| 26/03/2013 | **100.9** |
| 26/03/2013 | **Not detectable** |
| 26/03/2013 | **101.5** |
| 26/03/2013 | **94.4** |
| 26/03/2013 | **101.0** |

| | |
|---|---|
| 26/03/2013 | **92.4** |
| 26/03/2013 | **104.2** |
| 26/03/2013 | **102.0** |
| 26/03/2013 | **107.4** |
| 26/03/2013 | **103.0** |
| 26/03/2013 | Not detectable |
| 26/03/2013 | **101.0** |
| 26/03/2013 | **100.2** |
| 26/03/2013 | **103.1** |
| 26/03/2013 | **102.7** |
| 26/03/2013 | **112.2** |
| 27/03/2013 | Not detectable |
| 27/03/2013 | **99.7** |
| 27/03/2013 | **98.5** |
| 27/03/2013 | **91.6** |
| 27/03/2013 | **98.6** |
| 27/03/2013 | **97.8** |
| 27/03/2013 | **91.2** |
| 27/03/2013 | **97.1** |
| 27/03/2013 | **102.9** |
| 27/03/2013 | **100.1** |
| 27/03/2013 | **104.8** |
| 27/03/2013 | **98.9** |
| 27/03/2013 | **91.8** |
| 27/03/2013 | **99.5** |
| 27/03/2013 | **104.4** |
| 27/03/2013 | Not detectable |
| 27/03/2013 | **114.0** |
| 27/03/2013 | **98.1** |
| 27/03/2013 | **104.8** |
| 27/03/2013 | **108.8** |
| 27/03/2013 | **99.8** |
| 27/03/2013 | **101.2** |
| 27/03/2013 | **108.3** |
| 27/03/2013 | **108.6** |
| 27/03/2013 | **103.8** |
| 27/03/2013 | **106.7** |
| 27/03/2013 | **104.8** |
| 03/04/2013 | Not detectable |
| 03/04/2013 | **105.0** |
| 03/04/2013 | **99.8** |
| 03/04/2013 | **98.8** |
| 03/04/2013 | **107.6** |
| 03/04/2013 | **102.6** |
| 03/04/2013 | **106.4** |
| 03/04/2013 | **107.1** |
| 03/04/2013 | **109.5** |
| 03/04/2013 | **112.9** |
| 03/04/2013 | **110.8** |
| 03/04/2013 | **107.2** |

| | | |
|---|---|---|
| | 03/04/2013 | **102.0** |
| | 03/04/2013 | **99.5** |
| | 03/04/2013 | **101.5** |
| | 03/04/2013 | **91.2** |
| | 03/04/2013 | **Not detectable** |
| | 03/04/2013 | **Not detectable** |
| | 03/04/2013 | **Not detectable** |
| | 03/04/2013 | **106.8** |
| | 03/04/2013 | **99.0** |
| | 03/04/2013 | **Not detectable** |
| | 03/04/2013 | **109.6** |
| | 03/04/2013 | **Not detectable** |
| | 03/04/2013 | **96.7** |
| | 03/04/2013 | **114.7** |
| | 03/04/2013 | **104.5** |
| | 03/04/2013 | **97.8** |
| | 03/04/2013 | **91.2** |
| | 03/04/2013 | **98.1** |
| | 03/04/2013 | **102.9** |
| | 03/04/2013 | **100.1** |
| | 03/04/2013 | **104.8** |
| | 03/04/2013 | **106.2** |
| | 03/04/2013 | **96.9** |
| | 03/04/2013 | **90.8** |
| | 03/04/2013 | **99.5** |
| | 03/04/2013 | **104.4** |
| | 03/04/2013 | **100.6** |
| | 03/04/2013 | **114.0** |
| | 03/04/2013 | **98.1** |
| | 03/04/2013 | **104.8** |
| | 03/04/2013 | **108.8** |
| | 03/04/2013 | **99.8** |
| | 03/04/2013 | **101.2** |
| | 03/04/2013 | **108.3** |
| | 03/04/2013 | **108.6** |
| | 03/04/2013 | **103.8** |
| | 03/04/2013 | **106.7** |
| | 03/04/2013 | **104.8** |
| 6.70 | 08/04/2013 | **92.1** |
| 6.73 | 08/04/2013 | **91.2** |
| 6.74 | 08/04/2013 | **86.6** |
| | 08/04/2013 | **88.2** |
| | 08/04/2013 | **91.7** |
| | 08/04/2013 | **85.0** |
| 6.69 | 08/04/2013 | **88.0** |
| 6.69 | 08/04/2013 | **88.9** |
| 6.68 | 08/04/2013 | **91.5** |
| | 08/04/2013 | **98.6** |
| | 08/04/2013 | **91.9** |
| | 08/04/2013 | **92.8** |

| | | |
|---|---|---|
| 8.26 | 08/04/2013 | **97.8** |
| 8.26 | 08/04/2013 | **101.0** |
| 8.21 | 08/04/2013 | **91.7** |
| 8.09 | 08/04/2013 | **89.7** |
| 8.10 | 08/04/2013 | **86.6** |
| 4.96 | 08/04/2013 | **99.8** |
| 4.97 | 08/04/2013 | **98.4** |
| 4.98 | 08/04/2013 | **98.4** |
| | 08/04/2013 | **98.5** |
| | 08/04/2013 | **105.4** |
| 4.49 | 08/04/2013 | **41.1** |
| 4.79 | 08/04/2013 | **Not detectable** |
| 4.89 | 08/04/2013 | **98.3** |
| 4.72 | 08/04/2013 | **92.1** |
| 8.14 | 08/04/2013 | **99.1** |
| 8.11 | 08/04/2013 | **97.3** |
| 7.21 | 08/04/2013 | **100.9** |
| 7.03 | 08/04/2013 | **103.1** |
| 6.98 | 08/04/2013 | **100.4** |
| | 08/04/2013 | **99.6** |
| | 04/04/2013 | **94.2** |
| | 04/04/2013 | **92.5** |
| | 04/04/2013 | **90.4** |
| | 04/04/2013 | **91.1** |
| | 04/04/2013 | **97.1** |
| | 04/04/2013 | **97.1** |
| | 04/04/2013 | **91.2** |
| | 04/04/2013 | **96.7** |
| | 04/04/2013 | **85.3** |
| | 04/04/2013 | **97.5** |
| | 04/04/2013 | **94.6** |
| | 04/04/2013 | **92.3** |
| | 04/04/2013 | **97.6** |
| | 04/04/2013 | **99.3** |
| | 04/04/2013 | **96.0** |
| | 04/04/2013 | **99.7** |
| | 04/04/2013 | **95.5** |
| | 04/04/2013 | **94.5** |
| | 04/04/2013 | **108.6** |
| | 04/04/2013 | **98.7** |
| | 04/04/2013 | **93.4** |
| | 04/04/2013 | **96.0** |
| | 04/04/2013 | **101.4** |
| | 04/04/2013 | **98.7** |
| | 04/04/2013 | **101.4** |
| | 04/04/2013 | **107.1** |
| | 04/04/2013 | **103.5** |
| | 04/04/2013 | **106.1** |
| | 04/04/2013 | **104.9** |
| | 04/04/2013 | **102.4** |

| | | |
|---|---|---|
| | 04/04/2013 | **99.7** |
| | 04/04/2013 | **107.8** |
| | 04/04/2013 | **106.3** |
| | 04/04/2013 | **103.3** |
| | 04/04/2013 | **100.0** |
| | 04/04/2013 | **101.0** |
| | 04/04/2013 | **99.8** |
| | 04/04/2013 | **91.5** |
| | 04/04/2013 | **100.8** |
| | 04/04/2013 | **95.6** |
| | 04/04/2013 | **94.4** |
| | 04/04/2013 | **100.5** |
| | 04/04/2013 | **99.2** |
| | 04/04/2013 | **104.5** |
| | 04/04/2013 | **95.0** |
| | 04/04/2013 | **98.2** |
| | 04/04/2013 | **91.0** |
| | 04/04/2013 | **90.9** |
| | 04/04/2013 | **90.8** |
| | 04/04/2013 | **94.9** |
| | 04/04/2013 | **106.1** |
| | 04/04/2013 | **99.4** |
| | 04/04/2013 | **105.2** |
| | 04/04/2013 | **101.9** |
| | 04/04/2013 | **100.7** |
| | 04/04/2013 | **97.0** |
| | 04/04/2013 | **91.1** |
| | 04/04/2013 | **106.2** |
| | 04/04/2013 | **105.9** |
| | 04/04/2013 | **103.2** |
| | 04/04/2013 | **101.4** |
| | 04/04/2013 | **104.3** |
| | 04/04/2013 | **102.7** |
| | 04/04/2013 | **100.0** |
| | 04/04/2013 | **104.4** |
| | 04/04/2013 | **98.2** |
| | 04/04/2013 | **104.2** |
| | 04/04/2013 | **102.9** |
| | 04/04/2013 | **107.4** |
| | 04/04/2013 | **108.7** |
| | 04/04/2013 | **110.1** |
| | 04/04/2013 | **98.0** |
| | 04/04/2013 | **103.8** |
| | 04/04/2013 | **106.0** |
| | 04/04/2013 | **101.9** |
| 5.03 | 04/04/2013 | **114.8** |
| 5.01 | 04/04/2013 | **103.5** |
| 5.11 | 04/04/2013 | **96.6** |
| 2.24 | 04/04/2013 | **93.4** |
| 5.24 | 04/04/2013 | **98.2** |

| | | | |
|---|---|---|---|
| 8.26 | | 09/04/2013 | **101.3** |
| 8.30 | | 09/04/2013 | **95.6** |
| 8.31 | | 09/04/2013 | **90.1** |
| | | 09/04/2013 | **86** |
| | | 09/04/2013 | **88.3** |
| | | 09/04/2013 | **85.6** |
| 4.72 | | 09/04/2013 | **94.4** |
| 4.68 | | 09/04/2013 | **93.5** |
| 4.64 | | 09/04/2013 | **90.0** |
| | | 09/04/2013 | **93.0** |
| | | 09/04/2013 | **88.8** |
| 8.16 | | 09/04/2013 | **91.1** |
| 8.22 | | 09/04/2013 | **98.0** |
| 8.17 | | 09/04/2013 | **85.0** |
| | | 09/04/2013 | **89.7** |
| | | 09/04/2013 | **85.8** |
| | | 09/04/2013 | **91.8** |
| | | 09/04/2013 | **94.9** |
| | | 09/04/2013 | **86.4** |
| | | 09/04/2013 | **87.8** |
| 8.12 | | 09/04/2013 | **75.6** |
| | | 01/07/2013 | **109.9** |
| | CDC | | **105.3** |
| | | 01/07/2013 | **115.0** |
| | | 01/07/2013 | **109.5** |
| | | 01/07/2013 | **109.2** |
| | | 01/07/2013 | **105.0** |
| | | 01/07/2013 | **106.3** |
| | | 01/07/2013 | **101.4** |
| | CDC | | **112.3** |
| | | 01/07/2013 | **111.5** |
| | | 01/07/2013 | **92.0** |
| | | 01/07/2013 | **92.2** |
| | CDC | | **84.5** |
| | CDC | | **87.5** |
| | | 01/07/2013 | **114.6** |
| | | 01/07/2013 | **89.2** |
| | | 01/07/2013 | **105.9** |
| | | 01/07/2013 | **97.7** |
| | CDC | | **117.0** |
| | CDC | | **102.9** |
| | | 01/07/2013 | **96.7** |
| | CDC | | **111.2** |
| | CDC | | **102.9** |
| | | 01/07/2013 | **98.8** |
| | | 01/07/2013 | **96.0** |
| | | 01/07/2013 | **108.4** |
| | | 01/07/2013 | **114.6** |
| | CDC | | **100.9** |
| | CDC | | **104.9** |

| | | |
|---|---|---|
| | 01/07/2013 | **104.6** |
| | 01/07/2013 | **105.9** |
| CDC | | **112.7** |
| CDC | | **118.6** |
| | 01/07/2013 | **95.7** |
| | 01/07/2013 | **92.2** |
| | 01/07/2013 | **106.4** |
| | 01/07/2013 | **101.1** |
| | 01/07/2013 | **95.1** |
| CDC | | **117.6** |
| CDC | | **98.8** |
| | 01/07/2013 | **93.7** |
| | 01/07/2013 | **101.2** |
| | 01/07/2013 | **95.6** |
| CDC | | **131.0** |
| CDC | | **134.4** |
| | 01/07/2013 | **100.8** |
| | 01/07/2013 | **103.1** |
| | 01/07/2013 | **99.1** |
| | 01/07/2013 | **78.9** |
| | 01/07/2013 | **92.8** |
| | 01/07/2013 | **112.9** |
| | 01/07/2013 | **107.9** |
| | 01/07/2013 | **106.7** |
| | 01/07/2013 | **113.0** |
| | 01/07/2013 | **102.6** |
| | 01/07/2013 | **101.3** |
| | 01/07/2013 | **108.3** |
| | 01/07/2013 | **103.1** |
| | 01/07/2013 | **98.5** |
| | 01/07/2013 | **102.9** |
| | 01/07/2013 | **109.4** |
| | 01/07/2013 | **103.7** |
| | 01/07/2013 | **106.9** |
| | 01/07/2013 | **105.5** |
| | 01/07/2013 | **94.9** |
| | 01/07/2013 | **101.8** |
| | 01/07/2013 | **98.4** |
| | 01/07/2013 | **102.5** |
| | 01/07/2013 | **113.6** |
| CDC | | **118.2** |
| CDC | | **81.5** |
| CDC | | **93.8** |
| CDC | | **88.9** |
| CDC | | **84.4** |
| CDC | | **117.0** |
| CDC | | **93.3** |
| CDC | | **90.6** |
| CDC | | **90.8** |
| CDC | | **90.7** |

| | | | |
|---|---|---|---|
| | CDC | | **75.4** |
| | CDC | | **86.7** |
| | CDC | | **114.6** |
| | CDC | | **106.4** |
| | CDC | | **101.1** |
| | CDC | | **80.0** |
| | CDC | | **87.5** |
| | | 01/07/2013 | **99.8** |
| | | 01/07/2013 | **99.8** |
| | | 01/07/2013 | **103.8** |
| | | 01/07/2013 | **98.5** |
| | CDC | | **85.4** |
| | CDC | | **104.8** |
| 6.60 | | 08/03/2013 | **85.0** |
| 6.42 | | 08/03/2013 | **75.1** |
| | | 08/03/2013 | **85.9** |
| | | 08/03/2013 | **89.7** |
| | | 08/03/2013 | **87.6** |
| 9.02 | | 08/03/2013 | **73.0** |
| 8.59 | | 08/03/2013 | **34.6** |
| 8.70 | | 08/03/2013 | **78.9** |
| | | 08/03/2013 | **82.5** |
| | | 08/03/2013 | **54.0** |
| | | 08/03/2013 | **73.1** |
| | CDC | | **117.6** |
| | | 28/06/2013 | **107.5** |
| | CDC | | **94.9** |
| | | 28/06/2013 | **84.6** |
| | CDC | | **100.2** |
| | | 28/06/2013 | **107.2** |
| | CDC | | **81.9** |
| | | 28/06/2013 | **104.0** |
| | CDC | | **92.2** |
| | | 28/06/2013 | **92.8** |
| | CDC | | **112.8** |
| | | 28/06/2013 | **105.3** |
| | CDC | | **105.5** |
| | | 28/06/2013 | **111.9** |
| | CDC | | **102.0** |
| | | 28/06/2013 | **93.6** |
| | CDC | | **115.4** |
| | | 01/07/2013 | **98.7** |
| | | 16/08/2013 | **209.9** |
| | | 16/08/2013 | **105.6** |
| | CDC | | **60.4** |
| | | 16/08/2013 | **110.4** |
| | | 16/08/2013 | **109.1** |
| | CDC | | **43.8** |
| | | 16/08/2013 | **125.3** |
| | | 16/08/2013 | **112.1** |

| | | | |
|---|---|---|---|
| | CDC | | **30.7** |
| | | 16/08/2013 | **121.3** |
| | CDC | | **36.1** |
| | | 16/08/2013 | **77.9** |
| | | 16/08/2013 | **115.6** |
| | | 16/08/2013 | **110.5** |
| | CDC | | **92.7** |
| | | 16/08/2013 | **105.8** |
| | CDC | | **111.2** |
| | | 16/08/2013 | **57.2** |
| | | 16/08/2013 | **115.6** |
| | CDC | | **81.1** |
| | | 16/08/2013 | **43.5** |
| | CDC | | **73.5** |
| | | 16/08/2013 | **113.0** |
| | CDC | | **69.8** |
| | | 16/08/2013 | **109.0** |
| | CDC | | **71.1** |
| | | 16/08/2013 | **104.7** |
| | CDC | | **74.8** |
| | | 16/08/2013 | **94.5** |
| | CDC | | **95.1** |
| | | 16/08/2013 | **56.0** |
| | CDC | | **52.1** |
| | | 16/08/2013 | **23.0** |
| | CDC | | **82.9** |
| | | 16/08/2013 | **66.9** |
| | CDC | | **87.0** |
| | | 16/08/2013 | **101.2** |
| | CDC | | **74.8** |
| | | 16/08/2013 | **86.0** |
| | CDC | | **101.6** |
| | | 16/08/2013 | **114.4** |
| | CDC | | **99.1** |
| | | 16/08/2013 | **118.8** |
| | | 16/08/2013 | **71.6** |
| 3.56 | | 02/05/2013 | **106.6** |
| 3.01 | | 02/05/2013 | **93.6** |
| 3.05 | | 02/05/2013 | **101.3** |
| 3.16 | | 02/05/2013 | **105.7** |
| 2.97 | | 02/05/2013 | **96.9** |
| 3.04 | | 02/05/2013 | **108.0** |
| | | 02/05/2013 | **104.6** |
| | | 02/05/2013 | **107.4** |
| | | 02/05/2013 | **110.5** |
| | | 02/05/2013 | **110.1** |
| | | 02/05/2013 | **102.8** |
| | | 02/05/2013 | **86.9** |
| | | 02/05/2013 | **105.0** |
| | | 02/05/2013 | **111.3** |

| | | |
|---|---|---|
| | 02/05/2013 | **110.8** |
| | 02/05/2013 | **106.0** |
| | 02/05/2013 | **105.1** |
| | 02/05/2013 | **109.5** |
| | 02/05/2013 | **107.3** |
| | 02/05/2013 | **105.0** |
| | 02/05/2013 | **113.0** |
| | 02/05/2013 | **109.1** |
| | 02/05/2013 | **106.3** |
| | 02/05/2013 | **104.0** |
| | 02/05/2013 | **106.7** |
| | 02/05/2013 | **107.5** |
| | 02/05/2013 | **109.7** |
| | 02/05/2013 | **113.8** |
| | 02/05/2013 | **109.3** |
| | 02/05/2013 | **105.1** |
| | 02/05/2013 | **104.1** |
| | 02/05/2013 | **102.3** |
| | 02/05/2013 | **103.6** |
| | 02/05/2013 | **108.4** |
| | 02/05/2013 | **111.4** |
| | 02/05/2013 | **106.9** |
| | 02/05/2013 | **107.6** |
| | 02/05/2013 | **103.4** |
| | 02/05/2013 | **103.8** |
| | 02/05/2013 | **103.8** |
| | 02/05/2013 | **105.6** |
| | 02/05/2013 | **104.6** |
| | 02/05/2013 | **86.4** |
| | 02/05/2013 | **105.7** |
| | 02/05/2013 | **111.3** |
| | 02/05/2013 | **106.0** |
| | 02/05/2013 | **106.7** |
| | 02/05/2013 | **101.4** |
| | 02/05/2013 | **103.6** |
| | 02/05/2013 | **106.9** |
| | 02/05/2013 | **109.3** |
| 3.68 | 02/05/2013 | **90.1** |
| 3.63 | 02/05/2013 | **106.5** |
| 3.66 | 02/05/2013 | **106.9** |
| 3.69 | 02/05/2013 | **65.9** |
| 3.64 | 02/05/2013 | **51.7** |
| | 02/05/2013 | **98.2** |
| | 02/05/2013 | **78.4** |
| | 02/05/2013 | **105.2** |
| | 02/05/2013 | **104.3** |
| | 02/05/2013 | **94.0** |
| 4.62 | 02/05/2013 | **0.6** |
| 4.50 | 02/05/2013 | **0.1** |
| 4.58 | 02/05/2013 | **1.1** |

| | | | |
|---|---|---|---|
| 4.61 | | 02/05/2013 | **0.7** |
| 3.53 | | 02/05/2013 | **103.4** |
| 3.62 | | 02/05/2013 | **108.9** |
| 3.52 | | 02/05/2013 | **102.0** |
| 3.42 | | 02/05/2013 | **106.3** |
| 3.47 | | 02/05/2013 | **114.2** |
| 3.43 | | 02/05/2013 | **106.3** |
| 2.98 | | 02/05/2013 | **112.0** |
| 3.17 | | 02/05/2013 | **108.0** |
| 3.07 | | 02/05/2013 | **94.6** |
| 3.08 | | 03/05/2013 | **101.8** |
| 3.24 | | 03/05/2013 | **106.5** |
| 3.18 | | 03/05/2013 | **107.5** |
| 3.16 | | 03/05/2013 | **105.0** |
| 3.21 | | 03/05/2013 | **107.2** |
| | | 03/05/2013 | **107.8** |
| | | 03/05/2013 | **105.9** |
| | | 03/05/2013 | **105.2** |
| | | 03/05/2013 | **104.9** |
| | | 03/05/2013 | **107.0** |
| | | 03/05/2013 | **104.7** |
| | | 03/05/2013 | **107.7** |
| | | 03/05/2013 | **113.5** |
| | | 03/05/2013 | **97.9** |
| | | 03/05/2013 | **108.7** |
| | | 03/05/2013 | **104.2** |
| | | 03/05/2013 | **105.8** |
| | | 03/05/2013 | **107.0** |
| | | 03/05/2013 | **110.4** |
| | | 03/05/2013 | **110.3** |
| | | 03/05/2013 | **104.0** |
| | | 03/05/2013 | **110.8** |
| | | 03/05/2013 | **104.3** |
| | | 03/05/2013 | **107.8** |
| | | 03/05/2013 | **111.0** |
| | | 03/05/2013 | **100.6** |
| | | 03/05/2013 | **102.2** |
| | | 03/05/2013 | **109.8** |
| | | 03/05/2013 | **107.8** |
| | | 03/05/2013 | **109.9** |
| | | 03/05/2013 | **107.3** |
| | | 03/05/2013 | **105.2** |
| | | 03/05/2013 | **112.2** |
| | | 03/05/2013 | **109.4** |
| | | 03/05/2013 | **112.2** |
| | | 03/05/2013 | **109.9** |
| | | 03/05/2013 | **106.1** |
| | | 03/05/2013 | **109.0** |
| | | 03/05/2013 | **105.2** |
| | | 03/05/2013 | **107.8** |

| | | |
|---|---|---|
| | 03/05/2013 | Not detectable |
| | 03/05/2013 | **113.7** |
| | 03/05/2013 | **106.0** |
| | 03/05/2013 | **105.5** |
| | 03/05/2013 | **111.1** |
| | 03/05/2013 | **108.0** |
| | 03/05/2013 | **102.0** |
| 3.45 | 03/05/2013 | Not detectable |
| 3.43 | 03/05/2013 | Not detectable |
| | 03/05/2013 | **103.1** |
| | 03/05/2013 | **100.6** |
| 3.12 | 03/05/2013 | **109.8** |
| | 03/05/2013 | **102.5** |
| 3.14 | 03/05/2013 | **108.1** |
| | 03/05/2013 | **111.7** |
| | 03/05/2013 | **108.6** |
| | 03/05/2013 | **108.7** |
| | 03/05/2013 | **107.7** |
| | 03/05/2013 | **104.2** |
| | 03/05/2013 | **103.0** |
| | 03/05/2013 | **107.2** |
| | 03/05/2013 | **112.7** |
| | 03/05/2013 | **111.8** |
| | 03/05/2013 | **106.0** |
| | 03/05/2013 | **107.4** |
| 4.21 | 03/05/2013 | **109.6** |
| 4.25 | 03/05/2013 | **103.7** |
| 4.28 | 03/05/2013 | **106.2** |
| 4.27 | 03/05/2013 | **108.8** |
| 4.21 | 03/05/2013 | **108.9** |
| | 03/05/2013 | **106.2** |
| | 03/05/2013 | **109.7** |
| | 03/05/2013 | **110.5** |
| | 03/05/2013 | **113.2** |
| | 03/05/2013 | **101.2** |
| | 03/05/2013 | **98.7** |
| | 03/05/2013 | **104.8** |
| | 03/05/2013 | **103.5** |
| | 03/05/2013 | **109.7** |
| | 03/05/2013 | **107.4** |
| | 03/05/2013 | **103.8** |
| 3.12 | 07/05/2013 | **95.3** |
| 3.17 | 07/05/2013 | **97.1** |
| 3.13 | 07/05/2013 | **92.3** |
| 3.15 | 07/05/2013 | **94.0** |
| 3.13 | 07/05/2013 | **94.9** |
| 3.13 | 07/05/2013 | **96.6** |
| 3.13 | 07/05/2013 | **92.6** |
| 3.19 | 07/05/2013 | **94.5** |
| 3.23 | 07/05/2013 | **85.2** |

| | | |
|---|---|---|
| 3.18 | 07/05/2013 | **98.6** |
| 3.26 | 07/05/2013 | **99.9** |
| 3.20 | 07/05/2013 | **95.2** |
| 3.61 | 07/05/2013 | **111.8** |
| 3.54 | 07/05/2013 | **104.3** |
| 3.56 | 07/05/2013 | **98.9** |
| 3.63 | 07/05/2013 | **96.8** |
| 3.47 | 07/05/2013 | **95.9** |
| 3.50 | 07/05/2013 | **110.9** |
| | 07/05/2013 | **102.9** |
| | 07/05/2013 | **110.3** |
| | 07/05/2013 | **111.6** |
| | 07/05/2013 | **108.6** |
| | 07/05/2013 | **111.2** |
| | 07/05/2013 | **106.1** |
| | 07/05/2013 | **112.5** |
| | 07/05/2013 | **104.9** |
| | 07/05/2013 | **113.3** |
| | 07/05/2013 | **109.9** |
| | 07/05/2013 | **106.7** |
| | 07/05/2013 | **112.7** |
| | 07/05/2013 | **111.6** |
| | 07/05/2013 | **110.1** |
| | 07/05/2013 | **106.8** |
| | 07/05/2013 | **110.7** |
| | 07/05/2013 | **94.9** |
| | 07/05/2013 | **105.6** |
| | 07/05/2013 | **108.0** |
| | 07/05/2013 | **109.8** |
| | 07/05/2013 | **98.6** |
| | 07/05/2013 | **108.0** |
| | 07/05/2013 | **109.7** |
| | 07/05/2013 | **113.5** |
| | 07/05/2013 | **105.0** |
| | 07/05/2013 | **106.6** |
| | 07/05/2013 | **114.0** |
| | 07/05/2013 | **99.2** |
| | 07/05/2013 | **103.6** |
| | 07/05/2013 | **113.3** |
| | 07/05/2013 | **107.7** |
| | 07/05/2013 | **99.6** |
| | 07/05/2013 | **98.5** |
| | 07/05/2013 | **106.5** |
| | 07/05/2013 | **99.5** |
| | 07/05/2013 | **99.7** |
| | 07/05/2013 | **103.5** |
| | 07/05/2013 | **110.6** |
| | 07/05/2013 | **108.7** |
| | 07/05/2013 | **102.1** |
| | 07/05/2013 | **110.5** |

| | | |
|---|---|---|
| | 07/05/2013 | **105.7** |
| | 07/05/2013 | **113.5** |
| | 07/05/2013 | **111.5** |
| | 07/05/2013 | **106.1** |
| | 07/05/2013 | **99.2** |
| | 07/05/2013 | **103.6** |
| | 07/05/2013 | **113.3** |
| | 07/05/2013 | **107.7** |
| | 07/05/2013 | **99.6** |
| | 07/05/2013 | **98.5** |
| | 07/05/2013 | **106.5** |
| | 07/05/2013 | **99.5** |
| | 07/05/2013 | **99.7** |
| | 07/05/2013 | **103.5** |
| | 07/05/2013 | **110.6** |
| | 07/05/2013 | **108.7** |
| | 07/05/2013 | **102.1** |
| | 07/05/2013 | **110.0** |
| | 07/05/2013 | **105.6** |
| | 07/05/2013 | **112.8** |
| | 07/05/2013 | **110.9** |
| | 07/05/2013 | **106.1** |
| | 07/05/2013 | **105.8** |
| | 07/05/2013 | **108.8** |
| 3.83 | 08/05/2013 | **89.7** |
| 3.28 | 08/05/2013 | **98.5** |
| 3.83 | 08/05/2013 | **69.0** |
| 3.50 | 08/05/2013 | **104.0** |
| 3.48 | 08/05/2013 | **102.4** |
| 3.53 | 08/05/2013 | **103.7** |
| 3.47 | 08/05/2013 | **96.7** |
| 3.51 | 08/05/2013 | **104.2** |
| 3.52 | 08/05/2013 | **105.2** |
| 3.49 | 08/05/2013 | **103.2** |
| 4.34 | 09/05/2013 | **109.6** |
| 4.72 | 09/05/2013 | **100.8** |
| 4.79 | 09/05/2013 | **86.3** |
| 5.04 | 09/05/2013 | **108.8** |
| 4.95 | 09/05/2013 | **100.5** |
| 4.99 | 09/05/2013 | **102.3** |
| 5.01 | 09/05/2013 | **106.4** |
| 4.98 | 09/05/2013 | **104.5** |
| | 09/05/2013 | **102.3** |
| | 09/05/2013 | **109.5** |
| | 09/05/2013 | **105.1** |
| | 09/05/2013 | **108.6** |
| | 09/05/2013 | **109.5** |
| | 09/05/2013 | **101.9** |
| | 09/05/2013 | **109.6** |
| | 09/05/2013 | **106.7** |

| | | |
|---|---|---|
| | 09/05/2013 | **114.2** |
| | 09/05/2013 | **101.6** |
| | 09/05/2013 | **104.9** |
| 6.53 | 09/05/2013 | **103.3** |
| 4.11 | 09/05/2013 | **107.3** |
| 3.56 | 09/05/2013 | **107.7** |
| 3.68 | 09/05/2013 | **101.7** |
| 4.57 | 09/05/2013 | **92.3** |
| 5.48 | 09/05/2013 | **102.6** |
| 4.71 | 09/05/2013 | **98.1** |
| 4.98 | 10/05/2013 | **106.1** |
| 5.00 | 10/05/2013 | **101.5** |
| 4.93 | 10/05/2013 | **104.4** |
| 4.92 | 10/05/2013 | **104.4** |
| 4.92 | 10/05/2013 | **100.1** |
| | 10/05/2013 | **113.3** |
| | 10/05/2013 | **103.4** |
| | 10/05/2013 | **101.0** |
| | 10/05/2013 | **102.3** |
| | 10/05/2013 | **109.6** |
| | 10/05/2013 | **91.3** |
| | 10/05/2013 | **104.1** |
| | 10/05/2013 | **102.6** |
| | 10/05/2013 | **110.7** |
| | 10/05/2013 | **110.3** |
| | 10/05/2013 | **110.3** |
| | 10/05/2013 | **109.1** |
| | 10/05/2013 | **106.2** |
| | 10/05/2013 | **110.6** |
| 6.06 | 10/05/2013 | **110.0** |
| 6.24 | 10/05/2013 | **106.6** |
| 6.15 | 10/05/2013 | **105.6** |
| 6.04 | 10/05/2013 | **104.2** |
| 6.13 | 10/05/2013 | **109.1** |
| | 10/05/2013 | **108.5** |
| | 10/05/2013 | **110.6** |
| | 10/05/2013 | **108.2** |
| 6.08 | 10/05/2013 | **87.9** |
| 6.07 | 10/05/2013 | **90.7** |
| 6.22 | 10/05/2013 | **97.1** |
| 6.25 | 10/05/2013 | **96.4** |
| 6.37 | 10/05/2013 | **98.5** |
| | 10/05/2013 | **97.8** |
| 7.76 | 10/05/2013 | **97.0** |
| 7.77 | 10/05/2013 | **109.6** |
| 7.81 | 10/05/2013 | **101.6** |
| 7.85 | 10/05/2013 | **99.2** |
| 6.59 | 10/05/2013 | **114.6** |
| 4.73 | 10/05/2013 | **96.7** |
| 6.58 | 10/05/2013 | **99.1** |

| | | |
|---|---|---|
| 6.61 | 10/05/2013 | **110.3** |
| 6.60 | 10/05/2013 | **112.8** |
| 6.33 | 10/05/2013 | **114.0** |
| 6.30 | 10/05/2013 | **98.9** |
| 6.30 | 10/05/2013 | **108.9** |
| 4.20 | 10/05/2013 | **96.0** |
| 4.34 | 10/05/2013 | **111.3** |
| 4.24 | 10/05/2013 | **113.9** |
| 4.48 | 10/05/2013 | **115.0** |
| 4.41 | 10/05/2013 | **110.5** |
| | 10/05/2013 | **109.9** |
| | 10/05/2013 | **100.7** |
| 5.97 | 10/05/2013 | **106.4** |
| 6.04 | 10/05/2013 | **105.8** |
| 6.04 | 10/05/2013 | **106.9** |
| 6.11 | 10/05/2013 | **111.6** |
| 6.01 | 10/05/2013 | **107.9** |
| | 10/05/2013 | **108.2** |
| | 10/05/2013 | **104.8** |
| 6.04 | 10/05/2013 | **105.6** |
| 5.80 | 10/05/2013 | **114.3** |
| 6.44 | 14/05/2013 | **103.1** |
| 5.81 | 14/05/2013 | **100.0** |
| 6.11 | 14/05/2013 | **104.8** |
| 5.94 | 14/05/2013 | **92.1** |
| 6.14 | 14/05/2013 | **108.7** |
| 5.93 | 14/05/2013 | **93.9** |
| 6.10 | 14/05/2013 | **113.6** |
| 6.33 | 14/05/2013 | **114.7** |
| 6.28 | 14/05/2013 | **113.2** |
| 6.23 | 14/05/2013 | **110.9** |
| 6.16 | 14/05/2013 | **114.8** |
| 6.25 | 14/05/2013 | **111.9** |
| 6.23 | 14/05/2013 | **114.6** |
| | 14/05/2013 | **110.1** |
| | 14/05/2013 | **114.3** |
| | 14/05/2013 | **113.5** |
| | 14/05/2013 | **113.4** |
| | 14/05/2013 | **113.4** |
| 9.23 | 14/05/2013 | **96.3** |
| 5.21 | 14/05/2013 | **113.7** |
| 5.22 | 14/05/2013 | **106.6** |
| 6.45 | 14/05/2013 | **102.4** |
| 6.37 | 14/05/2013 | **105.2** |
| 6.70 | 14/05/2013 | **103.4** |
| 6.33 | 14/05/2013 | **103.9** |
| 6.49 | 14/05/2013 | **108.9** |
| | 14/05/2013 | **106.1** |
| | 14/05/2013 | **92.4** |
| | 14/05/2013 | **106.8** |

| | | |
|---|---|---|
| | 14/05/2013 | **109.6** |
| 7.14 | 14/05/2013 | **105.1** |
| 7.17 | 14/05/2013 | **107.8** |
| 7.18 | 14/05/2013 | **114.9** |
| 7.14 | 14/05/2013 | **112.0** |
| 7.17 | 14/05/2013 | **108.0** |
| | 14/05/2013 | **107.7** |
| | 14/05/2013 | **95.4** |
| | 14/05/2013 | **108.7** |
| | 14/05/2013 | **106.4** |
| | 14/05/2013 | **110.7** |
| | 14/05/2013 | **113.3** |
| | 14/05/2013 | **112.8** |
| | 14/05/2013 | **114.0** |
| | 14/05/2013 | **107.3** |
| | 14/05/2013 | **109.8** |
| 6.50 | 15/05/2013 | **109.6** |
| 6.07 | 15/05/2013 | **106.3** |
| 6.28 | 15/05/2013 | **108.2** |
| 6.46 | 15/05/2013 | **110.8** |
| 6.24 | 15/05/2013 | **110.8** |
| | 15/05/2013 | **108.0** |
| | 15/05/2013 | **107.6** |
| | 15/05/2013 | **97.8** |
| | 15/05/2013 | **97.9** |
| | 15/05/2013 | **98.9** |
| | 15/05/2013 | **96.0** |
| | 15/05/2013 | **94.7** |
| | 15/05/2013 | **92.5** |
| | 15/05/2013 | **89.0** |
| | 15/05/2013 | **87.0** |
| | 15/05/2013 | **86.0** |
| | 15/05/2013 | **86.9** |
| | 15/05/2013 | **87.9** |
| | 15/05/2013 | **89.5** |
| | 15/05/2013 | **86.8** |
| | 15/05/2013 | **94.5** |
| | 15/05/2013 | **108.3** |
| | 15/05/2013 | **109.6** |
| | 15/05/2013 | **111.4** |
| | 15/05/2013 | **103.4** |
| | 15/05/2013 | **87.2** |
| | 15/05/2013 | **104.4** |
| | 15/05/2013 | **86.4** |
| | 15/05/2013 | **104.3** |
| | 15/05/2013 | **99.0** |
| | 15/05/2013 | **97.9** |
| | 15/05/2013 | **95.7** |
| | 15/05/2013 | **96.8** |
| | 15/05/2013 | **93.1** |

| | | |
|---|---|---|
| | 15/05/2013 | **95.2** |
| | 15/05/2013 | **101.1** |
| | 15/05/2013 | **103.0** |
| | 15/05/2013 | **95.7** |
| | 15/05/2013 | **100.7** |
| | 15/05/2013 | **104.0** |
| | 15/05/2013 | **99.1** |
| | 15/05/2013 | **96.5** |
| | 15/05/2013 | **92.0** |
| | 15/05/2013 | **92.6** |
| | 15/05/2013 | **97.3** |
| | 15/05/2013 | **94.2** |
| | 15/05/2013 | **87.6** |
| | 15/05/2013 | **105.8** |
| | 15/05/2013 | **90.8** |
| | 15/05/2013 | **90.8** |
| | 15/05/2013 | **94.9** |
| | 15/05/2013 | **94.4** |
| | 15/05/2013 | **97.3** |
| | 15/05/2013 | **97.9** |
| | 15/05/2013 | **97.2** |
| | 15/05/2013 | **99.5** |
| | 15/05/2013 | **99.9** |
| | 15/05/2013 | **95.7** |
| | 15/05/2013 | **102.0** |
| | 15/05/2013 | **98.3** |
| | 15/05/2013 | **90.9** |
| | 15/05/2013 | **96.8** |
| | 15/05/2013 | **98.2** |
| | 15/05/2013 | **94.5** |
| | 15/05/2013 | **107.0** |
| | 15/05/2013 | **104.1** |
| | 15/05/2013 | **99.3** |
| | 15/05/2013 | **99.3** |
| | 15/05/2013 | **100.1** |
| | 15/05/2013 | **98.3** |
| | 15/05/2013 | **106.5** |
| | 15/05/2013 | **96.6** |
| | 15/05/2013 | **94.0** |
| | 15/05/2013 | **93.6** |
| | 15/05/2013 | **97.7** |
| 6.13 | 16/05/2013 | **99.7** |
| 6.03 | 16/05/2013 | **97.7** |
| 5.94 | 16/05/2013 | **101.4** |
| 6.08 | 16/05/2013 | **103.5** |
| 7.62 | 16/05/2013 | **91.8** |
| 7.7 | 16/05/2013 | **101.1** |
| 7.7 | 16/05/2013 | **91.5** |
| 7.67 | 16/05/2013 | **95.8** |
| 7.64 | 16/05/2013 | **95.7** |

| | | |
|---|---|---|
| | 16/05/2013 | **103.9** |
| | 16/05/2013 | **114.3** |
| | 16/05/2013 | **108.6** |
| | 16/05/2013 | **107.3** |
| | 16/05/2013 | **104.6** |
| | 16/05/2013 | **89.5** |
| | 16/05/2013 | **94.9** |
| | 16/05/2013 | **91.1** |
| | 16/05/2013 | **100.2** |
| | 16/05/2013 | **89.0** |
| 5.59 | 16/05/2013 | **88.8** |
| 5.49 | 16/05/2013 | **91.2** |
| 5.55 | 16/05/2013 | **86.5** |
| 5.7 | 16/05/2013 | **93.9** |
| 5.52 | 16/05/2013 | **101.4** |
| 6.13 | 16/05/2013 | **93.5** |
| 6.4 | 16/05/2013 | **90.4** |
| 6.06 | 16/05/2013 | **86.6** |
| 6.21 | 16/05/2013 | **94.0** |
| 6.09 | 16/05/2013 | **87.6** |
| 6.35 | 16/05/2013 | **94.7** |
| | 16/05/2013 | **97.7** |
| | 16/05/2013 | **95.6** |
| | 16/05/2013 | **85.5** |
| | 16/05/2013 | **94.6** |
| | 16/05/2013 | **92.7** |
| 6.21 | 16/05/2013 | **94.3** |
| 6.21 | 16/05/2013 | **103.3** |
| 6.28 | 16/05/2013 | **101.7** |
| 6.29 | 16/05/2013 | **94.9** |
| 6.22 | 16/05/2013 | **97.6** |
| | 16/05/2013 | **101.1** |
| | 16/05/2013 | **96.4** |
| 6.35 | 16/05/2013 | **98.3** |
| 6.61 | 16/05/2013 | **95.9** |
| 6.58 | 16/05/2013 | **102.5** |
| 6.56 | 16/05/2013 | **102.7** |
| 6.32 | 16/05/2013 | **97.3** |
| | 16/05/2013 | **107.8** |
| | 16/05/2013 | **101.5** |
| | 16/05/2013 | **103.0** |
| | 16/05/2013 | **109.0** |
| 8.09 | 16/05/2013 | **102.3** |
| 8.11 | 16/05/2013 | **102.1** |
| 8.13 | 16/05/2013 | **106.6** |
| 8.17 | 16/05/2013 | **107.4** |
| 8.07 | 16/05/2013 | **107.5** |
| | 16/05/2013 | **100.4** |
| 4.63 | 28/05/2013 | **100.5** |
| 4.39 | 28/05/2013 | **94.4** |

| | | |
|---|---|---|
| 2.91 | 28/05/2013 | **99.9** |
| 2.93 | 28/05/2013 | **101.6** |
| 2.88 | 28/05/2013 | **100.8** |
| 2.94 | 28/05/2013 | **100.3** |
| 2.99 | 28/05/2013 | **101.3** |
| | 28/05/2013 | **98.1** |
| | 28/05/2013 | **98.8** |
| | 28/05/2013 | **100.2** |
| | 28/05/2013 | **100.6** |
| | 28/05/2013 | **99.7** |
| | 28/05/2013 | **98.0** |
| | 28/05/2013 | **103.4** |
| | 28/05/2013 | **99.9** |
| | 28/05/2013 | **102.0** |
| | 28/05/2013 | **101.2** |
| | 28/05/2013 | **98.8** |
| | 28/05/2013 | **100.5** |
| | 28/05/2013 | **100.8** |
| | 28/05/2013 | **98.6** |
| | 28/05/2013 | **96.2** |
| | 28/05/2013 | **98.3** |
| | 28/05/2013 | **99.6** |
| | 28/05/2013 | **104.3** |
| | 28/05/2013 | **100.9** |
| | 28/05/2013 | **100.8** |
| | 28/05/2013 | **102.5** |
| | 28/05/2013 | **98.1** |
| | 28/05/2013 | **100.2** |
| | 28/05/2013 | **99.7** |
| | 28/05/2013 | **100.9** |
| | 28/05/2013 | **99.9** |
| | 28/05/2013 | **99.7** |
| | 28/05/2013 | **99.4** |
| | 28/05/2013 | **101.7** |
| | 28/05/2013 | **99.1** |
| 4.68 | 28/05/2013 | **91.3** |
| 4.45 | 28/05/2013 | **93.7** |
| 3.85 | 28/05/2013 | **96.4** |
| 4.02 | 28/05/2013 | **102.4** |
| 3.50 | 06/06/2013 | **107.3** |
| 3.55 | 06/06/2013 | **105.9** |
| 3.53 | 06/06/2013 | **102.6** |
| | 06/06/2013 | **92.4** |
| | 06/06/2013 | **98.3** |
| | 06/06/2013 | **103.4** |
| | 06/06/2013 | **109.6** |
| | 06/06/2013 | **101.4** |
| | 06/06/2013 | **103.1** |
| | 06/06/2013 | **110.1** |
| | 06/06/2013 | **106.0** |

| | | |
|---|---|---|
| 3.72 | 06/06/2013 | **96.6** |
| | 06/06/2013 | **111.0** |
| | 06/06/2013 | **110.7** |
| | 06/06/2013 | **107.7** |
| 4.27 | 06/06/2013 | **107.3** |
| 4.27 | 06/06/2013 | **106.6** |
| 4.27 | 06/06/2013 | **103.9** |
| 4.24 | 06/06/2013 | **107.5** |
| 4.29 | 06/06/2013 | **106.4** |
| | 06/06/2013 | **103.5** |
| | 06/06/2013 | **103.2** |
| | 06/06/2013 | **107.7** |
| | 06/06/2013 | **104.3** |
| | 06/06/2013 | **107.0** |
| | 06/06/2013 | **109.1** |
| | 06/06/2013 | **107.4** |
| | 06/06/2013 | **88.0** |
| | 06/06/2013 | **107.3** |
| | 06/06/2013 | **103.2** |
| | 06/06/2013 | **108.3** |
| | 06/06/2013 | **103.6** |
| | 06/06/2013 | **104.9** |
| | 06/06/2013 | **106.7** |
| | 06/06/2013 | **112.2** |
| | 06/06/2013 | **105.6** |
| | 06/06/2013 | **99.5** |
| | 06/06/2013 | **106.1** |
| | 06/06/2013 | **111.6** |
| 4.25 | 06/06/2013 | **104.2** |
| 4.28 | 06/06/2013 | **106.0** |
| 4.24 | 06/06/2013 | **106.8** |
| 4.24 | 06/06/2013 | **106.0** |
| 4.16 | 06/06/2013 | **102.1** |
| 4.24 | 06/06/2013 | **102.9** |
| 4.29 | 06/06/2013 | **104.1** |
| 4.38 | 06/06/2013 | **103.6** |
| 4.16 | 06/06/2013 | **100.1** |
| 4.48 | 06/06/2013 | **102.5** |
| 4.13 | 06/06/2013 | **103.2** |
| 4.57 | 06/06/2013 | **100.0** |
| | 06/06/2013 | **101.4** |
| | 06/06/2013 | **99.9** |
| | 06/06/2013 | **101.2** |
| | 06/06/2013 | **102.2** |
| | 06/06/2013 | **101.7** |
| | 06/06/2013 | **99.1** |
| | 06/06/2013 | **102.2** |
| | 06/06/2013 | **90.4** |
| | 06/06/2013 | **103.2** |
| | 06/06/2013 | **102.9** |

| | | |
|---|---|---|
| | 06/06/2013 | **102.3** |
| 4.20 | 06/06/2013 | **105.2** |
| 4.24 | 06/06/2013 | **107.8** |
| 4.20 | 06/06/2013 | **104.7** |
| 4.38 | 06/06/2013 | **101.3** |
| 4.17 | 06/06/2013 | **99.1** |
| 3.98 | 06/06/2013 | **101.1** |
| 4.00 | 06/06/2013 | **104.3** |
| 7.17 | 06/06/2013 | **107.4** |
| 7.19 | 06/06/2013 | **94.3** |
| 7.20 | 06/06/2013 | **98.2** |
| 5.55 | 18/06/2013 | **104.2** |
| 5.56 | 18/06/2013 | **104.7** |
| 5.57 | 18/06/2013 | **104.4** |
| 5.56 | 18/06/2013 | **104.6** |
| 5.55 | 18/06/2013 | **106.9** |
| 7.14 | 18/06/2013 | **105.8** |
| 7.09 | 18/06/2013 | **108.1** |
| 7.15 | 18/06/2013 | **106.6** |
| 7.11 | 18/06/2013 | **106.3** |
| 7.15 | 18/06/2013 | **104.8** |
| | 18/06/2013 | **107.8** |
| | 18/06/2013 | **108.1** |
| | 18/06/2013 | **106.9** |
| | 18/06/2013 | **111.3** |
| | 18/06/2013 | **104.5** |
| | 18/06/2013 | **106.9** |
| | 18/06/2013 | **105.5** |
| | 18/06/2013 | **106.3** |
| | 18/06/2013 | **102.1** |
| | 18/06/2013 | **108.2** |
| | 18/06/2013 | **107.9** |
| | 18/06/2013 | **101.5** |
| | 18/06/2013 | **107.9** |
| | 18/06/2013 | **114.9** |
| | 18/06/2013 | **100.0** |
| 7.11 | 18/06/2013 | **108.7** |
| | 18/06/2013 | **0.1** |
| 3.80 | 18/06/2013 | **99.9** |
| | 26/09/2013 | **97.9** |
| 4.14 | 25/06/2013 | **93.2** |
| 4.16 | 25/06/2013 | **95.2** |
| 4.27 | 25/06/2013 | **90.4** |
| 4.17 | 25/06/2013 | **92.9** |
| 4.27 | 25/06/2013 | **93.2** |
| | 25/06/2013 | **90.4** |
| 10.43 | 25/06/2013 | **90.3** |
| 10.45 | 25/06/2013 | **92.2** |
| 10.43 | 25/06/2013 | **88.2** |
| 10.43 | 25/06/2013 | **90.4** |

| | | |
|---|---|---|
| 10.58 | 25/06/2013 | **94.4** |
| | 25/06/2013 | **95.3** |
| | 25/06/2013 | **96.9** |
| | 25/06/2013 | **92.3** |
| | 25/06/2013 | **92.3** |
| | 25/06/2013 | **98.1** |
| | 25/06/2013 | **96.5** |
| | 25/06/2013 | **96.8** |
| | 25/06/2013 | **92.8** |
| | 25/06/2013 | **95.2** |
| | 25/06/2013 | **92.8** |
| 3.90 | 25/04/2013 | **93.5** |
| 3.46 | 25/04/2013 | **98.3** |
| 3.55 | 25/04/2013 | **92.6** |
| 3.62 | 25/04/2013 | **100.0** |
| 3.80 | 25/04/2013 | **96.3** |
| 3.57 | 25/04/2013 | **100.8** |
| | 25/04/2013 | **94.6** |
| | 25/04/2013 | **Not detectable** |
| | 25/04/2013 | **94.2** |
| | 25/04/2013 | **99.4** |
| | 25/04/2013 | **105.6** |
| | 25/04/2013 | **99.0** |
| | 25/04/2013 | **97.6** |
| | 25/04/2013 | **91.9** |
| | 25/04/2013 | **97.3** |
| | 25/04/2013 | **100.9** |
| | 25/04/2013 | **98.0** |
| | 25/04/2013 | **105.7** |
| | 25/04/2013 | **99.9** |
| | 25/04/2013 | **96.1** |
| | 25/04/2013 | **103.3** |
| | 25/04/2013 | **93.4** |
| | 25/04/2013 | **Not detectable** |
| | 25/04/2013 | **94.8** |
| | 25/04/2013 | **Not detectable** |
| | 25/04/2013 | **103.3** |
| | 25/04/2013 | **98.7** |
| | 25/04/2013 | **93.9** |
| | 25/04/2013 | **107.9** |
| | 25/04/2013 | **105.0** |
| | 25/04/2013 | **105.7** |
| | 25/04/2013 | **106.8** |
| | 25/04/2013 | **106.1** |
| | 25/04/2013 | **Not detectable** |
| | 25/04/2013 | **111.4** |
| | 25/04/2013 | **112.9** |
| | 25/04/2013 | **102.2** |
| | 25/04/2013 | **104.3** |
| | 25/04/2013 | **105.3** |

| | | |
|---|---|---|
| | 25/04/2013 | **104.0** |
| | 25/04/2013 | **99.0** |
| | 25/04/2013 | **96.3** |
| | 25/04/2013 | **108.1** |
| | 25/04/2013 | **112.5** |
| | 25/04/2013 | **97.0** |
| | 25/04/2013 | **97.5** |
| | 25/04/2013 | **97.2** |
| | 25/04/2013 | **106.0** |
| | 25/04/2013 | **97.1** |
| | 25/04/2013 | **100.9** |
| | 25/04/2013 | **111.4** |
| | 25/04/2013 | **109.9** |
| | 25/04/2013 | **106.0** |
| | 25/04/2013 | **93.6** |
| | 25/04/2013 | **96.6** |
| | 25/04/2013 | **104.7** |
| | 25/04/2013 | **90.3** |
| | 25/04/2013 | **106.7** |
| | 25/04/2013 | **99.5** |
| | 25/04/2013 | **103.9** |
| | 25/04/2013 | **104.3** |
| | 25/04/2013 | **97.1** |
| | 25/04/2013 | **94.7** |
| | 25/04/2013 | **109.4** |
| | 25/04/2013 | **100.0** |
| | 25/04/2013 | **98.5** |
| | 25/04/2013 | **102.9** |
| | 25/04/2013 | **92.5** |
| | 25/04/2013 | **94.8** |
| | 25/04/2013 | **100.6** |
| 6.72 | 01/05/2013 | **98.0** |
| 6.81 | 01/05/2013 | **90.1** |
| 6.70 | 01/05/2013 | **90.7** |
| 6.70 | 01/05/2013 | **93.6** |
| 8.28 | 01/05/2013 | **96.6** |
| 7.22 | 01/05/2013 | **100.8** |
| 8.31 | 01/05/2013 | **96.4** |
| 8.29 | 01/05/2013 | **83.7** |
| 8.31 | 01/05/2013 | **95.3** |
| 8.25 | 01/05/2013 | **85.3** |
| 8.25 | 01/05/2013 | **87.3** |
| 5.23 | 30/04/2013 | **104.3** |
| 5.10 | 30/04/2013 | **93.9** |
| 5.27 | 30/04/2013 | **95.7** |
| 5.06 | 30/04/2013 | **90.4** |
| 5.07 | 30/04/2013 | **95.4** |
| | 30/04/2013 | **101.7** |
| | 30/04/2013 | **104.3** |
| | 30/04/2013 | **90.5** |

| | | | |
|---|---|---|---|
| | | 30/04/2013 | **95.1** |
| | | 30/04/2013 | **90.4** |
| | | 30/04/2013 | **96.1** |
| | | 30/04/2013 | **99.4** |
| | | 30/04/2013 | **98.6** |
| | | 30/04/2013 | **90.3** |
| | | 30/04/2013 | **95.8** |
| | | 30/04/2013 | **100.9** |
| | | 30/04/2013 | **102.1** |
| | | 30/04/2013 | **102.7** |
| | | 30/04/2013 | **100.5** |
| | | 30/04/2013 | **99.6** |
| | | 30/04/2013 | **101.7** |
| | | 30/04/2013 | **103.1** |
| | | 30/04/2013 | **102.3** |
| | | 30/04/2013 | **103.1** |
| | | 30/04/2013 | **104.4** |
| | | 30/04/2013 | **103.8** |
| | | 30/04/2013 | **103.3** |
| | | 30/04/2013 | **104.2** |
| | | 30/04/2013 | **99.2** |
| | | 30/04/2013 | **104.8** |
| | | 30/04/2013 | **109.9** |
| | | 30/04/2013 | **105.2** |
| | | 30/04/2013 | **98.5** |
| | | 30/04/2013 | **103.0** |
| | | 30/04/2013 | **106.3** |
| | | 30/04/2013 | **102.6** |
| | | 30/04/2013 | **106.9** |
| | | 30/04/2013 | **102.0** |
| | | 30/04/2013 | **103.5** |
| | | 30/04/2013 | **109.7** |
| | | 30/04/2013 | **104.7** |
| | | 30/04/2013 | **104.2** |
| | | 30/04/2013 | **107.4** |
| | | 30/04/2013 | **107.9** |
| | | 30/04/2013 | **109.7** |
| | | 30/04/2013 | **102.6** |
| | | 30/04/2013 | **102.2** |
| 4.69 | | 01/05/2013 | **89.5** |
| 8.18 | | 01/05/2013 | **98.1** |
| 8.20 | | 01/05/2013 | **99.9** |
| 8.22 | | 01/05/2013 | **98.3** |
| 8.18 | | 01/05/2013 | **93.5** |
| 6.5 | | 17/05/2013 | **86.8** |
| 8.81 | | 17/05/2013 | **67.0** |
| 8.94 | | 17/05/2013 | **104.2** |
| | | 17/05/2013 | **102.2** |
| | | 01/08/2013 | **80.3** |
| | CDC | | **90.6** |

| | | |
|---|---|---|
| | 01/08/2013 | **79.0** |
| CDC | | **79.5** |
| | 01/08/2013 | **83.2** |
| CDC | | **52.8** |
| | 01/08/2013 | **90.5** |
| | 01/08/2013 | **103.3** |
| CDC | | **80.7** |
| | 01/08/2013 | **100.8** |
| | 01/08/2013 | **93.8** |
| CDC | | **113** |
| | 01/08/2013 | **88.0** |
| | 01/08/2013 | **90.9** |
| | 01/08/2013 | **99.3** |
| CDC | | **116.7** |
| | 01/08/2013 | **97.3** |
| CDC | | **111.8** |
| | 01/08/2013 | **90.2** |
| CDC | | **89** |
| | 01/08/2013 | **92.1** |
| | 01/08/2013 | **97.3** |
| CDC | | **101.4** |
| | 01/08/2013 | **88.9** |
| CDC | | **90.2** |
| | 01/08/2013 | **91.7** |
| CDC | | **94.5** |
| | 01/08/2013 | **101.5** |
| CDC | | **100.3** |
| | 01/08/2013 | **93.5** |
| | 01/08/2013 | **97.6** |
| | 01/08/2013 | **79.7** |
| | 01/08/2013 | **96.2** |
| CDC | | **106.8** |
| | 01/08/2013 | **98.3** |
| CDC | | **104.4** |
| | 01/08/2013 | **52.7** |
| | 01/08/2013 | **75.5** |
| | 01/08/2013 | **80.9** |
| CDC | | **134.7** |
| | 01/08/2013 | **69** |
| CDC | | **122.4** |
| | 01/08/2013 | **67.6** |
| | 01/08/2013 | **95.4** |
| | 01/08/2013 | **98.2** |
| | 02/08/2013 | **89.3** |
| | 02/08/2013 | **92.4** |
| CDC | | **104.5** |
| | 02/08/2013 | **89.4** |
| CDC | | **94.5** |
| | 02/08/2013 | **85.4** |
| CDC | | **97.4** |

| | | |
|---|---|---|
| | 02/08/2013 | **90.3** |
| CDC | | **108.7** |
| | 02/08/2013 | **93.3** |
| | 02/08/2013 | **93.7** |
| | 02/08/2013 | **74.5** |
| CDC | | **95.2** |
| | 02/08/2013 | **98.4** |
| | 02/08/2013 | **106.5** |
| CDC | | **110.6** |
| | 02/08/2013 | **91.8** |
| CDC | | **101.5** |
| | 02/08/2013 | **88.9** |
| CDC | | **95.4** |
| | 02/08/2013 | **89.8** |
| CDC | | **102.0** |
| | 02/08/2013 | **93.8** |
| CDC | | **96.9** |
| | 02/08/2013 | **98.8** |
| CDC | | **111.6** |
| | 02/08/2013 | **99.4** |
| CDC | | **101.6** |
| | 02/08/2013 | **86.1** |
| CDC | | **99.0** |
| | 02/08/2013 | **107.3** |
| CDC | | **109.7** |
| | 02/08/2013 | **92.0** |
| CDC | | **95.7** |
| | 02/08/2013 | **97.6** |
| | 02/08/2013 | **100.7** |
| | 06/06/2013 | **72.0** |
| | 06/06/2013 | **58.1** |
| | 06/06/2013 | **80.1** |
| | 06/06/2013 | **79.9** |
| | 06/06/2013 | **109.2** |
| | 06/06/2013 | **83.8** |
| | 06/06/2013 | **92.7** |
| | 06/06/2013 | **107.6** |
| | 06/06/2013 | **85.4** |
| | 06/06/2013 | **78.5** |
| | 06/06/2013 | **67.3** |
| | 06/06/2013 | **108.6** |
| | 28/06/2013 | **101.5** |
| CDC | | **92.5** |
| | 28/06/2013 | **97.8** |
| CDC | | **105.0** |
| | 28/06/2013 | **98.3** |
| CDC | | **110.0** |
| | 28/06/2013 | **87.6** |
| CDC | | **103.0** |
| | 28/06/2013 | **98.0** |

| | | |
|---|---|---|
| CDC | | **109.5** |
| | 28/06/2013 | **102.9** |
| CDC | | **115.0** |
| | 28/06/2013 | **104.7** |
| CDC | | **99.5** |
| | 28/06/2013 | **101.1** |
| CDC | | **103.5** |
| | 28/06/2013 | **101.7** |
| CDC | | **98.5** |
| | 28/06/2013 | **103.8** |
| | 16/08/2013 | **92.9** |
| | 16/08/2013 | **74.5** |
| | 16/08/2013 | **84.1** |
| CDC | | **65.9** |
| | 16/08/2013 | **82.3** |
| | 16/08/2013 | **60.6** |
| CDC | | **73.9** |
| | 16/08/2013 | **84** |
| CDC | | **64.4** |
| | 16/08/2013 | **92.6** |
| CDC | | **72.1** |
| | 16/08/2013 | **79.8** |
| | 16/08/2013 | **90.8** |
| | 16/08/2013 | **81.2** |
| CDC | | **70.8** |
| | 16/08/2013 | **76** |
| CDC | | **67** |
| | 16/08/2013 | **110.3** |
| | 16/08/2013 | **61.9** |
| CDC | | **67.6** |
| | 16/08/2013 | **93.3** |
| CDC | | **73.9** |
| | 16/08/2013 | **98.5** |
| CDC | | **63.1** |

# u_Nigeria Data base

## venience Sampling Approach

| permlgen2 | genname3 | strengthgen3 | permlgen3 | doseform | manufby2 |
|---|---|---|---|---|---|
| | | | | tablet | XX |
| | | | | tablet | XX |
| | | | | tablet | XX |
| | | | | tablet | XX |
| | | | | tablet | XX |
| | | | | tablet | XX |
| | | | | tablet | XX |
| | | | | tablet | XX |
| | | | | tablet | XX |
| | | | | tablet | XX |
| | | | | tablet | XX |
| | | | | tablet | XX |
| | | | | tablet | XX |
| | | | | tablet | XX |
| | | | | tablet | XX |
| | | | | tablet | XX |
| | | | | tablet | XX |
| | | | | tablet | XX |
| | | | | tablet | XX |
| | | | | tablet | XX |
| | | | | tablet | XX |
| | | | | tablet | XX |
| | | | | tablet | XX |
| | | | | tablet | XX |
| | | | | tablet | XX |
| | | | | tablet | XX |
| | | | | tablet | XX |
| | | | | tablet | XX |
| | | | | tablet | XX |
| | | | | tablet | XX |
| | | | | tablet | XX |
| | | | | tablet | XX |
| | | | | tablet | XX |
| | | | | tablet | XX |
| | | | | tablet | XX |
| | | | | tablet | XX |
| | | | | tablet | XX |
| | | | | tablet | XX |
| | | | | tablet | XX |
| | | | | tablet | XX |
| | | | | tablet | XX |
| | | | | tablet | XX |
| | | | | tablet | XX |
| | | | | tablet | XX |
| | | | | tablet | XX |
| | | | | tablet | XX |

| | |
|---|---|
| tablet | XX |
| tablet | XX |
| tablet | XX |
| tablet | XX |
| tablet | XX |
| tablet | XX |
| tablet | XX |
| tablet | XX |
| tablet | XX |
| tablet | XX |
| tablet | XX |
| powder | XX |
| tablet | XX |
| tablet | XX |
| tablet | XX |
| tablet | XX |
| tablet | XX |
| tablet | XX |
| tablet | XX |
| tablet | XX |
| tablet | XX |
| tablet | XX |
| tablet | XX |
| tablet | XX |
| tablet | XX |
| tablet | XX |
| tablet | XX |
| tablet | XX |
| tablet | XX |
| tablet | XX |
| tablet | XX |
| tablet | XX |
| tablet | XX |
| tablet | XX |
| tablet | XX |
| tablet | XX |
| tablet | XX |
| tablet | XX |
| tablet | XX |
| tablet | XX |
| tablet | XX |
| tablet | XX |
| tablet | XX |
| tablet | XX |
| tablet | XX |
| tablet | XX |
| tablet | XX |
| tablet | XX |
| tablet | XX |
| tablet | XX |

| | | | | |
|---|---|---|---|---|
| | | | tablet | XX |
| | | | tablet | XX |
| | | | tablet | XX |
| | | | tablet | XX |
| | | | tablet | XX |
| | | | tablet | XX |
| | | | tablet | XX |
| | | | tablet | XX |
| | | | tablet | XX |
| | | | tablet | XX |
| | | | tablet | XX |
| | | | tablet | XX |
| | | | tablet | XX |
| | | | tablet | XX |
| | | | tablet | XX |
| | | | tablet | XX |
| | | | tablet | XX |
| | | | tablet | XX |
| | | | tablet | XX |
| | | | tablet | XX |
| | | | tablet | XX |
| | | | tablet | XX |
| | | | tablet | XX |
| | | | tablet | XX |
| | | | tablet | XX |
| | | | tablet | XX |
| | | | tablet | XX |
| | | | tablet | XX |
| | | | tablet | XX |
| 120/5 | | | suspension | XX |
| 120/5 | | | suspension | XX |
| 120/5 | | | suspension | XX |
| 120/5 | | | suspension | XX |
| | Pyrimethamine | 25 | tablet | XX |
| | | | tablet | XX |
| | | | tablet | XX |
| | | | tablet | XX |
| | | | tablet | XX |
| | | | tablet | XX |
| | | | tablet | XX |
| | | | tablet | XX |
| | | | tablet | XX |
| | | | tablet | XX |
| | | | tablet | XX |
| | | | tablet | XX |
| | | | tablet | XX |
| | | | tablet | XX |
| | | | tablet | XX |
| | | | tablet | XX |

| | | | |
|---|---|---|---|
| | | tablet | XX |
| | | tablet | XX |
| | | tablet | XX |
| | | tablet | XX |
| | | tablet | XX |
| | | tablet | XX |
| | | tablet | XX |
| | | tablet | XX |
| | | tablet | XX |
| | | tablet | XX |
| | | tablet | XX |
| | | tablet | XX |
| | | tablet | XX |
| | | tablet | XX |
| | | tablet | XX |
| | | tablet | XX |
| | | tablet | XX |
| | | tablet | XX |
| | | tablet | XX |
| | | tablet | XX |
| | | tablet | XX |
| | | tablet | XX |
| Pyrimethamine | 25 | tablet | XX |
| Pyrimethamine | 25 | tablet | XX |
| Pyrimethamine | 25 | tablet | XX |
| Pyrimethamine | 25 | tablet | XX |
| Pyrimethamine | 25 | tablet | XX |
| Pyrimethamine | 25 | tablet | XX |
| Pyrimethamine | 25 | tablet | XX |
| Pyrimethamine | 25 | tablet | XX |
| | | tablet | XX |
| | | tablet | XX |
| | | tablet | XX |
| | | tablet | XX |
| | | tablet | XX |
| | | tablet | XX |
| | | tablet | XX |
| | | tablet | XX |
| | | tablet | XX |
| | | tablet | XX |
| | | tablet | XX |
| | | tablet | XX |
| | | tablet | XX |
| | | tablet | XX |
| | | tablet | XX |
| | | tablet | XX |
| | | tablet | XX |
| | | tablet | XX |

| | |
|---|---|
| tablet | XX |
| tablet | XX |
| tablet | XX |
| tablet | XX |
| tablet | XX |
| tablet | XX |
| tablet | XX |

## Data of Mystery  and Overt Sampling Approach

| CDC % API1 | genname2 | strengthegen2 | Weight (g) | Width (mm) | Height (mm) |
|---|---|---|---|---|---|
| | lumefantrine | 120 | | | |
| | lumefantrine | 120 | | | |
| | lumefantrine | 120 | | | |
| | lumefantrine | 120 | | | |
| | lumefantrine | 120 | | | |
| | lumefantrine | 120 | | | |
| | lumefantrine | 120 | | | |
| | lumefantrine | 120 | | | |
| | lumefantrine | 120 | | | |
| | lumefantrine | 120 | | | |
| | lumefantrine | 120 | | | |
| | lumefantrine | 120 | | | |
| | lumefantrine | 120 | | | |
| | lumefantrine | 120 | | | |
| | lumefantrine | 120 | | | |
| | lumefantrine | 120 | | | |
| | lumefantrine | 120 | | | |
| | lumefantrine | 120 | | | |
| | lumefantrine | 120 | | | |
| | lumefantrine | 120 | | | |
| | lumefantrine | 120 | | | |
| | lumefantrine | 120 | | | |
| | lumefantrine | 120 | | | |
| | lumefantrine | 120 | | | |
| | lumefantrine | 120 | | | |
| | lumefantrine | 120 | | | |
| | lumefantrine | 120 | | | |
| 109.8 | lumefantrine | 120 | | | |
| | lumefantrine | 120 | | | |
| | lumefantrine | 120 | | | |
| | lumefantrine | 120 | | | |
| | lumefantrine | 120 | | | |
| | lumefantrine | 120 | | | |
| | lumefantrine | 120 | | | |
| | lumefantrine | 120 | | | |
| | lumefantrine | 120 | | | |
| | lumefantrine | 120 | | | |
| | lumefantrine | 120 | | | |
| | lumefantrine | 120 | | | |

| | | | |
|---|---|---|---|
| | lumefantrine | 120 | |
| | lumefantrine | 120 | |
| | lumefantrine | 120 | |
| | lumefantrine | 120 | |
| | lumefantrine | 120 | |
| | lumefantrine | 120 | |
| | lumefantrine | 120 | |
| | lumefantrine | 120 | |
| 108.7 | lumefantrine | 120 | |
| 110.9 | lumefantrine | 120 | |
| | lumefantrine | 120 | |
| 104.8 | lumefantrine | 120 | |
| | lumefantrine | 120 | |
| | lumefantrine | 120 | |
| | lumefantrine | 120 | |
| 105.0 | lumefantrine | 120 | |
| | lumefantrine | 120 | |
| 106.9 | lumefantrine | 120 | |
| | lumefantrine | 120 | |
| | lumefantrine | 120 | |
| | lumefantrine | 120 | |
| | lumefantrine | 120 | |
| | lumefantrine | 120 | |
| | lumefantrine | | 120 |
| | lumefantrine | 120 | |
| | lumefantrine | 120 | |
| | lumefantrine | 120 | |
| | lumefantrine | 120 | |
| | lumefantrine | 120 | |
| | lumefantrine | 120 | |
| | lumefantrine | 120 | |
| 105.6 | lumefantrine | 120 | |
| | lumefantrine | 120 | |
| | lumefantrine | 120 | |
| | lumefantrine | 120 | |
| | lumefantrine | 120 | |
| | lumefantrine | 120 | |
| | lumefantrine | 120 | |
| | lumefantrine | 120 | |
| | lumefantrine | 120 | |
| | lumefantrine | 120 | |
| | lumefantrine | 120 | |
| 103.2 | lumefantrine | 120 | |
| | lumefantrine | 120 | |
| | lumefantrine | 120 | |
| | lumefantrine | 120 | |
| | lumefantrine | 120 | |
| | lumefantrine | 120 | |
| | lumefantrine | 120 | |
| | lumefantrine | 120 | |

| | | |
|---|---|---|
| | lumefantrine | 120 |
| | lumefantrine | 120 |
| 105.4 | lumefantrine | 120 |
| | lumefantrine | 120 |
| | lumefantrine | 120 |
| | lumefantrine | 120 |
| | lumefantrine | 120 |
| | lumefantrine | 120 |
| | lumefantrine | 120 |
| | lumefantrine | 120 |
| | lumefantrine | 120 |
| | lumefantrine | 120 |
| | lumefantrine | 120 |
| | lumefantrine | 120 |
| | lumefantrine | 120 |
| | lumefantrine | 120 |
| | lumefantrine | 120 |
| | lumefantrine | 120 |
| | lumefantrine | 120 |
| | lumefantrine | 120 |
| | lumefantrine | 120 |
| | lumefantrine | 120 |
| | lumefantrine | 120 |
| | lumefantrine | 120 |
| | lumefantrine | 120 |
| | lumefantrine | 120 |
| | lumefantrine | 120 |
| | lumefantrine | 120 |
| | lumefantrine | 120 |
| | lumefantrine | 120 |
| | lumefantrine | 120 |
| | lumefantrine | 120 |
| | lumefantrine | 120 |
| | lumefantrine | 120 |
| | lumefantrine | 120 |
| | lumefantrine | 120 |
| | lumefantrine | 120 |
| 103.0 | lumefantrine | 120 |
| | lumefantrine | 120 |
| | lumefantrine | 120 |
| | lumefantrine | 120 |
| | lumefantrine | 120 |
| | lumefantrine | 120 |
| | lumefantrine | 120 |
| | lumefantrine | 120 |
| | lumefantrine | 120 |
| | lumefantrine | 120 |
| | lumefantrine | 120 |
| | lumefantrine | 120 |
| | lumefantrine | 120 |

| | | |
|---|---|---|
| | lumefantrine | 120 |
| | lumefantrine | 120 |
| | lumefantrine | 120 |
| | lumefantrine | 120 |
| | lumefantrine | 120 |
| 102.0 | lumefantrine | 120 |
| | lumefantrine | 120 |
| | lumefantrine | 120 |
| | lumefantrine | 120 |
| | lumefantrine | 120 |
| | lumefantrine | 120 |
| 106.6 | lumefantrine | 120 |
| | lumefantrine | 120 |
| | lumefantrine | 120 |
| 112.8 | lumefantrine | 120 |
| | lumefantrine | 120 |
| 105.0 | lumefantrine | 120 |
| 105.3 | lumefantrine | 120 |
| | lumefantrine | 120 |
| | lumefantrine | 120 |
| | lumefantrine | 120 |
| | lumefantrine | 120 |
| | lumefantrine | 120 |
| | lumefantrine | 120 |
| 110.0 | lumefantrine | 120 |
| | lumefantrine | 120 |
| | lumefantrine | 120 |
| | lumefantrine | 120 |
| | lumefantrine | 120 |
| | lumefantrine | 120 |
| | lumefantrine | 120 |
| | lumefantrine | 120 |
| | lumefantrine | 120 |
| | lumefantrine | 120 |
| | lumefantrine | 120 |
| | lumefantrine | 120 |
| | lumefantrine | 480 |
| | lumefantrine | 120 |
| | lumefantrine | 120 |
| | lumefantrine | 120 |
| | lumefantrine | 120 |
| | lumefantrine | 120 |
| | lumefantrine | 120 |
| | lumefantrine | 120 |
| | lumefantrine | 120 |
| | lumefantrine | 120 |
| | lumefantrine | 120 |
| | lumefantrine | 120 |
| | lumefantrine | 120 |
| 95.6 | lumefantrine | 120 |

| | | |
|---|---|---|
| | lumefantrine | 120 |
| | lumefantrine | 120 |
| | lumefantrine | 120 |
| | lumefantrine | 120 |
| | lumefantrine | 120 |
| 103.2 | lumefantrine | 120 |
| | lumefantrine | 120 |
| | lumefantrine | 120 |
| | lumefantrine | 120 |
| | lumefantrine | 120 |
| | lumefantrine | 120 |
| | lumefantrine | 120 |
| 114.8 | lumefantrine | 120 |
| | lumefantrine | 120 |
| | lumefantrine | 120 |
| | lumefantrine | 120 |
| | lumefantrine | 120 |
| | lumefantrine | 120 |
| | lumefantrine | 120 |
| | lumefantrine | 120 |
| 113.8 | lumefantrine | 120 |
| | lumefantrine | 120 |
| | lumefantrine | 120 |
| | lumefantrine | 120 |
| | lumefantrine | 120 |
| | lumefantrine | 120 |
| | lumefantrine | 120 |
| | lumefantrine | 120 |
| | lumefantrine | 120 |
| 117.5 | lumefantrine | 120 |
| | lumefantrine | 120 |
| | lumefantrine | 120 |
| | lumefantrine | 120 |
| | lumefantrine | 120 |
| | lumefantrine | 120 |
| | lumefantrine | 120 |
| | lumefantrine | 120 |
| | lumefantrine | 120 |
| | lumefantrine | 120 |
| | lumefantrine | 120 |
| | lumefantrine | 120 |
| | lumefantrine | 120 |
| | lumefantrine | 120 |
| | lumefantrine | 120 |
| | lumefantrine | 120 |
| | lumefantrine | 120 |
| | lumefantrine | 120 |
| | lumefantrine | 120 |
| | lumefantrine | 120 |
| | lumefantrine | 120 |

| | | |
|---|---|---|
| | lumefantrine | 120 |
| | lumefantrine | 120 |
| | lumefantrine | 120 |
| | lumefantrine | 120 |
| 96.4 | lumefantrine | 120 |
| | lumefantrine | 120 |
| | lumefantrine | 120 |
| | lumefantrine | 120 |
| 104.5 | lumefantrine | 120 |
| | lumefantrine | 120 |
| 107.2 | lumefantrine | 120 |
| | lumefantrine | 120 |
| | lumefantrine | 120 |
| 97.9 | lumefantrine | 120 |
| | lumefantrine | 120 |
| | lumefantrine | 120 |
| | lumefantrine | 120 |
| | lumefantrine | 120 |
| 93.8 | lumefantrine | 120 |
| | lumefantrine | 120 |
| | lumefantrine | 120 |
| | lumefantrine | 120 |
| | lumefantrine | 120 |
| 94.7 | lumefantrine | 120 |
| | lumefantrine | 120 |
| | lumefantrine | 120 |
| | lumefantrine | 120 |
| | lumefantrine | 120 |
| | lumefantrine | 120 |
| | lumefantrine | 120 |
| | lumefantrine | 120 |
| | lumefantrine | 120 |
| 105.4 | lumefantrine | 120 |
| | lumefantrine | 120 |
| 104.5 | lumefantrine | 120 |
| | lumefantrine | 120 |
| | lumefantrine | 120 |
| | lumefantrine | 120 |
| | lumefantrine | 120 |
| | lumefantrine | 120 |
| | lumefantrine | 120 |
| | lumefantrine | 120 |
| | lumefantrine | 120 |
| | lumefantrine | 120 |
| | lumefantrine | 120 |
| | lumefantrine | 120 |
| | lumefantrine | 120 |
| | lumefantrine | 120 |
| | lumefantrine | 120 |
| | lumefantrine | 120 |

| | | |
|---|---|---|
| | lumefantrine | 120 |
| | lumefantrine | 120 |
| | lumefantrine | 120 |
| | lumefantrine | 120 |
| 109.4 | lumefantrine | 120 |
| 99.7 | lumefantrine | 120 |
| | lumefantrine | 120 |
| | lumefantrine | 120 |
| | lumefantrine | 120 |
| 101.7 | lumefantrine | 120 |
| | lumefantrine | 120 |
| 115.2 | lumefantrine | 120 |
| | lumefantrine | 120 |
| | lumefantrine | 120 |
| | lumefantrine | 120 |
| | lumefantrine | 120 |
| | lumefantrine | 120 |
| 93.3 | lumefantrine | 120 |
| | lumefantrine | 120 |
| | lumefantrine | 120 |
| | lumefantrine | 120 |
| | lumefantrine | 120 |
| | lumefantrine | 120 |
| | lumefantrine | 120 |
| | lumefantrine | 120 |
| | lumefantrine | 120 |
| | lumefantrine | 120 |
| | lumefantrine | 120 |
| | lumefantrine | 120 |
| | lumefantrine | 120 |
| | lumefantrine | 120 |
| **lot detectabl** | lumefantrine | 120 |
| | lumefantrine | 120 |
| | lumefantrine | 120 |
| | lumefantrine | 120 |
| | lumefantrine | 120 |
| | lumefantrine | 120 |
| | lumefantrine | 120 |
| | lumefantrine | 120 |
| | lumefantrine | 120 |
| | lumefantrine | 120 |
| | lumefantrine | 120 |
| | lumefantrine | 120 |
| | lumefantrine | 120 |
| | lumefantrine | 120 |
| | lumefantrine | 120 |
| | lumefantrine | 120 |
| 111.0 | lumefantrine | 120 |
| 108.4 | lumefantrine | 120 |
| | lumefantrine | 120 |

| | | |
|---|---|---|
| | lumefantrine | 120 |
| | lumefantrine | 120 |
| | lumefantrine | 120 |
| | lumefantrine | 120 |
| | lumefantrine | 120 |
| | lumefantrine | 120 |
| | lumefantrine | 120 |
| | lumefantrine | 120 |
| | lumefantrine | 120 |
| | lumefantrine | 120 |
| | lumefantrine | 120 |
| | lumefantrine | 120 |
| | lumefantrine | 120 |
| | lumefantrine | 120 |
| | lumefantrine | 120 |
| | lumefantrine | 120 |
| | lumefantrine | 120 |
| | lumefantrine | 120 |
| | lumefantrine | 120 |
| | lumefantrine | 120 |
| | lumefantrine | 120 |
| | lumefantrine | 120 |
| | lumefantrine | 120 |
| | lumefantrine | 120 |
| | lumefantrine | 120 |
| | lumefantrine | 120 |
| | lumefantrine | 120 |
| | lumefantrine | 120 |
| | lumefantrine | 120 |
| | lumefantrine | 120 |
| | lumefantrine | 120 |
| | lumefantrine | 120 |
| | lumefantrine | 120 |
| | lumefantrine | 120 |
| | lumefantrine | 120 |
| 102.9 | lumefantrine | 120 |
| | lumefantrine | 120 |
| | lumefantrine | 120 |
| | lumefantrine | 120 |
| | lumefantrine | 120 |
| | lumefantrine | 120 |
| | lumefantrine | 120 |
| 86.8 | lumefantrine | 120 |
| | lumefantrine | 120 |
| | lumefantrine | 120 |
| | lumefantrine | 120 |
| | lumefantrine | 120 |
| | lumefantrine | 120 |
| 93.6 | lumefantrine | 120 |
| | lumefantrine | 120 |

| | | | |
|---|---|---|---|
| 96.7 | lumefantrine | 120 | |
| | lumefantrine | 120 | |
| | lumefantrine | 120 | |
| | lumefantrine | 120 | |
| | lumefantrine | 120 | |
| | lumefantrine | 120 | |
| | lumefantrine | 120 | |
| | lumefantrine | 120 | |
| 94.1 | lumefantrine | 120 | |
| | lumefantrine | 120 | |
| | lumefantrine | 120 | |
| 128.0 | lumefantrine | 120 | |
| | lumefantrine | 120 | |
| | lumefantrine | 120 | |
| | lumefantrine | 120 | |
| | lumefantrine | 120 | |
| | lumefantrine | 120 | |
| 102.3 | lumefantrine | 120 | |
| | lumefantrine | 120 | |
| | lumefantrine | 120 | |
| | lumefantrine | 120 | |
| | lumefantrine | 120 | |
| | lumefantrine | 120 | |
| 105.2 | lumefantrine | 120 | |
| | lumefantrine | 120 | |
| | lumefantrine | 120 | |
| | lumefantrine | 120 | |
| | lumefantrine | 120 | |
| | lumefantrine | 120 | |
| 104.0 | lumefantrine | 120 | |
| | lumefantrine | 120 | |
| | lumefantrine | 120 | |
| | lumefantrine | 120 | |
| 102.6 | lumefantrine | 120 | |
| | lumefantrine | 120 | |
| 104.9 | lumefantrine | 120 | |
| | lumefantrine | 120 | |
| | lumefantrine | 120 | |
| | lumefantrine | 120 | |
| | lumefantrine | 120 | |
| | lumefantrine | 120 | |
| 101.8 | lumefantrine | 120 | |
| | lumefantrine | 120 | |
| 111.5 | lumefantrine | 120 | |
| | lumefantrine | | 120 |
| | lumefantrine | 120 | |
| | lumefantrine | 120 | |
| | lumefantrine | 120 | |
| | lumefantrine | 120 | |
| | lumefantrine | 120 | |

| | | |
|---|---|---|
| | lumefantrine | 120 |
| | lumefantrine | 120 |
| | lumefantrine | 120 |
| | lumefantrine | 120 |
| | lumefantrine | 120 |
| | lumefantrine | 120 |
| | lumefantrine | 120 |
| | lumefantrine | 120 |
| | lumefantrine | 120 |
| | lumefantrine | 120 |
| | lumefantrine | 120 |
| | lumefantrine | 120 |
| | lumefantrine | 120 |
| | lumefantrine | 120 |
| | lumefantrine | 120 |
| | lumefantrine | 120 |
| | lumefantrine | 120 |
| | lumefantrine | 120 |
| | lumefantrine | 120 |
| | lumefantrine | 120 |
| | lumefantrine | 120 |
| | lumefantrine | 120 |
| | lumefantrine | 120 |
| | lumefantrine | 120 |
| | lumefantrine | 120 |
| | lumefantrine | 120 |
| | lumefantrine | 120 |
| | | |
| 95.9 | lumefantrine | 120 |
| | lumefantrine | 120 |
| 105.8 | lumefantrine | 120 |
| | lumefantrine | 120 |
| | lumefantrine | 120 |
| | lumefantrine | 120 |
| | lumefantrine | 120 |
| | lumefantrine | 120 |
| | lumefantrine | 120 |
| | lumefantrine | 120 |
| | lumefantrine | 120 |
| | lumefantrine | 120 |
| | lumefantrine | 120 |
| | lumefantrine | 120 |
| | lumefantrine | 120 |
| | lumefantrine | 120 |
| | lumefantrine | 120 |
| 93.9 | lumefantrine | 120 |
| | lumefantrine | 120 |
| | lumefantrine | 120 |
| | lumefantrine | 120 |
| | lumefantrine | 120 |

| | | |
|---|---|---|
| | lumefantrine | 120 |
| | lumefantrine | 120 |
| | lumefantrine | 120 |
| | lumefantrine | 120 |
| | lumefantrine | 120 |
| | lumefantrine | 120 |
| | lumefantrine | 120 |
| | lumefantrine | 120 |
| | lumefantrine | 120 |
| | lumefantrine | 120 |
| | lumefantrine | 120 |
| | lumefantrine | 120 |
| | lumefantrine | 120 |
| | lumefantrine | 120 |
| | lumefantrine | 120 |
| | lumefantrine | 120 |
| | lumefantrine | 120 |
| 102.4 | lumefantrine | 120 |
| | lumefantrine | 120 |
| | lumefantrine | 120 |
| | lumefantrine | 120 |
| 90.4 | lumefantrine | 120 |
| | lumefantrine | 120 |
| | lumefantrine | 120 |
| | lumefantrine | 120 |
| | lumefantrine | 120 |
| | lumefantrine | 120 |
| 83.2 | lumefantrine | 120 |
| | lumefantrine | 120 |
| | lumefantrine | 120 |
| | lumefantrine | 120 |
| | lumefantrine | 120 |
| | lumefantrine | 120 |
| | lumefantrine | 120 |
| | lumefantrine | 120 |
| | lumefantrine | 120 |
| | lumefantrine | 120 |
| 85.1 | lumefantrine | 120 |
| | lumefantrine | 120 |
| | lumefantrine | 120 |
| | lumefantrine | 120 |
| | lumefantrine | 120 |
| | lumefantrine | 120 |
| | lumefantrine | 120 |
| | lumefantrine | 120 |
| | lumefantrine | 120 |
| | lumefantrine | 120 |
| | lumefantrine | 120 |
| | lumefantrine | 120 |
| | lumefantrine | 120 |

| | | |
|---|---|---|
| | lumefantrine | 120 |
| | lumefantrine | 120 |
| | lumefantrine | 120 |
| | lumefantrine | 120 |
| | lumefantrine | 120 |
| | lumefantrine | 120 |
| | lumefantrine | 120 |
| | lumefantrine | 120 |
| 94.4 | lumefantrine | 120 |
| | lumefantrine | 120 |
| | lumefantrine | 120 |
| | lumefantrine | 120 |
| | lumefantrine | 120 |
| | lumefantrine | 120 |
| | lumefantrine | 120 |
| | lumefantrine | 120 |
| | lumefantrine | 120 |
| | lumefantrine | 120 |
| 89.7 | lumefantrine | 120 |
| 95.9 | lumefantrine | 120 |
| | lumefantrine | 120 |
| | lumefantrine | 120 |
| | lumefantrine | 120 |
| 102.2 | lumefantrine | 120 |
| | lumefantrine | 120 |
| | lumefantrine | 120 |
| | lumefantrine | 120 |
| | lumefantrine | 120 |
| | lumefantrine | 120 |
| | lumefantrine | 120 |
| 119.8 | lumefantrine | 120 |
| | lumefantrine | 120 |
| | lumefantrine | 120 |
| 108.6 | lumefantrine | 120 |
| | lumefantrine | 120 |
| | lumefantrine | 120 |
| | lumefantrine | 120 |
| | lumefantrine | 120 |
| | lumefantrine | 120 |
| | lumefantrine | 120 |
| | lumefantrine | 120 |
| | lumefantrine | 120 |
| | lumefantrine | 120 |
| | lumefantrine | 120 |
| | lumefantrine | 240 |
| | lumefantrine | 240 |
| | lumefantrine | 240 |
| | lumefantrine | 240 |
| | lumefantrine | 240 |
| | lumefantrine | 240 |

| | | |
|---|---|---|
| | lumefantrine | 240 |
| | lumefantrine | 240 |
| | lumefantrine | 240 |
| | lumefantrine | 240 |
| | lumefantrine | 240 |
| 100.6 | lumefantrine | 240 |
| | lumefantrine | 240 |
| | lumefantrine | 240 |
| | lumefantrine | 240 |
| | lumefantrine | 240 |
| | lumefantrine | 240 |
| | lumefantrine | 240 |
| | lumefantrine | 240 |
| | lumefantrine | 240 |
| | lumefantrine | 240 |
| 92.7 | lumefantrine | 240 |
| | lumefantrine | 240 |
| | lumefantrine | 240 |
| | lumefantrine | 240 |
| | lumefantrine | 240 |
| | lumefantrine | 240 |
| | lumefantrine | 240 |
| | lumefantrine | 240 |
| | lumefantrine | 240 |
| | lumefantrine | 240 |
| | lumefantrine | 240 |
| | lumefantrine | 240 |
| | lumefantrine | 480 |
| | lumefantrine | 480 |
| | lumefantrine | 480 |
| 91.6 | lumefantrine | 480 |
| | lumefantrine | 480 |
| 96.2 | lumefantrine | 480 |
| | lumefantrine | 480 |
| | lumefantrine | 480 |
| | lumefantrine | 480 |
| | lumefantrine | 480 |
| | lumefantrine | 480 |
| | lumefantrine | 480 |
| 83.2 | lumefantrine | 480 |
| 105.2 | lumefantrine | 480 |
| | lumefantrine | 480 |
| | lumefantrine | 480 |
| | lumefantrine | 480 |
| | lumefantrine | 480 |
| | lumefantrine | 480 |
| | lumefantrine | 480 |
| | lumefantrine | 480 |
| | lumefantrine | 480 |
| | lumefantrine | 480 |

| | | | |
|---|---|---|---|
| | lumefantrine | 480 | |
| | lumefantrine | 480 | |
| | lumefantrine | 480 | |
| | lumefantrine | 480 | |
| | lumefantrine | 480 | |
| | lumefantrine | 480 | |
| | lumefantrine | 480 | |
| | lumefantrine | 480 | |
| | lumefantrine | 480 | |
| | lumefantrine | 480 | |
| | lumefantrine | 480 | |
| | lumefantrine | 480 | |
| | lumefantrine | 480 | |
| | lumefantrine | 480 | |
| | lumefantrine | 480 | |
| | lumefantrine | 480 | |
| | lumefantrine | 480 | |
| | lumefantrine | 480 | |
| 100.9 | lumefantrine | 480 | |
| | lumefantrine | 480 | |
| | lumefantrine | 480 | |
| | lumefantrine | 480 | |
| | lumefantrine | 480 | |
| | lumefantrine | 480 | |
| | lumefantrine | 480 | |
| | lumefantrine | 480 | |
| 100.7 | lumefantrine | 480 | |
| | lumefantrine | 480 | |
| | lumefantrine | 480 | |
| | lumefantrine | | 480 |
| | lumefantrine | 480 | |
| | lumefantrine | 480 | |
| | lumefantrine | 480 | |
| 99.9 | lumefantrine | 480 | |
| | lumefantrine | 480 | |
| | lumefantrine | | 240 |
| | lumefantrine | 480 | |
| | lumefantrine | 480 | |
| | lumefantrine | 480 | |
| | lumefantrine | 480 | |
| | lumefantrine | 480 | |
| | lumefantrine | 480 | |
| | lumefantrine | 480 | |
| | lumefantrine | 480 | |
| | lumefantrine | 480 | |
| | lumefantrine | 480 | |
| | lumefantrine | 480 | |
| | lumefantrine | 480 | |
| | lumefantrine | 480 | |
| | lumefantrine | 480 | |

| | | |
|---|---|---|
| | lumefantrine | 480 |
| | lumefantrine | 480 |
| | lumefantrine | 480 |
| | lumefantrine | 480 |
| | lumefantrine | 480 |
| | lumefantrine | 480 |
| | lumefantrine | 480 |
| | lumefantrine | 480 |
| | lumefantrine | 480 |
| | lumefantrine | 480 |
| | lumefantrine | 480 |
| | lumefantrine | 480 |
| | lumefantrine | 480 |
| | lumefantrine | 480 |
| | lumefantrine | 480 |
| | lumefantrine | 480 |
| | lumefantrine | 480 |
| | lumefantrine | 480 |
| | lumefantrine | 480 |
| 105.4 | lumefantrine | 480 |
| | lumefantrine | 480 |
| | lumefantrine | 480 |
| | lumefantrine | 480 |
| | lumefantrine | 480 |
| | lumefantrine | 480 |
| 92.3 | lumefantrine | 480 |
| | lumefantrine | 480 |
| | lumefantrine | 480 |
| | lumefantrine | 480 |
| | lumefantrine | 480 |
| | lumefantrine | 480 |
| | lumefantrine | 480 |
| | lumefantrine | 480 |
| | lumefantrine | 480 |
| | lumefantrine | 480 |
| | lumefantrine | 480 |
| | lumefantrine | 480 |
| | lumefantrine | 480 |
| | lumefantrine | 480 |
| | lumefantrine | 480 |
| | lumefantrine | 480 |
| | lumefantrine | 480 |
| | lumefantrine | 480 |
| | lumefantrine | 480 |
| | lumefantrine | 480 |
| | lumefantrine | 480 |
| 96.2 | lumefantrine | 480 |
| | lumefantrine | 480 |
| | lumefantrine | 480 |
| | lumefantrine | 480 |

| | | |
|---|---|---|
| | lumefantrine | 480 |
| | lumefantrine | 480 |
| | lumefantrine | 480 |
| | lumefantrine | 480 |
| | lumefantrine | 480 |
| 97.7 | lumefantrine | 480 |
| | lumefantrine | 480 |
| | lumefantrine | 480 |
| | lumefantrine | 480 |
| | lumefantrine | 480 |
| | lumefantrine | 480 |
| | lumefantrine | 480 |
| | lumefantrine | 480 |
| | lumefantrine | 480 |
| 98.5 | lumefantrine | 480 |
| | lumefantrine | 480 |
| | lumefantrine | 480 |
| | lumefantrine | 480 |
| | lumefantrine | 480 |
| | lumefantrine | 480 |
| | lumefantrine | 480 |
| | lumefantrine | 480 |
| | lumefantrine | 480 |
| | lumefantrine | 480 |
| | lumefantrine | 480 |
| | lumefantrine | 480 |
| | lumefantrine | 480 |
| | lumefantrine | 480 |
| 101.9 | lumefantrine | 480 |
| | lumefantrine | 480 |
| | lumefantrine | 480 |
| | lumefantrine | 480 |
| | lumefantrine | 480 |
| | lumefantrine | 480 |
| 118.2 | lumefantrine | 480 |
| | lumefantrine | 480 |
| | lumefantrine | 480 |
| 98.1 | lumefantrine | 480 |
| | lumefantrine | 480 |
| | lumefantrine | 480 |
| | lumefantrine | 480 |
| | lumefantrine | 480 |
| | lumefantrine | 480 |
| | lumefantrine | 480 |
| | lumefantrine | 480 |
| | lumefantrine | 480 |
| | lumefantrine | 480 |
| | lumefantrine | 480 |
| | lumefantrine | 480 |
| | lumefantrine | 480 |

| | | |
|---|---|---|
| | lumefantrine | 480 |
| | lumefantrine | 480 |
| | lumefantrine | 480 |
| | lumefantrine | 480 |
| | lumefantrine | 480 |
| | lumefantrine | 480 |
| | lumefantrine | 480 |
| | lumefantrine | 480 |
| | lumefantrine | 480 |
| | lumefantrine | 480 |
| | lumefantrine | 480 |
| | lumefantrine | 480 |
| | lumefantrine | 480 |
| 98.1 | lumefantrine | 480 |
| | lumefantrine | 480 |
| | lumefantrine | 480 |
| | lumefantrine | 480 |
| | lumefantrine | 480 |
| | lumefantrine | 480 |
| | lumefantrine | 480 |
| | lumefantrine | 480 |
| | lumefantrine | 480 |
| | lumefantrine | 480 |
| | lumefantrine | 480 |
| | lumefantrine | 480 |
| | lumefantrine | 480 |
| | lumefantrine | 480 |
| | lumefantrine | 480 |
| | lumefantrine | 480 |
| | lumefantrine | 480 |
| | lumefantrine | 480 |
| | lumefantrine | 480 |
| | lumefantrine | 480 |
| | lumefantrine | 480 |
| | lumefantrine | 480 |
| | lumefantrine | 480 |
| | lumefantrine | 480 |
| | lumefantrine | 480 |
| | lumefantrine | 480 |
| | lumefantrine | 480 |
| | lumefantrine | 480 |
| | lumefantrine | 480 |
| | lumefantrine | 480 |
| **lot detectabl** | lumefantrine | 480 |
| | lumefantrine | 480 |
| | lumefantrine | 480 |
| | lumefantrine | 480 |
| | lumefantrine | 480 |
| | lumefantrine | 480 |
| 103.6 | lumefantrine | 480 |

| | | |
|---|---|---|
| | lumefantrine | 480 |
| | lumefantrine | 480 |
| | lumefantrine | 480 |
| | lumefantrine | 480 |
| | lumefantrine | 480 |
| 101.2 | lumefantrine | 480 |
| | lumefantrine | 480 |
| | lumefantrine | 480 |
| | lumefantrine | 480 |
| | lumefantrine | 480 |
| 102.7 | lumefantrine | 480 |
| | lumefantrine | 480 |
| 98.7 | lumefantrine | 480 |
| | lumefantrine | 480 |
| | lumefantrine | 480 |
| | lumefantrine | 480 |
| | lumefantrine | 480 |
| | lumefantrine | 480 |
| | lumefantrine | 480 |
| | lumefantrine | 480 |
| | lumefantrine | 480 |
| | lumefantrine | 480 |
| | lumefantrine | 480 |
| | lumefantrine | 480 |
| 105.5 | lumefantrine | 480 |
| | lumefantrine | 480 |
| | lumefantrine | 480 |
| | lumefantrine | 480 |
| | lumefantrine | 480 |
| | lumefantrine | 480 |
| | lumefantrine | 480 |
| | lumefantrine | 480 |
| | lumefantrine | 480 |
| | lumefantrine | 480 |
| | lumefantrine | 480 |
| | lumefantrine | 480 |
| | lumefantrine | 480 |
| | lumefantrine | 480 |
| | lumefantrine | 480 |
| | lumefantrine | 480 |
| | lumefantrine | 480 |
| | lumefantrine | 480 |
| | lumefantrine | 480 |
| | lumefantrine | 480 |
| | lumefantrine | 480 |
| | lumefantrine | 480 |
| | lumefantrine | 480 |
| | lumefantrine | 480 |
| | lumefantrine | 480 |
| | lumefantrine | 480 |

| | | | |
|---|---|---|---|
| | lumefantrine | 480 | |
| 103.5 | lumefantrine | 480 | |
| | lumefantrine | 480 | |
| | lumefantrine | 480 | |
| **lot detectabl** | lumefantrine | 480 | |
| | lumefantrine | 480 | |
| | lumefantrine | 480 | |
| | lumefantrine | 480 | |
| | lumefantrine | 480 | |
| | lumefantrine | 480 | |
| | lumefantrine | 480 | |
| | lumefantrine | 480 | |
| | lumefantrine | 480 | |
| | lumefantrine | 480 | |
| | lumefantrine | 480 | |
| 98.0 | lumefantrine | 480 | |
| | lumefantrine | 480 | |
| | lumefantrine | | 120 |
| | lumefantrine | 480 | |
| | lumefantrine | 480 | |
| | lumefantrine | 480 | |
| | lumefantrine | 480 | |
| | lumefantrine | 480 | |
| | lumefantrine | 480 | |
| | lumefantrine | 480 | |
| | lumefantrine | 480 | |
| | lumefantrine | 480 | |
| | lumefantrine | 480 | |
| | lumefantrine | 480 | |
| 107.9 | lumefantrine | 480 | |
| 116.5 | lumefantrine | 480 | |
| | lumefantrine | 480 | |
| | lumefantrine | 480 | |
| | lumefantrine | 480 | |
| | lumefantrine | 480 | |
| | lumefantrine | 480 | |
| | lumefantrine | 480 | |
| | lumefantrine | 480 | |
| | lumefantrine | 480 | |
| | lumefantrine | 480 | |
| | lumefantrine | 480 | |
| | lumefantrine | 480 | |
| | lumefantrine | 480 | |
| | lumefantrine | 480 | |
| | lumefantrine | 1080 | |
| | lumefantrine | 480 | |
| 91.4 | lumefantrine | 1080 | |
| | lumefantrine | 480 | |
| | lumefantrine | 480 | |
| | lumefantrine | 480 | |

| | | |
|---|---|---|
| | lumefantrine | 480 |
| | lumefantrine | 480 |
| | lumefantrine | 480 |
| | lumefantrine | 480 |
| | lumefantrine | 480 |
| | lumefantrine | 480 |
| | lumefantrine | 480 |
| | lumefantrine | 480 |
| | lumefantrine | 480 |
| | lumefantrine | 480 |
| 0.0 | lumefantrine | 480 |
| | lumefantrine | 480 |
| | lumefantrine | 480 |
| | lumefantrine | 480 |
| | lumefantrine | 480 |
| | lumefantrine | 480 |
| | lumefantrine | 480 |
| | lumefantrine | 480 |
| | lumefantrine | 480 |
| | lumefantrine | 480 |
| | lumefantrine | 480 |
| | lumefantrine | 480 |
| | lumefantrine | 480 |
| | lumefantrine | 480 |
| | lumefantrine | 480 |
| | lumefantrine | 480 |
| | lumefantrine | 480 |
| | lumefantrine | 480 |
| | lumefantrine | 480 |
| | lumefantrine | 480 |
| | lumefantrine | 480 |
| | lumefantrine | 480 |
| | lumefantrine | 480 |
| | lumefantrine | 480 |
| | lumefantrine | 480 |
| | lumefantrine | 480 |
| | lumefantrine | 480 |
| | lumefantrine | 480 |
| 103.7 | lumefantrine | 480 |
| | lumefantrine | 480 |
| | lumefantrine | 480 |
| | lumefantrine | 480 |
| | lumefantrine | 480 |
| | lumefantrine | 480 |
| 104.6 | lumefantrine | 480 |
| | lumefantrine | 480 |
| | lumefantrine | 480 |
| | lumefantrine | 480 |
| | lumefantrine | 480 |
| | lumefantrine | 480 |

| | | |
|---|---|---|
| | lumefantrine | 480 |
| | lumefantrine | 480 |
| | lumefantrine | 480 |
| | lumefantrine | 480 |
| | lumefantrine | 480 |
| | lumefantrine | 480 |
| | lumefantrine | 480 |
| 104.1 | lumefantrine | 480 |
| | lumefantrine | 480 |
| | lumefantrine | 480 |
| | lumefantrine | 480 |
| | lumefantrine | 480 |
| | lumefantrine | 480 |
| 96.0 | | |
| 89.0 | | |
| 92.0 | | |

**lot detectabl**

96.0
88.0
94.0

91.0
93.0

| | | | | | |
|---|---|---|---|---|---|
| | amodiaquine | 67.5 | | | |
| | amodiaquine | 67.5 | | | |
| | amodiaquine | 135 | | | |
| | amodiaquine | 135 | | | |
| | amodiaquine | 135 | | | |
| | amodiaquine | 135 | | | |
| | amodiaquine | 135 | | | |
| | amodiaquine | 135 | | | |
| | amodiaquine | 135 | | | |
| | amodiaquine | 135 | | | |
| | amodiaquine | 135 | | | |
| | amodiaquine | 135 | | | |
| | amodiaquine | 135 | | | |
| 87.0 | amodiaquine | 135 | | | |
| | amodiaquine | 135 | | | |
| 89.0 | amodiaquine | 135 | | | |
| | amodiaquine | 135 | | | |
| | amodiaquine | 135 | | | |
| | amodiaquine | 135 | | | |
| | amodiaquine | 135 | | | |
| | amodiaquine | 135 | | | |
| | amodiaquine | 135 | | | |
| | amodiaquine | 135 | | | |
| 93.0 | amodiaquine | 135 | | | |
| | amodiaquine | 135 | | | |
| | amodiaquine | 135 | | | |
| | amodiaquine | 150 | 0.2499 | 9.16 | 4.34 |
| | amodiaquine | 150 | 0.2552 | 9.19 | 4.15 |
| | amodiaquine | 150 | 0.2644 | 9.17 | 3.68 |
| | amodiaquine | 150 | 0.2704 | 9.08 | 3.56 |
| | amodiaquine | 150 | 0.2702 | 9.09 | 3.55 |
| | amodiaquine | 150 | 0.2633 | 9.1 | 3.51 |
| | amodiaquine | 150 | 0.2654 | 9.07 | 3.56 |
| 84.0 | amodiaquine | 150 | | | |
| 90.0 | amodiaquine | 150 | | | |
| | amodiaquine | 150 | | | |
| | amodiaquine | 150 | | | |
| | amodiaquine | 150 | | | |
| | amodiaquine | 150 | | | |
| | amodiaquine | 150 | | | |
| | amodiaquine | 150 | | | |
| | amodiaquine | 150 | | | |
| 87.0 | amodiaquine | 150 | 0.2674 | 9.19 | 3.7 |
| | amodiaquine | 150 | 0.2677 | 9.07 | 3.51 |
| | amodiaquine | 150 | 0.2691 | 9.09 | 3.53 |

| | | | | | | |
|---|---|---|---|---|---|---|
| | amodiaquine | 150 | | 0.2657 | 9.17 | 3.63 |
| | amodiaquine | 150 | | 0.2669 | 9.12 | 3.48 |
| 91.0 | amodiaquine | 150 | | 0.2682 | 9.09 | 3.55 |
| | amodiaquine | 150 | | 0.2788 | 9.1 | 3.63 |
| | amodiaquine | 150 | | | | |
| | amodiaquine | 150 | | | | |
| | amodiaquine | 150 | | | | |
| | amodiaquine | 150 | | | | |
| | amodiaquine | 150 | | | | |
| | amodiaquine | 150 | | | | |
| | amodiaquine | 150 | | | | |
| | amodiaquine | 150 | | | | |
| | amodiaquine | 150 | | | | |
| | amodiaquine | 150 | | | | |
| | amodiaquine | 150 | | 0.2635 | 9.08 | 3.53 |
| | amodiaquine | | 153 | 0.267 | 11.06 | 3.41 |
| | amodiaquine | 153 | | 0.2735 | 11.02 | 3.42 |
| | amodiaquine | 153 | | 0.2618 | 11.05 | 3.4 |
| | amodiaquine | 153.1 | | 0.3097 | 9.08 | 3.87 |
| | amodiaquine | 153.1 | | 0.3062 | 9.09 | 3.83 |
| 96.0 | amodiaquine | 153.1 | | 0.3048 | 9.09 | 3.81 |
| | amodiaquine | 153.1 | | 0.3073 | 9.06 | 3.85 |
| | amodiaquine | 153.1 | | 0.2952 | 9.13 | 3.73 |
| | amodiaquine | 270 | | | | |
| | amodiaquine | 270 | | | | |
| | amodiaquine | 270 | | | | |
| 96.2 | amodiaquine | 270 | | | | |
| | amodiaquine | 270 | | | | |
| | amodiaquine | 270 | | | | |
| | amodiaquine | 270 | | | | |
| | amodiaquine | 270 | | | | |
| | amodiaquine | 270 | | | | |
| | amodiaquine | 270 | | | | |
| | amodiaquine | 270 | | | | |
| | amodiaquine | 270 | | | | |
| | amodiaquine | 270 | | | | |
| | amodiaquine | 270 | | | | |
| | amodiaquine | 270 | | | | |
| | amodiaquine | 270 | | | | |
| | amodiaquine | 270 | | | | |
| | amodiaquine | 270 | | | | |
| | amodiaquine | 270 | | | | |
| | amodiaquine | 270 | | | | |
| | amodiaquine | 270 | | | | |
| | amodiaquine | 270 | | | | |
| | amodiaquine | 270 | | | | |
| | amodiaquine | 270 | | | | |
| | amodiaquine | 270 | | | | |
| | amodiaquine | 270 | | | | |
| | amodiaquine | 270 | | | | |

| | | | | | | |
|---|---|---|---|---|---|---|
| | amodiaquine | 270 | | | | |
| | amodiaquine | 270 | | | | |
| | amodiaquine | 270 | | | | |
| | amodiaquine | 270 | | | | |
| | amodiaquine | 270 | | | | |
| | amodiaquine | 270 | | | | |
| | amodiaquine | 270 | | | | |
| | amodiaquine | 270 | | | | |
| | amodiaquine | 270 | | | | |
| | amodiaquine | 270 | | | | |
| | amodiaquine | 270 | | | | |
| | amodiaquine | 270 | | | | |
| | amodiaquine | 270 | | | | |
| | amodiaquine | 270 | | | | |
| | amodiaquine | 270 | | | | |
| | amodiaquine | 270 | | | | |
| | amodiaquine | 270 | | | | |
| | amodiaquine | 270 | | | | |
| | amodiaquine | 270 | | | | |
| | amodiaquine | 270 | | | | |
| | amodiaquine | 270 | | | | |
| | amodiaquine | 270 | | | | |
| | amodiaquine | 270 | | | | |
| | amodiaquine | 270 | | | | |
| | amodiaquine | 270 | | | | |
| | amodiaquine | 270 | | | | |
| | amodiaquine | 270 | | | | |
| | amodiaquine | 270 | | | | |
| | amodiaquine | 270 | | | | |
| | amodiaquine | 270 | | | | |
| | amodiaquine | 270 | | | | |
| | amodiaquine | 270 | | | | |
| 99.6 | amodiaquine | 270 | | | | |
| | amodiaquine | 270 | | | | |
| 107.4 | amodiaquine | 270 | | | | |
| 101.0 | amodiaquine | 270 | | | | |
| 98.6 | amodiaquine | 270 | | | | |
| | amodiaquine | 270 | | | | |
| | amodiaquine | 270 | | | | |
| | amodiaquine | | 300 | 0.5068 | 12.7 | 4.89 |
| 89.0 | amodiaquine | 300 | | 0.511 | 12.76 | 4.75 |
| 82.0 | amodiaquine | 300 | | 0.5065 | 12.74 | 4.8 |
| | amodiaquine | 300 | | 0.5042 | 12.71 | 4.76 |
| | amodiaquine | 300 | | 0.5122 | 12.72 | 4.76 |
| | amodiaquine | | 300 | 0.4954 | 12.7 | 4.89 |
| | amodiaquine | 300 | | 0.5227 | 12.71 | 4.79 |
| | amodiaquine | 300 | | 0.506 | 12.63 | 4.67 |
| | amodiaquine | | 300 | 0.5 | 12.67 | 4.68 |
| | amodiaquine | 300 | | 0.5153 | 12.72 | 4.91 |
| | amodiaquine | 300 | | 0.5022 | 12.62 | 4.69 |

| | | | | | | |
|---|---|---|---|---|---|---|
| 90.0 | amodiaquine | | 300 | 0.5079 | 12.63 | 4.67 |
| | amodiaquine | | 300 | | | |
| | amodiaquine | | 300 | | | |
| | amodiaquine | | 300 | | | |
| | amodiaquine | | 300 | | | |
| | amodiaquine | | 300 | | | |
| 92.0 | amodiaquine | | 300 | | | |
| 81.0 | amodiaquine | 300 | | | | |
| | amodiaquine | | 300 | | | |
| | amodiaquine | | 300 | | | |
| | amodiaquine | 300 | | | | |
| | amodiaquine | | 300 | | | |
| | amodiaquine | 300 | | | | |
| | amodiaquine | | 300 | | | |
| | amodiaquine | | 300 | | | |
| | amodiaquine | | 300 | | | |
| | amodiaquine | | 300 | | | |
| | amodiaquine | | 300 | | | |
| | amodiaquine | 300 | | | | |
| | amodiaquine | | 300 | | | |
| | amodiaquine | | 300 | | | |
| | amodiaquine | | 300 | 0.5086 | 12.68 | 4.74 |
| | amodiaquine | 300 | | 0.4956 | 12.69 | 4.91 |
| | amodiaquine | 300 | | 0.5005 | 12.63 | 4.66 |
| | amodiaquine | | 300 | 0.514 | 12.7 | 4.76 |
| | amodiaquine | 300 | | 0.5077 | 12.7 | 4.76 |
| | amodiaquine | | 300 | | | |
| | amodiaquine | | 300 | | | |
| | amodiaquine | | 300 | | | |
| | amodiaquine | | 300 | | | |
| | amodiaquine | 300 | | | | |
| | amodiaquine | | 300 | | | |
| | amodiaquine | | 300 | | | |
| | amodiaquine | 300 | | | | |
| | amodiaquine | | 300 | | | |
| | amodiaquine | | 300 | | | |
| | amodiaquine | 300 | | | | |
| | amodiaquine | | 300 | | | |
| 89.0 | amodiaquine | | 300 | | | |
| 90.0 | amodiaquine | | 300 | | | |
| 91.0 | amodiaquine | | 300 | | | |
| | amodiaquine | 300 | | | | |
| 91.0 | amodiaquine | | 300 | | | |
| | amodiaquine | 300 | | | | |
| 89.0 | amodiaquine | 300 | | | | |
| | amodiaquine | 300 | | | | |
| | amodiaquine | 300 | | | | |
| | amodiaquine | 306.3 | | | | |
| | amodiaquine | | 600 | | | |
| | amodiaquine | 600 | | 0.861 | 19.18 | 9.1 |

| | | | | | | |
|---|---|---|---|---|---|---|
| | amodiaquine | | 600 | | | |
| | amodiaquine | | 75 | 0.512 | | |
| | amodiaquine | | 75 | 0.5136 | | |
| | amodiaquine | | 75 | 0.4903 | | |
| | amodiaquine | | 75 | 0.5036 | | |
| | amodiaquine | | 75 | 0.4689 | | |
| | amodiaquine | | 75 | 0.5193 | | |
| | amodiaquine | | 75 | 0.501 | | |
| | amodiaquine | | 75 | | | |
| | amodiaquine | | 75 | | | |
| | amodiaquine | | 75 | | | |
| | amodiaquine | 150 | | 0.9547 | | |
| | amodiaquine | 150 | | 0.9848 | | |
| | amodiaquine | | 150 | 1.0268 | | |
| | amodiaquine | 150 | | 0.9882 | | |
| | amodiaquine | | 150 | 0.9918 | | |
| | amodiaquine | 150 | | | | |
| | amodiaquine | 150 | | | | |
| | amodiaquine | 150 | | | | |
| | amodiaquine | 150 | | | | |
| | amodiaquine | | 150 | | | |
| | amodiaquine | | 150 | | | |
| | amodiaquine | | 150 | | | |
| | amodiaquine | 150 | | | | |
| | amodiaquine | | 150 | | | |
| | amodiaquine | | 150 | | | |
| | amodiaquine | 150 | | | | |
| | amodiaquine | 150 | | | | |
| | amodiaquine | | 150 | | | |
| | amodiaquine | 150 | | | | |
| | amodiaquine | 150 | | | | |
| | amodiaquine | 150 | | | | |
| | amodiaquine | | 150 | | | |
| | amodiaquine | 150 | | | | |
| | amodiaquine | | 150 | | | |
| | amodiaquine | | 150 | | | |
| 93.0 | mefloquine | 125 | | 0.265 | 9.59 | 4.1 |
| | mefloquine | 125 | | 0.2701 | 9.57 | 4.14 |
| 96.0 | mefloquine | 125 | | 0.2683 | 9.59 | 4.18 |
| 94.0 | mefloquine | 125 | | 0.2683 | 9.56 | 4.14 |
| | mefloquine | 125 | | 0.2707 | 9.6 | 4.05 |
| | mefloquine | 125 | | | | |
| | mefloquine | 125 | | | | |
| | mefloquine | 125 | | | | |
| | mefloquine | 125 | | | | |
| 77.0 | mefloquine | 250 | | 0.5091 | 12.2 | 4.86 |
| | mefloquine | 250 | | 0.5288 | 12.32 | 5.07 |
| | mefloquine | 250 | | 0.5184 | 12.23 | 4.99 |
| | mefloquine | 250 | | 0.5451 | 12.11 | 4.36 |
| | mefloquine | 250 | | 0.5398 | 12.1 | 4.71 |

| | | | | | | |
|---|---|---|---|---|---|---|
| 89.0 | mefloquine | 250 | | 0.5301 | 12.07 | 4.4 |
| | mefloquine | 250 | | 0.5515 | 12.08 | 4.67 |
| | mefloquine | 250 | | 0.5454 | 12.07 | 4.65 |
| | mefloquine | 250 | | | | |
| 92.0 | mefloquine | 250 | | | | |
| | mefloquine | 250 | | | | |
| | mefloquine | 250 | | | | |
| | mefloquine | 250 | | | | |
| | mefloquine | 250 | | | | |
| | mefloquine | 250 | | | | |
| | mefloquine | 250 | | | | |
| | mefloquine | 250 | | | | |
| | mefloquine | 250 | | | | |
| | mefloquine | 250 | | | | |
| | mefloquine | 250 | | | | |
| | mefloquine | 250 | | | | |
| | mefloquine | 250 | | | | |
| 75.0 | mefloquine | 250 | | | | |
| | mefloquine | 250 | | | | |
| | mefloquine | 250 | | | | |
| | mefloquine | 250 | | | | |
| | mefloquine | 250 | | | | |
| | mefloquine | 250 | | | | |
| | mefloquine | 250 | | | | |
| | mefloquine | 250 | | | | |
| 95.0 | mefloquine | 250 | | | | |
| | mefloquine | 250 | | | | |
| | mefloquine | 250 | | | | |
| | mefloquine | 250 | | | | |
| | mefloquine | 250 | | | | |
| | mefloquine | 250 | | | | |
| | mefloquine | 250 | | | | |
| | mefloquine | 275 | | | 19.25 | 9.13 |
| | sulfadoxine | 500 | | 0.7037 | 13.1 | 4.23 |
| | sulfadoxine | 500 | | 0.6427 | 12.85 | 4.29 |
| | sulfadoxine | 500 | | 0.6571 | 12.61 | 4.22 |
| | sulfadoxine | 500 | | 0.6415 | 12.57 | 4.16 |
| | sulfadoxine | 500 | | 0.6782 | 12.61 | 4.28 |
| | sulfadoxine | 500 | | 0.6535 | 12.6 | 4.13 |
| | sulfadoxine | 500 | | 0.6525 | 12.57 | 4.23 |
| | sulfadoxine | 500 | | 0.6911 | 13.1 | 4.33 |
| | sulfadoxine | 500 | | 0.6949 | 13.1 | 4.39 |
| | sulfadoxine | 500 | | 0.6788 | 19.28 | 7.14 |
| | sulfadoxine | 500 | | 0.6411 | 19.04 | 7.08 |
| | sulfamethoxypyrazine | 250 | | | | |
| | sulfamethoxypyrazine | | 250 | | | |
| | sulfamethoxypyrazine | 250 | | | | |
| | sulfamethoxypyrazine | 250 | | | | |
| | sulfamethoxypyrazine | 250 | | | | |
| | sulfamethoxypyrazine | | 250 | | | |

| | | | | | |
|---|---|---|---|---|---|
| | sulfamethoxypyrazine | 250 | | | |
| | sulfamethoxypyrazine | 250 | | | |
| | sulfamethoxypyrazine | 250 | | | |
| | piperaquine | 225 | | | |
| | sulfamethoxypyrazine | 500 | | | |
| | sulfamethoxypyrazine | 500 | | | |
| | sulfamethoxypyrazine | 500 | | | |
| | sulfamethoxypyrazine | 500 | | | |
| | sulfamethoxypyrazine | 500 | | | |
| | sulfamethoxypyrazine | 500 | | | |
| | sulfamethoxypyrazine | 500 | | | |
| | sulfamethoxypyrazine | 500 | | | |
| | sulfamethoxypyrazine | 500 | | | |
| | sulfamethoxypyrazine | 500 | | | |
| | sulfamethoxypyrazine | 500 | | | |
| | sulfamethoxypyrazine | 500 | | | |
| | sulfamethoxypyrazine | 500 | | | |
| | sulfamethoxypyrazine | 500 | | | |
| | sulfamethoxypyrazine | 500 | | | |
| | sulfamethoxypyrazine | 500 | | | |
| | sulfamethoxypyrazine | 500 | | | |
| | sulfamethoxypyrazine | 250 | | | |
| | sulfamethoxypyrazine | 500 | | | |
| 80.9 | | | | | |
| | amodiaquine | 300 | 0.4941 | 12.69 | 4.86 |
| | amodiaquine | 300 | 0.4908 | 12.71 | 4.84 |
| | amodiaquine | 300 | 0.5034 | 12.69 | 4.84 |
| | piperaquine | 225 | | | |
| | piperaquine | 225 | | | |
| | piperaquine | 225 | | | |
| | piperaquine | 225 | | | |
| | piperaquine | 225 | | | |
| | piperaquine | 225 | | | |
| | piperaquine | 225 | | | |
| | piperaquine | 225 | | | |
| | piperaquine | 225 | | | |
| | piperaquine | 225 | | | |
| 88.2 | piperaquine | 225 | | | |

| | | |
|---|---|---|
| 94.7 | piperaquine | 225 |
| 82.1 | piperaquine | 225 |
| | piperaquine | 225 |
| | piperaquine | 225 |
| | piperaquine | 225 |
| | piperaquine | 225 |
| | piperaquine | 225 |
| | piperaquine | 225 |
| | piperaquine | 225 |
| | piperaquine | 225 |
| 85.0 | piperaquine | 225 |
| | piperaquine | 225 |
| 86.2 | piperaquine | 225 |
| | piperaquine | 225 |
| | piperaquine | 225 |
| | piperaquine | 225 |
| | piperaquine | 225 |
| | piperaquine | 225 |
| | piperaquine | 225 |
| | piperaquine | 225 |
| | piperaquine | 225 |
| | piperaquine | 225 |
| | piperaquine | 225 |
| | piperaquine | 225 |
| | piperaquine | 225 |
| | piperaquine | 225 |
| | piperaquine | 225 |
| | piperaquine | 225 |
| | piperaquine | 225 |
| | piperaquine | 225 |
| | piperaquine | 225 |
| **lot detectabl** | piperaquine | 225 |
| | piperaquine | 225 |
| | piperaquine | 225 |
| | piperaquine | 225 |
| | piperaquine | 225 |
| | piperaquine | 225 |
| **lot detectabl** | piperaquine | 225 |
| | piperaquine | 225 |
| | piperaquine | 225 |
| | piperaquine | 225 |
| | piperaquine | 225 |
| | piperaquine | 225 |
| | piperaquine | 225 |
| | piperaquine | 225 |
| | piperaquine | 225 |
| | piperaquine | 225 |
| | piperaquine | 225 |
| | piperaquine | 225 |
| 90.1 | piperaquine | 225 |

| | | |
|---|---|---|
| 91.6 | piperaquine | 225 |
| | piperaquine | 225 |
| | piperaquine | 225 |
| | piperaquine | 225 |
| | piperaquine | 225 |
| lot detectabl | piperaquine | 225 |
| 93.2 | piperaquine | 225 |
| | piperaquine | 225 |
| | piperaquine | 225 |
| | piperaquine | 225 |
| 88.6 | piperaquine | 225 |
| lot detectabl | piperaquine | 225 |
| | piperaquine | 225 |
| | piperaquine | 225 |
| | piperaquine | 225 |
| | piperaquine | 225 |
| 94.2 | piperaquine | 225 |
| | piperaquine | 225 |
| | piperaquine | 225 |
| | piperaquine | 225 |
| | piperaquine | 225 |
| | piperaquine | 225 |
| | piperaquine | 225 |
| | piperaquine | 225 |
| | piperaquine | 225 |
| | piperaquine | 225 |
| lot detectabl | piperaquine | 225 |
| | piperaquine | 225 |
| | piperaquine | 225 |
| | piperaquine | 225 |
| | piperaquine | 225 |
| | piperaquine | 225 |
| | piperaquine | 225 |
| | piperaquine | 225 |
| 85.7 | piperaquine | 225 |
| | piperaquine | 225 |
| | piperaquine | 225 |
| | piperaquine | 225 |
| | piperaquine | 225 |
| | piperaquine | 225 |
| | piperaquine | 225 |
| | piperaquine | 225 |
| 83.7 | piperaquine | 225 |
| | piperaquine | 225 |
| | piperaquine | 225 |
| | piperaquine | 225 |
| 95.1 | piperaquine | 225 |
| | piperaquine | 225 |
| | piperaquine | 225 |
| | piperaquine | 225 |

| | | |
|---|---|---|
| | piperaquine | 225 |
| | piperaquine | 225 |
| | piperaquine | 225 |
| | piperaquine | 225 |
| | piperaquine | 225 |
| lot detectabl | piperaquine | 225 |
| lot detectabl | piperaquine | 225 |
| | piperaquine | 225 |
| | piperaquine | 225 |
| lot detectabl | piperaquine | 225 |
| | piperaquine | 225 |
| lot detectabl | piperaquine | 225 |
| | piperaquine | 225 |
| | piperaquine | 225 |
| | piperaquine | 225 |
| 102.9 | piperaquine | 225 |
| | piperaquine | 225 |
| | piperaquine | 225 |
| | piperaquine | 225 |
| | piperaquine | 225 |
| | piperaquine | 225 |
| | piperaquine | 225 |
| | piperaquine | 225 |
| | piperaquine | 225 |
| | piperaquine | 225 |
| | piperaquine | 225 |
| | piperaquine | 225 |
| | piperaquine | 225 |
| | piperaquine | 225 |
| | piperaquine | 225 |
| | piperaquine | 225 |
| | piperaquine | 225 |
| | piperaquine | 225 |
| 88.6 | piperaquine | 225 |
| | piperaquine | 225 |
| | piperaquine | 225 |
| 89.4 | piperaquine | 225 |
| | piperaquine | 225 |
| | piperaquine | 320 |
| | piperaquine | 320 |
| | piperaquine | 320 |
| | piperaquine | 320 |
| | piperaquine | 320 |
| | piperaquine | 320 |
| 94.1 | piperaquine | 320 |
| | piperaquine | 320 |
| 89.9 | piperaquine | 320 |
| 88.3 | piperaquine | 320 |
| | piperaquine | 320 |
| | piperaquine | 320 |

| | | | |
|---|---|---|---|
| | piperaquine | 320 | |
| 109.2 | piperaquine | | 320 |
| | piperaquine | 320 | |
| | piperaquine | 320 | |
| | piperaquine | 320 | |
| | piperaquine | 320 | |
| | piperaquine | 320 | |
| | piperaquine | 320 | |
| | piperaquine | 320 | |
| | piperaquine | 320 | |
| | piperaquine | 320 | |
| | piperaquine | 320 | |
| | piperaquine | 320 | |
| | piperaquine | 320 | |
| | piperaquine | 320 | |
| | piperaquine | 320 | |
| 103.0 | piperaquine | 320 | |
| | piperaquine | 320 | |
| | piperaquine | 320 | |
| | piperaquine | 320 | |
| | piperaquine | 320 | |
| | piperaquine | 320 | |
| | piperaquine | 320 | |
| | piperaquine | 320 | |
| | piperaquine | 320 | |
| | piperaquine | 320 | |
| | piperaquine | 320 | |
| | piperaquine | 320 | |
| | piperaquine | 320 | |
| | piperaquine | 320 | |
| | piperaquine | 320 | |
| | piperaquine | 320 | |
| | piperaquine | 320 | |
| | piperaquine | 320 | |
| | piperaquine | 320 | |
| 102.7 | piperaquine | 320 | |
| | piperaquine | 320 | |
| | piperaquine | 320 | |
| | piperaquine | 320 | |
| | piperaquine | 320 | |
| | piperaquine | 320 | |
| | piperaquine | 320 | |
| | piperaquine | 320 | |
| 83.0 | piperaquine | 320 | |
| 74.4 | piperaquine | 320 | |
| | piperaquine | 320 | |
| | piperaquine | 320 | |
| | piperaquine | 320 | |
| | piperaquine | 320 | |
| 92.7 | piperaquine | 320 | |

| | | |
|---|---|---|
| | piperaquine | 320 |
| | piperaquine | 320 |
| | piperaquine | 320 |
| 101.5 | piperaquine | 320 |
| | piperaquine | 320 |
| 93.6 | piperaquine | 320 |
| 92.3 | piperaquine | 320 |
| | piperaquine | 320 |
| | piperaquine | 320 |
| | piperaquine | 320 |
| | piperaquine | 320 |
| | piperaquine | 320 |
| | piperaquine | 320 |
| | piperaquine | 320 |
| | piperaquine | 320 |
| | piperaquine | 320 |
| 79.7 | piperaquine | 320 |
| | piperaquine | 320 |
| | piperaquine | 320 |
| | piperaquine | 320 |
| | piperaquine | 320 |
| | piperaquine | 320 |
| | piperaquine | 320 |
| | piperaquine | 320 |
| | piperaquine | 320 |
| | piperaquine | 320 |
| | piperaquine | 320 |
| | piperaquine | 320 |
| | piperaquine | 320 |
| | piperaquine | 320 |
| | piperaquine | 320 |
| | piperaquine | 320 |
| | piperaquine | 320 |
| | piperaquine | 320 |
| | piperaquine | 320 |
| | piperaquine | 320 |
| | piperaquine | 320 |
| | piperaquine | 320 |
| | piperaquine | 320 |
| | piperaquine | 320 |
| | piperaquine | 320 |
| | piperaquine | 320 |
| | piperaquine | 320 |
| | piperaquine | 320 |
| | piperaquine | 320 |
| | piperaquine | 320 |
| | piperaquine | 320 |
| | piperaquine | 320 |
| | piperaquine | 320 |
| | piperaquine | 320 |

| | | | |
|---|---|---|---|
| 65.5 | piperaquine | 320 | |
| | piperaquine | | 320 |
| | piperaquine | 320 | |
| | piperaquine | 320 | |
| | piperaquine | 320 | |
| | piperaquine | 320 | |
| | piperaquine | 320 | |
| | piperaquine | 320 | |
| | piperaquine | 320 | |
| | piperaquine | 320 | |
| 89.5 | piperaquine | 320 | |
| | piperaquine | 320 | |
| | piperaquine | 320 | |
| | piperaquine | 320 | |
| | piperaquine | 320 | |
| | piperaquine | 320 | |
| | piperaquine | 320 | |
| | piperaquine | 320 | |
| | piperaquine | 320 | |
| | piperaquine | 320 | |
| | piperaquine | 320 | |
| | lumefantrine | | 1080 |
| CDC | lumefantrine | | 1080 |
| | lumefantrine | | 1080 |
| | lumefantrine | | 1080 |
| | lumefantrine | | 1080 |
| | lumefantrine | | 1080 |
| | lumefantrine | | 1080 |
| | lumefantrine | | 1080 |
| CDC | lumefantrine | | 1080 |
| | lumefantrine | | 1080 |
| | lumefantrine | 1080 | |
| | lumefantrine | 1080 | |
| CDC | lumefantrine | 1080 | |
| CDC | lumefantrine | 1080 | |
| | lumefantrine | 1080 | |
| | lumefantrine | 1080 | |
| | lumefantrine | 1080 | |
| | lumefantrine | 1080 | |
| CDC | lumefantrine | 1080 | |
| CDC | lumefantrine | 1080 | |
| | lumefantrine | 1080 | |
| CDC | lumefantrine | 1080 | |
| CDC | lumefantrine | 1080 | |
| | lumefantrine | 1080 | |
| | lumefantrine | 1080 | |
| | lumefantrine | 1080 | |
| | lumefantrine | 1080 | |
| CDC | lumefantrine | 1080 | |
| CDC | lumefantrine | 1080 | |

| | | | |
|---|---|---|---|
| | lumefantrine | 1080 | |
| | lumefantrine | 180 | |
| CDC | lumefantrine | 1080 | |
| CDC | lumefantrine | 1080 | |
| | lumefantrine | 1080 | |
| | lumefantrine | 1080 | |
| | lumefantrine | 1080 | |
| | lumefantrine | 1080 | |
| | lumefantrine | | 1080 |
| CDC | lumefantrine | 1080 | |
| CDC | lumefantrine | 1080 | |
| | lumefantrine | 1080 | |
| | lumefantrine | 1080 | |
| | lumefantrine | 1080 | |
| CDC | lumefantrine | 1080 | |
| CDC | lumefantrine | 1080 | |
| | lumefantrine | 1080 | |
| | lumefantrine | 1080 | |
| | lumefantrine | 1080 | |
| | lumefantrine | 1080 | |
| | lumefantrine | 1080 | |
| | lumefantrine | 1080 | |
| | lumefantrine | 90 | |
| | lumefantrine | 1080 | |
| | lumefantrine | 1440 | |
| | lumefantrine | 1440 | |
| | lumefantrine | 1440 | |
| | lumefantrine | 1440 | |
| | lumefantrine | 1440 | |
| | lumefantrine | 1440 | |
| | lumefantrine | 1440 | |
| | lumefantrine | 1440 | |
| | lumefantrine | 1440 | |
| | lumefantrine | 1440 | |
| | lumefantrine | 1440 | |
| | lumefantrine | 1440 | |
| | lumefantrine | 1440 | |
| | lumefantrine | 1440 | |
| | lumefantrine | 1440 | |
| | lumefantrine | 1440 | |
| CDC | lumefantrine | 1440 | |
| CDC | lumefantrine | 1440 | |
| CDC | lumefantrine | 1440 | |
| CDC | lumefantrine | 1440 | |
| CDC | lumefantrine | 1440 | |
| CDC | lumefantrine | 1440 | |
| CDC | lumefantrine | 1440 | |
| CDC | lumefantrine | 1440 | |
| CDC | lumefantrine | 1440 | |
| CDC | lumefantrine | 1440 | |

| | | | |
|---|---|---|---|
| CDC | lumefantrine | 1440 | |
| CDC | lumefantrine | 1440 | |
| CDC | lumefantrine | 1440 | |
| CDC | lumefantrine | 1440 | |
| CDC | lumefantrine | 1440 | |
| CDC | lumefantrine | 1440 | |
| CDC | lumefantrine | 1440 | |
| | lumefantrine | 1440 | |
| | lumefantrine | 1440 | |
| | lumefantrine | 1440 | |
| | lumefantrine | 1440 | |
| CDC | lumefantrine | 1440 | |
| CDC | lumefantrine | | 1440 |
| | lumefantrine | 240 | |
| | lumefantrine | 240 | |
| | lumefantrine | 240 | |
| | lumefantrine | 240 | |
| | lumefantrine | 240 | |
| | lumefantrine | 480 | |
| | lumefantrine | 240 | |
| | lumefantrine | 240 | |
| | lumefantrine | 120 | |
| | lumefantrine | 120 | |
| | lumefantrine | 120 | |
| CDC | | | |
| CDC | | | |
| CDC | | | |
| CDC | | | |
| CDC | | | |
| CDC | | | |
| CDC | | | |
| CDC | | | |
| CDC | amodiaquine | | 612.4 |
| | amodiaquine | | 612.4 |
| | piperaquine | 640 | |
| | piperaquine | 640 | |
| | piperaquine | 640 | |
| CDC | piperaquine | 640 | |
| | piperaquine | 640 | |
| | piperaquine | 640 | |
| CDC | piperaquine | 640 | |
| | piperaquine | 640 | |

| | | | |
|---|---|---|---|
| | piperaquine | 640 | |
| CDC | piperaquine | 640 | |
| | piperaquine | 640 | |
| CDC | piperaquine | 640 | |
| | piperaquine | 640 | |
| | piperaquine | 640 | |
| | piperaquine | 640 | |
| CDC | piperaquine | 640 | |
| | piperaquine | 640 | |
| CDC | piperaquine | 640 | |
| | piperaquine | 640 | |
| | piperaquine | 640 | |
| CDC | piperaquine | 640 | |
| | piperaquine | 640 | |
| CDC | piperaquine | 640 | |
| | piperaquine | 640 | |
| CDC | piperaquine | 640 | |
| | piperaquine | 640 | |
| CDC | piperaquine | | 640 |
| | piperaquine | 640 | |
| CDC | piperaquine | 640 | |
| | piperaquine | 640 | |
| CDC | piperaquine | 640 | |
| | piperaquine | 640 | |
| CDC | piperaquine | 640 | |
| | piperaquine | 640 | |
| CDC | piperaquine | 640 | |
| | piperaquine | 640 | |
| CDC | piperaquine | 640 | |
| | piperaquine | 640 | |
| CDC | piperaquine | | 640 |
| | piperaquine | 720 | |
| CDC | piperaquine | 720 | |
| | piperaquine | 720 | |
| CDC | piperaquine | 720 | |
| | piperaquine | 1920 | |
| | lumefantrine | 120 | |
| | lumefantrine | 120 | |
| 103.0 | lumefantrine | 120 | |
| | lumefantrine | 120 | |
| | lumefantrine | 120 | |
| | lumefantrine | 120 | |
| | lumefantrine | 120 | |
| | lumefantrine | 120 | |
| | lumefantrine | 120 | |
| | lumefantrine | 120 | |
| | lumefantrine | 120 | |
| | lumefantrine | 120 | |
| | lumefantrine | 120 | |
| | lumefantrine | 120 | |

| | | |
|---|---|---|
| | lumefantrine | 120 |
| | lumefantrine | 120 |
| 108.0 | lumefantrine | 120 |
| | lumefantrine | 120 |
| | lumefantrine | 120 |
| | lumefantrine | 120 |
| | lumefantrine | 120 |
| | lumefantrine | 120 |
| 104.3 | lumefantrine | 120 |
| | lumefantrine | 120 |
| | lumefantrine | 120 |
| | lumefantrine | 120 |
| | lumefantrine | 120 |
| | lumefantrine | 120 |
| | lumefantrine | 120 |
| | lumefantrine | 120 |
| | lumefantrine | 120 |
| | lumefantrine | 120 |
| | lumefantrine | 120 |
| | lumefantrine | 120 |
| | lumefantrine | 120 |
| | lumefantrine | 120 |
| | lumefantrine | 120 |
| | lumefantrine | 120 |
| 111.2 | lumefantrine | 120 |
| | lumefantrine | 120 |
| | lumefantrine | 120 |
| | lumefantrine | 120 |
| 101.8 | lumefantrine | 120 |
| | lumefantrine | 120 |
| | lumefantrine | 120 |
| | lumefantrine | 120 |
| | lumefantrine | 120 |
| | lumefantrine | 120 |
| | lumefantrine | 120 |
| | lumefantrine | 120 |
| | lumefantrine | 120 |
| | lumefantrine | 120 |
| 102.9 | lumefantrine | 120 |
| | lumefantrine | 120 |
| 91.1 | lumefantrine | 120 |
| | lumefantrine | 120 |
| 98.0 | lumefantrine | 120 |
| | lumefantrine | 120 |
| | lumefantrine | 120 |
| | lumefantrine | 120 |
| | lumefantrine | 120 |
| | lumefantrine | 120 |
| | lumefantrine | 120 |
| | lumefantrine | 120 |

| | | |
|---|---|---|
| | lumefantrine | 120 |
| | lumefantrine | 120 |
| | lumefantrine | 120 |
| | lumefantrine | 120 |
| | lumefantrine | 120 |
| | lumefantrine | 120 |
| | lumefantrine | 120 |
| | lumefantrine | 120 |
| | lumefantrine | 120 |
| | lumefantrine | 120 |
| | lumefantrine | 120 |
| | lumefantrine | 120 |
| | lumefantrine | 120 |
| | lumefantrine | 120 |
| | lumefantrine | 120 |
| | lumefantrine | 120 |
| | lumefantrine | 120 |
| | lumefantrine | 120 |
| | lumefantrine | 120 |
| | lumefantrine | 120 |
| | lumefantrine | 120 |
| | lumefantrine | 120 |
| | lumefantrine | 120 |
| | lumefantrine | 120 |
| | lumefantrine | 120 |
| | lumefantrine | 120 |
| | lumefantrine | 120 |
| | lumefantrine | 120 |
| | lumefantrine | 120 |
| | lumefantrine | 120 |
| | lumefantrine | 120 |
| 112.1 | lumefantrine | 120 |
| | lumefantrine | 120 |
| 109.1 | lumefantrine | 120 |
| | lumefantrine | 120 |
| | lumefantrine | 120 |
| | lumefantrine | 120 |
| | lumefantrine | 120 |
| | lumefantrine | 120 |
| | lumefantrine | 120 |
| | lumefantrine | 120 |
| | lumefantrine | 120 |
| | lumefantrine | 120 |
| | lumefantrine | 120 |
| | lumefantrine | 120 |
| | lumefantrine | 120 |
| | lumefantrine | 120 |
| | lumefantrine | 120 |
| | lumefantrine | 120 |
| | lumefantrine | 120 |

| | | |
|---|---|---|
| | lumefantrine | 120 |
| 0.0 | lumefantrine | 120 |
| | lumefantrine | 120 |
| | lumefantrine | 120 |
| | lumefantrine | 120 |
| | lumefantrine | 120 |
| | lumefantrine | 120 |
| | lumefantrine | 120 |
| lot detectabl | lumefantrine | 120 |
| lot detectabl | lumefantrine | 120 |
| lot detectabl | lumefantrine | 120 |
| lot detectabl | lumefantrine | 120 |
| 117.8 | lumefantrine | 120 |
| 111.9 | lumefantrine | 120 |
| | lumefantrine | 120 |
| | lumefantrine | 120 |
| | lumefantrine | 120 |
| | lumefantrine | 120 |
| | lumefantrine | 120 |
| | lumefantrine | 120 |
| | lumefantrine | 120 |
| | lumefantrine | 120 |
| | lumefantrine | 120 |
| | lumefantrine | 120 |
| | lumefantrine | 120 |
| | lumefantrine | 120 |
| | lumefantrine | 120 |
| | lumefantrine | 120 |
| | lumefantrine | 120 |
| | lumefantrine | 120 |
| | lumefantrine | 120 |
| | lumefantrine | 120 |
| | lumefantrine | 120 |
| | lumefantrine | 120 |
| | lumefantrine | 120 |
| | lumefantrine | 120 |
| | lumefantrine | 120 |
| | lumefantrine | 120 |
| | lumefantrine | 120 |
| | lumefantrine | 120 |
| | lumefantrine | 120 |
| | lumefantrine | 120 |
| 112.8 | lumefantrine | 120 |
| | lumefantrine | 120 |
| | lumefantrine | 120 |
| | lumefantrine | 120 |
| | lumefantrine | 120 |
| | lumefantrine | 120 |
| | lumefantrine | 120 |
| | lumefantrine | 120 |
| | lumefantrine | 120 |
| | lumefantrine | 120 |

| | | |
|---|---|---|
| | lumefantrine | 120 |
| | lumefantrine | 120 |
| | lumefantrine | 120 |
| | lumefantrine | 120 |
| | lumefantrine | 120 |
| | lumefantrine | 120 |
| | lumefantrine | 120 |
| | lumefantrine | 120 |
| | lumefantrine | 120 |
| | lumefantrine | 120 |
| 100.5 | lumefantrine | 120 |
| | lumefantrine | 120 |
| | lumefantrine | 120 |
| 105.2 | lumefantrine | 120 |
| 100.9 | lumefantrine | 120 |
| | lumefantrine | 120 |
| | lumefantrine | 120 |
| | lumefantrine | 120 |
| | lumefantrine | 120 |
| | lumefantrine | 120 |
| | lumefantrine | 120 |
| | lumefantrine | 120 |
| | lumefantrine | 120 |
| | lumefantrine | 120 |
| | lumefantrine | 120 |
| | lumefantrine | 120 |
| | lumefantrine | 120 |
| | lumefantrine | 120 |
| | lumefantrine | 120 |
| | lumefantrine | 120 |
| | lumefantrine | 120 |
| | lumefantrine | 120 |
| | lumefantrine | 120 |
| | lumefantrine | 120 |
| | lumefantrine | 120 |
| | lumefantrine | 120 |
| 102.2 | lumefantrine | 120 |
| | lumefantrine | 120 |
| | lumefantrine | 120 |
| | lumefantrine | 120 |
| | lumefantrine | 120 |
| | lumefantrine | 120 |
| | lumefantrine | 120 |
| 96.6 | lumefantrine | 120 |
| 99.2 | lumefantrine | 120 |
| 111.8 | lumefantrine | 120 |
| 113.8 | lumefantrine | 120 |
| | lumefantrine | 120 |
| | lumefantrine | 120 |
| | lumefantrine | 120 |

| | | | |
|---|---|---|---|
| | lumefantrine | 120 | |
| | lumefantrine | 120 | |
| | lumefantrine | 120 | |
| | lumefantrine | 120 | |
| | lumefantrine | 120 | |
| | lumefantrine | 120 | |
| | lumefantrine | 120 | |
| | lumefantrine | 120 | |
| | lumefantrine | 120 | |
| | lumefantrine | 120 | |
| | lumefantrine | 120 | |
| | lumefantrine | 120 | |
| | lumefantrine | 120 | |
| | lumefantrine | 120 | |
| | lumefantrine | 120 | |
| | lumefantrine | 120 | |
| | lumefantrine | 120 | |
| | lumefantrine | 120 | |
| | lumefantrine | 120 | |
| | lumefantrine | 120 | |
| | lumefantrine | 120 | |
| | lumefantrine | 120 | |
| | lumefantrine | 120 | |
| | lumefantrine | 120 | |
| | lumefantrine | 120 | |
| | lumefantrine | 120 | |
| | lumefantrine | | 120 |
| | lumefantrine | 120 | |
| | lumefantrine | 120 | |
| | lumefantrine | 120 | |
| | lumefantrine | 120 | |
| 111.4 | lumefantrine | 120 | |
| | lumefantrine | 120 | |
| | lumefantrine | 120 | |
| | lumefantrine | 240 | |
| | lumefantrine | 240 | |
| | lumefantrine | 240 | |
| 91.4 | lumefantrine | 240 | |
| | lumefantrine | 240 | |
| | lumefantrine | 240 | |
| | lumefantrine | 240 | |
| | lumefantrine | 240 | |
| 95.9 | lumefantrine | 240 | |
| | lumefantrine | 240 | |
| | lumefantrine | 240 | |
| | lumefantrine | 240 | |
| | lumefantrine | 240 | |
| | lumefantrine | 240 | |
| | lumefantrine | 240 | |
| | lumefantrine | 240 | |

| | | | |
|---|---|---|---|
| | lumefantrine | 240 | |
| | lumefantrine | 240 | |
| | lumefantrine | 240 | |
| | lumefantrine | | 240 |
| | lumefantrine | 240 | |
| 103.7 | lumefantrine | 240 | |
| 102.5 | lumefantrine | 240 | |
| | lumefantrine | 240 | |
| | lumefantrine | 240 | |
| | lumefantrine | 240 | |
| | lumefantrine | 480 | |
| | lumefantrine | 480 | |
| | lumefantrine | 480 | |
| | lumefantrine | 480 | |
| | lumefantrine | 480 | |
| | lumefantrine | 480 | |
| | lumefantrine | 480 | |
| | lumefantrine | 480 | |
| | lumefantrine | 480 | |
| | lumefantrine | 480 | |
| | lumefantrine | 480 | |
| | lumefantrine | 480 | |
| | lumefantrine | 480 | |
| | lumefantrine | 480 | |
| | lumefantrine | 480 | |
| | lumefantrine | 480 | |
| | lumefantrine | 480 | |
| | lumefantrine | 480 | |
| | lumefantrine | 480 | |
| | lumefantrine | 480 | |
| | lumefantrine | 480 | |
| | lumefantrine | 480 | |
| | lumefantrine | 480 | |
| | lumefantrine | 480 | |
| | lumefantrine | 480 | |
| | lumefantrine | 480 | |
| | lumefantrine | 480 | |
| 93.9 | lumefantrine | 480 | |
| | lumefantrine | 480 | |
| | lumefantrine | 480 | |
| | lumefantrine | 480 | |
| | lumefantrine | 480 | |
| | lumefantrine | 480 | |
| | lumefantrine | 480 | |
| | lumefantrine | 480 | |
| | lumefantrine | 480 | |
| | lumefantrine | 480 | |
| | lumefantrine | 480 | |
| | lumefantrine | 480 | |

| | | |
|---|---|---|
| 84.8 | lumefantrine | 480 |
| | lumefantrine | 480 |
| | lumefantrine | 480 |
| | lumefantrine | 480 |
| | lumefantrine | 480 |
| 104.5 | lumefantrine | 480 |
| | lumefantrine | 480 |
| | lumefantrine | 480 |
| | lumefantrine | 480 |
| | lumefantrine | 480 |
| 95.3 | lumefantrine | 480 |
| | lumefantrine | 480 |
| 104.7 | lumefantrine | 480 |
| | lumefantrine | 480 |
| | lumefantrine | 480 |
| | lumefantrine | 480 |
| | lumefantrine | 480 |
| | lumefantrine | 480 |
| | lumefantrine | 480 |
| 91.4 | lumefantrine | 480 |
| | lumefantrine | 480 |
| | lumefantrine | 480 |
| | lumefantrine | 480 |
| | lumefantrine | 480 |
| | lumefantrine | 480 |
| | lumefantrine | 480 |
| | lumefantrine | 480 |
| 86.1 | lumefantrine | 480 |
| | lumefantrine | 480 |
| | lumefantrine | 480 |
| | lumefantrine | 480 |
| | lumefantrine | 480 |
| | lumefantrine | 480 |
| | lumefantrine | 480 |
| | lumefantrine | 480 |
| | lumefantrine | 480 |
| | lumefantrine | 480 |
| | lumefantrine | 480 |
| | lumefantrine | 480 |
| | lumefantrine | 480 |
| | lumefantrine | 480 |
| | lumefantrine | 480 |
| | lumefantrine | 480 |
| | lumefantrine | 480 |
| | lumefantrine | 480 |
| | lumefantrine | 480 |
| | lumefantrine | 480 |
| | lumefantrine | 480 |
| | lumefantrine | 480 |
| | lumefantrine | 480 |

| | | |
|---|---|---|
| | lumefantrine | 480 |
| | lumefantrine | 480 |
| | lumefantrine | 480 |
| | lumefantrine | 480 |
| | lumefantrine | 480 |
| | lumefantrine | 480 |
| | lumefantrine | 480 |
| | lumefantrine | 480 |
| | lumefantrine | 480 |
| | lumefantrine | 480 |
| | lumefantrine | 480 |
| | lumefantrine | 480 |
| | lumefantrine | 480 |
| 105.8 | lumefantrine | 480 |
| | lumefantrine | 480 |
| | lumefantrine | 480 |
| | lumefantrine | 480 |
| | lumefantrine | 480 |
| | lumefantrine | 480 |
| | lumefantrine | 480 |
| | lumefantrine | 480 |
| | lumefantrine | 480 |
| | lumefantrine | 480 |
| | lumefantrine | 480 |
| | lumefantrine | 480 |
| | lumefantrine | 480 |
| | lumefantrine | 480 |
| | lumefantrine | 480 |
| | lumefantrine | 480 |
| | lumefantrine | 480 |
| | lumefantrine | 480 |
| | lumefantrine | 480 |
| | lumefantrine | 480 |
| | lumefantrine | 480 |
| | lumefantrine | 480 |
| | lumefantrine | 480 |
| | lumefantrine | 480 |
| | lumefantrine | 480 |
| | lumefantrine | 480 |
| | lumefantrine | 480 |
| | lumefantrine | 480 |
| | lumefantrine | 480 |
| 98.0 | lumefantrine | 480 |
| | lumefantrine | 480 |
| 98.9 | lumefantrine | 480 |
| 97.9 | lumefantrine | 480 |
| | lumefantrine | 480 |
| 97.0 | lumefantrine | 480 |
| | lumefantrine | 480 |
| | lumefantrine | 480 |

| | | |
|---|---|---|
| | lumefantrine | 480 |
| | lumefantrine | 480 |
| | lumefantrine | 480 |
| | lumefantrine | 480 |
| 97.6 | lumefantrine | 480 |
| 98.8 | lumefantrine | 480 |
| | lumefantrine | 480 |
| | lumefantrine | 480 |
| | lumefantrine | 480 |
| | lumefantrine | 480 |
| | lumefantrine | 480 |
| | lumefantrine | 480 |
| | lumefantrine | 480 |
| | lumefantrine | 480 |
| 99.4 | lumefantrine | 480 |
| | lumefantrine | 480 |
| | lumefantrine | 480 |
| | lumefantrine | 480 |
| | lumefantrine | 480 |
| 111.5 | lumefantrine | 480 |
| | lumefantrine | 480 |
| | lumefantrine | 480 |
| | lumefantrine | 480 |
| | lumefantrine | 480 |
| | lumefantrine | 480 |
| | lumefantrine | 480 |
| | lumefantrine | 480 |
| | lumefantrine | 480 |
| | lumefantrine | 480 |
| | lumefantrine | 480 |
| | lumefantrine | 480 |
| | lumefantrine | 480 |
| | lumefantrine | 480 |
| | lumefantrine | 480 |
| | lumefantrine | 480 |
| | lumefantrine | 480 |
| | lumefantrine | 480 |
| | lumefantrine | 480 |
| | lumefantrine | 480 |
| | lumefantrine | 480 |
| | lumefantrine | 480 |
| | lumefantrine | 480 |
| | lumefantrine | 480 |
| | lumefantrine | 480 |
| | lumefantrine | 480 |
| | lumefantrine | 480 |
| | lumefantrine | 480 |
| | lumefantrine | 480 |
| | lumefantrine | 480 |
| | lumefantrine | 480 |

| | | |
|---|---|---|
| | lumefantrine | 480 |
| | lumefantrine | 480 |
| | lumefantrine | 480 |
| | lumefantrine | 480 |
| | lumefantrine | 480 |
| | lumefantrine | 480 |
| | lumefantrine | 480 |
| | lumefantrine | 480 |
| 97.4 | lumefantrine | 480 |
| | lumefantrine | 480 |
| | lumefantrine | 480 |
| | lumefantrine | 480 |
| | lumefantrine | 480 |
| | lumefantrine | 480 |
| | lumefantrine | 480 |
| | lumefantrine | 480 |
| | lumefantrine | 480 |
| | lumefantrine | 480 |
| | lumefantrine | 480 |
| | lumefantrine | 480 |
| | lumefantrine | 480 |
| 98.0 | lumefantrine | 480 |
| | lumefantrine | 480 |
| 97.0 | lumefantrine | 480 |
| | lumefantrine | 480 |
| | lumefantrine | 480 |
| | lumefantrine | 480 |
| | lumefantrine | 480 |
| | lumefantrine | 480 |
| | lumefantrine | 480 |
| 101.3 | lumefantrine | 480 |
| | lumefantrine | 480 |
| 101.6 | lumefantrine | 480 |
| | lumefantrine | 480 |
| | lumefantrine | 480 |
| | lumefantrine | 480 |
| | lumefantrine | 480 |
| 87.7 | lumefantrine | 480 |
| 88.5 | lumefantrine | 480 |
| | lumefantrine | 480 |
| | lumefantrine | 480 |
| | lumefantrine | 480 |
| | lumefantrine | 480 |
| | lumefantrine | 480 |
| | lumefantrine | 480 |
| | lumefantrine | 480 |
| 93.6 | lumefantrine | 480 |
| | lumefantrine | 480 |

91.0

89.0

| | |
|---|---|
| amodiaquine | 135 |
| amodiaquine | 135 |
| amodiaquine | 135 |
| amodiaquine | 150 |
| amodiaquine | 150 |
| amodiaquine | 150 |
| amodiaquine | 150 |
| amodiaquine | 150 |
| amodiaquine | 150 |
| amodiaquine | 150 |
| amodiaquine | 150 |

| | | | | | | |
|---|---|---|---|---|---|---|
| | amodiaquine | 150 | | | | |
| | amodiaquine | 150 | | | | |
| | amodiaquine | 150 | | | | |
| | amodiaquine | 153 | | | | |
| | amodiaquine | 270 | | | | |
| 101.4 | amodiaquine | 270 | | | | |
| | amodiaquine | 270 | | | | |
| | amodiaquine | 270 | | | | |
| | amodiaquine | 270 | | | | |
| 96.2 | amodiaquine | 270 | | | | |
| | amodiaquine | 270 | | | | |
| | amodiaquine | 270 | | | | |
| | amodiaquine | 270 | | | | |
| | amodiaquine | 270 | | | | |
| | amodiaquine | 270 | | | | |
| | amodiaquine | 270 | | | | |
| | amodiaquine | 270 | | | | |
| | amodiaquine | 270 | | | | |
| | amodiaquine | 270 | | | | |
| | amodiaquine | 270 | | | | |
| | amodiaquine | 270 | | | | |
| | amodiaquine | 270 | | | | |
| | amodiaquine | 270 | | | | |
| 109.8 | amodiaquine | 270 | | | | |
| | amodiaquine | 270 | | | | |
| | amodiaquine | 270 | | | | |
| | amodiaquine | 270 | | | | |
| | amodiaquine | 270 | | | | |
| | amodiaquine | 270 | | | | |
| 103.5 | amodiaquine | 270 | | | | |
| | amodiaquine | 270 | | | | |
| | amodiaquine | 270 | | | | |
| | amodiaquine | | 300 | 0.5198 | 12.69 | 4.76 |
| | amodiaquine | | 300 | 0.5128 | 12.84 | 4.78 |
| | amodiaquine | | 300 | 0.5016 | 12.74 | 4.81 |
| | amodiaquine | 300 | | 0.5108 | 12.69 | 4.73 |
| | amodiaquine | 300 | | 0.5076 | 12.66 | 4.88 |
| 102.0 | amodiaquine | 300 | | 0.5078 | 12.75 | 4.73 |
| | amodiaquine | 300 | | 0.5108 | 12.73 | 4.74 |
| | amodiaquine | 300 | | 0.5166 | 12.7 | 4.75 |
| | amodiaquine | 300 | | | | |
| | amodiaquine | 300 | | | | |
| | amodiaquine | 300 | | | | |
| | amodiaquine | 300 | | | | |
| 98.0 | amodiaquine | 300 | | | | |
| 94.9 | amodiaquine | 300 | | | | |
| | amodiaquine | 300 | | | | |
| | amodiaquine | 300 | | | | |
| 93.9 | amodiaquine | 300 | | | | |
| | amodiaquine | 300 | | | | |

| | | | | | |
|---|---|---|---|---|---|
| | amodiaquine | 300 | | | |
| | amodiaquine | 300 | 0.5094 | 12.59 | 4.87 |
| | amodiaquine | 300 | 0.5062 | 12.56 | 4.83 |
| | amodiaquine | 300 | 0.5166 | 12.58 | 4.92 |
| | amodiaquine | 300 | 0.5036 | 12.65 | 4.67 |
| | amodiaquine | 300 | 0.5211 | 12.56 | 4.88 |
| | amodiaquine | 300 | 0.568 | 18.42 | 8.13 |
| | amodiaquine | 300 | 0.5802 | 18.34 | 8.12 |
| | amodiaquine | 780 | 0.8553 | 19.12 | 9.08 |
| 93.0 | amodiaquine | 780 | 0.8614 | 19.12 | 9.07 |
| 90.0 | amodiaquine | 780 | 0.8549 | 19.13 | 9.08 |
| | mefloquine | 125 | 0.2683 | 9.6 | 4.02 |
| | mefloquine | 125 | 0.2060 | 9.59 | 4.2 |
| | mefloquine | 125 | 0.2744 | 9.62 | 4.2 |
| | mefloquine | 125 | 0.2668 | 9.59 | 4.08 |
| | mefloquine | 125 | 0.2661 | 9.57 | 4.17 |
| | mefloquine | 250 | 0.5333 | 12.07 | 4.62 |
| | mefloquine | 250 | 0.5400 | 12.07 | 4.69 |
| | mefloquine | 250 | 0.5318 | 12.07 | 4.62 |
| | mefloquine | 250 | 0.5347 | 12.14 | 4.69 |
| | mefloquine | 250 | 0.5260 | 12.12 | 4.6 |
| | mefloquine | 250 | | | |
| | mefloquine | 250 | | | |
| | mefloquine | 250 | | | |
| | mefloquine | 250 | | | |
| | mefloquine | 250 | | | |
| | mefloquine | 250 | | | |
| | mefloquine | 250 | | | |
| | mefloquine | 250 | | | |
| | mefloquine | 250 | | | |
| | mefloquine | 250 | | | |
| | mefloquine | 250 | | | |
| | mefloquine | 250 | | | |
| | mefloquine | 250 | | | |
| | mefloquine | 250 | | | |
| | mefloquine | 275 | 0.9594 | 19.14 | 9.09 |
| | sulfadoxine | 500 | 0.7044 | 13.08 | 4.31 |
| | sulfadoxine | 500 | 0.6587 | 19.11 | 7.05 |
| | sulfadoxine | 500 | 0.667 | 12.57 | 4.21 |
| | sulfadoxine | 500 | 0.6661 | 12.59 | 4.22 |
| | sulfamethoxypyrazine | 250 | | | |
| | sulfamethoxypyrazine | 250 | | | |
| | sulfamethoxypyrazine | 250 | | | |
| | sulfamethoxypyrazine | 250 | | | |
| | sulfamethoxypyrazine | 250 | | | |
| | sulfamethoxypyrazine | 250 | | | |
| | sulfamethoxypyrazine | 500 | | | |
| | sulfamethoxypyrazine | 500 | | | |
| | sulfamethoxypyrazine | 500 | | | |
| | sulfamethoxypyrazine | 500 | | | |

| | | |
|---|---|---|
| | sulfamethoxypyrazine | 500 |
| | sulfamethoxypyrazine | 500 |
| | sulfamethoxypyrazine | 500 |
| | sulfamethoxypyrazine | 500 |
| | sulfamethoxypyrazine | 500 |
| | sulfamethoxypyrazine | 500 |
| | sulfamethoxypyrazine | 500 |
| | sulfamethoxypyrazine | 500 |
| | sulfamethoxypyrazine | 500 |
| | sulfamethoxypyrazine | 500 |
| | sulfamethoxypyrazine | 500 |
| | | |
| | piperaquine phosphate | 225 |
| | piperaquine phosphate | 225 |
| 92.6 | piperaquine phosphate | 225 |
| | piperaquine phosphate | 225 |
| 89.5 | piperaquine phosphate | 225 |
| | piperaquine phosphate | 225 |
| | piperaquine phosphate | 225 |
| 0.0 | piperaquine phosphate | 225 |
| | piperaquine phosphate | 225 |
| | piperaquine phosphate | 225 |
| | piperaquine phosphate | 225 |
| | piperaquine phosphate | 225 |
| | piperaquine phosphate | 225 |
| | piperaquine phosphate | 225 |
| | piperaquine phosphate | 225 |
| | piperaquine phosphate | 225 |
| | piperaquine phosphate | 225 |
| | piperaquine phosphate | 225 |
| | piperaquine phosphate | 225 |
| | piperaquine phosphate | 225 |
| | piperaquine phosphate | 225 |
| | piperaquine phosphate | 225 |
| 0.0 | piperaquine phosphate | 225 |
| | piperaquine phosphate | 225 |
| 0.0 | piperaquine phosphate | 225 |
| | piperaquine phosphate | 225 |
| | piperaquine phosphate | 225 |
| | piperaquine phosphate | 225 |
| | piperaquine phosphate | 225 |
| | piperaquine phosphate | 225 |
| | piperaquine phosphate | 225 |
| | piperaquine phosphate | 225 |
| | piperaquine phosphate | 225 |
| 0.0 | piperaquine phosphate | 225 |
| | piperaquine phosphate | 225 |
| | piperaquine phosphate | 225 |
| | piperaquine phosphate | 225 |
| | piperaquine phosphate | 225 |

| | | |
|---|---|---|
| | piperaquine phosphate | 225 |
| 94.6 | piperaquine phosphate | 225 |
| 90.6 | piperaquine phosphate | 225 |
| | piperaquine phosphate | 225 |
| | piperaquine phosphate | 225 |
| | piperaquine phosphate | 225 |
| | piperaquine phosphate | 225 |
| | piperaquine phosphate | 225 |
| | piperaquine phosphate | 225 |
| | piperaquine phosphate | 225 |
| | piperaquine phosphate | 225 |
| | piperaquine phosphate | 225 |
| | piperaquine phosphate | 225 |
| | piperaquine phosphate | 225 |
| | piperaquine phosphate | 225 |
| | piperaquine phosphate | 225 |
| 80.9 | piperaquine phosphate | 225 |
| 94.1 | piperaquine phosphate | 225 |
| | piperaquine phosphate | 225 |
| | piperaquine phosphate | 225 |
| | piperaquine phosphate | 225 |
| | piperaquine phosphate | 225 |
| | piperaquine phosphate | 225 |
| 93.4 | piperaquine phosphate | 225 |
| | piperaquine phosphate | 225 |
| | piperaquine phosphate | 225 |
| | piperaquine phosphate | 225 |
| | piperaquine phosphate | 225 |
| | piperaquine phosphate | 225 |
| | piperaquine phosphate | 225 |
| | piperaquine phosphate | 225 |
| | piperaquine | 320 |
| | piperaquine | 320 |
| | piperaquine phosphate | 320 |
| | piperaquine phosphate | 320 |
| 83.7 | piperaquine | 320 |
| | piperaquine | 320 |
| 111.5 | piperaquine | 320 |
| | piperaquine | 320 |
| 97.5 | piperaquine | 320 |
| | piperaquine | 320 |
| | piperaquine | 320 |
| | piperaquine phosphate | 320 |
| | piperaquine phosphate | 320 |
| | piperaquine phosphate | 320 |
| | piperaquine phosphate | 320 |
| | piperaquine phosphate | 320 |
| | piperaquine phosphate | 320 |
| | piperaquine phosphate | 320 |
| | piperaquine phosphate | 320 |

| | |
|---|---|
| piperaquine phosphate | 320 |
| piperaquine phosphate | 320 |
| piperaquine phosphate | 320 |
| piperaquine phosphate | 320 |
| piperaquine phosphate | 320 |
| piperaquine phosphate | 320 |
| piperaquine phosphate | 320 |
| piperaquine phosphate | 320 |
| piperaquine phosphate | 320 |
| piperaquine phosphate | 320 |
| piperaquine phosphate | 320 |
| piperaquine phosphate | 320 |
| piperaquine phosphate | 320 |
| piperaquine phosphate | 320 |
| piperaquine phosphate | 320 |
| piperaquine phosphate | 320 |
| piperaquine phosphate | 320 |
| piperaquine phosphate | 320 |
| piperaquine phosphate | 320 |
| piperaquine phosphate | 320 |
| piperaquine phosphate | 320 |
| piperaquine phosphate | 320 |
| piperaquine phosphate | 320 |
| piperaquine phosphate | 320 |
| piperaquine phosphate | 320 |
| piperaquine phosphate | 320 |
| piperaquine phosphate | 320 |
| piperaquine phosphate | 320 |
| piperaquine phosphate | 320 |
| piperaquine phosphate | 320 |
| piperaquine phosphate | 320 |
| piperaquine phosphate | 320 |
| piperaquine phosphate | 320 |
| piperaquine phosphate | 320 |
| piperaquine phosphate | 320 |
| piperaquine phosphate | 320 |
| piperaquine phosphate | 320 |
| piperaquine phosphate | 320 |
| piperaquine phosphate | 320 |
| piperaquine phosphate | 320 |
| piperaquine phosphate | 320 |
| piperaquine phosphate | 320 |
| piperaquine phosphate | 320 |
| piperaquine phosphate | 320 |
| lumefantrine | 240 |
| lumefantrine | 240 |
| lumefantrine | 240 |
| lumefantrine | 120 |
| lumefantrine | 1080 |
| lumefantrine | 1080 |

| | | | |
|---|---|---|---|
| CDC | lumefantrine | 1080 | |
| | lumefantrine | 1080 | |
| CDC | lumefantrine | 1080 | |
| | lumefantrine | 1080 | |
| CDC | lumefantrine | 1080 | |
| | lumefantrine | 1080 | |
| | lumefantrine | 1080 | |
| CDC | lumefantrine | 1080 | |
| | lumefantrine | | 1080 |
| | lumefantrine | | 1080 |
| CDC | lumefantrine | 1080 | |
| | lumefantrine | 1080 | |
| | lumefantrine | 1080 | |
| | lumefantrine | 1080 | |
| CDC | lumefantrine | 1080 | |
| | lumefantrine | 1080 | |
| CDC | lumefantrine | | 1080 |
| | lumefantrine | | 1080 |
| CDC | lumefantrine | | 1080 |
| | lumefantrine | | 1080 |
| | lumefantrine | | 1080 |
| CDC | lumefantrine | | 1080 |
| | lumefantrine | | 1080 |
| CDC | lumefantrine | | 1080 |
| | lumefantrine | | 1080 |
| CDC | lumefantrine | | 1080 |
| | lumefantrine | | 1080 |
| CDC | lumefantrine | | 1080 |
| | lumefantrine | | 1080 |
| | lumefantrine | | 1080 |
| | lumefantrine | 1080 | |
| | lumefantrine | 1080 | |
| CDC | lumefantrine | 1080 | |
| | lumefantrine | 1080 | |
| CDC | lumefantrine | 1080 | |
| | lumefantrine | 1080 | |
| | lumefantrine | 1080 | |
| | lumefantrine | 1080 | |
| CDC | lumefantrine | 1080 | |
| | lumefantrine | 1080 | |
| CDC | lumefantrine | 1080 | |
| | lumefantrine | | 1080 |
| | lumefantrine | 2160 | |
| | lumefantrine | 1440 | |
| | lumefantrine | 1440 | |
| | lumefantrine | 1440 | |
| CDC | lumefantrine | 1440 | |
| | lumefantrine | 1440 | |
| CDC | lumefantrine | 1440 | |
| | lumefantrine | 1440 | |

| | | | | |
|---|---|---|---|---|
| CDC | lumefantrine | 1440 | | |
| | lumefantrine | 1440 | | |
| CDC | lumefantrine | 1440 | | |
| | lumefantrine | 1440 | | |
| | lumefantrine | 1440 | | |
| | lumefantrine | 1440 | | |
| CDC | lumefantrine | 1440 | | |
| | lumefantrine | 1440 | | |
| | lumefantrine | 1440 | | |
| CDC | lumefantrine | 1440 | | |
| | lumefantrine | 1440 | | |
| CDC | lumefantrine | 1440 | | |
| | lumefantrine | 1440 | | |
| CDC | lumefantrine | 1440 | | |
| | lumefantrine | 1440 | | |
| CDC | lumefantrine | 1440 | | |
| | lumefantrine | 1440 | | |
| CDC | lumefantrine | 1440 | | |
| | lumefantrine | 1440 | | |
| CDC | lumefantrine | 1440 | | |
| | lumefantrine | 1440 | | |
| CDC | lumefantrine | 1440 | | |
| | lumefantrine | 1440 | | |
| CDC | lumefantrine | 1440 | | |
| | lumefantrine | 1440 | | |
| CDC | lumefantrine | 1440 | | |
| | lumefantrine | 1440 | | |
| CDC | lumefantrine | 1440 | | |
| | lumefantrine | 1440 | | |
| | amodiaquine | | 75 | 0.474 |
| | amodiaquine | 75 | | 0.4832 |
| | amodiaquine | 75 | | 0.5265 |
| | amodiaquine | 75 | | 0.516 |
| | amodiaquine | 150 | | 0.9376 |
| | amodiaquine | 150 | | 1.0417 |
| | amodiaquine | 150 | | 0.9444 |
| | amodiaquine | 150 | | 1.0215 |
| | amodiaquine | 150 | | 1.0089 |
| | amodiaquine | 150 | | |
| | amodiaquine | 150 | | |
| | amodiaquine | 150 | | |
| CDC | | | | |
| CDC | | | | |
| CDC | | | | |
| CDC | | | | |

| | | |
|---|---|---|
| CDC | | |
| CDC | | |
| CDC | | |
| CDC | | |
| CDC | | |
| | piperaquine phosphate | 640 |
| | piperaquine phosphate | 640 |
| | piperaquine phosphate | 640 |
| | piperaquine phosphate | 640 |
| CDC | piperaquine phosphate | 640 |
| | piperaquine phosphate | 640 |
| | piperaquine phosphate | 640 |
| CDC | piperaquine phosphate | 640 |
| | piperaquine phosphate | 640 |
| CDC | piperaquine phosphate | 640 |
| | piperaquine phosphate | 640 |
| CDC | piperaquine phosphate | 640 |
| | piperaquine phosphate | 640 |
| | piperaquine phosphate | 640 |
| | piperaquine phosphate | 640 |
| CDC | piperaquine phosphate | 640 |
| | piperaquine phosphate | 640 |
| CDC | piperaquine phosphate | 640 |
| | piperaquine phosphate | 640 |
| | piperaquine phosphate | 640 |
| CDC | piperaquine | 720 |
| | piperaquine | 720 |
| CDC | piperaquine | 720 |
| | piperaquine | 720 |

| manufbyother | manufcountry | licensor | fdc | amfm | batchno |
|---|---|---|---|---|---|
| | XXX | | y | n | XXXXX |
| | XXX | | y | n | XXXXX |
| | XXX | | y | n | XXXXX |
| | XXX | | y | n | XXXXX |
| | XXX | | y | n | XXXXX |
| | XXX | | y | n | XXXXX |
| | XXX | XXXX | y | y | XXXXX |
| | XXX | XXXX | y | n | XXXXX |
| | XXX | XXXX | y | n | XXXXX |
| | XXX | XXXX | y | n | XXXXX |
| | XXX | XXXX | y | n | XXXXX |
| | XXX | XXXX | y | n | XXXXX |
| | XXX | XXXX | y | n | XXXXX |
| | XXX | XXXX | y | n | XXXXX |
| | XXX | XXXX | y | n | XXXXX |
| | XXX | XXXX | y | n | XXXXX |
| | XXX | XXXX | y | n | XXXXX |
| | XXX | XXXX | y | n | XXXXX |
| | XXX | XXXX | y | n | XXXXX |
| | XXX | XXXX | y | y | XXXXX |
| | XXX | XXXX | y | y | XXXXX |
| | XXX | XXXX | y | y | XXXXX |
| | XXX | XXXX | y | y | XXXXX |
| | XXX | XXXX | y | y | XXXXX |
| | XXX | XXXX | y | y | XXXXX |
| | XXX | XXXX | y | y | XXXXX |
| | XXX | XXXX | y | y | XXXXX |
| | XXX | XXXX | y | y | XXXXX |
| | XXX | XXXX | y | y | XXXXX |
| | XXX | XXXX | y | y | XXXXX |
| | XXX | XXXX | y | y | XXXXX |
| | XXX | XXXX | y | y | XXXXX |
| | XXX | XXXX | y | y | XXXXX |
| | XXX | XXXX | y | y | XXXXX |
| | XXX | XXXX | y | y | XXXXX |
| | XXX | | y | y | XXXXX |
| | XXX | | y | n | XXXXX |
| | XXX | | y | y | XXXXX |
| | XXX | | y | y | XXXXX |
| | XXX | | y | y | XXXXX |
| | XXX | | y | y | XXXXX |
| | XXX | | y | y | XXXXX |
| | XXX | | y | y | XXXXX |
| | XXX | | y | y | XXXXX |
| | XXX | | y | y | XXXXX |
| | XXX | | y | y | XXXXX |

| | | | | |
|---|---|---|---|---|
| XXX | | y | n | XXXXX |
| XXX | | y | y | XXXXX |
| XXX | | y | y | XXXXX |
| XXX | | y | y | XXXXX |
| XXX | | y | y | XXXXX |
| XXX | | y | y | XXXXX |
| XXX | | y | y | XXXXX |
| XXX | | y | y | XXXXX |
| XXX | | y | y | XXXXX |
| XXX | | y | n | XXXXX |
| XXX | | y | n | XXXXX |
| XXX | | y | n | XXXXX |
| XXX | | y | n | XXXXX |
| XXX | | y | n | XXXXX |
| XXX | | y | n | XXXXX |
| XXX | | y | n | XXXXX |
| XXX | | y | n | XXXXX |
| XXX | | y | n | XXXXX |
| XXX | | y | n | XXXXX |
| XXX | | y | n | XXXXX |
| XXX | XXXX | y | n | XXXXX |
| XXX | XXXX | y | n | XXXXX |
| XXX | XXXX | y | n | XXXXX |
| XXX | XXXX | y | n | XXXXX |
| XXX | XXXX | y | n | XXXXX |
| XXX | XXXX | y | n | XXXXX |
| XXX | | y | n | XXXXX |
| XXX | | y | n | XXXXX |
| XXX | | y | n | XXXXX |
| XXX | | y | n | XXXXX |
| XXX | | y | n | XXXXX |
| XXX | | y | n | XXXXX |
| XXX | | y | n | XXXXX |
| XXX | XXXX | y | n | XXXXX |
| XXX | XXXX | y | n | XXXXX |
| XXX | XXXX | y | n | XXXXX |
| XXX | | y | n | XXXXX |
| XXX | | y | n | XXXXX |
| XXX | | y | n | XXXXX |
| XXX | | y | n | XXXXX |
| XXX | | y | n | XXXXX |
| XXX | | y | n | XXXXX |
| XXX | | y | n | XXXXX |
| XXX | | y | n | XXXXX |
| XXX | | y | n | XXXXX |
| XXX | | y | n | XXXXX |
| XXX | | y | n | XXXXX |
| XXX | | y | n | XXXXX |
| XXX | | y | n | XXXXX |
| XXX | | y | n | XXXXX |

| | | | | |
|---|---|---|---|---|
| XXX | | y | n | XXXXX |
| XXX | | y | n | XXXXX |
| XXX | | y | n | XXXXX |
| XXX | | y | n | XXXXX |
| XXX | | y | n | XXXXX |
| XXX | | y | n | XXXXX |
| XXX | XXXX | y | n | XXXXX |
| XXX | XXXX | y | n | XXXXX |
| XXX | XXXX | y | n | XXXXX |
| XXX | XXXX | y | n | XXXXX |
| XXX | XXXX | y | n | XXXXX |
| XXX | | y | n | XXXXX |
| XXX | | y | n | XXXXX |
| XXX | | y | n | XXXXX |
| XXX | | y | n | XXXXX |
| XXX | | y | n | XXXXX |
| XXX | | y | n | XXXXX |
| XXX | | y | n | XXXXX |
| XXX | XXXX | y | n | XXXXX |
| XXX | XXXX | y | n | XXXXX |
| XXX | XXXX | y | n | XXXXX |
| XXX | XXXX | y | n | XXXXX |
| XXX | | y | n | XXXXX |
| XXX | | y | n | XXXXX |
| XXX | | y | n | XXXXX |
| XXX | | y | n | XXXXX |
| XXX | | y | n | XXXXX |
| XXX | | y | n | XXXXX |
| XXX | | y | n | XXXXX |
| XXX | | y | n | XXXXX |
| XXX | | y | n | XXXXX |
| XXX | | y | n | XXXXX |
| XXX | | y | n | XXXXX |
| XXX | XXXX | n | n | XXXXX |
| XXX | | y | n | XXXXX |
| XXX | | n | y | XXXXX |
| XXX | | n | y | XXXXX |
| XXX | | n | y | XXXXX |
| XXX | | y | n | XXXXX |
| XXX | | y | n | XXXXX |
| XXX | | y | n | XXXXX |
| XXX | | y | n | XXXXX |
| XXX | | y | n | XXXXX |
| XXX | XXXX | y | y | XXXXX |
| XXX | XXXX | y | y | XXXXX |
| XXX | XXXX | y | y | XXXXX |
| XXX | XXXX | y | y | XXXXX |
| XXX | | n | n | XXXXX |
| XXX | | y | n | XXXXX |

| | | | | |
|---|---|---|---|---|
| XXX | | n | N | XXXXX |
| XXX | | n | n | XXXXX |
| XXX | | n | n | XXXXX |
| XXX | | n | n | XXXXX |
| XXX | | n | n | XXXXX |
| XXX | | y | n | XXXXX |
| XXX | | n | n | XXXXX |
| XXX | | n | n | XXXXX |
| XXX | | n | n | XXXXX |
| XXX | | y | n | XXXXX |
| XXX | | y | n | XXXXX |
| XXX | XXXX | y | n | XXXXX |
| XXX | XXXX | n | n | XXXXX |
| XXX | XXXX | y | n | XXXXX |
| XXX | | n | n | XXXXX |
| XXX | | n | n | XXXXX |
| XXX | | n | n | XXXXX |
| XXX | | n | n | XXXXX |
| XXX | | n | n | XXXXX |
| XXX | | n | n | XXXXX |
| XXX | | n | n | XXXXX |
| XXX | | n | n | XXXXX |
| XXX | XXXX | n | n | XXXXX |
| XXX | | n | n | XXXXX |
| XXX | | n | y | XXXXX |
| XXX | XXXX | y | n | XXXXX |
| XXX | XXXX | y | n | XXXXX |
| XXX | XXXX | y | n | XXXXX |
| XXX | XXXX | y | n | XXXXX |
| XXX | XXXX | y | n | XXXXX |
| XXX | | y | n | XXXXX |
| XXX | | y | n | XXXXX |
| XXX | | y | n | XXXXX |
| XXX | | y | n | XXXXX |
| XXX | | y | n | XXXXX |
| XXX | | y | n | XXXXX |
| XXX | | y | n | XXXXX |
| XXX | | y | n | XXXXX |
| XXX | | y | n | XXXXX |
| XXX | | y | n | XXXXX |
| XXX | | y | n | XXXXX |
| XXX | | y | n | XXXXX |
| XXX | | y | n | XXXXX |
| XXX | | y | n | XXXXX |
| XXX | | y | n | XXXXX |
| XXX | | y | n | XXXXX |
| XXX | | y | n | XXXXX |
| XXX | | y | n | XXXXX |

| | | | | | |
|---|---|---|---|---|---|
| | XXX | | y | n | XXXXX |
| | XXX | | y | n | XXXXX |
| | XXX | | y | n | XXXXX |
| | XXX | | y | n | XXXXX |
| | XXX | | y | n | XXXXX |
| | XXX | | y | n | XXXXX |
| | XXX | | y | n | XXXXX |

| analysis date | mean of two deterr | CDC % API2 | gennar | strengthg | analysis date |
|---|---|---|---|---|---|
| 06/02/2013 | **83.6** | | | | |
| 06/02/2013 | **83.8** | | | | |
| 06/02/2013 | **86.7** | | | | |
| 01/02/2013 | **94.4** | | | | |
| 01/02/2013 | **92.9** | | | | |
| 01/02/2013 | **95.8** | | | | |
| 01/02/2013 | **99.8** | | | | |
| 01/02/2013 | **88.3** | | | | |
| 01/02/2013 | **92.4** | | | | |
| 01/02/2013 | **93.3** | | | | |
| 01/02/2013 | **93.4** | | | | |
| 01/02/2013 | **92.6** | | | | |
| 01/02/2013 | **94.8** | | | | |
| 01/02/2013 | **96.5** | | | | |
| 01/02/2013 | **86.8** | | | | |
| 01/02/2013 | **98.3** | | | | |
| 01/02/2013 | **91.7** | | | | |
| 01/02/2013 | **95.3** | | | | |
| 01/02/2013 | **90.6** | | | | |
| 01/02/2013 | **92.9** | | | | |
| 01/02/2013 | **90.1** | | | | |
| 01/02/2013 | **91.9** | | | | |
| 01/02/2013 | **92.7** | | | | |
| 01/02/2013 | **98.8** | | | | |
| 01/02/2013 | **100.4** | | | | |
| 01/02/2013 | **92.7** | | | | |
| 01/02/2013 | **90.3** | | | | |
| 01/02/2013 | **95.3** | 93.0 | | | |
| 01/02/2013 | **94.6** | | | | |
| 01/02/2013 | **92.1** | | | | |
| 01/02/2013 | **94.9** | | | | |
| 01/02/2013 | **96.6** | | | | |
| 01/02/2013 | **95.7** | | | | |
| 01/02/2013 | **98.3** | | | | |
| 01/02/2013 | **96.4** | | | | |
| 01/02/2013 | **96.2** | | | | |
| 01/02/2013 | **96.7** | | | | |
| 01/02/2013 | **93.6** | | | | |
| 01/02/2013 | **89.5** | | | | |

| | | |
|---|---|---|
| 01/02/2013 | **97.7** | |
| 01/02/2013 | **95.5** | |
| 01/02/2013 | **96.9** | |
| 01/02/2013 | **93.5** | |
| 01/02/2013 | **95.1** | |
| 01/02/2013 | **91.4** | |
| 01/02/2013 | **87.7** | |
| 04/02/2013 | **94.0** | |
| 04/02/2013 | **100.7** | 95.8 |
| 04/02/2013 | **96.7** | 102.3 |
| 04/02/2013 | **95.8** | |
| 04/02/2013 | **92.4** | 87.4 |
| 04/02/2013 | **93.0** | |
| 04/02/2013 | **98.0** | |
| 04/02/2013 | **97.2** | |
| 04/02/2013 | **95.5** | 102.6 |
| 04/02/2013 | **94.0** | |
| 04/02/2013 | **106.1** | 100.7 |
| 04/02/2013 | **98.1** | |
| 04/02/2013 | **95.8** | |
| 04/02/2013 | **94.6** | |
| 04/02/2013 | **93.8** | |
| 04/02/2013 | **94.7** | |
| 04/02/2013 | **98.8** | |
| 04/02/2013 | **98.7** | |
| 04/02/2013 | **96.3** | |
| 04/02/2013 | **97.8** | |
| 04/02/2013 | **100.3** | |
| 04/02/2013 | **95.3** | |
| 04/02/2013 | **97.8** | |
| 04/02/2013 | **98.9** | |
| 04/02/2013 | **104.7** | 99.4 |
| 04/02/2013 | **98.0** | |
| 04/02/2013 | **97.0** | |
| 04/02/2013 | **93.8** | |
| 04/02/2013 | **102.8** | |
| 04/02/2013 | **98.3** | |
| 04/02/2013 | **97.7** | |
| 04/02/2013 | **91.0** | |
| 04/02/2013 | **99.2** | |
| 04/02/2013 | **100.8** | |
| 04/02/2013 | **93.8** | |
| 04/02/2013 | **98.1** | 98.6 |
| 04/02/2013 | **96.3** | |
| 04/02/2013 | **99.8** | |
| 04/02/2013 | **91.4** | |
| 04/02/2013 | **96.8** | |
| 04/02/2013 | **99.2** | |
| 04/02/2013 | **94.9** | |
| 04/02/2013 | **98.3** | |

| | | |
|---|---|---|
| 04/02/2013 | **97.0** | |
| 04/02/2013 | **94.1** | |
| 04/02/2013 | **96.2** | 100.5 |
| 04/02/2013 | **93.1** | |
| 04/02/2013 | **92.9** | |
| 04/02/2013 | **101.9** | |
| 04/02/2013 | **100.9** | |
| 04/02/2013 | **104.5** | |
| 04/02/2013 | **93.1** | |
| 04/02/2013 | **93.0** | |
| 04/02/2013 | **95.4** | |
| 04/02/2013 | **100.0** | |
| 04/02/2013 | **94.2** | |
| 04/02/2013 | **98.2** | |
| 04/02/2013 | **98.1** | |
| 05/02/2013 | **92.1** | |
| 05/02/2013 | **95.4** | |
| 05/02/2013 | **93.4** | |
| 05/02/2013 | **89.8** | |
| 05/02/2013 | **97.2** | |
| 05/02/2013 | **95.1** | |
| 05/02/2013 | **93.5** | |
| 05/02/2013 | **91.6** | |
| 05/02/2013 | **96.8** | |
| 05/02/2013 | **96.4** | |
| 05/02/2013 | **95.9** | |
| 05/02/2013 | **97.9** | |
| 05/02/2013 | **97.8** | |
| 05/02/2013 | **95.0** | |
| 05/02/2013 | **98.9** | |
| 05/02/2013 | **96.8** | |
| 05/02/2013 | **96.8** | |
| 05/02/2013 | **95.5** | |
| 05/02/2013 | **100.4** | |
| 05/02/2013 | **96.0** | |
| 05/02/2013 | **99.3** | |
| 05/02/2013 | **97.2** | |
| 05/02/2013 | **98.1** | 105.8 |
| 05/02/2013 | **94.8** | |
| 05/02/2013 | **99.3** | |
| 05/02/2013 | **94.9** | |
| 05/02/2013 | **90.1** | |
| 05/02/2013 | **93.5** | |
| 05/02/2013 | **92.7** | |
| 05/02/2013 | **95.9** | |
| 05/02/2013 | **96.6** | |
| 05/02/2013 | **96.4** | |
| 05/02/2013 | **96.1** | |
| 05/02/2013 | **100.7** | |
| 05/02/2013 | **95.5** | |

| | | |
|---|---|---|
| 05/02/2013 | **96.6** | |
| 05/02/2013 | **96.4** | |
| 05/02/2013 | **94.3** | |
| 05/02/2013 | **98.0** | |
| 05/02/2013 | **95.6** | |
| 05/02/2013 | **96.7** | 100.5 |
| 05/02/2013 | **95.1** | |
| 05/02/2013 | **90.9** | |
| 05/02/2013 | **92.3** | |
| 05/02/2013 | **93.7** | |
| 05/02/2013 | **97.9** | |
| 05/02/2013 | **94.6** | 99.2 |
| 05/02/2013 | **97.6** | |
| 05/02/2013 | **97.1** | |
| 05/02/2013 | **95.8** | 96.7 |
| 05/02/2013 | **97.6** | |
| 05/02/2013 | **98.4** | 96.8 |
| 05/02/2013 | **98.3** | 102.3 |
| 06/02/2013 | **82.4** | |
| 06/02/2013 | **81.0** | |
| 06/02/2013 | **98.9** | |
| 06/02/2013 | **71.0** | |
| 06/02/2013 | **84.4** | |
| 06/02/2013 | **90.0** | |
| 06/02/2013 | **90.0** | 100.2 |
| 06/02/2013 | **89.5** | |
| 06/02/2013 | **88.1** | |
| 06/02/2013 | **88.7** | |
| 06/02/2013 | **86.6** | |
| 06/02/2013 | **91.1** | |
| 06/02/2013 | **99.3** | |
| 06/02/2013 | **89.8** | |
| 06/02/2013 | **91.4** | |
| 06/02/2013 | **105.6** | |
| 06/02/2013 | **87.9** | |
| 06/02/2013 | **86.0** | |
| 06/02/2013 | **88.4** | |
| 06/02/2013 | **90.0** | |
| 06/02/2013 | **93.2** | |
| 06/02/2013 | **90.1** | |
| 06/02/2013 | **87.0** | |
| 06/02/2013 | **92.5** | |
| 06/02/2013 | **94.2** | |
| 06/02/2013 | **86.4** | |
| 06/02/2013 | **95.6** | |
| 06/02/2013 | **94.4** | |
| 06/02/2013 | **91.6** | |
| 06/02/2013 | **97.3** | |
| 06/02/2013 | **93.9** | |
| 06/02/2013 | **96.6** | 99.1 |

| | | |
|---|---|---|
| 06/02/2013 | **96.5** | |
| 06/02/2013 | **93.5** | |
| 06/02/2013 | **99.1** | |
| 06/02/2013 | **96.4** | |
| 06/02/2013 | **90.7** | |
| 06/02/2013 | **85.7** | 88.4 |
| 06/02/2013 | **86.8** | |
| 06/02/2013 | **76.6** | |
| 07/02/2013 | **98.5** | |
| 07/02/2013 | **92.0** | |
| 07/02/2013 | **94.3** | |
| 07/02/2013 | **95.4** | |
| 07/02/2013 | **94.4** | 112.2 |
| 07/02/2013 | **95.0** | |
| 07/02/2013 | **101.8** | |
| 07/02/2013 | **95.0** | |
| 07/02/2013 | **95.2** | |
| 07/02/2013 | **97.6** | |
| 07/02/2013 | **91.1** | |
| 07/02/2013 | **92.9** | |
| 07/02/2013 | **100.8** | 91.5 |
| 07/02/2013 | **100.8** | |
| 07/02/2013 | **92.3** | |
| 07/02/2013 | **96.9** | |
| 08/02/2013 | **96.5** | |
| 08/02/2013 | **95.7** | |
| 08/02/2013 | **93.1** | |
| 08/02/2013 | **94.6** | |
| 08/02/2013 | **94.7** | |
| 08/02/2013 | **96.0** | 109.2 |
| 08/02/2013 | **96.9** | |
| 08/02/2013 | **98.0** | |
| 08/02/2013 | **92.8** | |
| 08/02/2013 | **95.1** | |
| 08/02/2013 | **95.2** | |
| 08/02/2013 | **96.0** | |
| 08/02/2013 | **93.6** | |
| 08/02/2013 | **95.8** | |
| 08/02/2013 | **96.0** | |
| 08/02/2013 | **88.3** | |
| 08/02/2013 | **93.0** | |
| 08/02/2013 | **97.2** | |
| 08/02/2013 | **97.9** | |
| 08/02/2013 | **96.8** | |
| 08/02/2013 | **96.4** | |
| 08/02/2013 | **93.7** | |
| 08/02/2013 | **94.3** | |
| 08/02/2013 | **96.1** | |
| 08/02/2013 | **92.7** | |
| 08/02/2013 | **97.9** | |

| | | |
|---|---|---|
| 08/02/2013 | **99.3** | |
| 08/02/2013 | **98.1** | |
| 08/02/2013 | **95.9** | |
| 08/02/2013 | **94.5** | |
| 08/02/2013 | **98.7** | 96.8 |
| 08/02/2013 | **96.3** | |
| 08/02/2013 | **102.2** | |
| 08/02/2013 | **95.2** | |
| 08/02/2013 | **96.0** | 107.8 |
| 08/02/2013 | **94.5** | |
| 08/02/2013 | **97.0** | 94.2 |
| 08/02/2013 | **105.2** | |
| 08/02/2013 | **103.3** | |
| 08/02/2013 | **103.4** | 111.0 |
| 11/02/2013 | **101.2** | |
| 11/02/2013 | **96.7** | |
| 11/02/2013 | **98.2** | |
| 11/02/2013 | **96.0** | |
| 11/02/2013 | **93.3** | 110.2 |
| 11/02/2013 | **95.2** | |
| 11/02/2013 | **93.9** | |
| 11/02/2013 | **98.3** | |
| 11/02/2013 | **99.4** | |
| 11/02/2013 | **97.0** | 98.2 |
| 11/02/2013 | **96.4** | |
| 11/02/2013 | **96.1** | |
| 11/02/2013 | **102.2** | |
| 11/02/2013 | **88.4** | |
| 11/02/2013 | **99.6** | |
| 11/02/2013 | **100.4** | |
| 11/02/2013 | **94.4** | |
| 11/02/2013 | **98.2** | |
| 11/02/2013 | **99.2** | 103.2 |
| 11/02/2013 | **97.9** | |
| 11/02/2013 | **95.4** | 101.2 |
| 11/02/2013 | **97.6** | |
| 11/02/2013 | **97.2** | |
| 11/02/2013 | **106.3** | |
| 11/02/2013 | **100.8** | |
| 11/02/2013 | **107.0** | |
| 11/02/2013 | **91.8** | |
| 11/02/2013 | **90.4** | |
| 11/02/2013 | **98.3** | |
| 11/02/2013 | **97.8** | |
| 11/02/2013 | **91.3** | |
| 11/02/2013 | **93.7** | |
| 11/02/2013 | **93.3** | |
| 11/02/2013 | **Not detectable** | |
| 11/02/2013 | **92.1** | |
| 11/02/2013 | **95.2** | |

| | | |
|---|---|---|
| 11/02/2013 | **98.7** | |
| 11/02/2013 | **93.1** | |
| 11/02/2013 | **99.6** | |
| 11/02/2013 | **88.5** | |
| 11/02/2013 | **92.3** | 98.6 |
| 11/02/2013 | **94.7** | 105.5 |
| 11/02/2013 | **90.5** | |
| 11/02/2013 | **99.9** | |
| 11/02/2013 | **98.2** | |
| 11/02/2013 | **90.5** | 88.0 |
| 11/02/2013 | **94.3** | |
| 11/02/2013 | **92.7** | 100.9 |
| 11/02/2013 | **97.7** | |
| 11/02/2013 | **95.5** | |
| 11/02/2013 | **95.4** | |
| 11/02/2013 | **96.4** | |
| 11/02/2013 | **94.6** | |
| 11/02/2013 | **91.8** | 93.0 |
| 11/02/2013 | **88.4** | |
| 11/02/2013 | **91.1** | |
| 11/02/2013 | **95.8** | |
| 11/02/2013 | **93.5** | |
| 11/02/2013 | **104.2** | |
| 11/02/2013 | **88.0** | |
| 11/02/2013 | **99.3** | |
| 11/02/2013 | **96.0** | |
| 11/02/2013 | **95.3** | |
| 11/02/2013 | **85.7** | |
| 11/02/2013 | **100.2** | |
| 11/02/2013 | **95.0** | |
| 11/02/2013 | **100.3** | |
| 11/02/2013 | **Not detectable** | **Not detectable** |
| 11/02/2013 | **86.4** | |
| 11/02/2013 | **93.7** | |
| 11/02/2013 | **98.6** | |
| 11/02/2013 | **91.2** | |
| 11/02/2013 | **98.5** | |
| 11/02/2013 | **95.0** | |
| 11/02/2013 | **84.0** | |
| 11/02/2013 | **102.5** | |
| 11/02/2013 | **87.4** | |
| 12/02/2013 | **96.1** | |
| 12/02/2013 | **95.6** | |
| 12/02/2013 | **98.3** | |
| 12/02/2013 | **89.8** | |
| 12/02/2013 | **102.1** | |
| 12/02/2013 | **97.6** | |
| 12/02/2013 | **106.7** | 103.9 |
| 12/02/2013 | **101.0** | 103.9 |
| 12/02/2013 | **89.6** | |

| | | |
|---|---|---|
| 12/02/2013 | **86.1** | |
| 12/02/2013 | **91.3** | |
| 12/02/2013 | **98.0** | |
| 12/02/2013 | **100.3** | |
| 12/02/2013 | **102.9** | |
| 12/02/2013 | **91.0** | |
| 12/02/2013 | **98.3** | |
| 12/02/2013 | **95.6** | |
| 12/02/2013 | **97.0** | |
| 12/02/2013 | **99.0** | |
| 12/02/2013 | **101.6** | |
| 12/02/2013 | **102.2** | |
| 12/02/2013 | **93.8** | |
| 12/02/2013 | **105.2** | |
| 12/02/2013 | **104.4** | |
| 12/02/2013 | **92.0** | |
| 12/02/2013 | **100.0** | |
| 12/02/2013 | **99.8** | |
| 12/02/2013 | **103.6** | |
| 12/02/2013 | **102.2** | |
| 12/02/2013 | **103.7** | |
| 12/02/2013 | **94.8** | |
| 12/02/2013 | **90.4** | |
| 12/02/2013 | **107.1** | |
| 12/02/2013 | **107.3** | |
| 12/02/2013 | **101.8** | |
| 12/02/2013 | **105.0** | |
| 12/02/2013 | **101.7** | |
| 12/02/2013 | **101.7** | |
| 12/02/2013 | **103.1** | |
| 12/02/2013 | **104.4** | |
| 12/02/2013 | **97.8** | |
| 12/02/2013 | **101.0** | |
| 12/02/2013 | **98.8** | |
| 12/02/2013 | **106.2** | |
| 12/02/2013 | **103.3** | 103.6 |
| 12/02/2013 | **96.2** | |
| 12/02/2013 | **99.5** | |
| 12/02/2013 | **101.4** | |
| 12/02/2013 | **101.3** | |
| 12/02/2013 | **102.8** | |
| 12/02/2013 | **101.3** | |
| 12/02/2013 | **101.1** | 110.8 |
| 12/02/2013 | **101.9** | |
| 12/02/2013 | **100.8** | |
| 12/02/2013 | **99.2** | |
| 12/02/2013 | **102.0** | |
| 12/02/2013 | **104.6** | |
| 12/02/2013 | **99.2** | 96.1 |
| 12/02/2013 | **101.2** | |

| | | |
|---|---|---|
| 12/02/2013 | **98.8** | 108.0 |
| 12/02/2013 | **101.2** | |
| 12/02/2013 | **95.0** | |
| 12/02/2013 | **99.4** | |
| 12/02/2013 | **106.9** | |
| 12/02/2013 | **102.0** | |
| 12/02/2013 | **101.6** | |
| 12/02/2013 | **104.1** | |
| 12/02/2013 | **99.4** | 107.6 |
| 07/02/2013 | **52.1** | |
| 07/02/2013 | **53.1** | |
| 07/02/2013 | **96.7** | 104.0 |
| 07/02/2013 | **97.5** | |
| 07/02/2013 | **96.5** | |
| 07/02/2013 | **88.4** | |
| 07/02/2013 | **97.6** | |
| 07/02/2013 | **95.8** | |
| 07/02/2013 | **102.9** | 104.3 |
| 07/02/2013 | **96.4** | |
| 07/02/2013 | **97.6** | |
| 07/02/2013 | **98.2** | |
| 07/02/2013 | **96.7** | |
| 07/02/2013 | **99.4** | |
| 07/02/2013 | **99.8** | 104.2 |
| 07/02/2013 | **99.4** | |
| 07/02/2013 | **96.4** | |
| 07/02/2013 | **99.4** | |
| 07/02/2013 | **94.9** | |
| 07/02/2013 | **94.9** | |
| 07/02/2013 | **99.1** | 103.2 |
| 07/02/2013 | **97.4** | |
| 07/02/2013 | **95.1** | |
| 07/02/2013 | **99.7** | |
| 07/02/2013 | **90.4** | 100.9 |
| 07/02/2013 | **97.9** | |
| 07/02/2013 | **94.7** | 100.5 |
| 07/02/2013 | **92.7** | |
| 07/02/2013 | **93.6** | |
| 07/02/2013 | **97.8** | |
| 07/02/2013 | **98.0** | |
| 07/02/2013 | **100.1** | |
| 07/02/2013 | **100.4** | 98.6 |
| 07/02/2013 | **98.3** | |
| 07/02/2013 | **91.0** | 101.9 |
| 19/02/2013 | **95.5** | |
| 13/02/2013 | **99.4** | |
| 13/02/2013 | **96.0** | |
| 13/02/2013 | **99.2** | |
| 13/02/2013 | **104.6** | |
| 13/02/2013 | **99.5** | |

| | | |
|---|---|---|
| 13/02/2013 | **90.5** | |
| 13/02/2013 | **95.8** | |
| 13/02/2013 | **104.9** | |
| 13/02/2013 | **97.1** | |
| 13/02/2013 | **103.9** | |
| 13/02/2013 | **98.5** | |
| 13/02/2013 | **103.3** | |
| 13/02/2013 | **92.9** | |
| 13/02/2013 | **100.9** | |
| 13/02/2013 | **99.4** | |
| 13/02/2013 | **103.7** | |
| 13/02/2013 | **108.0** | |
| 13/02/2013 | **98.9** | |
| 13/02/2013 | **94.3** | |
| 13/02/2013 | **99.0** | |
| 13/02/2013 | **98.7** | |
| 13/02/2013 | **103.8** | |
| 13/02/2013 | **94.2** | |
| 13/02/2013 | **99.2** | |
| 13/02/2013 | **101.6** | |
| 13/02/2013 | **111.9** | |
| 13/02/2013 | **101.9** | |
| 13/02/2013 | **100.7** | |
| 13/02/2013 | **108.5** | |
| 13/02/2013 | **99.5** | |
| 13/02/2013 | **102.6** | |
| 13/02/2013 | **89.8** | |
| - | **-** | |
| 13/02/2013 | **106.1** | 96.3 |
| 13/02/2013 | **105.4** | |
| 13/02/2013 | **95.9** | 99.7 |
| 13/02/2013 | **101.9** | |
| 13/02/2013 | **92.7** | |
| 13/02/2013 | **105.8** | |
| 13/02/2013 | **90.7** | |
| 13/02/2013 | **104.6** | |
| 13/02/2013 | **105.2** | |
| 13/02/2013 | **97.5** | |
| 13/02/2013 | **94.5** | |
| 13/02/2013 | **100.6** | |
| 14/02/2013 | **108.7** | |
| 14/02/2013 | **99.6** | |
| 14/02/2013 | **105.8** | |
| 14/02/2013 | **95.1** | |
| 14/02/2013 | **104.3** | |
| 14/02/2013 | **101.9** | 103.1 |
| 14/02/2013 | **100.6** | |
| 14/02/2013 | **98.6** | |
| 14/02/2013 | **101.8** | |
| 14/02/2013 | **106.1** | |

| | | |
|---|---|---|
| 14/02/2013 | **101.7** | |
| 14/02/2013 | **97.1** | |
| 14/02/2013 | **107.3** | |
| 14/02/2013 | **98.0** | |
| 14/02/2013 | **105.3** | |
| 14/02/2013 | **101.3** | |
| 14/02/2013 | **105.6** | |
| 14/02/2013 | **101.0** | |
| 14/02/2013 | **101.8** | |
| 14/02/2013 | **98.7** | |
| 14/02/2013 | **110.9** | |
| 14/02/2013 | **106.5** | |
| 14/02/2013 | **101.5** | |
| 14/02/2013 | **95.5** | |
| 14/02/2013 | **102.6** | |
| 14/02/2013 | **102.4** | |
| 14/02/2013 | **107.6** | |
| 14/02/2013 | **91.3** | 104.3 |
| 14/02/2013 | **109.1** | |
| 14/02/2013 | **100.1** | |
| 14/02/2013 | **102.8** | |
| 14/02/2013 | **109.9** | 102.4 |
| 14/02/2013 | **104.8** | |
| 14/02/2013 | **106.2** | |
| 14/02/2013 | **103.7** | |
| 14/02/2013 | **99.0** | |
| 14/02/2013 | **110.0** | |
| 14/02/2013 | **114.5** | 91.5 |
| 14/02/2013 | **97.1** | |
| 14/02/2013 | **95.9** | |
| 14/02/2013 | **96.7** | |
| 14/02/2013 | **97.8** | |
| 14/02/2013 | **99.9** | |
| 14/02/2013 | **96.7** | |
| 14/02/2013 | **103.7** | |
| 14/02/2013 | **103.2** | |
| 14/02/2013 | **107.5** | |
| 14/02/2013 | **91.1** | 98.5 |
| 14/02/2013 | **108.5** | |
| 14/02/2013 | **105.1** | |
| 14/02/2013 | **112.9** | |
| 14/02/2013 | **101.1** | |
| 14/02/2013 | **105.4** | |
| 14/02/2013 | **104.0** | |
| 14/02/2013 | **104.7** | |
| 14/02/2013 | **103.7** | |
| 14/02/2013 | **102.5** | |
| 14/02/2013 | **105.6** | |
| 14/02/2013 | **110.6** | |
| 14/02/2013 | **99.6** | |

| | | |
|---|---|---|
| 14/02/2013 | **105.3** | |
| 14/02/2013 | **105.6** | |
| 14/02/2013 | **108.6** | |
| 14/02/2013 | **111.9** | |
| 14/02/2013 | **105.4** | |
| 14/02/2013 | **93.6** | |
| 14/02/2013 | **100.9** | |
| 14/02/2013 | **105.1** | |
| 14/02/2013 | **111.3** | 85.0 |
| 14/02/2013 | **102.0** | |
| 14/02/2013 | **109.5** | |
| 14/02/2013 | **114.1** | |
| 14/02/2013 | **111.3** | |
| 14/02/2013 | **100.6** | |
| 14/02/2013 | **111.0** | |
| 14/02/2013 | **98.8** | |
| 14/02/2013 | **106.6** | |
| 14/02/2013 | **110.5** | |
| 14/02/2013 | **102.2** | 104.2 |
| 18/02/2013 | **89.6** | 102.4 |
| 18/02/2013 | **92.5** | |
| 18/02/2013 | **88.7** | |
| 18/02/2013 | **92.5** | |
| 18/02/2013 | **88.2** | 94.3 |
| 18/02/2013 | **97.3** | |
| 18/02/2013 | **94.9** | |
| 18/02/2013 | **91.5** | |
| 18/02/2013 | **95.7** | |
| 18/02/2013 | **94.8** | |
| 18/02/2013 | **86.9** | |
| 18/02/2013 | **93.0** | 98.1 |
| 18/02/2013 | **94.1** | |
| 18/02/2013 | **88.5** | |
| 18/02/2013 | **97.0** | 100.8 |
| 18/02/2013 | **88.3** | |
| 18/02/2013 | **89.5** | |
| 18/02/2013 | **87.5** | |
| 18/02/2013 | **92.1** | |
| 18/02/2013 | **110.9** | |
| 18/02/2013 | **86.7** | |
| 18/02/2013 | **86.1** | |
| 18/02/2013 | **91.8** | |
| 18/02/2013 | **92.9** | |
| 18/02/2013 | **95.1** | |
| 19/02/2013 | **89.7** | |
| 19/02/2013 | **95.1** | |
| 19/02/2013 | **88.8** | |
| 19/02/2013 | **89.4** | |
| 19/02/2013 | **86.5** | |
| 19/02/2013 | **90.8** | |

| | | |
|---|---|---|
| 19/02/2013 | **92.7** | |
| 19/02/2013 | **89.3** | |
| 19/02/2013 | **95.2** | |
| 19/02/2013 | **90.7** | |
| 19/02/2013 | **94.3** | |
| 19/02/2013 | **93.7** | 98.6 |
| 19/02/2013 | **93.3** | |
| 19/02/2013 | **93.5** | |
| 19/02/2013 | **93.5** | |
| 19/02/2013 | **92.5** | |
| 19/02/2013 | **93.7** | |
| 19/02/2013 | **86.8** | |
| 19/02/2013 | **90.9** | |
| 19/02/2013 | **85.8** | |
| 19/02/2013 | **95.5** | |
| 19/02/2013 | **96.5** | 78.6 |
| 19/02/2013 | **94.1** | |
| 19/02/2013 | **94.5** | |
| 19/02/2013 | **99.4** | |
| 19/02/2013 | **98.5** | |
| 19/02/2013 | **93.4** | |
| 19/02/2013 | **102.3** | |
| 19/02/2013 | **109.7** | |
| 19/02/2013 | **94.1** | |
| 19/02/2013 | **89.9** | |
| 19/02/2013 | **104.0** | |
| 19/02/2013 | **97.3** | |
| 20/02/2013 | **99.4** | |
| 20/02/2013 | **98.4** | |
| 20/02/2013 | **88.0** | |
| 20/02/2013 | **98.5** | 99.1 |
| 20/02/2013 | **96.0** | |
| 20/02/2013 | **89.5** | 108.6 |
| 20/02/2013 | **92.4** | |
| 20/02/2013 | **95.3** | |
| 20/02/2013 | **88.9** | |
| 20/02/2013 | **91.8** | |
| 20/02/2013 | **92.4** | |
| 20/02/2013 | **90.4** | |
| 20/02/2013 | **91.2** | 70.9 |
| 20/02/2013 | **90.1** | 67.4 |
| 20/02/2013 | **96.2** | |
| 20/02/2013 | **90.7** | |
| 20/02/2013 | **91.7** | |
| 20/02/2013 | **94.1** | |
| 20/02/2013 | **86.2** | |
| 20/02/2013 | **95.7** | |
| 20/02/2013 | **97.7** | |
| 20/02/2013 | **99.5** | |
| 20/02/2013 | **86.9** | |

| | | |
|---|---|---|
| 20/02/2013 | **85.2** | |
| 20/02/2013 | **90.5** | |
| 20/02/2013 | **98.8** | |
| 20/02/2013 | **86.6** | |
| 20/02/2013 | **86.0** | |
| 20/02/2013 | **92.5** | |
| 20/02/2013 | **98.4** | |
| 20/02/2013 | **104.3** | |
| 20/02/2013 | **95.7** | |
| 20/02/2013 | **96.8** | |
| 20/02/2013 | **99.6** | |
| 20/02/2013 | **88.8** | |
| 20/02/2013 | **86.1** | |
| 20/02/2013 | **88.7** | |
| 20/02/2013 | **87.9** | |
| 20/02/2013 | **94.9** | |
| 20/02/2013 | **86.3** | |
| 20/02/2013 | **103.7** | |
| 20/02/2013 | **98.6** | 91.7 |
| 20/02/2013 | **103.6** | |
| 20/02/2013 | **95.6** | |
| 20/02/2013 | **90.9** | |
| 20/02/2013 | **104.7** | |
| 20/02/2013 | **86.5** | |
| 20/02/2013 | **85.6** | |
| 20/02/2013 | **92.0** | |
| 20/02/2013 | **85.7** | 96.8 |
| 20/02/2013 | **92.3** | |
| 20/02/2013 | **98.9** | |
| 20/02/2013 | **99.6** | |
| 20/02/2013 | **98.1** | |
| 20/02/2013 | **91.5** | |
| 22/02/2013 | **96.3** | |
| 22/02/2013 | **88.2** | 98.6 |
| 22/02/2013 | **90.2** | |
| 22/02/2013 | **95.0** | |
| 22/02/2013 | **36.3** | |
| 22/02/2013 | **38.2** | |
| 22/02/2013 | **51.0** | |
| 22/02/2013 | **34.0** | |
| 22/02/2013 | **87.7** | |
| 22/02/2013 | **90.8** | |
| 22/02/2013 | **91.9** | |
| 22/02/2013 | **85.7** | |
| 22/02/2013 | **92.0** | |
| 22/02/2013 | **96.5** | |
| 22/02/2013 | **92.0** | |
| 22/02/2013 | **90.2** | |
| 22/02/2013 | **93.5** | |
| 22/02/2013 | **95.4** | |

| | | |
|---|---|---|
| 22/02/2013 | **86.7** | |
| 22/02/2013 | **89.3** | |
| 22/02/2013 | **86.2** | |
| 22/02/2013 | **88.5** | |
| 22/02/2013 | **89.9** | |
| 22/02/2013 | **99.2** | |
| 22/02/2013 | **90.0** | |
| 22/02/2013 | **93.9** | |
| 22/02/2013 | **90.7** | |
| 22/02/2013 | **86.7** | |
| 22/02/2013 | **89.5** | |
| 22/02/2013 | **88.9** | |
| 22/02/2013 | **95.5** | |
| 22/02/2013 | **93.1** | |
| 22/02/2013 | **99.6** | |
| 22/02/2013 | **99.3** | |
| 22/02/2013 | **87.7** | |
| 22/02/2013 | **86.6** | |
| 22/02/2013 | **89.7** | |
| 22/02/2013 | **108.3** | 98.6 |
| 22/02/2013 | **90.9** | |
| 22/02/2013 | **89.5** | |
| 22/02/2013 | **90.4** | |
| 22/02/2013 | **89.5** | |
| 22/02/2013 | **85.8** | |
| 22/02/2013 | **89.8** | 112.4 |
| 22/02/2013 | **98.0** | |
| 22/02/2013 | **92.8** | |
| 22/02/2013 | **89.6** | |
| 22/02/2013 | **87.6** | |
| 22/02/2013 | **91.8** | |
| 22/02/2013 | **92.1** | |
| 22/02/2013 | **92.7** | |
| 22/02/2013 | **99.8** | |
| 22/02/2013 | **87.1** | |
| 22/02/2013 | **92.7** | |
| 22/02/2013 | **92.5** | |
| 22/02/2013 | **88.3** | |
| 25/02/2013 | **91.7** | |
| 25/02/2013 | **88.9** | |
| 25/02/2013 | **91.2** | |
| 25/02/2013 | **90.8** | |
| 25/02/2013 | **93.5** | |
| 25/02/2013 | **87.6** | |
| 25/02/2013 | **86.2** | |
| 25/02/2013 | **96.9** | |
| 25/02/2013 | **92.6** | 97.6 |
| 25/02/2013 | **90.8** | |
| 25/02/2013 | **99.4** | |
| 25/02/2013 | **90.2** | |

| | | |
|---|---|---|
| 25/02/2013 | **93.8** | |
| 25/02/2013 | **92.7** | |
| 25/02/2013 | **98.5** | |
| 25/02/2013 | **93.2** | |
| 25/02/2013 | **87.0** | |
| 25/02/2013 | **94.9** | 105.5 |
| 25/02/2013 | **99.9** | |
| 25/02/2013 | **90.5** | |
| 25/02/2013 | **90.4** | |
| 25/02/2013 | **94.5** | |
| 25/02/2013 | **89.3** | |
| 25/02/2013 | **86.9** | |
| 25/02/2013 | **90.1** | |
| 25/02/2013 | **85.9** | |
| 25/02/2013 | **88.3** | 107.7 |
| 25/02/2013 | **90.7** | |
| 25/02/2013 | **93.4** | |
| 25/02/2013 | **93.1** | |
| 25/02/2013 | **90.6** | |
| 25/02/2013 | **92.3** | |
| 25/02/2013 | **93.9** | |
| 25/02/2013 | **95.8** | |
| 25/02/2013 | **86.6** | |
| 25/02/2013 | **91.8** | |
| 25/02/2013 | **91.4** | |
| 25/02/2013 | **92.3** | |
| 25/02/2013 | **90.7** | |
| 25/02/2013 | **89.0** | |
| 25/02/2013 | **101.8** | 76.3 |
| 25/02/2013 | **90.6** | |
| 25/02/2013 | **86.6** | |
| 25/02/2013 | **95.2** | |
| 25/02/2013 | **97.9** | |
| 25/02/2013 | **94.0** | |
| 25/02/2013 | **87.9** | 98.6 |
| 25/02/2013 | **101.5** | |
| 25/02/2013 | **90.8** | |
| 25/02/2013 | **108.7** | 91.8 |
| 25/02/2013 | **105.4** | |
| 25/02/2013 | **94.0** | |
| 25/02/2013 | **102.2** | |
| 25/02/2013 | **87.7** | |
| 25/02/2013 | **101.5** | |
| 25/02/2013 | **86.3** | |
| 25/02/2013 | **92.8** | |
| 25/02/2013 | **109.7** | |
| 25/02/2013 | **99.5** | |
| 25/02/2013 | **104.1** | |
| 25/02/2013 | **94.1** | |
| 25/02/2013 | **107.0** | |

| | | |
|---|---|---|
| 25/02/2013 | **91.8** | |
| 25/02/2013 | **98.2** | |
| 27/02/2013 | **87.8** | |
| 27/02/2013 | **88.6** | |
| 27/02/2013 | **97.0** | |
| 27/02/2013 | **92.4** | |
| 27/02/2013 | **95.7** | |
| 27/02/2013 | **98.4** | |
| 27/02/2013 | **89.7** | |
| 27/02/2013 | **94.9** | |
| 27/02/2013 | **101.2** | |
| 27/02/2013 | **91.5** | |
| 27/02/2013 | **Not detectable** | |
| 27/02/2013 | **87.3** | 108.0 |
| 27/02/2013 | **86.8** | |
| 27/02/2013 | **94.1** | |
| 27/02/2013 | **94.2** | |
| 27/02/2013 | **91.2** | |
| 27/02/2013 | **86.5** | |
| 27/02/2013 | **99.1** | |
| 27/02/2013 | **94.8** | |
| 27/02/2013 | **90.3** | |
| 27/02/2013 | **92.6** | |
| 27/02/2013 | **89.6** | |
| 27/02/2013 | **93.5** | |
| 27/02/2013 | **92.4** | |
| 27/02/2013 | **87.9** | |
| 27/02/2013 | **93.7** | |
| 27/02/2013 | **91.7** | |
| 27/02/2013 | **90.3** | |
| 27/02/2013 | **88.5** | |
| 27/02/2013 | **91.0** | |
| 27/02/2013 | **90.2** | |
| 27/02/2013 | **98.8** | |
| 27/02/2013 | **91.1** | |
| 27/02/2013 | **92.1** | |
| 27/02/2013 | **93.8** | |
| 27/02/2013 | **93.2** | |
| 27/02/2013 | **91.5** | |
| 27/02/2013 | **87.7** | |
| 27/02/2013 | **94.5** | |
| 27/02/2013 | **87.7** | |
| 27/02/2013 | **88.0** | |
| 27/02/2013 | **Not detectable** | **Not detectable** |
| 27/02/2013 | **92.7** | |
| 27/02/2013 | **95.3** | |
| 27/02/2013 | **90.4** | |
| 27/02/2013 | **90.7** | |
| 27/02/2013 | **94.1** | |
| 28/02/2013 | **92.9** | 111.7 |

| | | |
|---|---|---|
| 28/02/2013 | **85.7** | |
| 28/02/2013 | **90.3** | |
| 28/02/2013 | **88.9** | |
| 28/02/2013 | **91.5** | |
| 28/02/2013 | **91.3** | |
| 28/02/2013 | **86.8** | 104.4 |
| 28/02/2013 | **89.4** | |
| 28/02/2013 | **87.2** | |
| 28/02/2013 | **85.2** | |
| 28/02/2013 | **90.9** | |
| 28/02/2013 | **89.3** | 96.3 |
| 28/02/2013 | **90.5** | |
| 28/02/2013 | **91.1** | 99.1 |
| 28/02/2013 | **85.9** | |
| 28/02/2013 | **94.6** | |
| 28/02/2013 | **92.1** | |
| 28/02/2013 | **94.5** | |
| 28/02/2013 | **85.4** | |
| 28/02/2013 | **87.0** | |
| 28/02/2013 | **99.4** | |
| 28/02/2013 | **90.9** | |
| 28/02/2013 | **100.8** | |
| 28/02/2013 | **103.8** | |
| 28/02/2013 | **88.9** | |
| 28/02/2013 | **104.3** | 104.9 |
| 28/02/2013 | **103.5** | |
| 28/02/2013 | **92.8** | |
| 28/02/2013 | **86.4** | |
| 28/02/2013 | **93.7** | |
| 28/02/2013 | **99.6** | |
| 28/02/2013 | **89.4** | |
| 28/02/2013 | **104.4** | |
| 28/02/2013 | **90.7** | |
| 28/02/2013 | **100.7** | |
| 28/02/2013 | **87.5** | |
| 28/02/2013 | **104.8** | |
| 28/02/2013 | **94.3** | |
| 28/02/2013 | **103.9** | |
| 28/02/2013 | **93.2** | |
| 28/02/2013 | **102.7** | |
| 28/02/2013 | **92.6** | |
| 28/02/2013 | **94.4** | |
| 28/02/2013 | **90.2** | |
| 28/02/2013 | **92.1** | |
| 28/02/2013 | **94.7** | |
| 28/02/2013 | **93.8** | |
| 28/02/2013 | **94.7** | |
| 28/02/2013 | **94.9** | |
| 28/02/2013 | **89.8** | |
| 28/02/2013 | **96.2** | |

| | | |
|---|---|---|
| 28/02/2013 | **102.1** | |
| 28/02/2013 | **86.1** | 108.3 |
| 28/02/2013 | **104.8** | |
| 28/02/2013 | **106.4** | |
| 28/02/2013 | **Not detectable** | **Not detectable** |
| 28/02/2013 | **91.5** | |
| 28/02/2013 | **93.3** | |
| 28/02/2013 | **90.8** | |
| 28/02/2013 | **91.0** | |
| 28/02/2013 | **85.3** | |
| 28/02/2013 | **95.9** | |
| 28/02/2013 | **87.8** | |
| 28/02/2013 | **90.9** | |
| 28/02/2013 | **85.7** | |
| 28/02/2013 | **93.2** | |
| 28/02/2013 | **92.0** | 85.5 |
| 28/02/2013 | **93.1** | |
| 28/02/2013 | **101.5** | |
| 25/02/2013 | **102.9** | |
| 25/02/2013 | **102.4** | |
| 25/02/2013 | **93.7** | |
| 25/02/2013 | **109.1** | |
| 25/02/2013 | **107.7** | |
| 25/02/2013 | **108.1** | |
| 26/02/2013 | **95.8** | |
| 26/02/2013 | **91.9** | |
| 26/02/2013 | **88.9** | |
| 26/02/2013 | **88.3** | |
| 26/02/2013 | **85.1** | |
| 26/02/2013 | **89.8** | 95.1 |
| 26/02/2013 | **92.4** | 83.9 |
| 26/02/2013 | **99.9** | |
| 26/02/2013 | **88.4** | |
| 26/02/2013 | **97.4** | |
| 26/02/2013 | **88.6** | |
| 26/02/2013 | **89.6** | |
| 26/02/2013 | **92.0** | |
| 26/02/2013 | **89.1** | |
| 26/02/2013 | **91.3** | |
| 26/02/2013 | **97.8** | |
| 26/02/2013 | **100.9** | |
| 26/02/2013 | **95.9** | |
| 26/02/2013 | **85.3** | |
| 26/02/2013 | **94.6** | |
| 26/02/2013 | **90.4** | |
| 26/02/2013 | **86.6** | |
| 26/02/2013 | **97.4** | 66.0 |
| 26/02/2013 | **93.9** | |
| 26/02/2013 | **94.0** | |
| 26/02/2013 | **98.2** | |

| | | |
|---|---|---|
| 26/02/2013 | **90.1** | |
| 26/02/2013 | **111.2** | |
| 26/02/2013 | **88.0** | |
| 26/02/2013 | **85.9** | |
| 26/02/2013 | **94.2** | |
| 26/02/2013 | **85.2** | |
| 26/02/2013 | **85.1** | |
| 26/02/2013 | **90.9** | |
| 26/02/2013 | **88.5** | |
| 26/02/2013 | **89.7** | |
| 26/02/2013 | **83.9** | 79.6 |
| 26/02/2013 | **91.0** | |
| 26/02/2013 | **86.7** | |
| 26/02/2013 | **86.2** | |
| 26/02/2013 | **87.5** | |
| 26/02/2013 | **87.5** | |
| 26/02/2013 | **86.1** | |
| 26/02/2013 | **85.6** | |
| 26/02/2013 | **90.8** | |
| 26/02/2013 | **88.2** | |
| 26/02/2013 | **91.5** | |
| 26/02/2013 | **86.3** | |
| 26/02/2013 | **88.3** | |
| 26/02/2013 | **89.1** | |
| 26/02/2013 | **92.3** | |
| 26/02/2013 | **87.5** | |
| 26/02/2013 | **86.0** | |
| 26/02/2013 | **94.8** | |
| 26/02/2013 | **100.6** | |
| 26/02/2013 | **100.9** | |
| 26/02/2013 | **108.2** | |
| 26/02/2013 | **91.4** | |
| 26/02/2013 | **91.4** | |
| 26/02/2013 | **94.1** | |
| 26/02/2013 | **98.4** | |
| 26/02/2013 | **90.8** | |
| 26/02/2013 | **88.5** | |
| 26/02/2013 | **98.6** | |
| 26/02/2013 | **88.6** | 99.0 |
| 26/02/2013 | **88.8** | |
| 26/02/2013 | **99.7** | |
| 26/02/2013 | **89.7** | |
| 26/02/2013 | **89.6** | |
| 26/02/2013 | **97.8** | |
| 26/02/2013 | **91.2** | 86.3 |
| 26/02/2013 | **92.3** | |
| 26/02/2013 | **86.1** | |
| 26/02/2013 | **85.0** | |
| 26/02/2013 | **88.4** | |
| 26/02/2013 | **94.1** | |

| | | |
|---|---|---|
| 26/02/2013 | **91.7** | |
| 26/02/2013 | **93.2** | |
| 26/02/2013 | **88.6** | |
| 26/02/2013 | **91.3** | |
| 26/02/2013 | **88.3** | |
| 26/02/2013 | **95.0** | |
| 26/02/2013 | **91.7** | |
| 26/02/2013 | **87.2** | 77.8 |
| 26/02/2013 | **88.8** | |
| 26/02/2013 | **96.2** | |
| 26/02/2013 | **88.9** | |
| 26/02/2013 | **89.0** | |
| 26/02/2013 | **85.8** | |
| | - | |
| | - | |
| | - | |
| | - | |
| | - | |
| | - | |
| | - | |
| | - | |
| | - | |
| | - | |
| | - | |
| | - | |
| | - | |
| | - | |
| | - | |
| | - | |
| | - | |
| | - | |
| | - | |
| | - | |
| | - | |
| | - | |
| | - | |
| | - | |
| | - | |
| | - | |
| | - | |
| | - | |
| | - | |
| | - | |
| | - | |
| | - | |
| | - | |

-
-
-
-
-
-
-
-
-
-
-
-
-
-
-
-
-
-
-
-
-
-
-
-
-
-
-
-
-
-
-
-
-
-
-
-
-
-
-
-
-
-
-
-
-
-
-
-
-
-

| | | |
|---|---|---|
| | - | |
| | - | |
| | - | |
| | - | |
| | - | |
| 03/06/2013 | **93.8** | |
| 03/06/2013 | **103.8** | |
| 03/06/2013 | **101.4** | |
| 03/06/2013 | **99.9** | |
| 03/06/2013 | **101.9** | |
| 03/06/2013 | **103.8** | |
| 03/06/2013 | **100.8** | |
| 03/06/2013 | **98.1** | |
| 03/06/2013 | **104.7** | |
| 03/06/2013 | **104.1** | |
| 03/06/2013 | **106.3** | |
| 03/06/2013 | **107.1** | |
| 03/06/2013 | **105.0** | |
| 03/06/2013 | **102.1** | 98.5 |
| 03/06/2013 | **102.3** | |
| 03/06/2013 | **107.1** | 96.6 |
| 03/06/2013 | **105.6** | |
| 03/06/2013 | **110.1** | |
| 03/06/2013 | **104.2** | |
| 03/06/2013 | **98.6** | |
| 03/06/2013 | **101.0** | |
| 03/06/2013 | **101.9** | |
| 03/06/2013 | **105.9** | |
| 03/06/2013 | **106.8** | 92.1 |
| 03/06/2013 | **101.7** | |
| 03/06/2013 | **106.5** | |
| 30/05/2013 | **102.8** | |
| 30/05/2013 | **106.2** | |
| 30/05/2013 | **104.1** | |
| 30/05/2013 | **107.1** | |
| 30/05/2013 | **108.8** | |
| 30/05/2013 | **101.7** | |
| 30/05/2013 | **103.2** | |
| 30/05/2013 | **107.3** | 101.5 |
| 30/05/2013 | **107.8** | 97.4 |
| 30/05/2013 | **106.9** | |
| 31/05/2013 | **110.8** | |
| 31/05/2013 | **103.2** | |
| 31/05/2013 | **98.4** | |
| 31/05/2013 | **101.4** | |
| 31/05/2013 | **102.5** | |
| 31/05/2013 | **107.7** | |
| 31/05/2013 | **103.8** | 104.8 |
| 31/05/2013 | **104.9** | |
| 31/05/2013 | **101.7** | |

| | | |
|---|---|---|
| 31/05/2013 | **104.4** | |
| 31/05/2013 | **99.9** | |
| 31/05/2013 | **100.2** | 103.9 |
| 31/05/2013 | **103.1** | |
| 31/05/2013 | **102.5** | |
| 31/05/2013 | **105.3** | |
| 31/05/2013 | **109.4** | |
| 31/05/2013 | **105.8** | |
| 31/05/2013 | **101.0** | |
| 31/05/2013 | **101.8** | |
| 31/05/2013 | **104.6** | |
| 04/06/2013 | **100.2** | |
| 04/06/2013 | **102.1** | |
| 04/06/2013 | **100.2** | |
| 31/05/2013 | **104.1** | |
| 31/05/2013 | **100.4** | |
| 31/05/2013 | **103.7** | |
| 31/05/2013 | **99.5** | |
| 31/05/2013 | **105.7** | |
| 31/05/2013 | **103.4** | |
| 31/05/2013 | **101.0** | 107.0 |
| 31/05/2013 | **99.9** | |
| 31/05/2013 | **103.8** | |
| 03/06/2013 | **95.6** | |
| 03/06/2013 | **96.8** | |
| 03/06/2013 | **102.0** | |
| 03/06/2013 | **96.7** | 92.1 |
| 03/06/2013 | **98.3** | |
| 03/06/2013 | **105.5** | |
| 03/06/2013 | **110.3** | |
| 03/06/2013 | **108.1** | |
| 03/06/2013 | **106.1** | |
| 03/06/2013 | **96.2** | |
| 03/06/2013 | **97.0** | |
| 03/06/2013 | **98.3** | |
| 03/06/2013 | **91.6** | |
| 03/06/2013 | **98.8** | |
| 03/06/2013 | **112.9** | |
| 03/06/2013 | **105.4** | |
| 03/06/2013 | **103.0** | |
| 03/06/2013 | **103.1** | |
| 04/06/2013 | **107.3** | |
| 04/06/2013 | **108.0** | |
| 04/06/2013 | **95.6** | |
| 04/06/2013 | **101.6** | |
| 04/06/2013 | **99.7** | |
| 04/06/2013 | **102.5** | |
| 04/06/2013 | **102.5** | |
| 04/06/2013 | **109.5** | |
| 04/06/2013 | **103.2** | |

| | | |
|---|---|---|
| 04/06/2013 | **100.3** | |
| 04/06/2013 | **100.0** | |
| 04/06/2013 | **103.1** | |
| 04/06/2013 | **106.5** | |
| 04/06/2013 | **112.2** | |
| 04/06/2013 | **105.1** | |
| 04/06/2013 | **96.7** | |
| 04/06/2013 | **100.6** | |
| 04/06/2013 | **104.2** | |
| 04/06/2013 | **109.2** | |
| 04/06/2013 | **106.6** | |
| 04/06/2013 | **106.8** | |
| 04/06/2013 | **105.7** | |
| 04/06/2013 | **96.9** | |
| 04/06/2013 | **98.5** | |
| 04/06/2013 | **97.3** | |
| 04/06/2013 | **104.8** | |
| 04/06/2013 | **107.7** | |
| 04/06/2013 | **106.3** | |
| 04/06/2013 | **103.9** | |
| 04/06/2013 | **106.0** | |
| 04/06/2013 | **96.0** | |
| 04/06/2013 | **102.6** | |
| 04/06/2013 | **112.6** | |
| 04/06/2013 | **114.0** | |
| 04/06/2013 | **102.1** | |
| 04/06/2013 | **109.8** | |
| 04/06/2013 | **100.2** | |
| 04/06/2013 | **106.9** | |
| 04/06/2013 | **104.3** | |
| 04/06/2013 | **103.7** | |
| 04/06/2013 | **113.9** | |
| 04/06/2013 | **114.1** | 93.7 |
| 04/06/2013 | **111.4** | |
| 04/06/2013 | **109.9** | 96.6 |
| 04/06/2013 | **110.4** | 98.5 |
| 04/06/2013 | **101.4** | 99.4 |
| 04/06/2013 | **106.2** | |
| 04/06/2013 | **106.8** | |
| 31/05/2013 | **105.1** | |
| 31/05/2013 | **104.4** | 116.0 |
| 31/05/2013 | **108.6** | 101.6 |
| 31/05/2013 | **104.7** | |
| 31/05/2013 | **107.6** | |
| 31/05/2013 | **100.5** | |
| 31/05/2013 | **104.4** | |
| 31/05/2013 | **103.5** | |
| 31/05/2013 | **107.7** | |
| 31/05/2013 | **106.3** | |
| 31/05/2013 | **108.9** | |

| | | |
|---|---|---|
| 31/05/2013 | **108.5** | 96.0 |
| 31/05/2013 | **109.6** | |
| 31/05/2013 | **107.4** | |
| 31/05/2013 | **101.4** | |
| 31/05/2013 | **103.9** | |
| 31/05/2013 | **104.0** | |
| 31/05/2013 | **109.4** | 99.7 |
| 31/05/2013 | **108.0** | 96.8 |
| 31/05/2013 | **108.5** | |
| 31/05/2013 | **104.2** | |
| 31/05/2013 | **103.4** | |
| 31/05/2013 | **93.6** | |
| 31/05/2013 | **103.3** | |
| 31/05/2013 | **106.8** | |
| 31/05/2013 | **108.7** | |
| 31/05/2013 | **110.4** | |
| 31/05/2013 | **108.5** | |
| 31/05/2013 | **103.9** | |
| 31/05/2013 | **102.3** | |
| 31/05/2013 | **100.6** | |
| 31/05/2013 | **109.1** | |
| 31/05/2013 | **110.9** | |
| 31/05/2013 | **108.0** | |
| 31/05/2013 | **110.8** | |
| 31/05/2013 | **109.3** | |
| 31/05/2013 | **102.5** | |
| 31/05/2013 | **103.7** | |
| 31/05/2013 | **99.3** | |
| 31/05/2013 | **108.0** | |
| 31/05/2013 | **108.9** | |
| 31/05/2013 | **110.7** | |
| 31/05/2013 | **110.3** | |
| 31/05/2013 | **108.9** | |
| 31/05/2013 | **105.7** | |
| 31/05/2013 | **98.8** | |
| 31/05/2013 | **101.7** | |
| 31/05/2013 | **101.3** | |
| 31/05/2013 | **109.7** | |
| 31/05/2013 | **106.1** | 96.0 |
| 31/05/2013 | **107.8** | 99.8 |
| 31/05/2013 | **100.0** | 102.1 |
| 15/10/2013 | **98.5** | |
| 15/10/2013 | **97.6** | 100.7 |
| 05/06/2013 | **106.2** | |
| 05/06/2013 | **90.8** | 91.0 |
| 05/06/2013 | **103.5** | |
| 05/06/2013 | **105.7** | |
| 05/06/2013 | **102.5** | |
| 05/06/2013 | **102.2** | |
| 05/06/2013 | **106.4** | |

| | | |
|---|---|---|
| 05/06/2013 | **94.1** | |
| 05/06/2013 | **23.9** | |
| 05/06/2013 | **24.7** | |
| 05/06/2013 | **21.3** | |
| 05/06/2013 | **21.8** | |
| 05/06/2013 | **21.6** | |
| 05/06/2013 | **14.0** | |
| 05/06/2013 | **17.3** | |
| 05/06/2013 | **19.2** | |
| 05/06/2013 | **20.9** | |
| 05/06/2013 | **17.5** | |
| 05/06/2013 | **19.3** | |
| 05/06/2013 | **18.9** | |
| 05/06/2013 | **18.6** | |
| 05/06/2013 | **18.3** | |
| 05/06/2013 | **20.1** | |
| 05/06/2013 | **19.4** | |
| 05/06/2013 | **18.6** | |
| 05/06/2013 | **20.4** | |
| 05/06/2013 | **22.5** | |
| 05/06/2013 | **24.1** | |
| 05/06/2013 | **21.8** | |
| 05/06/2013 | **16.8** | |
| 05/06/2013 | **20.6** | |
| 05/06/2013 | **22.8** | |
| 05/06/2013 | **20.9** | |
| 05/06/2013 | **25.0** | |
| 05/06/2013 | **20.7** | |
| 05/06/2013 | **26.8** | |
| 05/06/2013 | **22.1** | |
| 05/06/2013 | **23.0** | |
| 05/06/2013 | **23.8** | |
| 05/06/2013 | **18.3** | |
| 05/06/2013 | **21.9** | |
| 05/06/2013 | **23.6** | |
| 05/06/2013 | **22.2** | |
| 24/06/2013 | **98.9** | 114.0 |
| 24/06/2013 | **96.6** | |
| 24/06/2013 | **113.1** | 105.0 |
| 24/06/2013 | **95.3** | 107.0 |
| 24/06/2013 | **90.5** | |
| 24/06/2013 | **92.2** | |
| 24/06/2013 | **98.3** | |
| 24/06/2013 | **92.7** | |
| 24/06/2013 | **91.1** | |
| 24/06/2013 | **98.7** | 97.0 |
| 24/06/2013 | **105.1** | |
| 24/06/2013 | **97.2** | |
| 24/06/2013 | **96.4** | |
| 24/06/2013 | **104.1** | |

| | | | | | |
|---|---|---|---|---|---|
| 24/06/2013 | **95.4** | 104.0 | | | |
| 24/06/2013 | **100.9** | | | | |
| 24/06/2013 | **98.0** | | | | |
| 24/06/2013 | **89.9** | | | | |
| 24/06/2013 | **100.3** | 106.0 | | | |
| 24/06/2013 | **93.7** | | | | |
| 24/06/2013 | **98.8** | | | | |
| 24/06/2013 | **90.6** | | | | |
| 24/06/2013 | **95.0** | | | | |
| 24/06/2013 | **102.2** | | | | |
| 24/06/2013 | **90.9** | | | | |
| 24/06/2013 | **97.6** | | | | |
| 24/06/2013 | **95.1** | | | | |
| 24/06/2013 | **103.5** | | | | |
| 24/06/2013 | **92.5** | | | | |
| 24/06/2013 | **99.4** | | | | |
| 24/06/2013 | **93.5** | | | | |
| 24/06/2013 | **95.0** | 101.0 | | | |
| 24/06/2013 | **92.5** | | | | |
| 24/06/2013 | **90.7** | | | | |
| 24/06/2013 | **93.1** | | | | |
| 24/06/2013 | **92.2** | | | | |
| 24/06/2013 | **90.1** | | | | |
| 24/06/2013 | **93.3** | | | | |
| 24/06/2013 | **93.9** | | | | |
| 24/06/2013 | **99.0** | 113.0 | | | |
| 24/06/2013 | **94.8** | | | | |
| 24/06/2013 | **94.4** | | | | |
| 24/06/2013 | **95.8** | | | | |
| 24/06/2013 | **91.6** | | | | |
| 24/06/2013 | **96.8** | | | | |
| 24/06/2013 | **93.6** | | | | |
| 24/06/2013 | **90.8** | | | | |
| 25/06/2013 | **96.1** | | pyrime 25 | | 25/06/2013 |
| 25/06/2013 | **102.3** | | pyrime 25 | | 25/06/2013 |
| 25/06/2013 | **99.1** | | pyrime 25 | | 25/06/2013 |
| 25/06/2013 | **91.6** | | pyrime 25 | | 25/06/2013 |
| 25/06/2013 | **108.0** | | pyrime 25 | | 25/06/2013 |
| 25/06/2013 | **111.2** | | pyrime 25 | | 25/06/2013 |
| 25/06/2013 | **103.8** | | pyrime 25 | | 25/06/2013 |
| 25/06/2013 | **81.1** | | pyrime 25 | | 25/06/2013 |
| 25/06/2013 | **92.7** | | Pyrime 20 | | 25/06/2013 |
| 25/06/2013 | **74.7** | | pyrime 25 | | 25/06/2013 |
| 25/06/2013 | **72.1** | | pyrime 25 | | 25/06/2013 |
| 25/06/2013 | **95.0** | | pyrime 12.5 | | 25/06/2013 |
| 25/06/2013 | **104.4** | | pyrime | 12.5 | 25/06/2013 |
| 25/06/2013 | **104.1** | | pyrime 12.5 | | 25/06/2013 |
| 25/06/2013 | **98.2** | | pyrime 12.5 | | 25/06/2013 |
| 25/06/2013 | **96.4** | | pyrime 12.5 | | 25/06/2013 |
| 25/06/2013 | **98.2** | | pyrime | 12.5 | 25/06/2013 |

| | | | | |
|---|---|---|---|---|
| 25/06/2013 | **94.4** | | pyrime 12.5 | 25/06/2013 |
| 25/06/2013 | **91.9** | | pyrime 12.5 | 25/06/2013 |
| 25/06/2013 | **97.0** | | pyrime 12.5 | 25/06/2013 |
| 27/03/2013 | **95.5** | | | |
| 25/06/2013 | **104.3** | | pyrime 25 | 25/06/2013 |
| 25/06/2013 | **91.1** | | pyrime 25 | 25/06/2013 |
| 25/06/2013 | **106.4** | | pyrime 25 | 25/06/2013 |
| 25/06/2013 | **108.2** | | pyrime 25 | 25/06/2013 |
| 25/06/2013 | **105.1** | | pyrime 25 | 25/06/2013 |
| 25/06/2013 | **91.5** | | pyrime 25 | 25/06/2013 |
| 25/06/2013 | **96.7** | | pyrime 26 | 25/06/2013 |
| 25/06/2013 | **105.1** | | pyrime 25 | 25/06/2013 |
| 25/06/2013 | **108.6** | | pyrime 25 | 25/06/2013 |
| 25/06/2013 | **101.2** | | pyrime 25 | 25/06/2013 |
| 25/06/2013 | **88.1** | | pyrime 25 | 25/06/2013 |
| 26/09/2013 | **95.3** | | pyrime 25 | 26/09/2013 |
| 26/09/2013 | **114.3** | | pyrime 25 | 26/09/2013 |
| 26/09/2013 | **114.2** | | pyrime 25 | 26/09/2013 |
| 26/09/2013 | **101.1** | | pyrime 25 | 26/09/2013 |
| 26/09/2013 | **104.5** | | pyrime 25 | 26/09/2013 |
| 26/09/2013 | **110.6** | | pyrime 25 | 26/09/2013 |
| 25/06/2013 | **100.9** | | pyrime 12.5 | 25/06/2013 |
| 26/09/2013 | **110.5** | | pyrime 25 | 26/09/2013 |
| | **-** | | | |
| | **-** | | | |
| | **-** | | | |
| | **-** | | | |
| | **-** | | | |
| | **-** | | | |
| | **-** | | | |
| | **-** | | | |
| | **-** | | | |
| | **-** | | | |
| | **-** | | | |
| | **-** | | | |
| | **-** | | | |
| 09/04/2013 | **92.1** | | | |
| 09/04/2013 | **95.3** | | | |
| 09/04/2013 | **94.5** | | | |
| 26/03/2013 | **96.2** | | | |
| 26/03/2013 | **95.6** | | | |
| 26/03/2013 | **100.7** | | | |
| 26/03/2013 | **93.8** | | | |
| 26/03/2013 | **104.7** | | | |
| 26/03/2013 | **110.1** | | | |
| 26/03/2013 | **Not detectable** | | | |
| 26/03/2013 | **91.2** | | | |
| 26/03/2013 | **106.4** | | | |
| 26/03/2013 | **105.3** | | | |
| 26/03/2013 | **98.0** | 99.5 | | |

| | | |
|---|---|---|
| 26/03/2013 | **95.6** | 86.0 |
| 26/03/2013 | **110.3** | 80.0 |
| 26/03/2013 | **111.8** | |
| 26/03/2013 | **102.4** | |
| 26/03/2013 | **102.8** | |
| 26/03/2013 | **103.7** | |
| 26/03/2013 | **103.7** | |
| 26/03/2013 | **108.2** | |
| 26/03/2013 | **108.0** | |
| 26/03/2013 | **99.2** | |
| 26/03/2013 | **96.8** | 83.5 |
| 26/03/2013 | **105.1** | |
| 26/03/2013 | **103.3** | 111.4 |
| 26/03/2013 | **102.3** | |
| 26/03/2013 | **107.3** | |
| 26/03/2013 | **101.6** | |
| 26/03/2013 | **88.5** | |
| 26/03/2013 | **98.5** | |
| 26/03/2013 | **104.6** | |
| 26/03/2013 | **99.6** | |
| 26/03/2013 | **97.6** | |
| 26/03/2013 | **101.7** | |
| 26/03/2013 | **106.7** | |
| 26/03/2013 | **102.5** | |
| 26/03/2013 | **98.0** | |
| 26/03/2013 | **100.5** | |
| 26/03/2013 | **103.2** | |
| 26/03/2013 | **104.5** | |
| 26/03/2013 | **86.3** | |
| 26/03/2013 | **104.1** | |
| 26/03/2013 | **102.1** | |
| 26/03/2013 | **Not detectable** | **Not detectable** |
| 26/03/2013 | **99.0** | |
| 26/03/2013 | **102.4** | |
| 26/03/2013 | **101.9** | |
| 26/03/2013 | **108.0** | |
| 26/03/2013 | **91.4** | |
| 26/03/2013 | **Not detectable** | **Not detectable** |
| 26/03/2013 | **112.7** | |
| 26/03/2013 | **111.4** | |
| 26/03/2013 | **93.4** | |
| 26/03/2013 | **106.9** | |
| 26/03/2013 | **102.4** | |
| 26/03/2013 | **111.1** | |
| 26/03/2013 | **111.8** | |
| 26/03/2013 | **108.3** | |
| 26/03/2013 | **Not detectable** | |
| 26/03/2013 | **107.5** | |
| 26/03/2013 | **107.9** | |
| 26/03/2013 | **105.1** | 92.0 |

| | | |
|---|---|---|
| 26/03/2013 | **99.2** | 80.0 |
| 26/03/2013 | **110.4** | |
| 26/03/2013 | **93.3** | |
| 26/03/2013 | **112.4** | |
| 26/03/2013 | **109.4** | |
| 26/03/2013 | **Not detectable** | **Not detectable** |
| 26/03/2013 | **105.9** | 91.3 |
| 26/03/2013 | **112.8** | |
| 26/03/2013 | **106.4** | |
| 26/03/2013 | **110.5** | |
| 26/03/2013 | **111.8** | 84.3 |
| 27/03/2013 | **Not detectable** | **Not detectable** |
| 27/03/2013 | **105.0** | |
| 27/03/2013 | **108.6** | |
| 27/03/2013 | **95.4** | |
| 27/03/2013 | **110.0** | |
| 27/03/2013 | **112.0** | 87.4 |
| 27/03/2013 | **106.5** | |
| 27/03/2013 | **107.6** | |
| 27/03/2013 | **105.3** | |
| 27/03/2013 | **106.0** | |
| 27/03/2013 | **104.0** | |
| 27/03/2013 | **106.7** | |
| 27/03/2013 | **112.1** | |
| 27/03/2013 | **98.7** | |
| 27/03/2013 | **93.1** | |
| 27/03/2013 | **Not detectable** | **Not detectable** |
| 27/03/2013 | **108.4** | |
| 27/03/2013 | **110.0** | |
| 27/03/2013 | **90.3** | |
| 27/03/2013 | **95.7** | |
| 27/03/2013 | **111.3** | |
| 27/03/2013 | **108.0** | |
| 27/03/2013 | **100.3** | |
| 27/03/2013 | **102.8** | 84.1 |
| 27/03/2013 | **105.8** | |
| 27/03/2013 | **107.8** | |
| 27/03/2013 | **108.4** | |
| 03/04/2013 | **Not detectable** | |
| 03/04/2013 | **98.8** | |
| 03/04/2013 | **91.4** | |
| 03/04/2013 | **109.2** | |
| 03/04/2013 | **98.4** | 100.2 |
| 03/04/2013 | **107.6** | |
| 03/04/2013 | **100.2** | |
| 03/04/2013 | **106.9** | |
| 03/04/2013 | **104.0** | 83.0 |
| 03/04/2013 | **102.9** | |
| 03/04/2013 | **105.8** | |
| 03/04/2013 | **104.6** | |

| | | | |
|---|---|---|---|
| 03/04/2013 | **106.8** | | |
| 03/04/2013 | **106.2** | | |
| 03/04/2013 | **97.9** | | |
| 03/04/2013 | **104.9** | | |
| 03/04/2013 | **Not detectable** | | |
| 03/04/2013 | **Not detectable** | **Not detectable** | |
| 03/04/2013 | **Not detectable** | **Not detectable** | |
| 03/04/2013 | **100.4** | | |
| 03/04/2013 | **100.6** | | |
| 03/04/2013 | **Not detectable** | **Not detectable** | |
| 03/04/2013 | **101.6** | | |
| 03/04/2013 | **Not detectable** | **Not detectable** | |
| 03/04/2013 | **104.2** | | |
| 03/04/2013 | **106.0** | | |
| 03/04/2013 | **97.1** | | |
| 03/04/2013 | **93.3** | 88.6 | |
| 03/04/2013 | **102.3** | | |
| 03/04/2013 | **99.7** | | |
| 03/04/2013 | **105.8** | | |
| 03/04/2013 | **98.7** | | |
| 03/04/2013 | **104.8** | | |
| 03/04/2013 | **100.7** | | |
| 03/04/2013 | **100.3** | | |
| 03/04/2013 | **102.2** | | |
| 03/04/2013 | **99.9** | | |
| 03/04/2013 | **105.4** | | |
| 03/04/2013 | **104.7** | | |
| 03/04/2013 | **100.8** | | |
| 03/04/2013 | **105.5** | | |
| 03/04/2013 | **99.5** | | |
| 03/04/2013 | **99.5** | | |
| 03/04/2013 | **101.9** | | |
| 03/04/2013 | **107.7** | | |
| 03/04/2013 | **107.9** | 81.9 | |
| 03/04/2013 | **109.5** | | |
| 03/04/2013 | **100.9** | | |
| 03/04/2013 | **101.6** | 82.9 | |
| 03/04/2013 | **95.6** | | |
| 09/04/2013 | **109.0** | | trimetł 90 |
| 09/04/2013 | **105.1** | | trimetł 90 |
| 09/04/2013 | **101.1** | | trimetł 90 |
| 09/04/2013 | **91.1** | | trimetł 90 |
| 09/04/2013 | **90.2** | | trimetł 90 |
| 09/04/2013 | **86.2** | | trimetł 90 |
| 09/04/2013 | **91.0** | 108.2 | trimetł 90 |
| 09/04/2013 | **94.6** | | trimetł 90 |
| 09/04/2013 | **95.5** | 79.9 | trimetł 90 |
| 09/04/2013 | **96.4** | 114.0 | trimetł 90 |
| 09/04/2013 | **94.7** | | trimetł 90 |
| 09/04/2013 | **95.4** | | trimetł 90 |

| | | |
|---|---|---|
| 09/04/2013 | **95.9** | |
| 09/04/2013 | **92.8** | 109.9 |
| 09/04/2013 | **91.6** | |
| 09/04/2013 | **82.2** | |
| 09/04/2013 | **89.7** | |
| 09/04/2013 | **92.5** | |
| 09/04/2013 | **95.4** | |
| 09/04/2013 | **88.3** | |
| 09/04/2013 | **92.8** | |
| 09/04/2013 | **91.6** | |
| 09/04/2013 | **53.4** | |
| 09/04/2013 | **Not detectable** | |
| 09/04/2013 | **89.5** | |
| 09/04/2013 | **88.0** | |
| 09/04/2013 | **90.9** | |
| 09/04/2013 | **90.3** | |
| 09/04/2013 | **96.6** | 93.7 |
| 09/04/2013 | **92.2** | |
| 09/04/2013 | **94.2** | |
| 09/04/2013 | **91.9** | |
| 05/04/2013 | **113.6** | |
| 05/04/2013 | **110.8** | |
| 05/04/2013 | **104.8** | |
| 05/04/2013 | **94.3** | |
| 05/04/2013 | **113.5** | |
| 05/04/2013 | **104.2** | |
| 05/04/2013 | **110.8** | |
| 05/04/2013 | **94.8** | |
| 05/04/2013 | **95.3** | |
| 05/04/2013 | **108.8** | |
| 05/04/2013 | **111.7** | |
| 05/04/2013 | **95.7** | |
| 05/04/2013 | **101.7** | |
| 05/04/2013 | **107.2** | |
| 05/04/2013 | **109.2** | |
| 05/04/2013 | **91.7** | 104.1 |
| 05/04/2013 | **110.7** | |
| 05/04/2013 | **110.6** | |
| 05/04/2013 | **115.0** | |
| 05/04/2013 | **105.3** | |
| 05/04/2013 | **102.9** | |
| 05/04/2013 | **110.2** | |
| 05/04/2013 | **105.7** | |
| 05/04/2013 | **110.9** | 96.5 |
| 05/04/2013 | **102.5** | 97.4 |
| 05/04/2013 | **109.4** | |
| 05/04/2013 | **103.8** | |
| 05/04/2013 | **97.8** | |
| 05/04/2013 | **109.4** | |
| 05/04/2013 | **110.0** | 85.4 |

| | | |
|---|---|---|
| 05/04/2013 | **101.8** | |
| 05/04/2013 | **90.4** | |
| 05/04/2013 | **109.7** | |
| 05/04/2013 | **111.3** | 101.8 |
| 05/04/2013 | **105.7** | |
| 05/04/2013 | **96.3** | 95.2 |
| 05/04/2013 | **96.5** | 84.5 |
| 05/04/2013 | **105.5** | |
| 05/04/2013 | **113.8** | |
| 05/04/2013 | **86.6** | |
| 05/04/2013 | **101.5** | |
| 05/04/2013 | **110.4** | |
| 05/04/2013 | **107.1** | |
| 05/04/2013 | **91.8** | |
| 05/04/2013 | **100.0** | |
| 05/04/2013 | **109.9** | |
| 05/04/2013 | **105.2** | 89.6 |
| 05/04/2013 | **90.8** | |
| 05/04/2013 | **88.7** | |
| 05/04/2013 | **103.7** | |
| 05/04/2013 | **104.2** | |
| 05/04/2013 | **91.6** | |
| 05/04/2013 | **112.0** | |
| 05/04/2013 | **101.2** | |
| 05/04/2013 | **106.5** | |
| 05/04/2013 | **98.8** | |
| 05/04/2013 | **103.0** | |
| 05/04/2013 | **98.4** | |
| 05/04/2013 | **93.5** | |
| 05/04/2013 | **93.7** | |
| 05/04/2013 | **90.6** | |
| 05/04/2013 | **93.2** | |
| 05/04/2013 | **96.9** | |
| 05/04/2013 | **91.8** | |
| 05/04/2013 | **101.5** | |
| 05/04/2013 | **109.5** | |
| 05/04/2013 | **105.4** | |
| 05/04/2013 | **99.5** | |
| 05/04/2013 | **97.4** | |
| 05/04/2013 | **108.9** | |
| 05/04/2013 | **100.9** | |
| 05/04/2013 | **91.2** | |
| 05/04/2013 | **114.8** | |
| 05/04/2013 | **111.9** | |
| 05/04/2013 | **114.1** | |
| 05/04/2013 | **97.5** | |
| 05/04/2013 | **109.7** | |
| 05/04/2013 | **109.2** | |
| 05/04/2013 | **105.2** | |
| 05/04/2013 | **91.4** | |

| | | |
|---|---|---|
| 09/04/2013 | **100.6** | 89.1 |
| 09/04/2013 | **97.7** | |
| 09/04/2013 | **92.1** | |
| 09/04/2013 | **82.2** | |
| 09/04/2013 | **86.7** | |
| 09/04/2013 | **86.2** | |
| 09/04/2013 | **92.5** | |
| 09/04/2013 | **88.5** | |
| 09/04/2013 | **94.9** | |
| 09/04/2013 | **94.9** | |
| 09/04/2013 | **88.7** | 102.7 |
| 09/04/2013 | **85.2** | |
| 09/04/2013 | **92.9** | |
| 09/04/2013 | **92.4** | |
| 09/04/2013 | **91.0** | |
| 09/04/2013 | **88.8** | |
| 09/04/2013 | **89.4** | |
| 09/04/2013 | **92.5** | |
| 09/04/2013 | **85.0** | |
| 09/04/2013 | **88.2** | |
| 09/04/2013 | **84.8** | |
| 01/07/2013 | **102.8** | |
| CDC | **109.5** | CDC |
| 01/07/2013 | **107.9** | |
| 01/07/2013 | **105.1** | |
| 01/07/2013 | **100.3** | |
| 01/07/2013 | **104.3** | |
| 01/07/2013 | **107.7** | |
| 01/07/2013 | **105.1** | |
| CDC | **101.7** | CDC |
| 01/07/2013 | **113.5** | |
| 01/07/2013 | **107.0** | |
| 01/07/2013 | **112.1** | |
| CDC | **98.7** | CDC |
| CDC | **80.0** | CDC |
| 01/07/2013 | **107.1** | |
| 01/07/2013 | **92.4** | |
| 01/07/2013 | **94.3** | |
| 01/07/2013 | **103.9** | |
| CDC | **117.0** | CDC |
| CDC | **102.9** | CDC |
| 01/07/2013 | **98.6** | |
| CDC | **111.9** | CDC |
| CDC | **102.9** | CDC |
| 01/07/2013 | **110.3** | |
| 01/07/2013 | **107.7** | |
| 01/07/2013 | **99.9** | |
| 01/07/2013 | **107.0** | |
| CDC | **96.9** | CDC |
| CDC | **108.8** | CDC |

| | | |
|---|---|---|
| 01/07/2013 | **107.9** | |
| 01/07/2013 | **110.8** | |
| CDC | **113.4** | CDC |
| CDC | **108.4** | CDC |
| 01/07/2013 | **101.5** | |
| 01/07/2013 | **103.7** | |
| 01/07/2013 | **106.5** | |
| 01/07/2013 | **108.8** | |
| 01/07/2013 | **100.1** | |
| CDC | **113.2** | CDC |
| CDC | **88.2** | CDC |
| 01/07/2013 | **109.8** | |
| 01/07/2013 | **109.0** | |
| 01/07/2013 | **90.9** | |
| CDC | **87.6** | CDC |
| CDC | **90.7** | CDC |
| 01/07/2013 | **93.9** | |
| 01/07/2013 | **113.7** | |
| 01/07/2013 | **107.9** | |
| 01/07/2013 | **98.1** | |
| 01/07/2013 | **96.1** | |
| 26/07/2013 | **98.8** | |
| 26/07/2013 | **96.4** | |
| 26/07/2013 | **100.8** | |
| 26/07/2013 | **93.4** | |
| 26/07/2013 | **94.6** | |
| 26/07/2013 | **97.6** | |
| 26/07/2013 | **94.6** | |
| 26/07/2013 | **97.1** | |
| 26/07/2013 | **94.0** | |
| 26/07/2013 | **96.5** | |
| 26/07/2013 | **97.5** | |
| 26/07/2013 | **94.2** | |
| 26/07/2013 | **97.8** | |
| 26/07/2013 | **92.9** | |
| 26/07/2013 | **91.9** | |
| 26/07/2013 | **103.4** | |
| 26/07/2013 | **98.9** | |
| 26/07/2013 | **103.0** | |
| 26/07/2013 | **97.0** | |
| CDC | **108.6** | CDC |
| CDC | **88.9** | CDC |
| CDC | **90.4** | CDC |
| CDC | **90.6** | CDC |
| CDC | **97.1** | CDC |
| CDC | **105.0** | CDC |
| CDC | **93.6** | CDC |
| CDC | **90.5** | CDC |
| CDC | **112.0** | CDC |
| CDC | **93.3** | CDC |

| | | |
|---|---|---|
| CDC | **80.7** | CDC |
| CDC | **100.1** | CDC |
| CDC | **97.0** | CDC |
| CDC | **95.0** | CDC |
| CDC | **91.2** | CDC |
| CDC | **92.3** | CDC |
| CDC | **95.5** | CDC |
| 26/07/2013 | **111.0** | |
| 26/07/2013 | **91.5** | |
| 26/07/2013 | **97.6** | |
| 26/07/2013 | **97.1** | |
| CDC | **98.3** | CDC |
| CDC | **97.5** | CDC |
| 08/03/2013 | **76.4** | |
| 08/03/2013 | **68.6** | |
| 08/03/2013 | **88.2** | |
| 08/03/2013 | **92.0** | |
| 08/03/2013 | **88.6** | |
| 08/03/2013 | **75.0** | |
| 08/03/2013 | **28.6** | |
| 08/03/2013 | **83.5** | |
| 08/03/2013 | **100.4** | |
| 08/03/2013 | **87.7** | |
| 08/03/2013 | **87.4** | |
| | **-** | |
| | **-** | |
| | **-** | |
| | **-** | |
| | **-** | |
| | **-** | |
| | **-** | |
| | **-** | |
| | **-** | |
| | **-** | |
| | **-** | |
| | **-** | |
| | **-** | |
| | **-** | |
| | **-** | |
| | **-** | |
| CDC | **104** | CDC |
| 01/07/2013 | **101.0** | |
| 16/08/2013 | **92.3** | |
| 16/08/2013 | **97.4** | |
| CDC | **67.4** | |
| 16/08/2013 | **101.0** | CDC |
| 16/08/2013 | **94.7** | |
| CDC | **73.6** | |
| 16/08/2013 | **98.7** | CDC |
| 16/08/2013 | **98.3** | |

| | | |
|---|---|---|
| CDC | **79.3** | |
| 16/08/2013 | **98.7** | CDC |
| CDC | **46.0** | |
| 16/08/2013 | **95.0** | CDC |
| 16/08/2013 | **110.5** | |
| 16/08/2013 | **88.5** | |
| CDC | **102.9** | |
| 16/08/2013 | **94.4** | CDC |
| CDC | **110** | |
| 16/08/2013 | **91.7** | CDC |
| 16/08/2013 | **96.8** | |
| CDC | **108.2** | |
| 16/08/2013 | **98.2** | CDC |
| CDC | **102.6** | |
| 16/08/2013 | **104.8** | CDC |
| CDC | **110.6** | |
| 16/08/2013 | **93.2** | CDC |
| CDC | **107.9** | |
| 16/08/2013 | **90.4** | CDC |
| CDC | **113.5** | |
| 16/08/2013 | **91.4** | CDC |
| CDC | **119.6** | |
| 16/08/2013 | **96.6** | CDC |
| CDC | **119.6** | |
| 16/08/2013 | **96.4** | CDC |
| CDC | **108.9** | |
| 16/08/2013 | **94.9** | CDC |
| CDC | **105.8** | |
| 16/08/2013 | **103.2** | CDC |
| CDC | **106.3** | |
| 16/08/2013 | **91.3** | CDC |
| CDC | **106.3** | |
| 16/08/2013 | **94.6** | CDC |
| CDC | **102.3** | |
| 16/08/2013 | **102.8** | CDC |
| 16/08/2013 | **88.0** | |
| 02/05/2013 | **92.9** | |
| 02/05/2013 | **86.1** | |
| 02/05/2013 | **89.1** | 90.7 |
| 02/05/2013 | **90.5** | |
| 02/05/2013 | **90.1** | |
| 02/05/2013 | **94.6** | |
| 02/05/2013 | **91.6** | |
| 02/05/2013 | **87.9** | |
| 02/05/2013 | **88.3** | |
| 02/05/2013 | **98.8** | |
| 02/05/2013 | **89.6** | |
| 02/05/2013 | **96.3** | |
| 02/05/2013 | **83.1** | |
| 02/05/2013 | **85.8** | |

| | | |
|---|---|---|
| 02/05/2013 | **97.5** | |
| 02/05/2013 | **95.7** | |
| 02/05/2013 | **94.5** | 72.0 |
| 02/05/2013 | **88.9** | |
| 02/05/2013 | **95.1** | |
| 02/05/2013 | **96.4** | |
| 02/05/2013 | **91.1** | |
| 02/05/2013 | **91.6** | |
| 02/05/2013 | **94.5** | 87.5 |
| 02/05/2013 | **90.0** | |
| 02/05/2013 | **90.8** | |
| 02/05/2013 | **94.8** | |
| 02/05/2013 | **86.5** | |
| 02/05/2013 | **89.7** | |
| 02/05/2013 | **93.9** | |
| 02/05/2013 | **89.8** | |
| 02/05/2013 | **93.0** | |
| 02/05/2013 | **91.1** | |
| 02/05/2013 | **96.6** | |
| 02/05/2013 | **92.2** | |
| 02/05/2013 | **90.7** | |
| 02/05/2013 | **85.4** | |
| 02/05/2013 | **92.0** | |
| 02/05/2013 | **87.4** | |
| 02/05/2013 | **95.5** | 106.1 |
| 02/05/2013 | **93.5** | |
| 02/05/2013 | **93.1** | |
| 02/05/2013 | **94.8** | |
| 02/05/2013 | **94.8** | 81.6 |
| 02/05/2013 | **88.0** | |
| 02/05/2013 | **85.8** | |
| 02/05/2013 | **86.3** | |
| 02/05/2013 | **88.3** | |
| 02/05/2013 | **92.2** | |
| 02/05/2013 | **89.9** | |
| 02/05/2013 | **88.4** | |
| 02/05/2013 | **86.7** | |
| 02/05/2013 | **84.7** | |
| 02/05/2013 | **85.1** | 71.4 |
| 02/05/2013 | **88.7** | |
| 02/05/2013 | **91.7** | 100.7 |
| 02/05/2013 | **92.2** | |
| 02/05/2013 | **88.6** | 102.2 |
| 02/05/2013 | **96.0** | |
| 02/05/2013 | **90.2** | |
| 02/05/2013 | **88.5** | |
| 02/05/2013 | **100.5** | |
| 02/05/2013 | **92.2** | |
| 02/05/2013 | **97.9** | |
| 02/05/2013 | **85.8** | |

| | | |
|---|---|---|
| 02/05/2013 | **84.0** | |
| 02/05/2013 | **71.5** | |
| 02/05/2013 | **75.9** | |
| 02/05/2013 | **76.3** | |
| 02/05/2013 | **87.0** | |
| 02/05/2013 | **80.4** | |
| 02/05/2013 | **84.1** | |
| 02/05/2013 | **80.0** | |
| 02/05/2013 | **104.8** | |
| 02/05/2013 | **102.6** | |
| 03/05/2013 | **101.7** | |
| 03/05/2013 | **94.5** | |
| 03/05/2013 | **93.5** | |
| 03/05/2013 | **90.2** | |
| 03/05/2013 | **90.0** | |
| 03/05/2013 | **91.2** | |
| 03/05/2013 | **90.9** | |
| 03/05/2013 | **96.0** | |
| 03/05/2013 | **90.3** | |
| 03/05/2013 | **93.6** | |
| 03/05/2013 | **96.3** | |
| 03/05/2013 | **95.0** | |
| 03/05/2013 | **96.6** | |
| 03/05/2013 | **88.8** | |
| 03/05/2013 | **93.1** | |
| 03/05/2013 | **94.3** | |
| 03/05/2013 | **93.6** | |
| 03/05/2013 | **93.1** | |
| 03/05/2013 | **93.7** | |
| 03/05/2013 | **94.7** | |
| 03/05/2013 | **92.3** | |
| 03/05/2013 | **95.2** | 109.8 |
| 03/05/2013 | **96.5** | |
| 03/05/2013 | **92.2** | 105.0 |
| 03/05/2013 | **93.0** | |
| 03/05/2013 | **95.9** | |
| 03/05/2013 | **94.9** | |
| 03/05/2013 | **96.7** | |
| 03/05/2013 | **97.8** | |
| 03/05/2013 | **96.5** | |
| 03/05/2013 | **97.1** | |
| 03/05/2013 | **96.5** | |
| 03/05/2013 | **98.4** | |
| 03/05/2013 | **94.9** | |
| 03/05/2013 | **94.0** | |
| 03/05/2013 | **95.9** | |
| 03/05/2013 | **92.8** | |
| 03/05/2013 | **96.2** | |
| 03/05/2013 | **96.7** | |
| 03/05/2013 | **96.9** | |

| | | |
|---|---|---|
| 03/05/2013 | **Not detectable** | |
| 03/05/2013 | **96.0** | 0.0 |
| 03/05/2013 | **96.8** | |
| 03/05/2013 | **95.5** | |
| 03/05/2013 | **95.8** | |
| 03/05/2013 | **98.1** | |
| 03/05/2013 | **96.5** | |
| 03/05/2013 | **Not detectable** | |
| 03/05/2013 | **Not detectable** | **Not detectable** |
| 03/05/2013 | **97.7** | 0.0 |
| 03/05/2013 | **102.7** | |
| 03/05/2013 | **97.8** | |
| 03/05/2013 | **100.8** | 100.7 |
| 03/05/2013 | **92.9** | 98.2 |
| 03/05/2013 | **97.7** | |
| 03/05/2013 | **94.9** | |
| 03/05/2013 | **105.1** | |
| 03/05/2013 | **102.5** | |
| 03/05/2013 | **100.9** | |
| 03/05/2013 | **99.4** | |
| 03/05/2013 | **102.9** | |
| 03/05/2013 | **95.1** | |
| 03/05/2013 | **101.0** | |
| 03/05/2013 | **97.7** | |
| 03/05/2013 | **105.4** | |
| 03/05/2013 | **105.7** | |
| 03/05/2013 | **101.3** | |
| 03/05/2013 | **102.4** | |
| 03/05/2013 | **100.9** | |
| 03/05/2013 | **103.1** | |
| 03/05/2013 | **108.4** | |
| 03/05/2013 | **111.5** | |
| 03/05/2013 | **101.2** | |
| 03/05/2013 | **105.8** | |
| 03/05/2013 | **103.3** | |
| 03/05/2013 | **95.1** | |
| 03/05/2013 | **107.6** | |
| 03/05/2013 | **106.6** | |
| 03/05/2013 | **105.7** | |
| 03/05/2013 | **87.5** | |
| 03/05/2013 | **102.7** | 104.8 |
| 07/05/2013 | **99.0** | |
| 07/05/2013 | **96.8** | |
| 07/05/2013 | **95.7** | |
| 07/05/2013 | **92.2** | |
| 07/05/2013 | **92.3** | |
| 07/05/2013 | **93.1** | |
| 07/05/2013 | **95.7** | |
| 07/05/2013 | **97.9** | |
| 07/05/2013 | **93.0** | |

| | | |
|---|---|---|
| 07/05/2013 | **94.7** | |
| 07/05/2013 | **96.8** | |
| 07/05/2013 | **96.3** | |
| 07/05/2013 | **91.4** | |
| 07/05/2013 | **93.1** | |
| 07/05/2013 | **97.5** | |
| 07/05/2013 | **96.8** | |
| 07/05/2013 | **87.5** | |
| 07/05/2013 | **90.3** | |
| 07/05/2013 | **92.9** | |
| 07/05/2013 | **95.0** | 100.7 |
| 07/05/2013 | **89.3** | |
| 07/05/2013 | **93.1** | |
| 07/05/2013 | **96.9** | 92.3 |
| 07/05/2013 | **99.6** | 87.9 |
| 07/05/2013 | **85.0** | |
| 07/05/2013 | **91.8** | |
| 07/05/2013 | **99.1** | |
| 07/05/2013 | **97.1** | |
| 07/05/2013 | **94.5** | |
| 07/05/2013 | **87.0** | |
| 07/05/2013 | **90.9** | |
| 07/05/2013 | **96.6** | |
| 07/05/2013 | **99.4** | |
| 07/05/2013 | **102.2** | |
| 07/05/2013 | **85.3** | |
| 07/05/2013 | **95.9** | |
| 07/05/2013 | **97.8** | |
| 07/05/2013 | **88.6** | |
| 07/05/2013 | **94.6** | |
| 07/05/2013 | **90.3** | |
| 07/05/2013 | **90.9** | |
| 07/05/2013 | **89.6** | |
| 07/05/2013 | **90.4** | |
| 07/05/2013 | **95.0** | |
| 07/05/2013 | **93.8** | |
| 07/05/2013 | **86.9** | 102.9 |
| 07/05/2013 | **88.1** | |
| 07/05/2013 | **105.7** | |
| 07/05/2013 | **89.9** | |
| 07/05/2013 | **90.6** | |
| 07/05/2013 | **93.5** | |
| 07/05/2013 | **95.5** | |
| 07/05/2013 | **93.6** | 102.5 |
| 07/05/2013 | **94.2** | 103.1 |
| 07/05/2013 | **97.2** | 103.3 |
| 07/05/2013 | **95.0** | 91.5 |
| 07/05/2013 | **88.4** | |
| 07/05/2013 | **85.0** | |
| 07/05/2013 | **89.6** | |

| | | |
|---|---|---|
| 07/05/2013 | **82.6** | |
| 07/05/2013 | **87.9** | |
| 07/05/2013 | **90.8** | |
| 07/05/2013 | **94.8** | |
| 07/05/2013 | **101.6** | |
| 07/05/2013 | **87.5** | |
| 07/05/2013 | **91.8** | |
| 07/05/2013 | **90.3** | |
| 07/05/2013 | **92.3** | |
| 07/05/2013 | **94.9** | |
| 07/05/2013 | **90.9** | |
| 07/05/2013 | **90.7** | |
| 07/05/2013 | **93.2** | |
| 07/05/2013 | **88.4** | |
| 07/05/2013 | **92.8** | |
| 07/05/2013 | **90.7** | |
| 07/05/2013 | **85.1** | |
| 07/05/2013 | **90.0** | |
| 07/05/2013 | **99.4** | |
| 07/05/2013 | **88.9** | |
| 07/05/2013 | **95.0** | |
| 07/05/2013 | **92.1** | |
| 07/05/2013 | **91.0** | |
| 07/05/2013 | **90.8** | |
| 08/05/2013 | **106.4** | |
| 08/05/2013 | **92.0** | |
| 08/05/2013 | **77.8** | |
| 08/05/2013 | **91.0** | |
| 08/05/2013 | **91.9** | |
| 08/05/2013 | **93.7** | |
| 08/05/2013 | **90.8** | |
| 08/05/2013 | **96.2** | 84.2 |
| 08/05/2013 | **90.5** | |
| 08/05/2013 | **91.6** | |
| 09/05/2013 | **100.6** | |
| 09/05/2013 | **88.0** | |
| 09/05/2013 | **80.0** | |
| 09/05/2013 | **89.2** | 102.8 |
| 09/05/2013 | **85.7** | |
| 09/05/2013 | **90.7** | |
| 09/05/2013 | **85.2** | |
| 09/05/2013 | **91.1** | |
| 09/05/2013 | **89.7** | 78.1 |
| 09/05/2013 | **94.0** | |
| 09/05/2013 | **91.0** | |
| 09/05/2013 | **91.7** | |
| 09/05/2013 | **92.1** | |
| 09/05/2013 | **92.7** | |
| 09/05/2013 | **90.9** | |
| 09/05/2013 | **99.6** | |

| | | |
|---|---|---|
| 09/05/2013 | **94.4** | |
| 09/05/2013 | **86.0** | |
| 09/05/2013 | **89.8** | |
| 09/05/2013 | **97.0** | |
| 09/05/2013 | **91.4** | |
| 09/05/2013 | **90.2** | 95.9 |
| 09/05/2013 | **92.1** | 85.7 |
| 09/05/2013 | **85.5** | |
| 09/05/2013 | **97.1** | |
| 09/05/2013 | **89.0** | |
| 10/05/2013 | **93.6** | |
| 10/05/2013 | **94.2** | |
| 10/05/2013 | **93.5** | |
| 10/05/2013 | **98.6** | |
| 11/05/2013 | **97.2** | |
| 10/05/2013 | **92.6** | |
| 10/05/2013 | **99.5** | |
| 10/05/2013 | **91.6** | |
| 10/05/2013 | **90.2** | |
| 10/05/2013 | **89.2** | |
| 10/05/2013 | **88.2** | |
| 10/05/2013 | **91.5** | |
| 10/05/2013 | **97.6** | |
| 10/05/2013 | **92.8** | |
| 10/05/2013 | **95.0** | |
| 10/05/2013 | **99.7** | |
| 10/05/2013 | **93.4** | |
| 10/05/2013 | **92.3** | |
| 10/05/2013 | **94.6** | |
| 10/05/2013 | **93.0** | |
| 10/05/2013 | **93.8** | |
| 10/05/2013 | **96.2** | |
| 10/05/2013 | **91.2** | |
| 10/05/2013 | **94.8** | |
| 10/05/2013 | **94.6** | |
| 10/05/2013 | **91.9** | |
| 10/05/2013 | **103.1** | |
| 10/05/2013 | **97.0** | |
| 10/05/2013 | **96.2** | 68.7 |
| 10/05/2013 | **90.4** | |
| 10/05/2013 | **108.3** | |
| 10/05/2013 | **100.6** | |
| 10/05/2013 | **98.2** | |
| 10/05/2013 | **95.7** | |
| 10/05/2013 | **112.2** | |
| 10/05/2013 | **105.5** | |
| 10/05/2013 | **113.8** | |
| 10/05/2013 | **91.9** | |
| 10/05/2013 | **98.7** | |
| 10/05/2013 | **95.2** | |

| | | |
|---|---|---|
| 10/05/2013 | **111.1** | 70.6 |
| 10/05/2013 | **94.3** | |
| 10/05/2013 | **108.1** | |
| 10/05/2013 | **99.0** | |
| 10/05/2013 | **112.4** | |
| 10/05/2013 | **98.8** | 105.1 |
| 10/05/2013 | **113.7** | |
| 10/05/2013 | **108.7** | |
| 10/05/2013 | **98.8** | |
| 10/05/2013 | **99.3** | |
| 10/05/2013 | **95.9** | 112.4 |
| 10/05/2013 | **105.1** | |
| 10/05/2013 | **97.3** | 81.8 |
| 10/05/2013 | **97.3** | |
| 10/05/2013 | **100.0** | |
| 10/05/2013 | **96.3** | |
| 10/05/2013 | **106.1** | |
| 10/05/2013 | **109.1** | |
| 10/05/2013 | **111.4** | |
| 10/05/2013 | **93.7** | 94.5 |
| 10/05/2013 | **95.3** | |
| 14/05/2013 | **87.2** | |
| 14/05/2013 | **92.0** | |
| 14/05/2013 | **91.8** | |
| 14/05/2013 | **88.1** | |
| 14/05/2013 | **98.4** | |
| 14/05/2013 | **92.1** | |
| 14/05/2013 | **96.5** | 95.2 |
| 14/05/2013 | **99.3** | |
| 14/05/2013 | **109.5** | |
| 14/05/2013 | **110.3** | |
| 14/05/2013 | **109.3** | |
| 14/05/2013 | **98.2** | |
| 14/05/2013 | **100.5** | |
| 14/05/2013 | **111.6** | |
| 14/05/2013 | **103.3** | |
| 14/05/2013 | **111.7** | |
| 14/05/2013 | **111.0** | |
| 14/05/2013 | **105.5** | |
| 14/05/2013 | **101.4** | |
| 14/05/2013 | **106.6** | |
| 14/05/2013 | **110.4** | |
| 14/05/2013 | **94.0** | |
| 14/05/2013 | **105.0** | |
| 14/05/2013 | **107.3** | |
| 14/05/2013 | **97.3** | |
| 14/05/2013 | **99.5** | |
| 14/05/2013 | **102.0** | |
| 14/05/2013 | **91.5** | |
| 14/05/2013 | **99.6** | |

| | | |
|---|---|---|
| 14/05/2013 | **103.2** | |
| 14/05/2013 | **102.6** | |
| 14/05/2013 | **102.1** | |
| 14/05/2013 | **111.9** | |
| 14/05/2013 | **103.3** | |
| 14/05/2013 | **103.7** | |
| 14/05/2013 | **102.9** | |
| 14/05/2013 | **103.1** | |
| 14/05/2013 | **107.7** | |
| 14/05/2013 | **108.4** | |
| 14/05/2013 | **105.1** | |
| 14/05/2013 | **100.1** | |
| 14/05/2013 | **108.0** | |
| 14/05/2013 | **106.5** | 93.8 |
| 14/05/2013 | **94.0** | |
| 14/05/2013 | **108.4** | |
| 15/05/2013 | **110.4** | |
| 15/05/2013 | **104.2** | |
| 15/05/2013 | **101.6** | |
| 15/05/2013 | **97.0** | |
| 15/05/2013 | **97.8** | |
| 15/05/2013 | **90.0** | |
| 15/05/2013 | **105.0** | |
| 15/05/2013 | **96.1** | |
| 15/05/2013 | **97.1** | |
| 15/05/2013 | **100.3** | |
| 15/05/2013 | **95.3** | |
| 15/05/2013 | **98.8** | |
| 15/05/2013 | **102.6** | |
| 15/05/2013 | **95.2** | |
| 15/05/2013 | **103.9** | |
| 15/05/2013 | **100.7** | |
| 15/05/2013 | **94.6** | |
| 15/05/2013 | **96.6** | |
| 15/05/2013 | **100.0** | |
| 15/05/2013 | **89.6** | |
| 15/05/2013 | **108.9** | |
| 15/05/2013 | **102.2** | |
| 15/05/2013 | **94.5** | |
| 15/05/2013 | **100.3** | |
| 15/05/2013 | **93.6** | |
| 15/05/2013 | **100.8** | |
| 15/05/2013 | **100.1** | 94.1 |
| 15/05/2013 | **84.3** | |
| 15/05/2013 | **99.8** | 106.4 |
| 15/05/2013 | **104.0** | 102.3 |
| 15/05/2013 | **105.5** | |
| 15/05/2013 | **95.6** | 99.1 |
| 15/05/2013 | **95.4** | |
| 15/05/2013 | **102.1** | |

| | | |
|---|---|---|
| 15/05/2013 | **106.5** | |
| 15/05/2013 | **103.9** | |
| 15/05/2013 | **100.1** | |
| 15/05/2013 | **101.3** | |
| 15/05/2013 | **103.3** | 100.9 |
| 15/05/2013 | **98.4** | 100.0 |
| 15/05/2013 | **102.8** | |
| 15/05/2013 | **100.5** | |
| 15/05/2013 | **103.9** | |
| 15/05/2013 | **100.3** | |
| 15/05/2013 | **110.3** | |
| 15/05/2013 | **105.0** | |
| 15/05/2013 | **105.8** | |
| 15/05/2013 | **97.7** | |
| 15/05/2013 | **95.8** | 99.9 |
| 15/05/2013 | **92.3** | |
| 15/05/2013 | **100.1** | |
| 15/05/2013 | **94.4** | |
| 15/05/2013 | **93.8** | |
| 15/05/2013 | **101.5** | 109.1 |
| 15/05/2013 | **105.5** | |
| 15/05/2013 | **98.9** | |
| 15/05/2013 | **100.1** | |
| 15/05/2013 | **93.8** | |
| 15/05/2013 | **92.8** | |
| 15/05/2013 | **94.9** | |
| 15/05/2013 | **97.1** | |
| 15/05/2013 | **99.5** | |
| 15/05/2013 | **92.1** | |
| 15/05/2013 | **95.0** | |
| 15/05/2013 | **106.1** | |
| 15/05/2013 | **99.2** | |
| 15/05/2013 | **102.0** | |
| 15/05/2013 | **96.0** | |
| 15/05/2013 | **104.6** | |
| 15/05/2013 | **97.1** | |
| 15/05/2013 | **94.6** | |
| 15/05/2013 | **91.0** | |
| 15/05/2013 | **93.7** | |
| 15/05/2013 | **104.9** | |
| 15/05/2013 | **99.7** | |
| 16/05/2013 | **85.3** | |
| 16/05/2013 | **93.9** | |
| 16/05/2013 | **94.9** | |
| 16/05/2013 | **96.1** | |
| 16/05/2013 | **94.9** | |
| 16/05/2013 | **92.3** | |
| 16/05/2013 | **90.4** | |
| 16/05/2013 | **94.2** | |
| 16/05/2013 | **91.9** | |

| | | |
|---|---|---|
| 16/05/2013 | **97.6** | |
| 16/05/2013 | **86.4** | |
| 16/05/2013 | **99.5** | |
| 16/05/2013 | **96.5** | |
| 16/05/2013 | **98.3** | |
| 16/05/2013 | **88.2** | |
| 16/05/2013 | **89.2** | |
| 16/05/2013 | **85.0** | |
| 16/05/2013 | **101.1** | 96.3 |
| 16/05/2013 | **88.0** | |
| 16/05/2013 | **94.7** | |
| 16/05/2013 | **92.5** | |
| 16/05/2013 | **92.3** | |
| 16/05/2013 | **95.8** | |
| 16/05/2013 | **89.7** | |
| 16/05/2013 | **94.6** | |
| 16/05/2013 | **88.5** | |
| 16/05/2013 | **87.2** | |
| 16/05/2013 | **89.6** | |
| 16/05/2013 | **85.5** | |
| 16/05/2013 | **87.5** | |
| 16/05/2013 | **92.4** | 99.5 |
| 16/05/2013 | **91.5** | |
| 16/05/2013 | **88.1** | 99.3 |
| 16/05/2013 | **87.0** | |
| 16/05/2013 | **89.6** | |
| 16/05/2013 | **93.7** | |
| 16/05/2013 | **92.7** | |
| 16/05/2013 | **85.5** | |
| 16/05/2013 | **88.8** | |
| 16/05/2013 | **90.9** | 86.4 |
| 16/05/2013 | **95.6** | |
| 16/05/2013 | **97.5** | 91.3 |
| 16/05/2013 | **89.5** | |
| 16/05/2013 | **88.7** | |
| 16/05/2013 | **91.0** | |
| 16/05/2013 | **96.4** | |
| 16/05/2013 | **86.9** | 92.7 |
| 16/05/2013 | **86.2** | 100.0 |
| 16/05/2013 | **93.2** | |
| 16/05/2013 | **85.4** | |
| 16/05/2013 | **85.7** | |
| 16/05/2013 | **90.3** | |
| 16/05/2013 | **84.1** | |
| 16/05/2013 | **100.3** | |
| 16/05/2013 | **86.2** | |
| 16/05/2013 | **85.3** | 79.6 |
| 16/05/2013 | **96.6** | |
| | **-** | |
| | **-** | |

| | |
|---|---|
| | - |
| | - |
| | - |
| | - |
| | - |
| | - |
| | - |
| | - |
| | - |
| | - |
| | - |
| | - |
| | - |
| | - |
| | - |
| | - |
| | - |
| | - |
| | - |
| | - |
| | - |
| | - |
| | - |
| | - |
| | - |
| | - |
| | - |
| | - |
| | - |
| | - |
| | - |
| | - |
| | - |
| | - |
| | - |
| | - |
| | - |
| | - |
| | - |
| 06/06/2013 | **106.5** |
| 06/06/2013 | **111.9** |
| 06/06/2013 | **112.0** |
| 06/06/2013 | **102.6** |
| 06/06/2013 | **91.5** |
| 06/06/2013 | **91.5** |
| 06/06/2013 | **94.6** |
| 06/06/2013 | **93.8** |
| 06/06/2013 | **94.1** |
| 06/06/2013 | **102.4** |
| 06/06/2013 | **98.6** |

| | | |
|---|---|---|
| 06/06/2013 | **107.7** | |
| 06/06/2013 | **94.0** | |
| 06/06/2013 | **85.1** | |
| 06/06/2013 | **105.5** | |
| 06/06/2013 | **109.2** | |
| 06/06/2013 | **112.8** | 92.4 |
| 06/06/2013 | **106.9** | |
| 06/06/2013 | **114.2** | |
| 06/06/2013 | **112.0** | |
| 06/06/2013 | **115.0** | 96.2 |
| 06/06/2013 | **113.5** | |
| 06/06/2013 | **111.3** | |
| 06/06/2013 | **105.7** | |
| 06/06/2013 | **109.5** | |
| 06/06/2013 | **105.8** | |
| 06/06/2013 | **101.7** | |
| 06/06/2013 | **107.7** | |
| 06/06/2013 | **104.3** | |
| 06/06/2013 | **114.9** | |
| 06/06/2013 | **107.3** | |
| 06/06/2013 | **103.1** | |
| 06/06/2013 | **101.8** | |
| 06/06/2013 | **105.6** | |
| 06/06/2013 | **105.1** | 100.6 |
| 06/06/2013 | **112.9** | |
| 06/06/2013 | **112.9** | |
| 06/06/2013 | **107.6** | |
| 06/06/2013 | **103.3** | |
| 06/06/2013 | **109.0** | |
| 06/06/2013 | **114.6** | 101.1 |
| 06/06/2013 | **113.7** | |
| 06/06/2013 | **113.3** | |
| 06/06/2013 | **91.2** | |
| 06/06/2013 | **88.2** | |
| 06/06/2013 | **94.4** | |
| 06/06/2013 | **98.8** | |
| 06/06/2013 | **93.0** | |
| 06/06/2013 | **95.8** | 102.0 |
| 06/06/2013 | **87.1** | |
| 06/06/2013 | **99.2** | |
| 06/06/2013 | **97.4** | |
| 06/06/2013 | **96.4** | |
| 06/06/2013 | **90.3** | |
| 06/06/2013 | **90.3** | |
| 06/06/2013 | **91.1** | 98.0 |
| 06/06/2013 | **92.1** | 95.0 |
| 06/06/2013 | **94.6** | |
| 06/06/2013 | **90.2** | |
| 06/06/2013 | **91.0** | 94.0 |
| 06/06/2013 | **93.1** | |

| | | | | |
|---|---|---|---|---|
| 06/06/2013 | **99.9** | | | |
| 06/06/2013 | **97.4** | | | |
| 06/06/2013 | **100.7** | | | |
| 06/06/2013 | **95.8** | | | |
| 06/06/2013 | **94.3** | | | |
| 06/06/2013 | **95.1** | | | |
| 06/06/2013 | **89.4** | | | |
| 06/06/2013 | **99.0** | | | |
| 06/06/2013 | **97.9** | | | |
| 06/06/2013 | **95.6** | 89.0 | | |
| 06/06/2013 | **94.4** | 90.0 | | |
| 24/06/2013 | **99.2** | | | |
| 24/06/2013 | **106.0** | | | |
| 24/06/2013 | **98.2** | | | |
| 24/06/2013 | **94.7** | | | |
| 24/06/2013 | **95.2** | | | |
| 24/06/2013 | **96.1** | | | |
| 24/06/2013 | **96.0** | | | |
| 24/06/2013 | **101.3** | | | |
| 24/06/2013 | **97.3** | | | |
| 24/06/2013 | **92.5** | | | |
| 24/06/2013 | **96.0** | | | |
| 24/06/2013 | **102.9** | | | |
| 24/06/2013 | **96.3** | | | |
| 24/06/2013 | **97.1** | | | |
| 24/06/2013 | **93.4** | | | |
| 24/06/2013 | **86.9** | | | |
| 24/06/2013 | **91.1** | | | |
| 24/06/2013 | **93.8** | | | |
| 24/06/2013 | **90.1** | | | |
| 24/06/2013 | **91.0** | | | |
| 24/06/2013 | **88.1** | | | |
| 24/06/2013 | **96.3** | | | |
| 24/06/2013 | **95.7** | | | |
| 24/06/2013 | **97.4** | | | |
| 24/06/2013 | **92.3** | | | |
| 25/06/2013 | **92.4** | | pyrime 25 | 25/06/2013 |
| 25/06/2013 | **87.7** | | pyrime 25 | 25/06/2013 |
| 25/06/2013 | **90.0** | | pyrime 25 | 25/06/2013 |
| 25/06/2013 | **106.5** | | pyrime 25 | 25/06/2013 |
| 25/06/2013 | **97.1** | | pyrime 12.5 | 25/06/2013 |
| 25/06/2013 | **90.2** | | pyrime 12.5 | 25/06/2013 |
| 25/06/2013 | **92.8** | | pyrime 12.5 | 25/06/2013 |
| 25/06/2013 | **93.4** | | pyrime 12.5 | 25/06/2013 |
| 25/06/2013 | **94.1** | | pyrime 12.5 | 25/06/2013 |
| 25/06/2013 | **93.1** | | pyrime 12.5 | 25/06/2013 |
| 25/06/2013 | **94.2** | | pyrime 25 | 25/06/2013 |
| 25/06/2013 | **109.1** | | pyrime 25 | 25/06/2013 |
| 25/06/2013 | **90.1** | | pyrime 25 | 25/06/2013 |
| 25/06/2013 | **96.8** | | pyrime 25 | 25/06/2013 |

| | | | | |
|---|---|---|---|---|
| 25/06/2013 | **94.6** | | pyrime 25 | 25/06/2013 |
| 25/06/2013 | **97.7** | | pyrime 25 | 25/06/2013 |
| 25/06/2013 | **93.2** | | pyrime 25 | 25/06/2013 |
| 25/06/2013 | **95.8** | | pyrime 25 | 25/06/2013 |
| 25/06/2013 | **108.5** | | pyrime 25 | 25/06/2013 |
| 25/06/2013 | **91.7** | | pyrime 25 | 25/06/2013 |
| 25/06/2013 | **97.6** | | pyrime 25 | 25/06/2013 |
| 25/06/2013 | **100.2** | | pyrime 25 | 25/06/2013 |
| 25/06/2013 | **90.1** | | pyrime 25 | 25/06/2013 |
| 25/06/2013 | **98.3** | | pyrime 25 | 25/06/2013 |
| 25/06/2013 | **99.1** | | pyrime 25 | 25/06/2013 |
| | **-** | | | |
| 25/04/2013 | **91.5** | | | |
| 25/04/2013 | **97.5** | | | |
| 25/04/2013 | **98.2** | 85.6 | | |
| 25/04/2013 | **96.0** | | | |
| 25/04/2013 | **111.0** | 92.5 | | |
| 25/04/2013 | **98.8** | | | |
| 25/04/2013 | **Not detectable** | | | |
| 25/04/2013 | **105.4** | 0.0 | | |
| 25/04/2013 | **112.7** | | | |
| 25/04/2013 | **112.3** | | | |
| 25/04/2013 | **104.2** | | | |
| 25/04/2013 | **100.3** | | | |
| 25/04/2013 | **109.4** | | | |
| 25/04/2013 | **104.6** | | | |
| 25/04/2013 | **98.5** | | | |
| 25/04/2013 | **102.0** | | | |
| 25/04/2013 | **111.3** | | | |
| 25/04/2013 | **103.6** | | | |
| 25/04/2013 | **102.2** | | | |
| 25/04/2013 | **96.6** | | | |
| 25/04/2013 | **109.4** | | | |
| 25/04/2013 | **Not detectable** | | | |
| 25/04/2013 | **99.5** | 0.0 | | |
| 25/04/2013 | **Not detectable** | | | |
| 25/04/2013 | **114.7** | 0.0 | | |
| 25/04/2013 | **85.4** | | | |
| 25/04/2013 | **92.7** | | | |
| 25/04/2013 | **100.8** | | | |
| 25/04/2013 | **96.1** | | | |
| 25/04/2013 | **113.9** | | | |
| 25/04/2013 | **94.2** | | | |
| 25/04/2013 | **101.0** | | | |
| 25/04/2013 | **Not detectable** | | | |
| 25/04/2013 | **105.9** | 0.0 | | |
| 25/04/2013 | **114.2** | | | |
| 25/04/2013 | **114.5** | | | |
| 25/04/2013 | **99.7** | | | |
| 25/04/2013 | **106.3** | | | |

| | | | |
|---|---|---|---|
| 25/04/2013 | **107.0** | | |
| 25/04/2013 | **100.7** | 83.1 | |
| 25/04/2013 | **116.9** | 85.0 | |
| 25/04/2013 | **100.6** | | |
| 25/04/2013 | **113.7** | | |
| 25/04/2013 | **95.2** | | |
| 25/04/2013 | **114.1** | | |
| 25/04/2013 | **114.1** | | |
| 25/04/2013 | **107.8** | | |
| 25/04/2013 | **91.9** | | |
| 25/04/2013 | **97.4** | | |
| 25/04/2013 | **110.7** | | |
| 25/04/2013 | **107.8** | | |
| 25/04/2013 | **101.0** | | |
| 25/04/2013 | **106.1** | | |
| 25/04/2013 | **105.9** | | |
| 25/04/2013 | **109.3** | 80.6 | |
| 25/04/2013 | **101.7** | 80.8 | |
| 25/04/2013 | **101.8** | | |
| 25/04/2013 | **109.4** | | |
| 25/04/2013 | **106.1** | | |
| 25/04/2013 | **106.3** | | |
| 25/04/2013 | **108.5** | | |
| 25/04/2013 | **109.6** | 80.4 | |
| 25/04/2013 | **100.0** | | |
| 25/04/2013 | **112.1** | | |
| 25/04/2013 | **113.7** | | |
| 25/04/2013 | **103.5** | | |
| 25/04/2013 | **99.9** | | |
| 25/04/2013 | **106.4** | | |
| 25/04/2013 | **100.3** | | |
| 01/05/2013 | **104.6** | | trimetl 90 |
| 01/05/2013 | **106.4** | | trimetl 90 |
| 01/05/2013 | **96.0** | | trimetl 90 |
| 01/05/2013 | **95.6** | | trimetl 90 |
| 01/05/2013 | **94.5** | 77.1 | |
| 01/05/2013 | **90.1** | | |
| 01/05/2013 | **98.7** | 100.9 | |
| 01/05/2013 | **86.9** | | |
| 01/05/2013 | **89.1** | 96.0 | |
| 01/05/2013 | **98.8** | | |
| 01/05/2013 | **96.5** | | |
| 30/04/2013 | **95.2** | | |
| 30/04/2013 | **91.0** | | |
| 30/04/2013 | **96.9** | | |
| 30/04/2013 | **112.3** | | |
| 30/04/2013 | **88.5** | | |
| 30/04/2013 | **107.2** | | |
| 30/04/2013 | **101.1** | | |
| 30/04/2013 | **109.9** | | |

| | |
|---|---|
| 30/04/2013 | **94.7** |
| 30/04/2013 | **107.6** |
| 30/04/2013 | **106.5** |
| 30/04/2013 | **106.8** |
| 30/04/2013 | **107.8** |
| 30/04/2013 | **97.1** |
| 30/04/2013 | **92.4** |
| 30/04/2013 | **101.7** |
| 30/04/2013 | **94.9** |
| 30/04/2013 | **101.8** |
| 30/04/2013 | **109.6** |
| 30/04/2013 | **105.0** |
| 30/04/2013 | **95.9** |
| 30/04/2013 | **112.8** |
| 30/04/2013 | **113.8** |
| 30/04/2013 | **106.8** |
| 30/04/2013 | **84.1** |
| 30/04/2013 | **97.3** |
| 30/04/2013 | **108.6** |
| 30/04/2013 | **106.2** |
| 30/04/2013 | **86.4** |
| 30/04/2013 | **93.2** |
| 30/04/2013 | **96.7** |
| 30/04/2013 | **91.6** |
| 30/04/2013 | **95.4** |
| 30/04/2013 | **97.7** |
| 30/04/2013 | **96.3** |
| 30/04/2013 | **111.3** |
| 30/04/2013 | **94.9** |
| 30/04/2013 | **99.4** |
| 30/04/2013 | **114.9** |
| 30/04/2013 | **111.0** |
| 30/04/2013 | **94.8** |
| 30/04/2013 | **91.1** |
| 30/04/2013 | **87.7** |
| 30/04/2013 | **94.4** |
| 30/04/2013 | **112.1** |
| 30/04/2013 | **110.6** |
| 30/04/2013 | **92.6** |
| 01/05/2013 | **97.0** |
| 01/05/2013 | **87.6** |
| 01/05/2013 | **98.6** |
| 01/05/2013 | **101.3** |
| 01/05/2013 | **100.6** |
| 17/05/2013 | **96.5** |
| 17/05/2013 | **52.8** |
| 17/05/2013 | **98.7** |
| 17/05/2013 | **92.8** |
| 02/08/2013 | **97.8** |
| CDC | **85.7** |

| | | |
|---|---|---|
| 02/08/2013 | **97.9** | CDC |
| CDC | **87** | |
| 02/08/2013 | **100.0** | CDC |
| CDC | **91.4** | |
| 02/08/2013 | **98.1** | CDC |
| 02/08/2013 | **97.2** | |
| CDC | **88.9** | |
| 02/08/2013 | **99.2** | CDC |
| 02/08/2013 | **81.1** | |
| CDC | **94.9** | |
| 02/08/2013 | **90.1** | CDC |
| 02/08/2013 | **98.1** | |
| 02/08/2013 | **99.0** | |
| CDC | **87** | |
| 02/08/2013 | **95.5** | CDC |
| CDC | **97** | |
| 02/08/2013 | **99.4** | CDC |
| CDC | **89.3** | |
| 02/08/2013 | **99.4** | CDC |
| 02/08/2013 | **99.8** | |
| CDC | **92.6** | |
| 02/08/2013 | **99.6** | CDC |
| CDC | **90.9** | |
| 02/08/2013 | **102.3** | CDC |
| CDC | **90.3** | |
| 02/08/2013 | **100.1** | CDC |
| CDC | **88.9** | |
| 02/08/2013 | **100.3** | CDC |
| 02/08/2013 | **99.8** | |
| 02/08/2013 | **84.1** | |
| 02/08/2013 | **87.5** | |
| CDC | **85.0** | |
| 02/08/2013 | **95.5** | CDC |
| CDC | **89** | |
| 02/08/2013 | **97.1** | CDC |
| 02/08/2013 | **88.1** | |
| 02/08/2013 | **95.6** | |
| CDC | **90.2** | |
| 02/08/2013 | **87.9** | CDC |
| CDC | **89** | |
| 02/08/2013 | **93.4** | CDC |
| 02/08/2013 | **106.1** | |
| 02/08/2013 | **97.0** | |
| 05/08/2013 | **110.6** | |
| 05/08/2013 | **109.1** | |
| CDC | **107.4** | |
| 05/08/2013 | **104.5** | CDC |
| CDC | **100.9** | |
| 05/08/2013 | **105.7** | CDC |
| CDC | **105.8** | |

| | | |
|---|---|---|
| 05/08/2013 | **89.5** | CDC |
| CDC | **105.8** | |
| 05/08/2013 | **112.6** | CDC |
| 05/08/2013 | **109.3** | |
| 05/08/2013 | **106.9** | |
| CDC | **96.6** | |
| 05/08/2013 | **103.1** | CDC |
| 05/08/2013 | **110.4** | |
| CDC | **98.6** | |
| 05/08/2013 | **113.0** | CDC |
| CDC | **83.6** | |
| 05/08/2013 | **110.8** | CDC |
| CDC | **93.0** | |
| 05/08/2013 | **86.4** | CDC |
| CDC | **101.0** | |
| 05/08/2013 | **109.5** | CDC |
| CDC | **101.1** | |
| 05/08/2013 | **110.0** | CDC |
| CDC | **110.2** | |
| 05/08/2013 | **111.4** | CDC |
| CDC | **104.4** | |
| 05/08/2013 | **114.0** | CDC |
| CDC | **103.1** | |
| 05/08/2013 | **106.6** | CDC |
| CDC | **89.7** | |
| 05/08/2013 | **109.3** | CDC |
| CDC | **102.5** | |
| 05/08/2013 | **109.9** | CDC |
| 05/08/2013 | **97.1** | |
| 06/06/2013 | **27.9** | |
| 06/06/2013 | **28.3** | |
| 06/06/2013 | **28.5** | |
| 06/06/2013 | **26.6** | |
| 06/06/2013 | **32.4** | |
| 06/06/2013 | **29.2** | |
| 06/06/2013 | **35.0** | |
| 06/06/2013 | **32.2** | |
| 06/06/2013 | **41.7** | |
| 06/06/2013 | **39.9** | |
| 06/06/2013 | **38.4** | |
| 06/06/2013 | **106.2** | |
| | **-** | |
| | **-** | |
| | **-** | |
| | **-** | |
| | **-** | |
| | **-** | |
| - | **-** | |
| - | **-** | |
| | **-** | |

| | | |
|---|---|---|
| | **-** | |
| | **-** | |
| | **-** | |
| | **-** | |
| | **-** | |
| | **-** | |
| | **-** | |
| | **-** | |
| | **-** | |
| | **-** | |
| 16/08/2013 | **102.8** | |
| 16/08/2013 | **99.1** | |
| 16/08/2013 | **101.9** | |
| CDC | **94.7** | |
| 16/08/2013 | **94.8** | CDC |
| 16/08/2013 | **93.3** | |
| CDC | **97.8** | |
| 16/08/2013 | **95.1** | CDC |
| CDC | **87.3** | |
| 16/08/2013 | **102.3** | CDC |
| CDC | **92.2** | |
| 16/08/2013 | **94.0** | CDC |
| 16/08/2013 | **94.8** | |
| 16/08/2013 | **89.7** | |
| CDC | **96.6** | |
| 16/08/2013 | **90.7** | CDC |
| CDC | **94.8** | |
| 16/08/2013 | **84.3** | CDC |
| 16/08/2013 | **90.6** | |
| CDC | **95** | |
| 16/08/2013 | **102.9** | CDC |
| CDC | **93.2** | |
| 16/08/2013 | **102.2** | CDC |
| CDC | **90.5** | |

| mfddate | expdate | packsize | retailprice | comments |
|---|---|---|---|---|
| XXXXXX | XXXXXXX | 24 | 99999999 | |
| XXXXXX | XXXXXXX | 24 | 600 | |
| XXXXXX | XXXXXXX | 24 | 550 | |
| XXXXXX | XXXXXXX | 24 | 550 | |
| XXXXXX | XXXXXXX | 24 | 450 | |
| XXXXXX | XXXXXXX | 24 | 550 | |
| XXXXXX | XXXXXXX | 24 | 250 | |
| XXXXXX | XXXXXXX | 24 | 600 | |
| XXXXXX | XXXXXXX | 24 | 580 | |
| XXXXXX | XXXXXXX | 24 | 700 | |
| XXXXXX | XXXXXXX | 24 | 99999999 | |
| XXXXXX | XXXXXXX | 24 | 980 | |
| XXXXXX | XXXXXXX | 24 | 1000 | |
| XXXXXX | XXXXXXX | 24 | 950 | |
| XXXXXX | XXXXXXX | 24 | 900 | |
| XXXXXX | XXXXXXX | 24 | 1000 | |
| XXXXXX | XXXXXXX | 24 | 900 | |
| XXXXXX | XXXXXXX | 24 | 1000 | |
| XXXXXX | XXXXXXX | 24 | 1100 | |
| XXXXXX | XXXXXXX | 24 | 450 | |
| XXXXXX | XXXXXXX | 24 | 300 | |
| XXXXXX | XXXXXXX | 24 | 400 | |
| XXXXXX | XXXXXXX | 24 | 250 | |
| XXXXXX | XXXXXXX | 24 | 300 | |
| XXXXXX | XXXXXXX | 24 | 300 | |
| XXXXXX | XXXXXXX | 24 | 300 | |
| XXXXXX | XXXXXXX | 24 | 300 | |
| XXXXXX | XXXXXXX | 24 | 350 | |
| XXXXXX | XXXXXXX | 24 | 250 | |
| XXXXXX | XXXXXXX | 24 | 99999999 | |
| XXXXXX | XXXXXXX | 6 | 200 | |
| XXXXXX | XXXXXXX | 12 | 200 | |
| XXXXXX | XXXXXXX | 6 | 250 | |
| XXXXXX | XXXXXXX | 6 | 300 | |
| XXXXXX | XXXXXXX | 18 | 250 | |
| XXXXXX | XXXXXXX | 12 | 250 | |
| XXXXXX | XXXXXXX | 24 | 200 | |
| XXXXXX | XXXXXXX | 24 | 250 | |
| XXXXXX | XXXXXXX | 24 | 250 | |
| XXXXXX | XXXXXXX | 24 | 450 | |
| XXXXXX | XXXXXXX | 24 | 400 | |
| XXXXXX | XXXXXXX | 24 | 200 | |
| XXXXXX | XXXXXXX | 24 | 99999999 | |
| XXXXXX | XXXXXXX | 24 | 99999999 | |
| XXXXXX | XXXXXXX | 18 | 300 | |
| XXXXXX | XXXXXXX | 6 | 350 | |

| | | | | |
|---|---|---|---|---|
| XXXXXX | XXXXXXX | 24 | 700 | Expired |
| XXXXXX | XXXXXXX | 24 | 300 | |
| XXXXXX | XXXXXXX | 24 | 350 | |
| XXXXXX | XXXXXXX | 24 | 300 | |
| XXXXXX | XXXXXXX | 24 | 500 | |
| XXXXXX | XXXXXXX | 24 | 350 | |
| XXXXXX | XXXXXXX | 12 | 150 | only 12 tablets from 24tab pack |
| XXXXXX | XXXXXXX | 24 | 200 | |
| XXXXXX | XXXXXXX | 24 | 250 | |
| XXXXXX | XXXXXXX | 24 | 99999999 | |
| XXXXXX | XXXXXXX | 24 | 600 | |
| XXXXXX | XXXXXXX | 6 | 400 | |
| XXXXXX | XXXXXXX | 12 | 99999999 | |
| XXXXXX | XXXXXXX | 12 | 800 | |
| XXXXXX | XXXXXXX | 12 | 650 | |
| XXXXXX | XXXXXXX | 12 | 620 | |
| XXXXXX | XXXXXXX | 12 | 99999999 | |
| XXXXXX | XXXXXXX | 12 | 550 | |
| XXXXXX | XXXXXXX | 12 | 650 | |
| XXXXXX | XXXXXXX | 6 | 450 | |
| XXXXXX | XXXXXXX | 6 | 99999999 | |
| XXXXXX | XXXXXXX | 6 | 550 | |
| XXXXXX | XXXXXXX | 6 | 700 | |
| XXXXXX | XXXXXXX | 6 | 550 | |
| XXXXXX | XXXXXXX | 6 | 99999999 | |
| XXXXXX | XXXXXXX | 6 | 700 | |
| XXXXXX | XXXXXXX | 6 | 99999999 | |
| XXXXXX | XXXXXXX | 6 | 650 | |
| XXXXXX | XXXXXXX | 6 | 550 | The original pack is in a bad condition |
| XXXXXX | XXXXXXX | 6 | 650 | |
| XXXXXX | XXXXXXX | 6 | 450 | |
| XXXXXX | XXXXXXX | 6 | 99999999 | |
| XXXXXX | XXXXXXX | 6 | 700 | |
| XXXXXX | XXXXXXX | 6 | 500 | |
| XXXXXX | XXXXXXX | 6 | 99999999 | |
| XXXXXX | XXXXXXX | 6 | 700 | |
| XXXXXX | XXXXXXX | 6 | 99999999 | |
| XXXXXX | XXXXXXX | 6 | 650 | |
| XXXXXX | XXXXXXX | 6 | 650 | |
| XXXXXX | XXXXXXX | 6 | 550 | |
| XXXXXX | XXXXXXX | 6 | 700 | |
| XXXXXX | XXXXXXX | 6 | 99999999 | Have an identifying barcode on the blister |
| XXXXXX | XXXXXXX | 6 | 800 | |
| XXXXXX | XXXXXXX | 6 | 750 | |
| XXXXXX | XXXXXXX | 6 | 750 | |
| XXXXXX | XXXXXXX | 6 | 700 | |
| XXXXXX | XXXXXXX | 6 | 750 | |
| XXXXXX | XXXXXXX | 6 | 750 | |
| XXXXXX | XXXXXXX | 6 | 750 | |
| XXXXXX | XXXXXXX | 6 | 750 | |

| | | | | |
|---|---|---|---|---|
| XXXXXX | XXXXXXX | 6 | 750 | original pack is in a bad condition |
| XXXXXX | XXXXXXX | 6 | 750 | |
| XXXXXX | XXXXXXX | 6 | 750 | |
| XXXXXX | XXXXXXX | 6 | 730 | |
| XXXXXX | XXXXXXX | 6 | 850 | |
| XXXXXX | XXXXXXX | 6 | 99999999 | |
| XXXXXX | XXXXXXX | 6 | 99999999 | |
| XXXXXX | XXXXXXX | 6 | 500 | The original pack is in a bad condition |
| XXXXXX | XXXXXXX | 6 | 600 | |
| XXXXXX | XXXXXXX | 6 | 630 | |
| XXXXXX | XXXXXXX | 6 | 650 | |
| XXXXXX | XXXXXXX | 6 | 99999999 | |
| XXXXXX | XXXXXXX | 6 | 600 | |
| XXXXXX | XXXXXXX | 6 | 500 | |
| XXXXXX | XXXXXXX | 6 | 650 | |
| XXXXXX | XXXXXXX | 6 | 600 | |
| XXXXXX | XXXXXXX | 6 | 700 | |
| XXXXXX | XXXXXXX | 6 | 99999999 | |
| XXXXXX | XXXXXXX | 6 | 99999999 | |
| XXXXXX | XXXXXXX | 6 | 550 | |
| XXXXXX | XXXXXXX | 6 | 600 | |
| XXXXXX | XXXXXXX | 6 | 500 | |
| XXXXXX | XXXXXXX | 6 | 99999999 | |
| XXXXXX | XXXXXXX | 6 | 650 | |
| XXXXXX | XXXXXXX | 6 | 600 | |
| XXXXXX | XXXXXXX | 6 | 500 | |
| XXXXXX | XXXXXXX | 6 | 450 | |
| XXXXXX | XXXXXXX | 6 | 550 | |
| XXXXXX | XXXXXXX | 6 | 550 | |
| XXXXXX | XXXXXXX | 1 | 550 | |
| XXXXXX | XXXXXXX | 1 | 600 | |
| XXXXXX | XXXXXXX | 1 | 650 | |
| XXXXXX | XXXXXXX | 1 | 650 | |
| XXXXXX | XXXXXXX | 4 | 250 | |
| XXXXXX | XXXXXXX | 12 | 300 | monotherapy |
| XXXXXX | XXXXXXX | 24 | 450 | |
| XXXXXX | XXXXXXX | 24 | 450 | |
| XXXXXX | XXXXXXX | 6 | 250 | |
| XXXXXX | XXXXXXX | 12 | 320 | |
| XXXXXX | XXXXXXX | 12 | 300 | |
| XXXXXX | XXXXXXX | 12 | 270 | |
| XXXXXX | XXXXXXX | 12 | 300 | monotherapy |
| XXXXXX | XXXXXXX | 12 | 300 | |
| XXXXXX | XXXXXXX | 6 | 500 | |
| XXXXXX | XXXXXXX | 6 | 300 | |
| XXXXXX | XXXXXXX | 6 | 99999999 | The blister is without its original pack |
| XXXXXX | XXXXXXX | 6 | 99999999 | |
| XXXXXX | XXXXXXX | 12 | 99999999 | |
| XXXXXX | XXXXXXX | 12 | 600 | |

| | | | |
|---|---|---|---|
| XXXXXX | XXXXXXX | 12 | 500 |
| XXXXXX | XXXXXXX | 12 | 500 |
| XXXXXX | XXXXXXX | 12 | 500 |
| XXXXXX | XXXXXXX | 12 | 500 |
| XXXXXX | XXXXXXX | 6 | 99999999 |
| XXXXXX | XXXXXXX | 6 | 99999999 |
| XXXXXX | XXXXXXX | 12 | 350 |
| XXXXXX | XXXXXXX | 6 | 700 |
| XXXXXX | XXXXXXX | 6 | 650 |
| XXXXXX | XXXXXXX | 6 | 300 |
| XXXXXX | XXXXXXX | 6 | 270 |
| XXXXXX | XXXXXXX | 6 | 99999999 |
| XXXXXX | XXXXXXX | 6 | 650 |
| XXXXXX | XXXXXXX | 6 | 580 |
| XXXXXX | XXXXXXX | 6 | 99999999 |
| XXXXXX | XXXXXXX | 6 | 850 |
| XXXXXX | XXXXXXX | 6 | 800 |
| XXXXXX | XXXXXXX | 6 | 1000 |
| XXXXXX | XXXXXXX | 6 | 900 |
| XXXXXX | XXXXXXX | 6 | 1100 |
| XXXXXX | XXXXXXX | 6 | 1000 |
| XXXXXX | XXXXXXX | 6 | 99999999 |
| XXXXXX | XXXXXXX | 6 | 99999999 |
| XXXXXX | XXXXXXX | 6 | 99999999 |
| XXXXXX | XXXXXXX | 6 | 500 |
| XXXXXX | XXXXXXX | 3 | 99999999 |
| XXXXXX | XXXXXXX | 3 | 750 |
| XXXXXX | XXXXXXX | 3 | 750 |
| XXXXXX | XXXXXXX | 3 | 730 |
| XXXXXX | XXXXXXX | 3 | 800 |
| XXXXXX | XXXXXXX | 12 | 99999999 |
| XXXXXX | XXXXXXX | 12 | 300 |
| XXXXXX | XXXXXXX | 12 | 300 |
| XXXXXX | XXXXXXX | 12 | 300 |
| XXXXXX | XXXXXXX | 12 | 300 |
| XXXXXX | XXXXXXX | 12 | 350 |
| XXXXXX | XXXXXXX | 12 | 300 |
| XXXXXX | XXXXXXX | 12 | 300 |
| XXXXXX | XXXXXXX | 12 | 300 |
| XXXXXX | XXXXXXX | 12 | 300 |
| XXXXXX | XXXXXXX | 12 | 300 |
| XXXXXX | XXXXXXX | 12 | 300 |
| XXXXXX | XXXXXXX | 12 | 300 |
| XXXXXX | XXXXXXX | 12 | 350 |
| XXXXXX | XXXXXXX | 12 | 99999999 |
| XXXXXX | XXXXXXX | 8 | 99999999 |
| XXXXXX | XXXXXXX | 8 | 500 |
| XXXXXX | XXXXXXX | 8 | 550 |

| | | | | |
|---|---|---|---|---|
| XXXXXX | XXXXXXX | 8 | 550 | |
| XXXXXX | XXXXXXX | 8 | 550 | |
| XXXXXX | XXXXXXX | 8 | 500 | |
| XXXXXX | XXXXXXX | 8 | 500 | |
| XXXXXX | XXXXXXX | 8 | 500 | |
| XXXXXX | XXXXXXX | 8 | 99999999 | MONOTHERAPY |
| XXXXXX | XXXXXXX | 8 | 350 | |

| HPLC (%) me | APIs found b | doseform | manufby | manufcountry |
|---|---|---|---|---|
| - | Artemether-l | tablets | XX | XXX |
| - | Artemether-l | tablets | XX | XXX |
| - | Artemether-l | tablets | XX | XXX |
| - | Artemether-l | tablets | XX | XXX |
| - | Artemether-l | tablets | XX | XXX |
| - | Artemether-l | tablets | XX | XXX |
| - | Artemether-l | tablets | XX | XXX |
| - | Artemether-l | tablets | XX | XXX |
| - | Artemether-l | tablets | XX | XXX |
| - | Artemether-l | tablets | XX | XXX |
| - | Artemether-l | tablets | XX | XXX |
| - | Artemether-l | tablets | XX | XXX |
| - | Artemether-l | tablets | XX | XXX |
| - | Artemether-l | tablets | XX | XXX |
| - | Artemether-l | tablets | XX | XXX |
| - | Artemether-l | tablets | XX | XXX |
| - | Artemether-l | tablets | XX | XXX |
| - | Artemether-l | tablets | XX | XXX |
| - | Artemether-l | tablets | XX | XXX |
| - | Artemether-l | tablets | XX | XXX |
| - | Artemether-l | tablets | XX | XXX |
| - | Artemether-l | tablets | XX | XXX |
| - | Artemether-l | tablets | XX | XXX |
| - | Artemether-l | tablets | XX | XXX |
| - | Artemether-l | tablets | XX | XXX |
| - | Artemether-l | tablets | XX | XXX |
| - | Artemether-l | tablets | XX | XXX |
| - | Artemether-l | tablets | XX | XXX |
| - | Artemether-l | tablets | XX | XXX |
| - | Artemether-l | tablets | XX | XXX |
| - | Artemether-l | tablets | XX | XXX |
| - | Artemether-l | tablets | XX | XXX |
| - | Artemether-l | tablets | XX | XXX |
| - | Artemether-l | tablets | XX | XXX |
| - | Artemether-l | tablets | XX | XXX |
| - | Artemether-l | tablets | XX | XXX |
| - | Artemether-l | tablets | XX | XXX |
| - | Artemether-l | tablets | XX | XXX |

| | | | |
|---|---|---|---|
| - | Artemether-l tablets | XX | XXX |
| - | Artemether-l tablets | XX | XXX |
| - | Artemether-l tablets | XX | XXX |
| - | Artemether-l tablets | XX | XXX |
| - | Artemether-l tablets | XX | XXX |
| - | Artemether-l tablets | XX | XXX |
| - | Artemether-l tablets | XX | XXX |
| - | Artemether-l tablets | XX | XXX |
| - | Artemether-l tablets | XX | XXX |
| - | Artemether-l tablets | XX | XXX |
| - | Artemether-l tablets | XX | XXX |
| - | Artemether-l tablets | XX | XXX |
| - | Artemether-l tablets | XX | XXX |
| - | Artemether-l tablets | XX | XXX |
| - | Artemether-l tablets | XX | XXX |
| - | Artemether-l tablets | XX | XXX |
| - | Artemether-l tablets | XX | XXX |
| - | Artemether-l tablets | XX | XXX |
| - | Artemether-l tablets | XX | XXX |
| - | Artemether-l tablets | XX | XXX |
| - | Artemether-l tablets | XX | XXX |
| - | Artemether-l tablets | XX | XXX |
| - | Artemether-l tablets | XX | XXX |
| - | Artemether-l tablets | XX | XXX |
| - | Artemether-l tablets | XX | XXX |
| - | Artemether-l tablets | XX | XXX |
| - | Artemether-l tablets | XX | XXX |
| - | Artemether-l tablets | XX | XXX |
| - | Artemether-l tablets | XX | XXX |
| - | Artemether-l tablets | XX | XXX |
| - | Artemether-l tablets | XX | XXX |
| - | Artemether-l tablets | XX | XXX |
| - | Artemether-l tablets | XX | XXX |
| - | Artemether-l tablets | XX | XXX |
| - | Artemether-l tablets | XX | XXX |
| - | Artemether-l tablets | XX | XXX |
| - | Artemether-l tablets | XX | XXX |
| - | Artemether-l tablets | XX | XXX |
| - | Artemether-l tablets | XX | XXX |
| - | Artemether-l tablets | XX | XXX |
| - | Artemether-l tablets | XX | XXX |
| - | Artemether-l tablets | XX | XXX |
| - | Artemether-l tablets | XX | XXX |
| - | Artemether-l tablets | XX | XXX |
| - | Artemether-l tablets | XX | XXX |
| - | Artemether-l tablets | XX | XXX |
| - | Artemether-l tablets | XX | XXX |
| - | Artemether-l tablets | XX | XXX |
| - | Artemether-l tablets | XX | XXX |
| - | Artemether-l tablets | XX | XXX |

| | | | |
|---|---|---|---|
| - | Artemether-l tablets | XX | XXX |
| - | Artemether-l tablets | XX | XXX |
| - | Artemether-l tablets | XX | XXX |
| - | Artemether-l tablets | XX | XXX |
| - | Artemether-l tablets | XX | XXX |
| - | Artemether-l tablets | XX | XXX |
| - | Artemether-l tablets | XX | XXX |
| - | Artemether-l tablets | XX | XXX |
| - | Artemether-l tablets | XX | XXX |
| - | Artemether-l tablets | XX | XXX |
| - | Artemether-l tablets | XX | XXX |
| - | Artemether-l tablets | XX | XXX |
| - | Artemether-l tablets | XX | XXX |
| - | Artemether-l tablets | XX | XXX |
| - | Artemether-l tablets | XX | XXX |
| - | Artemether-l tablets | XX | XXX |
| - | Artemether-l tablets | XX | XXX |
| - | Artemether-l tablets | XX | XXX |
| - | Artemether-l tablets | XX | XXX |
| - | Artemether-l tablets | XX | XXX |
| - | Artemether-l tablets | XX | XXX |
| - | Artemether-l tablets | XX | XXX |
| - | Artemether-l tablets | XX | XXX |
| - | Artemether-l tablets | XX | XXX |
| - | Artemether-l tablets | XX | XXX |
| - | Artemether-l tablets | XX | XXX |
| - | Artemether-l tablets | XX | XXX |
| - | Artemether-l tablets | XX | XXX |
| - | Artemether-l tablets | XX | XXX |
| - | Artemether-l tablets | XX | XXX |
| - | Artemether-l tablets | XX | XXX |
| - | Artemether-l tablets | XX | XXX |
| - | Artemether-l tablets | XX | XXX |
| - | Artemether-l tablets | XX | XXX |
| - | Artemether-l tablets | XX | XXX |
| - | Artemether-l tablets | XX | XXX |
| - | Artemether-l tablets | XX | XXX |
| - | Artemether-l tablets | XX | XXX |
| - | Artemether-l tablets | XX | XXX |
| - | Artemether-l tablets | XX | XXX |
| - | Artemether-l tablets | XX | XXX |
| - | Artemether-l tablets | XX | XXX |
| - | Artemether-l tablets | XX | XXX |
| - | Artemether-l tablets | XX | XXX |
| - | Artemether-l tablets | XX | XXX |
| - | Artemether-l tablets | XX | XXX |
| - | Artemether-l tablets | XX | XXX |
| - | Artemether-l tablets | XX | XXX |
| - | Artemether-l tablets | XX | XXX |
| - | Artemether-l tablets | XX | XXX |

| | | | |
|---|---|---|---|
| - | Artemether-l tablets | XX | XXX |
| - | Artemether-l tablets | XX | XXX |
| - | Artemether-l tablets | XX | XXX |
| - | Artemether-l tablets | XX | XXX |
| - | Artemether-l tablets | XX | XXX |
| - | Artemether-l tablets | XX | XXX |
| - | Artemether-l tablets | XX | XXX |
| - | Artemether-l tablets | XX | XXX |
| - | Artemether-l tablets | XX | XXX |
| - | Artemether-l tablets | XX | XXX |
| - | Artemether-l tablets | XX | XXX |
| - | Artemether-l tablets | XX | XXX |
| - | Artemether-l tablets | XX | XXX |
| - | Artemether-l tablets | XX | XXX |
| - | Artemether-l tablets | XX | XXX |
| - | Artemether-l tablets | XX | XXX |
| - | Artemether-l tablets | XX | XXX |
| - | Artemether-l tablets | XX | XXX |
| - | Artemether-l tablets | XX | XXX |
| - | Artemether-l tablets | XX | XXX |
| - | Artemether-l tablets | XX | XXX |
| - | Artemether-l tablets | XX | XXX |
| - | Artemether-l tablets | XX | XXX |
| - | Artemether-l tablets | XX | XXX |
| - | Artemether-l tablets | XX | XXX |
| - | Artemether-l tablets | XX | XXX |
| - | Artemether-l tablets | XX | XXX |
| - | Artemether-l tablets | XX | XXX |
| - | Artemether-l tablets | XX | XXX |
| - | Artemether-l tablets | XX | XXX |
| - | Artemether-l tablets | XX | XXX |
| - | Artemether-l tablets | XX | XXX |
| - | Artemether-l tablets | XX | XXX |
| - | Artemether-l tablets | XX | XXX |
| - | Artemether-l tablets | XX | XXX |
| - | Artemether-l tablets | XX | XXX |
| - | Artemether-l tablets | XX | XXX |
| - | Artemether-l tablets | XX | XXX |
| - | Artemether-l tablets | XX | XXX |
| - | Artemether-l tablets | XX | XXX |
| - | Artemether-l tablets | XX | XXX |
| - | Artemether-l tablets | XX | XXX |
| - | Artemether-l tablets | XX | XXX |
| - | Artemether-l tablets | XX | XXX |
| - | Artemether-l tablets | XX | XXX |
| - | Artemether-l tablets | XX | XXX |
| - | Artemether-l tablets | XX | XXX |
| - | Artemether-l tablets | XX | XXX |
| - | Artemether-l tablets | XX | XXX |
| - | Artemether-l tablets | XX | XXX |

| | | | |
|---|---|---|---|
| - | Artemether-l tablets | XX | XXX |
| - | Artemether-l tablets | XX | XXX |
| - | Artemether-l tablets | XX | XXX |
| - | Artemether-l tablets | XX | XXX |
| - | Artemether-l tablets | XX | XXX |
| - | Artemether-l tablets | XX | XXX |
| - | Artemether-l tablets | XX | XXX |
| - | Artemether-l tablets | XX | XXX |
| - | Artemether-l tablets | XX | XXX |
| - | Artemether-l tablets | XX | XXX |
| - | Artemether-l tablets | XX | XXX |
| - | Artemether-l tablets | XX | XXX |
| - | Artemether-l tablets | XX | XXX |
| - | Artemether-l tablets | XX | XXX |
| - | Artemether-l tablets | XX | XXX |
| - | Artemether-l tablets | XX | XXX |
| - | Artemether-l tablets | XX | XXX |
| - | Artemether-l tablets | XX | XXX |
| - | Artemether-l tablets | XX | XXX |
| - | Artemether-l tablets | XX | XXX |
| - | Artemether-l tablets | XX | XXX |
| - | Artemether-l tablets | XX | XXX |
| - | Artemether-l tablets | XX | XXX |
| - | Artemether-l tablets | XX | XXX |
| - | Artemether-l tablets | XX | XXX |
| - | Artemether-l tablets | XX | XXX |
| - | Artemether-l tablets | XX | XXX |
| - | Artemether-l tablets | XX | XXX |
| - | Artemether-l tablets | XX | XXX |
| - | Artemether-l tablets | XX | XXX |
| - | Artemether-l tablets | XX | XXX |
| - | Artemether-l tablets | XX | XXX |
| - | Artemether-l tablets | XX | XXX |
| - | Artemether-l tablets | XX | XXX |
| - | Artemether-l tablets | XX | XXX |
| - | Artemether-l tablets | XX | XXX |
| - | Artemether-l tablets | XX | XXX |
| - | Artemether-l tablets | XX | XXX |
| - | Artemether-l tablets | XX | XXX |
| - | Artemether-l tablets | XX | XXX |
| - | Artemether-l tablets | XX | XXX |
| - | Artemether-l tablets | XX | XXX |
| - | Artemether-l tablets | XX | XXX |
| - | Artemether-l tablets | XX | XXX |
| - | Artemether-l tablets | XX | XXX |
| - | Artemether-l tablets | XX | XXX |
| - | Artemether-l tablets | XX | XXX |
| - | Artemether-l tablets | XX | XXX |
| - | Artemether-l tablets | XX | XXX |
| - | Artemether-l tablets | XX | XXX |

| | | | |
|---|---|---|---|
| - | Artemether-l tablets | XX | XXX |
| - | Artemether-l tablets | XX | XXX |
| - | Artemether-l tablets | XX | XXX |
| - | Artemether-l tablets | XX | XXX |
| - | Artemether-l tablets | XX | XXX |
| - | Artemether-l tablets | XX | XXX |
| - | Artemether-l tablets | XX | XXX |
| - | Artemether-l tablets | XX | XXX |
| - | Artemether-l tablets | XX | XXX |
| - | Artemether-l tablets | XX | XXX |
| - | Artemether-l tablets | XX | XXX |
| - | Artemether-l tablets | XX | XXX |
| - | Artemether-l tablets | XX | XXX |
| - | Artemether-l tablets | XX | XXX |
| - | Artemether-l tablets | XX | XXX |
| - | Artemether-l tablets | XX | XXX |
| - | Artemether-l tablets | XX | XXX |
| - | Artemether-l tablets | XX | XXX |
| - | Artemether-l tablets | XX | XXX |
| - | Artemether-l tablets | XX | XXX |
| - | Artemether-l tablets | XX | XXX |
| - | Artemether-l tablets | XX | XXX |
| - | Artemether-l tablets | XX | XXX |
| - | Artemether-l tablets | XX | XXX |
| - | Artemether-l tablets | XX | XXX |
| - | Artemether-l tablets | XX | XXX |
| - | Artemether-l tablets | XX | XXX |
| - | Artemether-l tablets | XX | XXX |
| - | Artemether-l tablets | XX | XXX |
| - | Artemether-l tablets | XX | XXX |
| - | Artemether-l tablets | XX | XXX |
| - | Artemether-l tablets | XX | XXX |
| - | Artemether-l tablets | XX | XXX |
| - | Artemether-l tablets | XX | XXX |
| - | Artemether-l tablets | XX | XXX |
| - | Artemether-l tablets | XX | XXX |
| - | Artemether-l tablets | XX | XXX |
| - | Artemether-l tablets | XX | XXX |
| - | Artemether-l tablets | XX | XXX |
| - | Artemether-l tablets | XX | XXX |
| - | Artemether-l tablets | XX | XXX |
| - | Artemether-l tablets | XX | XXX |
| - | Artemether-l tablets | XX | XXX |
| - | Artemether-l tablets | XX | XXX |
| - | Artemether-l tablets | XX | XXX |
| - | Artemether-l tablets | XX | XXX |
| - | Artemether-l tablets | XX | XXX |
| - | CHLORZOXAZ tablets | XX | XXX |
| - | Artemether-l tablets | XX | XXX |
| - | Artemether-l tablets | XX | XXX |

| | | | |
|---|---|---|---|
| - | Artemether-l tablets | XX | XXX |
| - | Artemether-l tablets | XX | XXX |
| - | Artemether-l tablets | XX | XXX |
| - | Artemether-l tablets | XX | XXX |
| - | Artemether-l tablets | XX | XXX |
| - | Artemether-l tablets | XX | XXX |
| - | Artemether-l tablets | XX | XXX |
| - | Artemether-l tablets | XX | XXX |
| - | Artemether-l tablets | XX | XXX |
| - | Artemether-l tablets | XX | XXX |
| - | Artemether-l tablets | XX | XXX |
| - | Artemether-l tablets | XX | XXX |
| - | Artemether-l tablets | XX | XXX |
| - | Artemether-l tablets | XX | XXX |
| - | Artemether-l tablets | XX | XXX |
| - | Artemether-l tablets | XX | XXX |
| - | Artemether-l tablets | XX | XXX |
| - | Artemether-l tablets | XX | XXX |
| - | Artemether-l tablets | XX | XXX |
| - | Artemether-l tablets | XX | XXX |
| - | Artemether-l tablets | XX | XXX |
| - | Artemether-l tablets | XX | XXX |
| - | Artemether-l tablets | XX | XXX |
| - | Artemether-l tablets | XX | XXX |
| - | Artemether-l tablets | XX | XXX |
| - | Artemether-l tablets | XX | XXX |
| - | Artemether-l tablets | XX | XXX |
| - | Artemether-l tablets | XX | XXX |
| - | Artemether-l tablets | XX | XXX |
| - | Artemether-l tablets | XX | XXX |
| - | Artemether-l tablets | XX | XXX |
| - | CHLORZOXAZ tablets | XX | XXX |
| - | Artemether-l tablets | XX | XXX |
| - | Artemether-l tablets | XX | XXX |
| - | Artemether-l tablets | XX | XXX |
| - | Artemether-l tablets | XX | XXX |
| - | Artemether-l tablets | XX | XXX |
| - | Artemether-l tablets | XX | XXX |
| - | Artemether-l tablets | XX | XXX |
| - | Artemether-l tablets | XX | XXX |
| - | Artemether-l tablets | XX | XXX |
| - | Artemether-l tablets | XX | XXX |
| - | Artemether-l tablets | XX | XXX |
| - | Artemether-l tablets | XX | XXX |
| - | Artemether-l tablets | XX | XXX |
| - | Artemether-l tablets | XX | XXX |
| - | Artemether-l tablets | XX | XXX |
| - | Artemether-l tablets | XX | XXX |
| - | Artemether-l tablets | XX | XXX |
| - | Artemether-l tablets | XX | XXX |

| | | | |
|---|---|---|---|
| - | Artemether-l tablets | XX | XXX |
| - | Artemether-l tablets | XX | XXX |
| - | Artemether-l tablets | XX | XXX |
| - | Artemether-l tablets | XX | XXX |
| - | Artemether-l tablets | XX | XXX |
| - | Artemether-l tablets | XX | XXX |
| - | Artemether-l tablets | XX | XXX |
| - | Artemether-l tablets | XX | XXX |
| - | Artemether-l tablets | XX | XXX |
| - | Artemether-l tablets | XX | XXX |
| - | Artemether-l tablets | XX | XXX |
| - | Artemether-l tablets | XX | XXX |
| - | Artemether-l tablets | XX | XXX |
| - | Artemether-l tablets | XX | XXX |
| - | Artemether-l tablets | XX | XXX |
| - | Artemether-l tablets | XX | XXX |
| - | Artemether-l tablets | XX | XXX |
| - | Artemether-l tablets | XX | XXX |
| - | Artemether-l tablets | XX | XXX |
| - | Artemether-l tablets | XX | XXX |
| - | Artemether-l tablets | XX | XXX |
| - | Artemether-l tablets | XX | XXX |
| - | Artemether-l tablets | XX | XXX |
| - | Artemether-l tablets | XX | XXX |
| - | Artemether-l tablets | XX | XXX |
| - | Artemether-l tablets | XX | XXX |
| - | Artemether-l tablets | XX | XXX |
| - | Artemether-l tablets | XX | XXX |
| - | Artemether-l tablets | XX | XXX |
| - | Artemether-l tablets | XX | XXX |
| - | Artemether-l tablets | XX | XXX |
| - | Artemether-l tablets | XX | XXX |
| - | Artemether-l tablets | XX | XXX |
| - | Artemether-l tablets | XX | XXX |
| - | Artemether-l tablets | XX | XXX |
| - | Artemether-l tablets | XX | XXX |
| - | Artemether-l tablets | XX | XXX |
| - | Artemether-l tablets | XX | XXX |
| - | Artemether-l tablets | XX | XXX |
| - | Artemether-l tablets | XX | XXX |
| - | Artemether-l tablets | XX | XXX |
| - | Artemether-l tablets | XX | XXX |
| - | Artemether-l tablets | XX | XXX |
| - | Artemether-l tablets | XX | XXX |
| - | Artemether-l tablets | XX | XXX |
| - | Artemether-l tablets | XX | XXX |
| - | Artemether-l tablets | XX | XXX |
| - | Artemether-l tablets | XX | XXX |
| - | Artemether-l tablets | XX | XXX |
| - | Artemether-l tablets | XX | XXX |

| | | | |
|---|---|---|---|
| - | Artemether-l tablets | XX | XXX |
| - | Artemether-l tablets | XX | XXX |
| - | Artemether-l tablets | XX | XXX |
| - | Artemether-l tablets | XX | XXX |
| - | Artemether-l tablets | XX | XXX |
| - | Artemether-l tablets | XX | XXX |
| - | Artemether-l tablets | XX | XXX |
| - | Artemether-l tablets | XX | XXX |
| - | Artemether-l tablets | XX | XXX |
| - | Artemether-l tablets | XX | XXX |
| - | Artemether-l tablets | XX | XXX |
| - | Artemether-l tablets | XX | XXX |
| - | Artemether-l tablets | XX | XXX |
| - | Artemether-l tablets | XX | XXX |
| - | Artemether-l tablets | XX | XXX |
| - | Artemether-l tablets | XX | XXX |
| - | Artemether-l tablets | XX | XXX |
| - | Artemether-l tablets | XX | XXX |
| - | Artemether-l tablets | XX | XXX |
| - | Artemether-l tablets | XX | XXX |
| - | Artemether-l tablets | XX | XXX |
| - | Artemether-l tablets | XX | XXX |
| - | Artemether-l tablets | XX | XXX |
| - | Artemether-l tablets | XX | XXX |
| - | Artemether-l tablets | XX | XXX |
| - | Artemether-l tablets | XX | XXX |
| - | Artemether-l tablets | XX | XXX |
| - | Artemether-l tablets | XX | XXX |
| - | Artemether-l tablets | XX | XXX |
| - | Artemether-l tablets | XX | XXX |
| - | Artemether-l tablets | XX | XXX |
| - | Artemether-l tablets | XX | XXX |
| - | Artemether-l tablets | XX | XXX |
| - | Artemether-l tablets | XX | XXX |
| - | Artemether-l tablets | XX | XXX |
| - | Artemether-l tablets | XX | XXX |
| - | Artemether-l tablets | XX | XXX |
| - | Artemether-l tablets | XX | XXX |
| - | Artemether-l tablets | XX | XXX |
| - | Artemether-l tablets | XX | XXX |
| - | Artemether-l tablets | XX | XXX |
| - | Artemether-l tablets | XX | XXX |
| - | Artemether-l tablets | XX | XXX |
| - | Artemether-l tablets | XX | XXX |
| - | Artemether-l tablets | XX | XXX |
| - | Artemether-l tablets | XX | XXX |
| - | Artemether-l tablets | XX | XXX |
| - | Artemether-l tablets | XX | XXX |
| - | Artemether-l tablets | XX | XXX |
| - | Artemether-l tablets | XX | XXX |

| | | | |
|---|---|---|---|
| - | Artemether-l tablets | XX | XXX |
| - | Artemether-l tablets | XX | XXX |
| - | Artemether-l tablets | XX | XXX |
| - | Artemether-l tablets | XX | XXX |
| - | Artemether-l tablets | XX | XXX |
| - | Artemether-l tablets | XX | XXX |
| - | Artemether-l tablets | XX | XXX |
| - | Artemether-l tablets | XX | XXX |
| - | Artemether-l tablets | XX | XXX |
| - | Artemether-l tablets | XX | XXX |
| - | Artemether-l tablets | XX | XXX |
| - | Artemether-l tablets | XX | XXX |
| - | Artemether-l tablets | XX | XXX |
| - | Artemether-l tablets | XX | XXX |
| - | Artemether-l tablets | XX | XXX |
| - | Artemether-l tablets | XX | XXX |
| - | Artemether-l tablets | XX | XXX |
| - | Artemether-l tablets | XX | XXX |
| - | Artemether-l tablets | XX | XXX |
| - | Artemether-l tablets | XX | XXX |
| - | Artemether-l tablets | XX | XXX |
| - | Artemether-l tablets | XX | XXX |
| - | Artemether-l tablets | XX | XXX |
| - | Artemether-l tablets | XX | XXX |
| - | Artemether-l tablets | XX | XXX |
| - | Artemether-l tablets | XX | XXX |
| - | Artemether-l tablets | XX | XXX |
| - | Artesunate tablets | XX | XXX |
| - | Artemether-l tablets | XX | XXX |
| - | Artemether-l tablets | XX | XXX |
| - | Artemether-l tablets | XX | XXX |
| - | Artemether-l tablets | XX | XXX |
| - | Artemether-l tablets | XX | XXX |
| - | Artemether-l tablets | XX | XXX |
| - | Artemether-l tablets | XX | XXX |
| - | Artemether-l tablets | XX | XXX |
| - | Artemether-l tablets | XX | XXX |
| - | Artemether-l tablets | XX | XXX |
| - | Artemether-l tablets | XX | XXX |
| - | Artemether-l tablets | XX | XXX |
| - | Artemether-l tablets | XX | XXX |
| - | Artemether-l tablets | XX | XXX |
| - | Artemether-l tablets | XX | XXX |
| - | Artemether-l tablets | XX | XXX |
| - | Artemether-l tablets | XX | XXX |
| - | Artemether-l tablets | XX | XXX |
| - | Artemether-l tablets | XX | XXX |
| - | Artemether-l tablets | XX | XXX |
| - | Artemether-l tablets | XX | XXX |
| - | Artemether-l tablets | XX | XXX |

| | | | |
|---|---|---|---|
| - | Artemether-l tablets | XX | XXX |
| - | Artemether-l tablets | XX | XXX |
| - | Artemether-l tablets | XX | XXX |
| - | Artemether-l tablets | XX | XXX |
| - | Artemether-l tablets | XX | XXX |
| - | Artemether-l tablets | XX | XXX |
| - | Artemether-l tablets | XX | XXX |
| - | Artemether-l tablets | XX | XXX |
| - | Artemether-l tablets | XX | XXX |
| - | Artemether-l tablets | XX | XXX |
| - | Artemether-l tablets | XX | XXX |
| - | Artemether-l tablets | XX | XXX |
| - | Artemether-l tablets | XX | XXX |
| - | Artemether-l tablets | XX | XXX |
| - | Artemether-l tablets | XX | XXX |
| - | Artemether-l tablets | XX | XXX |
| - | Artemether-l tablets | XX | XXX |
| - | Artemether-l tablets | XX | XXX |
| - | Artemether-l tablets | XX | XXX |
| - | Artemether-l tablets | XX | XXX |
| - | Artemether-l tablets | XX | XXX |
| - | Artemether-l tablets | XX | XXX |
| - | Artemether-l tablets | XX | XXX |
| - | Artemether-l tablets | XX | XXX |
| - | Artemether-l tablets | XX | XXX |
| - | Artemether-l tablets | XX | XXX |
| - | Artemether-l tablets | XX | XXX |
| - | Artemether-l tablets | XX | XXX |
| - | Artemether-l tablets | XX | XXX |
| - | Artemether-l tablets | XX | XXX |
| - | Artemether-l tablets | XX | XXX |
| - | Artemether-l tablets | XX | XXX |
| - | Artemether-l tablets | XX | XXX |
| - | Artemether-l tablets | XX | XXX |
| - | Artemether-l tablets | XX | XXX |
| - | Artemether-l tablets | XX | XXX |
| - | Artemether-l tablets | XX | XXX |
| - | Artemether-l tablets | XX | XXX |
| - | Artemether-l tablets | XX | XXX |
| - | Artemether-l tablets | XX | XXX |
| - | Artemether-l tablets | XX | XXX |
| - | Artemether-l tablets | XX | XXX |
| - | Artemether-l tablets | XX | XXX |
| - | Artemether-l tablets | XX | XXX |
| - | Artemether-l tablets | XX | XXX |
| - | Artemether-l tablets | XX | XXX |
| - | Artemether-l tablets | XX | XXX |
| - | Artemether-l tablets | XX | XXX |
| - | Artemether-l tablets | XX | XXX |
| - | Artemether-l tablets | XX | XXX |

| | | | |
|---|---|---|---|
| - | Artemether-l tablets | XX | XXX |
| - | Artemether-l tablets | XX | XXX |
| - | Artemether-l tablets | XX | XXX |
| - | Artemether-l tablets | XX | XXX |
| - | Artemether-l tablets | XX | XXX |
| - | Artemether-l tablets | XX | XXX |
| - | Artemether-l tablets | XX | XXX |
| - | Artemether-l tablets | XX | XXX |
| - | Artemether-l tablets | XX | XXX |
| - | Artemether-l tablets | XX | XXX |
| - | Artemether-l tablets | XX | XXX |
| - | Artemether-l tablets | XX | XXX |
| - | Artemether-l tablets | XX | XXX |
| - | Artemether-l tablets | XX | XXX |
| - | Artemether-l tablets | XX | XXX |
| - | Artemether-l tablets | XX | XXX |
| - | Artemether-l tablets | XX | XXX |
| - | Artemether-l tablets | XX | XXX |
| - | Artemether-l tablets | XX | XXX |
| - | Artemether-l tablets | XX | XXX |
| - | Artemether-l tablets | XX | XXX |
| - | Artemether-l tablets | XX | XXX |
| - | Artemether-l tablets | XX | XXX |
| - | Artemether-l tablets | XX | XXX |
| - | Artemether-l tablets | XX | XXX |
| - | Artemether-l tablets | XX | XXX |
| - | Artemether-l tablets | XX | XXX |
| - | Artemether-l tablets | XX | XXX |
| - | Artemether-l tablets | XX | XXX |
| - | Artemether-l tablets | XX | XXX |
| - | Artemether-l tablets | XX | XXX |
| - | Artemether-l tablets | XX | XXX |
| - | Artemether-l tablets | XX | XXX |
| - | Artemether-l tablets | XX | XXX |
| - | Artemether-l tablets | XX | XXX |
| - | Artemether-l tablets | XX | XXX |
| - | Artemether-l tablets | XX | XXX |
| - | Artemether-l tablets | XX | XXX |
| - | Artemether-l tablets | XX | XXX |
| - | Artemether-l tablets | XX | XXX |
| - | Artemether-l tablets | XX | XXX |
| - | Artemether-l tablets | XX | XXX |
| - | Artemether-l tablets | XX | XXX |
| - | Artemether-l tablets | XX | XXX |
| - | Artemether-l tablets | XX | XXX |
| - | Artemether-l tablets | XX | XXX |
| - | Artemether-l tablets | XX | XXX |
| - | Artemether-l tablets | XX | XXX |
| - | Artemether-l tablets | XX | XXX |
| - | Artemether-l tablets | XX | XXX |

| | | | |
|---|---|---|---|
| - | Artemether-l tablets | XX | XXX |
| - | Artemether-l tablets | XX | XXX |
| - | Artemether-l tablets | XX | XXX |
| - | Artemether-l tablets | XX | XXX |
| - | Artemether-l tablets | XX | XXX |
| - | Artemether-l tablets | XX | XXX |
| - | Artemether-l tablets | XX | XXX |
| - | Artemether-l tablets | XX | XXX |
| - | Artemether-l tablets | XX | XXX |
| - | Artemether-l tablets | XX | XXX |
| - | Artemether-l tablets | XX | XXX |
| - | Artemether-l tablets | XX | XXX |
| - | Artemether-l tablets | XX | XXX |
| - | Artemether-l tablets | XX | XXX |
| - | Artemether-l tablets | XX | XXX |
| - | Artemether-l tablets | XX | XXX |
| - | Artemether-l tablets | XX | XXX |
| - | Artemether-l tablets | XX | XXX |
| - | Artemether-l tablets | XX | XXX |
| - | Artemether-l tablets | XX | XXX |
| - | Artemether-l tablets | XX | XXX |
| - | Artemether-l tablets | XX | XXX |
| - | Artemether-l tablets | XX | XXX |
| - | Artemether-l tablets | XX | XXX |
| - | Artemether-l tablets | XX | XXX |
| - | Artemether-l tablets | XX | XXX |
| - | Artemether-l tablets | XX | XXX |
| - | Artemether-l tablets | XX | XXX |
| - | Artemether-l tablets | XX | XXX |
| - | Artemether-l tablets | XX | XXX |
| - | Artemether-l tablets | XX | XXX |
| - | Artemether-l tablets | XX | XXX |
| - | Artemether-l tablets | XX | XXX |
| - | Artemether-l tablets | XX | XXX |
| - | Artemether-l tablets | XX | XXX |
| - | Artemether-l tablets | XX | XXX |
| - | Artemether-l tablets | XX | XXX |
| - | Artemether-l tablets | XX | XXX |
| - | Artemether-l tablets | XX | XXX |
| - | Artemether-l tablets | XX | XXX |
| - | Artemether-l tablets | XX | XXX |
| - | Artemether-l tablets | XX | XXX |
| - | Artemether-l tablets | XX | XXX |
| - | Artemether-l tablets | XX | XXX |
| - | Artemether-l tablets | XX | XXX |
| - | Artemether-l tablets | XX | XXX |
| - | Artemether-l tablets | XX | XXX |
| - | Artemether-l tablets | XX | XXX |
| - | Artemether-l tablets | XX | XXX |
| - | Artemether-l tablets | XX | XXX |

| | | | |
|---|---|---|---|
| - | Artemether-l tablets | XX | XXX |
| - | Artemether-l tablets | XX | XXX |
| - | Artemether-l tablets | XX | XXX |
| - | Artemether-l tablets | XX | XXX |
| - | Artemether-l tablets | XX | XXX |
| - | Artemether-l tablets | XX | XXX |
| - | Artemether-l tablets | XX | XXX |
| - | Artemether-l tablets | XX | XXX |
| - | Artemether-l tablets | XX | XXX |
| - | Artemether-l tablets | XX | XXX |
| - | Artemether-l tablets | XX | XXX |
| - | Artemether-l tablets | XX | XXX |
| - | Artemether-l tablets | XX | XXX |
| - | Artemether-l tablets | XX | XXX |
| - | Artemether-l tablets | XX | XXX |
| - | Artemether-l tablets | XX | XXX |
| - | Artemether-l tablets | XX | XXX |
| - | Artemether-l tablets | XX | XXX |
| - | Artemether-l tablets | XX | XXX |
| - | Artemether-l tablets | XX | XXX |
| - | Artemether-l tablets | XX | XXX |
| - | Artemether-l tablets | XX | XXX |
| - | Artemether-l tablets | XX | XXX |
| - | Artemether-l tablets | XX | XXX |
| - | Artemether-l tablets | XX | XXX |
| - | Artemether-l tablets | XX | XXX |
| - | Artemether-l tablets | XX | XXX |
| - | Artemether-l tablets | XX | XXX |
| - | Artemether-l tablets | XX | XXX |
| - | Artemether-l tablets | XX | XXX |
| - | Artemether-l tablets | XX | XXX |
| - | Artemether-l tablets | XX | XXX |
| - | Artemether-l tablets | XX | XXX |
| - | Artemether-l tablets | XX | XXX |
| - | Artemether-l tablets | XX | XXX |
| - | Artemether-l tablets | XX | XXX |
| - | Artemether-l tablets | XX | XXX |
| - | Artemether-l tablets | XX | XXX |
| - | Artemether-l tablets | XX | XXX |
| - | Artemether-l tablets | XX | XXX |
| - | Artemether-l tablets | XX | XXX |
| - | Artemether-l tablets | XX | XXX |
| - | Artemether-l tablets | XX | XXX |
| - | Artemether-l tablets | XX | XXX |
| - | Artemether-l tablets | XX | XXX |
| - | Artemether-l tablets | XX | XXX |
| - | Artemether-l tablets | XX | XXX |
| - | Artemether-l tablets | XX | XXX |
| - | Artemether-l tablets | XX | XXX |
| - | Artemether-l tablets | XX | XXX |

| | | | |
|---|---|---|---|
| - | Artemether-l tablets | XX | XXX |
| - | Artemether-l tablets | XX | XXX |
| - | Artemether-l tablets | XX | XXX |
| - | Artemether-l tablets | XX | XXX |
| - | Artemether-l tablets | XX | XXX |
| - | Artemether-l tablets | XX | XXX |
| - | Artemether-l tablets | XX | XXX |
| - | Artemether-l tablets | XX | XXX |
| - | Artemether-l tablets | XX | XXX |
| - | Artemether-l tablets | XX | XXX |
| - | Artemether-l tablets | XX | XXX |
| - | Artemether-l tablets | XX | XXX |
| - | Artemether-l tablets | XX | XXX |
| - | Artemether-l tablets | XX | XXX |
| - | Artemether-l tablets | XX | XXX |
| - | Artemether-l tablets | XX | XXX |
| - | Artemether-l tablets | XX | XXX |
| - | Artemether-l tablets | XX | XXX |
| - | Artemether-l tablets | XX | XXX |
| - | Artemether-l tablets | XX | XXX |
| - | Artemether-l tablets | XX | XXX |
| - | Artemether-l tablets | XX | XXX |
| - | Artemether-l tablets | XX | XXX |
| - | Artemether-l tablets | XX | XXX |
| - | Artemether-l tablets | XX | XXX |
| - | Artemether-l tablets | XX | XXX |
| - | Artemether-l tablets | XX | XXX |
| - | Artemether-l tablets | XX | XXX |
| - | Artemether-l tablets | XX | XXX |
| - | Artemether-l tablets | XX | XXX |
| - | Artemether-l tablets | XX | XXX |
| - | Artemether-l tablets | XX | XXX |
| - | Artemether-l tablets | XX | XXX |
| - | Artemether-l tablets | XX | XXX |
| - | Artemether-l tablets | XX | XXX |
| - | Artemether-l tablets | XX | XXX |
| - | Artemether-l tablets | XX | XXX |
| - | Artemether-l tablets | XX | XXX |
| - | Artemether-l tablets | XX | XXX |
| - | Artemether-l tablets | XX | XXX |
| - | Artemether-l tablets | XX | XXX |
| - | Artemether-l tablets | XX | XXX |
| - | Artemether-l tablets | XX | XXX |
| - | Artemether-l tablets | XX | XXX |
| - | Artemether-l tablets | XX | XXX |
| - | Artemether-l tablets | XX | XXX |
| - | Artemether-l tablets | XX | XXX |
| - | Artemether-l tablets | XX | XXX |
| - | Artemether-l tablets | XX | XXX |
| - | Artemether-l tablets | XX | XXX |

| | | | |
|---|---|---|---|
| - | Artemether-l tablets | XX | XXX |
| - | Artemether-l tablets | XX | XXX |
| - | Artemether-l tablets | XX | XXX |
| - | Artemether-l tablets | XX | XXX |
| - | Artemether-l tablets | XX | XXX |
| - | Artemether-l tablets | XX | XXX |
| - | Artemether-l tablets | XX | XXX |
| - | Artemether-l tablets | XX | XXX |
| - | Artemether-l tablets | XX | XXX |
| - | Artemether-l tablets | XX | XXX |
| - | Artemether-l tablets | XX | XXX |
| - | Artemether-l tablets | XX | XXX |
| - | Artemether-l tablets | XX | XXX |
| - | Artemether-l tablets | XX | XXX |
| - | Artemether-l tablets | XX | XXX |
| - | Artemether-l tablets | XX | XXX |
| - | Artemether-l tablets | XX | XXX |
| - | Artemether-l tablets | XX | XXX |
| - | Artemether-l tablets | XX | XXX |
| - | Artemether-l tablets | XX | XXX |
| - | Artemether-l tablets | XX | XXX |
| - | Artemether-l tablets | XX | XXX |
| - | Artemether-l tablets | XX | XXX |
| - | Artemether-l tablets | XX | XXX |
| - | Artemether-l tablets | XX | XXX |
| - | Artemether-l tablets | XX | XXX |
| - | Artemether-l tablets | XX | XXX |
| - | Artemether-l tablets | XX | XXX |
| - | Artemether-l tablets | XX | XXX |
| - | Artemether-l tablets | XX | XXX |
| - | Artemether-l tablets | XX | XXX |
| - | Artemether-l tablets | XX | XXX |
| - | Artemether-l tablets | XX | XXX |
| - | Artemether-l tablets | XX | XXX |
| - | Artemether-l tablets | XX | XXX |
| - | Artemether-l tablets | XX | XXX |
| - | Artemether-l tablets | XX | XXX |
| - | Artemether-l tablets | XX | XXX |
| - | Artemether-l tablets | XX | XXX |
| - | Artemether-l tablets | XX | XXX |
| - | Artemether-l tablets | XX | XXX |
| - | Artemether-l tablets | XX | XXX |
| - | Artemether-l tablets | XX | XXX |
| - | Artemether-l tablets | XX | XXX |
| - | Artemether-l tablets | XX | XXX |
| - | Artemether-l tablets | XX | XXX |
| - | Artemether-l tablets | XX | XXX |
| - | Artemether-l tablets | XX | XXX |
| - | Artemether-l tablets | XX | XXX |
| - | Artemether-l tablets | XX | XXX |

| | | | |
|---|---|---|---|
| - | Artemether-l tablets | XX | XXX |
| - | Artemether-l tablets | XX | XXX |
| - | Artemether-l tablets | XX | XXX |
| - | Artemether-l tablets | XX | XXX |
| - | Artemether-l tablets | XX | XXX |
| - | Artemether-l tablets | XX | XXX |
| - | Artemether-l tablets | XX | XXX |
| - | Artemether-l tablets | XX | XXX |
| - | Artemether-l tablets | XX | XXX |
| - | Artemether-l tablets | XX | XXX |
| - | Artemether-l tablets | XX | XXX |
| - | Artemether-l tablets | XX | XXX |
| - | CIPROFLOXA( tablets | XX | XXX |
| - | Artemether-l tablets | XX | XXX |
| - | Artemether-l tablets | XX | XXX |
| - | Artemether-l tablets | XX | XXX |
| - | Artemether-l tablets | XX | XXX |
| - | Artemether-l tablets | XX | XXX |
| - | Artemether-l tablets | XX | XXX |
| - | Artemether-l tablets | XX | XXX |
| - | Artemether-l tablets | XX | XXX |
| - | Artemether-l tablets | XX | XXX |
| - | Artemether-l tablets | XX | XXX |
| - | Artemether-l tablets | XX | XXX |
| - | Artemether-l tablets | XX | XXX |
| - | Artemether-l tablets | XX | XXX |
| - | Artemether-l tablets | XX | XXX |
| - | Artemether-l tablets | XX | XXX |
| - | Artemether-l tablets | XX | XXX |
| - | Artemether-l tablets | XX | XXX |
| - | Artemether-l tablets | XX | XXX |
| - | Artemether-l tablets | XX | XXX |
| - | Artemether-l tablets | XX | XXX |
| - | Artemether-l tablets | XX | XXX |
| - | Artemether-l tablets | XX | XXX |
| - | Artemether-l tablets | XX | XXX |
| - | Artemether-l tablets | XX | XXX |
| - | Artemether-l tablets | XX | XXX |
| - | Artemether-l tablets | XX | XXX |
| - | Artemether-l tablets | XX | XXX |
| - | Artemether-l tablets | XX | XXX |
| - | Artemether-l tablets | XX | XXX |
| - | Artemether-l tablets | XX | XXX |
| - | CIPROFLOXA( tablets | XX | XXX |
| - | Artemether-l tablets | XX | XXX |
| - | Artemether-l tablets | XX | XXX |
| - | Artemether-l tablets | XX | XXX |
| - | Artemether-l tablets | XX | XXX |
| - | Artemether-l tablets | XX | XXX |
| - | Artemether-l tablets | XX | XXX |

| | | | |
|---|---|---|---|
| - | Artemether-l tablets | XX | XXX |
| - | Artemether-l tablets | XX | XXX |
| - | Artemether-l tablets | XX | XXX |
| - | Artemether-l tablets | XX | XXX |
| - | Artemether-l tablets | XX | XXX |
| - | Artemether-l tablets | XX | XXX |
| - | Artemether-l tablets | XX | XXX |
| - | Artemether-l tablets | XX | XXX |
| - | Artemether-l tablets | XX | XXX |
| - | Artemether-l tablets | XX | XXX |
| - | Artemether-l tablets | XX | XXX |
| - | Artemether-l tablets | XX | XXX |
| - | Artemether-l tablets | XX | XXX |
| - | Artemether-l tablets | XX | XXX |
| - | Artemether-l tablets | XX | XXX |
| - | Artemether-l tablets | XX | XXX |
| - | Artemether-l tablets | XX | XXX |
| - | Artemether-l tablets | XX | XXX |
| - | Artemether-l tablets | XX | XXX |
| - | Artemether-l tablets | XX | XXX |
| - | Artemether-l tablets | XX | XXX |
| - | Artemether-l tablets | XX | XXX |
| - | Artemether-l tablets | XX | XXX |
| - | Artemether-l tablets | XX | XXX |
| - | Artemether-l tablets | XX | XXX |
| - | Artemether-l tablets | XX | XXX |
| - | Artemether-l tablets | XX | XXX |
| - | Artemether-l tablets | XX | XXX |
| - | Artemether-l tablets | XX | XXX |
| - | Artemether-l tablets | XX | XXX |
| - | Artemether-l tablets | XX | XXX |
| - | Artemether-l tablets | XX | XXX |
| - | Artemether-l tablets | XX | XXX |
| - | Artemether-l tablets | XX | XXX |
| - | Artemether-l tablets | XX | XXX |
| - | Artemether-l tablets | XX | XXX |
| - | Artemether-l tablets | XX | XXX |
| - | Artemether-l tablets | XX | XXX |
| - | Artemether-l tablets | XX | XXX |
| - | Artemether-l tablets | XX | XXX |
| - | Artemether-l tablets | XX | XXX |
| - | Artemether-l tablets | XX | XXX |
| - | Artemether-l tablets | XX | XXX |
| - | Artemether-l tablets | XX | XXX |
| - | Artemether-l tablets | XX | XXX |
| - | Artemether-l tablets | XX | XXX |
| - | Artemether-l tablets | XX | XXX |
| - | Artemether-l tablets | XX | XXX |
| - | Artemether-l tablets | XX | XXX |
| - | Artemether-l tablets | XX | XXX |

| | | | | |
|---|---|---|---|---|
| - | Artemether-l | tablets | XX | XXX |
| - | Artemether-l | tablets | XX | XXX |
| - | Artemether-l | tablets | XX | XXX |
| - | Artemether-l | tablets | XX | XXX |
| - | CIPROFLOXA( | tablets | XX | XXX |
| - | Artemether-l | tablets | XX | XXX |
| - | Artemether-l | tablets | XX | XXX |
| - | Artemether-l | tablets | XX | XXX |
| - | Artemether-l | tablets | XX | XXX |
| - | Artemether-l | tablets | XX | XXX |
| - | Artemether-l | tablets | XX | XXX |
| - | Artemether-l | tablets | XX | XXX |
| - | Artemether-l | tablets | XX | XXX |
| - | Artemether-l | tablets | XX | XXX |
| - | Artemether-l | tablets | XX | XXX |
| - | Artemether-l | tablets | XX | XXX |
| - | Artemether-l | tablets | XX | XXX |
| - | Artemether-l | tablets | XX | XXX |
| - | Artemether-l | tablets | XX | XXX |
| - | Artemether-l | tablets | XX | XXX |
| - | Artemether-l | tablets | XX | XXX |
| - | Artemether-l | tablets | XX | XXX |
| - | Artemether-l | tablets | XX | XXX |
| - | Artemether-l | tablets | XX | XXX |
| - | Artemether-l | tablets | XX | XXX |
| - | Artemether-l | tablets | XX | XXX |
| - | Artemether-l | tablets | XX | XXX |
| - | Artemether-l | tablets | XX | XXX |
| - | Artemether-l | tablets | XX | XXX |
| - | Artemether-l | tablets | XX | XXX |
| - | Artemether-l | tablets | XX | XXX |
| - | Artemether-l | tablets | XX | XXX |
| - | Artemether-l | tablets | XX | XXX |
| - | Artemether-l | tablets | XX | XXX |
| - | Artemether-l | tablets | XX | XXX |
| - | Artemether-l | tablets | XX | XXX |
| - | Artemether-l | tablets | XX | XXX |
| - | Artemether-l | tablets | XX | XXX |
| - | Artemether-l | tablets | XX | XXX |
| - | Artemether-l | tablets | XX | XXX |
| - | Artemether-l | tablets | XX | XXX |
| - | Artemether-l | tablets | XX | XXX |
| - | Artemether-l | tablets | XX | XXX |
| - | Artemether-l | tablets | XX | XXX |
| - | Artemether-l | suspension | XX | XXX |
| - | Artemether-l | tablets | XX | XXX |
| - | Artemether-l | suspension | XX | XXX |
| - | Artemether-l | tablets | XX | XXX |
| - | Artemether-l | tablets | XX | XXX |
| - | Artemether-l | tablets | XX | XXX |

| | | | |
|---|---|---|---|
| - | Artemether-l tablets | XX | XXX |
| - | Artemether-l tablets | XX | XXX |
| - | Artemether-l tablets | XX | XXX |
| - | Artemether-l tablets | XX | XXX |
| - | Artemether-l tablets | XX | XXX |
| - | Artemether-l tablets | XX | XXX |
| - | Artemether-l tablets | XX | XXX |
| - | Artemether-l tablets | XX | XXX |
| - | Artemether-l tablets | XX | XXX |
| - | Artemether-l tablets | XX | XXX |
| - | Artemether-l tablets | XX | XXX |
| - | Artemether-l tablets | XX | XXX |
| - | Artemether-l tablets | XX | XXX |
| - | Artemether-l tablets | XX | XXX |
| - | Artemether-l tablets | XX | XXX |
| - | Artemether-l tablets | XX | XXX |
| - | Artemether-l tablets | XX | XXX |
| - | Artemether-l tablets | XX | XXX |
| - | Artemether-l tablets | XX | XXX |
| - | Artemether-l tablets | XX | XXX |
| - | Artemether-l tablets | XX | XXX |
| - | Artemether-l tablets | XX | XXX |
| - | Artemether-l tablets | XX | XXX |
| - | Artemether-l tablets | XX | XXX |
| - | Artemether-l tablets | XX | XXX |
| - | Artemether-l tablets | XX | XXX |
| - | Artemether-l tablets | XX | XXX |
| - | Artemether-l tablets | XX | XXX |
| - | Artemether-l tablets | XX | XXX |
| - | Artemether-l tablets | XX | XXX |
| - | Artemether-l tablets | XX | XXX |
| - | Artemether-l tablets | XX | XXX |
| - | Artemether-l tablets | XX | XXX |
| - | Artemether-l tablets | XX | XXX |
| - | Artemether-l tablets | XX | XXX |
| - | Artemether-l tablets | XX | XXX |
| - | Artemether-l tablets | XX | XXX |
| - | Artemether-l tablets | XX | XXX |
| - | Artemether-l tablets | XX | XXX |
| - | Artemether-l tablets | XX | XXX |
| - | Artemether-l tablets | XX | XXX |
| - | Artemether-l tablets | XX | XXX |
| - | Artemether-l tablets | XX | XXX |
| - | Artemether-l tablets | XX | XXX |
| - | Artemether-l tablets | XX | XXX |
| - | Artemether-l tablets | XX | XXX |
| - | Artemether-l tablets | XX | XXX |
| - | Artemether-l tablets | XX | XXX |
| - | Artemether-l tablets | XX | XXX |
| - | Artemether-l tablets | XX | XXX |

| | | | | |
|---|---|---|---|---|
| - | Artemether-l | tablets | XX | XXX |
| - | Artemether-l | tablets | XX | XXX |
| - | Artemether-l | tablets | XX | XXX |
| - | Artemether-l | tablets | XX | XXX |
| - | Artemether-l | tablets | XX | XXX |
| - | Artemether-l | tablets | XX | XXX |
| - | Artemether-l | tablets | XX | XXX |
| - | Artemether-l | tablets | XX | XXX |
| - | Artemether-l | tablets | XX | XXX |
| - | Artemether-l | tablets | XX | XXX |
| - | Artemether-l | tablets | XX | XXX |
| - | Artemether-l | tablets | XX | XXX |
| - | Artemether-l | tablets | XX | XXX |
| - | Artesunate | tablets | XX | XXX |
| - | Artesunate | tablets | XX | XXX |
| - | Artesunate | tablets | XX | XXX |
| - | Artesunate | tablets | XX | XXX |
| - | Artesunate | tablets | XX | XXX |
| - | Artesunate | tablets | XX | XXX |
| - | Artesunate | tablets | XX | XXX |
| - | Artesunate | tablets | XX | XXX |
| - | Artesunate | tablets | XX | XXX |
| - | ACETAMINOI | tablets | XX | XXX |
| - | Artesunate | tablets | XX | XXX |
| - | Artesunate | tablets | XX | XXX |
| - | Artesunate | tablets | XX | XXX |
| - | Artesunate | tablets | XX | XXX |
| - | Artesunate | tablets | XX | XXX |
| - | Artesunate | tablets | XX | XXX |
| - | Artesunate | tablets | XX | XXX |
| - | Artesunate | tablets | XX | XXX |
| - | DEHA or DOA | tablets | XX | XXX |
| - | Artesunate | tablets | XX | XXX |
| - | Artesunate | tablets | XX | XXX |
| - | Artesunate | tablets | XX | XXX |
| - | Artesunate | tablets | XX | XXX |
| - | Artesunate | tablets | XX | XXX |
| - | Artesunate | tablets | XX | XXX |
| - | Artesunate | tablets | XX | XXX |
| - | Artesunate | tablets | XX | XXX |
| - | Artesunate | tablets | XX | XXX |
| - | Artesunate | tablets | XX | XXX |
| - | Artesunate | tablets | XX | XXX |
| - | Artesunate | tablets | XX | XXX |
| - | Artesunate | tablets | XX | XXX |
| - | Artesunate | tablets | XX | XXX |
| - | Artesunate | tablets | XX | XXX |
| - | Artesunate | tablets | XX | XXX |
| - | Artesunate | tablets | XX | XXX |
| - | Artesunate | tablets | XX | XXX |

| | | | | |
|---|---|---|---|---|
| - | Artesunate | tablets | XX | XXX |
| - | DEHA or DOA | tablets | XX | XXX |
| - | Artesunate | tablets | XX | XXX |
| - | Artesunate | tablets | XX | XXX |
| - | Artesunate | tablets | XX | XXX |
| - | Artesunate | tablets | XX | XXX |
| - | Artesunate | tablets | XX | XXX |
| - | Artesunate | tablets | XX | XXX |
| - | Artesunate | tablets | XX | XXX |
| - | Artesunate | tablets | XX | XXX |
| - | Artesunate | tablets | XX | XXX |
| - | Artesunate | tablets | XX | XXX |
| - | Artesunate | tablets | XX | XXX |
| - | Artesunate | tablets | XX | XXX |
| - | Artesunate | tablets | XX | XXX |
| - | Artesunate | tablets | XX | XXX |
| - | Artesunate | tablets | XX | XXX |
| - | Artesunate | tablets | XX | XXX |
| - | Artesunate | tablets | XX | XXX |
| - | Artesunate | tablets | XX | XXX |
| - | Artesunate | tablets | XX | XXX |
| - | Artesunate | tablets | XX | XXX |
| - | Artesunate | tablets | XX | XXX |
| - | Artesunate | tablets | XX | XXX |
| - | Artesunate | tablets | XX | XXX |
| - | Artesunate | tablets | XX | XXX |
| - | Artesunate | tablets | XX | XXX |
| - | Artesunate | tablets | XX | XXX |
| - | Artesunate | tablets | XX | XXX |
| - | Artesunate | tablets | XX | XXX |
| - | Artesunate | tablets | XX | XXX |
| - | Artesunate | tablets | XX | XXX |
| - | Artesunate | tablets | XX | XXX |
| - | Artesunate | tablets | XX | XXX |
| - | Artesunate | tablets | XX | XXX |
| - | Artesunate | tablets | XX | XXX |
| - | Artesunate | tablets | XX | XXX |
| - | Artesunate | tablets | XX | XXX |
| - | Artesunate | tablets | XX | XXX |
| - | Artesunate | tablets | XX | XXX |
| - | Artesunate | tablets | XX | XXX |
| - | Artesunate | tablets | XX | XXX |
| - | Artesunate | tablets | XX | XXX |
| - | Artesunate | tablets | XX | XXX |
| - | Artesunate | tablets | XX | XXX |
| - | Artesunate | tablets | XX | XXX |
| - | Artesunate | tablets | XX | XXX |
| - | Artesunate | tablets | XX | XXX |
| - | Artesunate | tablets | XX | XXX |
| - | Artesunate | tablets | XX | XXX |

| | | | | |
|---|---|---|---|---|
| - | Artesunate | tablets | XX | XXX |
| - | Artesunate | tablets | XX | XXX |
| - | Artesunate | tablets | XX | XXX |
| - | Artesunate | tablets | XX | XXX |
| - | Artesunate | tablets | XX | XXX |
| - | Artesunate-A | tablets | XX | XXX |
| - | Artesunate-A | tablets | XX | XXX |
| - | Artesunate-A | tablets | XX | XXX |
| - | Artesunate-A | tablets | XX | XXX |
| - | Artesunate-A | tablets | XX | XXX |
| - | Artesunate-A | tablets | XX | XXX |
| - | Artesunate-A | tablets | XX | XXX |
| - | Artesunate-A | tablets | XX | XXX |
| - | Artesunate-A | tablets | XX | XXX |
| - | Artesunate-A | tablets | XX | XXX |
| - | Artesunate-A | tablets | XX | XXX |
| - | Artesunate-A | tablets | XX | XXX |
| - | Artesunate-A | tablets | XX | XXX |
| - | Artesunate-A | tablets | XX | XXX |
| - | Artesunate-A | tablets | XX | XXX |
| - | Artesunate-A | tablets | XX | XXX |
| - | Artesunate-A | tablets | XX | XXX |
| - | Artesunate-A | tablets | XX | XXX |
| - | Artesunate-A | tablets | XX | XXX |
| - | Artesunate-A | tablets | XX | XXX |
| - | Artesunate-A | tablets | XX | XXX |
| - | Artesunate-A | tablets | XX | XXX |
| - | Artesunate-A | tablets | XX | XXX |
| - | Artesunate-A | tablets | XX | XXX |
| - | Artesunate-A | tablets | XX | XXX |
| - | Artesunate-A | tablets | XX | XXX |
| - | Artesunate-A | tablets | XX | XXX |
| - | Artesunate-A | tablets | XX | XXX |
| - | Artesunate-A | tablets | XX | XXX |
| - | Artesunate-A | tablets | XX | XXX |
| - | Artesunate-A | tablets | XX | XXX |
| - | Artesunate-A | tablets | XX | XXX |
| - | Artesunate-A | tablets | XX | XXX |
| - | Artesunate-A | tablets | XX | XXX |
| - | Artesunate-A | tablets | XX | XXX |
| - | Artesunate-A | tablets | XX | XXX |
| - | Artesunate-A | tablets | XX | XXX |
| - | Artesunate-A | tablets | XX | XXX |
| - | Artesunate-A | tablets | XX | XXX |
| - | Artesunate-A | tablets | XX | XXX |
| - | Artesunate-A | tablets | XX | XXX |
| - | Artesunate-A | tablets | XX | XXX |
| - | Artesunate-A | tablets | XX | XXX |
| - | Artesunate-A | tablets | XX | XXX |
| - | Artesunate-A | tablets | XX | XXX |

| | | | |
|---|---|---|---|
| - | Artesunate-A tablets | XX | XXX |
| - | Artesunate-A tablets | XX | XXX |
| - | Artesunate-A tablets | XX | XXX |
| - | Artesunate-A tablets | XX | XXX |
| - | Artesunate-A tablets | XX | XXX |
| - | Artesunate-A tablets | XX | XXX |
| - | Artesunate-A tablets | XX | XXX |
| - | Artesunate-A tablets | XX | XXX |
| - | Artesunate-A tablets | XX | XXX |
| - | Artesunate-A tablets | XX | XXX |
| - | Artesunate-A tablets | XX | XXX |
| - | Artesunate-A tablets | XX | XXX |
| - | Artesunate-A tablets | XX | XXX |
| - | Artesunate-A tablets | XX | XXX |
| - | Artesunate-A tablets | XX | XXX |
| - | Artesunate-A tablets | XX | XXX |
| - | Artesunate-A tablets | XX | XXX |
| - | Artesunate-A tablets | XX | XXX |
| - | Artesunate-A tablets | XX | XXX |
| - | Artesunate-A tablets | XX | XXX |
| - | Artesunate-A tablets | XX | XXX |
| - | Artesunate-A tablets | XX | XXX |
| - | Artesunate-A tablets | XX | XXX |
| - | Artesunate-A tablets | XX | XXX |
| - | Artesunate-A tablets | XX | XXX |
| - | Artesunate-A tablets | XX | XXX |
| - | Artesunate-A tablets | XX | XXX |
| - | Artesunate-A tablets | XX | XXX |
| - | Artesunate-A tablets | XX | XXX |
| - | Artesunate-A tablets | XX | XXX |
| - | Artesunate-A tablets | XX | XXX |
| - | Artesunate-A tablets | XX | XXX |
| - | Artesunate-A tablets | XX | XXX |
| - | Artesunate-A tablets | XX | XXX |
| - | Artesunate-A tablets | XX | XXX |
| - | Artesunate-A tablets | XX | XXX |
| - | Artesunate-A tablets | XX | XXX |
| - | Artesunate-A tablets | XX | XXX |
| - | Artesunate-A tablets | XX | XXX |
| - | Artesunate-A tablets | XX | XXX |
| - | Artesunate-A tablets | XX | XXX |
| - | Artesunate-A tablets | XX | XXX |
| - | Artesunate-A tablets | XX | XXX |
| - | Artesunate-A tablets | XX | XXX |
| - | Artesunate-A tablets | XX | XXX |
| - | Artesunate-A tablets | XX | XXX |
| - | Artesunate-A tablets | XX | XXX |
| - | Artesunate-A tablets | XX | XXX |
| - | Artesunate-A tablets | XX | XXX |
| - | Artesunate-A tablets | XX | XXX |

| | | | |
|---|---|---|---|
| - | Artesunate-A tablets | XX | XXX |
| - | Artesunate-A tablets | XX | XXX |
| - | Artesunate-A tablets | XX | XXX |
| - | Artesunate-A tablets | XX | XXX |
| - | Artesunate-A tablets | XX | XXX |
| - | Artesunate-A tablets | XX | XXX |
| - | Artesunate-A tablets | XX | XXX |
| - | Artesunate-A tablets | XX | XXX |
| - | Artesunate-A tablets | XX | XXX |
| - | Artesunate-A tablets | XX | XXX |
| - | Artesunate-A tablets | XX | XXX |
| - | Artesunate-A tablets | XX | XXX |
| - | Artesunate-A tablets | XX | XXX |
| - | Artesunate-A tablets | XX | XXX |
| - | Artesunate-A tablets | XX | XXX |
| - | Artesunate-A tablets | XX | XXX |
| - | Artesunate-A tablets | XX | XXX |
| - | Artesunate-A tablets | XX | XXX |
| - | Artesunate-A tablets | XX | XXX |
| - | Artesunate-A tablets | XX | XXX |
| - | Artesunate-A tablets | XX | XXX |
| - | Artesunate-A tablets | XX | XXX |
| - | Artesunate-A tablets | XX | XXX |
| - | Artesunate-A tablets | XX | XXX |
| - | Artesunate-A tablets | XX | XXX |
| - | Artesunate-A tablets | XX | XXX |
| - | Artesunate-A tablets | XX | XXX |
| - | Artesunate-A tablets | XX | XXX |
| - | Artesunate-A tablets | XX | XXX |
| - | Artesunate-A tablets | XX | XXX |
| - | Artesunate-A tablets | XX | XXX |
| - | Artesunate-A tablets | XX | XXX |
| - | Artesunate-A tablets | XX | XXX |
| - | Artesunate-A tablets | XX | XXX |
| - | Artesunate-A tablets | XX | XXX |
| - | Artesunate-A tablets | XX | XXX |
| - | Artesunate-A tablets | XX | XXX |
| - | Artesunate-A tablets | XX | XXX |
| - | Artesunate-A tablets | XX | XXX |
| - | Artesunate-A tablets | XX | XXX |
| - | Artesunate-A tablets | XX | XXX |
| - | Artesunate-A tablets | XX | XXX |
| - | Artesunate-A tablets | XX | XXX |
| - | Artesunate-A tablets | XX | XXX |
| - | Artesunate-A tablets | XX | XXX |
| - | Artesunate-A tablets | XX | XXX |
| - | Artesunate-A tablets | XX | XXX |
| - | Artesunate-A tablets | XX | XXX |
| - | Artesunate-A tablets | XX | XXX |
| - | Artesunate-A tablets | XX | XXX |

| | | | |
|---|---|---|---|
| - | Artesunate-A tablets | XX | XXX |
| - | Artesunate-A tablets | XX | XXX |
| - | Artesunate-A tablets | XX | XXX |
| - | Artesunate-A tablets | XX | XXX |
| - | Artesunate-A tablets | XX | XXX |
| - | Artesunate-A tablets | XX | XXX |
| - | Artesunate-A tablets | XX | XXX |
| - | Artesunate-A tablets | XX | XXX |
| - | Artesunate-A tablets | XX | XXX |
| - | Artesunate-A tablets | XX | XXX |
| - | Artesunate-A tablets | XX | XXX |
| - | Artesunate-A tablets | XX | XXX |
| - | Artesunate-A tablets | XX | XXX |
| - | Artesunate-A tablets | XX | XXX |
| - | Artesunate-A tablets | XX | XXX |
| - | Artesunate-A tablets | XX | XXX |
| - | Artesunate-A tablets | XX | XXX |
| - | Artesunate-A tablets | XX | XXX |
| - | Artesunate-A tablets | XX | XXX |
| - | Artesunate-A tablets | XX | XXX |
| - | Artesunate-A tablets | XX | XXX |
| - | Artesunate-A tablets | XX | XXX |
| - | Artesunate-A tablets | XX | XXX |
| - | Artesunate-A tablets | XX | XXX |
| - | Artesunate-A tablets | XX | XXX |
| - | Artesunate-A tablets | XX | XXX |
| - | Artesunate-A tablets | XX | XXX |
| - | Artesunate-A tablets | XX | XXX |
| - | Artesunate-A tablets | XX | XXX |
| - | Artesunate-A tablets | XX | XXX |
| - | Artesunate-A tablets | XX | XXX |
| - | Artesunate-A tablets | XX | XXX |
| - | Artesunate-A tablets | XX | XXX |
| - | Artesunate-A tablets | XX | XXX |
| - | Artesunate-A tablets | XX | XXX |
| - | Artesunate-A tablets | XX | XXX |
| - | Artesunate-A tablets | XX | XXX |
| - | Artesunate-A tablets | XX | XXX |
| - | Artesunate-A tablets | XX | XXX |
| - | Artesunate-A tablets | XX | XXX |
| - | Artesunate-A tablets | XX | XXX |
| - | Artesunate-A tablets | XX | XXX |
| - | Artesunate-A tablets | XX | XXX |
| - | Artesunate-A tablets | XX | XXX |
| - | Artesunate-A tablets | XX | XXX |
| - | Artesunate-A tablets | XX | XXX |
| - | Artesunate-A tablets | XX | XXX |
| - | Artesunate-A tablets | XX | XXX |
| - | Artesunate-A tablets | XX | XXX |
| - | Artesunate-A tablets | XX | XXX |

| | | | | |
|---|---|---|---|---|
| - | Artesunate-A | tablets | XX | XXX |
| - | Artesunate-A | granule | XX | XXX |
| - | Artesunate-A | powder | XX | XXX |
| - | Artesunate-A | powder | XX | XXX |
| - | Artesunate-A | powder | XX | XXX |
| - | Artesunate-A | powder | XX | XXX |
| - | Artesunate-A | powder | XX | XXX |
| - | Artesunate-A | powder | XX | XXX |
| - | Artesunate-A | powder | XX | XXX |
| - | Artesunate-A | powder | XX | XXX |
| - | Artesunate-A | powder | XX | XXX |
| - | Artesunate-A | powder | XX | XXX |
| - | Artesunate-A | powder | XX | XXX |
| - | Artesunate-A | powder | XX | XXX |
| - | Artesunate-A | powder | XX | XXX |
| - | Artesunate-A | powder | XX | XXX |
| - | Artesunate-A | powder | XX | XXX |
| - | Artesunate-A | powder | XX | XXX |
| - | Artesunate-A | powder | XX | XXX |
| - | Artesunate-A | powder | XX | XXX |
| - | Artesunate-A | powder | XX | XXX |
| - | Artesunate-A | powder | XX | XXX |
| - | Artesunate-A | powder | XX | XXX |
| - | Artesunate-A | powder | XX | XXX |
| - | Artesunate-A | powder | XX | XXX |
| - | Artesunate-A | powder | XX | XXX |
| - | Amodiaquine | powder | XX | XXX |
| - | Amodiaquine | powder | XX | XXX |
| - | Artesunate | powder | XX | XXX |
| - | Artesunate | powder | XX | XXX |
| - | Amodiaquine | powder | XX | XXX |
| - | Amodiaquine | powder | XX | XXX |
| - | Artesunate-A | powder | XX | XXX |
| - | Artesunate-A | powder | XX | XXX |
| - | Artesunate-A | powder | XX | XXX |
| - | Artesunate-A | powder | XX | XXX |
| - | Artesunate-N | tablets | XX | XXX |
| - | Artesunate-N | tablets | XX | XXX |
| - | Artesunate-N | tablets | XX | XXX |
| - | Artesunate-N | tablets | XX | XXX |
| - | Artesunate-N | tablets | XX | XXX |
| - | Artesunate-N | tablets | XX | XXX |
| - | Artesunate-N | tablets | XX | XXX |
| - | Artesunate-N | tablets | XX | XXX |
| - | Artesunate-N | tablets | XX | XXX |
| - | Artesunate-N | tablets | XX | XXX |
| - | Artesunate-N | tablets | XX | XXX |
| - | Artesunate-N | tablets | XX | XXX |
| - | Artesunate-N | tablets | XX | XXX |
| - | Artesunate-N | tablets | XX | XXX |

| | | | |
|---|---|---|---|
| - | Artesunate-N tablets | XX | XXX |
| - | Artesunate-N tablets | XX | XXX |
| - | Artesunate-N tablets | XX | XXX |
| - | Artesunate-N tablets | XX | XXX |
| - | Artesunate-N tablets | XX | XXX |
| - | Artesunate-N tablets | XX | XXX |
| - | Artesunate-N tablets | XX | XXX |
| - | Artesunate-N tablets | XX | XXX |
| - | Artesunate-N tablets | XX | XXX |
| - | Artesunate-N tablets | XX | XXX |
| - | Artesunate-N tablets | XX | XXX |
| - | Artesunate-N tablets | XX | XXX |
| - | Artesunate-N tablets | XX | XXX |
| - | Artesunate-N tablets | XX | XXX |
| - | Artesunate-N tablets | XX | XXX |
| - | Artesunate-N tablets | XX | XXX |
| - | Artesunate-N tablets | XX | XXX |
| - | Artesunate-N tablets | XX | XXX |
| - | Artesunate-N tablets | XX | XXX |
| - | Artesunate-N tablets | XX | XXX |
| - | Artesunate-N tablets | XX | XXX |
| - | Artesunate-N tablets | XX | XXX |
| - | Artesunate-N tablets | XX | XXX |
| - | Artesunate-N tablets | XX | XXX |
| - | Artesunate-N tablets | XX | XXX |
| - | Artesunate-N tablets | XX | XXX |
| - | Artesunate-N tablets | XX | XXX |
| - | Artesunate-N tablets | XX | XXX |
| - | Artesunate-N tablets | XX | XXX |
| - | Artesunate-N tablets | XX | XXX |
| - | Artesunate-N tablets | XX | XXX |
| - | Artesunate-N tablets | XX | XXX |
| - | Artesunate-N tablets | XX | XXX |
| 94.2 | Artesunate-P tablets | XX | XXX |
| 101.6 | Artesunate-S tablets | XX | XXX |
| 98.9 | Artesunate-S tablets | XX | XXX |
| 92.8 | Artesunate-S tablets | XX | XXX |
| 105.5 | Artesunate-S tablets | XX | XXX |
| 112.6 | Artesunate-S tablets | XX | XXX |
| 101.6 | Artesunate-S tablets | XX | XXX |
| 83.3 | Artesunate-S tablets | XX | XXX |
| 95.2 | Artesunate-S tablets | XX | XXX |
| 76.8 | Artesunate-S tablets | XX | XXX |
| 77.4 | Artesunate-S tablets | XX | XXX |
| 93.5 | Artesunate-S tablets | XX | XXX |
| 102.6 | Artesunate-S tablets | XX | XXX |
| 106.3 | Artesunate-S tablets | XX | XXX |
| 96.3 | Artesunate-S tablets | XX | XXX |
| 98.6 | Artesunate-S tablets | XX | XXX |
| 100.6 | Artesunate-S tablets | XX | XXX |

| | | | | |
|---|---|---|---|---|
| 95.1 | Artesunate-S | tablets | XX | XXX |
| 93 | Artesunate-S | tablets | XX | XXX |
| 98.6 | Artesunate-S | tablets | XX | XXX |
| - | Dihydroarten | tablets | XX | XXX |
| 103.5 | Artesunate-S | tablets | XX | XXX |
| 94.1 | Artesunate-S | tablets | XX | XXX |
| 104.9 | Artesunate-S | tablets | XX | XXX |
| 106.1 | Artesunate-S | tablets | XX | XXX |
| 101.5 | Artesunate-S | tablets | XX | XXX |
| 90.1 | Artesunate-S | tablets | XX | XXX |
| 95.1 | Artesunate-S | tablets | XX | XXX |
| 108.4 | Artesunate-S | tablets | XX | XXX |
| 107.7 | Artesunate-S | tablets | XX | XXX |
| 99.4 | Artesunate-S | tablets | XX | XXX |
| 90.2 | Artesunate-S | tablets | XX | XXX |
| 97.3 | Artesunate-S | tablets | XX | XXX |
| 110.0 | Artesunate-S | tablets | XX | XXX |
| 112.1 | Artesunate-S | tablets | XX | XXX |
| 99.1 | Artesunate-S | tablets | XX | XXX |
| 105.1 | Artesunate-S | tablets | XX | XXX |
| 106.7 | Artesunate-S | tablets | XX | XXX |
| 104.3 | Artesunate-S | tablets | XX | XXX |
| 109.7 | Artesunate-S | tablets | XX | XXX |
| - | Dihydroarten | tablets | XX | XXX |
| - | Dihydroarten | tablets | XX | XXX |
| - | Dihydroarten | tablets | XX | XXX |
| - | Dihydroarten | tablets | XX | XXX |
| - | Dihydroarten | tablets | XX | XXX |
| - | Dihydroarten | tablets | XX | XXX |
| - | Dihydroarten | tablets | XX | XXX |
| - | Dihydroarten | tablets | XX | XXX |
| - | Dihydroarten | tablets | XX | XXX |
| - | Dihydroarten | tablets | XX | XXX |
| - | Dihydroarten | tablets | XX | XXX |
| - | Dihydroarten | tablets | XX | XXX |
| - | Dihydroarten | tablets | XX | XXX |
| - | Dihydroarten | tablets | XX | XXX |
| - | Dihydroarten | tablets | XX | XXX |
| - | Dihydroarten | tablets | XX | XXX |
| - | Dihydroarten | tablets | XX | XXX |
| - | Dihydroarten | tablets | XX | XXX |
| - | Dihydroarten | tablets | XX | XXX |
| - | Dihydroarten | tablets | XX | XXX |
| - | Dihydroarten | tablets | XX | XXX |
| - | Dihydroarten | tablets | XX | XXX |
| - | ACETAMINOI | tablets | XX | XXX |
| - | Dihydroarten | tablets | XX | XXX |
| - | Dihydroarten | tablets | XX | XXX |
| - | Dihydroarten | tablets | XX | XXX |
| - | Dihydroarten | tablets | XX | XXX |

| | | | | |
|---|---|---|---|---|
| - | Dihydroarten | tablets | XX | XXX |
| - | Dihydroarten | tablets | XX | XXX |
| - | Dihydroarten | tablets | XX | XXX |
| - | Dihydroarten | tablets | XX | XXX |
| - | Dihydroarten | tablets | XX | XXX |
| - | Dihydroarten | tablets | XX | XXX |
| - | Dihydroarten | tablets | XX | XXX |
| - | Dihydroarten | tablets | XX | XXX |
| - | Dihydroarten | tablets | XX | XXX |
| - | Dihydroarten | tablets | XX | XXX |
| - | Dihydroarten | tablets | XX | XXX |
| - | Dihydroarten | tablets | XX | XXX |
| - | Dihydroarten | tablets | XX | XXX |
| - | Dihydroarten | tablets | XX | XXX |
| - | Dihydroarten | tablets | XX | XXX |
| - | Dihydroarten | tablets | XX | XXX |
| - | Dihydroarten | tablets | XX | XXX |
| - | Dihydroarten | tablets | XX | XXX |
| - | Dihydroarten | tablets | XX | XXX |
| - | Dihydroarten | tablets | XX | XXX |
| - | Dihydroarten | tablets | XX | XXX |
| - | Dihydroarten | tablets | XX | XXX |
| - | Dihydroarten | tablets | XX | XXX |
| - | Dihydroarten | tablets | XX | XXX |
| - | Dihydroarten | tablets | XX | XXX |
| - | Dihydroarten | tablets | XX | XXX |
| - | Dihydroarten | tablets | XX | XXX |
| - | Dihydroarten | tablets | XX | XXX |
| - | Dihydroarten | tablets | XX | XXX |
| - | Dihydroarten | tablets | XX | XXX |
| - | Dihydroarten | tablets | XX | XXX |
| - | ACETAMINOP | tablets | XX | XXX |
| - | Dihydroarten | tablets | XX | XXX |
| - | Dihydroarten | tablets | XX | XXX |
| - | Dihydroarten | tablets | XX | XXX |
| - | Dihydroarten | tablets | XX | XXX |
| - | Dihydroarten | tablets | XX | XXX |
| - | ACETAMINOP | tablets | XX | XXX |
| - | Dihydroarten | tablets | XX | XXX |
| - | Dihydroarten | tablets | XX | XXX |
| - | Dihydroarten | tablets | XX | XXX |
| - | Dihydroarten | tablets | XX | XXX |
| - | Dihydroarten | tablets | XX | XXX |
| - | Dihydroarten | tablets | XX | XXX |
| - | Dihydroarten | tablets | XX | XXX |
| - | Dihydroarten | tablets | XX | XXX |
| - | DISACCHARID | tablets | XX | XXX |
| - | Dihydroarten | tablets | XX | XXX |
| - | Dihydroarten | tablets | XX | XXX |
| - | Dihydroarten | tablets | XX | XXX |

| | | | | |
|---|---|---|---|---|
| - | Dihydroarten | tablets | XX | XXX |
| - | Dihydroarten | tablets | XX | XXX |
| - | Dihydroarten | tablets | XX | XXX |
| - | Dihydroarten | tablets | XX | XXX |
| - | Dihydroarten | tablets | XX | XXX |
| - | DISACCHARIE | tablets | XX | XXX |
| - | Dihydroarten | tablets | XX | XXX |
| - | Dihydroarten | tablets | XX | XXX |
| - | Dihydroarten | tablets | XX | XXX |
| - | Dihydroarten | tablets | XX | XXX |
| - | Dihydroarten | tablets | XX | XXX |
| - | ACETAMINOI | tablets | XX | XXX |
| - | Dihydroarten | tablets | XX | XXX |
| - | Dihydroarten | tablets | XX | XXX |
| - | Dihydroarten | tablets | XX | XXX |
| - | Dihydroarten | tablets | XX | XXX |
| - | Dihydroarten | tablets | XX | XXX |
| - | Dihydroarten | tablets | XX | XXX |
| - | Dihydroarten | tablets | XX | XXX |
| - | Dihydroarten | tablets | XX | XXX |
| - | Dihydroarten | tablets | XX | XXX |
| - | Dihydroarten | tablets | XX | XXX |
| - | Dihydroarten | tablets | XX | XXX |
| - | Dihydroarten | tablets | XX | XXX |
| - | Dihydroarten | tablets | XX | XXX |
| - | Dihydroarten | tablets | XX | XXX |
| - | ACETAMINOI | tablets | XX | XXX |
| - | Dihydroarten | tablets | XX | XXX |
| - | Dihydroarten | tablets | XX | XXX |
| - | Dihydroarten | tablets | XX | XXX |
| - | Dihydroarten | tablets | XX | XXX |
| - | Dihydroarten | tablets | XX | XXX |
| - | Dihydroarten | tablets | XX | XXX |
| - | Dihydroarten | tablets | XX | XXX |
| - | Dihydroarten | tablets | XX | XXX |
| - | Dihydroarten | tablets | XX | XXX |
| - | Dihydroarten | tablets | XX | XXX |
| - | Dihydroarten | tablets | XX | XXX |
| - | ACETAMINOI | tablets | XX | XXX |
| - | Dihydroarten | tablets | XX | XXX |
| - | Dihydroarten | tablets | XX | XXX |
| - | Dihydroarten | tablets | XX | XXX |
| - | Dihydroarten | tablets | XX | XXX |
| - | Dihydroarten | tablets | XX | XXX |
| - | Dihydroarten | tablets | XX | XXX |
| - | Dihydroarten | tablets | XX | XXX |
| - | Dihydroarten | tablets | XX | XXX |
| - | Dihydroarten | tablets | XX | XXX |
| - | Dihydroarten | tablets | XX | XXX |
| - | Dihydroarten | tablets | XX | XXX |

| | | | | |
|---|---|---|---|---|
| - | Dihydroarten | tablets | XX | XXX |
| - | Dihydroarten | tablets | XX | XXX |
| - | Dihydroarten | tablets | XX | XXX |
| - | Dihydroarten | tablets | XX | XXX |
| - | ACETAMINOI | tablets | XX | XXX |
| - | DISACCHARII | tablets | XX | XXX |
| - | ACETAMINOI | tablets | XX | XXX |
| - | Dihydroarten | tablets | XX | XXX |
| - | Dihydroarten | tablets | XX | XXX |
| - | ACETAMINOI | tablets | XX | XXX |
| - | Dihydroarten | tablets | XX | XXX |
| - | ACETAMINOI | tablets | XX | XXX |
| - | Dihydroarten | tablets | XX | XXX |
| - | Dihydroarten | tablets | XX | XXX |
| - | Dihydroarten | tablets | XX | XXX |
| - | Dihydroarten | tablets | XX | XXX |
| - | Dihydroarten | tablets | XX | XXX |
| - | Dihydroarten | tablets | XX | XXX |
| - | Dihydroarten | tablets | XX | XXX |
| - | Dihydroarten | tablets | XX | XXX |
| - | Dihydroarten | tablets | XX | XXX |
| - | Dihydroarten | tablets | XX | XXX |
| - | Dihydroarten | tablets | XX | XXX |
| - | Dihydroarten | tablets | XX | XXX |
| - | Dihydroarten | tablets | XX | XXX |
| - | Dihydroarten | tablets | XX | XXX |
| - | Dihydroarten | tablets | XX | XXX |
| - | Dihydroarten | tablets | XX | XXX |
| - | Dihydroarten | tablets | XX | XXX |
| - | Dihydroarten | tablets | XX | XXX |
| - | Dihydroarten | tablets | XX | XXX |
| - | Dihydroarten | tablets | XX | XXX |
| - | Dihydroarten | tablets | XX | XXX |
| - | Dihydroarten | tablets | XX | XXX |
| - | Dihydroarten | tablets | XX | XXX |
| - | Dihydroarten | tablets | XX | XXX |
| - | Dihydroarten | tablets | XX | XXX |
| - | Dihydroarten | tablets | XX | XXX |
| - | Dihydroarten | tablets | XX | XXX |
| - | Dihydroarten | tablets | XX | XXX |
| - | Dihydroarten | tablets | XX | XXX |
| - | Dihydroarten | tablets | XX | XXX |
| - | Dihydroarten | tablets | XX | XXX |
| - | Dihydroarten | tablets | XX | XXX |
| - | Dihydroarten | tablets | XX | XXX |
| - | Dihydroarten | tablets | XX | XXX |
| - | Dihydroarten | tablets | XX | XXX |
| - | Dihydroarten | tablets | XX | XXX |
| - | Dihydroarten | tablets | XX | XXX |
| - | Dihydroarten | tablets | XX | XXX |

| | | | |
|---|---|---|---|
| - | Dihydroarten tablets | XX | XXX |
| - | Dihydroarten tablets | XX | XXX |
| - | Dihydroarten tablets | XX | XXX |
| - | Dihydroarten tablets | XX | XXX |
| - | Dihydroarten tablets | XX | XXX |
| - | Dihydroarten tablets | XX | XXX |
| - | Dihydroarten tablets | XX | XXX |
| - | Dihydroarten tablets | XX | XXX |
| - | Dihydroarten tablets | XX | XXX |
| - | Dihydroarten tablets | XX | XXX |
| - | Dihydroarten tablets | XX | XXX |
| - | DEHA or DOA tablets | XX | XXX |
| - | Dihydroarten tablets | XX | XXX |
| - | Dihydroarten tablets | XX | XXX |
| - | Dihydroarten tablets | XX | XXX |
| - | Dihydroarten tablets | XX | XXX |
| - | Dihydroarten tablets | XX | XXX |
| - | Dihydroarten tablets | XX | XXX |
| - | Dihydroarten tablets | XX | XXX |
| - | Dihydroarten tablets | XX | XXX |
| - | Dihydroarten tablets | XX | XXX |
| - | Dihydroarten tablets | XX | XXX |
| - | Dihydroarten tablets | XX | XXX |
| - | Dihydroarten tablets | XX | XXX |
| - | Dihydroarten tablets | XX | XXX |
| - | Dihydroarten tablets | XX | XXX |
| - | Dihydroarten tablets | XX | XXX |
| - | Dihydroarten tablets | XX | XXX |
| - | Dihydroarten tablets | XX | XXX |
| - | Dihydroarten tablets | XX | XXX |
| - | Dihydroarten tablets | XX | XXX |
| - | Dihydroarten tablets | XX | XXX |
| - | Dihydroarten tablets | XX | XXX |
| - | Dihydroarten tablets | XX | XXX |
| - | Dihydroarten tablets | XX | XXX |
| - | Dihydroarten tablets | XX | XXX |
| - | Dihydroarten tablets | XX | XXX |
| - | Dihydroarten tablets | XX | XXX |
| - | Dihydroarten tablets | XX | XXX |
| - | Dihydroarten tablets | XX | XXX |
| - | Dihydroarten tablets | XX | XXX |
| - | Dihydroarten tablets | XX | XXX |
| - | Dihydroarten tablets | XX | XXX |
| - | Dihydroarten tablets | XX | XXX |
| - | Dihydroarten tablets | XX | XXX |
| - | Dihydroarten tablets | XX | XXX |
| - | Dihydroarten tablets | XX | XXX |
| - | Dihydroarten tablets | XX | XXX |
| - | Dihydroarten tablets | XX | XXX |
| - | Dihydroarten tablets | XX | XXX |

| | | | |
|---|---|---|---|
| - | Dihydroarten tablets | XX | XXX |
| - | Dihydroarten tablets | XX | XXX |
| - | Dihydroarten tablets | XX | XXX |
| - | Dihydroarten tablets | XX | XXX |
| - | Dihydroarten tablets | XX | XXX |
| - | Dihydroarten tablets | XX | XXX |
| - | Dihydroarten tablets | XX | XXX |
| - | Dihydroarten tablets | XX | XXX |
| - | Dihydroarten tablets | XX | XXX |
| - | Dihydroarten tablets | XX | XXX |
| - | Dihydroarten tablets | XX | XXX |
| - | Dihydroarten tablets | XX | XXX |
| - | Dihydroarten tablets | XX | XXX |
| - | Dihydroarten tablets | XX | XXX |
| - | Dihydroarten tablets | XX | XXX |
| - | Dihydroarten tablets | XX | XXX |
| - | Dihydroarten tablets | XX | XXX |
| - | Dihydroarten tablets | XX | XXX |
| - | Dihydroarten tablets | XX | XXX |
| - | Dihydroarten tablets | XX | XXX |
| - | Dihydroarten tablets | XX | XXX |
| - | Dihydroarten tablets | XX | XXX |
| - | Dihydroarten tablets | XX | XXX |
| - | Dihydroarten tablets | XX | XXX |
| - | Dihydroarten tablets | XX | XXX |
| - | Dihydroarten tablets | XX | XXX |
| - | Dihydroarten tablets | XX | XXX |
| - | Dihydroarten tablets | XX | XXX |
| - | Dihydroarten tablets | XX | XXX |
| - | Dihydroarten tablets | XX | XXX |
| - | Dihydroarten tablets | XX | XXX |
| - | Dihydroarten tablets | XX | XXX |
| - | Dihydroarten tablets | XX | XXX |
| - | Dihydroarten tablets | XX | XXX |
| - | Dihydroarten tablets | XX | XXX |
| - | Dihydroarten tablets | XX | XXX |
| - | Dihydroarten tablets | XX | XXX |
| - | Dihydroarten tablets | XX | XXX |
| - | Dihydroarten tablets | XX | XXX |
| - | Dihydroarten tablets | XX | XXX |
| - | Dihydroarten tablets | XX | XXX |
| - | Dihydroarten tablets | XX | XXX |
| - | Dihydroarten tablets | XX | XXX |
| - | Dihydroarten tablets | XX | XXX |
| - | Dihydroarten tablets | XX | XXX |
| - | Dihydroarten tablets | XX | XXX |
| - | Dihydroarten tablets | XX | XXX |
| - | Dihydroarten tablets | XX | XXX |
| - | Dihydroarten tablets | XX | XXX |
| - | Dihydroarten tablets | XX | XXX |

| | | | | |
|---|---|---|---|---|
| - | Dihydroarten | tablets | XX | XXX |
| - | Dihydroarten | tablets | XX | XXX |
| - | Dihydroarten | tablets | XX | XXX |
| - | Dihydroarten | tablets | XX | XXX |
| - | Dihydroarten | tablets | XX | XXX |
| - | Dihydroarten | tablets | XX | XXX |
| - | Dihydroarten | tablets | XX | XXX |
| - | Dihydroarten | tablets | XX | XXX |
| - | Dihydroarten | tablets | XX | XXX |
| - | Dihydroarten | tablets | XX | XXX |
| - | Dihydroarten | tablets | XX | XXX |
| - | Dihydroarten | tablets | XX | XXX |
| - | Dihydroarten | tablets | XX | XXX |
| - | Dihydroarten | tablets | XX | XXX |
| - | Dihydroarten | tablets | XX | XXX |
| - | Dihydroarten | tablets | XX | XXX |
| - | Dihydroarten | tablets | XX | XXX |
| - | Dihydroarten | tablets | XX | XXX |
| - | Dihydroarten | tablets | XX | XXX |
| - | Dihydroarten | tablets | XX | XXX |
| - | Dihydroarten | tablets | XX | XXX |
| - | not sent to A | suspension | XX | XXX |
| - | Artemether-l | suspension | XX | XXX |
| - | not sent to A | suspension | XX | XXX |
| - | not sent to A | suspension | XX | XXX |
| - | not sent to A | suspension | XX | XXX |
| - | not sent to A | suspension | XX | XXX |
| - | not sent to A | suspension | XX | XXX |
| - | not sent to A | suspension | XX | XXX |
| - | Artemether-l | suspension | XX | XXX |
| - | not sent to A | suspension | XX | XXX |
| - | not sent to A | suspension | XX | XXX |
| - | not sent to A | suspension | XX | XXX |
| - | Artemether-l | suspension | XX | XXX |
| - | Artemether-l | suspension | XX | XXX |
| - | not sent to A | suspension | XX | XXX |
| - | not sent to A | suspension | XX | XXX |
| - | not sent to A | suspension | XX | XXX |
| - | not sent to A | suspension | XX | XXX |
| - | Artemether-l | suspension | XX | XXX |
| - | Artemether-l | suspension | XX | XXX |
| - | not sent to A | suspension | XX | XXX |
| - | Artemether-l | suspension | XX | XXX |
| - | Artemether-l | suspension | XX | XXX |
| - | not sent to A | suspension | XX | XXX |
| - | not sent to A | suspension | XX | XXX |
| - | not sent to A | suspension | XX | XXX |
| - | not sent to A | suspension | XX | XXX |
| - | Artemether-l | suspension | XX | XXX |
| - | Artemether-l | suspension | XX | XXX |

| | | | |
|---|---|---|---|
| - | not sent to A suspension | XX | XXX |
| - | not sent to A Suspension | XX | XXX |
| - | Artemether-l suspension | XX | XXX |
| - | Artemether-l suspension | XX | XXX |
| - | not sent to A suspension | XX | XXX |
| - | not sent to A suspension | XX | XXX |
| - | not sent to A suspension | XX | XXX |
| - | not sent to A suspension | XX | XXX |
| - | not sent to A suspension | XX | XXX |
| - | Artemether-l suspension | XX | XXX |
| - | Artemether-l suspension | XX | XXX |
| - | not sent to A suspension | XX | XXX |
| - | not sent to A suspension | XX | XXX |
| - | not sent to A suspension | XX | XXX |
| - | Artemether-l suspension | XX | XXX |
| - | Artemether-l suspension | XX | XXX |
| - | not sent to A suspension | XX | XXX |
| - | not sent to A suspension | XX | XXX |
| - | not sent to A suspension | XX | XXX |
| - | not sent to A suspension | XX | XXX |
| - | not sent to A suspension | XX | XXX |
| - | not sent to A suspension | XX | XXX |
| - | not sent to A suspension | XX | XXX |
| - | not sent to A suspension | XX | XXX |
| - | not sent to A suspension | XX | XXX |
| - | not sent to A suspension | XX | XXX |
| - | not sent to A suspension | XX | XXX |
| - | not sent to A suspension | XX | XXX |
| - | not sent to A suspension | XX | XXX |
| - | not sent to A suspension | XX | XXX |
| - | not sent to A suspension | XX | XXX |
| - | not sent to A suspension | XX | XXX |
| - | not sent to A suspension | XX | XXX |
| - | not sent to A suspension | XX | XXX |
| - | not sent to A suspension | XX | XXX |
| - | not sent to A suspension | XX | XXX |
| - | not sent to A suspension | XX | XXX |
| - | not sent to A suspension | XX | XXX |
| - | not sent to A suspension | XX | XXX |
| - | not sent to A suspension | XX | XXX |
| - | Artemether-l suspension | XX | XXX |
| - | Artemether-l suspension | XX | XXX |
| - | Artemether-l suspension | XX | XXX |
| - | Artemether-l suspension | XX | XXX |
| - | Artemether-l suspension | XX | XXX |
| - | Artemether-l suspension | XX | XXX |
| - | Artemether-l suspension | XX | XXX |
| - | Artemether-l suspension | XX | XXX |
| - | Artemether-l suspension | XX | XXX |
| - | Artemether-l suspension | XX | XXX |

| | | | | |
|---|---|---|---|---|
| - | Artemether-l | suspension | XX | XXX |
| - | Artemether-l | suspension | XX | XXX |
| - | Artemether-l | suspension | XX | XXX |
| - | Artemether-l | suspension | XX | XXX |
| - | Artemether-l | suspension | XX | XXX |
| - | Artemether-l | suspension | XX | XXX |
| - | Artemether-l | suspension | XX | XXX |
| - | not sent to A | suspension | XX | XXX |
| - | not sent to A | suspension | XX | XXX |
| - | not sent to A | suspension | XX | XXX |
| - | not sent to A | suspension | XX | XXX |
| - | Artemether-l | suspension | XX | XXX |
| - | Artemether-l | suspension | XX | XXX |
| - | Artemether-l | capsules | XX | XXX |
| - | Artemether-l | capsules | XX | XXX |
| - | Artemether-l | capsules | XX | XXX |
| - | Artemether-l | capsules | XX | XXX |
| - | Artemether-l | capsules | XX | XXX |
| - | Artemether-l | soft gel | XX | XXX |
| - | Artemether-l | soft gel | XX | XXX |
| - | Artemether-l | soft gel | XX | XXX |
| - | Artemether-l | granule | XX | XXX |
| - | Artemether-l | granule | XX | XXX |
| - | Artemether-l | granule | XX | XXX |
| - | Artesunate | suspension | XX | XXX |
| - | not sent to A | suspension | XX | XXX |
| - | Artesunate | suspension | XX | XXX |
| - | not sent to A | suspension | XX | XXX |
| - | Artesunate | suspension | XX | XXX |
| - | not sent to A | suspension | XX | XXX |
| - | Artesunate | suspension | XX | XXX |
| - | not sent to A | suspension | XX | XXX |
| - | Artesunate | suspension | XX | XXX |
| - | not sent to A | suspension | XX | XXX |
| - | Artesunate | suspension | XX | XXX |
| - | not sent to A | suspension | XX | XXX |
| - | Artesunate | suspension | XX | XXX |
| - | not sent to A | suspension | XX | XXX |
| - | Artesunate | suspension | XX | XXX |
| - | not sent to A | suspension | XX | XXX |
| - | Artesunate-A | suspension | XX | XXX |
| - | not sent to A | suspension | XX | XXX |
| - | not sent to A | suspension | XX | XXX |
| - | not sent to A | suspension | XX | XXX |
| - | Dihydroarten | suspension | XX | XXX |
| - | not sent to A | suspension | XX | XXX |
| - | not sent to A | suspension | XX | XXX |
| - | Dihydroarten | suspension | XX | XXX |
| - | not sent to A | suspension | XX | XXX |
| - | not sent to A | suspension | XX | XXX |

| | | | | |
|---|---|---|---|---|
| - | Dihydroarten | suspension | XX | XXX |
| - | not sent to A | suspension | XX | XXX |
| - | Dihydroarten | suspension | XX | XXX |
| - | not sent to A | suspension | XX | XXX |
| - | not sent to A | suspension | XX | XXX |
| - | not sent to A | suspension | XX | XXX |
| - | Dihydroarten | suspension | XX | XXX |
| - | not sent to A | suspension | XX | XXX |
| - | Dihydroarten | suspension | XX | XXX |
| - | not sent to A | suspension | XX | XXX |
| - | not sent to A | suspension | XX | XXX |
| - | Dihydroarten | suspension | XX | XXX |
| - | not sent to A | suspension | XX | XXX |
| - | Dihydroarten | suspension | XX | XXX |
| - | not sent to A | suspension | XX | XXX |
| - | Dihydroarten | suspension | XX | XXX |
| - | not sent to A | suspension | XX | XXX |
| - | Dihydroarten | suspension | XX | XXX |
| - | not sent to A | suspension | XX | XXX |
| - | Dihydroarten | suspension | XX | XXX |
| - | not sent to A | suspension | XX | XXX |
| - | Dihydroarten | suspension | XX | XXX |
| - | not sent to A | suspension | XX | XXX |
| - | Dihydroarten | suspension | XX | XXX |
| - | not sent to A | suspension | XX | XXX |
| - | Dihydroarten | suspension | XX | XXX |
| - | not sent to A | suspension | XX | XXX |
| - | Dihydroarten | suspension | XX | XXX |
| - | not sent to A | suspension | XX | XXX |
| - | Dihydroarten | suspension | XX | XXX |
| - | not sent to A | suspension | XX | XXX |
| - | Dihydroarten | suspension | XX | XXX |
| - | not sent to A | suspension | XX | XXX |
| - | Dihydroarten | suspension | XX | XXX |
| - | not sent to A | suspension | XX | XXX |
| - | not sent to A | suspension | XX | XXX |
| - | Artemether-l | tablets | XX | XXX |
| - | Artemether-l | tablets | XX | XXX |
| - | Artemether-l | tablets | XX | XXX |
| - | Artemether-l | tablets | XX | XXX |
| - | Artemether-l | tablets | XX | XXX |
| - | Artemether-l | tablets | XX | XXX |
| - | Artemether-l | tablets | XX | XXX |
| - | Artemether-l | tablets | XX | XXX |
| - | Artemether-l | tablets | XX | XXX |
| - | Artemether-l | tablets | XX | XXX |
| - | Artemether-l | tablets | XX | XXX |
| - | Artemether-l | tablets | XX | XXX |
| - | Artemether-l | tablets | XX | XXX |
| - | Artemether-l | tablets | XX | XXX |

| | | | |
|---|---|---|---|
| - | Artemether-l tablets | XX | XXX |
| - | Artemether-l tablets | XX | XXX |
| - | Artemether-l tablets | XX | XXX |
| - | Artemether-l tablets | XX | XXX |
| - | Artemether-l tablets | XX | XXX |
| - | Artemether-l tablets | XX | XXX |
| - | Artemether-l tablets | XX | XXX |
| - | Artemether-l tablets | XX | XXX |
| - | Artemether-l tablets | XX | XXX |
| - | Artemether-l tablets | XX | XXX |
| - | Artemether-l tablets | XX | XXX |
| - | Artemether-l tablets | XX | XXX |
| - | Artemether-l tablets | XX | XXX |
| - | Artemether-l tablets | XX | XXX |
| - | Artemether-l tablets | XX | XXX |
| - | Artemether-l tablets | XX | XXX |
| - | Artemether-l tablets | XX | XXX |
| - | Artemether-l tablets | XX | XXX |
| - | Artemether-l tablets | XX | XXX |
| - | Artemether-l tablets | XX | XXX |
| - | Artemether-l tablets | XX | XXX |
| - | Artemether-l tablets | XX | XXX |
| - | Artemether-l tablets | XX | XXX |
| - | Artemether-l tablets | XX | XXX |
| - | Artemether-l tablets | XX | XXX |
| - | Artemether-l tablets | XX | XXX |
| - | Artemether-l tablets | XX | XXX |
| - | Artemether-l tablets | XX | XXX |
| - | Artemether-l tablets | XX | XXX |
| - | Artemether-l tablets | XX | XXX |
| - | Artemether-l tablets | XX | XXX |
| - | Artemether-l tablets | XX | XXX |
| - | Artemether-l tablets | XX | XXX |
| - | Artemether-l tablets | XX | XXX |
| - | Artemether-l tablets | XX | XXX |
| - | Artemether-l tablets | XX | XXX |
| - | Artemether-l tablets | XX | XXX |
| - | Artemether-l tablets | XX | XXX |
| - | Artemether-l tablets | XX | XXX |
| - | Artemether-l tablets | XX | XXX |
| - | Artemether-l tablets | XX | XXX |
| - | Artemether-l tablets | XX | XXX |
| - | Artemether-l tablets | XX | XXX |
| - | Artemether-l tablets | XX | XXX |
| - | Artemether-l tablets | XX | XXX |
| - | Artemether-l tablets | XX | XXX |
| - | Artemether-l tablets | XX | XXX |
| - | Artemether-l tablets | XX | XXX |
| - | Artemether-l tablets | XX | XXX |
| - | Artemether-l tablets | XX | XXX |

| | | | |
|---|---|---|---|
| - | Artemether-l tablets | XX | XXX |
| - | Artemether-l tablets | XX | XXX |
| - | Artemether-l tablets | XX | XXX |
| - | Artemether-l tablets | XX | XXX |
| - | Artemether-l tablets | XX | XXX |
| - | Artemether-l tablets | XX | XXX |
| - | Artemether-l tablets | XX | XXX |
| - | Artemether-l tablets | XX | XXX |
| - | Artemether-l tablets | XX | XXX |
| - | Artemether-l tablets | XX | XXX |
| - | Artemether-l tablets | XX | XXX |
| - | Artemether-l tablets | XX | XXX |
| - | Artemether-l tablets | XX | XXX |
| - | Artemether-l tablets | XX | XXX |
| - | Artemether-l tablets | XX | XXX |
| - | Artemether-l tablets | XX | XXX |
| - | Artemether-l tablets | XX | XXX |
| - | Artemether-l tablets | XX | XXX |
| - | Artemether-l tablets | XX | XXX |
| - | Artemether-l tablets | XX | XXX |
| - | Artemether-l tablets | XX | XXX |
| - | Artemether-l tablets | XX | XXX |
| - | Artemether-l tablets | XX | XXX |
| - | Artemether-l tablets | XX | XXX |
| - | Artemether-l tablets | XX | XXX |
| - | Artemether-l tablets | XX | XXX |
| - | Artemether-l tablets | XX | XXX |
| - | Artemether-l tablets | XX | XXX |
| - | Artemether-l tablets | XX | XXX |
| - | Artemether-l tablets | XX | XXX |
| - | Artemether-l tablets | XX | XXX |
| - | Artemether-l tablets | XX | XXX |
| - | Artemether-l tablets | XX | XXX |
| - | Artemether-l tablets | XX | XXX |
| - | Artemether-l tablets | XX | XXX |
| - | Artemether-l tablets | XX | XXX |
| - | Artemether-l tablets | XX | XXX |
| - | Artemether-l tablets | XX | XXX |
| - | Artemether-l tablets | XX | XXX |
| - | Artemether-l tablets | XX | XXX |
| - | Artemether-l tablets | XX | XXX |
| - | Artemether-l tablets | XX | XXX |
| - | Artemether-l tablets | XX | XXX |
| - | Artemether-l tablets | XX | XXX |
| - | Artemether-l tablets | XX | XXX |
| - | Artemether-l tablets | XX | XXX |
| - | Artemether-l tablets | XX | XXX |
| - | Artemether-l tablets | XX | XXX |
| - | Artemether-l tablets | XX | XXX |
| - | Artemether-l tablets | XX | XXX |

| | | | | |
|---|---|---|---|---|
| - | CHLORZOXAZ | tablets | XX | XXX |
| - | Artemether-l | tablets | XX | XXX |
| - | Artemether-l | tablets | XX | XXX |
| - | Artemether-l | tablets | XX | XXX |
| - | Artemether-l | tablets | XX | XXX |
| - | Artemether-l | tablets | XX | XXX |
| - | Artemether-l | tablets | XX | XXX |
| - | CHLORZOXAZ | tablets | XX | XXX |
| - | CHLORZOXAZ | tablets | XX | XXX |
| - | Artemether-l | tablets | XX | XXX |
| - | Artemether-l | tablets | XX | XXX |
| - | Artemether-l | tablets | XX | XXX |
| - | Artemether-l | tablets | XX | XXX |
| - | Artemether-l | tablets | XX | XXX |
| - | Artemether-l | tablets | XX | XXX |
| - | Artemether-l | tablets | XX | XXX |
| - | Artemether-l | tablets | XX | XXX |
| - | Artemether-l | tablets | XX | XXX |
| - | Artemether-l | tablets | XX | XXX |
| - | Artemether-l | tablets | XX | XXX |
| - | Artemether-l | tablets | XX | XXX |
| - | Artemether-l | tablets | XX | XXX |
| - | Artemether-l | tablets | XX | XXX |
| - | Artemether-l | tablets | XX | XXX |
| - | Artemether-l | tablets | XX | XXX |
| - | Artemether-l | tablets | XX | XXX |
| - | Artemether-l | tablets | XX | XXX |
| - | Artemether-l | tablets | XX | XXX |
| - | Artemether-l | tablets | XX | XXX |
| - | Artemether-l | tablets | XX | XXX |
| - | Artemether-l | tablets | XX | XXX |
| - | Artemether-l | tablets | XX | XXX |
| - | Artemether-l | tablets | XX | XXX |
| - | Artemether-l | tablets | XX | XXX |
| - | Artemether-l | tablets | XX | XXX |
| - | Artemether-l | tablets | XX | XXX |
| - | Artemether-l | tablets | XX | XXX |
| - | Artemether-l | tablets | XX | XXX |
| - | Artemether-l | tablets | XX | XXX |
| - | Artemether-l | tablets | XX | XXX |
| - | Artemether-l | tablets | XX | XXX |
| - | Artemether-l | tablets | XX | XXX |
| - | Artemether-l | tablets | XX | XXX |
| - | Artemether-l | tablets | XX | XXX |
| - | Artemether-l | tablets | XX | XXX |
| - | Artemether-l | tablets | XX | XXX |
| - | Artemether-l | tablets | XX | XXX |
| - | Artemether-l | tablets | XX | XXX |
| - | Artemether-l | tablets | XX | XXX |
| - | Artemether-l | tablets | XX | XXX |

| | | | |
|---|---|---|---|
| - | Artemether-l tablets | XX | XXX |
| - | Artemether-l tablets | XX | XXX |
| - | Artemether-l tablets | XX | XXX |
| - | Artemether-l tablets | XX | XXX |
| - | Artemether-l tablets | XX | XXX |
| - | Artemether-l tablets | XX | XXX |
| - | Artemether-l tablets | XX | XXX |
| - | Artemether-l tablets | XX | XXX |
| - | Artemether-l tablets | XX | XXX |
| - | Artemether-l tablets | XX | XXX |
| - | Artemether-l tablets | XX | XXX |
| - | Artemether-l tablets | XX | XXX |
| - | Artemether-l tablets | XX | XXX |
| - | Artemether-l tablets | XX | XXX |
| - | Artemether-l tablets | XX | XXX |
| - | Artemether-l tablets | XX | XXX |
| - | Artemether-l tablets | XX | XXX |
| - | Artemether-l tablets | XX | XXX |
| - | Artemether-l tablets | XX | XXX |
| - | Artemether-l tablets | XX | XXX |
| - | Artemether-l tablets | XX | XXX |
| - | Artemether-l tablets | XX | XXX |
| - | Artemether-l tablets | XX | XXX |
| - | Artemether-l tablets | XX | XXX |
| - | Artemether-l tablets | XX | XXX |
| - | Artemether-l tablets | XX | XXX |
| - | Artemether-l tablets | XX | XXX |
| - | Artemether-l tablets | XX | XXX |
| - | Artemether-l tablets | XX | XXX |
| - | Artemether-l tablets | XX | XXX |
| - | Artemether-l tablets | XX | XXX |
| - | Artemether-l tablets | XX | XXX |
| - | Artemether-l tablets | XX | XXX |
| - | Artemether-l tablets | XX | XXX |
| - | Artemether-l tablets | XX | XXX |
| - | Artemether-l tablets | XX | XXX |
| - | Artemether-l tablets | XX | XXX |
| - | Artemether-l tablets | XX | XXX |
| - | Artemether-l tablets | XX | XXX |
| - | Artemether-l tablets | XX | XXX |
| - | Artemether-l tablets | XX | XXX |
| - | Artemether-l tablets | XX | XXX |
| - | Artemether-l tablets | XX | XXX |
| - | Artemether-l tablets | XX | XXX |
| - | Artemether-l tablets | XX | XXX |
| - | Artemether-l tablets | XX | XXX |
| - | Artemether-l tablets | XX | XXX |
| - | Artemether-l tablets | XX | XXX |
| - | Artemether-l tablets | XX | XXX |
| - | Artemether-l tablets | XX | XXX |

| | | | |
|---|---|---|---|
| - | Artemether-l tablets | XX | XXX |
| - | Artemether-l tablets | XX | XXX |
| - | Artemether-l tablets | XX | XXX |
| - | Artemether-l tablets | XX | XXX |
| - | Artemether-l tablets | XX | XXX |
| - | Artemether-l tablets | XX | XXX |
| - | Artemether-l tablets | XX | XXX |
| - | Artemether-l tablets | XX | XXX |
| - | Artemether-l tablets | XX | XXX |
| - | Artemether-l tablets | XX | XXX |
| - | Artemether-l tablets | XX | XXX |
| - | Artemether-l tablets | XX | XXX |
| - | Artemether-l tablets | XX | XXX |
| - | Artemether-l tablets | XX | XXX |
| - | Artemether-l tablets | XX | XXX |
| - | Artemether-l tablets | XX | XXX |
| - | Artemether-l tablets | XX | XXX |
| - | Artemether-l tablets | XX | XXX |
| - | Artemether-l tablets | XX | XXX |
| - | Artemether-l tablets | XX | XXX |
| - | Artemether-l tablets | XX | XXX |
| - | Artemether-l tablets | XX | XXX |
| - | Artemether-l tablets | XX | XXX |
| - | Artemether-l tablets | XX | XXX |
| - | Artemether-l tablets | XX | XXX |
| - | Artemether-l tablets | XX | XXX |
| - | Artemether-l tablets | XX | XXX |
| - | Artemether-l tablets | XX | XXX |
| - | Artemether-l tablets | XX | XXX |
| - | Artemether-l tablets | XX | XXX |
| - | Artemether-l tablets | XX | XXX |
| - | Artemether-l tablets | XX | XXX |
| - | Artemether-l tablets | XX | XXX |
| - | Artemether-l tablets | XX | XXX |
| - | Artemether-l tablets | XX | XXX |
| - | Artemether-l tablets | XX | XXX |
| - | Artemether-l tablets | XX | XXX |
| - | Artemether-l tablets | XX | XXX |
| - | Artemether-l tablets | XX | XXX |
| - | Artemether-l tablets | XX | XXX |
| - | Artemether-l tablets | XX | XXX |
| - | Artemether-l tablets | XX | XXX |
| - | Artemether-l tablets | XX | XXX |
| - | Artemether-l tablets | XX | XXX |
| - | Artemether-l tablets | XX | XXX |
| - | Artemether-l tablets | XX | XXX |
| - | Artemether-l tablets | XX | XXX |
| - | Artemether-l tablets | XX | XXX |
| - | Artemether-l tablets | XX | XXX |
| - | Artemether-l tablets | XX | XXX |

| | | | |
|---|---|---|---|
| - | Artemether-l tablets | XX | XXX |
| - | Artemether-l tablets | XX | XXX |
| - | Artemether-l tablets | XX | XXX |
| - | Artemether-l tablets | XX | XXX |
| - | Artemether-l tablets | XX | XXX |
| - | Artemether-l tablets | XX | XXX |
| - | Artemether-l tablets | XX | XXX |
| - | Artemether-l tablets | XX | XXX |
| - | Artemether-l tablets | XX | XXX |
| - | Artemether-l tablets | XX | XXX |
| - | Artemether-l tablets | XX | XXX |
| - | Artemether-l tablets | XX | XXX |
| - | Artemether-l tablets | XX | XXX |
| - | Artemether-l tablets | XX | XXX |
| - | Artemether-l tablets | XX | XXX |
| - | Artemether-l tablets | XX | XXX |
| - | Artemether-l tablets | XX | XXX |
| - | Artemether-l tablets | XX | XXX |
| - | Artemether-l tablets | XX | XXX |
| - | Artemether-l tablets | XX | XXX |
| - | Artemether-l tablets | XX | XXX |
| - | Artemether-l tablets | XX | XXX |
| - | Artemether-l tablets | XX | XXX |
| - | Artemether-l tablets | XX | XXX |
| - | Artemether-l tablets | XX | XXX |
| - | Artemether-l tablets | XX | XXX |
| - | Artemether-l tablets | XX | XXX |
| - | Artemether-l tablets | XX | XXX |
| - | Artemether-l tablets | XX | XXX |
| - | Artemether-l tablets | XX | XXX |
| - | Artemether-l tablets | XX | XXX |
| - | Artemether-l tablets | XX | XXX |
| - | Artemether-l tablets | XX | XXX |
| - | Artemether-l tablets | XX | XXX |
| - | Artemether-l tablets | XX | XXX |
| - | Artemether-l tablets | XX | XXX |
| - | Artemether-l tablets | XX | XXX |
| - | Artemether-l tablets | XX | XXX |
| - | Artemether-l tablets | XX | XXX |
| - | Artemether-l tablets | XX | XXX |
| - | Artemether-l tablets | XX | XXX |
| - | Artemether-l tablets | XX | XXX |
| - | Artemether-l tablets | XX | XXX |
| - | Artemether-l tablets | XX | XXX |
| - | Artemether-l tablets | XX | XXX |
| - | Artemether-l tablets | XX | XXX |
| - | Artemether-l tablets | XX | XXX |
| - | Artemether-l tablets | XX | XXX |
| - | Artemether-l tablets | XX | XXX |
| - | Artemether-l tablets | XX | XXX |

| | | | |
|---|---|---|---|
| - | Artemether-l tablets | XX | XXX |
| - | Artemether-l tablets | XX | XXX |
| - | Artemether-l tablets | XX | XXX |
| - | Artemether-l tablets | XX | XXX |
| - | Artemether-l tablets | XX | XXX |
| - | Artemether-l tablets | XX | XXX |
| - | Artemether-l tablets | XX | XXX |
| - | Artemether-l tablets | XX | XXX |
| - | Artemether-l tablets | XX | XXX |
| - | Artemether-l tablets | XX | XXX |
| - | Artemether-l tablets | XX | XXX |
| - | Artemether-l tablets | XX | XXX |
| - | Artemether-l tablets | XX | XXX |
| - | Artemether-l tablets | XX | XXX |
| - | Artemether-l tablets | XX | XXX |
| - | Artemether-l tablets | XX | XXX |
| - | Artemether-l tablets | XX | XXX |
| - | Artemether-l tablets | XX | XXX |
| - | Artemether-l tablets | XX | XXX |
| - | Artemether-l tablets | XX | XXX |
| - | Artemether-l tablets | XX | XXX |
| - | Artemether-l tablets | XX | XXX |
| - | Artemether-l tablets | XX | XXX |
| - | Artemether-l tablets | XX | XXX |
| - | Artemether-l tablets | XX | XXX |
| - | Artemether-l tablets | XX | XXX |
| - | Artemether-l tablets | XX | XXX |
| - | Artemether-l tablets | XX | XXX |
| - | Artemether-l tablets | XX | XXX |
| - | Artemether-l tablets | XX | XXX |
| - | Artemether-l tablets | XX | XXX |
| - | Artemether-l tablets | XX | XXX |
| - | Artemether-l tablets | XX | XXX |
| - | Artemether-l tablets | XX | XXX |
| - | Artemether-l tablets | XX | XXX |
| - | Artemether-l tablets | XX | XXX |
| - | Artemether-l tablets | XX | XXX |
| - | Artemether-l tablets | XX | XXX |
| - | Artemether-l tablets | XX | XXX |
| - | Artemether-l tablets | XX | XXX |
| - | Artemether-l tablets | XX | XXX |
| - | Artemether-l tablets | XX | XXX |
| - | Artemether-l tablets | XX | XXX |
| - | Artemether-l tablets | XX | XXX |
| - | Artemether-l tablets | XX | XXX |
| - | Artemether-l tablets | XX | XXX |
| - | Artemether-l tablets | XX | XXX |
| - | Artemether-l tablets | XX | XXX |
| - | Artemether-l tablets | XX | XXX |
| - | Artemether-l tablets | XX | XXX |

| | | | |
|---|---|---|---|
| - | Artemether-l tablets | XX | XXX |
| - | Artemether-l tablets | XX | XXX |
| - | Artemether-l tablets | XX | XXX |
| - | Artemether-l tablets | XX | XXX |
| - | Artemether-l tablets | XX | XXX |
| - | Artemether-l tablets | XX | XXX |
| - | Artemether-l tablets | XX | XXX |
| - | Artemether-l tablets | XX | XXX |
| - | Artemether-l tablets | XX | XXX |
| - | Artemether-l tablets | XX | XXX |
| - | Artemether-l tablets | XX | XXX |
| - | Artemether-l tablets | XX | XXX |
| - | Artemether-l tablets | XX | XXX |
| - | Artemether-l tablets | XX | XXX |
| - | Artemether-l tablets | XX | XXX |
| - | Artemether-l tablets | XX | XXX |
| - | Artemether-l tablets | XX | XXX |
| - | Artemether-l tablets | XX | XXX |
| - | Artemether-l tablets | XX | XXX |
| - | Artemether-l tablets | XX | XXX |
| - | Artemether-l tablets | XX | XXX |
| - | Artemether-l tablets | XX | XXX |
| - | Artemether-l tablets | XX | XXX |
| - | Artemether-l tablets | XX | XXX |
| - | Artemether-l tablets | XX | XXX |
| - | Artemether-l tablets | XX | XXX |
| - | Artemether-l tablets | XX | XXX |
| - | Artemether-l tablets | XX | XXX |
| - | Artemether-l tablets | XX | XXX |
| - | Artemether-l tablets | XX | XXX |
| - | Artemether-l tablets | XX | XXX |
| - | Artemether-l tablets | XX | XXX |
| - | Artemether-l tablets | XX | XXX |
| - | Artemether-l tablets | XX | XXX |
| - | Artemether-l tablets | XX | XXX |
| - | Artemether-l tablets | XX | XXX |
| - | Artemether-l tablets | XX | XXX |
| - | Artemether-l tablets | XX | XXX |
| - | Artemether-l tablets | XX | XXX |
| - | Artemether-l tablets | XX | XXX |
| - | Artemether-l tablets | XX | XXX |
| - | Artemether-l tablets | XX | XXX |
| - | Artemether-l tablets | XX | XXX |
| - | Artemether-l tablets | XX | XXX |
| - | Artemether-l tablets | XX | XXX |
| - | Artemether-l tablets | XX | XXX |
| - | Artemether-l tablets | XX | XXX |
| - | Artemether-l tablets | XX | XXX |
| - | Artemether-l tablets | XX | XXX |
| - | Artemether-l tablets | XX | XXX |

| | | | |
|---|---|---|---|
| - | Artemether-l tablets | XX | XXX |
| - | Artemether-l tablets | XX | XXX |
| - | Artemether-l tablets | XX | XXX |
| - | Artemether-l tablets | XX | XXX |
| - | Artemether-l tablets | XX | XXX |
| - | Artemether-l tablets | XX | XXX |
| - | Artemether-l tablets | XX | XXX |
| - | Artemether-l tablets | XX | XXX |
| - | Artemether-l tablets | XX | XXX |
| - | Artemether-l tablets | XX | XXX |
| - | Artemether-l tablets | XX | XXX |
| - | Artemether-l tablets | XX | XXX |
| - | Artemether-l tablets | XX | XXX |
| - | Artemether-l tablets | XX | XXX |
| - | Artemether-l tablets | XX | XXX |
| - | Artemether-l tablets | XX | XXX |
| - | Artemether-l tablets | XX | XXX |
| - | Artemether-l tablets | XX | XXX |
| - | Artemether-l tablets | XX | XXX |
| - | Artemether-l tablets | XX | XXX |
| - | Artemether-l tablets | XX | XXX |
| - | Artemether-l tablets | XX | XXX |
| - | Artemether-l tablets | XX | XXX |
| - | Artemether-l tablets | XX | XXX |
| - | Artemether-l tablets | XX | XXX |
| - | Artemether-l tablets | XX | XXX |
| - | Artemether-l tablets | XX | XXX |
| - | Artemether-l tablets | XX | XXX |
| - | Artemether-l tablets | XX | XXX |
| - | Artemether-l tablets | XX | XXX |
| - | Artemether-l tablets | XX | XXX |
| - | Artemether-l tablets | XX | XXX |
| - | Artemether-l tablets | XX | XXX |
| - | Artemether-l tablets | XX | XXX |
| - | Artemether-l tablets | XX | XXX |
| - | Artemether-l tablets | XX | XXX |
| - | Artemether-l tablets | XX | XXX |
| - | Artemether-l tablets | XX | XXX |
| - | Artemether-l tablets | XX | XXX |
| - | Artemether-l tablets | XX | XXX |
| - | Artemether-l tablets | XX | XXX |
| - | Artemether-l tablets | XX | XXX |
| - | Artemether-l tablets | XX | XXX |
| - | Artemether-l tablets | XX | XXX |
| - | Artemether-l tablets | XX | XXX |
| - | Artemether-l tablets | XX | XXX |
| - | Artemether-l tablets | XX | XXX |
| - | Artemether-l tablets | XX | XXX |
| - | Artemether-l tablets | XX | XXX |
| - | Artemether-l tablets | XX | XXX |

| | | | | |
|---|---|---|---|---|
| - | Artemether-l | tablets | XX | XXX |
| - | Artemether-l | tablets | XX | XXX |
| - | Artemether-l | tablets | XX | XXX |
| - | Artemether-l | tablets | XX | XXX |
| - | Artemether-l | tablets | XX | XXX |
| - | Artemether-l | tablets | XX | XXX |
| - | Artemether-l | tablets | XX | XXX |
| - | Artemether-l | tablets | XX | XXX |
| - | Artemether-l | tablets | XX | XXX |
| - | Artemether-l | tablets | XX | XXX |
| - | Artemether-l | tablets | XX | XXX |
| - | Artemether-l | tablets | XX | XXX |
| - | Artemether-l | tablets | XX | XXX |
| - | Artemether-l | tablets | XX | XXX |
| - | Artemether-l | tablets | XX | XXX |
| - | Artemether-l | tablets | XX | XXX |
| - | Artemether-l | tablets | XX | XXX |
| - | Artemether-l | tablets | XX | XXX |
| - | Artemether-l | tablets | XX | XXX |
| - | Artemether-l | tablets | XX | XXX |
| - | Artemether-l | tablets | XX | XXX |
| - | Artemether-l | tablets | XX | XXX |
| - | Artemether-l | tablets | XX | XXX |
| - | Artemether-l | tablets | XX | XXX |
| - | Artemether-l | tablets | XX | XXX |
| - | Artemether-l | tablets | XX | XXX |
| - | Artemether-l | tablets | XX | XXX |
| - | Artemether-l | tablets | XX | XXX |
| - | Artemether-l | tablets | XX | XXX |
| - | Artemether-l | tablets | XX | XXX |
| - | Artemether-l | tablets | XX | XXX |
| - | Artemether-l | tablets | XX | XXX |
| - | Artemether-l | tablets | XX | XXX |
| - | Artemether-l | tablets | XX | XXX |
| - | Artemether-l | tablets | XX | XXX |
| - | Artemether-l | tablets | XX | XXX |
| - | Artemether-l | tablets | XX | XXX |
| - | Artemether-l | tablets | XX | XXX |
| - | Artemether-l | tablets | XX | XXX |
| - | Artemether-l | tablets | XX | XXX |
| - | Artemether-l | tablets | XX | XXX |
| - | Artemether-l | tablets | XX | XXX |
| - | Artemether-l | tablets | XX | XXX |
| - | Artemether-l | tablets | XX | XXX |
| - | Artemether-l | tablets | XX | XXX |
| - | Artemether-l | tablets | XX | XXX |
| - | Artemether-l | tablets | XX | XXX |
| - | Artemether-l | tablets | XX | XXX |
| - | Artesunate | tablets | XX | XXX |
| - | Artesunate | tablets | XX | XXX |

| | | | | |
|---|---|---|---|---|
| - | Artesunate | tablets | XX | XXX |
| - | Artesunate | tablets | XX | XXX |
| - | Artesunate | tablets | XX | XXX |
| - | Artesunate | tablets | XX | XXX |
| - | Artesunate | tablets | XX | XXX |
| - | Artesunate | tablets | XX | XXX |
| - | Artesunate | tablets | XX | XXX |
| - | Artesunate | tablets | XX | XXX |
| - | Artesunate | tablets | XX | XXX |
| - | Artesunate | tablets | XX | XXX |
| - | Artesunate | tablets | XX | XXX |
| - | Artesunate | tablets | XX | XXX |
| - | Artesunate | tablets | XX | XXX |
| - | Artesunate | tablets | XX | XXX |
| - | Artesunate | tablets | XX | XXX |
| - | Artesunate | tablets | XX | XXX |
| - | Artesunate | tablets | XX | XXX |
| - | Artesunate | tablets | XX | XXX |
| - | Artesunate | tablets | XX | XXX |
| - | Artesunate | tablets | XX | XXX |
| - | Artesunate | tablets | XX | XXX |
| - | Artesunate | tablets | XX | XXX |
| - | Artesunate | tablets | XX | XXX |
| - | Artesunate | tablets | XX | XXX |
| - | Artesunate | tablets | XX | XXX |
| - | Artesunate | tablets | XX | XXX |
| - | Artesunate | tablets | XX | XXX |
| - | Artesunate | tablets | XX | XXX |
| - | Artesunate | tablets | XX | XXX |
| - | Artesunate | tablets | XX | XXX |
| - | Artesunate | tablets | XX | XXX |
| - | Artesunate | tablets | XX | XXX |
| - | Artesunate | tablets | XX | XXX |
| - | Artesunate | tablets | XX | XXX |
| - | Artesunate | tablets | XX | XXX |
| - | Artesunate | tablets | XX | XXX |
| - | Artesunate | tablets | XX | XXX |
| - | Artesunate | tablets | XX | XXX |
| - | Artesunate | tablets | XX | XXX |
| - | Artesunate-A | tablets | XX | XXX |
| - | Artesunate-A | tablets | XX | XXX |
| - | Artesunate-A | tablets | XX | XXX |
| - | Artesunate-A | tablets | XX | XXX |
| - | Artesunate-A | tablets | XX | XXX |
| - | Artesunate-A | tablets | XX | XXX |
| - | Artesunate-A | tablets | XX | XXX |
| - | Artesunate-A | tablets | XX | XXX |
| - | Artesunate-A | tablets | XX | XXX |
| - | Artesunate-A | tablets | XX | XXX |
| - | Artesunate-A | tablets | XX | XXX |

| | | | |
|---|---|---|---|
| - | Artesunate-A tablets | XX | XXX |
| - | Artesunate-A tablets | XX | XXX |
| - | Artesunate-A tablets | XX | XXX |
| - | Artesunate-A tablets | XX | XXX |
| - | Artesunate-A tablets | XX | XXX |
| - | Artesunate-A tablets | XX | XXX |
| - | Artesunate-A tablets | XX | XXX |
| - | Artesunate-A tablets | XX | XXX |
| - | Artesunate-A tablets | XX | XXX |
| - | Artesunate-A tablets | XX | XXX |
| - | Artesunate-A tablets | XX | XXX |
| - | Artesunate-A tablets | XX | XXX |
| - | Artesunate-A tablets | XX | XXX |
| - | Artesunate-A tablets | XX | XXX |
| - | Artesunate-A tablets | XX | XXX |
| - | Artesunate-A tablets | XX | XXX |
| - | Artesunate-A tablets | XX | XXX |
| - | Artesunate-A tablets | XX | XXX |
| - | Artesunate-A tablets | XX | XXX |
| - | Artesunate-A tablets | XX | XXX |
| - | Artesunate-A tablets | XX | XXX |
| - | Artesunate-A tablets | XX | XXX |
| - | Artesunate-A tablets | XX | XXX |
| - | Artesunate-A tablets | XX | XXX |
| - | Artesunate-A tablets | XX | XXX |
| - | Artesunate-A tablets | XX | XXX |
| - | Artesunate-A tablets | XX | XXX |
| - | Artesunate-A tablets | XX | XXX |
| - | Artesunate-A tablets | XX | XXX |
| - | Artesunate-A tablets | XX | XXX |
| - | Artesunate-A tablets | XX | XXX |
| - | Artesunate-A tablets | XX | XXX |
| - | Artesunate-A tablets | XX | XXX |
| - | Artesunate-A tablets | XX | XXX |
| - | Artesunate-A tablets | XX | XXX |
| - | Artesunate-A tablets | XX | XXX |
| - | Artesunate-A tablets | XX | XXX |
| - | Artesunate-A tablets | XX | XXX |
| - | Artesunate-A tablets | XX | XXX |
| - | Artesunate-A tablets | XX | XXX |
| - | Artesunate-A tablets | XX | XXX |
| - | Artesunate-A tablets | XX | XXX |
| - | Artesunate-A tablets | XX | XXX |
| - | Artesunate-A tablets | XX | XXX |
| - | Artesunate-A tablets | XX | XXX |
| - | Artesunate-A tablets | XX | XXX |
| - | Artesunate-A tablets | XX | XXX |
| - | Artesunate-A tablets | XX | XXX |
| - | Artesunate-A tablets | XX | XXX |
| - | Artesunate-A tablets | XX | XXX |

| | | | | |
|---|---|---|---|---|
| - | Artesunate-A | tablets | XX | XXX |
| - | Artesunate-A | tablets | XX | XXX |
| - | Artesunate-A | tablets | XX | XXX |
| - | Artesunate-A | tablets | XX | XXX |
| - | Artesunate-A | tablets | XX | XXX |
| - | Artesunate-A | tablets | XX | XXX |
| - | Artesunate-A | tablets | XX | XXX |
| - | Artesunate-A | tablets | XX | XXX |
| - | Artesunate-A | tablets | XX | XXX |
| - | Artesunate-A | tablets | XX | XXX |
| - | Artesunate-A | tablets | XX | XXX |
| - | Artesunate-N | tablets | XX | XXX |
| - | Artesunate-N | tablets | XX | XXX |
| - | Artesunate-N | tablets | XX | XXX |
| - | Artesunate-N | tablets | XX | XXX |
| - | Artesunate-N | tablets | XX | XXX |
| - | Artesunate-N | tablets | XX | XXX |
| - | Artesunate-N | tablets | XX | XXX |
| - | Artesunate-N | tablets | XX | XXX |
| - | Artesunate-N | tablets | XX | XXX |
| - | Artesunate-N | tablets | XX | XXX |
| - | Artesunate-N | tablets | XX | XXX |
| - | Artesunate-N | tablets | XX | XXX |
| - | Artesunate-N | tablets | XX | XXX |
| - | Artesunate-N | tablets | XX | XXX |
| - | Artesunate-N | tablets | XX | XXX |
| - | Artesunate-N | tablets | XX | XXX |
| - | Artesunate-N | tablets | XX | XXX |
| - | Artesunate-N | tablets | XX | XXX |
| - | Artesunate-N | tablets | XX | XXX |
| - | Artesunate-N | tablets | XX | XXX |
| - | Artesunate-N | tablets | XX | XXX |
| - | Artesunate-N | tablets | XX | XXX |
| - | Artesunate-N | tablets | XX | XXX |
| - | Artesunate-N | tablets | XX | XXX |
| - | Artesunate-N | tablets | XX | XXX |
| 93.6 | Artesunate-S | tablets | XX | XXX |
| 89.9 | Artesunate-S | tablets | XX | XXX |
| 89.4 | Artesunate | tablets | XX | XXX |
| 102.5 | Artesunate-S | tablets | XX | XXX |
| 98.7 | Artesunate-S | tablets | XX | XXX |
| 92.6 | Artesunate-S | tablets | XX | XXX |
| 95.6 | Artesunate-S | tablets | XX | XXX |
| 96.4 | Artesunate-S | tablets | XX | XXX |
| 96.1 | Artesunate-S | tablets | XX | XXX |
| 97.5 | Artesunate-S | tablets | XX | XXX |
| 91 | Artesunate-S | tablets | XX | XXX |
| 110.2 | Artesunate-S | tablets | XX | XXX |
| 89.1 | Artesunate-S | tablets | XX | XXX |
| 98.3 | Artesunate-S | tablets | XX | XXX |

| | | | | |
|---|---|---|---|---|
| 99.2 | Artesunate-S | tablets | XX | XXX |
| 98.8 | Artesunate-S | tablets | XX | XXX |
| 91.7 | Artesunate-S | tablets | XX | XXX |
| 94.1 | Artesunate-S | tablets | XX | XXX |
| 106.4 | Artesunate-S | tablets | XX | XXX |
| 96.4 | Artesunate-S | tablets | XX | XXX |
| 95.8 | Artesunate-S | tablets | XX | XXX |
| 103.3 | Artesunate-S | tablets | XX | XXX |
| 93.4 | Artesunate-S | tablets | XX | XXX |
| 95.3 | Artesunate-S | tablets | XX | XXX |
| 97.4 | Artesunate-S | tablets | XX | XXX |
| - | Dihydroarten | tablets | XX | XXX |
| - | Dihydroarten | tablets | XX | XXX |
| - | Dihydroarten | tablets | XX | XXX |
| - | Dihydroarten | tablets | XX | XXX |
| - | Dihydroarten | tablets | XX | XXX |
| - | Dihydroarten | tablets | XX | XXX |
| - | Dihydroarten | tablets | XX | XXX |
| - | ACETAMINOI | tablets | XX | XXX |
| - | Dihydroarten | tablets | XX | XXX |
| - | Dihydroarten | tablets | XX | XXX |
| - | Dihydroarten | tablets | XX | XXX |
| - | Dihydroarten | tablets | XX | XXX |
| - | Dihydroarten | tablets | XX | XXX |
| - | Dihydroarten | tablets | XX | XXX |
| - | Dihydroarten | tablets | XX | XXX |
| - | Dihydroarten | tablets | XX | XXX |
| - | Dihydroarten | tablets | XX | XXX |
| - | Dihydroarten | tablets | XX | XXX |
| - | Dihydroarten | tablets | XX | XXX |
| - | Dihydroarten | tablets | XX | XXX |
| - | Dihydroarten | tablets | XX | XXX |
| - | Dihydroarten | tablets | XX | XXX |
| - | ACETAMINOI | tablets | XX | XXX |
| - | Dihydroarten | tablets | XX | XXX |
| - | ACETAMINOI | tablets | XX | XXX |
| - | Dihydroarten | tablets | XX | XXX |
| - | Dihydroarten | tablets | XX | XXX |
| - | Dihydroarten | tablets | XX | XXX |
| - | Dihydroarten | tablets | XX | XXX |
| - | Dihydroarten | tablets | XX | XXX |
| - | Dihydroarten | tablets | XX | XXX |
| - | Dihydroarten | tablets | XX | XXX |
| - | Dihydroarten | tablets | XX | XXX |
| - | ACETAMINOI | tablets | XX | XXX |
| - | Dihydroarten | tablets | XX | XXX |
| - | Dihydroarten | tablets | XX | XXX |
| - | Dihydroarten | tablets | XX | XXX |
| - | Dihydroarten | tablets | XX | XXX |
| - | Dihydroarten | tablets | XX | XXX |

| | | | |
|---|---|---|---|
| - | Dihydroarten tablets | XX | XXX |
| - | Dihydroarten tablets | XX | XXX |
| - | Dihydroarten tablets | XX | XXX |
| - | Dihydroarten tablets | XX | XXX |
| - | Dihydroarten tablets | XX | XXX |
| - | Dihydroarten tablets | XX | XXX |
| - | Dihydroarten tablets | XX | XXX |
| - | Dihydroarten tablets | XX | XXX |
| - | Dihydroarten tablets | XX | XXX |
| - | Dihydroarten tablets | XX | XXX |
| - | Dihydroarten tablets | XX | XXX |
| - | Dihydroarten tablets | XX | XXX |
| - | Dihydroarten tablets | XX | XXX |
| - | Dihydroarten tablets | XX | XXX |
| - | Dihydroarten tablets | XX | XXX |
| - | Dihydroarten tablets | XX | XXX |
| - | Dihydroarten tablets | XX | XXX |
| - | Dihydroarten tablets | XX | XXX |
| - | Dihydroarten tablets | XX | XXX |
| - | Dihydroarten tablets | XX | XXX |
| - | Dihydroarten tablets | XX | XXX |
| - | Dihydroarten tablets | XX | XXX |
| - | Dihydroarten tablets | XX | XXX |
| - | Dihydroarten tablets | XX | XXX |
| - | Dihydroarten tablets | XX | XXX |
| - | Dihydroarten tablets | XX | XXX |
| - | Dihydroarten tablets | XX | XXX |
| - | Dihydroarten tablets | XX | XXX |
| - | Dihydroarten tablets | XX | XXX |
| - | Dihydroarten tablets | XX | XXX |
| - | Dihydroarten tablets | XX | XXX |
| - | Dihydroarten tablets | XX | XXX |
| - | Dihydroarten tablets | XX | XXX |
| - | Dihydroarten tablets | XX | XXX |
| - | Dihydroarten tablets | XX | XXX |
| - | Dihydroarten tablets | XX | XXX |
| - | Dihydroarten tablets | XX | XXX |
| - | Dihydroarten tablets | XX | XXX |
| - | Dihydroarten tablets | XX | XXX |
| - | Dihydroarten tablets | XX | XXX |
| - | Dihydroarten tablets | XX | XXX |
| - | Dihydroarten tablets | XX | XXX |
| - | Dihydroarten tablets | XX | XXX |
| - | Dihydroarten tablets | XX | XXX |
| - | Dihydroarten tablets | XX | XXX |
| - | Dihydroarten tablets | XX | XXX |
| - | Dihydroarten tablets | XX | XXX |
| - | Dihydroarten tablets | XX | XXX |
| - | Dihydroarten tablets | XX | XXX |
| - | Dihydroarten tablets | XX | XXX |

| | | | | |
|---|---|---|---|---|
| - | Dihydroarten | tablets | XX | XXX |
| - | Dihydroarten | tablets | XX | XXX |
| - | Dihydroarten | tablets | XX | XXX |
| - | Dihydroarten | tablets | XX | XXX |
| - | Dihydroarten | tablets | XX | XXX |
| - | Dihydroarten | tablets | XX | XXX |
| - | Dihydroarten | tablets | XX | XXX |
| - | Dihydroarten | tablets | XX | XXX |
| - | Dihydroarten | tablets | XX | XXX |
| - | Dihydroarten | tablets | XX | XXX |
| - | Dihydroarten | tablets | XX | XXX |
| - | Dihydroarten | tablets | XX | XXX |
| - | Dihydroarten | tablets | XX | XXX |
| - | Dihydroarten | tablets | XX | XXX |
| - | Dihydroarten | tablets | XX | XXX |
| - | Dihydroarten | tablets | XX | XXX |
| - | Dihydroarten | tablets | XX | XXX |
| - | Dihydroarten | tablets | XX | XXX |
| - | Dihydroarten | tablets | XX | XXX |
| - | Dihydroarten | tablets | XX | XXX |
| - | Dihydroarten | tablets | XX | XXX |
| - | Dihydroarten | tablets | XX | XXX |
| - | Dihydroarten | tablets | XX | XXX |
| - | Dihydroarten | tablets | XX | XXX |
| - | Dihydroarten | tablets | XX | XXX |
| - | Dihydroarten | tablets | XX | XXX |
| - | Dihydroarten | tablets | XX | XXX |
| - | Dihydroarten | tablets | XX | XXX |
| - | Dihydroarten | tablets | XX | XXX |
| - | Dihydroarten | tablets | XX | XXX |
| - | Dihydroarten | tablets | XX | XXX |
| - | Dihydroarten | tablets | XX | XXX |
| - | Dihydroarten | tablets | XX | XXX |
| - | Dihydroarten | tablets | XX | XXX |
| - | Dihydroarten | tablets | XX | XXX |
| - | Dihydroarten | tablets | XX | XXX |
| - | Dihydroarten | tablets | XX | XXX |
| - | Dihydroarten | tablets | XX | XXX |
| - | Dihydroarten | tablets | XX | XXX |
| - | Dihydroarten | tablets | XX | XXX |
| - | Dihydroarten | tablets | XX | XXX |
| - | Dihydroarten | tablets | XX | XXX |
| - | Dihydroarten | tablets | XX | XXX |
| - | Artemether-l | capsules | XX | XXX |
| - | Artemether-l | other (capsul | XX | XXX |
| - | Artemether-l | other (capsul | XX | XXX |
| - | Artemether-l | granule | XX | XXX |
| - | not sent to A | suspension | XX | XXX |
| - | sent to Atlan | suspension | XX | XXX |

| | | | |
|---|---|---|---|
| - | not sent to A suspension | XX | XXX |
| - | sent to Atlan suspension | XX | XXX |
| - | not sent to A suspension | XX | XXX |
| - | sent to Atlan suspension | XX | XXX |
| - | not sent to A suspension | XX | XXX |
| - | not sent to A suspension | XX | XXX |
| - | sent to Atlan suspension | XX | XXX |
| - | not sent to A suspension | XX | XXX |
| - | not sent to A suspension | XX | XXX |
| - | sent to Atlan suspension | XX | XXX |
| - | not sent to A suspension | XX | XXX |
| - | not sent to A suspension | XX | XXX |
| - | not sent to A suspension | XX | XXX |
| - | sent to Atlan suspension | XX | XXX |
| - | not sent to A suspension | XX | XXX |
| - | sent to Atlan suspension | XX | XXX |
| - | not sent to A suspension | XX | XXX |
| - | sent to Atlan suspension | XX | XXX |
| - | not sent to A suspension | XX | XXX |
| - | not sent to A suspension | XX | XXX |
| - | sent to Atlan suspension | XX | XXX |
| - | not sent to A suspension | XX | XXX |
| - | sent to Atlan suspension | XX | XXX |
| - | not sent to A suspension | XX | XXX |
| - | sent to Atlan suspension | XX | XXX |
| - | not sent to A suspension | XX | XXX |
| - | sent to Atlan suspension | XX | XXX |
| - | not sent to A suspension | XX | XXX |
| - | not sent to A suspension | XX | XXX |
| - | not sent to A suspension | XX | XXX |
| - | not sent to A suspension | XX | XXX |
| - | sent to Atlan suspension | XX | XXX |
| - | not sent to A suspension | XX | XXX |
| - | sent to Atlan suspension | XX | XXX |
| - | not sent to A suspension | XX | XXX |
| - | not sent to A suspension | XX | XXX |
| - | not sent to A suspension | XX | XXX |
| - | sent to Atlan suspension | XX | XXX |
| - | not sent to A suspension | XX | XXX |
| - | sent to Atlan suspension | XX | XXX |
| - | not sent to A suspension | XX | XXX |
| - | not sent to A suspension | XX | XXX |
| - | not sent to A suspension | XX | XXX |
| - | not sent to A suspension | XX | XXX |
| - | not sent to A suspension | XX | XXX |
| - | sent to Atlan suspension | XX | XXX |
| - | not sent to A suspension | XX | XXX |
| - | sent to Atlan suspension | XX | XXX |
| - | not sent to A suspension | XX | XXX |
| - | sent to Atlan suspension | XX | XXX |

| | | | |
|---|---|---|---|
| - | not sent to A suspension | XX | XXX |
| - | sent to Atlan suspension | XX | XXX |
| - | not sent to A suspension | XX | XXX |
| - | not sent to A suspension | XX | XXX |
| - | not sent to A suspension | XX | XXX |
| - | sent to Atlan suspension | XX | XXX |
| - | not sent to A suspension | XX | XXX |
| - | not sent to A suspension | XX | XXX |
| - | sent to Atlan suspension | XX | XXX |
| - | not sent to A suspension | XX | XXX |
| - | sent to Atlan suspension | XX | XXX |
| - | not sent to A suspension | XX | XXX |
| - | sent to Atlan suspension | XX | XXX |
| - | not sent to A suspension | XX | XXX |
| - | sent to Atlan suspension | XX | XXX |
| - | not sent to A suspension | XX | XXX |
| - | sent to Atlan suspension | XX | XXX |
| - | not sent to A suspension | XX | XXX |
| - | sent to Atlan suspension | XX | XXX |
| - | not sent to A suspension | XX | XXX |
| - | sent to Atlan suspension | XX | XXX |
| - | not sent to A suspension | XX | XXX |
| - | sent to Atlan suspension | XX | XXX |
| - | not sent to A suspension | XX | XXX |
| - | sent to Atlan suspension | XX | XXX |
| - | not sent to A suspension | XX | XXX |
| - | sent to Atlan suspension | XX | XXX |
| - | not sent to A suspension | XX | XXX |
| - | not sent to A suspension | XX | XXX |
| - | Artesunate-A granule | XX | XXX |
| - | Artesunate-A granule | XX | XXX |
| - | Artesunate-A granule | XX | XXX |
| - | Artesunate-A granule | XX | XXX |
| - | Artesunate-A granule | XX | XXX |
| - | Artesunate-A granule | XX | XXX |
| - | Artesunate-A granule | XX | XXX |
| - | Artesunate-A granule | XX | XXX |
| - | Artesunate-A granule | XX | XXX |
| - | Artesunate-A granule | XX | XXX |
| - | Artesunate-A granule | XX | XXX |
| - | Artesunate-A granule | XX | XXX |
| - | not sent to A suspension | XX | XXX |
| - | sent to Atlan suspension | XX | XXX |
| - | not sent to A suspension | XX | XXX |
| - | sent to Atlan suspension | XX | XXX |
| - | not sent to A suspension | XX | XXX |
| - | sent to Atlan suspension | XX | XXX |
| - | not sent to A suspension | XX | XXX |
| - | sent to Atlan suspension | XX | XXX |
| - | not sent to A suspension | XX | XXX |

| | | | | |
|---|---|---|---|---|
| - | sent to Atlan | suspension | XX | XXX |
| - | not sent to A | suspension | XX | XXX |
| - | sent to Atlan | suspension | XX | XXX |
| - | not sent to A | suspension | XX | XXX |
| - | sent to Atlan | suspension | XX | XXX |
| - | not sent to A | suspension | XX | XXX |
| - | sent to Atlan | suspension | XX | XXX |
| - | not sent to A | suspension | XX | XXX |
| - | sent to Atlan | suspension | XX | XXX |
| - | not sent to A | suspension | XX | XXX |
| - | not sent to A | suspension | XX | XXX |
| - | not sent to A | suspension | XX | XXX |
| - | not sent to A | suspension | XX | XXX |
| - | sent to Atlan | suspension | XX | XXX |
| - | not sent to A | suspension | XX | XXX |
| - | not sent to A | suspension | XX | XXX |
| - | sent to Atlan | suspension | XX | XXX |
| - | not sent to A | suspension | XX | XXX |
| - | sent to Atlan | suspension | XX | XXX |
| - | not sent to A | suspension | XX | XXX |
| - | sent to Atlan | suspension | XX | XXX |
| - | not sent to A | suspension | XX | XXX |
| - | not sent to A | suspension | XX | XXX |
| - | not sent to A | suspension | XX | XXX |
| - | sent to Atlan | suspension | XX | XXX |
| - | not sent to A | suspension | XX | XXX |
| - | sent to Atlan | suspension | XX | XXX |
| - | not sent to A | suspension | XX | XXX |
| - | not sent to A | suspension | XX | XXX |
| - | sent to Atlan | suspension | XX | XXX |
| - | not sent to A | suspension | XX | XXX |
| - | sent to Atlan | suspension | XX | XXX |
| - | not sent to A | suspension | XX | XXX |
| - | sent to Atlan | suspension | XX | XXX |

| UniqueKey | Recstatus | dateofcapture | otherbname | altitude | coordinateN |
|---|---|---|---|---|---|
| 1093 | 1 | 26/06/2012 | | 174 | 6.43651 |
| 1135 | 1 | 28/06/2012 | | 173 | 6.458 |
| 1148 | 1 | 28/06/2012 | | 174 | 6.41256 |
| 1166 | 1 | 29/06/2012 | | 243 | 6.48775 |
| 1189 | 1 | 29/06/2012 | | 137 | 6.47443 |
| 1204 | 1 | 29/06/2012 | | 176 | 6.49644 |
| 1195 | 1 | 29/06/2012 | | 137 | 6.47443 |
| 1128 | 1 | 28/06/2012 | | 173 | 6.458 |
| 1192 | 1 | 29/06/2012 | | 137 | 6.47443 |
| 1271 | 1 | 29/06/2012 | | 101 | 6.41292 |
| 1113 | 1 | 26/06/2012 | | 174 | 6.43651 |
| 1137 | 1 | 28/06/2012 | | 173 | 6.458 |
| 1145 | 1 | 28/06/2012 | | 125 | 6.46048 |
| 1155 | 1 | 29/06/2012 | | 144 | 6.46361 |
| 1167 | 1 | 29/06/2012 | | 243 | 6.48775 |
| 1182 | 1 | 29/06/2012 | | 198 | 6.47732 |
| 1191 | 1 | 29/06/2012 | | 137 | 6.47443 |
| 1209 | 1 | 29/06/2012 | | 176 | 6.49644 |
| 1280 | 1 | 02/07/2012 | | 156 | 6.42479 |
| 1125 | 1 | 28/06/2012 | | 173 | 6.458 |
| 1154 | 1 | 29/06/2012 | | 144 | 6.46361 |
| 1181 | 1 | 29/06/2012 | | 198 | 6.47732 |
| 1194 | 1 | 29/06/2012 | | 137 | 6.47443 |
| 1203 | 1 | 29/06/2012 | | 155 | 6.47633 |
| 1208 | 1 | 29/06/2012 | | 176 | 6.49644 |
| 1221 | 1 | 29/06/2012 | | 165 | 6.43893 |
| 1230 | 1 | 29/06/2012 | | 116 | 6.37491 |
| 1234 | 1 | 29/06/2012 | | 174 | 6.4105 |
| 1250 | 1 | 29/06/2012 | | 164 | 6.42491 |
| 1296 | 1 | 02/07/2012 | | 0 | 0 |
| 1130 | 1 | 28/06/2012 | | 173 | 6.458 |
| 1222 | 1 | 29/06/2012 | | 165 | 6.43893 |
| 1238 | 1 | 29/06/2012 | | 122 | 6.42468 |
| 1245 | 1 | 29/06/2012 | | 174 | 6.41548 |
| 1264 | 1 | 29/06/2012 | | 101 | 6.41292 |
| 1270 | 1 | 29/06/2012 | | 101 | 6.41292 |
| 1151 | 1 | 28/06/2012 | | 174 | 6.41256 |
| 1165 | 1 | 29/06/2012 | | 243 | 6.48775 |
| 1231 | 1 | 29/06/2012 | | 116 | 6.37491 |
| 1241 | 1 | 29/06/2012 | | 122 | 6.42468 |
| 1246 | 1 | 29/06/2012 | | 174 | 6.41548 |
| 1277 | 1 | 02/07/2012 | | 156 | 6.42479 |
| 1289 | 1 | 02/07/2012 | | 138 | 6.44296 |
| 1293 | 1 | 02/07/2012 | | 0 | 0 |
| 1220 | 1 | 29/06/2012 | | 165 | 6.43893 |
| 1217 | 1 | 29/06/2012 | | 135 | 6.44303 |

| | | | | |
|---|---|---|---|---|
| 1211 | 1 | 29/06/2012 | 176 | 6.49644 |
| 1141 | 1 | 28/06/2012 | 173 | 6.458 |
| 1161 | 1 | 29/06/2012 | 144 | 6.46361 |
| 1176 | 1 | 29/06/2012 | 198 | 6.47732 |
| 1210 | 1 | 29/06/2012 | 176 | 6.49644 |
| 1216 | 1 | 29/06/2012 | 135 | 6.44303 |
| 1224 | 1 | 29/06/2012 | 145 | 6.39289 |
| 1239 | 1 | 29/06/2012 | 122 | 6.42468 |
| 1261 | 1 | 29/06/2012 | 101 | 6.41292 |
| 1101 | 1 | 26/06/2012 | 174 | 6.43651 |
| 1199 | 1 | 29/06/2012 | 137 | 6.47443 |
| 1122 | 1 | 28/06/2012 | 173 | 6.458 |
| 1103 | 1 | 26/06/2012 | 174 | 6.43651 |
| 1119 | 1 | 28/06/2012 | 173 | 6.458 |
| 1257 | 1 | 29/06/2012 | 164 | 6.42491 |
| 1283 | 1 | 02/07/2012 | 156 | 6.42479 |
| 1104 | 1 | 26/06/2012 | 174 | 6.43651 |
| 1172 | 1 | 29/06/2012 | 243 | 6.48775 |
| 1212 | 1 | 29/06/2012 | 135 | 6.44303 |
| 1285 | 1 | 02/07/2012 | 156 | 6.42479 |
| 1116 | 1 | 26/06/2012 | 174 | 6.43651 |
| 1170 | 1 | 29/06/2012 | 243 | 6.48775 |
| 1183 | 1 | 29/06/2012 | 198 | 6.47732 |
| 1254 | 1 | 29/06/2012 | 164 | 6.42491 |
| 1115 | 1 | 26/06/2012 | 174 | 6.43651 |
| 1180 | 1 | 29/06/2012 | 198 | 6.47732 |
| 1109 | 1 | 26/06/2012 | 174 | 6.43651 |
| 1136 | 1 | 28/06/2012 | 173 | 6.458 |
| 1163 | 1 | 29/06/2012 | 144 | 6.46361 |
| 1262 | 1 | 29/06/2012 | 101 | 6.41292 |
| 1276 | 1 | 02/07/2012 | 156 | 6.42479 |
| 1110 | 1 | 26/06/2012 | 174 | 6.43651 |
| 1124 | 1 | 28/06/2012 | 173 | 6.458 |
| 1247 | 1 | 29/06/2012 | 174 | 6.41548 |
| 1100 | 1 | 26/06/2012 | 174 | 6.43651 |
| 1126 | 1 | 28/06/2012 | 173 | 6.458 |
| 1112 | 1 | 26/06/2012 | 174 | 6.43651 |
| 1138 | 1 | 28/06/2012 | 173 | 6.458 |
| 1144 | 1 | 28/06/2012 | 125 | 6.46048 |
| 1156 | 1 | 29/06/2012 | 144 | 6.46361 |
| 1226 | 1 | 29/06/2012 | 145 | 6.39289 |
| 1092 | 1 | 26/06/2012 | 174 | 6.43651 |
| 1129 | 1 | 28/06/2012 | 173 | 6.458 |
| 1146 | 1 | 28/06/2012 | 125 | 6.46048 |
| 1150 | 1 | 28/06/2012 | 174 | 6.41256 |
| 1169 | 1 | 29/06/2012 | 243 | 6.48775 |
| 1184 | 1 | 29/06/2012 | 198 | 6.47732 |
| 1196 | 1 | 29/06/2012 | 137 | 6.47443 |
| 1201 | 1 | 29/06/2012 | 155 | 6.47633 |
| 1206 | 1 | 29/06/2012 | 176 | 6.49644 |

| | | | | |
|---|---|---|---|---|
| 1223 | 1 | 29/06/2012 | 165 | 6.43893 |
| 1228 | 1 | 29/06/2012 | 145 | 6.39289 |
| 1248 | 1 | 29/06/2012 | 164 | 6.42491 |
| 1267 | 1 | 29/06/2012 | 101 | 6.41292 |
| 1281 | 1 | 02/07/2012 | 156 | 6.42479 |
| 1291 | 1 | 02/07/2012 | 0 | 0 |
| 1095 | 1 | 26/06/2012 | 174 | 6.43651 |
| 1120 | 1 | 28/06/2012 | 173 | 6.458 |
| 1168 | 1 | 29/06/2012 | 243 | 6.48775 |
| 1265 | 1 | 29/06/2012 | 101 | 6.41292 |
| 1133 | 1 | 28/06/2012 | 173 | 6.458 |
| 1091 | 1 | 26/06/2012 | 174 | 6.43651 |
| 1131 | 1 | 28/06/2012 | 173 | 6.458 |
| 1200 | 1 | 29/06/2012 | 137 | 6.47443 |
| 1249 | 1 | 29/06/2012 | 164 | 6.42491 |
| 1269 | 1 | 29/06/2012 | 101 | 6.41292 |
| 1274 | 1 | 02/07/2012 | 156 | 6.42479 |
| 1295 | 1 | 02/07/2012 | 0 | 0 |
| 1090 | 1 | 26/06/2012 | 174 | 6.43651 |
| 1118 | 1 | 28/06/2012 | 173 | 6.458 |
| 1158 | 1 | 29/06/2012 | 144 | 6.46361 |
| 1190 | 1 | 29/06/2012 | 137 | 6.47443 |
| 1098 | 1 | 26/06/2012 | 174 | 6.43651 |
| 1117 | 1 | 28/06/2012 | 173 | 6.458 |
| 1153 | 1 | 29/06/2012 | 144 | 6.46361 |
| 1175 | 1 | 29/06/2012 | 198 | 6.47732 |
| 1197 | 1 | 29/06/2012 | 137 | 6.47443 |
| 1251 | 1 | 29/06/2012 | 164 | 6.42491 |
| 1258 | 1 | 29/06/2012 | 101 | 6.41292 |
| 1173 | 1 | 29/06/2012 | 243 | 6.48775 |
| 1236 | 1 | 29/06/2012 | 174 | 6.4105 |
| 1244 | 1 | 29/06/2012 | 122 | 6.42468 |
| 1273 | 1 | 02/07/2012 | 156 | 6.42479 |
| 1275 | 1 | 02/07/2012 | 156 | 6.42479 |
| 1152 | 1 | 28/06/2012 | 174 | 6.41256 |
| 1232 | 1 | 29/06/2012 | 116 | 6.37491 |
| 1260 | 1 | 29/06/2012 | 101 | 6.41292 |
| 1243 | 1 | 29/06/2012 | 122 | 6.42468 |
| 1185 | 1 | 29/06/2012 | 198 | 6.47732 |
| 1297 | 1 | 02/07/2012 | 198 | 6.47732 |
| 1299 | 1 | 02/07/2012 | 243 | 6.48775 |
| 1219 | 1 | 29/06/2012 | 165 | 6.43893 |
| 1227 | 1 | 29/06/2012 | 145 | 6.39289 |
| 1121 | 1 | 28/06/2012 | 173 | 6.458 |
| 1259 | 1 | 29/06/2012 | 101 | 6.41292 |
| 1290 | 1 | 02/07/2012 | 138 | 6.44296 |
| 1292 | 1 | 02/07/2012 | 0 | 0 |
| 1097 | 1 | 26/06/2012 | 174 | 6.43651 |
| 1134 | 1 | 28/06/2012 | 173 | 6.458 |

| | | | | |
|---|---|---|---|---|
| 1159 | 1 | 29/06/2012 | 144 | 6.46361 |
| 1198 | 1 | 29/06/2012 | 137 | 6.47443 |
| 1207 | 1 | 29/06/2012 | 176 | 6.49644 |
| 1214 | 1 | 29/06/2012 | 135 | 6.44303 |
| 1107 | 1 | 26/06/2012 | 174 | 6.43651 |
| 1288 | 1 | 02/07/2012 | 138 | 6.44296 |
| 1213 | 1 | 29/06/2012 | 135 | 6.44303 |
| 1140 | 1 | 28/06/2012 | 173 | 6.458 |
| 1253 | 1 | 29/06/2012 | 164 | 6.42491 |
| 1298 | 1 | 02/07/2012 | 198 | 6.47732 |
| 1301 | 1 | 02/07/2012 | 243 | 6.48775 |
| 1106 | 1 | 26/06/2012 | 174 | 6.43651 |
| 1215 | 1 | 29/06/2012 | 135 | 6.44303 |
| 1268 | 1 | 29/06/2012 | 101 | 6.41292 |
| 1096 | 1 | 26/06/2012 | 174 | 6.43651 |
| 1143 | 1 | 28/06/2012 | 125 | 6.46048 |
| 1160 | 1 | 29/06/2012 | 144 | 6.46361 |
| 1178 | 1 | 29/06/2012 | 198 | 6.47732 |
| 1266 | 1 | 29/06/2012 | 101 | 6.41292 |
| 1284 | 1 | 02/07/2012 | 156 | 6.42479 |
| 1286 | 1 | 02/07/2012 | 157 | 6.42643 |
| 1111 | 1 | 26/06/2012 | 174 | 6.43651 |
| 1105 | 1 | 26/06/2012 | 174 | 6.43651 |
| 1099 | 1 | 26/06/2012 | 174 | 6.43651 |
| 1177 | 1 | 29/06/2012 | 198 | 6.47732 |
| 1108 | 1 | 26/06/2012 | 174 | 6.43651 |
| 1123 | 1 | 28/06/2012 | 173 | 6.458 |
| 1179 | 1 | 29/06/2012 | 198 | 6.47732 |
| 1263 | 1 | 29/06/2012 | 101 | 6.41292 |
| 1287 | 1 | 02/07/2012 | 157 | 6.42643 |
| 1114 | 1 | 26/06/2012 | 174 | 6.43651 |
| 1132 | 1 | 28/06/2012 | 173 | 6.458 |
| 1149 | 1 | 28/06/2012 | 174 | 6.41256 |
| 1157 | 1 | 29/06/2012 | 144 | 6.46361 |
| 1171 | 1 | 29/06/2012 | 243 | 6.48775 |
| 1187 | 1 | 29/06/2012 | 198 | 6.47732 |
| 1193 | 1 | 29/06/2012 | 137 | 6.47443 |
| 1202 | 1 | 29/06/2012 | 155 | 6.47633 |
| 1218 | 1 | 29/06/2012 | 165 | 6.43893 |
| 1225 | 1 | 29/06/2012 | 145 | 6.39289 |
| 1229 | 1 | 29/06/2012 | 116 | 6.37491 |
| 1235 | 1 | 29/06/2012 | 174 | 6.4105 |
| 1242 | 1 | 29/06/2012 | 122 | 6.42468 |
| 1282 | 1 | 02/07/2012 | 156 | 6.42479 |
| 1294 | 1 | 02/07/2012 | 0 | 0 |
| 1102 | 1 | 26/06/2012 | 174 | 6.43651 |
| 1127 | 1 | 28/06/2012 | 173 | 6.458 |
| 1162 | 1 | 29/06/2012 | 144 | 6.46361 |

| | | | | |
|---|---|---|---|---|
| 1164 | 1 | 29/06/2012 | 243 | 6.48775 |
| 1186 | 1 | 29/06/2012 | 198 | 6.47732 |
| 1205 | 1 | 29/06/2012 | 176 | 6.49644 |
| 1240 | 1 | 29/06/2012 | 122 | 6.42468 |
| 1252 | 1 | 29/06/2012 | 164 | 6.42491 |
| 1094 | 1 | 26/06/2012 | 174 | 6.43651 |
| 1300 | 1 | 02/07/2012 | 243 | 6.48775 |

| licensor | fdc | amfm | batchno | mfddate | expdate |
|---|---|---|---|---|---|
| XXXX | y | n | XXXXX | XXXXXX | XXXXXXX |
| XXXX | y | n | XXXXX | XXXXXX | XXXXXXX |
| XXXX | y | n | XXXXX | XXXXXX | XXXXXXX |
| XXXX | y | y | XXXXX | XXXXXX | XXXXXXX |
| XXXX | y | y | XXXXX | XXXXXX | XXXXXXX |
| XXXX | y | y | XXXXX | XXXXXX | XXXXXXX |
| XXXX | y | y | XXXXX | XXXXXX | XXXXXXX |
| XXXX | y | y | XXXXX | XXXXXX | XXXXXXX |
| XXXX | y | y | XXXXX | XXXXXX | XXXXXXX |
| XXXX | y | y | XXXXX | XXXXXX | XXXXXXX |
| XXXX | y | y | XXXXX | XXXXXX | XXXXXXX |
| XXXX | y | y | XXXXX | XXXXXX | XXXXXXX |
| XXXX | y | y | XXXXX | XXXXXX | XXXXXXX |
| XXXX | y | y | XXXXX | XXXXXX | XXXXXXX |
| XXXX | y | y | XXXXX | XXXXXX | XXXXXXX |
| XXXX | y | y | XXXXX | XXXXXX | XXXXXXX |
| XXXX | y | y | XXXXX | XXXXXX | XXXXXXX |
| XXXX | y | y | XXXXX | XXXXXX | XXXXXXX |
| XXXX | y | y | XXXXX | XXXXXX | XXXXXXX |
| XXXX | y | y | XXXXX | XXXXXX | XXXXXXX |
| XXXX | y | y | XXXXX | XXXXXX | XXXXXXX |
| XXXX | y | y | XXXXX | XXXXXX | XXXXXXX |
| XXXX | y | y | XXXXX | XXXXXX | XXXXXXX |
| XXXX | y | y | XXXXX | XXXXXX | XXXXXXX |
| XXXX | y | y | XXXXX | XXXXXX | XXXXXXX |
| XXXX | y | y | XXXXX | XXXXXX | XXXXXXX |
| XXXX | y | y | XXXXX | XXXXXX | XXXXXXX |
| XXXX | y | y | XXXXX | XXXXXX | XXXXXXX |
| XXXX | y | y | XXXXX | XXXXXX | XXXXXXX |
| XXXX | y | y | XXXXX | XXXXXX | XXXXXXX |
| XXXX | y | y | XXXXX | XXXXXX | XXXXXXX |
| XXXX | y | y | XXXXX | XXXXXX | XXXXXXX |
| XXXX | y | y | XXXXX | XXXXXX | XXXXXXX |
| XXXX | y | y | XXXXX | XXXXXX | XXXXXXX |
| XXXX | y | y | XXXXX | XXXXXX | XXXXXXX |
| XXXX | y | y | XXXXX | XXXXXX | XXXXXXX |
| XXXX | y | y | XXXXX | XXXXXX | XXXXXXX |
| XXXX | y | y | XXXXX | XXXXXX | XXXXXXX |
| XXXX | y | y | XXXXX | XXXXXX | XXXXXXX |

| | | | | | |
|---|---|---|---|---|---|
| XXXX | y | y | XXXXX | XXXXXX | XXXXXXX |
| XXXX | y | y | XXXXX | XXXXXX | XXXXXXX |
| XXXX | y | y | XXXXX | XXXXXX | XXXXXXX |
| XXXX | y | y | XXXXX | XXXXXX | XXXXXXX |
| XXXX | y | y | XXXXX | XXXXXX | XXXXXXX |
| XXXX | y | y | XXXXX | XXXXXX | XXXXXXX |
| XXXX | y | y | XXXXX | XXXXXX | XXXXXXX |
| XXXX | y | y | XXXXX | XXXXXX | XXXXXXX |
| XXXX | y | y | XXXXX | XXXXXX | XXXXXXX |
| XXXX | y | y | XXXXX | XXXXXX | XXXXXXX |
| XXXX | y | y | XXXXX | XXXXXX | XXXXXXX |
| XXXX | y | y | XXXXX | XXXXXX | XXXXXXX |
| XXXX | y | y | XXXXX | XXXXXX | XXXXXXX |
| XXXX | y | y | XXXXX | XXXXXX | XXXXXXX |
| XXXX | y | y | XXXXX | XXXXXX | XXXXXXX |
| XXXX | y | y | XXXXX | XXXXXX | XXXXXXX |
| XXXX | y | y | XXXXX | XXXXXX | XXXXXXX |
| XXXX | y | y | XXXXX | XXXXXX | XXXXXXX |
| XXXX | y | y | XXXXX | XXXXXX | XXXXXXX |
| XXXX | y | y | XXXXX | XXXXXX | XXXXXXX |
| XXXX | y | y | XXXXX | XXXXXX | XXXXXXX |
| XXXX | y | y | XXXXX | XXXXXX | XXXXXXX |
| XXXX | y | y | XXXXX | XXXXXX | XXXXXXX |
| XXXX | y | y | XXXXX | XXXXXX | XXXXXXX |
| XXXX | y | y | XXXXX | XXXXXX | XXXXXXX |
| XXXX | y | y | XXXXX | XXXXXX | XXXXXXX |
| XXXX | y | y | XXXXX | XXXXXX | XXXXXXX |
| XXXX | y | y | XXXXX | XXXXXX | XXXXXXX |
| XXXX | y | y | XXXXX | XXXXXX | XXXXXXX |
| XXXX | y | y | XXXXX | XXXXXX | XXXXXXX |
| XXXX | y | y | XXXXX | XXXXXX | XXXXXXX |
| XXXX | y | y | XXXXX | XXXXXX | XXXXXXX |
| XXXX | y | y | XXXXX | XXXXXX | XXXXXXX |
| XXXX | y | y | XXXXX | XXXXXX | XXXXXXX |
| XXXX | y | y | XXXXX | XXXXXX | XXXXXXX |
| XXXX | y | y | XXXXX | XXXXXX | XXXXXXX |
| XXXX | y | y | XXXXX | XXXXXX | XXXXXXX |
| XXXX | y | y | XXXXX | XXXXXX | XXXXXXX |
| XXXX | y | y | XXXXX | XXXXXX | XXXXXXX |
| XXXX | y | y | XXXXX | XXXXXX | XXXXXXX |
| XXXX | y | y | XXXXX | XXXXXX | XXXXXXX |
| XXXX | y | y | XXXXX | XXXXXX | XXXXXXX |
| XXXX | y | y | XXXXX | XXXXXX | XXXXXXX |
| XXXX | y | y | XXXXX | XXXXXX | XXXXXXX |
| XXXX | y | y | XXXXX | XXXXXX | XXXXXXX |
| XXXX | y | y | XXXXX | XXXXXX | XXXXXXX |
| XXXX | y | y | XXXXX | XXXXXX | XXXXXXX |
| XXXX | y | y | XXXXX | XXXXXX | XXXXXXX |
| XXXX | y | y | XXXXX | XXXXXX | XXXXXXX |
| XXXX | y | y | XXXXX | XXXXXX | XXXXXXX |

| | | | | | |
|---|---|---|---|---|---|
| XXXX | y | y | XXXXX | XXXXXX | XXXXXXX |
| XXXX | y | y | XXXXX | XXXXXX | XXXXXXX |
| XXXX | y | y | XXXXX | XXXXXX | XXXXXXX |
| XXXX | y | y | XXXXX | XXXXXX | XXXXXXX |
| XXXX | y | y | XXXXX | XXXXXX | XXXXXXX |
| XXXX | y | y | XXXXX | XXXXXX | XXXXXXX |
| XXXX | y | y | XXXXX | XXXXXX | XXXXXXX |
| XXXX | y | y | XXXXX | XXXXXX | XXXXXXX |
| XXXX | y | y | XXXXX | XXXXXX | XXXXXXX |
| XXXX | y | y | XXXXX | XXXXXX | XXXXXXX |
| XXXX | y | y | XXXXX | XXXXXX | XXXXXXX |
| XXXX | y | y | XXXXX | XXXXXX | XXXXXXX |
| XXXX | y | y | XXXXX | XXXXXX | XXXXXXX |
| XXXX | y | y | XXXXX | XXXXXX | XXXXXXX |
| XXXX | y | y | XXXXX | XXXXXX | XXXXXXX |
| XXXX | y | y | XXXXX | XXXXXX | XXXXXXX |
| XXXX | y | y | XXXXX | XXXXXX | XXXXXXX |
| XXXX | y | y | XXXXX | XXXXXX | XXXXXXX |
| XXXX | y | y | XXXXX | XXXXXX | XXXXXXX |
| XXXX | y | y | XXXXX | XXXXXX | XXXXXXX |
| XXXX | y | y | XXXXX | XXXXXX | XXXXXXX |
| XXXX | y | y | XXXXX | XXXXXX | XXXXXXX |
| XXXX | y | y | XXXXX | XXXXXX | XXXXXXX |
| XXXX | y | y | XXXXX | XXXXXX | XXXXXXX |
| XXXX | y | y | XXXXX | XXXXXX | XXXXXXX |
| XXXX | y | y | XXXXX | XXXXXX | XXXXXXX |
| XXXX | y | y | XXXXX | XXXXXX | XXXXXXX |
| XXXX | y | y | XXXXX | XXXXXX | XXXXXXX |
| XXXX | y | y | XXXXX | XXXXXX | XXXXXXX |
| XXXX | y | y | XXXXX | XXXXXX | XXXXXXX |
| XXXX | y | y | XXXXX | XXXXXX | XXXXXXX |
| XXXX | y | y | XXXXX | XXXXXX | XXXXXXX |
| XXXX | y | y | XXXXX | XXXXXX | XXXXXXX |
| XXXX | y | y | XXXXX | XXXXXX | XXXXXXX |
| XXXX | y | y | XXXXX | XXXXXX | XXXXXXX |
| XXXX | y | y | XXXXX | XXXXXX | XXXXXXX |
| XXXX | y | y | XXXXX | XXXXXX | XXXXXXX |
| XXXX | y | y | XXXXX | XXXXXX | XXXXXXX |
| XXXX | y | y | XXXXX | XXXXXX | XXXXXXX |
| XXXX | y | y | XXXXX | XXXXXX | XXXXXXX |
| XXXX | y | y | XXXXX | XXXXXX | XXXXXXX |
| XXXX | y | y | XXXXX | XXXXXX | XXXXXXX |
| XXXX | y | y | XXXXX | XXXXXX | XXXXXXX |
| XXXX | y | y | XXXXX | XXXXXX | XXXXXXX |
| XXXX | y | y | XXXXX | XXXXXX | XXXXXXX |
| XXXX | y | y | XXXXX | XXXXXX | XXXXXXX |
| XXXX | y | y | XXXXX | XXXXXX | XXXXXXX |
| XXXX | y | y | XXXXX | XXXXXX | XXXXXXX |
| XXXX | y | y | XXXXX | XXXXXX | XXXXXXX |
| XXXX | y | y | XXXXX | XXXXXX | XXXXXXX |

| | | | | | |
|---|---|---|---|---|---|
| XXXX | y | y | XXXXX | XXXXXX | XXXXXXX |
| XXXX | y | y | XXXXX | XXXXXX | XXXXXXX |
| XXXX | y | y | XXXXX | XXXXXX | XXXXXXX |
| XXXX | y | y | XXXXX | XXXXXX | XXXXXXX |
| XXXX | y | y | XXXXX | XXXXXX | XXXXXXX |
| XXXX | y | y | XXXXX | XXXXXX | XXXXXXX |
| XXXX | y | y | XXXXX | XXXXXX | XXXXXXX |
| XXXX | y | y | XXXXX | XXXXXX | XXXXXXX |
| XXXX | y | y | XXXXX | XXXXXX | XXXXXXX |
| XXXX | y | y | XXXXX | XXXXXX | XXXXXXX |
| XXXX | y | y | XXXXX | XXXXXX | XXXXXXX |
| XXXX | y | y | XXXXX | XXXXXX | XXXXXXX |
| XXXX | y | y | XXXXX | XXXXXX | XXXXXXX |
| XXXX | y | y | XXXXX | XXXXXX | XXXXXXX |
| XXXX | y | y | XXXXX | XXXXXX | XXXXXXX |
| XXXX | y | y | XXXXX | XXXXXX | XXXXXXX |
| XXXX | y | y | XXXXX | XXXXXX | XXXXXXX |
| XXXX | y | y | XXXXX | XXXXXX | XXXXXXX |
| XXXX | y | n | XXXXX | XXXXXX | XXXXXXX |
| XXXX | y | n | XXXXX | XXXXXX | XXXXXXX |
| XXXX | y | n | XXXXX | XXXXXX | XXXXXXX |
| XXXX | y | n | XXXXX | XXXXXX | XXXXXXX |
| XXXX | y | n | XXXXX | XXXXXX | XXXXXXX |
| XXXX | y | n | XXXXX | XXXXXX | XXXXXXX |
| XXXX | y | n | XXXXX | XXXXXX | XXXXXXX |
| XXXX | y | n | XXXXX | XXXXXX | XXXXXXX |
| XXXX | y | n | XXXXX | XXXXXX | XXXXXXX |
| XXXX | y | n | XXXXX | XXXXXX | XXXXXXX |
| XXXX | y | n | XXXXX | XXXXXX | XXXXXXX |
| XXXX | y | n | XXXXX | XXXXXX | XXXXXXX |
| XXXX | y | n | XXXXX | XXXXXX | XXXXXXX |
| XXXX | y | n | XXXXX | XXXXXX | XXXXXXX |
| XXXX | y | n | XXXXX | XXXXXX | XXXXXXX |
| XXXX | y | n | XXXXX | XXXXXX | XXXXXXX |
| XXXX | y | n | XXXXX | XXXXXX | XXXXXXX |
| XXXX | y | n | XXXXX | XXXXXX | XXXXXXX |
| XXXX | y | n | XXXXX | XXXXXX | XXXXXXX |
| XXXX | y | n | XXXXX | XXXXXX | XXXXXXX |
| XXXX | y | n | XXXXX | XXXXXX | XXXXXXX |
| XXXX | y | n | XXXXX | XXXXXX | XXXXXXX |
| XXXX | y | n | XXXXX | XXXXXX | XXXXXXX |
| XXXX | y | n | XXXXX | XXXXXX | XXXXXXX |
| XXXX | y | n | XXXXX | XXXXXX | XXXXXXX |
| XXXX | y | n | XXXXX | XXXXXX | XXXXXXX |
| XXXX | y | y | XXXXX | XXXXXX | XXXXXXX |
| XXXX | y | y | XXXXX | XXXXXX | XXXXXXX |
| XXXX | y | y | XXXXX | XXXXXX | XXXXXXX |
| XXXX | y | y | XXXXX | XXXXXX | XXXXXXX |
| XXXX | y | y | XXXXX | XXXXXX | XXXXXXX |
| XXXX | y | y | XXXXX | XXXXXX | XXXXXXX |

| | | | | | |
|---|---|---|---|---|---|
| XXXX | y | y | XXXXX | XXXXXX | XXXXXXX |
| XXXX | y | y | XXXXX | XXXXXX | XXXXXXX |
| XXXX | y | y | XXXXX | XXXXXX | XXXXXXX |
| XXXX | y | y | XXXXX | XXXXXX | XXXXXXX |
| XXXX | y | y | XXXXX | XXXXXX | XXXXXXX |
| XXXX | y | y | XXXXX | XXXXXX | XXXXXXX |
| XXXX | y | y | XXXXX | XXXXXX | XXXXXXX |
| XXXX | y | n | XXXXX | XXXXXX | XXXXXXX |
| XXXX | y | n | XXXXX | XXXXXX | XXXXXXX |
| XXXX | y | n | XXXXX | XXXXXX | XXXXXXX |
| XXXX | y | n | XXXXX | XXXXXX | XXXXXXX |
| XXXX | y | n | XXXXX | XXXXXX | XXXXXXX |
| XXXX | y | n | XXXXX | XXXXXX | XXXXXXX |
| XXXX | y | n | XXXXX | XXXXXX | XXXXXXX |
| XXXX | y | n | XXXXX | XXXXXX | XXXXXXX |
| XXXX | y | n | XXXXX | XXXXXX | XXXXXXX |
| XXXX | y | n | XXXXX | XXXXXX | XXXXXXX |
| XXXX | y | n | XXXXX | XXXXXX | XXXXXXX |
| XXXX | y | n | XXXXX | XXXXXX | XXXXXXX |
| XXXX | y | n | XXXXX | XXXXXX | XXXXXXX |
| XXXX | y | n | XXXXX | XXXXXX | XXXXXXX |
| XXXX | y | n | XXXXX | XXXXXX | XXXXXXX |
| XXXX | y | n | XXXXX | XXXXXX | XXXXXXX |
| XXXX | y | n | XXXXX | XXXXXX | XXXXXXX |
| XXXX | y | n | XXXXX | XXXXXX | XXXXXXX |
| XXXX | y | n | XXXXX | XXXXXX | XXXXXXX |
| XXXX | y | n | XXXXX | XXXXXX | XXXXXXX |
| XXXX | y | n | XXXXX | XXXXXX | XXXXXXX |
| XXXX | y | n | XXXXX | XXXXXX | XXXXXXX |
| XXXX | y | n | XXXXX | XXXXXX | XXXXXXX |
| XXXX | y | n | XXXXX | XXXXXX | XXXXXXX |
| XXXX | y | n | XXXXX | XXXXXX | XXXXXXX |
| XXXX | y | n | XXXXX | XXXXXX | XXXXXXX |
| XXXX | y | n | XXXXX | XXXXXX | XXXXXXX |
| XXXX | y | n | XXXXX | XXXXXX | XXXXXXX |
| XXXX | y | n | XXXXX | XXXXXX | XXXXXXX |
| XXXX | y | n | XXXXX | XXXXXX | XXXXXXX |
| XXXX | y | n | XXXXX | XXXXXX | XXXXXXX |
| XXXX | y | n | XXXXX | XXXXXX | XXXXXXX |
| XXXX | y | n | XXXXX | XXXXXX | XXXXXXX |
| XXXX | y | n | XXXXX | XXXXXX | XXXXXXX |
| XXXX | y | n | XXXXX | XXXXXX | XXXXXXX |
| XXXX | y | n | XXXXX | XXXXXX | XXXXXXX |
| XXXX | y | n | XXXXX | XXXXXX | XXXXXXX |
| XXXX | y | n | XXXXX | XXXXXX | XXXXXXX |
| XXXX | y | n | XXXXX | XXXXXX | XXXXXXX |
| XXXX | y | n | XXXXX | XXXXXX | XXXXXXX |
| XXXX | y | n | XXXXX | XXXXXX | XXXXXXX |
| XXXX | y | n | XXXXX | XXXXXX | XXXXXXX |
| XXXX | y | n | XXXXX | XXXXXX | XXXXXXX |

| | | | | | |
|---|---|---|---|---|---|
| XXXX | y | n | XXXXX | XXXXXX | XXXXXXX |
| XXXX | y | n | XXXXX | XXXXXX | XXXXXXX |
| XXXX | y | n | XXXXX | XXXXXX | XXXXXXX |
| XXXX | y | n | XXXXX | XXXXXX | XXXXXXX |
| XXXX | y | n | XXXXX | XXXXXX | XXXXXXX |
| XXXX | y | n | XXXXX | XXXXXX | XXXXXXX |
| XXXX | y | n | XXXXX | XXXXXX | XXXXXXX |
| XXXX | y | n | XXXXX | XXXXXX | XXXXXXX |
| XXXX | y | n | XXXXX | XXXXXX | XXXXXXX |
| XXXX | y | n | XXXXX | XXXXXX | XXXXXXX |
| XXXX | y | n | XXXXX | XXXXXX | XXXXXXX |
| XXXX | y | n | XXXXX | XXXXXX | XXXXXXX |
| XXXX | y | n | XXXXX | XXXXXX | XXXXXXX |
| XXXX | y | n | XXXXX | XXXXXX | XXXXXXX |
| XXXX | y | y | XXXXX | XXXXXX | XXXXXXX |
| XXXX | y | y | XXXXX | XXXXXX | XXXXXXX |
| XXXX | y | y | XXXXX | XXXXXX | XXXXXXX |
| XXXX | y | y | XXXXX | XXXXXX | XXXXXXX |
| XXXX | y | y | XXXXX | XXXXXX | XXXXXXX |
| XXXX | y | y | XXXXX | XXXXXX | XXXXXXX |
| XXXX | y | y | XXXXX | XXXXXX | XXXXXXX |
| XXXX | y | y | XXXXX | XXXXXX | XXXXXXX |
| XXXX | y | y | XXXXX | XXXXXX | XXXXXXX |
| XXXX | y | y | XXXXX | XXXXXX | XXXXXXX |
| XXXX | y | y | XXXXX | XXXXXX | XXXXXXX |
| XXXX | y | y | XXXXX | XXXXXX | XXXXXXX |
| XXXX | y | n | XXXXX | XXXXXX | XXXXXXX |
| XXXX | y | y | XXXXX | XXXXXX | XXXXXXX |
| XXXX | y | y | XXXXX | XXXXXX | XXXXXXX |
| XXXX | y | y | XXXXX | XXXXXX | XXXXXXX |
| XXXX | y | y | XXXXX | XXXXXX | XXXXXXX |
| XXXX | y | y | XXXXX | XXXXXX | XXXXXXX |
| XXXX | y | y | XXXXX | XXXXXX | XXXXXXX |
| XXXX | y | y | XXXXX | XXXXXX | XXXXXXX |
| XXXX | y | y | XXXXX | XXXXXX | XXXXXXX |
| XXXX | y | y | XXXXX | XXXXXX | XXXXXXX |
| XXXX | y | y | XXXXX | XXXXXX | XXXXXXX |
| XXXX | y | y | XXXXX | XXXXXX | XXXXXXX |
| XXXX | y | y | XXXXX | XXXXXX | XXXXXXX |
| XXXX | y | n | XXXXX | XXXXXX | XXXXXXX |
| XXXX | y | y | XXXXX | XXXXXX | XXXXXXX |
| XXXX | y | y | XXXXX | XXXXXX | XXXXXXX |
| XXXX | y | y | XXXXX | XXXXXX | XXXXXXX |
| XXXX | y | y | XXXXX | XXXXXX | XXXXXXX |
| XXXX | y | y | XXXXX | XXXXXX | XXXXXXX |
| XXXX | y | y | XXXXX | XXXXXX | XXXXXXX |
| XXXX | y | y | XXXXX | XXXXXX | XXXXXXX |
| XXXX | y | n | XXXXX | XXXXXX | XXXXXXX |
| XXXX | y | y | XXXXX | XXXXXX | XXXXXXX |
| XXXX | y | y | XXXXX | XXXXXX | XXXXXXX |

| | | | | | |
|---|---|---|---|---|---|
| XXXX | y | y | XXXXX | XXXXXX | XXXXXXX |
| XXXX | y | y | XXXXX | XXXXXX | XXXXXXX |
| XXXX | y | y | XXXXX | XXXXXX | XXXXXXX |
| XXXX | y | y | XXXXX | XXXXXX | XXXXXXX |
| XXXX | y | y | XXXXX | XXXXXX | XXXXXXX |
| XXXX | y | y | XXXXX | XXXXXX | XXXXXXX |
| XXXX | y | y | XXXXX | XXXXXX | XXXXXXX |
| XXXX | y | y | XXXXX | XXXXXX | XXXXXXX |
| XXXX | y | y | XXXXX | XXXXXX | XXXXXXX |
| XXXX | y | y | XXXXX | XXXXXX | XXXXXXX |
| XXXX | y | y | XXXXX | XXXXXX | XXXXXXX |
| XXXX | y | y | XXXXX | XXXXXX | XXXXXXX |
| XXXX | y | y | XXXXX | XXXXXX | XXXXXXX |
| XXXX | y | y | XXXXX | XXXXXX | XXXXXXX |
| XXXX | y | y | XXXXX | XXXXXX | XXXXXXX |
| XXXX | y | y | XXXXX | XXXXXX | XXXXXXX |
| XXXX | y | y | XXXXX | XXXXXX | XXXXXXX |
| XXXX | y | y | XXXXX | XXXXXX | XXXXXXX |
| XXXX | y | y | XXXXX | XXXXXX | XXXXXXX |
| XXXX | y | y | XXXXX | XXXXXX | XXXXXXX |
| XXXX | y | y | XXXXX | XXXXXX | XXXXXXX |
| XXXX | y | y | XXXXX | XXXXXX | XXXXXXX |
| XXXX | y | y | XXXXX | XXXXXX | XXXXXXX |
| XXXX | y | y | XXXXX | XXXXXX | XXXXXXX |
| XXXX | y | y | XXXXX | XXXXXX | XXXXXXX |
| XXXX | y | y | XXXXX | XXXXXX | XXXXXXX |
| XXXX | y | y | XXXXX | XXXXXX | XXXXXXX |
| XXXX | y | y | XXXXX | XXXXXX | XXXXXXX |
| XXXX | y | y | XXXXX | XXXXXX | XXXXXXX |
| XXXX | y | n | XXXXX | XXXXXX | XXXXXXX |
| XXXX | y | y | XXXXX | XXXXXX | XXXXXXX |
| XXXX | y | y | XXXXX | XXXXXX | XXXXXXX |
| XXXX | y | y | XXXXX | XXXXXX | XXXXXXX |
| XXXX | y | y | XXXXX | XXXXXX | XXXXXXX |
| XXXX | y | y | XXXXX | XXXXXX | XXXXXXX |
| XXXX | y | y | XXXXX | XXXXXX | XXXXXXX |
| XXXX | y | y | XXXXX | XXXXXX | XXXXXXX |
| XXXX | y | y | XXXXX | XXXXXX | XXXXXXX |
| XXXX | y | y | XXXXX | XXXXXX | XXXXXXX |
| XXXX | y | y | XXXXX | XXXXXX | XXXXXXX |
| XXXX | y | y | XXXXX | XXXXXX | XXXXXXX |
| XXXX | y | n | XXXXX | XXXXXX | XXXXXXX |
| XXXX | y | y | XXXXX | XXXXXX | XXXXXXX |
| XXXX | y | y | XXXXX | XXXXXX | XXXXXXX |
| XXXX | y | y | XXXXX | XXXXXX | XXXXXXX |
| XXXX | y | n | XXXXX | XXXXXX | XXXXXXX |
| XXXX | y | n | XXXXX | XXXXXX | XXXXXXX |
| XXXX | y | y | XXXXX | XXXXXX | XXXXXXX |
| XXXX | y | y | XXXXX | XXXXXX | XXXXXXX |
| XXXX | y | y | XXXXX | XXXXXX | XXXXXXX |

| | | | | | |
|---|---|---|---|---|---|
| XXXX | y | y | XXXXX | XXXXXX | XXXXXXX |
| XXXX | y | y | XXXXX | XXXXXX | XXXXXXX |
| XXXX | y | y | XXXXX | XXXXXX | XXXXXXX |
| XXXX | y | n | XXXXX | XXXXXX | XXXXXXX |
| XXXX | y | y | XXXXX | XXXXXX | XXXXXXX |
| XXXX | y | n | XXXXX | XXXXXX | XXXXXXX |
| XXXX | y | y | XXXXX | XXXXXX | XXXXXXX |
| XXXX | y | n | XXXXX | XXXXXX | XXXXXXX |
| XXXX | y | y | XXXXX | XXXXXX | XXXXXXX |
| XXXX | y | y | XXXXX | XXXXXX | XXXXXXX |
| XXXX | y | y | XXXXX | XXXXXX | XXXXXXX |
| XXXX | y | y | XXXXX | XXXXXX | XXXXXXX |
| XXXX | y | y | XXXXX | XXXXXX | XXXXXXX |
| XXXX | y | y | XXXXX | XXXXXX | XXXXXXX |
| XXXX | y | y | XXXXX | XXXXXX | XXXXXXX |
| XXXX | y | n | XXXXX | XXXXXX | XXXXXXX |
| XXXX | y | y | XXXXX | XXXXXX | XXXXXXX |
| XXXX | y | y | XXXXX | XXXXXX | XXXXXXX |
| XXXX | y | n | XXXXX | XXXXXX | XXXXXXX |
| XXXX | y | n | XXXXX | XXXXXX | XXXXXXX |
| XXXX | y | n | XXXXX | XXXXXX | XXXXXXX |
| XXXX | y | y | XXXXX | XXXXXX | XXXXXXX |
| XXXX | y | y | XXXXX | XXXXXX | XXXXXXX |
| XXXX | y | y | XXXXX | XXXXXX | XXXXXXX |
| XXXX | y | y | XXXXX | XXXXXX | XXXXXXX |
| XXXX | y | y | XXXXX | XXXXXX | XXXXXXX |
| XXXX | y | y | XXXXX | XXXXXX | XXXXXXX |
| XXXX | y | y | XXXXX | XXXXXX | XXXXXXX |
| XXXX | y | y | XXXXX | XXXXXX | XXXXXXX |
| XXXX | y | y | XXXXX | XXXXXX | XXXXXXX |
| XXXX | y | n | XXXXX | XXXXXX | XXXXXXX |
| XXXX | y | y | XXXXX | XXXXXX | XXXXXXX |
| XXXX | y | y | XXXXX | XXXXXX | XXXXXXX |
| XXXX | y | y | XXXXX | XXXXXX | XXXXXXX |
| XXXX | y | y | XXXXX | XXXXXX | XXXXXXX |
| XXXX | y | n | XXXXX | XXXXXX | XXXXXXX |
| XXXX | y | y | XXXXX | XXXXXX | XXXXXXX |
| XXXX | y | y | XXXXX | XXXXXX | XXXXXXX |
| XXXX | y | n | XXXXX | XXXXXX | XXXXXXX |
| XXXX | y | y | XXXXX | XXXXXX | XXXXXXX |
| XXXX | y | y | XXXXX | XXXXXX | XXXXXXX |
| XXXX | y | y | XXXXX | XXXXXX | XXXXXXX |
| XXXX | y | n | XXXXX | XXXXXX | XXXXXXX |
| XXXX | y | y | XXXXX | XXXXXX | XXXXXXX |
| XXXX | y | n | XXXXX | XXXXXX | XXXXXXX |
| XXXX | y | y | XXXXX | XXXXXX | XXXXXXX |
| XXXX | y | y | XXXXX | XXXXXX | XXXXXXX |
| XXXX | y | y | XXXXX | XXXXXX | XXXXXXX |
| XXXX | y | y | XXXXX | XXXXXX | XXXXXXX |
| XXXX | y | y | XXXXX | XXXXXX | XXXXXXX |

| | | | | | |
|---|---|---|---|---|---|
| XXXX | y | y | XXXXX | XXXXXX | XXXXXXX |
| XXXX | y | n | XXXXX | XXXXXX | XXXXXXX |
| XXXX | y | y | XXXXX | XXXXXX | XXXXXXX |
| XXXX | y | n | XXXXX | XXXXXX | XXXXXXX |
| XXXX | y | n | XXXXX | XXXXXX | XXXXXXX |
| XXXX | y | y | XXXXX | XXXXXX | XXXXXXX |
| XXXX | y | y | XXXXX | XXXXXX | XXXXXXX |
| XXXX | y | y | XXXXX | XXXXXX | XXXXXXX |
| XXXX | y | y | XXXXX | XXXXXX | XXXXXXX |
| XXXX | y | n | XXXXX | XXXXXX | XXXXXXX |
| XXXX | y | n | XXXXX | XXXXXX | XXXXXXX |
| XXXX | y | n | XXXXX | XXXXXX | XXXXXXX |
| XXXX | y | n | XXXXX | XXXXXX | XXXXXXX |
| XXXX | y | n | XXXXX | XXXXXX | XXXXXXX |
| XXXX | y | n | XXXXX | XXXXXX | XXXXXXX |
| XXXX | y | n | XXXXX | XXXXXX | XXXXXXX |
| XXXX | y | n | XXXXX | XXXXXX | XXXXXXX |
| XXXX | y | n | XXXXX | XXXXXX | XXXXXXX |
| XXXX | y | n | XXXXX | XXXXXX | XXXXXXX |
| XXXX | y | n | XXXXX | XXXXXX | XXXXXXX |
| XXXX | y | n | XXXXX | XXXXXX | XXXXXXX |
| XXXX | y | n | XXXXX | XXXXXX | XXXXXXX |
| XXXX | y | y | XXXXX | XXXXXX | XXXXXXX |
| XXXX | y | y | XXXXX | XXXXXX | XXXXXXX |
| XXXX | y | y | XXXXX | XXXXXX | XXXXXXX |
| XXXX | y | y | XXXXX | XXXXXX | XXXXXXX |
| XXXX | y | y | XXXXX | XXXXXX | XXXXXXX |
| XXXX | y | y | XXXXX | XXXXXX | XXXXXXX |
| XXXX | y | y | XXXXX | XXXXXX | XXXXXXX |
| XXXX | y | y | XXXXX | XXXXXX | XXXXXXX |
| XXXX | y | n | XXXXX | XXXXXX | XXXXXXX |
| XXXX | y | n | XXXXX | XXXXXX | XXXXXXX |
| XXXX | y | n | XXXXX | XXXXXX | XXXXXXX |
| XXXX | y | y | XXXXX | XXXXXX | XXXXXXX |
| XXXX | y | y | XXXXX | XXXXXX | XXXXXXX |
| XXXX | y | y | XXXXX | XXXXXX | XXXXXXX |
| XXXX | y | y | XXXXX | XXXXXX | XXXXXXX |
| XXXX | y | y | XXXXX | XXXXXX | XXXXXXX |
| XXXX | y | y | XXXXX | XXXXXX | XXXXXXX |
| XXXX | y | n | XXXXX | XXXXXX | XXXXXXX |
| XXXX | y | n | XXXXX | XXXXXX | XXXXXXX |
| XXXX | y | n | XXXXX | XXXXXX | XXXXXXX |
| XXXX | y | n | XXXXX | XXXXXX | XXXXXXX |
| XXXX | y | n | XXXXX | XXXXXX | XXXXXXX |
| XXXX | y | n | XXXXX | XXXXXX | XXXXXXX |
| XXXX | y | y | XXXXX | XXXXXX | XXXXXXX |
| XXXX | y | y | XXXXX | XXXXXX | XXXXXXX |
| XXXX | y | y | XXXXX | XXXXXX | XXXXXXX |
| XXXX | y | y | XXXXX | XXXXXX | XXXXXXX |
| XXXX | y | y | XXXXX | XXXXXX | XXXXXXX |

| | | | | | |
|---|---|---|---|---|---|
| XXXX | y | y | XXXXX | XXXXXX | XXXXXXX |
| XXXX | y | y | XXXXX | XXXXXX | XXXXXXX |
| XXXX | y | y | XXXXX | XXXXXX | XXXXXXX |
| XXXX | y | y | XXXXX | XXXXXX | XXXXXXX |
| XXXX | y | y | XXXXX | XXXXXX | XXXXXXX |
| XXXX | y | y | XXXXX | XXXXXX | XXXXXXX |
| XXXX | y | y | XXXXX | XXXXXX | XXXXXXX |
| XXXX | y | y | XXXXX | XXXXXX | XXXXXXX |
| XXXX | y | y | XXXXX | XXXXXX | XXXXXXX |
| XXXX | y | y | XXXXX | XXXXXX | XXXXXXX |
| XXXX | y | y | XXXXX | XXXXXX | XXXXXXX |
| XXXX | y | y | XXXXX | XXXXXX | XXXXXXX |
| XXXX | y | y | XXXXX | XXXXXX | XXXXXXX |
| XXXX | y | y | XXXXX | XXXXXX | XXXXXXX |
| XXXX | y | y | XXXXX | XXXXXX | XXXXXXX |
| XXXX | y | y | XXXXX | XXXXXX | XXXXXXX |
| XXXX | y | y | XXXXX | XXXXXX | XXXXXXX |
| XXXX | y | y | XXXXX | XXXXXX | XXXXXXX |
| XXXX | y | y | XXXXX | XXXXXX | XXXXXXX |
| XXXX | y | y | XXXXX | XXXXXX | XXXXXXX |
| XXXX | y | y | XXXXX | XXXXXX | XXXXXXX |
| XXXX | y | y | XXXXX | XXXXXX | XXXXXXX |
| XXXX | y | y | XXXXX | XXXXXX | XXXXXXX |
| XXXX | y | y | XXXXX | XXXXXX | XXXXXXX |
| XXXX | y | y | XXXXX | XXXXXX | XXXXXXX |
| XXXX | y | y | XXXXX | XXXXXX | XXXXXXX |
| XXXX | y | y | XXXXX | XXXXXX | XXXXXXX |
| XXXX | na | n | XXXXX | XXXXXX | XXXXXXX |
| XXXX | y | y | XXXXX | XXXXXX | XXXXXXX |
| XXXX | y | y | XXXXX | XXXXXX | XXXXXXX |
| XXXX | y | y | XXXXX | XXXXXX | XXXXXXX |
| XXXX | y | y | XXXXX | XXXXXX | XXXXXXX |
| XXXX | y | y | XXXXX | XXXXXX | XXXXXXX |
| XXXX | y | y | XXXXX | XXXXXX | XXXXXXX |
| XXXX | y | y | XXXXX | XXXXXX | XXXXXXX |
| XXXX | y | y | XXXXX | XXXXXX | XXXXXXX |
| XXXX | y | y | XXXXX | XXXXXX | XXXXXXX |
| XXXX | y | y | XXXXX | XXXXXX | XXXXXXX |
| XXXX | y | y | XXXXX | XXXXXX | XXXXXXX |
| XXXX | y | y | XXXXX | XXXXXX | XXXXXXX |
| XXXX | y | y | XXXXX | XXXXXX | XXXXXXX |
| XXXX | y | y | XXXXX | XXXXXX | XXXXXXX |
| XXXX | y | y | XXXXX | XXXXXX | XXXXXXX |
| XXXX | y | y | XXXXX | XXXXXX | XXXXXXX |
| XXXX | y | y | XXXXX | XXXXXX | XXXXXXX |
| XXXX | y | y | XXXXX | XXXXXX | XXXXXXX |
| XXXX | y | y | XXXXX | XXXXXX | XXXXXXX |
| XXXX | y | y | XXXXX | XXXXXX | XXXXXXX |
| XXXX | y | y | XXXXX | XXXXXX | XXXXXXX |
| XXXX | y | y | XXXXX | XXXXXX | XXXXXXX |

| | | | | | |
|---|---|---|---|---|---|
| XXXX | y | y | XXXXX | XXXXXX | XXXXXXX |
| XXXX | y | y | XXXXX | XXXXXX | XXXXXXX |
| XXXX | y | y | XXXXX | XXXXXX | XXXXXXX |
| XXXX | y | y | XXXXX | XXXXXX | XXXXXXX |
| XXXX | y | y | XXXXX | XXXXXX | XXXXXXX |
| XXXX | y | y | XXXXX | XXXXXX | XXXXXXX |
| XXXX | y | y | XXXXX | XXXXXX | XXXXXXX |
| XXXX | y | y | XXXXX | XXXXXX | XXXXXXX |
| XXXX | y | y | XXXXX | XXXXXX | XXXXXXX |
| XXXX | y | y | XXXXX | XXXXXX | XXXXXXX |
| XXXX | y | y | XXXXX | XXXXXX | XXXXXXX |
| XXXX | y | y | XXXXX | XXXXXX | XXXXXXX |
| XXXX | y | y | XXXXX | XXXXXX | XXXXXXX |
| XXXX | y | y | XXXXX | XXXXXX | XXXXXXX |
| XXXX | y | y | XXXXX | XXXXXX | XXXXXXX |
| XXXX | y | y | XXXXX | XXXXXX | XXXXXXX |
| XXXX | y | y | XXXXX | XXXXXX | XXXXXXX |
| XXXX | y | y | XXXXX | XXXXXX | XXXXXXX |
| XXXX | y | y | XXXXX | XXXXXX | XXXXXXX |
| XXXX | y | y | XXXXX | XXXXXX | XXXXXXX |
| XXXX | y | y | XXXXX | XXXXXX | XXXXXXX |
| XXXX | y | y | XXXXX | XXXXXX | XXXXXXX |
| XXXX | y | y | XXXXX | XXXXXX | XXXXXXX |
| XXXX | y | y | XXXXX | XXXXXX | XXXXXXX |
| XXXX | y | y | XXXXX | XXXXXX | XXXXXXX |
| XXXX | y | y | XXXXX | XXXXXX | XXXXXXX |
| XXXX | y | y | XXXXX | XXXXXX | XXXXXXX |
| XXXX | y | y | XXXXX | XXXXXX | XXXXXXX |
| XXXX | y | y | XXXXX | XXXXXX | XXXXXXX |
| XXXX | y | y | XXXXX | XXXXXX | XXXXXXX |
| XXXX | y | y | XXXXX | XXXXXX | XXXXXXX |
| XXXX | y | y | XXXXX | XXXXXX | XXXXXXX |
| XXXX | y | y | XXXXX | XXXXXX | XXXXXXX |
| XXXX | y | y | XXXXX | XXXXXX | XXXXXXX |
| XXXX | y | y | XXXXX | XXXXXX | XXXXXXX |
| XXXX | y | y | XXXXX | XXXXXX | XXXXXXX |
| XXXX | y | y | XXXXX | XXXXXX | XXXXXXX |
| XXXX | y | y | XXXXX | XXXXXX | XXXXXXX |
| XXXX | y | y | XXXXX | XXXXXX | XXXXXXX |
| XXXX | y | y | XXXXX | XXXXXX | XXXXXXX |
| XXXX | y | y | XXXXX | XXXXXX | XXXXXXX |
| XXXX | y | y | XXXXX | XXXXXX | XXXXXXX |
| XXXX | y | y | XXXXX | XXXXXX | XXXXXXX |
| XXXX | y | y | XXXXX | XXXXXX | XXXXXXX |
| XXXX | y | y | XXXXX | XXXXXX | XXXXXXX |
| XXXX | y | y | XXXXX | XXXXXX | XXXXXXX |
| XXXX | y | y | XXXXX | XXXXXX | XXXXXXX |
| XXXX | y | y | XXXXX | XXXXXX | XXXXXXX |
| XXXX | y | y | XXXXX | XXXXXX | XXXXXXX |
| XXXX | y | y | XXXXX | XXXXXX | XXXXXXX |

| | | | | | |
|---|---|---|---|---|---|
| XXXX | y | y | XXXXX | XXXXXX | XXXXXXX |
| XXXX | y | y | XXXXX | XXXXXX | XXXXXXX |
| XXXX | y | y | XXXXX | XXXXXX | XXXXXXX |
| XXXX | y | y | XXXXX | XXXXXX | XXXXXXX |
| XXXX | y | y | XXXXX | XXXXXX | XXXXXXX |
| XXXX | y | y | XXXXX | XXXXXX | XXXXXXX |
| XXXX | y | y | XXXXX | XXXXXX | XXXXXXX |
| XXXX | y | y | XXXXX | XXXXXX | XXXXXXX |
| XXXX | y | y | XXXXX | XXXXXX | XXXXXXX |
| XXXX | y | y | XXXXX | XXXXXX | XXXXXXX |
| XXXX | y | y | XXXXX | XXXXXX | XXXXXXX |
| XXXX | y | y | XXXXX | XXXXXX | XXXXXXX |
| XXXX | y | y | XXXXX | XXXXXX | XXXXXXX |
| XXXX | y | y | XXXXX | XXXXXX | XXXXXXX |
| XXXX | y | y | XXXXX | XXXXXX | XXXXXXX |
| XXXX | y | y | XXXXX | XXXXXX | XXXXXXX |
| XXXX | y | y | XXXXX | XXXXXX | XXXXXXX |
| XXXX | y | y | XXXXX | XXXXXX | XXXXXXX |
| XXXX | y | y | XXXXX | XXXXXX | XXXXXXX |
| XXXX | y | n | XXXXX | XXXXXX | XXXXXXX |
| XXXX | y | n | XXXXX | XXXXXX | XXXXXXX |
| XXXX | y | n | XXXXX | XXXXXX | XXXXXXX |
| XXXX | y | n | XXXXX | XXXXXX | XXXXXXX |
| XXXX | y | n | XXXXX | XXXXXX | XXXXXXX |
| XXXX | y | n | XXXXX | XXXXXX | XXXXXXX |
| XXXX | y | n | XXXXX | XXXXXX | XXXXXXX |
| XXXX | y | n | XXXXX | XXXXXX | XXXXXXX |
| XXXX | y | n | XXXXX | XXXXXX | XXXXXXX |
| XXXX | y | n | XXXXX | XXXXXX | XXXXXXX |
| XXXX | y | n | XXXXX | XXXXXX | XXXXXXX |
| XXXX | y | n | XXXXX | XXXXXX | XXXXXXX |
| XXXX | y | n | XXXXX | XXXXXX | XXXXXXX |
| XXXX | y | n | XXXXX | XXXXXX | XXXXXXX |
| XXXX | y | n | XXXXX | XXXXXX | XXXXXXX |
| XXXX | y | n | XXXXX | XXXXXX | XXXXXXX |
| XXXX | y | n | XXXXX | XXXXXX | XXXXXXX |
| XXXX | y | n | XXXXX | XXXXXX | XXXXXXX |
| XXXX | y | n | XXXXX | XXXXXX | XXXXXXX |
| XXXX | y | n | XXXXX | XXXXXX | XXXXXXX |
| XXXX | y | n | XXXXX | XXXXXX | XXXXXXX |
| XXXX | y | n | XXXXX | XXXXXX | XXXXXXX |
| XXXX | y | n | XXXXX | XXXXXX | XXXXXXX |
| XXXX | y | n | XXXXX | XXXXXX | XXXXXXX |
| XXXX | y | n | XXXXX | XXXXXX | XXXXXXX |
| XXXX | y | n | XXXXX | XXXXXX | XXXXXXX |
| XXXX | y | n | XXXXX | XXXXXX | XXXXXXX |
| XXXX | y | n | XXXXX | XXXXXX | XXXXXXX |
| XXXX | y | n | XXXXX | XXXXXX | XXXXXXX |
| XXXX | y | n | XXXXX | XXXXXX | XXXXXXX |
| XXXX | y | n | XXXXX | XXXXXX | XXXXXXX |

| | | | | | |
|---|---|---|---|---|---|
| XXXX | y | n | XXXXX | XXXXXX | XXXXXXX |
| XXXX | y | n | XXXXX | XXXXXX | XXXXXXX |
| XXXX | y | n | XXXXX | XXXXXX | XXXXXXX |
| XXXX | y | n | XXXXX | XXXXXX | XXXXXXX |
| XXXX | y | n | XXXXX | XXXXXX | XXXXXXX |
| XXXX | y | n | XXXXX | XXXXXX | XXXXXXX |
| XXXX | y | n | XXXXX | XXXXXX | XXXXXXX |
| XXXX | y | n | XXXXX | XXXXXX | XXXXXXX |
| XXXX | y | n | XXXXX | XXXXXX | XXXXXXX |
| XXXX | y | n | XXXXX | XXXXXX | XXXXXXX |
| XXXX | y | n | XXXXX | XXXXXX | XXXXXXX |
| XXXX | y | n | XXXXX | XXXXXX | XXXXXXX |
| XXXX | y | n | XXXXX | XXXXXX | XXXXXXX |
| XXXX | y | n | XXXXX | XXXXXX | XXXXXXX |
| XXXX | y | n | XXXXX | XXXXXX | XXXXXXX |
| XXXX | y | n | XXXXX | XXXXXX | XXXXXXX |
| XXXX | y | n | XXXXX | XXXXXX | XXXXXXX |
| XXXX | y | n | XXXXX | XXXXXX | XXXXXXX |
| XXXX | y | n | XXXXX | XXXXXX | XXXXXXX |
| XXXX | y | n | XXXXX | XXXXXX | XXXXXXX |
| XXXX | y | n | XXXXX | XXXXXX | XXXXXXX |
| XXXX | y | n | XXXXX | XXXXXX | XXXXXXX |
| XXXX | y | n | XXXXX | XXXXXX | XXXXXXX |
| XXXX | y | n | XXXXX | XXXXXX | XXXXXXX |
| XXXX | y | n | XXXXX | XXXXXX | XXXXXXX |
| XXXX | y | n | XXXXX | XXXXXX | XXXXXXX |
| XXXX | y | n | XXXXX | XXXXXX | XXXXXXX |
| XXXX | y | n | XXXXX | XXXXXX | XXXXXXX |
| XXXX | y | n | XXXXX | XXXXXX | XXXXXXX |
| XXXX | y | n | XXXXX | XXXXXX | XXXXXXX |
| XXXX | y | n | XXXXX | XXXXXX | XXXXXXX |
| XXXX | y | n | XXXXX | XXXXXX | XXXXXXX |
| XXXX | y | n | XXXXX | XXXXXX | XXXXXXX |
| XXXX | y | n | XXXXX | XXXXXX | XXXXXXX |
| XXXX | y | n | XXXXX | XXXXXX | XXXXXXX |
| XXXX | y | n | XXXXX | XXXXXX | XXXXXXX |
| XXXX | y | n | XXXXX | XXXXXX | XXXXXXX |
| XXXX | y | n | XXXXX | XXXXXX | XXXXXXX |
| XXXX | y | n | XXXXX | XXXXXX | XXXXXXX |
| XXXX | y | n | XXXXX | XXXXXX | XXXXXXX |
| XXXX | y | n | XXXXX | XXXXXX | XXXXXXX |
| XXXX | y | n | XXXXX | XXXXXX | XXXXXXX |
| XXXX | y | n | XXXXX | XXXXXX | XXXXXXX |
| XXXX | y | n | XXXXX | XXXXXX | XXXXXXX |
| XXXX | y | n | XXXXX | XXXXXX | XXXXXXX |
| XXXX | y | n | XXXXX | XXXXXX | XXXXXXX |
| XXXX | y | n | XXXXX | XXXXXX | XXXXXXX |
| XXXX | y | n | XXXXX | XXXXXX | XXXXXXX |
| XXXX | y | n | XXXXX | XXXXXX | XXXXXXX |
| XXXX | y | n | XXXXX | XXXXXX | XXXXXXX |

| | | | | | |
|---|---|---|---|---|---|
| XXXX | y | n | XXXXX | XXXXXX | XXXXXXX |
| XXXX | y | n | XXXXX | XXXXXX | XXXXXXX |
| XXXX | y | n | XXXXX | XXXXXX | XXXXXXX |
| XXXX | y | n | XXXXX | XXXXXX | XXXXXXX |
| XXXX | y | n | XXXXX | XXXXXX | XXXXXXX |
| XXXX | y | n | XXXXX | XXXXXX | XXXXXXX |
| XXXX | y | n | XXXXX | XXXXXX | XXXXXXX |
| XXXX | y | n | XXXXX | XXXXXX | XXXXXXX |
| XXXX | y | n | XXXXX | XXXXXX | XXXXXXX |
| XXXX | y | n | XXXXX | XXXXXX | XXXXXXX |
| XXXX | y | n | XXXXX | XXXXXX | XXXXXXX |
| XXXX | y | n | XXXXX | XXXXXX | XXXXXXX |
| XXXX | y | n | XXXXX | XXXXXX | XXXXXXX |
| XXXX | y | n | XXXXX | XXXXXX | XXXXXXX |
| XXXX | y | n | XXXXX | XXXXXX | XXXXXXX |
| XXXX | y | n | XXXXX | XXXXXX | XXXXXXX |
| XXXX | y | n | XXXXX | XXXXXX | XXXXXXX |
| XXXX | y | n | XXXXX | XXXXXX | XXXXXXX |
| XXXX | y | n | XXXXX | XXXXXX | XXXXXXX |
| XXXX | y | n | XXXXX | XXXXXX | XXXXXXX |
| XXXX | y | n | XXXXX | XXXXXX | XXXXXXX |
| XXXX | y | n | XXXXX | XXXXXX | XXXXXXX |
| XXXX | y | n | XXXXX | XXXXXX | XXXXXXX |
| XXXX | y | n | XXXXX | XXXXXX | XXXXXXX |
| XXXX | y | n | XXXXX | XXXXXX | XXXXXXX |
| XXXX | y | n | XXXXX | XXXXXX | XXXXXXX |
| XXXX | y | n | XXXXX | XXXXXX | XXXXXXX |
| XXXX | y | n | XXXXX | XXXXXX | XXXXXXX |
| XXXX | y | n | XXXXX | XXXXXX | XXXXXXX |
| XXXX | y | n | XXXXX | XXXXXX | XXXXXXX |
| XXXX | y | n | XXXXX | XXXXXX | XXXXXXX |
| XXXX | y | n | XXXXX | XXXXXX | XXXXXXX |
| XXXX | y | n | XXXXX | XXXXXX | XXXXXXX |
| XXXX | y | n | XXXXX | XXXXXX | XXXXXXX |
| XXXX | y | n | XXXXX | XXXXXX | XXXXXXX |
| XXXX | y | n | XXXXX | XXXXXX | XXXXXXX |
| XXXX | y | n | XXXXX | XXXXXX | XXXXXXX |
| XXXX | y | n | XXXXX | XXXXXX | XXXXXXX |
| XXXX | y | n | XXXXX | XXXXXX | XXXXXXX |
| XXXX | y | n | XXXXX | XXXXXX | XXXXXXX |
| XXXX | y | n | XXXXX | XXXXXX | XXXXXXX |
| XXXX | y | n | XXXXX | XXXXXX | XXXXXXX |
| XXXX | y | n | XXXXX | XXXXXX | XXXXXXX |
| XXXX | y | n | XXXXX | XXXXXX | XXXXXXX |
| XXXX | y | n | XXXXX | XXXXXX | XXXXXXX |
| XXXX | y | n | XXXXX | XXXXXX | XXXXXXX |
| XXXX | y | n | XXXXX | XXXXXX | XXXXXXX |
| XXXX | y | n | XXXXX | XXXXXX | XXXXXXX |
| XXXX | y | n | XXXXX | XXXXXX | XXXXXXX |
| XXXX | y | n | XXXXX | XXXXXX | XXXXXXX |

| | | | | | |
|---|---|---|---|---|---|
| XXXX | y | n | XXXXX | XXXXXX | XXXXXXX |
| XXXX | y | n | XXXXX | XXXXXX | XXXXXXX |
| XXXX | y | n | XXXXX | XXXXXX | XXXXXXX |
| XXXX | y | n | XXXXX | XXXXXX | XXXXXXX |
| XXXX | y | n | XXXXX | XXXXXX | XXXXXXX |
| XXXX | y | n | XXXXX | XXXXXX | XXXXXXX |
| XXXX | y | n | XXXXX | XXXXXX | XXXXXXX |
| XXXX | y | n | XXXXX | XXXXXX | XXXXXXX |
| XXXX | y | n | XXXXX | XXXXXX | XXXXXXX |
| XXXX | y | n | XXXXX | XXXXXX | XXXXXXX |
| XXXX | y | n | XXXXX | XXXXXX | XXXXXXX |
| XXXX | y | n | XXXXX | XXXXXX | XXXXXXX |
| XXXX | y | n | XXXXX | XXXXXX | XXXXXXX |
| XXXX | y | n | XXXXX | XXXXXX | XXXXXXX |
| XXXX | y | n | XXXXX | XXXXXX | XXXXXXX |
| XXXX | y | n | XXXXX | XXXXXX | XXXXXXX |
| XXXX | y | n | XXXXX | XXXXXX | XXXXXXX |
| XXXX | y | n | XXXXX | XXXXXX | XXXXXXX |
| XXXX | y | n | XXXXX | XXXXXX | XXXXXXX |
| XXXX | y | n | XXXXX | XXXXXX | XXXXXXX |
| XXXX | y | n | XXXXX | XXXXXX | XXXXXXX |
| XXXX | y | n | XXXXX | XXXXXX | XXXXXXX |
| XXXX | y | n | XXXXX | XXXXXX | XXXXXXX |
| XXXX | y | n | XXXXX | XXXXXX | XXXXXXX |
| XXXX | y | n | XXXXX | XXXXXX | XXXXXXX |
| XXXX | y | n | XXXXX | XXXXXX | XXXXXXX |
| XXXX | y | n | XXXXX | XXXXXX | XXXXXXX |
| XXXX | y | n | XXXXX | XXXXXX | XXXXXXX |
| XXXX | y | n | XXXXX | XXXXXX | XXXXXXX |
| XXXX | y | n | XXXXX | XXXXXX | XXXXXXX |
| XXXX | y | n | XXXXX | XXXXXX | XXXXXXX |
| XXXX | y | n | XXXXX | XXXXXX | XXXXXXX |
| XXXX | y | n | XXXXX | XXXXXX | XXXXXXX |
| XXXX | y | n | XXXXX | XXXXXX | XXXXXXX |
| XXXX | y | n | XXXXX | XXXXXX | XXXXXXX |
| XXXX | y | n | XXXXX | XXXXXX | XXXXXXX |
| XXXX | y | n | XXXXX | XXXXXX | XXXXXXX |
| XXXX | y | n | XXXXX | XXXXXX | XXXXXXX |
| XXXX | y | n | XXXXX | XXXXXX | XXXXXXX |
| XXXX | y | n | XXXXX | XXXXXX | XXXXXXX |
| XXXX | y | n | XXXXX | XXXXXX | XXXXXXX |
| XXXX | y | n | XXXXX | XXXXXX | XXXXXXX |
| XXXX | y | n | XXXXX | XXXXXX | XXXXXXX |
| XXXX | y | n | XXXXX | XXXXXX | XXXXXXX |
| XXXX | y | n | XXXXX | XXXXXX | XXXXXXX |
| XXXX | y | n | XXXXX | XXXXXX | XXXXXXX |
| XXXX | y | n | XXXXX | XXXXXX | XXXXXXX |
| XXXX | y | n | XXXXX | XXXXXX | XXXXXXX |
| XXXX | y | n | XXXXX | XXXXXX | XXXXXXX |
| XXXX | y | n | XXXXX | XXXXXX | XXXXXXX |

| | | | | | |
|---|---|---|---|---|---|
| XXXX | y | n | XXXXX | XXXXXX | XXXXXXX |
| XXXX | y | n | XXXXX | XXXXXX | XXXXXXX |
| XXXX | y | n | XXXXX | XXXXXX | XXXXXXX |
| XXXX | y | n | XXXXX | XXXXXX | XXXXXXX |
| XXXX | y | n | XXXXX | XXXXXX | XXXXXXX |
| XXXX | y | n | XXXXX | XXXXXX | XXXXXXX |
| XXXX | y | n | XXXXX | XXXXXX | XXXXXXX |
| XXXX | y | n | XXXXX | XXXXXX | XXXXXXX |
| XXXX | y | n | XXXXX | XXXXXX | XXXXXXX |
| XXXX | y | n | XXXXX | XXXXXX | XXXXXXX |
| XXXX | y | n | XXXXX | XXXXXX | XXXXXXX |
| XXXX | y | n | XXXXX | XXXXXX | XXXXXXX |
| XXXX | y | n | XXXXX | XXXXXX | XXXXXXX |
| XXXX | y | n | XXXXX | XXXXXX | XXXXXXX |
| XXXX | y | n | XXXXX | XXXXXX | XXXXXXX |
| XXXX | y | n | XXXXX | XXXXXX | XXXXXXX |
| XXXX | y | n | XXXXX | XXXXXX | XXXXXXX |
| XXXX | y | n | XXXXX | XXXXXX | XXXXXXX |
| XXXX | y | n | XXXXX | XXXXXX | XXXXXXX |
| XXXX | y | n | XXXXX | XXXXXX | XXXXXXX |
| XXXX | y | n | XXXXX | XXXXXX | XXXXXXX |
| XXXX | y | n | XXXXX | XXXXXX | XXXXXXX |
| XXXX | y | n | XXXXX | XXXXXX | XXXXXXX |
| XXXX | y | n | XXXXX | XXXXXX | XXXXXXX |
| XXXX | y | n | XXXXX | XXXXXX | XXXXXXX |
| XXXX | y | n | XXXXX | XXXXXX | XXXXXXX |
| XXXX | y | n | XXXXX | XXXXXX | XXXXXXX |
| XXXX | y | n | XXXXX | XXXXXX | XXXXXXX |
| XXXX | y | n | XXXXX | XXXXXX | XXXXXXX |
| XXXX | y | n | XXXXX | XXXXXX | XXXXXXX |
| XXXX | y | n | XXXXX | XXXXXX | XXXXXXX |
| XXXX | y | n | XXXXX | XXXXXX | XXXXXXX |
| XXXX | y | n | XXXXX | XXXXXX | XXXXXXX |
| XXXX | y | n | XXXXX | XXXXXX | XXXXXXX |
| XXXX | y | n | XXXXX | XXXXXX | XXXXXXX |
| XXXX | y | n | XXXXX | XXXXXX | XXXXXXX |
| XXXX | y | n | XXXXX | XXXXXX | XXXXXXX |
| XXXX | y | n | XXXXX | XXXXXX | XXXXXXX |
| XXXX | y | n | XXXXX | XXXXXX | XXXXXXX |
| XXXX | y | n | XXXXX | XXXXXX | XXXXXXX |
| XXXX | y | n | XXXXX | XXXXXX | XXXXXXX |
| XXXX | y | n | XXXXX | XXXXXX | XXXXXXX |
| XXXX | y | n | XXXXX | XXXXXX | XXXXXXX |
| XXXX | y | n | XXXXX | XXXXXX | XXXXXXX |
| XXXX | y | n | XXXXX | XXXXXX | XXXXXXX |
| XXXX | y | n | XXXXX | XXXXXX | XXXXXXX |
| XXXX | y | n | XXXXX | XXXXXX | XXXXXXX |
| XXXX | y | n | XXXXX | XXXXXX | XXXXXXX |
| XXXX | y | n | XXXXX | XXXXXX | XXXXXXX |
| XXXX | y | n | XXXXX | XXXXXX | XXXXXXX |

| | | | | | |
|---|---|---|---|---|---|
| XXXX | y | n | XXXXX | XXXXXX | XXXXXXX |
| XXXX | y | n | XXXXX | XXXXXX | XXXXXXX |
| XXXX | y | n | XXXXX | XXXXXX | XXXXXXX |
| XXXX | y | n | XXXXX | XXXXXX | XXXXXXX |
| XXXX | y | n | XXXXX | XXXXXX | XXXXXXX |
| XXXX | y | n | XXXXX | XXXXXX | XXXXXXX |
| XXXX | y | n | XXXXX | XXXXXX | XXXXXXX |
| XXXX | y | n | XXXXX | XXXXXX | XXXXXXX |
| XXXX | y | n | XXXXX | XXXXXX | XXXXXXX |
| XXXX | y | n | XXXXX | XXXXXX | XXXXXXX |
| XXXX | y | n | XXXXX | XXXXXX | XXXXXXX |
| XXXX | y | n | XXXXX | XXXXXX | XXXXXXX |
| XXXX | y | n | XXXXX | XXXXXX | XXXXXXX |
| XXXX | y | n | XXXXX | XXXXXX | XXXXXXX |
| XXXX | y | n | XXXXX | XXXXXX | XXXXXXX |
| XXXX | y | n | XXXXX | XXXXXX | XXXXXXX |
| XXXX | y | n | XXXXX | XXXXXX | XXXXXXX |
| XXXX | y | n | XXXXX | XXXXXX | XXXXXXX |
| XXXX | y | n | XXXXX | XXXXXX | XXXXXXX |
| XXXX | y | n | XXXXX | XXXXXX | XXXXXXX |
| XXXX | y | n | XXXXX | XXXXXX | XXXXXXX |
| XXXX | y | n | XXXXX | XXXXXX | XXXXXXX |
| XXXX | y | n | XXXXX | XXXXXX | XXXXXXX |
| XXXX | y | n | XXXXX | XXXXXX | XXXXXXX |
| XXXX | y | n | XXXXX | XXXXXX | XXXXXXX |
| XXXX | y | n | XXXXX | XXXXXX | XXXXXXX |
| XXXX | y | n | XXXXX | XXXXXX | XXXXXXX |
| XXXX | y | n | XXXXX | XXXXXX | XXXXXXX |
| XXXX | y | n | XXXXX | XXXXXX | XXXXXXX |
| XXXX | y | n | XXXXX | XXXXXX | XXXXXXX |
| XXXX | y | n | XXXXX | XXXXXX | XXXXXXX |
| XXXX | y | n | XXXXX | XXXXXX | XXXXXXX |
| XXXX | y | n | XXXXX | XXXXXX | XXXXXXX |
| XXXX | y | n | XXXXX | XXXXXX | XXXXXXX |
| XXXX | y | n | XXXXX | XXXXXX | XXXXXXX |
| XXXX | y | n | XXXXX | XXXXXX | XXXXXXX |
| XXXX | y | n | XXXXX | XXXXXX | XXXXXXX |
| XXXX | y | n | XXXXX | XXXXXX | XXXXXXX |
| XXXX | y | n | XXXXX | XXXXXX | XXXXXXX |
| XXXX | y | n | XXXXX | XXXXXX | XXXXXXX |
| XXXX | y | n | XXXXX | XXXXXX | XXXXXXX |
| XXXX | y | n | XXXXX | XXXXXX | XXXXXXX |
| XXXX | y | n | XXXXX | XXXXXX | XXXXXXX |
| XXXX | y | n | XXXXX | XXXXXX | XXXXXXX |
| XXXX | y | n | XXXXX | XXXXXX | XXXXXXX |
| XXXX | y | n | XXXXX | XXXXXX | XXXXXXX |
| XXXX | y | n | XXXXX | XXXXXX | XXXXXXX |
| XXXX | y | n | XXXXX | XXXXXX | XXXXXXX |
| XXXX | y | n | XXXXX | XXXXXX | XXXXXXX |
| XXXX | y | n | XXXXX | XXXXXX | XXXXXXX |

| | | | | | |
|---|---|---|---|---|---|
| XXXX | y | n | XXXXX | XXXXXX | XXXXXXX |
| XXXX | y | n | XXXXX | XXXXXX | XXXXXXX |
| XXXX | y | n | XXXXX | XXXXXX | XXXXXXX |
| XXXX | y | n | XXXXX | XXXXXX | XXXXXXX |
| XXXX | y | n | XXXXX | XXXXXX | XXXXXXX |
| XXXX | y | n | XXXXX | XXXXXX | XXXXXXX |
| XXXX | y | n | XXXXX | XXXXXX | XXXXXXX |
| XXXX | y | n | XXXXX | XXXXXX | XXXXXXX |
| XXXX | y | n | XXXXX | XXXXXX | XXXXXXX |
| XXXX | y | n | XXXXX | XXXXXX | XXXXXXX |
| XXXX | y | n | XXXXX | XXXXXX | XXXXXXX |
| XXXX | y | n | XXXXX | XXXXXX | XXXXXXX |
| XXXX | y | n | XXXXX | XXXXXX | XXXXXXX |
| XXXX | y | n | XXXXX | XXXXXX | XXXXXXX |
| XXXX | y | n | XXXXX | XXXXXX | XXXXXXX |
| XXXX | y | n | XXXXX | XXXXXX | XXXXXXX |
| XXXX | y | n | XXXXX | XXXXXX | XXXXXXX |
| XXXX | y | n | XXXXX | XXXXXX | XXXXXXX |
| XXXX | y | n | XXXXX | XXXXXX | XXXXXXX |
| XXXX | y | n | XXXXX | XXXXXX | XXXXXXX |
| XXXX | y | n | XXXXX | XXXXXX | XXXXXXX |
| XXXX | y | n | XXXXX | XXXXXX | XXXXXXX |
| XXXX | y | n | XXXXX | XXXXXX | XXXXXXX |
| XXXX | y | n | XXXXX | XXXXXX | XXXXXXX |
| XXXX | y | n | XXXXX | XXXXXX | XXXXXXX |
| XXXX | y | n | XXXXX | XXXXXX | XXXXXXX |
| XXXX | y | n | XXXXX | XXXXXX | XXXXXXX |
| XXXX | y | n | XXXXX | XXXXXX | XXXXXXX |
| XXXX | y | n | XXXXX | XXXXXX | XXXXXXX |
| XXXX | y | n | XXXXX | XXXXXX | XXXXXXX |
| XXXX | y | n | XXXXX | XXXXXX | XXXXXXX |
| XXXX | y | n | XXXXX | XXXXXX | XXXXXXX |
| XXXX | y | n | XXXXX | XXXXXX | XXXXXXX |
| XXXX | y | n | XXXXX | XXXXXX | XXXXXXX |
| XXXX | y | n | XXXXX | XXXXXX | XXXXXXX |
| XXXX | y | n | XXXXX | XXXXXX | XXXXXXX |
| XXXX | y | n | XXXXX | XXXXXX | XXXXXXX |
| XXXX | y | n | XXXXX | XXXXXX | XXXXXXX |
| XXXX | y | n | XXXXX | XXXXXX | XXXXXXX |
| XXXX | y | n | XXXXX | XXXXXX | XXXXXXX |
| XXXX | y | n | XXXXX | XXXXXX | XXXXXXX |
| XXXX | y | n | XXXXX | XXXXXX | XXXXXXX |
| XXXX | y | n | XXXXX | XXXXXX | XXXXXXX |
| XXXX | y | n | XXXXX | XXXXXX | XXXXXXX |
| XXXX | y | n | XXXXX | XXXXXX | XXXXXXX |
| XXXX | y | n | XXXXX | XXXXXX | XXXXXXX |
| XXXX | y | n | XXXXX | XXXXXX | XXXXXXX |
| XXXX | y | n | XXXXX | XXXXXX | XXXXXXX |
| XXXX | y | n | XXXXX | XXXXXX | XXXXXXX |
| XXXX | y | n | XXXXX | XXXXXX | XXXXXXX |

| | | | | | |
|---|---|---|---|---|---|
| XXXX | y | n | XXXXX | XXXXXX | XXXXXXX |
| XXXX | y | n | XXXXX | XXXXXX | XXXXXXX |
| XXXX | y | n | XXXXX | XXXXXX | XXXXXXX |
| XXXX | y | n | XXXXX | XXXXXX | XXXXXXX |
| XXXX | y | n | XXXXX | XXXXXX | XXXXXXX |
| XXXX | y | n | XXXXX | XXXXXX | XXXXXXX |
| XXXX | y | n | XXXXX | XXXXXX | XXXXXXX |
| XXXX | y | n | XXXXX | XXXXXX | XXXXXXX |
| XXXX | y | n | XXXXX | XXXXXX | XXXXXXX |
| XXXX | y | n | XXXXX | XXXXXX | XXXXXXX |
| XXXX | y | n | XXXXX | XXXXXX | XXXXXXX |
| XXXX | y | n | XXXXX | XXXXXX | XXXXXXX |
| XXXX | y | n | XXXXX | XXXXXX | XXXXXXX |
| XXXX | y | n | XXXXX | XXXXXX | XXXXXXX |
| XXXX | y | n | XXXXX | XXXXXX | XXXXXXX |
| XXXX | y | n | XXXXX | XXXXXX | XXXXXXX |
| XXXX | y | n | XXXXX | XXXXXX | XXXXXXX |
| XXXX | y | n | XXXXX | XXXXXX | XXXXXXX |
| XXXX | y | n | XXXXX | XXXXXX | XXXXXXX |
| XXXX | y | n | XXXXX | XXXXXX | XXXXXXX |
| XXXX | y | n | XXXXX | XXXXXX | XXXXXXX |
| XXXX | y | n | XXXXX | XXXXXX | XXXXXXX |
| XXXX | y | n | XXXXX | XXXXXX | XXXXXXX |
| XXXX | y | n | XXXXX | XXXXXX | XXXXXXX |
| XXXX | y | n | XXXXX | XXXXXX | XXXXXXX |
| XXXX | y | n | XXXXX | XXXXXX | XXXXXXX |
| XXXX | y | n | XXXXX | XXXXXX | XXXXXXX |
| XXXX | y | n | XXXXX | XXXXXX | XXXXXXX |
| XXXX | y | n | XXXXX | XXXXXX | XXXXXXX |
| XXXX | y | n | XXXXX | XXXXXX | XXXXXXX |
| XXXX | y | n | XXXXX | XXXXXX | XXXXXXX |
| XXXX | y | n | XXXXX | XXXXXX | XXXXXXX |
| XXXX | y | n | XXXXX | XXXXXX | XXXXXXX |
| XXXX | y | n | XXXXX | XXXXXX | XXXXXXX |
| XXXX | y | n | XXXXX | XXXXXX | XXXXXXX |
| XXXX | y | n | XXXXX | XXXXXX | XXXXXXX |
| XXXX | y | n | XXXXX | XXXXXX | XXXXXXX |
| XXXX | y | n | XXXXX | XXXXXX | XXXXXXX |
| XXXX | y | n | XXXXX | XXXXXX | XXXXXXX |
| XXXX | y | n | XXXXX | XXXXXX | XXXXXXX |
| XXXX | y | n | XXXXX | XXXXXX | XXXXXXX |
| XXXX | y | n | XXXXX | XXXXXX | XXXXXXX |
| XXXX | y | n | XXXXX | XXXXXX | XXXXXXX |
| XXXX | y | n | XXXXX | XXXXXX | XXXXXXX |
| XXXX | y | n | XXXXX | XXXXXX | XXXXXXX |
| XXXX | y | n | XXXXX | XXXXXX | XXXXXXX |
| XXXX | y | n | XXXXX | XXXXXX | XXXXXXX |
| XXXX | y | n | XXXXX | XXXXXX | XXXXXXX |
| XXXX | y | n | XXXXX | XXXXXX | XXXXXXX |
| XXXX | y | n | XXXXX | XXXXXX | XXXXXXX |

| | | | | | |
|---|---|---|---|---|---|
| XXXX | y | n | XXXXX | XXXXXX | XXXXXXX |
| XXXX | y | n | XXXXX | XXXXXX | XXXXXXX |
| XXXX | y | n | XXXXX | XXXXXX | XXXXXXX |
| XXXX | y | n | XXXXX | XXXXXX | XXXXXXX |
| XXXX | y | n | XXXXX | XXXXXX | XXXXXXX |
| XXXX | y | n | XXXXX | XXXXXX | XXXXXXX |
| XXXX | y | n | XXXXX | XXXXXX | XXXXXXX |
| XXXX | y | n | XXXXX | XXXXXX | XXXXXXX |
| XXXX | y | n | XXXXX | XXXXXX | XXXXXXX |
| XXXX | y | n | XXXXX | XXXXXX | XXXXXXX |
| XXXX | y | n | XXXXX | XXXXXX | XXXXXXX |
| XXXX | y | n | XXXXX | XXXXXX | XXXXXXX |
| XXXX | y | n | XXXXX | XXXXXX | XXXXXXX |
| XXXX | y | n | XXXXX | XXXXXX | XXXXXXX |
| XXXX | y | n | XXXXX | XXXXXX | XXXXXXX |
| XXXX | y | n | XXXXX | XXXXXX | XXXXXXX |
| XXXX | y | n | XXXXX | XXXXXX | XXXXXXX |
| XXXX | y | n | XXXXX | XXXXXX | XXXXXXX |
| XXXX | y | n | XXXXX | XXXXXX | XXXXXXX |
| XXXX | y | n | XXXXX | XXXXXX | XXXXXXX |
| XXXX | y | n | XXXXX | XXXXXX | XXXXXXX |
| XXXX | y | n | XXXXX | XXXXXX | XXXXXXX |
| XXXX | y | n | XXXXX | XXXXXX | XXXXXXX |
| XXXX | y | n | XXXXX | XXXXXX | XXXXXXX |
| XXXX | y | n | XXXXX | XXXXXX | XXXXXXX |
| XXXX | y | n | XXXXX | XXXXXX | XXXXXXX |
| XXXX | y | n | XXXXX | XXXXXX | XXXXXXX |
| XXXX | y | n | XXXXX | XXXXXX | XXXXXXX |
| XXXX | y | n | XXXXX | XXXXXX | XXXXXXX |
| XXXX | y | n | XXXXX | XXXXXX | XXXXXXX |
| XXXX | y | n | XXXXX | XXXXXX | XXXXXXX |
| XXXX | y | n | XXXXX | XXXXXX | XXXXXXX |
| XXXX | y | n | XXXXX | XXXXXX | XXXXXXX |
| XXXX | y | n | XXXXX | XXXXXX | XXXXXXX |
| XXXX | y | n | XXXXX | XXXXXX | XXXXXXX |
| XXXX | y | n | XXXXX | XXXXXX | XXXXXXX |
| XXXX | y | n | XXXXX | XXXXXX | XXXXXXX |
| XXXX | y | n | XXXXX | XXXXXX | XXXXXXX |
| XXXX | y | n | XXXXX | XXXXXX | XXXXXXX |
| XXXX | y | n | XXXXX | XXXXXX | XXXXXXX |
| XXXX | y | n | XXXXX | XXXXXX | XXXXXXX |
| XXXX | y | n | XXXXX | XXXXXX | XXXXXXX |
| XXXX | y | n | XXXXX | XXXXXX | XXXXXXX |
| XXXX | y | n | XXXXX | XXXXXX | XXXXXXX |
| XXXX | y | n | XXXXX | XXXXXX | XXXXXXX |
| XXXX | y | n | XXXXX | XXXXXX | XXXXXXX |
| XXXX | y | n | XXXXX | XXXXXX | XXXXXXX |
| XXXX | y | n | XXXXX | XXXXXX | XXXXXXX |
| XXXX | y | n | XXXXX | XXXXXX | XXXXXXX |
| XXXX | y | n | XXXXX | XXXXXX | XXXXXXX |

| | | | | | |
|---|---|---|---|---|---|
| XXXX | y | n | XXXXX | XXXXXX | XXXXXXX |
| XXXX | y | n | XXXXX | XXXXXX | XXXXXXX |
| XXXX | y | n | XXXXX | XXXXXX | XXXXXXX |
| XXXX | y | n | XXXXX | XXXXXX | XXXXXXX |
| XXXX | y | n | XXXXX | XXXXXX | XXXXXXX |
| XXXX | y | n | XXXXX | XXXXXX | XXXXXXX |
| XXXX | y | n | XXXXX | XXXXXX | XXXXXXX |
| XXXX | y | n | XXXXX | XXXXXX | XXXXXXX |
| XXXX | y | n | XXXXX | XXXXXX | XXXXXXX |
| XXXX | y | n | XXXXX | XXXXXX | XXXXXXX |
| XXXX | y | n | XXXXX | XXXXXX | XXXXXXX |
| XXXX | y | n | XXXXX | XXXXXX | XXXXXXX |
| XXXX | y | n | XXXXX | XXXXXX | XXXXXXX |
| XXXX | na | n | XXXXX | XXXXXX | XXXXXXX |
| XXXX | na | n | XXXXX | XXXXXX | XXXXXXX |
| XXXX | na | n | XXXXX | XXXXXX | XXXXXXX |
| XXXX | na | n | XXXXX | XXXXXX | XXXXXXX |
| XXXX | na | n | XXXXX | XXXXXX | XXXXXXX |
| XXXX | na | n | XXXXX | XXXXXX | XXXXXXX |
| XXXX | na | n | XXXXX | XXXXXX | XXXXXXX |
| XXXX | na | n | XXXXX | XXXXXX | XXXXXXX |
| XXXX | na | n | XXXXX | XXXXXX | XXXXXXX |
| XXXX | na | n | XXXXX | XXXXXX | XXXXXXX |
| XXXX | na | n | XXXXX | XXXXXX | XXXXXXX |
| XXXX | na | n | XXXXX | XXXXXX | XXXXXXX |
| XXXX | na | n | XXXXX | XXXXXX | XXXXXXX |
| XXXX | na | n | XXXXX | XXXXXX | XXXXXXX |
| XXXX | na | n | XXXXX | XXXXXX | XXXXXXX |
| XXXX | na | n | XXXXX | XXXXXX | XXXXXXX |
| XXXX | na | n | XXXXX | XXXXXX | XXXXXXX |
| XXXX | na | n | XXXXX | XXXXXX | XXXXXXX |
| XXXX | na | n | XXXXX | XXXXXX | XXXXXXX |
| XXXX | na | n | XXXXX | XXXXXX | XXXXXXX |
| XXXX | na | n | XXXXX | XXXXXX | XXXXXXX |
| XXXX | na | n | XXXXX | XXXXXX | XXXXXXX |
| XXXX | na | n | XXXXX | XXXXXX | XXXXXXX |
| XXXX | na | n | XXXXX | XXXXXX | XXXXXXX |
| XXXX | na | n | XXXXX | XXXXXX | XXXXXXX |
| XXXX | na | n | XXXXX | XXXXXX | XXXXXXX |
| XXXX | na | n | XXXXX | XXXXXX | XXXXXXX |
| XXXX | na | n | XXXXX | XXXXXX | XXXXXXX |
| XXXX | na | n | XXXXX | XXXXXX | XXXXXXX |
| XXXX | na | n | XXXXX | XXXXXX | XXXXXXX |
| XXXX | na | n | XXXXX | XXXXXX | XXXXXXX |
| XXXX | na | n | XXXXX | XXXXXX | XXXXXXX |
| XXXX | na | n | XXXXX | XXXXXX | XXXXXXX |
| XXXX | na | n | XXXXX | XXXXXX | XXXXXXX |
| XXXX | na | n | XXXXX | XXXXXX | XXXXXXX |
| XXXX | na | n | XXXXX | XXXXXX | XXXXXXX |
| XXXX | na | n | XXXXX | XXXXXX | XXXXXXX |
| XXXX | na | n | XXXXX | XXXXXX | XXXXXXX |

| | | | | | |
|---|---|---|---|---|---|
| XXXX | na | n | XXXXX | XXXXXX | XXXXXXX |
| XXXX | na | n | XXXXX | XXXXXX | XXXXXXX |
| XXXX | na | n | XXXXX | XXXXXX | XXXXXXX |
| XXXX | na | n | XXXXX | XXXXXX | XXXXXXX |
| XXXX | na | n | XXXXX | XXXXXX | XXXXXXX |
| XXXX | na | n | XXXXX | XXXXXX | XXXXXXX |
| XXXX | na | n | XXXXX | XXXXXX | XXXXXXX |
| XXXX | na | n | XXXXX | XXXXXX | XXXXXXX |
| XXXX | na | n | XXXXX | XXXXXX | XXXXXXX |
| XXXX | na | n | XXXXX | XXXXXX | XXXXXXX |
| XXXX | na | n | XXXXX | XXXXXX | XXXXXXX |
| XXXX | na | n | XXXXX | XXXXXX | XXXXXXX |
| XXXX | na | n | XXXXX | XXXXXX | XXXXXXX |
| XXXX | na | n | XXXXX | XXXXXX | XXXXXXX |
| XXXX | na | n | XXXXX | XXXXXX | XXXXXXX |
| XXXX | na | n | XXXXX | XXXXXX | XXXXXXX |
| XXXX | na | n | XXXXX | XXXXXX | XXXXXXX |
| XXXX | na | n | XXXXX | XXXXXX | XXXXXXX |
| XXXX | na | n | XXXXX | XXXXXX | XXXXXXX |
| XXXX | na | n | XXXXX | XXXXXX | XXXXXXX |
| XXXX | na | n | XXXXX | XXXXXX | XXXXXXX |
| XXXX | na | n | XXXXX | XXXXXX | XXXXXXX |
| XXXX | na | n | XXXXX | XXXXXX | XXXXXXX |
| XXXX | na | n | XXXXX | XXXXXX | XXXXXXX |
| XXXX | na | n | XXXXX | XXXXXX | XXXXXXX |
| XXXX | na | n | XXXXX | XXXXXX | XXXXXXX |
| XXXX | na | n | XXXXX | XXXXXX | XXXXXXX |
| XXXX | na | n | XXXXX | XXXXXX | XXXXXXX |
| XXXX | na | n | XXXXX | XXXXXX | XXXXXXX |
| XXXX | na | n | XXXXX | XXXXXX | XXXXXXX |
| XXXX | na | n | XXXXX | XXXXXX | XXXXXXX |
| XXXX | na | n | XXXXX | XXXXXX | XXXXXXX |
| XXXX | na | n | XXXXX | XXXXXX | XXXXXXX |
| XXXX | na | n | XXXXX | XXXXXX | XXXXXXX |
| XXXX | na | n | XXXXX | XXXXXX | XXXXXXX |
| XXXX | na | n | XXXXX | XXXXXX | XXXXXXX |
| XXXX | na | n | XXXXX | XXXXXX | XXXXXXX |
| XXXX | na | n | XXXXX | XXXXXX | XXXXXXX |
| XXXX | na | n | XXXXX | XXXXXX | XXXXXXX |
| XXXX | na | n | XXXXX | XXXXXX | XXXXXXX |
| XXXX | na | n | XXXXX | XXXXXX | XXXXXXX |
| XXXX | na | n | XXXXX | XXXXXX | XXXXXXX |
| XXXX | na | n | XXXXX | XXXXXX | XXXXXXX |
| XXXX | na | n | XXXXX | XXXXXX | XXXXXXX |
| XXXX | na | n | XXXXX | XXXXXX | XXXXXXX |
| XXXX | na | n | XXXXX | XXXXXX | XXXXXXX |
| XXXX | na | n | XXXXX | XXXXXX | XXXXXXX |
| XXXX | na | n | XXXXX | XXXXXX | XXXXXXX |
| XXXX | na | n | XXXXX | XXXXXX | XXXXXXX |
| XXXX | na | n | XXXXX | XXXXXX | XXXXXXX |

| | | | | | |
|---|---|---|---|---|---|
| XXXX | na | n | XXXXX | XXXXXX | XXXXXXX |
| XXXX | na | n | XXXXX | XXXXXX | XXXXXXX |
| XXXX | na | n | XXXXX | XXXXXX | XXXXXXX |
| XXXX | na | n | XXXXX | XXXXXX | XXXXXXX |
| XXXX | na | n | XXXXX | XXXXXX | XXXXXXX |
| XXXX | y | y | XXXXX | XXXXXX | XXXXXXX |
| XXXX | y | y | XXXXX | XXXXXX | XXXXXXX |
| XXXX | y | y | XXXXX | XXXXXX | XXXXXXX |
| XXXX | y | y | XXXXX | XXXXXX | XXXXXXX |
| XXXX | y | y | XXXXX | XXXXXX | XXXXXXX |
| XXXX | y | y | XXXXX | XXXXXX | XXXXXXX |
| XXXX | y | y | XXXXX | XXXXXX | XXXXXXX |
| XXXX | y | y | XXXXX | XXXXXX | XXXXXXX |
| XXXX | y | y | XXXXX | XXXXXX | XXXXXXX |
| XXXX | y | y | XXXXX | XXXXXX | XXXXXXX |
| XXXX | y | y | XXXXX | XXXXXX | XXXXXXX |
| XXXX | y | y | XXXXX | XXXXXX | XXXXXXX |
| XXXX | y | y | XXXXX | XXXXXX | XXXXXXX |
| XXXX | y | y | XXXXX | XXXXXX | XXXXXXX |
| XXXX | y | y | XXXXX | XXXXXX | XXXXXXX |
| XXXX | y | y | XXXXX | XXXXXX | XXXXXXX |
| XXXX | y | y | XXXXX | XXXXXX | XXXXXXX |
| XXXX | y | y | XXXXX | XXXXXX | XXXXXXX |
| XXXX | y | y | XXXXX | XXXXXX | XXXXXXX |
| XXXX | y | y | XXXXX | XXXXXX | XXXXXXX |
| XXXX | y | y | XXXXX | XXXXXX | XXXXXXX |
| XXXX | y | y | XXXXX | XXXXXX | XXXXXXX |
| XXXX | y | y | XXXXX | XXXXXX | XXXXXXX |
| XXXX | y | y | XXXXX | XXXXXX | XXXXXXX |
| XXXX | y | y | XXXXX | XXXXXX | XXXXXXX |
| XXXX | y | y | XXXXX | XXXXXX | XXXXXXX |
| XXXX | n | n | XXXXX | XXXXXX | XXXXXXX |
| XXXX | n | n | XXXXX | XXXXXX | XXXXXXX |
| XXXX | n | y | XXXXX | XXXXXX | XXXXXXX |
| XXXX | n | y | XXXXX | XXXXXX | XXXXXXX |
| XXXX | n | y | XXXXX | XXXXXX | XXXXXXX |
| XXXX | n | y | XXXXX | XXXXXX | XXXXXXX |
| XXXX | n | y | XXXXX | XXXXXX | XXXXXXX |
| XXXX | n | y | XXXXX | XXXXXX | XXXXXXX |
| XXXX | n | y | XXXXX | XXXXXX | XXXXXXX |
| XXXX | n | y | XXXXX | XXXXXX | XXXXXXX |
| XXXX | n | y | XXXXX | XXXXXX | XXXXXXX |
| XXXX | n | y | XXXXX | XXXXXX | XXXXXXX |
| XXXX | n | y | XXXXX | XXXXXX | XXXXXXX |
| XXXX | n | y | XXXXX | XXXXXX | XXXXXXX |
| XXXX | n | y | XXXXX | XXXXXX | XXXXXXX |
| XXXX | n | y | XXXXX | XXXXXX | XXXXXXX |
| XXXX | n | y | XXXXX | XXXXXX | XXXXXXX |
| XXXX | n | y | XXXXX | XXXXXX | XXXXXXX |
| XXXX | n | y | XXXXX | XXXXXX | XXXXXXX |

| | | | | | |
|---|---|---|---|---|---|
| XXXX | n | y | XXXXX | XXXXXX | XXXXXXX |
| XXXX | n | y | XXXXX | XXXXXX | XXXXXXX |
| XXXX | n | y | XXXXX | XXXXXX | XXXXXXX |
| XXXX | n | y | XXXXX | XXXXXX | XXXXXXX |
| XXXX | n | y | XXXXX | XXXXXX | XXXXXXX |
| XXXX | n | y | XXXXX | XXXXXX | XXXXXXX |
| XXXX | n | y | XXXXX | XXXXXX | XXXXXXX |
| XXXX | n | y | XXXXX | XXXXXX | XXXXXXX |
| XXXX | n | y | XXXXX | XXXXXX | XXXXXXX |
| XXXX | n | y | XXXXX | XXXXXX | XXXXXXX |
| XXXX | n | y | XXXXX | XXXXXX | XXXXXXX |
| XXXX | y | n | XXXXX | XXXXXX | XXXXXXX |
| XXXX | y | n | XXXXX | XXXXXX | XXXXXXX |
| XXXX | y | n | XXXXX | XXXXXX | XXXXXXX |
| XXXX | n | y | XXXXX | XXXXXX | XXXXXXX |
| XXXX | n | n | XXXXX | XXXXXX | XXXXXXX |
| XXXX | n | n | XXXXX | XXXXXX | XXXXXXX |
| XXXX | n | n | XXXXX | XXXXXX | XXXXXXX |
| XXXX | n | n | XXXXX | XXXXXX | XXXXXXX |
| XXXX | n | n | XXXXX | XXXXXX | XXXXXXX |
| XXXX | n | n | XXXXX | XXXXXX | XXXXXXX |
| XXXX | n | n | XXXXX | XXXXXX | XXXXXXX |
| XXXX | n | n | XXXXX | XXXXXX | XXXXXXX |
| XXXX | y | y | XXXXX | XXXXXX | XXXXXXX |
| XXXX | y | y | XXXXX | XXXXXX | XXXXXXX |
| XXXX | y | y | XXXXX | XXXXXX | XXXXXXX |
| XXXX | y | y | XXXXX | XXXXXX | XXXXXXX |
| XXXX | y | y | XXXXX | XXXXXX | XXXXXXX |
| XXXX | y | y | XXXXX | XXXXXX | XXXXXXX |
| XXXX | y | y | XXXXX | XXXXXX | XXXXXXX |
| XXXX | y | y | XXXXX | XXXXXX | XXXXXXX |
| XXXX | y | y | XXXXX | XXXXXX | XXXXXXX |
| XXXX | y | y | XXXXX | XXXXXX | XXXXXXX |
| XXXX | y | y | XXXXX | XXXXXX | XXXXXXX |
| XXXX | y | y | XXXXX | XXXXXX | XXXXXXX |
| XXXX | y | y | XXXXX | XXXXXX | XXXXXXX |
| XXXX | y | y | XXXXX | XXXXXX | XXXXXXX |
| XXXX | y | y | XXXXX | XXXXXX | XXXXXXX |
| XXXX | y | y | XXXXX | XXXXXX | XXXXXXX |
| XXXX | y | y | XXXXX | XXXXXX | XXXXXXX |
| XXXX | y | y | XXXXX | XXXXXX | XXXXXXX |
| XXXX | y | y | XXXXX | XXXXXX | XXXXXXX |
| XXXX | y | y | XXXXX | XXXXXX | XXXXXXX |
| XXXX | y | y | XXXXX | XXXXXX | XXXXXXX |
| XXXX | y | y | XXXXX | XXXXXX | XXXXXXX |
| XXXX | y | y | XXXXX | XXXXXX | XXXXXXX |
| XXXX | y | y | XXXXX | XXXXXX | XXXXXXX |
| XXXX | y | y | XXXXX | XXXXXX | XXXXXXX |
| XXXX | y | y | XXXXX | XXXXXX | XXXXXXX |
| XXXX | y | y | XXXXX | XXXXXX | XXXXXXX |

| | | | | | |
|---|---|---|---|---|---|
| XXXX | y | y | XXXXX | XXXXXX | XXXXXXX |
| XXXX | y | y | XXXXX | XXXXXX | XXXXXXX |
| XXXX | y | y | XXXXX | XXXXXX | XXXXXXX |
| XXXX | y | y | XXXXX | XXXXXX | XXXXXXX |
| XXXX | y | y | XXXXX | XXXXXX | XXXXXXX |
| XXXX | y | y | XXXXX | XXXXXX | XXXXXXX |
| XXXX | y | y | XXXXX | XXXXXX | XXXXXXX |
| XXXX | y | y | XXXXX | XXXXXX | XXXXXXX |
| XXXX | y | y | XXXXX | XXXXXX | XXXXXXX |
| XXXX | y | y | XXXXX | XXXXXX | XXXXXXX |
| XXXX | y | y | XXXXX | XXXXXX | XXXXXXX |
| XXXX | y | y | XXXXX | XXXXXX | XXXXXXX |
| XXXX | y | y | XXXXX | XXXXXX | XXXXXXX |
| XXXX | y | y | XXXXX | XXXXXX | XXXXXXX |
| XXXX | y | y | XXXXX | XXXXXX | XXXXXXX |
| XXXX | y | y | XXXXX | XXXXXX | XXXXXXX |
| XXXX | y | y | XXXXX | XXXXXX | XXXXXXX |
| XXXX | y | y | XXXXX | XXXXXX | XXXXXXX |
| XXXX | y | y | XXXXX | XXXXXX | XXXXXXX |
| XXXX | y | y | XXXXX | XXXXXX | XXXXXXX |
| XXXX | y | y | XXXXX | XXXXXX | XXXXXXX |
| XXXX | y | y | XXXXX | XXXXXX | XXXXXXX |
| XXXX | y | y | XXXXX | XXXXXX | XXXXXXX |
| XXXX | y | y | XXXXX | XXXXXX | XXXXXXX |
| XXXX | y | y | XXXXX | XXXXXX | XXXXXXX |
| XXXX | y | y | XXXXX | XXXXXX | XXXXXXX |
| XXXX | y | y | XXXXX | XXXXXX | XXXXXXX |
| XXXX | y | y | XXXXX | XXXXXX | XXXXXXX |
| XXXX | y | y | XXXXX | XXXXXX | XXXXXXX |
| XXXX | y | y | XXXXX | XXXXXX | XXXXXXX |
| XXXX | y | y | XXXXX | XXXXXX | XXXXXXX |
| XXXX | y | y | XXXXX | XXXXXX | XXXXXXX |
| XXXX | y | y | XXXXX | XXXXXX | XXXXXXX |
| XXXX | y | y | XXXXX | XXXXXX | XXXXXXX |
| XXXX | y | y | XXXXX | XXXXXX | XXXXXXX |
| XXXX | y | y | XXXXX | XXXXXX | XXXXXXX |
| XXXX | y | y | XXXXX | XXXXXX | XXXXXXX |
| XXXX | y | y | XXXXX | XXXXXX | XXXXXXX |
| XXXX | y | y | XXXXX | XXXXXX | XXXXXXX |
| XXXX | n | n | XXXXX | XXXXXX | XXXXXXX |
| XXXX | n | n | XXXXX | XXXXXX | XXXXXXX |
| XXXX | n | n | XXXXX | XXXXXX | XXXXXXX |
| XXXX | n | n | XXXXX | XXXXXX | XXXXXXX |
| XXXX | n | n | XXXXX | XXXXXX | XXXXXXX |
| XXXX | n | n | XXXXX | XXXXXX | XXXXXXX |
| XXXX | n | n | XXXXX | XXXXXX | XXXXXXX |
| XXXX | n | n | XXXXX | XXXXXX | XXXXXXX |
| XXXX | n | n | XXXXX | XXXXXX | XXXXXXX |
| XXXX | n | n | XXXXX | XXXXXX | XXXXXXX |
| XXXX | n | n | XXXXX | XXXXXX | XXXXXXX |

| | | | | | |
|---|---|---|---|---|---|
| XXXX | n | n | XXXXX | XXXXXX | XXXXXXX |
| XXXX | n | n | XXXXX | XXXXXX | XXXXXXX |
| XXXX | n | n | XXXXX | XXXXXX | XXXXXXX |
| XXXX | n | n | XXXXX | XXXXXX | XXXXXXX |
| XXXX | n | n | XXXXX | XXXXXX | XXXXXXX |
| XXXX | n | n | XXXXX | XXXXXX | XXXXXXX |
| XXXX | n | n | XXXXX | XXXXXX | XXXXXXX |
| XXXX | n | n | XXXXX | XXXXXX | XXXXXXX |
| XXXX | n | n | XXXXX | XXXXXX | XXXXXXX |
| XXXX | n | n | XXXXX | XXXXXX | XXXXXXX |
| XXXX | n | n | XXXXX | XXXXXX | XXXXXXX |
| XXXX | n | n | XXXXX | XXXXXX | XXXXXXX |
| XXXX | n | n | XXXXX | XXXXXX | XXXXXXX |
| XXXX | n | n | XXXXX | XXXXXX | XXXXXXX |
| XXXX | n | n | XXXXX | XXXXXX | XXXXXXX |
| XXXX | n | n | XXXXX | XXXXXX | XXXXXXX |
| XXXX | n | n | XXXXX | XXXXXX | XXXXXXX |
| XXXX | n | n | XXXXX | XXXXXX | XXXXXXX |
| XXXX | n | n | XXXXX | XXXXXX | XXXXXXX |
| XXXX | n | n | XXXXX | XXXXXX | XXXXXXX |
| XXXX | n | n | XXXXX | XXXXXX | XXXXXXX |
| XXXX | n | n | XXXXX | XXXXXX | XXXXXXX |
| XXXX | n | n | XXXXX | XXXXXX | XXXXXXX |
| XXXX | n | n | XXXXX | XXXXXX | XXXXXXX |
| XXXX | n | n | XXXXX | XXXXXX | XXXXXXX |
| XXXX | n | n | XXXXX | XXXXXX | XXXXXXX |
| XXXX | n | n | XXXXX | XXXXXX | XXXXXXX |
| XXXX | n | n | XXXXX | XXXXXX | XXXXXXX |
| XXXX | n | n | XXXXX | XXXXXX | XXXXXXX |
| XXXX | n | n | XXXXX | XXXXXX | XXXXXXX |
| XXXX | n | n | XXXXX | XXXXXX | XXXXXXX |
| XXXX | n | n | XXXXX | XXXXXX | XXXXXXX |
| XXXX | n | n | XXXXX | XXXXXX | XXXXXXX |
| XXXX | n | n | XXXXX | XXXXXX | XXXXXXX |
| XXXX | n | n | XXXXX | XXXXXX | XXXXXXX |
| XXXX | n | n | XXXXX | XXXXXX | XXXXXXX |
| XXXX | n | n | XXXXX | XXXXXX | XXXXXXX |
| XXXX | n | n | XXXXX | XXXXXX | XXXXXXX |
| XXXX | n | n | XXXXX | XXXXXX | XXXXXXX |
| XXXX | n | n | XXXXX | XXXXXX | XXXXXXX |
| XXXX | n | n | XXXXX | XXXXXX | XXXXXXX |
| XXXX | n | n | XXXXX | XXXXXX | XXXXXXX |
| XXXX | n | n | XXXXX | XXXXXX | XXXXXXX |
| XXXX | n | n | XXXXX | XXXXXX | XXXXXXX |
| XXXX | n | n | XXXXX | XXXXXX | XXXXXXX |
| XXXX | n | n | XXXXX | XXXXXX | XXXXXXX |
| XXXX | n | n | XXXXX | XXXXXX | XXXXXXX |
| XXXX | y | n | XXXXX | XXXXXX | XXXXXXX |
| XXXX | n | n | XXXXX | XXXXXX | XXXXXXX |
| XXXX | n | n | XXXXX | XXXXXX | XXXXXXX |

| | | | | | |
|---|---|---|---|---|---|
| XXXX | n | n | XXXXX | XXXXXX | XXXXXXX |
| XXXX | n | n | XXXXX | XXXXXX | XXXXXXX |
| XXXX | n | n | XXXXX | XXXXXX | XXXXXXX |
| XXXX | n | n | XXXXX | XXXXXX | XXXXXXX |
| XXXX | n | n | XXXXX | XXXXXX | XXXXXXX |
| XXXX | n | n | XXXXX | XXXXXX | XXXXXXX |
| XXXX | n | n | XXXXX | XXXXXX | XXXXXXX |
| XXXX | n | n | XXXXX | XXXXXX | XXXXXXX |
| XXXX | n | n | XXXXX | XXXXXX | XXXXXXX |
| XXXX | n | n | XXXXX | XXXXXX | XXXXXXX |
| XXXX | n | n | XXXXX | XXXXXX | XXXXXXX |
| XXXX | n | n | XXXXX | XXXXXX | XXXXXXX |
| XXXX | n | n | XXXXX | XXXXXX | XXXXXXX |
| XXXX | n | n | XXXXX | XXXXXX | XXXXXXX |
| XXXX | n | n | XXXXX | XXXXXX | XXXXXXX |
| XXXX | n | n | XXXXX | XXXXXX | XXXXXXX |
| XXXX | n | n | XXXXX | XXXXXX | XXXXXXX |
| XXXX | n | n | XXXXX | XXXXXX | XXXXXXX |
| XXXX | n | n | XXXXX | XXXXXX | XXXXXXX |
| XXXX | n | n | XXXXX | XXXXXX | XXXXXXX |
| XXXX | n | n | XXXXX | XXXXXX | XXXXXXX |
| XXXX | n | n | XXXXX | XXXXXX | XXXXXXX |
| XXXX | n | n | XXXXX | XXXXXX | XXXXXXX |
| XXXX | n | n | XXXXX | XXXXXX | XXXXXXX |
| XXXX | n | n | XXXXX | XXXXXX | XXXXXXX |
| XXXX | n | n | XXXXX | XXXXXX | XXXXXXX |
| XXXX | n | n | XXXXX | XXXXXX | XXXXXXX |
| XXXX | n | n | XXXXX | XXXXXX | XXXXXXX |
| XXXX | n | n | XXXXX | XXXXXX | XXXXXXX |
| XXXX | n | n | XXXXX | XXXXXX | XXXXXXX |
| XXXX | n | n | XXXXX | XXXXXX | XXXXXXX |
| XXXX | n | n | XXXXX | XXXXXX | XXXXXXX |
| XXXX | n | n | XXXXX | XXXXXX | XXXXXXX |
| XXXX | n | n | XXXXX | XXXXXX | XXXXXXX |
| XXXX | n | n | XXXXX | XXXXXX | XXXXXXX |
| XXXX | n | n | XXXXX | XXXXXX | XXXXXXX |
| XXXX | n | n | XXXXX | XXXXXX | XXXXXXX |
| XXXX | n | n | XXXXX | XXXXXX | XXXXXXX |
| XXXX | n | n | XXXXX | XXXXXX | XXXXXXX |
| XXXX | n | n | XXXXX | XXXXXX | XXXXXXX |
| XXXX | n | n | XXXXX | XXXXXX | XXXXXXX |
| XXXX | n | n | XXXXX | XXXXXX | XXXXXXX |
| XXXX | n | n | XXXXX | XXXXXX | XXXXXXX |
| XXXX | n | n | XXXXX | XXXXXX | XXXXXXX |
| XXXX | n | n | XXXXX | XXXXXX | XXXXXXX |
| XXXX | n | n | XXXXX | XXXXXX | XXXXXXX |
| XXXX | n | n | XXXXX | XXXXXX | XXXXXXX |
| XXXX | n | n | XXXXX | XXXXXX | XXXXXXX |
| XXXX | n | n | XXXXX | XXXXXX | XXXXXXX |
| XXXX | n | n | XXXXX | XXXXXX | XXXXXXX |

| | | | | | |
|---|---|---|---|---|---|
| XXXX | n | n | XXXXX | XXXXXX | XXXXXXX |
| XXXX | n | n | XXXXX | XXXXXX | XXXXXXX |
| XXXX | n | n | XXXXX | XXXXXX | XXXXXXX |
| XXXX | n | n | XXXXX | XXXXXX | XXXXXXX |
| XXXX | n | n | XXXXX | XXXXXX | XXXXXXX |
| XXXX | n | n | XXXXX | XXXXXX | XXXXXXX |
| XXXX | n | n | XXXXX | XXXXXX | XXXXXXX |
| XXXX | n | n | XXXXX | XXXXXX | XXXXXXX |
| XXXX | n | n | XXXXX | XXXXXX | XXXXXXX |
| XXXX | n | n | XXXXX | XXXXXX | XXXXXXX |
| XXXX | n | n | XXXXX | XXXXXX | XXXXXXX |
| XXXX | n | n | XXXXX | XXXXXX | XXXXXXX |
| XXXX | n | n | XXXXX | XXXXXX | XXXXXXX |
| XXXX | n | n | XXXXX | XXXXXX | XXXXXXX |
| XXXX | n | n | XXXXX | XXXXXX | XXXXXXX |
| XXXX | n | n | XXXXX | XXXXXX | XXXXXXX |
| XXXX | n | n | XXXXX | XXXXXX | XXXXXXX |
| XXXX | n | n | XXXXX | XXXXXX | XXXXXXX |
| XXXX | n | n | XXXXX | XXXXXX | XXXXXXX |
| XXXX | n | n | XXXXX | XXXXXX | XXXXXXX |
| XXXX | n | n | XXXXX | XXXXXX | XXXXXXX |
| XXXX | n | n | XXXXX | XXXXXX | XXXXXXX |
| XXXX | n | n | XXXXX | XXXXXX | XXXXXXX |
| XXXX | n | n | XXXXX | XXXXXX | XXXXXXX |
| XXXX | n | n | XXXXX | XXXXXX | XXXXXXX |
| XXXX | n | n | XXXXX | XXXXXX | XXXXXXX |
| XXXX | n | n | XXXXX | XXXXXX | XXXXXXX |
| XXXX | n | n | XXXXX | XXXXXX | XXXXXXX |
| XXXX | n | n | XXXXX | XXXXXX | XXXXXXX |
| XXXX | n | n | XXXXX | XXXXXX | XXXXXXX |
| XXXX | n | n | XXXXX | XXXXXX | XXXXXXX |
| XXXX | n | n | XXXXX | XXXXXX | XXXXXXX |
| XXXX | n | n | XXXXX | XXXXXX | XXXXXXX |
| XXXX | n | n | XXXXX | XXXXXX | XXXXXXX |
| XXXX | n | n | XXXXX | XXXXXX | XXXXXXX |
| XXXX | n | n | XXXXX | XXXXXX | XXXXXXX |
| XXXX | n | n | XXXXX | XXXXXX | XXXXXXX |
| XXXX | n | n | XXXXX | XXXXXX | XXXXXXX |
| XXXX | n | n | XXXXX | XXXXXX | XXXXXXX |
| XXXX | n | n | XXXXX | XXXXXX | XXXXXXX |
| XXXX | n | n | XXXXX | XXXXXX | XXXXXXX |
| XXXX | n | n | XXXXX | XXXXXX | XXXXXXX |
| XXXX | n | n | XXXXX | XXXXXX | XXXXXXX |
| XXXX | n | n | XXXXX | XXXXXX | XXXXXXX |
| XXXX | y | n | XXXXX | XXXXXX | XXXXXXX |
| XXXX | y | n | XXXXX | XXXXXX | XXXXXXX |
| XXXX | y | n | XXXXX | XXXXXX | XXXXXXX |
| XXXX | y | n | XXXXX | XXXXXX | XXXXXXX |
| XXXX | y | n | XXXXX | XXXXXX | XXXXXXX |
| XXXX | y | n | XXXXX | XXXXXX | XXXXXXX |

| | | | | | |
|---|---|---|---|---|---|
| XXXX | y | n | XXXXX | XXXXXX | XXXXXXX |
| XXXX | y | n | XXXXX | XXXXXX | XXXXXXX |
| XXXX | y | n | XXXXX | XXXXXX | XXXXXXX |
| XXXX | y | n | XXXXX | XXXXXX | XXXXXXX |
| XXXX | y | n | XXXXX | XXXXXX | XXXXXXX |
| XXXX | y | n | XXXXX | XXXXXX | XXXXXXX |
| XXXX | y | n | XXXXX | XXXXXX | XXXXXXX |
| XXXX | y | n | XXXXX | XXXXXX | XXXXXXX |
| XXXX | y | n | XXXXX | XXXXXX | XXXXXXX |
| XXXX | y | n | XXXXX | XXXXXX | XXXXXXX |
| XXXX | y | n | XXXXX | XXXXXX | XXXXXXX |
| XXXX | y | n | XXXXX | XXXXXX | XXXXXXX |
| XXXX | y | n | XXXXX | XXXXXX | XXXXXXX |
| XXXX | y | n | XXXXX | XXXXXX | XXXXXXX |
| XXXX | y | n | XXXXX | XXXXXX | XXXXXXX |
| XXXX | y | n | XXXXX | XXXXXX | XXXXXXX |
| XXXX | y | n | XXXXX | XXXXXX | XXXXXXX |
| XXXX | y | n | XXXXX | XXXXXX | XXXXXXX |
| XXXX | y | n | XXXXX | XXXXXX | XXXXXXX |
| XXXX | y | n | XXXXX | XXXXXX | XXXXXXX |
| XXXX | y | n | XXXXX | XXXXXX | XXXXXXX |
| XXXX | y | n | XXXXX | XXXXXX | XXXXXXX |
| XXXX | y | n | XXXXX | XXXXXX | XXXXXXX |
| XXXX | na | n | XXXXX | XXXXXX | XXXXXXX |
| XXXX | na | n | XXXXX | XXXXXX | XXXXXXX |
| XXXX | na | n | XXXXX | XXXXXX | XXXXXXX |
| XXXX | na | n | XXXXX | XXXXXX | XXXXXXX |
| XXXX | na | n | XXXXX | XXXXXX | XXXXXXX |
| XXXX | na | n | XXXXX | XXXXXX | XXXXXXX |
| XXXX | na | n | XXXXX | XXXXXX | XXXXXXX |
| XXXX | na | n | XXXXX | XXXXXX | XXXXXXX |
| XXXX | na | n | XXXXX | XXXXXX | XXXXXXX |
| XXXX | na | n | XXXXX | XXXXXX | XXXXXXX |
| XXXX | na | n | XXXXX | XXXXXX | XXXXXXX |
| XXXX | na | n | XXXXX | XXXXXX | XXXXXXX |
| XXXX | na | n | XXXXX | XXXXXX | XXXXXXX |
| XXXX | n | n | XXXXX | XXXXXX | XXXXXXX |
| XXXX | n | n | XXXXX | XXXXXX | XXXXXXX |
| XXXX | n | n | XXXXX | XXXXXX | XXXXXXX |
| XXXX | y | n | XXXXX | XXXXXX | XXXXXXX |
| XXXX | y | n | XXXXX | XXXXXX | XXXXXXX |
| XXXX | y | n | XXXXX | XXXXXX | XXXXXXX |
| XXXX | y | n | XXXXX | XXXXXX | XXXXXXX |
| XXXX | y | n | XXXXX | XXXXXX | XXXXXXX |
| XXXX | y | n | XXXXX | XXXXXX | XXXXXXX |
| XXXX | y | n | XXXXX | XXXXXX | XXXXXXX |
| XXXX | y | n | XXXXX | XXXXXX | XXXXXXX |
| XXXX | y | n | XXXXX | XXXXXX | XXXXXXX |
| XXXX | y | n | XXXXX | XXXXXX | XXXXXXX |
| XXXX | y | n | XXXXX | XXXXXX | XXXXXXX |

| | | | | | |
|---|---|---|---|---|---|
| XXXX | y | n | XXXXX | XXXXXX | XXXXXXX |
| XXXX | y | n | XXXXX | XXXXXX | XXXXXXX |
| XXXX | y | n | XXXXX | XXXXXX | XXXXXXX |
| XXXX | y | n | XXXXX | XXXXXX | XXXXXXX |
| XXXX | y | n | XXXXX | XXXXXX | XXXXXXX |
| XXXX | y | n | XXXXX | XXXXXX | XXXXXXX |
| XXXX | y | n | XXXXX | XXXXXX | XXXXXXX |
| XXXX | y | n | XXXXX | XXXXXX | XXXXXXX |
| XXXX | y | n | XXXXX | XXXXXX | XXXXXXX |
| XXXX | y | n | XXXXX | XXXXXX | XXXXXXX |
| XXXX | y | n | XXXXX | XXXXXX | XXXXXXX |
| XXXX | y | n | XXXXX | XXXXXX | XXXXXXX |
| XXXX | y | n | XXXXX | XXXXXX | XXXXXXX |
| XXXX | y | n | XXXXX | XXXXXX | XXXXXXX |
| XXXX | y | n | XXXXX | XXXXXX | XXXXXXX |
| XXXX | y | n | XXXXX | XXXXXX | XXXXXXX |
| XXXX | y | n | XXXXX | XXXXXX | XXXXXXX |
| XXXX | y | n | XXXXX | XXXXXX | XXXXXXX |
| XXXX | y | n | XXXXX | XXXXXX | XXXXXXX |
| XXXX | y | n | XXXXX | XXXXXX | XXXXXXX |
| XXXX | y | n | XXXXX | XXXXXX | XXXXXXX |
| XXXX | y | n | XXXXX | XXXXXX | XXXXXXX |
| XXXX | y | n | XXXXX | XXXXXX | XXXXXXX |
| XXXX | y | n | XXXXX | XXXXXX | XXXXXXX |
| XXXX | y | n | XXXXX | XXXXXX | XXXXXXX |
| XXXX | y | n | XXXXX | XXXXXX | XXXXXXX |
| XXXX | y | n | XXXXX | XXXXXX | XXXXXXX |
| XXXX | y | n | XXXXX | XXXXXX | XXXXXXX |
| XXXX | y | n | XXXXX | XXXXXX | XXXXXXX |
| XXXX | y | n | XXXXX | XXXXXX | XXXXXXX |
| XXXX | y | n | XXXXX | XXXXXX | XXXXXXX |
| XXXX | y | n | XXXXX | XXXXXX | XXXXXXX |
| XXXX | y | n | XXXXX | XXXXXX | XXXXXXX |
| XXXX | y | n | XXXXX | XXXXXX | XXXXXXX |
| XXXX | y | n | XXXXX | XXXXXX | XXXXXXX |
| XXXX | y | n | XXXXX | XXXXXX | XXXXXXX |
| XXXX | y | n | XXXXX | XXXXXX | XXXXXXX |
| XXXX | y | n | XXXXX | XXXXXX | XXXXXXX |
| XXXX | y | n | XXXXX | XXXXXX | XXXXXXX |
| XXXX | y | n | XXXXX | XXXXXX | XXXXXXX |
| XXXX | y | n | XXXXX | XXXXXX | XXXXXXX |
| XXXX | y | n | XXXXX | XXXXXX | XXXXXXX |
| XXXX | y | n | XXXXX | XXXXXX | XXXXXXX |
| XXXX | y | n | XXXXX | XXXXXX | XXXXXXX |
| XXXX | y | n | XXXXX | XXXXXX | XXXXXXX |
| XXXX | y | n | XXXXX | XXXXXX | XXXXXXX |
| XXXX | y | n | XXXXX | XXXXXX | XXXXXXX |
| XXXX | y | n | XXXXX | XXXXXX | XXXXXXX |
| XXXX | y | n | XXXXX | XXXXXX | XXXXXXX |
| XXXX | y | n | XXXXX | XXXXXX | XXXXXXX |

| | | | | | |
|---|---|---|---|---|---|
| XXXX | y | n | XXXXX | XXXXXX | XXXXXXX |
| XXXX | y | n | XXXXX | XXXXXX | XXXXXXX |
| XXXX | y | n | XXXXX | XXXXXX | XXXXXXX |
| XXXX | y | n | XXXXX | XXXXXX | XXXXXXX |
| XXXX | y | n | XXXXX | XXXXXX | XXXXXXX |
| XXXX | y | n | XXXXX | XXXXXX | XXXXXXX |
| XXXX | y | n | XXXXX | XXXXXX | XXXXXXX |
| XXXX | y | n | XXXXX | XXXXXX | XXXXXXX |
| XXXX | y | n | XXXXX | XXXXXX | XXXXXXX |
| XXXX | y | n | XXXXX | XXXXXX | XXXXXXX |
| XXXX | y | n | XXXXX | XXXXXX | XXXXXXX |
| XXXX | y | n | XXXXX | XXXXXX | XXXXXXX |
| XXXX | y | n | XXXXX | XXXXXX | XXXXXXX |
| XXXX | y | n | XXXXX | XXXXXX | XXXXXXX |
| XXXX | y | n | XXXXX | XXXXXX | XXXXXXX |
| XXXX | y | n | XXXXX | XXXXXX | XXXXXXX |
| XXXX | y | n | XXXXX | XXXXXX | XXXXXXX |
| XXXX | y | n | XXXXX | XXXXXX | XXXXXXX |
| XXXX | y | n | XXXXX | XXXXXX | XXXXXXX |
| XXXX | y | n | XXXXX | XXXXXX | XXXXXXX |
| XXXX | y | n | XXXXX | XXXXXX | XXXXXXX |
| XXXX | y | n | XXXXX | XXXXXX | XXXXXXX |
| XXXX | y | n | XXXXX | XXXXXX | XXXXXXX |
| XXXX | y | n | XXXXX | XXXXXX | XXXXXXX |
| XXXX | y | n | XXXXX | XXXXXX | XXXXXXX |
| XXXX | y | n | XXXXX | XXXXXX | XXXXXXX |
| XXXX | y | n | XXXXX | XXXXXX | XXXXXXX |
| XXXX | y | n | XXXXX | XXXXXX | XXXXXXX |
| XXXX | y | n | XXXXX | XXXXXX | XXXXXXX |
| XXXX | y | n | XXXXX | XXXXXX | XXXXXXX |
| XXXX | y | n | XXXXX | XXXXXX | XXXXXXX |
| XXXX | y | n | XXXXX | XXXXXX | XXXXXXX |
| XXXX | y | n | XXXXX | XXXXXX | XXXXXXX |
| XXXX | y | n | XXXXX | XXXXXX | XXXXXXX |
| XXXX | y | n | XXXXX | XXXXXX | XXXXXXX |
| XXXX | y | n | XXXXX | XXXXXX | XXXXXXX |
| XXXX | y | n | XXXXX | XXXXXX | XXXXXXX |
| XXXX | y | n | XXXXX | XXXXXX | XXXXXXX |
| XXXX | y | n | XXXXX | XXXXXX | XXXXXXX |
| XXXX | y | n | XXXXX | XXXXXX | XXXXXXX |
| XXXX | y | n | XXXXX | XXXXXX | XXXXXXX |
| XXXX | y | n | XXXXX | XXXXXX | XXXXXXX |
| XXXX | y | n | XXXXX | XXXXXX | XXXXXXX |
| XXXX | y | n | XXXXX | XXXXXX | XXXXXXX |
| XXXX | y | n | XXXXX | XXXXXX | XXXXXXX |
| XXXX | y | n | XXXXX | XXXXXX | XXXXXXX |
| XXXX | y | n | XXXXX | XXXXXX | XXXXXXX |
| XXXX | y | n | XXXXX | XXXXXX | XXXXXXX |
| XXXX | y | n | XXXXX | XXXXXX | XXXXXXX |
| XXXX | y | n | XXXXX | XXXXXX | XXXXXXX |

| | | | | | |
|---|---|---|---|---|---|
| XXXX | y | n | XXXXX | XXXXXX | XXXXXXX |
| XXXX | y | n | XXXXX | XXXXXX | XXXXXXX |
| XXXX | y | n | XXXXX | XXXXXX | XXXXXXX |
| XXXX | y | n | XXXXX | XXXXXX | XXXXXXX |
| XXXX | y | n | XXXXX | XXXXXX | XXXXXXX |
| XXXX | y | n | XXXXX | XXXXXX | XXXXXXX |
| XXXX | y | n | XXXXX | XXXXXX | XXXXXXX |
| XXXX | y | n | XXXXX | XXXXXX | XXXXXXX |
| XXXX | y | n | XXXXX | XXXXXX | XXXXXXX |
| XXXX | y | n | XXXXX | XXXXXX | XXXXXXX |
| XXXX | y | n | XXXXX | XXXXXX | XXXXXXX |
| XXXX | y | n | XXXXX | XXXXXX | XXXXXXX |
| XXXX | y | n | XXXXX | XXXXXX | XXXXXXX |
| XXXX | y | n | XXXXX | XXXXXX | XXXXXXX |
| XXXX | y | n | XXXXX | XXXXXX | XXXXXXX |
| XXXX | y | n | XXXXX | XXXXXX | XXXXXXX |
| XXXX | y | n | XXXXX | XXXXXX | XXXXXXX |
| XXXX | y | n | XXXXX | XXXXXX | XXXXXXX |
| XXXX | y | n | XXXXX | XXXXXX | XXXXXXX |
| XXXX | y | n | XXXXX | XXXXXX | XXXXXXX |
| XXXX | y | n | XXXXX | XXXXXX | XXXXXXX |
| XXXX | y | n | XXXXX | XXXXXX | XXXXXXX |
| XXXX | y | n | XXXXX | XXXXXX | XXXXXXX |
| XXXX | y | n | XXXXX | XXXXXX | XXXXXXX |
| XXXX | y | n | XXXXX | XXXXXX | XXXXXXX |
| XXXX | y | n | XXXXX | XXXXXX | XXXXXXX |
| XXXX | y | n | XXXXX | XXXXXX | XXXXXXX |
| XXXX | y | n | XXXXX | XXXXXX | XXXXXXX |
| XXXX | y | n | XXXXX | XXXXXX | XXXXXXX |
| XXXX | y | n | XXXXX | XXXXXX | XXXXXXX |
| XXXX | y | n | XXXXX | XXXXXX | XXXXXXX |
| XXXX | y | n | XXXXX | XXXXXX | XXXXXXX |
| XXXX | y | n | XXXXX | XXXXXX | XXXXXXX |
| XXXX | y | n | XXXXX | XXXXXX | XXXXXXX |
| XXXX | y | n | XXXXX | XXXXXX | XXXXXXX |
| XXXX | y | n | XXXXX | XXXXXX | XXXXXXX |
| XXXX | y | n | XXXXX | XXXXXX | XXXXXXX |
| XXXX | y | n | XXXXX | XXXXXX | XXXXXXX |
| XXXX | y | n | XXXXX | XXXXXX | XXXXXXX |
| XXXX | y | n | XXXXX | XXXXXX | XXXXXXX |
| XXXX | y | n | XXXXX | XXXXXX | XXXXXXX |
| XXXX | y | n | XXXXX | XXXXXX | XXXXXXX |
| XXXX | y | n | XXXXX | XXXXXX | XXXXXXX |
| XXXX | y | n | XXXXX | XXXXXX | XXXXXXX |
| XXXX | y | n | XXXXX | XXXXXX | XXXXXXX |
| XXXX | y | n | XXXXX | XXXXXX | XXXXXXX |
| XXXX | y | n | XXXXX | XXXXXX | XXXXXXX |
| XXXX | y | n | XXXXX | XXXXXX | XXXXXXX |
| XXXX | y | n | XXXXX | XXXXXX | XXXXXXX |
| XXXX | y | n | XXXXX | XXXXXX | XXXXXXX |

| | | | | | |
|---|---|---|---|---|---|
| XXXX | y | n | XXXXX | XXXXXX | XXXXXXX |
| XXXX | y | n | XXXXX | XXXXXX | XXXXXXX |
| XXXX | y | n | XXXXX | XXXXXX | XXXXXXX |
| XXXX | y | n | XXXXX | XXXXXX | XXXXXXX |
| XXXX | y | n | XXXXX | XXXXXX | XXXXXXX |
| XXXX | y | n | XXXXX | XXXXXX | XXXXXXX |
| XXXX | y | n | XXXXX | XXXXXX | XXXXXXX |
| XXXX | y | n | XXXXX | XXXXXX | XXXXXXX |
| XXXX | y | n | XXXXX | XXXXXX | XXXXXXX |
| XXXX | y | n | XXXXX | XXXXXX | XXXXXXX |
| XXXX | y | n | XXXXX | XXXXXX | XXXXXXX |
| XXXX | y | n | XXXXX | XXXXXX | XXXXXXX |
| XXXX | y | n | XXXXX | XXXXXX | XXXXXXX |
| XXXX | y | n | XXXXX | XXXXXX | XXXXXXX |
| XXXX | y | n | XXXXX | XXXXXX | XXXXXXX |
| XXXX | y | n | XXXXX | XXXXXX | XXXXXXX |
| XXXX | y | n | XXXXX | XXXXXX | XXXXXXX |
| XXXX | y | n | XXXXX | XXXXXX | XXXXXXX |
| XXXX | y | n | XXXXX | XXXXXX | XXXXXXX |
| XXXX | y | n | XXXXX | XXXXXX | XXXXXXX |
| XXXX | y | n | XXXXX | XXXXXX | XXXXXXX |
| XXXX | y | n | XXXXX | XXXXXX | XXXXXXX |
| XXXX | y | n | XXXXX | XXXXXX | XXXXXXX |
| XXXX | y | n | XXXXX | XXXXXX | XXXXXXX |
| XXXX | y | n | XXXXX | XXXXXX | XXXXXXX |
| XXXX | y | n | XXXXX | XXXXXX | XXXXXXX |
| XXXX | y | n | XXXXX | XXXXXX | XXXXXXX |
| XXXX | y | n | XXXXX | XXXXXX | XXXXXXX |
| XXXX | y | n | XXXXX | XXXXXX | XXXXXXX |
| XXXX | y | n | XXXXX | XXXXXX | XXXXXXX |
| XXXX | y | n | XXXXX | XXXXXX | XXXXXXX |
| XXXX | y | n | XXXXX | XXXXXX | XXXXXXX |
| XXXX | y | n | XXXXX | XXXXXX | XXXXXXX |
| XXXX | y | n | XXXXX | XXXXXX | XXXXXXX |
| XXXX | y | n | XXXXX | XXXXXX | XXXXXXX |
| XXXX | y | n | XXXXX | XXXXXX | XXXXXXX |
| XXXX | y | n | XXXXX | XXXXXX | XXXXXXX |
| XXXX | y | n | XXXXX | XXXXXX | XXXXXXX |
| XXXX | y | n | XXXXX | XXXXXX | XXXXXXX |
| XXXX | y | n | XXXXX | XXXXXX | XXXXXXX |
| XXXX | y | n | XXXXX | XXXXXX | XXXXXXX |
| XXXX | y | n | XXXXX | XXXXXX | XXXXXXX |
| XXXX | y | n | XXXXX | XXXXXX | XXXXXXX |
| XXXX | y | n | XXXXX | XXXXXX | XXXXXXX |
| XXXX | y | n | XXXXX | XXXXXX | XXXXXXX |
| XXXX | y | n | XXXXX | XXXXXX | XXXXXXX |
| XXXX | y | n | XXXXX | XXXXXX | XXXXXXX |
| XXXX | y | n | XXXXX | XXXXXX | XXXXXXX |
| XXXX | y | n | XXXXX | XXXXXX | XXXXXXX |
| XXXX | y | n | XXXXX | XXXXXX | XXXXXXX |

| | | | | | |
|---|---|---|---|---|---|
| XXXX | y | n | XXXXX | XXXXXX | XXXXXXX |
| XXXX | y | n | XXXXX | XXXXXX | XXXXXXX |
| XXXX | y | n | XXXXX | XXXXXX | XXXXXXX |
| XXXX | y | n | XXXXX | XXXXXX | XXXXXXX |
| XXXX | y | n | XXXXX | XXXXXX | XXXXXXX |
| XXXX | y | n | XXXXX | XXXXXX | XXXXXXX |
| XXXX | y | n | XXXXX | XXXXXX | XXXXXXX |
| XXXX | y | n | XXXXX | XXXXXX | XXXXXXX |
| XXXX | y | n | XXXXX | XXXXXX | XXXXXXX |
| XXXX | y | n | XXXXX | XXXXXX | XXXXXXX |
| XXXX | y | n | XXXXX | XXXXXX | XXXXXXX |
| XXXX | y | n | XXXXX | XXXXXX | XXXXXXX |
| XXXX | y | n | XXXXX | XXXXXX | XXXXXXX |
| XXXX | y | n | XXXXX | XXXXXX | XXXXXXX |
| XXXX | y | n | XXXXX | XXXXXX | XXXXXXX |
| XXXX | y | n | XXXXX | XXXXXX | XXXXXXX |
| XXXX | y | n | XXXXX | XXXXXX | XXXXXXX |
| XXXX | y | n | XXXXX | XXXXXX | XXXXXXX |
| XXXX | y | n | XXXXX | XXXXXX | XXXXXXX |
| XXXX | y | n | XXXXX | XXXXXX | XXXXXXX |
| XXXX | y | n | XXXXX | XXXXXX | XXXXXXX |
| XXXX | y | n | XXXXX | XXXXXX | XXXXXXX |
| XXXX | y | n | XXXXX | XXXXXX | XXXXXXX |
| XXXX | y | n | XXXXX | XXXXXX | XXXXXXX |
| XXXX | y | n | XXXXX | XXXXXX | XXXXXXX |
| XXXX | y | n | XXXXX | XXXXXX | XXXXXXX |
| XXXX | y | n | XXXXX | XXXXXX | XXXXXXX |
| XXXX | y | n | XXXXX | XXXXXX | XXXXXXX |
| XXXX | y | n | XXXXX | XXXXXX | XXXXXXX |
| XXXX | y | n | XXXXX | XXXXXX | XXXXXXX |
| XXXX | y | n | XXXXX | XXXXXX | XXXXXXX |
| XXXX | y | n | XXXXX | XXXXXX | XXXXXXX |
| XXXX | y | n | XXXXX | XXXXXX | XXXXXXX |
| XXXX | y | n | XXXXX | XXXXXX | XXXXXXX |
| XXXX | y | n | XXXXX | XXXXXX | XXXXXXX |
| XXXX | y | n | XXXXX | XXXXXX | XXXXXXX |
| XXXX | y | n | XXXXX | XXXXXX | XXXXXXX |
| XXXX | y | n | XXXXX | XXXXXX | XXXXXXX |
| XXXX | y | n | XXXXX | XXXXXX | XXXXXXX |
| XXXX | y | n | XXXXX | XXXXXX | XXXXXXX |
| XXXX | y | n | XXXXX | XXXXXX | XXXXXXX |
| XXXX | y | n | XXXXX | XXXXXX | XXXXXXX |
| XXXX | y | n | XXXXX | XXXXXX | XXXXXXX |
| XXXX | y | n | XXXXX | XXXXXX | XXXXXXX |
| XXXX | y | n | XXXXX | XXXXXX | XXXXXXX |
| XXXX | y | n | XXXXX | XXXXXX | XXXXXXX |
| XXXX | y | n | XXXXX | XXXXXX | XXXXXXX |
| XXXX | y | n | XXXXX | XXXXXX | XXXXXXX |
| XXXX | y | n | XXXXX | XXXXXX | XXXXXXX |
| XXXX | y | n | XXXXX | XXXXXX | XXXXXXX |

| | | | | | |
|---|---|---|---|---|---|
| XXXX | y | n | XXXXX | XXXXXX | XXXXXXX |
| XXXX | y | n | XXXXX | XXXXXX | XXXXXXX |
| XXXX | y | n | XXXXX | XXXXXX | XXXXXXX |
| XXXX | y | n | XXXXX | XXXXXX | XXXXXXX |
| XXXX | y | n | XXXXX | XXXXXX | XXXXXXX |
| XXXX | y | n | XXXXX | XXXXXX | XXXXXXX |
| XXXX | y | n | XXXXX | XXXXXX | XXXXXXX |
| XXXX | y | n | XXXXX | XXXXXX | XXXXXXX |
| XXXX | y | n | XXXXX | XXXXXX | XXXXXXX |
| XXXX | y | n | XXXXX | XXXXXX | XXXXXXX |
| XXXX | y | n | XXXXX | XXXXXX | XXXXXXX |
| XXXX | y | n | XXXXX | XXXXXX | XXXXXXX |
| XXXX | y | n | XXXXX | XXXXXX | XXXXXXX |
| XXXX | y | n | XXXXX | XXXXXX | XXXXXXX |
| XXXX | y | n | XXXXX | XXXXXX | XXXXXXX |
| XXXX | y | n | XXXXX | XXXXXX | XXXXXXX |
| XXXX | y | n | XXXXX | XXXXXX | XXXXXXX |
| XXXX | y | n | XXXXX | XXXXXX | XXXXXXX |
| XXXX | y | n | XXXXX | XXXXXX | XXXXXXX |
| XXXX | y | n | XXXXX | XXXXXX | XXXXXXX |
| XXXX | y | n | XXXXX | XXXXXX | XXXXXXX |
| XXXX | y | n | XXXXX | XXXXXX | XXXXXXX |
| XXXX | y | n | XXXXX | XXXXXX | XXXXXXX |
| XXXX | y | n | XXXXX | XXXXXX | XXXXXXX |
| XXXX | y | n | XXXXX | XXXXXX | XXXXXXX |
| XXXX | y | n | XXXXX | XXXXXX | XXXXXXX |
| XXXX | y | n | XXXXX | XXXXXX | XXXXXXX |
| XXXX | y | n | XXXXX | XXXXXX | XXXXXXX |
| XXXX | y | n | XXXXX | XXXXXX | XXXXXXX |
| XXXX | y | n | XXXXX | XXXXXX | XXXXXXX |
| XXXX | y | n | XXXXX | XXXXXX | XXXXXXX |
| XXXX | y | n | XXXXX | XXXXXX | XXXXXXX |
| XXXX | y | n | XXXXX | XXXXXX | XXXXXXX |
| XXXX | y | n | XXXXX | XXXXXX | XXXXXXX |
| XXXX | y | n | XXXXX | XXXXXX | XXXXXXX |
| XXXX | y | n | XXXXX | XXXXXX | XXXXXXX |
| XXXX | y | n | XXXXX | XXXXXX | XXXXXXX |
| XXXX | y | n | XXXXX | XXXXXX | XXXXXXX |
| XXXX | y | n | XXXXX | XXXXXX | XXXXXXX |
| XXXX | y | n | XXXXX | XXXXXX | XXXXXXX |
| XXXX | y | n | XXXXX | XXXXXX | XXXXXXX |
| XXXX | y | n | XXXXX | XXXXXX | XXXXXXX |
| XXXX | y | n | XXXXX | XXXXXX | XXXXXXX |
| XXXX | y | n | XXXXX | XXXXXX | XXXXXXX |
| XXXX | y | n | XXXXX | XXXXXX | XXXXXXX |
| XXXX | y | n | XXXXX | XXXXXX | XXXXXXX |
| XXXX | y | n | XXXXX | XXXXXX | XXXXXXX |
| XXXX | y | n | XXXXX | XXXXXX | XXXXXXX |
| XXXX | y | n | XXXXX | XXXXXX | XXXXXXX |
| XXXX | y | n | XXXXX | XXXXXX | XXXXXXX |

| | | | | | |
|---|---|---|---|---|---|
| XXXX | y | n | XXXXX | XXXXXX | XXXXXXX |
| XXXX | y | n | XXXXX | XXXXXX | XXXXXXX |
| XXXX | y | n | XXXXX | XXXXXX | XXXXXXX |
| XXXX | y | n | XXXXX | XXXXXX | XXXXXXX |
| XXXX | y | n | XXXXX | XXXXXX | XXXXXXX |
| XXXX | y | n | XXXXX | XXXXXX | XXXXXXX |
| XXXX | y | n | XXXXX | XXXXXX | XXXXXXX |
| XXXX | y | n | XXXXX | XXXXXX | XXXXXXX |
| XXXX | y | n | XXXXX | XXXXXX | XXXXXXX |
| XXXX | y | n | XXXXX | XXXXXX | XXXXXXX |
| XXXX | y | n | XXXXX | XXXXXX | XXXXXXX |
| XXXX | y | n | XXXXX | XXXXXX | XXXXXXX |
| XXXX | y | n | XXXXX | XXXXXX | XXXXXXX |
| XXXX | y | n | XXXXX | XXXXXX | XXXXXXX |
| XXXX | y | n | XXXXX | XXXXXX | XXXXXXX |
| XXXX | y | n | XXXXX | XXXXXX | XXXXXXX |
| XXXX | y | n | XXXXX | XXXXXX | XXXXXXX |
| XXXX | y | n | XXXXX | XXXXXX | XXXXXXX |
| XXXX | y | n | XXXXX | XXXXXX | XXXXXXX |
| XXXX | y | n | XXXXX | XXXXXX | XXXXXXX |
| XXXX | y | n | XXXXX | XXXXXX | XXXXXXX |
| XXXX | y | n | XXXXX | XXXXXX | XXXXXXX |
| XXXX | y | n | XXXXX | XXXXXX | XXXXXXX |
| XXXX | y | n | XXXXX | XXXXXX | XXXXXXX |
| XXXX | y | n | XXXXX | XXXXXX | XXXXXXX |
| XXXX | y | n | XXXXX | XXXXXX | XXXXXXX |
| XXXX | y | n | XXXXX | XXXXXX | XXXXXXX |
| XXXX | y | n | XXXXX | XXXXXX | XXXXXXX |
| XXXX | y | n | XXXXX | XXXXXX | XXXXXXX |
| XXXX | y | n | XXXXX | XXXXXX | XXXXXXX |
| XXXX | y | n | XXXXX | XXXXXX | XXXXXXX |
| XXXX | y | n | XXXXX | XXXXXX | XXXXXXX |
| XXXX | y | n | XXXXX | XXXXXX | XXXXXXX |
| XXXX | y | n | XXXXX | XXXXXX | XXXXXXX |
| XXXX | y | n | XXXXX | XXXXXX | XXXXXXX |
| XXXX | y | n | XXXXX | XXXXXX | XXXXXXX |
| XXXX | y | n | XXXXX | XXXXXX | XXXXXXX |
| XXXX | y | n | XXXXX | XXXXXX | XXXXXXX |
| XXXX | y | n | XXXXX | XXXXXX | XXXXXXX |
| XXXX | y | n | XXXXX | XXXXXX | XXXXXXX |
| XXXX | y | n | XXXXX | XXXXXX | XXXXXXX |
| XXXX | y | n | XXXXX | XXXXXX | XXXXXXX |
| XXXX | y | n | XXXXX | XXXXXX | XXXXXXX |
| XXXX | y | n | XXXXX | XXXXXX | XXXXXXX |
| XXXX | y | n | XXXXX | XXXXXX | XXXXXXX |
| XXXX | y | n | XXXXX | XXXXXX | XXXXXXX |
| XXXX | y | n | XXXXX | XXXXXX | XXXXXXX |
| XXXX | y | n | XXXXX | XXXXXX | XXXXXXX |
| XXXX | y | n | XXXXX | XXXXXX | XXXXXXX |
| XXXX | y | n | XXXXX | XXXXXX | XXXXXXX |

| | | | | | |
|---|---|---|---|---|---|
| XXXX | y | n | XXXXX | XXXXXX | XXXXXXX |
| XXXX | y | n | XXXXX | XXXXXX | XXXXXXX |
| XXXX | y | n | XXXXX | XXXXXX | XXXXXXX |
| XXXX | y | n | XXXXX | XXXXXX | XXXXXXX |
| XXXX | y | n | XXXXX | XXXXXX | XXXXXXX |
| XXXX | y | n | XXXXX | XXXXXX | XXXXXXX |
| XXXX | y | n | XXXXX | XXXXXX | XXXXXXX |
| XXXX | y | n | XXXXX | XXXXXX | XXXXXXX |
| XXXX | y | n | XXXXX | XXXXXX | XXXXXXX |
| XXXX | y | n | XXXXX | XXXXXX | XXXXXXX |
| XXXX | y | n | XXXXX | XXXXXX | XXXXXXX |
| XXXX | y | n | XXXXX | XXXXXX | XXXXXXX |
| XXXX | y | n | XXXXX | XXXXXX | XXXXXXX |
| XXXX | y | n | XXXXX | XXXXXX | XXXXXXX |
| XXXX | y | n | XXXXX | XXXXXX | XXXXXXX |
| XXXX | y | n | XXXXX | XXXXXX | XXXXXXX |
| XXXX | y | n | XXXXX | XXXXXX | XXXXXXX |
| XXXX | y | n | XXXXX | XXXXXX | XXXXXXX |
| XXXX | y | n | XXXXX | XXXXXX | XXXXXXX |
| XXXX | y | n | XXXXX | XXXXXX | XXXXXXX |
| XXXX | y | n | XXXXX | XXXXXX | XXXXXXX |
| XXXX | y | n | XXXXX | XXXXXX | XXXXXXX |
| XXXX | y | n | XXXXX | XXXXXX | XXXXXXX |
| XXXX | y | n | XXXXX | XXXXXX | XXXXXXX |
| XXXX | na | n | XXXXX | XXXXXX | XXXXXXX |
| XXXX | na | n | XXXXX | XXXXXX | XXXXXXX |
| XXXX | na | n | XXXXX | XXXXXX | XXXXXXX |
| XXXX | na | n | XXXXX | XXXXXX | XXXXXXX |
| XXXX | na | n | XXXXX | XXXXXX | XXXXXXX |
| XXXX | na | n | XXXXX | XXXXXX | XXXXXXX |
| XXXX | na | n | XXXXX | XXXXXX | XXXXXXX |
| XXXX | na | n | XXXXX | XXXXXX | XXXXXXX |
| XXXX | na | n | XXXXX | XXXXXX | XXXXXXX |
| XXXX | na | n | XXXXX | XXXXXX | XXXXXXX |
| XXXX | na | n | XXXXX | XXXXXX | XXXXXXX |
| XXXX | na | n | XXXXX | XXXXXX | XXXXXXX |
| XXXX | na | n | XXXXX | XXXXXX | XXXXXXX |
| XXXX | na | n | XXXXX | XXXXXX | XXXXXXX |
| XXXX | na | n | XXXXX | XXXXXX | XXXXXXX |
| XXXX | na | n | XXXXX | XXXXXX | XXXXXXX |
| XXXX | y | n | XXXXX | XXXXXX | XXXXXXX |
| XXXX | y | n | XXXXX | XXXXXX | XXXXXXX |
| XXXX | y | n | XXXXX | XXXXXX | XXXXXXX |
| XXXX | y | n | XXXXX | XXXXXX | XXXXXXX |
| XXXX | y | n | XXXXX | XXXXXX | XXXXXXX |
| XXXX | y | n | XXXXX | XXXXXX | XXXXXXX |
| XXXX | y | n | XXXXX | XXXXXX | XXXXXXX |
| XXXX | y | n | XXXXX | XXXXXX | XXXXXXX |
| XXXX | y | n | XXXXX | XXXXXX | XXXXXXX |
| XXXX | y | n | XXXXX | XXXXXX | XXXXXXX |

| | | | | | |
|---|---|---|---|---|---|
| XXXX | y | n | XXXXX | XXXXXX | XXXXXXX |
| XXXX | y | n | XXXXX | XXXXXX | XXXXXXX |
| XXXX | y | n | XXXXX | XXXXXX | XXXXXXX |
| XXXX | y | n | XXXXX | XXXXXX | XXXXXXX |
| XXXX | y | n | XXXXX | XXXXXX | XXXXXXX |
| XXXX | y | n | XXXXX | XXXXXX | XXXXXXX |
| XXXX | y | n | XXXXX | XXXXXX | XXXXXXX |
| XXXX | y | n | XXXXX | XXXXXX | XXXXXXX |
| XXXX | y | n | XXXXX | XXXXXX | XXXXXXX |
| XXXX | y | n | XXXXX | XXXXXX | XXXXXXX |
| XXXX | y | n | XXXXX | XXXXXX | XXXXXXX |
| XXXX | y | n | XXXXX | XXXXXX | XXXXXXX |
| XXXX | y | n | XXXXX | XXXXXX | XXXXXXX |
| XXXX | y | n | XXXXX | XXXXXX | XXXXXXX |
| XXXX | y | n | XXXXX | XXXXXX | XXXXXXX |
| XXXX | y | n | XXXXX | XXXXXX | XXXXXXX |
| XXXX | y | n | XXXXX | XXXXXX | XXXXXXX |
| XXXX | y | n | XXXXX | XXXXXX | XXXXXXX |
| XXXX | y | n | XXXXX | XXXXXX | XXXXXXX |
| XXXX | y | n | XXXXX | XXXXXX | XXXXXXX |
| XXXX | y | n | XXXXX | XXXXXX | XXXXXXX |
| XXXX | y | n | XXXXX | XXXXXX | XXXXXXX |
| XXXX | y | n | XXXXX | XXXXXX | XXXXXXX |
| XXXX | y | n | XXXXX | XXXXXX | XXXXXXX |
| XXXX | y | n | XXXXX | XXXXXX | XXXXXXX |
| XXXX | y | n | XXXXX | XXXXXX | XXXXXXX |
| XXXX | y | n | XXXXX | XXXXXX | XXXXXXX |
| XXXX | y | n | XXXXX | XXXXXX | XXXXXXX |
| XXXX | y | n | XXXXX | XXXXXX | XXXXXXX |
| XXXX | y | n | XXXXX | XXXXXX | XXXXXXX |
| XXXX | y | n | XXXXX | XXXXXX | XXXXXXX |
| XXXX | y | n | XXXXX | XXXXXX | XXXXXXX |
| XXXX | y | n | XXXXX | XXXXXX | XXXXXXX |
| XXXX | y | n | XXXXX | XXXXXX | XXXXXXX |
| XXXX | y | n | XXXXX | XXXXXX | XXXXXXX |
| XXXX | y | n | XXXXX | XXXXXX | XXXXXXX |
| XXXX | y | n | XXXXX | XXXXXX | XXXXXXX |
| XXXX | y | y | XXXXX | XXXXXX | XXXXXXX |
| XXXX | y | y | XXXXX | XXXXXX | XXXXXXX |
| XXXX | y | y | XXXXX | XXXXXX | XXXXXXX |
| XXXX | y | y | XXXXX | XXXXXX | XXXXXXX |
| XXXX | y | y | XXXXX | XXXXXX | XXXXXXX |
| XXXX | y | y | XXXXX | XXXXXX | XXXXXXX |
| XXXX | y | y | XXXXX | XXXXXX | XXXXXXX |
| XXXX | y | y | XXXXX | XXXXXX | XXXXXXX |
| XXXX | y | y | XXXXX | XXXXXX | XXXXXXX |
| XXXX | y | y | XXXXX | XXXXXX | XXXXXXX |
| XXXX | y | y | XXXXX | XXXXXX | XXXXXXX |
| XXXX | y | y | XXXXX | XXXXXX | XXXXXXX |
| XXXX | y | y | XXXXX | XXXXXX | XXXXXXX |

| | | | | | |
|---|---|---|---|---|---|
| XXXX | y | y | XXXXX | XXXXXX | XXXXXXX |
| XXXX | y | y | XXXXX | XXXXXX | XXXXXXX |
| XXXX | y | y | XXXXX | XXXXXX | XXXXXXX |
| XXXX | y | y | XXXXX | XXXXXX | XXXXXXX |
| XXXX | y | y | XXXXX | XXXXXX | XXXXXXX |
| XXXX | y | y | XXXXX | XXXXXX | XXXXXXX |
| XXXX | y | y | XXXXX | XXXXXX | XXXXXXX |
| XXXX | y | y | XXXXX | XXXXXX | XXXXXXX |
| XXXX | y | y | XXXXX | XXXXXX | XXXXXXX |
| XXXX | y | y | XXXXX | XXXXXX | XXXXXXX |
| XXXX | y | y | XXXXX | XXXXXX | XXXXXXX |
| XXXX | y | y | XXXXX | XXXXXX | XXXXXXX |
| XXXX | y | y | XXXXX | XXXXXX | XXXXXXX |
| XXXX | y | y | XXXXX | XXXXXX | XXXXXXX |
| XXXX | y | y | XXXXX | XXXXXX | XXXXXXX |
| XXXX | y | y | XXXXX | XXXXXX | XXXXXXX |
| XXXX | y | y | XXXXX | XXXXXX | XXXXXXX |
| XXXX | y | y | XXXXX | XXXXXX | XXXXXXX |
| XXXX | y | y | XXXXX | XXXXXX | XXXXXXX |
| XXXX | y | y | XXXXX | XXXXXX | XXXXXXX |
| XXXX | y | y | XXXXX | XXXXXX | XXXXXXX |
| XXXX | y | y | XXXXX | XXXXXX | XXXXXXX |
| XXXX | y | y | XXXXX | XXXXXX | XXXXXXX |
| XXXX | y | y | XXXXX | XXXXXX | XXXXXXX |
| XXXX | y | y | XXXXX | XXXXXX | XXXXXXX |
| XXXX | y | y | XXXXX | XXXXXX | XXXXXXX |
| XXXX | y | n | XXXXX | XXXXXX | XXXXXXX |
| XXXX | y | y | XXXXX | XXXXXX | XXXXXXX |
| XXXX | y | y | XXXXX | XXXXXX | XXXXXXX |
| XXXX | y | y | XXXXX | XXXXXX | XXXXXXX |
| XXXX | y | y | XXXXX | XXXXXX | XXXXXXX |
| XXXX | y | n | XXXXX | XXXXXX | XXXXXXX |
| XXXX | y | y | XXXXX | XXXXXX | XXXXXXX |
| XXXX | y | y | XXXXX | XXXXXX | XXXXXXX |
| XXXX | y | y | XXXXX | XXXXXX | XXXXXXX |
| XXXX | y | y | XXXXX | XXXXXX | XXXXXXX |
| XXXX | y | y | XXXXX | XXXXXX | XXXXXXX |
| XXXX | y | n | XXXXX | XXXXXX | XXXXXXX |
| XXXX | y | n | XXXXX | XXXXXX | XXXXXXX |
| XXXX | y | n | XXXXX | XXXXXX | XXXXXXX |
| XXXX | y | n | XXXXX | XXXXXX | XXXXXXX |
| XXXX | y | n | XXXXX | XXXXXX | XXXXXXX |
| XXXX | y | n | XXXXX | XXXXXX | XXXXXXX |
| XXXX | y | n | XXXXX | XXXXXX | XXXXXXX |
| XXXX | y | n | XXXXX | XXXXXX | XXXXXXX |
| XXXX | y | n | XXXXX | XXXXXX | XXXXXXX |
| XXXX | y | n | XXXXX | XXXXXX | XXXXXXX |
| XXXX | y | n | XXXXX | XXXXXX | XXXXXXX |
| XXXX | y | n | XXXXX | XXXXXX | XXXXXXX |
| XXXX | y | n | XXXXX | XXXXXX | XXXXXXX |

| | | | | | |
|---|---|---|---|---|---|
| XXXX | y | n | XXXXX | XXXXXX | XXXXXXX |
| XXXX | y | y | XXXXX | XXXXXX | XXXXXXX |
| XXXX | y | y | XXXXX | XXXXXX | XXXXXXX |
| XXXX | y | n | XXXXX | XXXXXX | XXXXXXX |
| XXXX | y | y | XXXXX | XXXXXX | XXXXXXX |
| XXXX | y | y | XXXXX | XXXXXX | XXXXXXX |
| XXXX | y | y | XXXXX | XXXXXX | XXXXXXX |
| XXXX | y | n | XXXXX | XXXXXX | XXXXXXX |
| XXXX | y | n | XXXXX | XXXXXX | XXXXXXX |
| XXXX | y | n | XXXXX | XXXXXX | XXXXXXX |
| XXXX | y | n | XXXXX | XXXXXX | XXXXXXX |
| XXXX | y | n | XXXXX | XXXXXX | XXXXXXX |
| XXXX | y | n | XXXXX | XXXXXX | XXXXXXX |
| XXXX | y | n | XXXXX | XXXXXX | XXXXXXX |
| XXXX | y | n | XXXXX | XXXXXX | XXXXXXX |
| XXXX | y | n | XXXXX | XXXXXX | XXXXXXX |
| XXXX | y | n | XXXXX | XXXXXX | XXXXXXX |
| XXXX | y | n | XXXXX | XXXXXX | XXXXXXX |
| XXXX | y | n | XXXXX | XXXXXX | XXXXXXX |
| XXXX | y | n | XXXXX | XXXXXX | XXXXXXX |
| XXXX | y | n | XXXXX | XXXXXX | XXXXXXX |
| XXXX | y | n | XXXXX | XXXXXX | XXXXXXX |
| XXXX | y | n | XXXXX | XXXXXX | XXXXXXX |
| XXXX | y | n | XXXXX | XXXXXX | XXXXXXX |
| XXXX | y | n | XXXXX | XXXXXX | XXXXXXX |
| XXXX | y | n | XXXXX | XXXXXX | XXXXXXX |
| XXXX | y | n | XXXXX | XXXXXX | XXXXXXX |
| XXXX | y | n | XXXXX | XXXXXX | XXXXXXX |
| XXXX | y | n | XXXXX | XXXXXX | XXXXXXX |
| XXXX | y | n | XXXXX | XXXXXX | XXXXXXX |
| XXXX | y | n | XXXXX | XXXXXX | XXXXXXX |
| XXXX | y | n | XXXXX | XXXXXX | XXXXXXX |
| XXXX | y | n | XXXXX | XXXXXX | XXXXXXX |
| XXXX | y | n | XXXXX | XXXXXX | XXXXXXX |
| XXXX | y | n | XXXXX | XXXXXX | XXXXXXX |
| XXXX | y | n | XXXXX | XXXXXX | XXXXXXX |
| XXXX | y | n | XXXXX | XXXXXX | XXXXXXX |
| XXXX | y | n | XXXXX | XXXXXX | XXXXXXX |
| XXXX | y | n | XXXXX | XXXXXX | XXXXXXX |
| XXXX | y | n | XXXXX | XXXXXX | XXXXXXX |
| XXXX | y | n | XXXXX | XXXXXX | XXXXXXX |
| XXXX | y | n | XXXXX | XXXXXX | XXXXXXX |
| XXXX | y | n | XXXXX | XXXXXX | XXXXXXX |
| XXXX | y | y | XXXXX | XXXXXX | XXXXXXX |
| XXXX | y | y | XXXXX | XXXXXX | XXXXXXX |
| XXXX | y | y | XXXXX | XXXXXX | XXXXXXX |
| XXXX | y | y | XXXXX | XXXXXX | XXXXXXX |
| XXXX | y | y | XXXXX | XXXXXX | XXXXXXX |
| XXXX | y | y | XXXXX | XXXXXX | XXXXXXX |
| XXXX | y | y | XXXXX | XXXXXX | XXXXXXX |

| | | | | | |
|---|---|---|---|---|---|
| XXXX | y | y | XXXXX | XXXXXX | XXXXXXX |
| XXXX | y | y | XXXXX | XXXXXX | XXXXXXX |
| XXXX | y | y | XXXXX | XXXXXX | XXXXXXX |
| XXXX | y | n | XXXXX | XXXXXX | XXXXXXX |
| XXXX | y | y | XXXXX | XXXXXX | XXXXXXX |
| XXXX | y | y | XXXXX | XXXXXX | XXXXXXX |
| XXXX | y | y | XXXXX | XXXXXX | XXXXXXX |
| XXXX | y | y | XXXXX | XXXXXX | XXXXXXX |
| XXXX | y | y | XXXXX | XXXXXX | XXXXXXX |
| XXXX | y | y | XXXXX | XXXXXX | XXXXXXX |
| XXXX | y | y | XXXXX | XXXXXX | XXXXXXX |
| XXXX | y | y | XXXXX | XXXXXX | XXXXXXX |
| XXXX | y | y | XXXXX | XXXXXX | XXXXXXX |
| XXXX | y | y | XXXXX | XXXXXX | XXXXXXX |
| XXXX | y | y | XXXXX | XXXXXX | XXXXXXX |
| XXXX | y | y | XXXXX | XXXXXX | XXXXXXX |
| XXXX | y | n | XXXXX | XXXXXX | XXXXXXX |
| XXXX | y | y | XXXXX | XXXXXX | XXXXXXX |
| XXXX | y | n | XXXXX | XXXXXX | XXXXXXX |
| XXXX | y | n | XXXXX | XXXXXX | XXXXXXX |
| XXXX | y | y | XXXXX | XXXXXX | XXXXXXX |
| XXXX | y | y | XXXXX | XXXXXX | XXXXXXX |
| XXXX | y | n | XXXXX | XXXXXX | XXXXXXX |
| XXXX | y | y | XXXXX | XXXXXX | XXXXXXX |
| XXXX | y | n | XXXXX | XXXXXX | XXXXXXX |
| XXXX | y | n | XXXXX | XXXXXX | XXXXXXX |
| XXXX | y | y | XXXXX | XXXXXX | XXXXXXX |
| XXXX | y | y | XXXXX | XXXXXX | XXXXXXX |
| XXXX | y | y | XXXXX | XXXXXX | XXXXXXX |
| XXXX | y | n | XXXXX | XXXXXX | XXXXXXX |
| XXXX | y | y | XXXXX | XXXXXX | XXXXXXX |
| XXXX | y | n | XXXXX | XXXXXX | XXXXXXX |
| XXXX | y | y | XXXXX | XXXXXX | XXXXXXX |
| XXXX | y | y | XXXXX | XXXXXX | XXXXXXX |
| XXXX | y | y | XXXXX | XXXXXX | XXXXXXX |
| XXXX | y | n | XXXXX | XXXXXX | XXXXXXX |
| XXXX | y | n | XXXXX | XXXXXX | XXXXXXX |
| XXXX | y | n | XXXXX | XXXXXX | XXXXXXX |
| XXXX | y | n | XXXXX | XXXXXX | XXXXXXX |
| XXXX | y | y | XXXXX | XXXXXX | XXXXXXX |
| XXXX | y | y | XXXXX | XXXXXX | XXXXXXX |
| XXXX | y | n | XXXXX | XXXXXX | XXXXXXX |
| XXXX | y | n | XXXXX | XXXXXX | XXXXXXX |
| XXXX | y | n | XXXXX | XXXXXX | XXXXXXX |
| XXXX | y | n | XXXXX | XXXXXX | XXXXXXX |
| XXXX | y | n | XXXXX | XXXXXX | XXXXXXX |
| XXXX | y | n | XXXXX | XXXXXX | XXXXXXX |
| XXXX | y | y | XXXXX | XXXXXX | XXXXXXX |
| XXXX | y | y | XXXXX | XXXXXX | XXXXXXX |
| XXXX | y | y | XXXXX | XXXXXX | XXXXXXX |

| | | | | | |
|---|---|---|---|---|---|
| XXXX | y | y | XXXXX | XXXXXX | XXXXXXX |
| XXXX | y | y | XXXXX | XXXXXX | XXXXXXX |
| XXXX | y | y | XXXXX | XXXXXX | XXXXXXX |
| XXXX | y | y | XXXXX | XXXXXX | XXXXXXX |
| XXXX | y | y | XXXXX | XXXXXX | XXXXXXX |
| XXXX | y | y | XXXXX | XXXXXX | XXXXXXX |
| XXXX | y | y | XXXXX | XXXXXX | XXXXXXX |
| XXXX | y | y | XXXXX | XXXXXX | XXXXXXX |
| XXXX | y | y | XXXXX | XXXXXX | XXXXXXX |
| XXXX | y | y | XXXXX | XXXXXX | XXXXXXX |
| XXXX | y | y | XXXXX | XXXXXX | XXXXXXX |
| XXXX | y | y | XXXXX | XXXXXX | XXXXXXX |
| XXXX | y | y | XXXXX | XXXXXX | XXXXXXX |
| XXXX | y | y | XXXXX | XXXXXX | XXXXXXX |
| XXXX | y | y | XXXXX | XXXXXX | XXXXXXX |
| XXXX | y | y | XXXXX | XXXXXX | XXXXXXX |
| XXXX | y | y | XXXXX | XXXXXX | XXXXXXX |
| XXXX | y | y | XXXXX | XXXXXX | XXXXXXX |
| XXXX | y | y | XXXXX | XXXXXX | XXXXXXX |
| XXXX | y | y | XXXXX | XXXXXX | XXXXXXX |
| XXXX | y | y | XXXXX | XXXXXX | XXXXXXX |
| XXXX | y | y | XXXXX | XXXXXX | XXXXXXX |
| XXXX | y | y | XXXXX | XXXXXX | XXXXXXX |
| XXXX | y | y | XXXXX | XXXXXX | XXXXXXX |
| XXXX | y | y | XXXXX | XXXXXX | XXXXXXX |
| XXXX | y | y | XXXXX | XXXXXX | XXXXXXX |
| XXXX | y | y | XXXXX | XXXXXX | XXXXXXX |
| XXXX | y | y | XXXXX | XXXXXX | XXXXXXX |
| XXXX | y | y | XXXXX | XXXXXX | XXXXXXX |
| XXXX | y | y | XXXXX | XXXXXX | XXXXXXX |
| XXXX | y | y | XXXXX | XXXXXX | XXXXXXX |
| XXXX | y | y | XXXXX | XXXXXX | XXXXXXX |
| XXXX | y | y | XXXXX | XXXXXX | XXXXXXX |
| XXXX | y | y | XXXXX | XXXXXX | XXXXXXX |
| XXXX | y | y | XXXXX | XXXXXX | XXXXXXX |
| XXXX | y | y | XXXXX | XXXXXX | XXXXXXX |
| XXXX | y | y | XXXXX | XXXXXX | XXXXXXX |
| XXXX | y | n | XXXXX | XXXXXX | XXXXXXX |
| XXXX | y | y | XXXXX | XXXXXX | XXXXXXX |
| XXXX | y | y | XXXXX | XXXXXX | XXXXXXX |
| XXXX | y | y | XXXXX | XXXXXX | XXXXXXX |
| XXXX | y | y | XXXXX | XXXXXX | XXXXXXX |
| XXXX | y | y | XXXXX | XXXXXX | XXXXXXX |
| XXXX | y | y | XXXXX | XXXXXX | XXXXXXX |
| XXXX | y | y | XXXXX | XXXXXX | XXXXXXX |
| XXXX | y | y | XXXXX | XXXXXX | XXXXXXX |
| XXXX | y | y | XXXXX | XXXXXX | XXXXXXX |
| XXXX | y | y | XXXXX | XXXXXX | XXXXXXX |
| XXXX | y | y | XXXXX | XXXXXX | XXXXXXX |
| XXXX | y | y | XXXXX | XXXXXX | XXXXXXX |

| | | | | | |
|---|---|---|---|---|---|
| XXXX | y | y | XXXXX | XXXXXX | XXXXXXX |
| XXXX | y | y | XXXXX | XXXXXX | XXXXXXX |
| XXXX | y | y | XXXXX | XXXXXX | XXXXXXX |
| XXXX | y | y | XXXXX | XXXXXX | XXXXXXX |
| XXXX | y | y | XXXXX | XXXXXX | XXXXXXX |
| XXXX | y | y | XXXXX | XXXXXX | XXXXXXX |
| XXXX | y | y | XXXXX | XXXXXX | XXXXXXX |
| XXXX | y | y | XXXXX | XXXXXX | XXXXXXX |
| XXXX | y | y | XXXXX | XXXXXX | XXXXXXX |
| XXXX | y | y | XXXXX | XXXXXX | XXXXXXX |
| XXXX | y | y | XXXXX | XXXXXX | XXXXXXX |
| XXXX | y | y | XXXXX | XXXXXX | XXXXXXX |
| XXXX | y | y | XXXXX | XXXXXX | XXXXXXX |
| XXXX | y | y | XXXXX | XXXXXX | XXXXXXX |
| XXXX | y | y | XXXXX | XXXXXX | XXXXXXX |
| XXXX | y | y | XXXXX | XXXXXX | XXXXXXX |
| XXXX | y | y | XXXXX | XXXXXX | XXXXXXX |
| XXXX | y | y | XXXXX | XXXXXX | XXXXXXX |
| XXXX | y | y | XXXXX | XXXXXX | XXXXXXX |
| XXXX | y | y | XXXXX | XXXXXX | XXXXXXX |
| XXXX | y | y | XXXXX | XXXXXX | XXXXXXX |
| XXXX | y | y | XXXXX | XXXXXX | XXXXXXX |
| XXXX | y | y | XXXXX | XXXXXX | XXXXXXX |
| XXXX | y | y | XXXXX | XXXXXX | XXXXXXX |
| XXXX | y | n | XXXXX | XXXXXX | XXXXXXX |
| XXXX | y | n | XXXXX | XXXXXX | XXXXXXX |
| XXXX | y | n | XXXXX | XXXXXX | XXXXXXX |
| XXXX | y | n | XXXXX | XXXXXX | XXXXXXX |
| XXXX | y | n | XXXXX | XXXXXX | XXXXXXX |
| XXXX | y | n | XXXXX | XXXXXX | XXXXXXX |
| XXXX | y | n | XXXXX | XXXXXX | XXXXXXX |
| XXXX | y | n | XXXXX | XXXXXX | XXXXXXX |
| XXXX | y | n | XXXXX | XXXXXX | XXXXXXX |
| XXXX | y | n | XXXXX | XXXXXX | XXXXXXX |
| XXXX | y | n | XXXXX | XXXXXX | XXXXXXX |
| XXXX | y | n | XXXXX | XXXXXX | XXXXXXX |
| XXXX | y | n | XXXXX | XXXXXX | XXXXXXX |
| XXXX | y | n | XXXXX | XXXXXX | XXXXXXX |
| XXXX | y | n | XXXXX | XXXXXX | XXXXXXX |
| XXXX | y | n | XXXXX | XXXXXX | XXXXXXX |
| XXXX | y | n | XXXXX | XXXXXX | XXXXXXX |
| XXXX | y | n | XXXXX | XXXXXX | XXXXXXX |
| XXXX | y | n | XXXXX | XXXXXX | XXXXXXX |
| XXXX | y | n | XXXXX | XXXXXX | XXXXXXX |
| XXXX | y | n | XXXXX | XXXXXX | XXXXXXX |
| XXXX | y | n | XXXXX | XXXXXX | XXXXXXX |
| XXXX | y | n | XXXXX | XXXXXX | XXXXXXX |
| XXXX | y | n | XXXXX | XXXXXX | XXXXXXX |
| XXXX | y | n | XXXXX | XXXXXX | XXXXXXX |
| XXXX | y | n | XXXXX | XXXXXX | XXXXXXX |

| | | | | | |
|---|---|---|---|---|---|
| XXXX | y | n | XXXXX | XXXXXX | XXXXXXX |
| XXXX | y | n | XXXXX | XXXXXX | XXXXXXX |
| XXXX | y | n | XXXXX | XXXXXX | XXXXXXX |
| XXXX | y | n | XXXXX | XXXXXX | XXXXXXX |
| XXXX | y | n | XXXXX | XXXXXX | XXXXXXX |
| XXXX | y | n | XXXXX | XXXXXX | XXXXXXX |
| XXXX | y | n | XXXXX | XXXXXX | XXXXXXX |
| XXXX | y | n | XXXXX | XXXXXX | XXXXXXX |
| XXXX | y | n | XXXXX | XXXXXX | XXXXXXX |
| XXXX | y | n | XXXXX | XXXXXX | XXXXXXX |
| XXXX | y | n | XXXXX | XXXXXX | XXXXXXX |
| XXXX | y | n | XXXXX | XXXXXX | XXXXXXX |
| XXXX | y | n | XXXXX | XXXXXX | XXXXXXX |
| XXXX | y | n | XXXXX | XXXXXX | XXXXXXX |
| XXXX | y | n | XXXXX | XXXXXX | XXXXXXX |
| XXXX | y | n | XXXXX | XXXXXX | XXXXXXX |
| XXXX | y | n | XXXXX | XXXXXX | XXXXXXX |
| XXXX | y | n | XXXXX | XXXXXX | XXXXXXX |
| XXXX | y | n | XXXXX | XXXXXX | XXXXXXX |
| XXXX | y | n | XXXXX | XXXXXX | XXXXXXX |
| XXXX | y | n | XXXXX | XXXXXX | XXXXXXX |
| XXXX | y | n | XXXXX | XXXXXX | XXXXXXX |
| XXXX | y | n | XXXXX | XXXXXX | XXXXXXX |
| XXXX | y | n | XXXXX | XXXXXX | XXXXXXX |
| XXXX | y | n | XXXXX | XXXXXX | XXXXXXX |
| XXXX | y | n | XXXXX | XXXXXX | XXXXXXX |
| XXXX | y | n | XXXXX | XXXXXX | XXXXXXX |
| XXXX | y | n | XXXXX | XXXXXX | XXXXXXX |
| XXXX | y | n | XXXXX | XXXXXX | XXXXXXX |
| XXXX | y | n | XXXXX | XXXXXX | XXXXXXX |
| XXXX | y | n | XXXXX | XXXXXX | XXXXXXX |
| XXXX | y | n | XXXXX | XXXXXX | XXXXXXX |
| XXXX | y | n | XXXXX | XXXXXX | XXXXXXX |
| XXXX | y | n | XXXXX | XXXXXX | XXXXXXX |
| XXXX | y | n | XXXXX | XXXXXX | XXXXXXX |
| XXXX | y | n | XXXXX | XXXXXX | XXXXXXX |
| XXXX | y | n | XXXXX | XXXXXX | XXXXXXX |
| XXXX | y | n | XXXXX | XXXXXX | XXXXXXX |
| XXXX | y | n | XXXXX | XXXXXX | XXXXXXX |
| XXXX | y | n | XXXXX | XXXXXX | XXXXXXX |
| XXXX | y | n | XXXXX | XXXXXX | XXXXXXX |
| XXXX | y | n | XXXXX | XXXXXX | XXXXXXX |
| XXXX | y | n | XXXXX | XXXXXX | XXXXXXX |
| XXXX | y | n | XXXXX | XXXXXX | XXXXXXX |
| XXXX | y | n | XXXXX | XXXXXX | XXXXXXX |
| XXXX | y | n | XXXXX | XXXXXX | XXXXXXX |
| XXXX | y | n | XXXXX | XXXXXX | XXXXXXX |
| XXXX | y | n | XXXXX | XXXXXX | XXXXXXX |
| XXXX | y | n | XXXXX | XXXXXX | XXXXXXX |
| XXXX | y | n | XXXXX | XXXXXX | XXXXXXX |

| | | | | | |
|---|---|---|---|---|---|
| XXXX | y | n | XXXXX | XXXXXX | XXXXXXX |
| XXXX | y | n | XXXXX | XXXXXX | XXXXXXX |
| XXXX | y | n | XXXXX | XXXXXX | XXXXXXX |
| XXXX | y | n | XXXXX | XXXXXX | XXXXXXX |
| XXXX | y | n | XXXXX | XXXXXX | XXXXXXX |
| XXXX | y | n | XXXXX | XXXXXX | XXXXXXX |
| XXXX | y | n | XXXXX | XXXXXX | XXXXXXX |
| XXXX | y | n | XXXXX | XXXXXX | XXXXXXX |
| XXXX | y | n | XXXXX | XXXXXX | XXXXXXX |
| XXXX | y | n | XXXXX | XXXXXX | XXXXXXX |
| XXXX | y | n | XXXXX | XXXXXX | XXXXXXX |
| XXXX | y | n | XXXXX | XXXXXX | XXXXXXX |
| XXXX | y | n | XXXXX | XXXXXX | XXXXXXX |
| XXXX | y | n | XXXXX | XXXXXX | XXXXXXX |
| XXXX | y | n | XXXXX | XXXXXX | XXXXXXX |
| XXXX | y | n | XXXXX | XXXXXX | XXXXXXX |
| XXXX | y | n | XXXXX | XXXXXX | XXXXXXX |
| XXXX | y | n | XXXXX | XXXXXX | XXXXXXX |
| XXXX | y | n | XXXXX | XXXXXX | XXXXXXX |
| XXXX | y | n | XXXXX | XXXXXX | XXXXXXX |
| XXXX | y | n | XXXXX | XXXXXX | XXXXXXX |
| XXXX | y | n | XXXXX | XXXXXX | XXXXXXX |
| XXXX | y | n | XXXXX | XXXXXX | XXXXXXX |
| XXXX | y | n | XXXXX | XXXXXX | XXXXXXX |
| XXXX | y | n | XXXXX | XXXXXX | XXXXXXX |
| XXXX | y | n | XXXXX | XXXXXX | XXXXXXX |
| XXXX | y | n | XXXXX | XXXXXX | XXXXXXX |
| XXXX | y | n | XXXXX | XXXXXX | XXXXXXX |
| XXXX | y | n | XXXXX | XXXXXX | XXXXXXX |
| XXXX | y | n | XXXXX | XXXXXX | XXXXXXX |
| XXXX | y | n | XXXXX | XXXXXX | XXXXXXX |
| XXXX | y | n | XXXXX | XXXXXX | XXXXXXX |
| XXXX | y | n | XXXXX | XXXXXX | XXXXXXX |
| XXXX | y | n | XXXXX | XXXXXX | XXXXXXX |
| XXXX | y | n | XXXXX | XXXXXX | XXXXXXX |
| XXXX | y | n | XXXXX | XXXXXX | XXXXXXX |
| XXXX | y | n | XXXXX | XXXXXX | XXXXXXX |
| XXXX | y | n | XXXXX | XXXXXX | XXXXXXX |
| XXXX | y | n | XXXXX | XXXXXX | XXXXXXX |
| XXXX | y | n | XXXXX | XXXXXX | XXXXXXX |
| XXXX | y | n | XXXXX | XXXXXX | XXXXXXX |
| XXXX | y | n | XXXXX | XXXXXX | XXXXXXX |
| XXXX | y | n | XXXXX | XXXXXX | XXXXXXX |
| XXXX | y | n | XXXXX | XXXXXX | XXXXXXX |
| XXXX | y | n | XXXXX | XXXXXX | XXXXXXX |
| XXXX | y | n | XXXXX | XXXXXX | XXXXXXX |
| XXXX | y | n | XXXXX | XXXXXX | XXXXXXX |
| XXXX | y | n | XXXXX | XXXXXX | XXXXXXX |
| XXXX | y | n | XXXXX | XXXXXX | XXXXXXX |
| XXXX | y | n | XXXXX | XXXXXX | XXXXXXX |

| | | | | | |
|---|---|---|---|---|---|
| XXXX | y | n | XXXXX | XXXXXX | XXXXXXX |
| XXXX | y | n | XXXXX | XXXXXX | XXXXXXX |
| XXXX | y | n | XXXXX | XXXXXX | XXXXXXX |
| XXXX | y | n | XXXXX | XXXXXX | XXXXXXX |
| XXXX | y | n | XXXXX | XXXXXX | XXXXXXX |
| XXXX | y | n | XXXXX | XXXXXX | XXXXXXX |
| XXXX | y | n | XXXXX | XXXXXX | XXXXXXX |
| XXXX | y | n | XXXXX | XXXXXX | XXXXXXX |
| XXXX | y | n | XXXXX | XXXXXX | XXXXXXX |
| XXXX | y | n | XXXXX | XXXXXX | XXXXXXX |
| XXXX | y | n | XXXXX | XXXXXX | XXXXXXX |
| XXXX | y | n | XXXXX | XXXXXX | XXXXXXX |
| XXXX | y | n | XXXXX | XXXXXX | XXXXXXX |
| XXXX | y | n | XXXXX | XXXXXX | XXXXXXX |
| XXXX | y | n | XXXXX | XXXXXX | XXXXXXX |
| XXXX | y | n | XXXXX | XXXXXX | XXXXXXX |
| XXXX | y | n | XXXXX | XXXXXX | XXXXXXX |
| XXXX | y | n | XXXXX | XXXXXX | XXXXXXX |
| XXXX | y | n | XXXXX | XXXXXX | XXXXXXX |
| XXXX | y | n | XXXXX | XXXXXX | XXXXXXX |
| XXXX | y | n | XXXXX | XXXXXX | XXXXXXX |
| XXXX | y | n | XXXXX | XXXXXX | XXXXXXX |
| XXXX | y | n | XXXXX | XXXXXX | XXXXXXX |
| XXXX | y | n | XXXXX | XXXXXX | XXXXXXX |
| XXXX | y | n | XXXXX | XXXXXX | XXXXXXX |
| XXXX | y | n | XXXXX | XXXXXX | XXXXXXX |
| XXXX | y | n | XXXXX | XXXXXX | XXXXXXX |
| XXXX | y | n | XXXXX | XXXXXX | XXXXXXX |
| XXXX | y | n | XXXXX | XXXXXX | XXXXXXX |
| XXXX | y | n | XXXXX | XXXXXX | XXXXXXX |
| XXXX | y | n | XXXXX | XXXXXX | XXXXXXX |
| XXXX | y | n | XXXXX | XXXXXX | XXXXXXX |
| XXXX | y | n | XXXXX | XXXXXX | XXXXXXX |
| XXXX | y | n | XXXXX | XXXXXX | XXXXXXX |
| XXXX | y | n | XXXXX | XXXXXX | XXXXXXX |
| XXXX | y | n | XXXXX | XXXXXX | XXXXXXX |
| XXXX | y | n | XXXXX | XXXXXX | XXXXXXX |
| XXXX | y | n | XXXXX | XXXXXX | XXXXXXX |
| XXXX | y | n | XXXXX | XXXXXX | XXXXXXX |
| XXXX | y | n | XXXXX | XXXXXX | XXXXXXX |
| XXXX | y | n | XXXXX | XXXXXX | XXXXXXX |
| XXXX | y | n | XXXXX | XXXXXX | XXXXXXX |
| XXXX | y | n | XXXXX | XXXXXX | XXXXXXX |
| XXXX | y | n | XXXXX | XXXXXX | XXXXXXX |
| XXXX | y | n | XXXXX | XXXXXX | XXXXXXX |
| XXXX | y | n | XXXXX | XXXXXX | XXXXXXX |
| XXXX | y | n | XXXXX | XXXXXX | XXXXXXX |
| XXXX | y | n | XXXXX | XXXXXX | XXXXXXX |
| XXXX | y | n | XXXXX | XXXXXX | XXXXXXX |
| XXXX | y | n | XXXXX | XXXXXX | XXXXXXX |

| | | | | | |
|---|---|---|---|---|---|
| XXXX | y | n | XXXXX | XXXXXX | XXXXXXX |
| XXXX | y | n | XXXXX | XXXXXX | XXXXXXX |
| XXXX | y | n | XXXXX | XXXXXX | XXXXXXX |
| XXXX | y | n | XXXXX | XXXXXX | XXXXXXX |
| XXXX | y | n | XXXXX | XXXXXX | XXXXXXX |
| XXXX | y | n | XXXXX | XXXXXX | XXXXXXX |
| XXXX | y | n | XXXXX | XXXXXX | XXXXXXX |
| XXXX | y | n | XXXXX | XXXXXX | XXXXXXX |
| XXXX | y | n | XXXXX | XXXXXX | XXXXXXX |
| XXXX | y | n | XXXXX | XXXXXX | XXXXXXX |
| XXXX | y | n | XXXXX | XXXXXX | XXXXXXX |
| XXXX | y | n | XXXXX | XXXXXX | XXXXXXX |
| XXXX | y | n | XXXXX | XXXXXX | XXXXXXX |
| XXXX | y | n | XXXXX | XXXXXX | XXXXXXX |
| XXXX | y | n | XXXXX | XXXXXX | XXXXXXX |
| XXXX | y | n | XXXXX | XXXXXX | XXXXXXX |
| XXXX | y | n | XXXXX | XXXXXX | XXXXXXX |
| XXXX | y | n | XXXXX | XXXXXX | XXXXXXX |
| XXXX | y | n | XXXXX | XXXXXX | XXXXXXX |
| XXXX | y | n | XXXXX | XXXXXX | XXXXXXX |
| XXXX | y | n | XXXXX | XXXXXX | XXXXXXX |
| XXXX | y | n | XXXXX | XXXXXX | XXXXXXX |
| XXXX | y | n | XXXXX | XXXXXX | XXXXXXX |
| XXXX | y | n | XXXXX | XXXXXX | XXXXXXX |
| XXXX | y | n | XXXXX | XXXXXX | XXXXXXX |
| XXXX | y | n | XXXXX | XXXXXX | XXXXXXX |
| XXXX | y | n | XXXXX | XXXXXX | XXXXXXX |
| XXXX | y | n | XXXXX | XXXXXX | XXXXXXX |
| XXXX | y | n | XXXXX | XXXXXX | XXXXXXX |
| XXXX | y | n | XXXXX | XXXXXX | XXXXXXX |
| XXXX | y | n | XXXXX | XXXXXX | XXXXXXX |
| XXXX | y | n | XXXXX | XXXXXX | XXXXXXX |
| XXXX | y | n | XXXXX | XXXXXX | XXXXXXX |
| XXXX | y | n | XXXXX | XXXXXX | XXXXXXX |
| XXXX | y | n | XXXXX | XXXXXX | XXXXXXX |
| XXXX | y | n | XXXXX | XXXXXX | XXXXXXX |
| XXXX | y | n | XXXXX | XXXXXX | XXXXXXX |
| XXXX | y | n | XXXXX | XXXXXX | XXXXXXX |
| XXXX | y | n | XXXXX | XXXXXX | XXXXXXX |
| XXXX | y | n | XXXXX | XXXXXX | XXXXXXX |
| XXXX | y | n | XXXXX | XXXXXX | XXXXXXX |
| XXXX | y | n | XXXXX | XXXXXX | XXXXXXX |
| XXXX | y | n | XXXXX | XXXXXX | XXXXXXX |
| XXXX | y | n | XXXXX | XXXXXX | XXXXXXX |
| XXXX | y | n | XXXXX | XXXXXX | XXXXXXX |
| XXXX | y | n | XXXXX | XXXXXX | XXXXXXX |
| XXXX | y | n | XXXXX | XXXXXX | XXXXXXX |
| XXXX | y | n | XXXXX | XXXXXX | XXXXXXX |
| XXXX | y | n | XXXXX | XXXXXX | XXXXXXX |
| XXXX | y | n | XXXXX | XXXXXX | XXXXXXX |

| | | | | | |
|---|---|---|---|---|---|
| XXXX | y | n | XXXXX | XXXXXX | XXXXXXX |
| XXXX | y | n | XXXXX | XXXXXX | XXXXXXX |
| XXXX | y | n | XXXXX | XXXXXX | XXXXXXX |
| XXXX | y | n | XXXXX | XXXXXX | XXXXXXX |
| XXXX | y | n | XXXXX | XXXXXX | XXXXXXX |
| XXXX | y | n | XXXXX | XXXXXX | XXXXXXX |
| XXXX | y | n | XXXXX | XXXXXX | XXXXXXX |
| XXXX | y | n | XXXXX | XXXXXX | XXXXXXX |
| XXXX | y | n | XXXXX | XXXXXX | XXXXXXX |
| XXXX | y | n | XXXXX | XXXXXX | XXXXXXX |
| XXXX | y | n | XXXXX | XXXXXX | XXXXXXX |
| XXXX | y | n | XXXXX | XXXXXX | XXXXXXX |
| XXXX | y | n | XXXXX | XXXXXX | XXXXXXX |
| XXXX | y | n | XXXXX | XXXXXX | XXXXXXX |
| XXXX | y | n | XXXXX | XXXXXX | XXXXXXX |
| XXXX | y | n | XXXXX | XXXXXX | XXXXXXX |
| XXXX | y | n | XXXXX | XXXXXX | XXXXXXX |
| XXXX | y | n | XXXXX | XXXXXX | XXXXXXX |
| XXXX | y | n | XXXXX | XXXXXX | XXXXXXX |
| XXXX | y | n | XXXXX | XXXXXX | XXXXXXX |
| XXXX | y | n | XXXXX | XXXXXX | XXXXXXX |
| XXXX | y | n | XXXXX | XXXXXX | XXXXXXX |
| XXXX | y | n | XXXXX | XXXXXX | XXXXXXX |
| XXXX | y | n | XXXXX | XXXXXX | XXXXXXX |
| XXXX | y | n | XXXXX | XXXXXX | XXXXXXX |
| XXXX | y | n | XXXXX | XXXXXX | XXXXXXX |
| XXXX | y | n | XXXXX | XXXXXX | XXXXXXX |
| XXXX | y | n | XXXXX | XXXXXX | XXXXXXX |
| XXXX | y | n | XXXXX | XXXXXX | XXXXXXX |
| XXXX | y | n | XXXXX | XXXXXX | XXXXXXX |
| XXXX | y | n | XXXXX | XXXXXX | XXXXXXX |
| XXXX | y | n | XXXXX | XXXXXX | XXXXXXX |
| XXXX | y | n | XXXXX | XXXXXX | XXXXXXX |
| XXXX | y | n | XXXXX | XXXXXX | XXXXXXX |
| XXXX | y | n | XXXXX | XXXXXX | XXXXXXX |
| XXXX | y | n | XXXXX | XXXXXX | XXXXXXX |
| XXXX | y | n | XXXXX | XXXXXX | XXXXXXX |
| XXXX | y | n | XXXXX | XXXXXX | XXXXXXX |
| XXXX | y | n | XXXXX | XXXXXX | XXXXXXX |
| XXXX | y | n | XXXXX | XXXXXX | XXXXXXX |
| XXXX | y | n | XXXXX | XXXXXX | XXXXXXX |
| XXXX | y | n | XXXXX | XXXXXX | XXXXXXX |
| XXXX | y | n | XXXXX | XXXXXX | XXXXXXX |
| XXXX | y | n | XXXXX | XXXXXX | XXXXXXX |
| XXXX | y | n | XXXXX | XXXXXX | XXXXXXX |
| XXXX | y | n | XXXXX | XXXXXX | XXXXXXX |
| XXXX | y | n | XXXXX | XXXXXX | XXXXXXX |
| XXXX | y | n | XXXXX | XXXXXX | XXXXXXX |
| XXXX | na | N | XXXXX | XXXXXX | XXXXXXX |
| XXXX | na | N | XXXXX | XXXXXX | XXXXXXX |

| | | | | | |
|---|---|---|---|---|---|
| XXXX | na | N | XXXXX | XXXXXX | XXXXXXX |
| XXXX | na | N | XXXXX | XXXXXX | XXXXXXX |
| XXXX | na | N | XXXXX | XXXXXX | XXXXXXX |
| XXXX | na | N | XXXXX | XXXXXX | XXXXXXX |
| XXXX | na | N | XXXXX | XXXXXX | XXXXXXX |
| XXXX | na | N | XXXXX | XXXXXX | XXXXXXX |
| XXXX | na | N | XXXXX | XXXXXX | XXXXXXX |
| XXXX | na | N | XXXXX | XXXXXX | XXXXXXX |
| XXXX | na | N | XXXXX | XXXXXX | XXXXXXX |
| XXXX | na | N | XXXXX | XXXXXX | XXXXXXX |
| XXXX | na | N | XXXXX | XXXXXX | XXXXXXX |
| XXXX | na | N | XXXXX | XXXXXX | XXXXXXX |
| XXXX | na | N | XXXXX | XXXXXX | XXXXXXX |
| XXXX | na | N | XXXXX | XXXXXX | XXXXXXX |
| XXXX | na | N | XXXXX | XXXXXX | XXXXXXX |
| XXXX | na | N | XXXXX | XXXXXX | XXXXXXX |
| XXXX | na | N | XXXXX | XXXXXX | XXXXXXX |
| XXXX | na | N | XXXXX | XXXXXX | XXXXXXX |
| XXXX | na | N | XXXXX | XXXXXX | XXXXXXX |
| XXXX | na | N | XXXXX | XXXXXX | XXXXXXX |
| XXXX | na | N | XXXXX | XXXXXX | XXXXXXX |
| XXXX | na | N | XXXXX | XXXXXX | XXXXXXX |
| XXXX | na | N | XXXXX | XXXXXX | XXXXXXX |
| XXXX | na | N | XXXXX | XXXXXX | XXXXXXX |
| XXXX | na | N | XXXXX | XXXXXX | XXXXXXX |
| XXXX | na | N | XXXXX | XXXXXX | XXXXXXX |
| XXXX | na | N | XXXXX | XXXXXX | XXXXXXX |
| XXXX | na | N | XXXXX | XXXXXX | XXXXXXX |
| XXXX | na | N | XXXXX | XXXXXX | XXXXXXX |
| XXXX | na | N | XXXXX | XXXXXX | XXXXXXX |
| XXXX | na | N | XXXXX | XXXXXX | XXXXXXX |
| XXXX | na | N | XXXXX | XXXXXX | XXXXXXX |
| XXXX | na | N | XXXXX | XXXXXX | XXXXXXX |
| XXXX | na | N | XXXXX | XXXXXX | XXXXXXX |
| XXXX | na | N | XXXXX | XXXXXX | XXXXXXX |
| XXXX | na | N | XXXXX | XXXXXX | XXXXXXX |
| XXXX | na | N | XXXXX | XXXXXX | XXXXXXX |
| XXXX | na | N | XXXXX | XXXXXX | XXXXXXX |
| XXXX | na | n | XXXXX | XXXXXX | XXXXXXX |
| XXXX | y | y | XXXXX | XXXXXX | XXXXXXX |
| XXXX | y | y | XXXXX | XXXXXX | XXXXXXX |
| XXXX | y | y | XXXXX | XXXXXX | XXXXXXX |
| XXXX | n | N | XXXXX | XXXXXX | XXXXXXX |
| XXXX | n | Y | XXXXX | XXXXXX | XXXXXXX |
| XXXX | n | Y | XXXXX | XXXXXX | XXXXXXX |
| XXXX | n | y | XXXXX | XXXXXX | XXXXXXX |
| XXXX | n | Y | XXXXX | XXXXXX | XXXXXXX |
| XXXX | n | Y | XXXXX | XXXXXX | XXXXXXX |
| XXXX | n | Y | XXXXX | XXXXXX | XXXXXXX |
| XXXX | n | Y | XXXXX | XXXXXX | XXXXXXX |

| | | | | | |
|---|---|---|---|---|---|
| XXXX | y | n | XXXXX | XXXXXX | XXXXXXX |
| XXXX | n | y | XXXXX | XXXXXX | XXXXXXX |
| XXXX | n | Y | XXXXX | XXXXXX | XXXXXXX |
| XXXX | n | N | XXXXX | XXXXXX | XXXXXXX |
| XXXX | y | y | XXXXX | XXXXXX | XXXXXXX |
| XXXX | y | y | XXXXX | XXXXXX | XXXXXXX |
| XXXX | y | y | XXXXX | XXXXXX | XXXXXXX |
| XXXX | y | y | XXXXX | XXXXXX | XXXXXXX |
| XXXX | y | y | XXXXX | XXXXXX | XXXXXXX |
| XXXX | y | y | XXXXX | XXXXXX | XXXXXXX |
| XXXX | y | y | XXXXX | XXXXXX | XXXXXXX |
| XXXX | y | y | XXXXX | XXXXXX | XXXXXXX |
| XXXX | y | y | XXXXX | XXXXXX | XXXXXXX |
| XXXX | y | y | XXXXX | XXXXXX | XXXXXXX |
| XXXX | y | y | XXXXX | XXXXXX | XXXXXXX |
| XXXX | y | y | XXXXX | XXXXXX | XXXXXXX |
| XXXX | y | y | XXXXX | XXXXXX | XXXXXXX |
| XXXX | y | y | XXXXX | XXXXXX | XXXXXXX |
| XXXX | y | y | XXXXX | XXXXXX | XXXXXXX |
| XXXX | y | y | XXXXX | XXXXXX | XXXXXXX |
| XXXX | y | y | XXXXX | XXXXXX | XXXXXXX |
| XXXX | y | y | XXXXX | XXXXXX | XXXXXXX |
| XXXX | y | y | XXXXX | XXXXXX | XXXXXXX |
| XXXX | y | y | XXXXX | XXXXXX | XXXXXXX |
| XXXX | y | y | XXXXX | XXXXXX | XXXXXXX |
| XXXX | y | y | XXXXX | XXXXXX | XXXXXXX |
| XXXX | y | y | XXXXX | XXXXXX | XXXXXXX |
| XXXX | y | y | XXXXX | XXXXXX | XXXXXXX |
| XXXX | y | y | XXXXX | XXXXXX | XXXXXXX |
| XXXX | y | y | XXXXX | XXXXXX | XXXXXXX |
| XXXX | y | y | XXXXX | XXXXXX | XXXXXXX |
| XXXX | y | y | XXXXX | XXXXXX | XXXXXXX |
| XXXX | n | N | XXXXX | XXXXXX | XXXXXXX |
| XXXX | n | N | XXXXX | XXXXXX | XXXXXXX |
| XXXX | n | N | XXXXX | XXXXXX | XXXXXXX |
| XXXX | n | N | XXXXX | XXXXXX | XXXXXXX |
| XXXX | n | N | XXXXX | XXXXXX | XXXXXXX |
| XXXX | n | N | XXXXX | XXXXXX | XXXXXXX |
| XXXX | n | N | XXXXX | XXXXXX | XXXXXXX |
| XXXX | n | N | XXXXX | XXXXXX | XXXXXXX |
| XXXX | n | N | XXXXX | XXXXXX | XXXXXXX |
| XXXX | n | N | XXXXX | XXXXXX | XXXXXXX |
| XXXX | n | N | XXXXX | XXXXXX | XXXXXXX |
| XXXX | n | N | XXXXX | XXXXXX | XXXXXXX |
| XXXX | n | N | XXXXX | XXXXXX | XXXXXXX |
| XXXX | n | N | XXXXX | XXXXXX | XXXXXXX |
| XXXX | n | N | XXXXX | XXXXXX | XXXXXXX |
| XXXX | n | N | XXXXX | XXXXXX | XXXXXXX |
| XXXX | n | N | XXXXX | XXXXXX | XXXXXXX |
| XXXX | n | N | XXXXX | XXXXXX | XXXXXXX |

| | | | | | |
|---|---|---|---|---|---|
| XXXX | n | N | XXXXX | XXXXXX | XXXXXXX |
| XXXX | n | N | XXXXX | XXXXXX | XXXXXXX |
| XXXX | n | N | XXXXX | XXXXXX | XXXXXXX |
| XXXX | n | N | XXXXX | XXXXXX | XXXXXXX |
| XXXX | n | N | XXXXX | XXXXXX | XXXXXXX |
| XXXX | n | N | XXXXX | XXXXXX | XXXXXXX |
| XXXX | n | N | XXXXX | XXXXXX | XXXXXXX |
| XXXX | n | N | XXXXX | XXXXXX | XXXXXXX |
| XXXX | n | N | XXXXX | XXXXXX | XXXXXXX |
| XXXX | n | N | XXXXX | XXXXXX | XXXXXXX |
| XXXX | n | N | XXXXX | XXXXXX | XXXXXXX |
| XXXX | n | N | XXXXX | XXXXXX | XXXXXXX |
| XXXX | n | N | XXXXX | XXXXXX | XXXXXXX |
| XXXX | n | N | XXXXX | XXXXXX | XXXXXXX |
| XXXX | n | N | XXXXX | XXXXXX | XXXXXXX |
| XXXX | n | N | XXXXX | XXXXXX | XXXXXXX |
| XXXX | n | N | XXXXX | XXXXXX | XXXXXXX |
| XXXX | n | N | XXXXX | XXXXXX | XXXXXXX |
| XXXX | n | N | XXXXX | XXXXXX | XXXXXXX |
| XXXX | n | N | XXXXX | XXXXXX | XXXXXXX |
| XXXX | n | N | XXXXX | XXXXXX | XXXXXXX |
| XXXX | n | N | XXXXX | XXXXXX | XXXXXXX |
| XXXX | n | N | XXXXX | XXXXXX | XXXXXXX |
| XXXX | n | N | XXXXX | XXXXXX | XXXXXXX |
| XXXX | n | N | XXXXX | XXXXXX | XXXXXXX |
| XXXX | n | N | XXXXX | XXXXXX | XXXXXXX |
| XXXX | n | N | XXXXX | XXXXXX | XXXXXXX |
| XXXX | n | N | XXXXX | XXXXXX | XXXXXXX |
| XXXX | n | N | XXXXX | XXXXXX | XXXXXXX |
| XXXX | n | N | XXXXX | XXXXXX | XXXXXXX |
| XXXX | n | N | XXXXX | XXXXXX | XXXXXXX |
| XXXX | n | N | XXXXX | XXXXXX | XXXXXXX |
| XXXX | n | N | XXXXX | XXXXXX | XXXXXXX |
| XXXX | n | N | XXXXX | XXXXXX | XXXXXXX |
| XXXX | n | N | XXXXX | XXXXXX | XXXXXXX |
| XXXX | n | N | XXXXX | XXXXXX | XXXXXXX |
| XXXX | n | N | XXXXX | XXXXXX | XXXXXXX |
| XXXX | n | N | XXXXX | XXXXXX | XXXXXXX |
| XXXX | n | N | XXXXX | XXXXXX | XXXXXXX |
| XXXX | n | N | XXXXX | XXXXXX | XXXXXXX |
| XXXX | y | n | XXXXX | XXXXXX | XXXXXXX |
| XXXX | y | n | XXXXX | XXXXXX | XXXXXXX |
| XXXX | y | n | XXXXX | XXXXXX | XXXXXXX |
| XXXX | y | n | XXXXX | XXXXXX | XXXXXXX |
| XXXX | y | n | XXXXX | XXXXXX | XXXXXXX |
| XXXX | y | n | XXXXX | XXXXXX | XXXXXXX |
| XXXX | y | n | XXXXX | XXXXXX | XXXXXXX |
| XXXX | y | n | XXXXX | XXXXXX | XXXXXXX |
| XXXX | y | n | XXXXX | XXXXXX | XXXXXXX |
| XXXX | y | n | XXXXX | XXXXXX | XXXXXXX |

| | | | | | |
|---|---|---|---|---|---|
| XXXX | y | n | XXXXX | XXXXXX | XXXXXXX |
| XXXX | y | n | XXXXX | XXXXXX | XXXXXXX |
| XXXX | y | n | XXXXX | XXXXXX | XXXXXXX |
| XXXX | y | n | XXXXX | XXXXXX | XXXXXXX |
| XXXX | y | n | XXXXX | XXXXXX | XXXXXXX |
| XXXX | y | n | XXXXX | XXXXXX | XXXXXXX |
| XXXX | y | n | XXXXX | XXXXXX | XXXXXXX |
| XXXX | y | n | XXXXX | XXXXXX | XXXXXXX |
| XXXX | y | n | XXXXX | XXXXXX | XXXXXXX |
| XXXX | y | n | XXXXX | XXXXXX | XXXXXXX |
| XXXX | y | n | XXXXX | XXXXXX | XXXXXXX |
| XXXX | na | N | XXXXX | XXXXXX | XXXXXXX |
| XXXX | y | n | XXXXX | XXXXXX | XXXXXXX |
| XXXX | y | n | XXXXX | XXXXXX | XXXXXXX |
| XXXX | y | n | XXXXX | XXXXXX | XXXXXXX |
| XXXX | y | n | XXXXX | XXXXXX | XXXXXXX |
| XXXX | y | n | XXXXX | XXXXXX | XXXXXXX |
| XXXX | y | n | XXXXX | XXXXXX | XXXXXXX |
| XXXX | y | n | XXXXX | XXXXXX | XXXXXXX |
| XXXX | y | n | XXXXX | XXXXXX | XXXXXXX |
| XXXX | y | n | XXXXX | XXXXXX | XXXXXXX |
| XXXX | y | n | XXXXX | XXXXXX | XXXXXXX |
| XXXX | y | n | XXXXX | XXXXXX | XXXXXXX |
| XXXX | y | n | XXXXX | XXXXXX | XXXXXXX |
| XXXX | y | n | XXXXX | XXXXXX | XXXXXXX |
| XXXX | y | n | XXXXX | XXXXXX | XXXXXXX |
| XXXX | y | n | XXXXX | XXXXXX | XXXXXXX |
| XXXX | y | n | XXXXX | XXXXXX | XXXXXXX |
| XXXX | y | n | XXXXX | XXXXXX | XXXXXXX |
| XXXX | y | n | XXXXX | XXXXXX | XXXXXXX |
| XXXX | y | n | XXXXX | XXXXXX | XXXXXXX |
| XXXX | y | n | XXXXX | XXXXXX | XXXXXXX |
| XXXX | y | n | XXXXX | XXXXXX | XXXXXXX |
| XXXX | y | n | XXXXX | XXXXXX | XXXXXXX |
| XXXX | y | n | XXXXX | XXXXXX | XXXXXXX |
| XXXX | y | n | XXXXX | XXXXXX | XXXXXXX |
| XXXX | y | n | XXXXX | XXXXXX | XXXXXXX |
| XXXX | y | n | XXXXX | XXXXXX | XXXXXXX |
| XXXX | y | n | XXXXX | XXXXXX | XXXXXXX |
| XXXX | y | n | XXXXX | XXXXXX | XXXXXXX |
| XXXX | y | n | XXXXX | XXXXXX | XXXXXXX |
| XXXX | y | n | XXXXX | XXXXXX | XXXXXXX |
| XXXX | y | n | XXXXX | XXXXXX | XXXXXXX |
| XXXX | y | n | XXXXX | XXXXXX | XXXXXXX |
| XXXX | y | n | XXXXX | XXXXXX | XXXXXXX |
| XXXX | y | n | XXXXX | XXXXXX | XXXXXXX |
| XXXX | y | n | XXXXX | XXXXXX | XXXXXXX |
| XXXX | y | n | XXXXX | XXXXXX | XXXXXXX |
| XXXX | y | n | XXXXX | XXXXXX | XXXXXXX |
| XXXX | y | n | XXXXX | XXXXXX | XXXXXXX |

| | | | | | |
|---|---|---|---|---|---|
| XXXX | y | n | XXXXX | XXXXXX | XXXXXXX |
| XXXX | y | n | XXXXX | XXXXXX | XXXXXXX |
| XXXX | y | n | XXXXX | XXXXXX | XXXXXXX |
| XXXX | y | n | XXXXX | XXXXXX | XXXXXXX |
| XXXX | y | n | XXXXX | XXXXXX | XXXXXXX |
| XXXX | y | n | XXXXX | XXXXXX | XXXXXXX |
| XXXX | y | n | XXXXX | XXXXXX | XXXXXXX |
| XXXX | y | n | XXXXX | XXXXXX | XXXXXXX |
| XXXX | y | n | XXXXX | XXXXXX | XXXXXXX |
| XXXX | y | n | XXXXX | XXXXXX | XXXXXXX |
| XXXX | y | n | XXXXX | XXXXXX | XXXXXXX |
| XXXX | y | n | XXXXX | XXXXXX | XXXXXXX |
| XXXX | y | n | XXXXX | XXXXXX | XXXXXXX |
| XXXX | y | n | XXXXX | XXXXXX | XXXXXXX |
| XXXX | y | n | XXXXX | XXXXXX | XXXXXXX |
| XXXX | y | n | XXXXX | XXXXXX | XXXXXXX |
| XXXX | y | n | XXXXX | XXXXXX | XXXXXXX |
| XXXX | y | n | XXXXX | XXXXXX | XXXXXXX |
| XXXX | y | n | XXXXX | XXXXXX | XXXXXXX |
| XXXX | y | n | XXXXX | XXXXXX | XXXXXXX |
| XXXX | y | n | XXXXX | XXXXXX | XXXXXXX |
| XXXX | y | n | XXXXX | XXXXXX | XXXXXXX |
| XXXX | y | n | XXXXX | XXXXXX | XXXXXXX |
| XXXX | y | n | XXXXX | XXXXXX | XXXXXXX |
| XXXX | y | n | XXXXX | XXXXXX | XXXXXXX |
| XXXX | y | n | XXXXX | XXXXXX | XXXXXXX |
| XXXX | y | n | XXXXX | XXXXXX | XXXXXXX |
| XXXX | y | n | XXXXX | XXXXXX | XXXXXXX |
| XXXX | y | n | XXXXX | XXXXXX | XXXXXXX |
| XXXX | y | n | XXXXX | XXXXXX | XXXXXXX |
| XXXX | y | n | XXXXX | XXXXXX | XXXXXXX |
| XXXX | y | n | XXXXX | XXXXXX | XXXXXXX |
| XXXX | y | n | XXXXX | XXXXXX | XXXXXXX |
| XXXX | y | n | XXXXX | XXXXXX | XXXXXXX |
| XXXX | y | n | XXXXX | XXXXXX | XXXXXXX |
| XXXX | y | n | XXXXX | XXXXXX | XXXXXXX |
| XXXX | y | n | XXXXX | XXXXXX | XXXXXXX |
| XXXX | y | n | XXXXX | XXXXXX | XXXXXXX |
| XXXX | y | n | XXXXX | XXXXXX | XXXXXXX |
| XXXX | y | n | XXXXX | XXXXXX | XXXXXXX |
| XXXX | y | n | XXXXX | XXXXXX | XXXXXXX |
| XXXX | y | n | XXXXX | XXXXXX | XXXXXXX |
| XXXX | y | n | XXXXX | XXXXXX | XXXXXXX |
| XXXX | y | n | XXXXX | XXXXXX | XXXXXXX |
| XXXX | y | n | XXXXX | XXXXXX | XXXXXXX |
| XXXX | y | n | XXXXX | XXXXXX | XXXXXXX |
| XXXX | y | n | XXXXX | XXXXXX | XXXXXXX |
| XXXX | y | n | XXXXX | XXXXXX | XXXXXXX |
| XXXX | y | n | XXXXX | XXXXXX | XXXXXXX |
| XXXX | y | n | XXXXX | XXXXXX | XXXXXXX |

| | | | | | |
|---|---|---|---|---|---|
| XXXX | y | n | XXXXX | XXXXXX | XXXXXXX |
| XXXX | y | n | XXXXX | XXXXXX | XXXXXXX |
| XXXX | y | n | XXXXX | XXXXXX | XXXXXXX |
| XXXX | y | n | XXXXX | XXXXXX | XXXXXXX |
| XXXX | y | n | XXXXX | XXXXXX | XXXXXXX |
| XXXX | y | n | XXXXX | XXXXXX | XXXXXXX |
| XXXX | y | n | XXXXX | XXXXXX | XXXXXXX |
| XXXX | y | n | XXXXX | XXXXXX | XXXXXXX |
| XXXX | y | n | XXXXX | XXXXXX | XXXXXXX |
| XXXX | y | n | XXXXX | XXXXXX | XXXXXXX |
| XXXX | y | n | XXXXX | XXXXXX | XXXXXXX |
| XXXX | y | n | XXXXX | XXXXXX | XXXXXXX |
| XXXX | y | n | XXXXX | XXXXXX | XXXXXXX |
| XXXX | y | n | XXXXX | XXXXXX | XXXXXXX |
| XXXX | y | n | XXXXX | XXXXXX | XXXXXXX |
| XXXX | y | n | XXXXX | XXXXXX | XXXXXXX |
| XXXX | y | n | XXXXX | XXXXXX | XXXXXXX |
| XXXX | y | n | XXXXX | XXXXXX | XXXXXXX |
| XXXX | y | n | XXXXX | XXXXXX | XXXXXXX |
| XXXX | y | n | XXXXX | XXXXXX | XXXXXXX |
| XXXX | y | n | XXXXX | XXXXXX | XXXXXXX |
| XXXX | y | n | XXXXX | XXXXXX | XXXXXXX |
| XXXX | y | n | XXXXX | XXXXXX | XXXXXXX |
| XXXX | y | n | XXXXX | XXXXXX | XXXXXXX |
| XXXX | y | n | XXXXX | XXXXXX | XXXXXXX |
| XXXX | y | n | XXXXX | XXXXXX | XXXXXXX |
| XXXX | y | n | XXXXX | XXXXXX | XXXXXXX |
| XXXX | y | n | XXXXX | XXXXXX | XXXXXXX |
| XXXX | y | n | XXXXX | XXXXXX | XXXXXXX |
| XXXX | y | n | XXXXX | XXXXXX | XXXXXXX |
| XXXX | y | n | XXXXX | XXXXXX | XXXXXXX |
| XXXX | y | n | XXXXX | XXXXXX | XXXXXXX |
| XXXX | y | n | XXXXX | XXXXXX | XXXXXXX |
| XXXX | y | n | XXXXX | XXXXXX | XXXXXXX |
| XXXX | y | n | XXXXX | XXXXXX | XXXXXXX |
| XXXX | y | n | XXXXX | XXXXXX | XXXXXXX |
| XXXX | y | n | XXXXX | XXXXXX | XXXXXXX |
| XXXX | y | n | XXXXX | XXXXXX | XXXXXXX |
| XXXX | y | n | XXXXX | XXXXXX | XXXXXXX |
| XXXX | y | n | XXXXX | XXXXXX | XXXXXXX |
| XXXX | y | n | XXXXX | XXXXXX | XXXXXXX |
| XXXX | y | n | XXXXX | XXXXXX | XXXXXXX |
| XXXX | y | n | XXXXX | XXXXXX | XXXXXXX |
| XXXX | y | n | XXXXX | XXXXXX | XXXXXXX |
| XXXX | y | n | XXXXX | XXXXXX | XXXXXXX |
| XXXX | y | n | XXXXX | XXXXXX | XXXXXXX |
| XXXX | y | n | XXXXX | XXXXXX | XXXXXXX |
| XXXX | y | n | XXXXX | XXXXXX | XXXXXXX |
| XXXX | y | n | XXXXX | XXXXXX | XXXXXXX |
| XXXX | y | n | XXXXX | XXXXXX | XXXXXXX |

| | | | | | |
|---|---|---|---|---|---|
| XXXX | y | n | XXXXX | XXXXXX | XXXXXXX |
| XXXX | y | n | XXXXX | XXXXXX | XXXXXXX |
| XXXX | y | n | XXXXX | XXXXXX | XXXXXXX |
| XXXX | y | n | XXXXX | XXXXXX | XXXXXXX |
| XXXX | y | n | XXXXX | XXXXXX | XXXXXXX |
| XXXX | y | n | XXXXX | XXXXXX | XXXXXXX |
| XXXX | y | n | XXXXX | XXXXXX | XXXXXXX |
| XXXX | y | n | XXXXX | XXXXXX | XXXXXXX |
| XXXX | y | n | XXXXX | XXXXXX | XXXXXXX |
| XXXX | y | n | XXXXX | XXXXXX | XXXXXXX |
| XXXX | y | n | XXXXX | XXXXXX | XXXXXXX |
| XXXX | y | n | XXXXX | XXXXXX | XXXXXXX |
| XXXX | y | n | XXXXX | XXXXXX | XXXXXXX |
| XXXX | y | n | XXXXX | XXXXXX | XXXXXXX |
| XXXX | y | n | XXXXX | XXXXXX | XXXXXXX |
| XXXX | y | n | XXXXX | XXXXXX | XXXXXXX |
| XXXX | y | n | XXXXX | XXXXXX | XXXXXXX |
| XXXX | y | n | XXXXX | XXXXXX | XXXXXXX |
| XXXX | y | n | XXXXX | XXXXXX | XXXXXXX |
| XXXX | y | n | XXXXX | XXXXXX | XXXXXXX |
| XXXX | y | n | XXXXX | XXXXXX | XXXXXXX |
| XXXX | y | n | XXXXX | XXXXXX | XXXXXXX |
| XXXX | y | n | XXXXX | XXXXXX | XXXXXXX |
| XXXX | y | n | XXXXX | XXXXXX | XXXXXXX |
| XXXX | y | n | XXXXX | XXXXXX | XXXXXXX |
| XXXX | y | n | XXXXX | XXXXXX | XXXXXXX |
| XXXX | y | n | XXXXX | XXXXXX | XXXXXXX |
| XXXX | y | n | XXXXX | XXXXXX | XXXXXXX |
| XXXX | y | n | XXXXX | XXXXXX | XXXXXXX |
| XXXX | y | n | XXXXX | XXXXXX | XXXXXXX |
| XXXX | y | n | XXXXX | XXXXXX | XXXXXXX |
| XXXX | y | n | XXXXX | XXXXXX | XXXXXXX |
| XXXX | y | n | XXXXX | XXXXXX | XXXXXXX |
| XXXX | y | n | XXXXX | XXXXXX | XXXXXXX |
| XXXX | y | n | XXXXX | XXXXXX | XXXXXXX |
| XXXX | y | n | XXXXX | XXXXXX | XXXXXXX |
| XXXX | y | n | XXXXX | XXXXXX | XXXXXXX |
| XXXX | y | n | XXXXX | XXXXXX | XXXXXXX |
| XXXX | y | n | XXXXX | XXXXXX | XXXXXXX |
| XXXX | y | n | XXXXX | XXXXXX | XXXXXXX |
| XXXX | y | n | XXXXX | XXXXXX | XXXXXXX |
| XXXX | y | n | XXXXX | XXXXXX | XXXXXXX |
| XXXX | y | n | XXXXX | XXXXXX | XXXXXXX |
| XXXX | y | n | XXXXX | XXXXXX | XXXXXXX |
| XXXX | y | n | XXXXX | XXXXXX | XXXXXXX |
| XXXX | y | n | XXXXX | XXXXXX | XXXXXXX |
| XXXX | y | n | XXXXX | XXXXXX | XXXXXXX |
| XXXX | y | n | XXXXX | XXXXXX | XXXXXXX |
| XXXX | y | n | XXXXX | XXXXXX | XXXXXXX |
| XXXX | y | n | XXXXX | XXXXXX | XXXXXXX |

| | | | | | |
|---|---|---|---|---|---|
| XXXX | y | n | XXXXX | XXXXXX | XXXXXXX |
| XXXX | y | n | XXXXX | XXXXXX | XXXXXXX |
| XXXX | y | n | XXXXX | XXXXXX | XXXXXXX |
| XXXX | y | n | XXXXX | XXXXXX | XXXXXXX |
| XXXX | y | n | XXXXX | XXXXXX | XXXXXXX |
| XXXX | y | n | XXXXX | XXXXXX | XXXXXXX |
| XXXX | y | n | XXXXX | XXXXXX | XXXXXXX |
| XXXX | y | n | XXXXX | XXXXXX | XXXXXXX |
| XXXX | y | n | XXXXX | XXXXXX | XXXXXXX |
| XXXX | y | n | XXXXX | XXXXXX | XXXXXXX |
| XXXX | y | n | XXXXX | XXXXXX | XXXXXXX |
| XXXX | y | n | XXXXX | XXXXXX | XXXXXXX |
| XXXX | y | n | XXXXX | XXXXXX | XXXXXXX |
| XXXX | y | n | XXXXX | XXXXXX | XXXXXXX |
| XXXX | y | n | XXXXX | XXXXXX | XXXXXXX |
| XXXX | y | n | XXXXX | XXXXXX | XXXXXXX |
| XXXX | y | n | XXXXX | XXXXXX | XXXXXXX |
| XXXX | y | n | XXXXX | XXXXXX | XXXXXXX |
| XXXX | y | n | XXXXX | XXXXXX | XXXXXXX |
| XXXX | y | n | XXXXX | XXXXXX | XXXXXXX |
| XXXX | y | n | XXXXX | XXXXXX | XXXXXXX |
| XXXX | y | n | XXXXX | XXXXXX | XXXXXXX |
| XXXX | y | n | XXXXX | XXXXXX | XXXXXXX |
| XXXX | y | n | XXXXX | XXXXXX | XXXXXXX |
| XXXX | y | n | XXXXX | XXXXXX | XXXXXXX |
| XXXX | y | n | XXXXX | XXXXXX | XXXXXXX |
| XXXX | y | n | XXXXX | XXXXXX | XXXXXXX |
| XXXX | y | n | XXXXX | XXXXXX | XXXXXXX |
| XXXX | y | n | XXXXX | XXXXXX | XXXXXXX |
| XXXX | n | N | XXXXX | XXXXXX | XXXXXXX |
| XXXX | n | N | XXXXX | XXXXXX | XXXXXXX |
| XXXX | n | N | XXXXX | XXXXXX | XXXXXXX |
| XXXX | n | N | XXXXX | XXXXXX | XXXXXXX |
| XXXX | n | N | XXXXX | XXXXXX | XXXXXXX |
| XXXX | n | N | XXXXX | XXXXXX | XXXXXXX |
| XXXX | n | N | XXXXX | XXXXXX | XXXXXXX |
| XXXX | n | N | XXXXX | XXXXXX | XXXXXXX |
| XXXX | n | n | XXXXX | XXXXXX | XXXXXXX |
| XXXX | n | n | XXXXX | XXXXXX | XXXXXXX |
| XXXX | n | N | XXXXX | XXXXXX | XXXXXXX |
| XXXX | n | N | XXXXX | XXXXXX | XXXXXXX |
| XXXX | na | N | XXXXX | XXXXXX | XXXXXXX |
| XXXX | na | N | XXXXX | XXXXXX | XXXXXXX |
| XXXX | na | N | XXXXX | XXXXXX | XXXXXXX |
| XXXX | na | N | XXXXX | XXXXXX | XXXXXXX |
| XXXX | na | N | XXXXX | XXXXXX | XXXXXXX |
| XXXX | na | N | XXXXX | XXXXXX | XXXXXXX |
| XXXX | na | n | XXXXX | XXXXXX | XXXXXXX |
| XXXX | na | n | XXXXX | XXXXXX | XXXXXXX |
| XXXX | na | N | XXXXX | XXXXXX | XXXXXXX |

| | | | | | |
|---|---|---|---|---|---|
| XXXX | na | N | XXXXX | XXXXXX | XXXXXXX |
| XXXX | na | N | XXXXX | XXXXXX | XXXXXXX |
| XXXX | na | N | XXXXX | XXXXXX | XXXXXXX |
| XXXX | na | N | XXXXX | XXXXXX | XXXXXXX |
| XXXX | na | N | XXXXX | XXXXXX | XXXXXXX |
| XXXX | na | N | XXXXX | XXXXXX | XXXXXXX |
| XXXX | na | N | XXXXX | XXXXXX | XXXXXXX |
| XXXX | na | N | XXXXX | XXXXXX | XXXXXXX |
| XXXX | na | N | XXXXX | XXXXXX | XXXXXXX |
| XXXX | na | N | XXXXX | XXXXXX | XXXXXXX |
| XXXX | y | n | XXXXX | XXXXXX | XXXXXXX |
| XXXX | y | n | XXXXX | XXXXXX | XXXXXXX |
| XXXX | y | n | XXXXX | XXXXXX | XXXXXXX |
| XXXX | y | n | XXXXX | XXXXXX | XXXXXXX |
| XXXX | y | n | XXXXX | XXXXXX | XXXXXXX |
| XXXX | y | n | XXXXX | XXXXXX | XXXXXXX |
| XXXX | y | n | XXXXX | XXXXXX | XXXXXXX |
| XXXX | y | n | XXXXX | XXXXXX | XXXXXXX |
| XXXX | y | n | XXXXX | XXXXXX | XXXXXXX |
| XXXX | y | n | XXXXX | XXXXXX | XXXXXXX |
| XXXX | y | n | XXXXX | XXXXXX | XXXXXXX |
| XXXX | y | n | XXXXX | XXXXXX | XXXXXXX |
| XXXX | y | n | XXXXX | XXXXXX | XXXXXXX |
| XXXX | y | n | XXXXX | XXXXXX | XXXXXXX |
| XXXX | y | n | XXXXX | XXXXXX | XXXXXXX |
| XXXX | y | n | XXXXX | XXXXXX | XXXXXXX |
| XXXX | y | n | XXXXX | XXXXXX | XXXXXXX |
| XXXX | y | n | XXXXX | XXXXXX | XXXXXXX |
| XXXX | y | n | XXXXX | XXXXXX | XXXXXXX |
| XXXX | y | n | XXXXX | XXXXXX | XXXXXXX |
| XXXX | y | n | XXXXX | XXXXXX | XXXXXXX |
| XXXX | y | n | XXXXX | XXXXXX | XXXXXXX |
| XXXX | y | n | XXXXX | XXXXXX | XXXXXXX |
| XXXX | y | n | XXXXX | XXXXXX | XXXXXXX |

| |
|---|
| coordinateE |
| 7.47968 |
| 7.52716 |
| 7.53534 |
| 7.57523 |
| 7.58727 |
| 7.514 |
| 7.58727 |
| 7.52716 |
| 7.58727 |
| 7.49865 |
| 7.47968 |
| 7.52716 |
| 7.49823 |
| 7.49565 |
| 7.57523 |
| 7.4931 |
| 7.58727 |
| 7.514 |
| 7.52928 |
| 7.52716 |
| 7.49565 |
| 7.4931 |
| 7.58727 |
| 7.58271 |
| 7.514 |
| 7.49474 |
| 7.52198 |
| 7.48758 |
| 7.47795 |
| 0 |
| 7.52716 |
| 7.49474 |
| 7.49319 |
| 7.50597 |
| 7.49865 |
| 7.49865 |
| 7.53534 |
| 7.57523 |
| 7.52198 |
| 7.49319 |
| 7.50597 |
| 7.52928 |
| 7.50048 |
| 0 |
| 7.49474 |
| 7.49808 |

| |
|---|
| 7.514 |
| 7.52716 |
| 7.49565 |
| 7.4931 |
| 7.514 |
| 7.49808 |
| 7.4975 |
| 7.49319 |
| 7.49865 |
| 7.47968 |
| 7.58727 |
| 7.52716 |
| 7.47968 |
| 7.52716 |
| 7.47795 |
| 7.52928 |
| 7.47968 |
| 7.57523 |
| 7.49808 |
| 7.52928 |
| 7.47968 |
| 7.57523 |
| 7.4931 |
| 7.47795 |
| 7.47968 |
| 7.4931 |
| 7.47968 |
| 7.52716 |
| 7.49565 |
| 7.49865 |
| 7.52928 |
| 7.47968 |
| 7.52716 |
| 7.50597 |
| 7.47968 |
| 7.52716 |
| 7.47968 |
| 7.52716 |
| 7.49823 |
| 7.49565 |
| 7.4975 |
| 7.47968 |
| 7.52716 |
| 7.49823 |
| 7.53534 |
| 7.57523 |
| 7.4931 |
| 7.58727 |
| 7.58271 |
| 7.514 |

| |
|---|
| 7.49474 |
| 7.4975 |
| 7.47795 |
| 7.49865 |
| 7.52928 |
| 0 |
| 7.47968 |
| 7.52716 |
| 7.57523 |
| 7.49865 |
| 7.52716 |
| 7.47968 |
| 7.52716 |
| 7.58727 |
| 7.47795 |
| 7.49865 |
| 7.52928 |
| 0 |
| 7.47968 |
| 7.52716 |
| 7.49565 |
| 7.58727 |
| 7.47968 |
| 7.52716 |
| 7.49565 |
| 7.4931 |
| 7.58727 |
| 7.47795 |
| 7.49865 |
| 7.57523 |
| 7.48758 |
| 7.49319 |
| 7.52928 |
| 7.52928 |
| 7.53534 |
| 7.52198 |
| 7.49865 |
| 7.49319 |
| 7.4931 |
| 7.4931 |
| 7.57523 |
| 7.49474 |
| 7.4975 |
| 7.52716 |
| 7.49865 |
| 7.50048 |
| 0 |
| 7.47968 |
| 7.52716 |

| |
|---|
| 7.49565 |
| 7.58727 |
| 7.514 |
| 7.49808 |
| 7.47968 |
| 7.50048 |
| 7.49808 |
| 7.52716 |
| 7.47795 |
| 7.4931 |
| 7.57523 |
| 7.47968 |
| 7.49808 |
| 7.49865 |
| 7.47968 |
| 7.49823 |
| 7.49565 |
| 7.4931 |
| 7.49865 |
| 7.52928 |
| 7.48646 |
| 7.47968 |
| 7.47968 |
| 7.47968 |
| 7.4931 |
| 7.47968 |
| 7.52716 |
| 7.4931 |
| 7.49865 |
| 7.48646 |
| 7.47968 |
| 7.52716 |
| 7.53534 |
| 7.49565 |
| 7.57523 |
| 7.4931 |
| 7.58727 |
| 7.58271 |
| 7.49474 |
| 7.4975 |
| 7.52198 |
| 7.48758 |
| 7.49319 |
| 7.52928 |
| 0 |
| 7.47968 |
| 7.52716 |
| 7.49565 |

| |
|---|
| 7.57523 |
| 7.4931 |
| 7.514 |
| 7.49319 |
| 7.47795 |
| 7.47968 |
| 7.57523 |

| packsize | retailprice | UniqueKey | dateofcapt | AFDACregno |
|---|---|---|---|---|
| 24 | 550 | 1202 | 21-Jan-13 | XXXXXXXX |
| 24 | 500 | 399 | 09-Jan-13 | XXXXXXXX |
| 24 | 500 | 462 | 14-Jan-13 | XXXXXXXX |
| 6 | 350 | 833 | 17-Jan-13 | XXXXXXXX |
| 6 | 250 | 1015 | 17-Jan-13 | XXXXXXXX |
| 6 | 300 | 357 | 07-Jan-13 | XXXXXXXX |
| 6 | 150 | 505 | 10-Jan-13 | XXXXXXXX |
| 6 | 150 | 719 | 16-Jan-13 | XXXXXXXX |
| 6 | 200 | 983 | 17-Jan-13 | XXXXXXXX |
| 6 | 100 | 517 | 16-Jan-13 | XXXXXXXX |
| 6 | 200 | 1229 | 21-Jan-13 | XXXXXXXX |
| 6 | | 701 | 16-Jan-13 | XXXXXXXX |
| 6 | 100 | 1144 | 21-Jan-13 | XXXXXXXX |
| 6 | 200 | 521 | 16-Jan-13 | XXXXXXXX |
| 6 | 200 | 488 | 09-Jan-13 | XXXXXXXX |
| 6 | 350 | 715 | 16-Jan-13 | XXXXXXXX |
| 6 | 150 | 1237 | 21-Jan-13 | XXXXXXXX |
| 6 | | 678 | 16-Jan-13 | XXXXXXXX |
| 6 | 100 | 376 | 07-Jan-13 | XXXXXXXX |
| 6 | 150 | 1069 | 21-Jan-13 | XXXXXXXX |
| 6 | 150 | 1073 | 21-Jan-13 | XXXXXXXX |
| 6 | 150 | 616 | 15-Jan-13 | XXXXXXXX |
| 6 | 300 | 1254 | 22-Jan-13 | XXXXXXXX |
| 6 | 150 | 510 | 09-Jan-13 | XXXXXXXX |
| 6 | | 706 | 16-Jan-13 | XXXXXXXX |
| 6 | | 815 | 22-Jan-13 | XXXXXXXX |
| 6 | 300 | 598 | 16-Jan-13 | XXXXXXXX |
| 6 | | 750 | 16-Jan-13 | XXXXXXXX |
| 6 | 300 | 775 | 22-Jan-13 | XXXXXXXX |
| 6 | 100 | 1107 | 21-Jan-13 | XXXXXXXX |
| 6 | 150 | 333 | 07-Jan-13 | XXXXXXXX |
| 6 | 200 | 358 | 07-Jan-13 | XXXXXXXX |
| 6 | 200 | 752 | 21-Jan-13 | XXXXXXXX |
| 6 | 300 | 448 | 09-Jan-13 | XXXXXXXX |
| 6 | 150 | 861 | 17-Jan-13 | XXXXXXXX |
| 6 | 300 | 607 | 15-Jan-13 | XXXXXXXX |
| 6 | | 743 | 16-Jan-13 | XXXXXXXX |
| 6 | 150 | 350 | 08-Jan-13 | XXXXXXXX |
| 6 | 200 | 351 | 08-Jan-13 | XXXXXXXX |

| | | | | |
|---|---|---|---|---|
| 6 | 150 | 373 | 08-Jan-13 | XXXXXXXX |
| 6 | 250 | 452 | 09-Jan-13 | XXXXXXXX |
| 6 | 150 | 590 | 17-Jan-13 | XXXXXXXX |
| 6 | 150 | 513 | 10-Jan-13 | XXXXXXXX |
| 6 | | 1185 | 21-Jan-13 | XXXXXXXX |
| 6 | 100 | 704 | 21-Jan-13 | XXXXXXXX |
| 6 | | 728 | 16-Jan-13 | XXXXXXXX |
| 12 | 300 | 1016 | 17-Jan-13 | XXXXXXXX |
| 12 | 200 | 1238 | 22-Jan-13 | XXXXXXXX |
| 12 | 250 | 718 | 16-Jan-13 | XXXXXXXX |
| 12 | | 721 | 16-Jan-13 | XXXXXXXX |
| 12 | 200 | 692 | 21-Jan-13 | XXXXXXXX |
| 12 | 200 | 554 | 16-Jan-13 | XXXXXXXX |
| 12 | | 814 | 22-Jan-13 | XXXXXXXX |
| 12 | | 751 | 16-Jan-13 | XXXXXXXX |
| 12 | 250 | 1169 | 21-Jan-13 | XXXXXXXX |
| 12 | 200 | 335 | 07-Jan-13 | XXXXXXXX |
| 12 | 300 | 753 | 21-Jan-13 | XXXXXXXX |
| 12 | 400 | 644 | 17-Jan-13 | XXXXXXXX |
| 12 | 250 | 862 | 17-Jan-13 | XXXXXXXX |
| 12 | | 746 | 16-Jan-13 | XXXXXXXX |
| 12 | | 1184 | 21-Jan-13 | XXXXXXXX |
| 12 | | 730 | 16-Jan-13 | XXXXXXXX |
| 12 | | | | XXXXXXXX |
| 18 | 350 | 837 | 17-Jan-13 | XXXXXXXX |
| 18 | 350 | 1195 | 21-Jan-13 | XXXXXXXX |
| 18 | 250 | 1211 | 21-Jan-13 | XXXXXXXX |
| 18 | | 1137 | 21-Jan-13 | XXXXXXXX |
| 18 | | 1138 | 21-Jan-13 | XXXXXXXX |
| 18 | 300 | 748 | 21-Jan-13 | XXXXXXXX |
| 18 | 400 | 506 | 10-Jan-13 | XXXXXXXX |
| 18 | 300 | 1043 | 21-Jan-13 | XXXXXXXX |
| 18 | 250 | 516 | 16-Jan-13 | XXXXXXXX |
| 18 | 350 | 1181 | 21-Jan-13 | XXXXXXXX |
| 18 | | 703 | 16-Jan-13 | XXXXXXXX |
| 18 | 300 | 635 | 15-Jan-13 | XXXXXXXX |
| 18 | 250 | 1145 | 21-Jan-13 | XXXXXXXX |
| 18 | 250 | 770 | 16-Jan-13 | XXXXXXXX |
| 18 | | 683 | 16-Jan-13 | XXXXXXXX |
| 18 | 450 | 1080 | 21-Jan-13 | XXXXXXXX |
| 18 | 300 | 584 | 15-Jan-13 | XXXXXXXX |
| 18 | 350 | 591 | 15-Jan-13 | XXXXXXXX |
| 18 | 350 | 1255 | 22-Jan-13 | XXXXXXXX |
| 18 | 250 | 511 | 09-Jan-13 | XXXXXXXX |
| 18 | 300 | 603 | 17-Jan-13 | XXXXXXXX |
| 18 | 250 | 661 | 18-Jan-13 | XXXXXXXX |
| 18 | | 708 | 16-Jan-13 | XXXXXXXX |
| 18 | | 816 | 22-Jan-13 | XXXXXXXX |
| 18 | | 752 | 16-Jan-13 | XXXXXXXX |
| 18 | 400 | 776 | 22-Jan-13 | XXXXXXXX |

| | | | | |
|---|---|---|---|---|
| 18 | | 673 | 16-Jan-13 | XXXXXXXX |
| 18 | 250 | 694 | 21-Jan-13 | XXXXXXXX |
| 18 | 200 | 1104 | 21-Jan-13 | XXXXXXXX |
| 18 | 400 | 1017 | 17-Jan-13 | XXXXXXXX |
| 18 | | 336 | 07-Jan-13 | XXXXXXXX |
| 18 | 300 | 1045 | 21-Jan-13 | XXXXXXXX |
| 18 | 400 | 754 | 21-Jan-13 | XXXXXXXX |
| 18 | | 744 | 16-Jan-13 | XXXXXXXX |
| 18 | 400 | 677 | 17-Jan-13 | XXXXXXXX |
| 18 | 300 | 1172 | 21-Jan-13 | XXXXXXXX |
| 18 | 300 | 717 | 21-Jan-13 | XXXXXXXX |
| 18 | | 1182 | 21-Jan-13 | XXXXXXXX |
| 18 | 100 | 700 | 21-Jan-13 | XXXXXXXX |
| 18 | 300 | 363 | 07-Jan-13 | XXXXXXXX |
| 18 | | 727 | 16-Jan-13 | XXXXXXXX |
| 24 | 300 | 330 | 07-Jan-13 | XXXXXXXX |
| 24 | 400 | 398 | 08-Jan-13 | XXXXXXXX |
| 24 | 350 | 549 | 16-Jan-13 | XXXXXXXX |
| 24 | 450 | 1194 | 21-Jan-13 | XXXXXXXX |
| 24 | 500 | 339 | 07-Jan-13 | XXXXXXXX |
| 24 | 350 | 411 | 08-Jan-13 | XXXXXXXX |
| 24 | 250 | 1249 | 22-Jan-13 | XXXXXXXX |
| 24 | 400 | | 08-Jan-13 | XXXXXXXX |
| 24 | 800 | 1205 | 21-Jan-13 | XXXXXXXX |
| 24 | 350 | 353 | 07-Jan-13 | XXXXXXXX |
| 24 | 350 | 386 | 07-Jan-13 | XXXXXXXX |
| 24 | 350 | 800 | 16-Jan-13 | XXXXXXXX |
| 24 | 550 | 653 | 18-Jan-13 | XXXXXXXX |
| 24 | 300 | 469 | 15-Jan-13 | XXXXXXXX |
| 24 | 400 | 618 | 15-Jan-13 | XXXXXXXX |
| 24 | 350 | 565 | 14-Jan-13 | XXXXXXXX |
| 24 | 300 | 515 | 15-Jan-13 | XXXXXXXX |
| 24 | 400 | 635 | 17-Jan-13 | XXXXXXXX |
| 24 | 600 | 886 | 17-Jan-13 | XXXXXXXX |
| 24 | 250 | 768 | 16-Jan-13 | XXXXXXXX |
| 24 | 500 | 947 | 17-Jan-13 | XXXXXXXX |
| 24 | 700 | 627 | 17-Jan-13 | XXXXXXXX |
| 24 | | 682 | 16-Jan-13 | XXXXXXXX |
| 24 | 300 | 375 | 07-Jan-13 | XXXXXXXX |
| 24 | 450 | 1076 | 21-Jan-13 | XXXXXXXX |
| 24 | 250 | 784 | 16-Jan-13 | XXXXXXXX |
| 24 | 300 | 852 | 17-Jan-13 | XXXXXXXX |
| 24 | 400 | 486 | 10-Jan-13 | XXXXXXXX |
| 24 | 450 | 659 | 18-Jan-13 | XXXXXXXX |
| 24 | | 707 | 16-Jan-13 | XXXXXXXX |
| 24 | | 817 | 22-Jan-13 | XXXXXXXX |
| 24 | 400 | 578 | 17-Jan-13 | XXXXXXXX |
| 24 | 500 | 616 | 16-Jan-13 | XXXXXXXX |
| 24 | | 753 | 16-Jan-13 | XXXXXXXX |
| 24 | 400 | 355 | 07-Jan-13 | XXXXXXXX |

| | | | | |
|---|---|---|---|---|
| 24 | 450 | 424 | 08-Jan-13 | XXXXXXXX |
| 24 | 300 | 1034 | 21-Jan-13 | XXXXXXXX |
| 24 | 250 | 1105 | 21-Jan-13 | XXXXXXXX |
| 24 | 450 | 755 | 21-Jan-13 | XXXXXXXX |
| 24 | 300 | 697 | 21-Jan-13 | XXXXXXXX |
| 24 | 350 | 873 | 17-Jan-13 | XXXXXXXX |
| 24 | 600 | 621 | 15-Jan-13 | XXXXXXXX |
| 24 | 500 | 863 | 17-Jan-13 | XXXXXXXX |
| 24 | 450 | 605 | 15-Jan-13 | XXXXXXXX |
| 24 | | 745 | 16-Jan-13 | XXXXXXXX |
| 24 | 400 | 440 | 14-Jan-13 | XXXXXXXX |
| 24 | 350 | 652 | 17-Jan-13 | XXXXXXXX |
| 24 | 300 | 735 | 16-Jan-13 | XXXXXXXX |
| 24 | | 1183 | 21-Jan-13 | XXXXXXXX |
| 24 | 350 | 558 | 14-Jan-13 | XXXXXXXX |
| 24 | 350 | 426 | 14-Jan-13 | XXXXXXXX |
| 24 | 300 | 672 | 21-Jan-13 | XXXXXXXX |
| 24 | | 729 | 16-Jan-13 | XXXXXXXX |
| 24 | 600 | 677 | 21-Jan-13 | XXXXXXXX |
| 24 | 600 | 928 | 17-Jan-13 | XXXXXXXX |
| 24 | 550 | 919 | 17-Jan-13 | XXXXXXXX |
| 24 | 950 | 614 | 15-Jan-13 | XXXXXXXX |
| 24 | 500 | 644 | 18-Jan-13 | XXXXXXXX |
| 24 | 550 | 897 | 17-Jan-13 | XXXXXXXX |
| 24 | 550 | 472 | 10-Jan-13 | XXXXXXXX |
| 24 | 750 | 475 | 15-Jan-13 | XXXXXXXX |
| 24 | 550 | 858 | 17-Jan-13 | XXXXXXXX |
| 24 | 700 | 658 | 18-Jan-13 | XXXXXXXX |
| 24 | 200 | 353 | 07-Jan-13 | XXXXXXXX |
| 24 | 1000 | 356 | 08-Jan-13 | XXXXXXXX |
| 24 | 650 | 1192 | 21-Jan-13 | XXXXXXXX |
| 24 | 500 | 1227 | 21-Jan-13 | XXXXXXXX |
| 24 | | | | XXXXXXXX |
| 24 | 650 | 829 | 17-Jan-13 | XXXXXXXX |
| 24 | 750 | 391 | 07-Jan-13 | XXXXXXXX |
| 24 | 600 | 588 | 15-Jan-13 | XXXXXXXX |
| 24 | 600 | 432 | 08-Jan-13 | XXXXXXXX |
| 24 | 650 | 810 | 22-Jan-13 | XXXXXXXX |
| 24 | 650 | 441 | 14-Jan-13 | XXXXXXXX |
| 24 | 600 | 857 | 17-Jan-13 | XXXXXXXX |
| 24 | 600 | 483 | 10-Jan-13 | XXXXXXXX |
| 24 | 700 | 875 | 17-Jan-13 | XXXXXXXX |
| 24 | 700 | 880 | 17-Jan-13 | XXXXXXXX |
| 24 | 650 | 429 | 08-Jan-13 | XXXXXXXX |
| 24 | 400 | 627 | 15-Jan-13 | XXXXXXXX |
| 12 | 300 | 386 | 08-Jan-13 | XXXXXXXX |
| 24 | 400 | 385 | 07-Jan-13 | XXXXXXXX |
| 24 | 300 | 416 | 10-Jan-13 | XXXXXXXX |
| 24 | 300 | 907 | 17-Jan-13 | XXXXXXXX |
| 24 | 350 | 723 | 16-Jan-13 | XXXXXXXX |

| | | | | |
|---|---|---|---|---|
| 24 | 350 | 992 | 17-Jan-13 | XXXXXXXX |
| 24 | 350 | 401 | 08-Jan-13 | XXXXXXXX |
| 24 | 650 | 334 | 07-Jan-13 | XXXXXXXX |
| 24 | 350 | 525 | 11-Jan-13 | XXXXXXXX |
| 12 | 700 | 449 | 09-Jan-13 | XXXXXXXX |
| 12 | 300 | 507 | 15-Jan-13 | XXXXXXXX |
| 12 | 250 | 519 | 09-Jan-13 | XXXXXXXX |
| 24 | 800 | 708 | 21-Jan-13 | XXXXXXXX |
| 24 | 600 | 975 | 17-Jan-13 | XXXXXXXX |
| 24 | 600 | 412 | 10-Jan-13 | XXXXXXXX |
| 24 | 400 | 744 | 21-Jan-13 | XXXXXXXX |
| 24 | 650 | 811 | 22-Jan-13 | XXXXXXXX |
| 24 | 600 | 609 | 15-Jan-13 | XXXXXXXX |
| 24 | 650 | 859 | 17-Jan-13 | XXXXXXXX |
| 24 | 600 | 473 | 10-Jan-13 | XXXXXXXX |
| 24 | 450 | 476 | 09-Jan-13 | XXXXXXXX |
| 24 | 600 | 714 | 21-Jan-13 | XXXXXXXX |
| 24 | 500 | 619 | 17-Jan-13 | XXXXXXXX |
| 24 | 500 | 441 | 08-Jan-13 | XXXXXXXX |
| 24 | 600 | 495 | 09-Jan-13 | XXXXXXXX |
| 24 | 550 | 413 | 10-Jan-13 | XXXXXXXX |
| 24 | 700 | 484 | 09-Jan-13 | XXXXXXXX |
| 24 | 600 | 581 | 16-Jan-13 | XXXXXXXX |
| 24 | 650 | 612 | 17-Jan-13 | XXXXXXXX |
| 24 | 900 | 678 | 21-Jan-13 | XXXXXXXX |
| 24 | 1000 | 545 | 16-Jan-13 | XXXXXXXX |
| 24 | 480 | 372 | 07-Jan-13 | XXXXXXXX |
| 24 | 900 | 1273 | 22-Jan-13 | XXXXXXXX |
| 24 | 900 | 492 | 15-Jan-13 | XXXXXXXX |
| 24 | 950 | 980 | 17-Jan-13 | XXXXXXXX |
| 24 | 1100 | 933 | 17-Jan-13 | XXXXXXXX |
| 24 | 950 | 424 | 08-Jan-13 | XXXXXXXX |
| 24 | 900 | 556 | 16-Jan-13 | XXXXXXXX |
| 24 | 1000 | 1085 | 21-Jan-13 | XXXXXXXX |
| 24 | 1000 | 452 | 14-Jan-13 | XXXXXXXX |
| 24 | 1000 | 742 | 21-Jan-13 | XXXXXXXX |
| 24 | 1050 | 1269 | 22-Jan-13 | XXXXXXXX |
| 24 | 1200 | 1082 | 21-Jan-13 | XXXXXXXX |
| 24 | 1000 | 548 | 14-Jan-13 | XXXXXXXX |
| 24 | 1000 | 577 | 14-Jan-13 | XXXXXXXX |
| 24 | 980 | 973 | 17-Jan-13 | XXXXXXXX |
| 24 | 900 | 787 | 16-Jan-13 | XXXXXXXX |
| 24 | 900 | 854 | 17-Jan-13 | XXXXXXXX |
| 24 | 1150 | 594 | 16-Jan-13 | XXXXXXXX |
| 24 | 1000 | 936 | 17-Jan-13 | XXXXXXXX |
| 24 | 1000 | 442 | 08-Jan-13 | XXXXXXXX |
| 24 | 950 | 331 | 07-Jan-13 | XXXXXXXX |
| 24 | 1200 | 1046 | 21-Jan-13 | XXXXXXXX |
| 24 | 1000 | 1283 | 22-Jan-13 | XXXXXXXX |
| 24 | 950 | 516 | 11-Jan-13 | XXXXXXXX |

| | | | | |
|---|---|---|---|---|
| 24 | 1000 | 530 | 09-Jan-13 | XXXXXXXX |
| 24 | 950 | 872 | 17-Jan-13 | XXXXXXXX |
| 24 | 900 | 640 | 17-Jan-13 | XXXXXXXX |
| 24 | 1000 | 439 | 14-Jan-13 | XXXXXXXX |
| 24 | 1000 | 1126 | 21-Jan-13 | XXXXXXXX |
| 24 | 960 | 842 | 17-Jan-13 | XXXXXXXX |
| 24 | 1000 | 455 | 09-Jan-13 | XXXXXXXX |
| 24 | 1100 | 800 | 22-Jan-13 | XXXXXXXX |
| 24 | 1020 | 734 | 21-Jan-13 | XXXXXXXX |
| 24 | 1200 | 647 | 15-Jan-13 | XXXXXXXX |
| 24 | 1200 | 667 | 15-Jan-13 | XXXXXXXX |
| 24 | 1000 | 672 | 15-Jan-13 | XXXXXXXX |
| 24 | 1000 | 435 | 08-Jan-13 | XXXXXXXX |
| 24 | 1000 | 1301 | 22-Jan-13 | XXXXXXXX |
| 18 | 350 | 596 | 15-Jan-13 | XXXXXXXX |
| 18 | 350 | 497 | 09-Jan-13 | XXXXXXXX |
| 18 | 300 | 1228 | 21-Jan-13 | XXXXXXXX |
| 18 | 300 | 498 | 10-Jan-13 | XXXXXXXX |
| 18 | 300 | 675 | 17-Jan-13 | XXXXXXXX |
| 18 | 300 | 345 | 07-Jan-13 | XXXXXXXX |
| 18 | 350 | 698 | 21-Jan-13 | XXXXXXXX |
| 18 | 300 | 1117 | 21-Jan-13 | XXXXXXXX |
| 18 | 950 | 517 | 11-Jan-13 | XXXXXXXX |
| 18 | 300 | 366 | 08-Jan-13 | XXXXXXXX |
| 18 | 300 | 670 | 15-Jan-13 | XXXXXXXX |
| 18 | 250 | 695 | 16-Jan-13 | XXXXXXXX |
| 24 | 450 | 838 | 17-Jan-13 | XXXXXXXX |
| 24 | 300 | 1277 | 22-Jan-13 | XXXXXXXX |
| 24 | 600 | 1197 | 21-Jan-13 | XXXXXXXX |
| 24 | 450 | 341 | 07-Jan-13 | XXXXXXXX |
| 24 | 400 | 1250 | 22-Jan-13 | XXXXXXXX |
| 24 | 350 | 384 | 07-Jan-13 | XXXXXXXX |
| 24 | 500 | 932 | 17-Jan-13 | XXXXXXXX |
| 24 | 850 | 792 | 16-Jan-13 | XXXXXXXX |
| 24 | 300 | 615 | 15-Jan-13 | XXXXXXXX |
| 24 | 300 | 426 | 08-Jan-13 | XXXXXXXX |
| 24 | 300 | 1285 | 22-Jan-13 | XXXXXXXX |
| 24 | 300 | 894 | 17-Jan-13 | XXXXXXXX |
| 24 | 300 | 924 | 17-Jan-13 | XXXXXXXX |
| 24 | 550 | 541 | 09-Jan-13 | XXXXXXXX |
| 24 | 500 | 946 | 17-Jan-13 | XXXXXXXX |
| 24 | 400 | 1089 | 21-Jan-13 | XXXXXXXX |
| 24 | 350 | 687 | 16-Jan-13 | XXXXXXXX |
| 24 | 350 | 1164 | 21-Jan-13 | XXXXXXXX |
| 24 | 300 | 374 | 07-Jan-13 | XXXXXXXX |
| 24 | 350 | 1258 | 22-Jan-13 | XXXXXXXX |
| 24 | 480 | 963 | 17-Jan-13 | XXXXXXXX |
| 24 | 400 | 1214 | 21-Jan-13 | XXXXXXXX |
| 24 | 450 | 408 | 08-Jan-13 | XXXXXXXX |
| 24 | 400 | 346 | 07-Jan-13 | XXXXXXXX |

| | | | | |
|---|---|---|---|---|
| 24 | 400 | 396 | 08-Jan-13 | XXXXXXXX |
| 24 | 400 | 330 | 07-Jan-13 | XXXXXXXX |
| 24 | 400 | 1014 | 17-Jan-13 | XXXXXXXX |
| 24 | 350 | 501 | 09-Jan-13 | XXXXXXXX |
| 24 | 500 | 474 | 09-Jan-13 | XXXXXXXX |
| 24 | 700 | 884 | 17-Jan-13 | XXXXXXXX |
| 24 | 400 | 819 | 17-Jan-13 | XXXXXXXX |
| 24 | 500 | 1168 | 21-Jan-13 | XXXXXXXX |
| 24 | 500 | 451 | 09-Jan-13 | XXXXXXXX |
| 24 | 500 | 1190 | 21-Jan-13 | XXXXXXXX |
| 24 | 700 | 646 | 15-Jan-13 | XXXXXXXX |
| 24 | 300 | 1062 | 21-Jan-13 | XXXXXXXX |
| 24 | 500 | 789 | 22-Jan-13 | XXXXXXXX |
| 24 | 250 | 409 | 08-Jan-13 | XXXXXXXX |
| 24 | 600 | 389 | 08-Jan-13 | XXXXXXXX |
| 24 | 300 | 689 | 21-Jan-13 | XXXXXXXX |
| 24 | 400 | 691 | 21-Jan-13 | XXXXXXXX |
| 24 | 350 | 467 | 10-Jan-13 | XXXXXXXX |
| 24 | 350 | 374 | 07-Jan-13 | XXXXXXXX |
| 24 | 250 | 1118 | 21-Jan-13 | XXXXXXXX |
| 24 | 400 | 688 | 21-Jan-13 | XXXXXXXX |
| 24 | 350 | 497 | 10-Jan-13 | XXXXXXXX |
| 24 | 600 | 583 | 15-Jan-13 | XXXXXXXX |
| 24 | 500 | 531 | 16-Jan-13 | XXXXXXXX |
| 24 | 300 | 606 | 17-Jan-13 | XXXXXXXX |
| 24 | 500 | 674 | 17-Jan-13 | XXXXXXXX |
| 24 | 400 | 581 | 17-Jan-13 | XXXXXXXX |
| 24 | 400 | 387 | 09-Jan-13 | XXXXXXXX |
| 24 | 300 | 596 | 17-Jan-13 | XXXXXXXX |
| 24 | 400 | 615 | 16-Jan-13 | XXXXXXXX |
| 24 | 350 | 403 | 08-Jan-13 | XXXXXXXX |
| 24 | 400 | 406 | 10-Jan-13 | XXXXXXXX |
| 24 | 300 | 493 | 10-Jan-13 | XXXXXXXX |
| 24 | 400 | 614 | 17-Jan-13 | XXXXXXXX |
| 24 | 350 | 509 | 10-Jan-13 | XXXXXXXX |
| 24 | 520 | 735 | 21-Jan-13 | XXXXXXXX |
| 24 | 400 | 422 | 14-Jan-13 | XXXXXXXX |
| 24 | 300 | 478 | 10-Jan-13 | XXXXXXXX |
| 24 | 350 | 570 | 16-Jan-13 | XXXXXXXX |
| 24 | 350 | 665 | 21-Jan-13 | XXXXXXXX |
| 24 | 500 | 1175 | 21-Jan-13 | XXXXXXXX |
| 6 | 300 | 1012 | 17-Jan-13 | XXXXXXXX |
| 6 | 300 | 1125 | 21-Jan-13 | XXXXXXXX |
| 6 | 200 | 388 | 08-Jan-13 | XXXXXXXX |
| 6 | 200 | | | XXXXXXXX |
| 6 | 200 | 351 | 07-Jan-13 | XXXXXXXX |
| 6 | 250 | 1040 | 21-Jan-13 | XXXXXXXX |
| 6 | 250 | 931 | 17-Jan-13 | XXXXXXXX |
| 6 | 150 | 1287 | 22-Jan-13 | XXXXXXXX |
| 6 | 150 | 1141 | 21-Jan-13 | XXXXXXXX |

| | | | | |
|---|---|---|---|---|
| 6 | 350 | 771 | 16-Jan-13 | XXXXXXXX |
| 6 | 350 | 540 | 09-Jan-13 | XXXXXXXX |
| 6 | 150 | 1235 | 21-Jan-13 | XXXXXXXX |
| 6 | 300 | 485 | 15-Jan-13 | XXXXXXXX |
| 6 | 200 | 499 | 10-Jan-13 | XXXXXXXX |
| 6 | 250 | 788 | 16-Jan-13 | XXXXXXXX |
| 6 | 300 | 585 | 17-Jan-13 | XXXXXXXX |
| 6 | 220 | 597 | 17-Jan-13 | XXXXXXXX |
| 6 | 350 | 407 | 08-Jan-13 | XXXXXXXX |
| 6 | 150 | 343 | 07-Jan-13 | XXXXXXXX |
| 6 | 150 | 400 | 08-Jan-13 | XXXXXXXX |
| 6 | 150 | 1119 | 21-Jan-13 | XXXXXXXX |
| 6 | 200 | 604 | 16-Jan-13 | XXXXXXXX |
| 6 | 200 | 801 | 16-Jan-13 | XXXXXXXX |
| 6 | 350 | 405 | 10-Jan-13 | XXXXXXXX |
| 6 | 200 | 487 | 10-Jan-13 | XXXXXXXX |
| 6 | 1250 | 640 | 15-Jan-13 | XXXXXXXX |
| 6 | 300 | 660 | 15-Jan-13 | XXXXXXXX |
| 6 | 300 | 1097 | 21-Jan-13 | XXXXXXXX |
| 6 | 250 | 347 | 08-Jan-13 | XXXXXXXX |
| 6 | 250 | 1024 | 21-Jan-13 | XXXXXXXX |
| 6 | 180 | 680 | 21-Jan-13 | XXXXXXXX |
| 6 | 200 | 421 | 14-Jan-13 | XXXXXXXX |
| 6 | 500 | 689 | 16-Jan-13 | XXXXXXXX |
| 6 | 250 | 376 | 08-Jan-13 | XXXXXXXX |
| 6 | 250 | 505 | 15-Jan-13 | XXXXXXXX |
| 6 | 100 | 765 | 21-Jan-13 | XXXXXXXX |
| 12 | 300 | 681 | 21-Jan-13 | XXXXXXXX |
| 12 | 300 | 1013 | 17-Jan-13 | XXXXXXXX |
| 12 | 250 | 550 | 16-Jan-13 | XXXXXXXX |
| 12 | 350 | 784 | 22-Jan-13 | XXXXXXXX |
| 12 | 300 | 1124 | 21-Jan-13 | XXXXXXXX |
| 12 | 200 | 1213 | 21-Jan-13 | XXXXXXXX |
| 12 | 250 | 352 | 07-Jan-13 | XXXXXXXX |
| 12 | 250 | 1041 | 21-Jan-13 | XXXXXXXX |
| 12 | 200 | 981 | 17-Jan-13 | XXXXXXXX |
| 12 | 250 | 425 | 08-Jan-13 | XXXXXXXX |
| 12 | 200 | 1286 | 22-Jan-13 | XXXXXXXX |
| 12 | 300 | 895 | 17-Jan-13 | XXXXXXXX |
| 12 | 200 | 633 | 15-Jan-13 | XXXXXXXX |
| 12 | 250 | 437 | 08-Jan-13 | XXXXXXXX |
| 12 | 280 | 725 | 16-Jan-13 | XXXXXXXX |
| 12 | 200 | 607 | 17-Jan-13 | XXXXXXXX |
| 12 | 250 | 614 | 16-Jan-13 | XXXXXXXX |
| 12 | 300 | 346 | 07-Jan-13 | XXXXXXXX |
| 12 | 200 | 344 | 07-Jan-13 | XXXXXXXX |
| 12 | 250 | 393 | 08-Jan-13 | XXXXXXXX |
| 12 | 150 | 1030 | 21-Jan-13 | XXXXXXXX |
| 12 | 250 | 814 | 16-Jan-13 | XXXXXXXX |
| 12 | 300 | 1044 | 21-Jan-13 | XXXXXXXX |

| | | | | |
|---|---|---|---|---|
| 12 | 200 | 359 | 07-Jan-13 | XXXXXXXX |
| 12 | 300 | 696 | 21-Jan-13 | XXXXXXXX |
| 12 | 450 | 447 | 09-Jan-13 | XXXXXXXX |
| 12 | 250 | 865 | 17-Jan-13 | XXXXXXXX |
| 12 | 350 | 407 | 10-Jan-13 | XXXXXXXX |
| 12 | 200 | 505 | 09-Jan-13 | XXXXXXXX |
| 12 | 250 | 461 | 08-Jan-13 | XXXXXXXX |
| 12 | 250 | 396 | 08-Jan-13 | XXXXXXXX |
| 12 | 350 | 367 | 07-Jan-13 | XXXXXXXX |
| 24 | 550 | 912 | 17-Jan-13 | XXXXXXXX |
| 24 | 500 | 454 | 09-Jan-13 | XXXXXXXX |
| 24 | 460 | 727 | 21-Jan-13 | XXXXXXXX |
| 24 | 600 | 913 | 17-Jan-13 | XXXXXXXX |
| 24 | 700 | 453 | 14-Jan-13 | XXXXXXXX |
| 24 | 650 | 743 | 21-Jan-13 | XXXXXXXX |
| 24 | 600 | 966 | 17-Jan-13 | XXXXXXXX |
| 24 | 650 | 941 | 17-Jan-13 | XXXXXXXX |
| 24 | 640 | 529 | 11-Jan-13 | XXXXXXXX |
| 24 | 650 | 586 | 16-Jan-13 | XXXXXXXX |
| 24 | 630 | 475 | 09-Jan-13 | XXXXXXXX |
| 24 | 750 | 1307 | 22-Jan-13 | XXXXXXXX |
| 24 | 750 | 431 | 08-Jan-13 | XXXXXXXX |
| 6 | 300 | 538 | 09-Jan-13 | XXXXXXXX |
| 6 | | 710 | 16-Jan-13 | XXXXXXXX |
| 6 | 200 | 523 | 16-Jan-13 | XXXXXXXX |
| 6 | 250 | 955 | 17-Jan-13 | XXXXXXXX |
| 12 | 300 | 793 | 16-Jan-13 | XXXXXXXX |
| 12 | 150 | 1106 | 21-Jan-13 | XXXXXXXX |
| 12 | 350 | 1008 | 17-Jan-13 | XXXXXXXX |
| 12 | 200 | 706 | 21-Jan-13 | XXXXXXXX |
| 24 | 350 | 1196 | 21-Jan-13 | XXXXXXXX |
| 24 | 350 | 976 | 17-Jan-13 | XXXXXXXX |
| 24 | 350 | 1230 | 21-Jan-13 | XXXXXXXX |
| 24 | 350 | 901 | 17-Jan-13 | XXXXXXXX |
| 24 | 250 | 804 | 16-Jan-13 | XXXXXXXX |
| 24 | 400 | 818 | 17-Jan-13 | XXXXXXXX |
| 24 | 300 | 488 | 10-Jan-13 | XXXXXXXX |
| 24 | 300 | 716 | 21-Jan-13 | XXXXXXXX |
| 24 | 280 | 392 | 08-Jan-13 | XXXXXXXX |
| 24 | 550 | 663 | 17-Jan-13 | XXXXXXXX |
| 24 | 600 | 507 | 09-Jan-13 | XXXXXXXX |
| 12 | 650 | 775 | 16-Jan-13 | XXXXXXXX |
| 24 | 350 | 599 | 17-Jan-13 | XXXXXXXX |
| 12 | | 757 | 16-Jan-13 | XXXXXXXX |
| 6 | 250 | 615 | 15-Jan-13 | XXXXXXXX |
| 6 | 150 | | | XXXXXXXX |
| 6 | 300 | 795 | 16-Jan-13 | XXXXXXXX |
| 6 | 200 | 598 | 15-Jan-13 | XXXXXXXX |
| 6 | 250 | 1322 | 22-Jan-13 | XXXXXXXX |
| 6 | 250 | 1178 | 21-Jan-13 | XXXXXXXX |

| | | | | |
|---|---|---|---|---|
| 6 | 100 | 909 | 17-Jan-13 | XXXXXXXX |
| 6 | 150 | 724 | 16-Jan-13 | XXXXXXXX |
| 6 | 200 | 1146 | 21-Jan-13 | XXXXXXXX |
| 6 | 250 | 769 | 16-Jan-13 | XXXXXXXX |
| 6 | 150 | 480 | 15-Jan-13 | XXXXXXXX |
| 6 | 250 | 512 | 09-Jan-13 | XXXXXXXX |
| 6 | 300 | 660 | 18-Jan-13 | XXXXXXXX |
| 6 | 100 | 592 | 16-Jan-13 | XXXXXXXX |
| 6 | 400 | 349 | 07-Jan-13 | XXXXXXXX |
| 6 | 300 | 499 | 09-Jan-13 | XXXXXXXX |
| 6 | 250 | 660 | 17-Jan-13 | XXXXXXXX |
| 6 | 200 | 733 | 16-Jan-13 | XXXXXXXX |
| 6 | 150 | 669 | 15-Jan-13 | XXXXXXXX |
| 6 | 200 | 425 | 14-Jan-13 | XXXXXXXX |
| 12 | 350 | 835 | 17-Jan-13 | XXXXXXXX |
| 12 | 350 | 1023 | 21-Jan-13 | XXXXXXXX |
| 12 | 150 | | | XXXXXXXX |
| 12 | 200 | 541 | 16-Jan-13 | XXXXXXXX |
| 12 | 250 | 1179 | 21-Jan-13 | XXXXXXXX |
| 12 | 250 | 410 | 10-Jan-13 | XXXXXXXX |
| 12 | 150 | 1233 | 21-Jan-13 | XXXXXXXX |
| 12 | 200 | 949 | 17-Jan-13 | XXXXXXXX |
| 12 | 250 | 529 | 16-Jan-13 | XXXXXXXX |
| 12 | 250 | 608 | 17-Jan-13 | XXXXXXXX |
| 12 | 400 | 937 | 17-Jan-13 | XXXXXXXX |
| 12 | 400 | 656 | 18-Jan-13 | XXXXXXXX |
| 12 | 350 | 773 | 22-Jan-13 | XXXXXXXX |
| 12 | | | | XXXXXXXX |
| 12 | 200 | 697 | 21-Jan-13 | XXXXXXXX |
| 12 | 150 | 1033 | 21-Jan-13 | XXXXXXXX |
| 12 | 250 | 821 | 17-Jan-13 | XXXXXXXX |
| 12 | 250 | 822 | 17-Jan-13 | XXXXXXXX |
| 12 | 160 | 469 | 09-Jan-13 | XXXXXXXX |
| 12 | 250 | 394 | 09-Jan-13 | XXXXXXXX |
| 12 | 200 | 348 | 08-Jan-13 | XXXXXXXX |
| 12 | 120 | 848 | 17-Jan-13 | XXXXXXXX |
| 12 | 200 | 1224 | 21-Jan-13 | XXXXXXXX |
| 18 | 230 | 445 | 14-Jan-13 | XXXXXXXX |
| 18 | 200 | 377 | 08-Jan-13 | XXXXXXXX |
| 18 | 100 | 728 | 21-Jan-13 | XXXXXXXX |
| 24 | 750 | 834 | 17-Jan-13 | XXXXXXXX |
| 24 | 300 | 680 | 21-Jan-13 | XXXXXXXX |
| 24 | 500 | 718 | 21-Jan-13 | XXXXXXXX |
| 24 | 300 | 1003 | 17-Jan-13 | XXXXXXXX |
| 24 | 300 | 548 | 16-Jan-13 | XXXXXXXX |
| 24 | 350 | 1198 | 21-Jan-13 | XXXXXXXX |
| 24 | 600 | 1121 | 21-Jan-13 | XXXXXXXX |
| 24 | 300 | 710 | 21-Jan-13 | XXXXXXXX |
| 24 | 200 | 368 | 07-Jan-13 | XXXXXXXX |
| 24 | 250 | 358 | 07-Jan-13 | XXXXXXXX |

| | | | | |
|---|---|---|---|---|
| 24 | 300 | 383 | 07-Jan-13 | XXXXXXXX |
| 24 | 300 | 1241 | 22-Jan-13 | XXXXXXXX |
| 24 | 350 | 717 | 16-Jan-13 | XXXXXXXX |
| 24 | 350 | 387 | 07-Jan-13 | XXXXXXXX |
| 24 | 30 | 1039 | 21-Jan-13 | XXXXXXXX |
| 24 | 350 | 417 | 14-Jan-13 | XXXXXXXX |
| 24 | 600 | 982 | 17-Jan-13 | XXXXXXXX |
| 24 | 300 | 468 | 14-Jan-13 | XXXXXXXX |
| 24 | 300 | 636 | 17-Jan-13 | XXXXXXXX |
| 24 | 300 | 419 | 08-Jan-13 | XXXXXXXX |
| 24 | 300 | 551 | 16-Jan-13 | XXXXXXXX |
| 24 | 350 | 535 | 09-Jan-13 | XXXXXXXX |
| 24 | 300 | 1150 | 21-Jan-13 | XXXXXXXX |
| 24 | 350 | 892 | 17-Jan-13 | XXXXXXXX |
| 24 | 300 | 925 | 17-Jan-13 | XXXXXXXX |
| 24 | 350 | 1180 | 21-Jan-13 | XXXXXXXX |
| 24 | 300 | 1147 | 21-Jan-13 | XXXXXXXX |
| 24 | 250 | 522 | 16-Jan-13 | XXXXXXXX |
| 24 | 500 | 945 | 17-Jan-13 | XXXXXXXX |
| 24 | 350 | 1090 | 21-Jan-13 | XXXXXXXX |
| 24 | 300 | 1160 | 21-Jan-13 | XXXXXXXX |
| 24 | 450 | 950 | 17-Jan-13 | XXXXXXXX |
| 24 | 300 | 443 | 14-Jan-13 | XXXXXXXX |
| 24 | 400 | 1256 | 22-Jan-13 | XXXXXXXX |
| 24 | 350 | 630 | 17-Jan-13 | XXXXXXXX |
| 24 | 350 | 597 | 16-Jan-13 | XXXXXXXX |
| 24 | 300 | 672 | 17-Jan-13 | XXXXXXXX |
| 24 | 350 | 580 | 17-Jan-13 | XXXXXXXX |
| 24 | 300 | 600 | 16-Jan-13 | XXXXXXXX |
| 24 | 450 | 774 | 22-Jan-13 | XXXXXXXX |
| 24 | 350 | 404 | 08-Jan-13 | XXXXXXXX |
| 24 | 450 | 425 | 08-Jan-13 | XXXXXXXX |
| 24 | 300 | 455 | 08-Jan-13 | XXXXXXXX |
| 24 | 250 | 698 | 21-Jan-13 | XXXXXXXX |
| 24 | 250 | 498 | 15-Jan-13 | XXXXXXXX |
| 24 | 250 | 1031 | 21-Jan-13 | XXXXXXXX |
| 24 | 500 | 1176 | 21-Jan-13 | XXXXXXXX |
| 24 | 350 | 1051 | 21-Jan-13 | XXXXXXXX |
| 24 | 300 | 476 | 10-Jan-13 | XXXXXXXX |
| 24 | 250 | 380 | 08-Jan-13 | XXXXXXXX |
| 24 | 350 | 695 | 21-Jan-13 | XXXXXXXX |
| 24 | 250 | 363 | 07-Jan-13 | XXXXXXXX |
| 24 | 800 | 617 | 15-Jan-13 | XXXXXXXX |
| 24 | 400 | 526 | 09-Jan-13 | XXXXXXXX |
| 24 | 300 | 500 | 09-Jan-13 | XXXXXXXX |
| 24 | 400 | 643 | 17-Jan-13 | XXXXXXXX |
| 24 | 500 | 645 | 17-Jan-13 | XXXXXXXX |
| 24 | 550 | 620 | 15-Jan-13 | XXXXXXXX |
| 24 | 300 | 570 | 14-Jan-13 | XXXXXXXX |
| 24 | 480 | 508 | 09-Jan-13 | XXXXXXXX |

| | | | | |
|---|---|---|---|---|
| 24 | 500 | 403 | 10-Jan-13 | XXXXXXXX |
| 24 | 400 | 823 | 17-Jan-13 | XXXXXXXX |
| 24 | 300 | 489 | 10-Jan-13 | XXXXXXXX |
| 24 | 250 | 370 | 08-Jan-13 | XXXXXXXX |
| 24 | 250 | 609 | 17-Jan-13 | XXXXXXXX |
| 24 | 500 | 462 | 09-Jan-13 | XXXXXXXX |
| 24 | 400 | 687 | 21-Jan-13 | XXXXXXXX |
| 24 | 250 | 732 | 21-Jan-13 | XXXXXXXX |
| 24 | 400 | 1226 | 21-Jan-13 | XXXXXXXX |
| 24 | 450 | 759 | 16-Jan-13 | XXXXXXXX |
| 24 | 550 | 652 | 15-Jan-13 | XXXXXXXX |
| 24 | 300 | 557 | 14-Jan-13 | XXXXXXXX |
| 24 | 300 | 424 | 14-Jan-13 | XXXXXXXX |
| 24 | 300 | 602 | 15-Jan-13 | XXXXXXXX |
| 24 | 300 | 702 | 21-Jan-13 | XXXXXXXX |
| 24 | 300 | 1060 | 21-Jan-13 | XXXXXXXX |
| 24 | 300 | 539 | 11-Jan-13 | XXXXXXXX |
| 24 | 200 | 569 | 16-Jan-13 | XXXXXXXX |
| 24 | 280 | 393 | 08-Jan-13 | XXXXXXXX |
| 18 | 450 | 488 | 15-Jan-13 | XXXXXXXX |
| 6 | 300 | 487 | 15-Jan-13 | XXXXXXXX |
| 12 | 350 | 560 | 16-Jan-13 | XXXXXXXX |
| 6 | 280 | 522 | 11-Jan-13 | XXXXXXXX |
| 6 | 350 | 459 | 09-Jan-13 | XXXXXXXX |
| 18 | 530 | 523 | 11-Jan-13 | XXXXXXXX |
| 24 | 650 | 524 | 11-Jan-13 | XXXXXXXX |
| 24 | 600 | 1006 | 17-Jan-13 | XXXXXXXX |
| 24 | 700 | 786 | 22-Jan-13 | XXXXXXXX |
| 24 | 700 | 592 | 15-Jan-13 | XXXXXXXX |
| 24 | 600 | 492 | 09-Jan-13 | XXXXXXXX |
| 24 | 600 | 638 | 18-Jan-13 | XXXXXXXX |
| 24 | 550 | 466 | 10-Jan-13 | XXXXXXXX |
| 24 | 600 | 550 | 11-Jan-13 | XXXXXXXX |
| 24 | 600 | 906 | 17-Jan-13 | XXXXXXXX |
| 24 | 650 | 401 | 09-Jan-13 | XXXXXXXX |
| 24 | 550 | 482 | 15-Jan-13 | XXXXXXXX |
| 24 | 650 | 860 | 17-Jan-13 | XXXXXXXX |
| 24 | 550 | 622 | 17-Jan-13 | XXXXXXXX |
| 24 | 550 | 690 | 21-Jan-13 | XXXXXXXX |
| 24 | 500 | 650 | 15-Jan-13 | XXXXXXXX |
| 24 | 600 | 506 | 15-Jan-13 | XXXXXXXX |
| 24 | 600 | 889 | 17-Jan-13 | XXXXXXXX |
| 24 | 400 | 687 | 21-Jan-13 | XXXXXXXX |
| 24 | 500 | 478 | 09-Jan-13 | XXXXXXXX |
| 12 | 650 | 537 | 16-Jan-13 | XXXXXXXX |
| 12 | 600 | 435 | 08-Jan-13 | XXXXXXXX |
| 12 | 650 | 1244 | 22-Jan-13 | XXXXXXXX |
| 12 | 650 | 338 | 07-Jan-13 | XXXXXXXX |
| 12 | 450 | 805 | 16-Jan-13 | XXXXXXXX |
| 12 | 500 | 633 | 17-Jan-13 | XXXXXXXX |

| | | | | |
|---|---|---|---|---|
| 12 | 550 | 1010 | 17-Jan-13 | XXXXXXXX |
| 12 | 700 | 790 | 22-Jan-13 | XXXXXXXX |
| 12 | 550 | 1042 | 21-Jan-13 | XXXXXXXX |
| 12 | 550 | 468 | 10-Jan-13 | XXXXXXXX |
| 12 | 500 | 898 | 17-Jan-13 | XXXXXXXX |
| 12 | 650 | 1245 | 22-Jan-13 | XXXXXXXX |
| 12 | 650 | 607 | 15-Jan-13 | XXXXXXXX |
| 12 | 600 | 405 | 08-Jan-13 | XXXXXXXX |
| 12 | 650 | 421 | 08-Jan-13 | XXXXXXXX |
| 12 | 600 | 823 | 22-Jan-13 | XXXXXXXX |
| 12 | 700 | 589 | 16-Jan-13 | XXXXXXXX |
| 12 | 700 | 881 | 17-Jan-13 | XXXXXXXX |
| 12 | 700 | 922 | 17-Jan-13 | XXXXXXXX |
| 12 | 500 | 845 | 17-Jan-13 | XXXXXXXX |
| 12 | 600 | 1309 | 22-Jan-13 | XXXXXXXX |
| 12 | 500 | 688 | 16-Jan-13 | XXXXXXXX |
| 12 | 380 | 764 | 16-Jan-13 | XXXXXXXX |
| 12 | 450 | 447 | 14-Jan-13 | XXXXXXXX |
| 12 | 550 | 652 | 18-Jan-13 | XXXXXXXX |
| 12 | 550 | 462 | 10-Jan-13 | XXXXXXXX |
| 12 | 400 | 451 | 14-Jan-13 | XXXXXXXX |
| 12 | 470 | 514 | 11-Jan-13 | XXXXXXXX |
| 12 | 650 | 628 | 17-Jan-13 | XXXXXXXX |
| 12 | 300 | 610 | 17-Jan-13 | XXXXXXXX |
| 12 | 600 | 585 | 15-Jan-13 | XXXXXXXX |
| 12 | 700 | 587 | 15-Jan-13 | XXXXXXXX |
| 12 | 650 | 458 | 14-Jan-13 | XXXXXXXX |
| 6 | 600 | 335 | 07-Jan-13 | XXXXXXXX |
| 6 | 600 | 381 | 07-Jan-13 | XXXXXXXX |
| 6 | 650 | 419 | 14-Jan-13 | XXXXXXXX |
| 6 | 600 | 984 | 17-Jan-13 | XXXXXXXX |
| 6 | 700 | 459 | 14-Jan-13 | XXXXXXXX |
| 6 | 650 | 606 | 15-Jan-13 | XXXXXXXX |
| 6 | 540 | 959 | 17-Jan-13 | XXXXXXXX |
| 6 | 700 | 667 | 17-Jan-13 | XXXXXXXX |
| 6 | | 450 | 08-Jan-13 | XXXXXXXX |
| 6 | 550 | 403 | 08-Jan-13 | XXXXXXXX |
| 6 | 750 | 824 | 22-Jan-13 | XXXXXXXX |
| 6 | 600 | 809 | 16-Jan-13 | XXXXXXXX |
| 6 | 750 | 619 | 15-Jan-13 | XXXXXXXX |
| 6 | 600 | 564 | 14-Jan-13 | XXXXXXXX |
| 6 | 700 | 622 | 17-Jan-13 | XXXXXXXX |
| 6 | 600 | 1099 | 21-Jan-13 | XXXXXXXX |
| 6 | 600 | 1155 | 21-Jan-13 | XXXXXXXX |
| 6 | 600 | 560 | 14-Jan-13 | XXXXXXXX |
| 6 | 650 | 696 | 16-Jan-13 | XXXXXXXX |
| 6 | 550 | 574 | 16-Jan-13 | XXXXXXXX |
| 6 | 500 | 664 | 21-Jan-13 | XXXXXXXX |
| 6 | 500 | 537 | 11-Jan-13 | XXXXXXXX |
| 6 | 550 | 364 | 07-Jan-13 | XXXXXXXX |

| | | | | |
|---|---|---|---|---|
| 6 | 600 | 645 | 18-Jan-13 | XXXXXXXX |
| 6 | 550 | 376 | 07-Jan-13 | XXXXXXXX |
| 6 | 600 | 481 | 09-Jan-13 | XXXXXXXX |
| 6 | 600 | 988 | 17-Jan-13 | XXXXXXXX |
| 6 | 600 | 442 | 14-Jan-13 | XXXXXXXX |
| 6 | 540 | 960 | 17-Jan-13 | XXXXXXXX |
| 6 | 600 | 634 | 17-Jan-13 | XXXXXXXX |
| 6 | 700 | 423 | 08-Jan-13 | XXXXXXXX |
| 6 | 650 | 1048 | 21-Jan-13 | XXXXXXXX |
| 6 | 550 | 1280 | 22-Jan-13 | XXXXXXXX |
| 6 | 600 | 518 | 11-Jan-13 | XXXXXXXX |
| 6 | 650 | 621 | 15-Jan-13 | XXXXXXXX |
| 6 | 600 | 587 | 16-Jan-13 | XXXXXXXX |
| 6 | 600 | 569 | 14-Jan-13 | XXXXXXXX |
| 6 | 650 | 620 | 17-Jan-13 | XXXXXXXX |
| 6 | 650 | 506 | 09-Jan-13 | XXXXXXXX |
| 6 | 600 | 1103 | 21-Jan-13 | XXXXXXXX |
| 6 | 650 | 620 | 17-Jan-13 | XXXXXXXX |
| 6 | 700 | 1303 | 22-Jan-13 | XXXXXXXX |
| 6 | 650 | 985 | 17-Jan-13 | XXXXXXXX |
| 6 | 650 | 1246 | 22-Jan-13 | XXXXXXXX |
| 6 | 650 | 613 | 15-Jan-13 | XXXXXXXX |
| 6 | 800 | 443 | 08-Jan-13 | XXXXXXXX |
| 6 | 700 | 402 | 08-Jan-13 | XXXXXXXX |
| 6 | 630 | 531 | 11-Jan-13 | XXXXXXXX |
| 6 | 700 | 513 | 15-Jan-13 | XXXXXXXX |
| 6 | 480 | 967 | 17-Jan-13 | XXXXXXXX |
| 6 | 570 | 533 | 11-Jan-13 | XXXXXXXX |
| 6 | 600 | 590 | 16-Jan-13 | XXXXXXXX |
| 6 | 600 | 1329 | 22-Jan-13 | XXXXXXXX |
| 6 | 700 | 551 | 14-Jan-13 | XXXXXXXX |
| 6 | 650 | 437 | 08-Jan-13 | XXXXXXXX |
| 6 | 750 | 479 | 15-Jan-13 | XXXXXXXX |
| 6 | 750 | 777 | 22-Jan-13 | XXXXXXXX |
| 6 | 750 | 760 | 16-Jan-13 | XXXXXXXX |
| 12 | 650 | 1084 | 21-Jan-13 | XXXXXXXX |
| 6 | 800 | 1120 | 21-Jan-13 | XXXXXXXX |
| 6 | 500 | 344 | 07-Jan-13 | XXXXXXXX |
| 6 | 600 | 582 | 15-Jan-13 | XXXXXXXX |
| 6 | 500 | 1057 | 21-Jan-13 | XXXXXXXX |
| 6 | 700 | 584 | 15-Jan-13 | XXXXXXXX |
| 6 | 550 | 469 | 10-Jan-13 | XXXXXXXX |
| 6 | 550 | 483 | 09-Jan-13 | XXXXXXXX |
| 6 | 550 | 399 | 08-Jan-13 | XXXXXXXX |
| 6 | 700 | 629 | 17-Jan-13 | XXXXXXXX |
| 6 | 550 | 570 | 14-Jan-13 | XXXXXXXX |
| 6 | 600 | 691 | 21-Jan-13 | XXXXXXXX |
| 6 | 600 | 1087 | 21-Jan-13 | XXXXXXXX |
| 6 | 600 | 382 | 07-Jan-13 | XXXXXXXX |
| 6 | | | | XXXXXXXX |

| | | | | |
|---|---|---|---|---|
| 6 | | 722 | 16-Jan-13 | XXXXXXXX |
| 6 | 550 | 595 | 16-Jan-13 | XXXXXXXX |
| 6 | 650 | 940 | 17-Jan-13 | XXXXXXXX |
| 6 | 750 | 827 | 22-Jan-13 | XXXXXXXX |
| 6 | 550 | 1094 | 21-Jan-13 | XXXXXXXX |
| 6 | 300 | 715 | 21-Jan-13 | XXXXXXXX |
| 6 | 500 | 617 | 16-Jan-13 | XXXXXXXX |
| 6 | 550 | 679 | 21-Jan-13 | XXXXXXXX |
| 6 | 600 | 1219 | 21-Jan-13 | XXXXXXXX |
| 6 | 500 | 644 | 15-Jan-13 | XXXXXXXX |
| 6 | 900 | 370 | 07-Jan-13 | XXXXXXXX |
| 6 | 500 | 595 | 15-Jan-13 | XXXXXXXX |
| 6 | 600 | 586 | 15-Jan-13 | XXXXXXXX |
| 6 | 550 | 549 | 11-Jan-13 | XXXXXXXX |
| 6 | 700 | 457 | 14-Jan-13 | XXXXXXXX |
| 6 | 750 | 972 | 17-Jan-13 | XXXXXXXX |
| 6 | 540 | 965 | 17-Jan-13 | XXXXXXXX |
| 6 | 600 | 628 | 17-Jan-13 | XXXXXXXX |
| 6 | 600 | 599 | 16-Jan-13 | XXXXXXXX |
| 6 | 800 | 451 | 08-Jan-13 | XXXXXXXX |
| 6 | 550 | 395 | 08-Jan-13 | XXXXXXXX |
| 6 | 650 | 812 | 16-Jan-13 | XXXXXXXX |
| 6 | 570 | 520 | 11-Jan-13 | XXXXXXXX |
| 6 | 650 | 625 | 15-Jan-13 | XXXXXXXX |
| 6 | 600 | 623 | 17-Jan-13 | XXXXXXXX |
| 6 | 600 | 371 | 08-Jan-13 | XXXXXXXX |
| 6 | 530 | 663 | 21-Jan-13 | XXXXXXXX |
| 6 | 650 | 832 | 17-Jan-13 | XXXXXXXX |
| 6 | 600 | 1201 | 21-Jan-13 | XXXXXXXX |
| 6 | 750 | 1112 | 21-Jan-13 | XXXXXXXX |
| 6 | 800 | 624 | 17-Jan-13 | XXXXXXXX |
| 6 | 550 | 402 | 08-Jan-13 | XXXXXXXX |
| 6 | 750 | 826 | 22-Jan-13 | XXXXXXXX |
| 6 | 500 | 1018 | 17-Jan-13 | XXXXXXXX |
| 6 | 600 | 337 | 07-Jan-13 | XXXXXXXX |
| 6 | 600 | 662 | 17-Jan-13 | XXXXXXXX |
| 6 | | | | XXXXXXXX |
| 6 | 550 | 957 | 17-Jan-13 | XXXXXXXX |
| 6 | 650 | 542 | 16-Jan-13 | XXXXXXXX |
| 6 | 650 | 373 | 07-Jan-13 | XXXXXXXX |
| 6 | 800 | 1324 | 22-Jan-13 | XXXXXXXX |
| 6 | 650 | 339 | 07-Jan-13 | XXXXXXXX |
| 6 | 500 | 347 | 07-Jan-13 | XXXXXXXX |
| 6 | 650 | 1314 | 22-Jan-13 | XXXXXXXX |
| 6 | 650 | 1200 | 21-Jan-13 | XXXXXXXX |
| 6 | 750 | 473 | 15-Jan-13 | XXXXXXXX |
| 6 | 700 | 485 | 09-Jan-13 | XXXXXXXX |
| 6 | 750 | 813 | 22-Jan-13 | XXXXXXXX |
| 6 | 600 | 604 | 15-Jan-13 | XXXXXXXX |
| 6 | 650 | 942 | 17-Jan-13 | XXXXXXXX |

| | | | | |
|---|---|---|---|---|
| 6 | 800 | 452 | 08-Jan-13 | XXXXXXXX |
| 6 | 570 | 528 | 11-Jan-13 | XXXXXXXX |
| 6 | 650 | 627 | 15-Jan-13 | XXXXXXXX |
| 6 | 750 | 662 | 15-Jan-13 | XXXXXXXX |
| 6 | 540 | 737 | 21-Jan-13 | XXXXXXXX |
| 6 | 600 | 489 | 09-Jan-13 | XXXXXXXX |
| 6 | 750 | 415 | 10-Jan-13 | XXXXXXXX |
| 6 | 600 | 551 | 11-Jan-13 | XXXXXXXX |
| 6 | 650 | 397 | 09-Jan-13 | XXXXXXXX |
| 6 | 650 | 581 | 15-Jan-13 | XXXXXXXX |
| 6 | 600 | 633 | 17-Jan-13 | XXXXXXXX |
| 6 | 600 | 534 | 11-Jan-13 | XXXXXXXX |
| 6 | 650 | 629 | 15-Jan-13 | XXXXXXXX |
| 6 | 600 | 582 | 16-Jan-13 | XXXXXXXX |
| 6 | 750 | 435 | 14-Jan-13 | XXXXXXXX |
| 6 | 700 | 681 | 21-Jan-13 | XXXXXXXX |
| 6 | 600 | 463 | 14-Jan-13 | XXXXXXXX |
| 6 | 650 | 797 | 16-Jan-13 | XXXXXXXX |
| 6 | 700 | 896 | 17-Jan-13 | XXXXXXXX |
| 6 | 850 | 765 | 16-Jan-13 | XXXXXXXX |
| 6 | 750 | 1068 | 21-Jan-13 | XXXXXXXX |
| 6 | 700 | 515 | 09-Jan-13 | XXXXXXXX |
| 6 | 650 | 1047 | 21-Jan-13 | XXXXXXXX |
| 6 | 750 | 527 | 09-Jan-13 | XXXXXXXX |
| 6 | 700 | 879 | 17-Jan-13 | XXXXXXXX |
| 6 | 600 | 817 | 17-Jan-13 | XXXXXXXX |
| 6 | 750 | 761 | 16-Jan-13 | XXXXXXXX |
| 6 | 600 | 561 | 14-Jan-13 | XXXXXXXX |
| 6 | 850 | 758 | 16-Jan-13 | XXXXXXXX |
| 6 | 600 | 799 | 16-Jan-13 | XXXXXXXX |
| 6 | 750 | 790 | 16-Jan-13 | XXXXXXXX |
| 6 | 600 | 535 | 16-Jan-13 | XXXXXXXX |
| 6 | 650 | 340 | 07-Jan-13 | XXXXXXXX |
| 6 | 550 | 682 | 21-Jan-13 | XXXXXXXX |
| 6 | 550 | 446 | 14-Jan-13 | XXXXXXXX |
| 6 | 500 | 371 | 07-Jan-13 | XXXXXXXX |
| 6 | 620 | 1208 | 21-Jan-13 | XXXXXXXX |
| 6 | 600 | 371 | 07-Jan-13 | XXXXXXXX |
| 6 | 600 | 1083 | 21-Jan-13 | XXXXXXXX |
| 6 | 570 | 990 | 17-Jan-13 | XXXXXXXX |
| 6 | 650 | 474 | 15-Jan-13 | XXXXXXXX |
| 6 | 600 | 1270 | 22-Jan-13 | XXXXXXXX |
| 6 | 500 | 776 | 16-Jan-13 | XXXXXXXX |
| 6 | 600 | 528 | 16-Jan-13 | XXXXXXXX |
| 6 | 600 | 629 | 17-Jan-13 | XXXXXXXX |
| 6 | 800 | 447 | 08-Jan-13 | XXXXXXXX |
| 6 | 570 | 515 | 11-Jan-13 | XXXXXXXX |
| 6 | 650 | 618 | 15-Jan-13 | XXXXXXXX |
| 6 | 600 | 473 | 09-Jan-13 | XXXXXXXX |
| 6 | 700 | 877 | 17-Jan-13 | XXXXXXXX |

| | | | | |
|---|---|---|---|---|
| 6 | 750 | 1221 | 21-Jan-13 | XXXXXXXX |
| 6 | 600 | 438 | 08-Jan-13 | XXXXXXXX |
| 6 | 750 | 836 | 17-Jan-13 | XXXXXXXX |
| 6 | 750 | 684 | 21-Jan-13 | XXXXXXXX |
| 6 | 800 | 724 | 21-Jan-13 | XXXXXXXX |
| 6 | 700 | 397 | 08-Jan-13 | XXXXXXXX |
| 6 | 700 | 1276 | 22-Jan-13 | XXXXXXXX |
| 6 | 750 | 418 | 08-Jan-13 | XXXXXXXX |
| 6 | 850 | 787 | 22-Jan-13 | XXXXXXXX |
| 6 | 750 | 340 | 07-Jan-13 | XXXXXXXX |
| 6 | 750 | 1252 | 22-Jan-13 | XXXXXXXX |
| 6 | 680 | 373 | 07-Jan-13 | XXXXXXXX |
| 6 | 750 | 359 | 07-Jan-13 | XXXXXXXX |
| 6 | 800 | 1111 | 21-Jan-13 | XXXXXXXX |
| 6 | 680 | 1272 | 22-Jan-13 | XXXXXXXX |
| 6 | 780 | 736 | 16-Jan-13 | XXXXXXXX |
| 6 | 850 | | | XXXXXXXX |
| 6 | 750 | 418 | 14-Jan-13 | XXXXXXXX |
| 6 | 750 | 979 | 17-Jan-13 | XXXXXXXX |
| 6 | 800 | 934 | 17-Jan-13 | XXXXXXXX |
| 6 | 500 | 916 | 17-Jan-13 | XXXXXXXX |
| 6 | 750 | 472 | 15-Jan-13 | XXXXXXXX |
| 6 | 950 | 613 | 15-Jan-13 | XXXXXXXX |
| 6 | 600 | 519 | 16-Jan-13 | XXXXXXXX |
| 6 | 750 | 420 | 08-Jan-13 | XXXXXXXX |
| 6 | 800 | 536 | 09-Jan-13 | XXXXXXXX |
| 6 | 750 | 890 | 17-Jan-13 | XXXXXXXX |
| 6 | 700 | 366 | 07-Jan-13 | XXXXXXXX |
| 6 | 750 | 411 | 10-Jan-13 | XXXXXXXX |
| 6 | 650 | 902 | 17-Jan-13 | XXXXXXXX |
| 6 | 850 | 716 | 16-Jan-13 | XXXXXXXX |
| 6 | 800 | 625 | 17-Jan-13 | XXXXXXXX |
| 6 | 750 | 1091 | 21-Jan-13 | XXXXXXXX |
| 6 | 750 | 986 | 17-Jan-13 | XXXXXXXX |
| 6 | | 681 | 16-Jan-13 | XXXXXXXX |
| 6 | 850 | 484 | 15-Jan-13 | XXXXXXXX |
| 6 | 800 | 740 | 21-Jan-13 | XXXXXXXX |
| 6 | 750 | 1264 | 22-Jan-13 | XXXXXXXX |
| 6 | 800 | 580 | 15-Jan-13 | XXXXXXXX |
| 6 | 800 | 550 | 14-Jan-13 | XXXXXXXX |
| 6 | 750 | 579 | 14-Jan-13 | XXXXXXXX |
| 6 | 200 | 467 | 14-Jan-13 | XXXXXXXX |
| 6 | 800 | 969 | 17-Jan-13 | XXXXXXXX |
| 6 | 750 | 786 | 16-Jan-13 | XXXXXXXX |
| 6 | 780 | 964 | 17-Jan-13 | XXXXXXXX |
| 6 | 250 | 604 | 17-Jan-13 | XXXXXXXX |
| 6 | 850 | 593 | 16-Jan-13 | XXXXXXXX |
| 6 | 850 | 673 | 17-Jan-13 | XXXXXXXX |
| 6 | 800 | 939 | 17-Jan-13 | XXXXXXXX |
| 6 | 800 | 579 | 17-Jan-13 | XXXXXXXX |

| | | | | |
|---|---|---|---|---|
| 6 | 700 | 593 | 17-Jan-13 | XXXXXXXX |
| 6 | 800 | 352 | 07-Jan-13 | XXXXXXXX |
| 6 | 950 | 782 | 22-Jan-13 | XXXXXXXX |
| 6 | 800 | 406 | 08-Jan-13 | XXXXXXXX |
| 6 | 600 | 694 | 21-Jan-13 | XXXXXXXX |
| 6 | 700 | 397 | 08-Jan-13 | XXXXXXXX |
| 6 | 700 | 1116 | 21-Jan-13 | XXXXXXXX |
| 6 | 750 | 821 | 22-Jan-13 | XXXXXXXX |
| 6 | 850 | 500 | 15-Jan-13 | XXXXXXXX |
| 6 | 750 | 332 | 07-Jan-13 | XXXXXXXX |
| 6 | 700 | 1028 | 21-Jan-13 | XXXXXXXX |
| 6 | 750 | 1000 | 17-Jan-13 | XXXXXXXX |
| 6 | 750 | 338 | 07-Jan-13 | XXXXXXXX |
| 6 | 700 | 1053 | 21-Jan-13 | XXXXXXXX |
| 6 | 700 | 477 | 10-Jan-13 | XXXXXXXX |
| 6 | 750 | 354 | 07-Jan-13 | XXXXXXXX |
| 6 | 570 | 381 | 08-Jan-13 | XXXXXXXX |
| 6 | 750 | 1278 | 22-Jan-13 | XXXXXXXX |
| 6 | 750 | 362 | 07-Jan-13 | XXXXXXXX |
| 6 | 800 | 770 | 21-Jan-13 | XXXXXXXX |
| 6 | 750 | 527 | 11-Jan-13 | XXXXXXXX |
| 6 | 800 | 568 | 14-Jan-13 | XXXXXXXX |
| 6 | 850 | 624 | 15-Jan-13 | XXXXXXXX |
| 6 | 800 | 580 | 16-Jan-13 | XXXXXXXX |
| 6 | 800 | 529 | 09-Jan-13 | XXXXXXXX |
| 6 | 750 | 565 | 14-Jan-13 | XXXXXXXX |
| 6 | 800 | 869 | 17-Jan-13 | XXXXXXXX |
| 6 | 650 | 642 | 17-Jan-13 | XXXXXXXX |
| 6 | 750 | 619 | 15-Jan-13 | XXXXXXXX |
| 6 | 750 | 477 | 09-Jan-13 | XXXXXXXX |
| 6 | 750 | 624 | 17-Jan-13 | XXXXXXXX |
| 6 | 750 | 606 | 15-Jan-13 | XXXXXXXX |
| 6 | 700 | 502 | 09-Jan-13 | XXXXXXXX |
| 6 | 850 | 438 | 14-Jan-13 | XXXXXXXX |
| 6 | 750 | 631 | 17-Jan-13 | XXXXXXXX |
| 6 | 800 | 883 | 17-Jan-13 | XXXXXXXX |
| 6 | 700 | 471 | 09-Jan-13 | XXXXXXXX |
| 6 | 700 | 641 | 15-Jan-13 | XXXXXXXX |
| 6 | 700 | 491 | 10-Jan-13 | XXXXXXXX |
| 6 | 700 | 768 | 21-Jan-13 | XXXXXXXX |
| 6 | 700 | 1128 | 21-Jan-13 | XXXXXXXX |
| 6 | 800 | 1098 | 21-Jan-13 | XXXXXXXX |
| 6 | 700 | 739 | 16-Jan-13 | XXXXXXXX |
| 6 | 750 | 349 | 08-Jan-13 | XXXXXXXX |
| 6 | 750 | 615 | 17-Jan-13 | XXXXXXXX |
| 6 | 800 | 453 | 09-Jan-13 | XXXXXXXX |
| 6 | 750 | 568 | 14-Jan-13 | XXXXXXXX |
| 6 | 800 | 587 | 17-Jan-13 | XXXXXXXX |
| 6 | 750 | 512 | 10-Jan-13 | XXXXXXXX |
| 6 | 820 | 801 | 22-Jan-13 | XXXXXXXX |

| | | | | |
|---|---|---|---|---|
| 6 | 800 | 1186 | 21-Jan-13 | XXXXXXXX |
| 6 | 800 | 688 | 21-Jan-13 | XXXXXXXX |
| 6 | 810 | 733 | 21-Jan-13 | XXXXXXXX |
| 6 | 600 | 1225 | 21-Jan-13 | XXXXXXXX |
| 6 | 800 | 1026 | 21-Jan-13 | XXXXXXXX |
| 6 | 800 | 1311 | 22-Jan-13 | XXXXXXXX |
| 6 | 800 | 659 | 15-Jan-13 | XXXXXXXX |
| 6 | 890 | 610 | 15-Jan-13 | XXXXXXXX |
| 6 | 750 | 562 | 14-Jan-13 | XXXXXXXX |
| 6 | 750 | 594 | 15-Jan-13 | XXXXXXXX |
| 6 | 750 | 433 | 08-Jan-13 | XXXXXXXX |
| 6 | 800 | 701 | 21-Jan-13 | XXXXXXXX |
| 6 | 750 | 479 | 10-Jan-13 | XXXXXXXX |
| 6 | 700 | 577 | 16-Jan-13 | XXXXXXXX |
| 6 | 600 | 674 | 21-Jan-13 | XXXXXXXX |
| 6 | 750 | 360 | 07-Jan-13 | XXXXXXXX |
| 6 | 800 | 1298 | 22-Jan-13 | XXXXXXXX |
| 6 | | | | XXXXXXXX |
| 6 | 600 | 370 | 07-Jan-13 | XXXXXXXX |
| 6 | 630 | 545 | 11-Jan-13 | XXXXXXXX |
| 6 | 700 | 885 | 17-Jan-13 | XXXXXXXX |
| 6 | 780 | 617 | 17-Jan-13 | XXXXXXXX |
| 6 | 550 | 1154 | 21-Jan-13 | XXXXXXXX |
| 6 | 650 | 691 | 16-Jan-13 | XXXXXXXX |
| 6 | 700 | 399 | 08-Jan-13 | XXXXXXXX |
| 6 | 600 | 491 | 15-Jan-13 | XXXXXXXX |
| 6 | 650 | 463 | 10-Jan-13 | XXXXXXXX |
| 6 | 650 | 436 | 08-Jan-13 | XXXXXXXX |
| 6 | 650 | 1093 | 21-Jan-13 | XXXXXXXX |
| 6 | 700 | 812 | 22-Jan-13 | XXXXXXXX |
| 6 | 650 | 783 | 16-Jan-13 | XXXXXXXX |
| 6 | 700 | 853 | 17-Jan-13 | XXXXXXXX |
| 6 | 650 | 341 | 07-Jan-13 | XXXXXXXX |
| 6 | 750 | 1011 | 17-Jan-13 | XXXXXXXX |
| 6 | 650 | 1056 | 21-Jan-13 | XXXXXXXX |
| 6 | 700 | 585 | 16-Jan-13 | XXXXXXXX |
| 6 | 750 | 528 | 09-Jan-13 | XXXXXXXX |
| 6 | 700 | 503 | 09-Jan-13 | XXXXXXXX |
| 6 | 650 | 1127 | 21-Jan-13 | XXXXXXXX |
| 6 | 750 | 576 | 14-Jan-13 | XXXXXXXX |
| 6 | 750 | 692 | 16-Jan-13 | XXXXXXXX |
| 6 | 650 | 536 | 11-Jan-13 | XXXXXXXX |
| 6 | 690 | 662 | 21-Jan-13 | XXXXXXXX |
| 6 | 650 | 392 | 07-Jan-13 | XXXXXXXX |
| 6 | 550 | 438 | 08-Jan-13 | XXXXXXXX |
| 6 | 700 | 487 | 09-Jan-13 | XXXXXXXX |
| 6 | 450 | 530 | 11-Jan-13 | XXXXXXXX |
| 6 | 650 | 621 | 17-Jan-13 | XXXXXXXX |
| 6 | 650 | 389 | 07-Jan-13 | XXXXXXXX |
| 6 | 650 | 605 | 15-Jan-13 | XXXXXXXX |

| | | | | |
|---|---|---|---|---|
| 6 | 650 | 811 | 16-Jan-13 | XXXXXXXX |
| 6 | 500 | 514 | 15-Jan-13 | XXXXXXXX |
| 6 | 600 | 331 | 07-Jan-13 | XXXXXXXX |
| 6 | 650 | 796 | 16-Jan-13 | XXXXXXXX |
| 6 | 650 | 518 | 16-Jan-13 | XXXXXXXX |
| 6 | 600 | 591 | 15-Jan-13 | XXXXXXXX |
| 6 | 600 | 555 | 16-Jan-13 | XXXXXXXX |
| 6 | 600 | 433 | 08-Jan-13 | XXXXXXXX |
| 6 | 600 | 1243 | 22-Jan-13 | XXXXXXXX |
| 6 | 600 | 855 | 17-Jan-13 | XXXXXXXX |
| 6 | 750 | 825 | 22-Jan-13 | XXXXXXXX |
| 6 | 700 | 333 | 07-Jan-13 | XXXXXXXX |
| 6 | 600 | 810 | 16-Jan-13 | XXXXXXXX |
| 6 | 600 | 1049 | 21-Jan-13 | XXXXXXXX |
| 6 | 650 | 474 | 10-Jan-13 | XXXXXXXX |
| 6 | 600 | 1281 | 22-Jan-13 | XXXXXXXX |
| 6 | 600 | 584 | 16-Jan-13 | XXXXXXXX |
| 6 | 600 | 560 | 14-Jan-13 | XXXXXXXX |
| 6 | 600 | 639 | 17-Jan-13 | XXXXXXXX |
| 6 | 750 | 625 | 17-Jan-13 | XXXXXXXX |
| 6 | 550 | 1132 | 21-Jan-13 | XXXXXXXX |
| 6 | 650 | 436 | 08-Jan-13 | XXXXXXXX |
| 6 | 550 | 512 | 15-Jan-13 | XXXXXXXX |
| 6 | 600 | 918 | 17-Jan-13 | XXXXXXXX |
| 6 | 500 | 566 | 16-Jan-13 | XXXXXXXX |
| 6 | 600 | 910 | 17-Jan-13 | XXXXXXXX |
| 6 | 600 | 1290 | 22-Jan-13 | XXXXXXXX |
| 6 | 600 | 576 | 14-Jan-13 | XXXXXXXX |
| 6 | 800 | 974 | 17-Jan-13 | XXXXXXXX |
| 6 | 550 | 856 | 17-Jan-13 | XXXXXXXX |
| 6 | 750 | 669 | 17-Jan-13 | XXXXXXXX |
| 6 | 700 | 1001 | 17-Jan-13 | XXXXXXXX |
| 6 | 700 | 626 | 15-Jan-13 | XXXXXXXX |
| 6 | 500 | 601 | 15-Jan-13 | XXXXXXXX |
| 6 | 650 | 517 | 09-Jan-13 | XXXXXXXX |
| 6 | 600 | 978 | 17-Jan-13 | XXXXXXXX |
| 6 | 450 | 471 | 15-Jan-13 | XXXXXXXX |
| 6 | 700 | 415 | 08-Jan-13 | XXXXXXXX |
| 6 | 650 | 540 | 16-Jan-13 | XXXXXXXX |
| 6 | 550 | 900 | 17-Jan-13 | XXXXXXXX |
| 6 | 650 | 1079 | 21-Jan-13 | XXXXXXXX |
| 6 | 550 | 611 | 15-Jan-13 | XXXXXXXX |
| 6 | 450 | 594 | 17-Jan-13 | XXXXXXXX |
| 6 | 850 | 406 | 08-Jan-13 | XXXXXXXX |
| 6 | 600 | 521 | 11-Jan-13 | XXXXXXXX |
| 6 | 500 | 588 | 16-Jan-13 | XXXXXXXX |
| 6 | 1000 | 353 | 08-Jan-13 | XXXXXXXX |
| 6 | 500 | 1027 | 21-Jan-13 | XXXXXXXX |
| 6 | 550 | 466 | 14-Jan-13 | XXXXXXXX |
| 6 | 800 | 1108 | 21-Jan-13 | XXXXXXXX |

| | | | | |
|---|---|---|---|---|
| 6 | 600 | 929 | 17-Jan-13 | XXXXXXXX |
| 6 | 750 | 422 | 08-Jan-13 | XXXXXXXX |
| 6 | 600 | 590 | 15-Jan-13 | XXXXXXXX |
| 6 | 700 | 486 | 09-Jan-13 | XXXXXXXX |
| 6 | 650 | 483 | 15-Jan-13 | XXXXXXXX |
| 6 | 750 | 586 | 15-Jan-13 | XXXXXXXX |
| 6 | 750 | 670 | 17-Jan-13 | XXXXXXXX |
| 6 | 1000 | 822 | 22-Jan-13 | XXXXXXXX |
| 6 | 600 | 339 | 07-Jan-13 | XXXXXXXX |
| 6 | 600 | 1282 | 22-Jan-13 | XXXXXXXX |
| 6 | 700 | 627 | 17-Jan-13 | XXXXXXXX |
| 6 | 1000 | 355 | 08-Jan-13 | XXXXXXXX |
| 6 | 500 | 731 | 21-Jan-13 | XXXXXXXX |
| 12 | 350 | 772 | 21-Jan-13 | XXXXXXXX |
| 12 | 250 | 774 | 16-Jan-13 | XXXXXXXX |
| 12 | 350 | 830 | 17-Jan-13 | XXXXXXXX |
| 12 | 600 | 336 | 07-Jan-13 | XXXXXXXX |
| 12 | 300 | 679 | 21-Jan-13 | XXXXXXXX |
| 12 | 350 | 723 | 21-Jan-13 | XXXXXXXX |
| 12 | 350 | 417 | 08-Jan-13 | XXXXXXXX |
| 12 | 380 | 1021 | 21-Jan-13 | XXXXXXXX |
| 12 | 300 | 1199 | 21-Jan-13 | XXXXXXXX |
| 12 | 450 | 677 | 16-Jan-13 | XXXXXXXX |
| 12 | 300 | 1253 | 22-Jan-13 | XXXXXXXX |
| 12 | 400 | 1259 | 22-Jan-13 | XXXXXXXX |
| 12 | 300 | 711 | 21-Jan-13 | XXXXXXXX |
| 12 | 300 | 379 | 07-Jan-13 | XXXXXXXX |
| 12 | 350 | 1109 | 21-Jan-13 | XXXXXXXX |
| 12 | 350 | 751 | 21-Jan-13 | XXXXXXXX |
| 12 | 350 | 408 | 08-Jan-13 | XXXXXXXX |
| 12 | 350 | 338 | 07-Jan-13 | XXXXXXXX |
| 12 | 350 | 390 | 08-Jan-13 | XXXXXXXX |
| 12 | 300 | 493 | 15-Jan-13 | XXXXXXXX |
| 12 | 300 | 420 | 14-Jan-13 | XXXXXXXX |
| 12 | | 685 | 21-Jan-13 | XXXXXXXX |
| 12 | 280 | 641 | 18-Jan-13 | XXXXXXXX |
| 12 | 350 | 534 | 09-Jan-13 | XXXXXXXX |
| 12 | 300 | 1328 | 22-Jan-13 | XXXXXXXX |
| 12 | 780 | 369 | 07-Jan-13 | XXXXXXXX |
| 12 | 300 | 1288 | 22-Jan-13 | XXXXXXXX |
| 12 | 350 | 1174 | 21-Jan-13 | XXXXXXXX |
| 12 | 300 | 1265 | 22-Jan-13 | XXXXXXXX |
| 12 | 270 | 504 | 10-Jan-13 | XXXXXXXX |
| 12 | 350 | 1162 | 21-Jan-13 | XXXXXXXX |
| 12 | 350 | 948 | 17-Jan-13 | XXXXXXXX |
| 12 | 300 | 1257 | 22-Jan-13 | XXXXXXXX |
| 12 | 350 | 1302 | 22-Jan-13 | XXXXXXXX |
| 12 | 350 | 354 | 07-Jan-13 | XXXXXXXX |
| 12 | 400 | 385 | 08-Jan-13 | XXXXXXXX |
| 12 | 350 | 779 | 22-Jan-13 | XXXXXXXX |

| | | | | |
|---|---|---|---|---|
| 12 | 350 | 620 | 15-Jan-13 | XXXXXXXX |
| 12 | 300 | 693 | 21-Jan-13 | XXXXXXXX |
| 12 | 350 | 422 | 08-Jan-13 | XXXXXXXX |
| 12 | 300 | 342 | 07-Jan-13 | XXXXXXXX |
| 12 | 300 | 340 | 07-Jan-13 | XXXXXXXX |
| 12 | 300 | 401 | 08-Jan-13 | XXXXXXXX |
| 12 | 300 | 606 | 16-Jan-13 | XXXXXXXX |
| 12 | 300 | 1170 | 21-Jan-13 | XXXXXXXX |
| 12 | 300 | 1055 | 21-Jan-13 | XXXXXXXX |
| 12 | 350 | 378 | 08-Jan-13 | XXXXXXXX |
| 12 | 300 | 794 | 22-Jan-13 | XXXXXXXX |
| 12 | 300 | 525 | 09-Jan-13 | XXXXXXXX |
| 12 | 350 | 445 | 09-Jan-13 | XXXXXXXX |
| 12 | 330 | 464 | 09-Jan-13 | XXXXXXXX |
| 12 | 300 | 867 | 17-Jan-13 | XXXXXXXX |
| 12 | 300 | 509 | 09-Jan-13 | XXXXXXXX |
| 12 | 350 | 434 | 14-Jan-13 | XXXXXXXX |
| 12 | 300 | 632 | 17-Jan-13 | XXXXXXXX |
| 12 | 290 | 470 | 09-Jan-13 | XXXXXXXX |
| 12 | 400 | 661 | 15-Jan-13 | XXXXXXXX |
| 12 | 400 | 393 | 09-Jan-13 | XXXXXXXX |
| 12 | 350 | 678 | 17-Jan-13 | XXXXXXXX |
| 12 | 300 | 741 | 16-Jan-13 | XXXXXXXX |
| 12 | | | | XXXXXXXX |
| 12 | 300 | 346 | 07-Jan-13 | XXXXXXXX |
| 12 | 300 | 363 | 08-Jan-13 | XXXXXXXX |
| 12 | 300 | 843 | 17-Jan-13 | XXXXXXXX |
| 12 | 300 | 463 | 09-Jan-13 | XXXXXXXX |
| 12 | 300 | 571 | 14-Jan-13 | XXXXXXXX |
| 12 | 200 | 732 | 16-Jan-13 | XXXXXXXX |
| 12 | 320 | 796 | 22-Jan-13 | XXXXXXXX |
| 12 | 350 | 1193 | 21-Jan-13 | XXXXXXXX |
| 12 | 330 | 736 | 21-Jan-13 | XXXXXXXX |
| 12 | 800 | 1220 | 21-Jan-13 | XXXXXXXX |
| 12 | 350 | 1318 | 22-Jan-13 | XXXXXXXX |
| 12 | 370 | 611 | 15-Jan-13 | XXXXXXXX |
| 12 | 300 | 432 | 08-Jan-13 | XXXXXXXX |
| 12 | 350 | 374 | 08-Jan-13 | XXXXXXXX |
| 12 | 300 | 1061 | 21-Jan-13 | XXXXXXXX |
| 12 | 300 | 579 | 16-Jan-13 | XXXXXXXX |
| 12 | 350 | 518 | 09-Jan-13 | XXXXXXXX |
| 12 | 300 | 766 | 21-Jan-13 | XXXXXXXX |
| 12 | 300 | 637 | 15-Jan-13 | XXXXXXXX |
| 12 | 360 | 654 | 18-Jan-13 | XXXXXXXX |
| 12 | 250 | 498 | 09-Jan-13 | XXXXXXXX |
| 12 | 400 | 537 | 09-Jan-13 | XXXXXXXX |
| 12 | 350 | 1072 | 21-Jan-13 | XXXXXXXX |
| 12 | 300 | 484 | 10-Jan-13 | XXXXXXXX |
| 12 | 300 | 825 | 17-Jan-13 | XXXXXXXX |
| 12 | | 709 | 16-Jan-13 | XXXXXXXX |

| | | | | |
|---|---|---|---|---|
| 16 | 250 | 567 | 16-Jan-13 | XXXXXXXX |
| 12 | 250 | 431 | 08-Jan-13 | XXXXXXXX |
| 6 | 280 | 994 | 17-Jan-13 | XXXXXXXX |
| 6 | 420 | 427 | 08-Jan-13 | XXXXXXXX |
| 6 | 650 | 1078 | 21-Jan-13 | XXXXXXXX |
| 3 | 250 | 350 | 07-Jan-13 | XXXXXXXX |
| 3 | 200 | 684 | 21-Jan-13 | XXXXXXXX |
| 3 | 240 | 415 | 08-Jan-13 | XXXXXXXX |
| 3 | | 1139 | 21-Jan-13 | XXXXXXXX |
| 3 | 250 | 555 | 14-Jan-13 | XXXXXXXX |
| 3 | 200 | 624 | 15-Jan-13 | XXXXXXXX |
| 3 | 200 | 482 | 09-Jan-13 | XXXXXXXX |
| 3 | 150 | 746 | 21-Jan-13 | XXXXXXXX |
| 6 | 150 | 1262 | 22-Jan-13 | XXXXXXXX |
| 3 | 250 | 953 | 17-Jan-13 | XXXXXXXX |
| 3 | 200 | 547 | 14-Jan-13 | XXXXXXXX |
| 3 | | 705 | 16-Jan-13 | XXXXXXXX |
| 3 | | 818 | 22-Jan-13 | XXXXXXXX |
| 3 | | 749 | 16-Jan-13 | XXXXXXXX |
| 3 | | 674 | 16-Jan-13 | XXXXXXXX |
| 3 | 250 | 349 | 07-Jan-13 | XXXXXXXX |
| 3 | 300 | 998 | 17-Jan-13 | XXXXXXXX |
| 3 | 250 | | | XXXXXXXX |
| 3 | 150 | 793 | 22-Jan-13 | XXXXXXXX |
| 3 | 100 | 567 | 14-Jan-13 | XXXXXXXX |
| 3 | 300 | 573 | 14-Jan-13 | XXXXXXXX |
| 3 | 300 | 430 | 14-Jan-13 | XXXXXXXX |
| 3 | 150 | 816 | 17-Jan-13 | XXXXXXXX |
| 3 | 300 | 504 | 15-Jan-13 | XXXXXXXX |
| 3 | 200 | 675 | 21-Jan-13 | XXXXXXXX |
| 3 | 200 | 521 | 09-Jan-13 | XXXXXXXX |
| 24 | 500 | 581 | 15-Jan-13 | XXXXXXXX |
| 24 | 550 | 534 | 16-Jan-13 | XXXXXXXX |
| 6 | 200 | 759 | 21-Jan-13 | XXXXXXXX |
| 6 | 180 | 379 | 07-Jan-13 | XXXXXXXX |
| 6 | 150 | 1142 | 21-Jan-13 | XXXXXXXX |
| 6 | 150 | 1234 | 21-Jan-13 | XXXXXXXX |
| 6 | 250 | 1161 | 21-Jan-13 | XXXXXXXX |
| 6 | 100 | 377 | 07-Jan-13 | XXXXXXXX |
| 6 | 300 | 1020 | 17-Jan-13 | XXXXXXXX |
| 3 | 300 | 572 | 14-Jan-13 | XXXXXXXX |
| 6 | 300 | 465 | 09-Jan-13 | XXXXXXXX |
| 6 | 250 | 866 | 17-Jan-13 | XXXXXXXX |
| 6 | 150 | 650 | 17-Jan-13 | XXXXXXXX |
| 6 | 200 | 613 | 17-Jan-13 | XXXXXXXX |
| 6 | 200 | 731 | 16-Jan-13 | XXXXXXXX |
| 6 | 200 | 705 | 21-Jan-13 | XXXXXXXX |
| 12 | 180 | 385 | 08-Jan-13 | XXXXXXXX |
| 12 | 250 | 592 | 15-Jan-13 | XXXXXXXX |
| 24 | 450 | 1007 | 17-Jan-13 | XXXXXXXX |

| | | | | |
|---|---|---|---|---|
| 24 | 350 | 410 | 08-Jan-13 | XXXXXXXX |
| 24 | 150 | | | XXXXXXXX |
| 24 | 300 | 749 | 21-Jan-13 | XXXXXXXX |
| 24 | 300 | 561 | 16-Jan-13 | XXXXXXXX |
| 24 | 300 | 626 | 15-Jan-13 | XXXXXXXX |
| 24 | 250 | 1143 | 21-Jan-13 | XXXXXXXX |
| 24 | 500 | 539 | 09-Jan-13 | XXXXXXXX |
| 24 | 600 | 685 | 16-Jan-13 | XXXXXXXX |
| 24 | 300 | 600 | 17-Jan-13 | XXXXXXXX |
| 24 | 300 | 692 | 21-Jan-13 | XXXXXXXX |
| 24 | 350 | 460 | 08-Jan-13 | XXXXXXXX |
| 6 | 400 | 478 | 15-Jan-13 | XXXXXXXX |
| 6 | | 684 | 16-Jan-13 | XXXXXXXX |
| 6 | | 754 | 16-Jan-13 | XXXXXXXX |
| 24 | 300 | 564 | 14-Jan-13 | XXXXXXXX |
| 6 | 350 | 369 | 07-Jan-13 | XXXXXXXX |
| 6 | 250 | 532 | 16-Jan-13 | XXXXXXXX |
| 6 | 300 | 578 | 16-Jan-13 | XXXXXXXX |
| 6 | 180 | 556 | 11-Jan-13 | XXXXXXXX |
| 6 | 200 | 773 | 16-Jan-13 | XXXXXXXX |
| 6 | 200 | | | XXXXXXXX |
| 6 | 200 | 785 | 16-Jan-13 | XXXXXXXX |
| 6 | 250 | 495 | 10-Jan-13 | XXXXXXXX |
| 3 | | | | XXXXXXXX |
| 3 | | 1140 | 21-Jan-13 | XXXXXXXX |
| 3 | 250 | 1327 | 22-Jan-13 | XXXXXXXX |
| 3 | 100 | 1289 | 22-Jan-13 | XXXXXXXX |
| 3 | | 680 | 16-Jan-13 | XXXXXXXX |
| 3 | 150 | 745 | 21-Jan-13 | XXXXXXXX |
| 3 | 150 | 1261 | 22-Jan-13 | XXXXXXXX |
| 3 | 250 | 956 | 17-Jan-13 | XXXXXXXX |
| 3 | 200 | 546 | 14-Jan-13 | XXXXXXXX |
| 3 | | 748 | 16-Jan-13 | XXXXXXXX |
| 3 | 300 | 997 | 17-Jan-13 | XXXXXXXX |
| 3 | 150 | 792 | 22-Jan-13 | XXXXXXXX |
| 3 | 300 | 429 | 14-Jan-13 | XXXXXXXX |
| 3 | 150 | 815 | 17-Jan-13 | XXXXXXXX |
| 3 | 300 | 1096 | 21-Jan-13 | XXXXXXXX |
| 3 | 250 | 618 | 16-Jan-13 | XXXXXXXX |
| 3 | 200 | 589 | 17-Jan-13 | XXXXXXXX |
| 3 | 200 | | | XXXXXXXX |
| 6 | 400 | 413 | 08-Jan-13 | XXXXXXXX |
| 6 | | | | XXXXXXXX |
| 6 | 450 | 412 | 08-Jan-13 | XXXXXXXX |
| 6 | 300 | 1134 | 21-Jan-13 | XXXXXXXX |
| 6 | | | | XXXXXXXX |
| 6 | 300 | 599 | 15-Jan-13 | XXXXXXXX |
| 6 | 300 | 566 | 14-Jan-13 | XXXXXXXX |
| 6 | 300 | 520 | 16-Jan-13 | XXXXXXXX |
| 6 | | 720 | 16-Jan-13 | XXXXXXXX |

| | | | | |
|---|---|---|---|---|
| 6 | 350 | 494 | 09-Jan-13 | XXXXXXXX |
| 6 | 300 | 1149 | 21-Jan-13 | XXXXXXXX |
| 6 | 300 | 927 | 17-Jan-13 | XXXXXXXX |
| 6 | 280 | 378 | 07-Jan-13 | XXXXXXXX |
| 6 | | 702 | 16-Jan-13 | XXXXXXXX |
| 6 | 300 | 547 | 11-Jan-13 | XXXXXXXX |
| 6 | 200 | 747 | 21-Jan-13 | XXXXXXXX |
| 6 | 450 | 593 | 15-Jan-13 | XXXXXXXX |
| 6 | 450 | 578 | 14-Jan-13 | XXXXXXXX |
| 6 | 550 | 533 | 16-Jan-13 | XXXXXXXX |
| 6 | 350 | 485 | 10-Jan-13 | XXXXXXXX |
| 6 | | 704 | 16-Jan-13 | XXXXXXXX |
| 6 | | 819 | 22-Jan-13 | XXXXXXXX |
| 6 | 500 | 655 | 18-Jan-13 | XXXXXXXX |
| 6 | 300 | 595 | 17-Jan-13 | XXXXXXXX |
| 6 | 250 | 601 | 16-Jan-13 | XXXXXXXX |
| 6 | | 747 | 16-Jan-13 | XXXXXXXX |
| 6 | 500 | 426 | 08-Jan-13 | XXXXXXXX |
| 6 | 450 | 412 | 08-Jan-13 | XXXXXXXX |
| 6 | 500 | 348 | 07-Jan-13 | XXXXXXXX |
| 6 | 400 | 1115 | 21-Jan-13 | XXXXXXXX |
| 6 | 350 | 1035 | 21-Jan-13 | XXXXXXXX |
| 6 | 400 | 996 | 17-Jan-13 | XXXXXXXX |
| 6 | 200 | 699 | 21-Jan-13 | XXXXXXXX |
| 6 | | | | XXXXXXXX |
| 6 | 200 | 365 | 07-Jan-13 | XXXXXXXX |
| 6 | 400 | 646 | 17-Jan-13 | XXXXXXXX |
| 6 | 300 | 467 | 09-Jan-13 | XXXXXXXX |
| 6 | 350 | 806 | 16-Jan-13 | XXXXXXXX |
| 6 | 500 | 433 | 14-Jan-13 | XXXXXXXX |
| 6 | 300 | 820 | 17-Jan-13 | XXXXXXXX |
| 6 | 300 | 642 | 15-Jan-13 | XXXXXXXX |
| 6 | 400 | 1095 | 21-Jan-13 | XXXXXXXX |
| 6 | 150 | 368 | 08-Jan-13 | XXXXXXXX |
| 6 | 200 | 611 | 17-Jan-13 | XXXXXXXX |
| 6 | 400 | 1187 | 21-Jan-13 | XXXXXXXX |
| 6 | 300 | 1222 | 21-Jan-13 | XXXXXXXX |
| 6 | 300 | 600 | 15-Jan-13 | XXXXXXXX |
| 6 | 400 | 508 | 15-Jan-13 | XXXXXXXX |
| 6 | 350 | 414 | 08-Jan-13 | XXXXXXXX |
| 6 | 300 | 413 | 08-Jan-13 | XXXXXXXX |
| 6 | 400 | 1325 | 22-Jan-13 | XXXXXXXX |
| 6 | 400 | 514 | 09-Jan-13 | XXXXXXXX |
| 6 | 350 | 522 | 09-Jan-13 | XXXXXXXX |
| 12 | 500 | 640 | 18-Jan-13 | XXXXXXXX |
| 12 | | 516 | 09-Jan-13 | XXXXXXXX |
| 6 | 350 | 543 | 16-Jan-13 | XXXXXXXX |
| 6 | 350 | 1240 | 22-Jan-13 | XXXXXXXX |
| 6 | 350 | 497 | 15-Jan-13 | XXXXXXXX |
| 6 | 300 | 612 | 16-Jan-13 | XXXXXXXX |

| | | | | |
|---|---|---|---|---|
| 6 | 300 | 418 | 08-Jan-13 | XXXXXXXX |
| 6 | 350 | 646 | 18-Jan-13 | XXXXXXXX |
| 6 | 380 | 1231 | 21-Jan-13 | XXXXXXXX |
| 6 | 350 | | | XXXXXXXX |
| 6 | 550 | 911 | 17-Jan-13 | XXXXXXXX |
| 6 | 350 | 954 | 17-Jan-13 | XXXXXXXX |
| 6 | 300 | 782 | 16-Jan-13 | XXXXXXXX |
| 6 | 390 | 501 | 15-Jan-13 | XXXXXXXX |
| 6 | 400 | 566 | 14-Jan-13 | XXXXXXXX |
| 6 | 350 | 803 | 16-Jan-13 | XXXXXXXX |
| 6 | 400 | 432 | 14-Jan-13 | XXXXXXXX |
| 6 | 500 | 1102 | 21-Jan-13 | XXXXXXXX |
| 6 | 350 | 365 | 08-Jan-13 | XXXXXXXX |
| 6 | 450 | 840 | 17-Jan-13 | XXXXXXXX |
| 6 | 450 | 645 | 15-Jan-13 | XXXXXXXX |
| 6 | 500 | 668 | 15-Jan-13 | XXXXXXXX |
| 6 | 400 | 598 | 15-Jan-13 | XXXXXXXX |
| 6 | 450 | 442 | 08-Jan-13 | XXXXXXXX |
| 6 | 400 | 509 | 15-Jan-13 | XXXXXXXX |
| 6 | 400 | 540 | 11-Jan-13 | XXXXXXXX |
| 6 | 400 | 362 | 07-Jan-13 | XXXXXXXX |
| 12 | 600 | 683 | 21-Jan-13 | XXXXXXXX |
| 12 | 800 | 726 | 21-Jan-13 | XXXXXXXX |
| 12 | 450 | 547 | 16-Jan-13 | XXXXXXXX |
| 12 | 450 | 977 | 17-Jan-13 | XXXXXXXX |
| 12 | 550 | 611 | 16-Jan-13 | XXXXXXXX |
| 12 | 600 | 601 | 15-Jan-13 | XXXXXXXX |
| 12 | 550 | 567 | 14-Jan-13 | XXXXXXXX |
| 12 | 500 | 915 | 17-Jan-13 | XXXXXXXX |
| 12 | 500 | | | XXXXXXXX |
| 12 | 600 | 608 | 15-Jan-13 | XXXXXXXX |
| 12 | 550 | 632 | 17-Jan-13 | XXXXXXXX |
| 12 | 650 | | | XXXXXXXX |
| 12 | 580 | 502 | 15-Jan-13 | XXXXXXXX |
| 12 | 100 | 1284 | 22-Jan-13 | XXXXXXXX |
| 12 | | 563 | 14-Jan-13 | XXXXXXXX |
| 12 | 500 | 408 | 10-Jan-13 | XXXXXXXX |
| 12 | 500 | 643 | 15-Jan-13 | XXXXXXXX |
| 12 | 700 | | | XXXXXXXX |
| 12 | 600 | 658 | 15-Jan-13 | XXXXXXXX |
| 12 | 500 | 439 | 08-Jan-13 | XXXXXXXX |
| 12 | 600 | 575 | 16-Jan-13 | XXXXXXXX |
| 12 | 600 | 1293 | 22-Jan-13 | XXXXXXXX |
| 12 | 450 | 1019 | 17-Jan-13 | XXXXXXXX |
| 12 | 550 | 538 | 16-Jan-13 | XXXXXXXX |
| 12 | 500 | 628 | 15-Jan-13 | XXXXXXXX |
| 12 | 350 | 459 | 08-Jan-13 | XXXXXXXX |
| 6 | | 755 | 16-Jan-13 | XXXXXXXX |
| 6 | 650 | 414 | 10-Jan-13 | XXXXXXXX |
| 6 | 550 | 636 | 15-Jan-13 | XXXXXXXX |

| | | | | |
|---|---|---|---|---|
| 6 | 600 | 1004 | 17-Jan-13 | XXXXXXXX |
| 6 | 250 | 417 | 08-Jan-13 | XXXXXXXX |
| 6 | 300 | 342 | 07-Jan-13 | XXXXXXXX |
| 6 | 350 | 1239 | 22-Jan-13 | XXXXXXXX |
| 6 | 300 | 495 | 15-Jan-13 | XXXXXXXX |
| 6 | 500 | 501 | 10-Jan-13 | XXXXXXXX |
| 6 | 300 | 789 | 16-Jan-13 | XXXXXXXX |
| 6 | 400 | 431 | 14-Jan-13 | XXXXXXXX |
| 6 | 280 | 352 | 08-Jan-13 | XXXXXXXX |
| 6 | 300 | 648 | 15-Jan-13 | XXXXXXXX |
| 6 | 300 | 599 | 15-Jan-13 | XXXXXXXX |
| 6 | 350 | 361 | 07-Jan-13 | XXXXXXXX |
| 6 | 300 | 494 | 15-Jan-13 | XXXXXXXX |
| 6 | 300 | 888 | 17-Jan-13 | XXXXXXXX |
| 6 | 270 | 409 | 10-Jan-13 | XXXXXXXX |
| 6 | 250 | 628 | 15-Jan-13 | XXXXXXXX |
| 6 | 270 | 546 | 11-Jan-13 | XXXXXXXX |
| 6 | 600 | 400 | 09-Jan-13 | XXXXXXXX |
| 6 | 350 | 460 | 14-Jan-13 | XXXXXXXX |
| 6 | 500 | 502 | 10-Jan-13 | XXXXXXXX |
| 6 | 350 | 549 | 14-Jan-13 | XXXXXXXX |
| 6 | 250 | 951 | 17-Jan-13 | XXXXXXXX |
| 6 | 300 | 781 | 16-Jan-13 | XXXXXXXX |
| 6 | 300 | 341 | 07-Jan-13 | XXXXXXXX |
| 6 | 200 | 1032 | 21-Jan-13 | XXXXXXXX |
| 6 | 300 | 878 | 17-Jan-13 | XXXXXXXX |
| 6 | 250 | 630 | 17-Jan-13 | XXXXXXXX |
| 6 | 250 | 367 | 08-Jan-13 | XXXXXXXX |
| 6 | 300 | 839 | 17-Jan-13 | XXXXXXXX |
| 6 | 300 | 573 | 14-Jan-13 | XXXXXXXX |
| 6 | 300 | 510 | 10-Jan-13 | XXXXXXXX |
| 6 | 390 | 802 | 22-Jan-13 | XXXXXXXX |
| 6 | 270 | 1059 | 21-Jan-13 | XXXXXXXX |
| 6 | 270 | 395 | 08-Jan-13 | XXXXXXXX |
| 6 | 300 | 361 | 07-Jan-13 | XXXXXXXX |
| 6 | 300 | 1294 | 22-Jan-13 | XXXXXXXX |
| 6 | 900 | 719 | 21-Jan-13 | XXXXXXXX |
| 6 | 700 | 1009 | 17-Jan-13 | XXXXXXXX |
| 6 | 650 | 557 | 16-Jan-13 | XXXXXXXX |
| 6 | 650 | 903 | 17-Jan-13 | XXXXXXXX |
| 6 | | 395 | 09-Jan-13 | XXXXXXXX |
| 6 | 850 | 552 | 14-Jan-13 | XXXXXXXX |
| 6 | 750 | 558 | 14-Jan-13 | XXXXXXXX |
| 6 | 1000 | 636 | 17-Jan-13 | XXXXXXXX |
| 6 | 690 | 434 | 08-Jan-13 | XXXXXXXX |
| 6 | 750 | 707 | 21-Jan-13 | XXXXXXXX |
| 6 | 650 | 914 | 17-Jan-13 | XXXXXXXX |
| 6 | 650 | 623 | 17-Jan-13 | XXXXXXXX |
| 6 | 1100 | 722 | 21-Jan-13 | XXXXXXXX |
| 6 | 950 | 1005 | 17-Jan-13 | XXXXXXXX |

| | | | | |
|---|---|---|---|---|
| 6 | 1000 | 546 | 16-Jan-13 | XXXXXXXX |
| 6 | 950 | 496 | 15-Jan-13 | XXXXXXXX |
| 6 | 950 | 470 | 15-Jan-13 | XXXXXXXX |
| 6 | 900 | 623 | 15-Jan-13 | XXXXXXXX |
| 6 | 950 | 1088 | 21-Jan-13 | XXXXXXXX |
| 6 | 1000 | 1267 | 22-Jan-13 | XXXXXXXX |
| 6 | 960 | 962 | 17-Jan-13 | XXXXXXXX |
| 6 | 1000 | 543 | 14-Jan-13 | XXXXXXXX |
| 6 | 980 | 665 | 17-Jan-13 | XXXXXXXX |
| 6 | 1500 | 820 | 22-Jan-13 | XXXXXXXX |
| 6 | 950 | 1050 | 21-Jan-13 | XXXXXXXX |
| 6 | 1000 | 583 | 16-Jan-13 | XXXXXXXX |
| 6 | 1000 | 557 | 14-Jan-13 | XXXXXXXX |
| 6 | 950 | 870 | 17-Jan-13 | XXXXXXXX |
| 6 | 1000 | 637 | 17-Jan-13 | XXXXXXXX |
| 6 | 900 | 807 | 16-Jan-13 | XXXXXXXX |
| 6 | 900 | 496 | 10-Jan-13 | XXXXXXXX |
| 6 | 1000 | 1129 | 21-Jan-13 | XXXXXXXX |
| 6 | 1040 | 1100 | 21-Jan-13 | XXXXXXXX |
| 6 | 500 | 1166 | 21-Jan-13 | XXXXXXXX |
| 6 | 1000 | 847 | 17-Jan-13 | XXXXXXXX |
| 6 | 1000 | 1151 | 21-Jan-13 | XXXXXXXX |
| 6 | 1080 | 795 | 22-Jan-13 | XXXXXXXX |
| 6 | 1000 | 738 | 21-Jan-13 | XXXXXXXX |
| 6 | 950 | 1218 | 21-Jan-13 | XXXXXXXX |
| 6 | 1000 | 649 | 15-Jan-13 | XXXXXXXX |
| 6 | 1050 | 666 | 15-Jan-13 | XXXXXXXX |
| 6 | 1040 | 612 | 15-Jan-13 | XXXXXXXX |
| 6 | 950 | 450 | 14-Jan-13 | XXXXXXXX |
| 6 | 980 | 690 | 16-Jan-13 | XXXXXXXX |
| 6 | 900 | 576 | 16-Jan-13 | XXXXXXXX |
| 6 | 950 | 1300 | 22-Jan-13 | XXXXXXXX |
| 6 | 580 | 555 | 11-Jan-13 | XXXXXXXX |
| 4 | 200 | 559 | 14-Jan-13 | XXXXXXXX |
| 9 | 350 | 675 | 16-Jan-13 | XXXXXXXX |
| 6 | 380 | 421 | 08-Jan-13 | XXXXXXXX |
| 9 | 500 | 642 | 18-Jan-13 | XXXXXXXX |
| 9 | 400 | 1323 | 22-Jan-13 | XXXXXXXX |
| 9 | 550 | 1092 | 21-Jan-13 | XXXXXXXX |
| 9 | 500 | 347 | 07-Jan-13 | XXXXXXXX |
| 6 | 660 | 530 | 16-Jan-13 | XXXXXXXX |
| 6 | 550 | 1153 | 21-Jan-13 | XXXXXXXX |
| 6 | 500 | 583 | 15-Jan-13 | XXXXXXXX |
| 6 | 550 | 526 | 16-Jan-13 | XXXXXXXX |
| 3 | 500 | 632 | 15-Jan-13 | XXXXXXXX |
| 3 | 600 | | 20-Sep-13 | XXXXXXXX |
| 3 | 600 | 396 | 09-Jan-13 | XXXXXXXX |
| 3 | 650 | 1268 | 22-Jan-13 | XXXXXXXX |
| 3 | 520 | 614 | 15-Jan-13 | XXXXXXXX |
| 3 | 450 | | 20-Sep-13 | XXXXXXXX |

| | | | | |
|---|---|---|---|---|
| 3 | 650 | 561 | 14-Jan-13 | XXXXXXXX |
| 3 | 650 | 803 | 22-Jan-13 | XXXXXXXX |
| 3 | 650 | 1317 | 22-Jan-13 | XXXXXXXX |
| 12 | 300 | 999 | 17-Jan-13 | XXXXXXXX |
| 3 | 720 | | 20-Sep-13 | XXXXXXXX |
| 3 | 800 | 788 | 22-Jan-13 | XXXXXXXX |
| 3 | 700 | 493 | 09-Jan-13 | XXXXXXXX |
| 3 | 700 | 562 | 16-Jan-13 | XXXXXXXX |
| 3 | 850 | 455 | 14-Jan-13 | XXXXXXXX |
| 3 | 850 | 1266 | 22-Jan-13 | XXXXXXXX |
| 3 | 780 | 612 | 15-Jan-13 | XXXXXXXX |
| 3 | 850 | 671 | 17-Jan-13 | XXXXXXXX |
| 3 | 850 | 523 | 09-Jan-13 | XXXXXXXX |
| 3 | 600 | 562 | 14-Jan-13 | XXXXXXXX |
| 3 | 700 | 808 | 16-Jan-13 | XXXXXXXX |
| 3 | 800 | 920 | 17-Jan-13 | XXXXXXXX |
| 3 | 720 | 841 | 17-Jan-13 | XXXXXXXX |
| 3 | 950 | 1315 | 22-Jan-13 | XXXXXXXX |
| 3 | 800 | 657 | 15-Jan-13 | XXXXXXXX |
| 3 | 850 | 665 | 15-Jan-13 | XXXXXXXX |
| 3 | 650 | 694 | 16-Jan-13 | XXXXXXXX |
| 3 | 480 | 1067 | 21-Jan-13 | XXXXXXXX |
| 3 | 750 | 1304 | 22-Jan-13 | XXXXXXXX |
| 8 | 400 | 390 | 07-Jan-13 | XXXXXXXX |
| 8 | 380 | 565 | 16-Jan-13 | XXXXXXXX |
| 8 | 500 | 893 | 17-Jan-13 | XXXXXXXX |
| 8 | 400 | 602 | 17-Jan-13 | XXXXXXXX |
| 8 | 850 | 449 | 08-Jan-13 | XXXXXXXX |
| 8 | 500 | 428 | 14-Jan-13 | XXXXXXXX |
| 8 | 400 | 357 | 08-Jan-13 | XXXXXXXX |
| 8 | 450 | 618 | 17-Jan-13 | XXXXXXXX |
| 8 | 450 | 460 | 09-Jan-13 | XXXXXXXX |
| 8 | 480 | 428 | 08-Jan-13 | XXXXXXXX |
| 8 | 500 | 589 | 15-Jan-13 | XXXXXXXX |
| 8 | 550 | 454 | 14-Jan-13 | XXXXXXXX |
| 8 | 400 | 653 | 17-Jan-13 | XXXXXXXX |
| 12 | 350 | 758 | 21-Jan-13 | XXXXXXXX |
| 12 | 450 | 648 | 18-Jan-13 | XXXXXXXX |
| 12 | 450 | 634 | 15-Jan-13 | XXXXXXXX |
| 12 | 300 | 332 | 07-Jan-13 | XXXXXXXX |
| 12 | 300 | 682 | 21-Jan-13 | XXXXXXXX |
| 12 | 350 | 725 | 21-Jan-13 | XXXXXXXX |
| 12 | 350 | 1275 | 22-Jan-13 | XXXXXXXX |
| 12 | 350 | 785 | 22-Jan-13 | XXXXXXXX |
| 12 | 300 | 676 | 16-Jan-13 | XXXXXXXX |
| 12 | 300 | 1251 | 22-Jan-13 | XXXXXXXX |
| 12 | 350 | 1260 | 22-Jan-13 | XXXXXXXX |
| 12 | 350 | | 21-Jan-13 | XXXXXXXX |
| 12 | 300 | 360 | 07-Jan-13 | XXXXXXXX |
| 12 | 300 | 380 | 07-Jan-13 | XXXXXXXX |

| | | | | |
|---|---|---|---|---|
| 12 | 300 | 1110 | 21-Jan-13 | XXXXXXXX |
| 12 | 350 | 737 | 16-Jan-13 | XXXXXXXX |
| 12 | 300 | | | XXXXXXXX |
| 12 | | 1136 | 21-Jan-13 | XXXXXXXX |
| 12 | 350 | 750 | 21-Jan-13 | XXXXXXXX |
| 12 | 350 | 407 | 08-Jan-13 | XXXXXXXX |
| 12 | 350 | 336 | 07-Jan-13 | XXXXXXXX |
| 12 | 450 | 391 | 08-Jan-13 | XXXXXXXX |
| 12 | 300 | 490 | 15-Jan-13 | XXXXXXXX |
| 12 | 350 | 935 | 17-Jan-13 | XXXXXXXX |
| 12 | 300 | 794 | 16-Jan-13 | XXXXXXXX |
| 12 | 300 | 613 | 16-Jan-13 | XXXXXXXX |
| 12 | 300 | 616 | 15-Jan-13 | XXXXXXXX |
| 12 | 300 | 600 | 15-Jan-13 | XXXXXXXX |
| 12 | 350 | 686 | 21-Jan-13 | XXXXXXXX |
| 12 | 300 | 637 | 17-Jan-13 | XXXXXXXX |
| 12 | 300 | 414 | 08-Jan-13 | XXXXXXXX |
| 12 | 300 | 496 | 09-Jan-13 | XXXXXXXX |
| 12 | 400 | 690 | 21-Jan-13 | XXXXXXXX |
| 12 | 300 | 539 | 16-Jan-13 | XXXXXXXX |
| 12 | 280 | 647 | 18-Jan-13 | XXXXXXXX |
| 12 | 300 | 531 | 09-Jan-13 | XXXXXXXX |
| 12 | 300 | 1326 | 22-Jan-13 | XXXXXXXX |
| 12 | 300 | 887 | 17-Jan-13 | XXXXXXXX |
| 12 | 300 | 926 | 17-Jan-13 | XXXXXXXX |
| 12 | 300 | 464 | 10-Jan-13 | XXXXXXXX |
| 12 | 280 | 384 | 07-Jan-13 | XXXXXXXX |
| 12 | 280 | 631 | 15-Jan-13 | XXXXXXXX |
| 12 | 300 | 552 | 11-Jan-13 | XXXXXXXX |
| 12 | 280 | 904 | 17-Jan-13 | XXXXXXXX |
| 12 | 300 | 726 | 16-Jan-13 | XXXXXXXX |
| 12 | 300 | 1148 | 21-Jan-13 | XXXXXXXX |
| 12 | 350 | 772 | 16-Jan-13 | XXXXXXXX |
| 12 | 400 | 525 | 16-Jan-13 | XXXXXXXX |
| 12 | 280 | 479 | 09-Jan-13 | XXXXXXXX |
| 12 | 400 | 542 | 09-Jan-13 | XXXXXXXX |
| 12 | 350 | 714 | 16-Jan-13 | XXXXXXXX |
| 12 | 300 | 1236 | 21-Jan-13 | XXXXXXXX |
| 12 | 300 | 626 | 17-Jan-13 | XXXXXXXX |
| 12 | 300 | 993 | 17-Jan-13 | XXXXXXXX |
| 12 | 350 | 686 | 16-Jan-13 | XXXXXXXX |
| 12 | 350 | 456 | 14-Jan-13 | XXXXXXXX |
| 12 | 350 | 481 | 15-Jan-13 | XXXXXXXX |
| 12 | 300 | 739 | 21-Jan-13 | XXXXXXXX |
| 12 | 400 | 503 | 10-Jan-13 | XXXXXXXX |
| 12 | 350 | 1159 | 21-Jan-13 | XXXXXXXX |
| 12 | 350 | 1071 | 21-Jan-13 | XXXXXXXX |
| 12 | 350 | 579 | 15-Jan-13 | XXXXXXXX |
| 12 | 350 | 952 | 17-Jan-13 | XXXXXXXX |
| 12 | 350 | 580 | 14-Jan-13 | XXXXXXXX |

| | | | | |
|---|---|---|---|---|
| 12 | 300 | 777 | 16-Jan-13 | XXXXXXXX |
| 12 | 350 | 527 | 16-Jan-13 | XXXXXXXX |
| 12 | 300 | 605 | 17-Jan-13 | XXXXXXXX |
| 12 | 340 | 596 | 16-Jan-13 | XXXXXXXX |
| 12 | 350 | 666 | 17-Jan-13 | XXXXXXXX |
| 12 | 350 | 337 | 07-Jan-13 | XXXXXXXX |
| 12 | 400 | 938 | 17-Jan-13 | XXXXXXXX |
| 12 | 350 | 586 | 17-Jan-13 | XXXXXXXX |
| 12 | 400 | 657 | 18-Jan-13 | XXXXXXXX |
| 12 | 300 | 592 | 17-Jan-13 | XXXXXXXX |
| 12 | 350 | 610 | 16-Jan-13 | XXXXXXXX |
| 12 | 300 | 603 | 16-Jan-13 | XXXXXXXX |
| 12 | 385 | 446 | 08-Jan-13 | XXXXXXXX |
| 12 | 400 | 384 | 08-Jan-13 | XXXXXXXX |
| 12 | 300 | 1058 | 21-Jan-13 | XXXXXXXX |
| 12 | 300 | 456 | 08-Jan-13 | XXXXXXXX |
| 12 | 300 | 699 | 21-Jan-13 | XXXXXXXX |
| 12 | 300 | 1113 | 21-Jan-13 | XXXXXXXX |
| 12 | 300 | 503 | 15-Jan-13 | XXXXXXXX |
| 12 | 300 | 1036 | 21-Jan-13 | XXXXXXXX |
| 12 | 300 | 813 | 16-Jan-13 | XXXXXXXX |
| 12 | 300 | | | XXXXXXXX |
| 12 | 300 | 1171 | 21-Jan-13 | XXXXXXXX |
| 12 | 300 | 1052 | 21-Jan-13 | XXXXXXXX |
| 12 | 300 | 475 | 10-Jan-13 | XXXXXXXX |
| 12 | 300 | 356 | 07-Jan-13 | XXXXXXXX |
| 12 | 300 | 756 | 21-Jan-13 | XXXXXXXX |
| 12 | 300 | 771 | 21-Jan-13 | XXXXXXXX |
| 12 | 300 | 526 | 11-Jan-13 | XXXXXXXX |
| 12 | 350 | 622 | 15-Jan-13 | XXXXXXXX |
| 12 | 350 | 876 | 17-Jan-13 | XXXXXXXX |
| 12 | 300 | 641 | 17-Jan-13 | XXXXXXXX |
| 12 | 380 | 647 | 17-Jan-13 | XXXXXXXX |
| 12 | 380 | 622 | 15-Jan-13 | XXXXXXXX |
| 12 | 300 | 661 | 17-Jan-13 | XXXXXXXX |
| 12 | 450 | 446 | 09-Jan-13 | XXXXXXXX |
| 12 | | | | XXXXXXXX |
| 12 | 300 | 472 | 09-Jan-13 | XXXXXXXX |
| 12 | 300 | 626 | 17-Jan-13 | XXXXXXXX |
| 12 | 350 | 864 | 17-Jan-13 | XXXXXXXX |
| 12 | 300 | 802 | 16-Jan-13 | XXXXXXXX |
| 12 | 300 | 504 | 09-Jan-13 | XXXXXXXX |
| 12 | 350 | 436 | 14-Jan-13 | XXXXXXXX |
| 12 | 450 | 404 | 10-Jan-13 | XXXXXXXX |
| 12 | 300 | 824 | 17-Jan-13 | XXXXXXXX |
| 12 | 300 | 648 | 17-Jan-13 | XXXXXXXX |
| 12 | 300 | 468 | 09-Jan-13 | XXXXXXXX |
| 12 | 350 | 921 | 17-Jan-13 | XXXXXXXX |
| 12 | 300 | 639 | 15-Jan-13 | XXXXXXXX |
| 12 | 300 | 490 | 10-Jan-13 | XXXXXXXX |

| | | | | |
|---|---|---|---|---|
| 12 | 300 | 767 | 21-Jan-13 | XXXXXXXX |
| 12 | 400 | 386 | 08-Jan-13 | XXXXXXXX |
| 12 | 300 | 1133 | 21-Jan-13 | XXXXXXXX |
| 12 | 300 | 1101 | 21-Jan-13 | XXXXXXXX |
| 12 | 350 | 676 | 17-Jan-13 | XXXXXXXX |
| 12 | 300 | 740 | 16-Jan-13 | XXXXXXXX |
| 12 | 300 | 345 | 07-Jan-13 | XXXXXXXX |
| 12 | 300 | 359 | 08-Jan-13 | XXXXXXXX |
| 12 | 500 | 712 | 21-Jan-13 | XXXXXXXX |
| 12 | 350 | 372 | 08-Jan-13 | XXXXXXXX |
| 12 | 400 | 1167 | 21-Jan-13 | XXXXXXXX |
| 12 | 350 | 461 | 09-Jan-13 | XXXXXXXX |
| 12 | 300 | 572 | 14-Jan-13 | XXXXXXXX |
| 12 | 350 | 457 | 08-Jan-13 | XXXXXXXX |
| 12 | 350 | 511 | 10-Jan-13 | XXXXXXXX |
| 12 | 300 | 734 | 16-Jan-13 | XXXXXXXX |
| 12 | 500 | 354 | 08-Jan-13 | XXXXXXXX |
| 12 | 320 | 799 | 22-Jan-13 | XXXXXXXX |
| 12 | 350 | 1191 | 21-Jan-13 | XXXXXXXX |
| 12 | 350 | 686 | 21-Jan-13 | XXXXXXXX |
| 12 | 330 | 729 | 21-Jan-13 | XXXXXXXX |
| 12 | 800 | 1223 | 21-Jan-13 | XXXXXXXX |
| 12 | 350 | 656 | 15-Jan-13 | XXXXXXXX |
| 12 | 350 | 609 | 15-Jan-13 | XXXXXXXX |
| 12 | 300 | 563 | 14-Jan-13 | XXXXXXXX |
| 12 | 300 | 671 | 15-Jan-13 | XXXXXXXX |
| 12 | 350 | 423 | 14-Jan-13 | XXXXXXXX |
| 12 | 300 | 597 | 15-Jan-13 | XXXXXXXX |
| 12 | 300 | 465 | 14-Jan-13 | XXXXXXXX |
| 12 | 300 | 693 | 16-Jan-13 | XXXXXXXX |
| 12 | 350 | 375 | 08-Jan-13 | XXXXXXXX |
| 12 | 300 | 568 | 16-Jan-13 | XXXXXXXX |
| 12 | 300 | 671 | 21-Jan-13 | XXXXXXXX |
| 12 | 300 | 538 | 11-Jan-13 | XXXXXXXX |
| 12 | 350 | 520 | 09-Jan-13 | XXXXXXXX |
| 12 | 300 | 366 | 07-Jan-13 | XXXXXXXX |
| 12 | 300 | 764 | 21-Jan-13 | XXXXXXXX |
| 12 | 350 | 1299 | 22-Jan-13 | XXXXXXXX |
| 8 | 420 | 368 | 07-Jan-13 | XXXXXXXX |
| 8 | 400 | 630 | 15-Jan-13 | XXXXXXXX |
| 8 | 500 | 905 | 17-Jan-13 | XXXXXXXX |
| 8 | 300 | 1037 | 21-Jan-13 | XXXXXXXX |
| 8 | 650 | 871 | 17-Jan-13 | XXXXXXXX |
| 8 | 400 | 1188 | 21-Jan-13 | XXXXXXXX |
| 8 | 500 | 490 | 09-Jan-13 | XXXXXXXX |
| 8 | 550 | 643 | 18-Jan-13 | XXXXXXXX |
| 8 | 650 | 377 | 07-Jan-13 | XXXXXXXX |
| 8 | 600 | 908 | 17-Jan-13 | XXXXXXXX |
| 8 | 500 | 477 | 15-Jan-13 | XXXXXXXX |
| 8 | 500 | 1025 | 21-Jan-13 | XXXXXXXX |

| | | | | |
|---|---|---|---|---|
| 8 | 500 | 783 | 22-Jan-13 | XXXXXXXX |
| 8 | 500 | 923 | 17-Jan-13 | XXXXXXXX |
| 8 | 500 | 603 | 15-Jan-13 | XXXXXXXX |
| 8 | 650 | 809 | 22-Jan-13 | XXXXXXXX |
| 8 | 650 | 1075 | 21-Jan-13 | XXXXXXXX |
| 9 | 1280 | 382 | 07-Jan-13 | XXXXXXXX |
| 9 | 1000 | 899 | 17-Jan-13 | XXXXXXXX |
| 9 | 950 | 634 | 17-Jan-13 | XXXXXXXX |
| 9 | 750 | 369 | 08-Jan-13 | XXXXXXXX |
| 9 | 1070 | 440 | 08-Jan-13 | XXXXXXXX |
| 8 | 440 | 591 | 16-Jan-13 | XXXXXXXX |
| 8 | 500 | 629 | 15-Jan-13 | XXXXXXXX |
| 8 | 600 | 846 | 17-Jan-13 | XXXXXXXX |
| 9 | 750 | 630 | 15-Jan-13 | XXXXXXXX |
| 8 | 650 | 1316 | 22-Jan-13 | XXXXXXXX |
| 8 | 600 | 427 | 14-Jan-13 | XXXXXXXX |
| 8 | 450 | 553 | 16-Jan-13 | XXXXXXXX |
| 8 | 400 | 476 | 15-Jan-13 | XXXXXXXX |
| 8 | 450 | 578 | 15-Jan-13 | XXXXXXXX |
| 8 | 500 | 610 | 15-Jan-13 | XXXXXXXX |
| 8 | 500 | 334 | 07-Jan-13 | XXXXXXXX |
| 8 | 650 | 721 | 21-Jan-13 | XXXXXXXX |
| 8 | 550 | 416 | 08-Jan-13 | XXXXXXXX |
| 8 | 450 | 544 | 16-Jan-13 | XXXXXXXX |
| 8 | 600 | 791 | 22-Jan-13 | XXXXXXXX |
| 8 | 600 | 387 | 08-Jan-13 | XXXXXXXX |
| 8 | 450 | 1271 | 22-Jan-13 | XXXXXXXX |
| 8 | 650 | 738 | 16-Jan-13 | XXXXXXXX |
| 8 | 550 | | | XXXXXXXX |
| 8 | 700 | 343 | 07-Jan-13 | XXXXXXXX |
| 8 | 650 | 930 | 17-Jan-13 | XXXXXXXX |
| 8 | 550 | 798 | 16-Jan-13 | XXXXXXXX |
| 8 | 500 | 917 | 17-Jan-13 | XXXXXXXX |
| 8 | 650 | 651 | 18-Jan-13 | XXXXXXXX |
| 8 | 850 | 617 | 15-Jan-13 | XXXXXXXX |
| 8 | 500 | 593 | 15-Jan-13 | XXXXXXXX |
| 8 | 500 | 416 | 08-Jan-13 | XXXXXXXX |
| 8 | 600 | 532 | 09-Jan-13 | XXXXXXXX |
| 8 | 500 | 1330 | 22-Jan-13 | XXXXXXXX |
| 8 | 550 | 470 | 10-Jan-13 | XXXXXXXX |
| 8 | 520 | 402 | 09-Jan-13 | XXXXXXXX |
| 8 | 550 | 1086 | 21-Jan-13 | XXXXXXXX |
| 8 | 480 | 987 | 17-Jan-13 | XXXXXXXX |
| 8 | 700 | 461 | 14-Jan-13 | XXXXXXXX |
| 8 | 600 | 486 | 15-Jan-13 | XXXXXXXX |
| 8 | 550 | 1163 | 21-Jan-13 | XXXXXXXX |
| 8 | 650 | 1070 | 21-Jan-13 | XXXXXXXX |
| 8 | 650 | 582 | 15-Jan-13 | XXXXXXXX |
| 8 | 500 | 575 | 14-Jan-13 | XXXXXXXX |
| 8 | 600 | 970 | 17-Jan-13 | XXXXXXXX |

| | | | | |
|---|---|---|---|---|
| 8 | 450 | 779 | 16-Jan-13 | XXXXXXXX |
| 8 | 450 | 544 | 14-Jan-13 | XXXXXXXX |
| 8 | 500 | 601 | 17-Jan-13 | XXXXXXXX |
| 8 | 550 | 631 | 17-Jan-13 | XXXXXXXX |
| 8 | 550 | 582 | 17-Jan-13 | XXXXXXXX |
| 8 | 650 | 444 | 08-Jan-13 | XXXXXXXX |
| 8 | 500 | 696 | 21-Jan-13 | XXXXXXXX |
| 8 | 550 | 409 | 08-Jan-13 | XXXXXXXX |
| 8 | 550 | 499 | 15-Jan-13 | XXXXXXXX |
| 8 | 550 | 335 | 07-Jan-13 | XXXXXXXX |
| 8 | 450 | 1029 | 21-Jan-13 | XXXXXXXX |
| 8 | 500 | | | XXXXXXXX |
| 8 | 500 | 1054 | 21-Jan-13 | XXXXXXXX |
| 8 | 500 | 379 | 08-Jan-13 | XXXXXXXX |
| 8 | 500 | 1279 | 22-Jan-13 | XXXXXXXX |
| 8 | 500 | 364 | 07-Jan-13 | XXXXXXXX |
| 8 | 650 | 769 | 21-Jan-13 | XXXXXXXX |
| 8 | 480 | 535 | 11-Jan-13 | XXXXXXXX |
| 8 | 650 | 623 | 15-Jan-13 | XXXXXXXX |
| 8 | 600 | 524 | 09-Jan-13 | XXXXXXXX |
| 8 | 700 | 868 | 17-Jan-13 | XXXXXXXX |
| 8 | 500 | 635 | 17-Jan-13 | XXXXXXXX |
| 8 | 550 | 608 | 15-Jan-13 | XXXXXXXX |
| 8 | 550 | 437 | 14-Jan-13 | XXXXXXXX |
| 8 | 600 | 882 | 17-Jan-13 | XXXXXXXX |
| 8 | 500 | 651 | 17-Jan-13 | XXXXXXXX |
| 8 | 460 | 1131 | 21-Jan-13 | XXXXXXXX |
| 8 | 450 | 742 | 16-Jan-13 | XXXXXXXX |
| 8 | 600 | 1165 | 21-Jan-13 | XXXXXXXX |
| 8 | 500 | 844 | 17-Jan-13 | XXXXXXXX |
| 8 | 480 | 616 | 17-Jan-13 | XXXXXXXX |
| 8 | 530 | 457 | 09-Jan-13 | XXXXXXXX |
| 8 | 500 | 1152 | 21-Jan-13 | XXXXXXXX |
| 8 | 520 | 798 | 22-Jan-13 | XXXXXXXX |
| 8 | 550 | 689 | 21-Jan-13 | XXXXXXXX |
| 8 | 500 | 730 | 21-Jan-13 | XXXXXXXX |
| 8 | 550 | 762 | 16-Jan-13 | XXXXXXXX |
| 8 | 600 | 1310 | 22-Jan-13 | XXXXXXXX |
| 8 | 500 | 654 | 15-Jan-13 | XXXXXXXX |
| 8 | 560 | 654 | 17-Jan-13 | XXXXXXXX |
| 8 | 450 | 559 | 14-Jan-13 | XXXXXXXX |
| 8 | 500 | 596 | 15-Jan-13 | XXXXXXXX |
| 8 | 500 | 430 | 08-Jan-13 | XXXXXXXX |
| 8 | 500 | 703 | 21-Jan-13 | XXXXXXXX |
| 8 | 500 | 510 | 15-Jan-13 | XXXXXXXX |
| 8 | 550 | 481 | 10-Jan-13 | XXXXXXXX |
| 8 | 500 | 394 | 08-Jan-13 | XXXXXXXX |
| 8 | 550 | 365 | 07-Jan-13 | XXXXXXXX |
| 8 | 480 | 763 | 21-Jan-13 | XXXXXXXX |
| 8 | 500 | 1296 | 22-Jan-13 | XXXXXXXX |

| | | | | |
|---|---|---|---|---|
| 8 | 600 | 333 | 07-Jan-13 | XXXXXXXX |
| 8 | 580 | 553 | 11-Jan-13 | XXXXXXXX |
| 8 | 650 | 804 | 22-Jan-13 | XXXXXXXX |
| 8 | 570 | 532 | 11-Jan-13 | XXXXXXXX |
| 8 | 650 | 649 | 17-Jan-13 | XXXXXXXX |
| 8 | 500 | 1130 | 21-Jan-13 | XXXXXXXX |
| 8 | 550 | 831 | 17-Jan-13 | XXXXXXXX |
| 8 | 500 | 348 | 07-Jan-13 | XXXXXXXX |
| 8 | 750 | 1002 | 17-Jan-13 | XXXXXXXX |
| 8 | 550 | 664 | 17-Jan-13 | XXXXXXXX |
| 8 | 650 | 621 | 17-Jan-13 | XXXXXXXX |
| 8 | 650 | 1022 | 21-Jan-13 | XXXXXXXX |
| 8 | 450 | 709 | 21-Jan-13 | XXXXXXXX |
| 8 | 500 | 423 | 08-Jan-13 | XXXXXXXX |
| 8 | 650 | 1081 | 21-Jan-13 | XXXXXXXX |
| 8 | 650 | 874 | 17-Jan-13 | XXXXXXXX |
| 8 | 500 | | | XXXXXXXX |
| 8 | 500 | 663 | 15-Jan-13 | XXXXXXXX |
| 8 | 450 | 1189 | 21-Jan-13 | XXXXXXXX |
| 8 | 550 | 683 | 21-Jan-13 | XXXXXXXX |
| 8 | 400 | 604 | 15-Jan-13 | XXXXXXXX |
| | 800 | 533 | 09-Jan-13 | XXXXXXXX |
| | 680 | 380 | 07-Jan-13 | XXXXXXXX |
| | 800 | 767 | 16-Jan-13 | XXXXXXXX |
| | 850 | 1247 | 22-Jan-13 | XXXXXXXX |
| | 700 | 574 | 14-Jan-13 | XXXXXXXX |
| | 700 | 1308 | 22-Jan-13 | XXXXXXXX |
| | 800 | 448 | 14-Jan-13 | XXXXXXXX |
| | 700 | 572 | 16-Jan-13 | XXXXXXXX |
| | 750 | 669 | 21-Jan-13 | XXXXXXXX |
| | 750 | 1295 | 22-Jan-13 | XXXXXXXX |
| | 450 | 991 | 17-Jan-13 | XXXXXXXX |
| 1 | 600 | 807 | 22-Jan-13 | XXXXXXXX |
| | 550 | 791 | 16-Jan-13 | XXXXXXXX |
| 1 | 600 | 808 | 22-Jan-13 | XXXXXXXX |
| | 550 | 544 | 11-Jan-13 | XXXXXXXX |
| 1 | 550 | 536 | 16-Jan-13 | XXXXXXXX |
| 1 | 350 | 398 | 09-Jan-13 | XXXXXXXX |
| 1 | 500 | 351 | 07-Jan-13 | XXXXXXXX |
| | 600 | 454 | 08-Jan-13 | XXXXXXXX |
| | 300 | 1065 | 21-Jan-13 | XXXXXXXX |
| | 400 | 1122 | 21-Jan-13 | XXXXXXXX |
| | 600 | 344 | 07-Jan-13 | XXXXXXXX |
| | 500 | 554 | 11-Jan-13 | XXXXXXXX |
| | 600 | 944 | 17-Jan-13 | XXXXXXXX |
| | 400 | 989 | 17-Jan-13 | XXXXXXXX |
| | 500 | 361 | 08-Jan-13 | XXXXXXXX |
| | 550 | 577 | 14-Jan-13 | XXXXXXXX |
| | 450 | 699 | 16-Jan-13 | XXXXXXXX |
| | 450 | 571 | 16-Jan-13 | XXXXXXXX |

| | | | | |
|---|---|---|---|---|
| | 600 | 826 | 17-Jan-13 | XXXXXXXX |
| 1 | 500 | 594 | 15-Jan-13 | XXXXXXXX |
| | 520 | 564 | 16-Jan-13 | XXXXXXXX |
| | 500 | 1319 | 22-Jan-13 | XXXXXXXX |
| | 600 | 943 | 17-Jan-13 | XXXXXXXX |
| | | 679 | 16-Jan-13 | XXXXXXXX |
| | 500 | 778 | 16-Jan-13 | XXXXXXXX |
| | 650 | 445 | 08-Jan-13 | XXXXXXXX |
| | 500 | 394 | 08-Jan-13 | XXXXXXXX |
| | 300 | 1216 | 21-Jan-13 | XXXXXXXX |
| | 500 | 668 | 21-Jan-13 | XXXXXXXX |
| | 350 | 995 | 17-Jan-13 | XXXXXXXX |
| | 450 | 381 | 07-Jan-13 | XXXXXXXX |
| | 450 | 548 | 11-Jan-13 | XXXXXXXX |
| | 550 | 588 | 15-Jan-13 | XXXXXXXX |
| | 600 | 1217 | 21-Jan-13 | XXXXXXXX |
| | 600 | 337 | 07-Jan-13 | XXXXXXXX |
| | 600 | 1248 | 22-Jan-13 | XXXXXXXX |
| | 700 | 420 | 08-Jan-13 | XXXXXXXX |
| | 500 | 638 | 15-Jan-13 | XXXXXXXX |
| | 630 | 667 | 21-Jan-13 | XXXXXXXX |
| | 500 | 430 | 08-Jan-13 | XXXXXXXX |
| 1 | 400 | 443 | 08-Jan-13 | XXXXXXXX |
| | 600 | 587 | 15-Jan-13 | XXXXXXXX |
| | 600 | 343 | 07-Jan-13 | XXXXXXXX |
| | 650 | 350 | 07-Jan-13 | XXXXXXXX |
| | | 1135 | 21-Jan-13 | XXXXXXXX |
| | 600 | 489 | 15-Jan-13 | XXXXXXXX |
| | | | | XXXXXXXX |
| | 550 | 597 | 15-Jan-13 | XXXXXXXX |
| | 600 | 563 | 16-Jan-13 | XXXXXXXX |
| | 600 | 1320 | 22-Jan-13 | XXXXXXXX |
| | 650 | 585 | 15-Jan-13 | XXXXXXXX |
| | 800 | 1263 | 22-Jan-13 | XXXXXXXX |
| | 600 | 1158 | 21-Jan-13 | XXXXXXXX |
| | 750 | 1074 | 21-Jan-13 | XXXXXXXX |
| | 650 | 968 | 17-Jan-13 | XXXXXXXX |
| | 650 | 444 | 14-Jan-13 | XXXXXXXX |
| | 650 | 583 | 17-Jan-13 | XXXXXXXX |
| | 600 | 602 | 16-Jan-13 | XXXXXXXX |
| | 755 | 441 | 08-Jan-13 | XXXXXXXX |
| | 850 | 781 | 22-Jan-13 | XXXXXXXX |
| | 650 | 400 | 08-Jan-13 | XXXXXXXX |
| | 500 | 453 | 08-Jan-13 | XXXXXXXX |
| | 600 | 693 | 21-Jan-13 | XXXXXXXX |
| | 550 | 405 | 08-Jan-13 | XXXXXXXX |
| | 350 | 1173 | 21-Jan-13 | XXXXXXXX |
| | 600 | 360 | 08-Jan-13 | XXXXXXXX |
| | 630 | 591 | 17-Jan-13 | XXXXXXXX |
| | 650 | 456 | 09-Jan-13 | XXXXXXXX |

| | | | | |
|---|---|---|---|---|
| | 650 | 569 | 14-Jan-13 | XXXXXXXX |
| | 750 | 1312 | 22-Jan-13 | XXXXXXXX |
| | 600 | 653 | 15-Jan-13 | XXXXXXXX |
| | 600 | 556 | 14-Jan-13 | XXXXXXXX |
| | 600 | 1064 | 21-Jan-13 | XXXXXXXX |
| | 800 | 673 | 21-Jan-13 | XXXXXXXX |
| | 600 | 511 | 15-Jan-13 | XXXXXXXX |
| | 580 | 760 | 21-Jan-13 | XXXXXXXX |
| | 600 | 1292 | 22-Jan-13 | XXXXXXXX |
| 1 | 500 | 491 | 09-Jan-13 | XXXXXXXX |
| 1 | 450 | 1321 | 22-Jan-13 | XXXXXXXX |
| 1 | 550 | 766 | 16-Jan-13 | XXXXXXXX |
| 1 | 550 | 595 | 15-Jan-13 | XXXXXXXX |
| 12 | 500 | 639 | 18-Jan-13 | XXXXXXXX |
| 12 | 500 | 545 | 14-Jan-13 | XXXXXXXX |
| 12 | | 756 | 16-Jan-13 | XXXXXXXX |
| 12 | 550 | 519 | 11-Jan-13 | XXXXXXXX |
| 12 | 550 | 466 | 09-Jan-13 | XXXXXXXX |
| 6 | 700 | 603 | 15-Jan-13 | XXXXXXXX |
| 12 | 450 | 372 | 07-Jan-13 | XXXXXXXX |
| 12 | 500 | 602 | 15-Jan-13 | XXXXXXXX |
| 6 | 450 | 1291 | 22-Jan-13 | XXXXXXXX |
| 6 | 450 | 391 | 09-Jan-13 | XXXXXXXX |
| 6 | 400 | 685 | 21-Jan-13 | XXXXXXXX |
| 1 | 480 | 1123 | 21-Jan-13 | XXXXXXXX |
| 1 | 500 | 757 | 21-Jan-13 | XXXXXXXX |
| 1 | 500 | 1157 | 21-Jan-13 | XXXXXXXX |
| 1 | 850 | 439 | 08-Jan-13 | XXXXXXXX |
| 1 | 600 | 382 | 08-Jan-13 | XXXXXXXX |
| 1 | 550 | 780 | 22-Jan-13 | XXXXXXXX |
| 1 | 650 | 411 | 08-Jan-13 | XXXXXXXX |
| 1 | 450 | 404 | 08-Jan-13 | XXXXXXXX |
| 1 | 450 | 364 | 08-Jan-13 | XXXXXXXX |
| 1 | 500 | 851 | 17-Jan-13 | XXXXXXXX |
| 1 | 450 | 507 | 10-Jan-13 | XXXXXXXX |
| 1 | 650 | 797 | 22-Jan-13 | XXXXXXXX |
| 1 | 480 | 444 | 08-Jan-13 | XXXXXXXX |
| 1 | 460 | 1063 | 21-Jan-13 | XXXXXXXX |
| 1 | 480 | 670 | 21-Jan-13 | XXXXXXXX |
| 1 | 460 | 762 | 21-Jan-13 | XXXXXXXX |
| 1 | 500 | 778 | 22-Jan-13 | XXXXXXXX |
| 1 | 550 | 449 | 14-Jan-13 | XXXXXXXX |
| 1 | 600 | 1306 | 22-Jan-13 | XXXXXXXX |
| 1 | 650 | 828 | 17-Jan-13 | XXXXXXXX |
| 1 | 650 | 720 | 21-Jan-13 | XXXXXXXX |
| 1 | 550 | 345 | 07-Jan-13 | XXXXXXXX |
| 1 | 500 | 1232 | 21-Jan-13 | XXXXXXXX |
| 1 | 500 | 891 | 17-Jan-13 | XXXXXXXX |
| 1 | 500 | 1177 | 21-Jan-13 | XXXXXXXX |
| 1 | 650 | 806 | 22-Jan-13 | XXXXXXXX |

| | | | | |
|---|---|---|---|---|
| 1 | 500 | 1156 | 21-Jan-13 | XXXXXXXX |
| 1 | 650 | 1077 | 21-Jan-13 | XXXXXXXX |
| 1 | 500 | 554 | 14-Jan-13 | XXXXXXXX |
| 1 | 500 | 574 | 14-Jan-13 | XXXXXXXX |
| 1 | 450 | 780 | 16-Jan-13 | XXXXXXXX |
| 1 | 550 | 584 | 17-Jan-13 | XXXXXXXX |
| 1 | 650 | 598 | 17-Jan-13 | XXXXXXXX |
| 1 | 550 | 608 | 16-Jan-13 | XXXXXXXX |
| 1 | 650 | 440 | 08-Jan-13 | XXXXXXXX |
| 1 | 650 | 383 | 08-Jan-13 | XXXXXXXX |
| 1 | 500 | 695 | 21-Jan-13 | XXXXXXXX |
| 1 | 650 | 410 | 08-Jan-13 | XXXXXXXX |
| 1 | 500 | 398 | 08-Jan-13 | XXXXXXXX |
| 1 | 450 | 1114 | 21-Jan-13 | XXXXXXXX |
| 1 | 500 | 392 | 09-Jan-13 | XXXXXXXX |
| 1 | 480 | 358 | 08-Jan-13 | XXXXXXXX |
| 1 | 500 | 849 | 17-Jan-13 | XXXXXXXX |
| 1 | 550 | 458 | 09-Jan-13 | XXXXXXXX |
| 1 | 600 | 575 | 14-Jan-13 | XXXXXXXX |
| 1 | 550 | 508 | 10-Jan-13 | XXXXXXXX |
| 1 | 500 | 763 | 16-Jan-13 | XXXXXXXX |
| 1 | 600 | 1313 | 22-Jan-13 | XXXXXXXX |
| 1 | 500 | 655 | 15-Jan-13 | XXXXXXXX |
| 1 | 480 | 700 | 16-Jan-13 | XXXXXXXX |
| 1 | 500 | 1066 | 21-Jan-13 | XXXXXXXX |
| 1 | 500 | 573 | 16-Jan-13 | XXXXXXXX |
| 1 | 500 | 666 | 21-Jan-13 | XXXXXXXX |
| 1 | 500 | 542 | 11-Jan-13 | XXXXXXXX |
| 1 | 460 | 761 | 21-Jan-13 | XXXXXXXX |
| 1 | 500 | 1297 | 22-Jan-13 | XXXXXXXX |
| 1 | | | | XXXXXXXX |
| 1 | 500 | 429 | 08-Jan-13 | XXXXXXXX |
| 1 | 650 | 805 | 22-Jan-13 | XXXXXXXX |
| 1 | 650 | 958 | 17-Jan-13 | XXXXXXXX |
| 1 | 500 | 1305 | 22-Jan-13 | XXXXXXXX |
| 1 | 500 | 553 | 14-Jan-13 | XXXXXXXX |
| 24 | 600 | 1021 | 09-Apr-13 | XXXXXXXX |
| 6 | 150 | 1085 | 10-Apr-13 | XXXXXXXX |
| 6 | 100 | 1041 | 10-Apr-13 | XXXXXXXX |
| 6 | 250 | 1049 | 09-Apr-13 | XXXXXXXX |
| 6 | 250 | 909 | 09-Apr-13 | XXXXXXXX |
| 6 | 140 | 988 | 09-Apr-13 | XXXXXXXX |
| 6 | 150 | 825 | 05-Apr-13 | XXXXXXXX |
| 6 | 150 | 1242 | 10-Apr-13 | XXXXXXXX |
| 6 | 100 | 1286 | 10-Apr-13 | XXXXXXXX |
| 6 | 150 | 962 | 08-Apr-13 | XXXXXXXX |
| 6 | 200 | 923 | 09-Apr-13 | XXXXXXXX |
| 6 | 200 | 743 | 14-Mar-13 | XXXXXXXX |
| 6 | 250 | 879 | 08-Apr-13 | XXXXXXXX |
| 6 | 180 | 993 | 09-Apr-13 | XXXXXXXX |

| | | | | |
|---|---|---|---|---|
| 6 | 150 | 950 | 09-Apr-13 | XXXXXXXX |
| 6 | 80 | 708 | 14-Mar-13 | XXXXXXXX |
| 12 | 200 | 1345 | 11-Apr-13 | XXXXXXXX |
| 12 | 250 | 929 | 08-Apr-13 | XXXXXXXX |
| 12 | 300 | 1303 | 11-Apr-13 | XXXXXXXX |
| 12 | 300 | 880 | 08-Apr-13 | XXXXXXXX |
| 18 | 300 | 1024 | 10-Apr-13 | XXXXXXXX |
| 18 | 150 | 982 | 09-Apr-13 | XXXXXXXX |
| 18 | 400 | 1348 | 11-Apr-13 | XXXXXXXX |
| 18 | 650 | 1389 | 11-Apr-13 | XXXXXXXX |
| 18 | 400 | 1300 | 11-Apr-13 | XXXXXXXX |
| 18 | 250 | 1278 | 10-Apr-13 | XXXXXXXX |
| 18 | 300 | 882 | 08-Apr-13 | XXXXXXXX |
| 18 | 250 | 845 | 08-Apr-13 | XXXXXXXX |
| 18 | 350 | 767 | 05-Apr-13 | XXXXXXXX |
| 18 | 250 | 1321 | 11-Apr-13 | XXXXXXXX |
| 24 | 350 | 809 | 05-Apr-13 | XXXXXXXX |
| 24 | 350 | 1082 | 09-Apr-13 | XXXXXXXX |
| 24 | 300 | 980 | 09-Apr-13 | XXXXXXXX |
| 24 | 300 | 888 | 08-Apr-13 | XXXXXXXX |
| 24 | 400 | 725 | 14-Mar-13 | XXXXXXXX |
| 24 | 300 | 863 | 08-Apr-13 | XXXXXXXX |
| 24 | 350 | 990 | 09-Apr-13 | XXXXXXXX |
| 24 | 450 | 959 | 08-Apr-13 | XXXXXXXX |
| 24 | 350 | 924 | 09-Apr-13 | XXXXXXXX |
| 24 | 300 | 966 | 09-Apr-13 | XXXXXXXX |
| 24 | 300 | 1183 | 10-Apr-13 | XXXXXXXX |
| 24 | 250 | 1177 | 10-Apr-13 | XXXXXXXX |
| 24 | 350 | 883 | 08-Apr-13 | XXXXXXXX |
| 24 | 400 | 1114 | 09-Apr-13 | XXXXXXXX |
| 24 | 400 | 849 | 08-Apr-13 | XXXXXXXX |
| 24 | 300 | 1158 | 10-Apr-13 | XXXXXXXX |
| 24 | 300 | 846 | 08-Apr-13 | XXXXXXXX |
| 24 | 300 | 916 | 08-Apr-13 | XXXXXXXX |
| 24 | 300 | 951 | 09-Apr-13 | XXXXXXXX |
| 24 | 400 | 768 | 05-Apr-13 | XXXXXXXX |
| 24 | 300 | 930 | 09-Apr-13 | XXXXXXXX |
| 24 | 570 | 1089 | 10-Apr-13 | XXXXXXXX |
| 24 | 1000 | 752 | 14-Mar-13 | XXXXXXXX |
| 24 | 650 | 977 | 09-Apr-13 | XXXXXXXX |
| 24 | 600 | 1065 | 09-Apr-13 | XXXXXXXX |
| 24 | 600 | 837 | 05-Apr-13 | XXXXXXXX |
| 24 | 600 | 942 | 08-Apr-13 | XXXXXXXX |
| 24 | 700 | 1255 | 10-Apr-13 | XXXXXXXX |
| 24 | 500 | 913 | 08-Apr-13 | XXXXXXXX |
| 24 | 550 | 831 | 05-Apr-13 | XXXXXXXX |
| 24 | 550 | 819 | 05-Apr-13 | XXXXXXXX |
| 24 | 700 | 1333 | 11-Apr-13 | XXXXXXXX |
| 24 | 650 | 1230 | 10-Apr-13 | XXXXXXXX |
| 24 | 650 | 1187 | 10-Apr-13 | XXXXXXXX |

| | | | | |
|---|---|---|---|---|
| 24 | 600 | 799 | 05-Apr-13 | XXXXXXXX |
| 12 | 400 | 722 | 14-Mar-13 | XXXXXXXX |
| 12 | 700 | 1252 | 10-Apr-13 | XXXXXXXX |
| 24 | 550 | 1037 | 10-Apr-13 | XXXXXXXX |
| 24 | 250 | 1061 | 09-Apr-13 | XXXXXXXX |
| 24 | 350 | 889 | 08-Apr-13 | XXXXXXXX |
| 24 | 350 | 919 | 08-Apr-13 | XXXXXXXX |
| 24 | 500 | 938 | 09-Apr-13 | XXXXXXXX |
| 24 | 500 | 1037 | 09-Apr-13 | XXXXXXXX |
| 24 | 500 | 1124 | 09-Apr-13 | XXXXXXXX |
| 24 | 1000 | 1111 | 10-Apr-13 | XXXXXXXX |
| 24 | 1000 | 1094 | 10-Apr-13 | XXXXXXXX |
| 24 | 1030 | 1146 | 09-Apr-13 | XXXXXXXX |
| 24 | 950 | 1033 | 10-Apr-13 | XXXXXXXX |
| 24 | 1100 | 906 | 08-Apr-13 | XXXXXXXX |
| 24 | 1000 | 1336 | 11-Apr-13 | XXXXXXXX |
| 24 | 960 | 1072 | 10-Apr-13 | XXXXXXXX |
| 24 | 550 | 982 | 09-Apr-13 | XXXXXXXX |
| 24 | 1000 | 1055 | 09-Apr-13 | XXXXXXXX |
| 24 | 1000 | 1074 | 09-Apr-13 | XXXXXXXX |
| 24 | 1000 | 1083 | 09-Apr-13 | XXXXXXXX |
| 24 | 980 | 1100 | 09-Apr-13 | XXXXXXXX |
| 24 | 1000 | 1125 | 09-Apr-13 | XXXXXXXX |
| 24 | 1000 | 1377 | 11-Apr-13 | XXXXXXXX |
| 24 | 950 | 869 | 08-Apr-13 | XXXXXXXX |
| 24 | 1000 | 952 | 08-Apr-13 | XXXXXXXX |
| 24 | 1100 | 892 | 08-Apr-13 | XXXXXXXX |
| 24 | 1000 | 1169 | 10-Apr-13 | XXXXXXXX |
| 24 | 1000 | 1015 | 09-Apr-13 | XXXXXXXX |
| 24 | 1100 | 973 | 08-Apr-13 | XXXXXXXX |
| 24 | 1000 | 790 | 05-Apr-13 | XXXXXXXX |
| 24 | 950 | 1316 | 11-Apr-13 | XXXXXXXX |
| 24 | 1000 | 1386 | 11-Apr-13 | XXXXXXXX |
| 24 | 950 | 1216 | 10-Apr-13 | XXXXXXXX |
| 24 | 1000 | 912 | 08-Apr-13 | XXXXXXXX |
| 24 | 1000 | 1176 | 10-Apr-13 | XXXXXXXX |
| 24 | 900 | 872 | 08-Apr-13 | XXXXXXXX |
| 24 | 950 | 833 | 05-Apr-13 | XXXXXXXX |
| 24 | 950 | 1070 | 09-Apr-13 | XXXXXXXX |
| 24 | 900 | 818 | 05-Apr-13 | XXXXXXXX |
| 24 | 1000 | 761 | 14-Mar-13 | XXXXXXXX |
| 24 | 1000 | 933 | 09-Apr-13 | XXXXXXXX |
| 24 | 950 | 1359 | 11-Apr-13 | XXXXXXXX |
| 18 | 300 | 1202 | 10-Apr-13 | XXXXXXXX |
| 18 | 450 | 975 | 09-Apr-13 | XXXXXXXX |
| 18 | 190 | 991 | 09-Apr-13 | XXXXXXXX |
| 18 | 250 | 953 | 09-Apr-13 | XXXXXXXX |
| 18 | 300 | 1019 | 09-Apr-13 | XXXXXXXX |
| 24 | 400 | 714 | 14-Mar-13 | XXXXXXXX |
| 24 | 300 | 1100 | 10-Apr-13 | XXXXXXXX |

| | | | | |
|---|---|---|---|---|
| 24 | 450 | 812 | 05-Apr-13 | XXXXXXXX |
| 24 | 300 | 1051 | 10-Apr-13 | XXXXXXXX |
| 1 | 1150 | 920 | 09-Apr-13 | XXXXXXXX |
| 24 | 600 | 908 | 08-Apr-13 | XXXXXXXX |
| 24 | 400 | 979 | 09-Apr-13 | XXXXXXXX |
| 24 | 500 | 986 | 09-Apr-13 | XXXXXXXX |
| 24 | 650 | 1034 | 09-Apr-13 | XXXXXXXX |
| 24 | 350 | 892 | 08-Apr-13 | XXXXXXXX |
| 24 | 450 | 1003 | 09-Apr-13 | XXXXXXXX |
| 24 | 250 | 998 | 09-Apr-13 | XXXXXXXX |
| 24 | 350 | 742 | 14-Mar-13 | XXXXXXXX |
| 24 | 650 | 1391 | 11-Apr-13 | XXXXXXXX |
| 24 | 700 | 829 | 05-Apr-13 | XXXXXXXX |
| 24 | 800 | 891 | 08-Apr-13 | XXXXXXXX |
| 24 | 350 | 1279 | 10-Apr-13 | XXXXXXXX |
| 24 | 900 | 1258 | 10-Apr-13 | XXXXXXXX |
| 24 | 400 | 1191 | 10-Apr-13 | XXXXXXXX |
| 24 | 300 | 730 | 14-Mar-13 | XXXXXXXX |
| 24 | 450 | 1119 | 10-Apr-13 | XXXXXXXX |
| 24 | 450 | 940 | 08-Apr-13 | XXXXXXXX |
| 24 | 500 | 1004 | 10-Apr-13 | XXXXXXXX |
| 24 | 350 | 1110 | 09-Apr-13 | XXXXXXXX |
| 24 | 400 | 774 | 05-Apr-13 | XXXXXXXX |
| 24 | 450 | 958 | 08-Apr-13 | XXXXXXXX |
| 24 | 300 | 727 | 14-Mar-13 | XXXXXXXX |
| 6 | 150 | 1101 | 10-Apr-13 | XXXXXXXX |
| 6 | 250 | 1340 | 11-Apr-13 | XXXXXXXX |
| 6 | 300 | 985 | 09-Apr-13 | XXXXXXXX |
| 6 | 200 | 985 | 09-Apr-13 | XXXXXXXX |
| 6 | 450 | 1032 | 09-Apr-13 | XXXXXXXX |
| 6 | 300 | 1092 | 09-Apr-13 | XXXXXXXX |
| 6 | 150 | 1062 | 10-Apr-13 | XXXXXXXX |
| 6 | 550 | 1392 | 11-Apr-13 | XXXXXXXX |
| 6 | 300 | 935 | 08-Apr-13 | XXXXXXXX |
| 6 | 250 | 1297 | 11-Apr-13 | XXXXXXXX |
| 12 | 280 | 1047 | 09-Apr-13 | XXXXXXXX |
| 12 | 200 | 1062 | 09-Apr-13 | XXXXXXXX |
| 12 | 300 | 830 | 05-Apr-13 | XXXXXXXX |
| 12 | 250 | 1238 | 10-Apr-13 | XXXXXXXX |
| 12 | 200 | 967 | 09-Apr-13 | XXXXXXXX |
| 12 | 200 | 775 | 05-Apr-13 | XXXXXXXX |
| 24 | 700 | 981 | 09-Apr-13 | XXXXXXXX |
| 24 | 700 | 1054 | 09-Apr-13 | XXXXXXXX |
| 24 | 650 | 951 | 08-Apr-13 | XXXXXXXX |
| 24 | 650 | 896 | 08-Apr-13 | XXXXXXXX |
| 24 | 650 | 1190 | 10-Apr-13 | XXXXXXXX |
| 24 | 640 | 1213 | 10-Apr-13 | XXXXXXXX |
| 6 | 300 | 901 | 08-Apr-13 | XXXXXXXX |
| 12 | 300 | 1093 | 09-Apr-13 | XXXXXXXX |
| 12 | 300 | 937 | 08-Apr-13 | XXXXXXXX |

| | | | | |
|---|---|---|---|---|
| 24 | 250 | 1058 | 09-Apr-13 | XXXXXXXX |
| 24 | 300 | 862 | 08-Apr-13 | XXXXXXXX |
| 24 | 350 | 1236 | 10-Apr-13 | XXXXXXXX |
| 6 | 300 | 965 | 09-Apr-13 | XXXXXXXX |
| 6 | 350 | 846 | 08-Apr-13 | XXXXXXXX |
| 6 | 150 | 1224 | 10-Apr-13 | XXXXXXXX |
| 6 | 700 | 797 | 05-Apr-13 | XXXXXXXX |
| 12 | 200 | 947 | 09-Apr-13 | XXXXXXXX |
| 12 | 200 | 1089 | 09-Apr-13 | XXXXXXXX |
| 12 | 150 | 1059 | 09-Apr-13 | XXXXXXXX |
| 12 | 300 | 964 | 09-Apr-13 | XXXXXXXX |
| 12 | 300 | 904 | 08-Apr-13 | XXXXXXXX |
| 12 | 400 | 845 | 08-Apr-13 | XXXXXXXX |
| 12 | 200 | 766 | 05-Apr-13 | XXXXXXXX |
| 12 | 200 | 1087 | 10-Apr-13 | XXXXXXXX |
| 12 | 250 | 1026 | 10-Apr-13 | XXXXXXXX |
| 12 | 100 | 983 | 09-Apr-13 | XXXXXXXX |
| 12 | 100 | 1035 | 09-Apr-13 | XXXXXXXX |
| 12 | 300 | 1022 | 09-Apr-13 | XXXXXXXX |
| 12 | 300 | 1004 | 09-Apr-13 | XXXXXXXX |
| 12 | 150 | 1057 | 10-Apr-13 | XXXXXXXX |
| 12 | 250 | 836 | 05-Apr-13 | XXXXXXXX |
| 12 | 160 | 989 | 09-Apr-13 | XXXXXXXX |
| 12 | 300 | 1288 | 10-Apr-13 | XXXXXXXX |
| 12 | 250 | 764 | 05-Apr-13 | XXXXXXXX |
| 18 | 150 | 709 | 14-Mar-13 | XXXXXXXX |
| 24 | 300 | 721 | 14-Mar-13 | XXXXXXXX |
| 24 | 500 | 1032 | 10-Apr-13 | XXXXXXXX |
| 24 | 400 | 1344 | 11-Apr-13 | XXXXXXXX |
| 24 | 300 | 1029 | 09-Apr-13 | XXXXXXXX |
| 24 | 300 | 978 | 09-Apr-13 | XXXXXXXX |
| 24 | 350 | 1023 | 09-Apr-13 | XXXXXXXX |
| 24 | 250 | 1375 | 11-Apr-13 | XXXXXXXX |
| 24 | 200 | 824 | 05-Apr-13 | XXXXXXXX |
| 24 | 300 | 826 | 05-Apr-13 | XXXXXXXX |
| 24 | 850 | 931 | 08-Apr-13 | XXXXXXXX |
| 24 | 450 | 1347 | 11-Apr-13 | XXXXXXXX |
| 24 | 600 | 1118 | 10-Apr-13 | XXXXXXXX |
| 24 | 350 | 941 | 08-Apr-13 | XXXXXXXX |
| 24 | 350 | 1131 | 09-Apr-13 | XXXXXXXX |
| 24 | 750 | 854 | 08-Apr-13 | XXXXXXXX |
| 24 | 250 | 918 | 08-Apr-13 | XXXXXXXX |
| 24 | 400 | 765 | 05-Apr-13 | XXXXXXXX |
| 24 | 350 | 1309 | 11-Apr-13 | XXXXXXXX |
| 24 | 300 | 1086 | 10-Apr-13 | XXXXXXXX |
| 24 | 300 | 1208 | 10-Apr-13 | XXXXXXXX |
| 24 | 450 | 811 | 05-Apr-13 | XXXXXXXX |
| 24 | 500 | 877 | 08-Apr-13 | XXXXXXXX |
| 24 | 300 | 946 | 09-Apr-13 | XXXXXXXX |
| 24 | 300 | 979 | 09-Apr-13 | XXXXXXXX |

| | | | | |
|---|---|---|---|---|
| 24 | 300 | 910 | 09-Apr-13 | XXXXXXXX |
| 24 | 350 | 937 | 09-Apr-13 | XXXXXXXX |
| 24 | 120 | 984 | 09-Apr-13 | XXXXXXXX |
| 24 | 200 | 1038 | 09-Apr-13 | XXXXXXXX |
| 24 | 350 | 1101 | 09-Apr-13 | XXXXXXXX |
| 24 | 250 | 1245 | 10-Apr-13 | XXXXXXXX |
| 24 | 200 | 783 | 05-Apr-13 | XXXXXXXX |
| 24 | 250 | 777 | 05-Apr-13 | XXXXXXXX |
| 24 | 150 | 741 | 14-Mar-13 | XXXXXXXX |
| 24 | 350 | 954 | 09-Apr-13 | XXXXXXXX |
| 24 | 240 | 990 | 09-Apr-13 | XXXXXXXX |
| 24 | 300 | 861 | 08-Apr-13 | XXXXXXXX |
| 24 | 300 | 988 | 09-Apr-13 | XXXXXXXX |
| 24 | 250 | 887 | 08-Apr-13 | XXXXXXXX |
| 24 | 350 | 1281 | 10-Apr-13 | XXXXXXXX |
| 24 | 600 | 1251 | 10-Apr-13 | XXXXXXXX |
| 24 | 400 | 731 | 14-Mar-13 | XXXXXXXX |
| 24 | 300 | 1153 | 09-Apr-13 | XXXXXXXX |
| 24 | 400 | 1111 | 09-Apr-13 | XXXXXXXX |
| 24 | 300 | 832 | 05-Apr-13 | XXXXXXXX |
| 24 | 300 | 1016 | 09-Apr-13 | XXXXXXXX |
| 24 | 200 | 1353 | 11-Apr-13 | XXXXXXXX |
| 24 | 300 | 1366 | 11-Apr-13 | XXXXXXXX |
| 24 | 300 | 753 | 14-Mar-13 | XXXXXXXX |
| 24 | 800 | 1259 | 10-Apr-13 | XXXXXXXX |
| 24 | 400 | 957 | 08-Apr-13 | XXXXXXXX |
| 24 | 750 | 907 | 08-Apr-13 | XXXXXXXX |
| 24 | 670 | 1143 | 09-Apr-13 | XXXXXXXX |
| 24 | 580 | 1046 | 09-Apr-13 | XXXXXXXX |
| 24 | 630 | 1382 | 11-Apr-13 | XXXXXXXX |
| 24 | 650 | 1233 | 10-Apr-13 | XXXXXXXX |
| 24 | 650 | 970 | 09-Apr-13 | XXXXXXXX |
| 24 | 600 | 858 | 08-Apr-13 | XXXXXXXX |
| 24 | 500 | 1199 | 10-Apr-13 | XXXXXXXX |
| 12 | 720 | 905 | 08-Apr-13 | XXXXXXXX |
| 12 | 600 | 1263 | 10-Apr-13 | XXXXXXXX |
| 12 | 600 | 864 | 08-Apr-13 | XXXXXXXX |
| 12 | 500 | 1088 | 10-Apr-13 | XXXXXXXX |
| 12 | 500 | 1050 | 10-Apr-13 | XXXXXXXX |
| 12 | 1000 | 751 | 14-Mar-13 | XXXXXXXX |
| 12 | 520 | 902 | 08-Apr-13 | XXXXXXXX |
| 12 | 600 | 1068 | 10-Apr-13 | XXXXXXXX |
| 12 | 550 | 1016 | 10-Apr-13 | XXXXXXXX |
| 12 | 850 | 933 | 08-Apr-13 | XXXXXXXX |
| 12 | 500 | 859 | 08-Apr-13 | XXXXXXXX |
| 12 | 600 | 1166 | 10-Apr-13 | XXXXXXXX |
| 12 | 500 | 1324 | 11-Apr-13 | XXXXXXXX |
| 12 | 400 | 897 | 08-Apr-13 | XXXXXXXX |
| 12 | 450 | 1264 | 10-Apr-13 | XXXXXXXX |
| 12 | 500 | 711 | 14-Mar-13 | XXXXXXXX |

| | | | | |
|---|---|---|---|---|
| 12 | 550 | 822 | 05-Apr-13 | XXXXXXXX |
| 12 | 600 | 926 | 08-Apr-13 | XXXXXXXX |
| 12 | 500 | 925 | 09-Apr-13 | XXXXXXXX |
| 12 | 650 | 754 | 14-Mar-13 | XXXXXXXX |
| 12 | 600 | 867 | 08-Apr-13 | XXXXXXXX |
| 12 | 650 | 1065 | 10-Apr-13 | XXXXXXXX |
| 12 | 500 | 1327 | 11-Apr-13 | XXXXXXXX |
| 12 | 650 | 1229 | 10-Apr-13 | XXXXXXXX |
| 12 | 600 | 1231 | 10-Apr-13 | XXXXXXXX |
| 12 | 600 | 973 | 09-Apr-13 | XXXXXXXX |
| 6 | 500 | 1203 | 10-Apr-13 | XXXXXXXX |
| 6 | 480 | 1048 | 10-Apr-13 | XXXXXXXX |
| 6 | 650 | 903 | 08-Apr-13 | XXXXXXXX |
| 6 | 650 | 1064 | 10-Apr-13 | XXXXXXXX |
| 6 | 550 | 1018 | 10-Apr-13 | XXXXXXXX |
| 6 | 750 | 980 | 09-Apr-13 | XXXXXXXX |
| 6 | 650 | 886 | 08-Apr-13 | XXXXXXXX |
| 6 | 900 | 785 | 05-Apr-13 | XXXXXXXX |
| 6 | 600 | 864 | 08-Apr-13 | XXXXXXXX |
| 6 | 700 | 888 | 08-Apr-13 | XXXXXXXX |
| 6 | 600 | 1168 | 10-Apr-13 | XXXXXXXX |
| 6 | 600 | 792 | 05-Apr-13 | XXXXXXXX |
| 6 | 600 | 1387 | 11-Apr-13 | XXXXXXXX |
| 6 | 550 | 878 | 08-Apr-13 | XXXXXXXX |
| 6 | 600 | 1115 | 09-Apr-13 | XXXXXXXX |
| 6 | 650 | 844 | 08-Apr-13 | XXXXXXXX |
| 6 | 600 | 1071 | 09-Apr-13 | XXXXXXXX |
| 6 | 550 | 710 | 14-Mar-13 | XXXXXXXX |
| 6 | 600 | 926 | 09-Apr-13 | XXXXXXXX |
| 6 | 650 | 1023 | 10-Apr-13 | XXXXXXXX |
| 6 | 700 | 1332 | 11-Apr-13 | XXXXXXXX |
| 6 | 600 | 1014 | 10-Apr-13 | XXXXXXXX |
| 6 | 680 | 1044 | 09-Apr-13 | XXXXXXXX |
| 6 | 550 | 1379 | 11-Apr-13 | XXXXXXXX |
| 6 | 650 | 733 | 14-Mar-13 | XXXXXXXX |
| 6 | 700 | 1313 | 11-Apr-13 | XXXXXXXX |
| 6 | 650 | 1097 | 09-Apr-13 | XXXXXXXX |
| 6 | 630 | 1145 | 09-Apr-13 | XXXXXXXX |
| 6 | 750 | 1039 | 10-Apr-13 | XXXXXXXX |
| 6 | 1000 | 749 | 14-Mar-13 | XXXXXXXX |
| 6 | 600 | 1385 | 11-Apr-13 | XXXXXXXX |
| 6 | 630 | 1221 | 10-Apr-13 | XXXXXXXX |
| 6 | 800 | 927 | 09-Apr-13 | XXXXXXXX |
| 6 | 650 | 1063 | 10-Apr-13 | XXXXXXXX |
| 6 | 700 | 1107 | 09-Apr-13 | XXXXXXXX |
| 6 | 630 | 1121 | 09-Apr-13 | XXXXXXXX |
| 6 | 600 | 870 | 08-Apr-13 | XXXXXXXX |
| 6 | 650 | 1028 | 09-Apr-13 | XXXXXXXX |
| 6 | 300 | 1271 | 10-Apr-13 | XXXXXXXX |
| 6 | 500 | 1059 | 10-Apr-13 | XXXXXXXX |

| | | | | |
|---|---|---|---|---|
| 6 | 700 | 1235 | 10-Apr-13 | XXXXXXXX |
| 6 | 550 | 821 | 05-Apr-13 | XXXXXXXX |
| 6 | 550 | 1322 | 11-Apr-13 | XXXXXXXX |
| 6 | 500 | 1265 | 10-Apr-13 | XXXXXXXX |
| 6 | 600 | 755 | 14-Mar-13 | XXXXXXXX |
| 6 | 600 | 1036 | 09-Apr-13 | XXXXXXXX |
| 6 | 850 | 932 | 08-Apr-13 | XXXXXXXX |
| 6 | 650 | 1289 | 10-Apr-13 | XXXXXXXX |
| 6 | 600 | 1250 | 10-Apr-13 | XXXXXXXX |
| 6 | 500 | 871 | 08-Apr-13 | XXXXXXXX |
| 6 | 500 | 1180 | 10-Apr-13 | XXXXXXXX |
| 6 | 550 | 847 | 08-Apr-13 | XXXXXXXX |
| 6 | 540 | 1148 | 09-Apr-13 | XXXXXXXX |
| 6 | 600 | 1013 | 10-Apr-13 | XXXXXXXX |
| 6 | 700 | 1077 | 09-Apr-13 | XXXXXXXX |
| 6 | 680 | 995 | 09-Apr-13 | XXXXXXXX |
| 6 | 700 | 1103 | 09-Apr-13 | XXXXXXXX |
| 6 | 550 | 1060 | 10-Apr-13 | XXXXXXXX |
| 6 | 650 | 858 | 08-Apr-13 | XXXXXXXX |
| 6 | 650 | 956 | 09-Apr-13 | XXXXXXXX |
| 6 | 450 | 866 | 08-Apr-13 | XXXXXXXX |
| 6 | 650 | 969 | 08-Apr-13 | XXXXXXXX |
| 6 | 800 | 798 | 05-Apr-13 | XXXXXXXX |
| 6 | 550 | 1055 | 10-Apr-13 | XXXXXXXX |
| 6 | 600 | 1127 | 09-Apr-13 | XXXXXXXX |
| 6 | 550 | 1378 | 11-Apr-13 | XXXXXXXX |
| 6 | 600 | 867 | 08-Apr-13 | XXXXXXXX |
| 6 | 650 | 1283 | 10-Apr-13 | XXXXXXXX |
| 6 | 650 | 1012 | 10-Apr-13 | XXXXXXXX |
| 6 | 650 | 911 | 09-Apr-13 | XXXXXXXX |
| 6 | 600 | 1086 | 09-Apr-13 | XXXXXXXX |
| 6 | 700 | 997 | 09-Apr-13 | XXXXXXXX |
| 6 | 600 | 779 | 05-Apr-13 | XXXXXXXX |
| 6 | 750 | 964 | 08-Apr-13 | XXXXXXXX |
| 6 | 600 | 1212 | 10-Apr-13 | XXXXXXXX |
| 6 | 650 | 862 | 08-Apr-13 | XXXXXXXX |
| 6 | 600 | 902 | 08-Apr-13 | XXXXXXXX |
| 6 | 550 | 873 | 08-Apr-13 | XXXXXXXX |
| 6 | 500 | 848 | 08-Apr-13 | XXXXXXXX |
| 6 | 650 | 1181 | 10-Apr-13 | XXXXXXXX |
| 6 | 600 | 1064 | 09-Apr-13 | XXXXXXXX |
| 6 | 600 | 857 | 08-Apr-13 | XXXXXXXX |
| 6 | 700 | 996 | 09-Apr-13 | XXXXXXXX |
| 6 | 650 | 1033 | 09-Apr-13 | XXXXXXXX |
| 6 | 650 | 1020 | 09-Apr-13 | XXXXXXXX |
| 6 | 600 | 884 | 08-Apr-13 | XXXXXXXX |
| 6 | 600 | 963 | 08-Apr-13 | XXXXXXXX |
| 6 | 600 | 974 | 08-Apr-13 | XXXXXXXX |
| 6 | 650 | 900 | 08-Apr-13 | XXXXXXXX |
| 6 | 500 | 1182 | 10-Apr-13 | XXXXXXXX |

| | | | | |
|---|---|---|---|---|
| 6 | 750 | 769 | 05-Apr-13 | XXXXXXXX |
| 6 | 580 | 719 | 14-Mar-13 | XXXXXXXX |
| 6 | 650 | 1107 | 10-Apr-13 | XXXXXXXX |
| 1 | 500 | 1135 | 09-Apr-13 | XXXXXXXX |
| 6 | 550 | 1017 | 10-Apr-13 | XXXXXXXX |
| 6 | 550 | 1116 | 09-Apr-13 | XXXXXXXX |
| 6 | 600 | 885 | 08-Apr-13 | XXXXXXXX |
| 6 | 600 | 1005 | 09-Apr-13 | XXXXXXXX |
| 6 | 650 | 839 | 05-Apr-13 | XXXXXXXX |
| 6 | 500 | 860 | 08-Apr-13 | XXXXXXXX |
| 6 | 650 | 1287 | 10-Apr-13 | XXXXXXXX |
| 6 | 600 | 944 | 08-Apr-13 | XXXXXXXX |
| 6 | 570 | 1215 | 10-Apr-13 | XXXXXXXX |
| 6 | 600 | 771 | 05-Apr-13 | XXXXXXXX |
| 6 | 550 | 823 | 05-Apr-13 | XXXXXXXX |
| 6 | 570 | 925 | 08-Apr-13 | XXXXXXXX |
| 6 | 700 | 718 | 14-Mar-13 | XXXXXXXX |
| 6 | 800 | 1093 | 10-Apr-13 | XXXXXXXX |
| 6 | 700 | 1207 | 10-Apr-13 | XXXXXXXX |
| 6 | 800 | 804 | 05-Apr-13 | XXXXXXXX |
| 6 | 750 | 1028 | 10-Apr-13 | XXXXXXXX |
| 6 | 750 | 1047 | 10-Apr-13 | XXXXXXXX |
| 12 | 900 | 748 | 14-Mar-13 | XXXXXXXX |
| 6 | 840 | 893 | 08-Apr-13 | XXXXXXXX |
| 6 | 800 | 1331 | 11-Apr-13 | XXXXXXXX |
| 6 | 750 | 1071 | 10-Apr-13 | XXXXXXXX |
| 6 | 800 | 1105 | 09-Apr-13 | XXXXXXXX |
| 6 | 750 | 1007 | 10-Apr-13 | XXXXXXXX |
| 6 | 750 | 1030 | 09-Apr-13 | XXXXXXXX |
| 6 | 800 | 875 | 08-Apr-13 | XXXXXXXX |
| 6 | 850 | 984 | 09-Apr-13 | XXXXXXXX |
| 6 | 850 | 1053 | 09-Apr-13 | XXXXXXXX |
| 6 | 850 | 1078 | 09-Apr-13 | XXXXXXXX |
| 6 | 800 | 935 | 09-Apr-13 | XXXXXXXX |
| 6 | 750 | 1118 | 09-Apr-13 | XXXXXXXX |
| 6 | 800 | 1102 | 09-Apr-13 | XXXXXXXX |
| 6 | 800 | 890 | 08-Apr-13 | XXXXXXXX |
| 6 | 790 | 1128 | 09-Apr-13 | XXXXXXXX |
| 6 | 700 | 1094 | 09-Apr-13 | XXXXXXXX |
| 6 | 700 | 1246 | 10-Apr-13 | XXXXXXXX |
| 6 | 750 | 1381 | 11-Apr-13 | XXXXXXXX |
| 6 | 700 | 786 | 05-Apr-13 | XXXXXXXX |
| 6 | 750 | 968 | 09-Apr-13 | XXXXXXXX |
| 6 | 700 | 782 | 05-Apr-13 | XXXXXXXX |
| 6 | 750 | 734 | 14-Mar-13 | XXXXXXXX |
| 6 | 750 | 843 | 05-Apr-13 | XXXXXXXX |
| 6 | 700 | 1390 | 11-Apr-13 | XXXXXXXX |
| 6 | 750 | 957 | 09-Apr-13 | XXXXXXXX |
| 6 | 700 | 986 | 09-Apr-13 | XXXXXXXX |
| 6 | 700 | 856 | 08-Apr-13 | XXXXXXXX |

| | | | | |
|---|---|---|---|---|
| 6 | 750 | 1301 | 11-Apr-13 | XXXXXXXX |
| 6 | 750 | 1239 | 10-Apr-13 | XXXXXXXX |
| 6 | 800 | 886 | 08-Apr-13 | XXXXXXXX |
| 6 | 750 | 1163 | 10-Apr-13 | XXXXXXXX |
| 6 | 750 | 882 | 08-Apr-13 | XXXXXXXX |
| 6 | 850 | 968 | 08-Apr-13 | XXXXXXXX |
| 6 | 800 | 1192 | 10-Apr-13 | XXXXXXXX |
| 6 | 850 | 922 | 09-Apr-13 | XXXXXXXX |
| 6 | 750 | 729 | 14-Mar-13 | XXXXXXXX |
| 6 | 750 | 789 | 05-Apr-13 | XXXXXXXX |
| 6 | 900 | 1116 | 10-Apr-13 | XXXXXXXX |
| 6 | 700 | 958 | 09-Apr-13 | XXXXXXXX |
| 6 | 750 | 939 | 08-Apr-13 | XXXXXXXX |
| 6 | 750 | 1315 | 11-Apr-13 | XXXXXXXX |
| 6 | 700 | 1096 | 09-Apr-13 | XXXXXXXX |
| 6 | 750 | 745 | 14-Mar-13 | XXXXXXXX |
| 6 | 850 | 1002 | 10-Apr-13 | XXXXXXXX |
| 6 | 750 | 1388 | 11-Apr-13 | XXXXXXXX |
| 6 | 750 | 1217 | 10-Apr-13 | XXXXXXXX |
| 6 | 800 | 914 | 08-Apr-13 | XXXXXXXX |
| 6 | 750 | 1188 | 10-Apr-13 | XXXXXXXX |
| 6 | 700 | 875 | 08-Apr-13 | XXXXXXXX |
| 6 | 750 | 1112 | 09-Apr-13 | XXXXXXXX |
| 6 | 750 | 848 | 08-Apr-13 | XXXXXXXX |
| 6 | 750 | 851 | 08-Apr-13 | XXXXXXXX |
| 6 | 750 | 850 | 08-Apr-13 | XXXXXXXX |
| 6 | 700 | 942 | 09-Apr-13 | XXXXXXXX |
| 6 | 750 | 835 | 05-Apr-13 | XXXXXXXX |
| 6 | 700 | 1225 | 10-Apr-13 | XXXXXXXX |
| 6 | 750 | 1068 | 09-Apr-13 | XXXXXXXX |
| 6 | 900 | 773 | 05-Apr-13 | XXXXXXXX |
| 6 | 700 | 1017 | 09-Apr-13 | XXXXXXXX |
| 6 | 800 | 1310 | 11-Apr-13 | XXXXXXXX |
| 6 | 750 | 803 | 05-Apr-13 | XXXXXXXX |
| 6 | 700 | 817 | 05-Apr-13 | XXXXXXXX |
| 6 | 800 | 1364 | 11-Apr-13 | XXXXXXXX |
| 6 | 750 | 1228 | 10-Apr-13 | XXXXXXXX |
| 6 | 700 | 1317 | 11-Apr-13 | XXXXXXXX |
| 6 | 750 | 760 | 14-Mar-13 | XXXXXXXX |
| 6 | 750 | 932 | 09-Apr-13 | XXXXXXXX |
| 6 | 750 | 1363 | 11-Apr-13 | XXXXXXXX |
| 6 | 630 | 1134 | 09-Apr-13 | XXXXXXXX |
| 6 | 650 | 1020 | 10-Apr-13 | XXXXXXXX |
| 6 | 600 | 1015 | 10-Apr-13 | XXXXXXXX |
| 6 | 550 | 1323 | 11-Apr-13 | XXXXXXXX |
| 6 | 700 | 1108 | 10-Apr-13 | XXXXXXXX |
| 6 | 650 | 1211 | 10-Apr-13 | XXXXXXXX |
| 6 | 750 | 1049 | 10-Apr-13 | XXXXXXXX |
| 6 | 800 | 1343 | 11-Apr-13 | XXXXXXXX |
| 6 | 750 | 1067 | 10-Apr-13 | XXXXXXXX |

| | | | | |
|---|---|---|---|---|
| 6 | 800 | 876 | 08-Apr-13 | XXXXXXXX |
| 6 | 850 | 976 | 09-Apr-13 | XXXXXXXX |
| 6 | 670 | 1380 | 11-Apr-13 | XXXXXXXX |
| 6 | 700 | 974 | 09-Apr-13 | XXXXXXXX |
| 6 | 800 | 895 | 08-Apr-13 | XXXXXXXX |
| 6 | 650 | 1328 | 11-Apr-13 | XXXXXXXX |
| 6 | 650 | 706 | 14-Mar-13 | XXXXXXXX |
| 6 | 650 | 820 | 05-Apr-13 | XXXXXXXX |
| 6 | 800 | 1368 | 11-Apr-13 | XXXXXXXX |
| 6 | 650 | 756 | 14-Mar-13 | XXXXXXXX |
| 6 | 650 | 1046 | 10-Apr-13 | XXXXXXXX |
| 6 | 600 | 778 | 05-Apr-13 | XXXXXXXX |
| 6 | 600 | 1189 | 10-Apr-13 | XXXXXXXX |
| 6 | 600 | 863 | 08-Apr-13 | XXXXXXXX |
| 6 | 600 | 1312 | 11-Apr-13 | XXXXXXXX |
| 6 | 500 | 928 | 09-Apr-13 | XXXXXXXX |
| 6 | 630 | 1139 | 09-Apr-13 | XXXXXXXX |
| 6 | 580 | 1117 | 09-Apr-13 | XXXXXXXX |
| 6 | 650 | 987 | 09-Apr-13 | XXXXXXXX |
| 6 | 600 | 1196 | 10-Apr-13 | XXXXXXXX |
| 6 | 600 | 793 | 05-Apr-13 | XXXXXXXX |
| 6 | 600 | 861 | 08-Apr-13 | XXXXXXXX |
| 6 | 700 | 1113 | 09-Apr-13 | XXXXXXXX |
| 6 | 600 | 849 | 08-Apr-13 | XXXXXXXX |
| 6 | 650 | 1069 | 09-Apr-13 | XXXXXXXX |
| 6 | 550 | 931 | 09-Apr-13 | XXXXXXXX |
| 6 | 550 | 1034 | 10-Apr-13 | XXXXXXXX |
| 6 | 700 | 1104 | 09-Apr-13 | XXXXXXXX |
| 6 | 650 | 1123 | 09-Apr-13 | XXXXXXXX |
| 6 | 600 | 1000 | 09-Apr-13 | XXXXXXXX |
| 6 | 850 | 930 | 08-Apr-13 | XXXXXXXX |
| 6 | 500 | 1326 | 11-Apr-13 | XXXXXXXX |
| 6 | 700 | 941 | 09-Apr-13 | XXXXXXXX |
| 6 | 600 | 750 | 14-Mar-13 | XXXXXXXX |
| 6 | 600 | 1080 | 09-Apr-13 | XXXXXXXX |
| 6 | 850 | 934 | 08-Apr-13 | XXXXXXXX |
| 6 | 750 | 1347 | 11-Apr-13 | XXXXXXXX |
| 6 | 480 | 879 | 08-Apr-13 | XXXXXXXX |
| 6 | 500 | 1295 | 11-Apr-13 | XXXXXXXX |
| 6 | 600 | 966 | 08-Apr-13 | XXXXXXXX |
| 6 | 550 | 1179 | 10-Apr-13 | XXXXXXXX |
| 6 | 500 | 1194 | 10-Apr-13 | XXXXXXXX |
| 6 | 715 | 900 | 08-Apr-13 | XXXXXXXX |
| 6 | 550 | 978 | 09-Apr-13 | XXXXXXXX |
| 6 | 500 | 1234 | 10-Apr-13 | XXXXXXXX |
| 6 | 700 | 890 | 08-Apr-13 | XXXXXXXX |
| 6 | 800 | 1280 | 10-Apr-13 | XXXXXXXX |
| 6 | 480 | 1219 | 10-Apr-13 | XXXXXXXX |
| 12 | 300 | 1156 | 10-Apr-13 | XXXXXXXX |
| 12 | 300 | 1371 | 11-Apr-13 | XXXXXXXX |

| | | | | |
|---|---|---|---|---|
| 12 | 320 | 1099 | 10-Apr-13 | XXXXXXXX |
| 12 | 350 | 1073 | 10-Apr-13 | XXXXXXXX |
| 12 | 350 | 808 | 05-Apr-13 | XXXXXXXX |
| 12 | 320 | 896 | 08-Apr-13 | XXXXXXXX |
| 12 | 350 | 1342 | 11-Apr-13 | XXXXXXXX |
| 12 | 350 | 1081 | 09-Apr-13 | XXXXXXXX |
| 12 | 300 | 1085 | 09-Apr-13 | XXXXXXXX |
| 12 | 400 | 1350 | 11-Apr-13 | XXXXXXXX |
| 12 | 300 | 788 | 05-Apr-13 | XXXXXXXX |
| 12 | 300 | 728 | 14-Mar-13 | XXXXXXXX |
| 12 | 300 | 738 | 14-Mar-13 | XXXXXXXX |
| 12 | 320 | 1237 | 10-Apr-13 | XXXXXXXX |
| 12 | 300 | 1171 | 10-Apr-13 | XXXXXXXX |
| 12 | 300 | 1006 | 09-Apr-13 | XXXXXXXX |
| 12 | 350 | 946 | 08-Apr-13 | XXXXXXXX |
| 12 | 350 | 1260 | 10-Apr-13 | XXXXXXXX |
| 12 | 350 | 1113 | 10-Apr-13 | XXXXXXXX |
| 12 | 350 | 1314 | 11-Apr-13 | XXXXXXXX |
| 12 | 350 | 1099 | 09-Apr-13 | XXXXXXXX |
| 12 | 320 | 1223 | 10-Apr-13 | XXXXXXXX |
| 12 | 300 | 898 | 08-Apr-13 | XXXXXXXX |
| 12 | 350 | 881 | 08-Apr-13 | XXXXXXXX |
| 12 | 300 | 1150 | 09-Apr-13 | XXXXXXXX |
| 12 | 350 | 1157 | 10-Apr-13 | XXXXXXXX |
| 12 | 300 | 943 | 09-Apr-13 | XXXXXXXX |
| 12 | 350 | 917 | 08-Apr-13 | XXXXXXXX |
| 12 | 300 | 952 | 09-Apr-13 | XXXXXXXX |
| 12 | 300 | 1352 | 11-Apr-13 | XXXXXXXX |
| 12 | 300 | 802 | 05-Apr-13 | XXXXXXXX |
| 12 | 650 | 814 | 05-Apr-13 | XXXXXXXX |
| 12 | 350 | 1367 | 11-Apr-13 | XXXXXXXX |
| 12 | 300 | 1227 | 10-Apr-13 | XXXXXXXX |
| 12 | 300 | 1319 | 11-Apr-13 | XXXXXXXX |
| 12 | 300 | 757 | 14-Mar-13 | XXXXXXXX |
| 12 | 320 | 924 | 08-Apr-13 | XXXXXXXX |
| 12 | 300 | 1031 | 10-Apr-13 | XXXXXXXX |
| 12 | 300 | 954 | 08-Apr-13 | XXXXXXXX |
| 6 | 300 | 1383 | 11-Apr-13 | XXXXXXXX |
| 6 | 280 | 922 | 08-Apr-13 | XXXXXXXX |
| 3 | 200 | 1084 | 10-Apr-13 | XXXXXXXX |
| 3 | 100 | 784 | 05-Apr-13 | XXXXXXXX |
| 3 | 150 | 949 | 08-Apr-13 | XXXXXXXX |
| 24 | 500 | 1232 | 10-Apr-13 | XXXXXXXX |
| 6 | 150 | 1106 | 10-Apr-13 | XXXXXXXX |
| 6 | 200 | 1095 | 10-Apr-13 | XXXXXXXX |
| 6 | 150 | 945 | 09-Apr-13 | XXXXXXXX |
| 6 | 100 | 1248 | 10-Apr-13 | XXXXXXXX |
| 6 | 300 | 899 | 08-Apr-13 | XXXXXXXX |
| 12 | 250 | 1019 | 10-Apr-13 | XXXXXXXX |
| 24 | 500 | 955 | 08-Apr-13 | XXXXXXXX |

| | | | | |
|---|---|---|---|---|
| 6 | 300 | 1090 | 09-Apr-13 | XXXXXXXX |
| 24 | 300 | 949 | 09-Apr-13 | XXXXXXXX |
| 24 | 400 | 868 | 08-Apr-13 | XXXXXXXX |
| 6 | 300 | 720 | 14-Mar-13 | XXXXXXXX |
| 6 | 200 | 1075 | 10-Apr-13 | XXXXXXXX |
| 6 | 400 | 1052 | 10-Apr-13 | XXXXXXXX |
| 6 | 400 | 1109 | 09-Apr-13 | XXXXXXXX |
| 6 | 400 | 998 | 09-Apr-13 | XXXXXXXX |
| 6 | 250 | 981 | 09-Apr-13 | XXXXXXXX |
| 6 | 250 | 1120 | 09-Apr-13 | XXXXXXXX |
| 6 | 750 | 1395 | 11-Apr-13 | XXXXXXXX |
| 6 | 500 | 724 | 14-Mar-13 | XXXXXXXX |
| 6 | 300 | 878 | 08-Apr-13 | XXXXXXXX |
| 6 | 750 | 965 | 08-Apr-13 | XXXXXXXX |
| 6 | 350 | 963 | 09-Apr-13 | XXXXXXXX |
| 6 | 350 | 744 | 14-Mar-13 | XXXXXXXX |
| 6 | 250 | 866 | 08-Apr-13 | XXXXXXXX |
| 6 | 300 | 905 | 08-Apr-13 | XXXXXXXX |
| 6 | 250 | 884 | 08-Apr-13 | XXXXXXXX |
| 6 | 300 | 1154 | 09-Apr-13 | XXXXXXXX |
| 6 | 350 | 1132 | 09-Apr-13 | XXXXXXXX |
| 6 | 300 | 1159 | 10-Apr-13 | XXXXXXXX |
| 3 | 300 | 844 | 08-Apr-13 | XXXXXXXX |
| 6 | 500 | 940 | 09-Apr-13 | XXXXXXXX |
| 6 | 400 | 770 | 05-Apr-13 | XXXXXXXX |
| 6 | 350 | 1298 | 11-Apr-13 | XXXXXXXX |
| 6 | 300 | 1355 | 11-Apr-13 | XXXXXXXX |
| 6 | 400 | 929 | 09-Apr-13 | XXXXXXXX |
| 3 | 200 | 1074 | 10-Apr-13 | XXXXXXXX |
| 3 | 180 | 948 | 09-Apr-13 | XXXXXXXX |
| 3 | 150 | 948 | 08-Apr-13 | XXXXXXXX |
| 3 | 150 | 1318 | 11-Apr-13 | XXXXXXXX |
| 12 | 500 | 871 | 08-Apr-13 | XXXXXXXX |
| 12 | 600 | 800 | 05-Apr-13 | XXXXXXXX |
| 6 | 350 | 1275 | 10-Apr-13 | XXXXXXXX |
| 12 | 600 | 712 | 14-Mar-13 | XXXXXXXX |
| 12 | 500 | 1096 | 10-Apr-13 | XXXXXXXX |
| 6 | 450 | 1209 | 10-Apr-13 | XXXXXXXX |
| 12 | 550 | 1030 | 10-Apr-13 | XXXXXXXX |
| 12 | 520 | 1056 | 10-Apr-13 | XXXXXXXX |
| 12 | 650 | 913 | 09-Apr-13 | XXXXXXXX |
| 12 | 600 | 889 | 08-Apr-13 | XXXXXXXX |
| 12 | 500 | 857 | 08-Apr-13 | XXXXXXXX |
| 12 | 480 | 1011 | 09-Apr-13 | XXXXXXXX |
| 12 | 650 | 1282 | 10-Apr-13 | XXXXXXXX |
| 12 | 700 | 1262 | 10-Apr-13 | XXXXXXXX |
| 12 | 500 | 1197 | 10-Apr-13 | XXXXXXXX |
| 12 | 500 | 1222 | 10-Apr-13 | XXXXXXXX |
| 12 | 550 | 1161 | 10-Apr-13 | XXXXXXXX |
| 12 | 500 | 923 | 08-Apr-13 | XXXXXXXX |

| | | | | |
|---|---|---|---|---|
| 12 | 500 | 1362 | 11-Apr-13 | XXXXXXXX |
| 6 | 400 | 715 | 14-Mar-13 | XXXXXXXX |
| 6 | 350 | 1098 | 10-Apr-13 | XXXXXXXX |
| 6 | 350 | 1210 | 10-Apr-13 | XXXXXXXX |
| 6 | 370 | 1012 | 09-Apr-13 | XXXXXXXX |
| 6 | 380 | 921 | 08-Apr-13 | XXXXXXXX |
| 6 | 550 | 1040 | 10-Apr-13 | XXXXXXXX |
| 6 | 450 | 1008 | 10-Apr-13 | XXXXXXXX |
| 6 | 600 | 1141 | 09-Apr-13 | XXXXXXXX |
| 6 | 500 | 868 | 08-Apr-13 | XXXXXXXX |
| 6 | 550 | 1266 | 10-Apr-13 | XXXXXXXX |
| 6 | 690 | 1138 | 09-Apr-13 | XXXXXXXX |
| 6 | 600 | 1069 | 10-Apr-13 | XXXXXXXX |
| 6 | 750 | 1285 | 10-Apr-13 | XXXXXXXX |
| 6 | 720 | 992 | 09-Apr-13 | XXXXXXXX |
| 6 | 650 | 927 | 08-Apr-13 | XXXXXXXX |
| 6 | 1100 | 1112 | 10-Apr-13 | XXXXXXXX |
| 6 | 950 | 1042 | 10-Apr-13 | XXXXXXXX |
| 6 | 1100 | 895 | 08-Apr-13 | XXXXXXXX |
| 6 | 1050 | 1338 | 11-Apr-13 | XXXXXXXX |
| 6 | 1100 | 1106 | 09-Apr-13 | XXXXXXXX |
| 6 | 900 | 739 | 14-Mar-13 | XXXXXXXX |
| 6 | 50 | 828 | 05-Apr-13 | XXXXXXXX |
| 6 | 950 | 870 | 08-Apr-13 | XXXXXXXX |
| 6 | 950 | 1164 | 10-Apr-13 | XXXXXXXX |
| 1 | 1000 | 1291 | 10-Apr-13 | XXXXXXXX |
| 6 | 1020 | 1013 | 09-Apr-13 | XXXXXXXX |
| 6 | 1100 | 972 | 08-Apr-13 | XXXXXXXX |
| 6 | 1000 | 1254 | 10-Apr-13 | XXXXXXXX |
| 6 | 900 | 960 | 09-Apr-13 | XXXXXXXX |
| 6 | 900 | 1269 | 10-Apr-13 | XXXXXXXX |
| 6 | 1000 | 1354 | 11-Apr-13 | XXXXXXXX |
| 6 | 550 | 815 | 05-Apr-13 | XXXXXXXX |
| 6 | 900 | 759 | 14-Mar-13 | XXXXXXXX |
| 6 | 1000 | 1357 | 11-Apr-13 | XXXXXXXX |
| 6 | 760 | 904 | 08-Apr-13 | XXXXXXXX |
| 6 | 600 | 996 | 09-Apr-13 | XXXXXXXX |
| 6 | 650 | 898 | 08-Apr-13 | XXXXXXXX |
| 9 | 400 | 1274 | 10-Apr-13 | XXXXXXXX |
| 9 | 550 | 762 | 14-Mar-13 | XXXXXXXX |
| 3 | 600 | 716 | 14-Mar-13 | XXXXXXXX |
| 3 | 750 | 1053 | 10-Apr-13 | XXXXXXXX |
| 3 | 650 | 1341 | 11-Apr-13 | XXXXXXXX |
| 3 | 480 | 1014 | 09-Apr-13 | XXXXXXXX |
| 3 | 900 | 1256 | 10-Apr-13 | XXXXXXXX |
| 3 | 600 | 1003 | 10-Apr-13 | XXXXXXXX |
| 3 | 650 | 1109 | 10-Apr-13 | XXXXXXXX |
| 3 | 630 | 1142 | 09-Apr-13 | XXXXXXXX |
| 3 | 800 | 1025 | 10-Apr-13 | XXXXXXXX |
| 6 | 720 | 901 | 08-Apr-13 | XXXXXXXX |

| | | | | |
|---|---|---|---|---|
| 3 | 780 | 1108 | 09-Apr-13 | XXXXXXXX |
| 3 | 650 | 1011 | 10-Apr-13 | XXXXXXXX |
| 3 | 800 | 917 | 09-Apr-13 | XXXXXXXX |
| 3 | 850 | 1079 | 09-Apr-13 | XXXXXXXX |
| 3 | 800 | 1119 | 09-Apr-13 | XXXXXXXX |
| 3 | 650 | 1284 | 10-Apr-13 | XXXXXXXX |
| 6 | 650 | 865 | 08-Apr-13 | XXXXXXXX |
| 3 | 720 | 993 | 09-Apr-13 | XXXXXXXX |
| 3 | 700 | 1270 | 10-Apr-13 | XXXXXXXX |
| 3 | 750 | 928 | 08-Apr-13 | XXXXXXXX |
| 3 | 650 | 1361 | 11-Apr-13 | XXXXXXXX |
| 8 | 350 | 1320 | 11-Apr-13 | XXXXXXXX |
| 12 | 300 | 717 | 14-Mar-13 | XXXXXXXX |
| 12 | 350 | 1110 | 10-Apr-13 | XXXXXXXX |
| 12 | 330 | 1091 | 10-Apr-13 | XXXXXXXX |
| 12 | 300 | 1147 | 09-Apr-13 | XXXXXXXX |
| 12 | 350 | 1043 | 10-Apr-13 | XXXXXXXX |
| 12 | 350 | 1076 | 10-Apr-13 | XXXXXXXX |
| 12 | 350 | 806 | 05-Apr-13 | XXXXXXXX |
| 12 | 500 | 747 | 14-Mar-13 | XXXXXXXX |
| 12 | 300 | 1337 | 11-Apr-13 | XXXXXXXX |
| 12 | 300 | 1027 | 09-Apr-13 | XXXXXXXX |
| 12 | 300 | 874 | 08-Apr-13 | XXXXXXXX |
| 12 | 300 | 873 | 08-Apr-13 | XXXXXXXX |
| 12 | 300 | 1045 | 09-Apr-13 | XXXXXXXX |
| 1 | 300 | 944 | 09-Apr-13 | XXXXXXXX |
| 12 | 400 | 914 | 09-Apr-13 | XXXXXXXX |
| 12 | 350 | 912 | 09-Apr-13 | XXXXXXXX |
| 12 | 350 | 1076 | 09-Apr-13 | XXXXXXXX |
| 12 | 330 | 1084 | 09-Apr-13 | XXXXXXXX |
| 12 | 300 | 936 | 09-Apr-13 | XXXXXXXX |
| 12 | 350 | 977 | 09-Apr-13 | XXXXXXXX |
| 12 | 350 | 891 | 08-Apr-13 | XXXXXXXX |
| 12 | 300 | 1002 | 09-Apr-13 | XXXXXXXX |
| 12 | 350 | 1349 | 11-Apr-13 | XXXXXXXX |
| 12 | 300 | 1061 | 10-Apr-13 | XXXXXXXX |
| 12 | 300 | 1247 | 10-Apr-13 | XXXXXXXX |
| 12 | 300 | 780 | 05-Apr-13 | XXXXXXXX |
| 12 | 350 | 736 | 14-Mar-13 | XXXXXXXX |
| 12 | 550 | 1394 | 11-Apr-13 | XXXXXXXX |
| 12 | 300 | 726 | 14-Mar-13 | XXXXXXXX |
| 12 | 300 | 987 | 09-Apr-13 | XXXXXXXX |
| 12 | 350 | 827 | 05-Apr-13 | XXXXXXXX |
| 12 | | 855 | 08-Apr-13 | XXXXXXXX |
| 12 | 350 | 1304 | 11-Apr-13 | XXXXXXXX |
| 12 | 350 | 1240 | 10-Apr-13 | XXXXXXXX |
| 12 | 350 | 894 | 08-Apr-13 | XXXXXXXX |
| 12 | 300 | 1170 | 10-Apr-13 | XXXXXXXX |
| 12 | 300 | 881 | 08-Apr-13 | XXXXXXXX |
| 12 | 300 | 994 | 09-Apr-13 | XXXXXXXX |

| | | | | |
|---|---|---|---|---|
| 12 | 350 | 945 | 08-Apr-13 | XXXXXXXX |
| 12 | 350 | 1253 | 10-Apr-13 | XXXXXXXX |
| 12 | 300 | 1193 | 10-Apr-13 | XXXXXXXX |
| 12 | 350 | 921 | 09-Apr-13 | XXXXXXXX |
| 12 | 300 | 732 | 14-Mar-13 | XXXXXXXX |
| 12 | 350 | 791 | 05-Apr-13 | XXXXXXXX |
| 12 | 400 | 1117 | 10-Apr-13 | XXXXXXXX |
| 12 | 300 | 938 | 08-Apr-13 | XXXXXXXX |
| 12 | 300 | 1098 | 09-Apr-13 | XXXXXXXX |
| 12 | 350 | 746 | 14-Mar-13 | XXXXXXXX |
| 12 | 350 | 1000 | 10-Apr-13 | XXXXXXXX |
| 12 | 320 | 1220 | 10-Apr-13 | XXXXXXXX |
| 12 | 300 | 903 | 08-Apr-13 | XXXXXXXX |
| 12 | 300 | 908 | 08-Apr-13 | XXXXXXXX |
| 12 | 300 | 1186 | 10-Apr-13 | XXXXXXXX |
| 12 | 350 | 1178 | 10-Apr-13 | XXXXXXXX |
| 12 | 300 | 1151 | 09-Apr-13 | XXXXXXXX |
| 12 | 300 | 992 | 09-Apr-13 | XXXXXXXX |
| 12 | 300 | 877 | 08-Apr-13 | XXXXXXXX |
| 12 | 300 | 1160 | 10-Apr-13 | XXXXXXXX |
| 12 | 300 | 853 | 08-Apr-13 | XXXXXXXX |
| 12 | 300 | 834 | 05-Apr-13 | XXXXXXXX |
| 12 | 250 | 1307 | 11-Apr-13 | XXXXXXXX |
| 12 | 300 | 705 | 14-Mar-13 | XXXXXXXX |
| 12 | 350 | 1018 | 09-Apr-13 | XXXXXXXX |
| 12 | 300 | 1308 | 11-Apr-13 | XXXXXXXX |
| 12 | 350 | 953 | 08-Apr-13 | XXXXXXXX |
| 12 | 300 | 816 | 05-Apr-13 | XXXXXXXX |
| 12 | 300 | 1365 | 11-Apr-13 | XXXXXXXX |
| 12 | 300 | 763 | 14-Mar-13 | XXXXXXXX |
| 12 | 350 | 1360 | 11-Apr-13 | XXXXXXXX |
| 8 | 750 | 1043 | 09-Apr-13 | XXXXXXXX |
| 8 | 450 | 1370 | 11-Apr-13 | XXXXXXXX |
| 8 | 500 | 971 | 09-Apr-13 | XXXXXXXX |
| 8 | 500 | 1296 | 11-Apr-13 | XXXXXXXX |
| 8 | 500 | 999 | 10-Apr-13 | XXXXXXXX |
| 8 | 450 | 1091 | 09-Apr-13 | XXXXXXXX |
| 8 | 650 | 713 | 14-Mar-13 | XXXXXXXX |
| 8 | 540 | 1008 | 09-Apr-13 | XXXXXXXX |
| 8 | 350 | 1384 | 11-Apr-13 | XXXXXXXX |
| 8 | 600 | 874 | 08-Apr-13 | XXXXXXXX |
| 8 | 600 | 1372 | 11-Apr-13 | XXXXXXXX |
| 8 | 500 | 1092 | 10-Apr-13 | XXXXXXXX |
| 8 | 500 | 1205 | 10-Apr-13 | XXXXXXXX |
| 8 | 500 | 1038 | 10-Apr-13 | XXXXXXXX |
| 8 | 550 | 805 | 05-Apr-13 | XXXXXXXX |
| 8 | 500 | 1027 | 10-Apr-13 | XXXXXXXX |
| 8 | 500 | 1329 | 11-Apr-13 | XXXXXXXX |
| 8 | 500 | 1006 | 10-Apr-13 | XXXXXXXX |
| 8 | 530 | 1042 | 09-Apr-13 | XXXXXXXX |

| | | | | |
|---|---|---|---|---|
| 8 | 600 | 983 | 09-Apr-13 | XXXXXXXX |
| 8 | 600 | 1052 | 09-Apr-13 | XXXXXXXX |
| 8 | 450 | 1087 | 09-Apr-13 | XXXXXXXX |
| 8 | 600 | 994 | 09-Apr-13 | XXXXXXXX |
| 8 | 450 | 1063 | 09-Apr-13 | XXXXXXXX |
| 8 | 480 | 1376 | 11-Apr-13 | XXXXXXXX |
| 8 | 480 | 787 | 05-Apr-13 | XXXXXXXX |
| 8 | 550 | 841 | 05-Apr-13 | XXXXXXXX |
| 8 | 650 | 1393 | 11-Apr-13 | XXXXXXXX |
| 8 | 500 | 723 | 14-Mar-13 | XXXXXXXX |
| 8 | 500 | 853 | 08-Apr-13 | XXXXXXXX |
| 8 | 500 | 1299 | 11-Apr-13 | XXXXXXXX |
| 8 | 600 | 1241 | 10-Apr-13 | XXXXXXXX |
| 8 | 550 | 1167 | 10-Apr-13 | XXXXXXXX |
| 8 | 650 | 967 | 08-Apr-13 | XXXXXXXX |
| 8 | 460 | 1009 | 09-Apr-13 | XXXXXXXX |
| 8 | 500 | 1257 | 10-Apr-13 | XXXXXXXX |
| 8 | 500 | 1195 | 10-Apr-13 | XXXXXXXX |
| 8 | 500 | 1095 | 09-Apr-13 | XXXXXXXX |
| 8 | 500 | 1001 | 10-Apr-13 | XXXXXXXX |
| 8 | 480 | 1218 | 10-Apr-13 | XXXXXXXX |
| 8 | 500 | 910 | 08-Apr-13 | XXXXXXXX |
| 8 | 480 | 1175 | 10-Apr-13 | XXXXXXXX |
| 8 | 500 | 1152 | 09-Apr-13 | XXXXXXXX |
| 8 | 450 | 876 | 08-Apr-13 | XXXXXXXX |
| 8 | 555 | 847 | 08-Apr-13 | XXXXXXXX |
| 8 | 500 | 852 | 08-Apr-13 | XXXXXXXX |
| 8 | 450 | 1267 | 10-Apr-13 | XXXXXXXX |
| 8 | 450 | 707 | 14-Mar-13 | XXXXXXXX |
| 8 | 600 | 772 | 05-Apr-13 | XXXXXXXX |
| 8 | 500 | 1311 | 11-Apr-13 | XXXXXXXX |
| 8 | 500 | 801 | 05-Apr-13 | XXXXXXXX |
| 8 | 500 | 1369 | 11-Apr-13 | XXXXXXXX |
| 8 | 550 | 813 | 05-Apr-13 | XXXXXXXX |
| 8 | 450 | 1226 | 10-Apr-13 | XXXXXXXX |
| 8 | 550 | 758 | 14-Mar-13 | XXXXXXXX |
| 8 | 480 | 920 | 08-Apr-13 | XXXXXXXX |
| 8 | 500 | 934 | 09-Apr-13 | XXXXXXXX |
| 8 | 500 | 1358 | 11-Apr-13 | XXXXXXXX |
| 8 | 550 | 955 | 09-Apr-13 | XXXXXXXX |
| 8 | 400 | 1090 | 10-Apr-13 | XXXXXXXX |
| 8 | 550 | 1075 | 09-Apr-13 | XXXXXXXX |
| 8 | 550 | 1276 | 10-Apr-13 | XXXXXXXX |
| 8 | 450 | 855 | 08-Apr-13 | XXXXXXXX |
| 12 | 500 | 869 | 08-Apr-13 | XXXXXXXX |
| 12 | 550 | 865 | 08-Apr-13 | XXXXXXXX |
| 12 | 500 | 989 | 09-Apr-13 | XXXXXXXX |
| 6 | 550 | 1306 | 11-Apr-13 | XXXXXXXX |
| 1 | 600 | 1103 | 10-Apr-13 | XXXXXXXX |
| 1 | 600 | 1206 | 10-Apr-13 | XXXXXXXX |

| | | | | |
|---|---|---|---|---|
| 1 | 600 | 887 | 08-Apr-13 | XXXXXXXX |
| 1 | 600 | 976 | 08-Apr-13 | XXXXXXXX |
| 1 | 600 | 795 | 05-Apr-13 | XXXXXXXX |
| 1 | 500 | 703 | 14-Mar-13 | XXXXXXXX |
| 1 | 550 | 971 | 08-Apr-13 | XXXXXXXX |
| 1 | 570 | 1079 | 10-Apr-13 | XXXXXXXX |
| 1 | 750 | 919 | 09-Apr-13 | XXXXXXXX |
| 1 | 700 | 885 | 08-Apr-13 | XXXXXXXX |
| 1 | 400 | 1066 | 10-Apr-13 | XXXXXXXX |
| 1 | 500 | 1294 | 10-Apr-13 | XXXXXXXX |
| 1 | 550 | 1021 | 10-Apr-13 | XXXXXXXX |
| 1 | 500 | 939 | 09-Apr-13 | XXXXXXXX |
| 1 | 600 | 1056 | 09-Apr-13 | XXXXXXXX |
| 1 | 600 | 838 | 05-Apr-13 | XXXXXXXX |
| 1 | 550 | 1290 | 10-Apr-13 | XXXXXXXX |
| 1 | 650 | 947 | 08-Apr-13 | XXXXXXXX |
| 1 | 750 | 1081 | 10-Apr-13 | XXXXXXXX |
| 1 | 750 | 1137 | 09-Apr-13 | XXXXXXXX |
| 1 | 850 | 894 | 08-Apr-13 | XXXXXXXX |
| 1 | 800 | 915 | 09-Apr-13 | XXXXXXXX |
| 1 | 850 | 1072 | 09-Apr-13 | XXXXXXXX |
| 1 | 1000 | 1031 | 09-Apr-13 | XXXXXXXX |
| 1 | 850 | 840 | 05-Apr-13 | XXXXXXXX |
| 1 | 700 | 1302 | 11-Apr-13 | XXXXXXXX |
| 1 | 780 | 950 | 08-Apr-13 | XXXXXXXX |
| 1 | 850 | 1293 | 10-Apr-13 | XXXXXXXX |
| 1 | | 1102 | 10-Apr-13 | XXXXXXXX |
| 1 | 900 | 1249 | 10-Apr-13 | XXXXXXXX |
| 1 | 450 | 1272 | 10-Apr-13 | XXXXXXXX |
| 1 | 600 | 860 | 08-Apr-13 | XXXXXXXX |
| 1 | 500 | 1136 | 09-Apr-13 | XXXXXXXX |
| 1 | 600 | 1024 | 09-Apr-13 | XXXXXXXX |
| 1 | 500 | 1040 | 09-Apr-13 | XXXXXXXX |
| 1 | 500 | 776 | 05-Apr-13 | XXXXXXXX |
| 1 | | 943 | 08-Apr-13 | XXXXXXXX |
| 1 | 500 | 1041 | 09-Apr-13 | XXXXXXXX |
| 1 | 600 | 1165 | 10-Apr-13 | XXXXXXXX |
| 1 | 500 | 1373 | 11-Apr-13 | XXXXXXXX |
| 1 | 700 | 796 | 05-Apr-13 | XXXXXXXX |
| 1 | 550 | 962 | 09-Apr-13 | XXXXXXXX |
| 1 | 530 | 1214 | 10-Apr-13 | XXXXXXXX |
| 1 | 400 | 1122 | 09-Apr-13 | XXXXXXXX |
| 1 | 650 | 700 | 14-Mar-13 | XXXXXXXX |
| 1 | 400 | 1022 | 10-Apr-13 | XXXXXXXX |
| 1 | 650 | 1104 | 10-Apr-13 | XXXXXXXX |
| 1 | 600 | 1082 | 10-Apr-13 | XXXXXXXX |
| 1 | 600 | 1140 | 09-Apr-13 | XXXXXXXX |
| 1 | 600 | 1204 | 10-Apr-13 | XXXXXXXX |
| 1 | 630 | 1044 | 10-Apr-13 | XXXXXXXX |
| 1 | 650 | 807 | 05-Apr-13 | XXXXXXXX |

| | | | | |
|---|---|---|---|---|
| 1 | 600 | 1054 | 10-Apr-13 | XXXXXXXX |
| 1 | 650 | 899 | 08-Apr-13 | XXXXXXXX |
| 1 | 600 | 1330 | 11-Apr-13 | XXXXXXXX |
| 1 | 750 | 916 | 09-Apr-13 | XXXXXXXX |
| 1 | 600 | 1058 | 10-Apr-13 | XXXXXXXX |
| 1 | 600 | 1243 | 10-Apr-13 | XXXXXXXX |
| 1 | 550 | 969 | 09-Apr-13 | XXXXXXXX |
| 1 | 250 | 842 | 05-Apr-13 | XXXXXXXX |
| 1 | 600 | 1305 | 11-Apr-13 | XXXXXXXX |
| 1 | 700 | 893 | 08-Apr-13 | XXXXXXXX |
| 1 | 600 | 1162 | 10-Apr-13 | XXXXXXXX |
| 1 | 550 | 883 | 08-Apr-13 | XXXXXXXX |
| 1 | 650 | 1292 | 10-Apr-13 | XXXXXXXX |
| 1 | 650 | 960 | 08-Apr-13 | XXXXXXXX |
| 1 | 800 | 1261 | 10-Apr-13 | XXXXXXXX |
| 1 | 700 | 794 | 05-Apr-13 | XXXXXXXX |
| 1 | 850 | 1115 | 10-Apr-13 | XXXXXXXX |
| 1 | 550 | 959 | 09-Apr-13 | XXXXXXXX |
| 1 | 580 | 1325 | 11-Apr-13 | XXXXXXXX |
| 1 | 600 | 907 | 08-Apr-13 | XXXXXXXX |
| 1 | 600 | 915 | 08-Apr-13 | XXXXXXXX |
| 1 | 600 | 1184 | 10-Apr-13 | XXXXXXXX |
| 1 | 680 | 1174 | 10-Apr-13 | XXXXXXXX |
| 1 | 600 | 1149 | 09-Apr-13 | XXXXXXXX |
| 1 | | 850 | 08-Apr-13 | XXXXXXXX |
| 1 | 600 | 851 | 08-Apr-13 | XXXXXXXX |
| 1 | 650 | 1066 | 09-Apr-13 | XXXXXXXX |
| 1 | 580 | 702 | 14-Mar-13 | XXXXXXXX |
| 1 | 450 | 1088 | 09-Apr-13 | XXXXXXXX |
| 6 | 250 | 1273 | 10-Apr-13 | XXXXXXXX |
| 6 | 300 | 1201 | 10-Apr-13 | XXXXXXXX |
| 6 | 300 | 1029 | 10-Apr-13 | XXXXXXXX |
| 6 | 250 | 1010 | 09-Apr-13 | XXXXXXXX |
| 6 | 260 | 1097 | 10-Apr-13 | XXXXXXXX |
| 6 | 280 | 1144 | 09-Apr-13 | XXXXXXXX |
| 6 | 300 | 1200 | 10-Apr-13 | XXXXXXXX |
| 6 | 300 | 1339 | 11-Apr-13 | XXXXXXXX |
| 6 | 300 | 1048 | 09-Apr-13 | XXXXXXXX |
| 6 | 350 | 1001 | 09-Apr-13 | XXXXXXXX |
| 6 | 300 | 737 | 14-Mar-13 | XXXXXXXX |
| 6 | 260 | 995 | 09-Apr-13 | XXXXXXXX |
| 1 | 480 | 1105 | 10-Apr-13 | XXXXXXXX |
| 1 | 480 | 1083 | 10-Apr-13 | XXXXXXXX |
| 1 | 550 | 1045 | 10-Apr-13 | XXXXXXXX |
| 1 | 650 | 897 | 08-Apr-13 | XXXXXXXX |
| 1 | 500 | 1335 | 11-Apr-13 | XXXXXXXX |
| 1 | 470 | 1070 | 10-Apr-13 | XXXXXXXX |
| 1 | 430 | 1039 | 09-Apr-13 | XXXXXXXX |
| 1 | 450 | 999 | 09-Apr-13 | XXXXXXXX |
| 1 | 500 | 1244 | 10-Apr-13 | XXXXXXXX |

| | | | | |
|---|---|---|---|---|
| 1 | 450 | 781 | 05-Apr-13 | XXXXXXXX |
| 1 | 460 | 997 | 09-Apr-13 | XXXXXXXX |
| 1 | 500 | 975 | 08-Apr-13 | XXXXXXXX |
| 1 | 600 | 1114 | 10-Apr-13 | XXXXXXXX |
| 1 | 450 | 961 | 09-Apr-13 | XXXXXXXX |
| 1 | 500 | 936 | 08-Apr-13 | XXXXXXXX |
| 1 | 450 | 909 | 08-Apr-13 | XXXXXXXX |
| 1 | 550 | 1173 | 10-Apr-13 | XXXXXXXX |
| 1 | 450 | 1130 | 09-Apr-13 | XXXXXXXX |
| 1 | 500 | 1356 | 11-Apr-13 | XXXXXXXX |
| 1 | 480 | 1080 | 10-Apr-13 | XXXXXXXX |
| 1 | 490 | 1077 | 10-Apr-13 | XXXXXXXX |
| 1 | 500 | 1334 | 11-Apr-13 | XXXXXXXX |
| 1 | 500 | 1010 | 10-Apr-13 | XXXXXXXX |
| 1 | 550 | 1026 | 09-Apr-13 | XXXXXXXX |
| 1 | 650 | 918 | 09-Apr-13 | XXXXXXXX |
| 1 | 700 | 1050 | 09-Apr-13 | XXXXXXXX |
| 1 | 550 | 1073 | 09-Apr-13 | XXXXXXXX |
| 1 | 480 | 1126 | 09-Apr-13 | XXXXXXXX |
| 1 | 500 | 1057 | 09-Apr-13 | XXXXXXXX |
| 1 | 500 | 1374 | 11-Apr-13 | XXXXXXXX |
| 1 | 500 | 880 | 08-Apr-13 | XXXXXXXX |
| 1 | 460 | 991 | 09-Apr-13 | XXXXXXXX |
| 1 | 480 | 1172 | 10-Apr-13 | XXXXXXXX |
| 1 | 550 | 906 | 08-Apr-13 | XXXXXXXX |
| 1 | 500 | 911 | 08-Apr-13 | XXXXXXXX |
| 1 | 500 | 1185 | 10-Apr-13 | XXXXXXXX |
| 1 | 450 | 1129 | 09-Apr-13 | XXXXXXXX |
| 1 | 500 | 852 | 08-Apr-13 | XXXXXXXX |
| 1 | | | 15-Apr-13 | XXXXXXXX |
| 1 | 860 | 1078 | 10-Apr-13 | XXXXXXXX |
| 1 | 570 | 1133 | 09-Apr-13 | XXXXXXXX |
| 1 | 550 | 1009 | 10-Apr-13 | XXXXXXXX |
| 1 | 480 | 1007 | 09-Apr-13 | XXXXXXXX |
